# Data supporting the identification of anti-metastatic drug and natural compound targets in isogenic colorectal cancer cells

**DOI:** 10.1016/j.dib.2014.10.005

**Published:** 2014-11-04

**Authors:** Jin-Gyun Lee, Kimberly Q. McKinney, Antonis J. Pavlopoulos, Jeong-Hill Park, Sunil Hwang

**Affiliations:** a Proteomics Laboratory for Clinical and Translational Research, Carolinas HealthCare System, Charlotte, NC 28203, United States; b College of Pharmacy, Seoul National University, Seoul 151-742, South Korea

## Abstract

To investigate molecular therapeutic targets in cancer metastasis, comparative proteomic analysis was performed using the isogenic colorectal cancer cell lines SW480 and SW620. Two potential metastasis related molecular targets were identified: fatty acid synthase and histone H4. Subsequently, metastatic SW620 cells were treated with six anti-cancerous components and suppressive effects were observed in target protein expression. Through comprehensive proteomic analysis, three of the tested compounds, oxaliplatin, ginsenoside 20(S)-Rg_3_ and curcumin, were determined to have a suppressive effect on fatty acid synthase and histone H4 expression [1]. The current article contains one table exhibiting a list of proteins differentially expressed in metastatic SW620 cell lines compared to the primary SW480 cell line (Supplementary Table 1). Additionally, six tables demonstrate proteome changes in SW620 resulting from the treatment of three chemotherapeutics and three natural components (Supplementary Tables 1–7). The anti-metastatic components revealed by the current proteomic analysis represent promising chemotherapeutic candidates for the treatment of colorectal adenocarcinoma.

**Table:** 

**Specifications table**	

Subject area	Biochemistry
More specific subject area	Proteomics
Type of data	Tables
How data was acquired	Using a linear ion-trap mass spectrometry combined with nano-UPLC (Thermo Scientific LTQ/Orbitrap-XL+Waters Nanoacquity UPLC system)
Data format	Data were filtered by XCorr score after SEQUEST search, compiled using Scaffold^™^ and statistically analyzed by Power Law Global Error Model (PLGEM).
Experimental factors	SW620 cells were treated with oxaliplatin, sorafenib, 5-fluorouracil, ginsenoside 20(S)-Rg_3_, curcumin, and luteolin (see Supplementary Tables)
Experimental features	Proteomics data from SW480 and SW620 with various drug treatment
Consent	N/a
Data source location	Data are present with this article

Value of the data

**Table:** 

• The presented list of differentially expressed proteins from SW620 and SW480 could represent putative molecular targets involved in colorectal cancer metastasis. • Drug treatment of the metastatic SW620 cell line provides comprehensive information about cellular response to the tested drugs. • Current experimental datasets may provide insight as to whether combinational treatment might allow a better therapeutic effect via synergistic activity.

## 1 Experimental design, materials and methods

### 1.1 Cell culture and drug treatment

**Fig. 1:**
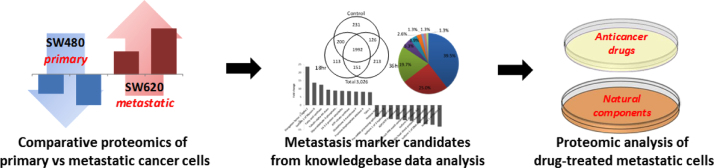
A graphical work flow of comprehensive proteomic analysis of colorectal cancer cells.

Fig. 1 shows a workflow of proteomic analysis for characterization of metastatic cancer biomarkers. Cells were cultured in a Dulbecco׳s modified eagle medium (DMEM, Gibco BRL Life Technologies, Grand Island, NY, USA) containing 10% fetal bovine serum (FBS), 50 units/mL of penicillin G and 50 mcg/mL of streptomycin which were purchased from Gibco BRL Life Technologies (Grand Island, NY, USA). Cells were maintained at 37 °C under humidified 95% air and 5% CO_2_ and grown to confluence in culture dishes (150 mm diameter) over 2 or 3 days and then trypsinized and used for the experiments. Stock solutions of tested drugs were diluted to the desired concentration and were administered in the presence of DMEM with reduced serum (0.5% FBS). Three chemotherapeutics, namely oxaliplatin, sorafenib and 5-fluorouracil were treated at concentrations of 10 μM, 0.15 μM and 10 μM, and herbal dietary components, namely ginsenoside 20(S)-Rg_3_, curcumin and luteolin were also treated at 10 μM, 20 μM and 50 μM, respectively.

### 1.2 Proteomic sample preparation

The PBS-washed pellet of trypsinized cells were lysed with radioimmunoprecipitation (RIPA) buffer containing 50 mM Tris (pH 8.0), 150 mM NaCl, 1.0% (v/v) Triton X-100, 0.5% (w/v) deoxycholate and 1× protease inhibitor. 50 μg of denatured protein with sample buffer containing 300 mM Tris–HCl, 0.01% (w/v) bromophenol blue, 15% (v/v) gycerol, 6% (w/v) SDS and 1% (v/v) β-mercaptoethanol were separated on 10% Bis–Tris NuPAGE gels. Gels were stained using 0.04% (w/v) Coomassie brilliant blue G in 3.5% (v/v) perchloric acid for 15 min and then destained using deionized water with several changes overnight. Each gel lane was cut into 20 slices, which were chopped into small pieces. Gel pieces were destained with 50% (v/v) acetonitrile (ACN) containing 25 mM ammonium bicarbonate several times, and then were dehydrated in 100% ACN. After being dried in a Centrivap (Labconco, Kansas City, MO, USA), gel pieces were rehydrated in 50 mM ammonium bicarbonate containing 12.5 ng/μL trypsin, and then incubated at 37 °C overnight. Peptides were extracted by adding 100 μL 50% (v/v) ACN containing 5% (v/v) formic acid and incubated at room temperature for 30 min 3 times. The extracts were dried under vacuum and then were suspended in 5% (v/v) ACN containing 3% (v/v) formic acid to be subjected to LC–MS/MS.

### 1.3 Nano-LC and mass spectrometry analysis

The LC–MS/MS system used consisted of an LTQ/Orbitrap-XL mass spectrometer (Thermo Scientific, Rockford, IL, USA) equipped with a NanoAcquity UPLC system (Waters, Milford, MA, USA). Peptides were separated on a reversed phase analytical column (Nanoacquity BEH C18, 1.7 μm, 150 mm, Waters, Milford, MA, USA) combined with trap column (NanoAcquity, Waters, Milford, MA, USA). Good chromatographic separation was observed with a 75 min linear gradient consisting of mobile phases solvent A (0.1% formic acid in water) and solvent B (0.1% formic acid in ACN) where the gradient was from 5% B at 0 min to 40% B at 65 min. MS spectra were acquired by data dependent scans consisting of MS/MS scans of the eight most intense ions from the full MS scan with dynamic exclusion of 30 s.

### 1.4 Database search and data compiling

The Human International Protein Index (IPI) v3.72 FASTA database (86,392 entries) was used within the Bioworks software v.3.3.1 sp1 applying the SEQUEST search algorithm (SRF v.5). Search parameters were as follows: parent mass tolerance of 10 ppm, fragment mass tolerance of 0.5 Da (monoisotopic), variable modification on methionine of 16 Da (oxidation) and maximum missed cleavage of 2 sites assuming the digestion enzyme trypsin. Search results were compiled using Scaffold software v4.0.5 (Proteome Software, Portland, OR, USA) which provided spectral counts for data comparison under the filter criteria of 2 peptides minimum: XCorr scores of greater than 1.9, 2.3, 3.4 for singly, doubly and triply charged peptides, deltaCn scores of greater than 0.10. Spectral counts from duplicate analysis were compared using the Power Law Global Error Model (PLGEM) in order to identify the significance of the protein changes [2].
