# Supplementary material for: Data supporting the identification of anti-metastatic drug and natural compound targets in isogenic colorectal cancer cells
Source: Data Brief. 2014 Nov 4;1:73–5. doi: 10.1016/j.dib.2014.10.005 (PMC4459770; doi:10.1016/j.dib.2014.10.005)
Supplement: Supplementary file 1 — Supplementary data [file mmc1.zip › CRC_Metastasis_DIB_Table01.pdf]

Table 1. List of proteins differentially expressed in SW620 comparing to those in SW480. ([1]STN and p-value were acquired from PLGEM analysis, [2]Raw spectral counts from data compilation using ScaffoldTM software)

No.	Description	Accession number	STN[1]	p-Value[1]	480_A[2]	480_B[2]	620_A[2]	620_B[2]
1	Fatty acid synthase	IPI00026781	22.910	0.00007	75	72	177	184
2	L-lactate dehydrogenase B chain	IPI00219217	21.905	0.00007	54	52	155	148
3	Elongation factor 1-alpha 2	IPI00014424	21.867	0.00007	180	194	258	344
4	Isoform M1 of Pyruvate kinase isozymes M1/M2	IPI00220644	20.060	0.00007	78	68	168	164
5	Histone H4	IPI00453473	19.568	0.00007	133	159	242	247
6	32 kDa protein	IPI00176692	18.367	0.00007	74	75	167	152
7	Isoform 1 of Splicing factor 3B subunit 3	IPI00300371	17.629	0.00007	50	58	128	137
8	Isoform 1 of L-lactate dehydrogenase A chain	IPI00217966	16.885	0.00007	109	98	186	183
9	Pre-mRNA-processing-splicing factor 8	IPI00007928	16.219	0.00007	80	75	151	154
10	Profilin-1	IPI00216691	14.257	0.00021	74	61	142	122
11	Isoform 1 of Myoferlin	IPI00021048	13.220	0.00021	53	54	106	117
12	Plastin-2	IPI00010471	13.003	0.00021	3	0	46	45
13	Neutral amino acid transporter B(0)	IPI00019472	12.868	0.00021	24	23	80	70
14	14-3-3 protein epsilon	IPI00000816	12.747	0.00021	100	105	161	165
15	Elongation factor 1-alpha	IPI00025447	11.822	0.00021	52	53	108	100
16	Galectin-1	IPI00219219	11.189	0.00021	24	19	63	68
17	Nestin	IPI00010800	10.848	0.00021	10	10	46	53
18	Translational activator GCN1	IPI00001159	10.804	0.00021	61	66	113	110
19	Villin-1	IPI00218852	10.654	0.00021	0	0	34	38
20	Isoform 1 of Nucleolar RNA helicase 2	IPI00015953	10.623	0.00021	27	25	73	64
21	DNA damage-binding protein 1	IPI00293464	9.625	0.00021	29	32	70	69
22	ATP-dependent RNA helicase A	IPI00844578	9.227	0.00021	102	89	139	138
23	Isoform 1 of Heterogeneous nuclear ribonucleoprotein K	IPI00216049	9.106	0.00021	66	68	96	119
24	Glyceraldehyde-3-phosphate dehydrogenase	IPI00219018	9.042	0.00021	273	292	340	323
25	NAD(P) transhydrogenase, mitochondrial	IPI00337541	8.967	0.00021	11	11	43	44
26	40S ribosomal protein S3	IPI00011253	8.909	0.00021	58	61	93	104
27	Serine hydroxymethyltransferase, mitochondrial	IPI00002520	8.827	0.00021	16	19	56	46
28	Nuclear pore complex protein Nup205	IPI00783781	8.649	0.00021	31	32	68	65
29	Inorganic pyrophosphatase	IPI00015018	8.603	0.00021	23	22	58	54
30	Isoform M2 of Pyruvate kinase isozymes M1/M2	IPI00479186	8.217	0.00021	21	25	57	53
31	NCL protein	IPI00183526	8.195	0.00021	42	28	65	72
32	Isoform 1 of Vinculin	IPI00291175	7.953	0.00021	45	35	69	77
33	Isoform C1 of Heterogeneous nuclear ribonucleoproteins C1/C2	IPI00216592	7.884	0.00021	24	27	59	54
34	N-acetyltransferase 10	IPI00300127	7.796	0.00021	15	16	42	47
35	Alanyl-tRNA synthetase, cytoplasmic	IPI00027442	7.669	0.00021	18	13	43	45
36	Isoform B1 of Heterogeneous nuclear ribonucleoproteins A2/B1	IPI00396378	7.595	0.00021	130	117	169	151
37	6-phosphofructokinase type C	IPI00009790	7.545	0.00021	14	12	37	44
38	Gamma-glutamyl hydrolase	IPI00023728	7.540	0.00021	6	6	32	31
39	Isoform 1 of Tensin-3	IPI00658152	7.485	0.00021	2	3	24	28
40	FACT complex subunit SPT16	IPI00026970	7.477	0.00021	39	42	71	72
41	T-complex protein 1 subunit alpha	IPI00290566	7.328	0.00021	33	31	61	62
42	T-complex protein 1 subunit beta	IPI00297779	7.222	0.00021	43	40	78	65
43	T-complex protein 1 subunit zeta	IPI00027626	7.022	0.00021	29	31	60	56
44	ATP-binding cassette sub-family E member 1	IPI00303207	7.008	0.00021	17	15	43	41
45	Elongation factor 2	IPI00186290	6.908	0.00021	89	82	118	116
46	Tubulin, beta	IPI00645452	6.666	0.00028	22	21	52	42
47	Eukaryotic initiation factor 4A-I	IPI00025491	6.655	0.00028	77	81	108	110
48	Wolframin	IPI00008711	6.560	0.00028	0	2	24	20
49	Alpha-actinin-4	IPI00013808	6.438	0.00028	81	98	116	122
50	Histone H1.2	IPI00217465	6.381	0.00028	29	34	54	60
51	Isoform Long of Sodium/potassium-transporting ATPase subunit alpha-1	IPI00006482	6.144	0.00028	41	45	64	73
52	Heterogeneous nuclear ribonucleoprotein F	IPI00003881	6.137	0.00028	12	8	32	31
53	cDNA FLJ40024 fis, clone STOMA2007745, highly similar to UBIQUITIN-ACTIVATING ENZYME E1	IPI00026119	6.018	0.00034	46	41	74	63
54	Cofilin-1	IPI00012011	6.002	0.00034	64	74	107	84
55	Gamma-enolase	IPI00216171	5.982	0.00034	43	35	65	62
56	Isoform 5 of Glycogen debranching enzyme	IPI00219065	5.917	0.00034	7	4	27	23
57	Histone H2B type 2-E	IPI00003935	5.741	0.00034	40	68	76	81
58	Ribonucleoside-diphosphate reductase large subunit	IPI00013871	5.708	0.00034	2	3	22	18
59	Isoform 1 of Chromodomain-helicase-DNA-binding protein 4	IPI00000846	5.666	0.00034	31	31	53	54
60	Isoform 2 of Splicing factor 3B subunit 3	IPI00179138	5.651	0.00034	2	2	15	23
61	40S ribosomal protein S3a	IPI00419880	5.629	0.00034	24	13	41	38
62	Endoplasmin	IPI00027230	5.603	0.00034	68	62	79	100
63	Isoform 1 of Acetyl-CoA carboxylase 1	IPI00011569	5.498	0.00034	2	2	18	19
64	Non-POU domain-containing octamer-binding protein	IPI00304596	5.302	0.00045	25	26	44	48
65	Small nuclear ribonucleoprotein Sm D1	IPI00302850	5.274	0.00045	17	11	31	35
66	Leucine-rich PPR motif-containing protein, mitochondrial	IPI00783271	5.221	0.00045	90	72	113	96
67	cDNA FLJ25678 fis, clone TST04067, highly similar to PURINE NUCLEOSIDE PHOSPHORYLASE	IPI00017672	5.214	0.00045	24	24	41	47
68	Tubulin alpha-4A chain	IPI00007750	5.201	0.00045	201	184	219	219
69	Isoform A1-B of Heterogeneous nuclear ribonucleoprotein A1	IPI00215965	5.175	0.00045	48	42	69	64
70	60S ribosomal protein L10a	IPI00412579	5.154	0.00045	23	20	45	37
71	Isoform 3 of Spectrin alpha chain, brain	IPI00843765	5.146	0.00045	98	82	112	115
72	T-complex protein 1 subunit gamma isoform b	IPI00290770	5.001	0.00045	23	22	39	44
73	Isoform 1 of ATP-dependent RNA helicase DDX19B	IPI00008943	4.951	0.00045	3	2	13	22
74	Isoform 3 of LIM domain only protein 7	IPI00291802	4.941	0.00045	8	11	25	28
75	Complement component 1 Q subcomponent-binding protein, mitochondrial	IPI00014230	4.927	0.00045	21	20	38	40
76	60 kDa heat shock protein, mitochondrial	IPI00784154	4.863	0.00045	173	185	199	208
77	Aspartate aminotransferase, mitochondrial	IPI00018206	4.813	0.00045	22	18	37	39
78	Isoform alpha-enolase of Alpha-enolase	IPI00465248	4.803	0.00045	199	191	216	223
79	Isoform 1 of Nuclear pore membrane glycoprotein 210	IPI00291755	4.690	0.00045	17	14	36	29
80	T-complex protein 1 subunit eta	IPI00018465	4.642	0.00048	33	34	50	54
81	X-ray repair cross-complementing protein 5	IPI00220834	4.614	0.00048	43	43	58	66
82	60S ribosomal protein L5	IPI00000494	4.592	0.00048	3	9	15	27
83	Isoform 1 of Plectin-1	IPI00014898	4.434	0.00052	398	368	424	392
84	Eukaryotic translation initiation factor 3, subunit E interacting protein	IPI00465233	4.397	0.00052	13	12	27	29
85	ATP synthase subunit beta, mitochondrial	IPI00303476	4.380	0.00058	80	73	92	100
86	HEAT repeat-containing protein 1	IPI00024279	4.374	0.00058	31	25	50	40
87	Isoform Short of Heterogeneous nuclear ribonucleoprotein U	IPI00479217	4.335	0.00058	39	36	57	53
88	Keratin, type I cytoskeletal 19	IPI00479145	4.287	0.00058	151	147	180	160
89	Probable ATP-dependent RNA helicase DDX6	IPI00030320	4.281	0.00058	11	13	26	28
90	Aspartyl-tRNA synthetase, cytoplasmic	IPI00216951	4.268	0.00058	26	28	44	43
91	Matrin-3	IPI00017297	4.214	0.00058	24	23	40	39

No.	Description	Accession number	STN[1]	p-Value[1]	480_A[2]	480_B[2]	620_A[2]	620_B[2]
92	ATP-dependent DNA helicase Q1	IPI00178431	4.182	0.00065	27	23	42	40
93	Isoform 1 of Exportin-2	IPI00022744	4.168	0.00065	59	65	84	76
94	Ubiquitin-like modifier-activating enzyme 1	IPI00645078	4.080	0.00069	33	28	46	47
95	Vimentin	IPI00418471	4.063	0.00069	62	60	78	79
96	Heterogeneous nuclear ribonucleoprotein C-like 1	IPI00027569	4.058	0.00069	13	16	30	28
97	14-3-3 protein zeta/delta	IPI00021263	4.056	0.00069	26	24	40	41
98	transcription activator BRG1 isoform D	IPI00029822	4.042	0.00069	6	10	23	20
99	Isoform 1 of Importin-5	IPI00793443	4.026	0.00072	23	18	34	37
100	Glutathione S-transferase P	IPI00219757	4.025	0.00072	91	102	111	119
101	Isoform 2 of Nuclear mitotic apparatus protein 1	IPI00006196	4.008	0.00072	37	33	54	48
102	DNA-directed RNA polymerase II subunit RPB1	IPI00031627	3.889	0.00076	9	14	24	26
103	Isoform 1 of Heterogeneous nuclear ribonucleoprotein R	IPI00012074	3.789	0.00083	8	13	23	24
104	Endoplasmic reticulum metallopeptidase 1	IPI00257903	3.783	0.00083	0	0	15	11
105	Isoform Cytoplasmic of Lysyl-tRNA synthetase	IPI00014238	3.751	0.00083	13	10	23	26
106	Isoform 2 of Filamin-A	IPI00302592	3.748	0.00083	234	217	252	238
107	Isoform 1 of Serine/arginine repetitive matrix protein 2	IPI00782992	3.744	0.00083	23	20	35	36
108	GTP-binding nuclear protein Ran	IPI00643041	3.733	0.00083	11	13	29	21
109	Histone H1.5	IPI00217468	3.723	0.00083	21	24	34	39
110	Glutathione synthetase	IPI00010706	3.722	0.00083	6	6	18	18
111	Isoform 2 of U5 small nuclear ribonucleoprotein 200 kDa helicase	IPI00168235	3.715	0.00089	32	28	42	47
112	Heat shock protein 75 kDa, mitochondrial	IPI00030275	3.684	0.00089	13	14	25	28
113	CAD protein	IPI00301263	3.672	0.00089	68	66	93	73
114	Isoform 1 of Myosin-Ib	IPI00376344	3.640	0.00093	4	6	15	18
115	Isoform Long of Spectrin beta chain, brain 1	IPI00005614	3.625	0.00093	92	90	101	114
116	Valyl-tRNA synthetase	IPI00000873	3.625	0.00093	17	14	28	29
117	Isoform SM-B' of Small nuclear ribonucleoprotein-associated proteins B and B'	IPI00027285	3.612	0.00100	16	16	27	31
118	Isoform SERCA1B of Sarcoplasmic/endoplasmic reticulum calcium ATPase 1	IPI00024804	3.592	0.00100	6	11	21	20
119	Coronin-1B	IPI00007058	3.570	0.00100	9	9	21	21
120	TC4 protein	IPI00044779	3.565	0.00107	5	3	13	17
121	Prohibitin	IPI00017334	3.539	0.00124	36	31	56	39
122	Proliferating cell nuclear antigen	IPI00021700	3.504	0.00124	28	27	45	37
123	Malate dehydrogenase, mitochondrial	IPI00291006	3.499	0.00124	61	59	71	79
124	Phospholipase A-2-activating protein	IPI00218465	3.495	0.00124	9	6	18	20
125	Putative pre-mRNA-splicing factor ATP-dependent RNA helicase DHX15	IPI00396435	3.480	0.00124	28	30	45	40
126	Importin-7	IPI00007402	3.465	0.00124	25	20	33	38
127	Succinyl-CoA:3-ketoacid-coenzyme A transferase 1, mitochondrial	IPI00026516	3.463	0.00124	2	2	11	13
128	T-complex protein 1 subunit epsilon	IPI00010720	3.446	0.00138	24	23	36	37
129	Isoform ASF-1 of Splicing factor, arginine/serine-rich 1	IPI00215884	3.446	0.00138	22	25	34	39
130	Isoform 1 of Polypyrimidine tract-binding protein 1	IPI00179964	3.443	0.00138	36	27	47	43
131	Isoform 1 of Spectrin beta chain, brain 2	IPI00012645	3.428	0.00138	5	7	16	18
132	Pre-mRNA-splicing factor ATP-dependent RNA helicase PRP16	IPI00294211	3.400	0.00138	3	2	14	11
133	Isoform 1 of Enhancer of mRNA-decapping protein 4	IPI00376317	3.383	0.00138	14	15	28	25
134	Isoform 1 of Adenylyl cyclase-associated protein 1	IPI00008274	3.364	0.00141	19	23	37	30
135	Isoform 2 of Bromodomain adjacent to zinc finger domain protein 1A	IPI00383565	3.302	0.00165	0	2	9	14
136	Myosin-Ie	IPI00329672	3.302	0.00165	2	0	10	13
137	Calcium-binding protein 39-like	IPI00026359	3.302	0.00165	0	0	12	11
138	Isoform 1 of Enoyl-CoA hydratase domain-containing protein 1	IPI00302688	3.302	0.00165	2	0	11	12
139	Isoform 1 of Calcyclin-binding protein	IPI00395627	3.289	0.00175	13	13	26	23
140	Plastin-1	IPI00032304	3.285	0.00175	7	11	22	18
141	Barrier-to-autointegration factor	IPI00026087	3.281	0.00175	7	5	14	19
142	Probable ATP-dependent RNA helicase DDX5	IPI00017617	3.274	0.00175	20	18	31	31
143	cDNA FLJ55482, highly similar to Annexin A11	IPI00414320	3.254	0.00179	7	6	19	15
144	DNA replication licensing factor MCM5	IPI00018350	3.229	0.00179	8	6	17	18
145	Tubulin--tyrosine ligase-like protein 12	IPI00029048	3.223	0.00179	4	5	13	16
146	Electron transfer flavoprotein subunit alpha, mitochondrial	IPI00010810	3.209	0.00179	15	17	28	27
147	Putative uncharacterized protein DKFZp686L20222	IPI00026689	3.185	0.00179	19	15	30	27
148	Methionyl-tRNA synthetase, cytoplasmic	IPI00008240	3.181	0.00179	11	13	23	23
149	Protein disulfide-isomerase A3	IPI00025252	3.179	0.00179	25	23	40	32
150	Putative heat shock protein HSP 90-alpha A2	IPI00031523	3.149	0.00182	43	50	59	60
151	Histone H2B type 1-L	IPI00018534	3.141	0.00182	0	0	10	12
152	U1 small nuclear ribonucleoprotein A	IPI00012382	3.132	0.00182	6	6	15	17
153	Isoform SERCA2A of Sarcoplasmic/endoplasmic reticulum calcium ATPase 2	IPI00177817	3.124	0.00182	13	6	23	17
154	Isoform 1 of Ras-related protein Rab-1A	IPI00005719	3.120	0.00182	38	37	55	45
155	Isoform 1 of Cysteine and histidine-rich domain-containing protein 1	IPI00015897	3.106	0.00182	8	5	15	18
156	DNA polymerase alpha catalytic subunit	IPI00220317	3.106	0.00182	4	4	14	13
157	annexin A4	IPI00793199	3.074	0.00182	17	15	29	25
158	Hypoxanthine-guanine phosphoribosyltransferase	IPI00218493	3.056	0.00193	35	30	48	41
159	Multifunctional protein ADE2	IPI00217223	3.051	0.00193	19	15	28	28
160	Protein disulfide-isomerase A4	IPI00009904	3.034	0.00193	29	21	38	35
161	Dolichyl-diphosphooligosaccharide--protein glycosyltransferase subunit 1 precursor	IPI00025874	3.026	0.00200	30	21	36	38
162	Mitochondrial carrier homolog 2	IPI00003833	3.019	0.00203	17	20	30	29
163	60S ribosomal protein L3	IPI00550021	2.999	0.00203	22	17	33	28
164	Heat shock protein HSP 90-beta	IPI00414676	2.999	0.00203	65	67	74	84
165	Visinin-like protein 1	IPI00216313	2.990	0.00203	3	4	13	12
166	Adenosylhomocysteinase	IPI00012007	2.971	0.00203	44	36	47	57
167	Isoform 1 of DNA-dependent protein kinase catalytic subunit	IPI00296337	2.960	0.00203	194	188	207	205
168	C-1-tetrahydrofolate synthase, cytoplasmic	IPI00218342	2.950	0.00206	35	27	45	40
169	Poly [ADP-ribose] polymerase 1	IPI00449049	2.942	0.00206	45	41	53	57
170	Probable ATP-dependent RNA helicase DDX23	IPI00006725	2.922	0.00206	3	0	11	11
171	Elongator complex protein 1	IPI00293735	2.922	0.00206	0	3	12	10
172	60S ribosomal protein L6	IPI00329389	2.917	0.00206	16	18	29	26
173	Nascent polypeptide-associated complex subunit alpha	IPI00023748	2.888	0.00210	12	13	20	25
174	40S ribosomal protein S8	IPI00216587	2.874	0.00210	12	14	21	25
175	482 kDa protein	IPI00179298	2.858	0.00220	73	68	83	83
176	Isoform 2 of Extended synaptotagmin-2	IPI00409635	2.821	0.00227	9	11	20	19
177	Glutamate dehydrogenase 1, mitochondrial	IPI00016801	2.799	0.00234	22	25	32	36
178	60S ribosomal protein L15	IPI00470528	2.776	0.00248	11	12	18	24
179	UMP-CMP kinase isoform a	IPI00219953	2.776	0.00248	12	11	19	23
180	Eukaryotic initiation factor 4A-III	IPI00009328	2.762	0.00248	17	19	26	30
181	Isoform 1 of ATP-binding cassette sub-family B member 7, mitochondrial	IPI00306748	2.761	0.00248	3	0	12	9
182	Signal recognition particle 54 kDa protein	IPI00009822	2.747	0.00251	9	7	16	18
183	Calreticulin	IPI00020599	2.716	0.00251	32	27	39	41
184	Superkiller viralicidic activity 2-like 2	IPI00647217	2.711	0.00251	15	13	29	18
185	DNA-directed RNA polymerase I subunit RPA1	IPI00031960	2.709	0.00261	5	6	12	16
186	Isoform 1 of Serine/threonine-protein kinase WNK1	IPI00004472	2.651	0.00268	2	2	9	10

No.	Description	Accession number	STN[1]	p-Value[1]	480_A[2]	480_B[2]	620_A[2]	620_B[2]
187	Isoform 1 of HBS1-like protein	IPI00009070	2.651	0.00268	0	0	6	13
188	Isoform 1 of Transcription factor BTF3	IPI00221035	2.637	0.00272	20	15	26	28
189	40S ribosomal protein S4, X isoform	IPI00217030	2.635	0.00272	13	10	20	21
190	Estradiol 17-beta-dehydrogenase 12	IPI00007676	2.622	0.00272	13	11	22	20
191	Monocarboxylate transporter 4	IPI00006666	2.611	0.00272	2	7	11	14
192	Isoform 1 of Heterogeneous nuclear ribonucleoprotein D-like	IPI00011274	2.601	0.00272	8	8	17	16
193	T-complex protein 1 subunit delta	IPI00302927	2.601	0.00272	15	24	27	31
194	Inosine triphosphate pyrophosphatase	IPI00018783	2.599	0.00272	3	0	9	11
195	E3 ubiquitin/ISG15 ligase TRIM25	IPI00029629	2.556	0.00275	0	4	11	10
196	Small subunit processome component 20 homolog	IPI00004970	2.553	0.00275	22	23	34	30
197	U2 small nuclear ribonucleoprotein A'	IPI00297477	2.551	0.00275	14	16	24	24
198	Phosphoribosylformylglycinamidine synthase	IPI00004534	2.541	0.00282	16	15	26	23
199	116 kDa U5 small nuclear ribonucleoprotein component	IPI00003519	2.531	0.00282	24	24	36	31
200	Isoform 1 of Electron transfer flavoprotein subunit beta	IPI00004902	2.530	0.00282	18	14	24	26
201	Isoform 5 of Protein polybromo-1	IPI00023097	2.519	0.00285	4	3	10	12
202	arylacetamide deacetylase-like 1 isoform b	IPI00002230	2.512	0.00285	8	5	15	14
203	Vacuolar protein sorting-associated protein 26A	IPI00411426	2.512	0.00285	7	6	11	18
204	Guanine nucleotide-binding protein subunit beta-2-like 1	IPI00848226	2.505	0.00285	27	25	34	37
205	Nucleoprotein TPR	IPI00742682	2.502	0.00285	36	40	40	56
206	Isoform 2 of Neutral alpha-glucosidase AB	IPI00011454	2.499	0.00289	56	52	63	66
207	Isoform 1 of 60S ribosomal protein L11	IPI00376798	2.491	0.00289	6	8	16	14
208	Importin-11	IPI00301107	2.486	0.00289	0	0	7	11
209	Dihydroorotate dehydrogenase, mitochondrial	IPI00024462	2.486	0.00289	0	2	9	9
210	Isoform 3 of Oxidation resistance protein 1	IPI00166807	2.486	0.00289	0	0	9	9
211	Collapsin response mediator protein 4 long variant	IPI00029111	2.486	0.00296	5	3	10	13
212	Small nuclear ribonucleoprotein E	IPI00029266	2.486	0.00296	4	4	12	11
213	Acetyl-CoA acetyltransferase, mitochondrial	IPI00030363	2.476	0.00296	19	19	31	25
214	60S ribosomal protein L7a	IPI00299573	2.468	0.00296	12	13	24	18
215	Heterogeneous nuclear ribonucleoprotein U-like protein 2	IPI00456887	2.468	0.00296	12	13	20	22
216	Nucleosome assembly protein 1-like 1	IPI00023860	2.457	0.00296	15	11	19	24
217	Isoform 1 of Myosin-10	IPI00397526	2.436	0.00299	19	24	32	29
218	DNA-directed RNA polymerase II subunit RPB2	IPI00027808	2.421	0.00306	8	10	17	17
219	Eukaryotic translation initiation factor 3 subunit A	IPI00029012	2.418	0.00306	52	45	57	60
220	DNA replication licensing factor MCM4	IPI00018349	2.401	0.00320	23	25	32	34
221	ATP-binding cassette sub-family D member 1	IPI00291373	2.395	0.00327	0	4	10	10
222	Isoform 2 of Spliceosome RNA helicase BAT1	IPI00641829	2.394	0.00334	16	16	23	26
223	Peptidyl-prolyl cis-trans isomerase FKBP3	IPI00024157	2.391	0.00334	10	10	19	17
224	60S ribosomal protein L9	IPI00031691	2.375	0.00334	19	15	25	26
225	Rho GDP-dissociation inhibitor 1	IPI00003815	2.366	0.00334	21	14	27	25
226	60S ribosomal protein L23	IPI00010153	2.352	0.00344	9	14	14	25
227	Isoform 1 of Peripherin	IPI00013164	2.352	0.00344	10	13	23	16
228	Protein RRP5 homolog	IPI00400922	2.350	0.00344	20	17	23	31
229	Isoform Beta of Lamina-associated polypeptide 2, isoforms beta/gamma	IPI00030131	2.350	0.00344	18	19	26	28
230	Ubiquitin carboxyl-terminal hydrolase 7	IPI00003965	2.339	0.00344	12	12	19	21
231	Phosphoglycerate kinase 1	IPI00169383	2.330	0.00347	42	46	52	55
232	WD40 repeat-containing protein SMU1	IPI00305833	2.328	0.00347	4	4	8	14
233	Putative myosin-XVB	IPI00786880	2.320	0.00361	0	0	6	11
234	Heat shock 70 kDa protein 12A	IPI00011932	2.320	0.00361	0	2	9	8
235	Core histone macro-H2A.2	IPI00220994	2.320	0.00361	2	0	10	7
236	Transmembrane protein 165	IPI00307572	2.320	0.00361	0	0	8	9
237	Isoform 2 of Oxidation resistance protein 1	IPI00298348	2.320	0.00361	0	0	7	10
238	Isoform 2 of 6-phosphofructokinase, muscle type	IPI00219585	2.300	0.00371	5	4	9	14
239	cDNA FLJ59758, highly similar to S-methyl-5-thioadenosine phosphorylase	IPI00011876	2.300	0.00371	5	4	13	10
240	Ubiquitin-like modifier activating enzyme 1	IPI00552452	2.291	0.00382	10	7	17	15
241	Aldehyde dehydrogenase, mitochondrial	IPI00006663	2.275	0.00385	17	13	24	22
242	Quinone oxidoreductase	IPI00000792	2.273	0.00389	3	0	9	9
243	40S ribosomal protein S6	IPI00021840	2.252	0.00396	6	5	12	13
244	Paladin	IPI00297212	2.252	0.00396	5	6	11	14
245	Phenylalanyl-tRNA synthetase beta chain	IPI00300074	2.252	0.00396	3	8	14	11
246	60S acidic ribosomal protein P0	IPI00008530	2.243	0.00396	42	37	53	44
247	Isoform 1 of Medium-chain specific acyl-CoA dehydrogenase, mitochondrial	IPI00005040	2.234	0.00396	3	3	10	9
248	AP-1 complex subunit mu-1	IPI00032516	2.200	0.00430	3	4	10	10
249	40S ribosomal protein S2	IPI00013485	2.199	0.00430	22	17	26	29
250	Prohibitin-2	IPI00027252	2.174	0.00440	30	36	40	43
251	Isoform 2 of Serine/threonine-protein phosphatase PGAM5, mitochondrial	IPI00063242	2.171	0.00440	5	3	9	12
252	Isoform Beta-4C of Integrin beta-4	IPI00027422	2.161	0.00444	34	35	45	41
253	probable ubiquitin carboxyl-terminal hydrolase FAF-X isoform 4	IPI00003964	2.159	0.00444	3	13	13	17
254	Isoform 1 of Transportin-1	IPI00024364	2.159	0.00444	9	7	15	15
255	Isoform 1 of Ubiquitin-like modifier-activating enzyme 6	IPI00023647	2.159	0.00444	8	8	16	14
256	Leucyl-tRNA synthetase, cytoplasmic	IPI00103994	2.158	0.00444	25	20	32	29
257	Growth arrest and DNA damage-inducible proteins-interacting protein 1	IPI00552587	2.152	0.00454	2	0	7	9
258	Isoform 2 of Formin-like protein 1	IPI00025202	2.152	0.00454	0	2	7	9
259	Isoform 1 of Roundabout homolog 1	IPI00219798	2.152	0.00454	0	0	9	7
260	Isoform Long of Delta-1-pyrroline-5-carboxylate synthase	IPI00008982	2.146	0.00461	13	16	20	24
261	Ubiquitin-conjugating enzyme E2 L3	IPI00021347	2.144	0.00461	5	4	14	8
262	Nucleolar GTP-binding protein 1	IPI00385042	2.144	0.00461	3	6	11	11
263	Isoform 1 of Splicing factor, arginine/serine-rich 7	IPI00003377	2.115	0.00471	10	9	18	15
264	magnesium transporter protein 1	IPI00301202	2.108	0.00471	0	3	7	10
265	Probable methylthioribulose-1-phosphate dehydratase	IPI00549730	2.108	0.00471	3	2	8	9
266	Phenylalanyl-tRNA synthetase alpha chain	IPI00031820	2.098	0.00478	8	3	10	14
267	cDNA FLJ55599, highly similar to DNA replication licensing factor MCM3	IPI00013214	2.095	0.00478	18	17	24	26
268	Isoform 2 of Eukaryotic translation initiation factor 5A-1	IPI00376005	2.079	0.00478	20	17	30	22
269	E3 ubiquitin-protein ligase UBR5	IPI00026320	2.078	0.00478	5	7	14	11
270	Isoform 2 of Voltage-dependent anion-selective channel protein 2	IPI00024145	2.078	0.00478	12	10	19	17
271	Solute carrier family 4 sodium bicarbonate cotransporter member 7	IPI00021058	2.071	0.00482	2	4	11	7
272	Early endosome antigen 1	IPI00329536	2.066	0.00492	12	11	15	22
273	Isoform 1 of 60S ribosomal protein L12	IPI00024933	2.059	0.00499	9	4	12	14
274	Insulin-degrading enzyme	IPI00220373	2.059	0.00499	7	6	13	13
275	Sorbitol dehydrogenase	IPI00216057	2.059	0.00499	7	6	12	14
276	40S ribosomal protein S15	IPI00479058	2.055	0.00499	15	9	21	17
277	Tripeptidyl-peptidase 2	IPI00020416	2.045	0.00499	13	12	20	19
278	Ubiquitin carboxyl-terminal hydrolase 24	IPI00902614	2.040	0.00499	3	4	12	7
279	TRIP12 protein	IPI00032342	2.025	0.00506	9	6	14	14
280	60S ribosomal protein L23a	IPI00021266	2.025	0.00506	14	13	19	22
281	probable E3 ubiquitin-protein ligase MYCBP2	IPI00289776	2.012	0.00519	4	4	8	12

No.	Description	Accession number	STN[1]	p-Value[1]	480_A[2]	480_B[2]	620_A[2]	620_B[2]
282	cDNA FLJ56825, highly similar to WD repeat protein 57	IPI00006723	2.012	0.00519	4	4	11	9
283	Protein RCC2	IPI00465044	2.012	0.00519	3	5	8	12
284	Putative uncharacterized protein KIAA0090	IPI00640734	1.984	0.00519	2	0	9	6
285	Transmembrane 9 superfamily member 2	IPI00018415	1.984	0.00519	2	2	8	7
286	Cell division protein kinase 6	IPI00023529	1.984	0.00519	0	0	8	7
287	Isoform 1 of Cleavage and polyadenylation specificity factor subunit 6	IPI00012998	1.964	0.00526	5	5	13	9
288	Isoform 2 of ATP-binding cassette sub-family F member 1	IPI00013495	1.964	0.00526	4	6	11	11
289	Enoyl-CoA hydratase, mitochondrial	IPI00024993	1.964	0.00526	5	5	9	13
290	Lupus La protein	IPI00009032	1.958	0.00543	16	19	24	25
291	Isoform 1 of Heterogeneous nuclear ribonucleoprotein M	IPI00171903	1.957	0.00543	30	28	33	40
292	Peroxiredoxin-4	IPI00011937	1.957	0.00543	10	10	17	16
293	Isoform 1 of CCR4-NOT transcription complex subunit 1	IPI00166010	1.945	0.00547	9	12	16	18
294	Isoform 1 of Cirhin	IPI00239815	1.943	0.00547	5	6	12	11
295	Condensin-2 complex subunit D3	IPI00747787	1.943	0.00547	5	6	13	10
296	Estradiol 17-beta-dehydrogenase 11	IPI00329598	1.943	0.00547	2	3	8	8
297	Isoform 2 of ATPase family AAA domain-containing protein 3A	IPI00295992	1.943	0.00547	3	0	8	8
298	TOB3	IPI00045921	1.943	0.00547	2	3	9	7
299	X-ray repair cross-complementing protein 6	IPI00644712	1.937	0.00547	50	48	56	58
300	tRNA (cytosine-5-)-methyltransferase NSUN2	IPI00306369	1.923	0.00585	10	13	21	15
301	Isoform 3 of Ribosome-binding protein 1	IPI00215743	1.913	0.00585	12	12	19	18
302	Cell division protein kinase 5	IPI00023530	1.908	0.00595	4	2	10	7
303	THO complex subunit 2	IPI00158615	1.907	0.00595	6	7	11	14
304	Delta(3,5)-Delta(2,4)-dienoyl-CoA isomerase, mitochondrial	IPI00011416	1.899	0.00595	20	24	28	30
305	Isoform 1 of Carnitine O-palmitoyltransferase 1, liver isoform	IPI00032038	1.899	0.00595	16	28	30	28
306	Isoform 1 of 3-hydroxyacyl-CoA dehydrogenase type-2	IPI00017726	1.888	0.00599	38	37	46	44
307	Isoform 2 of SWI/SNF complex subunit SMARCC2	IPI00150057	1.884	0.00599	13	14	22	18
308	Glycyl-tRNA synthetase	IPI00783097	1.884	0.00599	17	10	20	20
309	Bifunctional ATP-dependent dihydroxyacetone kinase/FAD-AMP lyase (cyclizing)	IPI00551024	1.878	0.00602	3	4	8	10
310	Isoform 1 of Exosome component 10	IPI00009464	1.878	0.00602	2	5	8	10
311	Palmitoyl-protein thioesterase 1	IPI00002412	1.878	0.00602	4	3	10	8
312	poly(rC) binding protein 2 isoform b	IPI00012066	1.871	0.00609	24	25	32	31
313	Isoform 2 of Ubiquinol-cytochrome c reductase complex chaperone CBP3 homolog	IPI00219889	1.852	0.00623	0	6	9	10
314	ATP synthase subunit alpha, mitochondrial	IPI00440493	1.851	0.00623	43	43	43	58
315	Ataxin-10	IPI00001636	1.847	0.00623	9	8	17	12
316	Isoform 5 of Interleukin enhancer-binding factor 3	IPI00219330	1.837	0.00623	24	32	34	36
317	Proliferation-associated protein 2G4	IPI00299000	1.835	0.00626	16	17	28	18
318	Histone H2A.V	IPI00018278	1.833	0.00633	49	43	54	53
319	Isoform 1 of BRCA2 and CDKN1A-interacting protein	IPI00002203	1.828	0.00633	3	6	11	9
320	59 kDa protein	IPI00302925	1.824	0.00633	29	30	38	35
321	Isoform 1 of Heterogeneous nuclear ribonucleoprotein H3	IPI00013877	1.821	0.00633	16	19	22	26
322	60S acidic ribosomal protein P2	IPI00008529	1.821	0.00633	20	15	24	24
323	Isoform 4 of Uncharacterized protein KIAA0090	IPI00642244	1.814	0.00633	0	0	7	7
324	GDP-mannose 4,6 dehydratase	IPI00030207	1.814	0.00633	2	0	6	8
325	Aladin	IPI00024143	1.814	0.00633	0	2	7	7
326	4-hydroxyphenylpyruvate dioxygenase-like protein	IPI00063762	1.814	0.00633	2	2	8	6
327	Epidermal growth factor receptor kinase substrate 8	IPI00290337	1.814	0.00633	0	0	5	9
328	Aspartyl/asparaginyl beta-hydroxylase	IPI00294834	1.814	0.00633	0	2	6	8
329	dCTP pyrophosphatase 1	IPI00012197	1.814	0.00633	2	0	7	7
330	Serine/threonine-protein kinase PRP4 homolog	IPI00013721	1.814	0.00633	0	0	8	6
331	Ladinin-1	IPI00514234	1.814	0.00633	0	2	7	7
332	Neprilysin	IPI00247063	1.814	0.00633	0	0	5	9
333	cDNA FLJ59211, highly similar to Glucosidase 2 subunit beta	IPI00026154	1.811	0.00643	11	9	15	17
334	Putative rRNA methyltransferase 3	IPI00217686	1.811	0.00643	9	11	18	14
335	treacle protein isoform a	IPI00165041	1.800	0.00654	12	9	15	18
336	Splicing factor 3B subunit 1	IPI00026089	1.792	0.00657	35	32	39	42
337	Isoform Long of Trifunctional purine biosynthetic protein adenosine-3	IPI00025273	1.777	0.00709	20	22	27	28
338	Gamma-aminobutyric acid receptor-associated protein-like 2	IPI00026358	1.776	0.00709	0	3	8	7
339	UDP-glucose 6-dehydrogenase	IPI00031420	1.769	0.00726	11	13	17	19
340	Isoform 1 of Nuclear pore complex protein Nup155	IPI00026625	1.760	0.00743	14	11	20	17
341	Large neutral amino acids transporter small subunit 1	IPI00008986	1.754	0.00746	7	6	10	14
342	Isoform 1 of Protein strawberry notch homolog 1	IPI00023649	1.743	0.00746	4	2	9	7
343	ADP-sugar pyrophosphatase	IPI00296913	1.743	0.00798	13	14	20	19
344	Poly(rC)-binding protein 1	IPI00016610	1.724	0.00819	8	7	16	10
345	Isoform 1 of Far upstream element-binding protein 1	IPI00375441	1.724	0.00819	9	6	15	11
346	Isoform D of Constitutive coactivator of PPAR-gamma-like protein 1	IPI00039626	1.724	0.00819	9	6	13	13
347	splicing factor 3B subunit 2	IPI00221106	1.719	0.00819	21	9	25	17
348	Isoform 1 of General transcription factor 3C polypeptide 1	IPI00414482	1.715	0.00826	2	5	6	11
349	Plastin-3	IPI00216694	1.715	0.00826	3	4	5	12
350	Isoform 1 of Transformer-2 protein homolog beta	IPI00301503	1.711	0.00826	6	10	15	12
351	39S ribosomal protein L13, mitochondrial	IPI00022403	1.691	0.00846	4	4	8	10
352	Transmembrane protein 2	IPI00170706	1.691	0.00846	4	4	8	10
353	GTP-binding protein SAR1a	IPI00015954	1.691	0.00846	3	5	10	8
354	40S ribosomal protein S5	IPI00008433	1.686	0.00856	8	10	18	11
355	DKFZP586J0619 protein	IPI00740961	1.686	0.00856	8	10	16	13
356	Transducin beta-like protein 3	IPI00477971	1.686	0.00856	7	11	13	16
357	Nuclear migration protein nudC	IPI00550746	1.686	0.00856	10	8	16	13
358	Dihydropyrimidinase-like 2	IPI00106642	1.675	0.00860	13	6	14	16
359	Isoform 2 of Basigin	IPI00019906	1.675	0.00860	10	9	16	14
360	Isoform 2 of Cat eye syndrome critical region protein 5	IPI00011511	1.669	0.00860	6	3	12	7
361	TDP43	IPI00025815	1.669	0.00860	4	5	10	9
362	Dihydrolipoyllysine-residue acetyltransferase component of pyruvate dehydrogenase complex	IPI00021338	1.650	0.00881	5	5	9	11
363	Isoform 1 of Eukaryotic translation initiation factor 3 subunit B	IPI00396370	1.648	0.00887	21	20	28	25
364	Probable cysteinyl-tRNA synthetase, mitochondrial	IPI00336016	1.643	0.00887	0	2	7	6
365	Isoform 1 of Protein timeless homolog	IPI00335541	1.643	0.00887	0	2	6	7
366	Very long-chain acyl-CoA synthetase	IPI00024787	1.643	0.00887	0	0	5	8
367	Major vault protein	IPI00000105	1.643	0.00887	0	0	6	7
368	Isoform NELF-C of Negative elongation factor C/D	IPI00164949	1.643	0.00887	0	0	5	8
369	Uncharacterized protein C19orf21	IPI00217121	1.643	0.00887	2	2	7	6
370	Galectin-3-binding protein	IPI00023673	1.643	0.00887	0	0	6	7
371	Isoform 1 of Rho GTPase-activating protein 18	IPI00296353	1.643	0.00887	0	0	5	8
372	Isoform 1 of Secretory carrier-associated membrane protein 3	IPI00306382	1.643	0.00887	0	0	9	4
373	Isoform 1 of U5 small nuclear ribonucleoprotein 200 kDa helicase	IPI00420014	1.632	0.00901	103	100	120	98
374	Stress-induced-phosphoprotein 1	IPI00013894	1.632	0.00901	24	20	30	26
375	cDNA FLJ45706 fis, clone FEBRA2028457, highly similar to Nucleolin	IPI00444262	1.632	0.00901	22	22	31	25
376	Phosphatidylethanolamine-binding protein 1	IPI00219446	1.626	0.00912	13	11	19	16

No.	Description	Accession number	STN[1]	p-Value[1]	480_A[2]	480_B[2]	620_A[2]	620_B[2]
377	von Hippel-Lindau binding protein 1, isoform CRA_b	IPI00334159	1.609	0.00953	16	10	20	17
378	Vacuolar protein-sorting-associated protein 25	IPI00031655	1.607	0.00953	3	2	6	8
379	DNA replication licensing factor MCM2	IPI00184330	1.599	0.00960	28	23	36	27
380	Peroxiredoxin-1	IPI00000874	1.596	0.00960	47	43	57	46
381	pyrroline-5-carboxylate reductase 1, mitochondrial isoform 2	IPI00376503	1.577	0.00977	3	3	8	7
382	Isoform 1 of Core-binding factor subunit beta	IPI00016746	1.560	0.00991	10	6	13	13
383	Mitochondrial 2-oxoglutarate/malate carrier protein	IPI00219729	1.559	0.01018	17	16	22	22
384	DNA mismatch repair protein Msh2	IPI00017303	1.552	0.01018	14	20	21	24
385	Dolichyl-diphosphooligosaccharide--protein glycosyltransferase subunit STT3A	IPI00297492	1.552	0.01018	2	5	8	8
386	cDNA FLJ55586, highly similar to MMS19-like protein	IPI00154451	1.552	0.01018	5	2	8	8
387	Polyribonucleotide nucleotidyltransferase 1, mitochondrial	IPI00744711	1.552	0.01018	4	3	10	6
388	Histone-binding protein RBBP4	IPI00328319	1.548	0.01018	9	8	12	15
389	Tyrosyl-tRNA synthetase, cytoplasmic	IPI00007074	1.548	0.01018	7	10	13	14
390	Putative uncharacterized protein SPTAN1	IPI00745092	1.546	0.01018	16	19	23	23
391	UPF0027 protein C22orf28	IPI00550689	1.540	0.01018	18	18	24	23
392	Putative deoxyribose-phosphate aldolase	IPI00219677	1.529	0.01056	4	4	9	8
393	Isoform 1 of RNA-binding protein Musashi homolog 2	IPI00073713	1.529	0.01056	4	4	8	9
394	Ran GTPase-activating protein 1	IPI00294879	1.529	0.01056	5	3	8	9
395	Isoform 1 of Lipopolysaccharide-responsive and beige-like anchor protein	IPI00002255	1.527	0.01056	9	10	15	14
396	Splicing factor, arginine/serine-rich 9	IPI00012340	1.527	0.01056	10	9	12	17
397	Thioredoxin-dependent peroxide reductase, mitochondrial	IPI00024919	1.527	0.01056	10	9	12	17
398	Cytochrome c oxidase subunit 2	IPI00017510	1.527	0.01056	8	11	15	14
399	Protein phosphatase 1G	IPI00006167	1.527	0.01056	10	9	11	18
400	cytochrome b5 type B precursor	IPI00303954	1.517	0.01066	12	8	14	16
401	Kinesin-like protein KIF11	IPI00305289	1.509	0.01087	4	5	9	9
402	Moesin	IPI00219365	1.498	0.01090	22	22	24	31
403	Isoform 1 of Polyadenylate-binding protein 1	IPI00008524	1.494	0.01125	22	23	30	26
404	Isoform 1AB of Catenin delta-1	IPI00182469	1.491	0.01125	6	4	8	11
405	High mobility group protein B2	IPI00219097	1.491	0.01125	0	8	10	9
406	Lactoylglutathione lyase	IPI00220766	1.481	0.01132	12	12	18	16
407	Inositol monophosphatase 1	IPI00020906	1.474	0.01139	6	5	8	12
408	cDNA FLJ56307, highly similar to Ubiquitin thioesterase protein OTUB1	IPI00000581	1.473	0.01139	12	13	18	17
409	Isoform 1 of Coatomer subunit alpha	IPI00295857	1.472	0.01139	43	48	54	49
410	CAAX prenyl protease 1 homolog	IPI00027180	1.470	0.01139	0	0	6	6
411	60S ribosomal protein L7-like 1	IPI00456940	1.470	0.01139	2	2	7	5
412	cDNA FLJ53927, highly similar to Beta-hexosaminidase alpha chain	IPI00027851	1.470	0.01139	2	2	6	6
413	Long-chain-fatty-acid--CoA ligase 3	IPI00031397	1.470	0.01139	0	0	5	7
414	Isoform 1 of Nucleolar protein 14	IPI00022613	1.470	0.01139	0	0	6	6
415	39S ribosomal protein L15, mitochondrial	IPI00023086	1.470	0.01139	0	2	3	9
416	74 kDa protein	IPI00290439	1.470	0.01139	0	2	7	5
417	Eukaryotic peptide chain release factor GTP-binding subunit ERF3A	IPI00218829	1.470	0.01139	2	0	5	7
418	Telomeric repeat-binding factor 2-interacting protein 1	IPI00008961	1.470	0.01139	0	2	8	4
419	Pterin-4-alpha-carbinolamine dehydratase	IPI00218568	1.470	0.01139	2	0	6	6
420	Calponin-3	IPI00216682	1.470	0.01139	0	0	7	5
421	Probable phosphoglycerate mutase 4	IPI00374975	1.459	0.01280	7	5	9	12
422	Isoform 1 of Malignant T cell-amplified sequence 1	IPI00179026	1.459	0.01280	7	5	12	9
423	Spermidine synthase	IPI00292020	1.459	0.01280	7	5	11	10
424	Peptidyl-prolyl cis-trans isomerase A	IPI00419585	1.459	0.01280	48	49	56	53
425	Interleukin enhancer-binding factor 2	IPI00005198	1.452	0.01280	27	28	33	33
426	Isoform 1 of Putative helicase MOV-10	IPI00444452	1.445	0.01300	5	8	10	12
427	Peptidyl-prolyl cis-trans isomerase FKBP4	IPI00219005	1.445	0.01300	29	28	33	35
428	WD repeat-containing protein 3	IPI00009471	1.438	0.01307	2	3	6	7
429	Small nuclear ribonucleoprotein F	IPI00220528	1.438	0.01307	3	0	7	6
430	Isoform 3 of Chitinase domain-containing protein 1	IPI00045536	1.438	0.01307	3	2	7	6
431	Tubulin-specific chaperone E	IPI00018402	1.438	0.01307	2	3	5	8
432	10 kDa heat shock protein, mitochondrial	IPI00220362	1.420	0.01314	8	7	10	14
433	Importin 5	IPI00514205	1.420	0.01314	8	7	10	14
434	Beta-hexosaminidase subunit beta	IPI00012585	1.410	0.01328	4	2	6	8
435	Methyltransferase like 7B	IPI00090807	1.410	0.01328	0	4	8	6
436	Isoform 1 of Chromodomain-helicase-DNA-binding protein 1	IPI00297851	1.410	0.01328	0	4	9	5
437	Isoform 1 of SEC23-interacting protein	IPI00026969	1.410	0.01328	4	2	8	6
438	ATP-dependent RNA helicase DDX50	IPI00031554	1.410	0.01328	3	3	8	6
439	Translin-associated protein X	IPI00293350	1.408	0.01397	11	5	13	12
440	Isoform 1 of RNA-binding protein 8A	IPI00001757	1.408	0.01397	8	8	14	11
441	6-phosphogluconolactonase	IPI00029997	1.408	0.01397	9	7	13	12
442	Chloride intracellular channel protein 1	IPI00010896	1.408	0.01397	18	17	25	20
443	Isoform A of AP-1 complex subunit beta-1	IPI00328257	1.397	0.01441	18	19	24	23
444	Ras-related protein Rab-10	IPI00016513	1.387	0.01462	7	11	15	12
445	vacuolar protein sorting-associated protein 13C isoform 2B	IPI00412216	1.387	0.01462	4	3	7	8
446	Mitochondrial 28S ribosomal protein S2	IPI00006970	1.387	0.01462	3	4	7	8
447	26S proteasome non-ATPase regulatory subunit 10	IPI00003565	1.387	0.01462	4	3	7	8
448	Isoform 1 of Replication factor C subunit 1	IPI00375358	1.387	0.01462	4	3	8	7
449	Periodic tryptophan protein 2 homolog	IPI00300078	1.378	0.01479	8	11	14	14
450	Isoform 2 of Exosome complex exonuclease RRP44	IPI00183462	1.378	0.01479	10	9	13	15
451	Isoform 1 of Poly(U)-binding-splicing factor PUF60	IPI00069750	1.378	0.01479	9	10	15	13
452	Laminin receptor-like protein LAMRL5	IPI00411639	1.378	0.01479	22	19	27	24
453	28 kDa heat- and acid-stable phosphoprotein	IPI00013297	1.369	0.01496	10	10	16	13
454	L-xylulose reductase	IPI00448095	1.366	0.01496	3	5	9	7
455	Brefeldin A-inhibited guanine nucleotide-exchange protein 2	IPI00002186	1.366	0.01496	5	3	8	8
456	Isoform 1 of Cell division cycle and apoptosis regulator protein 1	IPI00217357	1.366	0.01496	4	4	7	9
457	Isoform 1 of General transcription factor II-I	IPI00054042	1.360	0.01551	24	21	28	27
458	WD repeat-containing protein 36	IPI00169325	1.352	0.01555	12	10	18	13
459	ADP/ATP translocase 1	IPI00022891	1.348	0.01558	4	5	8	9
460	COP9 signalosome complex subunit 4	IPI00171844	1.348	0.01558	4	5	8	9
461	Isoform 1 of 5'-3' exoribonuclease 2	IPI00100151	1.336	0.01565	13	11	17	16
462	Isoform 1 of Cullin-associated NEDD8-dissociated protein 1	IPI00100160	1.332	0.01565	29	23	29	33
463	U3 small nucleolar ribonucleoprotein protein IMP3	IPI00019488	1.331	0.01565	7	3	10	8
464	ubiquitin and ribosomal protein S27a precursor	IPI00179330	1.328	0.01644	42	60	58	55
465	Mitochondrial ribonuclease P protein 1	IPI00099996	1.316	0.01661	5	6	11	8
466	Rho-associated protein kinase 2	IPI00307155	1.315	0.01661	14	13	17	19
467	Isoform 2 of Signal recognition particle 68 kDa protein	IPI00102936	1.302	0.01675	6	6	8	12
468	Isoform 1 of Tyrosine-protein kinase BAZ1B	IPI00069817	1.297	0.01737	16	14	18	21
469	Eukaryotic translation initiation factor 6	IPI00010105	1.297	0.01737	16	14	18	21
470	ATP-dependent RNA helicase DDX3X	IPI00215637	1.297	0.01737	17	13	20	19
471	Growth hormone inducible transmembrane protein	IPI00549970	1.296	0.01747	2	0	0	9

No.	Description	Accession number	STN[1]	p-Value[1]	480_A[2]	480_B[2]	620_A[2]	620_B[2]
472	Vitamin K epoxide reductase complex subunit 1-like protein 1	IPI00166079	1.296	0.01747	2	0	6	5
473	Isoform 1 of ATPase family AAA domain-containing protein 1	IPI00171445	1.296	0.01747	2	2	4	7
474	Isoform 2 of Chromodomain-helicase-DNA-binding protein 2	IPI00023109	1.296	0.01747	2	2	5	6
475	E3 ubiquitin-protein ligase HECTD1	IPI00328911	1.296	0.01747	0	2	3	8
476	Selenide, water dikinase 1	IPI00029056	1.296	0.01747	2	0	6	5
477	Molybdopterin synthase catalytic subunit	IPI00005218	1.296	0.01747	2	2	5	6
478	Uncharacterized protein KIAA1797	IPI00748360	1.296	0.01747	0	0	7	4
479	Isoform 1 of Pogo transposable element with ZNF domain	IPI00410717	1.296	0.01747	2	2	6	5
480	Transient receptor potential cation channel subfamily V member 2	IPI00183666	1.296	0.01747	0	0	4	7
481	Isoform 1 of Helicase-like transcription factor	IPI00339381	1.296	0.01747	0	0	7	4
482	Rho GDP-dissociation inhibitor 2	IPI00003817	1.296	0.01747	0	0	5	6
483	Exportin-T	IPI00306290	1.289	0.01758	6	7	12	9
484	Glutathione S-transferase kappa 1	IPI00219673	1.285	0.01758	16	16	21	20
485	Isoform 2 of Triosephosphate isomerase	IPI00451401	1.277	0.01813	37	33	41	39
486	Threonyl-tRNA synthetase, cytoplasmic	IPI00329633	1.266	0.01823	8	7	11	12
487	Protein DEK	IPI00020021	1.266	0.01823	8	7	12	11
488	Isoform Short of Adenosine kinase	IPI00234368	1.266	0.01823	3	2	7	5
489	Kinetochore-associated protein 1	IPI00001458	1.266	0.01823	2	3	5	7
490	Parafibromin	IPI00300659	1.266	0.01823	3	2	5	7
491	Isoform 2 of Endoplasmic reticulum aminopeptidase 1	IPI00165949	1.266	0.01823	3	0	5	7
492	Charged multivesicular body protein 5	IPI00100796	1.266	0.01823	3	2	7	5
493	cDNA FLJ54492, highly similar to Eukaryotic translation initiation factor 4B	IPI00012079	1.256	0.01826	8	8	13	11
494	Isoform Long of Splicing factor, proline- and glutamine-rich	IPI00010740	1.255	0.01864	22	16	24	23
495	CSNK2A1 protein	IPI00016613	1.246	0.01875	10	7	11	14
496	Isoform 1 of Cytosol aminopeptidase	IPI00419237	1.246	0.01875	8	9	13	12
497	Methylenetetrahydrofolate dehydrogenase (NADP+ dependent) 1-like	IPI00291646	1.246	0.01875	9	8	12	13
498	UV excision repair protein RAD23 homolog B	IPI00008223	1.241	0.01878	3	3	7	6
499	Ribosome biogenesis protein WDR12	IPI00304232	1.241	0.01878	4	2	7	6
500	Pre-mRNA-splicing factor SPF27	IPI00025178	1.241	0.01878	3	3	7	6
501	Isoform 1 of Specifically androgen-regulated gene protein	IPI00028392	1.241	0.01878	4	2	6	7
502	Isoform GTBP-alt of DNA mismatch repair protein Msh6	IPI00106847	1.238	0.01895	22	20	26	25
503	DNA-(apurinic or apyrimidinic site) lyase	IPI00215911	1.237	0.01895	10	8	11	15
504	Isoform 3 of Splicing factor, arginine/serine-rich 13A	IPI00009071	1.237	0.01895	9	9	14	12
505	Isoform 1 of 39S ribosomal protein L22, mitochondrial	IPI00414410	1.237	0.01895	9	9	15	11
506	Protein NipSnap homolog 2	IPI00016077	1.228	0.01930	10	9	15	12
507	EH domain-containing protein 4	IPI00005578	1.228	0.01930	8	11	12	15
508	Cullin-1	IPI00014310	1.220	0.01940	2	5	8	6
509	Alkyldihydroxyacetonephosphate synthase, peroxisomal	IPI00010349	1.220	0.01940	3	4	9	5
510	39S ribosomal protein L1, mitochondrial	IPI00549381	1.220	0.01940	2	5	6	8
511	Isoform 1 of Ribose-phosphate pyrophosphokinase 2	IPI00219617	1.220	0.01940	3	4	7	7
512	Gamma-tubulin complex component 2	IPI00029705	1.220	0.01940	4	3	7	7
513	Isoform 1 of Replication factor C subunit 2	IPI00017412	1.220	0.01940	11	9	17	11
514	Isoform 3 of Adenylate kinase 2, mitochondrial	IPI00172460	1.212	0.01974	11	10	16	13
515	SERPINE1 mRNA binding protein 1, isoform CRA_d	IPI00410693	1.201	0.02040	4	4	9	6
516	COP9 signalosome complex subunit 5	IPI00009958	1.201	0.02040	5	3	6	9
517	Isoform DPI of Desmoplakin	IPI00013933	1.198	0.02054	60	48	56	62
518	Condensin complex subunit 3	IPI00106495	1.198	0.02054	13	10	15	16
519	Isoform 1 of WD repeat-containing protein 1	IPI00746165	1.191	0.02071	12	12	13	19
520	Isoform 1 of Dynamin-2	IPI00033022	1.191	0.02071	12	12	16	16
521	Isoform 1 of Bcl-2-associated transcription factor 1	IPI00006079	1.185	0.02074	4	5	8	8
522	Probable ATP-dependent RNA helicase DDX10	IPI00297900	1.185	0.02074	4	5	5	11
523	Transaldolase	IPI00744692	1.182	0.02074	33	25	39	28
524	Isoform 2 of Inverted formin-2	IPI00876962	1.178	0.02074	12	14	17	17
525	cDNA FLJ60124, highly similar to Mitochondrial dicarboxylate carrier	IPI00005537	1.170	0.02105	3	7	5	12
526	Cold-inducible RNA-binding protein	IPI00180954	1.170	0.02105	6	4	10	7
527	Methylosome subunit pICln	IPI00004795	1.170	0.02105	5	5	10	7
528	40S ribosomal protein S10	IPI00008438	1.166	0.02119	11	17	17	19
529	Activated RNA polymerase II transcriptional coactivator p15	IPI00221222	1.156	0.02157	5	6	9	9
530	Isoform 2 of Mitochondrial import inner membrane translocase subunit TIM50	IPI00418497	1.156	0.02157	5	6	8	10
531	Lamin-B receptor	IPI00292135	1.144	0.02219	6	6	10	9
532	Isoform 1 of Cleavage and polyadenylation specificity factor subunit 7	IPI00550821	1.132	0.02250	6	7	10	10
533	Bifunctional aminoacyl-tRNA synthetase	IPI00013452	1.124	0.02263	38	44	46	45
534	Pre-mRNA-processing factor 19	IPI00004968	1.122	0.02263	8	6	11	10
535	40S ribosomal protein S15a	IPI00221091	1.122	0.02263	8	6	11	10
536	Anaphase-promoting complex subunit 1	IPI00033907	1.120	0.02263	0	0	6	4
537	Mediator of RNA polymerase II transcription subunit 12	IPI00004068	1.120	0.02263	2	0	6	4
538	PH-interacting protein	IPI00291916	1.120	0.02263	0	2	5	5
539	Isoform 1 of Cytochrome c oxidase assembly protein COX15 homolog	IPI00419869	1.120	0.02263	0	0	3	7
540	Isoform 1 of Cleavage stimulation factor subunit 2	IPI00013256	1.120	0.02263	0	0	5	5
541	Isoform 1 of Solute carrier family 12 member 2	IPI00022649	1.120	0.02263	2	0	5	5
542	Diphosphoinositol polyphosphate phosphohydrolase 1	IPI00009148	1.120	0.02263	0	0	6	4
543	poly [ADP-ribose] polymerase 14	IPI00291215	1.120	0.02263	0	2	5	5
544	Similar to Protein SAAL1. Isoform 2	IPI00304935	1.120	0.02263	0	0	5	5
545	Isoform 1 of Polyadenylate-binding protein 4	IPI00012726	1.120	0.02263	0	0	5	5
546	Isoform 1 of Protein FAM115A	IPI00006050	1.120	0.02263	0	0	8	0
547	Bifunctional methylenetetrahydrofolate dehydrogenase/cyclohydrolase, mitochondrial	IPI00011307	1.120	0.02263	2	2	4	6
548	Isoform 1 of Cullin-3	IPI00014312	1.120	0.02263	2	2	6	4
549	Isoform 1 of Phosphatidylinositol-3,4,5-trisphosphate 5-phosphatase 1	IPI00329213	1.120	0.02263	0	0	5	5
550	Isoform SMN of Survival motor neuron protein	IPI00003394	1.120	0.02263	0	0	6	4
551	Ribosomal RNA processing protein 1 homolog A	IPI00550766	1.120	0.02263	2	0	7	3
552	1-acyl-sn-glycerol-3-phosphate acyltransferase epsilon	IPI00028491	1.120	0.02263	0	0	5	5
553	Isoform 1 of tRNA guanosine-2'-O-methyltransferase TRM11 homolog	IPI00470606	1.120	0.02263	0	0	4	6
554	Synaptotagmin-1	IPI00009439	1.120	0.02263	0	0	3	7
555	SH3 domain-binding glutamic acid-rich-like protein	IPI00025318	1.120	0.02263	0	0	5	5
556	6-phosphogluconate dehydrogenase, decarboxylating	IPI00219525	1.106	0.02532	22	19	27	22
557	Isoform 1 of ATPase family AAA domain-containing protein 2	IPI00170548	1.103	0.02532	7	9	12	11
558	Src substrate cortactin	IPI00029601	1.103	0.02532	7	9	9	14
559	Proteasome subunit alpha type-5	IPI00291922	1.095	0.02549	20	24	25	27
560	Isoform 1 of 14-3-3 protein sigma	IPI00013890	1.094	0.02552	8	9	12	12
561	Isoform 1 of ER lumen protein retaining receptor 2	IPI00018248	1.093	0.02552	3	0	4	7
562	39S ribosomal protein L28, mitochondrial	IPI00172594	1.093	0.02552	3	2	6	5
563	Isoform 1 of 60S ribosome subunit biogenesis protein NIP7 homolog	IPI00007175	1.093	0.02552	2	3	6	5
564	Transmembrane protein 126A	IPI00031064	1.093	0.02552	3	2	6	5
565	Isoform 1 of Ubiquitin-protein ligase E3C	IPI00604464	1.093	0.02552	2	3	3	8
566	Peptidyl-prolyl cis-trans isomerase FKBP5	IPI00218775	1.093	0.02552	2	3	6	5

No.	Description	Accession number	STN[1]	p-Value[1]	480_A[2]	480_B[2]	620_A[2]	620_B[2]
567	Ras-related protein Rab-5B	IPI00017344	1.093	0.02552	2	3	6	5
568	Heat shock 70 kDa protein 14	IPI00292499	1.093	0.02552	3	0	5	6
569	Puromycin-sensitive aminopeptidase	IPI00026216	1.091	0.02559	23	22	25	28
570	40S ribosomal protein S7	IPI00013415	1.086	0.02566	8	10	11	14
571	Isoform Gamma-1 of Serine/threonine-protein phosphatase PP1-gamma catalytic subunit	IPI00005705	1.084	0.02566	21	26	32	23
572	Ribosome biogenesis protein BMS1 homolog	IPI00006099	1.078	0.02583	10	9	13	13
573	Aminoacyl tRNA synthase complex-interacting multifunctional protein 2	IPI00011916	1.071	0.02625	3	3	5	7
574	cDNA FLJ14048 fis, clone HEMBA1006650, weakly similar to ARP2/3 COMPLEX 20 KD SUBUNIT	IPI00386354	1.071	0.02625	2	4	5	7
575	Interferon-induced, double-stranded RNA-activated protein kinase	IPI00019463	1.071	0.02625	2	4	5	7
576	Protein tyrosine phosphatase-like protein PTPLAD1	IPI00008998	1.071	0.02779	9	11	14	13
577	Isoform 1 of ATP-dependent RNA helicase DDX42	IPI00409671	1.071	0.02779	10	10	13	14
578	Isoform 2 of Transportin-3	IPI00395694	1.071	0.02779	8	12	13	14
579	Calpain-2 catalytic subunit	IPI00289758	1.064	0.02779	10	11	13	15
580	Isoform 2 of DNA replication licensing factor MCM7	IPI00219740	1.064	0.02779	10	11	15	13
581	Isoform 1 of F-actin-capping protein subunit beta	IPI00026185	1.057	0.02786	13	9	16	13
582	Isoform 1 of Pleiotropic regulator 1	IPI00002624	1.052	0.02786	3	4	7	6
583	Isoform 1 of Fanconi anemia group D2 protein	IPI00075081	1.052	0.02786	4	3	6	7
584	Isoform 1 of Zinc finger Ran-binding domain-containing protein 2	IPI00029400	1.052	0.02786	0	5	3	10
585	Isoform 1 of Fatty aldehyde dehydrogenase	IPI00333619	1.052	0.02786	2	5	5	8
586	Replication factor C subunit 5	IPI00031514	1.051	0.02790	10	13	17	13
587	Voltage-dependent anion-selective channel protein 1	IPI00216308	1.044	0.02790	70	65	83	61
588	Coatomer subunit gamma	IPI00783982	1.039	0.02814	14	11	18	14
589	Isoform 2 of Dedicator of cytokinesis protein 7	IPI00183572	1.036	0.02814	3	5	6	8
590	Isoform 1 of Methyl-CpG-binding domain protein 3	IPI00439194	1.036	0.02814	5	3	7	7
591	cDNA FLJ60299, highly similar to Rab GDP dissociation inhibitor beta	IPI00031461	1.035	0.02917	31	34	31	42
592	Isoform 1 of Leukotriene A-4 hydrolase	IPI00219077	1.033	0.02917	16	10	16	17
593	40S ribosomal protein S14	IPI00026271	1.028	0.02938	14	13	20	14
594	Isoform 1 of UPF0424 protein C1orf128	IPI00015351	1.021	0.02944	4	5	8	7
595	Isoform 1 of ATP-binding cassette sub-family D member 3	IPI00002372	1.021	0.02944	5	4	4	11
596	cohesin subunit SA-2 isoform a	IPI00470883	1.021	0.02944	4	5	8	7
597	Isoform 1 of 3'(2'),5'-bisphosphate nucleotidase 1	IPI00410214	1.021	0.02944	5	4	8	7
598	Short heat shock protein 60 Hsp60s2	IPI00076042	1.018	0.02951	17	12	19	17
599	Isoform Short of RNA-binding protein FUS	IPI00221354	1.008	0.02958	5	5	8	8
600	Isoform 2 of Glutaminase kidney isoform, mitochondrial	IPI00215685	1.008	0.02958	5	5	9	7
601	Transcription initiation factor IIB	IPI00022820	1.008	0.02958	5	5	8	8
602	Isoform Long of Ubiquitin carboxyl-terminal hydrolase 5	IPI00024664	0.999	0.03106	14	19	21	19
603	Tubulin-specific chaperone A	IPI00217236	0.996	0.03113	5	6	7	10
604	Isoform Long of Cold shock domain-containing protein E1	IPI00470891	0.996	0.03113	7	4	10	7
605	Isocitrate dehydrogenase 3, beta subunit isoform a precursor	IPI00304417	0.985	0.03127	8	4	5	13
606	Putative ATP-dependent Clp protease proteolytic subunit, mitochondrial	IPI00003870	0.985	0.03127	7	5	10	8
607	Histone acetyltransferase type B catalytic subunit	IPI00024719	0.985	0.03127	5	7	11	7
608	Histone deacetylase 1	IPI00013774	0.985	0.03127	6	6	8	10
609	Monocarboxylate transporter 1	IPI00024650	0.975	0.03237	3	10	9	10
610	Eukaryotic translation initiation factor 1A, Y-chromosomal	IPI00023004	0.975	0.03237	8	5	12	7
611	60S ribosomal protein L21	IPI00247583	0.975	0.03237	7	6	10	9
612	Dual specificity mitogen-activated protein kinase kinase 2	IPI00003783	0.975	0.03237	6	7	7	12
613	U4/U6.U5 tri-snRNP-associated protein 1	IPI00021417	0.975	0.03237	7	6	9	10
614	ERO1-like protein alpha	IPI00386755	0.975	0.03237	8	5	9	10
615	Putative uncharacterized protein MDH1	IPI00915869	0.975	0.03237	7	6	9	10
616	28S ribosomal protein S26, mitochondrial	IPI00006606	0.975	0.03237	7	6	11	8
617	Isoform 1 of Heterogeneous nuclear ribonucleoprotein Q	IPI00018140	0.972	0.03237	20	20	27	20
618	Exocyst complex component 4	IPI00059279	0.965	0.03251	6	8	9	11
619	DnaJ homolog subfamily A member 1	IPI00012535	0.965	0.03251	5	9	11	9
620	Isoform 1 of Hydroxyacyl-coenzyme A dehydrogenase, mitochondrial	IPI00294398	0.957	0.03292	8	7	12	9
621	Isoform Mitochondrial of Glutathione reductase, mitochondrial	IPI00016862	0.948	0.03299	10	6	11	11
622	29 kDa protein	IPI00453476	0.948	0.03299	10	6	12	10
623	Isoform Long of Deoxyhypusine synthase	IPI00026829	0.948	0.03299	6	10	9	13
624	Dipeptidyl peptidase 1	IPI00022810	0.941	0.03416	0	0	4	5
625	Isoform Long of Inositol 1,4,5-trisphosphate receptor type 2	IPI00031545	0.941	0.03416	0	2	4	5
626	Isoform 1 of KH domain-containing, RNA-binding, signal transduction-associated protein 1	IPI00008575	0.941	0.03416	0	0	5	4
627	Thioredoxin-related transmembrane protein 1	IPI00395887	0.941	0.03416	2	0	5	4
628	Isoform 1 of Sorting nexin-12	IPI00438170	0.941	0.03416	2	0	5	4
629	RNA-binding protein 12	IPI00550308	0.941	0.03416	2	2	4	5
630	Brefeldin A-inhibited guanine nucleotide-exchange protein 1	IPI00002188	0.941	0.03416	0	2	4	5
631	Protein VAC14 homolog	IPI00025160	0.941	0.03416	0	0	5	4
632	Isoform 1 of Disco-interacting protein 2 homolog B	IPI00465045	0.941	0.03416	0	0	4	5
633	Adenylosuccinate synthetase isozyme 2	IPI00026833	0.941	0.03416	0	2	4	5
634	LanC-like protein 2	IPI00032995	0.941	0.03416	2	2	4	5
635	Isoform 2 of Golgi apparatus protein 1	IPI00414717	0.941	0.03416	2	2	6	3
636	Isoform 1 of Pre-mRNA-splicing factor 38A	IPI00171390	0.941	0.03416	2	2	4	5
637	Protein phosphatase 1F	IPI00291412	0.941	0.03416	0	0	5	4
638	Isoform 2 of Serine-protein kinase ATM	IPI00289986	0.941	0.03416	0	0	7	2
639	Isoform 1 of Coiled-coil domain-containing protein 47	IPI00024642	0.941	0.03416	2	2	4	5
640	NADH-ubiquinone oxidoreductase chain 5	IPI00008511	0.941	0.03416	0	0	3	6
641	Isoform 3 of Fermitin family homolog 1	IPI00220602	0.941	0.03416	0	0	0	7
642	Centromere/kinetochore protein zw10 homolog	IPI00011631	0.941	0.03416	0	0	3	6
643	Claudin-1	IPI00000691	0.941	0.03416	2	2	5	4
644	RNA-binding protein NOB1	IPI00022373	0.941	0.03416	0	0	4	5
645	71 kDa protein	IPI00062599	0.941	0.03416	2	0	5	4
646	Mitochondrial import inner membrane translocase subunit Tim13	IPI00001589	0.941	0.03416	0	0	5	4
647	Isoform 1 of Dehydrogenase/reductase SDR family member 7	IPI00006957	0.941	0.03416	0	0	3	6
648	DCN1-like protein 5	IPI00165361	0.941	0.03426	8	9	13	10
649	Putative uncharacterized protein NAP1L4	IPI00017763	0.941	0.03426	8	9	13	10
650	Probable ATP-dependent RNA helicase DDX47	IPI00023972	0.941	0.03426	8	9	12	11
651	cDNA FLJ55177, highly similar to Ras-related protein Ral-B	IPI00004397	0.941	0.03426	8	9	12	11
652	Actin-related protein 2/3 complex subunit 4	IPI00554811	0.934	0.03436	10	8	16	8
653	Isoform 1 of Proteasome activator complex subunit 4	IPI00005260	0.934	0.03436	8	10	12	12
654	60S ribosomal protein L24	IPI00306332	0.934	0.03436	9	9	13	11
655	Heterogeneous nuclear ribonucleoprotein L	IPI00027834	0.931	0.03488	28	26	33	28
656	Isoform 1 of Heterogeneous nuclear ribonucleoprotein A3	IPI00419373	0.929	0.03488	29	26	32	30
657	Isoform 1 of 40S ribosomal protein S24	IPI00029750	0.927	0.03488	10	9	12	13
658	Isoform 1 of Host cell factor 1	IPI00019848	0.927	0.03488	8	11	9	16
659	Isoform 1 of Splicing factor U2AF 65 kDa subunit	IPI00031556	0.927	0.03488	7	12	12	13
660	Isoform 1 of DNA replication licensing factor MCM7	IPI00299904	0.927	0.03488	11	8	15	10
661	Isoform 1 of Deoxyuridine 5'-triphosphate nucleotidohydrolase, mitochondrial	IPI00013679	0.927	0.03488	10	9	10	15

No.	Description	Accession number	STN[1]	p-Value[1]	480_A[2]	480_B[2]	620_A[2]	620_B[2]
662	Putative uncharacterized protein RPL17	IPI00394699	0.920	0.03491	11	9	13	13
663	Hepatoma-derived growth factor	IPI00020956	0.920	0.03491	9	11	12	14
664	Isoform Epsilon of Apoptosis regulator BAX	IPI00071059	0.920	0.03491	10	10	15	11
665	1,4-alpha-glucan-branching enzyme	IPI00296635	0.918	0.03557	0	3	5	5
666	Isoform 2 of Uncharacterized protein C3orf63	IPI00745978	0.918	0.03557	2	3	4	6
667	Lamina-associated polypeptide 2, isoform alpha	IPI00216230	0.918	0.03557	3	2	5	5
668	Isoform CSBP2 of Mitogen-activated protein kinase 14	IPI00002857	0.918	0.03557	0	3	4	6
669	Isoform 1 of Spermine synthase	IPI00005102	0.918	0.03557	2	3	5	5
670	C-terminal-binding protein 1	IPI00012835	0.918	0.03557	3	2	3	7
671	Isoform 1 of C-terminal-binding protein 2	IPI00010120	0.918	0.03557	2	3	6	4
672	ATP synthase subunit b, mitochondrial	IPI00029133	0.908	0.03567	10	12	13	15
673	Ubiquitin carboxyl-terminal hydrolase isozyme L3	IPI00011250	0.908	0.03567	11	11	16	12
674	Isoform Beta of Nucleolar and coiled-body phosphoprotein 1	IPI00216654	0.899	0.03619	0	4	4	7
675	Proteasome inhibitor PI31 subunit	IPI00009949	0.899	0.03619	4	2	5	6
676	Ribosome biogenesis protein BRX1 homolog	IPI00181728	0.899	0.03619	3	3	5	6
677	Isoform A of Nucleoporin SEH1	IPI00185533	0.899	0.03619	3	3	5	6
678	39S ribosomal protein L19, mitochondrial	IPI00027096	0.899	0.03619	4	0	5	6
679	Isoform 2 of Lysine-specific histone demethylase 1A	IPI00217540	0.899	0.03619	2	4	5	6
680	Isoform 2 of COP9 signalosome complex subunit 2	IPI00018813	0.899	0.03619	3	3	5	6
681	WD repeat and HMG-box DNA-binding protein 1	IPI00411614	0.899	0.03619	2	4	4	7
682	Immunoglobulin-binding protein 1	IPI00019148	0.899	0.03619	2	4	5	6
683	Isoform 1 of EH domain-binding protein 1	IPI00178187	0.899	0.03619	2	4	7	4
684	Metastasis-associated protein MTA2	IPI00171798	0.899	0.03619	4	2	6	5
685	Maspardin	IPI00010248	0.899	0.03619	3	3	3	8
686	40S ribosomal protein S18	IPI00013296	0.892	0.03698	14	11	16	15
687	Transmembrane emp24 domain-containing protein 10	IPI00028055	0.888	0.03698	11	15	16	16
688	Radixin, isoform CRA_a	IPI00017367	0.888	0.03698	14	12	16	16
689	TATA-binding protein-associated factor 172	IPI00024802	0.882	0.03767	4	3	7	5
690	Isoform 2 of Tyrosine-protein phosphatase non-receptor type 11	IPI00298347	0.882	0.03767	3	4	6	6
691	Pseudouridylate synthase 7 homolog	IPI00044761	0.882	0.03767	3	4	5	7
692	ATP-binding cassette sub-family F member 2	IPI00005045	0.882	0.03767	4	3	5	7
693	Isoform 2 of tRNA pseudouridine synthase A	IPI00001716	0.882	0.03767	2	5	7	5
694	Peptidyl-prolyl cis-trans isomerase F, mitochondrial	IPI00026519	0.882	0.03767	5	0	6	6
695	Isoform 1 of Prolyl 4-hydroxylase subunit alpha-1	IPI00009923	0.882	0.03767	3	4	7	5
696	Midasin	IPI00167941	0.870	0.03828	18	12	17	19
697	Uncharacterized protein C17orf25	IPI00007102	0.868	0.03873	5	3	6	7
698	Putative uncharacterized protein ENSP00000350479	IPI00069693	0.868	0.03873	4	4	6	7
699	Bystin	IPI00328987	0.868	0.03873	2	6	8	5
700	F-actin-capping protein subunit alpha-2	IPI00026182	0.868	0.03873	4	4	8	5
701	Isoform 1 of Annexin A7	IPI00002460	0.868	0.03873	4	4	6	7
702	Isoform 1 of Apoptosis-inducing factor 1, mitochondrial	IPI00000690	0.862	0.03873	16	16	20	18
703	NADH dehydrogenase [ubiquinone] 1 alpha subcomplex subunit 9, mitochondrial	IPI00003968	0.858	0.03908	15	18	18	21
704	triosephosphate isomerase 1 isoform 2	IPI00465028	0.855	0.03908	5	4	7	7
705	NADH dehydrogenase [ubiquinone] 1 alpha subcomplex subunit 10, mitochondrial	IPI00029561	0.855	0.03908	6	3	8	6
706	Scavenger mRNA-decapping enzyme DcpS	IPI00335385	0.855	0.03908	5	4	6	8
707	sorting nexin-1 isoform c	IPI00183274	0.855	0.03908	5	4	9	5
708	DnaJ homolog subfamily C member 9	IPI00154975	0.855	0.03908	4	5	8	6
709	Isoform 1 of Elongation factor 1-delta	IPI00023048	0.844	0.03963	23	14	20	23
710	Isoform 1 of Cleft lip and palate transmembrane protein 1-like proteir	IPI00151358	0.844	0.03963	4	6	6	9
711	Isoform 1 of 6-phosphofructokinase, liver type	IPI00332371	0.844	0.03963	6	4	7	8
712	Isoform 3 of Probable ATP-dependent RNA helicase DDX17	IPI00651653	0.844	0.03963	4	6	8	7
713	cDNA FLJ59367, highly similar to Adenylosuccinate lyase	IPI00026904	0.844	0.03963	5	5	9	6
714	Isoform A of Peptidyl-prolyl cis-trans isomerase E	IPI00009316	0.844	0.03963	6	4	8	7
715	histone deacetylase complex subunit SAP18	IPI00011698	0.844	0.03963	4	6	8	7
716	Putative RNA-binding protein 3	IPI00024320	0.844	0.03963	5	5	8	7
717	L-aminoadipate-semialdehyde dehydrogenase-phosphopantetheinyl transferase	IPI00250297	0.844	0.03963	4	6	9	6
718	Cytoplasmic dynein 1 heavy chain 1	IPI00456969	0.842	0.03966	291	266	279	287
719	40S ribosomal protein S9	IPI00221088	0.838	0.04000	20	19	23	22
720	DNA replication licensing factor MCM6	IPI00031517	0.835	0.04000	20	20	21	25
721	Eukaryotic translation initiation factor 3 subunit D	IPI00006181	0.834	0.04007	6	5	8	8
722	NADH dehydrogenase [ubiquinone] iron-sulfur protein 7, mitochondrial	IPI00307749	0.834	0.04007	8	3	8	8
723	UPF0368 protein Cxorf26	IPI00107104	0.834	0.04007	5	6	8	8
724	Sorting and assembly machinery component 50 homolog	IPI00412713	0.834	0.04007	7	4	8	8
725	Isoform 1 of Proteasome activator complex subunit 3	IPI00030243	0.826	0.04062	25	18	28	21
726	Isoform 2 of Cytoplasmic FMR1-interacting protein 1	IPI00550212	0.824	0.04066	6	6	8	9
727	Golgi-specific brefeldin A-resistance guanine nucleotide exchange factor 1	IPI00021954	0.824	0.04066	4	8	9	8
728	Isoform 2 of Serrate RNA effector molecule homolog	IPI00220038	0.824	0.04066	5	7	8	9
729	Eukaryotic translation elongation factor 1 epsilon-1	IPI00003588	0.824	0.04066	7	5	9	8
730	Protein dpy-30 homolog	IPI00028109	0.824	0.04066	8	4	9	8
731	Microsomal glutathione S-transferase 3	IPI00024266	0.824	0.04066	8	4	8	9
732	Protein C20orf11	IPI00016634	0.824	0.04066	6	6	10	7
733	Transcription factor A, mitochondrial	IPI00020928	0.816	0.04166	8	5	9	9
734	26S proteasome non-ATPase regulatory subunit 12	IPI00185374	0.808	0.04203	27	23	25	31
735	Isoform 1 of 5'(3')-deoxyribonucleotidase, cytosolic type	IPI00005573	0.808	0.04210	6	8	10	9
736	Ras-related protein Rab-1B	IPI00008964	0.808	0.04210	7	7	7	12
737	DNA ligase 1	IPI00219841	0.808	0.04210	5	9	10	9
738	Glutathione S-transferase omega-1	IPI00019755	0.800	0.04231	8	7	12	8
739	Similar to Signal peptidase complex subunit 2	IPI00452747	0.800	0.04231	7	8	10	10
740	Aldehyde dehydrogenase X, mitochondrial	IPI00103467	0.800	0.04231	6	9	9	11
741	Lysophospholipid acyltransferase 5	IPI00306419	0.800	0.04231	10	5	10	10
742	Isoform 1 of Alpha-aminoadipic semialdehyde dehydrogenase	IPI00221234	0.787	0.04313	8	9	11	11
743	Annexin A3	IPI00024095	0.783	0.04334	31	31	38	30
744	Isoform 4 of Tubulin-specific chaperone D	IPI00030774	0.781	0.04341	11	7	10	13
745	AP-1 complex subunit gamma-1 isoform a	IPI00293396	0.781	0.04341	8	10	10	13
746	SWI/SNF-related matrix-associated actin-dependent regulator of chromatin subfamily A member 5	IPI00297211	0.775	0.04382	6	13	11	13
747	Cytochrome c oxidase subunit 4 isoform 1, mitochondrial	IPI00006579	0.775	0.04382	8	11	12	12
748	Replication factor C subunit 4	IPI00017381	0.775	0.04382	10	9	10	14
749	Ras-related protein Rap-1b	IPI00015148	0.769	0.04389	8	12	14	11
750	ATP-dependent RNA helicase DDX18	IPI00301323	0.764	0.04427	12	9	16	10
751	Peptidyl-prolyl cis-trans isomerase FKBP11	IPI00009885	0.760	0.04454	2	0	3	5
752	protein transport protein Sec61 subunit alpha isoform 2 isoform b	IPI00182313	0.760	0.04454	0	0	0	6
753	Programmed cell death protein 6	IPI00025277	0.760	0.04454	0	2	4	4
754	Isoform 3 of Rapamycin-insensitive companion of mTOR	IPI00166528	0.760	0.04454	2	2	4	4
755	Coiled-coil-helix-coiled-coil-helix domain-containing protein 2, mitochondrial	IPI00007673	0.760	0.04454	0	0	0	6
756	Mimitin, mitochondrial	IPI00031109	0.760	0.04454	0	0	4	4

No.	Description	Accession number	STN[1]	p-Value[1]	480_A[2]	480_B[2]	620_A[2]	620_B[2]
757	28S ribosomal protein S25, mitochondrial	IPI00013167	0.760	0.04454	2	2	3	5
758	Isoform 1 of Golgin subfamily A member 3	IPI00305267	0.760	0.04454	2	2	2	6
759	Histone H1.0	IPI00550239	0.760	0.04454	0	0	0	6
760	Isoform 2 of Integrator complex subunit 3	IPI00418336	0.760	0.04454	2	2	3	5
761	cDNA FLJ60317, highly similar to Aminoacylase-1	IPI00009268	0.760	0.04454	0	2	5	3
762	Mitochondrial import receptor subunit TOM20 homolog	IPI00016676	0.760	0.04454	0	0	6	0
763	Phosphatidylinositide phosphatase SAC1	IPI00022275	0.760	0.04454	0	0	2	6
764	Thiosulfate sulfurtransferase	IPI00216293	0.760	0.04454	0	2	4	4
765	G-rich sequence factor 1	IPI00478657	0.760	0.04454	0	0	4	4
766	Isoform 1 of Citron Rho-interacting kinase	IPI00022465	0.760	0.04454	0	0	4	4
767	Isoform 1 of Regulator of microtubule dynamics protein 3	IPI00410079	0.760	0.04454	0	0	4	4
768	Isoform 1 of Gamma-tubulin complex component 3	IPI00033516	0.760	0.04454	2	2	4	4
769	Ubiquitin-conjugating enzyme E2 S	IPI00217949	0.760	0.04454	2	2	4	4
770	cDNA FLJ56468, highly similar to Kynurenine--oxoglutarate transaminase 1	IPI00002523	0.760	0.04454	0	2	3	5
771	Isoform 1 of Phosphatidylinositol glycan anchor biosynthesis class U protein	IPI00026044	0.760	0.04454	0	0	4	4
772	Kinesin-like protein KIF13B	IPI00021753	0.760	0.04454	0	0	3	5
773	NEDD8-activating enzyme E1 regulatory subunit	IPI00018968	0.760	0.04454	2	2	3	5
774	Catechol O-methyltransferase domain-containing protein 1	IPI00642041	0.760	0.04454	0	0	3	5
775	Isoform 1 of GTP-binding protein 10	IPI00167638	0.760	0.04454	0	0	6	2
776	Isoform 1 of Phosphatidate cytidylyltransferase 2	IPI00032150	0.760	0.04454	2	0	4	4
777	Isoform 1 of Ras GTPase-activating protein 1	IPI00026262	0.760	0.04454	2	2	3	5
778	BRO1 domain-containing protein BROX	IPI00065500	0.760	0.04454	2	2	3	5
779	ATP-dependent Clp protease ATP-binding subunit clpX-like, mitochondrial	IPI00008728	0.760	0.04454	0	0	4	4
780	cDNA FLJ78567	IPI00043678	0.760	0.04454	0	0	4	4
781	39S ribosomal protein L24, mitochondrial	IPI00514506	0.760	0.04454	2	0	3	5
782	28S ribosomal protein S36, mitochondrial	IPI00020495	0.760	0.04454	2	0	4	4
783	Isoform 2 of DNA-3-methyladenine glycosylase	IPI00218495	0.760	0.04454	2	0	4	4
784	Protein S100-A16	IPI00062120	0.760	0.04454	0	0	4	4
785	OTU domain-containing protein 6B	IPI00182180	0.760	0.04454	2	0	4	4
786	Isoform 1 of Peroxisomal membrane protein PEX14	IPI00025346	0.760	0.04454	0	0	5	3
787	2,4-dienoyl-CoA reductase, mitochondrial	IPI00003482	0.760	0.04454	0	0	3	5
788	Ethanolamine kinase 1	IPI00030090	0.760	0.04454	0	0	3	5
789	Coiled-coil domain-containing protein 6	IPI00000634	0.760	0.04454	0	0	0	6
790	Cob(I)yrinic acid a,c-diamide adenosyltransferase, mitochondrial	IPI00029665	0.760	0.04454	0	0	5	3
791	Isoform 1 of MYC-induced nuclear antigen	IPI00216737	0.760	0.04454	0	0	4	4
792	Ubiquilin-2	IPI00409659	0.760	0.04454	0	0	4	4
793	Isoform 1 of Required for meiotic nuclear division protein 1 homolog	IPI00329591	0.760	0.04454	0	0	3	5
794	Replication initiator 1	IPI00549171	0.760	0.04454	0	0	4	4
795	NEDD4-like E3 ubiquitin-protein ligase WWP2	IPI00013010	0.760	0.04454	0	0	4	4
796	Putative uncharacterized protein INF2	IPI00872508	0.759	0.05259	12	10	14	13
797	Isoform 1 of Dipeptidyl peptidase 3	IPI00020672	0.750	0.05325	11	13	14	15
798	Vacuolar protein sorting-associated protein 35	IPI00018931	0.750	0.05325	13	11	14	15
799	Calponin-2	IPI00015262	0.746	0.05325	12	13	15	15
800	ATP-dependent RNA helicase DDX1	IPI00293655	0.746	0.05325	13	12	14	16
801	Elongation factor 1-beta	IPI00178440	0.746	0.05325	13	12	12	18
802	60S ribosomal protein L8	IPI00012772	0.741	0.05366	3	0	2	7
803	Isoform 2 of Nipped-B-like protein	IPI00026466	0.741	0.05366	2	3	5	4
804	Ubiquitin-fold modifier-conjugating enzyme 1	IPI00294495	0.741	0.05366	2	3	3	6
805	Casein kinase II subunit beta	IPI00010865	0.741	0.05366	3	0	4	5
806	Protein MEMO1	IPI00032426	0.741	0.05366	3	0	3	6
807	Phosphoglycerate mutase 2	IPI00218570	0.741	0.05366	3	2	5	4
808	cDNA FLJ61739, highly similar to Serine/arginine repetitive matrix protein 1	IPI00328293	0.741	0.05366	3	0	5	4
809	ATPase ASNA1	IPI00013466	0.741	0.05366	3	2	5	4
810	Guanine nucleotide-binding protein G(k) subunit alpha	IPI00220578	0.741	0.05366	2	3	6	3
811	Probable saccharopine dehydrogenase	IPI00329600	0.741	0.05366	3	2	4	5
812	Isoform 1 of Apoptosis-inducing factor 2	IPI00013909	0.741	0.05366	3	2	6	3
813	39S ribosomal protein L40, mitochondrial	IPI00099871	0.741	0.05366	2	3	5	4
814	Protein transport protein Sec23B	IPI00017376	0.741	0.05366	0	3	4	5
815	Phosphoglucomutase-2	IPI00550364	0.741	0.05366	0	3	4	5
816	Isoform 1 of Luc7-like protein 3	IPI00107745	0.741	0.05366	2	3	4	5
817	Histone H1x	IPI00021924	0.741	0.05366	0	3	5	4
818	Nuclease-sensitive element-binding protein 1	IPI00031812	0.738	0.05369	14	13	15	17
819	Nucleolar protein 58	IPI00006379	0.734	0.05394	15	13	17	16
820	Isoform 1 of Fanconi anemia group I protein	IPI00019447	0.727	0.05421	12	18	19	16
821	Isoform 1 of DNA (cytosine-5)-methyltransferase 1	IPI00031519	0.727	0.05421	13	17	16	19
822	Isoform 2 of Mediator of DNA damage checkpoint protein 1	IPI00470805	0.725	0.05421	0	4	2	8
823	Isoform 1 of Proteasome assembly chaperone 1	IPI00030770	0.725	0.05421	4	2	4	6
824	Isoform A of Ras GTPase-activating protein-binding protein 2	IPI00009057	0.725	0.05421	2	4	5	5
825	Activity-dependent neuroprotector homeobox protein	IPI00022215	0.725	0.05421	2	4	5	5
826	Cullin-5	IPI00216003	0.725	0.05421	4	2	5	5
827	Isoform 2 of WASH complex subunit FAM21C	IPI00456853	0.725	0.05421	2	4	6	4
828	Isoform 1 of Acyl-CoA-binding protein	IPI00010182	0.725	0.05421	3	3	5	5
829	YrdC domain-containing protein, mitochondrial	IPI00384180	0.725	0.05421	3	3	6	4
830	Isoform 1 of Lymphoid-specific helicase	IPI00010590	0.725	0.05421	0	4	3	7
831	Isoform 1 of CUGBP Elav-like family member 1	IPI00034015	0.725	0.05421	3	3	7	3
832	myosin-IXb isoform 1	IPI00306933	0.711	0.05744	4	3	4	7
833	Cell division protein kinase 2	IPI00031681	0.711	0.05744	4	3	6	5
834	Isoform 2 of Peptidyl-prolyl cis-trans isomerase NIMA-interacting 4	IPI00006658	0.711	0.05744	4	3	6	5
835	Cleavage and polyadenylation specificity factor subunit 2	IPI00419531	0.711	0.05744	4	3	5	6
836	Osteoclast-stimulating factor 1	IPI00414836	0.711	0.05744	4	3	6	5
837	Putative uncharacterized protein	IPI00260769	0.711	0.05744	3	4	6	5
838	Isoform 1 of Rho guanine nucleotide exchange factor 2	IPI00291316	0.711	0.05744	3	4	5	6
839	Hippocalcin-like protein 1	IPI00219344	0.711	0.05744	2	5	6	5
840	Density-regulated protein	IPI00306280	0.711	0.05744	4	3	5	6
841	Putative uncharacterized protein KIAA0664	IPI00024425	0.702	0.05765	17	21	23	20
842	Lon protease homolog, mitochondrial	IPI00005158	0.699	0.05779	20	19	22	22
843	MLL1/MLL complex subunit C17orf49 isoform 1	IPI00373869	0.699	0.05786	2	6	8	4
844	Isoform 3 of Protein transport protein Sec31A	IPI00305152	0.699	0.05786	0	6	6	6
845	Coiled-coil domain-containing protein 25	IPI00396174	0.699	0.05786	4	4	5	7
846	Isoform 1 of CAP-Gly domain-containing linker protein 2	IPI00019642	0.699	0.05786	2	6	7	5
847	40S ribosomal protein S21	IPI00017448	0.699	0.05786	4	4	4	8
848	Tyrosyl-tRNA synthetase, mitochondrial	IPI00165092	0.699	0.05786	4	4	5	7
849	Sec1 family domain-containing protein 1	IPI00165261	0.699	0.05786	5	3	5	7
850	Putative uncharacterized protein DKFZp781K1356	IPI00412545	0.699	0.05786	4	4	7	5
851	HDCMD34P	IPI00001672	0.699	0.05786	3	5	6	6

No.	Description	Accession number	STN[1]	p-Value[1]	480_A[2]	480_B[2]	620_A[2]	620_B[2]
852	Isoform 1 of Protein SET	IPI00072377	0.694	0.06157	19	22	25	21
853	Platelet-activating factor acetylhydrolase IB subunit gamma	IPI00014808	0.688	0.06202	5	4	5	8
854	Prostaglandin E synthase 3	IPI00015029	0.688	0.06202	7	0	6	7
855	Isoform 2 of Ubiquitin conjugation factor E4 A	IPI00028957	0.688	0.06202	3	6	6	7
856	S-adenosylmethionine synthase isoform type-2	IPI00010157	0.688	0.06202	4	5	7	6
857	Isoform 3 of PCI domain-containing protein 2	IPI00072541	0.688	0.06202	5	4	7	6
858	26 kDa protein	IPI00219685	0.688	0.06202	5	4	6	7
859	Isoform A of Uncharacterized protein C21orf70	IPI00027898	0.688	0.06202	4	5	6	7
860	Isoform 1 of Glucosamine--fructose-6-phosphate aminotransferase [isomerizing] 1	IPI00217952	0.688	0.06202	5	4	7	6
861	mRNA turnover protein 4 homolog	IPI00106491	0.679	0.06226	5	5	6	8
862	Actin-related protein 2/3 complex subunit 3	IPI00005162	0.679	0.06226	6	4	6	8
863	Platelet-activating factor acetylhydrolase IB subunit beta	IPI00026546	0.679	0.06226	5	5	6	8
864	Mitochondrial import receptor subunit TOM34	IPI00009946	0.679	0.06226	3	7	7	7
865	Isoform 1 of COP9 signalosome complex subunit 7b	IPI00009301	0.679	0.06226	6	4	8	6
866	ADP-ribosylation factor-like protein 3	IPI00003327	0.679	0.06226	6	4	8	6
867	ADP-ribosylation factor-like protein 2	IPI00003326	0.679	0.06226	6	4	8	6
868	Coatomer subunit beta	IPI00295851	0.671	0.06594	25	27	30	27
869	SUMO-conjugating enzyme UBC9	IPI00032957	0.670	0.06594	6	5	7	8
870	Isoform Heart of ATP synthase subunit gamma, mitochondrial	IPI00395769	0.670	0.06594	7	4	8	7
871	Isoform 2 of Ubiquitin carboxyl-terminal hydrolase 47	IPI00165528	0.670	0.06594	4	7	7	8
872	Dihydropteridine reductase	IPI00014439	0.670	0.06594	5	6	9	6
873	Acidic leucine-rich nuclear phosphoprotein 32 family member E	IPI00165393	0.670	0.06594	6	5	7	8
874	Developmentally-regulated GTP-binding protein 2	IPI00022697	0.670	0.06594	7	4	7	8
875	Isoform 1 of Extended synaptotagmin-1	IPI00022143	0.665	0.06608	29	26	32	28
876	FACT complex subunit SSRP1	IPI00005154	0.663	0.06611	6	6	6	10
877	Rho-associated protein kinase 1	IPI00022542	0.663	0.06611	6	6	7	9
878	60S ribosomal protein L38	IPI00215790	0.663	0.06611	6	6	7	9
879	Protein NipSnap homolog 1	IPI00304435	0.663	0.06611	6	6	9	7
880	cDNA FLJ56153, highly similar to Homo sapiens transforming growth factor beta regulator 4 (TBRG4)	IPI00329625	0.663	0.06611	7	5	9	7
881	Isoform 2 of Histone deacetylase 2	IPI00289601	0.663	0.06611	7	5	8	8
882	cDNA FLJ55988, highly similar to RNA-binding protein Luc7-like 2	IPI00006932	0.663	0.06611	6	6	9	7
883	Condensin complex subunit 1	IPI00299524	0.657	0.06866	30	30	32	33
884	Isoform 1 of Gamma-glutamylcyclotransferase	IPI00031564	0.656	0.06866	6	7	8	9
885	ANKHD1-EIF4EBP3 protein	IPI00217442	0.656	0.06866	6	7	8	9
886	Casein kinase II subunit alpha'	IPI00020602	0.656	0.06866	6	7	8	9
887	Mitochondrial import inner membrane translocase subunit Tim23	IPI00007309	0.656	0.06866	7	6	8	9
888	Isoform 1 of Polymerase I and transcript release factor	IPI00176903	0.656	0.06866	7	6	10	7
889	Thyroid hormone receptor-associated protein 3	IPI00104050	0.649	0.06873	6	8	10	8
890	cDNA FLJ59571, highly similar to Eukaryotic translation initiation factor 4gamma 2	IPI00015952	0.649	0.06873	6	8	9	9
891	Peptidyl-prolyl cis-trans isomerase H	IPI00007346	0.649	0.06873	7	7	10	8
892	Isoform 1 of UDP-glucose:glycoprotein glucosyltransferase 1	IPI00024466	0.647	0.07124	34	32	39	32
893	NADH dehydrogenase [ubiquinone] iron-sulfur protein 3, mitochondrial	IPI00025796	0.643	0.07127	7	8	9	10
894	Isoform 1 of Voltage-dependent anion-selective channel protein 3	IPI00031804	0.643	0.07127	9	6	9	10
895	Isoform 3 of Protein VPRBP	IPI00181396	0.643	0.07127	6	9	8	11
896	Isoform Long of Eukaryotic translation initiation factor 4H	IPI00014263	0.637	0.07127	7	9	10	10
897	DnaJ homolog subfamily A member 2	IPI00032406	0.637	0.07127	7	9	10	10
898	Ras-related protein Rab-18	IPI00014577	0.637	0.07127	8	8	10	10
899	Isoform 3 of Glutaminase kidney isoform, mitochondrial	IPI00215687	0.632	0.07344	9	8	11	10
900	perilipin-3 isoform 3	IPI00106668	0.632	0.07344	9	8	10	11
901	HSPA5 protein	IPI00003362	0.628	0.07351	41	40	43	43
902	Ubiquitin-conjugating enzyme E2 N	IPI00003949	0.622	0.07564	9	10	11	12
903	Exosome complex exonuclease RRP4	IPI00015905	0.622	0.07564	11	8	10	13
904	Serine/threonine-protein kinase mTOR	IPI00031410	0.617	0.07567	9	11	12	12
905	40S ribosomal protein S13	IPI00221089	0.617	0.07567	11	9	13	11
906	Eukaryotic translation initiation factor 2 subunit 3	IPI00297982	0.617	0.07567	11	9	10	14
907	Actin-related protein 3	IPI00028091	0.617	0.07567	10	10	12	12
908	Importin subunit beta-1	IPI00001639	0.614	0.07671	45	49	50	49
909	Isoform 1 of ATP synthase subunit d, mitochondrial	IPI00220487	0.613	0.07671	11	10	12	13
910	Cleavage and polyadenylation specificity factor subunit 1	IPI00026219	0.609	0.07674	9	13	10	16
911	60S ribosomal protein L7	IPI00030179	0.605	0.07815	13	10	15	12
912	Isoform 1 of Acidic leucine-rich nuclear phosphoprotein 32 family member B	IPI00007423	0.605	0.07815	12	11	12	15
913	Transgelin	IPI00216138	0.605	0.07815	10	13	12	15
914	CTP synthase 1	IPI00290142	0.602	0.07815	10	14	14	14
915	Isoform 1 of Nuclear pore complex protein Nup160	IPI00748807	0.592	0.07973	13	14	16	15
916	Nucleolar protein 56	IPI00411937	0.592	0.07973	14	13	12	19
917	Isoform 1 of Protein KIAA1967	IPI00182757	0.585	0.08035	15	14	17	16
918	Isoform 1 of Peroxisomal membrane protein PEX16	IPI00006722	0.576	0.08162	0	2	4	3
919	Isoform HMG-I of High mobility group protein HMG-I/HMG-Y	IPI00179700	0.576	0.08162	0	0	5	0
920	Isoform 1 of Solute carrier family 12 member 7	IPI00008616	0.576	0.08162	0	0	0	5
921	Isoform 1 of Methylthioribose-1-phosphate isomerase	IPI00005948	0.576	0.08162	0	2	4	3
922	Isoform 2 of Cytosolic non-specific dipeptidase	IPI00165579	0.576	0.08162	0	2	3	4
923	Isoform 1 of Secretory carrier-associated membrane protein 1	IPI00005129	0.576	0.08162	0	0	4	3
924	Putative uncharacterized protein C20orf43	IPI00218962	0.576	0.08162	0	2	3	4
925	24 kDa protein	IPI00398057	0.576	0.08162	0	0	5	0
926	Ubiquitin-conjugating enzyme E2 T	IPI00023087	0.576	0.08162	2	2	4	3
927	NADH-ubiquinone oxidoreductase chain 2	IPI00007979	0.576	0.08162	0	0	4	3
928	Isoform 1 of ADP-ribosylation factor-like protein 2-binding protein	IPI00015866	0.576	0.08162	2	2	4	3
929	Isoform 1 of Uridine-cytidine kinase 2	IPI00065671	0.576	0.08162	0	2	4	3
930	mesencephalic astrocyte-derived neurotrophic factor	IPI00328748	0.576	0.08162	0	2	5	2
931	bifunctional protein NCOAT isoform b	IPI00181391	0.576	0.08162	2	2	3	4
932	Isoform 2 of cAMP-dependent protein kinase catalytic subunit alpha	IPI00217960	0.576	0.08162	0	0	5	0
933	Dolichyldiphosphatase 1	IPI00329410	0.576	0.08162	0	2	2	5
934	STE20/SPS1-related proline-alanine-rich protein kinase	IPI00004363	0.576	0.08162	2	0	2	5
935	Isoform 1 of Bromodomain-containing protein 4	IPI00440727	0.576	0.08162	0	0	2	5
936	Ribosomal protein S6 kinase alpha-3	IPI00020898	0.576	0.08162	0	0	4	3
937	Peptidase M20 domain-containing protein 2	IPI00217852	0.576	0.08162	0	2	4	3
938	Isoform Long of Transformer-2 protein homolog alpha	IPI00013891	0.576	0.08162	0	0	2	5
939	Isoform SRP40-1 of Splicing factor, arginine/serine-rich 5	IPI00012341	0.576	0.08162	2	2	3	4
940	Peptidyl-prolyl cis-trans isomerase D	IPI00003927	0.576	0.08162	2	2	2	5
941	Isoform 1 of Cellular tumor antigen p53	IPI00025087	0.576	0.08162	0	2	2	5
942	Isoform 1 of KDEL motif-containing protein 2	IPI00143921	0.576	0.08162	2	0	3	4
943	Scaffold attachment factor B1	IPI00300631	0.576	0.08162	2	0	3	4
944	Isoform C of Lethal(2) giant larvae protein homolog 2	IPI00465050	0.576	0.08162	0	0	2	5
945	ubiquitin-like with PHD and ring finger domains 1 isoform 2	IPI00797279	0.576	0.08162	2	0	3	4

No.	Description	Accession number	STN[1]	p-Value[1]	480_A[2]	480_B[2]	620_A[2]	620_B[2]
946	39S ribosomal protein L23, mitochondrial	IPI00293476	0.576	0.08162	2	2	4	3
947	Pre-mRNA cleavage complex 2 protein Pcf11	IPI00016387	0.576	0.08162	0	0	3	4
948	Isoform p27-L of 26S proteasome non-ATPase regulatory subunit 9	IPI00010860	0.576	0.08162	0	2	4	3
949	Sedoheptulokinase	IPI00005914	0.576	0.08162	0	0	3	4
950	Ubiquitin-conjugating enzyme E2 G1	IPI00219783	0.576	0.08162	2	2	4	3
951	Exosome complex exonuclease RRP41	IPI00745613	0.576	0.08162	2	2	4	3
952	Isoform 2 of Heme-binding protein 2	IPI00003799	0.576	0.08162	0	2	3	4
953	Transcription initiation factor TFIID subunit 9	IPI00002993	0.576	0.08162	2	2	4	3
954	D-tyrosyl-tRNA(Tyr) deacylase 1	IPI00152692	0.576	0.08162	0	0	4	3
955	Isoform 2 of Integrator complex subunit 7	IPI00645022	0.576	0.08162	0	0	5	2
956	WW domain-binding protein 11	IPI00170786	0.576	0.08162	0	0	4	3
957	GA-binding protein alpha chain	IPI00299413	0.576	0.08162	0	0	3	4
958	TBC1 domain family member 15 isoform 1	IPI00154645	0.576	0.08162	2	2	5	2
959	UPF0600 protein C5orf51	IPI00374272	0.576	0.08162	2	0	3	4
960	Isoform B of Arfaptin-1	IPI00021258	0.576	0.08162	0	0	3	4
961	Probable asparaginyl-tRNA synthetase, mitochondrial	IPI00101664	0.576	0.08162	0	0	4	3
962	Mediator of RNA polymerase II transcription subunit 13	IPI00021388	0.576	0.08162	0	0	4	3
963	Protein FAM128B	IPI00410094	0.576	0.08162	2	2	4	3
964	CGG triplet repeat-binding protein 1	IPI00295585	0.576	0.08162	0	2	0	5
965	Isoform 1 of Caldesmon	IPI00014516	0.576	0.08162	0	2	3	4
966	cDNA, FLJ96508, Homo sapiens SH3-domain GRB2-like 1 (SH3GL1), mRNA	IPI00019169	0.576	0.08162	2	2	3	4
967	Vacuolar protein sorting-associated protein 37B	IPI00002926	0.576	0.08162	0	0	4	3
968	Isoform 2 of Valacyclovir hydrolase	IPI00003990	0.576	0.08162	2	2	3	4
969	U6 snRNA-associated Sm-like protein LSm3	IPI00219229	0.576	0.08162	0	0	3	4
970	Isoform Long of Ras-related protein Rab-27A	IPI00016381	0.576	0.08162	0	2	0	5
971	Pleckstrin-2	IPI00009302	0.576	0.08162	0	2	3	4
972	Putative uncharacterized protein RBM12B	IPI00217626	0.576	0.08162	0	0	3	4
973	Carboxymethylenebutenolidase homolog	IPI00383046	0.576	0.08162	0	0	2	5
974	RWD domain-containing protein 1	IPI00034010	0.576	0.08162	0	0	2	5
975	Isoform Alpha of E3 ubiquitin-protein ligase TRIM33	IPI00010252	0.576	0.08162	0	0	3	4
976	Isoform 2 of Gamma-adducin	IPI00004408	0.576	0.08162	0	0	2	5
977	Protein C10	IPI00016925	0.576	0.08162	0	0	3	4
978	Serine/threonine-protein kinase Chk1	IPI00023664	0.576	0.08162	0	0	4	3
979	Isoform 1 of Protein kinase C and casein kinase substrate in neurons protein 2	IPI00027009	0.576	0.08162	0	0	2	5
980	Isoform 1 of ADP-ribosylation factor GTPase-activating protein 1	IPI00175169	0.576	0.08162	0	2	4	3
981	serine/threonine-protein kinase MST4 isoform 3	IPI00182383	0.576	0.08162	0	0	3	4
982	Eukaryotic translation initiation factor 3 subunit M	IPI00102069	0.575	0.08379	17	16	18	19
983	Intron-binding protein aquarius	IPI00297572	0.575	0.08379	15	18	20	17
984	Stomatin-like protein 2	IPI00334190	0.575	0.08379	16	17	18	19
985	Eukaryotic translation initiation factor 3 subunit C	IPI00016910	0.567	0.08448	16	20	17	23
986	Isoform LAMP-2A of Lysosome-associated membrane glycoprotein 2	IPI00009030	0.561	0.08524	2	3	5	3
987	Isoform Long of Long-chain-fatty-acid--CoA ligase 4	IPI00029737	0.561	0.08524	3	0	3	5
988	Ribosome biogenesis protein BOP1	IPI00028955	0.561	0.08524	2	3	3	5
989	Isoform 1 of SWI/SNF-related matrix-associated actin-dependent regulator of chromatin subfamily E	IPI00017669	0.561	0.08524	0	3	2	6
990	Translational activator of cytochrome c oxidase 1	IPI00019903	0.561	0.08524	3	2	4	4
991	39S ribosomal protein L48, mitochondrial	IPI00295066	0.561	0.08524	2	3	3	5
992	Mitochondrial glutamate carrier 1	IPI00003004	0.561	0.08524	3	2	5	3
993	13kDa differentiation-associated protein variant (Fragment)	IPI00005966	0.561	0.08524	3	2	5	3
994	N-alpha-acetyltransferase 20, NatB catalytic subunit	IPI00007174	0.561	0.08524	3	0	5	3
995	3-mercaptopyruvate sulfurtransferase	IPI00165360	0.561	0.08524	3	0	4	4
996	Synaptogyrin-2	IPI00013946	0.561	0.08524	3	2	5	3
997	cytochrome c oxidase subunit VIIa polypeptide 2 (liver) precursor	IPI00026570	0.561	0.08524	3	2	3	5
998	14 kDa protein	IPI00179589	0.561	0.08524	0	3	4	4
999	Similar to Ankyrin repeat and FYVE domain-containing protein 1	IPI00159899	0.561	0.08524	2	3	4	4
1000	U3 small nucleolar ribonucleoprotein protein MPP10	IPI00012149	0.561	0.08524	2	3	4	4
1001	Copine-3	IPI00024403	0.561	0.08524	3	0	4	4
1002	Proteasome activator complex subunit 1	IPI00479722	0.549	0.08617	25	20	25	24
1003	Cell growth-regulating nucleolar protein	IPI00015838	0.548	0.08617	2	4	6	3
1004	Coproporphyrinogen-III oxidase, mitochondrial	IPI00093057	0.548	0.08617	2	4	5	4
1005	GTPase NRas	IPI00000005	0.548	0.08617	2	4	4	5
1006	Isoform 1 of Nucleolar protein 3	IPI00105916	0.548	0.08617	3	3	4	5
1007	Isoform 1 of RNA-binding protein 4	IPI00003704	0.548	0.08617	3	3	4	5
1008	Serine/threonine-protein kinase VRK1	IPI00019640	0.548	0.08617	2	4	3	6
1009	Isoform 1 of Nicalin	IPI00470649	0.548	0.08617	4	2	5	4
1010	Isoform 2 of U1 small nuclear ribonucleoprotein 70 kDa	IPI00219483	0.548	0.08617	4	2	5	4
1011	Isoform 1 of 39S ribosomal protein L47, mitochondrial	IPI00030820	0.548	0.08617	3	3	4	5
1012	Double-strand-break repair protein rad21 homolog	IPI00006715	0.548	0.08617	4	0	5	4
1013	Aflatoxin B1 aldehyde reductase member 2	IPI00305978	0.548	0.08617	3	3	3	6
1014	Isoform 1 of Polyadenylate-binding protein 2	IPI00005792	0.548	0.08617	2	4	5	4
1015	Sulfide:quinone oxidoreductase, mitochondrial	IPI00009634	0.548	0.08617	3	3	5	4
1016	Isoform 2 of U4/U6 small nuclear ribonucleoprotein Prp31	IPI00167198	0.548	0.08617	2	4	5	4
1017	Isoform 1 of Dynamin-1	IPI00413140	0.548	0.08617	3	3	6	3
1018	Isoform 3 of UDP-N-acetylglucosamine--peptide N-acetylglucosaminyltransferase 110 kDa subunit	IPI00005780	0.537	0.08881	4	3	5	5
1019	LDLR chaperone MESD	IPI00399089	0.537	0.08881	4	3	6	4
1020	NADH dehydrogenase [ubiquinone] 1 beta subcomplex subunit 4	IPI00220059	0.537	0.08881	3	4	5	5
1021	Transmembrane emp24 domain-containing protein 5	IPI00294472	0.537	0.08881	0	5	4	6
1022	Nucleolar GTP-binding protein 2	IPI00015808	0.537	0.08881	3	4	4	6
1023	High mobility group protein B3	IPI00217477	0.537	0.08881	4	3	6	4
1024	Succinate dehydrogenase assembly factor 2, mitochondrial	IPI00016443	0.537	0.08881	3	4	5	5
1025	Isoform 3 of Centromere protein V	IPI00376481	0.537	0.08881	3	4	6	4
1026	Isoform Beta-2 of DNA topoisomerase 2-beta	IPI00027280	0.533	0.09033	23	32	30	29
1027	Histone-binding protein RBBP7	IPI00395865	0.528	0.09053	0	6	5	6
1028	40S ribosomal protein S25	IPI00012750	0.528	0.09053	4	4	5	6
1029	Mitochondrial import inner membrane translocase subunit Tim17-B	IPI00219833	0.528	0.09053	4	4	7	4
1030	Myeloid-associated differentiation marker	IPI00102685	0.528	0.09053	6	2	5	6
1031	Isoform 2 of Guanine nucleotide-binding protein-like 3	IPI00003886	0.528	0.09053	4	4	6	5
1032	Exosome complex exonuclease MTR3	IPI00073602	0.528	0.09053	4	4	7	4
1033	Isoform 1 of Regulator of nonsense transcripts 2	IPI00300504	0.528	0.09053	2	6	6	5
1034	SCY1-like protein 2	IPI00396218	0.528	0.09053	3	5	6	5
1035	Isoform 1 of U3 small nucleolar RNA-associated protein 14 homolog A	IPI00107113	0.528	0.09053	4	4	5	6
1036	Replication protein A 14 kDa subunit	IPI00017373	0.528	0.09053	4	4	7	4
1037	Translation initiation factor eIF-2B subunit alpha	IPI00221300	0.528	0.09053	4	4	3	8
1038	MACRO domain-containing protein 1	IPI00155601	0.528	0.09053	4	4	4	7
1039	SWI/SNF complex subunit SMARCC1	IPI00234252	0.520	0.09177	4	5	5	7

No.	Description	Accession number	STN[1]	p-Value[1]	480_A[2]	480_B[2]	620_A[2]	620_B[2]
1040	GrpE protein homolog 1, mitochondrial	IPI00029557	0.520	0.09177	3	6	6	6
1041	Isoform 1 of Calcineurin-like phosphoesterase domain-containing protein 1	IPI00305010	0.520	0.09177	5	4	6	6
1042	Phosphomannomutase 2	IPI00006092	0.520	0.09177	5	4	4	8
1043	Probable rRNA-processing protein EBP2	IPI00745955	0.520	0.09177	3	6	5	7
1044	Isoform 1 of DDRGK domain-containing protein 1	IPI00028387	0.520	0.09177	5	4	7	5
1045	Seryl-tRNA synthetase, mitochondrial	IPI00328361	0.520	0.09177	5	4	5	7
1046	Pyruvate dehydrogenase protein X component, mitochondrial	IPI00298423	0.520	0.09177	5	4	6	6
1047	Flotillin-1	IPI00027438	0.520	0.09177	4	5	6	6
1048	Eukaryotic translation initiation factor 5B	IPI00299254	0.514	0.09294	31	39	36	38
1049	Thioredoxin domain-containing protein 17	IPI00646689	0.512	0.09304	5	5	5	8
1050	DEAD (Asp-Glu-Ala-Asp) box polypeptide 39 transcript variant	IPI00062206	0.512	0.09304	4	6	7	6
1051	Protein phosphatase 1 regulatory subunit 14B	IPI00398922	0.512	0.09304	6	4	8	5
1052	Cytochrome c-type heme lyase	IPI00023406	0.512	0.09304	4	6	9	4
1053	Isoform 1 of Apolipoprotein O	IPI00042580	0.512	0.09304	5	5	8	5
1054	Deoxyribonucleoside 5'-monophosphate N-glycosidase	IPI00007926	0.512	0.09304	6	4	7	6
1055	NADH dehydrogenase [ubiquinone] iron-sulfur protein 2, mitochondrial	IPI00025239	0.512	0.09304	5	5	5	8
1056	Rho-related GTP-binding protein RhoG	IPI00017342	0.512	0.09304	5	5	6	7
1057	proteasome subunit beta type-5 isoform 3	IPI00383971	0.512	0.09304	5	5	7	6
1058	Programmed cell death protein 5	IPI00023640	0.512	0.09304	4	6	6	7
1059	CLASP2 protein	IPI00168165	0.506	0.09421	7	4	7	7
1060	DNA-directed RNA polymerase II subunit RPB3	IPI00018288	0.506	0.09421	5	6	7	7
1061	Isoform 1 of AP-3 complex subunit beta-1	IPI00021129	0.506	0.09421	6	5	7	7
1062	Tubulin gamma-1 chain	IPI00295081	0.506	0.09421	4	7	6	8
1063	Eukaryotic translation initiation factor 3 subunit J	IPI00290461	0.506	0.09421	5	6	5	9
1064	Pyrroline-5-carboxylate reductase 2	IPI00470610	0.506	0.09421	5	6	6	8
1065	E3 SUMO-protein ligase RanBP2	IPI00221325	0.500	0.09511	41	43	40	48
1066	Sepiapterin reductase	IPI00017469	0.500	0.09525	6	6	7	8
1067	Isoform 1 of Reticulon-4	IPI00021766	0.500	0.09525	5	7	3	12
1068	Isoform 1 of RNA-binding protein 39	IPI00163505	0.500	0.09525	7	5	8	7
1069	Isoform 1 of Methylcrotonoyl-CoA carboxylase beta chain, mitochondrial	IPI00784044	0.500	0.09525	4	8	6	9
1070	Isoform 1 of Protein virilizer homolog	IPI00036742	0.494	0.09628	6	7	8	8
1071	Thymidylate kinase	IPI00013862	0.494	0.09628	6	7	8	8
1072	Heme-binding protein 1	IPI00148063	0.494	0.09628	7	6	8	8
1073	Glucosamine 6-phosphate N-acetyltransferase	IPI00061525	0.494	0.09628	6	7	9	7
1074	Prolyl endopeptidase	IPI00008164	0.494	0.09628	6	7	8	8
1075	Isoform 1 of Growth factor receptor-bound protein 2	IPI00021327	0.489	0.09697	7	7	7	10
1076	Protein mago nashi homolog 2	IPI00059292	0.489	0.09697	6	8	9	8
1077	Isoform 1 of RNA-binding protein 25	IPI00004273	0.489	0.09697	6	8	7	10
1078	60S ribosomal protein L22	IPI00219153	0.489	0.09697	7	7	11	6
1079	Isoform 1 of Hematological and neurological expressed 1 protein	IPI00007764	0.489	0.09697	5	9	9	8
1080	UPF0160 protein MYG1, mitochondrial	IPI00029444	0.484	0.09721	10	5	9	9
1081	Isoform 1 of Oxysterol-binding protein 1	IPI00024971	0.484	0.09721	8	7	10	8
1082	60S ribosomal protein L26-like 1	IPI00007144	0.480	0.09789	8	8	8	11
1083	28S ribosomal protein S22, mitochondrial	IPI00013146	0.480	0.09789	8	8	10	9
1084	Heterogeneous nuclear ribonucleoprotein A0	IPI00011913	0.480	0.09789	9	7	9	10
1085	Hsc70-interacting protein	IPI00032826	0.480	0.09789	9	7	8	11
1086	Coiled-coil-helix-coiled-coil-helix domain-containing protein 3, mitochondrial	IPI00015833	0.480	0.09789	9	7	10	9
1087	Isoform 1 of Armadillo repeat-containing protein 10	IPI00166394	0.480	0.09789	8	8	11	8
1088	Proline synthetase co-transcribed homolog (Bacterial), isoform CRA_b	IPI00016346	0.476	0.09913	9	8	11	9
1089	Emerin	IPI00032003	0.472	0.09948	8	10	11	10
1090	Ribosome maturation protein SBDS	IPI00427330	0.472	0.09948	7	11	10	11
1091	NADH-ubiquinone oxidoreductase 75 kDa subunit	IPI00604664	0.472	0.09948	9	9	12	9
1092	Phosducin-like protein 3	IPI00031629	0.472	0.09948	8	10	14	7
1093	Isoform 1 of Apoptotic chromatin condensation inducer in the nucleus	IPI00007334	0.468	0.09982	8	11	10	12
1094	Leucine-rich repeat and WD repeat-containing protein 1	IPI00069309	0.468	0.09982	11	8	13	9
1095	Regulator of microtubule dynamics protein 1	IPI00329696	0.468	0.09982	12	7	10	12
1096	Flap endonuclease 1	IPI00026215	0.465	0.10051	10	10	13	10
1097	Adenine phosphoribosyltransferase	IPI00218693	0.465	0.10051	10	10	12	11
1098	Heme oxygenase 2	IPI00026824	0.465	0.10051	11	9	10	13
1099	Isoform 1 of Cytoplasmic FMR1-interacting protein 1	IPI00644231	0.465	0.10051	11	9	13	10
1100	Alcohol dehydrogenase [NADP+]	IPI00220271	0.465	0.10051	12	8	11	12
1101	cDNA FLJ36192 fis, clone TESTI2027450, highly similar to Eukaryotic translation initiation factor 3	IPI00654777	0.461	0.10109	11	10	11	13
1102	ADP-ribosylation factor-like protein 1	IPI00219518	0.461	0.10109	12	9	13	11
1103	UPF0568 protein C14orf166	IPI00006980	0.455	0.10161	11	12	15	11
1104	Isoform 1 of DNA-binding protein A	IPI00031801	0.453	0.10206	10	14	12	15
1105	Isoform 2 of S-phase kinase-associated protein 1	IPI00172421	0.450	0.10216	12	13	16	12
1106	Endoplasmic reticulum resident protein 29	IPI00024911	0.450	0.10216	13	12	15	13
1107	3-hydroxyisobutyrate dehydrogenase, mitochondrial	IPI00013860	0.450	0.10216	13	12	13	15
1108	Isochorismatase domain-containing protein 1	IPI00304082	0.447	0.10233	12	14	15	14
1109	Proteasome subunit alpha type-6	IPI00029623	0.440	0.10326	16	13	18	14
1110	Isoform 2 of Guanine nucleotide-binding protein G(i) subunit alpha-2	IPI00217906	0.440	0.10326	15	14	14	18
1111	Eukaryotic translation initiation factor 3 subunit E	IPI00013068	0.438	0.10364	11	19	15	18
1112	Isoform 1 of Tropomyosin alpha-4 chain	IPI00010779	0.436	0.10395	15	16	16	18
1113	40S ribosomal protein S17	IPI00221093	0.436	0.10395	16	15	16	18
1114	Glycogen phosphorylase, brain form	IPI00004358	0.432	0.10457	15	18	19	17
1115	Adenylate kinase isoenzyme 1	IPI00018342	0.421	0.10567	20	19	21	21
1116	Isoform Long of Glucose-6-phosphate 1-dehydrogenase	IPI00216008	0.420	0.10577	20	20	23	20
1117	Isoform 1 of Structural maintenance of chromosomes protein 2	IPI00007927	0.398	0.10770	32	25	29	31
1118	Protein disulfide-isomerase	IPI00010796	0.392	0.10808	35	28	36	30
1119	40S ribosomal protein S11	IPI00025091	0.389	0.10815	2	2	4	2
1120	cDNA FLJ59739, highly similar to Protein transport protein Sec61 subunit alpha isoform 1	IPI00218466	0.389	0.10815	0	0	3	3
1121	Protein S100-A6	IPI00027463	0.389	0.10815	2	2	4	0
1122	Actin-related protein 2/3 complex subunit 1B	IPI00005160	0.389	0.10815	0	2	2	4
1123	ADP/ATP translocase 3	IPI00291467	0.389	0.10815	0	0	0	4
1124	Putative uncharacterized protein DOCK6	IPI00184772	0.389	0.10815	0	2	3	3
1125	Isoform 4 of Dipeptidyl peptidase 9	IPI00604483	0.389	0.10815	0	0	2	4
1126	Putative uncharacterized protein THADA	IPI00412647	0.389	0.10815	2	0	2	4
1127	Phosphoserine phosphatase	IPI00019178	0.389	0.10815	0	0	2	4
1128	Ubiquitin carboxyl-terminal hydrolase 10	IPI00291946	0.389	0.10815	0	0	2	4
1129	Isoform 1 of Remodeling and spacing factor 1	IPI00290652	0.389	0.10815	2	2	3	3
1130	Exosome complex exonuclease RRP40	IPI00015956	0.389	0.10815	0	2	3	3
1131	Isoform 1 of HAUS augmin-like complex subunit 2	IPI00018198	0.389	0.10815	0	2	4	0
1132	WD repeat-containing protein 11	IPI00412224	0.389	0.10815	0	0	3	3
1133	UDP-glucose:glycoprotein glucosyltransferase 2	IPI00024467	0.389	0.10815	0	0	4	2
1134	Uncharacterized protein C11orf73	IPI00410091	0.389	0.10815	2	2	3	3

No.	Description	Accession number	STN[1]	p-Value[1]	480_A[2]	480_B[2]	620_A[2]	620_B[2]
1135	Chitobiosyldiphosphodolichol beta-mannosyltransferase	IPI00549761	0.389	0.10815	0	0	3	3
1136	Cyclin-G-associated kinase	IPI00298949	0.389	0.10815	2	2	2	4
1137	Retinol dehydrogenase 13	IPI00301204	0.389	0.10815	0	2	3	3
1138	RNA binding motif protein, X-linked-like 1	IPI00061178	0.389	0.10815	0	0	0	4
1139	Isoform Membrane-bound of Catechol O-methyltransferase	IPI00011284	0.389	0.10815	0	2	2	4
1140	Isoform 1 of 2-oxoglutarate and iron-dependent oxygenase domain-containing protein 1	IPI00170429	0.389	0.10815	2	2	3	3
1141	Cyclin-H	IPI00021305	0.389	0.10815	2	2	3	3
1142	Mitochondrial chaperone BCS1	IPI00003985	0.389	0.10815	0	2	4	2
1143	Isoform 2 of Beta-catenin-like protein 1	IPI00472981	0.389	0.10815	0	0	4	2
1144	Replication factor C subunit 3	IPI00031521	0.389	0.10815	0	0	0	4
1145	Aldose reductase	IPI00413641	0.389	0.10815	0	0	4	2
1146	Ras-related protein Rab-9A	IPI00016372	0.389	0.10815	0	2	4	2
1147	Isoform 1 of H/ACA ribonucleoprotein complex subunit 1	IPI00302176	0.389	0.10815	2	2	4	2
1148	Isoform 1 of Centrosomal protein of 170 kDa	IPI00186194	0.389	0.10815	0	0	0	4
1149	DNA polymerase subunit gamma-1	IPI00004317	0.389	0.10815	2	2	3	3
1150	ESF1 homolog	IPI00024167	0.389	0.10815	0	2	3	3
1151	Isoform 2 of Diphosphoinositol polyphosphate phosphohydrolase 2	IPI00021408	0.389	0.10815	2	0	3	3
1152	Isoform 4 of Nucleoporin NDC1	IPI00003455	0.389	0.10815	0	2	2	4
1153	Isoform II of Ubiquitin-protein ligase E3A	IPI00011609	0.389	0.10815	2	2	3	3
1154	Vacuolar protein sorting-associated protein 4B	IPI00182728	0.389	0.10815	0	0	3	3
1155	Isoform 1 of HCLS1-associated protein X-1	IPI00010440	0.389	0.10815	0	0	4	2
1156	SNARE-associated protein Snapin	IPI00018331	0.389	0.10815	0	0	2	4
1157	cDNA FLJ58308, highly similar to Alpha-1,2-mannosyltransferase ALG9	IPI00234857	0.389	0.10815	0	0	2	4
1158	Isoform 1 of Partner of Y14 and mago	IPI00305092	0.389	0.10815	2	2	3	3
1159	Serine/threonine-protein phosphatase 5	IPI00019812	0.389	0.10815	2	2	2	4
1160	39S ribosomal protein L2, mitochondrial	IPI00411816	0.389	0.10815	0	0	3	3
1161	Ribonuclease P protein subunit p38	IPI00019195	0.389	0.10815	2	2	4	2
1162	Vacuolar protein sorting-associated protein 4A	IPI00411356	0.389	0.10815	0	0	3	3
1163	Isoform 1 of DnaJ homolog subfamily C member 10	IPI00293260	0.389	0.10815	2	2	0	4
1164	Serine palmitoyltransferase 1	IPI00005745	0.389	0.10815	2	2	2	4
1165	Isoform Alpha-1 of Protein phosphatase 1A	IPI00020950	0.389	0.10815	0	0	4	0
1166	Isoform 1 of Translation initiation factor eIF-2B subunit delta	IPI00005979	0.389	0.10815	0	0	4	2
1167	Isoform 1 of Tuftelin-interacting protein 11	IPI00015924	0.389	0.10815	0	0	4	2
1168	Isoform 1 of Neurochondrin	IPI00549543	0.389	0.10815	0	2	3	3
1169	Putative uncharacterized protein	IPI00010402	0.389	0.10815	0	0	3	3
1170	39S ribosomal protein L20, mitochondrial	IPI00013706	0.389	0.10815	0	0	0	4
1171	Isoform 1 of Presenilin-1	IPI00028077	0.389	0.10815	2	2	4	0
1172	Isoform 2 of DNA-directed RNA polymerase I subunit RPA2	IPI00026445	0.389	0.10815	0	0	2	4
1173	Isoform 1 of HAUS augmin-like complex subunit 1	IPI00431082	0.389	0.10815	0	0	3	3
1174	Isoform 1 of Serine/threonine-protein kinase 4	IPI00011488	0.389	0.10815	0	0	2	4
1175	Isoform 1 of Telomeric repeat-binding factor 2	IPI00024214	0.389	0.10815	0	0	0	4
1176	importin subunit alpha-6	IPI00413214	0.389	0.10815	2	0	3	3
1177	Isoform 1 of SOSS complex subunit B1	IPI00031633	0.389	0.10815	0	2	3	3
1178	Aldehyde dehydrogenase, dimeric NADP-preferring	IPI00296183	0.389	0.10815	0	2	3	3
1179	CTP synthase 2	IPI00645702	0.389	0.10815	2	2	3	3
1180	Isoform 1 of RNA-binding protein 34	IPI00181617	0.389	0.10815	0	0	3	3
1181	114 kDa protein	IPI00166555	0.389	0.10815	2	2	4	2
1182	Brain-specific angiogenesis inhibitor 1-associated protein 2-like protein 1	IPI00179326	0.389	0.10815	0	0	2	4
1183	CDK-activating kinase assembly factor MAT1	IPI00294701	0.389	0.10815	0	2	3	3
1184	MARCKS-related protein	IPI00641181	0.389	0.10815	0	2	3	3
1185	Isoform 1 of Aldehyde dehydrogenase family 16 member A1	IPI00217920	0.389	0.10815	2	2	2	4
1186	cDNA FLJ54848, highly similar to tRNA-splicing endonuclease subunit Sen34	IPI00451941	0.389	0.10815	0	2	0	4
1187	Splicing factor 3B subunit 5	IPI00010404	0.389	0.10815	2	0	3	3
1188	JmjC domain-containing protein 7	IPI00382394	0.389	0.10815	2	0	3	3
1189	sphingomyelin phosphodiesterase 4 isoform 1	IPI00743121	0.389	0.10815	0	0	2	4
1190	Isoform 1 of Protein 4.1	IPI00003921	0.389	0.10815	0	0	4	2
1191	ATP-dependent RNA helicase SUPV3L1, mitochondrial	IPI00412404	0.389	0.10815	0	0	3	3
1192	Isoform 1 of Serologically defined colon cancer antigen 1	IPI00301618	0.389	0.10815	0	0	4	2
1193	Isoform 3 of Tyrosine-protein kinase-like 7	IPI00168813	0.389	0.10815	0	0	3	3
1194	Protein kinase C iota type	IPI00016639	0.389	0.10815	0	0	4	2
1195	Isoform 2 of Ankyrin repeat domain-containing protein 17	IPI00783186	0.389	0.10815	0	0	2	4
1196	Isoform 3 of Nuclear transcription factor Y subunit gamma	IPI00071697	0.389	0.10815	0	0	3	3
1197	ADP-ribosylation factor GTPase-activating protein 2	IPI00297322	0.389	0.10815	0	2	2	4
1198	Cytochrome c oxidase subunit 1	IPI00464968	0.389	0.10815	0	0	0	4
1199	Isoform 1 of Inositol-tetrakisphosphate 1-kinase	IPI00100329	0.389	0.10815	2	0	3	3
1200	Glia maturation factor gamma	IPI00028414	0.389	0.10815	2	0	4	2
1201	Isoform 2 of DNA replication complex GINS protein PSF3	IPI00185097	0.389	0.10815	0	0	3	3
1202	Methylmalonyl-CoA mutase, mitochondrial	IPI00024934	0.389	0.10815	0	0	2	4
1203	Isoform 2 of Arf-GAP with Rho-GAP domain, ANK repeat and PH domain-containing protein 1	IPI00220421	0.389	0.10815	0	0	3	3
1204	cDNA FLJ56184, highly similar to Proto-oncogene tyrosine-protein kinase LCK	IPI00394952	0.389	0.10815	0	0	3	3
1205	Glutathione peroxidase 2	IPI00298176	0.389	0.10815	0	0	2	4
1206	Myotubularin	IPI00748788	0.389	0.10815	0	0	3	3
1207	Vacuolar ATPase assembly integral membrane protein VMA21	IPI00146447	0.389	0.10815	0	0	3	3
1208	Isoform 2 of Tescalcin	IPI00791863	0.389	0.10815	0	0	3	3
1209	SHC-transforming protein 1 isoform 3	IPI00021326	0.389	0.10815	0	0	0	4
1210	Isoform 1 of Craniofacial development protein 1	IPI00007306	0.389	0.10815	0	0	3	3
1211	LIM and cysteine-rich domains protein 1	IPI00303258	0.389	0.10815	0	0	3	3
1212	Protein AF1q	IPI00011421	0.389	0.10815	0	0	0	4
1213	Isoform 1 of Protein LSM14 homolog B	IPI00032635	0.389	0.10815	0	0	4	0
1214	Serum deprivation-response protein	IPI00005809	0.389	0.10815	0	0	3	3
1215	Transmembrane emp24 domain-containing protein 2	IPI00016608	0.378	0.13295	2	3	4	3
1216	Transmembrane protein 14C	IPI00009346	0.378	0.13295	3	0	3	4
1217	28S ribosomal protein S34, mitochondrial	IPI00169413	0.378	0.13295	3	2	3	4
1218	Ribose-phosphate pyrophosphokinase 3	IPI00218371	0.378	0.13295	0	3	4	3
1219	Isoform 1 of Ral GTPase-activating protein subunit beta	IPI00409601	0.378	0.13295	2	3	4	3
1220	sulfatase modifying factor 2 isoform b precursor	IPI00171412	0.378	0.13295	3	2	2	5
1221	Probable O-sialoglycoprotein endopeptidase	IPI00015809	0.378	0.13295	0	3	4	3
1222	Protein	IPI00892529	0.378	0.13295	3	0	0	5
1223	Isoform 1 of PDZ domain-containing protein 11	IPI00550841	0.378	0.13295	3	2	4	3
1224	Isovaleryl-CoA dehydrogenase, mitochondrial	IPI00645805	0.378	0.13295	3	2	4	3
1225	Protein LYRIC	IPI00328715	0.378	0.13295	2	3	2	5
1226	54 kDa protein	IPI00177890	0.378	0.13295	0	3	3	4
1227	Copper chaperone for superoxide dismutase	IPI00021389	0.378	0.13295	3	2	5	2
1228	Ras-related protein Ral-A	IPI00217519	0.378	0.13295	2	3	4	3
1229	Heat shock factor-binding protein 1	IPI00935516	0.378	0.13295	3	2	0	5

No.	Description	Accession number	STN[1]	p-Value[1]	480_A[2]	480_B[2]	620_A[2]	620_B[2]
1230	Transducin beta-like protein 2	IPI00000948	0.378	0.13295	2	3	4	3
1231	Calcium-binding mitochondrial carrier protein Aralar1	IPI00386271	0.378	0.13295	0	3	3	4
1232	Thioredoxin-like protein 4B	IPI00016481	0.378	0.13295	3	2	3	4
1233	transcriptional regulator ATRX isoform 2	IPI00220109	0.369	0.13319	2	4	0	6
1234	RNA 3'-terminal phosphate cyclase-like protein	IPI00294229	0.369	0.13319	2	4	5	3
1235	Ribonucleases P/MRP protein subunit POP1	IPI00293331	0.369	0.13319	3	3	4	4
1236	Isoform 1 of Endophilin-B1	IPI00006558	0.369	0.13319	4	0	5	3
1237	DNA repair protein complementing XP-C cells	IPI00156793	0.369	0.13319	3	3	4	4
1238	Glutamate-rich WD repeat-containing protein 1	IPI00027831	0.369	0.13319	3	3	4	4
1239	Isoform 1 of Regulation of nuclear pre-mRNA domain-containing protein 2	IPI00384541	0.369	0.13319	2	4	3	5
1240	Isoform 1 of General transcription factor 3C polypeptide 3	IPI00015806	0.369	0.13319	3	3	3	5
1241	Poly(ADP-ribose) glycohydrolase ARH3	IPI00015865	0.369	0.13319	3	3	4	4
1242	Protein S100-A13	IPI00016179	0.369	0.13319	4	0	4	4
1243	BAG family molecular chaperone regulator 2	IPI00000643	0.361	0.14595	4	3	3	6
1244	Isoform 2C of Cytoplasmic dynein 1 intermediate chain 2	IPI00216348	0.361	0.14595	2	5	3	6
1245	Isoform 1 of HEAT repeat-containing protein 2	IPI00242630	0.361	0.14595	4	3	5	4
1246	Isoform Long of ES1 protein homolog, mitochondrial	IPI00024913	0.361	0.14595	5	2	5	4
1247	Isoform Short of TATA-binding protein-associated factor 2N	IPI00020194	0.361	0.14595	5	0	5	4
1248	Putative uncharacterized protein ATP5J2	IPI00219291	0.361	0.14595	3	4	4	5
1249	Cytochrome c oxidase subunit 5B, mitochondrial	IPI00021785	0.361	0.14595	4	3	5	4
1250	cDNA FLJ38069 fis, clone CTONG2015434, highly similar to DOUBLE-STRAND BREAK REPAIR PROTEIN	IPI00029159	0.361	0.14595	4	3	5	4
1251	Sperm-associated antigen 7	IPI00006863	0.361	0.14595	3	4	5	4
1252	Isoform 1 of Elongation factor Tu GTP-binding domain-containing protein 1	IPI00293026	0.361	0.14595	4	3	4	5
1253	Synapse-associated protein 1	IPI00059242	0.361	0.14595	3	4	4	5
1254	40S ribosomal protein S20	IPI00012493	0.355	0.14612	4	4	5	5
1255	Isoform 3 of Transcription elongation factor SPT6	IPI00456683	0.355	0.14612	6	0	5	5
1256	Isoform 2 of Nucleosome-remodeling factor subunit BPTF	IPI00254408	0.355	0.14612	6	2	3	7
1257	Isoform 1 of Metaxin-1	IPI00013678	0.355	0.14612	4	4	4	6
1258	Protein FAM98B	IPI00167572	0.355	0.14612	4	4	5	5
1259	cDNA FLJ78497	IPI00289535	0.355	0.14612	4	4	5	5
1260	WD repeat-containing protein 82	IPI00152695	0.355	0.14612	5	3	5	5
1261	Dihydrofolate reductase	IPI00030357	0.355	0.14612	4	4	5	5
1262	Isoform 2 of Ubiquitin-conjugating enzyme E2 K	IPI00019894	0.355	0.14612	4	4	5	5
1263	Hsp90 co-chaperone Cdc37	IPI00013122	0.355	0.14612	5	3	5	5
1264	cDNA FLJ56180, highly similar to Negative elongation factor E	IPI00000858	0.355	0.14612	3	5	5	5
1265	Coiled-coil domain-containing protein 124	IPI00060627	0.349	0.15451	4	5	4	7
1266	Splicing factor 3B subunit 4	IPI00017339	0.349	0.15451	4	5	5	6
1267	Signal recognition particle 72 kDa protein	IPI00215888	0.349	0.15451	4	5	5	6
1268	Isoform 1 of tRNA-nucleotidyltransferase 1, mitochondrial	IPI00289807	0.349	0.15451	5	4	7	4
1269	cAMP-dependent protein kinase type I-alpha regulatory subunit	IPI00021831	0.349	0.15451	5	4	5	6
1270	Isoform 1 of Inorganic pyrophosphatase 2, mitochondrial	IPI00301109	0.344	0.15458	6	4	5	7
1271	Aminoacyl tRNA synthase complex-interacting multifunctional protein 1	IPI00006252	0.344	0.15458	4	6	7	5
1272	Phosphomevalonate kinase	IPI00220648	0.344	0.15458	5	5	7	5
1273	60S ribosomal protein L30	IPI00219156	0.344	0.15458	6	4	7	5
1274	Isoform 1 of 39S ribosomal protein L4, mitochondrial	IPI00023334	0.339	0.16139	6	5	7	6
1275	cDNA FLJ10824 fis, clone NT2RP4001086 (Fragment)	IPI00294810	0.339	0.16139	5	6	5	8
1276	Uncharacterized protein C7orf50	IPI00031651	0.339	0.16139	6	5	7	6
1277	COP9 signalosome complex subunit 8	IPI00009480	0.339	0.16139	6	5	5	8
1278	Isoform 1 of DNA primase large subunit	IPI00027705	0.339	0.16139	4	7	7	6
1279	EF-hand domain-containing protein D2	IPI00060181	0.335	0.16139	7	5	7	7
1280	Isoform 1 of Protein CDV3 homolog	IPI00014197	0.335	0.16139	7	5	5	9
1281	Protein FAM49B	IPI00303318	0.335	0.16139	6	6	7	7
1282	Isoform 1 of Protein canopy homolog 2	IPI00443909	0.335	0.16139	5	7	7	7
1283	N(G),N(G)-dimethylarginine dimethylaminohydrolase 2	IPI00000760	0.335	0.16139	5	7	7	7
1284	Serine/threonine-protein phosphatase PP1-beta catalytic subunit	IPI00218236	0.335	0.16139	6	6	7	7
1285	CDGSH iron sulfur domain-containing protein 1	IPI00020510	0.335	0.16139	6	6	7	7
1286	Pirin	IPI00012575	0.331	0.16731	5	8	6	9
1287	UPF0553 protein C9orf64	IPI00170972	0.331	0.16731	6	7	8	7
1288	28S ribosomal protein S23, mitochondrial	IPI00032881	0.331	0.16731	7	6	8	7
1289	Isoform 1 of Vesicle-associated membrane protein-associated protein A	IPI00170692	0.331	0.16731	6	7	9	6
1290	Signal peptidase complex catalytic subunit SEC11A	IPI00104128	0.331	0.16731	6	7	8	7
1291	Isoform 2 of PERQ amino acid-rich with GYF domain-containing protein 2	IPI00647635	0.331	0.16731	5	8	7	8
1292	Isoform 2 of Ubiquilin-1	IPI00071180	0.331	0.16731	6	7	9	6
1293	Isoform 1 of Mitochondrial import receptor subunit TOM40 homolog	IPI00014053	0.327	0.16738	7	7	7	9
1294	PNAS-139	IPI00000477	0.327	0.16738	6	8	9	7
1295	Actin-related protein 2/3 complex subunit 2	IPI00005161	0.327	0.16738	7	7	9	7
1296	Nucleolar complex protein 3 homolog	IPI00102815	0.327	0.16738	8	6	9	7
1297	pyruvate dehydrogenase E1 alpha 1 isoform 2 precursor	IPI00306301	0.324	0.17157	9	6	9	8
1298	Ephrin type-A receptor 2	IPI00021267	0.321	0.17157	8	8	9	9
1299	Isoform 1 of Cullin-4B	IPI00179057	0.321	0.17157	5	11	9	9
1300	COP9 signalosome complex subunit 3	IPI00025721	0.321	0.17157	8	8	9	9
1301	Isoform Short of Proteasome subunit alpha type-1	IPI00016832	0.316	0.17525	10	8	11	9
1302	Proteasome subunit beta type-7	IPI00003217	0.316	0.17525	10	8	8	12
1303	Exportin-5	IPI00640703	0.316	0.17525	10	8	9	11
1304	Glutaredoxin-3	IPI00008552	0.313	0.17894	13	6	14	7
1305	Isoform 1 of Nuclear pore complex protein Nup98-Nup96	IPI00006038	0.311	0.17894	9	11	10	12
1306	cDNA FLJ60076, highly similar to ELAV-like protein 1	IPI00301936	0.311	0.17894	10	10	10	12
1307	Proteasome subunit beta type-5	IPI00479306	0.311	0.17894	9	11	11	11
1308	cDNA FLJ51909, highly similar to Serine-threonine kinase receptor-associatedprotein	IPI00294536	0.309	0.18107	10	11	15	8
1309	Importin-9	IPI00185146	0.309	0.18107	8	13	12	11
1310	Isoform 2 of Apoptosis inhibitor 5	IPI00554742	0.309	0.18107	11	10	12	11
1311	Trifunctional enzyme subunit beta, mitochondrial	IPI00022793	0.309	0.18107	12	9	11	12
1312	Isoform 2 of Tropomyosin alpha-3 chain	IPI00218319	0.306	0.18107	10	12	11	13
1313	Membrane-associated progesterone receptor component 1	IPI00220739	0.304	0.18313	13	10	14	11
1314	40S ribosomal protein S16	IPI00221092	0.296	0.18667	17	11	14	16
1315	Isoform 1 of Hexokinase-1	IPI00018246	0.294	0.18750	13	16	14	17
1316	Tu translation elongation factor, mitochondrial precursor	IPI00027107	0.291	0.18860	17	14	15	18
1317	Proteasome subunit beta type-2	IPI00028006	0.291	0.18860	13	18	16	17
1318	F-actin-capping protein subunit alpha-1	IPI00005969	0.288	0.18980	19	14	18	17
1319	Isoform 1 of Nucleoside diphosphate kinase B	IPI00026260	0.282	0.19115	20	18	21	19
1320	Ras-related protein Rab-11B	IPI00020436	0.281	0.19177	20	19	20	21
1321	26S proteasome non-ATPase regulatory subunit 3	IPI00011603	0.281	0.19177	18	21	19	22
1322	Proteasome 26S non-ATPase subunit 11 variant (Fragment)	IPI00105598	0.281	0.19177	19	20	23	18
1323	Eukaryotic translation initiation factor 2 subunit 1	IPI00219678	0.262	0.19490	31	31	32	32

No.	Description	Accession number	STN[1]	p-Value[1]	480_A[2]	480_B[2]	620_A[2]	620_B[2]
1324	NAD(P)H dehydrogenase [quinone] 1	IPI00012069	0.252	0.19541	40	40	43	39
1325	NADH-ubiquinone oxidoreductase chain 4	IPI00008495	0.197	0.19710	0	0	3	2
1326	Isoform 1 of ER lumen protein retaining receptor 3	IPI00013872	0.197	0.19710	0	2	2	3
1327	Isoform 1 of Neuroblastoma-amplified sequence	IPI00333913	0.197	0.19710	2	2	3	2
1328	COP9 signalosome complex subunit 6	IPI00163230	0.197	0.19710	2	0	3	2
1329	Isoform 1 of Multidrug resistance-associated protein 4	IPI00006675	0.197	0.19710	2	2	3	2
1330	Isoform 2 of Condensin-2 complex subunit G2	IPI00396058	0.197	0.19710	2	2	0	3
1331	Protein SCO1 homolog, mitochondrial	IPI00027233	0.197	0.19710	0	0	3	2
1332	Peroxisomal membrane protein PMP34	IPI00014440	0.197	0.19710	0	0	2	3
1333	Serine/threonine-protein kinase 12	IPI00176642	0.197	0.19710	2	0	3	0
1334	Exportin-6	IPI00465296	0.197	0.19710	0	0	2	3
1335	Nucleolar protein 9	IPI00002902	0.197	0.19710	0	0	2	3
1336	Adenylyl cyclase-associated protein	IPI00939159	0.197	0.19710	2	2	3	2
1337	Isoform 2 of Isopentenyl-diphosphate Delta-isomerase 1	IPI00220014	0.197	0.19710	2	0	0	3
1338	Isoform 1 of Vacuolar protein sorting-associated protein 8 homolog	IPI00464985	0.197	0.19710	0	2	3	2
1339	Isoform UBF1 of Nucleolar transcription factor 1	IPI00014533	0.197	0.19710	0	0	2	3
1340	DEAH (Asp-Glu-Ala-His) box polypeptide 16	IPI00292510	0.197	0.19710	0	2	2	3
1341	Isoform 1 of Transmembrane protein 41B	IPI00555703	0.197	0.19710	0	0	0	3
1342	Antigen peptide transporter 1	IPI00646625	0.197	0.19710	0	0	3	2
1343	Methylosome protein 50	IPI00012202	0.197	0.19710	2	0	3	0
1344	Neurolysin, mitochondrial	IPI00010346	0.197	0.19710	0	0	0	3
1345	COMM domain-containing protein 2	IPI00456048	0.197	0.19710	0	2	3	0
1346	Macrophage migration inhibitory factor	IPI00293276	0.197	0.19710	0	0	0	3
1347	Nuclear transport factor 2	IPI00009901	0.197	0.19710	0	0	0	3
1348	Isoform 2 of DnaJ homolog subfamily A member 3, mitochondrial	IPI00179187	0.197	0.19710	2	2	2	3
1349	39S ribosomal protein L44, mitochondrial	IPI00009680	0.197	0.19710	0	0	3	0
1350	Protein SEC13 homolog	IPI00375370	0.197	0.19710	0	0	0	3
1351	Transmembrane protein 49	IPI00062469	0.197	0.19710	2	0	3	2
1352	Isoform 3 of Nucleoporin NDC1	IPI00074330	0.197	0.19710	0	0	3	2
1353	Calcium-regulated heat stable protein 1	IPI00304409	0.197	0.19710	2	0	3	2
1354	Cytosolic Fe-S cluster assembly factor NUBP2	IPI00644674	0.197	0.19710	0	0	3	2
1355	Putative uncharacterized protein EIF4E2	IPI00556081	0.197	0.19710	0	2	3	0
1356	Syntaxin-binding protein 3	IPI00297626	0.197	0.19710	0	2	0	3
1357	Isoform 1 of FAD synthase	IPI00220299	0.197	0.19710	2	0	2	3
1358	Werner syndrome ATP-dependent helicase	IPI00029107	0.197	0.19710	0	0	0	3
1359	Isoform 1 of Creatine kinase U-type, mitochondrial	IPI00658109	0.197	0.19710	2	2	3	2
1360	NADH dehydrogenase [ubiquinone] 1 beta subcomplex subunit 7	IPI00219772	0.197	0.19710	0	0	3	0
1361	Protein SGT1	IPI00027034	0.197	0.19710	0	0	3	2
1362	Peroxisomal membrane protein 2	IPI00221002	0.197	0.19710	0	2	3	2
1363	Cathepsin Z	IPI00002745	0.197	0.19710	2	2	3	0
1364	Dimethyladenosine transferase 1, mitochondrial	IPI00291525	0.197	0.19710	0	0	3	2
1365	Isoform 1 of Nicastrin	IPI00021983	0.197	0.19710	0	0	3	2
1366	Isoform 1 of Microtubule-associated protein 4	IPI00396171	0.197	0.19710	0	0	2	3
1367	60S ribosomal protein L35	IPI00412607	0.197	0.19710	2	2	2	3
1368	119 kDa protein	IPI00297178	0.197	0.19710	0	2	0	3
1369	Telomere length regulation protein TEL2 homolog	IPI00016868	0.197	0.19710	0	0	0	3
1370	Isoform 2 of Epimerase family protein SDR39U1	IPI00643286	0.197	0.19710	2	2	3	2
1371	Protein CWC15 homolog	IPI00009009	0.197	0.19710	0	0	0	3
1372	Cytokine-like nuclear factor n-pac, isoform CRA_a	IPI00000155	0.197	0.19710	0	0	0	3
1373	Protein QIL1	IPI00329373	0.197	0.19710	2	2	2	3
1374	Isoform 2 of Transcription elongation factor A protein 1	IPI00218106	0.197	0.19710	0	2	2	3
1375	Isoform 1 of Fermitin family homolog 1	IPI00304754	0.197	0.19710	0	0	3	0
1376	Methylmalonate-semialdehyde dehydrogenase [acylating], mitochondria	IPI00024990	0.197	0.19710	0	2	3	2
1377	Isoform 1 of Porphobilinogen deaminase	IPI00028160	0.197	0.19710	2	2	0	3
1378	DNA polymerase epsilon subunit 3	IPI00010141	0.197	0.19710	2	0	0	3
1379	Uncharacterized protein C10orf58	IPI00296190	0.197	0.19710	2	2	2	3
1380	Isoform 1 of Serine/threonine-protein phosphatase 2A 55 kDa regulatory subunit B beta isoform	IPI00020850	0.197	0.19710	0	0	0	3
1381	Similar to Zinc finger CCCH domain-containing protein 15	IPI00000279	0.197	0.19710	2	2	2	3
1382	Isoform 1 of Pentatricopeptide repeat-containing protein 3, mitochondrial	IPI00783302	0.197	0.19710	2	2	2	3
1383	39S ribosomal protein L53, mitochondrial	IPI00061531	0.197	0.19710	0	2	3	2
1384	YEATS domain-containing protein 4	IPI00008536	0.197	0.19710	2	2	2	3
1385	Isoform 2A of GTPase KRas	IPI00423568	0.197	0.19710	0	0	3	0
1386	Isoform 1 of Actin-related protein 3B	IPI00007068	0.197	0.19710	0	0	0	3
1387	Isoform 2 of Membrane magnesium transporter 1	IPI00166785	0.197	0.19710	2	2	3	2
1388	NADH dehydrogenase [ubiquinone] 1 alpha subcomplex subunit 2	IPI00219381	0.197	0.19710	0	0	3	0
1389	Isoform 2 of Actin-related protein 2/3 complex subunit 5	IPI00007280	0.197	0.19710	2	0	2	3
1390	ATP-dependent RNA helicase DDX54 isoform 1	IPI00152510	0.197	0.19710	0	0	3	2
1391	UPF0554 protein C2orf43	IPI00030257	0.197	0.19710	0	2	3	2
1392	Coiled-coil domain-containing protein 58	IPI00046828	0.197	0.19710	0	0	3	2
1393	Isoform 2 of SWI/SNF-related matrix-associated actin-dependent regulator of chromatin subfamily A	IPI00008422	0.197	0.19710	0	0	3	0
1394	Acidic leucine-rich nuclear phosphoprotein 32 family member A	IPI00025849	0.197	0.19710	0	0	3	0
1395	Isoform 1 of Anaphase-promoting complex subunit 4	IPI00002551	0.197	0.19710	0	0	2	3
1396	Transmembrane and coiled-coil domain-containing protein 7	IPI00034201	0.197	0.19710	0	0	0	3
1397	Transcription initiation factor TFIID subunit 2	IPI00328144	0.197	0.19710	0	0	2	3
1398	Isoform 1 of COP9 signalosome complex subunit 1	IPI00156282	0.197	0.19710	0	0	0	3
1399	Isoform 1 of TraB domain-containing protein	IPI00008732	0.197	0.19710	0	0	2	3
1400	SAFB-like transcription modulator isoform b	IPI00019996	0.197	0.19710	2	0	2	3
1401	RAB4A, member RAS oncogene family variant	IPI00480056	0.197	0.19710	2	2	2	3
1402	DNA replication complex GINS protein PSF2	IPI00007146	0.197	0.19710	0	2	3	2
1403	Pre-mRNA-splicing factor SYF2	IPI00022963	0.197	0.19710	0	0	2	3
1404	ERBB2IP protein	IPI00438286	0.197	0.19710	2	2	3	0
1405	Trans-2-enoyl-CoA reductase, mitochondrial	IPI00306159	0.197	0.19710	2	0	3	0
1406	Ribosomal protein S6 kinase alpha-6	IPI00007123	0.197	0.19710	0	0	0	3
1407	Renin receptor	IPI00168884	0.197	0.19710	0	0	2	3
1408	Probable RNA-binding protein 19	IPI00000686	0.197	0.19710	0	2	0	3
1409	telomerase-binding protein EST1A isoform 2	IPI00014252	0.197	0.19710	0	0	2	3
1410	28S ribosomal protein S30, mitochondrial	IPI00010278	0.197	0.19710	0	2	3	2
1411	Ketosamine-3-kinase	IPI00099986	0.197	0.19710	2	2	3	0
1412	Isoform 6 of Ribosome-recycling factor, mitochondrial	IPI00030596	0.197	0.19710	2	2	3	2
1413	Transcription elongation factor B polypeptide 1	IPI00300341	0.197	0.19710	0	0	3	0
1414	Isoform 2 of Dynactin subunit 3	IPI00013654	0.197	0.19710	0	2	3	2
1415	Isoform 1 of IST1 homolog	IPI00024660	0.197	0.19710	0	2	2	3
1416	Ubiquitin carboxyl-terminal hydrolase 13	IPI00024401	0.197	0.19710	2	2	2	3
1417	Neuron-specific calcium-binding protein hippocalcin	IPI00219103	0.197	0.19710	0	0	3	2

No.	Description	Accession number	STN[1]	p-Value[1]	480_A[2]	480_B[2]	620_A[2]	620_B[2]
1418	DNA primase small subunit	IPI00027704	0.197	0.19710	0	2	0	3
1419	Translocation protein SEC63 homolog	IPI00218922	0.197	0.19710	2	0	3	2
1420	Isoform 1 of Nucleoporin p58/p45	IPI00107122	0.197	0.19710	0	0	3	2
1421	EF-hand domain-containing family member A1	IPI00640276	0.197	0.19710	0	0	2	3
1422	Isoform 1 of Protein FAM65A	IPI00418799	0.197	0.19710	0	0	3	0
1423	Cation-independent mannose-6-phosphate receptor	IPI00289819	0.197	0.19710	0	0	3	2
1424	Protein LTV1 homolog	IPI00153032	0.197	0.19710	0	0	2	3
1425	COMM domain-containing protein 10	IPI00412811	0.197	0.19710	0	2	2	3
1426	H/ACA ribonucleoprotein complex subunit 2	IPI00041325	0.197	0.19710	0	0	3	0
1427	Isoform 2 of Alpha-endosulfine	IPI00220797	0.197	0.19710	0	0	3	0
1428	Isoform 1 of Kinesin-like protein KIF21A	IPI00425404	0.197	0.19710	0	0	3	0
1429	Aldo-keto reductase family 1 member C2	IPI00005668	0.197	0.19710	0	0	0	3
1430	Vacuolar protein sorting-associated protein VTA1 homolog	IPI00017160	0.197	0.19710	0	2	3	2
1431	Aldo-keto reductase family 1 member C3	IPI00291483	0.197	0.19710	0	0	2	3
1432	ubiquitin-like protein fubi and ribosomal protein S30 precursor	IPI00019770	0.197	0.19710	0	0	2	3
1433	Cell division cycle protein 123 homolog	IPI00005670	0.197	0.19710	0	0	2	3
1434	WD repeat domain 57 (U5 snRNP specific), isoform CRA_b	IPI00385642	0.197	0.19710	0	0	3	0
1435	Isoform 2 of Vacuolar protein sorting-associated protein 53 homolog	IPI00181824	0.197	0.19710	2	0	0	3
1436	Isoform 2 of Torsin-1A-interacting protein 1	IPI00012280	0.197	0.19710	0	0	3	0
1437	Protein LLP homolog	IPI00031615	0.197	0.19710	0	0	0	3
1438	Coiled-coil domain-containing protein 127	IPI00060148	0.197	0.19710	2	2	3	0
1439	Serine/threonine-protein phosphatase 2A 56 kDa regulatory subunit alpha isoform	IPI00014978	0.197	0.19710	0	0	3	0
1440	Isoform 5 of Brain-specific angiogenesis inhibitor 1-associated protein 2	IPI00180292	0.197	0.19710	0	0	0	3
1441	E3 ubiquitin-protein ligase CBL	IPI00027269	0.197	0.19710	0	0	2	3
1442	Isoform 1 of Protein NDRG3	IPI00005605	0.197	0.19710	0	0	2	3
1443	N-acetylgalactosaminyltransferase 7	IPI00328391	0.197	0.19710	0	0	0	3
1444	Origin recognition complex subunit 6	IPI00001641	0.197	0.19710	0	0	3	0
1445	Activator of basal transcription 1	IPI00002938	0.197	0.19710	0	0	0	3
1446	Isoform 3 of F-box only protein 22	IPI00169168	0.197	0.19710	2	0	3	2
1447	Isoform 3 of Yorkie homolog	IPI00009326	0.197	0.19710	0	0	3	0
1448	Isoform 1 of WD repeat-containing protein 74	IPI00018192	0.197	0.19710	0	2	3	2
1449	U3 small nucleolar RNA-interacting protein 2	IPI00217862	0.197	0.19710	2	2	3	2
1450	Protein MAK16 homolog	IPI00332428	0.197	0.19710	0	0	3	2
1451	Isoform 2 of NAD-dependent deacetylase sirtuin-5	IPI00010331	0.197	0.19710	2	0	3	2
1452	UPF0534 protein C4orf43	IPI00019962	0.197	0.19710	0	0	0	3
1453	U4/U6.U5 small nuclear ribonucleoprotein 27 kDa protein	IPI00017289	0.197	0.19710	0	0	3	2
1454	25 kDa protein	IPI00010276	0.197	0.19710	0	0	3	2
1455	Isoform 2 of 39S ribosomal protein L55, mitochondrial	IPI00419626	0.197	0.19710	2	0	3	2
1456	Transmembrane protein C9orf46	IPI00307547	0.197	0.19710	0	0	0	3
1457	Uncharacterized protein C11orf84	IPI00106955	0.197	0.19710	0	0	0	3
1458	Isoform 1 of tRNA (guanine-N(7)-)-methyltransferase subunit WDR4	IPI00025718	0.197	0.19710	0	0	3	2
1459	Isoform 1 of Chaperone activity of bc1 complex-like, mitochondrial	IPI00176469	0.197	0.19710	2	2	3	0
1460	Uncharacterized protein C20orf72	IPI00001287	0.197	0.19710	0	0	3	2
1461	Isoform 1 of Probable threonyl-tRNA synthetase 2, cytoplasmic	IPI00328082	0.197	0.19710	0	0	3	2
1462	Isoform 1 of Peroxisomal biogenesis factor 19	IPI00441867	0.197	0.19710	0	0	0	3
1463	Isoform 1 of Tropomyosin beta chain	IPI00013991	0.197	0.19710	0	0	3	0
1464	Splicing factor 45	IPI00176706	0.197	0.19710	0	0	3	0
1465	sorting nexin-6 isoform a	IPI00258833	0.197	0.19710	0	0	2	3
1466	Isoform 1 of Vacuolar protein sorting-associated protein 16 homolog	IPI00305438	0.197	0.19710	0	0	0	3
1467	Isoform 1 of Rab GTPase-binding effector protein 1	IPI00293009	0.197	0.19710	2	0	3	2
1468	60 kDa protein	IPI00053288	0.197	0.19710	2	2	3	2
1469	Isoform 1 of Ran guanine nucleotide release factor	IPI00025081	0.197	0.19710	0	0	2	3
1470	Isoform 2 of Arf-GAP domain and FG repeats-containing protein 1	IPI00304693	0.197	0.19710	2	0	3	0
1471	Isoform 1 of Coronin-7	IPI00027996	0.197	0.19710	0	0	2	3
1472	1-phosphatidylinositol-4,5-bisphosphate phosphodiesterase delta-3	IPI00152701	0.197	0.19710	0	0	2	3
1473	Isoform 1 of Mixed lineage kinase domain-like protein	IPI00180781	0.197	0.19710	0	0	3	0
1474	ZW10 interactor	IPI00294008	0.197	0.19710	0	0	0	3
1475	Uncharacterized protein C7orf44	IPI00414548	0.197	0.19710	0	0	2	3
1476	Isoform 1 of GPN-loop GTPase 3	IPI00470580	0.197	0.19710	0	0	0	3
1477	Atlastin-1	IPI00103530	0.197	0.19710	0	0	2	3
1478	Isoform 2 of Rho GTPase-activating protein 5	IPI00013988	0.197	0.19710	0	0	3	2
1479	Isoform 2 of Kinesin-like protein KIF2C	IPI00216113	0.197	0.19710	0	0	0	3
1480	DUS1L protein (Fragment)	IPI00329754	0.197	0.19710	0	0	2	3
1481	175 kDa protein	IPI00328318	0.197	0.19710	0	0	3	0
1482	Checkpoint protein HUS1	IPI00004712	0.197	0.19710	0	0	3	2
1483	UPF0552 protein C15orf38	IPI00074225	0.197	0.19710	0	0	2	3
1484	NADH dehydrogenase [ubiquinone] iron-sulfur protein 4, mitochondrial	IPI00011217	0.197	0.19710	0	0	0	3
1485	BRISC complex subunit Abro1	IPI00299517	0.197	0.19710	0	0	3	2
1486	Methionyl-tRNA synthetase, mitochondrial	IPI00062839	0.197	0.19710	0	0	3	0
1487	Isoform 1 of Protein POF1B	IPI00103242	0.197	0.19710	0	0	2	3
1488	cDNA FLJ11251 fis, clone PLACE1008813	IPI00010953	0.197	0.19710	0	0	0	3
1489	2-oxoisovalerate dehydrogenase subunit alpha, mitochondrial	IPI00025100	0.197	0.19710	0	0	3	0
1490	Guanine nucleotide-binding protein G(i) subunit alpha-1	IPI00337415	0.197	0.19710	0	0	3	0
1491	FH1/FH2 domain-containing protein 1	IPI00001730	0.197	0.19710	0	0	0	3
1492	Endonuclease G, mitochondrial	IPI00290614	0.197	0.19710	0	0	0	3
1493	Isoform 1 of L-2-hydroxyglutarate dehydrogenase, mitochondrial	IPI00016458	0.197	0.19710	0	0	0	3
1494	Probable tRNA pseudouridine synthase 1	IPI00103341	0.197	0.19710	0	0	3	2
1495	Isoform 1 of Acyl-CoA-binding domain-containing protein 5	IPI00186681	0.197	0.19710	0	0	0	3
1496	Acyl-CoA:lysophosphatidylglycerol acyltransferase 1	IPI00022447	0.197	0.19710	0	0	0	3
1497	Ras-related C3 botulinum toxin substrate 2	IPI00010270	0.191	0.21392	3	0	0	4
1498	Ribosomal protein L1	IPI00035167	0.191	0.21392	0	3	3	3
1499	Isoform 1 of Pescadillo homolog	IPI00003768	0.191	0.21392	3	2	0	4
1500	Serine/threonine-protein phosphatase 2A 56 kDa regulatory subunit epsilon isoform	IPI00002853	0.191	0.21392	2	3	4	2
1501	Isoform 1 of Lysophospholipase-like protein 1	IPI00059762	0.191	0.21392	0	3	3	3
1502	Alcohol dehydrogenase class-3	IPI00746777	0.191	0.21392	0	3	3	3
1503	ribonucleoprotein PTB-binding 1	IPI00217661	0.191	0.21392	3	0	3	3
1504	Isoform 2 of Transcription elongation factor SPT6	IPI00430770	0.191	0.21392	0	3	2	4
1505	Isoform 1 of Ubiquitin conjugation factor E4 B	IPI00005715	0.191	0.21392	0	3	0	4
1506	RER1 protein	IPI00005728	0.191	0.21392	3	2	3	3
1507	Dynein, light chain, roadblock-type 1	IPI00412497	0.191	0.21392	3	0	0	4
1508	Neudesin	IPI00002525	0.191	0.21392	3	2	3	3
1509	Thimet oligopeptidase	IPI00549189	0.191	0.21392	3	0	0	4
1510	Isoform 1 of Glomulin	IPI00074604	0.191	0.21392	3	2	3	3
1511	Isoform 1 of Tumor suppressor p53-binding protein 1	IPI00029778	0.191	0.21392	2	3	4	2
1512	Thiopurine S-methyltransferase	IPI00019400	0.191	0.21392	2	3	4	2

No.	Description	Accession number	STN[1]	p-Value[1]	480_A[2]	480_B[2]	620_A[2]	620_B[2]
1513	Small nuclear ribonucleoprotein Sm D3	IPI00017964	0.191	0.21392	2	3	4	0
1514	AP-3 complex subunit mu-1	IPI00032459	0.191	0.21392	3	2	3	3
1515	Ethanolamine-phosphate cytidylyltransferase	IPI00015285	0.191	0.21392	3	2	3	3
1516	Isoform 2 of Oxidoreductase HTATIP2	IPI00383665	0.191	0.21392	3	2	3	3
1517	Dual specificity mitogen-activated protein kinase kinase 1	IPI00219604	0.191	0.21392	3	2	3	3
1518	Phosphatidylinositol 4-kinase type 2-alpha	IPI00020124	0.191	0.21392	3	2	4	2
1519	Isoform 1 of Translation initiation factor eIF-2B subunit gamma	IPI00006504	0.191	0.21392	2	3	3	3
1520	Ribosomal protein S6 kinase alpha-1	IPI00017305	0.191	0.21392	3	0	4	2
1521	cDNA FLJ56277, highly similar to Toll-like receptor 9	IPI00219489	0.191	0.21392	2	3	3	3
1522	similar to RAN binding protein 1	IPI00399212	0.191	0.21392	3	2	3	3
1523	ATP synthase subunit g, mitochondrial	IPI00027448	0.191	0.21392	2	3	4	2
1524	C-Myc-binding protein	IPI00871174	0.191	0.21392	2	3	2	4
1525	Probable histidyl-tRNA synthetase, mitochondrial	IPI00027445	0.191	0.21392	3	0	3	3
1526	Nuclear envelope pore membrane protein POM 121C	IPI00032358	0.191	0.21392	3	2	2	4
1527	Nuclear pore complex protein Nup50	IPI00026940	0.191	0.21392	0	3	3	3
1528	Acylphosphatase-1	IPI00221117	0.191	0.21392	3	2	3	3
1529	Isoform 1 of Homologous-pairing protein 2 homolog	IPI00009805	0.191	0.21392	0	3	2	4
1530	Putative uncharacterized protein DKFZp313O211	IPI00552186	0.186	0.22520	4	2	4	3
1531	60S ribosomal protein L19	IPI00025329	0.186	0.22520	2	4	3	4
1532	rho GTPase-activating protein 4 isoform 1	IPI00328842	0.186	0.22520	2	4	3	4
1533	Cleavage stimulation factor subunit 3	IPI00015195	0.186	0.22520	2	4	2	5
1534	bullous pemphigoid antigen 1, isoform 7 isoform 3	IPI00008756	0.186	0.22520	3	3	2	5
1535	Caspase-3	IPI00292140	0.186	0.22520	3	3	3	4
1536	NADH dehydrogenase [ubiquinone] iron-sulfur protein 8, mitochondrial	IPI00010845	0.186	0.22520	3	3	3	4
1537	Isoform 1 of WD repeat-containing protein 44	IPI00444371	0.186	0.22520	3	3	2	5
1538	Isoform 3 of Cytosolic 5'-nucleotidase 3	IPI00100192	0.186	0.22520	2	4	2	5
1539	GDP-L-fucose synthase	IPI00014361	0.186	0.22520	3	3	3	4
1540	Isoform 1 of Alpha-adducin	IPI00019901	0.186	0.22520	2	4	3	4
1541	37 kDa protein	IPI00032799	0.186	0.22520	3	3	3	4
1542	V-type proton ATPase subunit d 1	IPI00034159	0.186	0.22520	3	3	3	4
1543	Isoform 1 of 28S ribosomal protein S5, mitochondrial	IPI00169400	0.186	0.22520	4	2	3	4
1544	Isoform 1 of RNA-binding protein with serine-rich domain 1	IPI00033561	0.186	0.22520	3	3	3	4
1545	Isoform 1 of Zinc finger protein 207	IPI00013457	0.186	0.22520	2	4	4	3
1546	Cystatin-B	IPI00021828	0.186	0.22520	4	2	4	3
1547	nucleoside diphosphate kinase type 6	IPI00218214	0.186	0.22520	3	3	3	4
1548	Survival of motor neuron-related-splicing factor 30	IPI00025176	0.186	0.22520	3	3	4	3
1549	U2 small nuclear ribonucleoprotein B''	IPI00029267	0.182	0.23139	3	4	4	4
1550	Isoform 1 of Adenylate kinase 2, mitochondrial	IPI00215901	0.182	0.23139	4	3	4	4
1551	Golgi phosphoprotein 3	IPI00005490	0.182	0.23139	4	3	4	4
1552	Scaffold attachment factor B2	IPI00005648	0.182	0.23139	4	3	4	4
1553	Ras-related protein Rap-2c	IPI00009607	0.182	0.23139	4	3	4	4
1554	U6 snRNA-associated Sm-like protein LSm4	IPI00294955	0.182	0.23139	4	3	4	4
1555	Isoform 1 of Drebrin	IPI00003406	0.182	0.23139	4	3	5	3
1556	Plakophilin-3	IPI00026952	0.182	0.23139	4	3	4	4
1557	Isoform 1 of Acyl-coenzyme A thioesterase 2, mitochondrial	IPI00220906	0.182	0.23139	3	4	4	4
1558	Isoform 1 of Erlin-2	IPI00026942	0.182	0.23139	4	3	4	4
1559	Ras-related protein Rab-22A	IPI00007756	0.182	0.23139	4	3	5	3
1560	Isoform 2 of NADH dehydrogenase [ubiquinone] flavoprotein 3, mitochondrial	IPI00291016	0.182	0.23139	3	4	4	4
1561	Isoform 1 of Regulator of nonsense transcripts 3B	IPI00023409	0.182	0.23139	3	4	4	4
1562	Isoform 2 of Double-stranded RNA-specific adenosine deaminase	IPI00025057	0.179	0.23586	4	4	5	4
1563	U4/U6.U5 tri-snRNP-associated protein 2	IPI00419844	0.179	0.23586	4	4	6	3
1564	Isoform 1 of Mitochondrial antiviral-signaling protein	IPI00020719	0.179	0.23586	4	4	6	3
1565	Amidophosphoribosyltransferase	IPI00029534	0.179	0.23586	2	6	5	4
1566	Serine/threonine-protein phosphatase 2A 55 kDa regulatory subunit B alpha isoform	IPI00332511	0.179	0.23586	4	4	4	5
1567	NAD-dependent malic enzyme, mitochondrial	IPI00011201	0.179	0.23586	4	4	4	5
1568	Putative uncharacterized protein DKFZp686E2459	IPI00375731	0.179	0.23586	4	4	5	4
1569	Isoform 1 of COMM domain-containing protein 4	IPI00413500	0.179	0.23586	5	3	5	4
1570	cDNA FLJ56840, highly similar to Galactokinase	IPI00019383	0.179	0.23586	4	4	4	5
1571	Isoform 2 of Ubiquitin-associated domain-containing protein 2	IPI00007034	0.179	0.23586	4	4	3	6
1572	E3 ubiquitin-protein ligase BRE1A	IPI00251559	0.179	0.23586	4	4	4	5
1573	Protein FAM162A	IPI00023001	0.179	0.23586	4	4	6	3
1574	Isoform 3 of Protein PRRC1	IPI00217053	0.179	0.23586	4	4	5	4
1575	Isoform 1 of Transmembrane and coiled-coil domain-containing protein 1	IPI00026111	0.176	0.24009	4	5	4	6
1576	Isoform 1 of Enolase-phosphatase E1	IPI00038378	0.176	0.24009	5	4	7	3
1577	Metaxin-2	IPI00025717	0.176	0.24009	5	4	5	5
1578	Isoform 1 of E3 UFM1-protein ligase 1	IPI00844000	0.176	0.24009	3	6	4	6
1579	Ewing sarcoma breakpoint region 1 isoform 1	IPI00009841	0.176	0.24009	5	4	6	4
1580	Isoform 1 of Serine/threonine-protein phosphatase 2A 65 kDa regulatory subunit A beta isoform	IPI00294178	0.176	0.24009	3	6	4	6
1581	Uncharacterized protein C2orf47, mitochondrial	IPI00291751	0.176	0.24009	4	5	4	6
1582	39S ribosomal protein L37, mitochondrial	IPI00162330	0.176	0.24009	4	5	5	5
1583	39S ribosomal protein L11, mitochondrial	IPI00007001	0.176	0.24009	4	5	5	5
1584	Protein TFG	IPI00294619	0.176	0.24009	5	4	3	7
1585	Vasodilator-stimulated phosphoprotein	IPI00301058	0.176	0.24009	3	6	5	5
1586	Putative uncharacterized protein CNOT1	IPI00032299	0.173	0.24319	6	4	6	5
1587	Sorcin	IPI00027175	0.173	0.24319	5	5	5	6
1588	cDNA FLJ60607, highly similar to Acyl-protein thioesterase 1	IPI00007321	0.173	0.24319	5	5	6	5
1589	Non-functional aryl hydrocarbon receptor interacting protein (Fragment)	IPI00925804	0.173	0.24319	4	6	6	5
1590	Isoform 2 of NSFL1 cofactor p47	IPI00022830	0.173	0.24319	5	5	5	6
1591	cDNA FLJ54536, highly similar to Mitochondrial 28S ribosomal protein S27	IPI00022002	0.173	0.24319	5	5	4	7
1592	cDNA FLJ55829, highly similar to Homo sapiens leucine zipper and CTNNBIP1 domain containing	IPI00152900	0.173	0.24319	5	5	5	6
1593	tropomyosin alpha-3 chain isoform 1	IPI00183968	0.173	0.24319	5	5	7	4
1594	Isoform 1 of RNA-binding protein 14	IPI00013174	0.173	0.24319	7	3	6	5
1595	Nucleoporin Nup43	IPI00742943	0.173	0.24319	5	5	6	5
1596	Nuclear pore glycoprotein p62	IPI00293533	0.173	0.24319	5	5	5	6
1597	60S ribosomal protein L27	IPI00219155	0.171	0.24629	7	4	3	9
1598	Vesicle-fusing ATPase	IPI00006451	0.171	0.24629	5	6	6	6
1599	DNA-directed RNA polymerases I, II, and III subunit RPABC1	IPI00291093	0.171	0.24629	6	5	6	6
1600	28S ribosomal protein S18b, mitochondrial	IPI00022316	0.171	0.24629	3	8	5	7
1601	Transmembrane protein 43	IPI00301280	0.171	0.24629	3	8	5	7
1602	Dolichyl-diphosphooligosaccharide--protein glycosyltransferase subunit STT3B	IPI00152377	0.168	0.24849	5	7	7	6
1603	Isoform 2 of Myosin-XVIIIa	IPI00334410	0.168	0.24849	4	8	7	6
1604	Transmembrane emp24 domain-containing protein 9	IPI00023542	0.168	0.24849	9	3	8	5
1605	Isoform 1 of UBX domain-containing protein 1	IPI00027378	0.168	0.24849	6	6	8	5
1606	Isoform 1 of Oligoribonuclease, mitochondrial (Fragment)	IPI00032830	0.168	0.24849	6	6	6	7
1607	Dihydropyrimidinase-related protein 2	IPI00257508	0.168	0.24849	4	8	7	6

No.	Description	Accession number	STN[1]	p-Value[1]	480_A[2]	480_B[2]	620_A[2]	620_B[2]
1608	RNA-binding protein 28	IPI00304187	0.168	0.24849	6	6	6	7
1609	Isoform 3 of THO complex subunit 6 homolog	IPI00301252	0.168	0.24849	7	5	7	6
1610	Galectin-3	IPI00465431	0.166	0.25138	7	6	6	8
1611	Phosphoribosyl pyrophosphate synthase-associated protein 2	IPI00003168	0.166	0.25138	6	7	6	8
1612	ADP-ribosylation factor 6	IPI00215920	0.166	0.25138	7	6	7	7
1613	Guanine nucleotide-binding protein subunit alpha-13	IPI00290928	0.166	0.25138	6	7	7	7
1614	Cytochrome b-c1 complex subunit 1, mitochondrial	IPI00013847	0.166	0.25138	7	6	8	6
1615	Putative high mobility group protein B3-like-1	IPI00006437	0.166	0.25138	7	6	8	6
1616	cDNA FLJ56357, highly similar to Homo sapiens apolipoprotein A-I binding protein (APOA1BP), mRNA	IPI00168479	0.166	0.25138	7	6	7	7
1617	Isoform 1 of Nucleolar protein 6	IPI00152890	0.165	0.25330	5	9	7	8
1618	SDHA protein	IPI00217143	0.165	0.25330	6	8	5	10
1619	Isoform 2 of Myosin-VI	IPI00008455	0.165	0.25330	7	7	6	9
1620	UPF0468 protein C16orf80	IPI00001655	0.165	0.25330	7	7	7	8
1621	Stathmin	IPI00479997	0.165	0.25330	9	5	9	6
1622	WD repeat-containing protein 75	IPI00217240	0.165	0.25330	7	7	7	8
1623	Signal recognition particle 14 kDa protein	IPI00293434	0.165	0.25330	6	8	7	8
1624	Coatomer subunit delta variant 2	IPI00298520	0.163	0.25526	8	7	7	9
1625	NADH dehydrogenase [ubiquinone] flavoprotein 2, mitochondrial	IPI00291328	0.163	0.25526	8	7	6	10
1626	Isoform Short of Glycylpeptide N-tetradecanoyltransferase 1	IPI00218830	0.163	0.25526	7	8	10	6
1627	Fructose-bisphosphate aldolase	IPI00418262	0.163	0.25526	8	7	6	10
1628	Small acidic protein	IPI00003419	0.163	0.25526	8	7	8	8
1629	Mitochondrial-processing peptidase subunit alpha	IPI00166749	0.161	0.25671	8	8	7	10
1630	Ribonuclease P protein subunit p30	IPI00019196	0.161	0.25671	9	7	8	9
1631	Ubiquitin-conjugating enzyme E2 O	IPI00783378	0.161	0.25671	6	10	7	10
1632	cDNA FLJ55382, highly similar to Hsp70-binding protein 1	IPI00100748	0.161	0.25671	7	9	7	10
1633	14-3-3 protein eta	IPI00216319	0.161	0.25671	9	7	10	7
1634	WD repeat-containing protein 61	IPI00019269	0.160	0.25795	10	7	9	9
1635	Epithelial cell adhesion molecule	IPI00296215	0.160	0.25795	9	8	11	7
1636	Serine/threonine-protein kinase PAK 2	IPI00419979	0.158	0.25870	9	9	10	9
1637	60S ribosomal protein L36	IPI00216237	0.158	0.25870	8	10	11	8
1638	Isoform 2 of Obg-like ATPase 1	IPI00216105	0.157	0.25994	9	10	9	11
1639	Coatomer subunit zeta-1	IPI00032851	0.157	0.25994	9	10	10	10
1640	Cell division cycle 5-like protein	IPI00465294	0.157	0.25994	10	9	9	11
1641	Isoform 1 of Ubiquitin-conjugating enzyme E2 variant 1	IPI00019599	0.157	0.25994	9	10	9	11
1642	Isoform 1 of Abhydrolase domain-containing protein 14B	IPI00063827	0.155	0.26163	11	10	9	13
1643	ATP synthase subunit O, mitochondrial	IPI00007611	0.155	0.26163	12	9	11	11
1644	GTP:AMP phosphotransferase mitochondrial	IPI00465256	0.153	0.26314	11	12	12	12
1645	Isoform 1 of Heterogeneous nuclear ribonucleoprotein D0	IPI00028888	0.152	0.26359	13	11	11	14
1646	Activator of 90 kDa heat shock protein ATPase homolog 1	IPI00030706	0.150	0.26520	13	13	12	15
1647	Mitochondrial import receptor subunit TOM22 homolog	IPI00024976	0.150	0.26520	14	12	13	14
1648	Isoform Mitochondrial of Fumarate hydratase, mitochondrial	IPI00296053	0.150	0.26520	15	11	13	14
1649	rRNA 2'-O-methyltransferase fibrillarin	IPI00025039	0.149	0.26562	16	11	17	11
1650	Nodal modulator 1	IPI00329352	0.149	0.26562	11	16	14	14
1651	cysteinyl-tRNA synthetase, cytoplasmic isoform c	IPI00027443	0.149	0.26562	14	13	14	14
1652	Isoform 1 of Caprin-1	IPI00783872	0.147	0.26644	18	11	19	11
1653	Chromobox protein homolog 3	IPI00297579	0.147	0.26644	13	16	15	15
1654	Leucine-rich repeat-containing protein 59	IPI00396321	0.147	0.26672	14	16	14	17
1655	Actin-related protein 2	IPI00005159	0.146	0.26713	15	16	15	17
1656	Isoform 1 of Cytosolic acyl coenzyme A thioester hydrolase	IPI00010415	0.145	0.26796	16	17	16	18
1657	Leukocyte elastase inhibitor	IPI00027444	0.144	0.26830	17	17	17	18
1658	THO complex subunit 4	IPI00328840	0.143	0.26840	19	16	19	17
1659	Dihydrolipoyl dehydrogenase, mitochondrial	IPI00015911	0.143	0.26840	18	17	19	17
1660	Isoform 1 of Proteasome subunit alpha type-7	IPI00024175	0.141	0.26916	21	17	19	20
1661	Phosphoserine aminotransferase	IPI00001734	0.141	0.26916	18	20	19	20
1662	Proteasome subunit alpha type-2	IPI00219622	0.140	0.26971	21	19	20	21
1663	Lamin-B1	IPI00217975	0.140	0.26971	20	20	20	21
1664	Protein DJ-1	IPI00298547	0.137	0.27091	22	25	24	24
1665	Sodium/potassium-transporting ATPase subunit alpha-2	IPI00003021	0.137	0.27091	27	20	27	21
1666	Isoform 2 of Structural maintenance of chromosomes protein 4	IPI00328298	0.132	0.27201	29	30	29	31
1667	Heat shock 70 kDa protein 4	IPI00002966	0.121	0.27329	53	51	49	56
1668	Isoform 2 of Nucleophosmin	IPI00220740	0.118	0.27373	58	65	58	66
1669	Isoform 1 of Heat shock cognate 71 kDa protein	IPI00003865	0.108	0.27404	111	110	112	110
1670	Histone H3.2	IPI00171611	0.000	0.27422	2	3	3	2
1671	Proteasome subunit beta type-1	IPI00025019	0.000	0.27422	24	24	26	22
1672	Isoform 2 of Structural maintenance of chromosomes flexible hinge domain-containing protein 1	IPI00465022	0.000	0.27422	19	21	20	20
1673	Inosine-5'-monophosphate dehydrogenase 2	IPI00291510	0.000	0.27422	19	18	18	19
1674	Putative uncharacterized protein PSME2	IPI00384051	0.000	0.27422	22	21	22	21
1675	Programmed cell death 6-interacting protein	IPI00246058	0.000	0.27422	23	23	23	23
1676	Single-stranded DNA-binding protein, mitochondrial	IPI00029744	0.000	0.27422	15	9	12	12
1677	Isoform 1 of Regulator of nonsense transcripts 1	IPI00034049	0.000	0.27422	11	15	13	13
1678	60S ribosomal protein L18a	IPI00026202	0.000	0.27422	5	5	4	6
1679	SUMO-activating enzyme subunit 2	IPI00023234	0.000	0.27422	11	12	11	12
1680	26S proteasome non-ATPase regulatory subunit 14	IPI00024821	0.000	0.27422	19	14	15	18
1681	Isoform 1 of Nuclear autoantigenic sperm protein	IPI00179953	0.000	0.27422	14	12	12	14
1682	HEAT repeat containing 7B1	IPI00216817	0.000	0.27422	2	0	0	0
1683	26S protease regulatory subunit S10B	IPI00021926	0.000	0.27422	12	15	13	14
1684	Isoform 1 of AP-2 complex subunit beta	IPI00784156	0.000	0.27422	16	13	14	15
1685	Importin subunit alpha-2	IPI00002214	0.000	0.27422	11	12	12	11
1686	regulator of differentiation 1 isoform 2	IPI00159072	0.000	0.27422	2	0	2	0
1687	Isoform 1 of Phosphatidylinositol transfer protein beta isoform	IPI00334907	0.000	0.27422	11	10	12	9
1688	Isoform 5 of Dynamin-1-like protein	IPI00037283	0.000	0.27422	13	13	12	14
1689	Cytochrome b-c1 complex subunit 2, mitochondrial	IPI00305383	0.000	0.27422	14	15	14	15
1690	Histidyl-tRNA synthetase, cytoplasmic	IPI00021808	0.000	0.27422	15	11	13	13
1691	Protein transport protein Sec24C	IPI00024661	0.000	0.27422	11	10	11	10
1692	Nuclear cap-binding protein subunit 1	IPI00019380	0.000	0.27422	8	8	10	6
1693	cDNA FLJ35809 fis, clone TESTI2006016, highly similar to Eukaryotic translation initiation factor 3	IPI00647650	0.000	0.27422	9	11	9	11
1694	Aspartate aminotransferase, cytoplasmic	IPI00219029	0.000	0.27422	14	14	15	13
1695	60S ribosomal protein L13	IPI00465361	0.000	0.27422	3	5	4	4
1696	Vesicle transport protein GOT1B	IPI00007061	0.000	0.27422	0	3	0	3
1697	DNA polymerase delta catalytic subunit	IPI00002894	0.000	0.27422	10	13	13	10
1698	Splicing factor, arginine/serine-rich 2	IPI00005978	0.000	0.27422	13	10	14	9
1699	baculoviral IAP repeat-containing protein 6	IPI00299635	0.000	0.27422	9	6	8	7
1700	Keratin, type I cytoskeletal 24	IPI00004550	0.000	0.27422	2	0	2	0
1701	Isoform 1 of Trans-2,3-enoyl-CoA reductase	IPI00100656	0.000	0.27422	3	8	4	7

No.	Description	Accession number	STN[1]	p-Value[1]	480_A[2]	480_B[2]	620_A[2]	620_B[2]
1702	Programmed cell death protein 10	IPI00298558	0.000	0.27422	11	10	8	13
1703	Nuclear pore complex protein Nup153	IPI00292059	0.000	0.27422	10	9	10	9
1704	Transmembrane protein 33	IPI00299084	0.000	0.27422	5	8	6	7
1705	Malectin	IPI00029046	0.000	0.27422	6	7	5	8
1706	Isoform 1 of Heterogeneous nuclear ribonucleoprotein U-like protein 1	IPI00013070	0.000	0.27422	5	4	4	5
1707	Eukaryotic translation initiation factor 3 subunit G	IPI00290460	0.000	0.27422	7	9	8	8
1708	Peroxisomal multifunctional enzyme type 2	IPI00019912	0.000	0.27422	5	9	4	10
1709	Protein transport protein Sec23A	IPI00017375	0.000	0.27422	7	7	6	8
1710	Uroporphyrinogen decarboxylase	IPI00301489	0.000	0.27422	0	0	2	0
1711	V-type proton ATPase subunit E 1	IPI00003856	0.000	0.27422	6	7	8	5
1712	Thioredoxin domain-containing protein 12	IPI00026328	0.000	0.27422	7	7	7	7
1713	Uncharacterized protein KIAA0406	IPI00011702	0.000	0.27422	4	8	5	7
1714	Phosphatidylinositol transfer protein alpha isoform	IPI00216048	0.000	0.27422	8	6	7	7
1715	Isoform 1 of Hematological and neurological expressed 1-like protein	IPI00027397	0.000	0.27422	9	9	10	8
1716	Isoform 2 of Ubiquitin carboxyl-terminal hydrolase isozyme L5	IPI00219512	0.000	0.27422	8	7	7	8
1717	Superoxide dismutase [Mn], mitochondrial	IPI00022314	0.000	0.27422	8	8	8	8
1718	Twinfilin-2	IPI00550917	0.000	0.27422	11	7	10	8
1719	protein arginine N-methyltransferase 5 isoform b	IPI00064328	0.000	0.27422	5	5	6	4
1720	Malate dehydrogenase	IPI00916111	0.000	0.27422	4	5	6	3
1721	Polymerase delta-interacting protein 2	IPI00165506	0.000	0.27422	6	6	6	6
1722	Talin-2	IPI00219299	0.000	0.27422	5	5	6	4
1723	Ras suppressor protein 1	IPI00017256	0.000	0.27422	5	5	5	5
1724	Isoform 2 of Zinc finger CCCH domain-containing protein 13	IPI00016472	0.000	0.27422	0	0	0	2
1725	24 kDa protein	IPI00397611	0.000	0.27422	5	4	4	5
1726	Junction plakoglobin	IPI00554711	0.000	0.27422	3	0	3	2
1727	MKI67 FHA domain-interacting nucleolar phosphoprotein	IPI00154590	0.000	0.27422	9	7	7	9
1728	Transmembrane 9 superfamily member 3	IPI00030847	0.000	0.27422	0	0	0	2
1729	Isoform A of Protein CutA	IPI00034319	0.000	0.27422	8	7	9	6
1730	Isoform 1 of Tumor protein D52	IPI00619958	0.000	0.27422	7	9	9	7
1731	28S ribosomal protein S31, mitochondrial	IPI00294242	0.000	0.27422	7	6	5	8
1732	UPF0687 protein C20orf27	IPI00101095	0.000	0.27422	0	0	2	2
1733	Peptidyl-prolyl cis-trans isomerase NIMA-interacting 1	IPI00013723	0.000	0.27422	7	7	7	7
1734	PDZ domain-containing protein GIPC1	IPI00024705	0.000	0.27422	7	6	8	5
1735	V-type proton ATPase subunit F	IPI00004488	0.000	0.27422	7	8	7	8
1736	Isoform 1 of N-alpha-acetyltransferase 50, NatE catalytic subunit	IPI00018627	0.000	0.27422	7	3	5	5
1737	DNA-directed RNA polymerase II subunit RPB7	IPI00218895	0.000	0.27422	4	3	5	2
1738	Pre-mRNA-splicing factor SYF1	IPI00163084	0.000	0.27422	4	7	5	6
1739	Tubulin-folding cofactor B	IPI00293126	0.000	0.27422	6	5	7	4
1740	Isoform 2 of Fumarylacetoacetate hydrolase domain-containing protein 1	IPI00440828	0.000	0.27422	6	5	6	5
1741	ATP synthase mitochondrial F1 complex assembly factor 2	IPI00296999	0.000	0.27422	6	5	5	6
1742	Serine/threonine-protein kinase PLK1	IPI00021248	0.000	0.27422	0	0	2	2
1743	Isoform 1 of CDP-diacylglycerol--inositol 3-phosphatidyltransferase	IPI00645518	0.000	0.27422	2	0	0	2
1744	Isoform 2 of Vesicle-associated membrane protein 7	IPI00013236	0.000	0.27422	3	3	0	4
1745	Acyl-coenzyme A thioesterase 13	IPI00020530	0.000	0.27422	8	6	6	8
1746	Isoform 2 of Serine/threonine-protein kinase PAK 3	IPI00027382	0.000	0.27422	5	7	6	6
1747	Probable ATP-dependent RNA helicase DDX27	IPI00293078	0.000	0.27422	8	6	7	7
1748	SPRY domain-containing protein 4	IPI00291643	0.000	0.27422	7	5	6	6
1749	Isoform 2 of RANBP2-like and GRIP domain-containing protein 5/6	IPI00100787	0.000	0.27422	2	2	0	0
1750	Myosin light chain 6B	IPI00027255	0.000	0.27422	0	0	2	2
1751	Guanine nucleotide-binding protein G(I)/G(S)/G(T) subunit beta-2	IPI00003348	0.000	0.27422	3	2	2	3
1752	Isoform 2 of 3-hydroxyisobutyryl-CoA hydrolase, mitochondrial	IPI00377161	0.000	0.27422	5	7	7	5
1753	Isoform 1 of Ribonuclease H2 subunit C	IPI00382985	0.000	0.27422	3	5	6	0
1754	Nucleolar protein 16	IPI00032849	0.000	0.27422	5	5	5	5
1755	Importin 5	IPI00639960	0.000	0.27422	2	2	2	0
1756	Activating signal cointegrator 1 complex subunit 3	IPI00430472	0.000	0.27422	0	2	0	0
1757	Isoform 3 of Sorting nexin-3	IPI00029740	0.000	0.27422	5	5	5	5
1758	Isoform 1 of Transcription elongation factor SPT6	IPI00784161	0.000	0.27422	7	7	6	8
1759	Ribonuclease H2 subunit A	IPI00290192	0.000	0.27422	6	5	5	6
1760	RhoA activator C11orf59	IPI00016670	0.000	0.27422	4	0	2	4
1761	39S ribosomal protein L46, mitochondrial	IPI00023161	0.000	0.27422	6	2	4	4
1762	Isoform 2 of Pinin	IPI00002649	0.000	0.27422	3	2	2	3
1763	Isoform 1 of Transmembrane protein 111	IPI00020472	0.000	0.27422	6	2	4	4
1764	Isoform 2 of Sorting nexin-3	IPI00216508	0.000	0.27422	4	4	3	5
1765	L antigen family member 3	IPI00032314	0.000	0.27422	2	0	0	0
1766	Prostaglandin E synthase 2	IPI00303568	0.000	0.27422	4	6	3	7
1767	Pumilio domain-containing protein KIAA0020	IPI00791325	0.000	0.27422	3	3	4	2
1768	Protein FADD	IPI00011919	0.000	0.27422	2	3	0	3
1769	Isoform 4 of N-terminal kinase-like protein	IPI00062264	0.000	0.27422	0	3	2	3
1770	Heat shock 70 kDa protein 4L	IPI00295485	0.000	0.27422	7	6	4	9
1771	RNA-binding protein PNO1	IPI00024524	0.000	0.27422	4	5	3	6
1772	Isoform A of Kinesin light chain 1	IPI00020096	0.000	0.27422	3	2	2	3
1773	Actin-related protein 2/3 complex subunit 5-like protein	IPI00414554	0.000	0.27422	4	4	3	5
1774	28S ribosomal protein S10, mitochondrial	IPI00061245	0.000	0.27422	3	4	4	3
1775	SAP domain-containing ribonucleoprotein	IPI00014938	0.000	0.27422	4	5	5	4
1776	Isoform 1 of DAZ-associated protein 1	IPI00165230	0.000	0.27422	4	0	4	0
1777	Nucleolar complex protein 4 homolog	IPI00031661	0.000	0.27422	3	4	4	3
1778	Isoform Long of 60 kDa SS-A/Ro ribonucleoprotein	IPI00019450	0.000	0.27422	6	5	6	5
1779	Calcium-binding protein 39	IPI00032561	0.000	0.27422	3	3	3	3
1780	Uncharacterized protein C19orf43	IPI00031526	0.000	0.27422	0	0	2	2
1781	Isoform XLas-1 of Guanine nucleotide-binding protein G(s) subunit alpha isoforms XLas	IPI00095891	0.000	0.27422	0	4	2	4
1782	39S ribosomal protein L45, mitochondrial	IPI00185859	0.000	0.27422	3	2	3	2
1783	Isoform 1 of Autophagy-related protein 3	IPI00022254	0.000	0.27422	5	4	6	3
1784	Protein BUD31 homolog	IPI00013180	0.000	0.27422	3	3	3	3
1785	18 kDa protein	IPI00797709	0.000	0.27422	0	0	0	2
1786	Isoform 2 of PEST proteolytic signal-containing nuclear protein	IPI00060650	0.000	0.27422	0	2	2	2
1787	Isoamyl acetate-hydrolyzing esterase 1 homolog	IPI00419194	0.000	0.27422	3	0	0	3
1788	Isoform 1 of Serine/threonine-protein phosphatase 6 catalytic subunit	IPI00012970	0.000	0.27422	4	4	4	4
1789	Ribosome production factor 2 homolog	IPI00396329	0.000	0.27422	0	3	0	3
1790	Thioredoxin domain-containing protein 9	IPI00022386	0.000	0.27422	0	4	3	3
1791	TRMT61A protein (Fragment)	IPI00059718	0.000	0.27422	3	3	2	4
1792	Mitogen-activated protein kinase 1	IPI00003479	0.000	0.27422	5	4	5	4
1793	cDNA FLJ50992, highly similar to Coronin-1C	IPI00798401	0.000	0.27422	2	0	0	0
1794	Proteasome subunit beta type-10	IPI00027933	0.000	0.27422	2	2	0	0
1795	Adenylate kinase isoenzyme 6	IPI00032879	0.000	0.27422	0	0	2	2
1796	WD repeat-containing protein 18	IPI00032533	0.000	0.27422	3	3	3	3

No.	Description	Accession number	STN[1]	p-Value[1]	480_A[2]	480_B[2]	620_A[2]	620_B[2]
1797	Isoform 1 of Lysine-specific demethylase 3B	IPI00298935	0.000	0.27422	0	2	2	2
1798	Isoform 2 of Rap guanine nucleotide exchange factor 6	IPI00291839	0.000	0.27422	0	2	0	0
1799	Isoform 1 of 2',5'-phosphodiesterase 12	IPI00174390	0.000	0.27422	3	3	3	3
1800	Isoform 1 of Ribosome-recycling factor, mitochondrial	IPI00061108	0.000	0.27422	4	3	5	0
1801	DNA-directed RNA polymerase, mitochondrial precursor	IPI00298738	0.000	0.27422	0	2	2	2
1802	Isoform 2 of 1,2-dihydroxy-3-keto-5-methylthiopentene dioxygenase	IPI00470791	0.000	0.27422	0	0	2	2
1803	Isoform 1 of Syntaxin-7	IPI00289876	0.000	0.27422	5	4	5	4
1804	Microtubule-associated protein 1S	IPI00296485	0.000	0.27422	3	2	3	2
1805	erlin-1	IPI00007940	0.000	0.27422	6	3	5	4
1806	Isoform 1 of Mitogen-activated protein kinase kinase kinase kinase 4	IPI00006752	0.000	0.27422	0	0	0	2
1807	Vacuolar protein sorting-associated protein 26B	IPI00059264	0.000	0.27422	2	2	0	2
1808	Cation-dependent mannose-6-phosphate receptor	IPI00025049	0.000	0.27422	0	2	0	2
1809	Peptidyl-prolyl cis-trans isomerase-like 1	IPI00007019	0.000	0.27422	0	0	0	2
1810	28S ribosomal protein S18a, mitochondrial	IPI00018691	0.000	0.27422	2	0	2	2
1811	Isoform 1 of Transmembrane protein 70, mitochondrial	IPI00106966	0.000	0.27422	0	2	0	0
1812	Isoform 1 of E3 ubiquitin-protein ligase BRE1B	IPI00162563	0.000	0.27422	2	2	0	2
1813	RcDNAJ9 (Fragment)	IPI00014718	0.000	0.27422	4	5	4	5
1814	DNA topoisomerase 2-binding protein 1	IPI00293921	0.000	0.27422	0	0	0	2
1815	DCC-interacting protein 13-alpha	IPI00015836	0.000	0.27422	2	2	2	2
1816	Thioredoxin-interacting protein	IPI00007956	0.000	0.27422	0	0	2	0
1817	Dolichyl-diphosphooligosaccharide--protein glycosyltransferase subunit DAD1	IPI00009407	0.000	0.27422	3	4	2	5
1818	Isoform Alpha of Caveolin-1	IPI00009236	0.000	0.27422	2	0	0	2
1819	BRI3-binding protein	IPI00103599	0.000	0.27422	0	0	2	0
1820	39S ribosomal protein L17, mitochondrial	IPI00172591	0.000	0.27422	3	0	2	3
1821	Isoform 1 of Beta-enolase	IPI00218474	0.000	0.27422	0	0	2	0
1822	Isoform 1 of Phosphatidylinositol 4-kinase alpha	IPI00070943	0.000	0.27422	2	3	3	0
1823	Isoform 1 of Protein LSM12 homolog	IPI00410324	0.000	0.27422	6	4	5	5
1824	cDNA FLJ61162, highly similar to Ras-related protein R-Ras2	IPI00012512	0.000	0.27422	4	2	3	3
1825	Isoform 2 of Transportin-2	IPI00164417	0.000	0.27422	4	2	4	2
1826	Isoform 3 of Anoctamin-1	IPI00384668	0.000	0.27422	0	2	0	0
1827	Isoform 4 of Death-inducer obliterator 1	IPI00619921	0.000	0.27422	0	2	0	0
1828	Isoform 1 of Retinol dehydrogenase 11	IPI00339384	0.000	0.27422	4	4	4	4
1829	Isoform 2 of Low molecular weight phosphotyrosine protein phosphatase	IPI00218847	0.000	0.27422	4	5	5	4
1830	Full-length cDNA 5-PRIME end of clone CS0DJ009YL13 of T cells (Jurkat cell line) of Homo sapiens	IPI00384016	0.000	0.27422	3	0	0	3
1831	Small glutamine-rich tetratricopeptide repeat-containing protein alpha	IPI00013949	0.000	0.27422	0	2	0	0
1832	Syntaxin-8	IPI00009225	0.000	0.27422	0	2	0	2
1833	Isoform Brain of Clathrin light chain A	IPI00014587	0.000	0.27422	2	0	2	0
1834	serine/threonine-protein phosphatase PP1-alpha catalytic subunit isoform 3	IPI00027423	0.000	0.27422	6	4	5	5
1835	Zinc finger CCCH domain-containing protein 4	IPI00187011	0.000	0.27422	3	2	3	0
1836	Mitochondrial ribosomal protein L21 isoform d	IPI00375677	0.000	0.27422	3	2	2	3
1837	Isoform 1 of Mammalian ependymin-related protein 1	IPI00259102	0.000	0.27422	5	0	5	2
1838	Isoform 2 of Bromodomain adjacent to zinc finger domain protein 2A	IPI00296388	0.000	0.27422	0	0	0	2
1839	WD repeat-containing protein 5	IPI00005492	0.000	0.27422	2	3	3	0
1840	cDNA FLJ31776 fis, clone NT2RI2008141, highly similar to CALUMENIN	IPI00789155	0.000	0.27422	2	3	2	3
1841	Myosin-11	IPI00020501	0.000	0.27422	0	3	3	2
1842	Bifunctional 3'-phosphoadenosine 5'-phosphosulfate synthase 1	IPI00011619	0.000	0.27422	2	2	0	0
1843	Isoform 1 of Deoxycytidylate deaminase	IPI00296863	0.000	0.27422	0	3	3	0
1844	Isoform 1 of Fermitin family homolog 2	IPI00000856	0.000	0.27422	0	0	2	2
1845	Fragile X mental retardation syndrome-related protein 2	IPI00016250	0.000	0.27422	0	2	0	0
1846	Isoform 1 of HEAT repeat-containing protein 5A	IPI00783902	0.000	0.27422	2	0	0	0
1847	Isoform 1 of Cell division protein kinase 12	IPI00021175	0.000	0.27422	0	0	0	2
1848	Isoform 1 of Magnesium-dependent phosphatase 1	IPI00337556	0.000	0.27422	0	0	2	2
1849	Isoform 2 of 1-acyl-sn-glycerol-3-phosphate acyltransferase beta	IPI00221372	0.000	0.27422	3	3	2	4
1850	Thymidine kinase, cytosolic	IPI00299214	0.000	0.27422	2	2	2	2
1851	Isoform 1 of Neuroguidin	IPI00000162	0.000	0.27422	0	2	0	2
1852	Isoform 1 of DNA-directed RNA polymerases I and III subunit RPAC1	IPI00005179	0.000	0.27422	2	4	3	3
1853	Protein FAM50A	IPI00030098	0.000	0.27422	2	2	2	2
1854	Tetratricopeptide repeat protein 9C	IPI00175096	0.000	0.27422	3	3	0	4
1855	Isoform 2 of Heterogeneous nuclear ribonucleoprotein A/B	IPI00334587	0.000	0.27422	2	0	0	0
1856	Isoform SERCA3B of Sarcoplasmic/endoplasmic reticulum calcium ATPase 3	IPI00004092	0.000	0.27422	0	0	2	0
1857	Deoxyhypusine hydroxylase	IPI00171856	0.000	0.27422	4	0	4	2
1858	Isoform DFF45 of DNA fragmentation factor subunit alpha (Fragment)	IPI00010882	0.000	0.27422	3	3	3	3
1859	Calcium homeostasis endoplasmic reticulum protein	IPI00333010	0.000	0.27422	2	2	0	0
1860	cDNA FLJ56402, highly similar to Tripeptidyl-peptidase 1	IPI00298237	0.000	0.27422	3	3	3	3
1861	Transmembrane protein 11	IPI00012855	0.000	0.27422	2	0	2	2
1862	103 kDa protein	IPI00402008	0.000	0.27422	2	2	0	0
1863	EPS8L2 protein	IPI00414315	0.000	0.27422	3	3	3	3
1864	NADH dehydrogenase [ubiquinone] 1 alpha subcomplex subunit 4	IPI00011770	0.000	0.27422	0	0	0	2
1865	Splicing factor, arginine/serine-rich 4	IPI00000015	0.000	0.27422	0	0	0	2
1866	Isoform 1 of E3 ubiquitin-protein ligase RNF123	IPI00335085	0.000	0.27422	0	2	2	0
1867	ER lumen protein retaining receptor 1	IPI00028116	0.000	0.27422	0	0	0	2
1868	Negative elongation factor B	IPI00103483	0.000	0.27422	2	0	0	2
1869	RNA-binding motif protein, X-linked-like-2	IPI00004450	0.000	0.27422	0	3	3	2
1870	Choline/ethanolaminephosphotransferase 1	IPI00005775	0.000	0.27422	0	0	2	2
1871	NADH-ubiquinone oxidoreductase chain 1	IPI00007961	0.000	0.27422	0	0	2	0
1872	Calcium-binding protein p22	IPI00218924	0.000	0.27422	3	4	3	4
1873	Deoxynucleotidyltransferase terminal-interacting protein 2	IPI00290410	0.000	0.27422	0	3	2	3
1874	Putative uncharacterized protein NOL8	IPI00161085	0.000	0.27422	3	2	3	2
1875	Integrator complex subunit 5	IPI00304676	0.000	0.27422	0	0	2	2
1876	Isoform A of GC-rich sequence DNA-binding factor homolog	IPI00001364	0.000	0.27422	0	3	2	3
1877	Isoform 1 of Kinesin-like protein KIF1B	IPI00029011	0.000	0.27422	0	0	2	2
1878	Isoform 1 of Integrator complex subunit 6	IPI00015922	0.000	0.27422	0	0	2	2
1879	inosine-5'-monophosphate dehydrogenase 1 isoform a	IPI00375527	0.000	0.27422	3	4	3	4
1880	Isoform 2 of Mediator of RNA polymerase II transcription subunit 8	IPI00300278	0.000	0.27422	2	0	2	2
1881	Glutathione S-transferase theta 1	IPI00607741	0.000	0.27422	0	2	0	0
1882	Charged multivesicular body protein 2a	IPI00004416	0.000	0.27422	3	2	3	0
1883	mortality factor 4	IPI00001955	0.000	0.27422	0	0	0	2
1884	Isoform 2 of Mediator of RNA polymerase II transcription subunit 1	IPI00790747	0.000	0.27422	0	2	2	2
1885	DNA polymerase	IPI00744598	0.000	0.27422	0	0	2	0
1886	Isoform 1 of Uncharacterized protein C1orf77	IPI00300990	0.000	0.27422	2	2	2	2
1887	Peroxisomal membrane protein 11B	IPI00021978	0.000	0.27422	2	0	0	0
1888	Protein FRG1	IPI00004655	0.000	0.27422	2	2	2	2
1889	Huntingtin	IPI00002335	0.000	0.27422	2	0	2	0
1890	Protein FAM50B	IPI00015912	0.000	0.27422	3	0	0	3
1891	Origin recognition complex subunit 5	IPI00015143	0.000	0.27422	2	0	2	2

No.	Description	Accession number	STN[1]	p-Value[1]	480_A[2]	480_B[2]	620_A[2]	620_B[2]
1892	Complex I intermediate-associated protein 30, mitochondrial	IPI00032560	0.000	0.27422	0	2	0	2
1893	Exosome complex exonuclease RRP42	IPI00014198	0.000	0.27422	3	3	4	2
1894	Armadillo repeat-containing protein 1	IPI00018260	0.000	0.27422	4	2	4	2
1895	Vesicle-associated membrane protein 8	IPI00030911	0.000	0.27422	3	3	3	3
1896	V-type proton ATPase subunit G 1	IPI00025285	0.000	0.27422	5	4	5	4
1897	cDNA, FLJ79450, highly similar to 3-ketoacyl-CoA thiolase, peroxisomal	IPI00011522	0.000	0.27422	3	2	3	0
1898	E3 ubiquitin-protein ligase MARCH5	IPI00414168	0.000	0.27422	0	0	0	2
1899	Neighbor of COX4	IPI00005740	0.000	0.27422	0	2	0	2
1900	Translation initiation factor eIF-2B subunit epsilon	IPI00011898	0.000	0.27422	0	0	2	0
1901	Isoform 3 of 5'-3' exoribonuclease 1	IPI00328115	0.000	0.27422	2	2	2	0
1902	similar to unr-interacting protein	IPI00260209	0.000	0.27422	0	0	2	0
1903	aldehyde dehydrogenase 9A1	IPI00479877	0.000	0.27422	2	0	0	0
1904	Pyridoxal phosphate phosphatase	IPI00025340	0.000	0.27422	2	0	2	2
1905	Isoform 1 of Rho guanine nucleotide exchange factor 12	IPI00022164	0.000	0.27422	0	0	2	2
1906	G patch domain and KOW motifs-containing protein	IPI00024255	0.000	0.27422	2	3	2	3
1907	Isoform 1 of Apoptotic protease-activating factor 1	IPI00023630	0.000	0.27422	0	0	0	2
1908	Putative GTP-binding protein RAY-like variant (Fragment)	IPI00411514	0.000	0.27422	0	0	0	2
1909	Isoform 1 of Protein fto	IPI00028277	0.000	0.27422	2	0	2	0
1910	Isoform 2 of Zinc finger C3H1 domain-containing protein	IPI00175146	0.000	0.27422	0	0	0	2
1911	Isoform 2 of Phosphatidylinositol 4-kinase alpha	IPI00031424	0.000	0.27422	0	2	0	0
1912	Isoform 1 of DEP domain-containing protein 7	IPI00163266	0.000	0.27422	0	0	0	2
1913	Uncharacterized protein C6orf130	IPI00184871	0.000	0.27422	0	3	2	3
1914	Isoform 4 of Ubiquitin-conjugating enzyme E2 variant 1	IPI00007847	0.000	0.27422	0	0	0	2
1915	HIV Tat-specific factor 1	IPI00013788	0.000	0.27422	3	3	3	3
1916	Isoform 1 of U4/U6 small nuclear ribonucleoprotein Prp4	IPI00150269	0.000	0.27422	3	2	2	3
1917	Isoform 2 of Cytochrome b5	IPI00182933	0.000	0.27422	2	2	2	0
1918	Isoform 1 of Coiled-coil domain-containing protein 90B, mitochondrial	IPI00549236	0.000	0.27422	2	0	0	2
1919	39S ribosomal protein L18, mitochondrial	IPI00160421	0.000	0.27422	5	3	5	3
1920	NADH dehydrogenase [ubiquinone] 1 alpha subcomplex subunit 6	IPI00419266	0.000	0.27422	2	3	3	0
1921	Acyl-protein thioesterase 2	IPI00027032	0.000	0.27422	0	0	2	0
1922	Uncharacterized protein C21orf59	IPI00025710	0.000	0.27422	2	0	2	0
1923	Putative adenosylhomocysteinase 3	IPI00101645	0.000	0.27422	0	2	0	2
1924	Isoform 1 of Lysocardiolipin acyltransferase 1	IPI00419643	0.000	0.27422	0	0	2	2
1925	Non-histone chromosomal protein HMG-14	IPI00554761	0.000	0.27422	2	0	0	0
1926	Keratin, type I cytoskeletal 17	IPI00450768	0.000	0.27422	2	0	0	0
1927	cDNA FLJ52725, highly similar to Magnesium transporter MRS2L, mitochondrial	IPI00010188	0.000	0.27422	0	0	2	0
1928	DNA polymerase beta	IPI00219538	0.000	0.27422	2	0	0	2
1929	Laminin subunit alpha-5	IPI00783665	0.000	0.27422	2	0	0	0
1930	cDNA FLJ52756, highly similar to RNA polymerase transcriptional regulation mediator, subunit 6	IPI00102495	0.000	0.27422	0	0	2	0
1931	40S ribosomal protein S12	IPI00013917	0.000	0.27422	0	0	2	0
1932	Pumilio domain-containing protein C14orf21	IPI00216999	0.000	0.27422	0	2	0	0
1933	Isoform 1 of Crooked neck-like protein 1	IPI00177437	0.000	0.27422	3	3	0	4
1934	Beta-lactamase-like protein 2	IPI00006952	0.000	0.27422	0	0	2	2
1935	Pumilio homolog 1 (Drosophila), isoform CRA_c	IPI00032355	0.000	0.27422	2	2	0	0
1936	Peptidyl-tRNA hydrolase family protein	IPI00048572	0.000	0.27422	2	0	0	2
1937	Isoform 1 of Serine/threonine-protein phosphatase 4 regulatory subunit 3B	IPI00414323	0.000	0.27422	2	0	0	2
1938	Importin-8	IPI00007401	0.000	0.27422	0	0	0	2
1939	GPN-loop GTPase 1 isoform a	IPI00027035	0.000	0.27422	0	2	2	0
1940	Isoform 2 of UPF0585 protein C16orf13	IPI00065475	0.000	0.27422	2	0	0	0
1941	Farnesyl pyrophosphate synthetase like-4 protein (Fragment)	IPI00382869	0.000	0.27422	3	0	2	3
1942	U6 snRNA-associated Sm-like protein LSm1	IPI00004436	0.000	0.27422	0	3	3	2
1943	Protein S100-P	IPI00017526	0.000	0.27422	0	0	0	2
1944	Myosin-If	IPI00218638	0.000	0.27422	0	0	2	2
1945	Serine/threonine-protein kinase 25	IPI00012093	0.000	0.27422	2	0	0	0
1946	DNA-directed RNA polymerase III subunit RPC1	IPI00024163	0.000	0.27422	0	0	0	2
1947	Methionine aminopeptidase 1	IPI00022239	0.000	0.27422	0	0	0	2
1948	Isoform 1 of Vesicle-associated membrane protein 7	IPI00020887	0.000	0.27422	2	0	0	0
1949	Trafficking protein particle complex subunit 5	IPI00177509	0.000	0.27422	0	2	2	2
1950	Isoform 1 of Chromodomain-helicase-DNA-binding protein 8	IPI00398992	0.000	0.27422	0	0	2	2
1951	Isoform 1 of Structural maintenance of chromosomes protein 4	IPI00411559	0.000	0.27422	0	0	2	0
1952	Ribosome biogenesis protein NSA2 homolog	IPI00007089	0.000	0.27422	2	0	2	2
1953	Isoform Alpha of Caspase-6	IPI00023876	0.000	0.27422	0	0	0	2
1954	Dehydrogenase/reductase SDR family member on chromosome X	IPI00166860	0.000	0.27422	0	0	2	2
1955	Isoform 1 of NADH dehydrogenase [ubiquinone] flavoprotein 1, mitochondrial	IPI00028520	0.000	0.27422	2	0	2	0
1956	SNW domain-containing protein 1	IPI00013830	0.000	0.27422	2	3	2	3
1957	Isoform 2 of cAMP-dependent protein kinase catalytic subunit beta	IPI00376119	0.000	0.27422	0	0	2	0
1958	Isoform 1 of Zinc finger MYM-type protein 3	IPI00029484	0.000	0.27422	0	0	2	2
1959	Isoform 2 of mRNA cap guanine-N7 methyltransferase	IPI00410657	0.000	0.27422	2	2	0	2
1960	Proteasome assembly chaperone 2	IPI00644482	0.000	0.27422	2	0	0	0
1961	Chromosome-associated kinesin KIF4B	IPI00175193	0.000	0.27422	3	2	3	2
1962	Isoform 6 of E3 ubiquitin-protein ligase UBR4	IPI00386907	0.000	0.27422	2	2	0	0
1963	WD repeat-containing protein 81 isoform 1	IPI00917671	0.000	0.27422	0	0	0	2
1964	Isoform 1 of GPI transamidase component PIG-T	IPI00100030	0.000	0.27422	0	0	0	2
1965	Isoform 2 of DnaJ homolog subfamily C member 2	IPI00455199	0.000	0.27422	3	2	3	2
1966	Isoform 2 of 5'-3' exoribonuclease 1	IPI00657645	0.000	0.27422	2	3	3	2
1967	Isoform 2 of Treacle protein	IPI00298696	0.000	0.27422	0	0	0	2
1968	Isoform IIA of Myc box-dependent-interacting protein 1	IPI00186966	0.000	0.27422	0	0	2	2
1969	Deubiquitinating protein VCIP135	IPI00064162	0.000	0.27422	0	3	3	0
1970	Isoform 1 of Transcription termination factor 2	IPI00290812	0.000	0.27422	0	0	0	2
1971	[Pyruvate dehydrogenase [lipoamide]] kinase isozyme 3, mitochondrial	IPI00014849	0.000	0.27422	0	0	0	2
1972	Alpha-mannosidase 2	IPI00003802	0.000	0.27422	2	2	2	2
1973	UBX domain-containing protein 4	IPI00293946	0.000	0.27422	3	4	4	3
1974	Centromere protein H	IPI00009668	0.000	0.27422	0	0	2	0
1975	NADH dehydrogenase [ubiquinone] 1 alpha subcomplex assembly factor 4	IPI00023064	0.000	0.27422	0	3	2	3
1976	Golgi resident protein GCP60	IPI00009315	0.000	0.27422	3	3	3	3
1977	Syntaxin-17	IPI00012028	0.000	0.27422	0	0	2	0
1978	Probable 2-oxoglutarate dehydrogenase E1 component DHKTD1, mitochondrial	IPI00063408	0.000	0.27422	2	0	0	0
1979	Phosphatidylserine synthase 1	IPI00010746	0.000	0.27422	2	2	0	0
1980	Transmembrane protein 109	IPI00031697	0.000	0.27422	2	0	2	0
1981	Phospholipase D3	IPI00328243	0.000	0.27422	2	2	2	2
1982	PRA1 family protein 3	IPI00007426	0.000	0.27422	0	0	2	0
1983	Isoform 2 of V-type proton ATPase 116 kDa subunit a isoform 1	IPI00743576	0.000	0.27422	0	0	0	2
1984	Mitochondrial 18 kDa protein	IPI00784376	0.000	0.27422	0	0	0	2
1985	Isoform 1 of Thymocyte nuclear protein 1	IPI00383163	0.000	0.27422	3	3	2	4
1986	Isoform XD of Plasma membrane calcium-transporting ATPase 4	IPI00012490	0.000	0.27422	0	2	0	0

No.	Description	Accession number	STN[1]	p-Value[1]	480_A[2]	480_B[2]	620_A[2]	620_B[2]
1987	RNASEH2B protein	IPI00245135	0.000	0.27422	2	2	0	0
1988	Isoform 1 of GDP-fucose protein O-fucosyltransferase 1	IPI00058192	0.000	0.27422	0	0	0	2
1989	TATA-box-binding protein	IPI00022831	0.000	0.27422	0	0	2	2
1990	Isoform 1 of 1,2-dihydroxy-3-keto-5-methylthiopentene dioxygenase	IPI00651738	0.000	0.27422	0	2	0	0
1991	Isoform 2 of CLIP-associating protein 2	IPI00167067	0.000	0.27422	0	2	0	2
1992	Tyrosine-protein kinase CSK	IPI00013212	0.000	0.27422	0	0	2	0
1993	DNA/RNA-binding protein KIN17	IPI00017580	0.000	0.27422	0	2	0	0
1994	Putative uncharacterized protein FUBP3	IPI00063245	0.000	0.27422	2	2	2	0
1995	Isoform 1 of Voltage-gated potassium channel subunit beta-2	IPI00021088	0.000	0.27422	0	0	2	2
1996	Lysosomal Pro-X carboxypeptidase	IPI00001593	0.000	0.27422	0	0	2	0
1997	Angio-associated migratory cell protein	IPI00014481	0.000	0.27422	2	2	2	2
1998	UPF0361 protein C3orf37	IPI00024618	0.000	0.27422	0	3	2	3
1999	Coiled-coil domain-containing protein 86	IPI00012199	0.000	0.27422	0	0	2	2
2000	Microsomal glutathione S-transferase 1	IPI00021805	0.000	0.27422	0	0	2	2
2001	Isoform 1 of Serine-protein kinase ATM	IPI00298306	0.000	0.27422	0	0	2	0
2002	Isoform 1 of B-cell CLL/lymphoma 9-like protein	IPI00328798	0.000	0.27422	0	0	0	2
2003	Isoform 1 of 5-azacytidine-induced protein 1	IPI00298883	0.000	0.27422	0	2	0	0
2004	Ras GTPase-activating-like protein IQGAP3	IPI00328905	0.000	0.27422	2	2	0	0
2005	Ribonuclease H1	IPI00000077	0.000	0.27422	0	0	0	2
2006	Isoform 2 of Cytochrome P450 2S1	IPI00164018	0.000	0.27422	0	0	0	2
2007	Bloom syndrome protein	IPI00004859	0.000	0.27422	0	2	0	2
2008	Argininosuccinate lyase	IPI00220267	0.000	0.27422	0	2	0	0
2009	Probable ATP-dependent RNA helicase YTHDC2	IPI00010200	0.000	0.27422	0	2	0	2
2010	Transmembrane protein 97	IPI00020004	0.000	0.27422	0	2	0	0
2011	DnaJ homolog subfamily C member 3	IPI00006713	0.000	0.27422	0	0	2	2
2012	39S ribosomal protein L14, mitochondrial	IPI00418290	0.000	0.27422	2	2	0	2
2013	S-adenosyl-L-methionine-dependent methyltransferase FTSJD2	IPI00166153	0.000	0.27422	0	0	0	2
2014	Isoform Long of Beta-glucuronidase	IPI00027745	0.000	0.27422	0	0	2	2
2015	Isoform 1 of Phosphoribosyl pyrophosphate synthase-associated protein 1	IPI00291578	0.000	0.27422	2	3	2	3
2016	DNA repair protein RAD51 homolog 3	IPI00012829	0.000	0.27422	0	0	2	0
2017	Uncharacterized protein C13orf27	IPI00414985	0.000	0.27422	0	0	0	2
2018	Synaptojanin-2-binding protein	IPI00299193	0.000	0.27422	3	3	2	4
2019	cDNA FLJ61386, highly similar to Homo sapiens mitochondrial ribosomal protein L43 (MRPL43)	IPI00334579	0.000	0.27422	0	2	2	2
2020	Isoform 1 of RNA polymerase II subunit A C-terminal domain phosphatase SSU72	IPI00023556	0.000	0.27422	3	2	2	3
2021	MMP37-like protein, mitochondrial	IPI00060287	0.000	0.27422	0	0	2	0
2022	GTP cyclohydrolase 1 feedback regulatory protein	IPI00217253	0.000	0.27422	0	0	2	0
2023	D-dopachrome decarboxylase	IPI00293867	0.000	0.27422	0	2	0	0
2024	Isoform 2 of Protein diaphanous homolog 2	IPI00514075	0.000	0.27422	0	0	2	2
2025	Golgi phosphoprotein 3-like	IPI00012313	0.000	0.27422	2	0	0	0
2026	Endoribonuclease Dicer	IPI00219036	0.000	0.27422	2	2	0	0
2027	Isoform 1 of Long-chain-fatty-acid--CoA ligase 1	IPI00012728	0.000	0.27422	0	2	0	0
2028	NADH dehydrogenase [ubiquinone] 1 alpha subcomplex assembly factor 3	IPI00399053	0.000	0.27422	0	0	2	2
2029	Targeting protein for Xklp2	IPI00008477	0.000	0.27422	2	0	0	0
2030	Isoform 2 of Gamma-glutamylcyclotransferase	IPI00020301	0.000	0.27422	2	2	2	2
2031	Isoform 3 of Tyrosine-protein phosphatase non-receptor type 6	IPI00183046	0.000	0.27422	0	0	2	2
2032	Isoform 4 of Zinc finger protein 638	IPI00178953	0.000	0.27422	0	0	0	2
2033	Isoform 1 of Coiled-coil domain-containing protein 51	IPI00153023	0.000	0.27422	0	0	2	0
2034	tRNA (guanine-N(1)-)-methyltransferase	IPI00455268	0.000	0.27422	2	0	0	0
2035	Cytochrome c oxidase assembly protein COX11, mitochondrial	IPI00295394	0.000	0.27422	0	0	2	2
2036	Isoform 1 of Transmembrane emp24 domain-containing protein 4	IPI00296259	0.000	0.27422	2	0	2	2
2037	Isoform 1 of Histone-arginine methyltransferase CARM1	IPI00412880	0.000	0.27422	0	0	2	2
2038	RNA polymerase-associated protein RTF1 homolog	IPI00303832	0.000	0.27422	3	2	2	3
2039	Isoform 1 of Transmembrane protein 85	IPI00009320	0.000	0.27422	3	3	3	3
2040	Isoform 1 of 28S ribosomal protein S11, mitochondrial	IPI00010244	0.000	0.27422	0	2	2	0
2041	Huntingtin-interacting protein 1-related protein	IPI00024417	0.000	0.27422	2	0	0	2
2042	Torsin-1A-interacting protein 2	IPI00168878	0.000	0.27422	0	2	0	0
2043	Isoform 1 of Myosin-XVIIIa	IPI00760846	0.000	0.27422	0	2	0	0
2044	Melanoma-associated antigen G1	IPI00217104	0.000	0.27422	2	2	0	2
2045	Isoform 1 of Cullin-4A	IPI00419273	0.000	0.27422	0	0	2	2
2046	Isoform 1 of RNA 3'-terminal phosphate cyclase	IPI00011726	0.000	0.27422	3	0	3	2
2047	Acyl-CoA-binding domain-containing protein 6	IPI00031680	0.000	0.27422	0	2	0	0
2048	Golgi SNAP receptor complex member 1	IPI00029447	0.000	0.27422	2	2	2	0
2049	Glutamine-rich protein 1	IPI00328200	0.000	0.27422	2	2	2	2
2050	Protein kinase C and casein kinase substrate in neurons 3, isoform CRA_b	IPI00329572	0.000	0.27422	3	0	3	2
2051	Isoform 1 of OCIA domain-containing protein 2	IPI00555902	0.000	0.27422	0	0	2	0
2052	MAP kinase-activated protein kinase 3	IPI00005777	0.000	0.27422	0	0	2	0
2053	Protein FAM118B	IPI00002240	0.000	0.27422	2	2	0	2
2054	Isoform 1 of Tumor protein D53	IPI00383670	0.000	0.27422	0	2	2	0
2055	NudC domain-containing protein 2	IPI00103142	0.000	0.27422	2	2	0	0
2056	Ras-related protein Rap-2b	IPI00018364	0.000	0.27422	2	2	2	0
2057	Isoform 1 of Polyglutamine-binding protein 1	IPI00024698	0.000	0.27422	0	2	0	0
2058	Thioredoxin-related transmembrane protein 4	IPI00100247	0.000	0.27422	0	0	0	2
2059	glutathione peroxidase 1 isoform 2	IPI00398780	0.000	0.27422	0	2	0	0
2060	Isoform 2 of Tether containing UBX domain for GLUT4	IPI00065276	0.000	0.27422	0	0	2	2
2061	ELMO domain-containing protein 2	IPI00217918	0.000	0.27422	2	0	0	0
2062	Zinc finger CCCH domain-containing protein 11A	IPI00328306	0.000	0.27422	0	0	2	2
2063	Inner nuclear membrane protein Man1	IPI00032491	0.000	0.27422	0	0	2	2
2064	Transcription initiation factor TFIID subunit 10	IPI00030364	0.000	0.27422	3	0	3	0
2065	Probable U3 small nucleolar RNA-associated protein 11	IPI00180454	0.000	0.27422	0	0	2	0
2066	Mediator complex subunit 4	IPI00033546	0.000	0.27422	0	2	0	0
2067	PRMT3 protein (Fragment)	IPI00103026	0.000	0.27422	0	0	2	0
2068	Isoform 1 of Far upstream element-binding protein 3	IPI00377261	0.000	0.27422	0	2	2	2
2069	PIH1 domain-containing protein 1	IPI00550995	0.000	0.27422	2	0	2	0
2070	M-phase phosphoprotein 6	IPI00016074	0.000	0.27422	2	0	2	0
2071	Isoform B of Nuclear factor of activated T-cells, cytoplasmic 2	IPI00247309	0.000	0.27422	0	2	0	0
2072	Secretory carrier-associated membrane protein 2	IPI00218850	0.000	0.27422	2	2	2	0
2073	Isoform 2 of Protein FAM36A	IPI00103057	0.000	0.27422	2	2	2	2
2074	glycogen [starch] synthase, muscle isoform 2	IPI00157144	0.000	0.27422	2	2	0	0
2075	Isoform 1 of Phostensin	IPI00152853	0.000	0.27422	2	0	0	0
2076	Isoform 1 of Putative methyltransferase METT10D	IPI00163391	0.000	0.27422	0	0	0	2
2077	Exocyst complex component 2	IPI00783559	0.000	0.27422	3	2	3	2
2078	Isoform 3 of Pre-mRNA 3'-end-processing factor FIP1	IPI00008449	0.000	0.27422	2	0	0	2
2079	Isoform 4 of Phosphorylase b kinase regulatory subunit beta	IPI00181893	0.000	0.27422	2	0	0	2
2080	Isoform 2 of Protein diaphanous homolog 3	IPI00655865	0.000	0.27422	0	2	0	0
2081	RNA methyltransferase-like protein 1	IPI00335589	0.000	0.27422	0	0	2	2

No.	Description	Accession number	STN[1]	p-Value[1]	480_A[2]	480_B[2]	620_A[2]	620_B[2]
2082	Isoform 1 of Testin	IPI00024097	0.000	0.27422	0	2	0	2
2083	Isoform 3 of TP53RK-binding protein	IPI00217362	0.000	0.27422	2	0	2	0
2084	Isoform 1 of RNA polymerase II-associated factor 1 homolog	IPI00300333	0.000	0.27422	0	3	3	2
2085	Hexokinase-2	IPI00102864	0.000	0.27422	0	0	2	0
2086	Dual specificity protein phosphatase 23	IPI00306353	0.000	0.27422	2	0	0	0
2087	U8 snoRNA-decapping enzyme	IPI00783497	0.000	0.27422	0	3	2	3
2088	Chromatin accessibility complex protein 1	IPI00010158	0.000	0.27422	0	0	2	0
2089	PNAS-117	IPI00020827	0.000	0.27422	0	0	2	0
2090	3-ketoacyl-CoA thiolase, peroxisomal	IPI00012828	0.000	0.27422	2	0	0	0
2091	EIF4G3 protein	IPI00328268	0.000	0.27422	0	0	2	0
2092	Isoform Long of Ancient ubiquitous protein 1	IPI00001891	0.000	0.27422	4	2	4	0
2093	Guanine nucleotide-binding protein G(q) subunit alpha	IPI00288947	0.000	0.27422	0	3	3	2
2094	FAST kinase domain-containing protein 5	IPI00414973	0.000	0.27422	0	2	0	0
2095	Splicing factor, arginine/serine-rich 19	IPI00303343	0.000	0.27422	0	0	2	2
2096	Protein KTI12 homolog	IPI00061528	0.000	0.27422	0	2	2	2
2097	Peptide methionine sulfoxide reductase	IPI00006592	0.000	0.27422	0	0	2	0
2098	Vacuolar protein sorting-associated protein 45	IPI00090327	0.000	0.27422	2	0	0	0
2099	Protein FAM96A	IPI00030985	0.000	0.27422	2	0	2	0
2100	Centrin-2	IPI00215928	0.000	0.27422	0	0	2	0
2101	Farnesyltransferase, CAAX box, alpha, isoform CRA_a	IPI00026813	0.000	0.27422	2	2	0	0
2102	Isoform 1 of U4/U6 small nuclear ribonucleoprotein Prp3	IPI00005861	0.000	0.27422	0	0	2	2
2103	cDNA FLJ60091, highly similar to Hypoxia-inducible factor 1 alpha inhibitor	IPI00299906	0.000	0.27422	0	2	2	2
2104	Transcriptional repressor protein YY1	IPI00014513	0.000	0.27422	0	0	2	2
2105	Isoform 1 of Stimulated by retinoic acid gene 6 protein homolog	IPI00465247	0.000	0.27422	0	0	0	2
2106	WD repeat-containing protein 13	IPI00016988	0.000	0.27422	0	0	0	2
2107	Proteasomal ubiquitin receptor ADRM1	IPI00033030	0.000	0.27422	0	2	0	0
2108	Protein RFT1 homolog	IPI00059368	0.000	0.27422	0	0	0	2
2109	Isoform 3 of Ubiquitin-protein ligase E3C	IPI00411748	0.000	0.27422	2	0	0	0
2110	Isoform 1 of Casein kinase I isoform delta	IPI00011102	0.000	0.27422	0	0	0	2
2111	LanC-like protein 1	IPI00005724	0.000	0.27422	0	2	0	2
2112	Nuclear fragile X mental retardation-interacting protein 2	IPI00002349	0.000	0.27422	0	0	0	2
2113	Isoform 2 of Transcription factor p65	IPI00219084	0.000	0.27422	0	0	2	2
2114	Isoform 1 of Protein CIP2A	IPI00154283	0.000	0.27422	0	2	0	0
2115	Isoform 1 of Cell division protein kinase 8	IPI00026791	0.000	0.27422	2	0	0	0
2116	Beta-centractin	IPI00029469	0.000	0.27422	0	2	0	0
2117	UPF0428 protein CXorf56	IPI00005055	0.000	0.27422	2	2	2	2
2118	28S ribosomal protein S17, mitochondrial	IPI00744772	0.000	0.27422	0	0	2	0
2119	tyrosine-protein phosphatase non-receptor type 2	IPI00106928	0.000	0.27422	0	2	2	0
2120	DNA repair protein complementing XP-A cells	IPI00009694	0.000	0.27422	2	0	2	2
2121	Isoform 1 of Pantothenate kinase 2, mitochondrial	IPI00171176	0.000	0.27422	0	0	2	2
2122	Uridine diphosphate glucose pyrophosphatase	IPI00412878	0.000	0.27422	2	2	2	0
2123	Ubiquitin carboxyl-terminal hydrolase 8	IPI00030915	0.000	0.27422	0	0	2	0
2124	Origin recognition complex subunit 2	IPI00013216	0.000	0.27422	2	0	0	0
2125	Alpha-taxilin	IPI00470779	0.000	0.27422	3	2	3	2
2126	Isoform 1 of Arf-GAP with SH3 domain, ANK repeat and PH domain-containing protein 1	IPI00376976	0.000	0.27422	2	2	0	0
2127	Structural maintenance of chromosomes protein 5	IPI00413265	0.000	0.27422	2	2	0	0
2128	Glia maturation factor, beta	IPI00412987	0.000	0.27422	2	3	3	2
2129	Isoform 2 of Golgin subfamily A member 2	IPI00413895	0.000	0.27422	0	2	2	2
2130	Cleavage and polyadenylation specificity factor subunit 3	IPI00007818	0.000	0.27422	0	2	0	0
2131	Isoform 1 of Putative RNA-binding protein 15	IPI00102752	0.000	0.27422	2	2	0	0
2132	Isoform 1 of Cell division protein kinase 9	IPI00301923	0.000	0.27422	0	2	0	0
2133	Isoform 1 of MIF4G domain-containing protein	IPI00010240	0.000	0.27422	2	2	2	2
2134	Isoform 2 of Zinc finger CCCH-type antiviral protein 1	IPI00332936	0.000	0.27422	2	2	2	2
2135	Isoform 1 of Ribonucleoside-diphosphate reductase subunit M2 B	IPI00100213	0.000	0.27422	2	2	2	2
2136	Borealin	IPI00303099	0.000	0.27422	2	2	0	0
2137	Glioma tumor suppressor candidate region gene 2 protein	IPI00024567	0.000	0.27422	0	2	2	0
2138	NFU1 iron-sulfur cluster scaffold homolog, mitochondrial isoform 1	IPI00160021	0.000	0.27422	3	2	3	2
2139	NHP2-like protein 1	IPI00026167	0.000	0.27422	2	2	2	2
2140	Proteasome assembly chaperone 3	IPI00031106	0.000	0.27422	2	0	0	2
2141	Bleomycin hydrolase	IPI00219575	0.000	0.27422	0	2	0	0
2142	interleukin-1 receptor-associated kinase 1 isoform 3	IPI00060149	0.000	0.27422	0	0	2	0
2143	Plexin-A1	IPI00552671	0.000	0.27422	0	2	0	0
2144	Putative uncharacterized protein FUCA1	IPI00385751	0.000	0.27422	2	2	0	0
2145	Histone deacetylase 4	IPI00010088	0.000	0.27422	0	0	2	0
2146	Isoform 1 of Oxidoreductase HTATIP2	IPI00784029	0.000	0.27422	0	0	0	2
2147	Isoform 1 of TRM1-like protein	IPI00334914	0.000	0.27422	0	0	0	2
2148	Transcriptional enhancer factor TEF-1	IPI00002901	0.000	0.27422	0	2	0	2
2149	Nucleoside diphosphate kinase 3	IPI00012315	0.000	0.27422	2	0	0	0
2150	Isoform 1 of Transmembrane protein 55B	IPI00030530	0.000	0.27422	2	2	2	0
2151	DNA repair endonuclease XPF	IPI00219179	0.000	0.27422	0	0	0	2
2152	Isoform 1 of Microtubule-associated protein RP/EB family member 3	IPI00017597	0.000	0.27422	2	2	0	0
2153	Isoform 1 of ARF GTPase-activating protein GIT1	IPI00384861	0.000	0.27422	0	2	0	0
2154	Putative uncharacterized protein NMD3	IPI00101049	0.000	0.27422	0	2	0	0
2155	Isoform 1 of Ataxin-2	IPI00180154	0.000	0.27422	0	0	0	2
2156	Isoform 1 of Trafficking protein particle complex subunit 2	IPI00005119	0.000	0.27422	2	0	0	0
2157	Peptidylprolyl isomerase domain and WD repeat-containing protein 1	IPI00149650	0.000	0.27422	0	0	0	2
2158	Pleckstrin homology-like domain family A member 2 (Fragment)	IPI00019551	0.000	0.27422	2	2	0	0
2159	Uncharacterized protein C7orf30	IPI00061492	0.000	0.27422	0	0	2	0
2160	NEDD8-activating enzyme E1 catalytic subunit	IPI00328154	0.000	0.27422	0	2	2	0
2161	Ubiquitin protein ligase E3 component n-recognin 4	IPI00514902	0.000	0.27422	0	2	0	2
2162	Isoform 1 of Terminal uridylyltransferase 4	IPI00289861	0.000	0.27422	0	0	2	2
2163	Diphosphomevalonate decarboxylase	IPI00022745	0.000	0.27422	0	2	0	0
2164	Cytochrome b	IPI00015140	0.000	0.27422	0	0	0	2
2165	Diacylglycerol O-acyltransferase 1	IPI00015799	0.000	0.27422	0	0	2	2
2166	MIT domain-containing protein 1	IPI00103065	0.000	0.27422	2	0	2	2
2167	DNA excision repair protein ERCC-1	IPI00014040	0.000	0.27422	2	0	0	0
2168	Isoform 1 of DNA damage-binding protein 2	IPI00021518	0.000	0.27422	0	0	0	2
2169	Tetratricopeptide repeat protein 27	IPI00183938	0.000	0.27422	0	0	0	2
2170	Polyribonucleotide 5'-hydroxyl-kinase Clp1	IPI00024381	0.000	0.27422	0	0	2	2
2171	Ras-related protein Rab-43	IPI00329441	0.000	0.27422	2	0	0	0
2172	Isoform 1 of DNA repair protein XRCC4	IPI00007672	0.000	0.27422	2	0	0	0
2173	Kinesin-like protein KIF14	IPI00299554	0.000	0.27422	2	0	0	0
2174	Isoform 1 of Zinc finger protein 185	IPI00005688	0.000	0.27422	0	2	0	0
2175	cDNA FLJ55380, highly similar to Protein kinase C-binding protein 1	IPI00418316	0.000	0.27422	0	2	0	0
2176	Ubiquitin-like domain-containing CTD phosphatase 1	IPI00291669	0.000	0.27422	0	2	0	0

No.	Description	Accession number	STN[1]	p-Value[1]	480_A[2]	480_B[2]	620_A[2]	620_B[2]
2177	Acetyl-coenzyme A synthetase, cytoplasmic	IPI00413730	0.000	0.27422	0	0	0	2
2178	Isoform 1 of Protein disulfide-isomerase TMX3	IPI00064193	0.000	0.27422	0	0	2	2
2179	Coiled-coil domain-containing protein 134	IPI00302674	0.000	0.27422	0	2	2	0
2180	HSR1 protein	IPI00384745	0.000	0.27422	2	2	2	2
2181	Isoform 2 of DnaJ homolog subfamily A member 4	IPI00465105	0.000	0.27422	0	2	2	2
2182	Protein unc-119 homolog B	IPI00414629	0.000	0.27422	2	2	2	0
2183	cDNA FLJ59751, weakly similar to Mus musculus spermatogenesis associated, serine-rich 2 (Spats2)	IPI00023532	0.000	0.27422	0	0	0	2
2184	Notchless protein homolog 1	IPI00018196	0.000	0.27422	0	2	2	2
2185	CDGSH iron sulfur domain-containing protein 2	IPI00166865	0.000	0.27422	2	2	0	0
2186	Isoform 1 of Elongator complex protein 3	IPI00165477	0.000	0.27422	0	0	0	2
2187	Mitogen-activated protein kinase scaffold protein 1	IPI00030919	0.000	0.27422	0	2	0	0
2188	Protein spinster homolog 2	IPI00217163	0.000	0.27422	0	0	0	2
2189	Pyrroline-5-carboxylate reductase	IPI00550882	0.000	0.27422	0	0	0	2
2190	PDZ domain-containing protein 8	IPI00168698	0.000	0.27422	0	0	2	0
2191	Isoform 2 of Ribonuclease P protein subunit p40	IPI00332091	0.000	0.27422	0	2	0	0
2192	Isoform 1 of Dynamin-3	IPI00221332	0.000	0.27422	2	0	0	2
2193	homeobox protein MSX-1	IPI00002939	0.000	0.27422	2	0	0	0
2194	49 kDa protein	IPI00013651	0.000	0.27422	0	0	0	2
2195	Isoform 1 of WD repeat-containing protein 26	IPI00414197	0.000	0.27422	0	0	0	2
2196	N-myc-interactor	IPI00012450	0.000	0.27422	2	0	0	0
2197	Conserved hypothetical protein	IPI00477526	0.000	0.27422	0	0	2	0
2198	Amine oxidase [flavin-containing] B	IPI00328156	0.000	0.27422	2	0	0	0
2199	dehydrogenase/reductase SDR family member 4	IPI00106913	0.000	0.27422	2	0	0	0
2200	Isoform 1 of Citrate lyase subunit beta-like protein, mitochondrial	IPI00477957	0.000	0.27422	0	0	0	2
2201	Isoform 1 of Clathrin coat assembly protein AP180	IPI00006612	0.000	0.27422	0	2	0	0
2202	Isoform 1 of Transmembrane protein 126B	IPI00020540	0.000	0.27422	0	2	0	0
2203	Plasma alpha-L-fucosidase	IPI00012440	0.000	0.27422	0	2	0	0
2204	Isoform A of SWI/SNF-related matrix-associated actin-dependent regulator of chromatin subfamily B	IPI00029695	0.000	0.27422	2	2	2	0
2205	CDKN2A-interacting protein	IPI00020991	0.000	0.27422	2	2	0	0
2206	Isoform 2 of Lysocardiolipin acyltransferase 1	IPI00166225	0.000	0.27422	0	0	0	2
2207	F-box only protein 7	IPI00294567	0.000	0.27422	2	0	2	0
2208	Isoform 1 of Pre-mRNA-splicing factor RBM22	IPI00019046	0.000	0.27422	0	2	2	0
2209	Isoform 2 of Exosome complex exonuclease RRP45	IPI00029697	0.000	0.27422	0	2	0	0
2210	Isoform 7 of Serine/threonine-protein kinase MARK2	IPI00290158	0.000	0.27422	0	0	2	0
2211	Isoform 2 of Suppressor of SWI4 1 homolog	IPI00219793	0.000	0.27422	0	0	2	0
2212	Pentatricopeptide repeat-containing protein 1	IPI00171925	0.000	0.27422	0	2	2	2
2213	Carnitine O-palmitoyltransferase 2, mitochondrial	IPI00012912	0.000	0.27422	0	2	2	2
2214	Endonuclease/exonuclease/phosphatase family domain-containing protein 1	IPI00885036	0.000	0.27422	0	0	2	2
2215	Dynactin subunit 4	IPI00550852	0.000	0.27422	2	0	2	0
2216	Hydroxymethylglutaryl-CoA lyase, mitochondrial	IPI00293564	0.000	0.27422	0	2	0	0
2217	Isoform 1 of Protein GPR89	IPI00472858	0.000	0.27422	0	0	2	0
2218	Isoform 3 of Vesicle transport protein SEC20	IPI00030397	0.000	0.27422	0	0	2	0
2219	NF-kappa-B-repressing factor	IPI00005675	0.000	0.27422	0	2	0	0
2220	cDNA FLJ10079 fis, clone HEMBA1001896, weakly similar to DIMETHYLGLYCINE DEHYDROGENASE	IPI00017494	0.000	0.27422	0	0	2	0
2221	Isoform 2 of Nuclear factor NF-kappa-B p100 subunit	IPI00411715	0.000	0.27422	2	2	0	0
2222	Isoform C of Nuclear factor of activated T-cells 5	IPI00217710	0.000	0.27422	0	2	2	2
2223	Isoform 1 of HEAT repeat-containing protein 3	IPI00100984	0.000	0.27422	0	0	0	2
2224	Lanosterol synthase	IPI00009747	0.000	0.27422	2	2	0	0
2225	Isoform 1 of Uncharacterized protein C9orf142	IPI00030968	0.000	0.27422	2	0	2	0
2226	Cell division cycle protein 27 homolog	IPI00294575	0.000	0.27422	0	0	2	0
2227	Isoform 1 of Multiple myeloma tumor-associated protein 2	IPI00293746	0.000	0.27422	0	0	2	2
2228	Isoform 1 of OTU domain-containing protein 4	IPI00399254	0.000	0.27422	0	2	0	0
2229	Cytochrome P450 monooxygenase	IPI00010218	0.000	0.27422	0	2	2	2
2230	Wings apart-like homolog	IPI00103263	0.000	0.27422	0	2	0	0
2231	X-Pro aminopeptidase 1, soluble isoform 2	IPI00607814	0.000	0.27422	2	0	2	2
2232	Helicase ARIP4	IPI00294787	0.000	0.27422	0	2	0	2
2233	Ubiquitin-associated domain-containing protein 1	IPI00305442	0.000	0.27422	2	2	0	0
2234	15 kDa protein	IPI00000186	0.000	0.27422	0	2	0	2
2235	Isoform 1 of UPF0449 protein C19orf25	IPI00298662	0.000	0.27422	2	2	0	2
2236	Kinesin-like protein KIF3A	IPI00000455	0.000	0.27422	2	0	0	2
2237	Mediator of RNA polymerase II transcription subunit 28	IPI00097532	0.000	0.27422	0	0	0	2
2238	Neuronal calcium sensor 1	IPI00219110	0.000	0.27422	0	0	0	2
2239	WASH complex subunit CCDC53	IPI00032848	0.000	0.27422	0	0	2	2
2240	Ras-related protein Rab-23	IPI00008034	0.000	0.27422	0	0	2	0
2241	Isoform 2 of INO80 complex subunit C	IPI00167658	0.000	0.27422	0	0	0	2
2242	Isoform 1 of Cytosolic Fe-S cluster assembly factor NUBP1	IPI00021277	0.000	0.27422	0	0	2	2
2243	Sororin	IPI00061989	0.000	0.27422	0	2	2	0
2244	Armadillo repeat-containing protein 6	IPI00020196	0.000	0.27422	2	2	0	0
2245	Chloride intracellular channel protein 3	IPI00000692	0.000	0.27422	0	0	2	0
2246	MORC family CW-type zinc finger 3	IPI00436705	0.000	0.27422	2	0	0	0
2247	Isoform SV12 of Cell division protein kinase 11A	IPI00024413	0.000	0.27422	0	2	0	0
2248	Isoform 1 of BRCA1-A complex subunit MERIT40	IPI00101987	0.000	0.27422	0	0	0	2
2249	NmrA-like family protein	IPI00745792	0.000	0.27422	0	0	2	0
2250	Isoform 3 of Sorting nexin-27	IPI00328097	0.000	0.27422	0	0	2	0
2251	Ribosome production factor 1	IPI00292221	0.000	0.27422	0	0	2	0
2252	Isoform SV7 of Cell division protein kinase 11A	IPI00024414	0.000	0.27422	0	2	0	0
2253	Isoform 1 of Ubiquitin carboxyl-terminal hydrolase 28	IPI00045496	0.000	0.27422	0	0	0	2
2254	Isoform 1 of Inhibitor of nuclear factor kappa-B kinase-interacting protein	IPI00797136	0.000	0.27422	0	0	2	2
2255	Isoform 1 of Lysine-specific demethylase 5C	IPI00013185	0.000	0.27422	0	0	0	2
2256	GDH/6PGL endoplasmic bifunctional protein	IPI00607861	0.000	0.27422	0	2	0	0
2257	Isoform 2 of TSC22 domain family protein 1	IPI00019355	0.000	0.27422	0	2	0	0
2258	Isoform 1 of Nuclear ubiquitous casein and cyclin-dependent kinases substrate	IPI00022145	0.000	0.27422	0	0	2	0
2259	Gamma-taxilin	IPI00019994	0.000	0.27422	0	0	0	2
2260	Isoform 1 of Nicotinamide mononucleotide adenylyltransferase 3	IPI00290687	0.000	0.27422	2	0	0	0
2261	Sorting nexin-4	IPI00029403	0.000	0.27422	0	0	2	2
2262	FtsJ methyltransferase domain-containing protein 1	IPI00334846	0.000	0.27422	0	0	2	2
2263	Uncharacterized protein C19orf52	IPI00157215	0.000	0.27422	2	2	2	0
2264	Glycolipid transfer protein	IPI00184363	0.000	0.27422	0	0	2	2
2265	Uncharacterized protein C1orf198	IPI00013912	0.000	0.27422	0	0	0	2
2266	Isoform B of Syntaxin-16	IPI00023149	0.000	0.27422	2	2	0	0
2267	Isoform 2 of Alkylated DNA repair protein alkB homolog 5	IPI00413659	0.000	0.27422	0	2	0	0
2268	Isoform 1 of Septin-10	IPI00374970	0.000	0.27422	2	2	0	0
2269	Mediator of RNA polymerase II transcription subunit 30	IPI00063213	0.000	0.27422	2	2	0	0

No.	Description	Accession number	STN[1]	p-Value[1]	480_A[2]	480_B[2]	620_A[2]	620_B[2]
2270	Golgi integral membrane protein 4	IPI00004962	0.000	0.27422	0	2	2	0
2271	Isoform 2 of Insulin-like growth factor 2 mRNA-binding protein 3	IPI00165467	0.000	0.27422	0	0	2	0
2272	Kanadaptin	IPI00306749	0.000	0.27422	2	0	0	0
2273	Isoform 1 of Kinesin-like protein KIF2A	IPI00010368	0.000	0.27422	0	2	2	0
2274	Formin-binding protein 4	IPI00170778	0.000	0.27422	0	2	0	0
2275	B-cell lymphoma/leukemia 10	IPI00022477	0.000	0.27422	2	2	2	2
2276	Alba-like protein C9orf23	IPI00166873	0.000	0.27422	0	0	2	0
2277	Uncharacterized protein C3orf26	IPI00031679	0.000	0.27422	0	0	2	0
2278	Isoform 2 of ADP-ribosylation factor-binding protein GGA1	IPI00216337	0.000	0.27422	0	2	0	2
2279	A-kinase anchor protein 8	IPI00014474	0.000	0.27422	0	0	2	2
2280	Tumor necrosis factor receptor type 1-associated DEATH domain protein	IPI00018744	0.000	0.27422	0	2	0	0
2281	LLGL1 protein	IPI00791938	0.000	0.27422	2	0	0	0
2282	Isoform 1 of Adaptin ear-binding coat-associated protein 2	IPI00018188	0.000	0.27422	2	0	0	0
2283	Putative uncharacterized protein RANGRF	IPI00023552	0.000	0.27422	0	0	0	2
2284	Isoform 2 of Succinyl-CoA ligase [ADP-forming] subunit beta, mitochondrial	IPI00217232	0.000	0.27422	0	2	0	0
2285	Guanine nucleotide-binding protein subunit alpha-11	IPI00305551	0.000	0.27422	2	0	0	0
2286	Isoform 2 of VIP36-like protein	IPI00218337	0.000	0.27422	2	0	0	0
2287	Isoform 2 of 6-phosphofructo-2-kinase/fructose-2,6-biphosphatase 2	IPI00220808	0.000	0.27422	2	0	0	2
2288	Isoform 1 of Cytosolic non-specific dipeptidase	IPI00177728	0.000	0.27422	0	2	2	2
2289	Putative uncharacterized protein QTRTD1	IPI00074010	0.000	0.27422	2	2	2	0
2290	39S ribosomal protein L38, mitochondrial	IPI00783656	0.000	0.27422	0	0	2	2
2291	Isoform 1 of Peptidyl-prolyl cis-trans isomerase SDCCAG10	IPI00025174	0.000	0.27422	0	0	0	2
2292	Serine/threonine-protein kinase 6	IPI00298940	0.000	0.27422	2	0	0	0
2293	Inositol polyphosphate 1-phosphatase	IPI00027139	0.000	0.27422	0	0	2	0
2294	Isoform 1 of YTH domain-containing protein 1	IPI00144293	0.000	0.27422	0	2	0	2
2295	Isoform 3 of Histone lysine demethylase PHF8	IPI00183302	0.000	0.27422	0	0	2	0
2296	Glyoxylate reductase/hydroxypyruvate reductase, isoform CRA_c	IPI00026486	0.000	0.27422	0	2	0	0
2297	Putative uncharacterized protein LCMT1	IPI00296370	0.000	0.27422	0	0	0	2
2298	Isoform 1 of F-box only protein 38	IPI00456676	0.000	0.27422	2	0	0	0
2299	Isoform 1 of Breast cancer anti-estrogen resistance protein 3	IPI00179053	0.000	0.27422	0	2	0	0
2300	G1/S-specific cyclin-D1	IPI00028098	0.000	0.27422	0	2	0	0
2301	Xaa-Pro dipeptidase	IPI00257882	0.000	0.27422	0	0	2	0
2302	Isoform 2 of Disks large homolog 1	IPI00218729	0.000	0.27422	0	2	0	0
2303	Nuclear pore complex protein Nup88	IPI00001738	0.000	0.27422	0	2	0	0
2304	Isoform Long of Plakophilin-4	IPI00021076	0.000	0.27422	0	2	0	0
2305	Arylamine N-acetyltransferase 1	IPI00644361	0.000	0.27422	0	2	0	0
2306	Transcriptional activator protein Pur-beta	IPI00045051	0.000	0.27422	2	2	0	0
2307	Zinc finger protein 593	IPI00844193	0.000	0.27422	0	2	0	0
2308	Leukocyte receptor cluster member 1	IPI00100947	0.000	0.27422	2	0	0	0
2309	164 kDa protein	IPI00465246	0.000	0.27422	0	0	0	2
2310	Protein disulfide-isomerase A5	IPI00031479	0.000	0.27422	2	2	0	0
2311	Ribosomal protein S6 kinase alpha-5	IPI00335101	0.000	0.27422	2	2	0	0
2312	La-related protein 7	IPI00294742	0.000	0.27422	2	2	0	0
2313	Isoform 1 of Choline kinase alpha	IPI00409761	0.000	0.27422	0	0	0	2
2314	Guanine nucleotide exchange factor MSS4	IPI00023939	0.000	0.27422	0	0	0	2
2315	Ras-related protein Rab-39B	IPI00060801	0.000	0.27422	2	2	0	0
2316	Ubiquitin domain-containing protein UBFD1	IPI00005194	0.000	0.27422	0	0	2	2
2317	Isoform 1 of Type-1 angiotensin II receptor-associated protein	IPI00102501	0.000	0.27422	2	0	0	0
2318	Isoform 1 of Protein FAM177A1	IPI00166051	0.000	0.27422	0	2	0	0
2319	Isoform 1 of Vacuolar-sorting protein SNF8	IPI00101524	0.000	0.27422	0	0	2	0
2320	Isoform 1 of Nucleotide-binding protein-like	IPI00384517	0.000	0.27422	0	0	0	2
2321	3-oxoacyl-[acyl-carrier-protein] synthase, mitochondrial	IPI00016637	0.000	0.27422	0	2	0	0
2322	Isoform Long of Autophagy protein 5	IPI00006800	0.000	0.27422	0	0	0	2
2323	Alpha- and gamma-adaptin-binding protein p34	IPI00100193	0.000	0.27422	2	0	0	0
2324	cDNA FLJ10321 fis, clone NT2RM2000504, highly similar to Homo sapiens pitrilysin metallopeptidase	IPI00219613	0.000	0.27422	0	0	0	2
2325	Mitochondrial import inner membrane translocase subunit Tim8 A	IPI00028376	0.000	0.27422	2	0	2	2
2326	39S ribosomal protein L32, mitochondrial precursor	IPI00011077	0.000	0.27422	2	2	2	0
2327	cDNA FLJ35172 fis, clone PLACE6013232	IPI00385785	0.000	0.27422	2	2	0	0
2328	Pro-apoptotic protein BAKM variant	IPI00386229	0.000	0.27422	0	0	2	0
2329	Isoform 2 of Mitochondrial ribonuclease P protein 3	IPI00004584	0.000	0.27422	0	0	0	2
2330	Dynein light chain Tctex-type 1	IPI00019495	0.000	0.27422	2	0	0	0
2331	Isoform 1 of YTH domain family protein 2	IPI00306043	0.000	0.27422	0	2	0	0
2332	Kinetochore-associated protein NSL1 homolog	IPI00306330	0.000	0.27422	0	2	2	0
2333	Isoform 1 of Caspase-8	IPI00000149	0.000	0.27422	0	2	2	0
2334	Alpha-galactosidase A	IPI00025869	0.000	0.27422	0	2	2	2
2335	Isoform 1 of Anaphase-promoting complex subunit 7	IPI00008248	0.000	0.27422	0	2	0	2
2336	Heterochromatin protein 1, binding protein 3	IPI00640417	0.000	0.27422	0	0	0	2
2337	Isoform 4 of Sarcolemmal membrane-associated protein	IPI00026691	0.000	0.27422	2	0	0	0
2338	Proline-rich protein PRCC	IPI00294618	0.000	0.27422	0	0	0	2
2339	Isoform 1 of Serine/threonine-protein kinase TAO1	IPI00002232	0.000	0.27422	2	0	0	0
2340	Uncharacterized protein C20orf29	IPI00019941	0.000	0.27422	0	2	0	0
2341	Isoform 1 of Acetoacetyl-CoA synthetase	IPI00217272	0.000	0.27422	0	0	2	2
2342	Arfaptin-2	IPI00021257	0.000	0.27422	2	0	2	0
2343	Isoform A of Methyl-CpG-binding protein 2	IPI00418234	0.000	0.27422	0	2	0	2
2344	Putative transferase C1orf69, mitochondrial	IPI00145260	0.000	0.27422	0	0	2	2
2345	Isoform 1 of Quinone oxidoreductase PIG3	IPI00384643	0.000	0.27422	0	0	0	2
2346	Probable ATP-dependent RNA helicase DDX49	IPI00003739	0.000	0.27422	0	0	0	2
2347	High mobility group nucleosome-binding domain-containing protein 5	IPI00006157	0.000	0.27422	0	0	0	2
2348	Isoform 2 of Atlastin-2	IPI00007183	0.000	0.27422	0	0	2	0
2349	Ubiquilin-4	IPI00024502	0.000	0.27422	0	0	0	2
2350	Isoform 1 of Alpha-(1,6)-fucosyltransferase	IPI00004668	0.000	0.27422	2	2	0	0
2351	Cytochrome c oxidase subunit 7A-related protein, mitochondrial	IPI00022421	0.000	0.27422	0	0	2	2
2352	Kinesin light chain 2	IPI00021634	0.000	0.27422	0	2	0	0
2353	MON2 protein (Fragment)	IPI00439706	0.000	0.27422	0	0	0	2
2354	Isoform 3 of Sarcolemmal membrane-associated protein	IPI00030531	0.000	0.27422	0	2	0	0
2355	Isoform 2 of Coiled-coil domain-containing protein 132	IPI00103148	0.000	0.27422	0	2	2	0
2356	cDNA FLJ61629, highly similar to Clathrin interactor 1	IPI00291930	0.000	0.27422	0	2	2	0
2357	Isoform 1 of Nucleoporin GLE1	IPI00301647	0.000	0.27422	0	0	0	2
2358	69 kDa protein	IPI00172590	0.000	0.27422	0	0	0	2
2359	Isoform 1 of Bcl-2-like protein 12	IPI00019835	0.000	0.27422	2	2	0	0
2360	Integrin alpha-2	IPI00013744	0.000	0.27422	2	2	0	0
2361	Fructosamine-3-kinase	IPI00023729	0.000	0.27422	0	2	0	0
2362	Isoform 1 of Anoctamin-1	IPI00395839	0.000	0.27422	2	0	0	0
2363	Splicing factor, arginine/serine-rich 8	IPI00290094	0.000	0.27422	0	0	2	0

No.	Description	Accession number	STN[1]	p-Value[1]	480_A[2]	480_B[2]	620_A[2]	620_B[2]
2364	KIAA1033 protein	IPI00298991	0.000	0.27422	0	0	2	0
2365	Meiotic nuclear division protein 1 homolog	IPI00029810	0.000	0.27422	0	0	2	2
2366	Isoform Long of Vesicle transport through interaction with t-SNAREs homolog 1B	IPI00063784	0.000	0.27422	0	0	0	2
2367	Isoform 3 of PDZ and LIM domain protein 2	IPI00007983	0.000	0.27422	2	2	0	0
2368	Zinc finger protein ubi-d4	IPI00023322	0.000	0.27422	0	0	2	2
2369	V-type proton ATPase subunit S1	IPI00784119	0.000	0.27422	0	0	2	2
2370	Cytospin-A	IPI00178072	0.000	0.27422	0	2	0	0
2371	Digestive organ expansion factor homolog	IPI00004290	0.000	0.27422	0	0	2	0
2372	Perilipin-2	IPI00293307	0.000	0.27422	0	0	0	2
2373	Isoform 1 of Mucosa-associated lymphoid tissue lymphoma translocation protein 1	IPI00009540	0.000	0.27422	2	2	0	0
2374	Isoform 1 of Protein FAM169A	IPI00737638	0.000	0.27422	0	0	0	2
2375	RNA-binding protein 27	IPI00292975	0.000	0.27422	0	2	0	0
2376	Protein HEXIM1	IPI00007941	0.000	0.27422	0	0	2	2
2377	Isoform 3 of Epithelial splicing regulatory protein 1	IPI00184262	0.000	0.27422	0	0	0	2
2378	Isoform 1 of Uncharacterized protein C3orf63	IPI00790098	0.000	0.27422	0	0	2	0
2379	cDNA, FLJ79184, highly similar to Procollagen-lysine, 2-oxoglutarate 5-dioxygenase 1	IPI00027192	0.000	0.27422	2	2	0	0
2380	Isoform 1 of HLA class II histocompatibility antigen gamma chain	IPI00022933	0.000	0.27422	2	2	0	0
2381	Isoform 1 of Tripartite motif-containing protein 16	IPI00007955	0.000	0.27422	2	0	0	0
2382	Protein Niban	IPI00328350	0.000	0.27422	2	2	0	0
2383	Isoform 3 of Chromatin modification-related protein MEAF6	IPI00009373	0.000	0.27422	0	0	0	2
2384	Iron-sulfur cluster assembly 2 homolog, mitochondrial	IPI00376195	0.000	0.27422	0	2	0	0
2385	p53 and DNA damage-regulated protein 1	IPI00027887	0.000	0.27422	0	0	0	2
2386	Dipeptidase 1	IPI00059476	0.000	0.27422	0	0	0	2
2387	Splicing factor, arginine/serine-rich 11	IPI00464952	0.000	0.27422	0	0	2	0
2388	Active regulator of SIRT1	IPI00219006	0.000	0.27422	2	0	0	0
2389	Mitochondrial import inner membrane translocase subunit Tim10	IPI00001543	0.000	0.27422	0	0	0	2
2390	Isoform 2 of Coiled-coil-helix-coiled-coil-helix domain-containing protein 8	IPI00009817	0.000	0.27422	0	0	2	2
2391	Isoform 2 of Serine/threonine-protein kinase PAK 1	IPI00289746	0.000	0.27422	0	0	0	2
2392	Isoform 1 of Retinoid-inducible serine carboxypeptidase	IPI00012426	0.000	0.27422	0	0	2	0
2393	Neutrophil gelatinase-associated lipocalin	IPI00299547	0.000	0.27422	0	2	0	0
2394	17 kDa protein	IPI00643390	0.000	0.27422	0	0	2	2
2395	U3 small nucleolar RNA-associated protein 6 homolog	IPI00020128	0.000	0.27422	0	0	0	2
2396	Syntaxin-18	IPI00027194	0.000	0.27422	0	0	2	2
2397	Isoform B of Syntaxin-3	IPI00220099	0.000	0.27422	0	0	0	2
2398	Isoform 1 of Eukaryotic initiation factor 4A-II	IPI00328328	0.000	0.27422	2	2	0	0
2399	U3 small nucleolar ribonucleoprotein protein IMP4	IPI00181116	0.000	0.27422	0	2	0	2
2400	Interferon-related developmental regulator 2, isoform CRA_b	IPI00395667	0.000	0.27422	0	0	0	2
2401	Isoform 3 of Tropomyosin beta chain	IPI00218820	0.000	0.27422	0	0	2	2
2402	Isoform 1 of Endoplasmic reticulum-Golgi intermediate compartment protein 3	IPI00006438	0.000	0.27422	0	0	2	2
2403	Exosome complex exonuclease RRP46	IPI00015955	0.000	0.27422	2	0	0	0
2404	cDNA FLJ56469, highly similar to Propionyl-CoA carboxylase alpha chain, mitochondrial	IPI00552419	0.000	0.27422	2	0	0	0
2405	DNA polymerase delta subunit 3	IPI00394926	0.000	0.27422	0	0	2	0
2406	Isoform 1 of Polyadenylate-binding protein-interacting protein 1	IPI00021466	0.000	0.27422	0	0	2	0
2407	Zinc finger protein 622	IPI00056499	0.000	0.27422	0	0	0	2
2408	Isoform 1 of GPI transamidase component PIG-S	IPI00465308	0.000	0.27422	0	2	0	0
2409	Transmembrane protein 214	IPI00477118	0.000	0.27422	2	0	0	0
2410	Putative uncharacterized protein ZNF326	IPI00337602	0.000	0.27422	0	0	2	0
2411	Desmoglein-2	IPI00028931	0.000	0.27422	0	0	2	0
2412	ATP-dependent RNA helicase DDX55	IPI00185361	0.000	0.27422	0	0	0	2
2413	Isoform 1 of RNA-binding protein Raly	IPI00216044	0.000	0.27422	0	0	2	0
2414	Isoform 2 of ATP-binding cassette sub-family B member 6, mitochondrial	IPI00065486	0.000	0.27422	2	0	0	0
2415	Tetratricopeptide repeat protein 19	IPI00170855	0.000	0.27422	0	0	2	0
2416	ArgBPIB protein	IPI00299588	0.000	0.27422	0	0	2	0
2417	Isoform Mitochondrial of Cysteine desulfurase, mitochondrial	IPI00295240	0.000	0.27422	0	0	0	2
2418	cDNA: FLJ22221 fis, clone HRC01651	IPI00184854	0.000	0.27422	0	0	0	2
2419	Vacuolar protein sorting-associated protein 11 homolog	IPI00300697	0.000	0.27422	0	0	2	0
2420	Golgi-associated plant pathogenesis-related protein 1	IPI00007067	0.000	0.27422	2	2	0	0
2421	Isoform 4 of Inhibitor of nuclear factor kappa-B kinase-interacting protein	IPI00043598	0.000	0.27422	0	0	2	2
2422	Protein Njmu-R1	IPI00029473	0.000	0.27422	0	2	0	0
2423	Isoform 1 of Exocyst complex component 3	IPI00157734	0.000	0.27422	0	0	2	0
2424	Isoform 1 of Zinc finger protein 326	IPI00373877	0.000	0.27422	0	2	0	2
2425	Isoform 3 of Protein LAS1 homolog	IPI00009917	0.000	0.27422	0	0	2	0
2426	Isoform 1 of tRNA pseudouridine synthase-like 1	IPI00166125	0.000	0.27422	0	0	2	0
2427	Isoform 1 of Mitochondrial Rho GTPase 2	IPI00465059	0.000	0.27422	2	0	0	0
2428	DNA replication complex GINS protein PSF1	IPI00032387	0.000	0.27422	0	0	2	0
2429	ATP-binding domain-containing protein 4	IPI00063121	0.000	0.27422	0	0	2	2
2430	RNA-binding protein 7	IPI00001134	0.000	0.27422	0	0	2	2
2431	Isoform A of Ketohexokinase	IPI00029488	0.000	0.27422	0	2	0	0
2432	Isoform 1 of Kinesin-like protein KIF1A	IPI00604711	0.000	0.27422	2	0	0	0
2433	cDNA FLJ58610	IPI00100930	0.000	0.27422	0	0	2	0
2434	UV excision repair protein RAD23 homolog A	IPI00008219	0.000	0.27422	0	0	0	2
2435	Isoform 1 of Non-structural maintenance of chromosomes element 4 homolog A	IPI00014367	0.000	0.27422	2	2	0	0
2436	115 kDa protein	IPI00009322	0.000	0.27422	0	0	2	2
2437	Isoform 1 of Phosphoenolpyruvate carboxykinase [GTP], mitochondrial	IPI00797038	0.000	0.27422	0	0	2	2
2438	Disintegrin and metalloproteinase domain-containing protein 10	IPI00013897	0.000	0.27422	0	0	2	2
2439	Isoform 1 of NADH dehydrogenase [ubiquinone] 1 alpha subcomplex subunit 11	IPI00329301	0.000	0.27422	0	2	0	0
2440	Isoform 1 of Structural maintenance of chromosomes protein 6	IPI00154528	0.000	0.27422	0	0	0	2
2441	Adenosine monophosphate deaminase 2	IPI00007722	0.000	0.27422	0	0	2	0
2442	Isoform 1 of SH3KBP1-binding protein 1	IPI00163403	0.000	0.27422	2	2	0	0
2443	Thyroid receptor-interacting protein 6	IPI00301561	0.000	0.27422	2	0	0	0
2444	Uncharacterized protein KIAA0355	IPI00004527	0.000	0.27422	2	0	0	0
2445	Serum amyloid A protein	IPI00552578	0.000	0.27422	0	2	0	0
2446	Vacuolar fusion protein MON1 homolog B	IPI00848138	0.000	0.27422	0	0	2	0
2447	Isoform 1 of Protein max	IPI00018214	0.000	0.27422	0	0	0	2
2448	Isoform 1 of 5'-nucleotidase domain-containing protein 3	IPI00465170	0.000	0.27422	0	0	2	0
2449	Isoform B of Endothelin-converting enzyme 1	IPI00002478	0.000	0.27422	2	0	0	0
2450	Protein FAM96B	IPI00007024	0.000	0.27422	0	0	2	0
2451	Ribosomal protein 63, mitochondrial	IPI00031485	0.000	0.27422	0	0	0	2
2452	NTF2-related export protein 1	IPI00007605	0.000	0.27422	0	0	2	0
2453	Protein UXT	IPI00002646	0.000	0.27422	2	0	0	0
2454	Dihydropyrimidinase-related protein 1	IPI00414123	0.000	0.27422	0	2	0	0
2455	Nucleoside diphosphate kinase 7	IPI00294997	0.000	0.27422	0	0	0	2
2456	Isoform 1 of HAUS augmin-like complex subunit 4	IPI00302658	0.000	0.27422	0	0	0	2
2457	Isoform 2 of Rab9 effector protein with kelch motifs	IPI00384623	0.000	0.27422	0	0	0	2
2458	Isoform 1 of Golgin subfamily A member 5	IPI00294065	0.000	0.27422	0	2	0	0

No.	Description	Accession number	STN[1]	p-Value[1]	480_A[2]	480_B[2]	620_A[2]	620_B[2]
2459	Torsin A interacting protein 1	IPI00644766	0.000	0.27422	0	0	2	0
2460	RING1 and YY1-binding protein	IPI00296594	0.000	0.27422	0	2	0	0
2461	cDNA FLJ55772, highly similar to Rab5 GDP/GTP exchange factor	IPI00004974	0.000	0.27422	0	0	0	2
2462	Putative uncharacterized protein WDR43	IPI00892938	0.000	0.27422	2	0	0	0
2463	Isoform 3 of Dual specificity protein kinase CLK3	IPI00219341	0.000	0.27422	0	0	2	0
2464	Ribosomal RNA-processing protein 8	IPI00304932	0.000	0.27422	0	0	2	2
2465	Isoform 1 of Cytoskeleton-associated protein 2	IPI00071824	0.000	0.27422	2	2	0	0
2466	tropomyosin alpha-1 chain isoform 7	IPI00216134	0.000	0.27422	2	0	0	0
2467	Aldose 1-epimerase	IPI00060200	0.000	0.27422	2	2	0	0
2468	Isoform 1 of AN1-type zinc finger protein 1	IPI00291064	0.000	0.27422	0	0	2	0
2469	TBC1 domain family member 7	IPI00009425	0.000	0.27422	0	2	0	0
2470	Isoform 1 of Bromodomain-containing protein 8	IPI00019226	0.000	0.27422	2	0	0	0
2471	Centromere protein Q	IPI00018689	0.000	0.27422	2	0	0	0
2472	Isoform 2 of Proline-rich AKT1 substrate 1	IPI00306195	0.000	0.27422	0	2	0	0
2473	Pyruvate dehydrogenase phosphatase regulatory subunit, mitochondrial	IPI00168407	0.000	0.27422	0	0	0	2
2474	Mitochondrial transmembrane GTPase FZO-2	IPI00293073	0.000	0.27422	0	2	0	0
2475	Putative uncharacterized protein ATP6AP1	IPI00552748	0.000	0.27422	2	0	0	0
2476	Isoform Beta-1 of Protein phosphatase 1B	IPI00026612	0.000	0.27422	0	2	0	0
2477	Isoform Alpha of DNA fragmentation factor subunit beta (Fragment)	IPI00008794	0.000	0.27422	0	0	2	0
2478	Putative uncharacterized protein KIF20B	IPI00044751	0.000	0.27422	0	0	2	0
2479	Kinesin-like protein KIF20A	IPI00029422	0.000	0.27422	0	2	0	0
2480	Isoform 1 of Zinc finger CCCH-type antiviral protein 1	IPI00410067	0.000	0.27422	0	0	2	0
2481	Isoform 1 of Putative S-adenosyl-L-methionine-dependent methyltransferase METT5D1	IPI00783001	0.000	0.27422	0	0	0	2
2482	Coiled-coil domain-containing protein 137	IPI00401962	0.000	0.27422	2	0	0	0
2483	Isoform 3 of MAP7 domain-containing protein 3	IPI00217264	0.000	0.27422	2	2	0	0
2484	Isoform 1 of Autophagy-related protein 7	IPI00007404	0.000	0.27422	2	0	0	0
2485	Isoform 3 of Bromodomain-containing protein 8	IPI00016570	0.000	0.27422	0	2	0	0
2486	Ras-related protein Rap-2a	IPI00019346	0.000	0.27422	0	0	0	2
2487	Isoform 1 of Kelch domain-containing protein 4	IPI00152182	0.000	0.27422	0	0	2	0
2488	Putative uncharacterized protein DHX30	IPI00164906	0.000	0.27422	0	2	0	0
2489	Uncharacterized protein C8orf41	IPI00306207	0.000	0.27422	0	0	2	0
2490	Putative Ras-related protein Rab-12	IPI00419932	0.000	0.27422	2	2	0	0
2491	UPF0444 transmembrane protein C12orf23	IPI00184546	0.000	0.27422	0	0	0	2
2492	Isoform Delta 6 of Calcium/calmodulin-dependent protein kinase type II subunit delta	IPI00172636	0.000	0.27422	0	0	2	2
2493	Isoform 1 of Probable Xaa-Pro aminopeptidase 3	IPI00550192	0.000	0.27422	0	0	2	0
2494	Isoform 1 of SAC3 domain-containing protein 1	IPI00854724	0.000	0.27422	0	0	0	2
2495	Methylcrotonoyl-CoA carboxylase subunit alpha, mitochondrial	IPI00024580	0.000	0.27422	0	2	0	0
2496	Isoform 1 of Probable methyltransferase C20orf7, mitochondrial	IPI00106573	0.000	0.27422	0	0	0	2
2497	WD repeat-containing protein 70	IPI00300060	0.000	0.27422	0	0	0	2
2498	Isoform 1 of Pyridoxal-dependent decarboxylase domain-containing protein 1	IPI00384689	0.000	0.27422	2	0	0	0
2499	MORF4 family-associated protein 1	IPI00020915	0.000	0.27422	0	2	0	0
2500	Isoform 1 of UPF0366 protein C11orf67	IPI00100775	0.000	0.27422	0	2	0	0
2501	Thioredoxin-like protein 4A	IPI00216338	0.000	0.27422	0	2	0	0
2502	Putative uncharacterized protein PYCRL	IPI00604402	0.000	0.27422	0	0	0	2
2503	RCC1 domain-containing protein 1	IPI00399158	0.000	0.27422	0	2	0	0
2504	Isoform 1 of F-box only protein 22	IPI00183208	0.000	0.27422	2	0	0	0
2505	69 kDa protein	IPI00018116	0.000	0.27422	0	2	0	0
2506	Isoform 2 of Pre-mRNA-splicing regulator WTAP	IPI00014150	0.000	0.27422	0	2	0	0
2507	Isoform 1 of Rhotekin	IPI00029834	0.000	0.27422	0	0	2	0
2508	Isoform Long of Tyrosine-protein kinase SYK	IPI00018597	0.000	0.27422	0	0	2	0
2509	Histone acetyltransferase MYST2	IPI00180764	0.000	0.27422	0	0	2	0
2510	Isoform 1 of Mediator of RNA polymerase II transcription subunit 16	IPI00037401	0.000	0.27422	0	0	0	2
2511	My002 protein	IPI00023584	0.000	0.27422	0	0	2	0
2512	Cystatin-D	IPI00002851	0.000	0.27422	2	0	0	0
2513	ADP ribosylation factor-like protein 7	IPI00010149	0.000	0.27422	2	2	0	0
2514	Major centromere autoantigen B	IPI00010388	0.000	0.27422	0	0	2	2
2515	Isoform 1 of Biorientation of chromosomes in cell division protein 1	IPI00063667	0.000	0.27422	2	0	0	0
2516	Isoform 2 of Ras association domain-containing protein 6	IPI00176707	0.000	0.27422	0	0	2	0
2517	Acyl-CoA synthetase family member 2, mitochondrial	IPI00304071	0.000	0.27422	2	0	0	0
2518	Isoform 3 of FK506-binding protein 15	IPI00646791	0.000	0.27422	0	2	0	0
2519	Putative alpha-1,2-glucosyltransferase ALG10-B	IPI00105827	0.000	0.27422	0	0	2	0
2520	Ankyrin repeat and zinc finger domain-containing protein 1	IPI00335437	0.000	0.27422	2	0	0	0
2521	arf-GAP with GTPase, ANK repeat and PH domain-containing protein 3 isoform a	IPI00163185	0.000	0.27422	0	0	0	2
2522	Oxysterol-binding protein-related protein 10	IPI00032971	0.000	0.27422	0	0	2	0
2523	Uncharacterized protein C20orf111	IPI00010197	0.000	0.27422	0	0	0	2
2524	Sortilin	IPI00217882	0.000	0.27422	2	0	0	0
2525	Isoform 1 of FK506-binding protein 15	IPI00853400	0.000	0.27422	0	2	0	0
2526	CDC45-related protein	IPI00025695	0.000	0.27422	0	0	2	0
2527	Isoform 1 of RelA-associated inhibitor	IPI00439948	0.000	0.27422	2	0	0	0
2528	AP2-associated protein kinase 1	IPI00479760	0.000	0.27422	2	0	0	0
2529	Isoform 1 of Leucine zipper protein 1	IPI00296830	0.000	0.27422	0	2	0	0
2530	Protein yippee-like 5	IPI00429538	0.000	0.27422	0	0	2	0
2531	Isoform 1 of Lambda-crystallin homolog	IPI00006443	0.000	0.27422	0	2	0	0
2532	TRAF-interacting protein with FHA domain-containing protein A	IPI00060419	0.000	0.27422	2	0	0	0
2533	Dual specificity protein phosphatase 14	IPI00013031	0.000	0.27422	0	2	0	0
2534	Isoform 2 of Zinc transporter ZIP11	IPI00410249	0.000	0.27422	2	0	0	0
2535	Isoform 1 of Transmembrane protein 163	IPI00152253	0.000	0.27422	0	0	2	0
2536	Isoform 1 of Discoidin, CUB and LCCL domain-containing protein 2	IPI00419836	0.000	0.27422	0	0	2	0
2537	Isoform 2 of Ubiquitin-conjugating enzyme E2 variant 3	IPI00217342	0.000	0.27422	0	0	2	0
2538	Polypeptide N-acetylgalactosaminyltransferase 6	IPI00026991	0.000	0.27422	0	2	0	0
2539	Isoform 1 of Splicing factor, arginine/serine-rich 12	IPI00103497	0.000	0.27422	0	0	2	0
2540	Isoform 1 of Dynamin-1-like protein	IPI00146935	0.000	0.27422	0	0	2	0
2541	Putative uncharacterized protein MTM1	IPI00639863	0.000	0.27422	0	0	0	2
2542	Isoform 1 of General transcription factor 3C polypeptide 2	IPI00878252	0.000	0.27422	0	0	0	2
2543	Isoform 1 of Cingulin-like protein 1	IPI00307829	0.000	0.27422	0	0	0	2
2544	serum amyloid A2 isoform a	IPI00006146	0.000	0.27422	2	0	0	0
2545	Isoform 2 of Rho GTPase-activating protein 12	IPI00217418	0.000	0.27422	0	2	0	0
2546	Isoform 2 of Bifunctional arginine demethylase and lysyl-hydroxylase JMJD6	IPI00375496	0.000	0.27422	0	0	2	0
2547	Microfibrillar-associated protein 1	IPI00022790	0.000	0.27422	0	0	0	2
2548	Stress-70 protein, mitochondrial	IPI00007765	-0.128	0.28326	42	31	35	37
2549	RuvB-like 2	IPI00009104	-0.134	0.28220	29	25	28	25
2550	Isoform 1 of RuvB-like 1	IPI00021187	-0.135	0.28209	28	25	29	23
2551	Vigilin	IPI00022228	-0.136	0.28196	21	29	27	22
2552	Isoform 1 of Isocitrate dehydrogenase [NAD] subunit alpha, mitochondrial	IPI00030702	-0.145	0.27776	17	16	16	16
2553	Ras GTPase-activating protein-binding protein 1	IPI00012442	-0.146	0.27741	17	15	16	15

No.	Description	Accession number	STN[1]	p-Value[1]	480_A[2]	480_B[2]	620_A[2]	620_B[2]
2554	Eukaryotic translation initiation factor 3 subunit I	IPI00012795	-0.146	0.27741	17	15	17	14
2555	Cathepsin D	IPI00011229	-0.147	0.27690	17	14	18	12
2556	Isoform 1 of Mitotic checkpoint protein BUB3	IPI00013468	-0.147	0.27659	15	15	15	14
2557	V-type proton ATPase catalytic subunit A	IPI00007682	-0.156	0.27043	12	9	9	11
2558	Isoform 2 of Suppressor of G2 allele of SKP1 homolog	IPI00791573	-0.157	0.26974	10	10	7	12
2559	Ezrin	IPI00843975	-0.157	0.26974	10	10	10	9
2560	Splicing factor 3A subunit 3	IPI00029764	-0.157	0.26974	10	10	10	9
2561	Small nuclear ribonucleoprotein Sm D2	IPI00017963	-0.158	0.26778	10	9	9	9
2562	26S proteasome non-ATPase regulatory subunit 5	IPI00002134	-0.158	0.26778	9	10	7	11
2563	regulator of chromosome condensation 1 isoform a	IPI00001661	-0.160	0.26672	10	8	10	7
2564	Thymidylate synthetase, isoform CRA_a	IPI00103732	-0.160	0.26672	8	10	8	9
2565	Isoform 2 of Splicing factor 1	IPI00294627	-0.160	0.26672	8	10	7	10
2566	Asparaginyl-tRNA synthetase, cytoplasmic	IPI00306960	-0.160	0.26672	9	9	8	9
2567	Isocitrate dehydrogenase [NADP] cytoplasmic	IPI00027223	-0.161	0.26569	9	8	8	8
2568	Isoform 1 of Low molecular weight phosphotyrosine protein phosphatase	IPI00219861	-0.161	0.26569	9	8	7	9
2569	Translocon-associated protein subunit delta precursor	IPI00019385	-0.163	0.26493	10	6	8	7
2570	Insulin-like growth factor 2 mRNA-binding protein 1	IPI00008557	-0.163	0.26493	9	7	9	6
2571	Prefoldin subunit 5	IPI00015361	-0.163	0.26493	9	7	7	8
2572	Cytochrome c oxidase subunit 5A, mitochondrial	IPI00025086	-0.163	0.26493	7	9	7	8
2573	Histidine triad nucleotide-binding protein 1	IPI00239077	-0.165	0.26256	7	8	5	9
2574	Probable dimethyladenosine transferase	IPI00004459	-0.165	0.26256	7	8	6	8
2575	Serine/threonine-protein phosphatase 2A catalytic subunit alpha isoform	IPI00008380	-0.165	0.26256	7	8	8	6
2576	Isoform 1 of Paraspeckle component 1	IPI00103525	-0.165	0.26256	7	8	6	8
2577	Isoform 1 of 1-phosphatidylinositol-4,5-bisphosphate phosphodiesterase gamma-1	IPI00016736	-0.166	0.26111	6	8	6	7
2578	DnaJ homolog subfamily B member 1	IPI00015947	-0.166	0.26111	7	7	7	6
2579	Catalase	IPI00465436	-0.166	0.26111	5	9	7	6
2580	Isoform 1 of Elongation factor Ts, mitochondrial	IPI00021016	-0.166	0.26111	5	9	7	6
2581	Isoform 1 of STE20-like serine/threonine-protein kinase	IPI00022827	-0.168	0.25822	6	7	7	5
2582	Oxysterol-binding protein	IPI00163644	-0.168	0.25822	6	7	6	6
2583	UDP-galactose-4-epimerase	IPI00030229	-0.168	0.25822	7	6	5	7
2584	Isoform 1 of Ataxin-2-like protein	IPI00456359	-0.171	0.25626	6	6	4	7
2585	60S ribosomal protein L32	IPI00395998	-0.171	0.25626	6	6	6	5
2586	Isoform SCPx of Non-specific lipid-transfer protein	IPI00026105	-0.171	0.25626	7	5	6	5
2587	Pyridoxine-5'-phosphate oxidase	IPI00018272	-0.171	0.25626	6	6	5	6
2588	Isoform 1 of Pre-mRNA-processing factor 40 homolog A	IPI00337385	-0.171	0.25626	7	5	7	4
2589	Nucleoporin NUP53	IPI00329650	-0.171	0.25626	6	6	6	5
2590	Isoform 1 of Spermatid perinuclear RNA-binding protein	IPI00169430	-0.173	0.25316	7	4	3	7
2591	FKBP1A protein	IPI00413778	-0.173	0.25316	5	6	6	4
2592	Isoform 1 of Ras-related protein Rab-6A	IPI00023526	-0.173	0.25316	6	5	5	5
2593	Exosome complex exonuclease RRP43	IPI00552920	-0.173	0.25316	5	6	5	5
2594	cDNA FLJ59712, highly similar to Golgi reassembly-stacking protein 2	IPI00743931	-0.173	0.25316	6	5	5	5
2595	Isoform 1 of Uncharacterized methyltransferase WBSCR22	IPI00013810	-0.173	0.25316	5	6	5	5
2596	SRA stem-loop-interacting RNA-binding protein, mitochondrial	IPI00009922	-0.173	0.25316	5	6	5	5
2597	Na(+)/H(+) exchange regulatory cofactor NHE-RF1	IPI00003527	-0.173	0.25316	6	5	5	5
2598	NEDD8	IPI00020008	-0.176	0.25110	5	5	3	6
2599	Isoform 2 of Isochorismatase domain-containing protein 2, mitochondrial	IPI00003031	-0.176	0.25110	7	3	6	3
2600	Isoform 3 of Mediator of RNA polymerase II transcription subunit 23	IPI00413272	-0.176	0.25110	5	5	5	4
2601	60S ribosomal protein L27a	IPI00456758	-0.176	0.25110	5	5	3	6
2602	NDUFB10 protein	IPI00074489	-0.176	0.25110	3	7	5	4
2603	Ras-related protein Rab-5A	IPI00023510	-0.176	0.25110	7	3	4	5
2604	Nucleoporin 54kDa variant (Fragment)	IPI00172580	-0.176	0.25110	3	7	4	5
2605	DNA-directed RNA polymerases I, II, and III subunit RPABC3	IPI00003309	-0.176	0.25110	5	5	4	5
2606	Isoform 1 of Huntingtin-interacting protein K	IPI00335001	-0.176	0.25110	4	6	4	5
2607	Glutathione S-transferase theta-1	IPI00741097	-0.176	0.25110	6	4	5	4
2608	Cytochrome b-c1 complex subunit 7	IPI00220416	-0.176	0.25110	4	6	3	6
2609	Isoform 2 of Ribosomal RNA processing protein 1 homolog B	IPI00032374	-0.179	0.24604	5	4	0	6
2610	60S ribosomal protein L17	IPI00413324	-0.179	0.24604	6	3	4	4
2611	COP9 signalosome complex subunit 7a	IPI00301419	-0.179	0.24604	4	5	4	4
2612	28S ribosomal protein S28, mitochondrial	IPI00022276	-0.179	0.24604	4	5	4	4
2613	RNA polymerase-associated protein CTR9 homolog	IPI00477468	-0.179	0.24604	5	4	5	3
2614	tRNA methyltransferase 112 homolog	IPI00009010	-0.179	0.24604	5	4	4	4
2615	U6 snRNA-associated Sm-like protein LSm2	IPI00032460	-0.179	0.24604	5	4	3	5
2616	U3 small nucleolar RNA-associated protein 18 homolog	IPI00000733	-0.179	0.24604	4	5	4	4
2617	DnaJ homolog subfamily C member 7	IPI00329629	-0.179	0.24604	5	4	3	5
2618	NudC domain-containing protein 3	IPI00238209	-0.179	0.24604	6	3	6	0
2619	Pre-mRNA branch site protein p14	IPI00032827	-0.179	0.24604	5	4	4	4
2620	28S ribosomal protein S7, mitochondrial	IPI00006440	-0.182	0.24295	3	5	2	5
2621	synembryn-A	IPI00100106	-0.182	0.24295	4	4	3	4
2622	Mitotic spindle assembly checkpoint protein MAD2A	IPI00012369	-0.182	0.24295	0	6	3	4
2623	Ubiquitin-like protein 4A	IPI00005658	-0.182	0.24295	4	4	5	2
2624	7-dehydrocholesterol reductase	IPI00294501	-0.182	0.24295	6	2	3	4
2625	Isoform 1 of Caseinolytic peptidase B protein homolog	IPI00006615	-0.182	0.24295	2	6	3	4
2626	Isoform Beta of DNA ligase 3	IPI00000156	-0.182	0.24295	3	5	5	0
2627	NIF3L1 isoform gamma	IPI00451429	-0.182	0.24295	4	4	4	3
2628	Transmembrane emp24 domain-containing protein 7	IPI00032825	-0.182	0.24295	5	3	5	0
2629	Zinc finger protein ZPR1	IPI00025244	-0.182	0.24295	4	4	4	3
2630	Peflin	IPI00018235	-0.182	0.24295	5	3	3	4
2631	Eukaryotic translation initiation factor 1	IPI00015077	-0.182	0.24295	4	4	3	4
2632	PDZ and LIM domain protein 5	IPI00007935	-0.186	0.23573	3	4	2	4
2633	Isoform 2 of Leucine-rich repeat flightless-interacting protein 1	IPI00006207	-0.186	0.23573	4	3	3	3
2634	Isoform 1 of Wings apart-like protein homolog	IPI00375330	-0.186	0.23573	4	3	3	3
2635	Signal peptidase complex subunit 3	IPI00300299	-0.186	0.23573	5	2	3	3
2636	39S ribosomal protein L50, mitochondrial	IPI00329036	-0.186	0.23573	3	4	2	4
2637	cDNA FLJ12779 fis, clone NT2RP2001748	IPI00902799	-0.186	0.23573	4	3	3	3
2638	Isoform GN-1L of Glycogenin-1	IPI00180386	-0.186	0.23573	5	2	3	3
2639	Isoform 1 of E3 ubiquitin-protein ligase Itchy homolog	IPI00061780	-0.186	0.23573	3	4	2	4
2640	Isoform 2 of Phosphatidylinositol-binding clathrin assembly protein	IPI00216184	-0.186	0.23573	4	3	4	0
2641	Retinal rod rhodopsin-sensitive cGMP 3',5'-cyclic phosphodiesterase subunit delta	IPI00015161	-0.186	0.23573	0	5	3	3
2642	N-alpha-acetyltransferase 38, NatC auxiliary subunit	IPI00219871	-0.186	0.23573	3	4	2	4
2643	REST corepressor 1	IPI00008531	-0.186	0.23573	4	3	3	3
2644	coatomer subunit epsilon isoform c	IPI00399319	-0.186	0.23573	4	3	3	3
2645	Nuclear RNA export factor 1	IPI00033153	-0.186	0.23573	3	4	2	4
2646	Reticulocalbin-2	IPI00029628	-0.186	0.23573	4	3	0	4
2647	Cell division protein kinase 7	IPI00000685	-0.186	0.23573	4	3	4	2
2648	Isoform 1 of Alpha-parvin	IPI00018963	-0.186	0.23573	4	3	3	3

No.	Description	Accession number	STN[1]	p-Value[1]	480_A[2]	480_B[2]	620_A[2]	620_B[2]
2649	Isoform Sap-mu-0 of Proactivator polypeptide	IPI00012503	-0.191	0.23125	4	0	3	2
2650	Probable ATP-dependent RNA helicase DDX56	IPI00302281	-0.191	0.23125	3	3	2	3
2651	Isoform 1 of Dr1-associated corepressor	IPI00003084	-0.191	0.23125	3	3	0	3
2652	NADH dehydrogenase [ubiquinone] 1 beta subcomplex subunit 6	IPI00219385	-0.191	0.23125	0	4	3	2
2653	DEAD (Asp-Glu-Ala-Asp) box polypeptide 39, isoform CRA_c	IPI00166874	-0.191	0.23125	2	4	3	0
2654	Prefoldin subunit 2	IPI00006052	-0.191	0.23125	3	3	3	2
2655	Isoform 2 of 39S ribosomal protein L39, mitochondrial	IPI00084571	-0.191	0.23125	0	4	2	3
2656	SF3A2 protein (Fragment)	IPI00017341	-0.191	0.23125	3	3	3	2
2657	Isoform 1 of Splicing factor, arginine/serine-rich 15	IPI00181702	-0.191	0.23125	3	3	2	3
2658	Isoform 1 of Putative ATP-dependent RNA helicase DHX57	IPI00168885	-0.191	0.23125	0	4	2	3
2659	Probable ATP-dependent RNA helicase DDX52	IPI00032423	-0.191	0.23125	2	4	0	3
2660	NADP-dependent malic enzyme, mitochondrial	IPI00003970	-0.191	0.23125	2	4	3	2
2661	Deoxycytidine kinase	IPI00020454	-0.191	0.23125	2	4	0	3
2662	F-box only protein 2	IPI00007087	-0.191	0.23125	3	3	2	3
2663	Fumarylacetoacetate hydrolase domain-containing protein 2B	IPI00301994	-0.191	0.23125	3	3	3	2
2664	Isoform 2 of Basic leucine zipper and W2 domain-containing protein 1	IPI00180128	-0.191	0.23125	0	4	0	3
2665	Isoform 5 of Sigma non-opioid intracellular receptor 1	IPI00167206	-0.191	0.23125	3	3	2	3
2666	Isoform 1 of Probable aminopeptidase NPEPL1	IPI00100292	-0.191	0.23125	4	2	3	0
2667	U6 snRNA-associated Sm-like protein LSm7	IPI00007163	-0.191	0.23125	3	3	0	3
2668	39S ribosomal protein L27, mitochondrial	IPI00009444	-0.191	0.23125	4	2	3	0
2669	Cleavage stimulation factor subunit 1	IPI00011528	-0.191	0.23125	3	3	3	2
2670	Isoform 2 of CDK5 regulatory subunit-associated protein 3	IPI00018780	-0.191	0.23125	3	3	3	2
2671	Isoform 2 of Protein PAT1 homolog 1	IPI00760958	-0.191	0.23125	0	4	0	3
2672	Isoform 1 of Serine protease HTRA2, mitochondrial	IPI00001663	-0.191	0.23125	3	3	3	0
2673	Isoform 1 of Kinesin-like protein KIF15	IPI00024975	-0.191	0.23125	0	4	0	3
2674	WD repeat-containing protein 43	IPI00937477	-0.191	0.23125	4	0	0	3
2675	Isoform 2 of Pre-mRNA-splicing factor ISY1 homolog	IPI00063673	-0.191	0.23125	3	3	3	2
2676	Isoform 1 of Uncharacterized protein CXorf38	IPI00152089	-0.191	0.23125	2	4	3	0
2677	Splicing factor, arginine/serine-rich 3	IPI00010204	-0.197	0.21423	0	3	0	0
2678	Similar to Elongation factor 1-alpha 1	IPI00180730	-0.197	0.21423	0	3	0	0
2679	Isoform 1 of Serine/threonine-protein phosphatase PGAM5, mitochondrial	IPI00788907	-0.197	0.21423	0	3	0	0
2680	Isoform 1 of DNA repair protein complementing XP-G cells	IPI00477535	-0.197	0.21423	0	3	2	2
2681	CD9 antigen	IPI00215997	-0.197	0.21423	0	3	0	0
2682	Isoform 2 of Triple functional domain protein	IPI00479523	-0.197	0.21423	0	3	0	0
2683	Isoform 1 of Ubiquitin carboxyl-terminal hydrolase 34	IPI00297593	-0.197	0.21423	3	0	0	0
2684	Isoform 2 of Cell division protein kinase 13	IPI00029162	-0.197	0.21423	3	2	2	2
2685	Uncharacterized protein C18orf19	IPI00290799	-0.197	0.21423	2	3	0	0
2686	UPF0364 protein C6orf211	IPI00002270	-0.197	0.21423	2	3	0	2
2687	Isoform 4 of Neurofibromin	IPI00220514	-0.197	0.21423	3	2	0	2
2688	Isoform 4 of Mitochondrial fission factor	IPI00024627	-0.197	0.21423	2	3	2	2
2689	Charged multivesicular body protein 7	IPI00395463	-0.197	0.21423	3	2	2	2
2690	Grancalcin	IPI00004524	-0.197	0.21423	3	2	0	2
2691	Isoform 1 of Rab3 GTPase-activating protein catalytic subunit	IPI00014235	-0.197	0.21423	2	3	0	2
2692	Isoform 2 of Membrane-associated guanylate kinase, WW and PDZ domain-containing protein 1	IPI00165946	-0.197	0.21423	3	0	0	0
2693	Phosphoinositide 3-kinase regulatory subunit 4	IPI00024006	-0.197	0.21423	3	2	2	0
2694	16 kDa protein	IPI00293975	-0.197	0.21423	3	2	0	0
2695	Isoform 2 of Syntaxin-5	IPI00386786	-0.197	0.21423	3	2	2	2
2696	Trafficking protein particle complex subunit 4	IPI00007691	-0.197	0.21423	0	3	0	0
2697	Phosphopantothenate--cysteine ligase	IPI00023987	-0.197	0.21423	2	3	2	2
2698	NEDD8-conjugating enzyme Ubc12	IPI00022597	-0.197	0.21423	3	2	0	2
2699	Condensin complex subunit 2	IPI00299507	-0.197	0.21423	2	3	2	0
2700	Isoform 2 of Choline-phosphate cytidylyltransferase B	IPI00001562	-0.197	0.21423	2	3	2	0
2701	NADH dehydrogenase [ubiquinone] 1 alpha subcomplex subunit 8	IPI00219034	-0.197	0.21423	0	3	0	2
2702	DNA polymerase delta subunit 2	IPI00025616	-0.197	0.21423	2	3	2	2
2703	Lysosomal alpha-glucosidase	IPI00293088	-0.197	0.21423	0	3	0	2
2704	cDNA FLJ40287 fis, clone TESTI2027909, highly similar to 5'-AMP-ACTIVATED PROTEIN KINASE	IPI00473047	-0.197	0.21423	3	0	2	0
2705	Isoform 1 of Serine/threonine-protein phosphatase 6 regulatory ankyrin repeat subunit A	IPI00477505	-0.197	0.21423	2	3	0	0
2706	Proteasome maturation protein	IPI00006377	-0.197	0.21423	3	2	2	2
2707	Isoform 1 of Acylglycerol kinase, mitochondrial	IPI00019353	-0.197	0.21423	2	3	2	2
2708	Isoform 1 of Death-inducer obliterator 1	IPI00249982	-0.197	0.21423	2	3	0	2
2709	Isoform 1 of Lipase maturation factor 2	IPI00385495	-0.197	0.21423	3	2	2	2
2710	Cell differentiation protein RCD1 homolog	IPI00023101	-0.197	0.21423	3	2	0	2
2711	Translocated promoter region	IPI00514531	-0.197	0.21423	3	2	0	2
2712	Isoform 1 of GRIP and coiled-coil domain-containing protein 2	IPI00005631	-0.197	0.21423	0	3	2	2
2713	Cell division protein kinase 4	IPI00007811	-0.197	0.21423	0	3	0	2
2714	Choline-phosphate cytidylyltransferase A	IPI00329338	-0.197	0.21423	3	0	0	0
2715	Ras-related protein Rab-8B	IPI00024282	-0.197	0.21423	3	0	0	0
2716	Alpha-ketoglutarate dehydrogenase complex dihydrolipoyl succinyltransferase	IPI00033034	-0.197	0.21423	3	0	0	2
2717	Isoform 1 of ATP-binding cassette sub-family F member 3	IPI00465160	-0.197	0.21423	2	3	0	2
2718	Neuronal protein	IPI00472058	-0.197	0.21423	3	2	2	2
2719	Isoform 2 of Hydroxysteroid dehydrogenase-like protein 2	IPI00031107	-0.197	0.21423	3	2	0	0
2720	Protein tyrosine phosphatase type IVA 1	IPI00020164	-0.197	0.21423	3	2	2	0
2721	Isoform 2 of NudC domain-containing protein 1	IPI00306398	-0.197	0.21423	2	3	0	2
2722	FLJ00369 protein (Fragment)	IPI00166711	-0.197	0.21423	3	2	0	0
2723	Interferon regulatory factor 3	IPI00291901	-0.197	0.21423	0	3	0	0
2724	Isoform 1 of STIP1 homology and U box-containing protein 1	IPI00025156	-0.197	0.21423	2	3	0	2
2725	Probable ATP-dependent RNA helicase DDX20	IPI00005904	-0.197	0.21423	0	3	2	0
2726	Probable ergosterol biosynthetic protein 28	IPI00007730	-0.197	0.21423	3	2	2	2
2727	Isoform 1 of Glucosamine-6-phosphate isomerase 2	IPI00550894	-0.197	0.21423	3	2	2	0
2728	Claudin-3	IPI00007364	-0.197	0.21423	0	3	0	0
2729	Isoform 2 of Zinc finger CCCH domain-containing protein 18	IPI00293312	-0.197	0.21423	2	3	0	0
2730	Isoform 2 of E1A-binding protein p400	IPI00064931	-0.197	0.21423	3	0	0	0
2731	Translation initiation factor eIF-2B subunit beta	IPI00028083	-0.197	0.21423	2	3	0	2
2732	Isoform 1 of Protein IWS1 homolog	IPI00296432	-0.197	0.21423	3	0	0	0
2733	Nucleoporin Nup37	IPI00171665	-0.197	0.21423	3	2	2	2
2734	Haloacid dehalogenase-like hydrolase domain-containing protein 3	IPI00009931	-0.197	0.21423	3	2	2	0
2735	Isoform 1 of Nurim	IPI00217557	-0.197	0.21423	3	0	0	0
2736	Isoform 5 of Serine/threonine-protein kinase MRCK alpha	IPI00550263	-0.197	0.21423	0	3	0	0
2737	12 kDa protein	IPI00176698	-0.197	0.21423	0	3	0	2
2738	Casein kinase I isoform alpha-like	IPI00167096	-0.197	0.21423	2	3	0	0
2739	Isoform 1 of Carnitine O-acetyltransferase	IPI00016457	-0.197	0.21423	3	0	0	0
2740	Desmoglein-1	IPI00025753	-0.197	0.21423	2	3	0	0
2741	Aminopeptidase B	IPI00642211	-0.197	0.21423	0	3	0	2
2742	cDNA FLJ56152, highly similar to Rho guanine nucleotide exchange factor 7	IPI00449906	-0.197	0.21423	2	3	0	2
2743	Isoform 2 of Nitrilase homolog 1	IPI00023779	-0.197	0.21423	2	3	0	0

No.	Description	Accession number	STN[1]	p-Value[1]	480_A[2]	480_B[2]	620_A[2]	620_B[2]
2744	Isoform 1 of Mannose-1-phosphate guanyltransferase alpha	IPI00101782	-0.197	0.21423	0	3	2	2
2745	Isoform SRP55-1 of Splicing factor, arginine/serine-rich 6	IPI00012345	-0.197	0.21423	3	2	0	0
2746	cDNA FLJ60094, highly similar to F-actin capping protein subunit beta	IPI00218782	-0.197	0.21423	3	2	2	2
2747	Isoform Long of Metastasis-associated protein MTA1	IPI00012773	-0.197	0.21423	3	0	0	2
2748	Probable leucyl-tRNA synthetase, mitochondrial	IPI00014213	-0.197	0.21423	3	2	0	0
2749	cDNA FLJ55508, highly similar to Sad1/unc-84-like protein 2	IPI00295940	-0.197	0.21423	3	0	0	0
2750	Ubiquitin-associated protein 2	IPI00171127	-0.197	0.21423	0	3	0	0
2751	Isoform 1 of Motile sperm domain-containing protein 2	IPI00169283	-0.197	0.21423	0	3	2	2
2752	Isoform 1 of Transcription elongation factor A protein 1	IPI00333215	-0.197	0.21423	3	0	0	0
2753	Isoform 1 of NADH-cytochrome b5 reductase 2	IPI00008234	-0.197	0.21423	3	2	0	0
2754	Insulin receptor substrate 2 insertion mutant (Fragment)	IPI00464978	-0.197	0.21423	0	3	0	0
2755	Protein ariadne-2 homolog	IPI00007304	-0.197	0.21423	3	0	2	0
2756	Protein FAM98A	IPI00174442	-0.197	0.21423	3	0	0	2
2757	Uncharacterized protein C20orf4	IPI00166013	-0.197	0.21423	0	3	0	2
2758	Exportin-4	IPI00028357	-0.197	0.21423	3	0	0	0
2759	Transcription factor MafG	IPI00007311	-0.197	0.21423	3	0	2	0
2760	Isoform 2 of Protein SET	IPI00301311	-0.197	0.21423	3	2	2	2
2761	28S ribosomal protein S15, mitochondrial	IPI00550037	-0.197	0.21423	3	0	0	2
2762	N(G),N(G)-dimethylarginine dimethylaminohydrolase 1	IPI00220342	-0.197	0.21423	2	3	0	0
2763	Isoform 1 of BAG family molecular chaperone regulator 5	IPI00007731	-0.197	0.21423	0	3	0	0
2764	Biorientation of chromosomes in cell division protein 1-like	IPI00797574	-0.197	0.21423	0	3	0	0
2765	Isoform 2 of Inositol hexakisphosphate and diphosphoinositol-pentakisphosphate kinase 2	IPI00178375	-0.197	0.21423	3	2	0	2
2766	Isoform 2 of ELKS/Rab6-interacting/CAST family member 1	IPI00171230	-0.197	0.21423	0	3	0	0
2767	Isocitrate dehydrogenase [NAD] subunit gamma, mitochondrial	IPI00220150	-0.197	0.21423	3	0	2	2
2768	Cellular retinoic acid-binding protein 2	IPI00216088	-0.197	0.21423	3	2	0	0
2769	Uncharacterized protein C2orf79	IPI00430803	-0.197	0.21423	3	2	0	2
2770	Lipoma-preferred partner	IPI00023704	-0.197	0.21423	0	3	0	0
2771	Prostaglandin reductase 1	IPI00164901	-0.197	0.21423	0	3	0	0
2772	Receptor-type tyrosine-protein phosphatase eta precursor	IPI00290328	-0.197	0.21423	3	2	0	0
2773	DNA repair protein XRCC1	IPI00002564	-0.197	0.21423	0	3	0	0
2774	Ribosome biogenesis regulatory protein homolog	IPI00014253	-0.197	0.21423	3	2	2	2
2775	Protein Red	IPI00011875	-0.197	0.21423	3	0	2	0
2776	Isoform 1 of Mediator of RNA polymerase II transcription subunit 27	IPI00302652	-0.197	0.21423	3	2	2	0
2777	Glycogen synthase kinase-3 alpha	IPI00292228	-0.197	0.21423	0	3	0	0
2778	Isoform 1 of Protein SDA1 homolog	IPI00018240	-0.197	0.21423	3	2	0	0
2779	Glycogen phosphorylase, muscle form	IPI00218130	-0.197	0.21423	0	3	0	0
2780	Isoform 1 of Rho GTPase-activating protein 29	IPI00152011	-0.197	0.21423	3	2	0	0
2781	Isoform 1 of Adaptin ear-binding coat-associated protein 1	IPI00170916	-0.197	0.21423	2	3	0	0
2782	Isoform 1 of Serine/threonine-protein phosphatase 4 regulatory subunit 3A	IPI00217013	-0.197	0.21423	0	3	0	0
2783	Isoform 12 of CD44 antigen	IPI00297160	-0.197	0.21423	0	3	0	0
2784	Protein MIS12 homolog	IPI00031527	-0.197	0.21423	0	3	2	2
2785	Isoform 2 of Matrix-remodeling-associated protein 7	IPI00397645	-0.197	0.21423	2	3	2	0
2786	Cofilin-2	IPI00413344	-0.197	0.21423	2	3	2	2
2787	Transcriptional activator protein Pur-alpha	IPI00023591	-0.197	0.21423	3	2	2	0
2788	Transmembrane protein C3orf1	IPI00299387	-0.197	0.21423	2	3	0	0
2789	Isoform 1 of SH3 domain-containing kinase-binding protein 1	IPI00294962	-0.197	0.21423	0	3	0	0
2790	Isoform 2 of WASH complex subunit 7	IPI00164930	-0.197	0.21423	0	3	0	0
2791	Isoform B of Inositol polyphosphate 5-phosphatase OCRL-1	IPI00480049	-0.197	0.21423	2	3	0	0
2792	Basal cell adhesion molecule	IPI00002406	-0.197	0.21423	3	2	0	0
2793	StAR-related lipid transfer protein 4	IPI00061112	-0.197	0.21423	2	3	0	0
2794	Putative uncharacterized protein ISYNA1	IPI00385156	-0.197	0.21423	2	3	0	0
2795	Bcl-2-like protein 15	IPI00514645	-0.197	0.21423	3	2	0	0
2796	Male-enhanced antigen 1	IPI00157176	-0.197	0.21423	3	0	2	0
2797	Isoform 2 of Ankycorbin	IPI00292953	-0.197	0.21423	0	3	0	0
2798	COBW domain-containing protein 2	IPI00216734	-0.197	0.21423	3	0	0	0
2799	General transcription factor IIF subunit 1	IPI00017450	-0.197	0.21423	3	2	0	0
2800	Sorting nexin-8	IPI00001885	-0.197	0.21423	3	2	0	0
2801	Isoform 1 of Coiled-coil and C2 domain-containing protein 1A	IPI00302647	-0.197	0.21423	3	0	0	0
2802	cDNA FLJ61658, highly similar to Transmembrane 9 superfamily protein member 1	IPI00101374	-0.197	0.21423	3	2	2	2
2803	Isoform 1 of SLIT-ROBO Rho GTPase-activating protein 1	IPI00376259	-0.197	0.21423	0	3	0	0
2804	Myosin-Ia	IPI00294386	-0.197	0.21423	0	3	0	0
2805	Isoform 1 of Protein SMG9	IPI00012669	-0.197	0.21423	3	2	0	0
2806	Isoform AGX2 of UDP-N-acetylhexosamine pyrophosphorylase	IPI00000684	-0.197	0.21423	3	0	0	0
2807	Isoform 1 of Protein fat-free homolog	IPI00001710	-0.197	0.21423	3	0	0	2
2808	Isoform 1 of Arf-GAP with SH3 domain, ANK repeat and PH domain-containing protein 2	IPI00022058	-0.197	0.21423	3	2	0	0
2809	Ribosyldihydronicotinamide dehydrogenase [quinone]	IPI00219129	-0.197	0.21423	2	3	0	0
2810	Isoform 1 of Vesicle transport protein USE1	IPI00020515	-0.197	0.21423	0	3	0	0
2811	Isoform 3 of Motile sperm domain-containing protein 1	IPI00164251	-0.197	0.21423	3	2	0	0
2812	Laminin subunit gamma-1	IPI00298281	-0.197	0.21423	0	3	0	0
2813	Putative uncharacterized protein ZYX	IPI00658086	-0.197	0.21423	0	3	0	0
2814	Serine dehydratase-like	IPI00062419	-0.197	0.21423	3	0	0	0
2815	RAP1, GTP-GDP dissociation stimulator 1 isoform 6	IPI00424869	-0.197	0.21423	0	3	0	0
2816	Thrombospondin-1	IPI00296099	-0.197	0.21423	3	0	0	0
2817	Isoform 1 of Ras-related GTP-binding protein B	IPI00010317	-0.197	0.21423	0	3	0	0
2818	Isoform 1 of UAP56-interacting factor	IPI00289907	-0.197	0.21423	2	3	0	0
2819	Isoform 1 of Rho guanine nucleotide exchange factor 18	IPI00179437	-0.197	0.21423	3	0	0	0
2820	Alpha-1,3-mannosyl-glycoprotein 2-beta-N-acetylglucosaminyltransferase	IPI00000138	-0.197	0.21423	3	0	0	0
2821	Interferon-related developmental regulator 1	IPI00011735	-0.197	0.21423	0	3	0	0
2822	WD repeat-containing protein 46	IPI00023126	-0.197	0.21423	3	0	0	0
2823	Switch-associated protein 70	IPI00307200	-0.197	0.21423	2	3	0	0
2824	Isoform 1 of THO complex subunit 1	IPI00305374	-0.197	0.21423	0	3	0	0
2825	Fatty acid-binding protein, heart	IPI00219684	-0.197	0.21423	2	3	0	0
2826	15-hydroxyprostaglandin dehydrogenase isoform 2	IPI00290420	-0.197	0.21423	0	3	0	0
2828	Ras-related protein Rab-7a	IPI00016342	-0.261	0.19999	36	30	31	33
2829	Structural maintenance of chromosomes protein 1A	IPI00291939	-0.264	0.19968	30	31	28	31
2830	Isoform 3 of Core histone macro-H2A.1	IPI00059366	-0.269	0.19882	25	29	31	21
2831	Glycogen phosphorylase, liver form	IPI00783313	-0.277	0.19720	24	21	21	22
2832	Isoform 4 of Serine/threonine-protein phosphatase 6 regulatory subunit 3	IPI00019540	-0.293	0.19290	16	16	15	15
2833	Isoform 1 of Large proline-rich protein BAT3	IPI00465128	-0.293	0.19290	17	15	14	16
2834	Tetratricopeptide repeat protein 37	IPI00005634	-0.297	0.19056	14	15	11	16
2835	Isoform 1 of Protein-L-isoaspartate(D-aspartate) O-methyltransferase	IPI00411680	-0.297	0.19056	13	16	15	12
2836	Sideroflexin-1	IPI00009368	-0.299	0.19056	13	15	15	11
2837	Putative uncharacterized protein NOP2	IPI00294891	-0.299	0.19056	14	14	13	13
2838	Lamin-B2	IPI00009771	-0.301	0.18874	16	11	9	16
2839	Isoform Alpha of Signal transducer and activator of transcription 1-alpha/beta	IPI00030781	-0.313	0.18213	13	8	12	7

No.	Description	Accession number	STN[1]	p-Value[1]	480_A[2]	480_B[2]	620_A[2]	620_B[2]
2840	Isoform 3 of Serine/threonine-protein phosphatase 2A activator	IPI00217296	-0.316	0.18213	11	9	11	7
2841	Isoleucyl-tRNA synthetase, mitochondrial	IPI00017283	-0.318	0.17856	8	11	8	9
2842	coatomer subunit epsilon isoform b	IPI00399318	-0.318	0.17856	10	9	8	9
2843	Isoform 2 of AP-2 complex subunit alpha-2	IPI00016621	-0.321	0.17856	7	11	9	7
2844	Isoform 1 of Peroxisomal acyl-coenzyme A oxidase 1	IPI00296907	-0.324	0.17494	8	9	7	8
2845	Isoform 1 of 3,2-trans-enoyl-CoA isomerase, mitochondrial	IPI00300567	-0.324	0.17494	11	6	6	9
2846	Isoform HMG-Y of High mobility group protein HMG-I/HMG-Y	IPI00177716	-0.327	0.17494	7	9	6	8
2847	28S ribosomal protein S29, mitochondrial	IPI00018120	-0.327	0.17494	8	8	5	9
2848	Putative uncharacterized protein ZFR	IPI00748303	-0.327	0.17494	8	8	7	7
2849	protein ELYS	IPI00170594	-0.331	0.17054	7	8	7	6
2850	Protein of unknown function DUF410 family protein	IPI00419575	-0.331	0.17054	8	7	7	6
2851	33 kDa protein	IPI00413108	-0.331	0.17054	7	8	6	7
2852	Chromobox protein homolog 5	IPI00024662	-0.335	0.17054	7	7	6	6
2853	Isoform 1 of Putative deoxyribonuclease TATDN1	IPI00012463	-0.335	0.17054	7	7	6	6
2854	Ras-related protein Rab-6B	IPI00016891	-0.339	0.16463	8	5	5	6
2855	Succinyl-CoA ligase [GDP-forming] subunit alpha, mitochondrial	IPI00872762	-0.339	0.16463	6	7	5	6
2856	Isoform 3 of Drebrin-like protein	IPI00101968	-0.339	0.16463	8	5	4	7
2857	Coatomer subunit gamma-2	IPI00002557	-0.344	0.16449	5	7	6	4
2858	Isoform 1 of Dephospho-CoA kinase domain-containing protein	IPI00291417	-0.344	0.16449	7	5	6	4
2859	Thioredoxin domain-containing protein 5	IPI00171438	-0.349	0.15713	6	5	5	4
2860	Transcription factor BTF3 homolog 4	IPI00412792	-0.349	0.15713	6	5	6	3
2861	Fascin	IPI00163187	-0.349	0.15713	4	7	5	4
2862	Autophagy-related protein 101	IPI00305296	-0.349	0.15713	6	5	4	5
2863	Histidine triad nucleotide-binding protein 2, mitochondrial	IPI00000335	-0.349	0.15713	6	5	4	5
2864	ATP-dependent RNA helicase DHX29	IPI00217413	-0.355	0.15713	4	6	6	2
2865	Isoform 1 of Origin recognition complex subunit 3	IPI00294402	-0.355	0.15713	5	5	3	5
2866	Isoform 3 of Sperm-specific antigen 2	IPI00386170	-0.355	0.15713	5	5	3	5
2867	Heterogeneous nuclear ribonucleoprotein H2	IPI00026230	-0.355	0.15713	6	4	5	3
2868	Synaptosomal-associated protein 29	IPI00032831	-0.355	0.15713	4	6	4	4
2869	Methionine aminopeptidase 2	IPI00033036	-0.355	0.15713	4	6	4	4
2870	Phosphatidylinositol-5-phosphate 4-kinase type-2 gamma	IPI00152303	-0.355	0.15713	6	4	5	3
2871	Isoform 1 of Protein phosphatase 1 regulatory subunit 7	IPI00033600	-0.355	0.15713	6	4	3	5
2872	ADP-ribosylation factor 1	IPI00215914	-0.360	0.14798	58	55	60	50
2873	Isoform 1 of tRNA (adenine-N(1)-)-methyltransferase non-catalytic subunit TRM6	IPI00099311	-0.361	0.14794	4	5	4	3
2874	Leucine-rich repeat-containing protein 47	IPI00170935	-0.361	0.14794	4	5	3	4
2875	Toll-interacting protein	IPI00100154	-0.361	0.14794	5	4	4	3
2876	39S ribosomal protein L41, mitochondrial	IPI00217553	-0.361	0.14794	5	4	4	3
2877	Tetratricopeptide repeat protein 35	IPI00014149	-0.361	0.14794	4	5	4	3
2878	Isoform 1 of Serine/threonine-protein kinase N1	IPI00002803	-0.361	0.14794	5	4	0	5
2879	Mitochondrial import inner membrane translocase subunit Tim16	IPI00218463	-0.361	0.14794	5	4	4	3
2880	Ribonuclease inhibitor	IPI00550069	-0.361	0.14794	5	4	3	4
2881	Superoxide dismutase [Cu-Zn]	IPI00218733	-0.369	0.14781	4	4	4	0
2882	Isoform 3 of HEAT repeat-containing protein 5B	IPI00333696	-0.369	0.14781	3	5	4	0
2883	Isoform 1 of Dual specificity mitogen-activated protein kinase kinase 3	IPI00218857	-0.369	0.14781	5	3	3	3
2884	U3 small nucleolar RNA-associated protein 15 homolog	IPI00152708	-0.369	0.14781	5	3	0	4
2885	cDNA FLJ54775, highly similar to Syntaxin-binding protein 2	IPI00019971	-0.369	0.14781	3	5	3	3
2886	Charged multivesicular body protein 4b	IPI00025974	-0.369	0.14781	5	3	0	4
2887	UBX domain-containing protein 7	IPI00742124	-0.369	0.14781	4	4	2	4
2888	Isoform 1 of Serine hydroxymethyltransferase, cytosolic	IPI00002519	-0.369	0.14781	4	4	4	0
2889	Propionyl-CoA carboxylase beta chain, mitochondrial	IPI00007247	-0.369	0.14781	3	5	2	4
2890	Immature colon carcinoma transcript 1 protein	IPI00029114	-0.369	0.14781	4	4	3	3
2891	Syntaxin-12	IPI00329332	-0.369	0.14781	3	5	4	2
2892	NADP-dependent malic enzyme	IPI00008215	-0.369	0.14781	5	3	3	3
2893	Isoform 1 of RNA polymerase II-associated protein 3	IPI00002408	-0.369	0.14781	4	4	2	4
2894	Serine palmitoyltransferase 2	IPI00005751	-0.369	0.14781	4	4	3	3
2895	Ribonuclease UK114	IPI00005038	-0.369	0.14781	4	4	2	4
2896	Isoform 1 of Protein-tyrosine phosphatase mitochondrial 1	IPI00174190	-0.378	0.13418	0	5	0	3
2897	Isoform 1 of Protein transport protein Sec24A	IPI00873472	-0.378	0.13418	3	4	2	3
2898	cDNA FLJ56394, highly similar to N-acetylglucosamine kinase	IPI00296526	-0.378	0.13418	4	3	3	2
2899	WD repeat-containing protein 33	IPI00106567	-0.378	0.13418	0	5	3	0
2900	Mitochondrial import receptor subunit TOM70	IPI00015602	-0.378	0.13418	4	3	3	2
2901	Epoxide hydrolase 1	IPI00009896	-0.378	0.13418	2	5	2	3
2902	Multiple coagulation factor deficiency protein 2	IPI00328680	-0.378	0.13418	4	3	2	3
2903	cDNA FLJ54030, highly similar to Polymerase delta-interacting protein 3	IPI00440688	-0.378	0.13418	4	3	2	3
2904	Origin recognition complex subunit 4	IPI00015164	-0.378	0.13418	4	3	3	2
2905	Glucosamine--fructose-6-phosphate aminotransferase [isomerizing] 2	IPI00216159	-0.378	0.13418	4	3	0	3
2906	Isoform 2 of Peptidyl-prolyl cis-trans isomerase-like 3	IPI00032473	-0.378	0.13418	4	3	3	2
2907	Flotillin-2	IPI00789008	-0.378	0.13418	2	5	2	3
2908	Prefoldin subunit 6	IPI00005657	-0.378	0.13418	5	0	3	0
2909	Isoform 1 of PDZ and LIM domain protein 7	IPI00023122	-0.378	0.13418	3	4	2	3
2910	Probable DNA dC->dU-editing enzyme APOBEC-3C	IPI00555878	-0.378	0.13418	3	4	2	3
2911	Isoform Long of FAS-associated factor 1	IPI00070643	-0.378	0.13418	4	3	2	3
2912	Cytovillin 2 (Fragment)	IPI00384282	-0.378	0.13418	3	4	3	2
2913	Transcription initiation factor IIE subunit beta	IPI00019981	-0.378	0.13418	4	3	3	0
2914	Sorting nexin-2	IPI00299095	-0.378	0.13418	3	4	2	3
2915	Protein FAM49A	IPI00006574	-0.378	0.13418	3	4	3	2
2916	Isoform 3 of Parkinson disease 7 domain-containing protein 1	IPI00167976	-0.378	0.13418	3	4	0	3
2917	Protein AATF	IPI00302238	-0.378	0.13418	4	3	0	3
2918	H/ACA ribonucleoprotein complex subunit 4	IPI00221394	-0.389	0.13387	0	4	0	2
2919	Tubulin beta-4 chain	IPI00023598	-0.389	0.13387	0	4	0	0
2920	Nucleolar protein 11	IPI00303813	-0.389	0.13387	2	4	0	2
2921	CDNA FLJ20030 fis, clone ADSU02156	IPI00014402	-0.389	0.13387	2	4	2	0
2922	Myeloid leukemia factor 2	IPI00023095	-0.389	0.13387	2	4	0	0
2923	Protein of unknown function UPF0118 family protein	IPI00012429	-0.389	0.13387	2	4	2	2
2924	Isoform Long of Active breakpoint cluster region-related protein	IPI00030389	-0.389	0.13387	2	4	2	2
2925	Stromal cell-derived factor 2	IPI00293167	-0.389	0.13387	4	2	2	2
2926	Probable ATP-dependent RNA helicase DHX37	IPI00217630	-0.389	0.13387	3	3	2	2
2927	Phenol sulfotransferase 1A5*1A possible alternative splicing form	IPI00030730	-0.389	0.13387	3	3	0	0
2928	Plexin B2	IPI00852623	-0.389	0.13387	0	4	0	0
2929	Ras-related protein Rab-35	IPI00300096	-0.389	0.13387	3	3	0	2
2930	Derlin-1	IPI00013271	-0.389	0.13387	4	0	0	0
2931	Sulfotransferase 1A1	IPI00300026	-0.389	0.13387	4	0	0	0
2932	Isoform Delta-1 of Serine/threonine-protein phosphatase 2A 56 kDa regulatory subunit delta isoform	IPI00000030	-0.389	0.13387	2	4	0	0
2933	Protein CTF18 homolog	IPI00178203	-0.389	0.13387	2	4	2	2

No.	Description	Accession number	STN[1]	p-Value[1]	480_A[2]	480_B[2]	620_A[2]	620_B[2]
2934	Isoform UNPEL of Ubiquitin carboxyl-terminal hydrolase 4	IPI00011836	-0.389	0.13387	3	3	0	2
2935	Envoplakin	IPI00023711	-0.389	0.13387	3	3	0	0
2936	Isoform Mitochondrial of Phospholipid hydroperoxide glutathione peroxidase, mitochondrial	IPI00304814	-0.389	0.13387	4	0	0	0
2937	Isoform 1 of N-alpha-acetyltransferase 40, NatD catalytic subunit	IPI00328847	-0.389	0.13387	2	4	2	2
2938	Isoform 1 of Ubiquitin carboxyl-terminal hydrolase 15	IPI00000728	-0.389	0.13387	2	4	2	0
2939	Proto-oncogene tyrosine-protein kinase Yes	IPI00013981	-0.389	0.13387	0	4	0	2
2940	Utrophin	IPI00009329	-0.389	0.13387	4	0	0	0
2941	Isoform 6 of Terminal uridylyltransferase 7	IPI00336000	-0.389	0.13387	0	4	0	2
2942	Peptidyl-prolyl cis-trans isomerase FKBP2	IPI00002535	-0.389	0.13387	2	4	0	0
2943	Isoform 2 of Ras-related protein Rab-4B	IPI00187143	-0.389	0.13387	4	2	0	2
2944	Isoform 1 of Dedicator of cytokinesis protein 9	IPI00216408	-0.389	0.13387	3	3	0	0
2945	Isoform 1 of Glycogen synthase kinase-3 beta	IPI00028570	-0.389	0.13387	3	3	2	0
2946	Sulfiredoxin-1	IPI00168554	-0.389	0.13387	4	2	0	2
2947	Isoform 5 of Serine/threonine-protein phosphatase 4 regulatory subunit 3A	IPI00017290	-0.389	0.13387	4	0	0	0
2948	Isoform GTBP-N of DNA mismatch repair protein Msh6	IPI00384456	-0.389	0.13387	3	3	2	2
2949	cDNA FLJ56280, highly similar to Endoplasmic reticulum-Golgi intermediate compartment protein 1	IPI00003635	-0.389	0.13387	4	0	0	2
2950	Isoform 1 of UPF0598 protein C8orf82	IPI00166638	-0.389	0.13387	4	2	0	2
2951	Isoform 1 of Autophagy-related protein 9A	IPI00383396	-0.389	0.13387	0	4	0	0
2952	Isoform 1 of SUN domain-containing protein 1	IPI00783943	-0.389	0.13387	2	4	0	0
2953	Serine/threonine-protein phosphatase 2A 65 kDa regulatory subunit A alpha isoform	IPI00554737	-0.389	0.13387	3	3	2	2
2954	Isoform 1 of Hydroxyacylglutathione hydrolase, mitochondrial	IPI00003933	-0.389	0.13387	2	4	0	0
2955	protein Shroom3	IPI00152881	-0.389	0.13387	3	3	0	0
2956	cDNA FLJ56047, highly similar to A kinase anchor protein 1, mitochondrial	IPI00022585	-0.389	0.13387	3	3	0	0
2957	Isoform 1 of Casein kinase I isoform alpha	IPI00183400	-0.389	0.13387	3	3	0	0
2958	Isoform 1 of Cell surface glycoprotein MUC18	IPI00016334	-0.389	0.13387	4	0	0	0
2959	WD repeat-containing protein 62	IPI00470483	-0.389	0.13387	0	4	0	0
2960	Isoform 1 of Glucocorticoid receptor DNA-binding factor 1	IPI00334715	-0.389	0.13387	0	4	0	0
2961	cDNA FLJ43795 fis, clone TESTI4000079	IPI00171456	-0.389	0.13387	3	3	2	0
2962	Ephrin-B1	IPI00024307	-0.389	0.13387	2	4	0	0
2963	Serine/threonine-protein kinase 10	IPI00304742	-0.389	0.13387	0	4	2	0
2964	Isoform 2 of Protein transport protein Sec24A	IPI00178798	-0.389	0.13387	3	3	0	0
2965	Isoform 1 of Protein VPRBP	IPI00329528	-0.389	0.13387	3	3	0	0
2966	Centromere protein F	IPI00855998	-0.389	0.13387	4	2	0	0
2967	Isoform 1 of SET domain-containing protein 3	IPI00165026	-0.389	0.13387	3	3	0	0
2968	Isoform 1 of Surfeit locus protein 1	IPI00018034	-0.389	0.13387	4	2	2	2
2969	Isoform 1 of COMM domain-containing protein 7	IPI00743772	-0.389	0.13387	4	2	0	0
2970	Isoform RMO1abc of Ras-associated and pleckstrin homology domains-containing protein 1	IPI00402234	-0.389	0.13387	3	3	0	0
2971	Isoform 1 of Insulin-like growth factor 2 mRNA-binding protein 3	IPI00658000	-0.389	0.13387	3	3	2	2
2972	cDNA FLJ53160, highly similar to Zyxin	IPI00871311	-0.389	0.13387	3	3	2	2
2973	Ufm1-specific protease 2	IPI00305303	-0.389	0.13387	4	0	0	0
2974	Similar to Zinc finger MYM-type protein 5	IPI00294603	-0.389	0.13387	3	3	0	0
2975	Isoform 1 of Tropomyosin alpha-1 chain	IPI00014581	-0.389	0.13387	3	3	0	0
2976	cDNA FLJ56343, highly similar to Torsin A	IPI00413293	-0.389	0.13387	3	3	2	0
2977	cDNA FLJ54836	IPI00063160	-0.389	0.13387	4	2	0	0
2978	Fumarylacetoacetate hydrolase domain-containing protein 2A	IPI00329742	-0.389	0.13387	3	3	0	0
2979	Ganglioside GM2 activator	IPI00018236	-0.389	0.13387	0	4	0	0
2980	Cell division protein kinase 3	IPI00023503	-0.389	0.13387	3	3	0	2
2981	Glutaredoxin-related protein 5, mitochondrial	IPI00333763	-0.389	0.13387	3	3	0	0
2982	Isoform 1 of Epidermal growth factor receptor substrate 15	IPI00292134	-0.389	0.13387	0	4	0	0
2983	Isoform 1 of Actin-binding LIM protein 1	IPI00329495	-0.389	0.13387	0	4	0	0
2984	Glutaredoxin-1	IPI00219025	-0.389	0.13387	3	3	0	0
2985	Isoform 1 of Proteasomal ATPase-associated factor 1	IPI00743862	-0.389	0.13387	3	3	0	0
2986	Dual specificity protein kinase TTK	IPI00151170	-0.389	0.13387	0	4	0	0
2987	Microtubule-actin crosslinking factor 1	IPI00256859	-0.389	0.13387	0	4	0	0
2988	Isoform 2 of TATA element modulatory factor	IPI00010586	-0.389	0.13387	0	4	0	0
2989	myosin-14 isoform 1	IPI00607818	-0.389	0.13387	2	4	0	0
2990	DNA polymerase epsilon subunit 4	IPI00008436	-0.389	0.13387	4	0	0	0
2991	PRKC apoptosis WT1 regulator protein	IPI00001871	-0.389	0.13387	4	0	0	0
2992	Rab GDP dissociation inhibitor alpha	IPI00010154	-0.395	0.11251	32	31	28	32
2993	Calmodulin	IPI00075248	-0.412	0.11100	23	25	21	24
2994	Dolichyl-diphosphooligosaccharide--protein glycosyltransferase 48 kDa subunit	IPI00297084	-0.418	0.11024	20	24	21	20
2995	Peptidyl-prolyl cis-trans isomerase B	IPI00646304	-0.428	0.10914	23	15	21	14
2996	Glucosamine-6-phosphate isomerase 1	IPI00009305	-0.428	0.10914	20	18	18	17
2997	Isoform 1 of Pyruvate dehydrogenase E1 component subunit beta, mitochondrial	IPI00003925	-0.438	0.10811	14	19	11	19
2998	Destrin	IPI00473014	-0.442	0.10742	15	16	16	12
2999	Vesicle-trafficking protein SEC22b	IPI00006865	-0.442	0.10742	16	15	13	15
3000	Sodium/potassium-transporting ATPase subunit beta-3	IPI00008167	-0.445	0.10722	17	13	13	14
3001	Putative uncharacterized protein DKFZp451D234	IPI00031583	-0.447	0.10704	13	16	15	11
3002	60S ribosomal protein L18	IPI00215719	-0.450	0.10667	11	17	11	14
3003	S-formylglutathione hydrolase	IPI00411706	-0.455	0.10618	14	12	14	9
3004	Isoform 1 of Platelet-activating factor acetylhydrolase IB subunit alpha	IPI00218728	-0.461	0.10526	11	13	10	11
3005	Pre-rRNA-processing protein TSR1 homolog	IPI00292894	-0.461	0.10526	11	13	11	10
3006	Cytoplasmic dynein 1 light intermediate chain 1	IPI00007675	-0.465	0.10460	9	14	9	11
3007	proteasome 26S non-ATPase subunit 8	IPI00010201	-0.468	0.10405	10	12	10	9
3008	Splicing factor 3A subunit 1	IPI00017451	-0.468	0.10405	9	13	9	10
3009	WASH complex subunit strumpellin	IPI00029175	-0.472	0.10364	13	8	9	9
3010	Eukaryotic translation initiation factor 5	IPI00022648	-0.472	0.10364	10	11	9	9
3011	SUMO-activating enzyme subunit 1	IPI00033130	-0.476	0.10309	10	10	10	7
3012	cDNA FLJ14239 fis, clone NT2RP5003512, highly similar to Exportin-5	IPI00549861	-0.476	0.10309	12	8	9	8
3013	Isoform 2 of Vacuolar protein sorting-associated protein 13A	IPI00478586	-0.480	0.10257	10	9	5	11
3014	Proteasome subunit alpha type-4	IPI00299155	-0.480	0.10257	8	11	9	7
3015	Chromobox protein homolog 1	IPI00010320	-0.484	0.10168	11	7	8	7
3016	Acylamino-acid-releasing enzyme	IPI00337741	-0.484	0.10168	10	8	9	6
3017	Isoform 1 of Mps one binder kinase activator-like 1B	IPI00301518	-0.489	0.10065	6	11	7	7
3018	Isoform 1 of Transcription elongation regulator 1	IPI00247871	-0.489	0.10065	6	11	6	8
3019	Isoform A of Ras-related C3 botulinum toxin substrate 1	IPI00010271	-0.494	0.10034	7	9	7	6
3020	Dolichol-phosphate mannosyltransferase	IPI00022018	-0.494	0.10034	8	8	6	7
3021	nardilysin isoform a	IPI00243221	-0.500	0.09896	7	8	8	4
3022	Isocitrate dehydrogenase [NADP], mitochondrial	IPI00011107	-0.500	0.09896	9	6	6	6
3023	Mortality factor 4-like protein 2	IPI00014174	-0.506	0.09807	7	7	6	5
3024	Nitric oxide synthase-interacting protein	IPI00006408	-0.506	0.09807	7	7	6	5
3025	Isoform 3 of Myosin phosphatase Rho-interacting protein	IPI00166518	-0.512	0.09690	5	8	4	6
3026	Transcription elongation factor B polypeptide 2	IPI00026670	-0.512	0.09690	9	4	7	3
3027	Isoform B of Serine/threonine-protein kinase 24	IPI00002212	-0.512	0.09690	5	8	5	5

No.	Description	Accession number	STN[1]	p-Value[1]	480_A[2]	480_B[2]	620_A[2]	620_B[2]
3028	Testis-expressed sequence 10 protein	IPI00549664	-0.512	0.09690	5	8	6	4
3029	Bifunctional purine biosynthesis protein PURH	IPI00289499	-0.517	0.09593	36	35	33	34
3030	Citrate synthase, mitochondrial	IPI00025366	-0.520	0.09576	35	34	33	32
3031	Helicase SKI2W	IPI00414819	-0.520	0.09576	5	7	4	5
3032	Phosphatidylinositol-4-phosphate 3-kinase C2 domain-containing subunit alpha	IPI00002580	-0.520	0.09576	5	7	3	6
3033	N-alpha-acetyltransferase 10, NatA catalytic subunit	IPI00013184	-0.520	0.09576	5	7	3	6
3034	Isoform 1 of OCIA domain-containing protein 1	IPI00016405	-0.520	0.09576	6	6	4	5
3035	Prefoldin subunit 4	IPI00015891	-0.520	0.09576	6	6	6	3
3036	lanosterol 14-alpha demethylase isoform 1	IPI00295772	-0.520	0.09576	5	7	3	6
3037	cDNA FLJ56389, highly similar to Elongation factor 1-gamma	IPI00000875	-0.523	0.09466	35	31	32	30
3038	Isoform 1 of N-alpha-acetyltransferase 25, NatB auxiliary subunit	IPI00025890	-0.528	0.09442	5	6	3	5
3039	Isoform 1 of CLIP-associating protein 1	IPI00396279	-0.528	0.09442	6	5	4	4
3040	Proteasome subunit beta type-6	IPI00000811	-0.528	0.09442	5	6	5	3
3041	Pyruvate carboxylase, mitochondrial	IPI00299402	-0.528	0.09442	3	8	3	5
3042	Basic leucine zipper and W2 domain-containing protein 2	IPI00022305	-0.528	0.09442	5	6	3	5
3043	Isoform SNAP-23a of Synaptosomal-associated protein 23	IPI00010438	-0.528	0.09442	5	6	5	3
3044	Isoleucyl-tRNA synthetase, cytoplasmic	IPI00644127	-0.533	0.09349	30	29	26	29
3045	Structural maintenance of chromosomes protein 3	IPI00219420	-0.534	0.09349	30	28	25	29
3046	Isoform 1 of Vacuolar protein sorting-associated protein 29	IPI00170796	-0.537	0.09322	6	4	4	3
3047	Isoform A of DnaJ homolog subfamily B member 6	IPI00024523	-0.537	0.09322	4	6	5	0
3048	Isoform 2 of Putative methyltransferase NSUN5	IPI00101659	-0.537	0.09322	6	4	4	3
3049	NADH dehydrogenase [ubiquinone] 1 beta subcomplex subunit 5, mitochondrial	IPI00013459	-0.537	0.09322	5	5	5	0
3050	Isoform 1 of Cytoskeleton-associated protein 5	IPI00028275	-0.542	0.09115	27	26	24	25
3051	THO complex subunit 7 homolog	IPI00291131	-0.548	0.09071	3	6	4	2
3052	Isoform 1 of Actin-like protein 6A	IPI00003627	-0.548	0.09071	6	3	4	0
3053	Isoform 1 of Nucleoredoxin	IPI00304267	-0.548	0.09071	4	5	0	4
3054	Isoform 1 of Ubiquitin-conjugating enzyme E2 Z	IPI00011996	-0.548	0.09071	5	4	4	0
3055	Secernin-2	IPI00062266	-0.548	0.09071	4	5	3	3
3056	Isoform 1 of AP-2 complex subunit mu	IPI00022256	-0.561	0.08888	4	4	2	3
3057	N-acylsphingosine amidohydrolase (Acid ceramidase) 1, isoform CRA_c	IPI00013698	-0.561	0.08888	6	0	0	3
3058	Probable fructose-2,6-bisphosphatase TIGAR	IPI00006907	-0.561	0.08888	4	4	3	2
3059	39S ribosomal protein L49, mitochondrial	IPI00013195	-0.561	0.08888	5	3	2	3
3060	Cytochrome c1, heme protein, mitochondrial	IPI00029264	-0.561	0.08888	4	4	3	2
3061	DCN1-like protein 1	IPI00291893	-0.561	0.08888	5	3	3	2
3062	Calmodulin-regulated spectrin-associated protein 3	IPI00176702	-0.561	0.08888	4	4	3	2
3063	Cysteine and glycine-rich protein 2	IPI00002824	-0.561	0.08888	5	3	3	2
3064	ATP-dependent RNA helicase DHX8	IPI00031508	-0.561	0.08888	3	5	3	0
3065	Prolactin regulatory element-binding protein	IPI00033349	-0.561	0.08888	4	4	3	0
3066	Ubiquitin-conjugating enzyme E2 H	IPI00020965	-0.561	0.08888	5	3	3	2
3067	cDNA FLJ55543, highly similar to Phosphoacetylglucosamine mutase	IPI00030116	-0.561	0.08888	4	4	3	2
3068	Cysteine and glycine-rich protein 1	IPI00442073	-0.561	0.08888	4	4	3	0
3069	28S ribosomal protein S9, mitochondrial	IPI00641924	-0.561	0.08888	4	4	3	2
3070	Delta-1-pyrroline-5-carboxylate dehydrogenase, mitochondrial	IPI00217871	-0.561	0.08888	4	4	3	0
3071	Isoform 1 of Hepatocyte growth factor-regulated tyrosine kinase substrate	IPI00006176	-0.561	0.08888	2	6	0	3
3072	Ras-related protein Rab-24	IPI00056496	-0.561	0.08888	5	3	3	0
3073	Importin subunit alpha-3	IPI00299033	-0.561	0.08888	4	4	3	2
3074	Isoform Long of 14-3-3 protein beta/alpha	IPI00216318	-0.570	0.08795	18	21	19	16
3075	KH-type splicing regulatory protein	IPI00479786	-0.570	0.08795	19	20	17	18
3076	Isoform 1 of Serum paraoxonase/arylesterase 2	IPI00014958	-0.576	0.08716	4	3	2	0
3077	Probable E3 ubiquitin-protein ligase HERC2	IPI00005826	-0.576	0.08716	4	3	0	0
3078	Integrin-linked protein kinase	IPI00013219	-0.576	0.08716	3	4	2	2
3079	Isoform 2 of Dephospho-CoA kinase domain-containing protein	IPI00015737	-0.576	0.08716	3	4	0	0
3080	Isoform 1 of tRNA 2'-phosphotransferase 1	IPI00328580	-0.576	0.08716	4	3	0	2
3081	Ferritin	IPI00375676	-0.576	0.08716	5	2	0	0
3082	Tyrosine-protein kinase SgK223	IPI00739386	-0.576	0.08716	4	3	0	0
3083	14 kDa phosphohistidine phosphatase	IPI00299977	-0.576	0.08716	4	3	2	2
3084	NADH-cytochrome b5 reductase 1	IPI00470674	-0.576	0.08716	3	4	0	0
3085	Dolichyl-phosphate beta-glucosyltransferase	IPI00002506	-0.576	0.08716	4	3	0	0
3086	Ubiquitin-conjugating enzyme E2 variant 2	IPI00019600	-0.576	0.08716	4	3	0	0
3087	Isoform 1 of Beta-galactosidase	IPI00441344	-0.576	0.08716	3	4	0	2
3088	Isoform 1 of Pleckstrin homology-like domain family B member 1	IPI00413100	-0.576	0.08716	3	4	0	0
3089	Isoform 1 of Translocon-associated protein subunit alpha	IPI00301021	-0.576	0.08716	5	2	0	2
3090	COMM domain-containing protein 9	IPI00305212	-0.576	0.08716	5	0	0	2
3091	nuclear factor NF-kappa-B p100 subunit isoform b	IPI00807463	-0.576	0.08716	4	3	0	0
3092	Isoform 1 of Ubiquitin carboxyl-terminal hydrolase 19	IPI00016589	-0.576	0.08716	3	4	0	0
3093	PDXDC1 protein	IPI00329208	-0.576	0.08716	4	3	0	0
3094	CCR4-NOT transcription complex subunit 7	IPI00006552	-0.576	0.08716	0	5	2	2
3095	Isoform 1 of Protein sel-1 homolog 1	IPI00002790	-0.576	0.08716	4	3	0	0
3096	Isoform 1 of Methyltransferase-like protein 9	IPI00100239	-0.576	0.08716	4	3	0	2
3097	Ras-related protein Rab-13	IPI00016373	-0.576	0.08716	3	4	0	0
3098	Hemoglobin subunit alpha	IPI00410714	-0.576	0.08716	3	4	0	0
3099	Isoform 2 of Neural cell adhesion molecule L1	IPI00334532	-0.576	0.08716	2	5	0	0
3100	Isoform 1 of Kinesin-like protein KIF23	IPI00291579	-0.576	0.08716	2	5	2	2
3101	Y-box-binding protein 2	IPI00250153	-0.576	0.08716	5	2	2	2
3102	F-box-like/WD repeat-containing protein TBL1XR1	IPI00002922	-0.576	0.08716	5	2	2	2
3103	Isoform 1 of Tyrosine-protein kinase ZAP-70	IPI00329789	-0.576	0.08716	4	3	0	0
3104	cDNA FLJ56370, highly similar to Homo sapiens FK506 binding protein 8, 38kDa (FKBP8), mRNA	IPI00328161	-0.576	0.08716	4	3	0	2
3105	Torsin family protein C9orf167	IPI00015101	-0.576	0.08716	3	4	0	0
3106	Isoform 2 of Mannose-1-phosphate guanyltransferase beta	IPI00002496	-0.576	0.08716	3	4	0	0
3107	MAGUK p55 subfamily member 6	IPI00303280	-0.576	0.08716	5	2	0	0
3108	SLIT-ROBO Rho GTPase-activating protein 2	IPI00479125	-0.576	0.08716	3	4	0	0
3109	Isoform 1 of Cell division cycle protein 23 homolog	IPI00005822	-0.576	0.08716	0	5	0	0
3110	Isoform 1 of Pericentriolar material 1 protein	IPI00006213	-0.576	0.08716	3	4	0	0
3111	Amine oxidase [flavin-containing] A	IPI00008483	-0.576	0.08716	4	3	0	0
3112	Acyl-coenzyme A thioesterase 8	IPI00298202	-0.576	0.08716	3	4	0	0
3113	Isoform 1 of Uridine phosphorylase 1	IPI00004406	-0.576	0.08716	5	0	0	0
3114	nebulette isoform 2	IPI00418240	-0.576	0.08716	4	3	0	0
3115	2-hydroxyacyl-CoA lyase 1	IPI00296535	-0.576	0.08716	4	3	0	0
3116	Isoform 2 of Septin-8	IPI00022082	-0.576	0.08716	3	4	2	2
3117	Arf-GAP with coiled-coil, ANK repeat and PH domain-containing protein 2	IPI00014264	-0.576	0.08716	2	5	0	0
3118	Isoform 3 of Protein unc-13 homolog D	IPI00456635	-0.576	0.08716	3	4	0	0
3119	Cell adhesion molecule 4	IPI00176427	-0.576	0.08716	3	4	0	0
3120	Cystatin-S	IPI00032294	-0.576	0.08716	3	4	0	0
3121	Cleavage and polyadenylation specificity factor subunit 5	IPI00646917	-0.577	0.08592	17	19	17	15
3122	Ubiquitin carboxyl-terminal hydrolase 14	IPI00219913	-0.585	0.08396	16	17	14	15

No.	Description	Accession number	STN[1]	p-Value[1]	480_A[2]	480_B[2]	620_A[2]	620_B[2]
3123	Eukaryotic peptide chain release factor subunit 1	IPI00429191	-0.592	0.08321	14	17	11	16
3124	Microtubule-associated protein RP/EB family member 1	IPI00017596	-0.595	0.08310	16	14	12	14
3125	Isoform 1 of Transformation/transcription domain-associated protein	IPI00069084	-0.598	0.08200	16	13	11	14
3126	Nuclear pore complex protein Nup133	IPI00291200	-0.602	0.08197	14	14	11	13
3127	V-type proton ATPase subunit B, brain isoform	IPI00007812	-0.602	0.08197	13	15	13	11
3128	Isoform 1 of Pyridoxal kinase	IPI00013004	-0.605	0.08104	11	16	11	12
3129	Isoform 1a of Oxysterol-binding protein-related protein 3	IPI00023555	-0.605	0.08104	15	12	8	15
3130	Cytochrome c	IPI00465315	-0.609	0.08104	15	11	10	12
3131	Nuclear pore complex protein Nup107	IPI00028005	-0.609	0.08104	14	12	10	12
3132	EH domain-containing protein 1	IPI00017184	-0.609	0.08104	15	11	10	12
3133	Isoform 2 of Nuclear protein localization protein 4 homolog	IPI00001676	-0.613	0.07973	13	12	11	10
3134	Tyrosine-protein phosphatase non-receptor type 1	IPI00297261	-0.617	0.07973	13	11	10	10
3135	cDNA FLJ54957, highly similar to Transketolase	IPI00643920	-0.619	0.07867	50	44	47	42
3136	Isoform 3 of DNA topoisomerase 2-alpha	IPI00218753	-0.620	0.07867	46	47	47	41
3137	Myosin regulatory light chain 12B	IPI00033494	-0.622	0.07856	14	9	9	10
3138	Eukaryotic translation initiation factor 3 subunit K	IPI00033143	-0.622	0.07856	12	11	10	9
3139	Isoform 1 of LETM1 and EF-hand domain-containing protein 1, mitochondrial	IPI00017592	-0.627	0.07849	11	11	8	10
3140	Biliverdin reductase A	IPI00294158	-0.627	0.07849	11	11	11	7
3141	SWI/SNF related, matrix associated, actin dependent regulator of chromatin, subfamily a, member 1	IPI00216046	-0.632	0.07660	10	11	10	7
3142	Isoform 1 of U2-associated protein SR140	IPI00143753	-0.643	0.07447	9	10	6	9
3143	Tubulin beta-3 chain	IPI00013683	-0.643	0.07447	9	10	8	7
3144	Protein NipSnap homolog 3A	IPI00004845	-0.649	0.07416	8	10	5	9
3145	Isoform Alpha-6X1X2B of Integrin alpha-6	IPI00010697	-0.656	0.07186	13	4	5	8
3146	Cytochrome b-c1 complex subunit Rieske, mitochondrial	IPI00026964	-0.656	0.07186	9	8	5	8
3147	Isoform 1 of Uridine 5'-monophosphate synthase	IPI00003923	-0.656	0.07186	10	7	6	7
3148	Ras GTPase-activating-like protein IQGAP1	IPI00009342	-0.659	0.07144	97	104	97	98
3149	Isoform 1 of Transcription intermediary factor 1-beta	IPI00438229	-0.660	0.07144	33	30	30	28
3150	Rho GTPase-activating protein 1	IPI00020567	-0.663	0.07141	8	8	5	7
3151	Isoform 4 of Abhydrolase domain-containing protein 11	IPI00171152	-0.663	0.07141	9	7	7	5
3152	Succinate dehydrogenase [ubiquinone] iron-sulfur subunit, mitochondrial	IPI00294911	-0.663	0.07141	10	6	6	6
3153	39S ribosomal protein L9, mitochondrial	IPI00307409	-0.663	0.07141	8	8	5	7
3154	Isoform Rpn10A of 26S proteasome non-ATPase regulatory subunit 4	IPI00022694	-0.663	0.07141	9	7	8	4
3155	cDNA FLJ55574, highly similar to Calnexin	IPI00020984	-0.667	0.06941	28	31	25	29
3156	Nucleoporin 85	IPI00171542	-0.670	0.06921	7	8	7	4
3157	Asparagine synthetase [glutamine-hydrolyzing]	IPI00554777	-0.670	0.06921	6	9	5	6
3158	Copine-1	IPI00018452	-0.670	0.06921	7	8	5	6
3159	Mitochondrial import inner membrane translocase subunit TIM44	IPI00306516	-0.670	0.06921	8	7	6	5
3160	cDNA FLJ55034, highly similar to Dihydrolipoyllysine-residue succinyltransferase component	IPI00384122	-0.670	0.06921	7	8	5	6
3161	Isoform 1 of Nucleoside diphosphate kinase A	IPI00012048	-0.676	0.06907	25	29	24	25
3162	B-cell receptor-associated protein 31	IPI00218200	-0.679	0.06904	8	6	4	6
3163	Cytoplasmic dynein 1 light intermediate chain 2	IPI00011592	-0.679	0.06904	6	8	6	4
3164	Seryl-tRNA synthetase, cytoplasmic	IPI00220637	-0.679	0.06904	7	7	5	5
3165	Crk-like protein	IPI00004839	-0.679	0.06904	8	6	5	5
3166	Ubiquitin-conjugating enzyme E2 C	IPI00013002	-0.679	0.06904	9	5	5	5
3167	dynactin subunit 2	IPI00220503	-0.679	0.06904	7	7	6	4
3168	General transcription factor IIF subunit 2	IPI00477686	-0.688	0.06515	5	8	4	5
3169	cDNA FLJ30398 fis, clone BRACE2008402, highly similar to Steroid receptor RNA activator 1	IPI00102313	-0.688	0.06515	7	6	3	6
3170	Inositol 1,4,5-trisphosphate receptor type 3	IPI00291607	-0.699	0.06450	2	10	2	6
3171	zinc finger protein 294	IPI00783835	-0.699	0.06450	6	6	4	4
3172	DnaJ homolog subfamily B member 11	IPI00008454	-0.699	0.06450	6	6	4	4
3173	Isoform p26 of 7,8-dihydro-8-oxoguanine triphosphatase	IPI00004392	-0.699	0.06450	7	5	3	5
3174	cDNA FLJ53975, highly similar to Acetyl-CoA acetyltransferase, cytosolic	IPI00291419	-0.699	0.06450	6	6	4	4
3175	Serine/threonine-protein phosphatase 4 catalytic subunit	IPI00012833	-0.699	0.06450	6	6	5	3
3176	Isoform 1 of Rab GTPase-activating protein 1	IPI00016702	-0.699	0.06450	7	5	4	4
3177	Isoform 1 of Melanoma-associated antigen D2	IPI00009542	-0.699	0.06450	6	6	4	4
3178	Isoform 1 of PC4 and SFRS1-interacting protein	IPI00028122	-0.711	0.06009	5	6	5	0
3179	YLP motif-containing protein 1	IPI00165434	-0.711	0.06009	5	6	3	4
3180	GTP-binding protein Rheb	IPI00016669	-0.711	0.06009	6	5	4	3
3181	Sphingosine-1-phosphate lyase 1	IPI00099463	-0.711	0.06009	5	6	4	3
3182	Putative uncharacterized protein NAPRT1	IPI00412498	-0.711	0.06009	7	4	3	4
3183	UDP-glucose 4-epimerase	IPI00553131	-0.711	0.06009	5	6	4	3
3184	Endoplasmic reticulum resident protein 44	IPI00401264	-0.711	0.06009	2	9	3	4
3185	Importin subunit alpha-1	IPI00303292	-0.711	0.06009	6	5	3	4
3186	Isoform 1 of Putative splicing factor, arginine/serine-rich 14	IPI00158020	-0.711	0.06009	4	7	3	4
3187	26S proteasome non-ATPase regulatory subunit 6	IPI00014151	-0.714	0.06002	19	20	16	18
3188	Beta-actin-like protein 2	IPI00003269	-0.723	0.05968	57	55	55	51
3189	ADP-ribosylation factor 5	IPI00215919	-0.725	0.05958	5	5	3	3
3190	Sulfhydryl oxidase 2	IPI00376394	-0.725	0.05958	5	5	2	4
3191	Kinetochore protein Spc24	IPI00168317	-0.725	0.05958	6	4	3	3
3192	Nucleoside-triphosphatase C1orf57	IPI00031570	-0.725	0.05958	6	4	4	2
3193	Serine/threonine-protein kinase OSR1	IPI00010080	-0.725	0.05958	5	5	2	4
3194	Short/branched chain specific acyl-CoA dehydrogenase, mitochondrial	IPI00024623	-0.725	0.05958	5	5	3	3
3195	Isoform Non-muscle of Myosin light polypeptide 6	IPI00335168	-0.730	0.05658	19	15	14	15
3196	cDNA FLJ34068 fis, clone FCBBF3001918, highly similar to SERINE/THREONINE PROTEIN PHOSPHATASE	IPI00168184	-0.738	0.05624	16	16	14	13
3197	Isoform 2 of N-alpha-acetyltransferase 15, NatA auxiliary subunit	IPI00032158	-0.738	0.05624	15	17	12	15
3198	Isoform 1 of Probable ATP-dependent RNA helicase DHX36	IPI00027415	-0.741	0.05583	4	5	3	0
3199	V-type proton ATPase subunit D	IPI00001568	-0.741	0.05583	6	3	2	3
3200	Pre-mRNA-splicing factor CWC22 homolog	IPI00177381	-0.741	0.05583	3	6	0	3
3201	Cohesin subunit SA-1	IPI00025158	-0.741	0.05583	5	4	0	3
3202	Interferon-induced 17 kDa protein	IPI00375631	-0.741	0.05583	5	4	3	2
3203	Isoform 3 of Guanine nucleotide exchange factor VAV2	IPI00004977	-0.741	0.05583	4	5	3	2
3204	Putative uncharacterized protein ZYX	IPI00924931	-0.741	0.05583	4	5	2	3
3205	Isoform 2 of Late secretory pathway protein AVL9 homolog	IPI00022042	-0.741	0.05583	5	4	3	2
3206	26S proteasome non-ATPase regulatory subunit 13 isoform 2	IPI00375380	-0.746	0.05548	15	15	11	14
3207	sister chromatid cohesion protein PDS5 homolog A isoform 2	IPI00303063	-0.750	0.05541	14	15	10	14
3208	Peroxiredoxin-2	IPI00027350	-0.754	0.05521	14	14	14	9
3209	Vesicular integral-membrane protein VIP36	IPI00009950	-0.754	0.05521	13	15	11	12
3210	Isoform 7 of Protein BAT2-like 2	IPI00083708	-0.760	0.05483	4	4	2	2
3211	Isoform 1 of 28S ribosomal protein S35, mitochondrial	IPI00073779	-0.760	0.05483	3	5	0	2
3212	Carboxypeptidase D	IPI00027078	-0.760	0.05483	4	4	2	2
3213	ATP synthase subunit delta, mitochondrial	IPI00024920	-0.760	0.05483	4	4	0	0
3214	Isoform Bcl-X(L) of Bcl-2-like protein 1	IPI00019983	-0.760	0.05483	4	4	2	0
3215	Keratin-81-like protein	IPI00008669	-0.760	0.05483	4	4	0	0

No.	Description	Accession number	STN[1]	p-Value[1]	480_A[2]	480_B[2]	620_A[2]	620_B[2]
3216	Isoform 1 of Zinc phosphodiesterase ELAC protein 2	IPI00396627	-0.760	0.05483	4	4	2	2
3217	60S ribosomal protein L31	IPI00026302	-0.760	0.05483	6	0	2	2
3218	Serine/threonine-protein phosphatase 1 regulatory subunit 10	IPI00298731	-0.760	0.05483	3	5	0	2
3219	Huntingtin-interacting protein 1	IPI00782965	-0.760	0.05483	4	4	2	2
3220	AP-3 complex subunit beta-2	IPI00005793	-0.760	0.05483	4	4	0	2
3221	Argininosuccinate synthase	IPI00020632	-0.760	0.05483	4	4	0	2
3222	Isoform 1 of DNA dC->dU-editing enzyme APOBEC-3G	IPI00396656	-0.760	0.05483	4	4	0	0
3223	Isoform 1 of YTH domain family protein 1	IPI00221345	-0.760	0.05483	5	3	2	2
3224	5'-nucleotidase domain-containing protein 1	IPI00177965	-0.760	0.05483	3	5	0	0
3225	Isoform Long of Glutaryl-CoA dehydrogenase, mitochondrial	IPI00024317	-0.760	0.05483	4	4	2	2
3226	Sterol 26-hydroxylase, mitochondrial	IPI00025307	-0.760	0.05483	4	4	0	0
3227	Keratin, type I cytoskeletal 23	IPI00304458	-0.760	0.05483	4	4	0	0
3228	Leucine zipper transcription factor-like protein 1	IPI00299465	-0.760	0.05483	4	4	0	0
3229	Prostaglandin reductase 1	IPI00292657	-0.760	0.05483	4	4	0	0
3230	24 kDa protein	IPI00243338	-0.760	0.05483	4	4	0	0
3231	165 kDa protein	IPI00240812	-0.764	0.04823	12	14	10	11
3232	Isoform 1 of Squamous cell carcinoma antigen recognized by T-cells 3	IPI00006025	-0.769	0.04781	12	13	11	9
3233	Alpha-centractin	IPI00029468	-0.775	0.04774	14	10	10	9
3234	myosin regulatory light polypeptide 9 isoform b	IPI00030929	-0.781	0.04747	11	12	9	9
3235	Glucose-6-phosphate isomerase	IPI00027497	-0.787	0.04699	40	26	30	30
3236	Reticulocalbin-1	IPI00015842	-0.793	0.04654	9	12	7	9
3237	Isoform 1 of Acyl-coenzyme A thioesterase 9, mitochondrial	IPI00220710	-0.793	0.04654	13	8	8	8
3238	Isoform 1 of Methionine adenosyltransferase 2 subunit beta	IPI00002324	-0.793	0.04654	11	10	9	7
3239	DYNC1H1 protein	IPI00440177	-0.800	0.04592	9	11	9	6
3240	Succinyl-CoA ligase [GDP-forming] subunit beta, mitochondrial	IPI00096066	-0.800	0.04592	11	9	7	8
3241	Isoform 1 of Interferon-inducible double stranded RNA-dependent protein kinase activator A	IPI00021167	-0.808	0.04540	8	11	8	6
3242	Exportin-7	IPI00302458	-0.808	0.04540	8	11	6	8
3243	Macrophage-capping protein	IPI00027341	-0.808	0.04540	9	10	7	7
3244	Isoform 2 of Septin-11	IPI00019376	-0.808	0.04540	10	9	7	7
3245	Developmentally-regulated GTP-binding protein 1	IPI00031836	-0.816	0.04506	10	8	6	7
3246	Pre-mRNA-processing factor 6	IPI00305068	-0.816	0.04506	11	7	7	6
3247	Sialic acid synthase	IPI00147874	-0.816	0.04506	6	12	5	8
3248	Trifunctional enzyme subunit alpha, mitochondrial	IPI00031522	-0.818	0.04475	24	28	20	26
3249	Thioredoxin-like protein 1	IPI00305692	-0.824	0.04434	8	9	6	6
3250	Probable ribosome biogenesis protein NEP1	IPI00025347	-0.824	0.04434	8	9	6	6
3251	Eukaryotic translation initiation factor 2A	IPI00012462	-0.834	0.04386	7	9	7	4
3252	Tricarboxylate transport protein, mitochondrial	IPI00294159	-0.834	0.04386	8	8	6	5
3253	60S acidic ribosomal protein P1	IPI00008527	-0.834	0.04386	8	8	4	7
3254	Isoleucyl-tRNA synthetase	IPI00514082	-0.834	0.04386	10	6	5	6
3255	Isoform 1 of Large proline-rich protein BAT2	IPI00010700	-0.844	0.04300	7	8	5	5
3256	Diablo homolog, mitochondrial precursor	IPI00008418	-0.844	0.04300	8	7	5	5
3257	Cytoplasmic aconitate hydratase	IPI00008485	-0.855	0.04221	5	9	4	5
3258	Similar to nonhistone chromosomal protein HMG-1	IPI00418184	-0.855	0.04221	6	8	4	5
3259	Ribose-5-phosphate isomerase	IPI00026513	-0.855	0.04221	5	9	6	3
3260	Isoform 2 of Phosphoglucomutase-1	IPI00217872	-0.855	0.04221	6	8	5	4
3261	sideroflexin-3	IPI00793874	-0.855	0.04221	6	8	5	4
3262	Apolipoprotein O-like	IPI00394809	-0.868	0.04183	6	7	4	4
3263	Sterol-4-alpha-carboxylate 3-dehydrogenase, decarboxylating	IPI00019407	-0.868	0.04183	6	7	2	6
3264	PRA1 family protein 2	IPI00026994	-0.868	0.04183	6	7	4	4
3265	Isoform 1 of Syntenin-1	IPI00299086	-0.868	0.04183	6	7	3	5
3266	Protein ERGIC-53	IPI00026530	-0.868	0.04183	7	6	3	5
3267	Exportin-1	IPI00298961	-0.874	0.04135	46	44	39	44
3268	Isoform 1 of Septin-2	IPI00014177	-0.874	0.04135	18	17	14	15
3269	Isoform Complexed of Arginyl-tRNA synthetase, cytoplasmic	IPI00004860	-0.874	0.04135	18	17	16	13
3270	Isoform 3 of Obg-like ATPase 1	IPI00216106	-0.882	0.04049	6	6	4	3
3271	Isoform 1 of V-type proton ATPase subunit H	IPI00296191	-0.882	0.04049	7	5	0	5
3272	NADPH--cytochrome P450 reductase	IPI00470467	-0.882	0.04049	7	5	4	3
3273	Nucleolysin TIAR	IPI00005615	-0.882	0.04049	5	7	3	4
3274	DnaJ homolog subfamily C member 8	IPI00003438	-0.882	0.04049	6	6	4	3
3275	cDNA FLJ20475 fis, clone KAT07206	IPI00183065	-0.882	0.04049	5	7	4	3
3276	Heterogeneous nuclear ribonucleoprotein H	IPI00013881	-0.888	0.04038	17	15	17	9
3277	HMT1 hnRNP methyltransferase-like 2 isoform 1	IPI00018522	-0.888	0.04038	17	15	13	13
3278	Isoform 1 of Importin-4	IPI00156374	-0.888	0.04038	16	16	15	11
3279	Isoform 1 of Surfeit locus protein 4	IPI00005737	-0.892	0.03956	13	18	13	12
3280	Keratin-8-like protein 1	IPI00017870	-0.893	0.03956	37	42	30	42
3281	Isoform 1 of Serine/threonine-protein kinase ATR	IPI00412298	-0.899	0.03914	4	7	4	2
3282	Component of gems 4	IPI00027717	-0.899	0.03914	6	5	3	3
3283	mRNA export factor	IPI00019733	-0.899	0.03914	6	5	3	3
3284	Tyrosine-protein phosphatase non-receptor type 12	IPI00289082	-0.899	0.03914	6	5	0	4
3285	cDNA FLJ77422	IPI00011268	-0.903	0.03890	16	13	10	13
3286	Secernin-1	IPI00289862	-0.918	0.03828	7	3	3	0
3287	Isoform Del-701 of Signal transducer and activator of transcription 3	IPI00306436	-0.918	0.03828	5	5	3	2
3288	Vesicle-associated membrane protein 3	IPI00549343	-0.918	0.03828	5	5	2	3
3289	Sorting nexin-9	IPI00001883	-0.918	0.03828	5	5	3	2
3290	Syntaxin-4	IPI00029730	-0.918	0.03828	6	4	0	3
3291	Putative uncharacterized protein PYCR2	IPI00335061	-0.918	0.03828	5	5	2	3
3292	Isoform 2 of Nucleoporin NUP188 homolog	IPI00385001	-0.927	0.03732	13	12	10	9
3293	Aconitate hydratase, mitochondrial	IPI00017855	-0.927	0.03732	13	12	8	11
3294	Thioredoxin	IPI00216298	-0.927	0.03732	14	11	8	11
3295	Actin, aortic smooth muscle	IPI00008603	-0.931	0.03732	64	76	65	67
3296	Integrin beta-5	IPI00029741	-0.941	0.03670	4	5	0	0
3297	AP-3 complex subunit sigma-1	IPI00014624	-0.941	0.03670	6	3	0	2
3298	SEC24B protein	IPI00030851	-0.941	0.03670	3	6	0	0
3299	Adenylate kinase isoenzyme 4, mitochondrial	IPI00016568	-0.941	0.03670	5	4	0	0
3300	Isoform 2 of Sacsin	IPI00784002	-0.941	0.03670	5	4	0	0
3301	Thyroid receptor-interacting protein 11	IPI00003515	-0.941	0.03670	3	6	2	2
3302	[Pyruvate dehydrogenase [acetyl-transferring]]-phosphatase 1, mitochondria	IPI00218971	-0.941	0.03670	5	4	2	2
3303	Isoform 1 of Dynamin-binding protein	IPI00174025	-0.941	0.03670	3	6	2	0
3304	Glycerol-3-phosphate dehydrogenase 1-like protein	IPI00032959	-0.941	0.03670	4	5	2	0
3305	Nucleolar complex protein 2 homolog	IPI00411886	-0.941	0.03670	4	5	0	2
3306	Transmembrane protein 205	IPI00063130	-0.941	0.03670	3	6	0	0
3307	DDB1- and CUL4-associated factor 7	IPI00006754	-0.941	0.03670	5	4	2	0
3308	Serine/threonine-protein kinase N2	IPI00002804	-0.941	0.03670	6	3	0	2
3309	Isoform LMP2.L of Proteasome subunit beta type-9	IPI00000787	-0.941	0.03670	6	3	0	0
3310	Carbonic anhydrase 1	IPI00215983	-0.941	0.03670	3	6	0	0

No.	Description	Accession number	STN[1]	p-Value[1]	480_A[2]	480_B[2]	620_A[2]	620_B[2]
3311	Isoform Alpha-3A of Integrin alpha-3	IPI00215995	-0.941	0.03670	4	5	0	0
3312	Isoform 1 of Cytoskeleton-associated protein 4	IPI00141318	-0.941	0.03670	5	4	0	0
3313	Laminin subunit beta-3	IPI00299404	-0.941	0.03670	3	6	0	0
3314	Tubulin beta-2C chain	IPI00007752	-0.950	0.03577	125	136	124	128
3315	twinfilin-1	IPI00183508	-0.957	0.03557	11	10	6	9
3316	Isoform Long of Double-stranded RNA-binding protein Staufen homolog 1	IPI00000001	-0.957	0.03557	11	10	8	7
3317	Isoform 1 of Ubiquitin-conjugating enzyme E2 K	IPI00021370	-0.957	0.03557	11	10	8	7
3318	proteasome-associated protein ECM29 homolog	IPI00157790	-0.959	0.03557	25	26	27	17
3319	Isoform E of Eukaryotic translation initiation factor 4 gamma 1	IPI00386533	-0.962	0.03557	25	25	21	22
3320	cDNA FLJ56285, highly similar to ADP-ribosylation factor-like protein 8B	IPI00018871	-0.965	0.03539	9	11	7	7
3321	GTP-binding protein SAR1b	IPI00002149	-0.965	0.03539	11	9	9	5
3322	Isoform Crk-II of Adapter molecule crk	IPI00004838	-0.965	0.03539	10	10	7	7
3323	Glyoxylate reductase/hydroxypyruvate reductase	IPI00037448	-0.965	0.03539	9	11	7	7
3324	Isoform 2 of Ubiquitin-associated protein 2-like	IPI00029019	-0.965	0.03539	11	9	7	7
3325	Isoform 2 of 4F2 cell-surface antigen heavy chain	IPI00027493	-0.966	0.03495	23	26	20	22
3326	Proteasome subunit beta type-4	IPI00555956	-0.972	0.03484	27	20	21	19
3327	Ribosomal L1 domain-containing protein 1	IPI00008708	-0.975	0.03478	9	10	6	7
3328	cDNA FLJ78679, highly similar to Homo sapiens DEAD (Asp-Glu-Ala-Asp) box polypeptide 46 (DDX46)	IPI00329791	-0.976	0.03478	29	17	21	18
3329	Isoform 1 of N-acylneuraminate cytidylyltransferase	IPI00303158	-0.985	0.03478	8	10	7	5
3330	Cytosolic purine 5'-nucleotidase	IPI00029054	-0.985	0.03478	9	9	6	6
3331	Isoform 1 of Protein phosphatase 1 regulatory subunit 12A	IPI00183002	-0.985	0.03478	9	9	5	7
3332	Kinesin-1 heavy chain	IPI00012837	-0.987	0.03371	21	22	20	16
3333	Isoform Short of Ubiquitin fusion degradation protein 1 homolog	IPI00218292	-0.996	0.03350	10	7	6	5
3334	Isoform 1 of Replication protein A 32 kDa subunit	IPI00013939	-1.008	0.03306	9	7	7	3
3335	V-type proton ATPase subunit C 1	IPI00007814	-1.008	0.03306	9	7	4	6
3336	14-3-3 protein gamma	IPI00220642	-1.013	0.03196	20	17	15	15
3337	Putative uncharacterized protein ALB	IPI00022434	-1.021	0.03178	6	9	4	5
3338	Gamma-soluble NSF attachment protein	IPI00293817	-1.021	0.03178	8	7	4	5
3339	Ras-related protein Rab-14	IPI00291928	-1.023	0.03178	17	18	15	13
3340	Translin	IPI00018768	-1.028	0.03171	16	18	14	13
3341	Signal recognition particle receptor subunit beta	IPI00295098	-1.033	0.03140	18	15	14	12
3342	Isoform 2 of Actin-binding protein anillin	IPI00032958	-1.036	0.03140	5	9	3	5
3343	DNA topoisomerase 1	IPI00413611	-1.051	0.03013	17	13	12	11
3344	Trafficking protein particle complex subunit 3	IPI00004324	-1.052	0.03003	7	6	3	4
3345	Isoform 1 of Chromosome-associated kinesin KIF4A	IPI00178150	-1.052	0.03003	7	6	4	3
3346	Guanine deaminase	IPI00644409	-1.052	0.03003	6	7	3	4
3347	Isoform A of Phosphate carrier protein, mitochondrial	IPI00022202	-1.055	0.03003	35	30	30	27
3348	Protein phosphatase inhibitor 2	IPI00220402	-1.071	0.02986	6	6	3	3
3349	Isoform B of AP-2 complex subunit alpha-1	IPI00256684	-1.086	0.02779	10	15	9	9
3350	Isoform Mitochondrial of Peroxiredoxin-5, mitochondrial	IPI00024915	-1.091	0.02766	29	24	22	23
3351	Transmembrane 9 superfamily member 4	IPI00021985	-1.093	0.02762	4	7	3	2
3352	Heat shock-related 70 kDa protein 2	IPI00007702	-1.093	0.02762	5	6	2	3
3353	Calcium-binding mitochondrial carrier protein Aralar2	IPI00007084	-1.094	0.02762	11	13	10	7
3354	Staphylococcal nuclease domain-containing protein 1	IPI00140420	-1.095	0.02755	54	53	48	50
3355	Regulation of nuclear pre-mRNA domain-containing protein 1B	IPI00009659	-1.103	0.02745	11	12	9	7
3356	Isoform F of Protein SON	IPI00000192	-1.112	0.02704	11	11	9	6
3357	Transgelin-2	IPI00550363	-1.113	0.02704	25	22	19	20
3358	Cathepsin B	IPI00295741	-1.120	0.02680	3	7	0	2
3359	Isoform 1 of Peroxisomal proliferator-activated receptor A-interacting complex 285 kDa proteir	IPI00249304	-1.120	0.02680	7	3	0	0
3360	Isoform 3 of Protein scribble homolog	IPI00410666	-1.120	0.02680	5	5	2	2
3361	General transcription factor 3C polypeptide 4	IPI00016725	-1.120	0.02680	3	7	0	0
3362	Heat shock protein beta-11	IPI00098827	-1.120	0.02680	5	5	2	2
3363	Serine/threonine-protein kinase MRCK beta	IPI00477763	-1.120	0.02680	4	6	2	2
3364	Prenylcysteine oxidase 1	IPI00384280	-1.120	0.02680	4	6	2	2
3365	Isoform 1 of RNA polymerase II-associated protein 1	IPI00402657	-1.120	0.02680	4	6	2	2
3366	Retinol dehydrogenase 14	IPI00177940	-1.120	0.02680	5	5	2	2
3367	Isoform 3 of Serine/threonine-protein kinase SMG1	IPI00183368	-1.120	0.02680	5	5	2	2
3368	Unhealthy ribosome biogenesis protein 2 homolog	IPI00028980	-1.120	0.02680	5	5	0	2
3369	AFG3-like protein 2	IPI00001091	-1.120	0.02680	7	3	2	2
3370	Serine/threonine-protein kinase SRPK2	IPI00333420	-1.120	0.02680	5	5	0	0
3371	Isoform 4 of Afadin	IPI00023461	-1.120	0.02680	6	4	0	0
3372	Isoform 5 of Protein transport protein Sec16A	IPI00031242	-1.120	0.02680	5	5	0	0
3373	Isoform 1 of Endophilin-B2	IPI00024540	-1.120	0.02680	5	5	2	0
3374	Isoform 1 of Actin filament-associated protein 1-like 2	IPI00181905	-1.120	0.02680	4	6	0	0
3375	cDNA FLJ56176, highly similar to Poly(A) polymerase alpha	IPI00384028	-1.120	0.02680	5	5	2	2
3376	Isoform 1 of Striatin	IPI00014456	-1.120	0.02680	4	6	0	2
3377	Tumor necrosis factor alpha-induced protein 2	IPI00304866	-1.120	0.02680	5	5	0	0
3378	14-3-3 protein theta	IPI00018146	-1.122	0.02501	25	20	20	17
3379	Isoform 1 of Kinectin	IPI00328753	-1.126	0.02497	24	20	19	17
3380	Isoform 1 of Thyroid receptor-interacting protein 13	IPI00003505	-1.132	0.02480	10	10	5	8
3381	Isoform 2 of Cleft lip and palate transmembrane protein 1	IPI00107357	-1.156	0.02377	7	11	7	4
3382	Echinoderm microtubule-associated protein-like 4	IPI00001466	-1.185	0.02284	12	4	6	3
3383	D-3-phosphoglycerate dehydrogenase	IPI00011200	-1.185	0.02284	8	8	5	4
3384	Ras-related protein Rab-21	IPI00007755	-1.185	0.02284	8	8	4	5
3385	Glutamate--cysteine ligase regulatory subunit	IPI00010090	-1.185	0.02284	8	8	4	5
3386	Isoform 1 of Vesicle-associated membrane protein-associated protein B/C	IPI00006211	-1.191	0.02284	17	15	12	12
3387	Isoform 2 of Proteasome subunit alpha type-3	IPI00171199	-1.191	0.02284	17	15	13	11
3388	Eukaryotic translation initiation factor 2 subunit 2	IPI00021728	-1.198	0.02239	16	15	11	12
3389	Isoform 2 of AP-3 complex subunit delta-1	IPI00289608	-1.201	0.02236	6	9	3	5
3390	Armadillo repeat-containing X-linked protein 3	IPI00009906	-1.201	0.02236	7	8	6	0
3391	3-ketoacyl-CoA thiolase, mitochondrial	IPI00001539	-1.212	0.02167	16	13	9	12
3392	Isoform Short of NADPH:adrenodoxin oxidoreductase, mitochondrial	IPI00026958	-1.220	0.02146	7	7	3	4
3393	Protein S100-A11	IPI00013895	-1.228	0.02143	16	11	10	9
3394	Ornithine aminotransferase, mitochondrial	IPI00022334	-1.237	0.02136	14	12	10	8
3395	Isoform A1 of Tight junction protein ZO-2	IPI00003843	-1.256	0.02074	6	18	7	9
3396	D-beta-hydroxybutyrate dehydrogenase, mitochondrial	IPI00025341	-1.266	0.02016	6	6	3	2
3397	Isoform 1 of Phosphatidylinositol-3,4,5-trisphosphate 5-phosphatase 2	IPI00016932	-1.266	0.02016	6	6	0	3
3398	Branched-chain-amino-acid aminotransferase	IPI00181135	-1.266	0.02016	8	4	3	2
3399	Replication protein A 70 kDa DNA-binding subunit	IPI00020127	-1.266	0.02016	12	11	9	6
3400	Calpain small subunit 1	IPI00025084	-1.285	0.01954	22	19	16	16
3401	Maleylacetoacetate isomerase	IPI00013809	-1.296	0.01954	7	4	0	0
3402	Mitochondrial fission 1 protein	IPI00007052	-1.296	0.01954	4	7	2	2
3403	Ras-related protein R-Ras	IPI00020418	-1.296	0.01954	5	6	2	2
3404	Syntaxin-6	IPI00013930	-1.296	0.01954	6	5	2	0

No.	Description	Accession number	STN[1]	p-Value[1]	480_A[2]	480_B[2]	620_A[2]	620_B[2]
3405	Ras-related protein Rab-3D	IPI00032808	-1.296	0.01954	6	5	0	0
3406	Tetratricopeptide repeat protein 1	IPI00016912	-1.296	0.01954	5	6	2	2
3407	Isoform 1 of FYVE and coiled-coil domain-containing protein 1	IPI00001580	-1.296	0.01954	4	7	0	0
3408	Isoform 1 of Transcription elongation factor SPT5	IPI00298058	-1.302	0.01940	10	10	7	5
3409	Isoform 1 of Proteasome subunit beta type-8	IPI00000783	-1.302	0.01940	8	12	7	5
3410	Tropomodulin-3	IPI00005087	-1.302	0.01940	9	11	6	6
3411	Isoform 1 of Elongation factor G, mitochondrial	IPI00154473	-1.302	0.01940	10	10	4	8
3412	Isoform 1 of Telomere-associated protein RIF1	IPI00293845	-1.303	0.01882	19	19	13	16
3413	cDNA FLJ75085, highly similar to Homo sapiens glutaminyl-tRNA synthetase (QARS), mRNA	IPI00026665	-1.303	0.01882	17	21	13	16
3414	Hypoxia up-regulated protein 1	IPI00000877	-1.309	0.01868	40	29	31	28
3415	Proteasome subunit beta type-3	IPI00028004	-1.315	0.01861	20	16	15	12
3416	Ras-related protein Rab-5C	IPI00016339	-1.315	0.01861	17	19	16	11
3417	Isoform 2 of Tumor protein D54	IPI00221178	-1.315	0.01861	22	14	16	11
3418	DNA excision repair protein ERCC-6-like	IPI00552569	-1.316	0.01861	10	9	4	7
3419	Isoform 2 of Calumenin	IPI00045396	-1.331	0.01837	10	8	5	5
3420	Serpin B6	IPI00413451	-1.344	0.01778	17	15	13	10
3421	Protein kinase, cAMP-dependent, regulatory, type II, alpha, isoform CRA_b	IPI00063234	-1.348	0.01778	11	6	6	3
3422	Carbonyl reductase [NADPH] 3	IPI00290462	-1.348	0.01778	8	9	4	5
3423	Synaptic vesicle membrane protein VAT-1 homolog	IPI00156689	-1.378	0.01696	13	15	5	14
3424	Astrocytic phosphoprotein PEA-15	IPI00014850	-1.387	0.01665	8	7	4	3
3425	Isoform 2 of Neuropathy target esterase	IPI00217600	-1.387	0.01665	8	7	2	5
3426	HLA class I histocompatibility antigen, B-7 alpha chain	IPI00004657	-1.387	0.01665	7	8	3	4
3427	Carbonyl reductase [NADPH] 1	IPI00295386	-1.387	0.01665	14	13	10	8
3428	Isoform 1 of 26S proteasome non-ATPase regulatory subunit 1	IPI00299608	-1.409	0.01593	43	36	29	39
3429	26S proteasome non-ATPase regulatory subunit 7	IPI00019927	-1.432	0.01486	18	23	17	14
3430	Isoform 1 of SAM domain and HD domain-containing protein 1	IPI00294739	-1.438	0.01476	6	7	2	3
3431	Isoform CNPI of 2',3'-cyclic-nucleotide 3'-phosphodiesterase	IPI00220993	-1.438	0.01476	8	5	2	3
3432	Isoform 1 of NADH-cytochrome b5 reductase 3	IPI00328415	-1.444	0.01455	21	18	15	14
3433	Dolichyl-diphosphooligosaccharide--protein glycosyltransferase subunit 2	IPI00028635	-1.446	0.01455	60	55	52	51
3434	Isoform 1 of RRP12-like protein	IPI00101186	-1.451	0.01455	18	20	13	15
3435	60S ribosomal protein L4	IPI00003918	-1.458	0.01434	18	19	13	14
3436	Nuclear pore complex protein Nup93	IPI00397904	-1.459	0.01434	9	12	5	7
3437	UPF0556 protein C19orf10	IPI00056357	-1.470	0.01414	7	5	2	2
3438	Poly [ADP-ribose] polymerase 4	IPI00296909	-1.470	0.01414	5	7	0	0
3439	Protein FAM83H	IPI00784320	-1.470	0.01414	6	6	0	0
3440	Isoform Beta of LIM domain and actin-binding protein 1	IPI00008918	-1.470	0.01414	6	6	0	0
3441	Isoform 2 of Rho guanine nucleotide exchange factor 1	IPI00339379	-1.470	0.01414	5	7	2	2
3442	TBC1 domain family member 4	IPI00220901	-1.470	0.01414	4	8	0	0
3443	Keratin, type II cytoskeletal 6B	IPI00293665	-1.470	0.01414	6	6	0	0
3444	Isoform 1 of UTP--glucose-1-phosphate uridylyltransferase	IPI00329331	-1.473	0.01317	18	17	14	11
3445	Isoform 1 of Nck-associated protein 1	IPI00031982	-1.509	0.01238	6	12	5	4
3446	Tyrosine-protein phosphatase non-receptor type 23	IPI00034006	-1.509	0.01238	8	10	4	5
3447	Omega-amidase NIT2	IPI00549467	-1.517	0.01235	16	14	8	12
3448	Coatomer subunit beta'	IPI00220219	-1.536	0.01194	37	43	32	36
3449	Isoform 1 of BH3-interacting domain death agonist	IPI00413587	-1.537	0.01194	11	17	11	7
3450	Protein flightless-1 homolog	IPI00031023	-1.537	0.01194	16	12	9	9
3451	DnaJ homolog subfamily C member 13	IPI00307259	-1.540	0.01166	21	26	15	21
3452	Periplakin	IPI00298057	-1.546	0.01166	22	24	17	18
3453	cDNA FLJ56420, highly similar to Aspartyl aminopeptidase	IPI00015856	-1.552	0.01166	8	8	5	0
3454	Isoform 1 of Opioid growth factor receptor	IPI00021537	-1.552	0.01166	8	8	3	4
3455	40S ribosomal protein S19	IPI00215780	-1.560	0.01166	14	12	8	8
3456	Isoform 1 of Protein unc-45 homolog A	IPI00072534	-1.560	0.01166	12	14	7	9
3457	Acyl-CoA dehydrogenase family member 9, mitochondrial	IPI00152981	-1.572	0.01128	13	12	7	8
3458	Isoform 3 of DNA repair protein RAD50	IPI00107531	-1.577	0.01121	6	9	4	2
3459	Profilin	IPI00107555	-1.577	0.01121	8	7	4	2
3460	Isoform 4 of Heterogeneous nuclear ribonucleoprotein A/B	IPI00106509	-1.579	0.01121	20	21	11	19
3461	Isoform 1 of 26S protease regulatory subunit 6B	IPI00020042	-1.635	0.01032	15	19	11	12
3462	Isoform 1 of La-related protein 1	IPI00185919	-1.635	0.01032	20	14	12	11
3463	Isoform 1 of Bifunctional coenzyme A synthase	IPI00184821	-1.643	0.01018	7	6	2	2
3464	Isoform Beta-1C of Integrin beta-1	IPI00217561	-1.643	0.01018	9	4	0	0
3465	PDZ and LIM domain protein 1	IPI00010414	-1.643	0.01018	8	5	0	2
3466	dehydrogenase/reductase SDR family member 2 isoform 2	IPI00218235	-1.643	0.01018	7	6	0	0
3467	Isoform Long of Laminin subunit gamma-2	IPI00015117	-1.643	0.01018	5	8	0	0
3468	Coactosin-like protein	IPI00017704	-1.650	0.01015	13	7	6	4
3469	ribonucleotide reductase M2 polypeptide isoform 1	IPI00011118	-1.650	0.01015	10	10	5	5
3470	Nucleolar pre-ribosomal-associated protein 1	IPI00297241	-1.654	0.00998	15	17	8	13
3471	septin-9 isoform e	IPI00455033	-1.654	0.00998	15	17	9	12
3472	cDNA FLJ56414, highly similar to Homo sapiens proline-, glutamic acid-, leucine-rich protein 1	IPI00006702	-1.669	0.00984	9	10	4	5
3473	Annexin A5	IPI00329801	-1.680	0.00973	39	37	32	31
3474	Signal recognition particle receptor subunit alpha	IPI00385267	-1.691	0.00967	9	9	5	3
3475	Tubulin beta-1 chain	IPI00006510	-1.697	0.00932	22	23	17	16
3476	Isoform 1 of Liprin-alpha-1	IPI00163496	-1.743	0.00870	5	11	2	4
3477	Isoform 1 of CAP-Gly domain-containing linker protein 1	IPI00013455	-1.743	0.00870	8	8	0	4
3478	ATP-citrate synthase	IPI00021290	-1.744	0.00836	50	45	42	39
3479	Isoform 6 of GTPase-activating protein and VPS9 domain-containing protein 1	IPI00292753	-1.754	0.00836	10	14	4	9
3480	Alpha-soluble NSF attachment protein	IPI00009253	-1.769	0.00819	20	16	12	12
3481	Isoform 1 of Fragile X mental retardation syndrome-related protein 1	IPI00016249	-1.770	0.00808	13	10	8	4
3482	51 kDa protein	IPI00033025	-1.788	0.00767	9	13	5	6
3483	87 kDa protein	IPI00220365	-1.800	0.00726	18	15	14	7
3484	Isoform 1 of Integrin alpha-V	IPI00027505	-1.814	0.00702	9	5	2	0
3485	ATP-dependent RNA helicase DDX24	IPI00006987	-1.814	0.00702	6	8	0	2
3486	Serpin B9	IPI00032139	-1.814	0.00702	7	7	0	0
3487	Nicotinamide phosphoribosyltransferase	IPI00018873	-1.828	0.00688	22	25	17	17
3488	Importin subunit alpha-4	IPI00012578	-1.828	0.00688	10	10	5	4
3489	Isoform 1 of Calcium-binding mitochondrial carrier protein SCaMC-1	IPI00337494	-1.878	0.00633	8	10	3	4
3490	Actin, cytoplasmic 1	IPI00021439	-1.884	0.00633	188	206	178	197
3491	Tumor protein, translationally-controlled 1	IPI00009943	-1.884	0.00633	20	20	15	12
3492	Isoform 1 of Putative ATP-dependent RNA helicase DHX30	IPI00411733	-1.890	0.00633	11	15	5	9
3493	Calpain-1 catalytic subunit	IPI00011285	-1.903	0.00626	19	19	11	14
3494	Keratin, type I cytoskeletal 14	IPI00384444	-1.907	0.00626	12	13	9	4
3495	Eukaryotic translation initiation factor 4A, isoform 2, isoform CRA_b	IPI00030296	-1.908	0.00626	7	10	0	4
3496	tropomyosin alpha-1 chain isoform 2	IPI00000230	-1.957	0.00588	17	16	9	11
3497	Tumor necrosis factor receptor superfamily member 16	IPI00027436	-1.984	0.00571	7	8	0	0
3498	Synaptobrevin homolog YKT6	IPI00008569	-1.984	0.00571	8	7	0	2
3499	Isoform 1 of PDZ and LIM domain protein 4	IPI00032206	-1.984	0.00571	9	6	0	0

No.	Description	Accession number	STN[1]	p-Value[1]	480_A[2]	480_B[2]	620_A[2]	620_B[2]
3500	Glycylpeptide N-tetradecanoyltransferase 2	IPI00030223	-1.984	0.00571	8	7	2	0
3501	Isoform 1 of Rab3 GTPase-activating protein non-catalytic subunit	IPI00554590	-1.984	0.00571	7	8	0	0
3502	Isoform 1 of Nesprin-3	IPI00394994	-1.984	0.00571	4	11	0	0
3503	Isoform 1 of Myosin-14	IPI00337335	-1.984	0.00571	6	9	0	0
3504	26S protease regulatory subunit 8	IPI00023919	-2.065	0.00519	24	30	19	20
3505	Putative high mobility group protein 1-like 10	IPI00018755	-2.066	0.00516	19	18	12	11
3506	Eukaryotic translation initiation factor 5A-2	IPI00006935	-2.071	0.00516	14	4	3	3
3507	Isoform 1 of Symplekin	IPI00023344	-2.078	0.00506	14	22	11	11
3508	Isoform 1 of Glycerol-3-phosphate dehydrogenase, mitochondrial	IPI00017895	-2.098	0.00499	12	12	5	6
3509	Gem-associated protein 5	IPI00291783	-2.115	0.00485	17	16	10	9
3510	Isoform 1 of A-kinase anchor protein 9	IPI00019223	-2.152	0.00475	6	10	0	0
3511	Isoform 1 of Epidermal growth factor receptor	IPI00018274	-2.152	0.00475	9	7	0	0
3512	Isoform IIb of Prolyl 4-hydroxylase subunit alpha-2	IPI00003128	-2.152	0.00475	7	9	0	0
3513	Isoform 1 of Protein diaphanous homolog 1	IPI00852685	-2.156	0.00471	14	29	15	13
3514	1-phosphatidylinositol-4,5-bisphosphate phosphodiesterase beta-3	IPI00010400	-2.171	0.00468	10	11	3	5
3515	Fructose-bisphosphate aldolase A	IPI00465439	-2.188	0.00457	42	38	35	28
3516	Isoform Long of Antigen KI-67	IPI00004233	-2.229	0.00454	34	38	29	26
3517	protein ALO17 isoform 1	IPI00828098	-2.275	0.00413	12	12	5	5
3518	GMP synthase [glutamine-hydrolyzing]	IPI00029079	-2.295	0.00392	24	20	16	12
3519	46 kDa protein	IPI00641706	-2.320	0.00371	8	9	0	0
3520	Abhydrolase domain-containing protein 10, mitochondrial	IPI00020075	-2.324	0.00371	16	14	9	6
3521	FAS-associated factor 2	IPI00172656	-2.328	0.00368	11	11	3	5
3522	Isoform 1 of Minor histocompatibility antigen H13	IPI00152441	-2.342	0.00354	21	8	8	6
3523	Chloride intracellular channel protein 4	IPI00001960	-2.360	0.00351	11	10	3	4
3524	Ras-related protein Rab-2A	IPI00031169	-2.391	0.00341	19	17	11	9
3525	CCAAT/enhancer-binding protein zeta	IPI00306723	-2.456	0.00306	13	11	5	4
3526	Isoform 2 of Heat shock protein HSP 90-alpha	IPI00382470	-2.459	0.00306	161	162	143	156
3527	26S protease regulatory subunit 6A	IPI00018398	-2.498	0.00303	34	38	29	24
3528	Isoform 1 of Catenin alpha-1	IPI00215948	-2.502	0.00296	26	27	21	14
3529	Dual specificity protein phosphatase 3	IPI00018671	-2.512	0.00296	15	14	7	6
3530	Procollagen-lysine,2-oxoglutarate 5-dioxygenase 3	IPI00030255	-2.519	0.00296	12	10	5	2
3531	Protein FAM3C	IPI00334282	-2.519	0.00296	12	10	4	3
3532	Isoform 2 of Protein disulfide-isomerase A6	IPI00299571	-2.584	0.00279	22	23	13	14
3533	Isoform 1 of Filamin-C	IPI00178352	-2.592	0.00279	26	33	22	18
3534	Isoform 2 of Calpastatin	IPI00220857	-2.611	0.00279	13	12	4	5
3535	Band 4.1-like protein 2	IPI00015973	-2.651	0.00272	10	9	2	2
3536	Protein ETHE1, mitochondrial	IPI00003766	-2.651	0.00272	13	6	2	2
3537	Isoform 2 of Plakophilin-2	IPI00005264	-2.651	0.00272	8	11	0	0
3538	Peptidyl-tRNA hydrolase 2, mitochondrial	IPI00032903	-2.736	0.00255	14	13	8	0
3539	Isoform 1 of Disks large homolog 1	IPI00030351	-2.765	0.00248	13	13	5	4
3540	Peroxiredoxin-6	IPI00220301	-2.775	0.00248	61	59	53	44
3541	Isoform 1 of Nuclear pore complex protein Nup214	IPI00183294	-2.797	0.00241	10	15	3	5
3542	Isoform 1 of LIM and SH3 domain protein 1	IPI00000861	-2.797	0.00241	11	14	5	3
3543	Niban-like protein 1	IPI00456750	-2.803	0.00241	25	27	16	16
3544	Uveal autoantigen with coiled-coil domains and ankyrin repeats	IPI00173359	-2.815	0.00241	9	11	0	0
3545	Isoform 2 of Pleckstrin homology-like domain family B member 2	IPI00168459	-2.815	0.00241	10	10	0	0
3546	Annexin A1	IPI00218918	-2.849	0.00230	26	22	16	12
3547	ADP-ribosylation factor 4	IPI00215918	-2.927	0.00206	27	27	17	16
3548	Serpin H1	IPI00032140	-2.947	0.00206	19	22	8	13
3549	Cystatin-SN	IPI00305477	-2.978	0.00200	11	10	0	0
3550	Isoform 1 of Adipocyte plasma membrane-associated protein	IPI00031131	-2.984	0.00200	16	15	6	6
3551	Isoform 2 of Microtubule-associated protein 4	IPI00220113	-2.986	0.00200	26	23	16	12
3552	Paired amphipathic helix protein Sin3a	IPI00170596	-3.010	0.00200	15	15	7	4
3553	TUBA1C protein	IPI00166768	-3.062	0.00182	95	80	68	80
3554	Isoform 1 of Atlastin-3	IPI00550523	-3.141	0.00182	10	12	0	0
3555	Intercellular adhesion molecule 1	IPI00008494	-3.141	0.00182	9	13	0	0
3556	Heat shock 70 kDa protein 1A/1B	IPI00304925	-3.187	0.00175	44	45	28	36
3557	Dehydrogenase/reductase SDR family member 7B	IPI00550165	-3.241	0.00175	11	13	2	3
3558	Isoform 1 of 182 kDa tankyrase-1-binding protein	IPI00304589	-3.241	0.00175	11	13	2	3
3559	Isoform 5 of Thioredoxin reductase 1, cytoplasmic	IPI00554786	-3.247	0.00175	19	23	8	12
3560	Isoform 3 of Keratin, type II cytoskeletal 80	IPI00375843	-3.254	0.00175	17	17	7	6
3561	Isoform 1 of Golgin subfamily A member 4	IPI00013272	-3.302	0.00158	11	12	0	0
3562	Retinoblastoma-associated protein	IPI00302829	-3.341	0.00151	14	17	4	6
3563	Isoform 1 of Insulin-like growth factor 2 mRNA-binding protein 2	IPI00179713	-3.346	0.00148	14	12	0	4
3564	Isoform 1 of Tryptophanyl-tRNA synthetase, cytoplasmic	IPI00295400	-3.356	0.00144	25	30	13	18
3565	26S protease regulatory subunit 4	IPI00011126	-3.369	0.00138	28	26	14	16
3566	26S protease regulatory subunit 7	IPI00021435	-3.408	0.00131	21	21	9	10
3567	Tubulin beta-2A chain	IPI00013475	-3.428	0.00131	19	22	10	8
3568	2-oxoglutarate dehydrogenase, mitochondrial	IPI00098902	-3.453	0.00124	31	28	18	16
3569	Isoform 1 of Mitochondrial inner membrane protein	IPI00009960	-3.478	0.00120	30	27	16	16
3570	145 kDa protein	IPI00218097	-3.502	0.00120	11	16	2	4
3571	ADP/ATP translocase 2	IPI00007188	-3.513	0.00120	152	160	131	147
3572	Solute carrier family 2, facilitated glucose transporter member 1	IPI00220194	-3.516	0.00120	31	35	24	16
3573	Isoform p150 of Dynactin subunit 1	IPI00029485	-3.547	0.00120	19	17	6	7
3574	Isoform 1 of Probable DNA dC->dU-editing enzyme APOBEC-3B	IPI00005531	-3.570	0.00100	23	19	8	10
3575	Creatine kinase B-type	IPI00022977	-3.623	0.00086	12	13	2	2
3576	Ubiquitin carboxyl-terminal hydrolase 11	IPI00184533	-3.640	0.00079	16	17	3	7
3577	Talin-1	IPI00298994	-3.643	0.00079	104	105	97	79
3578	Mannosyl-oligosaccharide glucosidase	IPI00328170	-3.711	0.00072	24	19	9	9
3579	22 kDa protein	IPI00219910	-3.733	0.00069	22	28	11	13
3580	Isoform A of Lamin-A/C	IPI00021405	-3.751	0.00069	75	72	62	53
3581	cDNA FLJ56425, highly similar to Very-long-chain specific acyl-CoAdehydrogenase, mitochondrial	IPI00028031	-3.815	0.00065	45	48	32	31
3582	Isoform 1 of Clathrin heavy chain 2	IPI00022881	-3.899	0.00065	71	70	59	49
3583	Isoform 1 of Dynamin-like 120 kDa protein, mitochondrial	IPI00006721	-4.027	0.00058	27	24	12	11
3584	Isoform 9 of Sorbin and SH3 domain-containing protein 1	IPI00002491	-4.100	0.00045	14	14	0	0
3585	Isoform 1 of Apoptosis-associated speck-like protein containing a CARD	IPI00001699	-4.257	0.00041	15	14	2	2
3586	Keratin, type I cytoskeletal 16	IPI00217963	-4.343	0.00041	24	27	8	13
3587	Isoform Long of Tight junction protein ZO-1	IPI00216219	-4.467	0.00038	13	23	3	5
3588	Isoform Beta of Heat shock protein 105 kDa	IPI00218993	-4.613	0.00031	48	45	27	30
3589	Myosin-Id	IPI00329719	-4.726	0.00024	14	18	2	0
3590	Isoform 2 of Liprin-beta-1	IPI00179172	-4.823	0.00024	32	26	14	10
3591	Microtubule-associated protein 1B	IPI00008868	-5.037	0.00024	20	14	0	0
3592	Ras-related protein Rab-3B	IPI00300562	-5.345	0.00024	18	18	0	0
3593	Aldehyde dehydrogenase family 1 member A3	IPI00026663	-5.394	0.00014	22	23	6	4
3594	cDNA FLJ45400 fis, clone BRHIP3028570	IPI00151888	-5.498	0.00014	19	18	2	2

No.	Description	Accession number	STN[1]	p-Value[1]	480_A[2]	480_B[2]	620_A[2]	620_B[2]
3595	Isoform 2 of Leucyl-cystinyl aminopeptidase	IPI00221240	-5.708	0.00014	16	24	2	3
3596	Isoform 2 of Myosin-Ic	IPI00010418	-5.724	0.00014	41	41	18	21
3597	Golgin subfamily B member 1	IPI00004671	-5.804	0.00014	16	23	0	0
3598	Keratin, type I cytoskeletal 9	IPI00019359	-6.337	0.00010	183	165	146	140
3599	Heat shock protein beta-1	IPI00025512	-6.373	0.00010	37	35	14	12
3600	Histone H2A type 1-B/E	IPI00026272	-6.410	0.00010	69	54	69	0
3601	Isoform 4 of E3 ubiquitin-protein ligase UBR4	IPI00640981	-6.453	0.00010	47	35	16	18
3602	Alpha-actinin-1	IPI00013508	-6.542	0.00010	235	267	214	220
3603	Isoform 1 of Gelsolin	IPI00026314	-6.710	0.00010	21	24	0	0
3604	Isoform 1 of Myosin-9	IPI00019502	-6.804	0.00007	332	332	290	300
3605	Keratin, type II cytoskeletal 75	IPI00005859	-6.949	0.00007	43	37	7	22
3606	26S proteasome non-ATPase regulatory subunit 2	IPI00012268	-7.201	0.00007	48	48	20	21
3607	Isoform 5 of E3 ubiquitin-protein ligase UBR4	IPI00180305	-7.232	0.00007	44	37	10	18
3608	Isoform 1 of Myb-binding protein 1A	IPI00005024	-7.923	0.00007	45	45	13	18
3609	retinol-binding protein 1 isoform a	IPI00219718	-8.086	0.00007	65	61	28	33
3610	Isoform 1 of Filamin-B	IPI00289334	-8.290	0.00007	220	220	178	178
3611	Keratin, type II cytoskeletal 2 epiderma	IPI00021304	-8.327	0.00007	146	134	110	92
3612	Isoform 1 of Keratin, type I cytoskeletal 13	IPI00009866	-8.416	0.00007	69	71	37	34
3613	Solute carrier family 2, facilitated glucose transporter member 3	IPI00003909	-8.487	0.00007	30	27	0	2
3614	Isoform 1 of Catenin beta-1	IPI00017292	-8.610	0.00007	59	60	23	28
3615	epiplakin	IPI00010951	-8.673	0.00007	44	42	13	10
3616	annexin A6 isoform 2	IPI00002459	-8.779	0.00007	32	27	0	0
3617	Isoform 5 of Myosin-14	IPI00029818	-8.779	0.00007	27	32	0	0
3618	Transferrin receptor protein 1	IPI00022462	-9.148	0.00007	48	48	13	15
3619	Isoform 1 of Clathrin heavy chain 1	IPI00024067	-9.500	0.00007	244	223	193	177
3620	Transitional endoplasmic reticulum ATPase	IPI00022774	-9.504	0.00007	97	89	52	52
3621	Isoform 1 of Protein phosphatase methylesterase 1	IPI00007694	-10.445	0.00007	45	41	7	6
3622	A-kinase anchor protein 12 isoform 2	IPI00217683	-10.532	0.00007	50	41	6	10
3623	Keratin, type II cytoskeletal 5	IPI00009867	-11.485	0.00007	54	50	6	14
3624	Isoform 2 of Annexin A2	IPI00418169	-12.208	0.00007	66	61	18	15
3625	Isoform 2 of Microtubule-actin cross-linking factor 1, isoforms 1/2/3/5	IPI00256861	-12.988	0.00007	57	50	4	10
3626	Isoform 1 of Ras GTPase-activating-like protein IQGAP2	IPI00299048	-14.990	0.00007	53	50	0	0
3627	Keratin, type I cytoskeletal 10	IPI00009865	-16.640	0.00003	315	302	226	215
3628	Isoform 2 of Protein-glutamine gamma-glutamyltransferase 2	IPI00218251	-17.072	0.00003	61	69	4	5
3629	Keratin, type II cytoskeletal 8	IPI00554648	-17.206	0.00003	517	519	421	416
3630	Isoform 1 of Protein-glutamine gamma-glutamyltransferase 2	IPI00294578	-18.989	0.00003	82	67	6	5
3631	Putative annexin A2-like protein	IPI00334627	-19.214	0.00003	143	159	69	58
3632	Keratin, type II cytoskeletal 1	IPI00220327	-28.337	0.00003	488	484	336	316
3633	Keratin, type I cytoskeletal 18	IPI00554788	-41.439	0.00000	522	711	326	425
3634	Neuroblast differentiation-associated protein AHNAK	IPI00021812	-51.604	0.00000	428	402	130	158
