# Supplementary material for: Data supporting the identification of anti-metastatic drug and natural compound targets in isogenic colorectal cancer cells
Source: Data Brief. 2014 Nov 4;1:73–5. doi: 10.1016/j.dib.2014.10.005 (PMC4459770; doi:10.1016/j.dib.2014.10.005)
Supplement: Supplementary file 1 — Supplementary data [file mmc1.zip › CRC_Metastasis_DIB_Table02.pdf]

Table 2. List of proteins differentially expressed in SW620 by the treatment of oxaliplatin for 48hr. ([1]STN and p-value were acquired from PLGEM analysis, [2]Raw spectral counts from data compilation using ScaffoldTM software)

No.	Description	Accession number	STN[1]	p-Value[1]	Con_A[2]	Con_B[2]	OXA_A[2]	OXA_B[2]
1	Keratin, type I cytoskeletal 18	IPI00554788	-8.011	0.00008	550	610	412	420
2	Glyceraldehyde-3-phosphate dehydrogenase	IPI00219018	-3.829	0.00062	438	494	406	379
3	60 kDa heat shock protein, mitochondrial	IPI00784154	-5.314	0.00008	410	438	332	324
4	Keratin, type II cytoskeletal 1	IPI00220327	-0.192	0.32867	383	388	419	345
5	Cytoplasmic dynein 1 heavy chain 1	IPI00456969	1.100	0.03536	236	243	312	200
6	Keratin, type II cytoskeletal 8	IPI00554648	-6.627	0.00008	391	407	307	262
7	Isoform 1 of Myosin-9	IPI00019502	-3.922	0.00062	338	347	301	254
8	Elongation factor 1-alpha 2	IPI00014424	0.944	0.05163	286	222	292	245
9	Isoform 1 of Plectin-1	IPI00014898	-4.230	0.00012	281	281	241	194
10	Isoform 1 of DNA-dependent protein kinase catalytic subunit	IPI00296337	1.035	0.03929	230	214	272	202
11	Isoform B1 of Heterogeneous nuclear ribonucleoproteins A2/B1	IPI00396378	-5.669	0.00008	334	375	252	270
12	Isoform alpha-enolase of Alpha-enolase	IPI00465248	-3.612	0.00062	311	372	259	304
13	Isoform M1 of Pyruvate kinase isozymes M1/M2	IPI00220644	-3.769	0.00062	273	280	240	200
14	Histone H4	IPI00453473	-8.221	0.00008	286	166	189	55
15	Pre-mRNA-processing-splicing factor 8	IPI00007928	-2.849	0.00087	160	163	157	99
16	Fatty acid synthase	IPI00026781	-3.793	0.00062	193	188	156	130
17	Actin, cytoplasmic 1	IPI00021439	-3.657	0.00062	192	235	155	175
18	Isoform 1 of L-lactate dehydrogenase A chain	IPI00217966	-3.677	0.00062	198	198	179	123
19	Keratin, type I cytoskeletal 19	IPI00479145	-1.552	0.01246	198	196	175	178
20	Isoform 1 of Clathrin heavy chain 1	IPI00024067	-2.673	0.00095	174	170	156	123
21	Isoform 2 of Filamin-A	IPI00302592	-0.163	0.34440	162	158	163	153
22	Tubulin alpha-4A chain	IPI00007750	-1.404	0.01536	158	188	150	161
23	Keratin, type I cytoskeletal 9	IPI00019359	-0.839	0.06335	131	113	125	101
24	Isoform 1 of U5 small nuclear ribonucleoprotein 200 kDa helicase	IPI00420014	-2.435	0.00145	147	145	138	99
25	32 kDa protein	IPI00176692	-1.263	0.02083	131	157	124	135
26	ADP/ATP translocase 2	IPI00007188	2.087	0.00240	99	117	147	114
27	ATP-dependent RNA helicase A	IPI00844578	-1.535	0.01321	148	139	147	105
28	Isoform 2 of Heat shock protein HSP 90-alpha	IPI00382470	-4.740	0.00008	158	180	129	99
29	14-3-3 protein epsilon	IPI00000816	-3.004	0.00087	164	174	154	112
30	Short heat shock protein 60 Hsp60s2	IPI00076042	-3.996	0.00041	133	149	113	83
31	X-ray repair cross-complementing protein 5	IPI00220834	-1.735	0.00716	125	126	105	109
32	Tubulin beta-2C chain	IPI00007752	-5.953	0.00008	173	175	130	81
33	Isoform 1 of Heat shock cognate 71 kDa protein	IPI00003865	-6.043	0.00008	161	183	109	97
34	Isoform 3 of Spectrin alpha chain, brain	IPI00843765	-0.877	0.05482	120	102	116	88
35	Leucine-rich PPR motif-containing protein, mitochondrial	IPI00783271	-3.026	0.00087	154	142	128	100
36	Alpha-actinin-1	IPI00013508	-4.089	0.00025	135	148	103	92
37	Isoform 1 of Heterogeneous nuclear ribonucleoprotein K	IPI00216049	-4.397	0.00012	174	166	134	103
38	Translational activator GCN1	IPI00001159	-3.076	0.00083	116	121	100	75
39	Keratin, type I cytoskeletal 10	IPI00009865	0.160	0.34697	88	84	105	70
40	L-lactate dehydrogenase B chain	IPI00219217	-3.886	0.00062	147	143	120	85
41	Histone H2B type 2-E	IPI00003935	-4.536	0.00008	133	146	100	83
42	Isoform Long of Spectrin beta chain, brain 1	IPI00005614	-1.413	0.01536	125	120	105	110
43	X-ray repair cross-complementing protein 6	IPI00644712	-4.278	0.00012	126	123	89	74
44	Elongation factor 1-alpha	IPI00025447	-1.415	0.01536	92	103	96	72
45	Heat shock protein HSP 90-beta	IPI00414676	-4.724	0.00008	125	124	90	65
46	Elongation factor 2	IPI00186290	-3.081	0.00083	96	103	75	67
47	Endoplasmin	IPI00027230	-4.119	0.00025	103	111	66	71
48	Profilin-1	IPI00216691	-4.206	0.00025	131	114	99	62
49	ATP synthase subunit beta, mitochondrial	IPI00303476	-2.963	0.00087	112	110	80	84
50	482 kDa protein	IPI00179298	-2.929	0.00087	86	75	77	35
51	Isoform 2 of Neutral alpha-glucosidase AB	IPI00011454	-0.572	0.12674	79	75	79	65
52	Glutathione S-transferase P	IPI00219757	-2.943	0.00087	112	113	90	77
53	Complement component 1 Q subcomponent-binding protein, mitochondrial	IPI00014230	-4.269	0.00012	120	102	80	61
54	Talin-1	IPI00298994	0.000	0.37049	81	80	86	75
55	Isoform 1 of Polypyrimidine tract-binding protein 1	IPI00179964	-1.949	0.00406	66	71	56	50
56	Isoform 1 of Exportin-2	IPI00022744	-1.282	0.02058	96	90	80	82
57	Cofilin-1	IPI00012011	-2.145	0.00219	73	82	71	48
58	Isoform 2 of Nucleophosmin	IPI00220740	-5.576	0.00008	124	126	70	71
59	Isoform 1 of Filamin-B	IPI00289334	-0.504	0.16024	83	78	80	72
60	Isoform 1 of Splicing factor 3B subunit 3	IPI00300371	1.743	0.00650	74	88	103	92
61	Ras GTPase-activating-like protein IQGAP1	IPI00009342	-0.055	0.36854	83	80	87	75
62	CAD protein	IPI00301263	-0.179	0.33596	72	65	77	57
63	Phosphoglycerate kinase 1	IPI00169383	-2.899	0.00087	84	94	64	63
64	Isoform 1 of Heterogeneous nuclear ribonucleoprotein M	IPI00171903	-2.410	0.00145	95	101	73	78
65	Isoform Short of Heterogeneous nuclear ribonucleoprotein U	IPI00479217	-2.798	0.00091	95	103	81	65
66	Non-POU domain-containing octamer-binding protein	IPI00304596	-2.359	0.00145	102	112	95	73
67	Neuroblast differentiation-associated protein AHNAK	IPI00021812	2.972	0.00070	41	28	61	49
68	FACT complex subunit SPT16	IPI00026970	-0.633	0.11498	74	79	88	54
69	Isoform 1 of Chromodomain-helicase-DNA-binding protein 4	IPI00000846	-2.452	0.00132	57	70	54	36
70	Dolichyl-diphosphooligosaccharide--protein glycosyltransferase subunit 2	IPI00028635	-0.763	0.07569	63	61	67	45
71	Isoform A1-B of Heterogeneous nuclear ribonucleoprotein A1	IPI00215965	-1.382	0.01805	89	100	81	82
72	NCL protein	IPI00183526	-3.994	0.00041	100	109	68	67
73	cDNA FLJ40024 fis, clone STOMA2007745, highly similar to UBIQUITIN-ACTIVATING ENZYME	IPI00026119	-1.986	0.00393	84	86	74	61
74	Plastin-2	IPI00010471	-3.953	0.00050	77	98	63	45
75	Alanyl-tRNA synthetase, cytoplasmic	IPI00027442	-5.334	0.00008	106	101	54	58
76	T-complex protein 1 subunit zeta	IPI00027626	-2.590	0.00095	81	82	61	58
77	Eukaryotic initiation factor 4A-I	IPI00025491	-3.165	0.00083	104	99	76	68
78	Alpha-actinin-4	IPI00013808	-2.433	0.00145	85	82	64	61
79	Exportin-1	IPI00298961	0.434	0.18843	57	64	61	67
80	Stress-70 protein, mitochondrial	IPI00007765	-4.570	0.00008	95	107	59	61
81	Isoform 1 of Myosin-10	IPI00397526	0.623	0.12351	77	70	83	75
82	Actin, aortic smooth muscle	IPI00008603	-2.713	0.00095	66	70	48	46
83	Poly [ADP-ribose] polymerase 1	IPI00449049	-1.303	0.02025	71	51	50	52
84	Splicing factor 3B subunit 1	IPI00026089	0.188	0.33475	62	58	62	61
85	Isoform C1 of Heterogeneous nuclear ribonucleoproteins C1/C2	IPI00216592	-2.922	0.00087	94	103	67	76
86	Isoform 1 of Clathrin heavy chain 2	IPI00022881	-0.428	0.18789	60	72	70	55
87	HSPA5 protein	IPI00003362	-5.080	0.00008	104	107	65	54
88	Beta-actin-like protein 2	IPI00003269	-2.664	0.00095	73	61	53	40
89	Isoform M2 of Pyruvate kinase isozymes M1/M2	IPI00479186	2.135	0.00219	47	58	57	82
90	Keratin, type II cytoskeletal 2 epiderma	IPI00021304	1.743	0.00650	52	49	77	51
91	Gamma-enolase	IPI00216171	-0.113	0.36225	73	82	72	81

No.	Description	Accession number	STN[1]	p-Value[1]	Con_A[2]	Con_B[2]	OXA_A[2]	OXA_B[2]
92	Putative heat shock protein HSP 90-alpha A2	IPI00031523	-0.698	0.08529	66	58	57	56
93	Isoform DPI of Desmoplakin	IPI00013933	1.288	0.02033	44	49	63	49
94	T-complex protein 1 subunit eta	IPI00018465	-2.636	0.00095	82	83	64	56
95	Isoform 1 of Vinculin	IPI00291175	-0.183	0.33256	65	66	68	60
96	NAD(P) transhydrogenase, mitochondrial	IPI00337541	1.251	0.02153	44	44	65	41
97	Isoform Long of Inositol 1,4,5-trisphosphate receptor type 2	IPI00031545	2.863	0.00070	27	28	65	26
98	cDNA FLJ56389, highly similar to Elongation factor 1-gamma	IPI00000875	-1.179	0.03143	51	55	48	41
99	Heterogeneous nuclear ribonucleoprotein L	IPI00027834	-1.589	0.01172	65	66	59	47
100	ATP synthase subunit alpha, mitochondrial	IPI00440493	-2.442	0.00145	89	85	66	65
101	Isoform A of Lamin-A/C	IPI00021405	-0.622	0.12032	67	62	57	62
102	Prohibitin	IPI00017334	-2.374	0.00145	60	47	44	30
103	40S ribosomal protein S3	IPI00011253	-4.530	0.00008	87	77	49	42
104	DNA damage-binding protein 1	IPI00293464	0.745	0.07850	41	37	54	34
105	Transitional endoplasmic reticulum ATPase	IPI00022774	-3.358	0.00083	68	79	48	46
106	Isoform 2 of U5 small nuclear ribonucleoprotein 200 kDa helicase	IPI00168235	-0.905	0.05325	54	49	49	41
107	ADP/ATP translocase 3	IPI00291467	-0.213	0.31650	47	47	57	34
108	ubiquitin and ribosomal protein S27a precursor	IPI00179330	-0.210	0.31662	48	49	57	37
109	NAD(P)H dehydrogenase [quinone] 1	IPI00012069	-1.078	0.03586	58	54	55	41
110	Isoform 1 of Transcription intermediary factor 1-beta	IPI00438229	-3.068	0.00087	74	68	54	40
111	Neutral amino acid transporter B(0)	IPI00019472	-2.573	0.00095	49	61	48	26
112	Staphylococcal nuclease domain-containing protein 1	IPI00140420	-2.693	0.00095	65	60	42	43
113	Malate dehydrogenase, mitochondrial	IPI00291006	-2.346	0.00149	72	66	55	46
114	Isoform 1 of Cullin-associated NEDD8-dissociated protein 1	IPI00100160	-0.072	0.36767	46	44	45	44
115	Inorganic pyrophosphatase	IPI00015018	-1.752	0.00654	51	57	50	33
116	T-complex protein 1 subunit beta	IPI00297779	-1.300	0.02029	64	72	53	62
117	Isoform 1 of Coatomer subunit alpha	IPI00295857	-0.942	0.05060	39	41	36	32
118	TUBA1C protein	IPI00166768	-2.664	0.00095	65	69	53	40
119	cDNA FLJ54957, highly similar to Transketolase	IPI00643920	-2.898	0.00087	53	62	40	34
120	T-complex protein 1 subunit alpha	IPI00290566	-2.792	0.00091	61	68	45	42
121	Heterogeneous nuclear ribonucleoprotein F	IPI00003881	-2.499	0.00108	50	66	41	39
122	Isoform 1 of Keratin, type I cytoskeletal 13	IPI00009866	-1.158	0.03167	59	51	48	45
123	Importin-7	IPI00007402	0.076	0.36809	45	34	43	37
124	Protein disulfide-isomerase A4	IPI00009904	-1.092	0.03519	58	51	48	45
125	Matrin-3	IPI00017297	-0.471	0.16818	38	39	31	40
126	Ubiquitin-like modifier-activating enzyme 1	IPI00645078	-3.308	0.00083	71	80	48	50
127	HEAT repeat-containing protein 1	IPI00024279	-2.739	0.00091	47	46	36	22
128	Serine hydroxymethyltransferase, mitochondrial	IPI00002520	-2.466	0.00108	47	59	33	39
129	Bifunctional aminoacyl-tRNA synthetase	IPI00013452	-3.610	0.00062	67	58	40	33
130	Isoform 1 of Heterogeneous nuclear ribonucleoprotein R	IPI00012074	-3.362	0.00083	51	65	32	37
131	T-complex protein 1 subunit gamma isoform b	IPI00290770	-1.677	0.00816	46	53	39	37
132	Tubulin, beta	IPI00645452	-0.379	0.20748	60	63	61	56
133	Voltage-dependent anion-selective channel protein 1	IPI00216308	-1.677	0.00816	49	50	43	33
134	Importin subunit beta-1	IPI00001639	-0.850	0.06161	48	51	42	45
135	cDNA FLJ55574, highly similar to Calnexin	IPI00020984	-3.075	0.00083	56	63	38	37
136	Heat shock protein 75 kDa, mitochondrial	IPI00030275	-1.761	0.00654	56	60	46	44
137	Isoform 1 of Lipopolysaccharide-responsive and beige-like anchor protein	IPI00002255	3.034	0.00070	22	18	52	22
138	Structural maintenance of chromosomes protein 3	IPI00219420	3.563	0.00037	16	39	45	56
139	Putative annexin A2-like protein	IPI00334627	-2.161	0.00219	45	40	34	24
140	Lamin-B1	IPI00217975	-1.030	0.03954	53	54	49	43
141	Protein disulfide-isomerase A3	IPI00025252	-4.421	0.00012	71	62	39	30
142	Peptidyl-prolyl cis-trans isomerase A	IPI00419585	-1.213	0.02339	57	43	53	30
143	Keratin-8-like protein 1	IPI00017870	-2.284	0.00178	62	53	44	38
144	116 kDa U5 small nuclear ribonucleoprotein component	IPI00003519	0.813	0.06662	38	41	48	42
145	Eukaryotic translation initiation factor 3 subunit A	IPI00029012	0.384	0.20831	36	39	42	38
146	Vimentin	IPI00418471	0.343	0.24400	52	45	60	42
147	Hypoxia up-regulated protein 1	IPI00000877	-1.373	0.01814	36	52	33	37
148	Isoform 3 of DNA topoisomerase 2-alpha	IPI00218753	5.944	0.00004	16	15	60	40
149	Interleukin enhancer-binding factor 2	IPI00005198	-2.428	0.00145	54	55	39	36
150	Nucleoprotein TPR	IPI00742682	-2.252	0.00199	55	49	41	32
151	Protein disulfide-isomerase	IPI00010796	-2.151	0.00219	48	51	35	35
152	cDNA FLJ45706 fis, clone FEBRA2028457, highly similar to Nucleolin	IPI00444262	-3.916	0.00062	72	67	40	40
153	Prohibitin-2	IPI00027252	-3.367	0.00083	59	52	38	27
154	Glucose-6-phosphate isomerase	IPI00027497	-2.612	0.00095	49	58	38	33
155	Isoform Long of Sodium/potassium-transporting ATPase subunit alpha-1	IPI00006482	-0.578	0.12599	45	48	50	35
156	Isoform 1 of Heterogeneous nuclear ribonucleoprotein Q	IPI00018140	-1.823	0.00530	48	52	40	35
157	Galectin-1	IPI00219219	-0.972	0.04612	45	44	50	26
158	Gamma-glutamyl hydrolase	IPI00023728	-0.632	0.11506	44	33	37	32
159	6-phosphogluconate dehydrogenase, decarboxylating	IPI00219525	-0.210	0.31662	44	53	43	51
160	60S ribosomal protein L3	IPI00550021	-2.376	0.00145	40	37	31	18
161	Peroxiredoxin-6	IPI00220301	-1.212	0.02343	45	32	30	32
162	Small nuclear ribonucleoprotein Sm D1	IPI00302850	-4.424	0.00012	62	67	40	26
163	Isoleucyl-tRNA synthetase, cytoplasmic	IPI00644127	-2.500	0.00108	44	42	23	32
164	Heat shock 70 kDa protein 4	IPI00002966	-2.443	0.00132	60	61	41	44
165	Aspartate aminotransferase, mitochondrial	IPI00018206	-2.212	0.00207	47	47	34	31
166	Valyl-tRNA synthetase	IPI00000873	-1.763	0.00654	40	42	31	29
167	Isoform 1 of Serine/arginine repetitive matrix protein 2	IPI00782992	0.091	0.36552	24	29	30	24
168	ADP-ribosylation factor 1	IPI00215914	-1.811	0.00530	45	33	33	23
169	Nuclear pore complex protein Nup205	IPI00783781	-0.083	0.36680	32	34	37	28
170	Heterogeneous nuclear ribonucleoprotein C-like 1	IPI00027569	-2.053	0.00368	53	27	41	14
171	14-3-3 protein zeta/delta	IPI00021263	-1.782	0.00571	46	42	30	35
172	Isoform 1 of Myoferlin	IPI00021048	0.548	0.13365	39	32	47	31
173	Isoform 1 of Nucleoside diphosphate kinase A	IPI00012048	-1.052	0.03639	36	28	28	24
174	Calreticulin	IPI00020599	-1.099	0.03507	39	42	29	38
175	Isoform 1 of 3-hydroxyacyl-CoA dehydrogenase type-2	IPI00017726	-1.515	0.01342	46	54	39	40
176	Isoform 5 of Interleukin enhancer-binding factor 3	IPI00219330	-0.696	0.08633	45	36	35	37
177	Isoform 1 of Nucleolar RNA helicase 2	IPI00015953	-4.217	0.00025	58	50	33	20
178	Probable ATP-dependent RNA helicase DDX5	IPI00017617	-1.120	0.03453	40	38	33	31
179	Isoform 1 of Nuclear pore membrane glycoprotein 210	IPI00291755	-1.815	0.00530	40	45	39	23
180	C-1-tetrahydrofolate synthase, cytoplasmic	IPI00218342	-0.371	0.20831	43	43	38	43
181	Heterogeneous nuclear ribonucleoprotein H	IPI00013881	-0.778	0.06923	19	31	17	25
182	Isoform A of Phosphate carrier protein, mitochondrial	IPI00022202	2.100	0.00240	23	19	40	25
183	Isoform 1 of Polyadenylate-binding protein 1	IPI00008524	-1.182	0.03143	37	44	38	28
184	T-complex protein 1 subunit epsilon	IPI00010720	-1.761	0.00654	41	49	32	35
185	Moesin	IPI00219365	-2.244	0.00199	53	45	32	36
186	Isoform 1 of Protein SET	IPI00072377	-2.106	0.00248	51	52	39	35

No.	Description	Accession number	STN[1]	p-Value[1]	Con_A[2]	Con_B[2]	OXA_A[2]	OXA_B[2]
187	cDNA FLJ60299, highly similar to Rab GDP dissociation inhibitor beta	IPI00031461	-2.525	0.00099	44	46	32	26
188	Isoform 1 of Extended synaptotagmin-1	IPI00022143	1.787	0.00518	27	31	39	41
189	Isoform 2 of Eukaryotic translation initiation factor 5A-1	IPI00376005	-0.085	0.36655	24	39	35	27
190	poly(rC) binding protein 2 isoform b	IPI00012066	-1.110	0.03482	32	36	28	27
191	Isoform Long of Splicing factor, proline- and glutamine-rich	IPI00010740	-0.968	0.04757	32	31	24	28
192	Isoform 2 of Nuclear mitotic apparatus protein 1	IPI00006196	-1.415	0.01536	40	43	34	31
193	Isoform 1 of UDP-glucose:glycoprotein glucosyltransferase 1	IPI00024466	-1.974	0.00393	43	43	34	27
194	Glycyl-tRNA synthetase	IPI00783097	-2.955	0.00087	34	39	19	21
195	Trifunctional enzyme subunit alpha, mitochondrial	IPI00031522	0.249	0.29790	32	32	35	32
196	T-complex protein 1 subunit delta	IPI00302927	-3.563	0.00070	49	52	27	28
197	Lupus La protein	IPI00009032	-0.079	0.36717	35	39	35	38
198	Citrate synthase, mitochondrial	IPI00025366	-0.356	0.22888	31	27	26	28
199	Isoform ASF-1 of Splicing factor, arginine/serine-rich 1	IPI00215884	-0.169	0.34179	36	28	38	24
200	40S ribosomal protein S3a	IPI00419880	-3.961	0.00050	39	50	27	15
201	60S ribosomal protein L5	IPI00000494	-2.132	0.00232	33	25	21	15
202	Isoform 1 of Importin-5	IPI00793443	2.394	0.00120	20	28	36	40
203	Isoform 2 of Titin	IPI00023283	-1.664	0.01068	4	9	1	4
204	Adenosylhomocysteinase	IPI00012007	-3.568	0.00062	53	52	28	30
205	Isoform Long of Delta-1-pyrroline-5-carboxylate synthase	IPI00008982	-1.365	0.01814	44	45	36	35
206	59 kDa protein	IPI00302925	-2.505	0.00108	52	45	33	31
207	Nucleolar protein 56	IPI00411937	-0.992	0.04128	31	41	34	26
208	Isoform 1 of Catenin beta-1	IPI00017292	0.927	0.05205	18	18	24	21
209	Leucyl-tRNA synthetase, cytoplasmic	IPI00103994	-3.709	0.00062	42	38	17	21
210	Isoform 1 of 26S proteasome non-ATPase regulatory subunit 1	IPI00299608	-1.036	0.03946	34	32	25	29
211	Isoform 1 of Heterogeneous nuclear ribonucleoprotein H3	IPI00013877	-1.172	0.03147	29	32	24	24
212	Isoform Cytoplasmic of Lysyl-tRNA synthetase	IPI00014238	-1.127	0.03246	33	33	22	31
213	Delta(3,5)-Delta(2,4)-dienoyl-CoA isomerase, mitochondrial	IPI00011416	-1.470	0.01391	37	40	34	25
214	Coatomer subunit beta'	IPI00220219	-0.991	0.04132	29	31	30	19
215	Chromobox protein homolog 1	IPI00010320	-1.297	0.02029	35	42	38	23
216	Bifunctional purine biosynthesis protein PURH	IPI00289499	-0.087	0.36618	27	33	32	27
217	Laminin receptor-like protein LAMRL5	IPI00411639	-1.471	0.01391	49	37	33	34
218	Radixin, isoform CRA_a	IPI00017367	-1.110	0.03482	31	37	28	27
219	Putative pre-mRNA-splicing factor ATP-dependent RNA helicase DHX15	IPI00396435	-1.490	0.01354	36	39	27	30
220	Nucleolar protein 58	IPI00006379	-0.330	0.23870	32	36	36	28
221	Guanine nucleotide-binding protein subunit beta-2-like 1	IPI00848226	-1.247	0.02170	37	26	28	21
222	Proliferation-associated protein 2G4	IPI00299000	-0.898	0.05325	26	22	23	16
223	Annexin A3	IPI00024095	-2.718	0.00095	45	39	25	26
224	Isoform 1 of Apoptosis-inducing factor 1, mitochondrial	IPI00000690	-0.666	0.11121	28	41	32	29
225	Calcium-binding mitochondrial carrier protein Aralar2	IPI00007084	-0.287	0.27099	21	28	28	18
226	SUMO-activating enzyme subunit 2	IPI00023234	1.127	0.03312	22	31	33	33
227	Serpin H1	IPI00032140	0.420	0.19125	29	32	33	33
228	Electron transfer flavoprotein subunit alpha, mitochondrial	IPI00010810	-1.786	0.00571	32	34	24	22
229	vacuolar protein sorting-associated protein 13C isoform 2B	IPI00412216	6.967	0.00004	2	3	41	17
230	60S ribosomal protein L6	IPI00329389	-3.124	0.00083	28	28	17	9
231	Keratin, type II cytoskeletal 75	IPI00005859	0.121	0.36076	17	11	21	8
232	Annexin A5	IPI00329801	-1.191	0.03143	35	34	34	21
233	ATP-dependent RNA helicase DDX1	IPI00293655	-0.883	0.05441	27	35	27	25
234	Glutamate dehydrogenase 1, mitochondrial	IPI00016801	0.088	0.36643	28	29	27	31
235	60S acidic ribosomal protein P0	IPI00008530	-4.089	0.00025	41	54	21	24
236	Isoform 3 of Core histone macro-H2A.1	IPI00059366	-2.430	0.00145	34	40	24	22
237	Dolichyl-diphosphooligosaccharide--protein glycosyltransferase subunit 1 precursor	IPI00025874	-1.498	0.01354	27	39	27	22
238	Peptidyl-prolyl cis-trans isomerase FKBP4	IPI00219005	-1.557	0.01238	38	39	33	25
239	Aspartyl-tRNA synthetase, cytoplasmic	IPI00216951	-3.344	0.00083	35	32	17	15
240	Villin-1	IPI00218852	-1.637	0.01093	39	31	22	29
241	Isoform 1 of General transcription factor II-I	IPI00054042	0.337	0.24437	31	30	36	29
242	ATP-dependent RNA helicase DDX3X	IPI00215637	-1.537	0.01321	36	43	28	32
243	Acetyl-CoA acetyltransferase, mitochondrial	IPI00030363	-2.460	0.00132	37	46	27	26
244	Dihydropyrimidinase-like 2	IPI00106642	0.968	0.04749	25	27	34	29
245	Puromycin-sensitive aminopeptidase	IPI00026216	-0.768	0.07395	34	32	30	27
246	Stress-induced-phosphoprotein 1	IPI00013894	-2.285	0.00178	34	32	20	21
247	Peroxiredoxin-1	IPI00000874	-1.853	0.00497	34	34	23	24
248	cDNA FLJ25678 fis, clone TST04067, highly similar to PURINE NUCLEOSIDE PHOSPHORYLASE	IPI00017672	-1.905	0.00414	34	37	30	19
249	Lon protease homolog, mitochondrial	IPI00005158	-4.084	0.00025	44	41	21	17
250	Tripeptidyl-peptidase 2	IPI00020416	0.946	0.05130	22	22	29	25
251	Isoform Beta-2 of DNA topoisomerase 2-beta	IPI00027280	-1.260	0.02157	22	31	23	17
252	Heat shock protein beta-1	IPI00025512	0.083	0.36734	37	27	35	30
253	ATP-citrate synthase	IPI00021290	2.471	0.00099	18	22	29	38
254	Threonyl-tRNA synthetase, cytoplasmic	IPI00329633	-3.143	0.00083	41	41	24	21
255	Putative uncharacterized protein SPTAN1	IPI00745092	-1.325	0.02016	25	31	23	19
256	Coatomer subunit beta	IPI00295851	-1.377	0.01809	29	31	24	21
257	Isoform 4 of E3 ubiquitin-protein ligase UBR4	IPI00640981	1.887	0.00426	8	11	25	9
258	Proliferating cell nuclear antigen	IPI00021700	0.538	0.13746	25	27	33	25
259	Vacuolar protein sorting-associated protein 35	IPI00018931	-1.426	0.01524	33	31	25	23
260	N-acetyltransferase 10	IPI00300127	-3.192	0.00083	33	28	14	15
261	Isoform 1 of Nucleoside diphosphate kinase B	IPI00026260	-1.260	0.02157	28	25	22	18
262	Ubiquitin carboxyl-terminal hydrolase 7	IPI00003965	1.357	0.01818	15	18	21	25
263	Fructose-bisphosphate aldolase A	IPI00465439	-0.905	0.05325	30	29	24	25
264	Isoform 1 of Carnitine O-palmitoyltransferase 1, liver isoform	IPI00032038	-0.104	0.36452	18	22	26	13
265	40S ribosomal protein S15	IPI00479058	-1.192	0.03126	31	28	29	17
266	Heterogeneous nuclear ribonucleoprotein U-like protein 2	IPI00456887	-0.625	0.12011	29	30	25	27
267	Isoform 1 of Heterogeneous nuclear ribonucleoprotein A3	IPI00419373	-3.167	0.00083	39	42	22	22
268	Isoform 2 of Triosephosphate isomerase	IPI00451401	-3.706	0.00062	49	42	24	22
269	Histone H2A.V	IPI00018278	-2.242	0.00199	36	27	23	16
270	cDNA FLJ56425, highly similar to Very-long-chain specific acyl-CoAdehydrogenase	IPI00028031	-0.356	0.22888	28	30	31	23
271	40S ribosomal protein S2	IPI00013485	-3.399	0.00079	31	31	18	10
272	cysteinyl-tRNA synthetase, cytoplasmic isoform c	IPI00027443	-2.165	0.00219	32	35	24	19
273	Rho GDP-dissociation inhibitor 1	IPI00003815	-0.913	0.05316	29	29	24	24
274	Heat shock 70 kDa protein 1A/1B	IPI00304925	-1.291	0.02029	31	37	30	23
275	Transaldolase	IPI00744692	-2.725	0.00095	30	31	20	13
276	Isoform 2 of Microtubule-actin cross-linking factor 1, isoforms 1/2/3/5	IPI00256861	2.185	0.00215	14	3	22	12
277	Isoform 2 of 4F2 cell-surface antigen heavy chain	IPI00027493	-2.246	0.00199	35	33	23	20
278	Phosphoribosylformylglycinamidine synthase	IPI00004534	-0.913	0.05316	27	31	26	22
279	Isoform 1 of Eukaryotic translation initiation factor 3 subunit B	IPI00396370	1.353	0.01818	23	24	32	30
280	Eukaryotic translation initiation factor 5B	IPI00299254	-1.125	0.03279	29	27	24	20
281	Solute carrier family 2, facilitated glucose transporter member 1	IPI00220194	-0.095	0.36527	25	24	28	20

No.	Description	Accession number	STN[1]	p-Value[1]	Con_A[2]	Con_B[2]	OXA_A[2]	OXA_B[2]
282	26S proteasome non-ATPase regulatory subunit 2	IPI00012268	0.504	0.16177	12	28	24	21
283	KH-type splicing regulatory protein	IPI00479786	-1.020	0.03987	28	40	29	27
284	retinol-binding protein 1 isoform a	IPI00219718	-2.221	0.00203	33	31	24	16
285	Estradiol 17-beta-dehydrogenase 12	IPI00007676	-1.919	0.00414	34	36	28	20
286	Proteasome activator complex subunit 1	IPI00479722	-1.463	0.01420	34	27	24	21
287	annexin A4	IPI00793199	-1.118	0.03453	32	35	30	24
288	rRNA 2'-O-methyltransferase fibrillarin	IPI00025039	-1.943	0.00406	19	27	14	14
289	Keratin, type I cytoskeletal 16	IPI00217963	-0.554	0.13055	10	13	8	11
290	Isoform 1 of Myb-binding protein 1A	IPI00005024	-7.646	0.00008	51	44	12	4
291	Programmed cell death 6-interacting protein	IPI00246058	-1.268	0.02074	25	36	26	21
292	Isoform 1 of Ras-related protein Rab-1A	IPI00005719	-2.772	0.00091	35	37	21	20
293	Peptidyl-prolyl cis-trans isomerase B	IPI00646304	-1.342	0.01863	34	29	24	24
294	GTP-binding nuclear protein Ran	IPI00643041	-2.154	0.00219	27	25	21	10
295	Isoform 1 of Heterogeneous nuclear ribonucleoprotein D0	IPI00028888	-1.135	0.03201	35	30	29	23
296	DNA replication licensing factor MCM5	IPI00018350	-0.794	0.06873	22	26	23	17
297	cytochrome b5 type B precursor	IPI00303954	0.533	0.13796	20	15	21	19
298	THO complex subunit 4	IPI00328840	-0.272	0.28225	31	24	29	23
299	Isoform Long of 14-3-3 protein beta/alpha	IPI00216318	-2.657	0.00095	36	32	22	17
300	Isoform 1 of Adenylyl cyclase-associated protein 1	IPI00008274	-1.442	0.01520	36	35	29	25
301	Collapsin response mediator protein 4 long variant	IPI00029111	0.684	0.08774	17	25	23	26
302	Thioredoxin domain-containing protein 17	IPI00646689	-0.545	0.13328	7	6	7	3
303	Proteasome subunit beta type-4	IPI00555956	-0.983	0.04538	28	33	21	29
304	Chloride intracellular channel protein 1	IPI00010896	-1.236	0.02182	29	26	23	19
305	Isoform 2 of Voltage-dependent anion-selective channel protein 2	IPI00024145	-1.122	0.03283	25	22	20	16
306	ATP-binding cassette sub-family E member 1	IPI00303207	-2.955	0.00087	36	37	23	17
307	Aldehyde dehydrogenase, mitochondrial	IPI00006663	-1.864	0.00489	30	31	21	20
308	Histone H1.2	IPI00217465	-3.466	0.00075	46	39	23	21
309	Isoform Gamma-1 of Serine/threonine-protein phosphatase PP1-gamma catalytic subunit	IPI00005705	-2.043	0.00368	33	24	23	13
310	Methionyl-tRNA synthetase, cytoplasmic	IPI00008240	-3.006	0.00087	31	36	18	17
311	Isoform 2 of S-phase kinase-associated protein 1	IPI00172421	-0.107	0.36419	22	16	29	8
312	probable ubiquitin carboxyl-terminal hydrolase FAF-X isoform 4	IPI00003964	-0.479	0.16657	17	14	15	12
313	Probable phosphoglycerate mutase 4	IPI00374975	-0.820	0.06579	17	17	14	13
314	Glutathione S-transferase kappa 1	IPI00219673	-0.309	0.26221	20	22	24	15
315	Eukaryotic initiation factor 4A-III	IPI00009328	-0.917	0.05142	24	22	19	18
316	Eukaryotic translation initiation factor 2 subunit 1	IPI00219678	-1.364	0.01814	30	23	23	16
317	Tu translation elongation factor, mitochondrial precursor	IPI00027107	-2.242	0.00199	31	32	21	18
318	Isoform 1 of Far upstream element-binding protein 1	IPI00375441	-1.147	0.03176	22	23	19	15
319	Isoform 1 of Isocitrate dehydrogenase [NAD] subunit alpha, mitochondrial	IPI00030702	-0.880	0.05445	28	22	22	19
320	Isoform 1 of Cytosolic acyl coenzyme A thioester hydrolase	IPI00010415	-1.378	0.01809	28	24	19	19
321	cDNA FLJ34068 fis, clone FCBBF3001918	IPI00168184	-1.407	0.01536	23	20	16	14
322	Ras GTPase-activating protein-binding protein 1	IPI00012442	0.196	0.32796	23	21	25	21
323	Mitochondrial carrier homolog 2	IPI00003833	-0.907	0.05325	23	24	21	17
324	Structural maintenance of chromosomes protein 1A	IPI00291939	0.954	0.04815	20	23	28	25
325	cDNA FLJ59211, highly similar to Glucosidase 2 subunit beta	IPI00026154	-0.092	0.36544	28	24	25	26
326	Protein DJ-1	IPI00298547	-1.192	0.03143	28	22	23	15
327	Tyrosyl-tRNA synthetase, cytoplasmic	IPI00007074	-3.739	0.00062	28	29	13	9
328	Isoform 2 of SWI/SNF complex subunit SMARCC2	IPI00150057	-1.516	0.01342	23	27	19	16
329	Inosine-5'-monophosphate dehydrogenase 2	IPI00291510	-1.454	0.01445	22	25	19	14
330	Rab GDP dissociation inhibitor alpha	IPI00010154	-0.578	0.12645	26	24	21	23
331	26S proteasome non-ATPase regulatory subunit 3	IPI00011603	-0.336	0.23783	19	16	19	13
332	Isoform 1 of Acetyl-CoA carboxylase 1	IPI00011569	1.377	0.01797	11	10	24	8
333	Proteasome subunit alpha type-2	IPI00219622	-0.189	0.33190	26	24	25	23
334	Hepatoma-derived growth factor	IPI00020956	-1.236	0.02182	27	28	23	19
335	Hypoxanthine-guanine phosphoribosyltransferase	IPI00218493	-0.871	0.05490	27	24	24	18
336	E3 SUMO-protein ligase RanBP2	IPI00221325	-2.062	0.00323	24	27	17	14
337	Isoform Mitochondrial of Peroxiredoxin-5, mitochondrial	IPI00024915	-2.785	0.00091	26	29	21	7
338	Proteasome subunit beta type-1	IPI00025019	-1.180	0.03143	22	29	19	20
339	Isoform 1 of Splicing factor U2AF 65 kDa subunit	IPI00031556	-0.832	0.06356	25	31	21	26
340	RuvB-like 2	IPI00009104	-0.097	0.36498	21	26	23	23
341	Isoform 1 of ATP-binding cassette sub-family B member 7, mitochondrial	IPI00306748	-0.669	0.10815	20	17	25	6
342	Glycogen phosphorylase, brain form	IPI00004358	-1.626	0.01147	21	23	10	19
343	DNA-directed RNA polymerase II subunit RPB1	IPI00031627	-1.373	0.01814	24	21	20	12
344	60S ribosomal protein L23	IPI00010153	-4.269	0.00012	33	38	16	11
345	Leukocyte elastase inhibitor	IPI00027444	-0.946	0.05014	27	27	22	22
346	Eukaryotic translation initiation factor 3, subunit E interacting protein	IPI00465233	0.401	0.20599	20	21	27	18
347	Ras-related protein Rab-7a	IPI00016342	-1.604	0.01147	33	25	22	19
348	Protein tyrosine phosphatase-like protein PTPLAD1	IPI00008998	0.000	0.37049	13	13	18	8
349	Ezrin	IPI00843975	-1.684	0.00799	24	29	18	18
350	Eukaryotic translation initiation factor 3 subunit M	IPI00102069	0.202	0.32420	18	23	24	19
351	Lamin-B receptor	IPI00292135	-0.917	0.05184	18	18	19	9
352	Isoform 1 of Transcription factor BTF3	IPI00221035	-2.642	0.00095	23	25	15	9
353	2-oxoglutarate dehydrogenase, mitochondrial	IPI00098902	0.820	0.06637	22	15	22	23
354	Translin	IPI00018768	-1.014	0.03987	27	20	20	17
355	Isoform 1 of Elongation factor 1-delta	IPI00023048	0.000	0.37049	27	18	27	18
356	Multifunctional protein ADE2	IPI00217223	-2.925	0.00087	29	33	17	15
357	14-3-3 protein theta	IPI00018146	-2.198	0.00215	25	35	18	19
358	Isoform Beta of Lamina-associated polypeptide 2, isoforms beta/gamma	IPI00030131	-0.296	0.26689	24	22	20	23
359	Isoform Long of Trifunctional purine biosynthetic protein adenosine-3	IPI00025273	-1.471	0.01391	25	24	16	16
360	RAP1, GTP-GDP dissociation stimulator 1 isoform 6	IPI00424869	0.000	0.37049	1	0	1	1
361	Isoform Long of Glucose-6-phosphate 1-dehydrogenase	IPI00216008	-1.425	0.01524	19	23	16	13
362	Transmembrane emp24 domain-containing protein 10	IPI00028055	-1.853	0.00497	23	27	16	16
363	Putative uncharacterized protein DKFZp686L20222	IPI00026689	2.795	0.00079	13	12	28	23
364	Lamin-B2	IPI00009771	0.401	0.20599	20	21	22	23
365	Isoform 1 of Nuclear pore complex protein Nup160	IPI00748807	-0.115	0.36200	18	14	20	11
366	tRNA (cytosine-5-)-methyltransferase NSUN2	IPI00306369	-1.819	0.00530	19	22	13	12
367	Isoform 1 of 5'-3' exoribonuclease 2	IPI00100151	-0.192	0.32867	25	23	26	20
368	Isoform 1 of Leukotriene A-4 hydrolase	IPI00219077	-0.705	0.08475	22	24	19	20
369	arylacetamide deacetylase-like 1 isoform b	IPI00002230	-1.855	0.00497	18	17	11	9
370	Isoform 5 of E3 ubiquitin-protein ligase UBR4	IPI00180305	0.579	0.12914	10	7	16	5
371	Superkiller viralicidic activity 2-like 2	IPI00647217	-0.646	0.11390	11	16	13	9
372	proteasome-associated protein ECM29 homolog	IPI00157790	-2.774	0.00091	25	23	16	7
373	Phosphoserine aminotransferase	IPI00001734	-2.066	0.00323	25	21	16	11
374	60S ribosomal protein L15	IPI00470528	-3.047	0.00087	27	21	12	9
375	Isoform 1 of Mitochondrial inner membrane protein	IPI00009960	-0.305	0.26288	20	23	20	20
376	40S ribosomal protein S10	IPI00008438	-1.767	0.00646	19	19	14	9

No.	Description	Accession number	STN[1]	p-Value[1]	Con_A[2]	Con_B[2]	OXA_A[2]	OXA_B[2]
377	DNA replication licensing factor MCM2	IPI00184330	0.312	0.25228	17	21	20	21
378	Isoform 1 of Calcyclin-binding protein	IPI00395627	0.389	0.20702	24	20	25	23
379	Isoform Complexed of Arginyl-tRNA synthetase, cytoplasmic	IPI00004860	-1.157	0.03167	28	25	22	19
380	Nestin	IPI00010800	-2.312	0.00178	28	27	15	17
381	Isoform 2 of Apoptosis inhibitor 5	IPI00554742	0.299	0.26776	18	24	24	21
382	Isoform A of AP-1 complex subunit beta-1	IPI00328257	0.332	0.24491	19	14	19	17
383	Isoform 1 of Transportin-1	IPI00024364	0.000	0.37049	13	16	15	14
384	26S proteasome non-ATPase regulatory subunit 13 isoform 2	IPI00375380	-1.506	0.01354	17	21	12	13
385	Tubulin beta-1 chain	IPI00006510	-2.515	0.00099	30	35	19	19
386	ATP-dependent RNA helicase DDX18	IPI00301323	-1.272	0.02074	17	20	16	10
387	Phosphatidylethanolamine-binding protein 1	IPI00219446	-1.725	0.00733	21	24	17	12
388	26S protease regulatory subunit 6A	IPI00018398	0.000	0.37049	16	23	15	24
389	Plastin-1	IPI00032304	0.109	0.36266	18	17	21	15
390	Isoform 1 of RuvB-like 1	IPI00021187	-1.025	0.03979	22	24	19	17
391	Nascent polypeptide-associated complex subunit alpha	IPI00023748	-1.502	0.01354	29	29	24	18
392	Dipeptidyl peptidase 1	IPI00022810	-0.271	0.28225	12	11	15	6
393	Putative high mobility group protein 1-like 10	IPI00018755	-0.621	0.12040	24	19	16	21
394	Isoform 3 of Adenylate kinase 2, mitochondrial	IPI00172460	-1.919	0.00414	22	25	14	15
395	Plastin-3	IPI00216694	-0.983	0.04546	28	22	21	19
396	Dolichyl-diphosphooligosaccharide--protein glycosyltransferase subunit STT3B	IPI00152377	0.125	0.35939	13	13	15	12
397	cDNA FLJ55599, highly similar to DNA replication licensing factor MCM3	IPI00013214	-0.713	0.08186	22	23	15	23
398	Isoform Non-muscle of Myosin light polypeptide 6	IPI00335168	-0.108	0.36382	21	16	23	13
399	Cathepsin D	IPI00011229	-0.092	0.36544	27	25	27	24
400	Isoform Long of Ubiquitin carboxyl-terminal hydrolase 5	IPI00024664	-1.220	0.02323	21	19	13	16
401	GMP synthase [glutamine-hydrolyzing]	IPI00029079	-0.539	0.13535	16	23	15	19
402	Macrophage migration inhibitory factor	IPI00293276	-4.071	0.00025	15	15	2	3
403	Isoform E of Eukaryotic translation initiation factor 4 gamma 1	IPI00386533	-1.586	0.01172	24	22	15	16
404	CTP synthase 1	IPI00290142	0.109	0.36266	18	17	18	18
405	Isoform 1 of Electron transfer flavoprotein subunit beta	IPI00004902	-0.892	0.05391	24	14	16	14
406	Isoform 2 of Spliceosome RNA helicase BAT1	IPI00641829	-1.919	0.00414	20	27	16	13
407	Isoform 1 of Acidic leucine-rich nuclear phosphoprotein 32 family member B	IPI00007423	-0.577	0.12670	20	14	16	13
408	ATP synthase subunit b, mitochondrial	IPI00029133	-0.553	0.13059	19	18	17	15
409	Isoform 1 of Pyridoxal kinase	IPI00013004	-0.859	0.06124	23	18	18	15
410	60S acidic ribosomal protein P2	IPI00008529	-0.583	0.12591	24	25	22	21
411	Isoform 1 of Heterogeneous nuclear ribonucleoprotein D-like	IPI00011274	-1.049	0.03685	20	24	17	17
412	Actin-related protein 2/3 complex subunit 4	IPI00554811	-0.127	0.35922	15	11	18	7
413	Isoform 1 of ER lumen protein retaining receptor 2	IPI00018248	-0.289	0.27099	13	7	12	6
414	Isoform 1 of Proteasome activator complex subunit 3	IPI00030243	0.117	0.36121	15	15	19	12
415	60S ribosomal protein L7a	IPI00299573	-1.231	0.02323	26	21	18	17
416	Isoform 1 of Cytoskeleton-associated protein 5	IPI00028275	2.435	0.00120	6	4	15	11
417	40S ribosomal protein S4, X isoform	IPI00217030	-1.116	0.03466	23	16	17	12
418	Isoform 2 of Splicing factor 3B subunit 3	IPI00179138	-0.122	0.36030	12	16	19	8
419	Actin-related protein 2	IPI00005159	-0.628	0.11523	21	21	19	17
420	DNA replication licensing factor MCM4	IPI00018349	1.275	0.02062	16	16	25	19
421	V-type proton ATPase catalytic subunit A	IPI00007682	0.434	0.18847	17	17	22	16
422	60S ribosomal protein L23a	IPI00021266	-0.718	0.07842	15	17	12	14
423	Nuclease-sensitive element-binding protein 1	IPI00031812	-0.103	0.36461	26	15	21	19
424	HMT1 hnRNP methyltransferase-like 2 isoform 1	IPI00018522	-0.848	0.06186	20	22	17	17
425	WD repeat-containing protein 36	IPI00169325	-1.800	0.00563	12	16	12	3
426	Isoform 1 of Hydroxyacyl-coenzyme A dehydrogenase, mitochondrial	IPI00294398	0.000	0.37049	15	14	17	12
427	UDP-glucose 6-dehydrogenase	IPI00031420	-0.434	0.18719	20	18	15	19
428	Isoform 1 of Protein diaphanous homolog 1	IPI00852685	0.000	0.37049	10	14	14	10
429	SERPINE1 mRNA binding protein 1, isoform CRA_d	IPI00410693	-1.045	0.03743	18	10	9	11
430	Nodal modulator 1	IPI00329352	-0.121	0.36063	16	13	15	13
431	Nucleosome assembly protein 1-like 1	IPI00023860	1.182	0.03180	10	15	18	17
432	Eukaryotic translation initiation factor 3 subunit C	IPI00016910	1.102	0.03532	15	15	21	19
433	Isoform 1 of Pyruvate dehydrogenase E1 component subunit beta, mitochondrial	IPI00003925	-1.546	0.01250	21	21	14	14
434	Glycogen phosphorylase, liver form	IPI00783313	0.429	0.19013	16	19	17	22
435	DNA topoisomerase 1	IPI00413611	-1.466	0.01416	16	18	15	7
436	Isoform Beta-4C of Integrin beta-4	IPI00027422	0.112	0.36196	19	14	20	14
437	Calpain-1 catalytic subunit	IPI00011285	0.985	0.04501	13	18	21	19
438	DEAD (Asp-Glu-Ala-Asp) box polypeptide 39 transcript variant	IPI00062206	-1.545	0.01250	14	17	12	7
439	Isoform SM-B' of Small nuclear ribonucleoprotein-associated proteins B and B'	IPI00027285	-0.917	0.05184	17	19	16	12
440	Isoform 3 of Ribosome-binding protein 1	IPI00215743	0.105	0.36357	18	20	20	19
441	Transgelin-2	IPI00550363	-0.405	0.20330	25	19	19	21
442	Isoform 1 of Enhancer of mRNA-decapping protein 4	IPI00376317	-2.715	0.00095	25	21	13	9
443	Isoform Short of Adenosine kinase	IPI00234368	-0.542	0.13519	12	12	9	11
444	Isoform 1 of Proteasome subunit alpha type-7	IPI00024175	-0.829	0.06492	19	25	18	18
445	Mitochondrial 2-oxoglutarate/malate carrier protein	IPI00219729	-0.458	0.17117	16	18	18	12
446	Isoform 1 of Filamin-C	IPI00178352	-1.399	0.01553	18	19	14	11
447	Isoform 1 of Splicing factor, arginine/serine-rich 7	IPI00003377	-1.360	0.01818	18	21	16	11
448	Isoform 1 of Voltage-dependent anion-selective channel protein 3	IPI00031804	-1.182	0.03143	19	16	14	11
449	Cytochrome c oxidase subunit 2	IPI00017510	-0.127	0.35922	13	13	14	11
450	Single-stranded DNA-binding protein, mitochondrial	IPI00029744	-1.725	0.00733	25	20	17	12
451	Histone H1.5	IPI00217468	-2.205	0.00215	28	22	16	13
452	60S ribosomal protein L9	IPI00031691	-2.909	0.00087	27	28	15	12
453	Peptidyl-prolyl cis-trans isomerase FKBP3	IPI00024157	-0.546	0.13229	19	19	17	16
454	Elongation factor 1-beta	IPI00178440	0.209	0.31832	20	18	22	18
455	Activated RNA polymerase II transcriptional coactivator p15	IPI00221222	-0.930	0.05113	19	16	19	8
456	60S ribosomal protein L4	IPI00003918	-1.994	0.00389	24	20	15	11
457	Vigilin	IPI00022228	-0.634	0.11498	11	17	11	12
458	ATP-dependent DNA helicase Q1	IPI00178431	-0.904	0.05325	20	17	17	12
459	Thioredoxin-dependent peroxide reductase, mitochondrial	IPI00024919	-0.440	0.18528	19	18	21	12
460	40S ribosomal protein S17	IPI00221093	-0.927	0.05118	23	22	21	15
461	Tubulin--tyrosine ligase-like protein 12	IPI00029048	-0.243	0.29923	13	16	13	14
462	Isoform 1 of Deoxyuridine 5'-triphosphate nucleotidohydrolase, mitochondrial	IPI00013679	0.217	0.31724	18	17	20	17
463	Isoform 1 of RNA-binding protein 25	IPI00004273	0.494	0.16355	15	10	14	15
464	splicing factor 3B subunit 2	IPI00221106	1.008	0.04004	12	10	16	14
465	26S proteasome non-ATPase regulatory subunit 12	IPI00185374	0.414	0.19212	19	19	19	23
466	Dihydrolipoyl dehydrogenase, mitochondrial	IPI00015911	-0.316	0.24553	18	22	16	21
467	Isoform 1 of Dipeptidyl peptidase 3	IPI00020672	0.972	0.04604	15	17	18	23
468	DNA replication licensing factor MCM6	IPI00031517	0.796	0.06873	17	12	20	16
469	Putative uncharacterized protein PSME2	IPI00384051	0.125	0.35939	15	11	13	14
470	Isoform Beta of Heat shock protein 105 kDa	IPI00218993	0.832	0.06389	11	15	15	18
471	Isoform 1 of Importin-4	IPI00156374	-0.634	0.11498	14	14	13	10

No.	Description	Accession number	STN[1]	p-Value[1]	Con_A[2]	Con_B[2]	OXA_A[2]	OXA_B[2]
472	cDNA FLJ56307, highly similar to Ubiquitin thioesterase protein OTUB1	IPI00000581	0.464	0.16984	13	16	18	15
473	Isoform 5 of Glycogen debranching enzyme	IPI00219065	1.332	0.02004	11	12	17	17
474	Isoform 1 of WD repeat-containing protein 1	IPI00746165	0.755	0.07788	10	13	16	13
475	40S ribosomal protein S7	IPI00013415	-1.744	0.00712	20	14	11	9
476	Galectin-3	IPI00465431	0.871	0.05598	10	6	9	13
477	Isoform 1 of Cleavage and polyadenylation specificity factor subunit 6	IPI00012998	-0.997	0.04095	21	18	17	13
478	F-actin-capping protein subunit alpha-1	IPI00005969	0.000	0.37049	15	17	16	16
479	Isoform 1 of Poly(U)-binding-splicing factor PUF60	IPI00069750	-0.660	0.11279	21	17	17	15
480	Isoform 1 of Regulator of nonsense transcripts 1	IPI00034049	0.119	0.36101	16	13	16	14
481	cDNA FLJ59758, highly similar to S-methyl-5-thioadenosine phosphorylase	IPI00011876	-0.930	0.05113	16	19	14	13
482	High mobility group protein B2	IPI00219097	0.832	0.06389	12	6	11	13
483	26S proteasome non-ATPase regulatory subunit 6	IPI00014151	-0.464	0.16856	15	18	16	13
484	TC4 protein	IPI00044779	-0.718	0.07842	17	15	11	15
485	L-xylulose reductase	IPI00448095	0.768	0.07465	9	13	14	14
486	40S ribosomal protein S9	IPI00221088	-0.718	0.07842	16	16	14	12
487	Cleavage and polyadenylation specificity factor subunit 5	IPI00646917	-0.114	0.36225	17	16	17	15
488	Myosin regulatory light chain 12B	IPI00033494	-1.285	0.02050	17	13	13	7
489	Endoplasmic reticulum resident protein 29	IPI00024911	-1.921	0.00414	20	22	13	12
490	Glutathione synthetase	IPI00010706	0.553	0.13328	14	18	16	21
491	Isoform 2 of Structural maintenance of chromosomes protein 4	IPI00328298	2.050	0.00331	14	10	21	21
492	Phenylalanyl-tRNA synthetase beta chain	IPI00300074	-0.594	0.12314	16	16	17	10
493	Coatomer subunit gamma-2	IPI00002557	0.000	0.37049	4	4	2	6
494	Condensin complex subunit 1	IPI00299524	1.843	0.00460	11	6	19	12
495	Isoform 1 of Nuclear autoantigenic sperm protein	IPI00179953	-0.808	0.06662	16	19	13	15
496	Isoform 1 of 26S protease regulatory subunit 6B	IPI00020042	-0.380	0.20748	13	14	12	12
497	Isoform 2 of Splicing factor 1	IPI00294627	-0.579	0.12599	9	12	9	8
498	Protein RRP5 homolog	IPI00400922	-5.808	0.00008	26	30	8	0
499	cDNA FLJ78679, highly similar to Homo sapiens DEAD (Asp-Glu-Ala-Asp) box polypeptide 46	IPI00329791	-0.765	0.07569	22	17	14	18
500	Isoform 4 of Heterogeneous nuclear ribonucleoprotein A/B	IPI00106509	-0.808	0.06662	18	17	14	14
501	Isoform Mitochondrial of Fumarate hydratase, mitochondrial	IPI00296053	-1.182	0.03143	14	21	9	16
502	Dolichyl-diphosphooligosaccharide--protein glycosyltransferase 48 kDa subunit	IPI00297084	-0.328	0.23895	17	20	17	17
503	Isoform 1 of Mitochondrial import receptor subunit TOM40 homolog	IPI00014053	-0.594	0.12525	10	10	8	8
504	Isoform 1 of DNA-binding protein A	IPI00031801	-1.147	0.03176	15	22	15	12
505	Histone-binding protein RBBP4	IPI00328319	0.000	0.37049	11	18	14	15
506	Bifunctional ATP-dependent dihydroxyacetone kinase/FAD-AMP lyase (cyclizing)	IPI00551024	-0.561	0.13009	19	17	15	16
507	26S proteasome non-ATPase regulatory subunit 7	IPI00019927	-0.707	0.08451	19	14	13	14
508	cDNA FLJ77422, highly similar to Homo sapiens RNA binding protein	IPI00011268	-1.687	0.00799	18	18	12	10
509	40S ribosomal protein S5	IPI00008433	-1.998	0.00389	16	15	11	5
510	Isoform 1 of Large proline-rich protein BAT3	IPI00465128	0.000	0.37049	17	10	17	10
511	Proteasome subunit alpha type-6	IPI00029623	-0.239	0.30076	17	13	16	12
512	NAD-dependent malic enzyme, mitochondrial	IPI00011201	-0.297	0.26664	11	8	11	6
513	Isoform 2 of Protein disulfide-isomerase A6	IPI00299571	-0.869	0.05585	18	22	14	18
514	U2 small nuclear ribonucleoprotein A'	IPI00297477	-1.874	0.00489	27	22	17	14
515	Isoform 1 of Protein-L-isoaspartate(D-aspartate) O-methyltransferase	IPI00411680	-0.486	0.16611	16	14	16	10
516	Isoform 1 of Methylcrotonoyl-CoA carboxylase beta chain, mitochondrial	IPI00784044	-0.108	0.36382	19	18	19	17
517	Keratin, type I cytoskeletal 14	IPI00384444	-1.575	0.01230	17	13	10	8
518	Estradiol 17-beta-dehydrogenase 11	IPI00329598	0.119	0.36101	16	13	18	12
519	Isoform 1 of Transformer-2 protein homolog beta	IPI00301503	-0.440	0.18528	18	19	17	16
520	cDNA FLJ36192 fis, clone TESTI2027450	IPI00654777	0.000	0.37049	18	16	21	13
521	Isoform 1 of RNA-binding protein 8A	IPI00001757	-0.115	0.36200	17	15	15	16
522	Isoform 3 of Probable ATP-dependent RNA helicase DDX17	IPI00651653	0.542	0.13552	9	11	12	12
523	Protein transport protein Sec24C	IPI00024661	0.266	0.28664	12	10	10	14
524	40S ribosomal protein S13	IPI00221089	-1.959	0.00406	16	16	9	8
525	Signal recognition particle receptor subunit beta	IPI00295098	0.486	0.16541	9	17	13	17
526	Wolframin	IPI00008711	-1.716	0.00778	14	12	9	5
527	cDNA FLJ54492, highly similar to Eukaryotic translation initiation factor 4B	IPI00012079	0.117	0.36121	14	16	17	14
528	26S protease regulatory subunit 4	IPI00011126	0.687	0.08757	12	17	15	20
529	10 kDa heat shock protein, mitochondrial	IPI00220362	-0.451	0.18446	11	8	9	7
530	Heat shock protein beta (Fragment)	IPI00411633	-0.874	0.05490	16	14	14	9
531	Long-chain-fatty-acid--CoA ligase 3	IPI00031397	0.000	0.37049	7	12	10	9
532	Isoform 1 of 40S ribosomal protein S24	IPI00029750	-1.767	0.00646	20	18	12	11
533	Ras-related protein Rab-5C	IPI00016339	0.000	0.37049	15	13	16	12
534	Isoform 1 of ATP synthase subunit d, mitochondrial	IPI00220487	-0.974	0.04592	15	17	13	11
535	Stomatin-like protein 2	IPI00334190	-2.482	0.00108	22	23	12	11
536	Isoform 1 of NADH-cytochrome b5 reductase 3	IPI00328415	1.354	0.01818	10	12	17	16
537	Galectin-3-binding protein	IPI00023673	-0.277	0.28176	8	14	12	8
538	Midasin	IPI00167941	0.892	0.05436	6	9	16	5
539	Ubiquitin carboxyl-terminal hydrolase 14	IPI00219913	-1.189	0.03143	20	22	16	15
540	Isoform 5 of Dynamin-1-like protein	IPI00037283	0.341	0.24400	14	17	14	20
541	Heterogeneous nuclear ribonucleoprotein A0	IPI00011913	-0.368	0.21679	14	15	12	14
542	Putative uncharacterized protein NAP1L4	IPI00017763	-0.742	0.07668	19	11	12	12
543	Isoleucyl-tRNA synthetase, mitochondrial	IPI00017283	0.798	0.06757	11	9	15	11
544	Isoform 1 of Nuclear pore complex protein Nup155	IPI00026625	0.718	0.07974	14	12	17	15
545	transcription activator BRG1 isoform D	IPI00029822	-1.164	0.03155	15	21	14	12
546	NADH dehydrogenase [ubiquinone] 1 alpha subcomplex subunit 9, mitochondrial	IPI00003968	-1.377	0.01809	14	18	12	9
547	60S ribosomal protein L7	IPI00030179	-2.887	0.00087	19	23	10	8
548	probable E3 ubiquitin-protein ligase MYCBP2	IPI00289776	1.260	0.02087	4	8	15	5
549	sister chromatid cohesion protein PDS5 homolog A isoform 2	IPI00303063	0.685	0.08757	8	11	12	12
550	Destrin	IPI00473014	0.941	0.05188	9	10	15	11
551	Isoform 1 of Glycerol-3-phosphate dehydrogenase, mitochondrial	IPI00017895	1.200	0.03047	11	13	16	18
552	Sodium/potassium-transporting ATPase subunit alpha-2	IPI00003021	-0.464	0.16856	17	16	13	16
553	Echinoderm microtubule-associated protein-like 4	IPI00001466	-0.734	0.07743	11	10	8	8
554	Keratin, type II cytoskeletal 5	IPI00009867	1.076	0.03610	5	4	10	5
555	Histone H3.2	IPI00171611	-0.842	0.06318	3	3	2	0
556	Isoform 2 of Basigin	IPI00019906	-0.765	0.07569	16	23	16	16
557	Cytochrome c	IPI00465315	-1.808	0.00547	16	16	13	5
558	Uncharacterized protein C17orf25	IPI00007102	0.127	0.35881	11	14	14	12
559	Isoform 1 of Spectrin beta chain, brain 2	IPI00012645	-1.855	0.00497	11	12	7	4
560	60S ribosomal protein L27	IPI00219155	-1.921	0.00414	15	14	8	7
561	Isoform 1 of Tensin-3	IPI00658152	-3.854	0.00062	21	26	9	6
562	Isoform 1 of ATP-dependent RNA helicase DDX42	IPI00409671	-0.313	0.24578	9	8	7	8
563	Isoform 1 of Cytosol aminopeptidase	IPI00419237	0.471	0.16839	12	16	17	15
564	UPF0568 protein C14orf166	IPI00006980	-0.320	0.24516	20	19	18	18
565	Proteasome 26S non-ATPase subunit 11 variant (Fragment)	IPI00105598	-0.117	0.36125	13	18	14	16
566	septin-9 isoform e	IPI00455033	-0.585	0.12579	15	18	12	16

No.	Description	Accession number	STN[1]	p-Value[1]	Con_A[2]	Con_B[2]	OXA_A[2]	OXA_B[2]
567	Isoform 1 of Surfeit locus protein 4	IPI00005737	-0.127	0.35922	13	13	17	8
568	Isoform 1 of Cell division cycle and apoptosis regulator protein 1	IPI00217357	-0.134	0.35645	13	10	14	8
569	Ubiquitin-like modifier activating enzyme 1	IPI00552452	-1.354	0.01826	16	17	12	10
570	Isoform 2 of Annexin A2	IPI00418169	-1.011	0.04008	19	19	17	12
571	Structural maintenance of chromosomes flexible hinge domain-containing protein 1	IPI00465022	-0.941	0.05101	12	14	13	6
572	Isoform 2 of Signal recognition particle 68 kDa protein	IPI00102936	-0.796	0.06819	17	19	15	14
573	Splicing factor 3A subunit 3	IPI00029764	-0.985	0.04534	20	20	16	15
574	Isoform Beta of Nucleolar and coiled-body phosphoprotein 1	IPI00216654	-0.700	0.08529	12	11	10	8
575	E3 ubiquitin/ISG15 ligase TRIM25	IPI00029629	-1.497	0.01354	13	10	9	4
576	cDNA FLJ60076, highly similar to ELAV-like protein 1	IPI00301936	-0.808	0.06662	17	18	15	13
577	6-phosphofructokinase type C	IPI00009790	-2.418	0.00145	23	20	12	10
578	Isoform Short of Proteasome subunit alpha type-1	IPI00016832	-0.440	0.18628	9	11	9	8
579	40S ribosomal protein S16	IPI00221092	-2.552	0.00095	18	15	9	5
580	Succinyl-CoA:3-ketoacid-coenzyme A transferase 1, mitochondrial	IPI00026516	-0.115	0.36200	16	16	15	16
581	14-3-3 protein gamma	IPI00220642	-2.007	0.00389	20	19	11	11
582	Ras-related protein Rab-11B	IPI00020436	0.000	0.37049	16	13	15	14
583	TOB3	IPI00045921	0.122	0.36005	13	14	17	11
584	Tubulin beta-2A chain	IPI00013475	-1.425	0.01524	20	22	14	15
585	Isoform 3 of Splicing factor, arginine/serine-rich 13A	IPI00009071	-0.585	0.12579	16	17	15	13
586	Ubiquitin-conjugating enzyme E2 N	IPI00003949	-0.730	0.07743	17	14	16	9
587	Ras-related protein Rap-1b	IPI00015148	0.362	0.23501	14	13	17	13
588	Early endosome antigen 1	IPI00329536	-0.122	0.36030	15	13	12	15
589	Serpin B6	IPI00413451	-0.577	0.12670	15	19	14	15
590	Periodic tryptophan protein 2 homolog	IPI00300078	-1.964	0.00402	13	15	13	1
591	Isoform 1 of Cullin-4B	IPI00179057	0.387	0.20764	10	13	11	15
592	protein arginine N-methyltransferase 5 isoform b	IPI00064328	0.000	0.37049	17	11	14	14
593	Peroxiredoxin-2	IPI00027350	-0.531	0.13593	14	11	14	7
594	Coatomer subunit gamma	IPI00783982	-1.026	0.03967	12	17	9	12
595	Proteasome subunit alpha type-5	IPI00291922	-1.200	0.03068	17	17	10	14
596	Histidyl-tRNA synthetase, cytoplasmic	IPI00021808	-0.368	0.21679	16	13	13	13
597	NADH-ubiquinone oxidoreductase 75 kDa subunit	IPI00604664	-0.380	0.20748	11	16	12	12
598	UPF0027 protein C22orf28	IPI00550689	-1.379	0.01809	17	21	12	14
599	Isoform Mitochondrial of Glutathione reductase, mitochondrial	IPI00016862	-0.554	0.13055	13	10	11	8
600	Neurolysin, mitochondrial	IPI00010346	-1.726	0.00733	13	17	11	6
601	Transcription factor A, mitochondrial	IPI00020928	-0.671	0.10811	12	13	9	11
602	Importin subunit alpha-2	IPI00002214	2.830	0.00070	6	9	17	20
603	Isoform 1 of Malignant T cell-amplified sequence 1	IPI00179026	-1.189	0.03143	15	13	9	10
604	FACT complex subunit SSRP1	IPI00005154	-1.213	0.02343	12	15	7	11
605	UMP-CMP kinase isoform a	IPI00219953	-0.247	0.29898	15	13	15	11
606	Aspartate aminotransferase, cytoplasmic	IPI00219029	-0.577	0.12670	15	19	13	16
607	40S ribosomal protein S8	IPI00216587	-1.824	0.00530	19	17	10	11
608	Isoform 1 of ATP-dependent RNA helicase DDX19B	IPI00008943	0.718	0.07974	11	15	17	15
609	Ribosomal L1 domain-containing protein 1	IPI00008708	-4.576	0.00008	25	26	8	5
610	Microtubule-associated protein RP/EB family member 1	IPI00017596	-1.145	0.03184	16	14	11	10
611	cDNA FLJ51909, highly similar to Serine-threonine kinase receptor-associatedprotein	IPI00294536	-1.220	0.02323	16	17	11	12
612	Calpain small subunit 1	IPI00025084	-1.164	0.03155	17	19	12	14
613	Small nuclear ribonucleoprotein Sm D2	IPI00017963	-1.145	0.03184	18	12	15	6
614	Proteasome subunit alpha type-4	IPI00299155	-0.134	0.35645	13	10	11	11
615	26S protease regulatory subunit 8	IPI00023919	-0.351	0.22984	16	16	14	15
616	Splicing factor 3A subunit 1	IPI00017451	-0.420	0.18943	10	12	8	11
617	Thyroid hormone receptor-associated protein 3	IPI00104050	-1.066	0.03606	15	12	7	12
618	Probable ATP-dependent RNA helicase DDX6	IPI00030320	0.000	0.37049	17	15	17	15
619	Isoform 1 of F-actin-capping protein subunit beta	IPI00026185	0.243	0.29981	12	15	15	14
620	Transferrin receptor protein 1	IPI00022462	2.040	0.00331	7	8	17	13
621	Isoform 1 of Host cell factor 1	IPI00019848	-1.335	0.02012	16	12	9	9
622	Beta-hexosaminidase subunit beta	IPI00012585	0.814	0.06662	11	8	18	7
623	DYNC1H1 protein	IPI00440177	0.476	0.16727	6	8	11	6
624	Insulin-like growth factor 2 mRNA-binding protein 1	IPI00008557	-0.905	0.05325	14	14	12	9
625	ADP-sugar pyrophosphatase	IPI00296913	0.266	0.28664	12	10	13	11
626	Isoform 1 of DNA (cytosine-5)-methyltransferase 1	IPI00031519	1.583	0.01126	4	7	11	10
627	Peroxiredoxin-4	IPI00011937	-0.974	0.04592	17	15	13	11
628	SUMO-activating enzyme subunit 1	IPI00033130	-1.125	0.03279	17	14	11	11
629	Isoform 1 of RNA-binding protein 39	IPI00163505	-0.122	0.36030	13	15	12	15
630	cDNA FLJ75085, highly similar to Homo sapiens glutaminyl-tRNA synthetase (QARS), mRNA	IPI00026665	0.658	0.11709	13	8	11	15
631	Nicotinamide phosphoribosyltransferase	IPI00018873	0.000	0.37049	10	14	10	14
632	Glucosamine-6-phosphate isomerase 1	IPI00009305	-0.395	0.20516	8	17	12	10
633	6-phosphogluconolactonase	IPI00029997	-1.074	0.03594	20	22	17	15
634	Isoform 1 of Minor histocompatibility antigen H13	IPI00152441	0.428	0.19017	2	5	5	4
635	DNA-(apurinic or apyrimidinic site) lyase	IPI00215911	-2.500	0.00108	17	17	8	7
636	Isoform 1 of Protein KIAA1967	IPI00182757	-0.503	0.16131	11	17	12	12
637	Actin-related protein 2/3 complex subunit 3	IPI00005162	-1.088	0.03573	16	10	12	6
638	U1 small nuclear ribonucleoprotein A	IPI00012382	-0.859	0.06124	12	19	13	11
639	Isoform 1 of ATP-binding cassette sub-family D member 3	IPI00002372	1.518	0.01304	9	7	17	10
640	Isoform 1 of Transformation/transcription domain-associated protein	IPI00069084	2.616	0.00087	3	2	14	5
641	Programmed cell death protein 6	IPI00025277	-1.400	0.01553	10	11	6	6
642	NADH dehydrogenase [ubiquinone] iron-sulfur protein 3, mitochondrial	IPI00025796	-1.880	0.00489	19	11	8	8
643	Src substrate cortactin	IPI00029601	0.974	0.04538	10	14	14	18
644	40S ribosomal protein S18	IPI00013296	-0.718	0.07842	17	15	17	9
645	Isoform 1 of Catenin alpha-1	IPI00215948	-0.905	0.05325	15	13	11	10
646	Isoform 1 of Structural maintenance of chromosomes protein 2	IPI00007927	3.458	0.00046	4	4	14	17
647	Dihydrolipoyllysine-residue acetyltransferase component of pyruvate dehydrogenase complex	IPI00021338	-0.730	0.07743	17	14	15	10
648	cDNA FLJ55482, highly similar to Annexin A11	IPI00414320	-1.377	0.01809	15	17	11	10
649	Isoform 1 of 60S ribosomal protein L11	IPI00376798	-1.491	0.01354	19	14	11	10
650	Alcohol dehydrogenase [NADP+]	IPI00220271	-2.608	0.00095	14	18	8	5
651	Asparaginyl-tRNA synthetase, cytoplasmic	IPI00306960	-1.456	0.01445	15	14	10	8
652	ADP-ribosylation factor-like protein 1	IPI00219518	-1.998	0.00389	16	15	10	6
653	60S ribosomal protein L10a	IPI00412579	0.000	0.37049	12	8	9	11
654	E3 ubiquitin-protein ligase UBR5	IPI00026320	-0.743	0.07647	5	8	6	3
655	Proteasome subunit beta type-2	IPI00028006	-0.941	0.05101	15	11	12	7
656	mRNA turnover protein 4 homolog	IPI00106491	-1.152	0.03167	9	9	7	4
657	Isoform GTBP-alt of DNA mismatch repair protein Msh6	IPI00106847	-0.260	0.28532	12	13	13	10
658	Sideroflexin-1	IPI00009368	0.134	0.35674	10	12	12	11
659	DNA-directed RNA polymerase II subunit RPB2	IPI00027808	0.137	0.35554	13	8	11	11
660	Isoform 1 of Apolipoprotein O	IPI00042580	-0.402	0.20429	14	10	15	6
661	Isoform 1 of Methionine adenosyltransferase 2 subunit beta	IPI00002324	-1.182	0.03143	17	18	14	11

No.	Description	Accession number	STN[1]	p-Value[1]	Con_A[2]	Con_B[2]	OXA_A[2]	OXA_B[2]
662	Translin-associated protein X	IPI00293350	-0.140	0.35413	12	9	9	11
663	Tumor protein, translationally-controlled 1	IPI00009943	0.685	0.08757	8	11	11	13
664	Ubiquitin carboxyl-terminal hydrolase 24	IPI00902614	-0.163	0.34436	7	8	7	7
665	Phenylalanyl-tRNA synthetase alpha chain	IPI00031820	0.159	0.34718	7	8	8	8
666	Eukaryotic translation initiation factor 3 subunit E	IPI00013068	0.362	0.23501	13	14	18	12
667	cDNA FLJ59739, highly similar to Protein transport protein Sec61 subunit alpha isoform 1	IPI00218466	-0.566	0.12989	12	10	9	9
668	40S ribosomal protein S15a	IPI00221091	-1.213	0.02343	18	9	11	7
669	Leucine-rich repeat-containing protein 59	IPI00396321	0.000	0.37049	10	12	13	9
670	Trifunctional enzyme subunit beta, mitochondrial	IPI00022793	0.594	0.12682	10	6	10	10
671	Isoform 1 of 14-3-3 protein sigma	IPI00013890	0.134	0.35674	13	9	11	12
672	Isoform 1 of Ubiquitin-like modifier-activating enzyme 6	IPI00023647	1.583	0.01126	4	7	8	13
673	Putative uncharacterized protein NOP2	IPI00294891	-1.964	0.00402	15	13	9	5
674	Nucleoporin 85	IPI00171542	0.000	0.37049	8	13	9	12
675	Diablo homolog, mitochondrial precursor	IPI00008418	-0.134	0.35645	11	12	12	10
676	Importin-9	IPI00185146	1.934	0.00368	7	5	13	12
677	Isoform 1 of DNA replication licensing factor MCM7	IPI00299904	0.000	0.37049	10	10	10	10
678	Isoform 1 of Reticulon-4	IPI00021766	0.451	0.18504	7	9	10	9
679	Protein phosphatase 1G	IPI00006167	0.634	0.11871	10	13	19	9
680	Isoform 2 of Myosin-Ic	IPI00010418	0.000	0.37049	13	14	16	11
681	Aconitate hydratase, mitochondrial	IPI00017855	0.634	0.11871	10	13	14	14
682	26S protease regulatory subunit 7	IPI00021435	-0.283	0.27141	12	9	11	8
683	Adenylate kinase isoenzyme 1	IPI00018342	-0.479	0.16657	17	14	15	12
684	26S protease regulatory subunit S10B	IPI00021926	-0.634	0.11498	14	14	12	11
685	40S ribosomal protein S6	IPI00021840	-2.040	0.00368	15	15	12	3
686	AP-1 complex subunit gamma-1 isoform a	IPI00293396	0.140	0.35401	10	10	10	11
687	40S ribosomal protein S14	IPI00026271	-1.824	0.00530	18	18	12	9
688	Isoform 1 of CCR4-NOT transcription complex subunit 1	IPI00166010	-1.425	0.01524	16	9	11	4
689	Flap endonuclease 1	IPI00026215	-0.850	0.06161	10	13	8	9
690	Tricarboxylate transport protein, mitochondrial	IPI00294159	-1.111	0.03482	14	11	13	4
691	Proteasome subunit beta type-3	IPI00028004	0.236	0.30171	14	15	14	17
692	Peptidyl-prolyl cis-trans isomerase FKBP11	IPI00009885	-0.773	0.07341	13	6	7	7
693	Actin-related protein 3	IPI00028091	-1.631	0.01143	16	17	10	10
694	Isoform 1 of Squamous cell carcinoma antigen recognized by T-cells 3	IPI00006025	0.716	0.08037	7	10	12	10
695	proteasome 26S non-ATPase subunit 8	IPI00010201	-0.429	0.18789	11	10	12	6
696	Dolichyl-diphosphooligosaccharide--protein glycosyltransferase subunit STT3A	IPI00297492	-0.313	0.24578	11	6	10	5
697	Hsc70-interacting protein	IPI00032826	-0.579	0.12599	10	11	11	6
698	Isoform 1 of 3,2-trans-enoyl-CoA isomerase, mitochondrial	IPI00300567	-1.026	0.03967	17	12	12	9
699	Nuclear cap-binding protein subunit 1	IPI00019380	0.277	0.28271	10	10	8	14
700	Isoform 2 of Nuclear protein localization protein 4 homolog	IPI00001676	-0.289	0.27099	13	7	11	7
701	SWI/SNF related, matrix associated, actin dependent regulator of chromatin, subfamily a	IPI00216046	0.645	0.11809	5	8	7	10
702	26S proteasome non-ATPase regulatory subunit 14	IPI00024821	-0.503	0.16131	14	14	11	13
703	Histidine triad nucleotide-binding protein 1	IPI00239077	-0.507	0.15999	9	6	7	5
704	Eukaryotic peptide chain release factor subunit 1	IPI00429191	-0.494	0.16177	12	17	12	13
705	Nucleolar pre-ribosomal-associated protein 1	IPI00297241	0.169	0.34208	4	9	10	4
706	Nuclear pore complex protein Nup133	IPI00291200	-0.283	0.27141	10	11	8	11
707	Enoyl-CoA hydratase, mitochondrial	IPI00024993	-0.974	0.04592	18	14	12	12
708	Putative uncharacterized protein KIAA0664	IPI00024425	0.143	0.35293	11	8	11	9
709	Thiosulfate sulfurtransferase	IPI00216293	0.150	0.35065	8	9	10	8
710	Isoform 1 of Trans-2,3-enoyl-CoA reductase	IPI00100656	0.847	0.06306	7	4	9	7
711	WD repeat-containing protein 75	IPI00217240	-1.540	0.01255	9	9	8	1
712	Cullin-1	IPI00014310	0.658	0.11709	9	12	15	11
713	Vesicle-fusing ATPase	IPI00006451	-1.189	0.03143	13	15	10	9
714	Fascin	IPI00163187	0.283	0.27211	9	10	9	12
715	Putative uncharacterized protein MYO7B	IPI00738806	-0.525	0.15692	8	6	5	6
716	Abhydrolase domain-containing protein 10, mitochondrial	IPI00020075	-1.045	0.03743	12	16	12	8
717	Isocitrate dehydrogenase 3, beta subunit isoform a precursor	IPI00304417	-0.798	0.06807	12	14	11	9
718	Ras-related protein Rab-10	IPI00016513	-1.545	0.01250	16	15	9	10
719	Isoform 1 of N-alpha-acetyltransferase 25, NatB auxiliary subunit	IPI00025890	0.343	0.23774	11	1	10	4
720	Nuclear migration protein nudC	IPI00550746	-1.045	0.03743	13	15	9	11
721	Dihydropyrimidinase-related protein 2	IPI00257508	-0.236	0.30089	16	15	15	14
722	Activator of 90 kDa heat shock protein ATPase homolog 1	IPI00030706	-1.486	0.01366	14	14	9	8
723	Isoform 1 of Protein CDV3 homolog	IPI00014197	-1.363	0.01814	14	13	9	8
724	Isoform 1 of Sodium-coupled neutral amino acid transporter 2	IPI00410034	-2.936	0.00087	17	24	7	10
725	Isoform 1 of LIM and SH3 domain protein 1	IPI00000861	0.146	0.35161	8	10	12	7
726	Isoform 3 of Shootin-1	IPI00448751	-0.795	0.06844	8	10	8	5
727	Poly(rC)-binding protein 1	IPI00016610	-1.117	0.03466	13	6	7	5
728	Calmodulin	IPI00075248	-2.340	0.00153	17	17	9	7
729	Isoform 1 of Tyrosine-protein kinase BAZ1B	IPI00069817	0.277	0.28271	9	11	14	8
730	29 kDa protein	IPI00453476	-0.146	0.35082	10	9	9	9
731	Ribose-phosphate pyrophosphokinase 1	IPI00219616	-1.887	0.00489	19	15	11	8
732	Lactoylglutathione lyase	IPI00220766	-0.150	0.34970	11	7	11	6
733	Insulin-degrading enzyme	IPI00220373	0.773	0.07432	6	8	11	8
734	Isoform 2 of Guanine nucleotide-binding protein G(i) subunit alpha-2	IPI00217906	-0.594	0.12314	12	20	12	15
735	Alpha-centractin	IPI00029468	0.395	0.20648	10	12	11	14
736	Isoform 1 of Protein strawberry notch homolog 1	IPI00023649	0.842	0.06364	1	2	4	2
737	Uroporphyrinogen decarboxylase	IPI00301489	-0.916	0.05184	3	6	4	1
738	Seryl-tRNA synthetase, cytoplasmic	IPI00220637	-3.445	0.00079	18	11	3	4
739	Isoform 1 of Septin-2	IPI00014177	-0.974	0.04592	17	15	11	13
740	Glutathione S-transferase omega-1	IPI00019755	-0.440	0.18628	11	9	11	6
741	protein ALO17 isoform 1	IPI00828098	1.152	0.03225	7	4	15	3
742	Small subunit processome component 20 homolog	IPI00004970	-3.991	0.00050	14	13	3	0
743	Isoform 2 of Exosome complex exonuclease RRP44	IPI00183462	0.734	0.07933	9	7	10	11
744	V-type proton ATPase subunit B, brain isoform	IPI00007812	1.552	0.01205	9	14	18	18
745	Mitochondrial import receptor subunit TOM22 homolog	IPI00024976	-0.554	0.13055	11	12	12	7
746	Replication protein A 70 kDa DNA-binding subunit	IPI00020127	0.609	0.12446	8	7	12	7
747	H/ACA ribonucleoprotein complex subunit 4	IPI00221394	-1.362	0.01814	11	11	9	4
748	Isoform 1 of Adenylate kinase 2, mitochondrial	IPI00215901	-2.185	0.00215	19	15	10	7
749	Cleavage and polyadenylation specificity factor subunit 1	IPI00026219	-0.542	0.13519	14	10	14	6
750	Isoform 2 of NSFL1 cofactor p47	IPI00022830	1.145	0.03254	11	10	17	13
751	Isoform 1 of La-related protein 1	IPI00185919	-1.804	0.00563	12	12	7	5
752	Nuclear pore complex protein Nup93	IPI00397904	-0.322	0.24491	10	6	10	4
753	Isoform 2 of Transportin-3	IPI00395694	0.440	0.18752	6	11	7	13
754	D-3-phosphoglycerate dehydrogenase	IPI00011200	-4.247	0.00012	18	20	4	4
755	Acidic leucine-rich nuclear phosphoprotein 32 family member E	IPI00165393	-0.798	0.06807	12	14	12	8
756	THO complex subunit 2	IPI00158615	-1.400	0.01553	8	13	8	4

No.	Description	Accession number	STN[1]	p-Value[1]	Con_A[2]	Con_B[2]	OXA_A[2]	OXA_B[2]
757	CSNK2A1 protein	IPI00016613	0.322	0.24627	6	8	8	8
758	Serine/threonine-protein kinase PAK 2	IPI00419979	0.878	0.05544	4	6	10	5
759	Serine/threonine-protein kinase mTOR	IPI00031410	-1.400	0.01553	10	11	9	3
760	Ubiquitin carboxyl-terminal hydrolase isozyme L3	IPI00011250	-1.402	0.01553	16	15	10	10
761	PNAS-139	IPI00000477	0.163	0.34457	7	7	9	6
762	Inositol 1,4,5-trisphosphate receptor type 3	IPI00291607	0.000	0.37049	5	5	7	3
763	Adenine phosphoribosyltransferase	IPI00218693	-0.623	0.12032	16	13	14	10
764	Isoform 1 of Myosin-Ib	IPI00376344	1.164	0.03209	5	10	13	10
765	Sodium/potassium-transporting ATPase subunit beta-3	IPI00008167	0.122	0.36005	10	17	14	14
766	Isoform 1 of Vesicle-associated membrane protein-associated protein B/C	IPI00006211	0.374	0.20938	11	14	11	17
767	Eukaryotic translation initiation factor 2 subunit 3	IPI00297982	-1.266	0.02083	12	13	10	6
768	Protein DEK	IPI00020021	0.402	0.20524	12	9	11	13
769	Isoform 1 of Tryptophanyl-tRNA synthetase, cytoplasmic	IPI00295400	-2.340	0.00153	16	18	8	8
770	Isoform 1 of Mitotic checkpoint protein BUB3	IPI00013468	-0.512	0.15891	11	16	12	11
771	SDHA protein	IPI00217143	-0.277	0.28176	13	9	10	10
772	Isoform 1 of Hexokinase-1	IPI00018246	0.000	0.37049	14	9	13	10
773	Reticulocalbin-1	IPI00015842	-1.934	0.00406	13	12	6	6
774	Isoform 1 of Alpha-aminoadipic semialdehyde dehydrogenase	IPI00221234	0.322	0.24627	8	6	8	8
775	GDP-mannose 4,6 dehydratase	IPI00030207	-0.961	0.04803	9	16	8	10
776	Cytochrome b-c1 complex subunit 2, mitochondrial	IPI00305383	-2.273	0.00178	15	17	8	7
777	WASH complex subunit strumpellin	IPI00029175	-0.970	0.04753	9	9	7	5
778	7-dehydrocholesterol reductase	IPI00294501	0.531	0.13800	11	10	15	10
779	treacle protein isoform a	IPI00165041	-1.299	0.02029	8	11	5	6
780	Isoform 1 of Cysteine and histidine-rich domain-containing protein 1	IPI00015897	-0.847	0.06260	8	8	4	7
781	Isoform Heart of ATP synthase subunit gamma, mitochondrial	IPI00395769	-1.111	0.03482	9	16	11	6
782	Isoform Short of RNA-binding protein FUS	IPI00221354	-0.579	0.12599	10	11	8	9
783	Eukaryotic translation initiation factor 6	IPI00010105	-1.008	0.04066	15	15	11	11
784	3-ketoacyl-CoA thiolase, mitochondrial	IPI00001539	0.521	0.15816	12	10	12	14
785	Kinesin-like protein KIF11	IPI00305289	4.584	0.00004	0	1	16	10
786	Protein flightless-1 homolog	IPI00031023	1.442	0.01507	5	6	13	7
787	Isoform 1 of LETM1 and EF-hand domain-containing protein 1, mitochondrial	IPI00017592	-0.579	0.12599	11	10	8	9
788	Isoform 1 of Adipocyte plasma membrane-associated protein	IPI00031131	0.451	0.18504	9	7	8	11
789	Calponin-2	IPI00015262	0.000	0.37049	12	11	14	9
790	DNA mismatch repair protein Msh2	IPI00017303	1.066	0.03648	8	11	14	13
791	Copine-1	IPI00018452	0.000	0.37049	8	8	9	7
792	Sepiapterin reductase	IPI00017469	-0.905	0.05325	15	13	10	11
793	Casein kinase II subunit alpha'	IPI00020602	0.645	0.11809	8	5	7	10
794	Isoform 1 of Coiled-coil domain-containing protein 47	IPI00024642	-0.131	0.35773	9	15	12	11
795	Isoform 1 of Inorganic pyrophosphatase 2, mitochondrial	IPI00301109	-1.117	0.03466	10	9	7	5
796	Protein mago nashi homolog 2	IPI00059292	-0.163	0.34436	8	7	11	3
797	DNA polymerase delta catalytic subunit	IPI00002894	2.178	0.00215	5	5	13	11
798	cDNA FLJ60124, highly similar to Mitochondrial dicarboxylate carrier	IPI00005537	-2.045	0.00368	12	11	6	4
799	Isoform 1 of Platelet-activating factor acetylhydrolase IB subunit alpha	IPI00218728	-0.626	0.11788	9	9	6	8
800	Isoform 1 of Caprin-1	IPI00783872	-0.137	0.35558	12	10	10	11
801	Isoform Long of Eukaryotic translation initiation factor 4H	IPI00014263	-0.755	0.07585	13	16	10	13
802	cDNA FLJ59571, highly similar to Eukaryotic translation initiation factor 4gamma 2	IPI00015952	0.609	0.12446	9	6	10	9
803	Pre-mRNA-splicing factor ATP-dependent RNA helicase PRP16	IPI00294211	-0.411	0.19075	11	12	13	7
804	60S ribosomal protein L36	IPI00216237	-2.244	0.00199	17	9	9	2
805	Isoform 1 of Dynamin-2	IPI00033022	-0.752	0.07635	9	11	6	9
806	Putative uncharacterized protein DKFZp451D234	IPI00031583	0.982	0.04517	10	7	13	11
807	Cleavage stimulation factor subunit 3	IPI00015195	0.000	0.37049	9	9	8	10
808	U4/U6.U5 tri-snRNP-associated protein 2	IPI00419844	0.289	0.26859	11	7	9	11
809	Splicing factor, arginine/serine-rich 9	IPI00012340	0.277	0.28271	10	10	12	10
810	Aldose reductase	IPI00413641	-1.121	0.03445	6	8	6	2
811	Coatomer subunit delta variant 2	IPI00298520	-0.795	0.06844	10	8	9	4
812	Core histone macro-H2A.2	IPI00220994	0.420	0.19133	9	10	11	11
813	Putative RNA-binding protein 3	IPI00024320	-1.029	0.03967	13	9	8	7
814	Histone-binding protein RBBP7	IPI00395865	0.645	0.11809	6	7	8	9
815	Isoform 2 of DNA replication licensing factor MCM7	IPI00219740	1.363	0.01805	8	9	12	15
816	Small nuclear ribonucleoprotein E	IPI00029266	-1.164	0.03155	14	9	8	7
817	128 kDa protein	IPI00892839	-0.986	0.04534	4	4	2	2
818	Isoform 1 of Protein phosphatase 1 regulatory subunit 7	IPI00033600	-1.328	0.02012	14	9	8	6
819	60S ribosomal protein L21	IPI00247583	-1.677	0.00816	13	14	8	7
820	Isoform 1 of BH3-interacting domain death agonist	IPI00413587	-1.005	0.04066	14	9	13	3
821	Vacuolar protein sorting-associated protein 26A	IPI00411426	-1.425	0.01524	13	12	6	9
822	Protein dpy-30 homolog	IPI00028109	-2.045	0.00368	12	11	6	4
823	Histone acetyltransferase type B catalytic subunit	IPI00024719	1.393	0.01751	9	7	13	13
824	Eukaryotic translation elongation factor 1 epsilon-1	IPI00003588	-1.506	0.01354	8	7	4	3
825	ADP-ribosylation factor 4	IPI00215918	-0.140	0.35413	11	10	12	8
826	Asparagine synthetase [glutamine-hydrolyzing]	IPI00554777	-5.944	0.00008	19	22	2	1
827	Cytochrome c oxidase subunit 4 isoform 1, mitochondrial	IPI00006579	0.451	0.18504	10	6	12	7
828	Replication factor C subunit 5	IPI00031514	-0.143	0.35301	8	12	10	9
829	SWI/SNF-related matrix-associated actin-dependent regulator of chromatin subfamily A	IPI00297211	0.150	0.35065	6	11	10	8
830	Isoform 2 of Cytoplasmic FMR1-interacting protein 1	IPI00550212	-0.154	0.34838	7	10	8	8
831	Eukaryotic translation initiation factor 3 subunit I	IPI00012795	-1.056	0.03635	13	8	10	4
832	Isoform 2 of Obg-like ATPase 1	IPI00216105	-0.137	0.35558	11	11	9	12
833	Isoform 1 of Cleavage and polyadenylation specificity factor subunit 7	IPI00550821	-0.658	0.11320	12	14	11	10
834	Lysophospholipid acyltransferase 5	IPI00306419	0.163	0.34457	6	8	10	5
835	Sorbitol dehydrogenase	IPI00216057	-1.164	0.03155	13	10	8	7
836	Splicing factor, arginine/serine-rich 2	IPI00005978	-0.554	0.13055	13	10	9	10
837	Protein RCC2	IPI00465044	-0.313	0.24578	10	7	7	8
838	Isoform 1 of Tropomyosin alpha-4 chain	IPI00010779	-0.380	0.20748	14	13	15	9
839	Isoform 1 of 5'(3')-deoxyribonucleotidase, cytosolic type	IPI00005573	-1.640	0.01093	13	15	7	9
840	FKBP1A protein	IPI00413778	0.000	0.37049	8	7	10	5
841	Isoform 1 of Symplekin	IPI00023344	-0.912	0.05316	5	9	5	4
842	Signal recognition particle 54 kDa protein	IPI00009822	-0.832	0.06467	12	12	9	9
843	Isoform 1 of Glucosamine--fructose-6-phosphate aminotransferase [isomerizing] 1	IPI00217952	1.056	0.03677	8	6	11	10
844	Thioredoxin	IPI00216298	-0.429	0.18789	10	11	11	7
845	Vesicle-trafficking protein SEC22b	IPI00006865	-0.531	0.13593	13	12	11	10
846	Mannosyl-oligosaccharide glucosidase	IPI00328170	-0.773	0.07341	10	9	6	8
847	Prolyl endopeptidase	IPI00008164	0.000	0.37049	10	11	11	10
848	Isoform 5 of Thioredoxin reductase 1, cytoplasmic	IPI00554786	0.129	0.35831	13	11	11	14
849	Isoform 1 of RNA-binding protein 14	IPI00013174	-0.688	0.08641	6	9	6	5
850	Isoform SERCA2A of Sarcoplasmic/endoplasmic reticulum calcium ATPase 2	IPI00177817	-0.175	0.33798	4	9	8	4
851	Isoform 2 of Serine/threonine-protein phosphatase PGAM5, mitochondrial	IPI00063242	-1.085	0.03573	10	10	6	7

No.	Description	Accession number	STN[1]	p-Value[1]	Con_A[2]	Con_B[2]	OXA_A[2]	OXA_B[2]
852	DCN1-like protein 5	IPI00165361	-1.056	0.03635	10	11	7	7
853	Alpha-soluble NSF attachment protein	IPI00009253	0.154	0.34892	8	8	10	7
854	Rho-associated protein kinase 2	IPI00307155	-0.163	0.34436	6	9	9	5
855	3-hydroxyisobutyrate dehydrogenase, mitochondrial	IPI00013860	-0.531	0.13593	13	12	11	10
856	Isoform 1 of Peripherin	IPI00013164	-0.146	0.35082	10	9	11	7
857	Ras-related protein Rab-2A	IPI00031169	-0.814	0.06587	13	12	11	8
858	Isoform 1 of E3 UFM1-protein ligase 1	IPI00844000	-1.029	0.03967	10	12	10	5
859	Histone deacetylase 1	IPI00013774	1.285	0.02033	7	13	16	14
860	V-type proton ATPase subunit E 1	IPI00003856	0.313	0.25228	9	6	10	7
861	3-mercaptopyruvate sulfurtransferase	IPI00165360	0.154	0.34892	7	9	9	8
862	Platelet-activating factor acetylhydrolase IB subunit beta	IPI00026546	-0.140	0.35413	11	10	12	8
863	Probable saccharopine dehydrogenase	IPI00329600	0.289	0.26859	8	10	10	10
864	Catalase	IPI00465436	-0.159	0.34738	7	9	9	6
865	Isoform 1 of UTP--glucose-1-phosphate uridylyltransferase	IPI00329331	0.579	0.12914	8	9	11	10
866	Serine/threonine-protein kinase VRK1	IPI00019640	-0.169	0.34159	6	8	10	3
867	Protein NipSnap homolog 1	IPI00304435	-0.140	0.35413	10	11	9	11
868	165 kDa protein	IPI00240812	0.322	0.24627	7	7	10	6
869	Cytochrome c oxidase subunit 5A, mitochondrial	IPI00025086	0.159	0.34718	9	6	10	6
870	Microsomal glutathione S-transferase 3	IPI00024266	0.531	0.13800	11	10	15	10
871	Isoform Short of TATA-binding protein-associated factor 2N	IPI00020194	-0.332	0.23812	6	9	8	5
872	epiplakin	IPI00010951	-1.342	0.01958	6	8	5	2
873	Isoform 2 of Serrate RNA effector molecule homolog	IPI00220038	0.159	0.34718	8	7	9	7
874	Cell growth-regulating nucleolar protein	IPI00015838	-1.117	0.03466	13	6	7	5
875	protein ELYS	IPI00170594	-1.390	0.01805	8	9	8	1
876	Prenylcysteine oxidase 1	IPI00384280	0.476	0.16727	6	8	9	8
877	Regulation of nuclear pre-mRNA domain-containing protein 1B	IPI00009659	-0.154	0.34838	8	9	8	8
878	Ornithine aminotransferase, mitochondrial	IPI00022334	-0.542	0.13519	10	14	12	8
879	DnaJ homolog subfamily A member 1	IPI00012535	0.566	0.13096	8	10	11	11
880	Visinin-like protein 1	IPI00216313	-1.459	0.01445	11	13	8	6
881	Mitochondrial import inner membrane translocase subunit TIM44	IPI00306516	-0.143	0.35301	8	12	10	9
882	DnaJ homolog subfamily C member 13	IPI00307259	1.400	0.01540	6	6	12	9
883	Isoform 1AB of Catenin delta-1	IPI00182469	-1.121	0.03445	6	8	4	4
884	Cytoplasmic dynein 1 light intermediate chain 2	IPI00011592	-0.916	0.05300	9	11	8	6
885	Proteasome subunit beta type-5	IPI00479306	0.000	0.37049	10	13	12	11
886	Isoform 2 of Cat eye syndrome critical region protein 5	IPI00011511	-0.847	0.06260	7	9	4	7
887	Kinesin-1 heavy chain	IPI00012837	0.154	0.34892	6	10	7	10
888	Ras suppressor protein 1	IPI00017256	-1.121	0.03445	7	7	7	0
889	60S ribosomal protein L18	IPI00215719	-2.492	0.00108	14	17	6	7
890	Isoform 2 of Inverted formin-2	IPI00876962	-1.393	0.01805	15	11	8	8
891	High mobility group protein B3	IPI00217477	-0.850	0.06161	12	11	6	11
892	Isoform 1 of U2-associated protein SR140	IPI00143753	-1.152	0.03167	10	8	5	6
893	UPF0160 protein MYG1, mitochondrial	IPI00029444	-0.970	0.04753	10	8	4	8
894	Putative uncharacterized protein MDH1	IPI00915869	0.000	0.37049	7	12	10	9
895	Ubiquitin-conjugating enzyme E2 L3	IPI00021347	-0.970	0.04753	9	9	7	5
896	ADP/ATP translocase 1	IPI00022891	-0.743	0.07647	8	5	7	2
897	Isoform 2 of Microtubule-associated protein 4	IPI00220113	-2.588	0.00095	12	12	4	4
898	Isoform 2 of Double-stranded RNA-specific adenosine deaminase	IPI00025057	-0.387	0.20665	4	7	5	4
899	Mitochondrial 28S ribosomal protein S2	IPI00006970	-0.545	0.13328	7	6	5	5
900	SUMO-conjugating enzyme UBC9	IPI00032957	-0.645	0.11390	9	8	7	6
901	cDNA FLJ56285, highly similar to ADP-ribosylation factor-like protein 8B	IPI00018871	-0.714	0.08186	7	7	6	4
902	Paladin	IPI00297212	-0.491	0.16438	7	9	5	8
903	Polyribonucleotide nucleotidyltransferase 1, mitochondrial	IPI00744711	-0.579	0.12599	10	11	8	9
904	Protein NipSnap homolog 2	IPI00016077	-0.451	0.18446	9	10	8	8
905	Carbonyl reductase [NADPH] 1	IPI00295386	0.429	0.18963	9	9	12	9
906	Eukaryotic translation initiation factor 5A-2	IPI00006935	-1.193	0.03126	13	9	10	4
907	regulator of differentiation 1 isoform 2	IPI00159072	0.765	0.07536	2	2	2	5
908	Anaphase-promoting complex subunit 1	IPI00033907	-0.986	0.04534	3	5	3	1
909	Isoform 2 of Nucleoporin NUP188 homolog	IPI00385001	1.585	0.01126	3	1	6	5
910	Isoform 1 of Cytoplasmic FMR1-interacting protein 1	IPI00644231	0.716	0.08037	6	11	13	9
911	Protein VAC14 homolog	IPI00025160	-0.253	0.29716	2	4	3	2
912	Isoform 1 of Ubiquitin-conjugating enzyme E2 variant 1	IPI00019599	0.266	0.28664	12	10	15	9
913	Cation-independent mannose-6-phosphate receptor	IPI00289819	0.322	0.24627	7	7	8	8
914	Eukaryotic translation initiation factor 3 subunit D	IPI00006181	0.175	0.33894	6	6	6	7
915	Transmembrane emp24 domain-containing protein 2	IPI00016608	-0.175	0.33798	6	7	6	6
916	Superoxide dismutase [Mn], mitochondrial	IPI00022314	0.277	0.28271	9	11	11	11
917	Glutaredoxin-3	IPI00008552	-0.313	0.24578	7	10	7	8
918	Isoform 5 of Myosin-14	IPI00029818	-0.568	0.12806	7	5	6	3
919	cDNA FLJ61739, highly similar to Serine/arginine repetitive matrix protein 1	IPI00328293	-1.002	0.04087	7	10	5	6
920	Isoform 2 of Tropomyosin alpha-3 chain	IPI00218319	-0.313	0.24578	9	8	7	8
921	Isoform Crk-II of Adapter molecule crk	IPI00004838	-0.159	0.34738	7	9	7	8
922	TDP43	IPI00025815	-0.146	0.35082	10	9	8	10
923	Condensin complex subunit 3	IPI00106495	1.758	0.00584	8	5	13	12
924	Isoform 3 of Serine/threonine-protein phosphatase 2A activator	IPI00217296	-1.400	0.01553	12	9	7	5
925	Aminoacyl tRNA synthase complex-interacting multifunctional protein 2	IPI00011916	-0.491	0.16438	6	10	7	6
926	Mitochondrial-processing peptidase subunit alpha	IPI00166749	-0.343	0.23683	9	5	7	5
927	Proline synthetase co-transcribed homolog (Bacterial), isoform CRA_b	IPI00016346	0.451	0.18504	8	8	11	8
928	Isoform 2 of AP-2 complex subunit alpha-2	IPI00016621	0.714	0.08070	6	4	7	7
929	DNA-directed RNA polymerase I subunit RPA1	IPI00031960	1.121	0.03341	4	4	7	7
930	Pre-mRNA-processing factor 19	IPI00004968	-0.645	0.11390	10	7	8	5
931	von Hippel-Lindau binding protein 1, isoform CRA_b	IPI00334159	-0.283	0.27141	9	12	11	8
932	coatomer subunit epsilon isoform b	IPI00399318	0.146	0.35161	8	10	11	8
933	Vitamin K epoxide reductase complex subunit 1-like protein 1	IPI00166079	-1.117	0.03466	12	7	10	2
934	Isoform LAMP-2A of Lysosome-associated membrane glycoprotein 2	IPI00009030	-0.406	0.20280	3	7	4	4
935	Isoform 1 of Heterogeneous nuclear ribonucleoprotein U-like protein 1	IPI00013070	-0.773	0.07341	10	9	8	6
936	Eukaryotic translation initiation factor 3 subunit G	IPI00290460	0.313	0.25228	6	9	7	10
937	cohesin subunit SA-2 isoform a	IPI00470883	0.626	0.11941	6	8	10	8
938	histone deacetylase complex subunit SAP18	IPI00011698	-0.734	0.07743	10	11	8	8
939	N(G),N(G)-dimethylarginine dimethylaminohydrolase 2	IPI00000760	1.005	0.04037	8	8	12	11
940	Isoform 1 of Kinectin	IPI00328753	-1.442	0.01520	9	11	7	4
941	Dolichol-phosphate mannosyltransferase	IPI00022018	0.313	0.25228	8	7	11	6
942	Isoform 1 of Replication factor C subunit 2	IPI00017412	-0.734	0.07743	9	12	8	8
943	Alkyldihydroxyacetonephosphate synthase, peroxisomal	IPI00010349	0.451	0.18504	8	8	10	9
944	EH domain-containing protein 4	IPI00005578	0.000	0.37049	8	11	11	8
945	Isoform 1 of Peroxisomal acyl-coenzyme A oxidase 1	IPI00296907	1.152	0.03225	7	4	9	9
946	Isoform 2 of Leucyl-cystinyl aminopeptidase	IPI00221240	0.150	0.35065	6	11	10	8

No.	Description	Accession number	STN[1]	p-Value[1]	Con_A[2]	Con_B[2]	OXA_A[2]	OXA_B[2]
947	Isoform 2 of U1 small nuclear ribonucleoprotein 70 kDa	IPI00219483	-0.878	0.05474	5	10	5	5
948	Ran GTPase-activating protein 1	IPI00294879	1.972	0.00356	4	5	11	10
949	Isoform 1 of Vesicle-associated membrane protein-associated protein A	IPI00170692	-0.942	0.05060	8	11	9	4
950	Probable ATP-dependent RNA helicase DDX23	IPI00006725	1.121	0.03341	4	4	6	8
951	Probable cysteinyl-tRNA synthetase, mitochondrial	IPI00336016	-0.795	0.06844	7	11	7	6
952	Membrane-associated progesterone receptor component 1	IPI00220739	-1.111	0.03482	12	13	9	8
953	Barrier-to-autointegration factor	IPI00026087	-1.056	0.03635	12	9	10	4
954	Isoform 3 of Glutaminase kidney isoform, mitochondrial	IPI00215687	0.000	0.37049	9	8	7	10
955	Biliverdin reductase A	IPI00294158	-0.878	0.05474	6	9	8	2
956	cDNA FLJ35809 fis, clone TESTI2006016	IPI00647650	0.322	0.24627	7	7	8	8
957	Isoform Long of Long-chain-fatty-acid--CoA ligase 4	IPI00029737	0.150	0.35065	6	11	10	8
958	Isoform 3 of Obg-like ATPase 1	IPI00216106	-2.785	0.00091	16	16	6	6
959	Isoform 1 of Annexin A7	IPI00002460	-1.005	0.04066	12	11	9	7
960	Eukaryotic translation initiation factor 1A, Y-chromosomal	IPI00023004	-1.538	0.01321	11	11	7	5
961	Coatomer subunit zeta-1	IPI00032851	0.297	0.26789	10	7	12	7
962	cDNA FLJ56414, highly similar to Homo sapiens proline-, glutamic acid-, leucine-rich protein	IPI00006702	-1.037	0.03938	7	9	4	6
963	Cytoplasmic dynein 1 light intermediate chain 1	IPI00007675	-1.497	0.01354	11	12	6	7
964	Isoform Epsilon of Apoptosis regulator BAX	IPI00071059	-0.137	0.35558	12	10	11	10
965	Calpain-2 catalytic subunit	IPI00289758	1.672	0.00737	4	8	11	12
966	DnaJ homolog subfamily A member 2	IPI00032406	-0.297	0.26664	8	11	9	8
967	Heterogeneous nuclear ribonucleoprotein H2	IPI00026230	-1.672	0.00816	10	13	5	7
968	Isoform 2 of Proteasome subunit alpha type-3	IPI00171199	-0.832	0.06467	11	13	9	9
969	Ras-related protein Rab-35	IPI00300096	-1.260	0.02157	10	10	6	6
970	Putative uncharacterized protein ATP5J2	IPI00219291	-1.910	0.00414	11	11	8	2
971	cDNA FLJ50992, highly similar to Coronin-1C	IPI00798401	-0.626	0.12011	5	5	5	2
972	Stathmin	IPI00479997	0.000	0.37049	7	6	8	5
973	cDNA FLJ55586, highly similar to MMS19-like protein	IPI00154451	0.000	0.37049	9	3	5	7
974	Thioredoxin-related transmembrane protein 1	IPI00395887	0.609	0.12446	10	5	13	6
975	triosephosphate isomerase 1 isoform 2	IPI00465028	0.428	0.19017	3	4	4	5
976	Exosome complex exonuclease RRP4	IPI00015905	-1.056	0.03635	12	9	8	6
977	Pyruvate carboxylase, mitochondrial	IPI00299402	2.116	0.00219	1	3	5	9
978	Nuclear transport factor 2	IPI00009901	-0.595	0.12310	8	3	5	3
979	Very long-chain acyl-CoA synthetase	IPI00024787	-1.285	0.02058	8	7	7	1
980	Isoform Short of Glycylpeptide N-tetradecanoyltransferase 1	IPI00218830	-1.295	0.02029	12	12	8	7
981	S-adenosylmethionine synthase isoform type-2	IPI00010157	-0.773	0.07341	8	11	7	7
982	Pre-mRNA-processing factor 6	IPI00305068	-0.154	0.34838	9	8	9	7
983	Isoform 2 of Phosphoglucomutase-1	IPI00217872	0.734	0.07933	8	8	13	8
984	Cytochrome c-type heme lyase	IPI00023406	0.000	0.37049	6	7	11	2
985	Isochorismatase domain-containing protein 1	IPI00304082	-1.688	0.00787	9	10	6	3
986	Proteasome subunit beta type-7	IPI00003217	0.463	0.16984	9	6	9	9
987	Ras-related protein Rab-14	IPI00291928	-0.916	0.05300	11	9	8	6
988	ATP synthase subunit O, mitochondrial	IPI00007611	-0.146	0.35082	12	7	10	8
989	Isoform 1 of KH domain-containing, RNA-binding, signal transduction-associated protein 1	IPI00008575	-0.525	0.15692	7	7	6	5
990	Junction plakoglobin	IPI00554711	0.916	0.05271	3	2	6	3
991	Glucosamine 6-phosphate N-acetyltransferase	IPI00061525	-0.451	0.18446	11	8	10	6
992	NADH dehydrogenase [ubiquinone] iron-sulfur protein 8, mitochondrial	IPI00010845	-0.714	0.08186	9	5	8	2
993	28S ribosomal protein S22, mitochondrial	IPI00013146	1.037	0.03768	1	9	9	7
994	Isoform 1 of Core-binding factor subunit beta	IPI00016746	-0.146	0.35082	9	10	8	10
995	COP9 signalosome complex subunit 3	IPI00025721	-0.343	0.23683	5	9	5	7
996	Isoform Alpha of Signal transducer and activator of transcription 1-alpha/beta	IPI00030781	1.972	0.00356	4	5	10	11
997	Isoform 1 of Bcl-2-associated transcription factor 1	IPI00006079	-0.626	0.11788	9	9	6	8
998	Isoform 1 of Fanconi anemia group I protein	IPI00019447	1.050	0.03689	4	2	7	4
999	Putative uncharacterized protein RPL17	IPI00394699	-0.181	0.33571	6	6	6	5
1000	Putative uncharacterized protein ENSP00000382160	IPI00180956	0.163	0.34457	9	5	10	5
1001	Isoform 1 of Spermatid perinuclear RNA-binding protein	IPI00169430	-1.193	0.03126	12	10	9	5
1002	Lysosomal alpha-glucosidase	IPI00293088	-1.076	0.03586	7	8	8	1
1003	Guanine nucleotide-binding protein subunit alpha-13	IPI00290928	-0.440	0.18628	11	9	7	10
1004	EH domain-containing protein 1	IPI00017184	-0.356	0.22868	6	7	7	4
1005	Cullin-5	IPI00216003	-0.356	0.22868	6	7	5	6
1006	Isoform 1 of Acylglycerol kinase, mitochondrial	IPI00019353	-0.175	0.33798	8	5	5	7
1007	Signal recognition particle 72 kDa protein	IPI00215888	-0.154	0.34838	8	9	7	9
1008	2,4-dienoyl-CoA reductase, mitochondrial	IPI00003482	-0.714	0.08186	8	6	7	3
1009	Isoform 1 of Probable threonyl-tRNA synthetase 2, cytoplasmic	IPI00328082	-1.758	0.00654	13	12	7	6
1010	Isoform 1 of 39S ribosomal protein L4, mitochondrial	IPI00023334	-0.332	0.23812	7	8	9	4
1011	Importin subunit alpha-4	IPI00012578	0.181	0.33562	7	4	7	5
1012	Eukaryotic peptide chain release factor GTP-binding subunit ERF3A	IPI00218829	0.525	0.15696	6	5	6	8
1013	Isoform 1 of SAM domain and HD domain-containing protein 1	IPI00294739	1.191	0.03180	4	6	10	7
1014	Serine/threonine-protein phosphatase 2A catalytic subunit alpha isoform	IPI00008380	-0.594	0.12525	9	11	8	8
1015	Isoform 1 of Calcium-binding mitochondrial carrier protein SCaMC-1	IPI00337494	0.645	0.11809	6	7	8	9
1016	Exportin-7	IPI00302458	1.583	0.01126	4	7	7	14
1017	Putative uncharacterized protein FUBP3	IPI00063245	0.169	0.34208	7	6	7	7
1018	Nucleolar complex protein 3 homolog	IPI00102815	-0.970	0.04753	12	6	9	3
1019	ERO1-like protein alpha	IPI00386755	0.000	0.37049	9	9	9	9
1020	Isoform 1 of Transcription elongation factor SPT5	IPI00298058	0.000	0.37049	5	7	6	6
1021	myosin regulatory light polypeptide 9 isoform b	IPI00030929	-0.850	0.06161	10	13	9	8
1022	Actin-related protein 2/3 complex subunit 2	IPI00005161	-1.037	0.03938	9	7	6	4
1023	60S ribosomal protein L38	IPI00215790	-0.714	0.08186	8	6	5	5
1024	Mitogen-activated protein kinase 1	IPI00003479	0.626	0.11941	6	8	8	10
1025	Isoform 1 of Proteasome activator complex subunit 4	IPI00005260	0.000	0.37049	7	7	9	5
1026	NADPH--cytochrome P450 reductase	IPI00470467	1.390	0.01780	3	6	9	8
1027	Isoform SERCA1B of Sarcoplasmic/endoplasmic reticulum calcium ATPase 1	IPI00024804	-0.609	0.12148	10	9	8	7
1028	Eukaryotic translation initiation factor 2 subunit 2	IPI00021728	-0.566	0.12989	12	10	9	9
1029	Isoleucyl-tRNA synthetase	IPI00514082	-1.117	0.03466	10	9	7	5
1030	Isoform Long of Antigen KI-67	IPI00004233	-0.235	0.30503	3	4	3	3
1031	NADH dehydrogenase [ubiquinone] 1 alpha subcomplex subunit 10, mitochondrial	IPI00029561	-1.191	0.03143	7	10	6	4
1032	Chloride intracellular channel protein 4	IPI00001960	-1.540	0.01255	10	8	4	5
1033	Isoform 1 of Insulin-like growth factor 2 mRNA-binding protein 2	IPI00179713	-0.773	0.07341	10	9	8	6
1034	Carnitine O-palmitoyltransferase 2, mitochondrial	IPI00012912	0.912	0.05403	4	5	7	7
1035	Dual specificity mitogen-activated protein kinase kinase 2	IPI00003783	-0.743	0.07647	6	7	5	4
1036	SAP domain-containing ribonucleoprotein	IPI00014938	-0.685	0.08670	10	14	9	10
1037	Isoform Alpha-6X1X2B of Integrin alpha-6	IPI00010697	0.734	0.07933	10	6	12	9
1038	Tubulin-specific chaperone A	IPI00217236	-0.795	0.06844	9	9	6	7
1039	WD repeat-containing protein 61	IPI00019269	-0.594	0.12525	10	10	9	7
1040	Pirin	IPI00012575	-1.742	0.00716	8	7	2	4
1041	Chromobox protein homolog 3	IPI00297579	-0.143	0.35301	11	9	10	9

No.	Description	Accession number	STN[1]	p-Value[1]	Con_A[2]	Con_B[2]	OXA_A[2]	OXA_B[2]
1042	Acyl-CoA dehydrogenase family member 9, mitochondrial	IPI00152981	0.356	0.23584	4	7	8	5
1043	Quinone oxidoreductase	IPI00000792	-0.645	0.11390	10	7	8	5
1044	S-formylglutathione hydrolase	IPI00411706	-1.665	0.01068	8	8	3	4
1045	Intron-binding protein aquarius	IPI00297572	1.305	0.02021	4	1	7	4
1046	Isoform 1 of Abhydrolase domain-containing protein 14B	IPI00063827	-0.626	0.11788	9	9	7	7
1047	Proteasome inhibitor PI31 subunit	IPI00009949	-0.666	0.11121	7	9	6	6
1048	Isoform 1 of Translocon-associated protein subunit alpha	IPI00301021	-1.720	0.00749	10	12	7	4
1049	Isoform Long of ES1 protein homolog, mitochondrial	IPI00024913	0.181	0.33562	5	6	6	6
1050	baculoviral IAP repeat-containing protein 6	IPI00299635	0.530	0.13800	3	1	5	1
1051	Nuclear RNA export factor 1	IPI00033153	1.362	0.01805	6	7	13	9
1052	Heme oxygenase 2	IPI00026824	-1.506	0.01354	7	8	3	4
1053	Protein S100-A6	IPI00027463	1.394	0.01540	3	1	8	2
1054	Isoform 1 of Cleavage stimulation factor subunit 2	IPI00013256	-0.428	0.18789	4	5	3	4
1055	Isocitrate dehydrogenase [NADP] cytoplasmic	IPI00027223	-0.626	0.11788	9	9	7	7
1056	Phosphatidylinositol transfer protein alpha isoform	IPI00216048	0.000	0.37049	6	7	7	6
1057	Thimet oligopeptidase	IPI00549189	0.776	0.07022	5	3	6	6
1058	Isoform 2 of Mediator of DNA damage checkpoint protein 1	IPI00470805	1.235	0.02186	4	5	10	6
1059	145 kDa protein	IPI00218097	1.665	0.00994	3	4	7	9
1060	87 kDa protein	IPI00220365	-1.999	0.00389	6	9	3	2
1061	60S ribosomal protein L26-like 1	IPI00007144	-0.714	0.08186	6	8	6	4
1062	Isoform 1 of Enoyl-CoA hydratase domain-containing protein 1	IPI00302688	-0.916	0.05300	13	7	7	7
1063	Growth hormone inducible transmembrane protein	IPI00549970	-0.708	0.08447	3	5	4	1
1064	Isoform 1 of Gamma-glutamylcyclotransferase	IPI00031564	0.143	0.35293	9	10	11	9
1065	Isoform NELF-C of Negative elongation factor C/D	IPI00164949	-1.260	0.02157	9	11	6	6
1066	cDNA FLJ59367, highly similar to Adenylosuccinate lyase	IPI00026904	0.000	0.37049	7	5	6	6
1067	Isoform 1 of Calcineurin-like phosphoesterase domain-containing protein 1	IPI00305010	-1.085	0.03573	11	9	10	3
1068	Niban-like protein 1	IPI00456750	0.525	0.15696	5	6	9	5
1069	Testis-expressed sequence 10 protein	IPI00549664	-0.189	0.33144	5	6	5	5
1070	Eukaryotic translation initiation factor 3 subunit J	IPI00290461	-0.912	0.05316	5	9	4	5
1071	Basic leucine zipper and W2 domain-containing protein 2	IPI00022305	-0.595	0.12310	5	6	5	3
1072	Keratin, type II cytoskeletal 6B	IPI00293665	0.000	0.37049	1	0	1	0
1073	Transmembrane protein 43	IPI00301280	-0.370	0.21613	5	7	5	5
1074	Crk-like protein	IPI00004839	-0.688	0.08641	10	5	6	5
1075	39S ribosomal protein L28, mitochondrial	IPI00172594	-1.540	0.01255	11	7	6	3
1076	NADH dehydrogenase [ubiquinone] iron-sulfur protein 2, mitochondrial	IPI00025239	1.742	0.00658	2	4	8	7
1077	Transcription factor BTF3 homolog 4	IPI00412792	-2.182	0.00215	11	10	7	1
1078	Inosine triphosphate pyrophosphatase	IPI00018783	-0.322	0.24491	8	8	7	7
1079	Eukaryotic translation initiation factor 5	IPI00022648	-0.154	0.34838	8	9	9	7
1080	Activating signal cointegrator 1 complex subunit 3	IPI00430472	-0.220	0.31447	5	3	6	1
1081	Selenide, water dikinase 1	IPI00029056	-0.595	0.12310	3	8	2	6
1082	Ribosome maturation protein SBDS	IPI00427330	-0.568	0.12806	7	5	4	5
1083	Isoform 2 of tRNA pseudouridine synthase A	IPI00001716	-1.121	0.03445	8	6	5	3
1084	Isoform Long of Double-stranded RNA-binding protein Staufen homolog 1	IPI00000001	0.154	0.34892	9	7	9	8
1085	Methylenetetrahydrofolate dehydrogenase (NADP+ dependent) 1-like	IPI00291646	-2.045	0.00368	12	11	5	5
1086	Isoform 2 of Tumor protein D54	IPI00221178	-2.308	0.00178	13	12	5	5
1087	Isoform 1 of Pre-mRNA-processing factor 40 homolog A	IPI00337385	1.632	0.01039	6	4	10	10
1088	N-alpha-acetyltransferase 38, NatC auxiliary subunit	IPI00219871	-1.285	0.02058	6	9	3	5
1089	Ribosome biogenesis protein WDR12	IPI00304232	-0.847	0.06260	6	10	3	8
1090	sorting nexin-1 isoform c	IPI00183274	-0.688	0.08641	8	7	8	3
1091	Isoform 1 of Phosphatidylinositol transfer protein beta isoform	IPI00334907	0.181	0.33562	6	5	7	5
1092	Isoform p150 of Dynactin subunit 1	IPI00029485	0.714	0.08070	5	5	8	6
1093	Developmentally-regulated GTP-binding protein 1	IPI00031836	-0.507	0.15999	7	8	7	5
1094	ribonucleoprotein PTB-binding 1	IPI00217661	0.986	0.04066	1	3	4	4
1095	Dihydropteridine reductase	IPI00014439	-0.752	0.07635	10	10	8	7
1096	ATP-dependent RNA helicase DHX29	IPI00217413	0.189	0.32929	5	5	7	4
1097	Isoform 2 of Cytosolic non-specific dipeptidase	IPI00165579	-0.387	0.20665	7	4	4	5
1098	26S proteasome non-ATPase regulatory subunit 5	IPI00002134	0.666	0.11150	4	8	5	11
1099	Isoform 2 of cAMP-dependent protein kinase catalytic subunit alpha	IPI00217960	0.000	0.37049	6	7	6	7
1100	cDNA FLJ55177, highly similar to Ras-related protein Ral-B	IPI00004397	0.163	0.34457	6	8	8	7
1101	Monocarboxylate transporter 1	IPI00024650	-0.311	0.26205	2	2	1	2
1102	Isoform 1 of Phosphatidate cytidylyltransferase 2	IPI00032150	-0.942	0.05060	10	9	7	6
1103	Isoform 3 of Nucleoporin NDC1	IPI00074330	-0.708	0.08447	5	3	4	1
1104	Isoform 1 of Cirhin	IPI00239815	0.595	0.12525	4	4	7	4
1105	Importin 5	IPI00514205	1.193	0.03126	5	9	11	11
1106	39S ribosomal protein L13, mitochondrial	IPI00022403	-1.235	0.02182	8	8	7	2
1107	40S ribosomal protein S19	IPI00215780	-1.720	0.00749	13	9	8	3
1108	Vesicular integral-membrane protein VIP36	IPI00009950	-1.260	0.02157	11	9	5	7
1109	Chromobox protein homolog 5	IPI00024662	-0.626	0.11788	10	8	7	7
1110	Vacuolar protein-sorting-associated protein 25	IPI00031655	-1.342	0.01958	8	6	6	1
1111	Isoform 1 of Spermine synthase	IPI00005102	-0.181	0.33571	5	7	4	7
1112	Isoform 1 of Putative ATP-dependent RNA helicase DHX30	IPI00411733	0.861	0.05730	3	3	8	2
1113	Isoform 1 of 1-phosphatidylinositol-4,5-bisphosphate phosphodiesterase gamma-1	IPI00016736	1.191	0.03180	6	4	9	8
1114	Neprilysin	IPI00247063	1.972	0.00356	3	6	11	10
1115	Ataxin-10	IPI00001636	-0.304	0.26602	10	8	9	7
1116	Mannose-P-dolichol utilization defect 1 protein	IPI00025292	-0.220	0.31447	4	4	5	2
1117	Putative uncharacterized protein ZFR	IPI00748303	0.313	0.25228	8	7	6	11
1118	cDNA FLJ55988, highly similar to RNA-binding protein Luc7-like 2	IPI00006932	-0.304	0.26602	8	10	8	8
1119	pyrroline-5-carboxylate reductase 1, mitochondrial isoform 2	IPI00376503	-1.225	0.02323	12	9	7	6
1120	cDNA FLJ53927, highly similar to Beta-hexosaminidase alpha chain	IPI00027851	-0.776	0.07324	5	7	3	5
1121	Isoform 1 of CUGBP Elav-like family member 1	IPI00034015	-0.773	0.07341	10	9	7	7
1122	COP9 signalosome complex subunit 8	IPI00009480	-0.491	0.16438	9	7	9	4
1123	Nuclear pore complex protein Nup107	IPI00028005	0.820	0.06550	6	6	8	9
1124	Argininosuccinate synthase	IPI00020632	-2.358	0.00145	10	9	2	4
1125	Isoform 1 of UBX domain-containing protein 1	IPI00027378	-0.568	0.12806	6	6	4	5
1126	51 kDa protein	IPI00033025	0.181	0.33562	6	5	5	7
1127	Synaptic vesicle membrane protein VAT-1 homolog	IPI00156689	-0.343	0.23683	8	6	8	4
1128	Isocitrate dehydrogenase [NADP], mitochondrial	IPI00011107	-0.175	0.33798	6	7	7	5
1129	Translation initiation factor eIF-2B subunit alpha	IPI00221300	-0.343	0.23683	7	7	7	5
1130	Isovaleryl-CoA dehydrogenase, mitochondrial	IPI00645805	-1.121	0.03445	5	9	3	5
1131	Isoform 3 of DNA repair protein RAD50	IPI00107531	0.370	0.20967	5	5	6	6
1132	Transmembrane protein 33	IPI00299084	-0.545	0.13328	7	6	8	2
1133	tropomyosin alpha-1 chain isoform 2	IPI00000230	0.451	0.18504	7	9	10	9
1134	60S acidic ribosomal protein P1	IPI00008527	-0.609	0.12148	8	11	8	7
1135	Isoform 2 of Nipped-B-like protein	IPI00026466	0.189	0.32929	6	4	7	4
1136	Isoform 3 of LIM domain only protein 7	IPI00291802	-3.991	0.00050	17	10	2	2

No.	Description	Accession number	STN[1]	p-Value[1]	Con_A[2]	Con_B[2]	OXA_A[2]	OXA_B[2]
1137	Isoform 2 of ATP-binding cassette sub-family F member 1	IPI00013495	0.000	0.37049	6	8	6	8
1138	39S ribosomal protein L46, mitochondrial	IPI00023161	-0.847	0.06260	7	9	6	5
1139	Palmitoyl-protein thioesterase 1	IPI00002412	-0.181	0.33571	7	5	8	3
1140	Isoform 1 of Dual specificity mitogen-activated protein kinase kinase 3	IPI00218857	0.000	0.37049	7	5	7	5
1141	Inositol monophosphatase 1	IPI00020906	-0.645	0.11390	6	11	9	4
1142	regulator of chromosome condensation 1 isoform a	IPI00001661	-0.491	0.16438	7	9	7	6
1143	Isoform 5 of Protein polybromo-1	IPI00023097	1.487	0.01325	4	1	6	6
1144	Annexin A1	IPI00218918	-0.609	0.12148	10	9	8	7
1145	Mitochondrial import receptor subunit TOM34	IPI00009946	-0.688	0.08641	7	8	5	6
1146	Tyrosine-protein phosphatase non-receptor type 1	IPI00297261	0.000	0.37049	5	7	6	6
1147	Probable ATP-dependent RNA helicase DDX47	IPI00023972	-1.632	0.01143	11	9	7	3
1148	28S ribosomal protein S29, mitochondrial	IPI00018120	0.343	0.23774	8	4	4	10
1149	Isoform 4 of Tubulin-specific chaperone D	IPI00030774	1.342	0.01971	3	4	8	6
1150	cDNA FLJ53975, highly similar to Acetyl-CoA acetyltransferase, cytosolic	IPI00291419	-0.491	0.16438	8	8	8	5
1151	Isoform 1 of Acyl-coenzyme A thioesterase 2, mitochondrial	IPI00220906	0.356	0.23584	5	6	7	6
1152	cDNA FLJ56153, highly similar to Homo sapiens transforming growth factor beta regulator 4	IPI00329625	0.000	0.37049	5	8	9	4
1153	Peroxisomal multifunctional enzyme type 2	IPI00019912	0.847	0.06306	5	6	9	7
1154	Cystatin-B	IPI00021828	-0.163	0.34436	7	8	7	7
1155	Bleomycin hydrolase	IPI00219575	0.842	0.06364	2	1	3	3
1156	Ubiquitin-conjugating enzyme E2 O	IPI00783378	1.078	0.03610	0	2	3	4
1157	Peroxisomal membrane protein PMP34	IPI00014440	1.901	0.00414	1	2	10	1
1158	Isoform 1 of Fragile X mental retardation syndrome-related protein 1	IPI00016249	0.912	0.05403	5	4	7	7
1159	Translocon-associated protein subunit delta precursor	IPI00019385	0.181	0.33562	5	6	9	3
1160	Spermidine synthase	IPI00292020	-1.076	0.03586	8	7	6	3
1161	Exportin-5	IPI00640703	0.776	0.07022	4	4	7	5
1162	Ribosomal protein S6 kinase alpha-3	IPI00020898	0.370	0.20967	5	5	8	4
1163	Isoform 1 of AP-2 complex subunit beta	IPI00784156	0.322	0.24627	8	6	7	9
1164	Acylamino-acid-releasing enzyme	IPI00337741	-1.442	0.01520	10	10	8	3
1165	Isoform 1 of Transcription elongation regulator 1	IPI00247871	-1.305	0.02021	5	6	3	2
1166	Glyoxylate reductase/hydroxypyruvate reductase	IPI00037448	-0.545	0.13328	7	6	5	5
1167	Isoform 1 of General transcription factor 3C polypeptide 1	IPI00414482	0.861	0.05730	2	4	5	5
1168	Protein FAM49B	IPI00303318	0.743	0.07850	6	3	7	6
1169	Sialic acid synthase	IPI00147874	-0.175	0.33798	7	6	7	5
1170	Platelet-activating factor acetylhydrolase IB subunit gamma	IPI00014808	0.356	0.23584	6	5	5	8
1171	FAS-associated factor 2	IPI00172656	0.175	0.33894	5	7	5	8
1172	Isoform 1 of Acyl-CoA-binding protein	IPI00010182	0.000	0.37049	7	4	6	5
1173	Isoform 1 of Polyadenylate-binding protein 2	IPI00005792	-0.815	0.06587	5	6	5	2
1174	Isoform 4 of Serine/threonine-protein phosphatase 6 regulatory subunit 3	IPI00019540	-0.181	0.33571	6	6	5	6
1175	Isoform 1 of PC4 and SFRS1-interacting protein	IPI00028122	-1.394	0.01772	7	3	2	2
1176	Oxysterol-binding protein	IPI00163644	1.121	0.03341	5	3	6	8
1177	Isoform 1 of COP9 signalosome complex subunit 7b	IPI00009301	-0.507	0.15999	7	8	6	6
1178	Isoform 1 of RNA-binding protein Musashi homolog 2	IPI00073713	-0.146	0.35082	10	9	6	12
1179	Protein FAM98B	IPI00167572	0.645	0.11809	6	7	8	9
1180	Small nuclear ribonucleoprotein Sm D3	IPI00017964	0.220	0.31215	4	3	5	3
1181	Thioredoxin domain-containing protein 12	IPI00026328	-0.343	0.23683	8	6	7	5
1182	Cytosolic purine 5'-nucleotidase	IPI00029054	0.951	0.05080	3	5	6	7
1183	Talin-2	IPI00219299	-1.599	0.01155	8	9	5	3
1184	Isoform 1 of Caldesmon	IPI00014516	-1.299	0.02029	4	4	1	2
1185	Isoform SCPx of Non-specific lipid-transfer protein	IPI00026105	0.189	0.32929	7	3	6	5
1186	Mimitin, mitochondrial	IPI00031109	0.000	0.37049	4	3	4	3
1187	Rho GTPase-activating protein 1	IPI00020567	0.000	0.37049	3	5	4	4
1188	GrpE protein homolog 1, mitochondrial	IPI00029557	-0.491	0.16438	7	9	5	8
1189	perilipin-3 isoform 3	IPI00106668	0.491	0.16421	7	6	7	9
1190	Myosin-Ie	IPI00329672	1.037	0.03768	6	4	8	8
1191	NADH-ubiquinone oxidoreductase chain 5	IPI00008511	0.235	0.30171	2	4	4	3
1192	Isoform 1 of Fermitin family homolog 2	IPI00000856	-1.750	0.00687	9	9	7	1
1193	Acidic leucine-rich nuclear phosphoprotein 32 family member C	IPI00018262	-0.189	0.33144	4	7	4	6
1194	Aminoacyl tRNA synthase complex-interacting multifunctional protein 1	IPI00006252	0.208	0.31861	5	3	5	4
1195	Isoform 2 of ATPase family AAA domain-containing protein 3A	IPI00295992	-1.295	0.02029	11	13	8	7
1196	Prefoldin subunit 5	IPI00015361	0.000	0.37049	8	5	7	6
1197	Omega-amidase NIT2	IPI00549467	-0.743	0.07647	7	6	7	2
1198	Isoform 1 of Oxysterol-binding protein 1	IPI00024971	-0.626	0.11788	10	8	6	8
1199	UV excision repair protein RAD23 homolog B	IPI00008223	-0.175	0.33798	5	8	7	5
1200	Isoform 2 of Dedicator of cytokinesis protein 7	IPI00183572	0.220	0.31215	5	2	6	2
1201	Acidic leucine-rich nuclear phosphoprotein 32 family member A	IPI00025849	-0.175	0.33798	8	5	8	4
1202	tropomyosin alpha-3 chain isoform 1	IPI00183968	0.154	0.34892	9	7	8	9
1203	Phosphoglucomutase-2	IPI00550364	0.387	0.20826	5	4	6	5
1204	Isoform 1 of 60S ribosomal protein L12	IPI00024933	-0.666	0.11121	8	8	6	6
1205	AP-1 complex subunit mu-1	IPI00032516	0.545	0.13436	5	5	7	6
1206	Protein kinase, cAMP-dependent, regulatory, type II, alpha, isoform CRA_b	IPI00063234	0.000	0.37049	8	8	9	7
1207	Nucleolar GTP-binding protein 1	IPI00385042	-2.812	0.00087	9	5	0	1
1208	Isoform A of Ras GTPase-activating protein-binding protein 2	IPI00009057	-0.743	0.07647	8	5	4	5
1209	Isoform 1 of Serine hydroxymethyltransferase, cytosolic	IPI00002519	-1.191	0.03143	10	7	5	5
1210	Isoform 1 of Protein canopy homolog 2	IPI00443909	-0.332	0.23812	9	6	8	5
1211	Golgi phosphoprotein 3	IPI00005490	-1.191	0.03143	9	8	6	4
1212	Cell division protein kinase 6	IPI00023529	-1.506	0.01354	4	11	3	4
1213	cDNA FLJ60317, highly similar to Aminoacylase-1	IPI00009268	-0.220	0.31447	2	6	4	3
1214	Transducin beta-like protein 3	IPI00477971	-1.050	0.03677	6	5	3	3
1215	40S ribosomal protein S25	IPI00012750	-1.260	0.02157	11	9	7	5
1216	Isoform 2 of AP-3 complex subunit delta-1	IPI00289608	-0.951	0.05006	7	6	4	4
1217	Aldehyde dehydrogenase X, mitochondrial	IPI00103467	-2.045	0.00368	12	11	5	5
1218	Isoform A of Peptidyl-prolyl cis-trans isomerase E	IPI00009316	-1.305	0.02021	5	6	3	2
1219	Calcium-binding mitochondrial carrier protein Aralar1	IPI00386271	-0.545	0.13328	8	5	5	5
1220	Histone H1.0	IPI00550239	-1.002	0.04087	8	9	6	5
1221	Gamma-aminobutyric acid receptor-associated protein-like 2	IPI00026358	-0.332	0.23812	10	5	8	5
1222	Cell division protein kinase 5	IPI00023530	-0.208	0.32059	4	5	6	2
1223	Isoform 1 of Acyl-coenzyme A thioesterase 9, mitochondrial	IPI00220710	-1.342	0.01958	6	8	5	2
1224	Bifunctional methylenetetrahydrofolate dehydrogenase/cyclohydrolase, mitochondrial	IPI00011307	-2.058	0.00352	9	8	4	2
1225	Isoform 1 of DDRGK domain-containing protein 1	IPI00028387	-0.666	0.11121	6	10	6	6
1226	4-hydroxyphenylpyruvate dioxygenase-like protein	IPI00063762	-1.076	0.03586	7	8	5	4
1227	Tyrosyl-tRNA synthetase, mitochondrial	IPI00165092	-0.181	0.33571	4	8	6	5
1228	V-type proton ATPase subunit D	IPI00001568	-0.428	0.18789	4	5	5	2
1229	Replication factor C subunit 4	IPI00017381	-0.189	0.33144	5	6	4	6
1230	COP9 signalosome complex subunit 5	IPI00009958	1.076	0.03610	4	5	7	8
1231	cDNA FLJ56402, highly similar to Tripeptidyl-peptidase 1	IPI00298237	-1.225	0.02323	11	10	7	6

No.	Description	Accession number	STN[1]	p-Value[1]	Con_A[2]	Con_B[2]	OXA_A[2]	OXA_B[2]
1232	Transmembrane protein 165	IPI00307572	-0.645	0.11390	9	8	8	5
1233	Protein phosphatase 1 regulatory subunit 14B	IPI00398922	-0.820	0.06554	7	10	8	4
1234	Mitochondrial glutamate carrier 1	IPI00003004	-1.235	0.02182	9	7	6	3
1235	Peptidyl-prolyl cis-trans isomerase FKBP5	IPI00218775	-0.175	0.33798	5	8	7	5
1236	Isoform XLas-1 of Guanine nucleotide-binding protein G(s) subunit alpha isoforms XLas	IPI00095891	-0.189	0.33144	4	7	4	6
1237	Glucosamine--fructose-6-phosphate aminotransferase [isomerizing] 2	IPI00216159	-0.387	0.20665	5	6	6	3
1238	Isoform 2 of Ubiquitin thioesterase OTUB1	IPI00409750	-0.743	0.07647	9	4	7	2
1239	Isoform 1 of Pescadillo homolog	IPI00003768	-1.299	0.02029	4	4	2	1
1240	Isoform 2 of Histone deacetylase 2	IPI00289601	-0.304	0.26602	8	10	8	8
1241	Isoform Long of 60 kDa SS-A/Ro ribonucleoprotein	IPI00019450	-0.169	0.34159	5	9	7	6
1242	28S ribosomal protein S31, mitochondrial	IPI00294242	-0.169	0.34159	8	6	6	7
1243	GTP:AMP phosphotransferase mitochondrial	IPI00465256	0.000	0.37049	7	7	6	8
1244	Vesicle transport protein GOT1B	IPI00007061	-0.198	0.32751	5	5	6	3
1245	60S ribosomal protein L22	IPI00219153	-1.804	0.00563	14	10	6	6
1246	Endoplasmic reticulum metallopeptidase 1	IPI00257903	-1.115	0.03482	4	6	4	1
1247	Isoform 2 of Extended synaptotagmin-2	IPI00409635	0.525	0.15696	7	4	7	7
1248	cDNA FLJ56357, highly similar to Homo sapiens apolipoprotein A-I binding protein	IPI00168479	-0.463	0.16959	9	9	8	7
1249	NEDD8	IPI00020008	-0.198	0.32751	6	4	6	3
1250	Malate dehydrogenase	IPI00916111	-0.688	0.08641	8	7	6	5
1251	rho GTPase-activating protein 4 isoform 1	IPI00328842	-0.663	0.11221	3	6	4	2
1252	Isoform A of Protein CutA	IPI00034319	-0.175	0.33798	6	7	7	5
1253	Isoform A of Ras-related C3 botulinum toxin substrate 1	IPI00010271	-0.688	0.08641	7	8	6	5
1254	Putative uncharacterized protein INF2	IPI00872508	-1.910	0.00414	10	12	5	5
1255	Putative uncharacterized protein	IPI00260769	-1.235	0.02182	8	8	5	4
1256	Isoform 1 of RRP12-like protein	IPI00101186	-2.783	0.00091	12	10	5	1
1257	Isoform 1 of Protein unc-45 homolog A	IPI00072534	1.506	0.01321	3	4	8	7
1258	Splicing factor 3B subunit 4	IPI00017339	-0.406	0.20280	4	6	3	5
1259	myosin-IXb isoform 1	IPI00306933	-0.220	0.31447	5	3	5	2
1260	Zinc finger CCHC domain-containing protein 3	IPI00011550	-2.081	0.00323	2	8	1	1
1261	Isoform 1 of Protein AHNAK2	IPI00856045	-0.842	0.06318	4	2	2	1
1262	Sphingosine-1-phosphate lyase 1	IPI00099463	-0.525	0.15692	7	7	6	5
1263	Putative ATP-dependent Clp protease proteolytic subunit, mitochondrial	IPI00003870	-0.343	0.23683	9	5	6	6
1264	DNA-directed RNA polymerase II subunit RPB3	IPI00018288	-0.428	0.18789	4	5	4	3
1265	COP9 signalosome complex subunit 7a	IPI00301419	-1.172	0.03147	6	7	5	2
1266	UPF0468 protein C16orf80	IPI00001655	-0.332	0.23812	8	7	8	5
1267	Isoform 1 of Remodeling and spacing factor 1	IPI00290652	-0.997	0.04120	5	7	3	4
1268	magnesium transporter protein 1	IPI00301202	0.912	0.05403	4	5	8	6
1269	Isoform 2 of Ubiquitin-associated domain-containing protein 2	IPI00007034	1.664	0.00994	3	2	8	5
1270	N-acylsphingosine amidohydrolase (Acid ceramidase) 1, isoform CRA_c	IPI00013698	-1.585	0.01172	5	6	3	0
1271	Succinyl-CoA ligase [GDP-forming] subunit beta, mitochondrial	IPI00096066	-1.152	0.03167	9	9	5	6
1272	Isoform 1 of Paraspeckle component 1	IPI00103525	0.666	0.11150	6	6	7	9
1273	Leucine-rich repeat-containing protein 47	IPI00170935	0.663	0.11477	3	3	5	4
1274	Probable methylthioribulose-1-phosphate dehydratase	IPI00549730	0.356	0.23584	6	5	9	4
1275	Isoform 1 of Protein virilizer homolog	IPI00036742	-0.595	0.12310	8	3	5	3
1276	DKFZP586J0619 protein	IPI00740961	-0.253	0.29716	3	3	3	2
1277	Isoform 1 of Large proline-rich protein BAT2	IPI00010700	-1.208	0.03035	3	3	1	1
1278	B-cell receptor-associated protein 31	IPI00218200	0.000	0.37049	4	6	4	6
1279	40S ribosomal protein S11	IPI00025091	-0.588	0.12558	4	1	2	1
1280	Signal recognition particle 14 kDa protein	IPI00293434	-1.002	0.04087	8	9	9	2
1281	Ribosome biogenesis protein BRX1 homolog	IPI00181728	-1.834	0.00526	7	7	2	3
1282	Eukaryotic translation initiation factor 3 subunit K	IPI00033143	0.322	0.24627	8	6	10	6
1283	Protein SCO1 homolog, mitochondrial	IPI00027233	-1.305	0.02021	7	4	3	2
1284	DnaJ homolog subfamily B member 1	IPI00015947	-0.332	0.23812	8	7	8	5
1285	Aldo-keto reductase family 1 member C3	IPI00291483	-0.545	0.13328	6	7	4	6
1286	CDGSH iron sulfur domain-containing protein 1	IPI00020510	0.000	0.37049	7	3	7	3
1287	Pre-mRNA-splicing factor CWC22 homolog	IPI00177381	0.000	0.37049	1	2	2	0
1288	Isoform 1 of Lysocardiolipin acyltransferase 1	IPI00419643	0.198	0.32457	4	5	6	4
1289	PRA1 family protein 3	IPI00007426	0.387	0.20826	5	4	7	4
1290	Actin-related protein 2/3 complex subunit 1B	IPI00005160	0.000	0.37049	2	4	2	4
1291	Isoform 4 of Nucleoporin NDC1	IPI00003455	0.997	0.04049	5	2	9	3
1292	EF-hand domain-containing protein D2	IPI00060181	0.198	0.32457	5	4	5	5
1293	Coronin-1B	IPI00007058	0.545	0.13436	5	5	6	7
1294	Protein transport protein Sec23A	IPI00017375	0.912	0.05403	4	5	8	6
1295	Twinfilin-2	IPI00550917	0.568	0.12984	5	4	8	4
1296	Flotillin-1	IPI00027438	0.595	0.12525	4	4	7	4
1297	Isoform 2 of Ubiquitin carboxyl-terminal hydrolase isozyme L5	IPI00219512	-1.342	0.01958	8	6	3	4
1298	Thymidine kinase, cytosolic	IPI00299214	1.768	0.00522	1	3	6	6
1299	Signal peptidase complex catalytic subunit SEC11A	IPI00104128	-0.428	0.18789	5	4	5	2
1300	Importin-11	IPI00301107	-0.175	0.33798	7	6	6	6
1301	Isoform 1 of Sorting nexin-12	IPI00438170	-0.568	0.12806	6	6	6	3
1302	Serine/threonine-protein kinase OSR1	IPI00010080	-0.708	0.08447	6	2	4	1
1303	Isoform 2 of Beta-catenin-like protein 1	IPI00472981	-0.198	0.32751	5	5	5	4
1304	Isoform 1 of Pentatricopeptide repeat-containing protein 3, mitochondrial	IPI00783302	-0.356	0.22868	7	6	6	5
1305	Sec1 family domain-containing protein 1	IPI00165261	0.997	0.04049	3	4	6	6
1306	cDNA FLJ14239 fis, clone NT2RP5003512, highly similar to Exportin-5	IPI00549861	-0.387	0.20665	4	7	4	5
1307	Isoform D of Constitutive coactivator of PPAR-gamma-like protein 1	IPI00039626	0.986	0.04066	1	3	5	3
1308	ADP-ribosylation factor 6	IPI00215920	0.169	0.34208	7	6	9	5
1309	Isoform 1 of Methylthioribose-1-phosphate isomerase	IPI00005948	-0.912	0.05316	8	6	5	4
1310	Ferritin heavy chain	IPI00554521	0.743	0.07850	5	4	9	4
1311	Isoform 1 of Transmembrane and coiled-coil domain-containing protein 1	IPI00026111	-0.530	0.15514	5	0	3	1
1312	RNA-binding motif protein, X-linked-like-2	IPI00004450	-0.428	0.18789	3	6	3	4
1313	Phospholipase A-2-activating protein	IPI00218465	-1.172	0.03147	4	9	2	5
1314	Isoform F of Protein SON	IPI00000192	0.208	0.31861	2	6	7	2
1315	Small acidic protein	IPI00003419	-0.304	0.26602	10	8	8	8
1316	Putative uncharacterized protein ALB	IPI00022434	-0.776	0.07324	6	6	5	3
1317	60S ribosomal protein L24	IPI00306332	-1.037	0.03938	8	8	7	3
1318	Cytochrome b-c1 complex subunit Rieske, mitochondrial	IPI00026964	-0.507	0.15999	9	6	5	7
1319	Isoform 2 of Mitochondrial import inner membrane translocase subunit TIM50	IPI00418497	-0.568	0.12806	7	5	6	3
1320	Isoform 2 of Ubiquitin-conjugating enzyme E2 K	IPI00019894	-0.663	0.11221	6	3	4	2
1321	Isoform 1 of Ubiquitin-conjugating enzyme E2 K	IPI00021370	-0.159	0.34738	8	8	7	8
1322	Isoform 1 of ATPase family AAA domain-containing protein 1	IPI00171445	0.387	0.20826	4	5	5	6
1323	28S ribosomal protein S25, mitochondrial	IPI00013167	0.000	0.37049	4	5	5	4
1324	Isoform 1 of Serine/threonine-protein phosphatase 2A 65 kDa regulatory subunit A beta	IPI00294178	-0.568	0.12806	5	7	6	3
1325	Isoform 1 of Enolase-phosphatase E1	IPI00038378	0.406	0.19435	4	4	4	6
1326	Mitochondrial ribonuclease P protein 1	IPI00099996	-0.776	0.07324	6	6	6	2

No.	Description	Accession number	STN[1]	p-Value[1]	Con_A[2]	Con_B[2]	OXA_A[2]	OXA_B[2]
1327	Isoform 3 of Protein transport protein Sec31A	IPI00305152	1.115	0.03515	3	2	6	4
1328	CTP synthase 2	IPI00645702	-0.455	0.18350	6	2	4	2
1329	DNA-directed RNA polymerases I, II, and III subunit RPABC1	IPI00291093	-1.191	0.03143	7	10	5	5
1330	Calcium-binding protein 39-like	IPI00026359	-0.198	0.32751	6	4	3	6
1331	Cytoplasmic aconitate hydratase	IPI00008485	1.444	0.01433	4	4	7	9
1332	GTP-binding protein SAR1a	IPI00015954	-0.163	0.34436	6	9	6	8
1333	Parafibromin	IPI00300659	0.000	0.37049	3	5	5	3
1334	Peptidyl-prolyl cis-trans isomerase H	IPI00007346	0.000	0.37049	5	5	6	4
1335	Isoform 2 of Tyrosine-protein phosphatase non-receptor type 11	IPI00298347	1.394	0.01540	2	2	5	5
1336	Replication protein A 14 kDa subunit	IPI00017373	-0.743	0.07647	8	5	5	4
1337	RNA binding motif protein, X-linked-like 1	IPI00061178	-0.743	0.07647	7	6	5	4
1338	L antigen family member 3	IPI00032314	-0.387	0.20665	8	3	7	2
1339	40S ribosomal protein S20	IPI00012493	-1.121	0.03445	5	9	4	4
1340	Isoform 1 of RNA-binding protein with serine-rich domain 1	IPI00033561	-0.545	0.13328	8	5	5	5
1341	Eukaryotic translation initiation factor 2A	IPI00012462	0.000	0.37049	5	3	3	5
1342	NADH dehydrogenase [ubiquinone] 1 beta subcomplex subunit 6	IPI00219385	-0.455	0.18350	4	4	5	1
1343	Isoform 3 of Cytosolic 5'-nucleotidase 3	IPI00100192	-2.616	0.00095	10	9	3	2
1344	Isoform 2 of 6-phosphofructokinase, muscle type	IPI00219585	-1.305	0.02021	6	5	3	2
1345	Programmed cell death protein 5	IPI00023640	-0.491	0.16438	10	6	8	5
1346	SNW domain-containing protein 1	IPI00013830	0.000	0.37049	7	5	7	5
1347	Rho-related GTP-binding protein RhoC	IPI00027434	-0.220	0.31447	5	3	6	1
1348	Isoform 1 of 3'(2'),5'-bisphosphate nucleotidase 1	IPI00410214	-1.299	0.02029	9	10	6	5
1349	60S ribosomal protein L32	IPI00395998	-1.972	0.00393	10	8	3	4
1350	Putative high mobility group protein B3-like-1	IPI00006437	-0.343	0.23683	6	8	6	6
1351	Immunoglobulin-binding protein 1	IPI00019148	-0.277	0.27840	1	4	2	2
1352	Isoform 1 of Medium-chain specific acyl-CoA dehydrogenase, mitochondrial	IPI00005040	-1.076	0.03586	8	7	6	3
1353	Isoform 1 of Mps one binder kinase activator-like 1B	IPI00301518	0.488	0.16541	3	2	4	3
1354	ATP-dependent Clp protease ATP-binding subunit clpX-like, mitochondrial	IPI00008728	1.285	0.02045	4	4	7	8
1355	39S ribosomal protein L1, mitochondrial	IPI00549381	-0.198	0.32751	4	6	6	3
1356	Isoform 1 of Polyadenylate-binding protein 4	IPI00012726	0.220	0.31215	4	3	5	3
1357	Isoform 1 of Methyl-CpG-binding domain protein 3	IPI00439194	0.370	0.20967	6	4	7	5
1358	Growth arrest and DNA damage-inducible proteins-interacting protein 1	IPI00552587	-0.181	0.33571	6	6	4	7
1359	Putative nascent polypeptide-associated complex subunit alpha-like proteir	IPI00012479	-0.507	0.15999	8	7	6	6
1360	Isoform 1 of Dr1-associated corepressor	IPI00003084	-0.951	0.05006	9	4	5	3
1361	Putative uncharacterized protein CNOT1	IPI00032299	-1.585	0.01172	8	3	2	2
1362	Isoform 1 of Low molecular weight phosphotyrosine protein phosphatase	IPI00219861	-0.163	0.34436	7	8	8	6
1363	Isoform 1 of Putative helicase MOV-10	IPI00444452	0.000	0.37049	6	6	7	5
1364	Aflatoxin B1 aldehyde reductase member 2	IPI00305978	-0.525	0.15692	7	7	6	5
1365	Isoform 2C of Cytoplasmic dynein 1 intermediate chain 2	IPI00216348	-0.235	0.30503	3	4	2	4
1366	Myosin-Id	IPI00329719	0.765	0.07536	2	2	4	3
1367	pyruvate dehydrogenase E1 alpha 1 isoform 2 precursor	IPI00306301	-0.370	0.21613	7	5	6	4
1368	Golgin subfamily B member 1	IPI00004671	1.509	0.01308	2	1	5	4
1369	mesencephalic astrocyte-derived neurotrophic factor	IPI00328748	-0.861	0.06115	6	4	4	2
1370	L-aminoadipate-semialdehyde dehydrogenase-phosphopantetheinyl transferase	IPI00250297	-1.665	0.01068	8	8	4	3
1371	Deoxyribonucleoside 5'-monophosphate N-glycosidase	IPI00007926	-0.878	0.05474	6	9	7	3
1372	Isoform 2 of Isochorismatase domain-containing protein 2, mitochondrial	IPI00003031	0.000	0.37049	4	4	5	3
1373	SH3 domain-binding glutamic acid-rich-like protein	IPI00025318	-0.666	0.11121	10	6	9	3
1374	Isoform 1 of Dynamin-like 120 kDa protein, mitochondrial	IPI00006721	0.428	0.19017	5	2	4	5
1375	Mitochondrial ribosomal protein L21 isoform d	IPI00375677	-0.387	0.20665	5	6	4	5
1376	DnaJ homolog subfamily B member 11	IPI00008454	0.595	0.12525	3	5	7	4
1377	39S ribosomal protein L19, mitochondrial	IPI00027096	0.277	0.27228	3	1	4	1
1378	Pterin-4-alpha-carbinolamine dehydratase	IPI00218568	0.000	0.37049	7	3	6	4
1379	28S ribosomal protein S26, mitochondrial	IPI00006606	0.000	0.37049	7	7	8	6
1380	Isoform 3 of Chitinase domain-containing protein 1	IPI00045536	-0.951	0.05006	6	7	3	5
1381	Putative uncharacterized protein ENSP00000350479	IPI00069693	-1.191	0.03143	10	7	5	5
1382	33 kDa protein	IPI00413108	-1.999	0.00389	11	4	4	1
1383	Isoform 1 of Apoptotic chromatin condensation inducer in the nucleus	IPI00007334	-0.595	0.12310	5	6	4	4
1384	60S ribosomal protein L7-like 1	IPI00456940	-0.189	0.33144	5	6	4	6
1385	Guanine nucleotide-binding protein G(k) subunit alpha	IPI00220578	-0.175	0.33798	7	6	4	8
1386	Cell division cycle 5-like protein	IPI00465294	-1.285	0.02058	8	7	3	5
1387	Mitochondrial import inner membrane translocase subunit Tim23	IPI00007309	-0.595	0.12310	5	6	4	4
1388	Ribosome biogenesis protein BMS1 homolog	IPI00006099	-2.980	0.00087	5	10	1	0
1389	Metastasis-associated protein MTA2	IPI00171798	-0.776	0.07324	7	5	5	3
1390	Proteasome subunit beta type-6	IPI00000811	-0.743	0.07647	8	5	7	2
1391	COP9 signalosome complex subunit 6	IPI00163230	-0.916	0.05184	4	5	2	3
1392	Cold-inducible RNA-binding protein	IPI00180954	0.356	0.23584	5	6	7	6
1393	Calcium-binding protein 39	IPI00032561	-1.408	0.01536	5	8	3	3
1394	Isoform 2 of Succinyl-CoA ligase [ADP-forming] subunit beta, mitochondrial	IPI00217232	0.000	0.37049	3	6	4	5
1395	Protein of unknown function DUF410 family protein	IPI00419575	-1.115	0.03482	6	4	3	2
1396	Programmed cell death protein 10	IPI00298558	-0.568	0.12806	6	6	4	5
1397	Putative uncharacterized protein DKFZp781K1356	IPI00412545	-1.235	0.02182	9	7	7	2
1398	Integrator complex subunit 2	IPI00477759	-0.235	0.30503	4	3	4	2
1399	60S ribosomal protein L17	IPI00413324	-0.815	0.06587	5	6	3	4
1400	Isoform SRP40-1 of Splicing factor, arginine/serine-rich 5	IPI00012341	0.181	0.33562	5	6	6	6
1401	Peptidyl-prolyl cis-trans isomerase D	IPI00003927	-1.050	0.03677	5	6	4	2
1402	Ribonuclease P protein subunit p30	IPI00019196	-0.387	0.20665	7	4	5	4
1403	Uncharacterized protein C18orf19	IPI00290799	-0.387	0.20665	5	6	5	4
1404	Isoform CNPI of 2',3'-cyclic-nucleotide 3'-phosphodiesterase	IPI00220993	-0.189	0.33144	5	6	5	5
1405	SF3A2 protein (Fragment)	IPI00017341	0.220	0.31215	3	4	2	6
1406	Calcium-regulated heat stable protein 1	IPI00304409	-0.488	0.16467	3	4	2	3
1407	Isoform 1 of Armadillo repeat-containing protein 10	IPI00166394	-0.525	0.15692	5	9	8	3
1408	Putative rRNA methyltransferase 3	IPI00217686	-3.042	0.00087	8	14	2	3
1409	Isoform 2 of Calumenin	IPI00045396	-0.175	0.33798	5	8	6	6
1410	U4/U6.U5 tri-snRNP-associated protein 1	IPI00021417	-1.999	0.00389	7	8	4	1
1411	Exportin-T	IPI00306290	-1.342	0.01958	8	6	4	3
1412	39S ribosomal protein L49, mitochondrial	IPI00013195	0.000	0.37049	6	4	4	6
1413	Lysosome membrane protein 2	IPI00217766	-0.674	0.10802	2	2	1	1
1414	28S ribosomal protein S23, mitochondrial	IPI00032881	-0.428	0.18789	5	4	5	2
1415	Isoform 1 of AP-2 complex subunit mu	IPI00022256	0.952	0.04819	0	0	2	3
1416	Coactosin-like protein	IPI00017704	-0.626	0.12011	6	4	5	2
1417	Succinate dehydrogenase [ubiquinone] iron-sulfur subunit, mitochondrial	IPI00294911	-0.688	0.08641	10	5	6	5
1418	Negative elongation factor B	IPI00103483	0.916	0.05271	1	4	4	5
1419	Endoplasmic reticulum resident protein 44	IPI00401264	-0.235	0.30503	4	3	3	3
1420	Isoform 3 of PCI domain-containing protein 2	IPI00072541	-0.220	0.31447	4	4	2	5
1421	Protein MEMO1	IPI00032426	-0.488	0.16467	4	3	3	2

No.	Description	Accession number	STN[1]	p-Value[1]	Con_A[2]	Con_B[2]	OXA_A[2]	OXA_B[2]
1422	Isoform 1 of Ribonuclease H2 subunit C	IPI00382985	-0.861	0.06115	6	4	5	1
1423	Elongator complex protein 1	IPI00293735	1.394	0.01540	2	2	6	4
1424	Isoform 2 of 39S ribosomal protein L39, mitochondrial	IPI00084571	-0.708	0.08447	4	4	4	1
1425	DnaJ homolog subfamily C member 8	IPI00003438	-0.428	0.18789	4	5	6	1
1426	cDNA FLJ55829, highly similar to Homo sapiens leucine zipper and CTNNBIP1 domain	IPI00152900	-1.509	0.01342	6	3	1	2
1427	Transmembrane protein 126A	IPI00031064	-1.578	0.01221	8	6	5	1
1428	28S ribosomal protein S10, mitochondrial	IPI00061245	-0.861	0.06115	6	4	4	2
1429	NADH-ubiquinone oxidoreductase chain 1	IPI00007961	-0.986	0.04534	5	3	2	2
1430	Myeloid-associated differentiation marker	IPI00102685	1.050	0.03689	2	4	6	5
1431	Isoform 1 of Solute carrier family 12 member 2	IPI00022649	0.208	0.31861	4	4	4	5
1432	transcriptional regulator ATRX isoform 2	IPI00220109	-1.233	0.02323	11	1	4	2
1433	Prostaglandin E synthase 2	IPI00303568	-0.776	0.07324	8	4	4	4
1434	Isoform 1 of Replication protein A 32 kDa subunit	IPI00013939	-0.428	0.18789	5	4	6	0
1435	Cytochrome b-c1 complex subunit 1, mitochondrial	IPI00013847	0.189	0.32929	4	6	5	6
1436	Isoform 1 of N-acylneuraminate cytidylyltransferase	IPI00303158	-0.986	0.04534	4	4	2	2
1437	Isoform 1 of Nuclear pore complex protein Nup98-Nup96	IPI00006038	0.708	0.08397	2	3	3	5
1438	Isoform Delta-1 of Serine/threonine-protein phosphatase 2A 56 kDa regulatory subunit	IPI00000030	0.708	0.08397	2	3	4	4
1439	Small glutamine-rich tetratricopeptide repeat-containing protein alpha	IPI00013949	-1.585	0.01172	6	5	2	2
1440	26 kDa protein	IPI00219685	0.000	0.37049	3	1	3	0
1441	14-3-3 protein eta	IPI00216319	-1.342	0.01958	7	11	5	5
1442	U2 small nuclear ribonucleoprotein B''	IPI00029267	-0.815	0.06587	5	6	3	4
1443	Heat shock protein beta-11	IPI00098827	1.299	0.02029	1	2	6	2
1444	Coiled-coil-helix-coiled-coil-helix domain-containing protein 3, mitochondrial	IPI00015833	-0.970	0.04753	10	8	6	6
1445	Peptidyl-prolyl cis-trans isomerase NIMA-interacting 1	IPI00013723	0.000	0.37049	2	3	3	2
1446	Isoform 2 of COP9 signalosome complex subunit 2	IPI00018813	0.000	0.37049	5	2	5	2
1447	Serine/threonine-protein phosphatase 1 regulatory subunit 10	IPI00298731	0.277	0.27228	2	2	3	2
1448	Aldo-keto reductase family 1 member C1	IPI00029733	-0.356	0.22868	7	6	6	5
1449	Periplakin	IPI00298057	1.078	0.03610	2	1	5	2
1450	Aspartyl-tRNA synthetase, mitochondrial	IPI00100460	0.488	0.16541	2	3	5	2
1451	Isoform 1 of Bifunctional coenzyme A synthase	IPI00184821	0.406	0.19435	5	3	7	3
1452	Phosphoribosyl pyrophosphate synthase-associated protein 2	IPI00003168	0.406	0.19435	4	4	5	5
1453	Isoform 1 of Actin-like protein 6A	IPI00003627	0.387	0.20826	5	4	4	7
1454	Splicing factor, arginine/serine-rich 4	IPI00000015	-0.568	0.12806	6	6	4	5
1455	Cyclin-G-associated kinase	IPI00298949	-0.277	0.27840	3	2	3	0
1456	Epithelial cell adhesion molecule	IPI00296215	-1.121	0.03445	7	7	4	4
1457	39S ribosomal protein L23, mitochondrial	IPI00293476	-0.220	0.31447	5	3	4	3
1458	Isoform 1 of 6-phosphofructokinase, liver type	IPI00332371	-1.394	0.01772	5	5	1	3
1459	cAMP-dependent protein kinase type I-alpha regulatory subunit	IPI00021831	0.951	0.05080	3	5	5	8
1460	Isoform Rpn10A of 26S proteasome non-ATPase regulatory subunit 4	IPI00022694	-0.169	0.34159	6	8	7	6
1461	Rho-related GTP-binding protein RhoG	IPI00017342	-0.951	0.05006	8	5	4	4
1462	cDNA FLJ54536, highly similar to Mitochondrial 28S ribosomal protein S27	IPI00022002	-0.568	0.12806	6	6	4	5
1463	Isoform 1 of Protein 4.1	IPI00003921	0.000	0.37049	5	4	4	5
1464	Tropomodulin-3	IPI00005087	0.000	0.37049	4	5	5	4
1465	Scaffold attachment factor B2	IPI00005648	-0.568	0.12806	6	6	3	6
1466	Isoform 2 of 3-hydroxyisobutyryl-CoA hydrolase, mitochondrial	IPI00377161	-0.912	0.05316	9	5	6	3
1467	Protein BUD31 homolog	IPI00013180	-1.585	0.01172	6	5	3	0
1468	Isoform 2 of Golgi apparatus protein 1	IPI00414717	0.000	0.37049	5	4	6	3
1469	Isoform 1 of Mitochondrial antiviral-signaling protein	IPI00020719	-0.507	0.15999	7	8	6	6
1470	Isoform 1 of Elongation factor G, mitochondrial	IPI00154473	0.198	0.32457	5	4	5	5
1471	ATP-dependent RNA helicase SUPV3L1, mitochondrial	IPI00412404	-0.568	0.12806	6	6	3	6
1472	Isoform 2 of U4/U6 small nuclear ribonucleoprotein Prp31	IPI00167198	-0.912	0.05316	6	8	4	5
1473	Zinc finger protein ZPR1	IPI00025244	-0.507	0.15999	9	6	7	5
1474	Ribose-5-phosphate isomerase	IPI00026513	-1.742	0.00716	8	7	4	2
1475	Thioredoxin domain-containing protein 5	IPI00171438	0.000	0.37049	1	5	3	3
1476	Isoform 1 of tRNA-nucleotidyltransferase 1, mitochondrial	IPI00289807	-1.506	0.01354	7	8	4	3
1477	F-actin-capping protein subunit alpha-2	IPI00026182	-1.195	0.03093	6	3	1	3
1478	Isoform 2 of Septin-11	IPI00019376	0.198	0.32457	5	4	4	6
1479	28S ribosomal protein S28, mitochondrial	IPI00022276	-0.189	0.33144	5	6	6	4
1480	nardilysin isoform a	IPI00243221	2.752	0.00079	2	2	9	9
1481	Coproporphyrinogen-III oxidase, mitochondrial	IPI00093057	0.488	0.16541	4	0	2	5
1482	Isoform 1 of Nck-associated protein 1	IPI00031982	0.916	0.05271	2	3	4	5
1483	Solute carrier family 4 sodium bicarbonate cotransporter member 7	IPI00021058	-1.880	0.00489	5	4	0	0
1484	Ephrin type-A receptor 2	IPI00021267	0.198	0.32457	5	4	6	4
1485	Tubulin gamma-1 chain	IPI00295081	-0.488	0.16467	2	5	2	3
1486	Polymerase delta-interacting protein 2	IPI00165506	0.708	0.08397	3	2	4	4
1487	Paired amphipathic helix protein Sin3a	IPI00170596	-1.709	0.00778	4	6	2	0
1488	Isoform 2 of Suppressor of G2 allele of SKP1 homolog	IPI00791573	-1.664	0.01068	6	7	3	2
1489	Myosin-11	IPI00020501	-0.220	0.31447	5	3	5	2
1490	Isoform 2 of Serine/threonine-protein kinase PAK 3	IPI00027382	0.220	0.31215	4	3	5	3
1491	Isoform Beta-1C of Integrin beta-1	IPI00217561	0.220	0.31215	3	4	4	4
1492	HSR1 protein	IPI00384745	-0.406	0.20280	6	4	4	4
1493	cDNA FLJ78497	IPI00289535	-0.916	0.05184	5	4	2	3
1494	Interferon-induced, double-stranded RNA-activated protein kinase	IPI00019463	-0.189	0.33144	6	5	6	4
1495	Sorting nexin-2	IPI00299095	-0.455	0.18350	5	3	2	4
1496	Copine-3	IPI00024403	0.208	0.31861	2	6	5	4
1497	Isoform 2 of Ubiquinol-cytochrome c reductase complex chaperone CBP3 homolog	IPI00219889	0.181	0.33562	7	4	7	5
1498	Tetratricopeptide repeat protein 37	IPI00005634	0.000	0.37049	3	5	4	4
1499	UPF0368 protein Cxorf26	IPI00107104	0.181	0.33562	5	6	8	4
1500	SRA stem-loop-interacting RNA-binding protein, mitochondrial	IPI00009922	-0.181	0.33571	8	4	6	5
1501	DEAD (Asp-Glu-Ala-Asp) box polypeptide 39, isoform CRA_c	IPI00166874	-1.285	0.02058	6	9	4	4
1502	SWI/SNF complex subunit SMARCC1	IPI00234252	-0.568	0.12806	7	5	4	5
1503	Prostaglandin E synthase 3	IPI00015029	-0.568	0.12806	6	6	6	3
1504	Transmembrane emp24 domain-containing protein 9	IPI00023542	-0.406	0.20280	5	5	5	3
1505	Isoform 1 of Uncharacterized protein KIAA0528	IPI00465142	-0.235	0.30503	3	4	4	2
1506	GTPase NRas	IPI00000005	0.208	0.31861	5	3	5	4
1507	Isoform 1 of Protein-tyrosine phosphatase mitochondrial 1	IPI00174190	-0.663	0.11221	5	4	5	1
1508	Similar to nonhistone chromosomal protein HMG-1	IPI00418184	-0.743	0.07647	8	5	5	4
1509	Pseudouridylate synthase 7 homolog	IPI00044761	0.220	0.31215	2	5	3	5
1510	Ras-related protein Rab-6B	IPI00016891	-0.370	0.21613	6	6	4	6
1511	WD repeat-containing protein 82	IPI00152695	0.000	0.37049	5	5	4	6
1512	Isoform 1 of Phosphoenolpyruvate carboxykinase [GTP], mitochondrial	IPI00797038	-1.408	0.01536	7	6	3	3
1513	Isoform CSBP2 of Mitogen-activated protein kinase 14	IPI00002857	0.000	0.37049	2	4	3	3
1514	Probable O-sialoglycoprotein endopeptidase	IPI00015809	-0.208	0.32059	5	4	4	4
1515	Ribonucleoside-diphosphate reductase large subunit	IPI00013871	1.945	0.00368	2	2	7	6
1516	Isoform 1 of Thymocyte nuclear protein 1	IPI00383163	-1.195	0.03093	4	5	3	1

No.	Description	Accession number	STN[1]	p-Value[1]	Con_A[2]	Con_B[2]	OXA_A[2]	OXA_B[2]
1517	Isoform Long of FAS-associated factor 1	IPI00070643	0.000	0.37049	3	3	4	2
1518	Isoform 2 of Transcription elongation factor A protein 1	IPI00218106	-1.664	0.01068	6	7	4	1
1519	Tubulin-specific chaperone E	IPI00018402	0.663	0.11477	2	4	4	5
1520	V-type proton ATPase subunit F	IPI00004488	-1.342	0.01958	9	5	3	4
1521	Isoform 2 of Integrator complex subunit 3	IPI00418336	-0.455	0.18350	4	4	3	3
1522	Isoform 3 of Tyrosine-protein kinase-like 7	IPI00168813	0.277	0.27228	2	2	2	3
1523	Heme-binding protein 1	IPI00148063	0.356	0.23584	5	6	6	7
1524	Heat shock 70 kDa protein 12A	IPI00011932	0.861	0.05730	3	3	4	6
1525	Exosome complex exonuclease MTR3	IPI00073602	-0.198	0.32751	5	5	5	4
1526	Isoform 1 of Rab3 GTPase-activating protein non-catalytic subunit	IPI00554590	-0.189	0.33144	4	7	6	4
1527	UPF0553 protein C9orf64	IPI00170972	-0.198	0.32751	5	5	4	5
1528	proteasome subunit beta type-5 isoform 3	IPI00383971	-0.545	0.13328	7	6	6	4
1529	Isoform 1 of Vacuolar protein sorting-associated protein 29	IPI00170796	0.198	0.32457	4	5	5	5
1530	Isoform 2 of Peptidyl-prolyl cis-trans isomerase NIMA-interacting 4	IPI00006658	-0.986	0.04534	6	2	2	2
1531	Flotillin-2	IPI00789008	0.986	0.04066	2	2	5	3
1532	COP9 signalosome complex subunit 4	IPI00171844	0.387	0.20826	3	6	6	5
1533	39S ribosomal protein L50, mitochondrial	IPI00329036	-0.235	0.30503	5	2	5	0
1534	39S ribosomal protein L44, mitochondrial	IPI00009680	-0.861	0.06115	5	5	2	4
1535	Transcription elongation factor B polypeptide 2	IPI00026670	-0.568	0.12806	6	6	5	4
1536	Choline-phosphate cytidylyltransferase A	IPI00329338	-0.878	0.05474	7	8	4	6
1537	Isoform B of Serine/threonine-protein kinase 24	IPI00002212	-0.595	0.12310	5	6	4	4
1538	THO complex subunit 5 homolog	IPI00299417	0.311	0.25253	2	1	2	2
1539	cDNA FLJ56825, highly similar to WD repeat protein 57	IPI00006723	-0.488	0.16467	5	2	3	2
1540	C-terminal-binding protein 1	IPI00012835	-0.776	0.07324	5	7	3	5
1541	Probable rRNA-processing protein EBP2	IPI00745955	-1.834	0.00526	7	7	4	1
1542	Mitochondrial import receptor subunit TOM70	IPI00015602	-1.578	0.01221	5	9	0	5
1543	Lamina-associated polypeptide 2, isoform alpha	IPI00216230	0.708	0.08397	2	3	3	5
1544	Isoform 1 of DNA primase large subunit	IPI00027705	1.121	0.03341	3	5	7	7
1545	Poly(A)-specific ribonuclease PARN	IPI00294744	-0.362	0.22785	1	2	0	0
1546	Isoform 2 of Nucleosome-remodeling factor subunit BPTF	IPI00254408	1.445	0.01433	1	1	5	2
1547	Scaffold attachment factor B1	IPI00300631	0.000	0.37049	5	3	4	4
1548	Isoform 1 of Transcription elongation factor SPT6	IPI00784161	1.050	0.03689	3	3	9	2
1549	cDNA FLJ56184, highly similar to Proto-oncogene tyrosine-protein kinase LCK	IPI00394952	-0.198	0.32751	4	6	4	5
1550	Gem-associated protein 5	IPI00291783	-0.861	0.06115	6	4	2	4
1551	Condensin-2 complex subunit D3	IPI00747787	1.115	0.03515	2	3	7	3
1552	Isoform 1 of BRCA2 and CDKN1A-interacting protein	IPI00002203	-0.370	0.21613	7	5	5	5
1553	Monocarboxylate transporter 4	IPI00006666	-1.305	0.02021	6	5	4	1
1554	60S ribosomal protein L27a	IPI00456758	-1.945	0.00406	8	5	2	2
1555	Methyltransferase like 7B	IPI00090807	1.115	0.03515	1	4	5	5
1556	Ras-related C3 botulinum toxin substrate 2	IPI00010270	0.455	0.17237	3	3	4	4
1557	Isoform 1 of C-terminal-binding protein 2	IPI00010120	-0.406	0.20280	5	5	3	5
1558	Nucleolar protein 9	IPI00002902	-1.299	0.02029	3	5	1	2
1559	Adenylosuccinate synthetase isozyme 2	IPI00026833	0.000	0.37049	6	3	5	4
1560	Ubiquitin-fold modifier-conjugating enzyme 1	IPI00294495	-0.455	0.18350	5	3	5	1
1561	Splicing factor U2AF 35 kDa subunit	IPI00005613	-0.220	0.31447	5	3	4	3
1562	cDNA FLJ59712, highly similar to Golgi reassembly-stacking protein 2	IPI00743931	-0.595	0.12310	6	5	4	4
1563	Isoform 1 of Protein phosphatase methylesterase 1	IPI00007694	-0.208	0.32059	4	5	4	4
1564	DNA polymerase alpha catalytic subunit	IPI00220317	1.445	0.01433	1	1	3	4
1565	Helicase SKI2W	IPI00414819	-0.428	0.18789	5	4	4	3
1566	synembryn-A	IPI00100106	-0.488	0.16467	4	3	3	2
1567	24 kDa protein	IPI00397611	-1.834	0.00526	8	6	3	2
1568	Isoform 1 of STE20-like serine/threonine-protein kinase	IPI00022827	-0.189	0.33144	6	5	5	5
1569	Dihydroorotate dehydrogenase, mitochondrial	IPI00024462	-0.387	0.20665	6	5	3	6
1570	Isoform 1 of Porphobilinogen deaminase	IPI00028160	-0.208	0.32059	3	6	4	4
1571	Phosphoserine phosphatase	IPI00019178	-1.664	0.01068	7	6	3	2
1572	Phosphatidylinositol-4-phosphate 3-kinase C2 domain-containing subunit alpha	IPI00002580	-0.277	0.27840	4	1	2	2
1573	FAST kinase domain-containing protein 5	IPI00414973	-0.311	0.26205	3	1	1	2
1574	Lysosomal Pro-X carboxypeptidase	IPI00001593	0.220	0.31215	4	3	3	5
1575	Isoform 1 of Protein fto	IPI00028277	-0.776	0.07324	4	8	4	4
1576	Isoform UBF1 of Nucleolar transcription factor 1	IPI00014533	-0.588	0.12558	3	2	2	1
1577	Isoform 1 of KDEL motif-containing protein 2	IPI00143921	-0.277	0.27840	2	3	3	0
1578	Isoform 1 of DNA-directed RNA polymerases I and III subunit RPAC1	IPI00005179	-0.743	0.07647	7	6	5	4
1579	U6 snRNA-associated Sm-like protein LSm7	IPI00007163	0.000	0.37049	4	0	4	0
1580	Ribosomal protein S6 kinase alpha-6	IPI00007123	-0.198	0.32751	4	6	3	6
1581	Isoform 1 of Retinoid-inducible serine carboxypeptidase	IPI00012426	-0.568	0.12806	7	5	7	2
1582	60S ribosomal protein L19	IPI00025329	-1.999	0.00389	6	9	2	3
1583	Isoform 1 of Fermitin family homolog 1	IPI00304754	-0.997	0.04120	4	8	5	2
1584	Acyl-coenzyme A thioesterase 13	IPI00020530	0.406	0.19435	5	3	6	4
1585	Emerin	IPI00032003	0.000	0.37049	6	3	4	5
1586	cDNA FLJ40287 fis, clone TESTI2027909	IPI00473047	-0.815	0.06587	7	4	4	3
1587	Nucleolar protein 11	IPI00303813	-0.916	0.05184	3	6	4	1
1588	Isoform 1 of Nuclear-interacting partner of ALK	IPI00301421	0.000	0.37049	2	2	2	2
1589	Isoform 1 of Insulin-like growth factor 2 mRNA-binding protein 3	IPI00658000	0.000	0.37049	3	4	2	5
1590	60S ribosomal protein L30	IPI00219156	-1.408	0.01536	7	6	5	1
1591	Prefoldin subunit 2	IPI00006052	-2.434	0.00145	6	8	1	2
1592	Putative deoxyribose-phosphate aldolase	IPI00219677	-0.626	0.12011	4	6	3	4
1593	Pre-mRNA branch site protein p14	IPI00032827	-0.198	0.32751	5	5	5	4
1594	Isoform Del-701 of Signal transducer and activator of transcription 3	IPI00306436	0.455	0.17237	3	3	5	3
1595	Fructose-bisphosphate aldolase	IPI00418262	-0.743	0.07647	5	8	4	5
1596	Dual specificity mitogen-activated protein kinase kinase 1	IPI00219604	0.595	0.12525	3	5	6	5
1597	Putative uncharacterized protein DKFZp313O211	IPI00552186	-0.370	0.21613	5	7	6	4
1598	Aladin	IPI00024143	-0.488	0.16467	2	5	3	2
1599	Isoform 2 of N-alpha-acetyltransferase 15, NatA auxiliary subunit	IPI00032158	-0.588	0.12558	2	3	1	2
1600	Serine/threonine-protein kinase PRP4 homolog	IPI00013721	1.487	0.01325	2	3	7	5
1601	Isoform 1 of Telomeric repeat-binding factor 2	IPI00024214	-0.428	0.18789	4	5	4	3
1602	Isoform 1 of Serine/threonine-protein kinase ATR	IPI00412298	0.311	0.25253	2	0	2	2
1603	Ribosome production factor 2 homolog	IPI00396329	-1.585	0.01172	6	5	1	3
1604	Isoform 2 of 39S ribosomal protein L55, mitochondrial	IPI00419626	-0.815	0.06587	5	6	5	2
1605	Syntaxin-binding protein 3	IPI00297626	0.765	0.07536	2	2	5	2
1606	Isoform 1 of Elongation factor Ts, mitochondrial	IPI00021016	-0.455	0.18350	5	3	4	2
1607	DNA-directed RNA polymerase III subunit RPC1	IPI00024163	0.674	0.08856	1	1	2	2
1608	28S ribosomal protein S34, mitochondrial	IPI00169413	0.000	0.37049	2	2	1	3
1609	Isoform 1 of Nucleolar protein 6	IPI00152890	-2.274	0.00178	6	5	0	1
1610	Isoform 1 of THO complex subunit 1	IPI00305374	-0.235	0.30503	3	4	3	3
1611	ATP-binding cassette sub-family F member 2	IPI00005045	-1.394	0.01772	7	3	2	2

No.	Description	Accession number	STN[1]	p-Value[1]	Con_A[2]	Con_B[2]	OXA_A[2]	OXA_B[2]
1612	Fumarylacetoacetate hydrolase domain-containing protein 2B	IPI00301994	-0.530	0.15514	3	3	3	1
1613	TRMT61A protein (Fragment)	IPI00059718	0.428	0.19017	5	2	6	3
1614	SPRY domain-containing protein 4	IPI00291643	0.406	0.19435	5	3	5	5
1615	BAG family molecular chaperone regulator 2	IPI00000643	0.000	0.37049	3	3	3	3
1616	Isoform 2 of PEST proteolytic signal-containing nuclear protein	IPI00060650	0.000	0.37049	3	4	5	2
1617	Isoform 1 of Actin-related protein 3B	IPI00007068	-1.394	0.01772	8	2	3	1
1618	Cation-dependent mannose-6-phosphate receptor	IPI00025049	0.986	0.04066	1	3	4	4
1619	60S ribosomal protein L13	IPI00465361	-1.195	0.03093	5	4	3	1
1620	Cytokine-like nuclear factor n-pac, isoform CRA_a	IPI00000155	-1.121	0.03445	6	8	3	5
1621	Peflin	IPI00018235	-0.387	0.20665	5	6	6	3
1622	Histidine triad nucleotide-binding protein 2, mitochondrial	IPI00000335	-0.708	0.08447	3	5	4	1
1623	Isoform 1 of Oligoribonuclease, mitochondrial (Fragment)	IPI00032830	0.000	0.37049	5	5	4	6
1624	Signal recognition particle receptor subunit alpha	IPI00385267	-1.050	0.03677	5	6	3	3
1625	Thioredoxin-like protein 1	IPI00305692	-0.235	0.30503	5	2	3	3
1626	Phosphoglycerate mutase 2	IPI00218570	-1.945	0.00406	7	6	3	1
1627	TRIP12 protein	IPI00032342	-0.626	0.12011	4	6	5	2
1628	Isoform 1 of Thyroid receptor-interacting protein 13	IPI00003505	0.663	0.11477	3	3	6	3
1629	Heat shock-related 70 kDa protein 2	IPI00007702	1.394	0.01540	1	3	7	3
1630	Isoform 1 of Ataxin-2-like protein	IPI00456359	-2.081	0.00323	5	5	1	0
1631	Calponin-3	IPI00216682	-2.081	0.00323	6	4	1	1
1632	Isoform 1 of Acetolactate synthase-like protein	IPI00554541	0.708	0.08397	3	2	3	5
1633	Isoform 2 of Guanine nucleotide-binding protein-like 3	IPI00003886	-1.050	0.03677	5	6	4	2
1634	Isoform 1 of 2',5'-phosphodiesterase 12	IPI00174390	1.394	0.01540	1	3	4	6
1635	Non-functional aryl hydrocarbon receptor interacting protein (Fragment)	IPI00925804	-1.585	0.01172	6	5	3	1
1636	Pyridoxine-5'-phosphate oxidase	IPI00018272	-0.387	0.20665	6	5	4	5
1637	Isoform B of AP-2 complex subunit alpha-1	IPI00256684	0.663	0.11477	1	5	3	6
1638	AFG3-like protein 2	IPI00001091	-0.208	0.32059	4	5	3	5
1639	Carbonyl reductase [NADPH] 3	IPI00290462	0.387	0.20826	5	4	7	4
1640	Ubiquitin domain-containing protein UBFD1	IPI00005194	-0.545	0.13328	7	6	5	5
1641	Protein C20orf11	IPI00016634	-0.428	0.18789	6	3	3	4
1642	ADP-ribosylation factor-like protein 2	IPI00003326	-0.815	0.06587	7	4	5	2
1643	Thymidylate kinase	IPI00013862	0.455	0.17237	4	2	5	3
1644	U6 snRNA-associated Sm-like protein LSm4	IPI00294955	-0.997	0.04120	7	5	5	2
1645	Pyruvate dehydrogenase protein X component, mitochondrial	IPI00298423	-0.776	0.07324	6	6	3	5
1646	Isoform 1 of V-type proton ATPase subunit H	IPI00296191	-0.235	0.30503	2	5	2	4
1647	Xaa-Pro dipeptidase	IPI00257882	0.708	0.08397	2	3	3	5
1648	Ladinin-1	IPI00514234	-1.768	0.00646	6	6	1	3
1649	dCTP pyrophosphatase 1	IPI00012197	-0.595	0.12310	6	5	4	4
1650	Synaptobrevin homolog YKT6	IPI00008569	0.861	0.05730	3	3	6	4
1651	Uncharacterized protein C7orf50	IPI00031651	-0.997	0.04120	6	6	4	3
1652	RNA-binding protein 12	IPI00550308	0.220	0.31215	4	3	4	4
1653	Similar to Signal peptidase complex subunit 2	IPI00452747	-0.370	0.21613	7	5	5	5
1654	Isoform 1 of tRNA (adenine-N(1)-)-methyltransferase non-catalytic subunit TRM6	IPI00099311	-0.208	0.32228	3	6	3	5
1655	60S ribosomal protein L18a	IPI00026202	0.277	0.27228	3	1	3	2
1656	Isoform 1 of Origin recognition complex subunit 3	IPI00294402	1.487	0.01325	0	4	6	6
1657	Isoform 1 of L-2-hydroxyglutarate dehydrogenase, mitochondrial	IPI00016458	-0.220	0.31447	5	3	4	3
1658	Isoform 1 of Zinc finger ZZ-type and EF-hand domain-containing protein 1	IPI00385631	0.674	0.08856	0	0	3	1
1659	Adenosine monophosphate deaminase 2	IPI00007722	0.000	0.37049	2	0	0	2
1660	lanosterol 14-alpha demethylase isoform 1	IPI00295772	1.305	0.02021	1	4	5	6
1661	aldehyde dehydrogenase 9A1	IPI00479877	1.394	0.01540	2	2	4	6
1662	Cytochrome b-c1 complex subunit 7	IPI00220416	-0.743	0.07647	8	5	4	5
1663	Major centromere autoantigen B	IPI00010388	0.208	0.31861	4	4	4	5
1664	ATPase ASNA1	IPI00013466	0.000	0.37049	2	4	3	3
1665	Peroxisomal membrane protein 2	IPI00221002	0.488	0.16541	2	3	5	2
1666	Isoform 1 of Splicing factor, arginine/serine-rich 15	IPI00181702	-0.387	0.20665	6	5	6	3
1667	Large neutral amino acids transporter small subunit 1	IPI00008986	-3.189	0.00083	12	9	3	1
1668	Transmembrane protein 14C	IPI00009346	0.588	0.12889	2	1	3	2
1669	YLP motif-containing protein 1	IPI00165434	-1.509	0.01342	5	4	2	1
1670	Isoform 1 of 39S ribosomal protein L22, mitochondrial	IPI00414410	0.000	0.37049	5	4	6	3
1671	Catechol O-methyltransferase domain-containing protein 1	IPI00642041	0.000	0.37049	5	4	4	5
1672	Isoform 1 of Interferon-inducible double stranded RNA-dependent protein kinase activator	IPI00021167	-0.708	0.08447	3	5	2	3
1673	Interferon-induced 17 kDa protein	IPI00375631	-0.198	0.32751	6	4	4	5
1674	Cob(I)yrinic acid a,c-diamide adenosyltransferase, mitochondrial	IPI00029665	0.000	0.37049	4	6	3	7
1675	Density-regulated protein	IPI00306280	-0.235	0.30503	5	2	3	3
1676	Heterochromatin protein 1, binding protein 3	IPI00640417	-0.861	0.06115	6	4	4	2
1677	Isoform 2 of TIP41-like protein	IPI00641815	0.000	0.37049	2	2	3	1
1678	DnaJ homolog subfamily C member 7	IPI00329629	-1.487	0.01354	6	6	3	2
1679	Isoform 1 of Protein phosphatase 1 regulatory subunit 12A	IPI00183002	-1.195	0.03093	3	6	2	2
1680	Protein LYRIC	IPI00328715	-1.299	0.02029	4	4	1	2
1681	Isoform 3 of Ester hydrolase C11orf54	IPI00061507	-0.765	0.07552	2	5	2	2
1682	Isoform 1 of HEAT repeat-containing protein 3	IPI00100984	0.488	0.16541	1	4	3	4
1683	Heat shock 70 kDa protein 4L	IPI00295485	-1.078	0.03586	3	4	2	1
1684	Uncharacterized protein KIAA0406	IPI00011702	-0.455	0.18350	3	5	3	3
1685	Nuclear pore glycoprotein p62	IPI00293533	0.406	0.19435	4	4	5	5
1686	U3 small nucleolar RNA-associated protein 15 homolog	IPI00152708	-0.951	0.05006	8	5	3	5
1687	Similar to Protein SAAL1. Isoform 2	IPI00304935	-0.220	0.31447	2	6	3	4
1688	Procollagen-lysine,2-oxoglutarate 5-dioxygenase 3	IPI00030255	0.488	0.16541	3	2	3	4
1689	RhoA activator C11orf59	IPI00016670	-0.708	0.08447	3	5	3	2
1690	Uncharacterized protein C2orf47, mitochondrial	IPI00291751	0.406	0.19435	3	5	5	5
1691	Isoform 2 of Myosin-VI	IPI00008455	1.195	0.03114	3	1	5	4
1692	Ubiquilin-4	IPI00024502	0.000	0.37049	4	4	5	3
1693	Developmentally-regulated GTP-binding protein 2	IPI00022697	-0.455	0.18350	4	4	4	2
1694	Pyrroline-5-carboxylate reductase 2	IPI00470610	-0.568	0.12806	5	7	5	4
1695	Armadillo repeat-containing X-linked protein 3	IPI00009906	-0.428	0.18789	2	7	3	4
1696	Ubiquitin-conjugating enzyme E2 G1	IPI00219783	0.428	0.19017	3	4	5	4
1697	Transmembrane emp24 domain-containing protein 5	IPI00294472	0.000	0.37049	5	3	5	3
1698	Isoform 1 of Multidrug resistance-associated protein 4	IPI00006675	-0.663	0.11221	5	4	5	0
1699	Isoform 1 of HBS1-like protein	IPI00009070	-0.861	0.06115	5	5	4	2
1700	Mortality factor 4-like protein 2	IPI00014174	-0.220	0.31447	4	4	4	3
1701	cDNA FLJ55475	IPI00306017	-0.530	0.15514	3	3	1	3
1702	Guanine nucleotide-binding protein G(I)/G(S)/G(T) subunit beta-2	IPI00003348	-0.220	0.31447	4	4	4	3
1703	dynactin subunit 2	IPI00220503	0.626	0.12177	5	2	5	5
1704	Myristoylated alanine-rich C-kinase substrate	IPI00219301	0.208	0.31861	3	5	5	4
1705	Importin subunit alpha-1	IPI00303292	0.663	0.11477	2	4	5	4
1706	Isoform 1 of Vacuolar protein sorting-associated protein 8 homolog	IPI00464985	0.588	0.12889	1	2	4	1

No.	Description	Accession number	STN[1]	p-Value[1]	Con_A[2]	Con_B[2]	OXA_A[2]	OXA_B[2]
1707	Isoform 1 of 2-oxoglutarate and iron-dependent oxygenase domain-containing protein 1	IPI00170429	0.220	0.31215	3	4	4	4
1708	Isoform 1 of Serum paraoxonase/arylesterase 2	IPI00014958	-0.674	0.10802	2	2	1	1
1709	28S ribosomal protein S18b, mitochondrial	IPI00022316	-0.765	0.07552	3	4	3	1
1710	Isoform 1 of Chromodomain-helicase-DNA-binding protein 1	IPI00297851	-0.626	0.12011	5	5	5	2
1711	Mitochondrial import receptor subunit TOM20 homolog	IPI00016676	-0.235	0.30503	4	3	4	2
1712	Isoform 2 of Phosphoenolpyruvate carboxykinase [GTP], mitochondrial	IPI00384116	-2.315	0.00174	7	10	2	3
1713	WD40 repeat-containing protein SMU1	IPI00305833	0.663	0.11477	3	3	3	6
1714	Isoform 1 of U4/U6 small nuclear ribonucleoprotein Prp4	IPI00150269	-0.861	0.06115	6	4	4	2
1715	Isoform 1 of Serine/threonine-protein phosphatase 6 catalytic subunit	IPI00012970	-0.208	0.32059	3	6	4	4
1716	DnaJ homolog subfamily C member 9	IPI00154975	0.208	0.31861	4	4	4	5
1717	Isoform 2 of Ribosomal RNA processing protein 1 homolog B	IPI00032374	-0.765	0.07552	4	3	2	2
1718	Isoform 2 of NADH dehydrogenase [ubiquinone] flavoprotein 3, mitochondrial	IPI00291016	0.000	0.37049	4	3	4	3
1719	Brefeldin A-inhibited guanine nucleotide-exchange protein 2	IPI00002186	-0.530	0.15514	4	2	3	1
1720	tRNA methyltransferase 112 homolog	IPI00009010	-0.595	0.12310	5	6	4	4
1721	Eukaryotic translation initiation factor 4A, isoform 2, isoform CRA_b	IPI00030296	-0.708	0.08447	4	4	2	3
1722	Translocation protein SEC63 homolog	IPI00218922	-1.305	0.02021	5	6	1	4
1723	1,4-alpha-glucan-branching enzyme	IPI00296635	-0.488	0.16467	4	3	4	1
1724	28S ribosomal protein S9, mitochondrial	IPI00641924	-1.394	0.01772	5	5	2	2
1725	ADP-ribosylation factor-like protein 3	IPI00003327	0.000	0.37049	5	5	4	6
1726	Metaxin-2	IPI00025717	-0.626	0.12011	4	6	4	3
1727	Cell differentiation protein RCD1 homolog	IPI00023101	-0.530	0.15514	3	3	3	1
1728	28S ribosomal protein S6, mitochondrial	IPI00305668	-0.674	0.10802	3	0	1	1
1729	N-alpha-acetyltransferase 10, NatA catalytic subunit	IPI00013184	0.000	0.37049	2	1	2	1
1730	Isoform 1 of AP-3 complex subunit beta-1	IPI00021129	-0.455	0.18350	5	3	4	2
1731	Keratin, type I cytoskeletal 17	IPI00450768	0.000	0.37049	0	0	0	0
1732	Isoform A of Nucleoporin SEH1	IPI00185533	1.078	0.03610	2	1	5	2
1733	Histone H1x	IPI00021924	-1.664	0.01068	7	6	3	2
1734	Dolichyldiphosphatase 1	IPI00329410	-0.455	0.18350	5	3	5	0
1735	Thioredoxin-interacting protein	IPI00007956	-2.460	0.00132	7	5	1	0
1736	Isoform 2 of Tether containing UBX domain for GLUT4	IPI00065276	-1.394	0.01772	4	6	2	2
1737	Retinol dehydrogenase 13	IPI00301204	0.000	0.37049	5	2	4	3
1738	twinfilin-1	IPI00183508	0.000	0.37049	3	3	3	3
1739	Isoform 1 of Mammalian ependymin-related protein 1	IPI00259102	-0.253	0.29716	4	2	2	3
1740	X-Pro aminopeptidase 1, soluble isoform 2	IPI00607814	0.000	0.37049	2	3	4	0
1741	Cohesin subunit SA-1	IPI00025158	0.220	0.31215	4	3	5	3
1742	Full-length cDNA 5-PRIME end of clone CS0DJ009YL13 of T cells (Jurkat cell line)	IPI00384016	-0.220	0.31447	4	4	3	4
1743	Deoxyhypusine hydroxylase	IPI00171856	-0.815	0.06587	5	6	4	3
1744	Isoform 1 of Protein-glutamine gamma-glutamyltransferase 2	IPI00294578	0.588	0.12889	2	1	3	2
1745	Dehydrogenase/reductase SDR family member 7B	IPI00550165	0.626	0.12177	3	4	7	3
1746	Isoform 1 of Neuroblastoma-amplified sequence	IPI00333913	0.000	0.37049	1	1	1	1
1747	Isoform Long of Tight junction protein ZO-1	IPI00216219	0.000	0.37049	0	0	0	0
1748	Isoform 1 of Ras-related protein Rab-6A	IPI00023526	0.000	0.37049	4	5	5	4
1749	Hsp90 co-chaperone Cdc37	IPI00013122	-0.595	0.12310	6	5	5	3
1750	Synaptotagmin-1	IPI00009439	-0.277	0.27840	2	3	3	1
1751	Isoform 2 of Ubiquilin-1	IPI00071180	-0.595	0.12310	6	5	4	4
1752	Isoform 1 of IST1 homolog	IPI00024660	0.000	0.37049	5	2	3	4
1753	Isoform 3 of Guanine nucleotide exchange factor VAV2	IPI00004977	0.000	0.37049	2	3	3	2
1754	Isoform Long of Beta-glucuronidase	IPI00027745	1.078	0.03610	1	2	3	4
1755	V-type proton ATPase subunit C 1	IPI00007814	0.916	0.05271	2	3	5	4
1756	Isoform 1 of HEAT repeat-containing protein 2	IPI00242630	0.253	0.29095	2	3	2	4
1757	Vacuolar protein sorting-associated protein 33A	IPI00073179	1.195	0.03114	3	1	5	4
1758	UDP-galactose-4-epimerase	IPI00030229	-0.406	0.20280	4	6	3	5
1759	Isoform 1 of Putative deoxyribonuclease TATDN1	IPI00012463	-1.768	0.00646	7	5	3	0
1760	mRNA export factor	IPI00019733	0.000	0.37049	4	2	3	3
1761	Isoform Long of Cold shock domain-containing protein E1	IPI00470891	0.488	0.16541	3	2	4	3
1762	Ribosomal protein S6 kinase alpha-1	IPI00017305	1.880	0.00426	0	1	4	5
1763	Sterol-4-alpha-carboxylate 3-dehydrogenase, decarboxylating	IPI00019407	-1.445	0.01457	3	4	1	1
1764	Isoform 1 of Melanoma-associated antigen D2	IPI00009542	0.220	0.31215	3	4	5	3
1765	Peptidyl-prolyl cis-trans isomerase F, mitochondrial	IPI00026519	-0.455	0.18350	5	3	4	2
1766	Mitochondrial import inner membrane translocase subunit Tim17-B	IPI00219833	-0.428	0.18789	5	4	3	4
1767	Myosin light chain 6B	IPI00027255	0.455	0.17237	3	3	3	5
1768	Serine/threonine-protein phosphatase 2A 56 kDa regulatory subunit epsilon isoform	IPI00002853	-0.488	0.16467	4	3	1	4
1769	Diphosphoinositol polyphosphate phosphohydrolase 1	IPI00009148	-0.708	0.08447	4	4	3	2
1770	DNA-directed RNA polymerase II subunit RPB7	IPI00218895	0.997	0.04049	3	4	7	5
1771	Isoform 1 of Aldehyde dehydrogenase family 16 member A1	IPI00217920	0.000	0.37049	6	2	5	3
1772	Isoform 3 of Transcription elongation factor SPT6	IPI00456683	0.674	0.08856	0	0	3	1
1773	NFU1 iron-sulfur cluster scaffold homolog, mitochondrial isoform 1	IPI00160021	1.233	0.02186	2	4	6	6
1774	Activity-dependent neuroprotector homeobox protein	IPI00022215	-1.233	0.02323	4	8	3	3
1775	Leucine-rich repeat and WD repeat-containing protein 1	IPI00069309	0.986	0.04066	1	3	4	4
1776	Isoform 1 of Cullin-4A	IPI00419273	0.952	0.04819	0	1	2	3
1777	Ewing sarcoma breakpoint region 1 isoform 1	IPI00009841	0.952	0.04819	1	1	4	1
1778	Replication initiator 1	IPI00549171	0.455	0.17237	2	4	4	4
1779	Isoform 1 of Ribose-phosphate pyrophosphokinase 2	IPI00219617	-1.342	0.01958	7	7	3	4
1780	Protein transport protein Sec23B	IPI00017376	0.588	0.12889	2	1	2	3
1781	Isoform 1 of Peroxisomal membrane protein PEX16	IPI00006722	0.588	0.12889	2	0	4	1
1782	Phosphomannomutase 2	IPI00006092	-0.387	0.20665	5	6	5	4
1783	Isoform 1 of Leucine-rich repeat-containing protein 16A	IPI00014843	0.362	0.21837	1	0	2	1
1784	28 kDa heat- and acid-stable phosphoprotein	IPI00013297	-0.626	0.12011	6	4	4	3
1785	Hexokinase-2	IPI00102864	-2.081	0.00323	4	6	1	1
1786	Isoform 3 of Rapamycin-insensitive companion of mTOR	IPI00166528	0.000	0.37049	1	4	4	1
1787	NEDD8-activating enzyme E1 regulatory subunit	IPI00018968	0.663	0.11477	2	4	3	6
1788	Propionyl-CoA carboxylase beta chain, mitochondrial	IPI00007247	-0.842	0.06318	3	3	2	1
1789	Dual specificity protein phosphatase 3	IPI00018671	-0.815	0.06587	5	6	4	3
1790	Ran-binding protein 6	IPI00514622	1.709	0.00712	1	2	4	6
1791	Isoform 1 of Ras GTPase-activating protein 1	IPI00026262	0.000	0.37049	0	3	1	3
1792	Epoxide hydrolase 1	IPI00009896	0.674	0.08856	0	1	3	1
1793	Isoform 2 of Liprin-beta-1	IPI00179172	1.115	0.03515	4	0	7	3
1794	Isoform 1 of Dehydrogenase/reductase SDR family member 7	IPI00006957	0.000	0.37049	3	5	4	4
1795	39S ribosomal protein L38, mitochondrial	IPI00783656	-0.253	0.29716	2	4	3	2
1796	39S ribosomal protein L40, mitochondrial	IPI00099871	-1.195	0.03093	4	5	3	1
1797	Regulator of microtubule dynamics protein 1	IPI00329696	0.220	0.31215	4	3	4	4
1798	DNA mismatch repair protein Msh3	IPI00329605	1.445	0.01433	0	0	5	2
1799	Protein FAM162A	IPI00023001	-0.663	0.11221	5	4	4	2
1800	Isoform 1 of Cleft lip and palate transmembrane protein 1-like proteir	IPI00151358	-0.311	0.26205	3	0	2	1
1801	Hepatoma-derived growth factor-related protein 3	IPI00007063	-0.530	0.15514	3	3	2	2

No.	Description	Accession number	STN[1]	p-Value[1]	Con_A[2]	Con_B[2]	OXA_A[2]	OXA_B[2]
1802	Isoform 1 of Prolyl 4-hydroxylase subunit alpha-1	IPI00009923	-0.208	0.32059	4	5	3	5
1803	Isoform 2 of CDK5 regulatory subunit-associated protein 3	IPI00018780	-0.220	0.31447	3	5	4	3
1804	Isoform 1 of Pleiotropic regulator 1	IPI00002624	-0.842	0.06318	4	2	2	1
1805	Band 4.1-like protein 2	IPI00015973	1.445	0.01433	1	1	5	2
1806	Protein ERGIC-53	IPI00026530	0.000	0.37049	4	5	3	6
1807	Isoform A1 of Tight junction protein ZO-2	IPI00003843	-0.588	0.12558	4	1	2	1
1808	Isoform 2 of DnaJ homolog subfamily A member 3, mitochondrial	IPI00179187	-0.253	0.29716	3	3	2	3
1809	WD repeat-containing protein 3	IPI00009471	-2.274	0.00178	8	3	1	0
1810	Importin-8	IPI00007401	0.253	0.29095	3	2	4	2
1811	Scavenger mRNA-decapping enzyme DcpS	IPI00335385	-0.387	0.20665	5	6	4	5
1812	Cysteine and glycine-rich protein 1	IPI00442073	0.455	0.17237	3	3	4	4
1813	MARCKS-related protein	IPI00641181	0.000	0.37049	4	2	3	3
1814	Isoform 1 of 28S ribosomal protein S35, mitochondrial	IPI00073779	-1.115	0.03482	4	6	3	2
1815	Probable dimethyladenosine transferase	IPI00004459	-1.299	0.02029	4	4	2	0
1816	ADP-ribosylation factor 5	IPI00215919	0.428	0.19017	4	3	5	4
1817	Isoform 1 of CD109 antigen	IPI00152540	0.220	0.31215	4	3	7	0
1818	annexin A6 isoform 2	IPI00002459	0.674	0.08856	0	1	1	3
1819	cytochrome c oxidase subunit VIIa polypeptide 2 (liver) precursor	IPI00026570	0.000	0.37049	3	2	3	2
1820	Short/branched chain specific acyl-CoA dehydrogenase, mitochondrial	IPI00024623	-0.428	0.18789	4	5	3	4
1821	Macrophage-capping protein	IPI00027341	0.428	0.19017	3	4	4	5
1822	22 kDa protein	IPI00219910	-0.916	0.05184	5	4	2	3
1823	Isoform 4 of Abhydrolase domain-containing protein 11	IPI00171152	0.455	0.17237	3	3	5	3
1824	U6 snRNA-associated Sm-like protein LSm2	IPI00032460	-0.220	0.31447	5	3	4	3
1825	13kDa differentiation-associated protein variant (Fragment)	IPI00005966	-1.078	0.03586	4	3	2	1
1826	Isoform Long of Transformer-2 protein homolog alpha	IPI00013891	0.000	0.37049	4	6	6	4
1827	Phosphatidylserine synthase 1	IPI00010746	0.000	0.37049	3	2	3	2
1828	Peptidyl-tRNA hydrolase 2, mitochondrial	IPI00032903	0.000	0.37049	3	2	4	1
1829	Isoform 2 of Calpastatin	IPI00220857	-0.765	0.07552	3	4	2	2
1830	Retinoblastoma-associated protein	IPI00302829	1.901	0.00414	2	1	5	6
1831	Protein FAM3C	IPI00334282	0.626	0.12177	2	5	5	5
1832	Isoform 2 of Protein FAM36A	IPI00103057	0.708	0.08397	4	1	5	3
1833	Na(+)/H(+) exchange regulatory cofactor NHE-RF1	IPI00003527	-0.595	0.12310	8	3	4	4
1834	Ribose-phosphate pyrophosphokinase 3	IPI00218371	-0.663	0.11221	5	4	3	3
1835	Centromere/kinetochore protein zw10 homolog	IPI00011631	0.986	0.04066	3	0	5	3
1836	Neuron-specific calcium-binding protein hippocalcin	IPI00219103	-0.455	0.18350	4	4	5	1
1837	NEDD8-conjugating enzyme Ubc12	IPI00022597	0.235	0.30171	4	2	5	2
1838	Isoform 1 of Drebrin	IPI00003406	0.406	0.19435	5	3	6	4
1839	Toll-interacting protein	IPI00100154	-1.115	0.03482	5	5	3	2
1840	cDNA FLJ55382, highly similar to Hsp70-binding protein 1	IPI00100748	-0.588	0.12558	2	3	1	2
1841	Isoform 4 of Afadin	IPI00023461	-1.208	0.03035	3	3	0	1
1842	Cytovillin 2 (Fragment)	IPI00384282	0.428	0.19017	3	4	5	4
1843	CCAAT/enhancer-binding protein zeta	IPI00306723	-1.115	0.03482	6	4	2	3
1844	114 kDa protein	IPI00166555	-1.487	0.01354	6	6	2	3
1845	Isoform 1 of Serine/threonine-protein phosphatase 2A 55 kDa regulatory subunit B beta isoform	IPI00020850	-1.115	0.03482	5	5	2	3
1846	39S ribosomal protein L48, mitochondrial	IPI00295066	-0.208	0.32059	6	3	4	4
1847	Transmembrane protein 2	IPI00170706	0.952	0.04819	1	1	3	2
1848	Methylosome subunit pICln	IPI00004795	0.253	0.29095	3	2	3	3
1849	cDNA FLJ14048 fis, clone HEMBA1006650, weakly similar to ARP2/3 COMPLEX 20 KD	IPI00386354	-0.220	0.31447	5	3	5	2
1850	Isoform 2 of Myosin-XVIIIa	IPI00334410	1.078	0.03610	2	1	4	3
1851	Actin-related protein 2/3 complex subunit 5-like protein	IPI00414554	0.235	0.30171	3	3	5	2
1852	Isoform 2 of Protein SET	IPI00301311	-0.428	0.18789	4	5	3	4
1853	Isoform 2 of mRNA cap guanine-N7 methyltransferase	IPI00410657	-0.208	0.32059	5	4	4	4
1854	Isoform 1 of Growth factor receptor-bound protein 2	IPI00021327	0.000	0.37049	2	3	3	2
1855	Isoform 2 of Serine-protein kinase ATM	IPI00289986	0.000	0.37049	1	3	3	0
1856	Amidophosphoribosyltransferase	IPI00029534	-0.916	0.05184	5	4	3	2
1857	NADH dehydrogenase [ubiquinone] 1 alpha subcomplex subunit 8	IPI00219034	-0.311	0.26205	2	2	1	2
1858	HIV Tat-specific factor 1	IPI00013788	0.842	0.06364	2	0	3	3
1859	Epidermal growth factor receptor kinase substrate 8	IPI00290337	-0.708	0.08447	3	5	4	1
1860	Serine/threonine-protein phosphatase PP1-beta catalytic subunit	IPI00218236	-0.986	0.04534	3	5	3	1
1861	Isoform 1 of SET domain-containing protein 3	IPI00165026	-0.220	0.31447	4	4	4	3
1862	LanC-like protein 2	IPI00032995	-0.765	0.07552	2	5	3	1
1863	NADH dehydrogenase [ubiquinone] 1 beta subcomplex subunit 8, mitochondrial	IPI00028883	0.253	0.29095	2	3	4	2
1864	G-rich sequence factor 1	IPI00478657	0.674	0.08856	1	1	2	2
1865	Isoform 1 of Ras-related GTP-binding protein D	IPI00009737	-0.235	0.30503	4	3	3	3
1866	Isoform 1 of Serine protease HTRA2, mitochondrial	IPI00001663	0.000	0.37049	4	1	3	2
1867	F-box-like/WD repeat-containing protein TBL1XR1	IPI00002922	-0.428	0.18789	4	5	3	4
1868	Ethanolamine-phosphate cytidylyltransferase	IPI00015285	-0.220	0.31447	4	4	4	3
1869	UBX domain-containing protein 4	IPI00293946	0.000	0.37049	4	5	4	5
1870	Choline dehydrogenase, mitochondrial	IPI00168603	0.000	0.37049	3	2	2	3
1871	Isoform 1 of Nicalin	IPI00470649	1.195	0.03114	2	2	5	4
1872	BRI3-binding protein	IPI00103599	-0.235	0.30503	3	4	4	2
1873	Succinyl-CoA ligase [GDP-forming] subunit alpha, mitochondrial	IPI00872762	-0.663	0.11221	5	4	3	3
1874	Isoform 1 of Cell division cycle protein 23 homolog	IPI00005822	0.588	0.12889	2	1	3	2
1875	NADH dehydrogenase [ubiquinone] 1 alpha subcomplex subunit 4	IPI00011770	1.208	0.02377	1	1	4	2
1876	39S ribosomal protein L45, mitochondrial	IPI00185859	0.000	0.37049	2	2	3	0
1877	Isoform 1 of CDP-diacylglycerol--inositol 3-phosphatidyltransferase	IPI00645518	0.000	0.37049	6	0	5	2
1878	fatty acid desaturase 1	IPI00784651	0.311	0.25253	2	0	3	0
1879	18 kDa protein	IPI00797709	0.000	0.37049	2	2	2	2
1880	RER1 protein	IPI00005728	-0.663	0.11221	5	4	3	3
1881	Prefoldin subunit 4	IPI00015891	0.000	0.37049	4	4	4	4
1882	Isoform 1 of 60S ribosome subunit biogenesis protein NIP7 homolog	IPI00007175	-0.253	0.29716	3	3	3	2
1883	similar to RAN binding protein 1	IPI00399212	-0.626	0.12011	6	4	4	3
1884	Isoform 2 of Gamma-glutamylcyclotransferase	IPI00020301	0.000	0.37049	4	4	4	4
1885	Uncharacterized protein C11orf73	IPI00410091	-0.488	0.16467	4	3	4	1
1886	GTP-binding protein SAR1b	IPI00002149	0.000	0.37049	4	4	5	3
1887	Serine/threonine-protein phosphatase 2A 65 kDa regulatory subunit A alpha isoform	IPI00554737	-0.530	0.15514	2	4	2	2
1888	Peptidyl-prolyl cis-trans isomerase FKBP2	IPI00002535	-0.277	0.27840	4	0	1	3
1889	39S ribosomal protein L37, mitochondrial	IPI00162330	0.674	0.08856	1	1	2	2
1890	Probable fructose-2,6-bisphosphatase TIGAR	IPI00006907	-0.455	0.18350	5	3	3	3
1891	Isoform 1 of Serine/threonine-protein kinase WNK1	IPI00004472	-0.842	0.06318	2	4	2	0
1892	General transcription factor 3C polypeptide 4	IPI00016725	-0.765	0.07552	3	4	3	1
1893	Isoform 1 of Nuclear pore complex protein Nup214	IPI00183294	-1.208	0.03035	2	4	1	1
1894	Isoform 1 of Phosphatidylinositol-3,4,5-trisphosphate 5-phosphatase 2	IPI00016932	0.311	0.25253	2	0	2	2
1895	Leucine-rich repeat-containing protein 40	IPI00152998	-0.311	0.26205	2	2	2	0
1896	Glutamate--cysteine ligase catalytic subunit	IPI00215768	0.000	0.37049	3	1	3	0

No.	Description	Accession number	STN[1]	p-Value[1]	Con_A[2]	Con_B[2]	OXA_A[2]	OXA_B[2]
1897	Osteoclast-stimulating factor 1	IPI00414836	-0.235	0.30503	4	3	3	3
1898	cDNA FLJ56047, highly similar to A kinase anchor protein 1, mitochondrial	IPI00022585	0.000	0.37049	0	4	1	4
1899	Isoform 1 of Gelsolin	IPI00026314	0.952	0.04819	0	1	3	2
1900	Putative uncharacterized protein THADA	IPI00412647	0.000	0.37049	0	3	3	1
1901	Isoform 1 of Transmembrane protein 70, mitochondrial	IPI00106966	-1.208	0.03035	2	4	0	1
1902	Isoform 1 of Beta-galactosidase	IPI00441344	0.588	0.12889	1	2	2	3
1903	Probable ribosome biogenesis protein NEP1	IPI00025347	-3.143	0.00083	9	7	1	0
1904	Isoform 1 of Zinc phosphodiesterase ELAC protein 2	IPI00396627	-0.362	0.22785	1	2	1	1
1905	Isoform 1 of OCIA domain-containing protein 1	IPI00016405	0.235	0.30171	3	3	4	3
1906	Protein phosphatase inhibitor 2	IPI00220402	0.235	0.30171	4	2	4	3
1907	39S ribosomal protein L41, mitochondrial	IPI00217553	-1.445	0.01457	6	0	1	0
1908	Isoform 2 of Ubiquitin carboxyl-terminal hydrolase 47	IPI00165528	-0.952	0.04981	2	3	1	1
1909	Tubulin beta-3 chain	IPI00013683	1.901	0.00414	1	2	7	4
1910	ATP-dependent RNA helicase DDX50	IPI00031554	-0.488	0.16467	5	2	2	3
1911	Isoform 2 of Pinin	IPI00002649	0.000	0.37049	3	2	2	3
1912	Transcriptional activator protein Pur-alpha	IPI00023591	-0.253	0.29716	3	3	2	3
1913	Ras-related protein Rab-5A	IPI00023510	-0.488	0.16467	4	3	2	3
1914	cDNA, FLJ79450, highly similar to 3-ketoacyl-CoA thiolase, peroxisomal	IPI00011522	0.455	0.17237	3	3	4	4
1915	Cytochrome c1, heme protein, mitochondrial	IPI00029264	-0.588	0.12558	4	1	2	1
1916	ATP synthase mitochondrial F1 complex assembly factor 2	IPI00296999	0.235	0.30171	5	1	5	2
1917	Isoform 1 of 39S ribosomal protein L47, mitochondrial	IPI00030820	-0.253	0.29716	2	4	3	2
1918	Isoform 1 of RNA-binding protein 4	IPI00003704	-0.842	0.06318	3	3	1	2
1919	39S ribosomal protein L17, mitochondrial	IPI00172591	-0.986	0.04534	6	2	3	1
1920	Isoform 1 of Erlin-2	IPI00026942	-0.235	0.30503	2	5	3	3
1921	Ribonuclease UK114	IPI00005038	0.000	0.37049	4	4	4	4
1922	Sorting nexin-9	IPI00001883	-1.115	0.03482	4	6	1	4
1923	Pre-mRNA-splicing factor SPF27	IPI00025178	0.000	0.37049	3	5	3	5
1924	COMM domain-containing protein 2	IPI00456048	-0.530	0.15514	4	2	3	0
1925	Probable RNA-binding protein 19	IPI00000686	-0.362	0.22785	0	2	1	1
1926	Isoform 1 of Exosome component 10	IPI00009464	0.952	0.04819	1	0	2	3
1927	Importin 5	IPI00639960	-0.815	0.06587	4	7	3	4
1928	Translocated promoter region	IPI00514531	-0.208	0.32059	5	4	3	5
1929	Isoform 1 of Interleukin enhancer-binding factor 3	IPI00298788	-0.428	0.18789	5	4	3	4
1930	Isoform 1 of Histone-arginine methyltransferase CARM1	IPI00412880	0.000	0.37049	3	1	3	1
1931	Guanine nucleotide-binding protein G(olf) subunit alpha	IPI00006395	-0.916	0.05184	5	4	2	3
1932	Malectin	IPI00029046	-0.220	0.31447	5	3	4	3
1933	Glutamate-rich WD repeat-containing protein 1	IPI00027831	0.235	0.30171	3	3	4	3
1934	Isoform 2 of Low molecular weight phosphotyrosine protein phosphatase	IPI00218847	-0.406	0.20280	5	5	5	3
1935	Isoform 1 of DAZ-associated protein 1	IPI00165230	-1.305	0.02021	5	6	2	3
1936	Isoform 1 of Integrator complex subunit 4	IPI00446765	-0.674	0.10802	1	3	0	1
1937	Astrocytic phosphoprotein PEA-15	IPI00014850	0.952	0.04819	1	0	4	0
1938	39S ribosomal protein L27, mitochondrial	IPI00009444	-0.708	0.08447	4	4	2	3
1939	Isoform 3 of Serine/threonine-protein kinase SMG1	IPI00183368	1.509	0.01308	2	1	7	2
1940	Isoform 2 of Phosphatidylinositol-binding clathrin assembly protein	IPI00216184	-1.509	0.01342	4	5	1	2
1941	Gamma-taxilin	IPI00019994	-0.488	0.16467	3	4	3	2
1942	Aminopeptidase B	IPI00642211	-0.235	0.30503	3	4	5	1
1943	Charged multivesicular body protein 4b	IPI00025974	-0.861	0.06115	5	5	3	3
1944	Isoform 1 of Cell division protein kinase 9	IPI00301923	-0.674	0.10802	1	3	1	0
1945	Isoform 2 of Ubiquitin conjugation factor E4 A	IPI00028957	0.000	0.37049	2	5	3	4
1946	Isoform A of Kinesin light chain 1	IPI00020096	1.445	0.01433	1	1	3	4
1947	RNA-binding protein NOB1	IPI00022373	-0.986	0.04534	4	4	1	3
1948	U3 small nucleolar RNA-associated protein 18 homolog	IPI00000733	-0.488	0.16467	2	5	2	3
1949	NADH dehydrogenase [ubiquinone] 1 beta subcomplex subunit 4	IPI00220059	-0.488	0.16467	5	2	3	2
1950	Exosome complex exonuclease RRP43	IPI00552920	-0.428	0.18789	5	4	5	2
1951	Isoform 1 of Translation initiation factor eIF-2B subunit delta	IPI00005979	0.000	0.37049	2	2	2	2
1952	cDNA FLJ55158, highly similar to Thioredoxin, mitochondrial	IPI00017799	-0.842	0.06318	5	1	2	1
1953	TATA-binding protein-associated factor 172	IPI00024802	1.208	0.02377	1	0	5	1
1954	Isoform 1 of Uncharacterized protein C1orf77	IPI00300990	0.000	0.37049	2	5	3	4
1955	26S proteasome non-ATPase regulatory subunit 10	IPI00003565	0.530	0.13800	2	2	3	3
1956	Isoform 1 of Fatty aldehyde dehydrogenase	IPI00333619	0.000	0.37049	4	3	3	4
1957	Atlastin-1	IPI00103530	0.000	0.37049	2	3	2	3
1958	NDUFB10 protein	IPI00074489	-0.488	0.16467	5	2	3	2
1959	sideroflexin-3	IPI00793874	-1.115	0.03482	5	5	2	3
1960	Pyroglutamyl-peptidase 1	IPI00020539	0.277	0.27228	3	1	4	1
1961	Ras-related protein Rab-18	IPI00014577	0.406	0.19435	3	5	5	5
1962	16 kDa protein	IPI00293975	-0.765	0.07552	4	3	3	1
1963	Isoform 1 of Alpha-parvin	IPI00018963	0.626	0.12177	3	4	5	5
1964	Nitric oxide synthase-interacting protein	IPI00006408	0.000	0.37049	4	2	3	3
1965	ATP synthase subunit g, mitochondrial	IPI00027448	0.253	0.29095	2	3	4	2
1966	Isoform 1 of Regulator of microtubule dynamics protein 3	IPI00410079	0.000	0.37049	2	4	3	3
1967	Peptidylprolyl isomerase domain and WD repeat-containing protein 1	IPI00149650	2.081	0.00240	1	1	5	5
1968	Isoform 2 of Hydroxysteroid dehydrogenase-like protein 2	IPI00031107	0.220	0.31215	3	4	5	3
1969	Isoform 1 of Protein LSM12 homolog	IPI00410324	-0.488	0.16467	2	5	3	2
1970	Isoform 2 of Diphosphoinositol polyphosphate phosphohydrolase 2	IPI00021408	-1.050	0.03677	7	4	4	2
1971	LIM and cysteine-rich domains protein 1	IPI00303258	0.000	0.37049	3	1	1	3
1972	14 kDa protein	IPI00179589	-0.916	0.05184	5	4	3	2
1973	Kinetochore-associated protein 1	IPI00001458	1.208	0.02377	0	1	5	0
1974	Ribosome biogenesis protein BOP1	IPI00028955	-2.980	0.00087	8	7	1	0
1975	Isoform 1 of OCIA domain-containing protein 2	IPI00555902	-0.530	0.15514	5	1	3	1
1976	Tubulin-folding cofactor B	IPI00293126	-0.765	0.07552	4	3	2	2
1977	Isoform 1 of Nicastrin	IPI00021983	1.208	0.02377	1	1	4	2
1978	Isoform 1 of Luc7-like protein 3	IPI00107745	-1.233	0.02323	5	7	2	4
1979	Serine/threonine-protein phosphatase 2A 55 kDa regulatory subunit B alpha isoform	IPI00332511	0.277	0.27228	2	2	2	3
1980	Isoform Long of Metastasis-associated protein MTA1	IPI00012773	0.277	0.27228	2	2	2	3
1981	Similar to Zinc finger CCCH domain-containing protein 15	IPI00000279	-1.709	0.00778	3	7	1	2
1982	Isoform 2 of Serine/threonine-protein kinase PAK 1	IPI00289746	0.000	0.37049	3	3	3	3
1983	Thiopurine S-methyltransferase	IPI00019400	-0.488	0.16467	3	4	2	3
1984	Isoform 1 of SEC23-interacting protein	IPI00026969	0.362	0.21837	1	1	1	2
1985	Serine/threonine-protein phosphatase 4 catalytic subunit	IPI00012833	-0.455	0.18350	3	5	3	3
1986	Carboxymethylenebutenolidase homolog	IPI00383046	0.765	0.07536	0	3	3	4
1987	Isoform 1 of RNA polymerase II-associated protein 3	IPI00002408	1.299	0.02029	1	2	5	3
1988	Putative uncharacterized protein DKFZp686E2459	IPI00375731	0.000	0.37049	3	4	3	4
1989	Isoform 3 of Centromere protein V	IPI00376481	-1.305	0.02021	4	7	2	3
1990	A-kinase anchor protein 8	IPI00014474	0.000	0.37049	2	3	1	4
1991	sulfatase modifying factor 2 isoform b precursor	IPI00171412	-0.952	0.04981	2	3	1	1

No.	Description	Accession number	STN[1]	p-Value[1]	Con_A[2]	Con_B[2]	OXA_A[2]	OXA_B[2]
1992	Uncharacterized protein KIAA1797	IPI00748360	0.765	0.07536	1	3	4	3
1993	Heat shock 70 kDa protein 14	IPI00292499	0.952	0.04819	1	1	3	2
1994	Casein kinase I isoform alpha-like	IPI00167096	-0.253	0.29716	3	3	2	3
1995	Phosphoglycolate phosphatase	IPI00177008	-0.765	0.07552	3	4	2	2
1996	Pre-rRNA-processing protein TSR1 homolog	IPI00292894	-0.663	0.11221	5	4	3	3
1997	Tyrosine-protein phosphatase non-receptor type 23	IPI00034006	0.000	0.37049	3	1	3	1
1998	Exosome complex exonuclease RRP42	IPI00014198	-1.208	0.03035	3	3	1	1
1999	Nucleoporin 54kDa variant (Fragment)	IPI00172580	0.916	0.05271	2	3	5	4
2000	Sulfide:quinone oxidoreductase, mitochondrial	IPI00009634	-0.663	0.11221	5	4	3	3
2001	Isoform 1 of N-alpha-acetyltransferase 50, NatE catalytic subunit	IPI00018627	-0.455	0.18350	3	5	3	3
2002	Vacuolar protein sorting-associated protein VTA1 homolog	IPI00017160	-0.663	0.11221	5	4	3	3
2003	DNA-directed RNA polymerases I, II, and III subunit RPABC3	IPI00003309	0.000	0.37049	3	3	3	3
2004	Glutathione peroxidase 2	IPI00298176	0.235	0.30171	3	3	3	4
2005	Apolipoprotein O-like	IPI00394809	0.674	0.08856	0	1	2	2
2006	U6 snRNA-associated Sm-like protein LSm3	IPI00219229	0.311	0.25253	2	0	3	1
2007	Isoform 1 of Chromodomain-helicase-DNA-binding protein 8	IPI00398992	0.000	0.37049	0	0	0	1
2008	Isoform 2 of Lysine-specific histone demethylase 1A	IPI00217540	0.406	0.19435	5	3	5	5
2009	Plexin B2	IPI00852623	-0.277	0.27840	4	1	3	1
2010	TP53-regulating kinase	IPI00290305	-0.253	0.29716	4	2	3	2
2011	Isoform 1 of Prostaglandin reductase 2	IPI00167515	-0.277	0.27840	2	3	1	3
2012	Glycylpeptide N-tetradecanoyltransferase 2	IPI00030223	-0.663	0.11221	4	5	3	3
2013	Replication factor C subunit 3	IPI00031521	0.311	0.25253	2	1	2	2
2014	negative elongation factor A	IPI00394679	-0.530	0.15514	3	3	2	2
2015	Isoform GTBP-N of DNA mismatch repair protein Msh6	IPI00384456	-0.916	0.05184	3	6	3	2
2016	Peptidyl-prolyl cis-trans isomerase-like 1	IPI00007019	0.253	0.29095	3	2	3	3
2017	Acyl-protein thioesterase 2	IPI00027032	0.253	0.29095	2	3	3	3
2018	Isoform 1 of Partner of Y14 and mago	IPI00305092	0.311	0.25253	1	2	2	2
2019	Isoform Long of Tyrosine-protein kinase SYK	IPI00018597	1.195	0.03114	2	2	5	4
2020	Probable ATP-dependent RNA helicase DDX27	IPI00293078	-1.709	0.00778	5	5	2	0
2021	Cytochrome c oxidase subunit 5B, mitochondrial	IPI00021785	-0.488	0.16467	4	3	3	2
2022	Isoform 1 of HCLS1-associated protein X-1	IPI00010440	-0.488	0.16467	3	4	4	0
2023	40S ribosomal protein S21	IPI00017448	0.000	0.37049	3	5	5	3
2024	DNA polymerase	IPI00744598	0.674	0.08856	1	0	3	0
2025	Protein	IPI00892529	-0.253	0.29716	3	3	2	3
2026	BRO1 domain-containing protein BROX	IPI00065500	0.663	0.11477	3	3	4	5
2027	Vacuolar protein sorting-associated protein 4A	IPI00411356	-0.277	0.27840	2	3	2	2
2028	Isoform 2 of Vacuolar protein sorting-associated protein 13A	IPI00478586	0.952	0.04819	0	1	3	2
2029	39S ribosomal protein L15, mitochondrial	IPI00023086	-1.078	0.03586	4	3	2	1
2030	mortality factor 4	IPI00001955	0.253	0.29095	2	3	3	3
2031	cDNA FLJ54775, highly similar to Syntaxin-binding protein 2	IPI00019971	1.445	0.01433	1	1	2	5
2032	Isoform 1 of Probable ATP-dependent RNA helicase DHX36	IPI00027415	0.708	0.08397	3	2	4	4
2033	cDNA FLJ56420, highly similar to Aspartyl aminopeptidase	IPI00015856	1.115	0.03515	3	2	6	4
2034	Isoform 3 of Tyrosine-protein phosphatase non-receptor type 6	IPI00183046	0.765	0.07536	2	2	3	4
2035	Dihydrofolate reductase	IPI00030357	0.708	0.08397	3	2	5	3
2036	High mobility group nucleosome-binding domain-containing protein 5	IPI00006157	0.842	0.06364	1	2	2	4
2037	Dimethyladenosine transferase 1, mitochondrial	IPI00291525	-0.311	0.26205	2	2	2	1
2038	Synapse-associated protein 1	IPI00059242	-0.861	0.06115	5	5	2	4
2039	LDLR chaperone MESD	IPI00399089	0.588	0.12889	1	2	2	3
2040	Translation initiation factor eIF-2B subunit beta	IPI00028083	0.220	0.31215	2	5	3	5
2041	Uncharacterized protein C2orf79	IPI00430803	0.000	0.37049	4	4	4	4
2042	Isoform 3 of THO complex subunit 6 homolog	IPI00301252	0.000	0.37049	2	4	2	4
2043	NADH dehydrogenase [ubiquinone] 1 beta subcomplex subunit 9	IPI00255052	-0.277	0.27840	2	3	2	2
2044	Small nuclear ribonucleoprotein F	IPI00220528	-0.530	0.15514	5	1	3	0
2045	Isoform 1 of Far upstream element-binding protein 3	IPI00377261	0.277	0.27228	3	1	3	2
2046	Translation initiation factor eIF-2B subunit epsilon	IPI00011898	0.588	0.12889	0	2	1	4
2047	Nuclear pore complex protein Nup153	IPI00292059	-1.880	0.00489	5	4	0	1
2048	Isoform 1 of Chromosome-associated kinesin KIF4A	IPI00178150	1.208	0.02377	0	1	4	2
2049	ATP-binding cassette sub-family D member 1	IPI00291373	0.000	0.37049	3	3	3	3
2050	Isoform 3 of Drebrin-like protein	IPI00101968	-0.815	0.06587	5	6	3	4
2051	Choline/ethanolaminephosphotransferase 1	IPI00005775	-0.530	0.15514	3	3	3	1
2052	Isoform 1 of Magnesium-dependent phosphatase 1	IPI00337556	-1.195	0.03093	3	6	2	2
2053	cDNA FLJ61629, highly similar to Clathrin interactor 1	IPI00291930	0.220	0.31215	2	5	5	3
2054	Microsomal glutathione S-transferase 1	IPI00021805	0.000	0.37049	0	2	1	2
2055	Isoform 1 of Huntingtin-interacting protein K	IPI00335001	-0.235	0.30503	4	3	4	2
2056	Isoform 1 of ADP-ribosylation factor-like protein 2-binding protein	IPI00015866	-0.488	0.16467	3	4	3	2
2057	Isoform 1 of Uridine-cytidine kinase 2	IPI00065671	0.000	0.37049	3	1	3	1
2058	V-type proton ATPase subunit G 1	IPI00025285	-0.253	0.29716	4	2	4	1
2059	Isoform 1 of Tumor suppressor p53-binding protein 1	IPI00029778	-0.674	0.10802	2	2	1	1
2060	ADP-ribosylation factor-like protein 8A	IPI00060031	-0.488	0.16467	3	4	4	1
2061	Isoform 3 of HEAT repeat-containing protein 5B	IPI00333696	0.674	0.08856	0	0	3	0
2062	Isoform 1 of Protein fat-free homolog	IPI00001710	-0.588	0.12558	3	2	2	0
2063	Isoform 1 of Caseinolytic peptidase B protein homolog	IPI00006615	0.708	0.08397	3	2	3	5
2064	71 kDa protein	IPI00062599	0.986	0.04066	2	2	5	3
2065	Transcription initiation factor TFIID subunit 2	IPI00328144	0.000	0.37049	0	0	1	0
2066	Rho-associated protein kinase 1	IPI00022542	-0.277	0.27840	2	3	3	1
2067	Protein NipSnap homolog 3A	IPI00004845	-0.455	0.18350	5	3	2	4
2068	Sortilin-related receptor	IPI00022608	0.000	0.37049	3	0	3	1
2069	Isoform 1 of Coiled-coil domain-containing protein 109A	IPI00171573	0.000	0.37049	2	2	2	2
2070	Isoform 1 of Uridine 5'-monophosphate synthase	IPI00003923	0.428	0.19017	4	3	5	4
2071	Isoform 2 of Epimerase family protein SDR39U1	IPI00643286	0.000	0.37049	1	3	3	1
2072	Isoform 1a of Oxysterol-binding protein-related protein 3	IPI00023555	1.299	0.02029	1	2	4	4
2073	Isoform Short of NADPH:adrenodoxin oxidoreductase, mitochondrial	IPI00026958	1.585	0.01126	1	3	6	5
2074	Isoform 1 of Vacuolar protein sorting-associated protein 16 homolog	IPI00305438	-0.952	0.04981	2	3	1	1
2075	Isoform 3 of Mediator of RNA polymerase II transcription subunit 23	IPI00413272	0.362	0.21837	1	1	1	2
2076	cDNA FLJ38069 fis, clone CTONG2015434	IPI00029159	-0.530	0.15514	1	5	2	2
2077	Putative uncharacterized protein	IPI00010402	-0.277	0.27840	3	2	2	2
2078	Cyclin B1	IPI00294696	1.445	0.01433	0	0	4	3
2079	Integrin-linked protein kinase	IPI00013219	0.842	0.06364	1	2	3	3
2080	Transducin beta-like protein 2	IPI00000948	0.311	0.25253	1	2	3	1
2081	cDNA FLJ56840, highly similar to Galactokinase	IPI00019383	-0.842	0.06318	2	4	2	1
2082	Tetratricopeptide repeat protein 35	IPI00014149	-0.455	0.18350	3	5	4	2
2083	Protein tyrosine phosphatase type IVA 1	IPI00020164	-0.663	0.11221	5	4	4	2
2084	cDNA FLJ60607, highly similar to Acyl-protein thioesterase 1	IPI00007321	0.674	0.08856	1	1	1	3
2085	Perilipin-2	IPI00293307	0.000	0.37049	2	1	1	2
2086	Protein QIL1	IPI00329373	-1.299	0.02029	4	4	2	1

No.	Description	Accession number	STN[1]	p-Value[1]	Con_A[2]	Con_B[2]	OXA_A[2]	OXA_B[2]
2087	Ribosome biogenesis regulatory protein homolog	IPI00014253	-1.509	0.01342	3	6	1	2
2088	Small nuclear ribonucleoprotein G	IPI00016572	-0.455	0.18350	5	3	3	3
2089	bifunctional protein NCOAT isoform b	IPI00181391	0.362	0.21837	1	1	0	2
2090	Myosin-IIIa	IPI00185036	0.000	0.37049	3	1	3	1
2091	Golgi-specific brefeldin A-resistance guanine nucleotide exchange factor 1	IPI00021954	0.674	0.08856	1	0	3	0
2092	WD repeat-containing protein 11	IPI00412224	-0.311	0.26205	1	3	2	0
2093	Isoform 7 of Serine/threonine-protein kinase MARK2	IPI00290158	0.842	0.06364	2	1	4	2
2094	Protein FRG1	IPI00004655	0.000	0.37049	2	3	3	2
2095	Isoform 2 of PERQ amino acid-rich with GYF domain-containing protein 2	IPI00647635	-0.765	0.07552	4	3	3	1
2096	UPF0554 protein C2orf43	IPI00030257	-0.674	0.10802	1	3	1	1
2097	Isoform 2 of 1,2-dihydroxy-3-keto-5-methylthiopentene dioxygenase	IPI00470791	1.445	0.01433	1	1	1	6
2098	PCTP-like protein	IPI00186008	-0.530	0.15514	2	4	3	0
2099	Adenylyl cyclase-associated protein	IPI00939159	0.588	0.12889	1	2	1	4
2100	Mediator of RNA polymerase II transcription subunit 12	IPI00004068	0.362	0.21837	1	1	2	1
2101	Alpha-taxilin	IPI00470779	-0.488	0.16467	5	2	2	3
2102	Splicing factor, arginine/serine-rich 11	IPI00464952	-0.588	0.12558	3	2	1	2
2103	Isoform 2 of Actin-related protein 2/3 complex subunit 5	IPI00007280	-0.708	0.08447	4	4	4	1
2104	Protein FAM49A	IPI00006574	0.277	0.27228	1	3	4	1
2105	Isoform 2 of ATPase WRNIP1	IPI00102997	0.674	0.08856	1	1	2	2
2106	DNA ligase 1	IPI00219841	0.842	0.06364	1	2	2	4
2107	Exocyst complex component 4	IPI00059279	0.000	0.37049	4	2	3	3
2108	UPF0556 protein C19orf10	IPI00056357	-0.253	0.29716	3	3	4	1
2109	E3 ubiquitin-protein ligase BRE1A	IPI00251559	0.588	0.12889	1	2	3	2
2110	Charged multivesicular body protein 5	IPI00100796	0.000	0.37049	4	4	5	3
2111	Isoform 2 of Nitrilase homolog 1	IPI00023779	0.000	0.37049	2	2	3	1
2112	Nuclear pore complex protein Nup50	IPI00026940	0.765	0.07536	2	2	3	4
2113	tRNA (guanine-N(7)-)-methyltransferase	IPI00290184	-1.299	0.02029	4	4	1	2
2114	Ribonuclease H2 subunit A	IPI00290192	0.000	0.37049	3	4	4	3
2115	Isoform 1 of Glomulin	IPI00074604	1.078	0.03610	1	2	4	3
2116	Protein FAM50B	IPI00015912	0.488	0.16541	3	2	3	4
2117	Isoform 2 of Fumarylacetoacetate hydrolase domain-containing protein 1	IPI00440828	1.050	0.03689	3	3	6	5
2118	Isoform 2 of Carbohydrate kinase domain-containing protein	IPI00645172	0.000	0.37049	2	2	3	0
2119	Isoform Membrane-bound of Catechol O-methyltransferase	IPI00011284	-0.674	0.10802	2	2	0	1
2120	Exportin-6	IPI00465296	0.674	0.08856	0	0	3	1
2121	Envoplakin	IPI00023711	0.674	0.08856	1	1	3	0
2122	Alpha-galactosidase A	IPI00025869	1.208	0.02377	1	1	4	2
2123	Isoform 1 of Replication factor C subunit 1	IPI00375358	-0.530	0.15514	3	3	3	0
2124	Plakophilin-3	IPI00026952	-1.945	0.00406	6	7	0	3
2125	Isoform 1 of Retinol dehydrogenase 11	IPI00339384	-0.220	0.31447	5	3	3	4
2126	Telomeric repeat-binding factor 2-interacting protein 1	IPI00008961	-0.488	0.16467	3	4	2	3
2127	Desmoglein-2	IPI00028931	1.115	0.03515	3	2	4	6
2128	Sentrin-specific protease 3	IPI00171525	-0.986	0.04534	4	4	2	2
2129	U3 small nucleolar ribonucleoprotein protein IMP3	IPI00019488	-2.274	0.00178	6	5	1	1
2130	Probable asparaginyl-tRNA synthetase, mitochondrial	IPI00101664	-0.235	0.30503	4	3	3	3
2131	Isoform DFF45 of DNA fragmentation factor subunit alpha (Fragment)	IPI00010882	-1.115	0.03482	4	6	2	3
2132	Succinate dehydrogenase cytochrome b560 subunit, mitochondrial	IPI00016968	0.000	0.37049	2	0	2	1
2133	Isoform 2 of DnaJ homolog subfamily C member 2	IPI00455199	-0.277	0.27840	2	3	3	1
2134	Isoform 1 of RNA-binding Raly-like protein	IPI00166137	-0.842	0.06318	4	2	2	1
2135	Isoform 1 of Protein POF1B	IPI00103242	-0.916	0.05184	5	4	3	2
2136	Isoform 1 of Testin	IPI00024097	-0.986	0.04534	4	4	2	2
2137	MACRO domain-containing protein 1	IPI00155601	-1.115	0.03482	5	5	3	2
2138	Translational activator of cytochrome c oxidase 1	IPI00019903	-0.488	0.16467	5	2	3	2
2139	importin subunit alpha-6	IPI00413214	0.311	0.25253	1	2	3	1
2140	Isoform SRP55-1 of Splicing factor, arginine/serine-rich 6	IPI00012345	-0.311	0.26205	1	3	0	2
2141	Myosin-Ia	IPI00294386	0.530	0.13800	2	2	4	2
2142	Isoform 1 of CLIP-associating protein 1	IPI00396279	-0.765	0.07552	4	3	2	2
2143	Calcium homeostasis endoplasmic reticulum protein	IPI00333010	0.000	0.37049	2	2	2	2
2144	serine/threonine-protein phosphatase PP1-alpha catalytic subunit isoform 3	IPI00027423	-0.428	0.18789	5	4	4	3
2145	UDP-glucose 4-epimerase	IPI00553131	-0.674	0.10802	1	3	1	1
2146	Prefoldin subunit 6	IPI00005657	0.362	0.21837	1	1	2	0
2147	39S ribosomal protein L11, mitochondrial	IPI00007001	0.000	0.37049	1	1	1	0
2148	RcDNAJ9 (Fragment)	IPI00014718	0.277	0.27228	2	2	3	2
2149	Isoform 1 of Ubiquitin carboxyl-terminal hydrolase 15	IPI00000728	0.000	0.37049	2	1	2	0
2150	Isoform 1 of 28S ribosomal protein S11, mitochondrial	IPI00010244	0.000	0.37049	3	3	4	2
2151	28S ribosomal protein S36, mitochondrial	IPI00020495	-0.277	0.27840	2	3	2	2
2152	Isoform 1 of Rho guanine nucleotide exchange factor 2	IPI00291316	-0.277	0.27840	3	2	3	1
2153	RNA methyltransferase-like protein 1	IPI00335589	0.311	0.25253	2	1	2	2
2154	REST corepressor 1	IPI00008531	0.455	0.17237	3	3	5	3
2155	Isoform 2 of Mitochondrial intermembrane space import and assembly protein 40	IPI00177428	-0.253	0.29716	3	3	2	3
2156	Serine/threonine-protein phosphatase 5	IPI00019812	0.708	0.08397	3	2	3	5
2157	D-dopachrome decarboxylase	IPI00293867	0.000	0.37049	3	2	3	2
2158	Beta-adrenergic receptor kinase 1	IPI00012497	0.000	0.37049	1	1	1	0
2159	Nucleolar complex protein 4 homolog	IPI00031661	-2.081	0.00323	5	5	1	0
2160	Methylmalonyl-CoA mutase, mitochondrial	IPI00024934	1.299	0.02029	0	2	4	4
2161	Isoform 1 of E3 ubiquitin-protein ligase BRE1B	IPI00162563	1.078	0.03610	2	1	4	3
2162	ATP-dependent RNA helicase DDX24	IPI00006987	-0.674	0.10802	2	2	1	0
2163	1-acyl-sn-glycerol-3-phosphate acyltransferase epsilon	IPI00028491	0.000	0.37049	2	2	3	1
2164	Pyridoxal phosphate phosphatase	IPI00025340	-0.765	0.07552	4	3	3	1
2165	UPF0600 protein C5orf51	IPI00374272	-0.708	0.08447	3	5	2	3
2166	Transmembrane 9 superfamily member 4	IPI00021985	0.000	0.37049	1	2	2	0
2167	GTP-binding protein Rheb	IPI00016669	0.488	0.16541	2	3	4	3
2168	28S ribosomal protein S18a, mitochondrial	IPI00018691	-0.588	0.12558	2	3	2	1
2169	Isoform 1 of Proteasome assembly chaperone 1	IPI00030770	-0.311	0.26205	3	1	2	0
2170	Isoform 1 of Zinc finger MYM-type protein 3	IPI00029484	0.588	0.12889	2	0	2	3
2171	Isoform 1 of Hematological and neurological expressed 1 protein	IPI00007764	-0.455	0.18350	4	4	3	3
2172	Isoform Short of Probable global transcription activator SNF2L2	IPI00386718	-0.588	0.12558	2	3	1	2
2173	Isoform 2 of Peptidyl-prolyl cis-trans isomerase-like 3	IPI00032473	0.674	0.08856	1	1	3	1
2174	DnaJ homolog subfamily C member 3	IPI00006713	0.277	0.27228	2	2	2	3
2175	Isoform 1 of Protein dopey-2	IPI00294653	0.000	0.37049	1	1	1	0
2176	Isoform 1 of Polyadenylate-binding protein-interacting protein 1	IPI00021466	-1.299	0.02029	4	4	2	1
2177	Similar to Keratin, type II cytoskeletal 8	IPI00787323	-1.208	0.03035	2	4	1	1
2178	Mitochondrial import inner membrane translocase subunit Tim16	IPI00218463	-0.588	0.12558	3	2	2	1
2179	Isoform 2 of Chromodomain-helicase-DNA-binding protein 2	IPI00023109	0.000	0.37049	1	3	3	0
2180	Ribosomal protein L1	IPI00035167	-0.235	0.30503	3	4	2	4
2181	Isoform 1 of Regulator of nonsense transcripts 2	IPI00300504	0.674	0.08856	1	1	3	0

No.	Description	Accession number	STN[1]	p-Value[1]	Con_A[2]	Con_B[2]	OXA_A[2]	OXA_B[2]
2182	Ribonuclease P protein subunit p38	IPI00019195	0.000	0.37049	1	3	3	1
2183	ubiquitin-like protein fubi and ribosomal protein S30 precursor	IPI00019770	0.000	0.37049	3	1	3	0
2184	Brefeldin A-inhibited guanine nucleotide-exchange protein 1	IPI00002188	0.000	0.37049	1	2	2	0
2185	Aldo-keto reductase family 1 member C2	IPI00005668	-0.311	0.26205	2	2	2	0
2186	Isoform 2 of Choline-phosphate cytidylyltransferase B	IPI00001562	-0.530	0.15514	3	3	2	2
2187	Uncharacterized protein C3orf26	IPI00031679	-0.842	0.06318	3	3	2	1
2188	Protoporphyrinogen oxidase	IPI00031357	0.000	0.37049	0	2	2	1
2189	14 kDa phosphohistidine phosphatase	IPI00299977	-0.842	0.06318	2	4	1	2
2190	Mitochondrial import inner membrane translocase subunit Tim13	IPI00001589	0.000	0.37049	4	1	4	1
2191	General transcription factor IIF subunit 2	IPI00477686	-0.530	0.15514	3	3	2	2
2192	Importin subunit alpha-3	IPI00299033	0.000	0.37049	2	3	3	2
2193	Isoform 1 of GTP-binding protein 10	IPI00167638	-0.674	0.10802	1	3	0	1
2194	Phosphomevalonate kinase	IPI00220648	0.000	0.37049	2	2	3	1
2195	28S ribosomal protein S7, mitochondrial	IPI00006440	0.000	0.37049	2	1	2	1
2196	GTP cyclohydrolase 1 feedback regulatory protein	IPI00217253	0.000	0.37049	2	2	3	1
2197	Signal peptidase complex subunit 3	IPI00300299	0.588	0.12889	1	2	3	2
2198	N-alpha-acetyltransferase 20, NatB catalytic subunit	IPI00007174	-0.277	0.27840	3	2	2	2
2199	Isoform 2 of NudC domain-containing protein 1	IPI00306398	0.000	0.37049	0	0	1	0
2200	NADH dehydrogenase [ubiquinone] iron-sulfur protein 4, mitochondrial	IPI00011217	0.000	0.37049	2	1	1	2
2201	Profilin	IPI00107555	0.765	0.07536	3	1	3	4
2202	Histone acetyltransferase MYST2	IPI00180764	1.078	0.03610	2	1	3	4
2203	NADH dehydrogenase [ubiquinone] 1 beta subcomplex subunit 5, mitochondrial	IPI00013459	-0.842	0.06318	3	3	2	1
2204	Isoform 1 of Metaxin-1	IPI00013678	0.000	0.37049	1	1	1	1
2205	Isoform 1 of TP53RK-binding protein	IPI00301432	0.277	0.27228	3	1	4	0
2206	Putative uncharacterized protein DOCK6	IPI00184772	-0.362	0.22785	1	2	1	0
2207	Isoform 1 of Anaphase-promoting complex subunit 7	IPI00008248	0.952	0.04819	1	0	4	1
2208	NADH dehydrogenase [ubiquinone] flavoprotein 2, mitochondrial	IPI00291328	-0.588	0.12558	3	2	2	0
2209	Isoform 1 of Ubiquitin carboxyl-terminal hydrolase 34	IPI00297593	0.000	0.37049	1	0	0	0
2210	Isoform 1 of Wings apart-like protein homolog	IPI00375330	-0.952	0.04981	3	2	0	0
2211	Vasodilator-stimulated phosphoprotein	IPI00301058	-0.455	0.18350	4	4	2	4
2212	Isoform 1 of Zinc finger protein 207	IPI00013457	0.000	0.37049	3	2	2	3
2213	General transcription factor IIF subunit 1	IPI00017450	0.235	0.30171	3	3	4	3
2214	Isoform 3 of Keratin, type II cytoskeletal 80	IPI00375843	1.445	0.01433	1	1	4	3
2215	Transmembrane emp24 domain-containing protein 7	IPI00032825	-0.765	0.07552	3	4	1	3
2216	Ras-related protein Rap-2c	IPI00009607	0.362	0.21837	1	1	2	0
2217	Probable ergosterol biosynthetic protein 28	IPI00007730	-0.530	0.15514	4	2	3	1
2218	Ribonuclease inhibitor	IPI00550069	-0.765	0.07552	3	4	2	2
2219	SAFB-like transcription modulator isoform b	IPI00019996	0.362	0.21837	0	0	2	1
2220	Sorting and assembly machinery component 50 homolog	IPI00412713	-0.530	0.15514	4	2	3	1
2221	Golgi resident protein GCP60	IPI00009315	0.488	0.16541	3	2	3	4
2222	Isoform B of Arfaptin-1	IPI00021258	-0.253	0.29716	3	3	4	1
2223	Isoform 6 of E3 ubiquitin-protein ligase UBR4	IPI00386907	0.674	0.08856	1	0	2	2
2224	UV excision repair protein RAD23 homolog A	IPI00008219	0.000	0.37049	2	2	2	2
2225	Ras-related protein Ral-A	IPI00217519	-0.235	0.30503	3	4	3	3
2226	Ubiquitin carboxyl-terminal hydrolase 11	IPI00184533	0.952	0.04819	1	1	4	0
2227	NIF3L1 isoform gamma	IPI00451429	-1.208	0.03035	3	3	1	0
2228	Ras-related protein Rab-8A	IPI00028481	0.000	0.37049	2	1	2	1
2229	Cell division protein kinase 3	IPI00023503	0.277	0.27228	2	2	2	3
2230	Hexokinase-3	IPI00005118	-0.277	0.27840	1	4	1	3
2231	Methylosome protein 50	IPI00012202	-0.311	0.26205	2	2	1	2
2232	RNA 3'-terminal phosphate cyclase-like protein	IPI00294229	-0.765	0.07552	3	4	2	2
2233	Isoform SNAP-23a of Synaptosomal-associated protein 23	IPI00010438	0.220	0.31215	4	3	4	4
2234	Tyrosine-protein kinase CSK	IPI00013212	-0.277	0.27840	3	2	3	0
2235	1-phosphatidylinositol-4,5-bisphosphate phosphodiesterase beta-3	IPI00010400	0.311	0.25253	1	2	2	2
2236	Sulfhydryl oxidase 2	IPI00376394	-0.842	0.06318	3	3	2	0
2237	Peptidyl-prolyl cis-trans isomerase FKBP9	IPI00182126	-0.952	0.04981	3	2	0	0
2238	Serpin B9	IPI00032139	0.488	0.16541	3	2	4	3
2239	DNA polymerase delta subunit 2	IPI00025616	0.708	0.08397	2	3	3	5
2240	cDNA FLJ56370, highly similar to Homo sapiens FK506 binding protein 8, 38kDa	IPI00328161	0.765	0.07536	2	2	3	4
2241	peroxisomal 3,2-trans-enoyl-CoA isomerase isoform 1	IPI00419263	-0.765	0.07552	3	4	2	2
2242	Acylphosphatase-1	IPI00221117	0.000	0.37049	4	2	4	2
2243	Ras-related protein Rab-9A	IPI00016372	-0.277	0.27840	3	2	3	1
2244	Isoform 5 of Sigma non-opioid intracellular receptor 1	IPI00167206	0.311	0.25253	1	2	1	3
2245	Isoform 3 of Sorting nexin-3	IPI00029740	0.253	0.29095	2	3	3	3
2246	Cytochrome c oxidase subunit 6C	IPI00015972	-0.952	0.04981	2	3	1	1
2247	Isoform 1 of Set1/Ash2 histone methyltransferase complex subunit ASH2	IPI00328658	0.000	0.37049	1	2	2	1
2248	CCR4-NOT transcription complex subunit 7	IPI00006552	-0.588	0.12558	2	3	2	1
2249	Isoform 1 of UPF0598 protein C8orf82	IPI00166638	0.000	0.37049	4	2	3	3
2250	Isoform 1 of Uncharacterized methyltransferase WBSCR22	IPI00013810	-0.530	0.15514	3	3	3	0
2251	Isoform 1 of Serine/threonine-protein kinase 4	IPI00011488	0.253	0.29095	3	2	2	4
2252	Isoform 1 of ARF GTPase-activating protein GIT1	IPI00384861	-0.311	0.26205	1	3	0	2
2253	Isoform 1 of Transcription elongation factor A protein 1	IPI00333215	-0.952	0.04981	2	3	0	1
2254	Isoform 1 of Gamma-tubulin complex component 3	IPI00033516	0.000	0.37049	2	0	1	2
2255	D-beta-hydroxybutyrate dehydrogenase, mitochondrial	IPI00025341	-0.588	0.12558	3	2	2	0
2256	Isoform 1 of NHL repeat-containing protein 2	IPI00301051	1.208	0.02377	1	1	2	4
2257	Isoform 2 of Triple functional domain protein	IPI00479523	0.000	0.37049	1	0	1	0
2258	Isoform Long of Acidic fibroblast growth factor intracellular-binding protein	IPI00012443	-1.445	0.01457	4	3	1	1
2259	Isoform 2 of Basic leucine zipper and W2 domain-containing protein 1	IPI00180128	0.311	0.25253	2	0	3	0
2260	Isoform 1 of Heterochromatin protein 1-binding protein 3	IPI00642238	1.208	0.02377	0	0	3	3
2261	Isoform 1 of Melanoma inhibitory activity protein 3	IPI00455473	-0.362	0.22785	1	2	0	0
2262	Transmembrane protein 109	IPI00031697	-1.195	0.03093	5	4	3	1
2263	Isoform 1 of Hematological and neurological expressed 1-like protein	IPI00027397	-0.986	0.04534	4	4	2	2
2264	Phosphatidylinositide phosphatase SAC1	IPI00022275	-0.488	0.16467	4	3	3	2
2265	Isoform 1 of Translation initiation factor eIF-2B subunit gamma	IPI00006504	0.362	0.21837	1	1	1	2
2266	Cleavage and polyadenylation specificity factor subunit 2	IPI00419531	0.488	0.16541	2	3	3	4
2267	cDNA FLJ31776 fis, clone NT2RI2008141, highly similar to CALUMENIN	IPI00789155	0.952	0.04819	0	0	3	2
2268	cDNA FLJ56394, highly similar to N-acetylglucosamine kinase	IPI00296526	-0.674	0.10802	2	2	1	1
2269	Nucleolar protein 16	IPI00032849	-2.274	0.00178	5	6	1	1
2270	NADH dehydrogenase [ubiquinone] iron-sulfur protein 7, mitochondrial	IPI00307749	-0.253	0.29716	4	2	4	1
2271	cDNA FLJ56468, highly similar to Kynurenine--oxoglutarate transaminase 1	IPI00002523	-0.674	0.10802	2	2	1	1
2272	Reticulocalbin-2	IPI00029628	-1.195	0.03093	5	4	2	2
2273	Nucleoporin Nup37	IPI00171665	0.253	0.29095	2	3	4	2
2274	NADH dehydrogenase [ubiquinone] 1 alpha subcomplex assembly factor 3	IPI00399053	-1.668	0.01068	5	3	1	1
2275	NEDD8-activating enzyme E1 catalytic subunit	IPI00328154	0.588	0.12889	2	1	2	3
2276	Syntaxin-4	IPI00029730	-0.362	0.22785	2	1	1	1

No.	Description	Accession number	STN[1]	p-Value[1]	Con_A[2]	Con_B[2]	OXA_A[2]	OXA_B[2]
2277	Similar to Ankyrin repeat and FYVE domain-containing protein 1	IPI00159899	0.362	0.21837	0	1	1	2
2278	Coiled-coil domain-containing protein 58	IPI00046828	0.277	0.27228	2	2	3	2
2279	cDNA FLJ56152, highly similar to Rho guanine nucleotide exchange factor 7	IPI00449906	0.000	0.37049	1	2	2	1
2280	cDNA FLJ78567	IPI00043678	0.000	0.37049	1	1	1	0
2281	cDNA FLJ45232 fis, clone BRCAN2021718	IPI00170877	-0.952	0.04981	2	3	1	1
2282	RNA-binding protein 28	IPI00304187	-0.311	0.26205	2	2	1	2
2283	Isoform 1 of Regulator of nonsense transcripts 3B	IPI00023409	0.362	0.21837	1	1	2	1
2284	Nucleoside-triphosphatase C1orf57	IPI00031570	0.000	0.37049	2	2	2	2
2285	H/ACA ribonucleoprotein complex subunit 2	IPI00041325	-0.588	0.12558	2	3	2	0
2286	Isoform 3 of Nuclear transcription factor Y subunit gamma	IPI00071697	-0.277	0.27840	3	2	2	2
2287	Huntingtin-interacting protein 1	IPI00782965	0.708	0.08397	3	2	4	4
2288	Isoform 1 of Rab3 GTPase-activating protein catalytic subunit	IPI00014235	0.674	0.08856	1	1	3	1
2289	YrdC domain-containing protein, mitochondrial	IPI00384180	-0.488	0.16467	4	3	2	3
2290	Bifunctional 3'-phosphoadenosine 5'-phosphosulfate synthase 1	IPI00011619	0.674	0.08856	1	1	3	1
2291	cDNA FLJ60939, highly similar to NAD-dependent deacetylase sirtuin-3, mitochondrial	IPI00183171	0.000	0.37049	2	1	2	0
2292	Isocitrate dehydrogenase [NAD] subunit gamma, mitochondrial	IPI00220150	-0.277	0.27840	1	4	3	1
2293	Cytochrome c oxidase assembly protein COX11, mitochondrial	IPI00295394	-0.277	0.27840	3	2	3	0
2294	Tryptophanyl-tRNA synthetase, mitochondrial	IPI00025050	-1.208	0.03035	4	2	1	1
2295	Protein S100-A16	IPI00062120	0.000	0.37049	3	2	3	2
2296	cDNA FLJ30398 fis, clone BRACE2008402, highly similar to Steroid receptor RNA activator 1	IPI00102313	-0.488	0.16467	4	3	2	3
2297	Isoform 1 of 28S ribosomal protein S5, mitochondrial	IPI00169400	-0.842	0.06318	5	1	2	1
2298	NADH dehydrogenase [ubiquinone] 1 alpha subcomplex assembly factor 4	IPI00023064	0.277	0.27228	2	2	2	3
2299	Nucleoporin Nup43	IPI00742943	-0.588	0.12558	3	2	2	1
2300	C-Myc-binding protein	IPI00871174	-0.311	0.26205	2	2	2	1
2301	Isoform 1 of RNA 3'-terminal phosphate cyclase	IPI00011726	0.000	0.37049	2	3	2	3
2302	cDNA FLJ54710, highly similar to Target of Myb protein 1	IPI00023191	-1.078	0.03586	3	4	2	1
2303	Isoform 1 of H/ACA ribonucleoprotein complex subunit 1	IPI00302176	0.530	0.13800	2	2	3	3
2304	programmed cell death 4 isoform 2	IPI00240675	0.000	0.37049	2	2	2	2
2305	Isoform 1 of Mannose-6-phosphate isomerase	IPI00219358	-0.277	0.27840	1	4	1	3
2306	Guanine nucleotide-binding protein G(i) subunit alpha-1	IPI00337415	-0.952	0.04981	3	2	1	0
2307	RWD domain-containing protein 1	IPI00034010	-0.588	0.12558	3	2	2	1
2308	Casein kinase II subunit beta	IPI00010865	0.277	0.27228	2	2	3	2
2309	Isoform 1 of Phosphoribosyl pyrophosphate synthase-associated protein 1	IPI00291578	-0.530	0.15514	2	4	3	0
2310	Isoform 1 of Nucleolar protein 3	IPI00105916	0.277	0.27228	1	3	2	3
2311	Isoform 1 of Cullin-3	IPI00014312	0.842	0.06364	0	2	2	4
2312	2-oxoisovalerate dehydrogenase subunit alpha, mitochondrial	IPI00025100	-0.588	0.12552	2	3	0	2
2313	Survival of motor neuron-related-splicing factor 30	IPI00025176	-0.765	0.07552	3	4	3	1
2314	V-type proton ATPase 16 kDa proteolipid subunit	IPI00018855	0.362	0.21837	0	1	2	1
2315	Isoform SMN of Survival motor neuron protein	IPI00003394	-1.668	0.01068	6	2	1	1
2316	Bystin	IPI00328987	-0.952	0.04981	2	3	0	1
2317	Isoform 1 of Lysine-specific demethylase 3B	IPI00298935	0.362	0.21837	0	0	0	2
2318	cDNA FLJ56469, highly similar to Propionyl-CoA carboxylase alpha chain, mitochondrial	IPI00552419	0.311	0.25253	2	0	1	3
2319	28S ribosomal protein S16, mitochondrial	IPI00032872	-0.588	0.12558	0	4	0	2
2320	cDNA FLJ61658, highly similar to Transmembrane 9 superfamily protein member 1	IPI00101374	-0.311	0.26205	3	1	1	2
2321	Isoform 1 of Interferon regulatory factor 2-binding protein 2	IPI00376199	-0.253	0.29716	3	3	2	3
2322	Isoform 1 of Protein kinase C and casein kinase substrate in neurons protein 2	IPI00027009	-0.588	0.12558	3	2	2	0
2323	Phosphatidylinositol-5-phosphate 4-kinase type-2 gamma	IPI00152303	0.674	0.08856	1	0	2	2
2324	CDGSH iron sulfur domain-containing protein 2	IPI00166865	-0.842	0.06318	4	2	2	1
2325	Isoform 1 of Kinesin-like protein KIF2A	IPI00010368	0.530	0.13800	2	2	4	2
2326	Nucleolysin TIAR	IPI00005615	-0.588	0.12558	2	3	2	0
2327	Probable ATP-dependent RNA helicase DDX10	IPI00297900	-0.952	0.04981	3	2	1	1
2328	Isoform 3 of Pre-mRNA 3'-end-processing factor FIP1	IPI00008449	-1.208	0.03035	2	4	1	1
2329	Isoform 1 of Heterogeneous nuclear ribonucleoprotein L-like	IPI00103247	0.000	0.37049	1	2	2	1
2330	Uncharacterized protein C19orf43	IPI00031526	-0.588	0.12558	3	2	1	2
2331	Dihydroxyacetone phosphate acyltransferase	IPI00005677	0.530	0.13800	2	2	3	3
2332	Synaptojanin-2-binding protein	IPI00299193	0.000	0.37049	2	3	4	1
2333	Isoform 2 of UPF0465 protein C5orf33	IPI00431405	-0.277	0.27840	2	3	2	2
2334	Prolactin regulatory element-binding protein	IPI00033349	0.311	0.25253	2	1	2	2
2335	Farnesyl pyrophosphate synthetase like-4 protein (Fragment)	IPI00382869	-0.674	0.10802	3	1	1	0
2336	Ras-related protein Rap-2b	IPI00018364	0.311	0.25253	2	1	3	0
2337	Ras-related protein Rab-1B	IPI00008964	0.000	0.37049	4	1	3	2
2338	Nucleoporin NUP53	IPI00329650	-0.311	0.26205	2	2	2	1
2339	Claudin-1	IPI00000691	-1.078	0.03586	4	3	2	1
2340	Pumilio domain-containing protein C14orf21	IPI00216999	-0.674	0.10802	2	2	0	1
2341	Alcohol dehydrogenase class-3	IPI00746777	0.000	0.37049	1	2	2	1
2342	Elongation factor G 2, mitochondrial precursor	IPI00071703	0.362	0.21837	0	1	2	1
2343	119 kDa protein	IPI00297178	-0.530	0.15514	4	2	1	3
2344	Glutamate--cysteine ligase regulatory subunit	IPI00010090	0.588	0.12889	1	2	3	2
2345	General transcription factor IIH subunit 1	IPI00030380	-0.362	0.22785	1	2	1	1
2346	Ras-related protein Rab-21	IPI00007755	1.078	0.03610	1	2	3	4
2347	Vacuolar protein sorting-associated protein 26B	IPI00059264	0.277	0.27228	2	2	3	2
2348	Isoform 2 of WASH complex subunit 7	IPI00164930	-0.952	0.04981	3	2	1	1
2349	Acetyl-coenzyme A synthetase, cytoplasmic	IPI00413730	0.277	0.27228	2	2	3	2
2350	Transient receptor potential cation channel subfamily V member 2	IPI00183666	-0.362	0.22785	0	2	0	1
2351	Isoform 1 of Dynamin-1	IPI00413140	0.000	0.37049	1	3	1	3
2352	Isoform 1 of SWI/SNF-related matrix-associated actin-dependent regulator of chromatin	IPI00017669	0.674	0.08856	1	1	1	3
2353	Isoform 2 of Leucine-rich repeat flightless-interacting protein 1	IPI00006207	-0.311	0.26205	2	2	1	2
2354	Isoform 1 of Neurochondrin	IPI00549543	0.530	0.13800	2	2	3	3
2355	Hydroxymethylglutaryl-CoA lyase, mitochondrial	IPI00293564	-0.842	0.06318	3	3	1	2
2356	Isoform I of Septin-6	IPI00216139	0.253	0.29095	2	3	2	4
2357	Isoform 1 of Ubiquitin-conjugating enzyme E2 Z	IPI00011996	-1.509	0.01342	6	3	2	0
2358	NADP-dependent malic enzyme	IPI00008215	0.000	0.37049	3	1	2	2
2359	Isoform 1 of Serine/threonine-protein kinase N1	IPI00002803	1.208	0.02377	0	1	4	2
2360	V-type proton ATPase subunit d 1	IPI00034159	0.952	0.04819	1	0	2	3
2361	Mitochondrial 18 kDa protein	IPI00784376	-0.311	0.26205	3	1	2	0
2362	Isoform 6 of GTPase-activating protein and VPS9 domain-containing protein 1	IPI00292753	0.000	0.37049	2	2	2	2
2363	RAC-alpha serine/threonine-protein kinase	IPI00012866	0.362	0.21837	0	0	2	1
2364	telomerase-binding protein EST1A isoform 2	IPI00014252	-0.952	0.04981	0	4	1	1
2365	Isoform 3 of Protein VPRBP	IPI00181396	-0.674	0.10802	2	2	1	0
2366	Isoform 1 of NADH dehydrogenase [ubiquinone] flavoprotein 1, mitochondrial	IPI00028520	0.842	0.06364	1	2	3	3
2367	Isoform 1 of Ubiquitin-protein ligase E3C	IPI00604464	1.208	0.02377	1	0	3	3
2368	Isoform 1 of Elongation factor Tu GTP-binding domain-containing protein 1	IPI00293026	1.208	0.02377	1	0	2	4
2369	Ribonucleases P/MRP protein subunit POP1	IPI00293331	0.362	0.21837	0	1	2	1
2370	TFIIH basal transcription factor complex helicase subunit	IPI00029728	0.362	0.21837	0	1	0	2
2371	Filaggrin	IPI00026256	0.000	0.37049	0	0	0	0

No.	Description	Accession number	STN[1]	p-Value[1]	Con_A[2]	Con_B[2]	OXA_A[2]	OXA_B[2]
2372	39S ribosomal protein L14, mitochondrial	IPI00418290	-0.277	0.27840	2	3	3	1
2373	Ubiquitin-conjugating enzyme E2 E1	IPI00021346	0.588	0.12889	2	1	3	2
2374	Isoform 1 of Peroxisomal membrane protein PEX14	IPI00025346	0.588	0.12889	0	2	2	3
2375	Putative uncharacterized protein DKFZp686C1054	IPI00465054	-0.311	0.26205	3	1	1	2
2376	NHP2-like protein 1	IPI00026167	-0.488	0.16467	4	3	2	3
2377	Isoform 1 of RNA-binding protein Raly	IPI00216044	-0.588	0.12558	2	3	2	1
2378	Isoform 2 of Syntaxin-5	IPI00386786	-0.952	0.04981	3	2	1	0
2379	Isoform 1 of Pogo transposable element with ZNF domain	IPI00410717	-1.445	0.01457	5	2	1	1
2380	Ketosamine-3-kinase	IPI00099986	-0.253	0.29716	3	3	2	3
2381	cDNA FLJ12662 fis, clone NT2RM4002205, moderately similar to ELONGATION FACTOR G	IPI00026321	-0.362	0.22785	1	2	1	1
2382	erlin-1	IPI00007940	-0.253	0.29716	4	2	3	2
2383	PDZ domain-containing protein GIPC1	IPI00024705	0.277	0.27228	3	1	2	3
2384	Isoform 1 of Tumor protein D52	IPI00619958	-0.277	0.27840	3	2	2	2
2385	Isoform 1 of Protein IWS1 homolog	IPI00296432	-0.311	0.26205	1	3	1	2
2386	Isoform 1 of Protein disulfide-isomerase TMX3	IPI00064193	-0.588	0.12558	1	4	1	2
2387	cDNA FLJ56157, highly similar to Glucosylceramidase	IPI00021807	0.000	0.37049	0	2	2	1
2388	Isoform 1 of Dephospho-CoA kinase domain-containing protein	IPI00291417	-0.235	0.30503	3	4	3	3
2389	Isoform 2 of Syntaxin-binding protein 1	IPI00046057	0.362	0.21837	1	0	2	1
2390	Isoform 1 of UPF0557 protein C10orf119	IPI00478758	0.842	0.06364	0	2	4	2
2391	Isoform 1 of Alpha-adducin	IPI00019901	-0.530	0.15514	4	2	2	2
2392	Isoform 1 of Pre-mRNA-splicing factor RBM22	IPI00019046	-0.311	0.26205	2	2	2	1
2393	Ribosomal RNA-processing protein 8	IPI00304932	-0.311	0.26205	3	1	1	2
2394	Isoform 2A of GTPase KRas	IPI00423568	0.000	0.37049	3	2	2	3
2395	Isoform 1 of WD repeat-containing protein 74	IPI00018192	-0.588	0.12558	2	3	2	1
2396	WD repeat-containing protein 43	IPI00937477	-1.208	0.03035	2	4	1	1
2397	Isoform 1 of Protein tweety homolog 3	IPI00749429	-0.952	0.04981	1	4	0	1
2398	Isoform 2 of Plakophilin-2	IPI00005264	-0.253	0.29716	2	4	2	3
2399	Cysteine and glycine-rich protein 2	IPI00002824	-0.530	0.15514	3	3	2	2
2400	Isoform 1 of Transmembrane protein 111	IPI00020472	0.000	0.37049	3	2	3	2
2401	Calcium-binding protein p22	IPI00218924	0.000	0.37049	2	2	2	2
2402	Isoform 1 of Proteasome subunit beta type-8	IPI00000783	0.277	0.27228	3	1	2	3
2403	cDNA FLJ55034	IPI00384122	0.000	0.37049	2	3	2	3
2404	39S ribosomal protein L9, mitochondrial	IPI00307409	-0.842	0.06318	2	4	2	1
2405	Peptidase M20 domain-containing protein 2	IPI00217852	0.674	0.08856	0	1	2	2
2406	Isoform 1 of Ubiquinone biosynthesis protein COQ9, mitochondrial	IPI00470631	-0.362	0.22785	2	1	1	0
2407	Isoform AGX2 of UDP-N-acetylhexosamine pyrophosphorylase	IPI00000684	0.362	0.21837	1	1	2	1
2408	Coiled-coil domain-containing protein 124	IPI00060627	0.000	0.37049	1	2	2	1
2409	Isoform 2 of Isopentenyl-diphosphate Delta-isomerase 1	IPI00220014	0.277	0.27228	2	2	2	3
2410	Lanosterol synthase	IPI00009747	-0.588	0.12558	4	1	2	0
2411	39S ribosomal protein L16, mitochondrial	IPI00000821	-0.952	0.04981	3	2	0	1
2412	La-related protein 7	IPI00294742	-0.311	0.26205	2	2	1	2
2413	Pyrroline-5-carboxylate reductase	IPI00550882	-0.952	0.04981	3	2	0	0
2414	Isoform 1 of CUB domain-containing protein 1	IPI00290039	0.588	0.12889	0	2	3	2
2415	DNA-directed RNA polymerase, mitochondrial precursor	IPI00298738	0.000	0.37049	1	2	1	2
2416	Isoform 1 of Rho guanine nucleotide exchange factor 12	IPI00022164	-0.311	0.26205	1	3	2	0
2417	Pumilio domain-containing protein KIAA0020	IPI00791325	-1.668	0.01068	3	5	0	0
2418	Isoform 1 of Neuron navigator 3	IPI00217051	0.000	0.37049	0	0	1	1
2419	cDNA FLJ55484, highly similar to ATP-dependent RNA helicase DDX39	IPI00644431	0.277	0.27228	1	3	2	3
2420	Seryl-tRNA synthetase, mitochondrial	IPI00328361	0.000	0.37049	2	3	3	2
2421	progesterone receptor membrane component 2	IPI00005202	-0.530	0.15514	3	3	3	1
2422	Gamma-soluble NSF attachment protein	IPI00293817	-0.253	0.29716	3	3	4	1
2423	Putative uncharacterized protein NAPRT1	IPI00412498	-0.588	0.12558	1	4	2	1
2424	Isoform 1 of YTH domain family protein 1	IPI00221345	-0.952	0.04981	3	2	1	1
2425	Derlin-1	IPI00013271	0.000	0.37049	0	2	2	1
2426	Isoform p26 of 7,8-dihydro-8-oxoguanine triphosphatase	IPI00004392	0.000	0.37049	2	1	2	1
2427	Transcriptional repressor p66-beta	IPI00103554	0.362	0.21837	1	1	2	1
2428	Isoform 1 of Muscleblind-like protein 1	IPI00021692	-0.311	0.26205	2	2	1	2
2429	Isoform 1 of ATP-binding cassette sub-family F member 3	IPI00465160	0.000	0.37049	1	2	2	1
2430	Zinc finger protein 622	IPI00056499	-0.765	0.07552	3	4	2	2
2431	ATP-dependent RNA helicase DDX51	IPI00217541	-0.311	0.26205	0	3	1	2
2432	Isoform 1 of Malonyl-CoA-acyl carrier protein transacylase, mitochondrial	IPI00023359	-0.277	0.27840	2	3	1	3
2433	Serine palmitoyltransferase 1	IPI00005745	-0.362	0.22785	1	2	1	1
2434	Isoform 1 of GPI transamidase component PIG-T	IPI00100030	0.311	0.25253	2	1	1	3
2435	Isoform 1 of Cytosolic non-specific dipeptidase	IPI00177728	0.000	0.37049	2	2	2	2
2436	Hippocalcin-like protein 1	IPI00219344	-0.588	0.12558	2	3	2	1
2437	Splicing factor 45	IPI00176706	-0.277	0.27840	3	2	2	2
2438	Isoform 1 of Epidermal growth factor receptor kinase substrate 8-like protein 3	IPI00181833	-0.311	0.26205	3	0	0	2
2439	Isoform 2 of Putative methyltransferase NSUN5	IPI00101659	-0.952	0.04981	2	3	0	1
2440	Isoform 3 of Exocyst complex component 7	IPI00103064	0.000	0.37049	2	1	2	1
2441	Protein SGT1	IPI00027034	0.000	0.37049	2	1	1	2
2442	alpha-methylacyl-CoA racemase isoform 3	IPI00005918	0.000	0.37049	3	1	2	2
2443	Lipoamide acyltransferase component of branched-chain alpha-keto acid dehydrogenase	IPI00003944	0.000	0.37049	1	2	0	2
2444	Isoform 1 of Splicing factor, arginine/serine-rich 12	IPI00103497	-0.311	0.26205	0	3	2	1
2445	Isoform 1 of Transmembrane protein 85	IPI00009320	0.530	0.13800	2	2	3	3
2446	Isoform 1 of Polymerase I and transcript release factor	IPI00176903	0.530	0.13800	2	2	3	3
2447	similar to unr-interacting protein	IPI00260209	0.000	0.37049	1	2	2	0
2448	DNA polymerase epsilon subunit 3	IPI00010141	-0.311	0.26205	1	3	2	1
2449	Ras-related protein Rab-22A	IPI00007756	0.588	0.12889	1	2	2	3
2450	Arfaptin-2	IPI00021257	0.000	0.37049	3	2	3	2
2451	Fumarylacetoacetase	IPI00031708	0.000	0.37049	2	2	2	2
2452	Isoform 1 of Secretory carrier-associated membrane protein 1	IPI00005129	0.000	0.37049	1	2	2	1
2453	Splicing factor 3B subunit 5	IPI00010404	0.000	0.37049	2	1	2	0
2454	28S ribosomal protein S21, mitochondrial	IPI00014812	-0.952	0.04981	3	2	1	1
2455	SNARE-associated protein Snapin	IPI00018331	-0.277	0.27840	2	3	3	1
2456	Isoform 1 of COP9 signalosome complex subunit 1	IPI00156282	-0.311	0.26205	2	2	2	1
2457	Proteasome assembly chaperone 2	IPI00644482	0.362	0.21837	0	1	2	1
2458	Peptidyl-prolyl cis-trans isomerase FKBP10	IPI00303300	0.674	0.08856	1	1	3	0
2459	Coiled-coil domain-containing protein 6	IPI00000634	0.000	0.37049	3	2	2	3
2460	cDNA FLJ56277, highly similar to Toll-like receptor 9	IPI00219489	-0.952	0.04981	3	2	1	1
2461	Isoform Mitochondrial of Phospholipid hydroperoxide glutathione peroxidase, mitochondrial	IPI00304814	0.765	0.07536	3	1	3	4
2462	DEAH (Asp-Glu-Ala-His) box polypeptide 16	IPI00292510	0.000	0.37049	2	2	2	2
2463	Isoform 1 of Nucleoredoxin	IPI00304267	0.842	0.06364	2	1	4	2
2464	Cyclin-H	IPI00021305	0.000	0.37049	1	1	0	1
2465	Biogenesis of lysosome-related organelles complex 1 subunit 1	IPI00020319	-0.952	0.04981	4	1	1	1
2466	Isoform 1 of UPF0424 protein C1orf128	IPI00015351	-0.588	0.12558	1	4	1	2

No.	Description	Accession number	STN[1]	p-Value[1]	Con_A[2]	Con_B[2]	OXA_A[2]	OXA_B[2]
2467	Nuclear pore complex protein Nup88	IPI00001738	-0.952	0.04981	4	1	0	1
2468	U6 snRNA-associated Sm-like protein LSm1	IPI00004436	-0.311	0.26205	2	2	2	0
2469	Putative transferase C1orf69, mitochondrial	IPI00145260	0.674	0.08856	1	1	2	2
2470	Isoform 1 of ATPase family AAA domain-containing protein 2	IPI00170548	0.000	0.37049	0	0	1	1
2471	Vacuolar protein sorting-associated protein 45	IPI00090327	-0.362	0.22785	0	2	0	1
2472	N-acetylgalactosaminyltransferase 7	IPI00328391	-0.362	0.22785	2	0	1	1
2473	coatomer subunit epsilon isoform c	IPI00399319	0.362	0.21837	1	1	2	0
2474	Isoform 1 of Nucleolar and spindle-associated protein 1	IPI00000398	1.445	0.01433	0	0	3	4
2475	Unhealthy ribosome biogenesis protein 2 homolog	IPI00028980	0.000	0.37049	1	0	1	0
2476	Procollagen galactosyltransferase 1	IPI00168262	0.952	0.04819	0	1	3	2
2477	Isoform 1 of Rab GTPase-activating protein 1	IPI00016702	0.674	0.08856	0	1	2	2
2478	Ubiquitin carboxyl-terminal hydrolase 10	IPI00291946	-0.362	0.22785	0	2	0	1
2479	Branched-chain-amino-acid aminotransferase	IPI00181135	0.000	0.37049	2	3	2	3
2480	Ribonuclease P protein subunit p20	IPI00027142	-0.588	0.12558	3	2	2	1
2481	Diacylglycerol O-acyltransferase 1	IPI00015799	0.362	0.21837	0	0	2	1
2482	NudC domain-containing protein 2	IPI00103142	-0.235	0.30503	4	3	5	1
2483	Isoform MLC1 of Myosin light chain 1/3, skeletal muscle isoform	IPI00216070	0.311	0.25253	2	1	3	1
2484	HDCMD34P	IPI00001672	1.078	0.03610	2	1	6	1
2485	Isoform 2 of Treacle protein	IPI00298696	0.277	0.27228	0	3	2	3
2486	Phosphoenolpyruvate carboxykinase, cytosolic [GTP]	IPI00292709	-0.952	0.04981	2	3	1	1
2487	ribonucleotide reductase M2 polypeptide isoform 1	IPI00011118	0.952	0.04819	0	1	2	3
2488	Isoform 2 of Transcription elongation factor SPT6	IPI00430770	-0.277	0.27840	3	2	2	2
2489	Isoform 1 of Autophagy-related protein 3	IPI00022254	-0.311	0.26205	2	2	2	0
2490	Isoform 2 of Heme-binding protein 2	IPI00003799	-0.765	0.07552	4	3	2	2
2491	Isoform Beta-1 of Protein phosphatase 1B	IPI00026612	-0.311	0.26205	1	3	2	1
2492	Transcription initiation factor IIE subunit beta	IPI00019981	-0.952	0.04981	2	3	1	1
2493	Isoform 1 of Deoxycytidylate deaminase	IPI00296863	0.000	0.37049	2	1	2	0
2494	N(G),N(G)-dimethylarginine dimethylaminohydrolase 1	IPI00220342	-0.311	0.26205	2	2	2	0
2495	cDNA FLJ56343, highly similar to Torsin A	IPI00413293	0.530	0.13800	2	2	3	3
2496	Isoform IIA of Myc box-dependent-interacting protein 1	IPI00186966	0.000	0.37049	2	1	2	1
2497	Proteasomal ubiquitin receptor ADRM1	IPI00033030	-0.311	0.26205	2	2	2	0
2498	Isoform 3 of Oxidation resistance protein 1	IPI00166807	-1.208	0.03035	3	3	1	1
2499	Isoform 1 of Opioid growth factor receptor	IPI00021537	-0.311	0.26205	2	2	2	0
2500	Maspardin	IPI00010248	1.208	0.02377	1	1	3	3
2501	KIF1-binding protein	IPI00477355	-0.311	0.26205	2	2	2	0
2502	Zinc finger protein ubi-d4	IPI00023322	0.588	0.12889	2	1	2	3
2503	Isoform 1 of Pre-mRNA-splicing factor 38A	IPI00171390	-0.277	0.27840	2	3	2	2
2504	Isoform 1 of Syntenin-1	IPI00299086	-0.253	0.29716	2	4	3	2
2505	Isoform 1 of Syntaxin-7	IPI00289876	0.000	0.37049	2	3	3	2
2506	Isoform 1 of Disks large homolog 3	IPI00023343	-0.362	0.22785	2	1	1	1
2507	Dynein light chain Tctex-type 1	IPI00019495	-1.208	0.03035	3	3	1	1
2508	Eukaryotic translation initiation factor 1	IPI00015077	-1.208	0.03035	3	3	1	1
2509	Beta-lactamase-like protein 2	IPI00006952	-0.362	0.22785	2	1	1	1
2510	cDNA FLJ10079 fis, clone HEMBA1001896	IPI00017494	0.000	0.37049	1	1	1	1
2511	28S ribosomal protein S30, mitochondrial	IPI00010278	0.674	0.08856	0	1	2	2
2512	Maleylacetoacetate isomerase	IPI00013809	-0.842	0.06318	3	3	1	2
2513	ATP synthase subunit delta, mitochondrial	IPI00024920	0.000	0.37049	3	2	3	2
2514	Isoform B of Syntaxin-3	IPI00220099	0.362	0.21837	1	1	1	2
2515	NADH dehydrogenase [ubiquinone] 1 beta subcomplex subunit 3	IPI00219383	0.362	0.21837	1	1	2	1
2516	Isoform 2 of Sorting nexin-3	IPI00216508	0.000	0.37049	2	2	2	2
2517	Putative uncharacterized protein PYCR2	IPI00335061	-1.078	0.03586	3	4	2	1
2518	LEM domain-containing protein 2	IPI00168336	-1.445	0.01457	4	3	1	1
2519	MKI67 FHA domain-interacting nucleolar phosphoprotein	IPI00154590	-1.445	0.01457	3	4	1	1
2520	Isoform 1 of Endophilin-B1	IPI00006558	0.000	0.37049	2	2	2	2
2521	CLASP2 protein	IPI00168165	-0.674	0.10802	2	2	1	1
2522	Leucine zipper transcription factor-like protein 1	IPI00299465	-0.842	0.06318	3	3	2	1
2523	cDNA: FLJ22221 fis, clone HRC01651	IPI00184854	0.362	0.21837	1	0	1	2
2524	NADH-cytochrome b5 reductase 1	IPI00470674	-0.362	0.22785	1	2	0	1
2525	Niemann-Pick C1 protein	IPI00005107	0.362	0.21837	0	0	2	1
2526	GDH/6PGL endoplasmic bifunctional protein	IPI00607861	-0.842	0.06318	5	1	2	1
2527	USP48 protein	IPI00328815	0.674	0.08856	0	1	2	2
2528	Isoform 2 of AT-rich interactive domain-containing protein 1A	IPI00642705	-0.362	0.22785	2	0	0	0
2529	Protein S100-A13	IPI00016179	0.674	0.08856	1	1	2	2
2530	Deoxycytidine kinase	IPI00020454	0.362	0.21837	0	0	2	1
2531	Procollagen galactosyltransferase 2	IPI00514694	0.674	0.08856	0	0	1	3
2532	Isoform 1 of GDP-fucose protein O-fucosyltransferase 1	IPI00058192	0.000	0.37049	0	0	1	1
2533	Sedoheptulokinase	IPI00005914	0.000	0.37049	0	0	1	1
2534	MAGUK p55 subfamily member 6	IPI00303280	0.000	0.37049	1	1	1	1
2535	Probable histidyl-tRNA synthetase, mitochondrial	IPI00027445	0.842	0.06364	0	2	3	3
2536	Isoform 1 of Disks large homolog 1	IPI00030351	0.000	0.37049	1	2	0	2
2537	Probable methyltransferase TARBP1	IPI00298447	0.362	0.21837	1	0	2	0
2538	cDNA FLJ60094, highly similar to F-actin capping protein subunit beta	IPI00218782	0.000	0.37049	3	0	2	2
2539	Isoform 1 of Ubiquitin conjugation factor E4 B	IPI00005715	0.362	0.21837	1	0	2	1
2540	Kinesin-like protein KIF13B	IPI00021753	0.000	0.37049	0	0	1	0
2541	Isoform A of DnaJ homolog subfamily B member 6	IPI00024523	0.588	0.12889	1	2	3	2
2542	WD repeat-containing protein 5	IPI00005492	-1.078	0.03586	4	3	2	1
2543	Isoform p27-L of 26S proteasome non-ATPase regulatory subunit 9	IPI00010860	-1.445	0.01457	3	4	1	1
2544	Poly(ADP-ribose) glycohydrolase ARH3	IPI00015865	-0.588	0.12558	1	4	1	2
2545	U3 small nucleolar RNA-interacting protein 2	IPI00217862	0.000	0.37049	1	1	0	1
2546	Cell division protein kinase 7	IPI00000685	0.362	0.21837	1	1	2	1
2547	Protein TFG	IPI00294619	-0.253	0.29716	3	3	1	4
2548	UBX domain-containing protein 7	IPI00742124	0.000	0.37049	1	2	2	0
2549	Isoform 2 of cAMP-dependent protein kinase catalytic subunit beta	IPI00376119	0.277	0.27228	1	3	3	2
2550	Isoform 1 of Craniofacial development protein 1	IPI00007306	-0.277	0.27840	3	2	3	1
2551	cDNA FLJ43556 fis, clone PROST2018511	IPI00448767	-0.362	0.22785	0	2	1	1
2552	Ubiquitin-conjugating enzyme E2 variant 2	IPI00019600	0.362	0.21837	1	1	2	1
2553	Isoform 1 of Transmembrane emp24 domain-containing protein 4	IPI00296259	0.000	0.37049	1	2	1	2
2554	Ras-related protein Rab-5B	IPI00017344	0.000	0.37049	2	3	3	2
2555	Vesicle-associated membrane protein 3	IPI00549343	-0.253	0.29716	2	4	3	2
2556	glutathione peroxidase 1 isoform 2	IPI00398780	0.277	0.27228	1	3	3	2
2557	UPF0587 protein C1orf123	IPI00016605	-0.311	0.26205	3	1	2	1
2558	28S ribosomal protein S15, mitochondrial	IPI00550037	0.588	0.12889	2	1	3	2
2559	Isoform 2 of Tescalcin	IPI00791863	0.311	0.25253	1	2	2	2
2560	Isoform 1 of Protein tyrosine phosphatase type IVA 2	IPI00020191	0.000	0.37049	2	1	1	2
2561	Proteasome assembly chaperone 3	IPI00031106	0.000	0.37049	2	0	2	1

No.	Description	Accession number	STN[1]	p-Value[1]	Con_A[2]	Con_B[2]	OXA_A[2]	OXA_B[2]
2562	NADH dehydrogenase [ubiquinone] 1 alpha subcomplex subunit 6	IPI00419266	-0.362	0.22785	2	1	1	0
2563	Bifunctional polynucleotide phosphatase/kinase	IPI00290684	-0.674	0.10802	0	3	1	1
2564	Isoform 1 of U4/U6 small nuclear ribonucleoprotein Prp3	IPI00005861	-0.674	0.10802	3	1	1	0
2565	serine/threonine-protein phosphatase 2B catalytic subunit beta isoform isoform a	IPI00027809	0.000	0.37049	1	0	1	1
2566	cDNA FLJ10360 fis, clone NT2RM2001247	IPI00182406	0.000	0.37049	0	0	1	1
2567	Isoform 1 of Protein zwilch homolog	IPI00329679	1.208	0.02377	0	1	2	4
2568	Secernin-1	IPI00289862	0.952	0.04819	0	0	1	4
2569	Isoform 1 of WD repeat-containing protein 44	IPI00444371	0.362	0.21837	1	0	0	2
2570	Ubiquitin-conjugating enzyme E2 S	IPI00217949	1.445	0.01433	0	0	3	4
2571	cDNA, FLJ96508, Homo sapiens SH3-domain GRB2-like 1 (SH3GL1), mRNA	IPI00019169	-0.674	0.10802	2	2	1	0
2572	WD repeat-containing protein 46	IPI00023126	-1.880	0.00489	4	5	0	0
2573	Isoform 1 of Spindlin-3	IPI00174535	0.674	0.08856	1	1	3	1
2574	Isoform 4 of Protein LAS1 homolog	IPI00152781	0.277	0.27228	2	2	3	2
2575	alanyl-tRNA editing protein Aarsd1 isoform 1	IPI00748490	-0.588	0.12558	2	3	2	1
2576	39S ribosomal protein L20, mitochondrial	IPI00013706	0.311	0.25253	2	1	3	1
2577	Sperm-associated antigen 7	IPI00006863	-0.530	0.15514	2	4	2	2
2578	Cleavage stimulation factor subunit 1	IPI00011528	0.952	0.04819	0	1	2	3
2579	Nucleolar complex protein 2 homolog	IPI00411886	0.000	0.37049	1	1	0	1
2580	Succinate-semialdehyde dehydrogenase, mitochondrial	IPI00019888	-0.311	0.26205	2	2	1	2
2581	Isoform 2 of VIP36-like protein	IPI00218337	0.000	0.37049	1	1	1	1
2582	Isoform 2 of Valacyclovir hydrolase	IPI00003990	-0.311	0.26205	3	1	2	1
2583	Isoform Delta 6 of Calcium/calmodulin-dependent protein kinase type II subunit delta	IPI00172636	0.000	0.37049	2	2	2	2
2584	Actin-related protein 2/3 complex subunit 1A	IPI00333068	0.952	0.04819	0	1	2	3
2585	Mitochondrial chaperone BCS1	IPI00003985	-0.311	0.26205	2	2	1	2
2586	THO complex subunit 7 homolog	IPI00291131	0.311	0.25253	2	1	2	2
2587	Isoform 2 of NAD-dependent deacetylase sirtuin-5	IPI00010331	0.000	0.37049	3	1	2	2
2588	Guanine nucleotide-binding protein G(q) subunit alpha	IPI00288947	0.000	0.37049	2	2	2	2
2589	Dipeptidase 1	IPI00059476	0.311	0.25253	1	2	2	2
2590	Phosphopantothenate--cysteine ligase	IPI00023987	0.000	0.37049	2	1	2	1
2591	Multiple coagulation factor deficiency protein 2	IPI00328680	-0.674	0.10802	2	2	1	1
2592	Isoform 1 of Coiled-coil domain-containing protein 51	IPI00153023	0.000	0.37049	1	2	2	1
2593	Queuine tRNA-ribosyltransferase	IPI00215974	-0.311	0.26205	2	2	2	1
2594	Ubiquitin-like domain-containing CTD phosphatase 1	IPI00291669	0.000	0.37049	1	3	0	3
2595	Probable ATP-dependent RNA helicase DDX28	IPI00020050	0.000	0.37049	0	1	1	0
2596	U3 small nucleolar RNA-associated protein 6 homolog	IPI00020128	-0.362	0.22785	2	1	0	0
2597	Isoform 2 of Transportin-2	IPI00164417	0.674	0.08856	1	1	2	2
2598	Immature colon carcinoma transcript 1 protein	IPI00029114	-0.277	0.27840	3	2	3	1
2599	Glutamine-dependent NAD(+) synthetase	IPI00306689	-0.277	0.27840	3	2	2	2
2600	Isoform 1 of Metallo-beta-lactamase domain-containing protein 2	IPI00293336	-0.952	0.04981	3	2	1	1
2601	Phosducin-like protein 3	IPI00031629	-0.674	0.10802	1	3	1	0
2602	Isoform 1 of Rhotekin	IPI00029834	-0.588	0.12558	3	2	2	0
2603	CDKN2A-interacting protein	IPI00020991	0.362	0.21837	0	1	2	0
2604	39S ribosomal protein L24, mitochondrial	IPI00514506	-0.674	0.10802	3	0	1	1
2605	39S ribosomal protein L18, mitochondrial	IPI00160421	-0.588	0.12558	2	3	2	1
2606	Transmembrane protein 214	IPI00477118	0.362	0.21837	1	1	1	2
2607	cDNA FLJ59722, highly similar to Retinoblastoma-binding protein 5	IPI00478230	-0.362	0.22785	2	1	1	0
2608	Synaptosomal-associated protein 29	IPI00032831	0.362	0.21837	1	1	2	1
2609	Delta-1-pyrroline-5-carboxylate dehydrogenase, mitochondrial	IPI00217871	0.000	0.37049	1	0	0	1
2610	Isoform 2 of SAP30-binding protein	IPI00333699	0.000	0.37049	2	2	3	0
2611	Isoform 1 of Mucosa-associated lymphoid tissue lymphoma translocation protein 1	IPI00009540	0.362	0.21837	0	0	1	2
2612	Cell division protein kinase 2	IPI00031681	0.952	0.04819	1	0	2	3
2613	Ubiquitin-like protein 4A	IPI00005658	-0.362	0.22785	2	0	1	0
2614	Isoform Mitochondrial of Cysteine desulfurase, mitochondrial	IPI00295240	0.000	0.37049	0	1	0	0
2615	Isoform 1 of Trafficking protein particle complex subunit 2	IPI00005119	-0.362	0.22785	2	0	1	0
2616	3-ketoacyl-CoA thiolase, peroxisomal	IPI00012828	0.674	0.08856	0	0	2	2
2617	Isoform 1 of Nucleolar protein 14	IPI00022613	-0.674	0.10802	2	2	0	0
2618	Isoform 1 of 182 kDa tankyrase-1-binding protein	IPI00304589	-1.208	0.03035	3	3	0	0
2619	Isoform 1 of Polyglutamine-binding protein 1	IPI00024698	-0.530	0.15514	4	2	3	0
2620	Probable ATP-dependent RNA helicase DDX56	IPI00302281	-1.668	0.01068	4	4	1	1
2621	Ubiquitin-conjugating enzyme E2 H	IPI00020965	0.000	0.37049	1	2	2	1
2622	Isoform 1 of Microtubule-associated protein 4	IPI00396171	-1.208	0.03035	3	3	1	0
2623	Pyruvate dehydrogenase phosphatase regulatory subunit, mitochondrial	IPI00168407	0.277	0.27228	2	2	1	4
2624	cDNA FLJ61386, highly similar to Homo sapiens mitochondrial ribosomal protein L43	IPI00334579	-0.311	0.26205	2	2	2	1
2625	PIH1 domain-containing protein 1	IPI00550995	-0.952	0.04981	4	1	1	0
2626	Transcription initiation factor IIB	IPI00022820	-0.362	0.22785	2	1	1	0
2627	Cellular retinoic acid-binding protein 2	IPI00216088	0.362	0.21837	1	1	2	1
2628	Putative uncharacterized protein LCMT1	IPI00296370	0.842	0.06364	1	2	3	3
2629	Isoform 2 of Exosome complex exonuclease RRP45	IPI00029697	0.000	0.37049	1	2	2	0
2630	EPS8L2 protein	IPI00414315	-0.674	0.10802	1	3	1	1
2631	Non-histone chromosomal protein HMG-14	IPI00554761	-0.362	0.22785	2	1	1	1
2632	PNAS-117	IPI00020827	-0.362	0.22785	2	1	1	1
2633	Vacuolar protein sorting-associated protein 4B	IPI00182728	0.000	0.37049	1	2	1	2
2634	Receptor expression-enhancing protein 5	IPI00024670	0.000	0.37049	2	2	2	2
2635	Glia maturation factor, beta	IPI00412987	-0.952	0.04981	2	3	1	0
2636	Isoform 1 of Presenilin-1	IPI00028077	0.000	0.37049	3	1	3	1
2637	Isoform 1 of Lysophospholipase-like protein 1	IPI00059762	0.000	0.37049	1	1	1	1
2638	Isoamyl acetate-hydrolyzing esterase 1 homolog	IPI00419194	0.674	0.08856	1	1	1	3
2639	Serine/threonine-protein kinase Nek7	IPI00152658	-0.674	0.10802	3	1	1	1
2640	Isoform 1 of FAD synthase	IPI00220299	0.362	0.21837	1	1	1	2
2641	Retinol dehydrogenase 14	IPI00177940	0.362	0.21837	1	1	2	1
2642	Major vault protein	IPI00000105	0.000	0.37049	2	0	1	2
2643	Conserved hypothetical protein	IPI00477526	0.674	0.08856	1	1	2	2
2644	Vacuolar protein sorting-associated protein 28 homolog	IPI00007155	-0.311	0.26205	3	1	2	1
2645	Isoform 1 of STIP1 homology and U box-containing protein 1	IPI00025156	0.000	0.37049	2	1	2	0
2646	Isoform 1 of Transcriptional repressor p66-alpha	IPI00410330	-0.362	0.22785	2	1	1	0
2647	5-formyltetrahydrofolate cyclo-ligase	IPI00220567	0.000	0.37049	1	0	1	0
2648	Golgin subfamily A member 7	IPI00480022	0.674	0.08856	1	1	3	1
2649	Dehydrogenase/reductase SDR family member on chromosome X	IPI00166860	-0.362	0.22785	2	1	0	1
2650	Aldose 1-epimerase	IPI00060200	0.362	0.21837	0	1	1	2
2651	N-acetylglucosamine-6-sulfatase	IPI00012102	-0.362	0.22785	2	0	0	1
2652	Creatine kinase B-type	IPI00022977	0.674	0.08856	1	1	2	2
2653	U3 small nucleolar ribonucleoprotein protein MPP10	IPI00012149	-0.952	0.04981	3	2	1	1
2654	Dihydropyrimidinase-related protein 1	IPI00414123	0.952	0.04819	1	0	3	2
2655	Kanadaptin	IPI00306749	0.362	0.21837	0	0	2	0
2656	Isoform 1 of Required for meiotic nuclear division protein 1 homolog	IPI00329591	0.000	0.37049	1	1	0	1

No.	Description	Accession number	STN[1]	p-Value[1]	Con_A[2]	Con_B[2]	OXA_A[2]	OXA_B[2]
2657	Coiled-coil domain-containing protein 134	IPI00302674	0.952	0.04819	1	0	2	3
2658	Isoform Alpha of Nuclear inhibitor of protein phosphatase 1	IPI00030383	-0.674	0.10802	2	2	0	1
2659	LanC-like protein 1	IPI00005724	-0.311	0.26205	3	1	2	0
2660	Exosome complex exonuclease RRP46	IPI00015955	-0.311	0.26205	2	2	2	0
2661	KDEL motif-containing protein 1	IPI00005270	0.000	0.37049	0	2	2	0
2662	Isoform A of SWI/SNF-related matrix-associated actin-dependent regulator of chromatin	IPI00029695	0.674	0.08856	0	1	3	1
2663	Vacuolar protein sorting-associated protein 37B	IPI00002926	-0.952	0.04981	2	3	0	0
2664	cDNA FLJ54030, highly similar to Polymerase delta-interacting protein 3	IPI00440688	-0.362	0.22785	2	0	1	0
2665	Myotubularin	IPI00748788	0.362	0.21837	0	0	2	1
2666	Probable leucyl-tRNA synthetase, mitochondrial	IPI00014213	0.000	0.37049	2	0	0	2
2667	Serine/threonine-protein kinase PLK1	IPI00021248	0.362	0.21837	0	0	2	1
2668	Argininosuccinate lyase	IPI00220267	0.952	0.04819	0	0	3	2
2669	Isoform 14 of Dysferlin	IPI00020210	-0.311	0.26205	0	3	0	2
2670	ATP synthase-coupling factor 6, mitochondrial	IPI00002521	-0.674	0.10802	2	2	0	0
2671	Isoform 5 of Protein transport protein Sec16A	IPI00031242	-0.952	0.04981	2	3	1	0
2672	Isoform 2 of Condensin-2 complex subunit G2	IPI00396058	0.674	0.08856	0	0	2	2
2673	Isoform Long of Transcription intermediary factor 1-alpha	IPI00005184	0.362	0.21837	0	1	2	0
2674	Ribosomal RNA processing protein 1 homolog A	IPI00550766	-0.674	0.10802	2	2	1	1
2675	Torsin A interacting protein 1	IPI00644766	-0.588	0.12558	3	2	2	1
2676	Isoform 1 of Gamma-glutamyltransferase 7	IPI00030634	0.311	0.25253	1	2	2	2
2677	Mitochondrial import receptor subunit TOM7 homolog	IPI00000980	0.000	0.37049	2	0	2	1
2678	Estradiol 17-beta-dehydrogenase 8	IPI00021890	-0.362	0.22785	2	1	1	1
2679	Isoform 1 of Apoptosis-inducing factor 2	IPI00013909	0.000	0.37049	2	1	2	1
2680	Interferon regulatory factor 2-binding protein 1	IPI00645608	0.000	0.37049	1	1	1	1
2681	Isoform 1 of Transmembrane protein 163	IPI00152253	0.000	0.37049	2	2	2	2
2682	Charged multivesicular body protein 7	IPI00395463	0.000	0.37049	2	2	2	2
2683	Rho GDP-dissociation inhibitor 2	IPI00003817	0.311	0.25253	1	2	2	2
2684	Isoform 1 of Protein dpy-19 homolog 1	IPI00007461	-0.362	0.22785	2	0	1	0
2685	Transcription factor MafG	IPI00007311	0.000	0.37049	2	1	2	1
2686	Mitotic spindle assembly checkpoint protein MAD2A	IPI00012369	-0.311	0.26205	3	1	1	2
2687	Isoform 1 of ATP-dependent metalloprotease YME1L1	IPI00045946	0.952	0.04819	1	1	2	3
2688	NADH dehydrogenase [ubiquinone] 1 alpha subcomplex subunit 2	IPI00219381	-0.952	0.04981	3	2	1	1
2689	Vesicle-associated membrane protein 8	IPI00030911	-0.674	0.10802	2	2	1	1
2690	Isoform 3 of Yorkie homolog	IPI00009326	-0.952	0.04981	1	4	0	1
2691	cDNA FLJ58610	IPI00100930	-0.674	0.10802	2	2	1	1
2692	Transcription initiation factor TFIID subunit 9	IPI00002993	0.362	0.21837	1	1	2	0
2693	Isoform 1 of Ribulose-phosphate 3-epimerase	IPI00335280	0.000	0.37049	1	1	0	0
2694	NEDD4-like E3 ubiquitin-protein ligase WWP2	IPI00013010	0.674	0.08856	0	1	2	2
2695	Isoform 1 of Neuroguidin	IPI00000162	-0.674	0.10802	2	2	1	1
2696	Mitochondrial fission 1 protein	IPI00007052	-0.362	0.22785	2	0	1	0
2697	RAB4A, member RAS oncogene family variant	IPI00480056	0.311	0.25253	1	2	2	2
2698	Isoform Alpha of E3 ubiquitin-protein ligase TRIM33	IPI00010252	-0.362	0.22785	0	2	1	1
2699	Protein AATF	IPI00302238	-1.208	0.03035	3	3	0	1
2700	39S ribosomal protein L53, mitochondrial	IPI00061531	-0.952	0.04981	2	3	1	0
2701	Ubiquilin-2	IPI00409659	0.952	0.04819	1	0	2	3
2702	Isoform II of Ubiquitin-protein ligase E3A	IPI00011609	-0.362	0.22785	1	2	0	1
2703	MMP37-like protein, mitochondrial	IPI00060287	-0.952	0.04981	2	3	1	0
2704	cDNA FLJ55543, highly similar to Phosphoacetylglucosamine mutase	IPI00030116	0.362	0.21837	1	0	2	1
2705	Transmembrane protein C3orf1	IPI00299387	0.000	0.37049	0	1	0	0
2706	Origin recognition complex subunit 4	IPI00015164	-0.588	0.12558	2	3	0	2
2707	Isoform 2 of Neuropathy target esterase	IPI00217600	0.362	0.21837	0	0	2	1
2708	Isoform 1 of Carnitine O-acetyltransferase	IPI00016457	0.674	0.08856	0	0	0	3
2709	Activator of basal transcription 1	IPI00002938	-0.674	0.10802	3	1	1	0
2710	Isoform 1 of Probable E3 ubiquitin-protein ligase HERC4	IPI00333067	0.362	0.21837	0	0	0	2
2711	74 kDa protein	IPI00290439	-0.362	0.22785	0	2	0	1
2712	Isoform 2 of DNA-directed RNA polymerase I subunit RPA2	IPI00026445	0.362	0.21837	0	0	1	2
2713	Neudesin	IPI00002525	0.674	0.08856	0	0	3	0
2714	Serine/threonine-protein kinase N2	IPI00002804	0.674	0.08856	0	0	2	2
2715	Coiled-coil domain-containing protein 25	IPI00396174	-0.952	0.04981	4	1	1	0
2716	Isoform 2 of Integrator complex subunit 7	IPI00645022	0.000	0.37049	0	0	0	1
2717	Putative uncharacterized protein ZNF326	IPI00337602	0.674	0.08856	1	1	2	2
2718	E-cadherin	IPI00000513	-0.952	0.04981	2	3	1	1
2719	OTU domain-containing protein 6B	IPI00182180	-0.362	0.22785	1	2	1	0
2720	Isoform 1 of Alpha-globin transcription factor CP2	IPI00037599	-0.311	0.26205	1	3	2	1
2721	Proteasome maturation protein	IPI00006377	0.362	0.21837	1	0	2	0
2722	Protein phosphatase 1F	IPI00291412	0.674	0.08856	1	1	2	2
2723	Isoform 3 of Tyrosine-protein kinase Lck	IPI00515097	-0.311	0.26205	2	2	2	1
2724	X-prolyl aminopeptidase (Aminopeptidase P) 1, soluble	IPI00514564	0.588	0.12889	1	2	2	3
2725	Glycolipid transfer protein	IPI00184363	0.000	0.37049	2	0	2	0
2726	Putative uncharacterized protein LARP4B	IPI00847241	0.311	0.25253	1	2	2	2
2727	Isoform 1 of Copine-7	IPI00002657	-0.674	0.10802	2	2	1	0
2728	Trafficking protein particle complex subunit 4	IPI00007691	0.362	0.21837	1	1	2	1
2729	Isoform 1 of Nuclear ubiquitous casein and cyclin-dependent kinases substrate	IPI00022145	0.674	0.08856	1	1	2	2
2730	Syntaxin-8	IPI00009225	0.674	0.08856	1	1	2	2
2731	Isoform 3 of DnaJ homolog subfamily C member 11	IPI00333016	-0.311	0.26205	2	2	2	1
2732	Putative uncharacterized protein TXNRD2	IPI00157820	0.362	0.21837	1	0	1	2
2733	M-phase phosphoprotein 6	IPI00016074	-1.208	0.03035	3	3	0	0
2734	Thioredoxin-like protein 4A	IPI00216338	0.000	0.37049	1	1	1	1
2735	Small ubiquitin-related modifier 1	IPI00303105	0.000	0.37049	2	0	2	1
2736	Protein kinase C and casein kinase substrate in neurons 3, isoform CRA_b	IPI00329572	0.000	0.37049	1	2	1	2
2737	Isoform 1 of Ribosome-recycling factor, mitochondrial	IPI00061108	0.000	0.37049	2	2	2	2
2738	[Pyruvate dehydrogenase [lipoamide]] kinase isozyme 3, mitochondrial	IPI00014849	0.311	0.25253	2	1	2	2
2739	Isoform 2 of Torsin-1A-interacting protein 1	IPI00012280	-0.952	0.04981	3	2	1	1
2740	Inositol polyphosphate 1-phosphatase	IPI00027139	0.362	0.21837	1	1	2	1
2741	Isoform 1 of Ran-binding protein 3	IPI00026337	-0.674	0.10802	2	2	0	1
2742	Isoform 1 of Glycerol kinase	IPI00027424	0.000	0.37049	1	0	1	0
2743	Lysosomal alpha-mannosidase	IPI00012989	0.674	0.08856	0	0	3	0
2744	TBC1 domain family member 5	IPI00022450	-0.362	0.22785	2	1	0	0
2745	Isoform 3 of Mitochondrial Rho GTPase 1	IPI00217536	-0.674	0.10802	2	2	0	1
2746	Interferon-induced protein with tetratricopeptide repeats 5	IPI00012756	0.000	0.37049	1	0	0	1
2747	Probable ATP-dependent RNA helicase DDX52	IPI00032423	-0.588	0.12558	2	3	0	2
2748	Isoform Bcl-X(L) of Bcl-2-like protein 1	IPI00019983	0.277	0.27228	2	2	2	3
2749	Isoform 1 of Citrate lyase subunit beta-like protein, mitochondrial	IPI00477957	-0.674	0.10802	2	2	0	0
2750	Thioredoxin-related transmembrane protein 4	IPI00100247	0.000	0.37049	0	1	0	1
2751	37 kDa protein	IPI00032799	-1.445	0.01457	3	4	1	0

No.	Description	Accession number	STN[1]	p-Value[1]	Con_A[2]	Con_B[2]	OXA_A[2]	OXA_B[2]
2752	Isoform 1 of tRNA guanosine-2'-O-methyltransferase TRM11 homolog	IPI00470606	0.362	0.21837	0	1	1	2
2753	Borealin	IPI00303099	0.000	0.37049	0	1	0	1
2754	Isoform 1 of Quinone oxidoreductase PIG3	IPI00384643	0.000	0.37049	0	2	1	2
2755	Isoform 2 of Monoacylglycerol lipase ABHD12	IPI00060569	0.000	0.37049	1	0	1	1
2756	S-adenosyl-L-methionine-dependent methyltransferase FTSJD2	IPI00166153	-0.362	0.22785	2	1	1	0
2757	Isoform 1 of GPI transamidase component PIG-S	IPI00465308	0.362	0.21837	0	0	2	1
2758	Isoform 2 of Nuclear pore complex protein Nup160	IPI00221235	1.208	0.02377	0	1	3	3
2759	Patatin-like phospholipase domain-containing protein 4	IPI00013218	0.362	0.21837	1	0	0	2
2760	Uncharacterized protein C10orf58	IPI00296190	0.674	0.08856	1	1	3	0
2761	cDNA FLJ12528 fis, clone NT2RM4000155	IPI00018632	0.362	0.21837	0	1	1	2
2762	Ras-related protein Rab-32	IPI00014377	0.952	0.04819	0	0	3	2
2763	Isoform 1 of Crooked neck-like protein 1	IPI00177437	0.000	0.37049	1	0	0	1
2764	Protein FAM50A	IPI00030098	-0.674	0.10802	2	2	1	1
2765	Putative uncharacterized protein DOCK11	IPI00411452	0.000	0.37049	1	0	0	0
2766	Isoform A of Methyl-CpG-binding protein 2	IPI00418234	-0.362	0.22785	0	2	0	0
2767	Isoform 1 of Beta-enolase	IPI00218474	0.000	0.37049	1	0	1	0
2768	Isoform 1 of Kinesin-like protein KIF1B	IPI00029011	0.362	0.21837	0	0	2	1
2769	Isoform 1 of Protein GPR89	IPI00472858	0.362	0.21837	0	0	2	0
2770	Protein CTF18 homolog	IPI00178203	0.362	0.21837	0	0	1	2
2771	RNA polymerase-associated protein CTR9 homolog	IPI00477468	0.674	0.08856	0	1	3	1
2772	Isoform 1 of Dynamin-1-like protein	IPI00146935	0.000	0.37049	1	3	2	2
2773	Methionine aminopeptidase 2	IPI00033036	-0.952	0.04981	2	3	1	1
2774	Isoform 1 of UAP56-interacting factor	IPI00289907	-0.674	0.10802	1	3	1	1
2775	AP-3 complex subunit sigma-1	IPI00014624	-0.674	0.10802	2	2	1	1
2776	Isoform 1 of Mitochondrial Rho GTPase 2	IPI00465059	0.000	0.37049	0	1	1	1
2777	cDNA: FLJ22728 fis, clone HSI15617 (Fragment)	IPI00386139	-0.952	0.04981	3	2	1	1
2778	Isoform 1 of YTH domain family protein 2	IPI00306043	0.362	0.21837	1	1	0	2
2779	G patch domain and KOW motifs-containing protein	IPI00024255	-0.674	0.10802	2	2	1	1
2780	Putative uncharacterized protein DCP1A	IPI00164672	-0.674	0.10802	2	2	1	1
2781	NADH dehydrogenase [ubiquinone] 1 subunit C2	IPI00029558	0.000	0.37049	1	2	1	2
2782	Isoform 2 of Uncharacterized protein C3orf21	IPI00165665	-0.674	0.10802	2	2	1	1
2783	Death domain-containing protein CRADD	IPI00020364	0.000	0.37049	1	1	0	1
2784	Isoform 2 of Vesicle-associated membrane protein 7	IPI00013236	0.362	0.21837	1	1	2	1
2785	Isoform Long of Nucleolysin TIA-1 isoform p40	IPI00291398	0.362	0.21837	0	1	2	0
2786	164 kDa protein	IPI00465246	-0.362	0.22785	0	2	0	0
2787	Isoform 6 of Ribosome-recycling factor, mitochondrial	IPI00030596	0.000	0.37049	1	1	0	1
2788	Isoform 1 of Ubiquitin-like modifier-activating enzyme 5	IPI00015736	-0.362	0.22785	2	1	1	0
2789	Mitogen-activated protein kinase scaffold protein 1	IPI00030919	-0.674	0.10802	3	0	0	1
2790	Isoform 1 of Transmembrane protein 192	IPI00855873	0.000	0.37049	1	2	2	0
2791	Isoform 3 of Epithelial splicing regulatory protein 1	IPI00184262	-0.952	0.04981	3	2	1	1
2792	Isoform 2 of Oxidoreductase HTATIP2	IPI00383665	0.000	0.37049	2	0	1	2
2793	Isoform 1 of Adaptin ear-binding coat-associated protein 2	IPI00018188	0.362	0.21837	1	0	1	2
2794	tRNA-splicing endonuclease subunit Sen15	IPI00450071	-0.674	0.10802	2	2	0	0
2795	Protein FAM128B	IPI00410094	0.311	0.25253	1	2	2	2
2796	TATA box-binding protein-like protein 1	IPI00032911	0.362	0.21837	1	0	2	1
2797	Isoform 1 of Integrator complex subunit 11	IPI00063404	0.362	0.21837	1	0	2	0
2798	Isoform 1 of AP-1 complex subunit mu-2	IPI00002552	0.000	0.37049	2	1	2	0
2799	NADH dehydrogenase [ubiquinone] iron-sulfur protein 5	IPI00220063	-0.362	0.22785	2	1	1	0
2800	Methylcrotonoyl-CoA carboxylase subunit alpha, mitochondrial	IPI00024580	-0.362	0.22785	2	0	0	0
2801	Isoform 1 of CWF19-like protein 1	IPI00101600	0.362	0.21837	0	0	1	2
2802	Isoform 1 of Alanine aminotransferase 2	IPI00152432	-0.674	0.10802	2	2	0	0
2803	Isoform 1 of Lariat debranching enzyme	IPI00305545	0.674	0.08856	0	0	2	2
2804	Exportin-4	IPI00028357	0.000	0.37049	0	2	2	0
2805	Isoform 1 of Phosphatidylinositol 4-kinase alpha	IPI00070943	0.362	0.21837	0	0	1	2
2806	Histone deacetylase 4	IPI00010088	0.000	0.37049	0	1	0	1
2807	Translation initiation factor IF-2, mitochondrial	IPI00005039	0.000	0.37049	1	2	2	0
2808	MLL1/MLL complex subunit C17orf49 isoform 1	IPI00373869	-0.674	0.10802	2	2	0	0
2809	Isoform 1 of Mixed lineage kinase domain-like protein	IPI00180781	0.000	0.37049	0	2	1	2
2810	Eukaryotic translation initiation factor 4E	IPI00027485	-0.674	0.10802	0	3	0	1
2811	Isoform 1 of Nucleobindin-2	IPI00009123	0.362	0.21837	0	0	0	2
2812	Protein farnesyltransferase subunit beta	IPI00026817	0.000	0.37049	1	1	1	0
2813	Isoform 1 of Dehydrogenase/reductase SDR family member 11	IPI00034280	-0.362	0.22785	2	0	1	1
2814	Methylmalonate-semialdehyde dehydrogenase [acylating], mitochondria	IPI00024990	0.362	0.21837	0	0	2	0
2815	Isoform 1 of BAG family molecular chaperone regulator 5	IPI00007731	0.674	0.08856	0	1	2	2
2816	Ubiquitin-conjugating enzyme E2 C	IPI00013002	0.674	0.08856	0	0	3	0
2817	Carboxypeptidase D	IPI00027078	-0.362	0.22785	0	2	0	0
2818	Kinesin-like protein KIF22	IPI00000769	0.362	0.21837	0	0	2	1
2819	Isoform 1 of Putative hexokinase HKDC1	IPI00414612	-0.362	0.22785	2	1	0	0
2820	NF-kappa-B-repressing factor	IPI00005675	-0.952	0.04981	1	4	1	0
2821	Protein Red	IPI00011875	0.000	0.37049	0	1	0	0
2822	Keratin, type II cytoskeletal 3	IPI00290857	0.000	0.37049	0	0	0	0
2823	WD repeat-containing protein 70	IPI00300060	-0.674	0.10802	3	0	0	0
2824	PRKC apoptosis WT1 regulator protein	IPI00001871	-0.362	0.22785	2	0	0	0
2825	Endonuclease/exonuclease/phosphatase family domain-containing protein 1	IPI00885036	0.000	0.37049	2	1	1	2
2826	Biogenesis of lysosome-related organelles complex 1 subunit 3	IPI00397721	0.362	0.21837	1	1	1	2
2827	Isoform 2 of Core-binding factor subunit beta	IPI00024871	0.000	0.37049	1	2	1	2
2828	Mevalonate kinase	IPI00010717	0.362	0.21837	1	1	2	1
2829	D-tyrosyl-tRNA(Tyr) deacylase 1	IPI00152692	-0.362	0.22785	1	2	1	1
2830	Polymerase delta interacting protein 46	IPI00429180	0.000	0.37049	1	1	1	1
2831	Protein UXT	IPI00002646	-0.311	0.26205	2	2	2	0
2832	Putative uncharacterized protein QTRTD1	IPI00074010	0.362	0.21837	0	1	1	2
2833	Isoform 1 of Creatine kinase U-type, mitochondrial	IPI00658109	-0.362	0.22785	2	1	1	1
2834	Isoform 4 of Inhibitor of nuclear factor kappa-B kinase-interacting protein	IPI00043598	-0.674	0.10802	1	3	1	1
2835	Putative uncharacterized protein DKFZp686H16220	IPI00552191	-0.362	0.22785	1	2	0	1
2836	Isoform 1 of Cysteine protease ATG4B	IPI00554649	0.000	0.37049	1	1	1	1
2837	Ubiquitin-conjugating enzyme E2 R2	IPI00418603	0.674	0.08856	1	0	3	0
2838	Isoform 3 of Parkinson disease 7 domain-containing protein 1	IPI00167976	-0.311	0.26205	2	2	2	1
2839	Dynactin subunit 4	IPI00550852	-0.362	0.22785	1	2	1	1
2840	Probable serine carboxypeptidase CPVL	IPI00301395	0.000	0.37049	1	1	0	0
2841	Isoform 2 of RNA-binding protein 47	IPI00005042	0.362	0.21837	1	1	1	2
2842	Isoform 4 of Mitochondrial fission factor	IPI00024627	-0.362	0.22785	2	1	0	0
2843	Isoform 2 of tRNA-dihydrouridine synthase 3-like	IPI00640947	0.000	0.37049	0	2	1	2
2844	39S ribosomal protein L54, mitochondrial	IPI00332157	-0.674	0.10802	2	2	1	0
2845	Cytochrome P450 monooxygenase	IPI00010218	0.362	0.21837	1	1	2	0
2846	Isoform 1 of Selenocysteine lyase	IPI00101652	0.362	0.21837	0	1	2	1

No.	Description	Accession number	STN[1]	p-Value[1]	Con_A[2]	Con_B[2]	OXA_A[2]	OXA_B[2]
2847	GDP-L-fucose synthase	IPI00014361	-0.952	0.04981	2	3	0	0
2848	60S ribosomal protein L31	IPI00026302	-0.952	0.04981	3	2	1	0
2849	HLA class I histocompatibility antigen, B-7 alpha chain	IPI00004657	0.674	0.08856	1	1	3	1
2850	Isoform 2 of Rho guanine nucleotide exchange factor 1	IPI00339379	0.311	0.25253	1	2	2	2
2851	Uncharacterized protein C20orf4	IPI00166013	-0.674	0.10802	2	2	1	0
2852	Active regulator of SIRT1	IPI00219006	-0.311	0.26205	2	2	2	0
2853	Protein unc-119 homolog B	IPI00414629	0.000	0.37049	1	2	2	1
2854	Isoform 3 of Protein DDI1 homolog 2	IPI00031618	-0.674	0.10802	2	2	0	1
2855	AP-3 complex subunit mu-1	IPI00032459	-0.674	0.10802	0	3	1	1
2856	sorting nexin-6 isoform a	IPI00258833	0.000	0.37049	0	0	1	1
2857	Methionyl-tRNA synthetase, mitochondrial	IPI00062839	0.952	0.04819	0	0	3	2
2858	Transcription elongation factor A protein-like 2	IPI00002013	-0.362	0.22785	2	1	0	0
2859	UPF0480 protein C15orf24	IPI00024551	0.362	0.21837	1	0	2	0
2860	Isoform 1 of Elongator complex protein 3	IPI00165477	0.362	0.21837	1	0	0	2
2861	Sorting nexin-5	IPI00295209	0.000	0.37049	2	0	2	0
2862	Vacuolar protein sorting-associated protein 33B	IPI00032905	0.000	0.37049	0	0	1	0
2863	Serine/threonine-protein kinase 12	IPI00176642	0.362	0.21837	0	0	1	2
2864	Isoform 2 of Arf-GAP with Rho-GAP domain	IPI00220421	0.362	0.21837	0	0	2	1
2865	Isoform 4 of Zinc finger protein 638	IPI00178953	-0.674	0.10802	0	3	1	0
2866	Isoform 1 of Protein SDA1 homolog	IPI00018240	-0.362	0.22785	2	0	0	0
2867	High mobility group protein B1	IPI00419258	0.000	0.37049	0	1	1	1
2868	Ubiquitin protein ligase E3 component n-recognin 4	IPI00514902	0.362	0.21837	0	0	2	1
2869	Isoform 1 of Zinc finger protein 326	IPI00373877	-0.674	0.10802	2	2	1	1
2870	Stromal cell-derived factor 2	IPI00293167	0.000	0.37049	2	1	2	1
2871	Proteasome assembly chaperone 4	IPI00895892	0.362	0.21837	1	1	2	1
2872	Isoform 1 of COMM domain-containing protein 7	IPI00743772	0.000	0.37049	1	2	1	2
2873	Isoform 2 of DnaJ homolog subfamily C member 5	IPI00023780	0.000	0.37049	1	2	0	2
2874	B-cell lymphoma/leukemia 10	IPI00022477	0.362	0.21837	1	1	2	1
2875	Mitochondrial import inner membrane translocase subunit Tim8 A	IPI00028376	-0.674	0.10802	2	2	0	1
2876	Putative phospholipase B-like 2	IPI00169285	-0.362	0.22785	1	2	1	1
2877	Isoform 1 of ADP-ribosylation factor GTPase-activating protein 1	IPI00175169	-0.362	0.22785	0	2	1	1
2878	U8 snoRNA-decapping enzyme	IPI00783497	0.362	0.21837	1	1	0	2
2879	Ras-related protein Rab-6C	IPI00030304	0.000	0.37049	1	1	0	1
2880	GTP-binding protein 1	IPI00010463	-0.362	0.22785	0	2	1	1
2881	5'-nucleotidase domain-containing protein 1	IPI00177965	0.674	0.08856	1	0	1	3
2882	Isoform 1 of Ubiquinone biosynthesis methyltransferase COQ5, mitochondrial	IPI00456965	-0.674	0.10802	2	2	0	0
2883	RRP15-like protein	IPI00007004	0.362	0.21837	1	1	2	0
2884	KIAA1033 protein	IPI00298991	0.000	0.37049	0	1	1	1
2885	dehydrogenase/reductase SDR family member 4	IPI00106913	0.000	0.37049	0	2	2	1
2886	Iron-sulfur cluster assembly 2 homolog, mitochondrial	IPI00376195	-0.362	0.22785	2	0	1	0
2887	CD2 antigen cytoplasmic tail-binding protein 2	IPI00006103	-0.362	0.22785	2	0	0	1
2888	Alpha-mannosidase 2	IPI00003802	0.674	0.08856	0	0	2	2
2889	PRMT3 protein (Fragment)	IPI00103026	0.000	0.37049	0	1	1	1
2890	Isoform 1 of High mobility group protein 20A	IPI00018924	-0.362	0.22785	2	1	1	0
2891	Isoform 1 of General transcription factor 3C polypeptide 3	IPI00015806	0.000	0.37049	1	0	0	1
2892	cDNA FLJ20475 fis, clone KAT07206	IPI00183065	0.362	0.21837	1	0	0	2
2893	Kinesin-like protein KIFC1	IPI00306400	0.362	0.21837	0	0	1	2
2894	Uncharacterized protein C6orf130	IPI00184871	0.000	0.37049	1	0	1	0
2895	Isoform 1 of U3 small nucleolar RNA-associated protein 14 homolog A	IPI00107113	-0.952	0.04981	3	2	0	0
2896	Guanine deaminase	IPI00644409	0.674	0.08856	0	0	2	2
2897	Methionine aminopeptidase 1	IPI00022239	-0.674	0.10802	2	2	0	0
2898	Protein XRP2	IPI00026627	0.362	0.21837	1	0	1	2
2899	Isoform Long of Ancient ubiquitous protein 1	IPI00001891	0.000	0.37049	0	0	1	1
2900	Isoform 2 of Transcription factor p65	IPI00219084	0.674	0.08856	0	0	3	0
2901	Isoform 1 of Abhydrolase domain-containing protein FAM108B1	IPI00412592	0.000	0.37049	2	0	2	0
2902	Isoform 1 of Peptidyl-prolyl cis-trans isomerase SDCCAG10	IPI00025174	-0.362	0.22785	2	1	1	0
2903	Armadillo repeat-containing protein 1	IPI00018260	-0.362	0.22785	2	1	0	1
2904	Isoform 1 of COMM domain-containing protein 4	IPI00413500	-0.362	0.22785	0	2	0	0
2905	cDNA FLJ55508, highly similar to Sad1/unc-84-like protein 2	IPI00295940	0.362	0.21837	1	0	2	1
2906	Isoform 1 of Protein NDRG3	IPI00005605	-0.362	0.22785	1	2	0	0
2907	Isoform A of Uncharacterized protein C21orf70	IPI00027898	-1.445	0.01457	4	3	0	0
2908	ubiquitin-like with PHD and ring finger domains 1 isoform 2	IPI00797279	0.362	0.21837	0	0	1	2
2909	Isoform 1 of RNA polymerase II-associated factor 1 homolog	IPI00300333	-0.952	0.04981	1	4	0	0
2910	Gamma-tubulin complex component 2	IPI00029705	0.362	0.21837	0	1	0	2
2911	FLJ00369 protein (Fragment)	IPI00166711	0.362	0.21837	0	0	2	0
2912	Isoform 1 of Uncharacterized protein C1orf31	IPI00045660	-0.362	0.22785	1	2	1	1
2913	Protein yippee-like 5	IPI00429538	-0.362	0.22785	2	1	1	1
2914	Isoform 1 of MYC-induced nuclear antigen	IPI00216737	0.000	0.37049	1	2	2	1
2915	Isoform 2 of Frataxin, mitochondrial	IPI00217745	0.000	0.37049	1	1	1	0
2916	Isoform 1 of PHD finger protein 6	IPI00395568	-0.362	0.22785	0	2	1	1
2917	15 kDa selenoprotein isoform 1 precursor	IPI00030877	-0.674	0.10802	2	2	1	0
2918	Isoform 3 of Ubiquitin-protein ligase E3C	IPI00411748	0.000	0.37049	1	2	1	2
2919	33 kDa protein	IPI00386323	0.000	0.37049	1	1	1	1
2920	Bis(5'-nucleosyl)-tetraphosphatase [asymmetrical]	IPI00221231	0.362	0.21837	0	0	2	0
2921	Isoform 3 of Vesicle transport protein SEC20	IPI00030397	-0.362	0.22785	1	2	0	1
2922	Isoform 1 of SAC3 domain-containing protein 1	IPI00854724	0.362	0.21837	0	1	1	2
2923	CGG triplet repeat-binding protein 1	IPI00295585	-0.362	0.22785	2	1	1	0
2924	Isoform 1 of Serine/threonine-protein phosphatase 4 regulatory subunit 3A	IPI00217013	0.674	0.08856	0	0	1	3
2925	Thymidylate synthetase, isoform CRA_a	IPI00103732	0.362	0.21837	0	1	2	1
2926	Isoform 1 of Coronin-7	IPI00027996	0.362	0.21837	0	0	2	1
2927	Ras-related protein Rab-13	IPI00016373	0.362	0.21837	0	0	2	1
2928	Isoform 1 of Casein kinase I isoform alpha	IPI00183400	-0.362	0.22785	1	2	1	0
2929	Protein ariadne-1 homolog	IPI00294943	0.362	0.21837	1	1	2	1
2930	Isoform 5 of Methyltransferase-like protein 13	IPI00384061	0.362	0.21837	0	1	0	2
2931	Pro-apoptotic protein BAKM variant	IPI00386229	0.000	0.37049	1	0	0	1
2932	Isoform 2 of Evolutionarily conserved signaling intermediate in Toll pathway, mitochondria	IPI00063188	0.000	0.37049	1	0	0	1
2933	Isoform 1 of Nicotinamide mononucleotide adenylyltransferase 3	IPI00290687	-0.362	0.22785	2	0	0	0
2934	Isoform 1 of Protein syndesmos	IPI00031650	-0.674	0.10802	3	1	0	1
2935	Polypeptide N-acetylgalactosaminyltransferase 2	IPI00004669	0.000	0.37049	0	0	1	0
2936	Isoform 2 of Multiple inositol polyphosphate phosphatase 1	IPI00028553	0.362	0.21837	0	1	0	2
2937	Coilin	IPI00006442	-0.362	0.22785	0	2	0	1
2938	Coiled-coil domain-containing protein 22	IPI00022265	-0.362	0.22785	0	2	0	1
2939	Glypican-4	IPI00232571	0.362	0.21837	0	0	1	2
2940	Derlin-2	IPI00304264	0.362	0.21837	0	0	2	1
2941	Isoform 3 of Tropomyosin beta chain	IPI00218820	0.362	0.21837	0	0	1	2

No.	Description	Accession number	STN[1]	p-Value[1]	Con_A[2]	Con_B[2]	OXA_A[2]	OXA_B[2]
2942	Isoform 3 of Formin-binding protein 1-like	IPI00015580	0.674	0.08856	0	0	3	0
2943	Isoform 1 of Upstream-binding protein 1	IPI00005018	-0.362	0.22785	0	2	1	1
2944	Isoform 1 of Cell surface glycoprotein MUC18	IPI00016334	0.000	0.37049	0	0	0	1
2945	Isoform RON of Macrophage-stimulating protein receptor	IPI00030273	0.362	0.21837	0	0	2	0
2946	Isoform 1 of Anaphase-promoting complex subunit 5	IPI00008247	0.362	0.21837	0	0	2	1
2947	Armadillo repeat-containing protein 6	IPI00020196	-0.362	0.22785	0	2	1	0
2948	poly [ADP-ribose] polymerase 14	IPI00291215	0.362	0.21837	0	0	2	0
2949	Isoform 2 of Cytochrome P450 2S1	IPI00164018	0.000	0.37049	0	0	1	1
2950	Gamma-aminobutyric acid receptor-associated protein	IPI00027253	-0.362	0.22785	2	0	0	0
2951	Biotin--protein ligase	IPI00301907	-0.362	0.22785	2	1	1	0
2952	Uncharacterized protein C1orf198	IPI00013912	-0.362	0.22785	2	1	0	0
2953	Exocyst complex component 2	IPI00783559	0.674	0.08856	0	0	0	3
2954	Isoform 1 of B-cell CLL/lymphoma 7 protein family member C	IPI00006266	-0.362	0.22785	2	0	1	1
2955	RPB11a protein	IPI00003310	0.000	0.37049	1	1	1	1
2956	Threonine synthase-like 1	IPI00016287	0.362	0.21837	1	1	1	2
2957	Ras-related protein Rab-24	IPI00056496	0.000	0.37049	1	0	1	0
2958	Histidine triad nucleotide-binding protein 3	IPI00170924	-0.362	0.22785	1	2	0	0
2959	Glyoxylate reductase/hydroxypyruvate reductase, isoform CRA_c	IPI00026486	-0.362	0.22785	1	2	1	0
2960	Coiled-coil domain-containing protein 12	IPI00453463	-0.362	0.22785	1	2	0	0
2961	Isoform 1 of Putative S-adenosyl-L-methionine-dependent methyltransferase METT5D1	IPI00783001	-0.362	0.22785	2	0	0	0
2962	Fumarylacetoacetate hydrolase domain-containing protein 2A	IPI00329742	0.000	0.37049	0	2	0	2
2963	Secernin-2	IPI00062266	0.000	0.37049	1	2	2	0
2964	cDNA FLJ53160, highly similar to Zyxin	IPI00871311	-0.674	0.10802	2	2	0	0
2965	Ufm1-specific protease 2	IPI00305303	0.000	0.37049	0	0	1	1
2966	Isoform 2 of Septin-8	IPI00022082	0.000	0.37049	0	0	1	1
2967	tRNA-dihydrouridine synthase 2-like	IPI00015804	-0.362	0.22785	2	1	0	0
2968	Protein kinase, AMP-activated, alpha 1 catalytic subunit, isoform CRA_b	IPI00061282	0.362	0.21837	0	0	2	1
2969	Galectin-7	IPI00219221	0.000	0.37049	0	0	0	0
2970	Syntaxin-18	IPI00027194	0.362	0.21837	0	0	0	2
2971	Isoform Alpha of Caspase-6	IPI00023876	-0.362	0.22785	2	0	0	0
2972	F-box only protein 7	IPI00294567	0.362	0.21837	0	0	2	1
2973	Isoform 1 of Diphthamide biosynthesis protein 1	IPI00718991	-0.362	0.22785	0	2	0	0
2974	Isoform 3 of Leucine-rich repeat-containing protein 20	IPI00018929	0.000	0.37049	0	0	0	0
2975	Monoacylglycerol lipase ABHD6	IPI00107039	0.000	0.37049	0	0	1	0
2976	cDNA FLJ58333, highly similar to T-lymphokine-activated killer cell-originated protein kinase	IPI00306708	0.362	0.21837	0	0	2	0
2977	Isoform 1 of PDZ domain-containing protein 11	IPI00550841	0.000	0.37049	1	1	1	0
2978	DNA-directed RNA polymerase II subunit RPB4	IPI00007283	-0.362	0.22785	1	2	1	0
2979	Isoform 2 of Arf-GAP domain and FG repeats-containing protein 1	IPI00304693	-0.362	0.22785	2	1	0	1
2980	Desmoglein-1	IPI00025753	0.000	0.37049	1	0	1	0
2981	58 kDa protein	IPI00005806	0.362	0.21837	1	1	1	2
2982	Kinetochore-associated protein NSL1 homolog	IPI00306330	0.362	0.21837	0	1	2	1
2983	Isoform 1 of Beta-1-syntrophin	IPI00026059	0.000	0.37049	0	0	0	1
2984	Isoform 1 of N-alpha-acetyltransferase 40, NatD catalytic subunit	IPI00328847	-0.362	0.22785	0	2	0	1
2985	Melanoma-associated antigen B2	IPI00006726	-0.674	0.10802	2	2	0	0
2986	NEDD8 ultimate buster 1	IPI00157365	0.000	0.37049	1	2	0	2
2987	Isoform 1 of Nucleolar protein 7	IPI00007729	-0.362	0.22785	2	1	1	0
2988	Protein kinase C alpha type	IPI00385449	0.000	0.37049	0	0	1	0
2989	Keratin, type II cytoskeletal 6A	IPI00300725	0.000	0.37049	0	0	0	0
2990	Keratin, type II cuticular Hb4	IPI00300052	0.000	0.37049	0	0	0	0
2991	Isoform 1 of Serine/threonine-protein phosphatase 4 regulatory subunit 3B	IPI00414323	0.362	0.21837	0	0	0	2
2992	Pumilio homolog 1 (Drosophila), isoform CRA_c	IPI00032355	0.674	0.08856	0	0	3	1
2993	Isoform 3 of Prolyl 3-hydroxylase 1	IPI00045839	0.362	0.21837	0	0	0	2
2994	UPF0609 protein C4orf27	IPI00016532	-0.362	0.22785	0	2	0	0
2995	Isoform 2 of Late secretory pathway protein AVL9 homolog	IPI00022042	-0.362	0.22785	2	1	1	0
2996	Alba-like protein C9orf23	IPI00166873	0.000	0.37049	1	1	0	0
2997	Putative uncharacterized protein DKFZp686G0859	IPI00470477	0.674	0.08856	1	0	2	2
2998	Glutaredoxin-1	IPI00219025	-0.362	0.22785	2	0	0	0
2999	Carbonic anhydrase 1	IPI00215983	-0.674	0.10802	3	0	0	0
3000	Nuclear transcription factor Y subunit beta	IPI00013217	-0.362	0.22785	1	2	0	0
3001	Isoform 1 of Proteasomal ATPase-associated factor 1	IPI00743862	0.362	0.21837	0	0	2	0
3002	Ribosome biogenesis protein NSA2 homolog	IPI00007089	-0.362	0.22785	2	0	0	0
3003	Prostaglandin F2 receptor negative regulator	IPI00022048	0.000	0.37049	0	0	1	1
3004	Protein pelota homolog	IPI00106698	0.000	0.37049	1	1	0	0
3005	Syntaxin-12	IPI00329332	0.362	0.21837	0	0	2	0
3006	cDNA FLJ61655, highly similar to Phosphorylated CTD-interacting factor 1	IPI00014865	0.362	0.21837	0	0	0	2
3007	Short-chain specific acyl-CoA dehydrogenase, mitochondrial	IPI00027701	-0.362	0.22785	0	2	1	0
3008	Isoform 2 of Mannose-1-phosphate guanyltransferase beta	IPI00002496	0.362	0.21837	0	1	2	0
3009	Peroxisomal bifunctional enzyme	IPI00216164	0.362	0.21837	0	0	0	2
3010	UPF0552 protein C15orf38	IPI00074225	-0.362	0.22785	2	0	0	0
3011	Isoform 1 of Protein timeless homolog	IPI00335541	0.362	0.21837	0	0	2	0
3012	Isoform 1 of Protein LSM14 homolog B	IPI00032635	0.362	0.21837	0	0	2	0
3013	Isoform 2 of Dephospho-CoA kinase domain-containing protein	IPI00015737	-0.362	0.22785	2	0	0	0
3014	Threonyl-tRNA synthetase, mitochondrial	IPI00604527	0.000	0.37049	0	0	0	0
3015	Isoform 1 of Long-chain-fatty-acid--CoA ligase 5	IPI00008037	0.000	0.37049	0	0	0	0
3016	Nuclear envelope pore membrane protein POM 121C	IPI00032358	-0.362	0.22785	2	0	0	0
3017	My002 protein	IPI00023584	-0.362	0.22785	2	0	0	0
3018	Isoform 1 of Oxidoreductase HTATIP2	IPI00784029	-0.362	0.22785	2	0	0	0
