# Supplementary material for: Data supporting the identification of anti-metastatic drug and natural compound targets in isogenic colorectal cancer cells
Source: Data Brief. 2014 Nov 4;1:73–5. doi: 10.1016/j.dib.2014.10.005 (PMC4459770; doi:10.1016/j.dib.2014.10.005)
Supplement: Supplementary file 1 — Supplementary data [file mmc1.zip › CRC_Metastasis_DIB_Table03.pdf]

Table 3. List of proteins differentially expressed in SW620 by the treatment of sorafenib for 48hr. ([1]STN and p-value were acquired from PLGEM analysis, [2]Raw spectral counts from data compilation using ScaffoldTM software)

No.	Description	Accession number	STN[1]	p-Value[1]	Con_A[2]	Con_B[2]	SORA_A[2]	SORA_B[2]
1	ATP synthase subunit beta, mitochondrial	IPI00303476	5.158	0.00193	62	78	107	108
2	Isoform 2 of Filamin-A	IPI00302592	5.062	0.00193	146	140	180	193
3	Isoform 1 of Myosin-9	IPI00019502	5.040	0.00193	181	219	234	260
4	Isoform M1 of Pyruvate kinase isozymes M1/M2	IPI00220644	4.985	0.00193	157	194	189	252
5	Keratin, type II cytoskeletal 8	IPI00554648	3.966	0.00325	240	406	321	408
6	Neuroblast differentiation-associated protein AHNAK	IPI00021812	3.895	0.00336	75	66	80	117
7	Tubulin beta-2C chain	IPI00007752	3.586	0.00461	130	195	169	219
8	Actin, cytoplasmic 1	IPI00021439	3.470	0.00484	149	200	184	227
9	Peroxiredoxin-6	IPI00220301	3.454	0.00491	47	67	69	92
10	A-kinase anchor protein 12 isoform 2	IPI00217683	3.417	0.00503	7	10	22	26
11	Tubulin, beta	IPI00645452	3.344	0.00514	30	43	56	58
12	Isoform 1 of Heterogeneous nuclear ribonucleoprotein K	IPI00216049	3.144	0.00601	104	99	149	103
13	Aspartate aminotransferase, mitochondrial	IPI00018206	3.135	0.00612	30	49	44	74
14	Tubulin alpha-4A chain	IPI00007750	3.053	0.00688	205	244	235	272
15	Complement component 1 Q subcomponent-binding protein, mitochondrial	IPI00014230	2.976	0.00699	48	88	85	93
16	Putative annexin A2-like protein	IPI00334627	2.865	0.00733	30	54	58	62
17	retinol-binding protein 1 isoform a	IPI00219718	2.691	0.00794	23	44	41	58
18	Isoform alpha-enolase of Alpha-enolase	IPI00465248	2.664	0.00794	305	366	318	409
19	Isoform 1 of 60S ribosomal protein L11	IPI00376798	2.561	0.00835	10	13	29	18
20	T-complex protein 1 subunit delta	IPI00302927	2.433	0.00888	28	41	48	50
21	Isoform 2 of Splicing factor 3B subunit 3	IPI00179138	2.328	0.00915	3	6	9	18
22	Isoform 1 of Myoferlin	IPI00021048	2.267	0.00960	46	48	58	65
23	Profilin-1	IPI00216691	2.259	0.00968	90	139	117	148
24	cDNA FLJ60299, highly similar to Rab GDP dissociation inhibitor beta	IPI00031461	2.253	0.00968	31	30	40	47
25	EH domain-containing protein 1	IPI00017184	2.208	0.01005	14	12	20	27
26	Cofilin-1	IPI00012011	2.165	0.01028	86	157	122	156
27	59 kDa protein	IPI00302925	2.045	0.01183	36	43	56	48
28	TUBA1C protein	IPI00166768	1.947	0.01342	76	57	87	73
29	60S ribosomal protein L23	IPI00010153	1.926	0.01372	8	17	16	27
30	Galectin-1	IPI00219219	1.885	0.01429	20	24	32	32
31	40S ribosomal protein S8	IPI00216587	1.881	0.01436	14	14	24	22
32	Ubiquitin carboxyl-terminal hydrolase 7	IPI00003965	1.873	0.01444	11	11	16	23
33	Leucyl-tRNA synthetase, cytoplasmic	IPI00103994	1.858	0.01485	23	24	27	40
34	Trifunctional enzyme subunit alpha, mitochondrial	IPI00031522	1.814	0.01565	23	19	27	34
35	Glucose-6-phosphate isomerase	IPI00027497	1.802	0.01591	29	37	45	42
36	Tubulin beta-1 chain	IPI00006510	1.793	0.01614	20	15	28	25
37	Isoform 1 of Cullin-associated NEDD8-dissociated protein 1	IPI00100160	1.785	0.01625	31	25	27	49
38	Nucleoprotein TPR	IPI00742682	1.777	0.01644	35	35	42	49
39	Isoform 1 of Leukotriene A-4 hydrolase	IPI00219077	1.743	0.01693	16	15	19	29
40	Actin, aortic smooth muscle	IPI00008603	1.743	0.01693	28	48	32	65
41	Isoform 1 of Proteasome subunit alpha type-7	IPI00024175	1.720	0.01720	15	18	26	24
42	Phosphoglycerate mutase 2	IPI00218570	1.705	0.01758	0	0	8	7
43	Vacuolar protein sorting-associated protein 26A	IPI00411426	1.701	0.01769	3	7	10	13
44	Beta-actin-like protein 2	IPI00003269	1.692	0.01769	29	28	40	36
45	Isoform 1 of 6-phosphofructokinase, liver type	IPI00332371	1.684	0.01773	5	0	7	12
46	Isoform 1 of Filamin-C	IPI00178352	1.635	0.01845	8	16	15	24
47	Dihydrolipoyl dehydrogenase, mitochondrial	IPI00015911	1.635	0.01845	10	14	21	18
48	Serine hydroxymethyltransferase, mitochondrial	IPI00002520	1.626	0.01901	27	27	36	36
49	NCL protein	IPI00183526	1.602	0.01939	66	89	73	105
50	Citrate synthase, mitochondrial	IPI00025366	1.599	0.01943	27	31	32	44
51	Chloride intracellular channel protein 1	IPI00010896	1.567	0.02128	18	32	34	33
52	Isoform 2 of Formin-like protein 1	IPI00025202	1.556	0.02151	4	3	6	12
53	40S ribosomal protein S18	IPI00013296	1.546	0.02151	8	15	19	18
54	Tubulin beta-2A chain	IPI00013475	1.535	0.02162	5	12	18	12
55	Peroxiredoxin-1	IPI00000874	1.530	0.02189	28	42	46	42
56	RuvB-like 2	IPI00009104	1.530	0.02189	25	18	28	31
57	Isoform 2 of Triosephosphate isomerase	IPI00451401	1.514	0.02196	16	29	27	34
58	Elongation factor 2	IPI00186290	1.496	0.02226	79	125	98	129
59	Isoform 1 of Hexokinase-1	IPI00018246	1.484	0.02230	10	10	13	20
60	Eukaryotic translation initiation factor 2 subunit 2	IPI00021728	1.461	0.02347	6	9	11	16
61	Platelet-activating factor acetylhydrolase IB subunit beta	IPI00026546	1.459	0.02347	5	5	8	13
62	Isoform Long of Sodium/potassium-transporting ATPase subunit alpha-1	IPI00006482	1.448	0.02347	46	42	50	56
63	Isoform 1 of Adenylate kinase 2, mitochondrial	IPI00215901	1.432	0.02427	7	4	17	5
64	Fructose-bisphosphate aldolase	IPI00418262	1.427	0.02438	0	5	7	10
65	Isoform 1 of Vinculin	IPI00291175	1.414	0.02464	51	46	55	60
66	Fructose-bisphosphate aldolase A	IPI00465439	1.388	0.02559	21	29	27	38
67	14-3-3 protein epsilon	IPI00000816	1.379	0.02582	191	260	210	267
68	60S ribosomal protein L6	IPI00329389	1.376	0.02589	16	23	23	30
69	Isoform Long of Tight junction protein ZO-1	IPI00216219	1.363	0.02616	6	3	8	11
70	Endoplasmic reticulum resident protein 29	IPI00024911	1.362	0.02616	8	13	15	18
71	Putative uncharacterized protein ENSP00000350479	IPI00069693	1.348	0.02616	8	14	18	16
72	Elongation factor 1-alpha	IPI00025447	1.340	0.02653	78	105	107	96
73	cDNA FLJ59571, highly similar to Eukaryotic translation initiation factor 4gamma 2	IPI00015952	1.336	0.02672	5	5	9	11
74	cDNA FLJ78679, highly similar to Homo sapiens DEAD (Asp-Glu-Ala-Asp) box polypeptide 46 (DDX46)	IPI00329791	1.335	0.02699	12	11	19	16
75	Stomatin-like protein 2	IPI00334190	1.335	0.02699	11	12	19	16
76	Methionyl-tRNA synthetase, cytoplasmic	IPI00008240	1.322	0.02721	18	16	20	27
77	40S ribosomal protein S15a	IPI00221091	1.312	0.02737	6	11	15	13
78	Probable ATP-dependent RNA helicase DDX5	IPI00017617	1.304	0.02748	29	36	39	41
79	cDNA FLJ45400 fis, clone BRHIP3028570	IPI00151888	1.283	0.02767	2	2	4	8
80	Histone H2A.V	IPI00018278	1.259	0.02971	12	30	28	27
81	L-lactate dehydrogenase B chain	IPI00219217	1.256	0.02971	128	157	151	155
82	Endoplasmin	IPI00027230	1.219	0.03073	51	60	57	70
83	26S proteasome non-ATPase regulatory subunit 2	IPI00012268	1.215	0.03073	22	13	23	24
84	Cullin-1	IPI00014310	1.214	0.03081	7	9	13	13
85	Coatomer subunit delta variant 2	IPI00298520	1.211	0.03134	6	4	9	10
86	Niban-like protein 1	IPI00456750	1.211	0.03134	5	5	8	11
87	Isoform 1 of Myosin-10	IPI00397526	1.202	0.03137	22	29	28	36
88	Isoform 1 of Protein phosphatase 1 regulatory subunit 12A	IPI00183002	1.188	0.03273	6	5	9	11
89	40S ribosomal protein S5	IPI00008433	1.171	0.03315	24	17	28	25
90	SUMO-activating enzyme subunit 1	IPI00033130	1.167	0.03315	5	7	12	9

No.	Description	Accession number	STN[1]	p-Value[1]	Con_A[2]	Con_B[2]	SORA_A[2]	SORA_B[2]
91	Isoform 1 of Plectin-1	IPI00014898	1.167	0.03315	273	263	272	287
92	26S proteasome non-ATPase regulatory subunit 13 isoform 2	IPI00375380	1.167	0.03315	14	15	19	21
93	60S ribosomal protein L4	IPI00003918	1.167	0.03315	12	17	14	26
94	DnaJ homolog subfamily B member 11	IPI00008454	1.163	0.03319	3	4	6	9
95	Isoform Long of Splicing factor, proline- and glutamine-rich	IPI00010740	1.157	0.03330	9	11	14	16
96	Isoform 1 of Filamin-B	IPI00289334	1.152	0.03383	103	117	115	123
97	SERPINE1 mRNA binding protein 1, isoform CRA_d	IPI00410693	1.148	0.03387	5	8	9	13
98	Inorganic pyrophosphatase	IPI00015018	1.139	0.03387	37	49	50	50
99	Ribosomal protein S6 kinase alpha-6	IPI00007123	1.138	0.03406	0	2	4	7
100	Pre-mRNA-splicing factor ATP-dependent RNA helicase PRP16	IPI00294211	1.133	0.03451	9	13	15	17
101	Transitional endoplasmic reticulum ATPase	IPI00022774	1.114	0.03519	45	49	48	60
102	Isoform 1 of Nucleoside diphosphate kinase A	IPI00012048	1.112	0.03523	20	31	25	38
103	14-3-3 protein gamma	IPI00220642	1.111	0.03523	10	14	15	19
104	Isoform 1 of Insulin-like growth factor 2 mRNA-binding protein 2	IPI00179713	1.108	0.03564	2	7	9	8
105	ADP-ribosylation factor 1	IPI00215914	1.095	0.03610	119	149	130	156
106	Protein disulfide-isomerase	IPI00010796	1.092	0.03610	26	29	32	35
107	Gamma-enolase	IPI00216171	1.091	0.03629	61	73	67	82
108	CCAAT/enhancer-binding protein zeta	IPI00306723	1.085	0.03655	4	6	8	10
109	Laminin receptor-like protein LAMRL5	IPI00411639	1.083	0.03738	30	27	31	38
110	Structural maintenance of chromosomes protein 3	IPI00219420	1.077	0.03742	18	23	17	35
111	Lupus La protein	IPI00009032	1.071	0.03772	46	64	49	75
112	40S ribosomal protein S14	IPI00026271	1.065	0.03784	10	19	16	23
113	Developmentally-regulated GTP-binding protein 1	IPI00031836	1.064	0.03784	6	5	11	8
114	NADH dehydrogenase [ubiquinone] 1 beta subcomplex subunit 6	IPI00219385	1.058	0.03784	2	4	6	7
115	Isoform 2 of Heat shock protein HSP 90-alpha	IPI00382470	1.056	0.03787	114	194	142	184
116	ATP-dependent RNA helicase DDX1	IPI00293655	1.048	0.03814	18	13	15	26
117	Cleavage and polyadenylation specificity factor subunit 5	IPI00646917	1.048	0.03814	17	14	21	20
118	Eukaryotic translation initiation factor 3 subunit G	IPI00290460	1.045	0.03852	7	5	9	11
119	Enoyl-CoA hydratase, mitochondrial	IPI00024993	1.045	0.03852	5	7	11	9
120	Isoform 1 of 60S ribosome subunit biogenesis protein NIP7 homolog	IPI00007175	1.028	0.03961	2	5	7	7
121	Eukaryotic translation initiation factor 5A-2	IPI00006935	1.019	0.04033	14	21	25	20
122	40S ribosomal protein S10	IPI00008438	1.009	0.04139	24	52	33	55
123	tropomyosin alpha-1 chain isoform 2	IPI00000230	1.004	0.04147	7	17	17	16
124	Rho-associated protein kinase 1	IPI00022542	1.001	0.04165	4	4	7	8
125	Abhydrolase domain-containing protein 10, mitochondrial	IPI00020075	0.996	0.04188	9	6	12	11
126	Isoform 1 of Tryptophanyl-tRNA synthetase, cytoplasmic	IPI00295400	0.995	0.04188	12	13	14	20
127	Isoform 1 of Proteasome subunit beta type-8	IPI00000783	0.989	0.04203	2	2	8	0
128	LDLR chaperone MESD	IPI00399089	0.989	0.04203	0	0	5	5
129	Glycyl-tRNA synthetase	IPI00783097	0.987	0.04502	21	19	25	25
130	Isoform 1 of Protein-glutamine gamma-glutamyltransferase 2	IPI00294578	0.978	0.04581	4	5	5	11
131	Eukaryotic translation initiation factor 1A, Y-chromosomal	IPI00023004	0.976	0.04585	24	18	27	25
132	Neutral amino acid transporter B(0)	IPI00019472	0.960	0.04645	65	62	63	77
133	Serpin B6	IPI00413451	0.957	0.04645	10	8	11	15
134	1-phosphatidylinositol-4,5-bisphosphate phosphodiesterase beta-3	IPI00010400	0.957	0.04679	3	7	7	10
135	Calcium-binding mitochondrial carrier protein Aralar1	IPI00386271	0.957	0.04679	5	5	10	7
136	Isoform 1 of Electron transfer flavoprotein subunit beta	IPI00004902	0.955	0.04702	24	22	25	31
137	Heterogeneous nuclear ribonucleoprotein U-like protein 2	IPI00456887	0.954	0.04740	13	17	19	20
138	Calpain-2 catalytic subunit	IPI00289758	0.954	0.04740	15	15	19	20
139	Isoform 1 of Transmembrane and coiled-coil domain-containing protein 1	IPI00026111	0.950	0.04744	3	0	6	5
140	Thioredoxin	IPI00216298	0.935	0.04785	5	15	14	14
141	Asparagine synthetase [glutamine-hydrolyzing]	IPI00554777	0.918	0.04857	3	3	7	5
142	Isoform 1 of Serine/threonine-protein phosphatase 6 catalytic subunit	IPI00012970	0.918	0.04857	4	2	5	7
143	Isoform 2 of Extended synaptotagmin-2	IPI00409635	0.914	0.05050	17	19	19	26
144	Isoform Long of Ubiquitin carboxyl-terminal hydrolase 5	IPI00024664	0.905	0.05080	11	12	14	17
145	Vesicle transport protein GOT1B	IPI00007061	0.905	0.05080	6	7	8	12
146	Isoform 1 of Methionine adenosyltransferase 2 subunit beta	IPI00002324	0.905	0.05080	8	5	10	10
147	26S proteasome non-ATPase regulatory subunit 3	IPI00011603	0.891	0.05141	17	23	24	25
148	Parafibromin	IPI00300659	0.891	0.05144	6	8	13	8
149	Isoform 1 of Fatty aldehyde dehydrogenase	IPI00333619	0.890	0.05144	4	3	5	8
150	cDNA FLJ55574, highly similar to Calnexin	IPI00020984	0.888	0.05144	30	32	35	37
151	Isoform 1 of DNA-dependent protein kinase catalytic subunit	IPI00296337	0.887	0.05152	190	195	192	209
152	Alpha-actinin-4	IPI00013808	0.886	0.05152	83	91	87	100
153	60S acidic ribosomal protein P0	IPI00008530	0.874	0.05190	30	36	37	39
154	Isoform 1 of 3-hydroxyacyl-CoA dehydrogenase type-2	IPI00017726	0.867	0.05212	32	36	31	47
155	Cullin-5	IPI00216003	0.867	0.05216	3	5	6	8
156	Thimet oligopeptidase	IPI00549189	0.867	0.05216	5	3	8	6
157	Cleavage stimulation factor subunit 3	IPI00015195	0.867	0.05216	3	5	7	7
158	Isoform 1 of Putative ATP-dependent RNA helicase DHX30	IPI00411733	0.867	0.05216	4	4	7	7
159	Isoform 1 of Adenylyl cyclase-associated protein 1	IPI00008274	0.866	0.05330	16	29	21	33
160	Isoform 1 of Enhancer of mRNA-decapping protein 4	IPI00376317	0.865	0.05330	16	12	15	21
161	Probable phosphoglycerate mutase 4	IPI00374975	0.865	0.05349	5	11	10	13
162	Glutathione S-transferase omega-1	IPI00019755	0.865	0.05349	6	10	10	13
163	Isoform SERCA2A of Sarcoplasmic/endoplasmic reticulum calcium ATPase 2	IPI00177817	0.865	0.05349	11	5	7	16
164	Lamin-B receptor	IPI00292135	0.853	0.05379	7	10	13	11
165	N-acetyltransferase 10	IPI00300127	0.852	0.05386	26	22	29	28
166	Isoform 1 of U2-associated protein SR140	IPI00143753	0.846	0.05417	4	5	9	6
167	60S ribosomal protein L13	IPI00465361	0.846	0.05417	0	7	7	8
168	Isoform 2 of Septin-11	IPI00019376	0.846	0.05417	4	5	7	8
169	Isoform 3 of UDP-N-acetylglucosamine--peptide N-acetylglucosaminyltransferase 110 kDa subunit	IPI00005780	0.846	0.05417	7	2	8	7
170	Keratin-8-like protein 1	IPI00017870	0.844	0.05428	19	31	31	28
171	Isoform 1 of Catenin beta-1	IPI00017292	0.838	0.05454	14	18	15	25
172	Selenide, water dikinase 1	IPI00029056	0.837	0.05496	2	2	5	4
173	Serine/threonine-protein kinase PRP4 homolog	IPI00013721	0.837	0.05496	2	2	5	4
174	Isoform 1 of CUGBP Elav-like family member 1	IPI00034015	0.837	0.05496	0	0	5	4
175	Ras-related protein Rab-5A	IPI00023510	0.837	0.05496	0	2	5	4
176	SCY1-like protein 2	IPI00396218	0.837	0.05496	2	0	5	4
177	Interferon-induced 17 kDa protein	IPI00375631	0.837	0.05496	2	2	3	6
178	HSPA5 protein	IPI00003362	0.833	0.05598	51	65	55	72
179	cDNA FLJ14239 fis, clone NT2RP5003512, highly similar to Exportin-5	IPI00549861	0.827	0.05624	4	6	8	8
180	Mitochondrial ribonuclease P protein 1	IPI00099996	0.827	0.05624	6	4	9	7
181	Eukaryotic translation initiation factor 2 subunit 1	IPI00219678	0.826	0.05817	11	23	14	28
182	Nicotinamide phosphoribosyltransferase	IPI00018873	0.820	0.05851	19	16	20	23
183	Coatomer subunit gamma	IPI00783982	0.813	0.05870	11	10	13	15
184	Isoform 1 of Vesicle-associated membrane protein-associated protein B/C	IPI00006211	0.813	0.05870	9	12	10	18

No.	Description	Accession number	STN[1]	p-Value[1]	Con_A[2]	Con_B[2]	SORA_A[2]	SORA_B[2]
185	cDNA FLJ56425, highly similar to Very-long-chain specific acyl-CoAdehydrogenase, mitochondrial	IPI00028031	0.810	0.05870	29	30	28	40
186	cysteinyl-tRNA synthetase, cytoplasmic isoform c	IPI00027443	0.809	0.05870	19	18	18	27
187	Heat shock protein HSP 90-beta	IPI00414676	0.808	0.05878	78	105	86	109
188	Isoform 1 of Nuclear autoantigenic sperm protein	IPI00179953	0.804	0.05878	12	10	12	17
189	Periplakin	IPI00298057	0.804	0.05878	12	10	14	15
190	Isoform 1 of Sodium-coupled neutral amino acid transporter 2	IPI00410034	0.803	0.05915	3	2	3	7
191	Isoform 1 of UPF0424 protein C1orf128	IPI00015351	0.803	0.05915	3	2	6	4
192	Isoform 1 of Cullin-3	IPI00014312	0.803	0.05915	3	2	4	6
193	Seryl-tRNA synthetase, mitochondrial	IPI00328361	0.803	0.05915	2	3	6	4
194	X-ray repair cross-complementing protein 5	IPI00220834	0.797	0.05927	70	68	70	79
195	Malate dehydrogenase, mitochondrial	IPI00291006	0.787	0.06086	43	57	51	59
196	Non-POU domain-containing octamer-binding protein	IPI00304596	0.785	0.06086	31	36	37	39
197	Isoform 3 of Serine/threonine-protein phosphatase 2A activator	IPI00217296	0.781	0.06112	8	5	9	10
198	1,4-alpha-glucan-branching enzyme	IPI00296635	0.775	0.06138	3	3	5	6
199	Isoform 4 of Dipeptidyl peptidase 9	IPI00604483	0.775	0.06138	0	4	3	8
200	Bystin	IPI00328987	0.775	0.06138	3	3	5	6
201	Prenylcysteine oxidase 1	IPI00384280	0.775	0.06138	0	4	4	7
202	Isoform 1 of Glucosamine--fructose-6-phosphate aminotransferase [isomerizing] 1	IPI00217952	0.775	0.06138	3	3	6	5
203	Derlin-1	IPI00013271	0.775	0.06138	2	4	4	7
204	Putative uncharacterized protein ATP5J2	IPI00219291	0.775	0.06138	3	3	4	7
205	3-ketoacyl-CoA thiolase, mitochondrial	IPI00001539	0.775	0.06138	3	3	5	6
206	Isoform 3 of Core histone macro-H2A.1	IPI00059366	0.773	0.06199	14	12	19	14
207	Isoform 1 of Kinectin	IPI00328753	0.769	0.06199	9	5	10	10
208	Vesicle-fusing ATPase	IPI00006451	0.769	0.06199	7	7	10	10
209	Isoform 2 of Annexin A2	IPI00418169	0.768	0.06350	20	26	26	28
210	Isoform 2 of Nuclear mitotic apparatus protein 1	IPI00006196	0.763	0.06380	25	22	29	26
211	Isoform Short of Heterogeneous nuclear ribonucleoprotein U	IPI00479217	0.761	0.06380	33	43	40	45
212	Isoleucyl-tRNA synthetase, mitochondrial	IPI00017283	0.757	0.06422	7	8	6	15
213	Isoform 1 of N-acylneuraminate cytidylyltransferase	IPI00303158	0.757	0.06422	6	9	8	13
214	cDNA FLJ55586, highly similar to MMS19-like protein	IPI00154451	0.751	0.06448	4	3	5	7
215	Estradiol 17-beta-dehydrogenase 11	IPI00329598	0.751	0.06448	4	3	6	6
216	Ribosomal protein S6 kinase alpha-3	IPI00020898	0.751	0.06448	3	4	4	8
217	Isoform 1 of Serine/threonine-protein kinase WNK1	IPI00004472	0.751	0.06448	4	3	6	6
218	T-complex protein 1 subunit eta	IPI00018465	0.748	0.06448	56	66	57	75
219	Isoform 1 of Dynamin-2	IPI00033022	0.736	0.06626	9	8	11	12
220	Isoform 1 of AP-2 complex subunit beta	IPI00784156	0.736	0.06626	7	10	9	14
221	Proliferation-associated protein 2G4	IPI00299000	0.730	0.06626	16	17	19	21
222	Coatomer subunit gamma-2	IPI00002557	0.730	0.06634	5	3	6	7
223	Aminoacyl tRNA synthase complex-interacting multifunctional protein 1	IPI00006252	0.730	0.06634	5	3	7	6
224	Heterogeneous nuclear ribonucleoprotein H2	IPI00026230	0.730	0.06634	4	4	6	7
225	Acetyl-CoA acetyltransferase, mitochondrial	IPI00030363	0.725	0.06766	24	34	26	40
226	Isoform 1 of Heterogeneous nuclear ribonucleoprotein D0	IPI00028888	0.720	0.06800	14	21	20	22
227	60 kDa heat shock protein, mitochondrial	IPI00784154	0.717	0.06819	246	277	220	317
228	ADP-ribosylation factor 6	IPI00215920	0.716	0.06819	27	34	29	40
229	Histone H3.2	IPI00171611	0.712	0.06849	4	5	6	8
230	Malate dehydrogenase	IPI00916111	0.712	0.06849	5	4	7	7
231	Ras GTPase-activating-like protein IQGAP1	IPI00009342	0.706	0.07065	52	50	51	60
232	Plastin-3	IPI00216694	0.700	0.07102	12	9	13	14
233	Eukaryotic translation initiation factor 6	IPI00010105	0.700	0.07102	11	10	12	15
234	Solute carrier family 2, facilitated glucose transporter member 1	IPI00220194	0.700	0.07102	11	10	15	12
235	60S ribosomal protein L7a	IPI00299573	0.697	0.07102	16	24	20	27
236	Isoform 4 of Tubulin-specific chaperone D	IPI00030774	0.696	0.07129	5	5	5	10
237	5'-nucleotidase domain-containing protein 1	IPI00177965	0.696	0.07129	5	5	6	9
238	Isoform A of AP-1 complex subunit beta-1	IPI00328257	0.693	0.07155	22	19	19	29
239	Programmed cell death 6-interacting protein	IPI00246058	0.693	0.07155	23	18	23	25
240	Isoform 1 of Transportin-1	IPI00024364	0.693	0.07159	13	9	12	16
241	Isoform 1 of Dynamin-like 120 kDa protein, mitochondrial	IPI00006721	0.693	0.07159	11	11	14	14
242	Histone H4	IPI00453473	0.692	0.07257	60	179	98	152
243	Chromobox protein homolog 1	IPI00010320	0.682	0.07284	2	2	0	6
244	Programmed cell death protein 6	IPI00025277	0.682	0.07284	2	0	4	4
245	Isoform 2 of Calpastatin	IPI00220857	0.682	0.07284	0	0	5	3
246	Isoform 1 of Serine-protein kinase ATM	IPI00298306	0.682	0.07284	2	0	3	5
247	Isoform 1 of Protein LSM12 homolog	IPI00410324	0.682	0.07284	0	2	0	6
248	Isoform 1 of SEC23-interacting protein	IPI00026969	0.682	0.07284	2	2	4	4
249	ATP-dependent RNA helicase SUPV3L1, mitochondrial	IPI00412404	0.682	0.07284	0	0	5	3
250	Eukaryotic translation initiation factor 1	IPI00015077	0.682	0.07284	0	2	4	4
251	Isoform 3 of Glutaminase kidney isoform, mitochondrial	IPI00215687	0.678	0.08161	12	12	15	15
252	Isoform 1 of Alpha-aminoadipic semialdehyde dehydrogenase	IPI00221234	0.678	0.08161	12	12	12	18
253	Isoform B of Serine/threonine-protein kinase 24	IPI00002212	0.668	0.08206	6	6	9	8
254	Isoform 1 of Elongation factor G, mitochondrial	IPI00154473	0.668	0.08206	6	6	8	9
255	Keratin, type I cytoskeletal 19	IPI00479145	0.664	0.08248	116	163	124	166
256	60S ribosomal protein L27	IPI00219155	0.656	0.08274	7	6	11	7
257	Ubiquitin carboxyl-terminal hydrolase 14	IPI00219913	0.654	0.08293	14	14	18	16
258	Probable ATP-dependent RNA helicase DDX23	IPI00006725	0.652	0.08335	3	2	4	5
259	Propionyl-CoA carboxylase beta chain, mitochondrial	IPI00007247	0.652	0.08335	3	2	5	4
260	Tetratricopeptide repeat protein 35	IPI00014149	0.652	0.08335	2	3	4	5
261	28S ribosomal protein S30, mitochondrial	IPI00010278	0.652	0.08335	2	3	4	5
262	GrpE protein homolog 1, mitochondrial	IPI00029557	0.652	0.08335	2	3	4	5
263	Major vault protein	IPI00000105	0.652	0.08335	2	3	4	5
264	Perilipin-2	IPI00293307	0.652	0.08335	0	3	4	5
265	28S ribosomal protein S9, mitochondrial	IPI00641924	0.652	0.08335	3	0	5	4
266	Leukocyte elastase inhibitor	IPI00027444	0.648	0.08350	16	13	16	19
267	Cytosolic purine 5'-nucleotidase	IPI00029054	0.645	0.08361	9	5	9	10
268	Glutathione synthetase	IPI00010706	0.633	0.08516	17	15	20	18
269	Eukaryotic peptide chain release factor subunit 1	IPI00429191	0.628	0.08539	16	17	20	19
270	Probable ATP-dependent RNA helicase DDX52	IPI00032423	0.628	0.08539	2	4	5	5
271	Isoform 1 of Retinol dehydrogenase 11	IPI00339384	0.628	0.08539	3	3	0	8
272	Exocyst complex component 4	IPI00059279	0.628	0.08539	3	3	5	5
273	Isoform 1 of Translation initiation factor eIF-2B subunit delta	IPI00005979	0.628	0.08539	2	4	6	4
274	UDP-galactose-4-epimerase	IPI00030229	0.628	0.08539	3	3	5	5
275	60S ribosomal protein L7	IPI00030179	0.624	0.09079	13	21	17	23
276	Peptidyl-prolyl cis-trans isomerase A	IPI00419585	0.620	0.09117	63	106	85	93
277	Ubiquitin-like modifier activating enzyme 1	IPI00552452	0.617	0.09132	7	10	9	13
278	14-3-3 protein eta	IPI00216319	0.617	0.09132	7	10	9	13
279	Phosphatidylethanolamine-binding protein 1	IPI00219446	0.611	0.09185	14	23	21	22

No.	Description	Accession number	STN[1]	p-Value[1]	Con_A[2]	Con_B[2]	SORA_A[2]	SORA_B[2]
280	Proteasome activator complex subunit 1	IPI00479722	0.610	0.09185	29	39	37	38
281	Ribosomal L1 domain-containing protein 1	IPI00008708	0.608	0.09185	10	8	9	14
282	87 kDa protein	IPI00220365	0.608	0.09185	9	9	9	14
283	Eukaryotic translation initiation factor 3 subunit D	IPI00006181	0.608	0.09185	9	9	12	11
284	Reticulocalbin-1	IPI00015842	0.608	0.09185	4	3	6	5
285	Golgi-specific brefeldin A-resistance guanine nucleotide exchange factor 1	IPI00021954	0.608	0.09185	3	4	6	5
286	cDNA FLJ56357, highly similar to Homo sapiens apolipoprotein A-I binding protein (APOA1BP), mRNA	IPI00168479	0.608	0.09185	4	3	5	6
287	Isoform 2 of Putative methyltransferase NSUN5	IPI00101659	0.608	0.09185	2	5	3	8
288	26S protease regulatory subunit S10B	IPI00021926	0.601	0.09230	9	10	15	9
289	Heterogeneous nuclear ribonucleoprotein C-like 1	IPI00027569	0.599	0.09253	21	19	23	23
290	Isoform 1 of Myosin-Ib	IPI00376344	0.593	0.09272	8	12	9	16
291	Transmembrane protein 43	IPI00301280	0.591	0.09329	4	4	7	5
292	Isoform 1 of RuvB-like 1	IPI00021187	0.588	0.09775	22	21	23	26
293	Proteasome subunit alpha type-5	IPI00291922	0.588	0.09775	19	24	18	31
294	26S proteasome non-ATPase regulatory subunit 12	IPI00185374	0.585	0.09813	21	23	26	24
295	cDNA FLJ45706 fis, clone FEBRA2028457, highly similar to Nucleolin	IPI00444262	0.578	0.09899	41	43	45	46
296	Isoform 1 of 26S proteasome non-ATPase regulatory subunit 1	IPI00299608	0.575	0.09899	24	23	26	27
297	Isoleucyl-tRNA synthetase	IPI00514082	0.575	0.09899	4	5	4	9
298	Isoform Short of NADPH:adrenodoxin oxidoreductase, mitochondrial	IPI00026958	0.575	0.09899	4	5	6	7
299	Radixin, isoform CRA_a	IPI00017367	0.572	0.09915	24	24	26	28
300	Isoform 3 of LIM domain only protein 7	IPI00291802	0.562	0.10002	12	13	12	18
301	Ribosome biogenesis protein BRX1 homolog	IPI00181728	0.562	0.10020	4	6	7	7
302	Isoform 1 of E3 UFM1-protein ligase 1	IPI00844000	0.562	0.10020	5	5	7	7
303	Isoform 1 of Bcl-2-associated transcription factor 1	IPI00006079	0.562	0.10020	4	6	8	6
304	Isoform 2 of Serine-protein kinase ATM	IPI00289986	0.562	0.10020	6	4	8	6
305	Bifunctional purine biosynthesis protein PURH	IPI00289499	0.561	0.10357	45	50	44	58
306	Isoleucyl-tRNA synthetase, cytoplasmic	IPI00644127	0.558	0.10357	22	31	21	38
307	Signal recognition particle 54 kDa protein	IPI00009822	0.552	0.10391	13	14	11	21
308	cDNA FLJ60124, highly similar to Mitochondrial dicarboxylate carrier	IPI00005537	0.550	0.10448	5	6	6	9
309	Isoform Short of Glycylpeptide N-tetradecanoyltransferase 1	IPI00218830	0.550	0.10448	5	6	6	9
310	Chloride intracellular channel protein 4	IPI00001960	0.550	0.10448	6	5	8	7
311	Protein DJ-1	IPI00298547	0.547	0.10451	15	13	22	11
312	Rho GTPase-activating protein 1	IPI00020567	0.539	0.10504	6	6	6	10
313	Nucleolar complex protein 3 homolog	IPI00102815	0.539	0.10504	6	6	6	10
314	cDNA FLJ14048 fis, clone HEMBA1006650, weakly similar to ARP2/3 COMPLEX 20 KD SUBUNIT	IPI00386354	0.539	0.10504	3	9	5	11
315	Mitochondrial glutamate carrier 1	IPI00003004	0.539	0.10504	3	9	8	8
316	Isoform GTBP-alt of DNA mismatch repair protein Msh6	IPI00106847	0.534	0.10807	14	17	18	18
317	NADH dehydrogenase [ubiquinone] 1 alpha subcomplex subunit 9, mitochondrial	IPI00003968	0.529	0.10833	15	17	17	20
318	Isoform M2 of Pyruvate kinase isozymes M1/M2	IPI00479186	0.526	0.10848	50	72	59	70
319	E3 SUMO-protein ligase RanBP2	IPI00221325	0.525	0.10848	15	18	18	20
320	Translin	IPI00018768	0.525	0.10848	15	18	17	21
321	Alpha-actinin-1	IPI00013508	0.525	0.10852	156	163	160	168
322	H/ACA ribonucleoprotein complex subunit 4	IPI00221394	0.521	0.10852	2	2	2	5
323	Uroporphyrinogen decarboxylase	IPI00301489	0.521	0.10852	0	0	4	3
324	Aminopeptidase B	IPI00642211	0.521	0.10852	0	2	3	4
325	Isoform 1 of Phosphatidylinositol glycan anchor biosynthesis class U protein	IPI00026044	0.521	0.10852	2	2	5	0
326	Guanine nucleotide-binding protein G(I)/G(S)/G(T) subunit beta-2	IPI00003348	0.521	0.10852	2	2	5	2
327	Isoform 1 of Calcium-binding mitochondrial carrier protein SCaMC-1	IPI00337494	0.521	0.10852	0	2	4	3
328	RNA methyltransferase-like protein 1	IPI00335589	0.521	0.10852	2	2	2	5
329	Isoform A of Protein CutA	IPI00034319	0.521	0.10852	2	2	3	4
330	Isoform 1 of 28S ribosomal protein S35, mitochondrial	IPI00073779	0.521	0.10852	2	0	3	4
331	Isoform 1 of Rab3 GTPase-activating protein catalytic subunit	IPI00014235	0.521	0.10852	2	2	4	3
332	Astrocytic phosphoprotein PEA-15	IPI00014850	0.521	0.10852	2	2	3	4
333	Isoform 1 of Secretory carrier-associated membrane protein 3	IPI00306382	0.521	0.10852	0	0	4	3
334	Isoform 1 of Ubiquitin-protein ligase E3C	IPI00604464	0.521	0.10852	2	0	3	4
335	D-dopachrome decarboxylase	IPI00293867	0.521	0.10852	0	2	0	5
336	Retinol dehydrogenase 14	IPI00177940	0.521	0.10852	2	2	0	5
337	ATP synthase mitochondrial F1 complex assembly factor 2	IPI00296999	0.521	0.10852	2	2	4	3
338	Tetratricopeptide repeat protein 1	IPI00016912	0.521	0.10852	2	2	3	4
339	Isoform 1 of GTP-binding protein 10	IPI00167638	0.521	0.10852	0	2	4	3
340	BRISC complex subunit Abro1	IPI00299517	0.521	0.10852	0	0	5	0
341	Isoform 3 of Protein LAS1 homolog	IPI00009917	0.521	0.10852	2	0	3	4
342	AP-1 complex subunit gamma-1 isoform a	IPI00293396	0.520	0.11105	7	7	7	11
343	Isoform 1AB of Catenin delta-1	IPI00182469	0.520	0.11105	6	8	7	11
344	Protein DEK	IPI00020021	0.520	0.11105	7	7	9	9
345	Isocitrate dehydrogenase 3, beta subunit isoform a precursor	IPI00304417	0.520	0.11105	7	7	10	8
346	Exportin-1	IPI00298961	0.517	0.11396	39	33	36	42
347	Lon protease homolog, mitochondrial	IPI00005158	0.514	0.11400	17	19	20	21
348	Isoform 1 of Inorganic pyrophosphatase 2, mitochondrial	IPI00301109	0.512	0.11438	7	8	7	12
349	Isoform 1 of Apolipoprotein O	IPI00042580	0.504	0.11487	8	8	9	11
350	40S ribosomal protein S16	IPI00221092	0.501	0.11687	16	24	22	23
351	UPF0687 protein C20orf27	IPI00101095	0.497	0.11718	3	0	4	4
352	DNA polymerase subunit gamma-1	IPI00004317	0.497	0.11718	0	3	4	4
353	Sorting and assembly machinery component 50 homolog	IPI00412713	0.497	0.11718	2	3	5	3
354	Protein BUD31 homolog	IPI00013180	0.497	0.11718	0	3	3	5
355	Isoform 2 of Heme-binding protein 2	IPI00003799	0.497	0.11718	3	2	5	3
356	NADP-dependent malic enzyme, mitochondrial	IPI00003970	0.497	0.11718	0	3	0	6
357	Isoform 1 of HEAT repeat-containing protein 3	IPI00100984	0.497	0.11718	3	0	4	4
358	39S ribosomal protein L20, mitochondrial	IPI00013706	0.497	0.11718	3	2	5	3
359	Isoform 1 of Autophagy-related protein 3	IPI00022254	0.497	0.11718	0	3	3	5
360	erlin-1	IPI00007940	0.497	0.11718	2	3	4	4
361	Protein transport protein Sec24C	IPI00024661	0.497	0.11718	10	7	9	12
362	29 kDa protein	IPI00453476	0.490	0.11729	9	9	12	10
363	60S ribosomal protein L21	IPI00247583	0.490	0.11729	10	8	11	11
364	Poly(rC)-binding protein 1	IPI00016610	0.490	0.11729	10	8	12	10
365	Isoform 1 of Dipeptidyl peptidase 3	IPI00020672	0.483	0.11895	23	23	25	26
366	Translocon-associated protein subunit delta precursor	IPI00019385	0.483	0.11929	8	11	12	11
367	Hsc70-interacting protein	IPI00032826	0.483	0.11929	8	11	12	11
368	Probable ATP-dependent RNA helicase DDX47	IPI00023972	0.483	0.11929	11	8	11	12
369	GTP-binding nuclear protein Ran	IPI00643041	0.481	0.11929	20	27	25	27
370	Isoform 1 of Apoptosis-inducing factor 1, mitochondrial	IPI00000690	0.478	0.11933	26	22	21	32
371	Small nuclear ribonucleoprotein Sm D3	IPI00017964	0.478	0.11944	2	4	5	4
372	cDNA FLJ59712, highly similar to Golgi reassembly-stacking protein 2	IPI00743931	0.478	0.11944	3	3	3	6
373	lanosterol 14-alpha demethylase isoform 1	IPI00295772	0.478	0.11944	2	4	5	4

No.	Description	Accession number	STN[1]	p-Value[1]	Con_A[2]	Con_B[2]	SORA_A[2]	SORA_B[2]
374	6-phosphogluconolactonase	IPI00029997	0.477	0.12080	7	13	11	13
375	Polyribonucleotide nucleotidyltransferase 1, mitochondrial	IPI00744711	0.477	0.12080	11	9	11	13
376	cDNA FLJ25678 fis, clone TST04067, highly similar to PURINE NUCLEOSIDE PHOSPHORYLASE	IPI00017672	0.473	0.12201	22	28	28	27
377	Proteasome subunit alpha type-6	IPI00029623	0.472	0.12205	9	12	11	14
378	Electron transfer flavoprotein subunit alpha, mitochondrial	IPI00010810	0.471	0.12205	17	34	25	31
379	Isoform 1 of Protein SET	IPI00072377	0.471	0.12205	23	28	28	28
380	Isoform 1 of Cysteine and histidine-rich domain-containing protein 1	IPI00015897	0.466	0.12217	13	9	10	16
381	Small nuclear ribonucleoprotein Sm D2	IPI00017963	0.466	0.12217	9	13	13	13
382	Activity-dependent neuroprotector homeobox protein	IPI00022215	0.462	0.12319	3	4	5	5
383	RNA-binding protein 28	IPI00304187	0.462	0.12319	5	2	3	7
384	Protein SCO1 homolog, mitochondrial	IPI00027233	0.462	0.12319	4	3	5	5
385	cDNA FLJ61739, highly similar to Serine/arginine repetitive matrix protein 1	IPI00328293	0.462	0.12319	3	4	4	6
386	Isoform 1a of Oxysterol-binding protein-related protein 3	IPI00023555	0.462	0.12319	4	3	6	4
387	Isoform 1 of Mitochondrial antiviral-signaling protein	IPI00020719	0.462	0.12319	3	4	5	5
388	Negative elongation factor B	IPI00103483	0.462	0.12319	4	3	5	5
389	Isoform 1 of Regulator of nonsense transcripts 3B	IPI00023409	0.462	0.12319	3	4	6	4
390	Glucosamine-6-phosphate isomerase 1	IPI00009305	0.461	0.12511	12	11	13	14
391	Isoform 1 of Protein diaphanous homolog 1	IPI00852685	0.457	0.12511	12	12	13	15
392	ATP synthase subunit alpha, mitochondrial	IPI00440493	0.454	0.12579	55	64	55	70
393	26S proteasome non-ATPase regulatory subunit 7	IPI00019927	0.452	0.12579	11	14	10	19
394	DNA-directed RNA polymerase II subunit RPB2	IPI00027808	0.452	0.12579	14	11	13	16
395	Hepatoma-derived growth factor	IPI00020956	0.452	0.12579	13	12	15	14
396	Isoform 1 of N-alpha-acetyltransferase 25, NatB auxiliary subunit	IPI00025890	0.448	0.12598	4	4	5	6
397	Isoform 1 of Large proline-rich protein BAT2	IPI00010700	0.448	0.12598	4	4	6	5
398	Biliverdin reductase A	IPI00294158	0.448	0.12598	4	4	5	6
399	Isoform 1 of Nucleolar protein 6	IPI00152890	0.448	0.12598	4	4	7	4
400	Isoform 2 of CDK5 regulatory subunit-associated protein 3	IPI00018780	0.448	0.12598	4	4	4	7
401	Polymerase delta-interacting protein 2	IPI00165506	0.448	0.12598	2	6	3	8
402	Isoform 1 of STE20-like serine/threonine-protein kinase	IPI00022827	0.448	0.12598	4	4	5	6
403	UPF0368 protein Cxorf26	IPI00107104	0.448	0.12598	2	6	5	6
404	F-actin-capping protein subunit alpha-2	IPI00026182	0.448	0.12598	2	6	3	8
405	Protein transport protein Sec23B	IPI00017376	0.448	0.12598	3	5	5	6
406	similar to RAN binding protein 1	IPI00399212	0.448	0.12598	4	4	5	6
407	Sorting nexin-2	IPI00299095	0.448	0.12598	4	4	6	5
408	Vasodilator-stimulated phosphoprotein	IPI00301058	0.448	0.12598	4	4	7	4
409	HDCMD34P	IPI00001672	0.448	0.12598	6	2	5	6
410	Ribose-phosphate pyrophosphokinase 1	IPI00219616	0.448	0.12712	11	15	18	12
411	Isoform 3 of Adenylate kinase 2, mitochondrial	IPI00172460	0.443	0.12765	14	13	15	16
412	Cathepsin D	IPI00011229	0.443	0.12765	13	14	15	16
413	14-3-3 protein zeta/delta	IPI00021263	0.438	0.12829	21	47	29	44
414	Moesin	IPI00219365	0.438	0.12829	32	36	37	36
415	Isoform 1 of Mitochondrial import receptor subunit TOM40 homolog	IPI00014053	0.436	0.12829	7	2	7	5
416	Eukaryotic translation initiation factor 2A	IPI00012462	0.436	0.12829	5	4	5	7
417	Isoform 1 of Protein virilizer homolog	IPI00036742	0.436	0.12829	5	4	6	6
418	Protein phosphatase 1F	IPI00291412	0.436	0.12829	5	4	4	8
419	Serine/threonine-protein phosphatase PP1-beta catalytic subunit	IPI00218236	0.436	0.12829	4	5	6	6
420	Nucleolar protein 58	IPI00006379	0.432	0.12927	16	14	15	19
421	Isoform ASF-1 of Splicing factor, arginine/serine-rich 1	IPI00215884	0.431	0.12995	25	47	34	43
422	Isoform 1 of RNA-binding protein 25	IPI00004273	0.426	0.13007	5	5	7	6
423	39S ribosomal protein L28, mitochondrial	IPI00172594	0.426	0.13007	6	4	9	4
424	Isoform 1 of Tensin-3	IPI00658152	0.426	0.13007	7	3	5	8
425	Isoform 2 of Cytosolic non-specific dipeptidase	IPI00165579	0.426	0.13007	6	4	7	6
426	Isoform 1 of Ubiquitin-conjugating enzyme E2 variant 1	IPI00019599	0.426	0.13007	4	6	6	7
427	Signal recognition particle receptor subunit beta	IPI00295098	0.422	0.13226	17	16	19	18
428	cDNA FLJ59739, highly similar to Protein transport protein Sec61 subunit alpha isoform 1	IPI00218466	0.416	0.13286	7	4	6	8
429	Mitochondrial-processing peptidase subunit alpha	IPI00166749	0.416	0.13286	5	6	7	7
430	Isoform 1 of 2',5'-phosphodiesterase 12	IPI00174390	0.416	0.13286	6	5	6	8
431	Probable fructose-2,6-bisphosphatase TIGAR	IPI00006907	0.416	0.13286	5	6	7	7
432	Guanine nucleotide-binding protein subunit alpha-13	IPI00290928	0.416	0.13286	6	5	7	7
433	Twinfilin-2	IPI00550917	0.416	0.13286	5	6	6	8
434	Ephrin type-A receptor 2	IPI00021267	0.408	0.13403	6	6	8	7
435	NADH dehydrogenase [ubiquinone] 1 alpha subcomplex subunit 10, mitochondrial	IPI00029561	0.408	0.13403	6	6	7	8
436	Isoform 1 of 26S protease regulatory subunit 6B	IPI00020042	0.407	0.13517	20	18	17	25
437	Isoform 2 of 3-hydroxyisobutyryl-CoA hydrolase, mitochondrial	IPI00377161	0.400	0.13581	7	6	9	7
438	Isoform Long of 14-3-3 protein beta/alpha	IPI00216318	0.394	0.13717	17	26	21	26
439	60S ribosomal protein L15	IPI00470528	0.392	0.13834	18	26	24	24
440	Flap endonuclease 1	IPI00026215	0.387	0.13880	8	7	7	11
441	60S ribosomal protein L38	IPI00215790	0.387	0.13880	7	8	10	8
442	Putative uncharacterized protein DKFZp451D234	IPI00031583	0.387	0.13880	8	7	8	10
443	DNA topoisomerase 1	IPI00413611	0.387	0.13880	8	7	8	10
444	Splicing factor 3A subunit 1	IPI00017451	0.387	0.13880	9	6	7	11
445	28S ribosomal protein S22, mitochondrial	IPI00013146	0.387	0.13880	5	10	9	9
446	Catalase	IPI00465436	0.387	0.13880	7	8	10	8
447	UPF0160 protein MYG1, mitochondrial	IPI00029444	0.387	0.13880	8	7	10	8
448	cDNA FLJ36192 fis, clone TESTI2027450, highly similar to Eukaryotic translation initiation factor 3	IPI00654777	0.386	0.13925	22	25	23	28
449	14-3-3 protein theta	IPI00018146	0.386	0.13925	17	30	22	29
450	Putative uncharacterized protein NOP2	IPI00294891	0.380	0.13959	6	10	6	13
451	Ribonuclease P protein subunit p30	IPI00019196	0.380	0.13959	10	6	11	8
452	Very long-chain acyl-CoA synthetase	IPI00024787	0.380	0.13959	8	8	9	10
453	Isoform 2 of Suppressor of G2 allele of SKP1 homolog	IPI00791573	0.380	0.13959	7	9	8	11
454	pyruvate dehydrogenase E1 alpha 1 isoform 2 precursor	IPI00306301	0.380	0.13959	8	8	11	8
455	Isoform 1 of Phosphatidylinositol transfer protein beta isoform	IPI00334907	0.375	0.14069	9	8	9	11
456	Histone H2B type 1-L	IPI00018534	0.370	0.14163	5	13	11	10
457	Glutaredoxin-3	IPI00008552	0.370	0.14163	9	9	11	10
458	Annexin A3	IPI00024095	0.369	0.14288	25	31	28	32
459	Alpha-centractin	IPI00029468	0.365	0.14295	9	10	12	10
460	Isoform 2 of Cytoplasmic FMR1-interacting protein 1	IPI00550212	0.360	0.14348	9	11	10	13
461	Cytochrome b-c1 complex subunit 1, mitochondrial	IPI00013847	0.360	0.14348	13	7	8	15
462	Tubulin beta-3 chain	IPI00013683	0.360	0.14348	11	9	9	14
463	Heterogeneous nuclear ribonucleoprotein H	IPI00013881	0.356	0.14507	8	13	9	15
464	tRNA (cytosine-5-)-methyltransferase NSUN2	IPI00306369	0.356	0.14507	9	12	14	10
465	cDNA FLJ53927, highly similar to Beta-hexosaminidase alpha chain	IPI00027851	0.355	0.14571	0	2	3	3
466	Isoform Membrane-bound of Catechol O-methyltransferase	IPI00011284	0.355	0.14571	2	2	0	4
467	Isoform 1 of Chromodomain-helicase-DNA-binding protein 1	IPI00297851	0.355	0.14571	2	0	3	3

No.	Description	Accession number	STN[1]	p-Value[1]	Con_A[2]	Con_B[2]	SORA_A[2]	SORA_B[2]
468	Putative uncharacterized protein DKFZp313O211	IPI00552186	0.355	0.14571	2	0	3	3
469	FAST kinase domain-containing protein 5	IPI00414973	0.355	0.14571	2	0	0	4
470	Isoform 1 of Spermatid perinuclear RNA-binding protein	IPI00169430	0.355	0.14571	2	2	2	4
471	Eukaryotic translation initiation factor 3 subunit J	IPI00290461	0.355	0.14571	2	2	3	3
472	Small glutamine-rich tetratricopeptide repeat-containing protein alpha	IPI00013949	0.355	0.14571	2	2	3	3
473	Isoform 1 of Caseinolytic peptidase B protein homolog	IPI00006615	0.355	0.14571	2	2	3	3
474	Isoform 3 of HEAT repeat-containing protein 5B	IPI00333696	0.355	0.14571	0	2	3	3
475	Serine/threonine-protein phosphatase 2A 65 kDa regulatory subunit A alpha isoform	IPI00554737	0.355	0.14571	2	2	3	3
476	Isoform 1 of Insulin-like growth factor 2 mRNA-binding protein 3	IPI00658000	0.355	0.14571	2	2	2	4
477	Isoform 2 of cAMP-dependent protein kinase catalytic subunit alpha	IPI00217960	0.355	0.14571	2	2	0	4
478	Brefeldin A-inhibited guanine nucleotide-exchange protein 1	IPI00002188	0.355	0.14571	0	2	3	3
479	Aspartyl-tRNA synthetase, mitochondrial	IPI00100460	0.355	0.14571	2	2	2	4
480	Isoform 2 of NudC domain-containing protein 1	IPI00306398	0.355	0.14571	0	0	3	3
481	Actin-related protein 2/3 complex subunit 5-like protein	IPI00414554	0.355	0.14571	2	2	3	3
482	Protein TFG	IPI00294619	0.355	0.14571	0	0	3	3
483	Fragile X mental retardation syndrome-related protein 2	IPI00016250	0.355	0.14571	2	0	3	3
484	Myosin-11	IPI00020501	0.355	0.14571	2	2	3	3
485	Nucleolar complex protein 2 homolog	IPI00411886	0.355	0.14571	2	0	2	4
486	Isoform 1 of Putative splicing factor, arginine/serine-rich 14	IPI00158020	0.355	0.14571	0	0	2	4
487	Isoform 1 of Elongation factor Tu GTP-binding domain-containing protein 1	IPI00293026	0.355	0.14571	2	2	3	3
488	Isoform 1 of HCLS1-associated protein X-1	IPI00010440	0.355	0.14571	0	2	4	2
489	Isoform 3 of DNA repair protein RAD50	IPI00107531	0.355	0.14571	0	2	4	2
490	HIV Tat-specific factor 1	IPI00013788	0.355	0.14571	2	0	2	4
491	U3 small nucleolar RNA-interacting protein 2	IPI00217862	0.355	0.14571	0	0	3	3
492	Fascin	IPI00163187	0.355	0.14571	0	2	0	4
493	Protein FAM50B	IPI00015912	0.355	0.14571	0	0	0	4
494	Histone H1x	IPI00021924	0.355	0.14571	2	2	4	2
495	Glycylpeptide N-tetradecanoyltransferase 2	IPI00030223	0.355	0.14571	0	0	3	3
496	Isoform 3 of Pre-mRNA 3'-end-processing factor FIP1	IPI00008449	0.355	0.14571	2	2	3	3
497	RER1 protein	IPI00005728	0.355	0.14571	0	0	0	4
498	Isoform 1 of Probable threonyl-tRNA synthetase 2, cytoplasmic	IPI00328082	0.355	0.14571	0	0	3	3
499	tRNA (guanine-N(1)-)-methyltransferase	IPI00455268	0.355	0.14571	0	0	4	2
500	Isoform 1 of 28S ribosomal protein S5, mitochondrial	IPI00169400	0.355	0.14571	0	2	4	2
501	Isoform 2 of Protein diaphanous homolog 2	IPI00514075	0.355	0.14571	0	0	2	4
502	Isoform 1 of HAUS augmin-like complex subunit 2	IPI00018198	0.355	0.14571	0	0	3	3
503	Isoform 1 of U4/U6 small nuclear ribonucleoprotein Prp3	IPI00005861	0.355	0.14571	0	0	4	0
504	Transmembrane protein 49	IPI00062469	0.355	0.14571	2	2	0	4
505	Isoform 1 of Required for meiotic nuclear division protein 1 homolog	IPI00329591	0.355	0.14571	0	0	2	4
506	ATP synthase subunit g, mitochondrial	IPI00027448	0.355	0.14571	0	0	2	4
507	Ubiquitin carboxyl-terminal hydrolase 13	IPI00024401	0.355	0.14571	2	2	0	4
508	THUMP domain-containing protein 3	IPI00306127	0.355	0.14571	0	0	4	0
509	NADH dehydrogenase [ubiquinone] iron-sulfur protein 3, mitochondrial	IPI00025796	0.352	0.16851	10	12	10	15
510	Aconitate hydratase, mitochondrial	IPI00017855	0.348	0.16956	13	10	9	17
511	Isoform 1 of Platelet-activating factor acetylhydrolase IB subunit alpha	IPI00218728	0.344	0.17021	14	10	12	15
512	2-oxoglutarate dehydrogenase, mitochondrial	IPI00098902	0.341	0.17051	9	16	11	17
513	Splicing factor 3A subunit 3	IPI00029764	0.341	0.17051	11	14	13	15
514	Ras-related protein Rab-2A	IPI00031169	0.341	0.17051	13	12	15	13
515	histone deacetylase complex subunit SAP18	IPI00011698	0.338	0.17104	0	3	4	3
516	Protein S100-A6	IPI00027463	0.338	0.17104	3	0	5	0
517	Isoform 1 of Armadillo repeat-containing protein 10	IPI00166394	0.338	0.17104	2	3	5	0
518	Sedoheptulokinase	IPI00005914	0.338	0.17104	0	3	2	5
519	Pre-mRNA-processing factor 6	IPI00305068	0.338	0.17104	0	3	3	4
520	DNA primase small subunit	IPI00027704	0.338	0.17104	3	2	2	5
521	Aflatoxin B1 aldehyde reductase member 2	IPI00305978	0.338	0.17104	2	3	2	5
522	Signal peptidase complex subunit 3	IPI00300299	0.338	0.17104	2	3	4	3
523	Isoform 2 of NADH dehydrogenase [ubiquinone] flavoprotein 3, mitochondrial	IPI00291016	0.338	0.17104	3	0	0	5
524	Lipoamide acyltransferase component of branched-chain alpha-keto acid dehydrogenase complex	IPI00003944	0.338	0.17104	3	2	3	4
525	F-box only protein 2	IPI00007087	0.338	0.17104	0	3	3	4
526	Succinyl-CoA ligase [GDP-forming] subunit alpha, mitochondrial	IPI00872762	0.338	0.17104	0	3	0	5
527	Uncharacterized protein C7orf50	IPI00031651	0.338	0.17104	2	3	3	4
528	Putative uncharacterized protein DKFZp781K1356	IPI00412545	0.338	0.17104	0	3	3	4
529	Isoform B of Arfaptin-1	IPI00021258	0.338	0.17104	2	3	3	4
530	Tryptophanyl-tRNA synthetase, mitochondrial	IPI00025050	0.338	0.17104	0	3	3	4
531	Isoform 2 of Neuropathy target esterase	IPI00217600	0.338	0.17104	3	0	3	4
532	60S acidic ribosomal protein P2	IPI00008529	0.337	0.17104	12	14	13	16
533	Isoform Long of Glucose-6-phosphate 1-dehydrogenase	IPI00216008	0.331	0.17130	13	15	15	16
534	Putative pre-mRNA-splicing factor ATP-dependent RNA helicase DHX15	IPI00396435	0.330	0.17142	44	42	39	51
535	GDP-mannose 4,6 dehydratase	IPI00030207	0.328	0.17157	15	14	16	16
536	Putative adenosylhomocysteinase 3	IPI00101645	0.324	0.17282	4	2	4	4
537	Isoform 1 of Serine/threonine-protein phosphatase PGAM5, mitochondrial	IPI00788907	0.324	0.17282	3	3	4	4
538	Scavenger mRNA-decapping enzyme DcpS	IPI00335385	0.324	0.17282	3	3	5	3
539	Isoform CSBP2 of Mitogen-activated protein kinase 14	IPI00002857	0.324	0.17282	3	3	5	3
540	Telomeric repeat-binding factor 2-interacting protein 1	IPI00008961	0.324	0.17282	3	3	5	3
541	Isoform 1 of DNA-directed RNA polymerases I and III subunit RPAC1	IPI00005179	0.324	0.17282	2	4	4	4
542	SH3 domain-binding glutamic acid-rich-like protein	IPI00025318	0.324	0.17282	2	4	4	4
543	Serine/threonine-protein phosphatase 5	IPI00019812	0.324	0.17282	3	3	3	5
544	Isoform 3 of Protein transport protein Sec31A	IPI00305152	0.324	0.17282	3	3	3	5
545	Nuclear pore complex protein Nup50	IPI00026940	0.324	0.17282	2	4	4	4
546	Acyl-coenzyme A thioesterase 13	IPI00020530	0.324	0.17282	0	4	4	4
547	14 kDa protein	IPI00179589	0.324	0.17282	3	3	4	4
548	Phosphoribosylformylglycinamidine synthase	IPI00004534	0.318	0.18707	20	13	17	19
549	Isoform 2 of Nucleophosmin	IPI00220740	0.316	0.18737	38	63	42	63
550	T-complex protein 1 subunit alpha	IPI00290566	0.313	0.18763	53	52	48	61
551	RNA-binding protein NOB1	IPI00022373	0.313	0.18775	4	3	4	5
552	Isoform 2 of Sacsin	IPI00784002	0.313	0.18775	4	3	6	3
553	CAAX prenyl protease 1 homolog	IPI00027180	0.313	0.18775	5	0	0	7
554	Isoform 2 of Microtubule-associated protein 4	IPI00220113	0.313	0.18775	4	3	3	6
555	Isoform 1 of Roundabout homolog 1	IPI00219798	0.313	0.18775	4	3	4	5
556	Isoform 3 of Fermitin family homolog 1	IPI00220602	0.313	0.18775	5	0	2	7
557	Cytochrome c oxidase subunit 5B, mitochondrial	IPI00021785	0.313	0.18775	2	5	0	7
558	Isoform 1 of Deoxycytidylate deaminase	IPI00296863	0.313	0.18775	5	2	3	6
559	Probable ATP-dependent RNA helicase DDX27	IPI00293078	0.313	0.18775	4	3	7	2
560	Isoform 1 of Endophilin-B1	IPI00006558	0.313	0.18775	4	3	5	4
561	Probable saccharopine dehydrogenase	IPI00329600	0.303	0.18846	4	4	5	5
562	Isoform 2 of AP-2 complex subunit alpha-2	IPI00016621	0.303	0.18846	5	3	6	4

No.	Description	Accession number	STN[1]	p-Value[1]	Con_A[2]	Con_B[2]	SORA_A[2]	SORA_B[2]
563	cDNA FLJ54536, highly similar to Mitochondrial 28S ribosomal protein S27	IPI00022002	0.303	0.18846	4	4	3	7
564	cDNA FLJ56414, highly similar to Homo sapiens proline-, glutamic acid-, leucine-rich protein 1 (PELP1)	IPI00006702	0.303	0.18846	4	4	5	5
565	Isoform 1 of Serine hydroxymethyltransferase, cytosolic	IPI00002519	0.303	0.18846	4	4	6	4
566	Epidermal growth factor receptor kinase substrate 8	IPI00290337	0.303	0.18846	5	3	5	5
567	Isoform A of Nucleoporin SEH1	IPI00185533	0.303	0.18846	6	2	4	6
568	Density-regulated protein	IPI00306280	0.303	0.18846	3	5	4	6
569	Cell differentiation protein RCD1 homolog	IPI00023101	0.303	0.18846	5	3	5	5
570	Isoform 1 of Acyl-coenzyme A thioesterase 2, mitochondrial	IPI00220906	0.303	0.18846	4	4	5	5
571	cDNA FLJ56840, highly similar to Galactokinase	IPI00019383	0.303	0.18846	4	4	5	5
572	Isoform 2 of Low molecular weight phosphotyrosine protein phosphatase	IPI00218847	0.303	0.18846	3	5	3	7
573	Putative high mobility group protein B3-like-1	IPI00006437	0.303	0.18846	4	4	4	6
574	ATP-dependent DNA helicase Q1	IPI00178431	0.299	0.19875	26	16	20	25
575	Tripeptidyl-peptidase 2	IPI00020416	0.297	0.19897	18	25	16	30
576	Peroxiredoxin-2	IPI00027350	0.297	0.19897	12	31	20	26
577	Isoform 1 of Methylthioribose-1-phosphate isomerase	IPI00005948	0.294	0.19909	5	4	5	6
578	Mortality factor 4-like protein 2	IPI00014174	0.294	0.19909	4	5	5	6
579	Isoform 1 of AP-3 complex subunit beta-1	IPI00021129	0.294	0.19909	6	3	3	8
580	Transcription elongation factor B polypeptide 2	IPI00026670	0.294	0.19909	5	4	6	5
581	Lysophospholipid acyltransferase 5	IPI00306419	0.294	0.19909	0	7	7	4
582	Glutamate--cysteine ligase catalytic subunit	IPI00215768	0.294	0.19909	6	3	4	7
583	G-rich sequence factor 1	IPI00478657	0.294	0.19909	4	5	5	6
584	Synapse-associated protein 1	IPI00059242	0.294	0.19909	3	6	5	6
585	Probable dimethyladenosine transferase	IPI00004459	0.294	0.19909	4	5	7	4
586	Sialic acid synthase	IPI00147874	0.294	0.19909	5	4	6	5
587	Macrophage-capping protein	IPI00027341	0.294	0.19909	4	5	5	6
588	482 kDa protein	IPI00179298	0.292	0.19924	71	67	63	79
589	Aminoacyl tRNA synthase complex-interacting multifunctional protein 2	IPI00011916	0.287	0.19939	3	7	5	7
590	Protein transport protein Sec23A	IPI00017375	0.287	0.19939	5	5	5	7
591	51 kDa protein	IPI00033025	0.287	0.19939	6	4	8	4
592	V-type proton ATPase subunit C 1	IPI00007814	0.287	0.19939	5	5	5	7
593	Exosome complex exonuclease MTR3	IPI00073602	0.287	0.19939	5	5	6	6
594	HEAT repeat-containing protein 1	IPI00024279	0.287	0.20657	27	22	23	29
595	40S ribosomal protein S7	IPI00013415	0.285	0.20657	17	33	23	30
596	Isoform Long of Long-chain-fatty-acid--CoA ligase 4	IPI00029737	0.280	0.20680	5	6	5	8
597	proteasome subunit beta type-5 isoform 3	IPI00383971	0.280	0.20680	3	8	4	9
598	Prefoldin subunit 4	IPI00015891	0.280	0.20680	5	6	7	6
599	Coatomer subunit beta	IPI00295851	0.280	0.20680	31	23	26	31
600	Cytochrome c	IPI00465315	0.274	0.20751	6	6	7	7
601	Aldehyde dehydrogenase X, mitochondrial	IPI00103467	0.274	0.20751	7	5	7	7
602	Testis-expressed sequence 10 protein	IPI00549664	0.274	0.20751	6	6	6	8
603	Isoform 2 of ATPase family AAA domain-containing protein 3A	IPI00295992	0.274	0.20751	6	6	6	8
604	cDNA FLJ55034	IPI00384122	0.274	0.20751	5	7	7	7
605	Isoform 1 of Heterogeneous nuclear ribonucleoprotein Q	IPI00018140	0.271	0.21318	33	28	32	32
606	Isoform 1 of Adipocyte plasma membrane-associated protein	IPI00031131	0.269	0.21318	5	8	7	8
607	Cytochrome c oxidase subunit 5A, mitochondrial	IPI00025086	0.269	0.21318	8	5	6	9
608	Isoform 1 of Medium-chain specific acyl-CoA dehydrogenase, mitochondrial	IPI00005040	0.269	0.21318	8	5	8	7
609	Isoform 2 of Tumor protein D54	IPI00221178	0.269	0.21318	8	5	8	7
610	Isoform 3 of Keratin, type II cytoskeletal 80	IPI00375843	0.269	0.21318	6	7	8	7
611	Phosphoserine phosphatase	IPI00019178	0.269	0.21318	7	6	7	8
612	Cytochrome b-c1 complex subunit 7	IPI00220416	0.269	0.21318	7	6	8	7
613	DNA damage-binding protein 1	IPI00293464	0.268	0.21322	34	30	28	39
614	Thioredoxin domain-containing protein 17	IPI00646689	0.264	0.21337	7	7	9	7
615	Isoform 2 of ATP-binding cassette sub-family F member 1	IPI00013495	0.264	0.21337	5	9	7	9
616	Barrier-to-autointegration factor	IPI00026087	0.264	0.21337	6	8	7	9
617	Tyrosine-protein phosphatase non-receptor type 1	IPI00297261	0.264	0.21337	6	8	8	8
618	Isoform 3 of Splicing factor, arginine/serine-rich 13A	IPI00009071	0.260	0.21840	7	8	9	8
619	Sec1 family domain-containing protein 1	IPI00165261	0.260	0.21840	6	9	10	7
620	Isoform 1 of Transcription elongation regulator 1	IPI00247871	0.256	0.21851	7	9	9	9
621	Phosphoribosyl pyrophosphate synthase-associated protein 2	IPI00003168	0.256	0.21851	7	9	7	11
622	28S ribosomal protein S29, mitochondrial	IPI00018120	0.256	0.21851	8	8	8	10
623	Programmed cell death protein 10	IPI00298558	0.256	0.21851	7	9	8	10
624	Isoform Long of 60 kDa SS-A/Ro ribonucleoprotein	IPI00019450	0.256	0.21851	9	7	8	10
625	Isoform 1 of Clathrin heavy chain 2	IPI00022881	0.253	0.22252	37	43	36	47
626	L-xylulose reductase	IPI00448095	0.252	0.22252	8	9	7	12
627	Calpain small subunit 1	IPI00025084	0.252	0.22252	7	10	8	11
628	Isoform 1 of 39S ribosomal protein L22, mitochondrial	IPI00414410	0.252	0.22252	9	8	6	13
629	Isoform 1 of 39S ribosomal protein L4, mitochondrial	IPI00023334	0.252	0.22252	9	8	10	9
630	Small nuclear ribonucleoprotein Sm D1	IPI00302850	0.250	0.22252	36	47	40	46
631	Isoform 1 of Cullin-4B	IPI00179057	0.248	0.22252	12	6	11	9
632	Prohibitin-2	IPI00027252	0.247	0.22532	33	54	40	50
633	Protein mago nashi homolog 2	IPI00059292	0.239	0.22747	9	12	12	11
634	Peroxiredoxin-4	IPI00011937	0.239	0.22747	9	12	9	14
635	Isoform 1 of Catenin alpha-1	IPI00215948	0.239	0.22747	13	8	12	11
636	Cytoplasmic dynein 1 light intermediate chain 1	IPI00007675	0.239	0.22747	9	12	8	15
637	Isoform 2 of Splicing factor 1	IPI00294627	0.239	0.22747	9	12	10	13
638	Isoform 1 of Cytosol aminopeptidase	IPI00419237	0.239	0.22747	9	12	9	14
639	Isoform 2 of Exosome complex exonuclease RRP44	IPI00183462	0.236	0.22747	10	12	14	10
640	Isoform 1 of Cleavage and polyadenylation specificity factor subunit 6	IPI00012998	0.236	0.22747	10	12	12	12
641	E3 ubiquitin/ISG15 ligase TRIM25	IPI00029629	0.233	0.22891	10	13	10	15
642	Isoform 1 of Regulator of nonsense transcripts 1	IPI00034049	0.233	0.22891	13	10	10	15
643	Isoform 1 of Ubiquitin-like modifier-activating enzyme 6	IPI00023647	0.231	0.22891	12	12	11	15
644	Isoform 1 of Methylcrotonoyl-CoA carboxylase beta chain, mitochondrial	IPI00784044	0.231	0.22891	11	13	11	15
645	T-complex protein 1 subunit zeta	IPI00027626	0.230	0.23072	49	66	51	67
646	Eukaryotic translation initiation factor 3 subunit M	IPI00102069	0.228	0.23072	10	15	13	14
647	Isoform Beta-2 of DNA topoisomerase 2-beta	IPI00027280	0.228	0.23072	15	10	11	16
648	40S ribosomal protein S17	IPI00221093	0.228	0.23072	9	16	11	16
649	Acylamino-acid-releasing enzyme	IPI00337741	0.228	0.23072	12	13	17	10
650	Dihydropyrimidinase-like 2	IPI00106642	0.226	0.23072	12	14	13	15
651	Proteasome subunit beta type-3	IPI00028004	0.226	0.23072	10	16	13	15
652	40S ribosomal protein S13	IPI00221089	0.224	0.23163	11	16	14	15
653	E3 ubiquitin-protein ligase UBR5	IPI00026320	0.224	0.23163	14	13	14	15
654	Actin-related protein 3	IPI00028091	0.224	0.23163	11	16	14	15
655	UPF0027 protein C22orf28	IPI00550689	0.224	0.23163	13	14	15	14
656	Isoform 1 of Pyruvate dehydrogenase E1 component subunit beta, mitochondrial	IPI00003925	0.224	0.23163	9	18	14	15

No.	Description	Accession number	STN[1]	p-Value[1]	Con_A[2]	Con_B[2]	SORA_A[2]	SORA_B[2]
657	cDNA FLJ55482, highly similar to Annexin A11	IPI00414320	0.224	0.23163	10	17	15	14
658	Aspartate aminotransferase, cytoplasmic	IPI00219029	0.222	0.23163	14	14	15	15
659	UPF0568 protein C14orf166	IPI00006980	0.222	0.23163	14	14	14	16
660	Putative high mobility group protein 1-like 10	IPI00018755	0.218	0.23239	15	15	18	14
661	Putative uncharacterized protein NAP1L4	IPI00017763	0.216	0.23341	12	19	15	18
662	Isoform Mitochondrial of Fumarate hydratase, mitochondrial	IPI00296053	0.216	0.23341	16	15	16	17
663	Isoform 2 of Voltage-dependent anion-selective channel protein 2	IPI00024145	0.205	0.23537	19	19	21	19
664	Tu translation elongation factor, mitochondrial precursor	IPI00027107	0.203	0.23579	16	23	17	24
665	Aldehyde dehydrogenase, mitochondrial	IPI00006663	0.203	0.23579	22	17	21	20
666	Calpain-1 catalytic subunit	IPI00011285	0.203	0.23579	19	20	20	21
667	Isoform 1 of WD repeat-containing protein 1	IPI00746165	0.202	0.23579	21	19	19	23
668	Puromycin-sensitive aminopeptidase	IPI00026216	0.195	0.23685	26	20	26	22
669	Isoform 1 of Nucleoside diphosphate kinase B	IPI00026260	0.193	0.23722	17	31	20	30
670	32 kDa protein	IPI00176692	0.193	0.23741	97	128	102	126
671	Isoform Gamma-1 of Serine/threonine-protein phosphatase PP1-gamma catalytic subunit	IPI00005705	0.188	0.23783	23	30	24	31
672	Isoform Long of Delta-1-pyrroline-5-carboxylate synthase	IPI00008982	0.186	0.23790	26	29	25	32
673	Isoform 2 of Ubiquitin conjugation factor E4 A	IPI00028957	0.182	0.23806	2	2	3	2
674	cDNA FLJ54030, highly similar to Polymerase delta-interacting protein 3	IPI00440688	0.182	0.23806	2	0	2	3
675	Isoform SRP40-1 of Splicing factor, arginine/serine-rich 5	IPI00012341	0.182	0.23806	2	0	0	3
676	Isoform 2 of Serine/threonine-protein kinase PAK 3	IPI00027382	0.182	0.23806	0	0	3	0
677	Serine/threonine-protein kinase VRK1	IPI00019640	0.182	0.23806	2	2	3	2
678	Pumilio domain-containing protein C14orf21	IPI00216999	0.182	0.23806	2	2	3	2
679	Isoform 1 of Rho guanine nucleotide exchange factor 2	IPI00291316	0.182	0.23806	2	0	0	3
680	Protein LYRIC	IPI00328715	0.182	0.23806	2	0	2	3
681	Vacuolar protein sorting-associated protein 4B	IPI00182728	0.182	0.23806	0	2	3	0
682	Isoform 1 of Helicase-like transcription factor	IPI00339381	0.182	0.23806	0	0	2	3
683	Guanine nucleotide-binding protein G(k) subunit alpha	IPI00220578	0.182	0.23806	2	2	3	2
684	U6 snRNA-associated Sm-like protein LSm1	IPI00004436	0.182	0.23806	2	2	3	2
685	Isoform 1 of Zinc finger protein 207	IPI00013457	0.182	0.23806	0	2	2	3
686	Leucine-rich repeat-containing protein 40	IPI00152998	0.182	0.23806	0	2	3	0
687	Isoform 2 of Sorting nexin-3	IPI00216508	0.182	0.23806	0	2	0	3
688	Isoform 1 of UPF0667 protein C1orf55	IPI00167998	0.182	0.23806	0	2	2	3
689	Condensin complex subunit 2	IPI00299507	0.182	0.23806	2	0	2	3
690	Serine/threonine-protein kinase 25	IPI00012093	0.182	0.23806	2	0	2	3
691	Isoform 2 of Cytochrome b5	IPI00182933	0.182	0.23806	2	0	2	3
692	Eukaryotic translation initiation factor 4E	IPI00027485	0.182	0.23806	0	0	0	3
693	Cleavage and polyadenylation specificity factor subunit 2	IPI00419531	0.182	0.23806	2	2	3	0
694	Isoform 2 of Ribosomal RNA processing protein 1 homolog B	IPI00032374	0.182	0.23806	2	2	2	3
695	Isoamyl acetate-hydrolyzing esterase 1 homolog	IPI00419194	0.182	0.23806	2	2	3	2
696	Nucleolysin TIAR	IPI00005615	0.182	0.23806	2	0	3	0
697	Isoform XLas-1 of Guanine nucleotide-binding protein G(s) subunit alpha isoforms XLas	IPI00095891	0.182	0.23806	2	0	3	0
698	Isoform 1 of Protein phosphatase methylesterase 1	IPI00007694	0.182	0.23806	0	2	3	0
699	Gamma-soluble NSF attachment protein	IPI00293817	0.182	0.23806	0	2	0	3
700	Isoform 1 of COP9 signalosome complex subunit 1	IPI00156282	0.182	0.23806	0	2	2	3
701	Isoform 1 of Epidermal growth factor receptor kinase substrate 8-like protein 3	IPI00181833	0.182	0.23806	0	0	3	0
702	Wiskott-Aldrich syndrome protein family member 2	IPI00472164	0.182	0.23806	0	2	0	3
703	Isoform 1 of Choline kinase alpha	IPI00409761	0.182	0.23806	0	2	0	3
704	39S ribosomal protein L16, mitochondrial	IPI00000821	0.182	0.23806	0	2	0	3
705	Isoform DFF45 of DNA fragmentation factor subunit alpha (Fragment)	IPI00010882	0.182	0.23806	2	2	2	3
706	NADH dehydrogenase [ubiquinone] 1 beta subcomplex subunit 8, mitochondrial	IPI00028883	0.182	0.23806	2	0	3	2
707	mortality factor 4	IPI00001955	0.182	0.23806	2	0	0	3
708	Isoform Beta of DNA ligase 3	IPI00000156	0.182	0.23806	2	2	3	2
709	Isoform 1 of 5'-nucleotidase domain-containing protein 3	IPI00465170	0.182	0.23806	0	2	3	2
710	PIH1 domain-containing protein 1	IPI00550995	0.182	0.23806	0	0	2	3
711	Prefoldin subunit 2	IPI00006052	0.182	0.23806	0	2	2	3
712	CD9 antigen	IPI00215997	0.182	0.23806	0	0	3	0
713	Immature colon carcinoma transcript 1 protein	IPI00029114	0.182	0.23806	0	0	2	3
714	Short/branched chain specific acyl-CoA dehydrogenase, mitochondrial	IPI00024623	0.182	0.23806	0	2	3	0
715	Isoform 1 of Casein kinase I isoform alpha	IPI00183400	0.182	0.23806	0	0	3	0
716	PRA1 family protein 3	IPI00007426	0.182	0.23806	0	0	0	3
717	Isoform 1 of Serologically defined colon cancer antigen 1	IPI00301618	0.182	0.23806	0	0	3	0
718	Isoform 2 of Ral GTPase-activating protein subunit alpha-1	IPI00456722	0.182	0.23806	2	0	0	3
719	Gamma-tubulin complex component 2	IPI00029705	0.182	0.23806	2	2	3	0
720	Mitochondrial import inner membrane translocase subunit Tim13	IPI00001589	0.182	0.23806	0	0	0	3
721	Transcriptional repressor p66-beta	IPI00103554	0.182	0.23806	2	2	0	3
722	Cohesin subunit SA-1	IPI00025158	0.182	0.23806	0	2	0	3
723	Methionyl-tRNA synthetase, mitochondrial	IPI00062839	0.182	0.23806	2	0	3	2
724	Isoform II of Ubiquitin-protein ligase E3A	IPI00011609	0.182	0.23806	2	0	2	3
725	Isoform 1 of Disks large homolog 1	IPI00030351	0.182	0.23806	2	0	2	3
726	Isoform 1 of Methyltransferase-like protein 9	IPI00100239	0.182	0.23806	0	0	3	0
727	2,4-dienoyl-CoA reductase, mitochondrial	IPI00003482	0.182	0.23806	0	2	2	3
728	Serine/threonine-protein kinase N2	IPI00002804	0.182	0.23806	2	0	2	3
729	Dynein light chain Tctex-type 1	IPI00019495	0.182	0.23806	0	0	0	3
730	Isoform 2 of Triple functional domain protein	IPI00479523	0.182	0.23806	0	0	3	0
731	Isoform 2 of Tether containing UBX domain for GLUT4	IPI00065276	0.182	0.23806	2	2	3	2
732	Probable ATP-dependent RNA helicase DDX49	IPI00003739	0.182	0.23806	2	2	0	3
733	Fumarylacetoacetate hydrolase domain-containing protein 2A	IPI00329742	0.182	0.23806	0	2	2	3
734	Protein LLP homolog	IPI00031615	0.182	0.23806	2	2	0	3
735	Isoform 1 of Alpha-adducin	IPI00019901	0.182	0.23806	0	0	0	3
736	Probable arginyl-tRNA synthetase, mitochondrial	IPI00549566	0.182	0.23806	2	0	0	3
737	Wilm's tumour protein family protein	IPI00044748	0.182	0.23806	0	2	0	3
738	cDNA FLJ61629, highly similar to Clathrin interactor 1	IPI00291930	0.182	0.23806	0	2	2	3
739	aldehyde dehydrogenase 9A1	IPI00479877	0.182	0.23806	2	0	2	3
740	UDP-glucose 4-epimerase	IPI00553131	0.182	0.23806	2	0	3	2
741	General transcription factor IIH subunit 1	IPI00030380	0.182	0.23806	2	2	3	2
742	Isoform 1 of Transcription elongation factor A protein 1	IPI00333215	0.182	0.23806	0	0	3	0
743	cDNA FLJ55988, highly similar to RNA-binding protein Luc7-like 2	IPI00006932	0.182	0.23806	2	0	3	0
744	Uncharacterized protein C20orf4	IPI00166013	0.182	0.23806	0	0	0	3
745	Isoform 2 of Succinyl-CoA ligase [ADP-forming] subunit beta, mitochondrial	IPI00217232	0.182	0.23806	0	0	2	3
746	Isoform Long of Tyrosine-protein kinase SYK	IPI00018597	0.182	0.23806	0	0	0	3
747	Isoform 2 of Integrator complex subunit 7	IPI00645022	0.182	0.23806	0	0	2	3
748	Isoform 1 of Voltage-gated potassium channel subunit beta-2	IPI00021088	0.182	0.23806	2	2	0	3
749	D-tyrosyl-tRNA(Tyr) deacylase 1	IPI00152692	0.182	0.23806	0	0	2	3
750	Isoform 1 of Protein LSM14 homolog B	IPI00032635	0.182	0.23806	0	2	2	3
751	Isoform 1 of Kinesin-like protein KIF2A	IPI00010368	0.182	0.23806	0	2	2	3

No.	Description	Accession number	STN[1]	p-Value[1]	Con_A[2]	Con_B[2]	SORA_A[2]	SORA_B[2]
752	coatomer subunit epsilon isoform c	IPI00399319	0.182	0.23806	0	0	3	2
753	Isoform 1 of Cell division cycle protein 23 homolog	IPI00005822	0.182	0.23806	2	0	3	0
754	WD repeat domain 57 (U5 snRNP specific), isoform CRA_b	IPI00385642	0.182	0.23806	0	2	2	3
755	Isoform 1 of TRAF2 and NCK-interacting protein kinase	IPI00145805	0.182	0.23806	0	0	3	0
756	Disintegrin and metalloproteinase domain-containing protein 10	IPI00013897	0.182	0.23806	2	0	2	3
757	Isoform 4 of Zinc finger protein 638	IPI00178953	0.182	0.23806	2	0	0	3
758	cDNA FLJ56184, highly similar to Proto-oncogene tyrosine-protein kinase LCK	IPI00394952	0.182	0.23806	2	2	3	2
759	Protein FAM49A	IPI00006574	0.182	0.23806	2	2	3	2
760	Isoform 1 of Opioid growth factor receptor	IPI00021537	0.182	0.23806	2	0	3	2
761	Transcriptional activator protein Pur-alpha	IPI00023591	0.182	0.23806	0	0	2	3
762	MAP kinase-activated protein kinase 3	IPI00005777	0.182	0.23806	0	0	0	3
763	Ubiquitin-like domain-containing CTD phosphatase 1	IPI00291669	0.182	0.23806	2	0	3	0
764	Isoform 1 of Collagen type IV alpha-3-binding protein	IPI00024701	0.182	0.23806	0	2	2	3
765	La-related protein 7	IPI00294742	0.182	0.23806	2	0	3	2
766	Isoform 3 of Shootin-1	IPI00448751	0.182	0.23806	0	0	3	0
767	NF-kappa-B-repressing factor	IPI00005675	0.182	0.23806	0	0	0	3
768	TFIIH basal transcription factor complex helicase XPB subunit	IPI00747053	0.182	0.23806	0	0	0	3
769	Ribonuclease P protein subunit p38	IPI00019195	0.182	0.23806	0	2	0	3
770	Isoform 1 of CWF19-like protein 1	IPI00101600	0.182	0.23806	0	2	0	3
771	Vacuolar ATPase assembly integral membrane protein VMA21	IPI00146447	0.182	0.23806	2	2	0	3
772	40S ribosomal protein S21	IPI00017448	0.182	0.23806	0	0	2	3
773	Isoform 1 of Ataxin-2	IPI00180154	0.182	0.23806	0	2	2	3
774	Arfaptin-2	IPI00021257	0.182	0.23806	0	0	2	3
775	Putative uncharacterized protein KIF13B	IPI00939605	0.182	0.23806	0	2	2	3
776	Isoform 1 of Serine/threonine-protein phosphatase 6 regulatory ankyrin repeat subunit A	IPI00477505	0.182	0.23806	2	0	3	2
777	Secernin-1	IPI00289862	0.182	0.23806	0	0	3	2
778	Isoform 1 of FAD-dependent oxidoreductase domain-containing protein 1	IPI00549357	0.182	0.23806	0	2	0	3
779	Isoform 1 of Coiled-coil domain-containing protein 51	IPI00153023	0.182	0.23806	0	2	0	3
780	Isoform 1 of N6-adenosine-methyltransferase 70 kDa subunit	IPI00009755	0.182	0.23806	0	0	3	0
781	Isoform 1 of Cellular tumor antigen p53	IPI00025087	0.182	0.23806	0	0	2	3
782	Isoform 1 of Protein CIP2A	IPI00154283	0.182	0.23806	0	0	0	3
783	Isoform 2 of Ganglioside-induced differentiation-associated protein 1	IPI00290544	0.182	0.23806	2	2	3	2
784	Pyruvate dehydrogenase phosphatase regulatory subunit, mitochondrial	IPI00168407	0.182	0.23806	0	0	2	3
785	Isoform 2 of Fumarylacetoacetate hydrolase domain-containing protein 1	IPI00440828	0.182	0.23806	0	2	2	3
786	Isoform 2 of Late secretory pathway protein AVL9 homolog	IPI00022042	0.182	0.23806	2	0	3	0
787	Isoform 1 of Tumor suppressor p53-binding protein 1	IPI00029778	0.182	0.23806	0	0	2	3
788	Isoform SMN of Survival motor neuron protein	IPI00003394	0.182	0.23806	0	0	2	3
789	Alpha- and gamma-adaptin-binding protein p34	IPI00100193	0.182	0.23806	0	0	0	3
790	Putative uncharacterized protein DKFZp686H16220	IPI00552191	0.182	0.23806	2	0	0	3
791	Isoform 1 of Transcriptional repressor p66-alpha	IPI00410330	0.182	0.23806	0	0	0	3
792	Ras-related protein Rab-22A	IPI00007756	0.182	0.23806	0	2	0	3
793	Isoform 1 of Transmembrane protein 126B	IPI00020540	0.182	0.23806	0	0	0	3
794	tyrosine-protein phosphatase non-receptor type 2	IPI00106928	0.182	0.23806	0	2	0	3
795	Isoform 1 of Golgi-associated PDZ and coiled-coil motif-containing protein	IPI00186721	0.182	0.23806	0	0	3	0
796	Isoform 2 of Transducin-like enhancer protein 3	IPI00177938	0.182	0.23806	0	0	2	3
797	Hypoxia up-regulated protein 1	IPI00000877	0.180	0.25325	34	28	28	36
798	Ubiquitin-like modifier-activating enzyme 1	IPI00645078	0.175	0.25363	32	38	36	36
799	40S ribosomal protein S2	IPI00013485	0.173	0.25389	33	40	38	37
800	145 kDa protein	IPI00218097	0.173	0.25389	3	0	3	3
801	Isoform 2 of Serrate RNA effector molecule homolog	IPI00220038	0.173	0.25389	0	3	2	4
802	7-dehydrocholesterol reductase	IPI00294501	0.173	0.25389	3	2	0	4
803	39S ribosomal protein L17, mitochondrial	IPI00172591	0.173	0.25389	2	3	3	3
804	Isoform SCPx of Non-specific lipid-transfer protein	IPI00026105	0.173	0.25389	0	3	4	2
805	Integrin beta-5	IPI00029741	0.173	0.25389	0	3	2	4
806	YLP motif-containing protein 1	IPI00165434	0.173	0.25389	3	2	2	4
807	Isoform 1 of IST1 homolog	IPI00024660	0.173	0.25389	2	3	2	4
808	Isoform 1 of Phosphatidylinositol-3,4,5-trisphosphate 5-phosphatase 2	IPI00016932	0.173	0.25389	2	3	4	2
809	Isoform 2 of Phosphoglucomutase-1	IPI00217872	0.173	0.25389	0	3	3	3
810	Scaffold attachment factor B1	IPI00300631	0.173	0.25389	2	3	0	4
811	Ras-related protein Rab-21	IPI00007755	0.173	0.25389	3	2	2	4
812	Isoform 1 of Caldesmon	IPI00014516	0.173	0.25389	0	3	3	3
813	Microtubule-associated protein 1S	IPI00296485	0.173	0.25389	3	0	3	3
814	Isoform 3 of Oxidation resistance protein 1	IPI00166807	0.173	0.25389	3	0	4	2
815	Isoform 2 of Membrane magnesium transporter 1	IPI00166785	0.173	0.25389	3	2	2	4
816	Isoform 1 of Ubiquitin carboxyl-terminal hydrolase 15	IPI00000728	0.173	0.25389	3	0	0	4
817	Isoform 1 of RNA-binding protein 4	IPI00003704	0.173	0.25389	0	3	2	4
818	Calponin-3	IPI00216682	0.173	0.25389	2	3	0	4
819	Fumarylacetoacetate hydrolase domain-containing protein 2B	IPI00301994	0.173	0.25389	2	3	3	3
820	cDNA FLJ56037, highly similar to Cullin-2	IPI00014311	0.173	0.25389	3	0	2	4
821	Adenylyl cyclase-associated protein	IPI00939159	0.173	0.25389	2	3	3	3
822	Isoform 1 of YTH domain family protein 1	IPI00221345	0.173	0.25389	3	2	3	3
823	cDNA FLJ40287 fis, clone TESTI2027909, highly similar to 5'-AMP-ACTIVATED PROTEIN KINASE	IPI00473047	0.173	0.25389	2	3	0	4
824	Isoform 1 of Replication factor C subunit 1	IPI00375358	0.173	0.25389	2	3	3	3
825	Protein ERGIC-53	IPI00026530	0.173	0.25389	3	2	2	4
826	Transmembrane protein 214	IPI00477118	0.173	0.25389	3	2	3	3
827	39S ribosomal protein L2, mitochondrial	IPI00411816	0.173	0.25389	3	0	3	3
828	RcDNAJ9 (Fragment)	IPI00014718	0.173	0.25389	2	3	2	4
829	Transmembrane emp24 domain-containing protein 5	IPI00294472	0.173	0.25389	2	3	2	4
830	Isoform 2 of WASH complex subunit 7	IPI00164930	0.173	0.25389	2	3	0	4
831	Isoform 1 of Vacuolar-sorting protein SNF8	IPI00101524	0.173	0.25389	3	2	4	2
832	Isoform 1 of Mixed lineage kinase domain-like protein	IPI00180781	0.173	0.25389	3	2	2	4
833	Putative uncharacterized protein DKFZp761L1314 (Fragment)	IPI00183297	0.173	0.25389	0	3	4	2
834	Mitochondrial import inner membrane translocase subunit Tim16	IPI00218463	0.173	0.25389	0	3	3	3
835	Putative ATP-dependent Clp protease proteolytic subunit, mitochondrial	IPI00003870	0.173	0.25389	2	3	3	3
836	Glia maturation factor gamma	IPI00028414	0.173	0.25389	3	0	3	3
837	Ethanolamine-phosphate cytidylyltransferase	IPI00015285	0.173	0.25389	0	3	0	4
838	Heat shock 70 kDa protein 4	IPI00002966	0.170	0.26448	37	40	35	44
839	Importin subunit beta-1	IPI00001639	0.166	0.26470	40	45	38	49
840	PNAS-139	IPI00000477	0.165	0.26478	4	2	4	3
841	Isoform SRP55-1 of Splicing factor, arginine/serine-rich 6	IPI00012345	0.165	0.26478	3	3	2	5
842	Alcohol dehydrogenase class-3	IPI00746777	0.165	0.26478	4	0	2	5
843	Isoform 1 of Proteasome assembly chaperone 1	IPI00030770	0.165	0.26478	3	3	2	5
844	Ras-related C3 botulinum toxin substrate 2	IPI00010270	0.165	0.26478	0	4	3	4
845	Calcium-binding protein 39	IPI00032561	0.165	0.26478	3	3	4	3
846	Isoform 4 of Uncharacterized protein KIAA0090	IPI00642244	0.165	0.26478	2	4	3	4

No.	Description	Accession number	STN[1]	p-Value[1]	Con_A[2]	Con_B[2]	SORA_A[2]	SORA_B[2]
847	Isovaleryl-CoA dehydrogenase, mitochondrial	IPI00645805	0.165	0.26478	3	3	3	4
848	Isoform 1 of V-type proton ATPase subunit H	IPI00296191	0.165	0.26478	3	3	0	5
849	PDZ domain-containing protein GIPC1	IPI00024705	0.165	0.26478	3	3	5	2
850	Isoform 2 of Epimerase family protein SDR39U1	IPI00643286	0.165	0.26478	2	4	2	5
851	Cytoplasmic aconitate hydratase	IPI00008485	0.165	0.26478	3	3	4	3
852	HSR1 protein	IPI00384745	0.165	0.26478	4	0	4	3
853	U6 snRNA-associated Sm-like protein LSm3	IPI00219229	0.165	0.26478	0	4	3	4
854	RNA-binding protein 12	IPI00550308	0.165	0.26478	4	2	3	4
855	F-box-like/WD repeat-containing protein TBL1XR1	IPI00002922	0.165	0.26478	3	3	3	4
856	Osteoclast-stimulating factor 1	IPI00414836	0.165	0.26478	0	4	4	3
857	Isoform 1 of Serine/threonine-protein phosphatase 2A 55 kDa regulatory subunit B beta isoform	IPI00020850	0.165	0.26478	4	0	2	5
858	Isoform 2 of Lysine-specific histone demethylase 1A	IPI00217540	0.165	0.26478	3	3	3	4
859	Isoform 2 of Transportin-2	IPI00164417	0.165	0.26478	4	2	3	4
860	Protein S100-A13	IPI00016179	0.165	0.26478	3	3	3	4
861	RNA 3'-terminal phosphate cyclase-like protein	IPI00294229	0.165	0.26478	3	3	3	4
862	Transcription initiation factor IIB	IPI00022820	0.165	0.26478	3	3	5	0
863	WW domain-binding protein 11	IPI00170786	0.165	0.26478	3	3	3	4
864	cAMP-dependent protein kinase type I-alpha regulatory subunit	IPI00021831	0.165	0.26478	3	3	4	3
865	Isoform 1 of Phosphoenolpyruvate carboxykinase [GTP], mitochondrial	IPI00797038	0.165	0.26478	3	3	4	3
866	Isoform 1 of Putative methyltransferase METT10D	IPI00163391	0.165	0.26478	4	2	4	3
867	Cytovillin 2 (Fragment)	IPI00384282	0.165	0.26478	4	0	4	3
868	Splicing factor 45	IPI00176706	0.165	0.26478	3	3	2	5
869	cDNA FLJ56389, highly similar to Elongation factor 1-gamma	IPI00000875	0.164	0.27102	43	47	43	49
870	Isoform 1 of Nucleolar RNA helicase 2	IPI00015953	0.163	0.27113	44	48	42	52
871	Isoform 1 of ER lumen protein retaining receptor 2	IPI00018248	0.159	0.27132	3	4	5	3
872	Isoform 1 of Malignant T cell-amplified sequence 1	IPI00179026	0.159	0.27132	4	3	2	6
873	Long-chain-fatty-acid--CoA ligase 3	IPI00031397	0.159	0.27132	5	2	0	6
874	Transducin beta-like protein 3	IPI00477971	0.159	0.27132	4	3	2	6
875	Junction plakoglobin	IPI00554711	0.159	0.27132	3	4	2	6
876	Isoform 1 of Erlin-2	IPI00026942	0.159	0.27132	3	4	4	4
877	cDNA FLJ53975, highly similar to Acetyl-CoA acetyltransferase, cytosolic	IPI00291419	0.159	0.27132	3	4	3	5
878	Isoform 1 of Protein unc-45 homolog A	IPI00072534	0.159	0.27132	3	4	0	6
879	Isoform 1 of RNA-binding protein 14	IPI00013174	0.159	0.27132	3	4	0	6
880	EPS8L2 protein	IPI00414315	0.159	0.27132	3	4	4	4
881	Phosphatidylinositide phosphatase SAC1	IPI00022275	0.159	0.27132	2	5	4	4
882	Isoform 1 of EH domain-binding protein 1	IPI00178187	0.159	0.27132	3	4	3	5
883	Kinesin-like protein KIF13B	IPI00021753	0.159	0.27132	3	4	3	5
884	Superoxide dismutase [Mn], mitochondrial	IPI00022314	0.159	0.27132	3	4	3	5
885	Isoform 1 of Pentatricopeptide repeat-containing protein 3, mitochondrial	IPI00783302	0.159	0.27132	2	5	3	5
886	Isoform 1 of 182 kDa tankyrase-1-binding protein	IPI00304589	0.159	0.27132	3	4	3	5
887	treacle protein isoform a	IPI00165041	0.159	0.27132	0	5	0	6
888	Ras-related protein Rab-6B	IPI00016891	0.159	0.27132	2	5	2	6
889	Prefoldin subunit 5	IPI00015361	0.159	0.27132	5	0	0	6
890	DnaJ homolog subfamily C member 7	IPI00329629	0.159	0.27132	2	5	4	4
891	Transgelin	IPI00216138	0.159	0.27132	4	3	3	5
892	Isoform 1 of Uncharacterized methyltransferase WBSCR22	IPI00013810	0.159	0.27132	3	4	4	4
893	Cystatin-B	IPI00021828	0.159	0.27132	3	4	3	5
894	Isoform 1 of SWI/SNF-related matrix-associated actin-dependent regulator of chromatin subfamily E	IPI00017669	0.159	0.27132	4	3	4	4
895	Peptidase M20 domain-containing protein 2	IPI00217852	0.154	0.27548	5	3	6	3
896	Isoform 1 of Vacuolar protein sorting-associated protein 29	IPI00170796	0.154	0.27548	3	5	3	6
897	Isoform 2 of Isopentenyl-diphosphate Delta-isomerase 1	IPI00220014	0.154	0.27548	4	4	3	6
898	NADPH--cytochrome P450 reductase	IPI00470467	0.154	0.27548	4	4	2	7
899	Phosphomevalonate kinase	IPI00220648	0.154	0.27548	3	5	3	6
900	NADH dehydrogenase [ubiquinone] 1 beta subcomplex subunit 5, mitochondrial	IPI00013459	0.154	0.27548	4	4	4	5
901	serine/threonine-protein phosphatase PP1-alpha catalytic subunit isoform 3	IPI00027423	0.154	0.27548	4	4	4	5
902	Isoform 3 of Drebrin-like protein	IPI00101968	0.154	0.27548	4	4	5	4
903	Transcription elongation factor B polypeptide 1	IPI00300341	0.154	0.27548	4	4	5	4
904	Isoform 1 of Enolase-phosphatase E1	IPI00038378	0.149	0.28149	4	5	6	4
905	Isoform 3 of Mediator of RNA polymerase II transcription subunit 23	IPI00413272	0.149	0.28149	6	3	5	5
906	Cell division protein kinase 6	IPI00023529	0.149	0.28149	4	5	5	5
907	Isoform 1 of Nck-associated protein 1	IPI00031982	0.145	0.28459	5	5	5	6
908	Thiosulfate sulfurtransferase	IPI00216293	0.145	0.28459	6	4	5	6
909	Amidophosphoribosyltransferase	IPI00029534	0.145	0.28459	5	5	3	8
910	Isoform 1 of Transcription elongation factor SPT5	IPI00298058	0.145	0.28459	2	8	7	4
911	Isoform 1 of Protein fto	IPI00028277	0.145	0.28459	5	5	4	7
912	Isoform 1 of Abhydrolase domain-containing protein 14B	IPI00063827	0.145	0.28459	4	6	6	5
913	39S ribosomal protein L46, mitochondrial	IPI00023161	0.145	0.28459	6	4	3	8
914	V-type proton ATPase subunit D	IPI00001568	0.145	0.28459	4	6	5	6
915	Dihydropteridine reductase	IPI00014439	0.145	0.28459	4	6	5	6
916	Regulator of microtubule dynamics protein 1	IPI00329696	0.145	0.28459	3	7	4	7
917	40S ribosomal protein S11	IPI00025091	0.142	0.28791	4	7	5	7
918	Proteasome inhibitor PI31 subunit	IPI00009949	0.142	0.28791	4	7	5	7
919	Isoform 1 of COP9 signalosome complex subunit 7b	IPI00009301	0.142	0.28791	6	5	5	7
920	Peptidyl-prolyl cis-trans isomerase NIMA-interacting 1	IPI00013723	0.142	0.28791	5	6	5	7
921	Isoform 1 of Transformer-2 protein homolog beta	IPI00301503	0.139	0.28992	4	8	7	6
922	Exosome complex exonuclease RRP4	IPI00015905	0.139	0.28992	6	6	6	7
923	Cytochrome b-c1 complex subunit Rieske, mitochondrial	IPI00026964	0.139	0.28992	6	6	6	7
924	Omega-amidase NIT2	IPI00549467	0.139	0.28992	7	5	6	7
925	26 kDa protein	IPI00219685	0.139	0.28992	7	5	5	8
926	Rho-related GTP-binding protein RhoG	IPI00017342	0.139	0.28992	6	6	8	5
927	Coiled-coil-helix-coiled-coil-helix domain-containing protein 3, mitochondrial	IPI00015833	0.139	0.28992	5	7	5	8
928	Isoform 1 of tRNA-nucleotidyltransferase 1, mitochondrial	IPI00289807	0.139	0.28992	6	6	6	7
929	Mitochondrial import inner membrane translocase subunit TIM44	IPI00306516	0.139	0.28992	7	5	7	6
930	60S ribosomal protein L32	IPI00395998	0.139	0.28992	6	6	7	6
931	Isoform 1 of Acyl-coenzyme A thioesterase 9, mitochondrial	IPI00220710	0.136	0.29222	6	7	7	7
932	Ras-related protein Rab-5C	IPI00016339	0.136	0.29222	8	5	7	7
933	Quinone oxidoreductase	IPI00000792	0.136	0.29222	6	7	7	7
934	Phosphoglucomutase-2	IPI00550364	0.136	0.29222	8	5	6	8
935	Isoform 1 of Protein phosphatase 1 regulatory subunit 7	IPI00033600	0.136	0.29222	6	7	7	7
936	Eukaryotic translation elongation factor 1 epsilon-1	IPI00003588	0.133	0.29370	7	7	6	9
937	Ataxin-10	IPI00001636	0.133	0.29370	9	5	10	5
938	Isoform 1 of Oxysterol-binding protein 1	IPI00024971	0.133	0.29370	7	7	7	8
939	Protein kinase, cAMP-dependent, regulatory, type II, alpha, isoform CRA_b	IPI00063234	0.131	0.29562	8	7	9	7
940	Isoform 1 of 3'(2'),5'-bisphosphate nucleotidase 1	IPI00410214	0.131	0.29562	8	7	9	7

No.	Description	Accession number	STN[1]	p-Value[1]	Con_A[2]	Con_B[2]	SORA_A[2]	SORA_B[2]
941	40S ribosomal protein S6	IPI00021840	0.129	0.29687	6	10	5	12
942	Isoform 1 of Cleavage and polyadenylation specificity factor subunit 7	IPI00550821	0.129	0.29687	9	7	9	8
943	Pre-mRNA-processing factor 19	IPI00004968	0.129	0.29687	6	10	8	9
944	Isoform 1 of Thyroid receptor-interacting protein 13	IPI00003505	0.129	0.29687	7	9	10	7
945	L-aminoadipate-semialdehyde dehydrogenase-phosphopantetheinyl transferase	IPI00250297	0.129	0.29687	7	9	8	9
946	Isoform 2 of Myosin-VI	IPI00008455	0.127	0.29823	10	7	7	11
947	Isoform 1 of ATP-dependent RNA helicase DDX42	IPI00409671	0.125	0.29906	8	10	8	11
948	Isoform 3 of Ribosome-binding protein 1	IPI00215743	0.125	0.29906	8	10	7	12
949	Isoform 1 of LETM1 and EF-hand domain-containing protein 1, mitochondrial	IPI00017592	0.125	0.29906	10	8	7	12
950	Prolyl endopeptidase	IPI00008164	0.125	0.29906	8	10	9	10
951	Isoform 1 of Protein KIAA1967	IPI00182757	0.123	0.30042	11	8	8	12
952	Transmembrane emp24 domain-containing protein 10	IPI00028055	0.123	0.30042	9	10	8	12
953	Isoform 1 of 14-3-3 protein sigma	IPI00013890	0.122	0.30107	7	13	9	12
954	Isoform 1 of Surfeit locus protein 4	IPI00005737	0.122	0.30107	9	11	11	10
955	Src substrate cortactin	IPI00029601	0.122	0.30107	9	11	11	10
956	Isoform 2 of Dedicator of cytokinesis protein 7	IPI00183572	0.120	0.30175	11	10	8	14
957	Isoform Heart of ATP synthase subunit gamma, mitochondrial	IPI00395769	0.119	0.30235	8	14	13	10
958	Isoform Short of Proteasome subunit alpha type-1	IPI00016832	0.117	0.30337	10	13	10	14
959	Keratin, type II cytoskeletal 75	IPI00005859	0.117	0.30337	15	8	16	8
960	splicing factor 3B subunit 2	IPI00221106	0.117	0.30337	14	9	10	14
961	Actin-related protein 2/3 complex subunit 4	IPI00554811	0.116	0.30386	13	11	12	13
962	Isoform 1 of CCR4-NOT transcription complex subunit 1	IPI00166010	0.115	0.30500	14	11	6	20
963	Isoform Short of Adenosine kinase	IPI00234368	0.115	0.30500	14	11	14	12
964	Histone-binding protein RBBP4	IPI00328319	0.114	0.30564	14	12	14	13
965	40S ribosomal protein S9	IPI00221088	0.110	0.30685	12	17	15	15
966	Isoform 1 of Nuclear pore complex protein Nup155	IPI00026625	0.110	0.30685	16	13	11	19
967	Proteasome subunit alpha type-4	IPI00299155	0.110	0.30685	10	19	14	16
968	Actin-related protein 2	IPI00005159	0.110	0.30685	12	17	15	15
969	DNA replication licensing factor MCM5	IPI00018350	0.109	0.30704	19	11	10	21
970	THO complex subunit 4	IPI00328840	0.109	0.30704	14	16	17	14
971	Activator of 90 kDa heat shock protein ATPase homolog 1	IPI00030706	0.107	0.30844	12	21	15	19
972	Isoform 2 of Structural maintenance of chromosomes flexible hinge domain-containing protein 1	IPI00465022	0.106	0.30889	14	20	16	19
973	HMT1 hnRNP methyltransferase-like 2 isoform 1	IPI00018522	0.105	0.30900	15	20	17	19
974	Glycogen phosphorylase, brain form	IPI00004358	0.105	0.30900	17	18	16	20
975	Annexin A1	IPI00218918	0.099	0.31014	22	22	22	23
976	Glutamate dehydrogenase 1, mitochondrial	IPI00016801	0.095	0.31089	29	22	25	27
977	Proliferating cell nuclear antigen	IPI00021700	0.095	0.31089	22	29	26	26
978	C-1-tetrahydrofolate synthase, cytoplasmic	IPI00218342	0.092	0.31123	29	29	27	32
979	Heat shock protein 75 kDa, mitochondrial	IPI00030275	0.092	0.31135	28	31	27	33
980	Staphylococcal nuclease domain-containing protein 1	IPI00140420	0.083	0.31184	46	40	40	47
981	Adenosylhomocysteinase	IPI00012007	0.082	0.31184	39	50	44	46
982	Isoform B1 of Heterogeneous nuclear ribonucleoproteins A2/B1	IPI00396378	0.059	0.31207	143	167	153	158
983	NAD(P)H dehydrogenase [quinone] 1	IPI00012069	0.000	0.31207	46	55	50	51
984	Isoform A1-B of Heterogeneous nuclear ribonucleoprotein A1	IPI00215965	0.000	0.31207	50	58	49	59
985	Stress-70 protein, mitochondrial	IPI00007765	0.000	0.31207	47	51	44	54
986	FACT complex subunit SPT16	IPI00026970	0.000	0.31207	38	38	31	45
987	Eukaryotic translation initiation factor 3 subunit A	IPI00029012	0.000	0.31207	39	31	29	41
988	Transaldolase	IPI00744692	0.000	0.31207	29	34	26	37
989	Eukaryotic translation initiation factor 3, subunit E interacting protein	IPI00465233	0.000	0.31207	33	24	27	30
990	Gamma-glutamyl hydrolase	IPI00023728	0.000	0.31207	24	29	21	32
991	60S ribosomal protein L23a	IPI00021266	0.000	0.31207	18	25	15	28
992	Tyrosyl-tRNA synthetase, cytoplasmic	IPI00007074	0.000	0.31207	15	16	17	14
993	Isoform 2 of Titin	IPI00023283	0.000	0.31207	0	2	0	2
994	GMP synthase [glutamine-hydrolyzing]	IPI00029079	0.000	0.31207	19	16	15	20
995	Isoform Beta of Lamina-associated polypeptide 2, isoforms beta/gamma	IPI00030131	0.000	0.31207	20	21	18	23
996	Phosphoserine aminotransferase	IPI00001734	0.000	0.31207	15	19	17	17
997	Isoform SM-B' of Small nuclear ribonucleoprotein-associated proteins B and B'	IPI00027285	0.000	0.31207	15	19	15	19
998	Superkiller viralicidic activity 2-like 2	IPI00647217	0.000	0.31207	15	11	11	15
999	DNA replication licensing factor MCM4	IPI00018349	0.000	0.31207	20	18	15	23
1000	Isoform 1 of General transcription factor II-I	IPI00054042	0.000	0.31207	18	21	20	19
1001	60S ribosomal protein L18a	IPI00026202	0.000	0.31207	3	8	5	6
1002	Dolichyl-diphosphooligosaccharide--protein glycosyltransferase subunit STT3B	IPI00152377	0.000	0.31207	12	8	9	11
1003	KH-type splicing regulatory protein	IPI00479786	0.000	0.31207	17	16	16	17
1004	Isoform 2 of Structural maintenance of chromosomes protein 4	IPI00328298	0.000	0.31207	14	15	11	18
1005	10 kDa heat shock protein, mitochondrial	IPI00220362	0.000	0.31207	7	12	8	11
1006	DNA-directed RNA polymerase II subunit RPB1	IPI00031627	0.000	0.31207	14	11	10	15
1007	Isoform 1 of ATP-binding cassette sub-family D member 3	IPI00002372	0.000	0.31207	15	17	14	18
1008	Isoform 2 of 6-phosphofructokinase, muscle type	IPI00219585	0.000	0.31207	16	13	14	15
1009	DNA mismatch repair protein Msh2	IPI00017303	0.000	0.31207	14	11	15	10
1010	Replication factor C subunit 5	IPI00031514	0.000	0.31207	20	15	18	17
1011	Isoform Mitochondrial of Glutathione reductase, mitochondrial	IPI00016862	0.000	0.31207	12	10	11	11
1012	UMP-CMP kinase isoform a	IPI00219953	0.000	0.31207	10	19	12	17
1013	Isoform 2 of Myosin-Ic	IPI00010418	0.000	0.31207	12	14	13	13
1014	Putative RNA-binding protein 3	IPI00024320	0.000	0.31207	9	11	9	11
1015	Cytochrome c oxidase subunit 2	IPI00017510	0.000	0.31207	11	14	12	13
1016	DNA replication licensing factor MCM6	IPI00031517	0.000	0.31207	11	15	15	11
1017	Isoform 1 of Splicing factor U2AF 65 kDa subunit	IPI00031556	0.000	0.31207	13	11	8	16
1018	DnaJ homolog subfamily C member 13	IPI00307259	0.000	0.31207	13	12	13	12
1019	Isoform 1 of Proteasome activator complex subunit 3	IPI00030243	0.000	0.31207	13	12	11	14
1020	cDNA FLJ56307, highly similar to Ubiquitin thioesterase protein OTUB1	IPI00000581	0.000	0.31207	12	12	11	13
1021	40S ribosomal protein S19	IPI00215780	0.000	0.31207	8	13	11	10
1022	Insulin-like growth factor 2 mRNA-binding protein 1	IPI00008557	0.000	0.31207	10	15	13	12
1023	DKFZP586J0619 protein	IPI00740961	0.000	0.31207	9	11	10	10
1024	cDNA FLJ55599, highly similar to DNA replication licensing factor MCM3	IPI00013214	0.000	0.31207	13	11	11	13
1025	Isoform Alpha of Signal transducer and activator of transcription 1-alpha/beta	IPI00030781	0.000	0.31207	11	12	11	12
1026	Isoform 1 of Carnitine O-palmitoyltransferase 1, liver isoform	IPI00032038	0.000	0.31207	6	8	7	7
1027	40S ribosomal protein S25	IPI00012750	0.000	0.31207	5	12	9	8
1028	Echinoderm microtubule-associated protein-like 4	IPI00001466	0.000	0.31207	7	5	5	7
1029	Protein RCC2	IPI00465044	0.000	0.31207	6	4	4	6
1030	Eukaryotic translation initiation factor 2 subunit 3	IPI00297982	0.000	0.31207	9	8	8	9
1031	V-type proton ATPase catalytic subunit A	IPI00007682	0.000	0.31207	13	13	12	14
1032	Nuclear migration protein nudC	IPI00550746	0.000	0.31207	12	13	13	12
1033	Isoform 1 of Proteasome activator complex subunit 4	IPI00005260	0.000	0.31207	9	9	9	9
1034	Isoform 1 of 5'-3' exoribonuclease 2	IPI00100151	0.000	0.31207	9	4	5	8
1035	SWI/SNF-related matrix-associated actin-dependent regulator of chromatin subfamily A member 5	IPI00297211	0.000	0.31207	6	7	5	8

No.	Description	Accession number	STN[1]	p-Value[1]	Con_A[2]	Con_B[2]	SORA_A[2]	SORA_B[2]
1036	Ubiquitin-conjugating enzyme E2 N	IPI00003949	0.000	0.31207	7	10	8	9
1037	Chromobox protein homolog 3	IPI00297579	0.000	0.31207	8	10	7	11
1038	coatomer subunit epsilon isoform b	IPI00399318	0.000	0.31207	8	6	6	8
1039	Isoform 1 of Cytoplasmic FMR1-interacting protein 1	IPI00644231	0.000	0.31207	10	7	5	12
1040	Dolichyl-diphosphooligosaccharide--protein glycosyltransferase subunit STT3A	IPI00297492	0.000	0.31207	4	4	0	6
1041	Isoform 2 of Liprin-beta-1	IPI00179172	0.000	0.31207	6	9	5	10
1042	Isoform 1 of Serum paraoxonase/arylesterase 2	IPI00014958	0.000	0.31207	2	0	0	2
1043	Isoform 1 of UTP--glucose-1-phosphate uridylyltransferase	IPI00329331	0.000	0.31207	7	11	7	11
1044	Importin 5	IPI00514205	0.000	0.31207	7	9	6	10
1045	Ras-related protein Rab-14	IPI00291928	0.000	0.31207	7	9	7	9
1046	Actin-related protein 2/3 complex subunit 3	IPI00005162	0.000	0.31207	6	8	6	8
1047	Helicase SKI2W	IPI00414819	0.000	0.31207	6	6	4	8
1048	Isoform 1 of Bifunctional coenzyme A synthase	IPI00184821	0.000	0.31207	8	7	7	8
1049	Vesicle-trafficking protein SEC22b	IPI00006865	0.000	0.31207	9	8	8	9
1050	Inositol monophosphatase 1	IPI00020906	0.000	0.31207	6	5	5	6
1051	Isoform 1 of Peroxisomal acyl-coenzyme A oxidase 1	IPI00296907	0.000	0.31207	5	8	6	7
1052	twinfilin-1	IPI00183508	0.000	0.31207	9	7	8	8
1053	EF-hand domain-containing protein D2	IPI00060181	0.000	0.31207	8	7	8	7
1054	WASH complex subunit strumpellin	IPI00029175	0.000	0.31207	5	8	6	7
1055	Putative uncharacterized protein ENSP00000382160	IPI00180956	0.000	0.31207	0	0	0	0
1056	Putative uncharacterized protein FUBP3	IPI00063245	0.000	0.31207	5	0	0	5
1057	Isoform 1 of Uridine 5'-monophosphate synthase	IPI00003923	0.000	0.31207	6	6	7	5
1058	LEM domain-containing protein 2	IPI00168336	0.000	0.31207	0	2	0	2
1059	THO complex subunit 2	IPI00158615	0.000	0.31207	5	7	5	7
1060	Isoform 1 of Ubiquitin-conjugating enzyme E2 K	IPI00021370	0.000	0.31207	11	10	9	12
1061	Adenylate kinase isoenzyme 1	IPI00018342	0.000	0.31207	7	11	7	11
1062	Synaptotagmin-1	IPI00009439	0.000	0.31207	7	7	5	9
1063	Aspartyl/asparaginyl beta-hydroxylase	IPI00294834	0.000	0.31207	3	4	3	4
1064	Tubulin-specific chaperone A	IPI00217236	0.000	0.31207	7	9	8	8
1065	Importin-11	IPI00301107	0.000	0.31207	4	6	6	4
1066	triosephosphate isomerase 1 isoform 2	IPI00465028	0.000	0.31207	0	7	3	6
1067	Crk-like protein	IPI00004839	0.000	0.31207	6	9	7	8
1068	High mobility group protein B2	IPI00219097	0.000	0.31207	4	5	6	3
1069	Ras-related protein Rab-1B	IPI00008964	0.000	0.31207	3	8	4	7
1070	Vacuolar protein-sorting-associated protein 25	IPI00031655	0.000	0.31207	4	4	4	4
1071	Isoform A of Ras GTPase-activating protein-binding protein 2	IPI00009057	0.000	0.31207	6	4	5	5
1072	Isoform 2 of Obg-like ATPase 1	IPI00216105	0.000	0.31207	8	10	7	11
1073	Isoform A of Ras-related C3 botulinum toxin substrate 1	IPI00010271	0.000	0.31207	3	7	3	7
1074	Anaphase-promoting complex subunit 1	IPI00033907	0.000	0.31207	0	0	0	2
1075	24 kDa protein	IPI00398057	0.000	0.31207	5	9	7	7
1076	Histidine triad nucleotide-binding protein 1	IPI00239077	0.000	0.31207	5	5	5	5
1077	Cytochrome c1, heme protein, mitochondrial	IPI00029264	0.000	0.31207	4	5	4	5
1078	3-mercaptopyruvate sulfurtransferase	IPI00165360	0.000	0.31207	4	7	4	7
1079	Isoform 1 of Pescadillo homolog	IPI00003768	0.000	0.31207	6	3	6	3
1080	cDNA FLJ56825, highly similar to WD repeat protein 57	IPI00006723	0.000	0.31207	5	6	5	6
1081	Isoform Delta-1 of Serine/threonine-protein phosphatase 2A 56 kDa regulatory subunit delta isoform	IPI00000030	0.000	0.31207	4	3	3	4
1082	Casein kinase II subunit alpha'	IPI00020602	0.000	0.31207	5	3	5	3
1083	39S ribosomal protein L49, mitochondrial	IPI00013195	0.000	0.31207	5	8	6	7
1084	GTP-binding protein SAR1b	IPI00002149	0.000	0.31207	6	6	7	5
1085	Isoform 2 of Guanine nucleotide-binding protein-like 3	IPI00003886	0.000	0.31207	6	6	6	6
1086	Na(+)/H(+) exchange regulatory cofactor NHE-RF1	IPI00003527	0.000	0.31207	6	5	5	6
1087	ATP-binding cassette sub-family F member 2	IPI00005045	0.000	0.31207	5	3	3	5
1088	Protein NipSnap homolog 1	IPI00304435	0.000	0.31207	4	7	6	5
1089	UBX domain-containing protein 7	IPI00742124	0.000	0.31207	5	4	5	4
1090	Isoform 1 of UBX domain-containing protein 1	IPI00027378	0.000	0.31207	8	6	7	7
1091	Golgi phosphoprotein 3	IPI00005490	0.000	0.31207	6	6	6	6
1092	Isoform 2 of Phosphatidylinositol-binding clathrin assembly protein	IPI00216184	0.000	0.31207	5	3	0	6
1093	CDNA FLJ20030 fis, clone ADSU02156	IPI00014402	0.000	0.31207	2	2	0	2
1094	Inositol 1,4,5-trisphosphate receptor type 3	IPI00291607	0.000	0.31207	2	0	0	0
1095	Isoform 1 of Peripherin	IPI00013164	0.000	0.31207	4	6	4	6
1096	Ribose-5-phosphate isomerase	IPI00026513	0.000	0.31207	6	0	4	4
1097	CLASP2 protein	IPI00168165	0.000	0.31207	4	6	3	7
1098	Isoform 2 of RANBP2-like and GRIP domain-containing protein 5/6	IPI00100787	0.000	0.31207	0	0	0	0
1099	Nucleolar protein 11	IPI00303813	0.000	0.31207	3	3	2	4
1100	Uncharacterized protein C18orf19	IPI00290799	0.000	0.31207	0	5	0	5
1101	Ubiquitin-fold modifier-conjugating enzyme 1	IPI00294495	0.000	0.31207	3	0	3	0
1102	cDNA FLJ55382, highly similar to Hsp70-binding protein 1	IPI00100748	0.000	0.31207	5	3	3	5
1103	cohesin subunit SA-2 isoform a	IPI00470883	0.000	0.31207	6	4	6	4
1104	Isoform 1 of POTE ankyrin domain family member E	IPI00479743	0.000	0.31207	0	0	0	0
1105	28S ribosomal protein S31, mitochondrial	IPI00294242	0.000	0.31207	3	7	4	6
1106	Nuclear transport factor 2	IPI00009901	0.000	0.31207	4	0	4	0
1107	Putative uncharacterized protein KIAA0090	IPI00640734	0.000	0.31207	2	4	2	4
1108	Isoform 1 of Heterogeneous nuclear ribonucleoprotein U-like protein 1	IPI00013070	0.000	0.31207	6	4	4	6
1109	Ribose-phosphate pyrophosphokinase 3	IPI00218371	0.000	0.31207	4	5	3	6
1110	synembryn-A	IPI00100106	0.000	0.31207	4	7	4	7
1111	Isoform 3 of Protein VPRBP	IPI00181396	0.000	0.31207	2	5	3	4
1112	Isoform 1 of DAZ-associated protein 1	IPI00165230	0.000	0.31207	4	3	4	3
1113	myosin-IXb isoform 1	IPI00306933	0.000	0.31207	7	3	5	5
1114	NADH dehydrogenase [ubiquinone] iron-sulfur protein 8, mitochondrial	IPI00010845	0.000	0.31207	3	5	3	5
1115	Thioredoxin-like protein 1	IPI00305692	0.000	0.31207	0	2	0	2
1116	DNA-directed RNA polymerase, mitochondrial precursor	IPI00298738	0.000	0.31207	4	2	2	4
1117	Isoform 1 of Spermine synthase	IPI00005102	0.000	0.31207	5	7	7	5
1118	Isoform 1 of Apoptosis-inducing factor 2	IPI00013909	0.000	0.31207	5	2	5	2
1119	Methyltransferase like 7B	IPI00090807	0.000	0.31207	3	0	2	3
1120	Isoform 1 of RNA-binding protein with serine-rich domain 1	IPI00033561	0.000	0.31207	3	5	4	4
1121	Small acidic protein	IPI00003419	0.000	0.31207	5	6	5	6
1122	cDNA FLJ38069 fis, clone CTONG2015434, highly similar to DOUBLE-STRAND BREAK REPAIR PROTEIN	IPI00029159	0.000	0.31207	4	3	3	4
1123	Isoform 1 of Telomeric repeat-binding factor 2	IPI00024214	0.000	0.31207	4	4	4	4
1124	Isoform 1 of Cullin-4A	IPI00419273	0.000	0.31207	4	4	4	4
1125	COP9 signalosome complex subunit 6	IPI00163230	0.000	0.31207	4	0	3	3
1126	Heat shock-related 70 kDa protein 2	IPI00007702	0.000	0.31207	4	5	3	6
1127	Proteasome subunit beta type-7	IPI00003217	0.000	0.31207	3	4	5	0
1128	Isoform 1 of Transmembrane protein 70, mitochondrial	IPI00106966	0.000	0.31207	0	2	0	2

No.	Description	Accession number	STN[1]	p-Value[1]	Con_A[2]	Con_B[2]	SORA_A[2]	SORA_B[2]
1129	Isoform 2 of 1,2-dihydroxy-3-keto-5-methylthiopentene dioxygenase	IPI00470791	0.000	0.31207	0	0	0	0
1130	Isoform 1 of 1,2-dihydroxy-3-keto-5-methylthiopentene dioxygenase	IPI00651738	0.000	0.31207	0	2	0	0
1131	ATP-dependent RNA helicase DDX24	IPI00006987	0.000	0.31207	2	2	2	2
1132	Isoform 1 of Porphobilinogen deaminase	IPI00028160	0.000	0.31207	3	6	4	5
1133	COP9 signalosome complex subunit 7a	IPI00301419	0.000	0.31207	4	4	4	4
1134	SRA stem-loop-interacting RNA-binding protein, mitochondrial	IPI00009922	0.000	0.31207	3	7	4	6
1135	Peptidyl-prolyl cis-trans isomerase FKBP11	IPI00009885	0.000	0.31207	2	2	2	0
1136	39S ribosomal protein L44, mitochondrial	IPI00009680	0.000	0.31207	4	5	3	6
1137	WD repeat-containing protein 18	IPI00032533	0.000	0.31207	0	2	0	2
1138	Actin-related protein 2/3 complex subunit 1B	IPI00005160	0.000	0.31207	0	0	2	0
1139	74 kDa protein	IPI00290439	0.000	0.31207	4	3	2	5
1140	Isoform 1 of Trans-2,3-enoyl-CoA reductase	IPI00100656	0.000	0.31207	0	2	2	0
1141	Epithelial cell adhesion molecule	IPI00296215	0.000	0.31207	3	3	3	3
1142	60S ribosomal protein L19	IPI00025329	0.000	0.31207	4	0	0	4
1143	Pseudouridylate synthase 7 homolog	IPI00044761	0.000	0.31207	2	6	5	3
1144	Dual specificity mitogen-activated protein kinase kinase 1	IPI00219604	0.000	0.31207	3	5	4	4
1145	Mediator of RNA polymerase II transcription subunit 14	IPI00297191	0.000	0.31207	2	0	0	0
1146	Ribosomal protein L1	IPI00035167	0.000	0.31207	0	2	0	0
1147	Isoform 3 of Protein scribble homolog	IPI00410666	0.000	0.31207	4	5	3	6
1148	Isoform 1 of Protein-tyrosine phosphatase mitochondrial 1	IPI00174190	0.000	0.31207	0	0	0	2
1149	Protein FAM162A	IPI00023001	0.000	0.31207	5	2	4	3
1150	Isoform 6 of GTPase-activating protein and VPS9 domain-containing protein 1	IPI00292753	0.000	0.31207	4	5	2	7
1151	Nuclear pore glycoprotein p62	IPI00293533	0.000	0.31207	5	6	6	5
1152	cDNA FLJ50992, highly similar to Coronin-1C	IPI00798401	0.000	0.31207	2	0	2	2
1153	Sodium/myo-inositol cotransporter	IPI00296311	0.000	0.31207	0	2	0	2
1154	Isoform 1 of Origin recognition complex subunit 3	IPI00294402	0.000	0.31207	5	3	2	6
1155	Cytochrome c-type heme lyase	IPI00023406	0.000	0.31207	7	2	5	4
1156	Scaffold attachment factor B2	IPI00005648	0.000	0.31207	3	3	0	4
1157	Isoform 1 of Phosphatidylinositol-3,4,5-trisphosphate 5-phosphatase 1	IPI00329213	0.000	0.31207	4	3	3	4
1158	SF3A2 protein (Fragment)	IPI00017341	0.000	0.31207	3	2	2	3
1159	60S ribosomal protein L31	IPI00026302	0.000	0.31207	0	0	0	2
1160	Isoform 1 of Vacuolar protein sorting-associated protein 8 homolog	IPI00464985	0.000	0.31207	0	2	2	0
1161	Isoform 1 of Fermitin family homolog 1	IPI00304754	0.000	0.31207	0	0	0	2
1162	Isoform 1 of Apoptotic protease-activating factor 1	IPI00023630	0.000	0.31207	0	0	0	2
1163	Calcium-regulated heat stable protein 1	IPI00304409	0.000	0.31207	0	2	2	2
1164	TP53-regulating kinase	IPI00290305	0.000	0.31207	2	4	3	3
1165	TRMT61A protein (Fragment)	IPI00059718	0.000	0.31207	0	4	2	4
1166	Isoform 1 of Nicalin	IPI00470649	0.000	0.31207	2	3	2	3
1167	28S ribosomal protein S26, mitochondrial	IPI00006606	0.000	0.31207	3	6	4	5
1168	Isoform 2 of Neurofibromin	IPI00299512	0.000	0.31207	0	0	0	2
1169	Phosphatidylinositol-5-phosphate 4-kinase type-2 gamma	IPI00152303	0.000	0.31207	2	2	0	0
1170	Isoform Long of ES1 protein homolog, mitochondrial	IPI00024913	0.000	0.31207	2	4	2	4
1171	Isoform 1 of Specifically androgen-regulated gene protein	IPI00028392	0.000	0.31207	4	4	5	3
1172	Sterol-4-alpha-carboxylate 3-dehydrogenase, decarboxylating	IPI00019407	0.000	0.31207	3	4	4	3
1173	28S ribosomal protein S34, mitochondrial	IPI00169413	0.000	0.31207	3	3	3	3
1174	Synaptobrevin homolog YKT6	IPI00008569	0.000	0.31207	2	0	2	0
1175	Isoform 2 of Cleft lip and palate transmembrane protein 1	IPI00107357	0.000	0.31207	2	2	2	0
1176	Heat shock protein beta-11	IPI00098827	0.000	0.31207	4	5	4	5
1177	Nucleolar protein 9	IPI00002902	0.000	0.31207	0	0	2	0
1178	Isoform 2 of Chromodomain-helicase-DNA-binding protein 2	IPI00023109	0.000	0.31207	2	0	2	0
1179	Desmoglein-2	IPI00028931	0.000	0.31207	5	2	4	3
1180	Isoform 2 of Golgi apparatus protein 1	IPI00414717	0.000	0.31207	3	3	2	4
1181	60S ribosomal protein L30	IPI00219156	0.000	0.31207	4	5	4	5
1182	bifunctional protein NCOAT isoform b	IPI00181391	0.000	0.31207	3	0	3	2
1183	Isoform 1 of OCIA domain-containing protein 1	IPI00016405	0.000	0.31207	0	0	2	2
1184	Bifunctional methylenetetrahydrofolate dehydrogenase/cyclohydrolase, mitochondrial	IPI00011307	0.000	0.31207	3	5	3	5
1185	Splicing factor 3B subunit 4	IPI00017339	0.000	0.31207	3	2	0	3
1186	Peptidyl-prolyl cis-trans isomerase F, mitochondrial	IPI00026519	0.000	0.31207	6	4	4	6
1187	N-alpha-acetyltransferase 10, NatA catalytic subunit	IPI00013184	0.000	0.31207	2	0	2	0
1188	Ribosome production factor 2 homolog	IPI00396329	0.000	0.31207	0	2	2	0
1189	Talin-2	IPI00219299	0.000	0.31207	4	2	3	3
1190	Transmembrane protein 2	IPI00170706	0.000	0.31207	3	2	3	2
1191	Isoform 1 of Liprin-alpha-1	IPI00163496	0.000	0.31207	3	2	2	3
1192	Transcription initiation factor IIE subunit beta	IPI00019981	0.000	0.31207	3	0	3	0
1193	Nuclear RNA export factor 1	IPI00033153	0.000	0.31207	3	6	5	4
1194	Adenylate kinase isoenzyme 4, mitochondrial	IPI00016568	0.000	0.31207	0	0	2	0
1195	cDNA FLJ61162, highly similar to Ras-related protein R-Ras2	IPI00012512	0.000	0.31207	2	0	0	2
1196	U6 snRNA-associated Sm-like protein LSm4	IPI00294955	0.000	0.31207	2	4	2	4
1197	Isoform 2 of Peptidyl-prolyl cis-trans isomerase-like 3	IPI00032473	0.000	0.31207	4	3	4	3
1198	NADH dehydrogenase [ubiquinone] 1 beta subcomplex subunit 4	IPI00220059	0.000	0.31207	4	5	5	4
1199	DNA-directed RNA polymerases I, II, and III subunit RPABC3	IPI00003309	0.000	0.31207	2	6	4	4
1200	CDGSH iron sulfur domain-containing protein 1	IPI00020510	0.000	0.31207	5	4	5	4
1201	PH-interacting protein	IPI00291916	0.000	0.31207	2	2	0	0
1202	Isoform 2 of Condensin-2 complex subunit G2	IPI00396058	0.000	0.31207	2	0	0	0
1203	EF-hand domain-containing protein D1	IPI00031091	0.000	0.31207	2	2	2	2
1204	Isoform 1 of Exosome component 10	IPI00009464	0.000	0.31207	4	0	0	4
1205	Isoform 1 of Gamma-tubulin complex component 3	IPI00033516	0.000	0.31207	4	3	0	5
1206	Isoform Beta-1C of Integrin beta-1	IPI00217561	0.000	0.31207	3	0	0	3
1207	Ubiquilin-2	IPI00409659	0.000	0.31207	4	5	4	5
1208	Beta-lactamase-like protein 2	IPI00006952	0.000	0.31207	2	0	0	2
1209	Protein C20orf11	IPI00016634	0.000	0.31207	3	5	4	4
1210	cDNA FLJ55543, highly similar to Phosphoacetylglucosamine mutase	IPI00030116	0.000	0.31207	3	0	2	3
1211	Pre-mRNA branch site protein p14	IPI00032827	0.000	0.31207	2	6	4	4
1212	Splicing factor, arginine/serine-rich 4	IPI00000015	0.000	0.31207	0	2	0	2
1213	24-dehydrocholesterol reductase	IPI00016703	0.000	0.31207	2	2	2	2
1214	Cation-independent mannose-6-phosphate receptor	IPI00289819	0.000	0.31207	2	2	2	0
1215	Isoform 2 of Glutaminase kidney isoform, mitochondrial	IPI00215685	0.000	0.31207	0	3	3	0
1216	Chitobiosyldiphosphodolichol beta-mannosyltransferase	IPI00549761	0.000	0.31207	0	0	0	0
1217	Mediator of RNA polymerase II transcription subunit 12	IPI00004068	0.000	0.31207	2	0	2	0
1218	DCN1-like protein 1	IPI00291893	0.000	0.31207	4	4	4	4
1219	Isoform 1 of Zinc phosphodiesterase ELAC protein 2	IPI00396627	0.000	0.31207	2	3	2	3
1220	Aldo-keto reductase family 1 member C1	IPI00029733	0.000	0.31207	4	3	0	5
1221	U3 small nucleolar ribonucleoprotein protein IMP3	IPI00019488	0.000	0.31207	5	5	5	5
1222	cDNA FLJ55475	IPI00306017	0.000	0.31207	0	2	0	2
1223	General transcription factor 3C polypeptide 4	IPI00016725	0.000	0.31207	0	2	0	0

No.	Description	Accession number	STN[1]	p-Value[1]	Con_A[2]	Con_B[2]	SORA_A[2]	SORA_B[2]
1224	Isoform 1 of AP-3 complex subunit delta-1	IPI00411453	0.000	0.31207	2	2	0	2
1225	Large neutral amino acids transporter small subunit 1	IPI00008986	0.000	0.31207	2	2	0	0
1226	Copper chaperone for superoxide dismutase	IPI00021389	0.000	0.31207	0	0	2	0
1227	Plexin B2	IPI00852623	0.000	0.31207	0	0	2	2
1228	Isoform 1 of ADP-ribosylation factor-like protein 2-binding protein	IPI00015866	0.000	0.31207	2	0	0	0
1229	ATP-dependent RNA helicase DHX8	IPI00031508	0.000	0.31207	3	3	4	0
1230	Aldehyde dehydrogenase, dimeric NADP-preferring	IPI00296183	0.000	0.31207	5	3	3	5
1231	Isoform 1 of Probable ATP-dependent RNA helicase DHX36	IPI00027415	0.000	0.31207	3	3	2	4
1232	DNA-directed RNA polymerase III subunit RPC1	IPI00024163	0.000	0.31207	2	2	0	2
1233	Putative uncharacterized protein DOCK6	IPI00184772	0.000	0.31207	0	0	0	2
1234	18 kDa protein	IPI00797709	0.000	0.31207	0	0	2	2
1235	Isoform 1 of Lysophospholipase-like protein 1	IPI00059762	0.000	0.31207	0	0	0	0
1236	Probable ATP-dependent RNA helicase DDX56	IPI00302281	0.000	0.31207	0	0	0	2
1237	Serine/threonine-protein kinase 38-like	IPI00237011	0.000	0.31207	2	4	0	4
1238	Centromere/kinetochore protein zw10 homolog	IPI00011631	0.000	0.31207	4	4	4	4
1239	D-3-phosphoglycerate dehydrogenase	IPI00011200	0.000	0.31207	4	5	4	5
1240	V-type proton ATPase subunit G 1	IPI00025285	0.000	0.31207	0	5	3	4
1241	ubiquitin-like protein fubi and ribosomal protein S30 precursor	IPI00019770	0.000	0.31207	2	2	2	2
1242	Isoform 1 of Protein CDV3 homolog	IPI00014197	0.000	0.31207	2	0	0	2
1243	Isoform 2 of Actin-binding protein anillin	IPI00032958	0.000	0.31207	2	2	0	0
1244	Isoform 1 of Ral GTPase-activating protein subunit beta	IPI00409601	0.000	0.31207	0	0	2	0
1245	Putative uncharacterized protein ZFR	IPI00748303	0.000	0.31207	2	3	3	2
1246	Isoform 1 of L-2-hydroxyglutarate dehydrogenase, mitochondrial	IPI00016458	0.000	0.31207	5	2	3	4
1247	Survival of motor neuron-related-splicing factor 30	IPI00025176	0.000	0.31207	3	3	3	3
1248	Growth arrest and DNA damage-inducible proteins-interacting protein 1	IPI00552587	0.000	0.31207	3	4	4	3
1249	Isoform 2 of Syntaxin-5	IPI00386786	0.000	0.31207	0	2	0	0
1250	Serine/threonine-protein kinase PLK1	IPI00021248	0.000	0.31207	2	0	0	0
1251	Uncharacterized protein C18orf8	IPI00149964	0.000	0.31207	0	0	0	2
1252	ATP synthase subunit delta, mitochondrial	IPI00024920	0.000	0.31207	0	0	0	0
1253	3'-5' exoribonuclease CSL4 homolog	IPI00032823	0.000	0.31207	3	3	3	3
1254	Dimethyladenosine transferase 1, mitochondrial	IPI00291525	0.000	0.31207	2	0	2	0
1255	Pre-mRNA-splicing factor CWC22 homolog	IPI00177381	0.000	0.31207	2	0	2	2
1256	Uncharacterized protein KIAA1797	IPI00748360	0.000	0.31207	3	0	0	3
1257	Methylosome protein 50	IPI00012202	0.000	0.31207	2	3	3	2
1258	Isoform 1 of Ubiquitin conjugation factor E4 B	IPI00005715	0.000	0.31207	0	2	0	0
1259	Zinc finger protein 622	IPI00056499	0.000	0.31207	3	2	3	2
1260	Vacuolar protein sorting-associated protein VTA1 homolog	IPI00017160	0.000	0.31207	2	3	2	3
1261	Isoform 1 of WD repeat-containing protein 44	IPI00444371	0.000	0.31207	0	0	0	0
1262	NADH-ubiquinone oxidoreductase chain 5	IPI00008511	0.000	0.31207	0	2	2	2
1263	Integrator complex subunit 2	IPI00477759	0.000	0.31207	0	0	0	0
1264	39S ribosomal protein L37, mitochondrial	IPI00162330	0.000	0.31207	2	2	2	0
1265	DNA polymerase alpha subunit B	IPI00290272	0.000	0.31207	0	2	0	0
1266	mesencephalic astrocyte-derived neurotrophic factor	IPI00328748	0.000	0.31207	2	2	2	0
1267	Nucleoporin Nup37	IPI00171665	0.000	0.31207	2	2	0	0
1268	Isoform 1 of Short-chain dehydrogenase/reductase 3	IPI00006250	0.000	0.31207	3	2	3	0
1269	Probable ATP-dependent RNA helicase DDX28	IPI00020050	0.000	0.31207	3	2	0	3
1270	Sulfide:quinone oxidoreductase, mitochondrial	IPI00009634	0.000	0.31207	4	4	5	3
1271	Isoform 1 of Tumor susceptibility gene 101 protein	IPI00018434	0.000	0.31207	3	2	2	3
1272	cDNA FLJ55177, highly similar to Ras-related protein Ral-B	IPI00004397	0.000	0.31207	3	0	0	3
1273	39S ribosomal protein L18, mitochondrial	IPI00160421	0.000	0.31207	5	3	5	3
1274	Isoform Long of Inositol 1,4,5-trisphosphate receptor type 2	IPI00031545	0.000	0.31207	2	0	0	0
1275	Isoform 3 of Yorkie homolog	IPI00009326	0.000	0.31207	2	2	0	0
1276	M-phase phosphoprotein 6	IPI00016074	0.000	0.31207	0	0	0	2
1277	Isoform 3 of Anamorsin	IPI00025333	0.000	0.31207	0	0	0	0
1278	Isoform 1 of Glucosamine-6-phosphate isomerase 2	IPI00550894	0.000	0.31207	2	0	2	2
1279	cDNA FLJ52725, highly similar to Magnesium transporter MRS2L, mitochondrial	IPI00010188	0.000	0.31207	0	0	0	0
1280	Gamma-taxilin	IPI00019994	0.000	0.31207	0	2	0	0
1281	Isoform 4 of Death-inducer obliterator 1	IPI00619921	0.000	0.31207	0	2	0	0
1282	Isoform 1 of COMM domain-containing protein 4	IPI00413500	0.000	0.31207	2	3	0	3
1283	Probable O-sialoglycoprotein endopeptidase	IPI00015809	0.000	0.31207	2	2	2	0
1284	Phosphomannomutase 2	IPI00006092	0.000	0.31207	2	2	2	2
1285	Tetratricopeptide repeat protein 9C	IPI00175096	0.000	0.31207	2	0	0	0
1286	Cytochrome c oxidase subunit 1	IPI00464968	0.000	0.31207	0	0	2	2
1287	E3 ubiquitin-protein ligase KCMF1	IPI00306661	0.000	0.31207	0	0	0	0
1288	Putative uncharacterized protein DKFZp686E2459	IPI00375731	0.000	0.31207	0	0	0	2
1289	UPF0364 protein C6orf211	IPI00002270	0.000	0.31207	2	0	0	0
1290	WD repeat and HMG-box DNA-binding protein 1	IPI00411614	0.000	0.31207	2	2	2	2
1291	Protoporphyrinogen oxidase	IPI00031357	0.000	0.31207	0	0	2	0
1292	Cell division protein kinase 2	IPI00031681	0.000	0.31207	2	2	0	0
1293	Isoform 1 of NADH dehydrogenase [ubiquinone] flavoprotein 1, mitochondrial	IPI00028520	0.000	0.31207	3	2	3	2
1294	Isoform 2 of Suppressor of SWI4 1 homolog	IPI00219793	0.000	0.31207	0	0	0	2
1295	Hepatoma-derived growth factor-related protein 3	IPI00007063	0.000	0.31207	0	2	0	0
1296	Major facilitator superfamily domain-containing protein 10	IPI00103940	0.000	0.31207	2	2	0	0
1297	Isoform A of GC-rich sequence DNA-binding factor homolog	IPI00001364	0.000	0.31207	2	0	2	2
1298	Isoform 2 of Uncharacterized protein C3orf63	IPI00745978	0.000	0.31207	3	2	3	0
1299	Ubiquitin-like protein 4A	IPI00005658	0.000	0.31207	2	2	2	0
1300	Ras-related protein Rab-9A	IPI00016372	0.000	0.31207	3	4	3	4
1301	X-Pro aminopeptidase 1, soluble isoform 2	IPI00607814	0.000	0.31207	2	2	2	2
1302	Nucleoporin NUP53	IPI00329650	0.000	0.31207	0	4	2	4
1303	Isoform 1 of Syntenin-1	IPI00299086	0.000	0.31207	3	3	2	4
1304	Coiled-coil domain-containing protein 25	IPI00396174	0.000	0.31207	2	4	4	2
1305	Keratin, type II cytoskeletal 2 oral	IPI00008359	0.000	0.31207	0	2	0	0
1306	fatty acid desaturase 1	IPI00784651	0.000	0.31207	0	2	0	0
1307	Isoform 1 of E3 ubiquitin-protein ligase RNF123	IPI00335085	0.000	0.31207	2	2	0	2
1308	Protein FADD	IPI00011919	0.000	0.31207	0	2	0	0
1309	GDP-L-fucose synthase	IPI00014361	0.000	0.31207	2	0	0	2
1310	DNA mismatch repair protein Msh3	IPI00329605	0.000	0.31207	0	0	0	0
1311	inosine-5'-monophosphate dehydrogenase 1 isoform a	IPI00375527	0.000	0.31207	0	3	2	3
1312	Isoform 2 of DnaJ homolog subfamily C member 2	IPI00455199	0.000	0.31207	2	5	4	3
1313	Peroxisomal membrane protein PMP34	IPI00014440	0.000	0.31207	3	0	0	3
1314	E3 ubiquitin-protein ligase BRE1A	IPI00251559	0.000	0.31207	2	0	2	0
1315	Isoform 1 of Structural maintenance of chromosomes protein 4	IPI00411559	0.000	0.31207	2	2	0	2
1316	Isoform Alpha of Nuclear inhibitor of protein phosphatase 1	IPI00030383	0.000	0.31207	2	0	0	2
1317	Isoform 2 of Plakophilin-2	IPI00005264	0.000	0.31207	2	0	0	0
1318	Isoform 1 of Cytosolic non-specific dipeptidase	IPI00177728	0.000	0.31207	0	4	3	3

No.	Description	Accession number	STN[1]	p-Value[1]	Con_A[2]	Con_B[2]	SORA_A[2]	SORA_B[2]
1319	NADH dehydrogenase [ubiquinone] 1 beta subcomplex subunit 9	IPI00255052	0.000	0.31207	0	2	0	2
1320	Isoform 2 of Leucine-rich repeat flightless-interacting protein 1	IPI00006207	0.000	0.31207	0	0	2	2
1321	Armadillo repeat-containing protein 1	IPI00018260	0.000	0.31207	2	2	0	2
1322	Anoctamin-6	IPI00151710	0.000	0.31207	0	0	0	2
1323	Transmembrane 9 superfamily member 4	IPI00021985	0.000	0.31207	0	0	0	0
1324	Isoform 1 of Polyadenylate-binding protein-interacting protein 1	IPI00021466	0.000	0.31207	2	2	0	0
1325	Isoform 7 of Protein BAT2-like 2	IPI00083708	0.000	0.31207	0	0	0	0
1326	cDNA FLJ56047, highly similar to A kinase anchor protein 1, mitochondrial	IPI00022585	0.000	0.31207	0	0	0	0
1327	NEDD8-conjugating enzyme Ubc12	IPI00022597	0.000	0.31207	0	0	0	0
1328	Isoform 1 of Transmembrane emp24 domain-containing protein 4	IPI00296259	0.000	0.31207	2	2	2	2
1329	WD repeat-containing protein 33	IPI00106567	0.000	0.31207	0	2	0	0
1330	Isoform 1 of TIP41-like protein	IPI00745568	0.000	0.31207	0	0	0	2
1331	Putative uncharacterized protein HERC1	IPI00022479	0.000	0.31207	2	0	0	2
1332	Isoform 1 of Protein 4.1	IPI00003921	0.000	0.31207	2	2	2	2
1333	Isoform 1 of Serine/threonine-protein kinase ATR	IPI00412298	0.000	0.31207	2	2	0	0
1334	Isoform 1 of Phosphatidylinositol 4-kinase alpha	IPI00070943	0.000	0.31207	2	2	0	0
1335	Isoform 1 of Alanine aminotransferase 2	IPI00152432	0.000	0.31207	0	2	2	2
1336	37 kDa protein	IPI00032799	0.000	0.31207	2	2	2	2
1337	Probable ATP-dependent RNA helicase DHX37	IPI00217630	0.000	0.31207	2	2	0	2
1338	Transmembrane protein C3orf1	IPI00299387	0.000	0.31207	0	0	2	0
1339	High mobility group protein B3	IPI00217477	0.000	0.31207	2	4	3	3
1340	Isoform 1 of Endophilin-B2	IPI00024540	0.000	0.31207	0	0	2	2
1341	Isoform Long of Beta-glucuronidase	IPI00027745	0.000	0.31207	2	3	0	3
1342	39S ribosomal protein L24, mitochondrial	IPI00514506	0.000	0.31207	2	2	0	2
1343	Calcium homeostasis endoplasmic reticulum protein	IPI00333010	0.000	0.31207	0	2	2	2
1344	dCTP pyrophosphatase 1	IPI00012197	0.000	0.31207	0	2	0	2
1345	ESF1 homolog	IPI00024167	0.000	0.31207	2	0	0	2
1346	Replication protein A 14 kDa subunit	IPI00017373	0.000	0.31207	0	4	0	4
1347	Isoform 2 of Exosome complex exonuclease RRP45	IPI00029697	0.000	0.31207	2	0	2	2
1348	Isoform Sap-mu-0 of Proactivator polypeptide	IPI00012503	0.000	0.31207	0	0	2	0
1349	N-acylsphingosine amidohydrolase (Acid ceramidase) 1, isoform CRA_c	IPI00013698	0.000	0.31207	2	0	0	0
1350	Isoform 1 of FAST kinase domain-containing protein 2	IPI00013735	0.000	0.31207	2	2	0	0
1351	Acyl-protein thioesterase 2	IPI00027032	0.000	0.31207	0	2	0	0
1352	Isoform 3 of Protein PRRC1	IPI00217053	0.000	0.31207	5	2	4	3
1353	UPF0587 protein C1orf123	IPI00016605	0.000	0.31207	0	2	0	2
1354	Ufm1-specific protease 2	IPI00305303	0.000	0.31207	2	2	2	0
1355	Protein FAM91A1	IPI00152671	0.000	0.31207	2	2	2	2
1356	Ribonucleases P/MRP protein subunit POP1	IPI00293331	0.000	0.31207	2	2	0	0
1357	STE20/SPS1-related proline-alanine-rich protein kinase	IPI00004363	0.000	0.31207	0	0	0	2
1358	Isoform GTBP-N of DNA mismatch repair protein Msh6	IPI00384456	0.000	0.31207	0	3	3	0
1359	Similar to Protein SAAL1. Isoform 2	IPI00304935	0.000	0.31207	2	2	0	2
1360	Isoform 2 of Protein FAM36A	IPI00103057	0.000	0.31207	2	2	0	2
1361	DnaJ homolog subfamily C member 3	IPI00006713	0.000	0.31207	0	3	2	3
1362	Isoform 1 of Pre-mRNA-splicing factor 38A	IPI00171390	0.000	0.31207	0	3	0	3
1363	Coiled-coil domain-containing protein 58	IPI00046828	0.000	0.31207	0	0	2	2
1364	Mitochondrial 18 kDa protein	IPI00784376	0.000	0.31207	0	2	0	0
1365	Isoform 1 of Mitochondrial carrier homolog 1	IPI00386258	0.000	0.31207	2	0	0	0
1366	Guanine nucleotide-binding protein-like 3-like protein	IPI00005132	0.000	0.31207	2	0	0	0
1367	Isoform IIA of Myc box-dependent-interacting protein 1	IPI00186966	0.000	0.31207	0	0	0	2
1368	Thymidine kinase, cytosolic	IPI00299214	0.000	0.31207	0	2	0	0
1369	Transcription initiation factor TFIID subunit 2	IPI00328144	0.000	0.31207	0	2	0	0
1370	Putative uncharacterized protein THADA	IPI00412647	0.000	0.31207	0	0	0	0
1371	cDNA FLJ56176, highly similar to Poly(A) polymerase alpha	IPI00384028	0.000	0.31207	0	0	2	2
1372	rho GTPase-activating protein 4 isoform 1	IPI00328842	0.000	0.31207	2	0	2	2
1373	cDNA FLJ56152, highly similar to Rho guanine nucleotide exchange factor 7	IPI00449906	0.000	0.31207	0	0	0	2
1374	Ribosomal RNA processing protein 1 homolog A	IPI00550766	0.000	0.31207	2	2	0	2
1375	Isoform 1 of Putative ATP-dependent RNA helicase DHX57	IPI00168885	0.000	0.31207	0	0	0	0
1376	kinesin-like 8 isoform c	IPI00061476	0.000	0.31207	0	2	2	2
1377	Trans-2-enoyl-CoA reductase, mitochondrial	IPI00306159	0.000	0.31207	2	2	2	2
1378	Rho GDP-dissociation inhibitor 2	IPI00003817	0.000	0.31207	0	2	0	2
1379	Atlastin-1	IPI00103530	0.000	0.31207	2	2	2	2
1380	RAB4A, member RAS oncogene family variant	IPI00480056	0.000	0.31207	3	2	3	2
1381	Isoform 1 of Pre-mRNA-processing factor 40 homolog A	IPI00337385	0.000	0.31207	0	0	0	0
1382	AP-3 complex subunit mu-1	IPI00032459	0.000	0.31207	3	3	3	3
1383	Transcription factor BTF3 homolog 4	IPI00412792	0.000	0.31207	2	3	2	3
1384	cDNA FLJ61386, highly similar to Homo sapiens mitochondrial ribosomal protein L43 (MRPL43)	IPI00334579	0.000	0.31207	2	2	0	2
1385	Molybdopterin synthase catalytic subunit	IPI00005218	0.000	0.31207	2	2	2	2
1386	Isoform 2 of General transcription factor 3C polypeptide 5	IPI00411531	0.000	0.31207	0	0	0	2
1387	Isoform 2 of Protein SET	IPI00301311	0.000	0.31207	3	0	3	0
1388	Isoform 1 of Hepatocyte growth factor receptor	IPI00029273	0.000	0.31207	0	0	2	2
1389	Isoform 1 of Inositol monophosphatase 2	IPI00023635	0.000	0.31207	0	0	0	0
1390	Lysosomal alpha-glucosidase	IPI00293088	0.000	0.31207	0	0	0	2
1391	Histone chaperone ASF1A	IPI00292168	0.000	0.31207	2	0	0	0
1392	DNA-directed RNA polymerase II subunit RPB7	IPI00218895	0.000	0.31207	0	2	0	0
1393	Isoform 2 of Endoplasmic reticulum aminopeptidase 1	IPI00165949	0.000	0.31207	0	0	0	2
1394	DEAH (Asp-Glu-Ala-His) box polypeptide 16	IPI00292510	0.000	0.31207	2	0	2	2
1395	Uncharacterized protein C20orf72	IPI00001287	0.000	0.31207	0	2	2	0
1396	WD repeat-containing protein 5	IPI00005492	0.000	0.31207	2	2	0	2
1397	Isoform 2 of tRNA (adenine-N(1)-)-methyltransferase catalytic subunit TRMT61A	IPI00177856	0.000	0.31207	0	2	0	0
1398	AP-3 complex subunit sigma-1	IPI00014624	0.000	0.31207	0	2	2	2
1399	Importin-8	IPI00007401	0.000	0.31207	0	0	0	2
1400	Isoform 1 of Secretory carrier-associated membrane protein 1	IPI00005129	0.000	0.31207	0	2	0	0
1401	NIF3L1 isoform gamma	IPI00451429	0.000	0.31207	2	2	2	0
1402	Isoform 2 of Arf-GAP domain and FG repeats-containing protein 1	IPI00304693	0.000	0.31207	0	2	0	0
1403	Exosome complex exonuclease RRP40	IPI00015956	0.000	0.31207	0	2	0	0
1404	cDNA FLJ60607, highly similar to Acyl-protein thioesterase 1	IPI00007321	0.000	0.31207	2	2	2	0
1405	Brain-specific angiogenesis inhibitor 1-associated protein 2-like protein 1	IPI00179326	0.000	0.31207	2	2	0	2
1406	Centromere protein H	IPI00009668	0.000	0.31207	2	0	0	0
1407	Peroxisomal membrane protein 2	IPI00221002	0.000	0.31207	2	2	0	2
1408	Isoform 2 of Multidrug resistance-associated protein 1	IPI00008338	0.000	0.31207	0	0	2	0
1409	Angio-associated migratory cell protein	IPI00014481	0.000	0.31207	2	3	2	3
1410	Bis(5'-nucleosyl)-tetraphosphatase [asymmetrical]	IPI00221231	0.000	0.31207	3	3	3	3
1411	Splicing factor, arginine/serine-rich 11	IPI00464952	0.000	0.31207	2	2	2	0
1412	similar to unr-interacting protein	IPI00260209	0.000	0.31207	0	3	0	3
1413	Isoform 1 of H/ACA ribonucleoprotein complex subunit 1	IPI00302176	0.000	0.31207	2	2	2	2

No.	Description	Accession number	STN[1]	p-Value[1]	Con_A[2]	Con_B[2]	SORA_A[2]	SORA_B[2]
1414	Isoform 1 of Syntaxin-7	IPI00289876	0.000	0.31207	2	4	2	4
1415	Isoform Long of Ras-related protein Rab-27A	IPI00016381	0.000	0.31207	0	2	2	2
1416	DNA repair protein RAD51 homolog 3	IPI00012829	0.000	0.31207	0	2	0	0
1417	Inositol polyphosphate 1-phosphatase	IPI00027139	0.000	0.31207	0	0	0	2
1418	1-phosphatidylinositol-4,5-bisphosphate phosphodiesterase delta-3	IPI00152701	0.000	0.31207	0	0	2	2
1419	serine/threonine-protein kinase MST4 isoform 3	IPI00182383	0.000	0.31207	0	0	0	0
1420	Isoform 2 of Uncharacterized protein C7orf26	IPI00031632	0.000	0.31207	0	0	0	2
1421	Histone chaperone ASF1B	IPI00041127	0.000	0.31207	2	0	0	0
1422	Translocated promoter region	IPI00514531	0.000	0.31207	2	0	0	2
1423	SNF2 histone linker PHD RING helicase, isoform CRA_a	IPI00470627	0.000	0.31207	2	0	0	0
1424	Isoform 1 of RNA-binding protein 34	IPI00181617	0.000	0.31207	2	0	0	2
1425	Bifunctional 3'-phosphoadenosine 5'-phosphosulfate synthase 1	IPI00011619	0.000	0.31207	0	0	0	0
1426	Cleavage and polyadenylation specificity factor subunit 3	IPI00007818	0.000	0.31207	0	2	0	0
1427	Replication initiator 1	IPI00549171	0.000	0.31207	2	2	0	0
1428	Isoform 2 of AMSH-like protease	IPI00002208	0.000	0.31207	0	0	0	0
1429	Probable RNA-binding protein 19	IPI00000686	0.000	0.31207	0	0	0	0
1430	Transmembrane protein C9orf46	IPI00307547	0.000	0.31207	0	0	0	0
1431	Transcription initiation factor TFIID subunit 9	IPI00002993	0.000	0.31207	2	2	0	0
1432	cDNA FLJ31776 fis, clone NT2RI2008141, highly similar to CALUMENIN	IPI00789155	0.000	0.31207	0	2	0	0
1433	Isoform 1 of Pyridoxal-dependent decarboxylase domain-containing protein 1	IPI00384689	0.000	0.31207	0	2	0	0
1434	KIF1-binding protein	IPI00477355	0.000	0.31207	2	2	0	2
1435	Isoform Alpha of E3 ubiquitin-protein ligase TRIM33	IPI00010252	0.000	0.31207	3	2	2	3
1436	cDNA FLJ54710, highly similar to Target of Myb protein 1	IPI00023191	0.000	0.31207	2	3	2	3
1437	Cyclin-H	IPI00021305	0.000	0.31207	0	2	2	0
1438	cDNA FLJ56370, highly similar to Homo sapiens FK506 binding protein 8, 38kDa (FKBP8), mRNA	IPI00328161	0.000	0.31207	0	2	2	0
1439	GTP cyclohydrolase 1 feedback regulatory protein	IPI00217253	0.000	0.31207	0	0	0	0
1440	Pre-mRNA-splicing factor SYF1	IPI00163084	0.000	0.31207	2	0	0	0
1441	Isoform 2 of Atlastin-2	IPI00007183	0.000	0.31207	0	0	0	2
1442	Protein FAM50A	IPI00030098	0.000	0.31207	2	0	0	2
1443	Small nuclear ribonucleoprotein G	IPI00016572	0.000	0.31207	0	3	3	0
1444	Isoform 1 of Autophagy-related protein 9A	IPI00383396	0.000	0.31207	2	0	2	0
1445	Replication factor C subunit 3	IPI00031521	0.000	0.31207	2	2	0	0
1446	Phosphoglycolate phosphatase	IPI00177008	0.000	0.31207	2	2	0	0
1447	Origin recognition complex subunit 5	IPI00015143	0.000	0.31207	0	2	0	2
1448	GTP-binding protein Rheb	IPI00016669	0.000	0.31207	0	2	0	0
1449	Isoform 1 of Nesprin-3	IPI00394994	0.000	0.31207	0	0	2	2
1450	DNA polymerase subunit gamma-2, mitochondrial	IPI00033486	0.000	0.31207	0	0	2	0
1451	Isoform 2 of 39S ribosomal protein L55, mitochondrial	IPI00419626	0.000	0.31207	0	2	0	2
1452	Isoform 1 of ARF GTPase-activating protein GIT1	IPI00384861	0.000	0.31207	2	0	0	0
1453	sorting nexin-6 isoform a	IPI00258833	0.000	0.31207	0	0	0	2
1454	Isoform 1 of Solute carrier family 12 member 4	IPI00021057	0.000	0.31207	2	2	0	0
1455	Isoform 2 of Calumenin	IPI00045396	0.000	0.31207	0	0	0	2
1456	Isoform 1 of Regulator of nonsense transcripts 2	IPI00300504	0.000	0.31207	0	0	0	2
1457	Isoform 1 of Heterogeneous nuclear ribonucleoprotein L-like	IPI00103247	0.000	0.31207	0	0	0	2
1458	PDXDC1 protein	IPI00329208	0.000	0.31207	0	0	0	0
1459	Translation initiation factor eIF-2B subunit epsilon	IPI00011898	0.000	0.31207	0	0	0	2
1460	Isoform 1 of UPF0557 protein C10orf119	IPI00478758	0.000	0.31207	0	0	0	2
1461	Origin recognition complex subunit 2	IPI00013216	0.000	0.31207	0	0	2	2
1462	Proteasomal ubiquitin receptor ADRM1	IPI00033030	0.000	0.31207	2	0	0	0
1463	Sorcin	IPI00027175	0.000	0.31207	0	2	0	0
1464	Charged multivesicular body protein 4b	IPI00025974	0.000	0.31207	0	0	0	2
1465	Transducin beta-like protein 2	IPI00000948	0.000	0.31207	2	2	0	0
1466	Werner syndrome ATP-dependent helicase	IPI00029107	0.000	0.31207	2	0	0	2
1467	Isoform 1 of Mortality factor 4-like protein 1	IPI00409675	0.000	0.31207	0	2	0	2
1468	A-kinase anchor protein 8	IPI00014474	0.000	0.31207	3	0	2	3
1469	Isoform 3 of Myosin phosphatase Rho-interacting protein	IPI00166518	0.000	0.31207	0	0	0	2
1470	Glia maturation factor, beta	IPI00412987	0.000	0.31207	2	2	0	2
1471	COBW domain-containing protein 2	IPI00216734	0.000	0.31207	3	2	3	0
1472	Glutathione S-transferase theta-1	IPI00741097	0.000	0.31207	3	2	3	2
1473	Acetyl-coenzyme A synthetase, cytoplasmic	IPI00413730	0.000	0.31207	3	2	0	3
1474	Isoform 1 of TRM1-like protein	IPI00334914	0.000	0.31207	0	0	2	2
1475	Exportin-6	IPI00465296	0.000	0.31207	0	2	0	0
1476	Putative uncharacterized protein	IPI00027984	0.000	0.31207	0	0	0	0
1477	NADH dehydrogenase [ubiquinone] 1 alpha subcomplex assembly factor 3	IPI00399053	0.000	0.31207	0	2	0	0
1478	Synaptogyrin-2	IPI00013946	0.000	0.31207	0	0	0	0
1479	Phosphatidylserine synthase 1	IPI00010746	0.000	0.31207	0	2	0	0
1480	Isoform 1 of Abl interactor 1	IPI00431025	0.000	0.31207	0	0	0	2
1481	Agmatinase, mitochondrial	IPI00305360	0.000	0.31207	0	0	2	0
1482	Importin 5	IPI00639960	0.000	0.31207	0	2	2	0
1483	Protein FAM118B	IPI00002240	0.000	0.31207	0	0	2	0
1484	Isoform 1 of Mitogen-activated protein kinase kinase kinase kinase 4	IPI00006752	0.000	0.31207	2	0	0	0
1485	Eukaryotic translation initiation factor 4E-binding protein 1	IPI00002569	0.000	0.31207	0	0	0	0
1486	Pleckstrin-2	IPI00009302	0.000	0.31207	0	2	0	2
1487	Isoform 1 of Inositol-tetrakisphosphate 1-kinase	IPI00100329	0.000	0.31207	2	2	0	0
1488	telomerase-binding protein EST1A isoform 2	IPI00014252	0.000	0.31207	2	2	2	2
1489	Isoform 1 of Long-chain-fatty-acid--CoA ligase 1	IPI00012728	0.000	0.31207	2	0	0	0
1490	Caspase-3	IPI00292140	0.000	0.31207	0	2	0	0
1491	Target of EGR1 protein 1	IPI00549516	0.000	0.31207	0	0	2	0
1492	Isoform 1 of Rho guanine nucleotide exchange factor 12	IPI00022164	0.000	0.31207	2	0	0	0
1493	CDKN2A-interacting protein	IPI00020991	0.000	0.31207	2	0	0	0
1494	importin subunit alpha-6	IPI00413214	0.000	0.31207	2	2	2	0
1495	Isoform 1 of Putative RNA-binding protein 15	IPI00102752	0.000	0.31207	2	0	0	0
1496	Isoform 1 of Dynamin-1	IPI00413140	0.000	0.31207	2	0	0	2
1497	Isoform 2 of Dynactin subunit 3	IPI00013654	0.000	0.31207	2	0	0	0
1498	Origin recognition complex subunit 6	IPI00001641	0.000	0.31207	2	2	2	0
1499	Serine palmitoyltransferase 1	IPI00005745	0.000	0.31207	2	2	0	2
1500	Origin recognition complex subunit 4	IPI00015164	0.000	0.31207	0	2	2	0
1501	Isoform 1 of Protein zwilch homolog	IPI00329679	0.000	0.31207	0	0	2	0
1502	Isoform 2 of LisH domain and HEAT repeat-containing protein KIAA1468	IPI00023330	0.000	0.31207	0	0	0	0
1503	Isoform 2 of Protein PAT1 homolog 1	IPI00760958	0.000	0.31207	2	0	0	0
1504	Vacuolar protein sorting-associated protein 28 homolog	IPI00007155	0.000	0.31207	2	0	0	2
1505	Isoform 1 of N-alpha-acetyltransferase 40, NatD catalytic subunit	IPI00328847	0.000	0.31207	2	2	2	2
1506	Checkpoint protein HUS1	IPI00004712	0.000	0.31207	2	2	0	2
1507	peroxisomal 3,2-trans-enoyl-CoA isomerase isoform 1	IPI00419263	0.000	0.31207	0	2	2	0
1508	Isoform 1 of Transmembrane protein 192	IPI00855873	0.000	0.31207	0	2	0	2

No.	Description	Accession number	STN[1]	p-Value[1]	Con_A[2]	Con_B[2]	SORA_A[2]	SORA_B[2]
1509	Trafficking protein particle complex subunit 4	IPI00007691	0.000	0.31207	2	0	0	0
1510	Isoform I of Septin-6	IPI00216139	0.000	0.31207	0	2	0	0
1511	Exosome complex exonuclease RRP41	IPI00745613	0.000	0.31207	2	0	0	0
1512	Isoform 2 of Mediator of RNA polymerase II transcription subunit 8	IPI00300278	0.000	0.31207	0	2	0	0
1513	Gamma-aminobutyric acid receptor-associated protein	IPI00027253	0.000	0.31207	2	0	0	0
1514	Coiled-coil-helix-coiled-coil-helix domain-containing protein 2, mitochondrial	IPI00007673	0.000	0.31207	0	2	0	0
1515	Isoform 1 of Mannose-1-phosphate guanyltransferase alpha	IPI00101782	0.000	0.31207	2	2	0	0
1516	Bleomycin hydrolase	IPI00219575	0.000	0.31207	0	0	0	2
1517	Renin receptor	IPI00168884	0.000	0.31207	2	0	0	0
1518	HLA class I histocompatibility antigen, B-7 alpha chain	IPI00004657	0.000	0.31207	0	2	2	0
1519	Acidic leucine-rich nuclear phosphoprotein 32 family member A	IPI00025849	0.000	0.31207	2	0	0	0
1520	Isoform 2 of Polyhomeotic-like protein 2	IPI00419684	0.000	0.31207	0	2	0	0
1521	Isoform 1 of Poly [ADP-ribose] polymerase 2	IPI00026497	0.000	0.31207	0	0	0	0
1522	Coiled-coil domain-containing protein 86	IPI00012199	0.000	0.31207	0	0	2	0
1523	Polyribonucleotide 5'-hydroxyl-kinase Clp1	IPI00024381	0.000	0.31207	0	0	2	0
1524	Putative uncharacterized protein	IPI00010402	0.000	0.31207	0	0	0	0
1525	Calcium signal-modulating cyclophilin ligand	IPI00025729	0.000	0.31207	0	2	0	0
1526	Isoform 1 of Melanoma inhibitory activity protein 3	IPI00455473	0.000	0.31207	2	0	0	0
1527	Myosin-If	IPI00218638	0.000	0.31207	0	0	0	2
1528	cDNA FLJ55772, highly similar to Rab5 GDP/GTP exchange factor	IPI00004974	0.000	0.31207	0	0	0	2
1529	Isoform 2 of Probable palmitoyltransferase ZDHHC5	IPI00216546	0.000	0.31207	0	0	0	2
1530	Cell division protein kinase 4	IPI00007811	0.000	0.31207	2	0	0	0
1531	Isoform 1 of DNA damage-binding protein 2	IPI00021518	0.000	0.31207	2	0	0	0
1532	Isoform 2 of Actin-related protein 2/3 complex subunit 5	IPI00007280	0.000	0.31207	2	2	0	0
1533	Secretory carrier-associated membrane protein 2	IPI00218850	0.000	0.31207	2	2	0	0
1534	Pre-mRNA-splicing factor SYF2	IPI00022963	0.000	0.31207	0	2	0	0
1535	Isoform 1 of Lysine-specific demethylase 3B	IPI00298935	0.000	0.31207	0	0	0	2
1536	39S ribosomal protein L14, mitochondrial	IPI00418290	0.000	0.31207	2	2	2	2
1537	Isoform 2 of Hydroxysteroid dehydrogenase-like protein 2	IPI00031107	0.000	0.31207	2	3	0	3
1538	2-hydroxyacyl-CoA lyase 1	IPI00296535	0.000	0.31207	2	2	2	0
1539	Isoform 6 of Terminal uridylyltransferase 7	IPI00336000	0.000	0.31207	2	0	0	0
1540	Isoform 1 of Integrator complex subunit 6	IPI00015922	0.000	0.31207	0	0	0	0
1541	Stromal interaction molecule 1	IPI00299063	0.000	0.31207	0	0	0	2
1542	Cyclin B1	IPI00294696	0.000	0.31207	0	2	0	0
1543	Isoform Long of Metastasis-associated protein MTA1	IPI00012773	0.000	0.31207	2	0	0	0
1544	LanC-like protein 1	IPI00005724	0.000	0.31207	0	2	0	0
1545	cDNA FLJ58308, highly similar to Alpha-1,2-mannosyltransferase ALG9	IPI00234857	0.000	0.31207	2	0	0	0
1546	KRR1 small subunit processome component homolog	IPI00156032	0.000	0.31207	0	0	0	0
1547	46 kDa protein	IPI00641706	0.000	0.31207	2	0	0	0
1548	Isoform 2 of Bifunctional arginine demethylase and lysyl-hydroxylase JMJD6	IPI00375496	0.000	0.31207	0	2	2	2
1549	[Pyruvate dehydrogenase [acetyl-transferring]]-phosphatase 1, mitochondria	IPI00218971	0.000	0.31207	0	2	2	0
1550	Neuronal protein	IPI00472058	0.000	0.31207	2	0	0	0
1551	CDGSH iron sulfur domain-containing protein 2	IPI00166865	0.000	0.31207	2	2	0	2
1552	sulfatase modifying factor 2 isoform b precursor	IPI00171412	0.000	0.31207	0	0	0	2
1553	Isoform 1 of Alpha-(1,6)-fucosyltransferase	IPI00004668	0.000	0.31207	2	0	2	0
1554	N-acetylgalactosaminyltransferase 7	IPI00328391	0.000	0.31207	2	0	2	0
1555	GSK3-beta interaction protein	IPI00009374	0.000	0.31207	2	0	0	2
1556	Isoform 1 of Serine/threonine-protein kinase N1	IPI00002803	0.000	0.31207	2	0	2	0
1557	Isoform 1 of Protein tyrosine phosphatase type IVA 2	IPI00020191	0.000	0.31207	0	2	0	0
1558	Isoform 3 of Centromere protein V	IPI00376481	0.000	0.31207	0	2	2	0
1559	Isoform 4 of Afadin	IPI00023461	0.000	0.31207	0	0	0	0
1560	Mitochondrial ornithine transporter 1	IPI00003389	0.000	0.31207	0	2	0	0
1561	Isoform 2 of Torsin-1A-interacting protein 1	IPI00012280	0.000	0.31207	2	2	0	0
1562	High mobility group nucleosome-binding domain-containing protein 5	IPI00006157	0.000	0.31207	3	0	3	2
1563	Mediator of RNA polymerase II transcription subunit 13	IPI00021388	0.000	0.31207	0	0	0	0
1564	Probable ATP-dependent RNA helicase YTHDC2	IPI00010200	0.000	0.31207	0	0	0	2
1565	Isoform 3 of Parkinson disease 7 domain-containing protein 1	IPI00167976	0.000	0.31207	0	3	0	3
1566	Isoform 1 of YTH domain family protein 2	IPI00306043	0.000	0.31207	2	0	2	0
1567	Peptidylprolyl isomerase domain and WD repeat-containing protein 1	IPI00149650	0.000	0.31207	0	0	0	2
1568	Uncharacterized protein C1orf198	IPI00013912	0.000	0.31207	0	2	0	0
1569	NudC domain-containing protein 2	IPI00103142	0.000	0.31207	0	2	2	0
1570	Phosphatidylinositol 4-kinase type 2-alpha	IPI00020124	0.000	0.31207	0	0	0	0
1571	Isoform UBF1 of Nucleolar transcription factor 1	IPI00014533	0.000	0.31207	0	0	0	2
1572	Cytosolic Fe-S cluster assembly factor NUBP2	IPI00644674	0.000	0.31207	0	0	0	0
1573	Isoform Alpha-1 of Protein phosphatase 1A	IPI00020950	0.000	0.31207	0	0	0	0
1574	Isoform 1 of Tropomyosin beta chain	IPI00013991	0.000	0.31207	0	0	0	0
1575	Cell division protein kinase 3	IPI00023503	0.000	0.31207	2	0	0	2
1576	DDB1- and CUL4-associated factor 13	IPI00306642	0.000	0.31207	2	0	0	0
1577	Isoform A of SWI/SNF-related matrix-associated actin-dependent regulator of chromatin subfamily B	IPI00029695	0.000	0.31207	0	2	0	2
1578	RNASEH2B protein	IPI00245135	0.000	0.31207	0	2	2	2
1579	Isoform 1 of Hepatocyte growth factor-regulated tyrosine kinase substrate	IPI00006176	0.000	0.31207	0	2	0	2
1580	Isoform 1 of Serine/threonine-protein phosphatase 4 regulatory subunit 3B	IPI00414323	0.000	0.31207	2	0	0	0
1581	Isoform 1 of Signal transducing adapter molecule 1	IPI00020178	0.000	0.31207	2	2	0	0
1582	Exosome complex exonuclease RRP46	IPI00015955	0.000	0.31207	0	0	2	2
1583	Major centromere autoantigen B	IPI00010388	0.000	0.31207	0	2	0	0
1584	cDNA FLJ20475 fis, clone KAT07206	IPI00183065	0.000	0.31207	3	2	3	0
1585	Chloride intracellular channel protein 3	IPI00000692	0.000	0.31207	0	2	0	2
1586	Isoform 1 of Signal transducer and activator of transcription 6	IPI00030782	0.000	0.31207	2	0	0	0
1587	Ribosome biogenesis regulatory protein homolog	IPI00014253	0.000	0.31207	0	2	2	2
1588	CDK-activating kinase assembly factor MAT1	IPI00294701	0.000	0.31207	2	0	2	0
1589	Thioredoxin domain-containing protein 9	IPI00022386	0.000	0.31207	0	0	0	0
1590	Isocitrate dehydrogenase [NAD] subunit gamma, mitochondrial	IPI00220150	0.000	0.31207	2	2	0	2
1591	Protein DERPC	IPI00171540	0.000	0.31207	0	0	2	2
1592	Calcium-binding protein p22	IPI00218924	0.000	0.31207	0	2	2	0
1593	Isoform 2 of Carbohydrate kinase domain-containing protein	IPI00645172	0.000	0.31207	2	0	0	2
1594	Deoxynucleotidyltransferase terminal-interacting protein 2	IPI00290410	0.000	0.31207	2	2	2	0
1595	MLL1/MLL complex subunit C17orf49 isoform 1	IPI00373869	0.000	0.31207	2	2	2	0
1596	Proto-oncogene tyrosine-protein kinase Yes	IPI00013981	0.000	0.31207	0	0	0	0
1597	Isoform 1 of Ran-binding protein 3	IPI00026337	0.000	0.31207	0	2	0	2
1598	Isoform 1 of Myosin-XVIIIa	IPI00760846	0.000	0.31207	2	0	0	0
1599	Adenosine monophosphate deaminase 2	IPI00007722	0.000	0.31207	0	0	0	2
1600	Isoform 1 of Pre-mRNA-splicing factor RBM22	IPI00019046	0.000	0.31207	2	0	0	0
1601	Protein kinase C and casein kinase substrate in neurons 3, isoform CRA_b	IPI00329572	0.000	0.31207	0	0	0	2
1602	Isoform 1 of Rab GTPase-binding effector protein 1	IPI00293009	0.000	0.31207	0	0	0	2

No.	Description	Accession number	STN[1]	p-Value[1]	Con_A[2]	Con_B[2]	SORA_A[2]	SORA_B[2]
1603	Isoform 1 of Polyglutamine-binding protein 1	IPI00024698	0.000	0.31207	2	0	2	0
1604	cDNA FLJ59751, weakly similar to Mus musculus spermatogenesis associated, serine-rich 2 (Spats2)	IPI00023532	0.000	0.31207	0	0	0	0
1605	cDNA FLJ45232 fis, clone BRCAN2021718, highly similar to Homo sapiens mitochondrial ribosomal	IPI00170877	0.000	0.31207	0	2	0	0
1606	Isoform 1 of Pre-mRNA-splicing factor 38B	IPI00018098	0.000	0.31207	0	2	0	0
1607	Isoform 1 of BAG family molecular chaperone regulator 5	IPI00007731	0.000	0.31207	0	0	2	2
1608	Isoform 2 of Rho GTPase-activating protein 5	IPI00013988	0.000	0.31207	2	0	2	0
1609	Isoform 1 of STIP1 homology and U box-containing protein 1	IPI00025156	0.000	0.31207	2	2	0	0
1610	DNA-directed RNA polymerase II subunit RPB4	IPI00007283	0.000	0.31207	2	0	0	0
1611	F-box only protein 7	IPI00294567	0.000	0.31207	2	2	0	0
1612	p53 and DNA damage-regulated protein 1	IPI00027887	0.000	0.31207	2	0	0	0
1613	Isoform 2 of E1A-binding protein p400	IPI00064931	0.000	0.31207	0	2	0	0
1614	Polymerase delta interacting protein 46	IPI00429180	0.000	0.31207	2	0	0	2
1615	Isoform 1 of RNA polymerase II subunit A C-terminal domain phosphatase SSU72	IPI00023556	0.000	0.31207	0	2	0	2
1616	Isoform 1 of Surfeit locus protein 1	IPI00018034	0.000	0.31207	0	2	2	2
1617	Isoform 3 of F-box only protein 22	IPI00169168	0.000	0.31207	2	2	0	2
1618	Alpha-mannosidase 2	IPI00003802	0.000	0.31207	0	0	2	0
1619	serine/threonine-protein phosphatase 2B catalytic subunit beta isoform isoform a	IPI00027809	0.000	0.31207	0	2	0	0
1620	c-Maf-inducing protein isoform C-mip	IPI00028438	0.000	0.31207	0	0	0	0
1621	Cytoplasmic tRNA 2-thiolation protein 1	IPI00062882	0.000	0.31207	0	0	0	0
1622	Isoform 1 of AP-1 complex subunit mu-2	IPI00002552	0.000	0.31207	2	0	2	0
1623	Isoform 1 of Solute carrier family 12 member 2	IPI00022649	0.000	0.31207	0	0	0	0
1624	Isoform 1 of Prostaglandin reductase 2	IPI00167515	0.000	0.31207	2	2	0	0
1625	cDNA FLJ54836	IPI00063160	0.000	0.31207	2	0	0	2
1626	Isoform 1 of Translation initiation factor eIF-2B subunit gamma	IPI00006504	0.000	0.31207	0	0	2	0
1627	Uncharacterized protein C3orf26	IPI00031679	0.000	0.31207	0	0	0	0
1628	Isoform 1 of Protein disulfide-isomerase TMX3	IPI00064193	0.000	0.31207	0	0	0	0
1629	WD repeat-containing protein 43	IPI00937477	0.000	0.31207	2	0	2	2
1630	17 kDa protein	IPI00643390	0.000	0.31207	2	2	0	2
1631	DNA mismatch repair protein Mlh1	IPI00029754	0.000	0.31207	0	0	0	0
1632	Isoform 3 of Nuclear transcription factor Y subunit gamma	IPI00071697	0.000	0.31207	0	0	2	0
1633	Isoform 1 of Microtubule-associated protein 4	IPI00396171	0.000	0.31207	0	0	0	2
1634	Protein FAM114A2	IPI00329662	0.000	0.31207	0	2	0	0
1635	Putative uncharacterized protein ZYX	IPI00924931	0.000	0.31207	2	2	2	2
1636	Glycolipid transfer protein domain-containing protein 1	IPI00455408	0.000	0.31207	0	2	2	2
1637	Lipoma-preferred partner	IPI00023704	0.000	0.31207	0	2	0	2
1638	NHP2-like protein 1	IPI00026167	0.000	0.31207	0	2	2	2
1639	Isoform 1 of Spindle and kinetochore-associated protein 1	IPI00059912	0.000	0.31207	0	2	0	0
1640	Periodic tryptophan protein 1 homolog	IPI00014298	0.000	0.31207	0	2	0	0
1641	507 kDa protein	IPI00031411	0.000	0.31207	0	0	0	2
1642	Putative uncharacterized protein TXNRD2	IPI00157820	0.000	0.31207	2	2	0	2
1643	SNARE-associated protein Snapin	IPI00018331	0.000	0.31207	0	0	2	0
1644	WD repeat-containing protein 46	IPI00023126	0.000	0.31207	0	0	0	2
1645	Formin-binding protein 4	IPI00170778	0.000	0.31207	0	2	2	0
1646	cDNA FLJ10079 fis, clone HEMBA1001896, weakly similar to DIMETHYLGLYCINE DEHYDROGENASE	IPI00017494	0.000	0.31207	0	0	0	0
1647	Protein LTV1 homolog	IPI00153032	0.000	0.31207	2	2	0	2
1648	Isoform 1 of Lariat debranching enzyme	IPI00305545	0.000	0.31207	0	2	2	2
1649	cDNA FLJ55829, highly similar to Homo sapiens leucine zipper and CTNNBIP1 domain containing	IPI00152900	0.000	0.31207	2	0	0	0
1650	Nuclear pore complex protein Nup88	IPI00001738	0.000	0.31207	0	0	0	2
1651	Activator of basal transcription 1	IPI00002938	0.000	0.31207	0	0	0	0
1652	Isoform 1 of Striatin	IPI00014456	0.000	0.31207	0	0	0	2
1653	Putative transferase C1orf69, mitochondrial	IPI00145260	0.000	0.31207	0	2	0	0
1654	Tetratricopeptide repeat protein 19	IPI00170855	0.000	0.31207	0	0	2	0
1655	FH1/FH2 domain-containing protein 1	IPI00001730	0.000	0.31207	0	0	2	2
1656	Isoform 1 of Protein sel-1 homolog 1	IPI00002790	0.000	0.31207	0	0	0	0
1657	Isoform 1 of Elongator complex protein 3	IPI00165477	0.000	0.31207	2	2	2	0
1658	Chromosome-associated kinesin KIF4B	IPI00175193	0.000	0.31207	2	0	0	0
1659	Helicase ARIP4	IPI00294787	0.000	0.31207	2	0	0	0
1660	E-cadherin	IPI00000513	0.000	0.31207	2	2	0	0
1661	Kelch-like ECH-associated protein 1	IPI00106502	0.000	0.31207	2	2	0	0
1662	UPF0609 protein C4orf27	IPI00016532	0.000	0.31207	0	0	0	2
1663	ERBB2IP protein	IPI00438286	0.000	0.31207	0	0	0	2
1664	UPF0534 protein C4orf43	IPI00019962	0.000	0.31207	2	0	2	0
1665	Heterochromatin protein 1, binding protein 3	IPI00640417	0.000	0.31207	2	2	0	0
1666	Transmembrane and coiled-coil domain-containing protein 7	IPI00034201	0.000	0.31207	2	0	0	0
1667	Flotillin-2	IPI00789008	0.000	0.31207	0	0	0	2
1668	RNA-binding protein 7	IPI00001134	0.000	0.31207	0	0	0	0
1669	Isoform 2 of Pre-mRNA-splicing factor ISY1 homolog	IPI00063673	0.000	0.31207	0	2	0	0
1670	61 kDa protein	IPI00012486	0.000	0.31207	0	0	0	2
1671	Protein FAM96A	IPI00030985	0.000	0.31207	0	2	2	0
1672	Isoform 1 of Solute carrier family 35 member F2	IPI00293362	0.000	0.31207	0	0	0	2
1673	Coiled-coil-helix-coiled-coil-helix domain-containing protein 6	IPI00031622	0.000	0.31207	0	2	2	0
1674	Isoform 1 of Glycerophosphodiester phosphodiesterase domain-containing protein 1	IPI00298535	0.000	0.31207	2	0	0	0
1675	Protein ariadne-1 homolog	IPI00294943	0.000	0.31207	2	0	2	0
1676	Isoform 2 of 5'-AMP-activated protein kinase catalytic subunit alpha-1	IPI00410287	0.000	0.31207	2	2	0	2
1677	THO complex subunit 7 homolog	IPI00291131	0.000	0.31207	2	2	0	0
1678	Isoform 2 of Alkylated DNA repair protein alkB homolog 5	IPI00413659	0.000	0.31207	0	2	0	2
1679	Poly(ADP-ribose) glycohydrolase ARH3	IPI00015865	0.000	0.31207	0	2	0	2
1680	Isoform 1 of Uncharacterized protein CXorf38	IPI00152089	0.000	0.31207	2	0	2	2
1681	Krueppel-like factor 5	IPI00015934	0.000	0.31207	0	0	0	0
1682	Golgi resident protein GCP60	IPI00009315	0.000	0.31207	0	0	2	0
1683	Isoform 1 of DNA repair protein complementing XP-G cells	IPI00477535	0.000	0.31207	0	0	0	2
1684	Isoform 1 of Trafficking protein particle complex subunit 2	IPI00005119	0.000	0.31207	2	2	0	2
1685	Active regulator of SIRT1	IPI00219006	0.000	0.31207	0	2	2	2
1686	Synaptojanin-2-binding protein	IPI00299193	0.000	0.31207	0	2	2	0
1687	MMP37-like protein, mitochondrial	IPI00060287	0.000	0.31207	0	0	0	0
1688	Thioesterase superfamily member 4	IPI00064739	0.000	0.31207	2	0	0	2
1689	Prolactin regulatory element-binding protein	IPI00033349	0.000	0.31207	2	2	0	0
1690	119 kDa protein	IPI00297178	0.000	0.31207	2	0	0	0
1691	Inner nuclear membrane protein Man1	IPI00032491	0.000	0.31207	0	2	0	0
1692	ER lumen protein retaining receptor 1	IPI00028116	0.000	0.31207	0	0	2	2
1693	Haloacid dehalogenase-like hydrolase domain-containing protein 3	IPI00009931	0.000	0.31207	2	0	0	0
1694	Isoform 1 of RNA 3'-terminal phosphate cyclase	IPI00011726	0.000	0.31207	0	2	0	2
1695	Casein kinase I isoform alpha-like	IPI00167096	0.000	0.31207	0	2	0	0

No.	Description	Accession number	STN[1]	p-Value[1]	Con_A[2]	Con_B[2]	SORA_A[2]	SORA_B[2]
1696	Arginyl-tRNA synthetase 2, mitochondrial	IPI00514101	0.000	0.31207	2	2	0	2
1697	DNA polymerase delta subunit 3	IPI00394926	0.000	0.31207	0	0	0	2
1698	Vacuolar protein sorting-associated protein 45	IPI00090327	0.000	0.31207	0	0	0	0
1699	Isoform 2 of Valacyclovir hydrolase	IPI00003990	0.000	0.31207	0	0	0	2
1700	Isoform 1 of Mucosa-associated lymphoid tissue lymphoma translocation protein 1	IPI00009540	0.000	0.31207	2	0	2	0
1701	Prefoldin subunit 6	IPI00005657	0.000	0.31207	0	2	0	0
1702	NADH dehydrogenase [ubiquinone] 1 alpha subcomplex assembly factor 4	IPI00023064	0.000	0.31207	2	2	0	2
1703	Coiled-coil domain-containing protein 134	IPI00302674	0.000	0.31207	0	0	0	2
1704	Phospholysine phosphohistidine inorganic pyrophosphate phosphatase	IPI00005474	0.000	0.31207	0	0	0	2
1705	Mitochondrial folate transporter/carrier	IPI00300886	0.000	0.31207	2	0	2	0
1706	RWD domain-containing protein 1	IPI00034010	0.000	0.31207	0	0	0	2
1707	Nuclear envelope pore membrane protein POM 121C	IPI00032358	0.000	0.31207	2	0	0	0
1708	39S ribosomal protein L53, mitochondrial	IPI00061531	0.000	0.31207	2	0	0	0
1709	Ubiquitin-conjugating enzyme E2 J1	IPI00006937	0.000	0.31207	2	0	0	0
1710	Galectin-related protein	IPI00023549	0.000	0.31207	0	2	0	0
1711	Ubiquitin domain-containing protein UBFD1	IPI00005194	0.000	0.31207	2	0	2	2
1712	Nucleolar GTP-binding protein 2	IPI00015808	0.000	0.31207	2	0	0	0
1713	AP-3 complex subunit beta-2	IPI00005793	0.000	0.31207	2	0	0	0
1714	Methionine aminopeptidase 2	IPI00033036	0.000	0.31207	2	0	0	2
1715	Poly(A)-specific ribonuclease PARN	IPI00294744	0.000	0.31207	0	2	2	2
1716	Argininosuccinate synthase	IPI00020632	0.000	0.31207	2	0	2	2
1717	Isoform 2 of Peroxisomal acyl-coenzyme A oxidase 1	IPI00477729	0.000	0.31207	2	0	0	0
1718	Ribosomal RNA-processing protein 8	IPI00304932	0.000	0.31207	2	0	0	2
1719	cDNA FLJ54848, highly similar to tRNA-splicing endonuclease subunit Sen34	IPI00451941	0.000	0.31207	0	0	0	0
1720	Isoform 2 of Treacle protein	IPI00298696	0.000	0.31207	0	2	0	0
1721	Isoform Long of Autophagy protein 5	IPI00006800	0.000	0.31207	2	0	0	0
1722	Guanine nucleotide-binding protein subunit alpha-14	IPI00000695	0.000	0.31207	0	0	0	0
1723	Gigaxonin	IPI00022758	0.000	0.31207	0	0	0	0
1724	Uncharacterized protein C7orf30	IPI00061492	0.000	0.31207	0	0	0	0
1725	Isoform 1 of Disks large homolog 3	IPI00023343	0.000	0.31207	2	0	0	0
1726	Lysosome membrane protein 2	IPI00217766	0.000	0.31207	0	0	0	0
1727	Isoform 1 of Centrosomal protein of 55 kDa	IPI00101532	0.000	0.31207	0	2	0	0
1728	Endoribonuclease Dicer	IPI00219036	0.000	0.31207	0	0	0	0
1729	Isoform 1 of Leucine-rich repeat-containing protein 1	IPI00427739	0.000	0.31207	0	0	0	0
1730	Putative uncharacterized protein KIF20B	IPI00044751	0.000	0.31207	2	2	0	0
1731	Isoform Mitochondrial of Malonyl-CoA decarboxylase, mitochondrial	IPI00000663	0.000	0.31207	0	0	2	2
1732	Isoform 1 of ELAV-like protein 2	IPI00030250	0.000	0.31207	0	0	0	2
1733	Isoform 1 of Coiled-coil domain-containing protein 109A	IPI00171573	0.000	0.31207	2	0	0	0
1734	Alpha-galactosidase A	IPI00025869	0.000	0.31207	2	0	0	0
1735	Isoform 1 of E3 ubiquitin-protein ligase Itchy homolog	IPI00061780	0.000	0.31207	2	0	0	0
1736	Vacuolar protein sorting-associated protein 37B	IPI00002926	0.000	0.31207	0	2	0	0
1737	Reticulocalbin-2	IPI00029628	0.000	0.31207	0	2	2	2
1738	Protein FAM96B	IPI00007024	0.000	0.31207	0	0	0	0
1739	Isoform Long of Ancient ubiquitous protein 1	IPI00001891	0.000	0.31207	0	2	0	0
1740	Isoform 2 of Nucleolar protein 10	IPI00016494	0.000	0.31207	0	2	0	0
1741	Isoform 2 of Kinesin-like protein KIF2C	IPI00216113	0.000	0.31207	0	0	0	0
1742	Importin subunit alpha-3	IPI00299033	0.000	0.31207	2	2	0	2
1743	CTP synthase II	IPI00553127	0.000	0.31207	0	0	0	2
1744	Isoform 2 of Septin-8	IPI00022082	0.000	0.31207	2	0	0	0
1745	Isoform 1 of Copine-7	IPI00002657	0.000	0.31207	0	2	0	0
1746	Isoform 1 of Integrin alpha-V	IPI00027505	0.000	0.31207	0	0	2	2
1747	Isoform 2 of DnaJ homolog subfamily A member 4	IPI00465105	0.000	0.31207	0	0	0	0
1748	Isoform 1 of Kinesin-like protein KIF23	IPI00291579	0.000	0.31207	2	0	0	0
1749	Cation transport regulator-like protein 2	IPI00103047	0.000	0.31207	2	2	0	0
1750	Isoform 1 of Regulation of nuclear pre-mRNA domain-containing protein 2	IPI00384541	0.000	0.31207	2	2	0	0
1751	15 kDa protein	IPI00000186	0.000	0.31207	0	2	0	2
1752	Isoform 1 of Transmembrane protein 55B	IPI00030530	0.000	0.31207	0	0	0	2
1753	Isoform 1 of Nucleotide-binding protein-like	IPI00384517	0.000	0.31207	0	0	0	0
1754	Proteasome assembly chaperone 3	IPI00031106	0.000	0.31207	0	0	2	2
1755	C-Myc-binding protein	IPI00871174	0.000	0.31207	2	0	0	0
1756	Serpin B9	IPI00032139	0.000	0.31207	2	0	0	0
1757	Isoform 1 of Signal transducing adapter molecule 2	IPI00290542	0.000	0.31207	0	2	0	0
1758	nucleoside diphosphate kinase type 6	IPI00218214	0.000	0.31207	0	0	0	2
1759	Protein tyrosine phosphatase type IVA 1	IPI00020164	0.000	0.31207	0	2	0	2
1760	Isoform 1 of Cell division protein kinase 9	IPI00301923	0.000	0.31207	2	0	0	0
1761	Syntaxin-18	IPI00027194	0.000	0.31207	2	0	2	0
1762	Galactosylgalactosylxylosylprotein 3-beta-glucuronosyltransferase 3	IPI00304331	0.000	0.31207	0	2	0	2
1763	Isoform 1 of Eukaryotic initiation factor 4A-II	IPI00328328	0.000	0.31207	2	2	0	0
1764	Isoform 1 of Ubiquitin-like modifier-activating enzyme 5	IPI00015736	0.000	0.31207	2	2	0	2
1765	Acyl-coenzyme A thioesterase 8	IPI00298202	0.000	0.31207	2	0	2	0
1766	NEDD4-like E3 ubiquitin-protein ligase WWP2	IPI00013010	0.000	0.31207	0	2	2	0
1767	Isoform A of Probable cation-transporting ATPase 13A1	IPI00034277	0.000	0.31207	0	0	0	2
1768	Isoform 1 of Ubiquitin thioesterase OTUB2	IPI00302895	0.000	0.31207	0	0	2	0
1769	Death domain-containing protein CRADD	IPI00020364	0.000	0.31207	0	2	0	0
1770	cDNA FLJ30398 fis, clone BRACE2008402, highly similar to Steroid receptor RNA activator 1	IPI00102313	0.000	0.31207	0	0	0	0
1771	Isoform 1 of Protein IWS1 homolog	IPI00296432	0.000	0.31207	2	0	0	0
1772	Mitochondrial intermediate peptidase	IPI00241860	0.000	0.31207	0	0	0	0
1773	TBC1 domain family member 5	IPI00022450	0.000	0.31207	2	0	0	0
1774	Isoform 2 of Ankyrin repeat domain-containing protein 17	IPI00783186	0.000	0.31207	2	0	0	0
1775	Translation initiation factor eIF-2B subunit beta	IPI00028083	0.000	0.31207	0	0	0	2
1776	Torsin-1A-interacting protein 2	IPI00168878	0.000	0.31207	0	0	0	0
1777	Isoform 1 of ADP-ribose pyrophosphatase, mitochondrial	IPI00031558	0.000	0.31207	2	0	0	2
1778	Isoform 2 of Helicase SRCAP	IPI00009101	0.000	0.31207	0	0	0	0
1779	Borealin	IPI00303099	0.000	0.31207	2	0	0	0
1780	Isoform 1 of PDZ and LIM domain protein 4	IPI00032206	0.000	0.31207	0	0	0	0
1781	Protein Hook homolog 1	IPI00026305	0.000	0.31207	0	0	2	0
1782	Isoform 1 of WD repeat-containing protein 26	IPI00414197	0.000	0.31207	2	0	0	0
1783	Isoform 1 of CAP-Gly domain-containing linker protein 2	IPI00019642	0.000	0.31207	2	0	0	0
1784	DNA replication complex GINS protein PSF1	IPI00032387	0.000	0.31207	2	0	0	0
1785	Isoform 1 of Spastin	IPI00002707	0.000	0.31207	0	0	0	0
1786	Uridine diphosphate glucose pyrophosphatase	IPI00412878	0.000	0.31207	2	0	0	2
1787	Isoform 1 of Ribonucleoside-diphosphate reductase subunit M2 B	IPI00100213	0.000	0.31207	2	0	0	0
1788	Isoform 2 of Antigen peptide transporter 2	IPI00001382	0.000	0.31207	2	0	0	0
1789	Isoform 1 of Guanine nucleotide-binding protein subunit beta-like protein 1	IPI00107339	0.000	0.31207	0	2	0	0
1790	E3 ubiquitin-protein ligase MARCH5	IPI00414168	0.000	0.31207	0	2	0	0

No.	Description	Accession number	STN[1]	p-Value[1]	Con_A[2]	Con_B[2]	SORA_A[2]	SORA_B[2]
1791	Isoform 1 of tRNA (guanine-N(7)-)-methyltransferase subunit WDR4	IPI00025718	0.000	0.31207	0	2	2	0
1792	Isoform 1 of Tumor protein D52	IPI00619958	0.000	0.31207	2	0	0	2
1793	La ribonucleoprotein domain family, member 5	IPI00514141	0.000	0.31207	2	0	0	0
1794	Golgin subfamily A member 7	IPI00480022	0.000	0.31207	0	2	0	2
1795	Pentatricopeptide repeat-containing protein 1	IPI00171925	0.000	0.31207	0	2	0	2
1796	Isoform 1 of N-acylethanolamine-hydrolyzing acid amidase	IPI00024083	0.000	0.31207	0	0	0	0
1797	Putative uncharacterized protein LCMT1	IPI00296370	0.000	0.31207	0	0	0	0
1798	Isoform SNAP-23a of Synaptosomal-associated protein 23	IPI00010438	0.000	0.31207	0	2	2	2
1799	Isoform 1 of Methyl-CpG-binding domain protein 2	IPI00434623	0.000	0.31207	0	2	0	2
1800	Isoform 1 of Bridging integrator 3	IPI00032966	0.000	0.31207	0	0	0	2
1801	Isoform 1 of Casein kinase I isoform delta	IPI00011102	0.000	0.31207	2	0	0	0
1802	DnaJ homolog subfamily C member 17	IPI00018798	0.000	0.31207	2	2	0	0
1803	Protein UXT	IPI00002646	0.000	0.31207	2	0	0	2
1804	Isoform 1 of Protein GPR89	IPI00472858	0.000	0.31207	0	0	0	2
1805	Ribosome biogenesis protein NSA2 homolog	IPI00007089	0.000	0.31207	2	0	0	0
1806	Isoform 1 of Cytosolic Fe-S cluster assembly factor NUBP1	IPI00021277	0.000	0.31207	0	2	0	0
1807	Trafficking protein particle complex subunit 5	IPI00177509	0.000	0.31207	0	2	0	2
1808	PRKC apoptosis WT1 regulator protein	IPI00001871	0.000	0.31207	0	0	2	0
1809	Armadillo repeat-containing protein 6	IPI00020196	0.000	0.31207	0	0	2	0
1810	Pumilio homolog 1 (Drosophila), isoform CRA_c	IPI00032355	0.000	0.31207	2	0	0	0
1811	Uncharacterized protein C9orf114	IPI00844014	0.000	0.31207	0	2	0	2
1812	Isoform 1 of Unconventional prefoldin RPB5 interactor	IPI00477619	0.000	0.31207	2	2	0	0
1813	Isoform 1 of Ubiquitin carboxyl-terminal hydrolase 28	IPI00045496	0.000	0.31207	0	0	0	2
1814	Isoform 1 of Ran-binding protein 9	IPI00465275	0.000	0.31207	0	0	0	0
1815	Arf-GAP with coiled-coil, ANK repeat and PH domain-containing protein 2	IPI00014264	0.000	0.31207	0	2	0	2
1816	UPF0480 protein C15orf24	IPI00024551	0.000	0.31207	2	0	0	0
1817	Isoform 1 of Peripheral plasma membrane protein CASK	IPI00514301	0.000	0.31207	2	0	0	0
1818	Thioredoxin-related transmembrane protein 4	IPI00100247	0.000	0.31207	0	2	0	0
1819	MORF4 family-associated protein 1	IPI00020915	0.000	0.31207	0	2	0	0
1820	Putative uncharacterized protein NMD3	IPI00101049	0.000	0.31207	2	0	0	0
1821	Glyoxylate reductase/hydroxypyruvate reductase, isoform CRA_c	IPI00026486	0.000	0.31207	0	0	2	0
1822	Isoform 3 of Guanine nucleotide exchange factor VAV2	IPI00004977	0.000	0.31207	0	0	0	2
1823	Isoform 2 of Serine/threonine-protein kinase PAK 1	IPI00289746	0.000	0.31207	0	2	0	2
1824	Isoform 1 of Sodium-coupled neutral amino acid transporter 5	IPI00102509	0.000	0.31207	0	0	0	2
1825	Isoform 1 of Cytoskeleton-associated protein 4	IPI00141318	0.000	0.31207	0	2	0	0
1826	MIT domain-containing protein 1	IPI00103065	0.000	0.31207	0	2	2	2
1827	ZW10 interactor	IPI00294008	0.000	0.31207	2	0	2	0
1828	Vesicle-associated membrane protein 8	IPI00030911	0.000	0.31207	0	2	0	0
1829	Integrin alpha-2	IPI00013744	0.000	0.31207	0	0	2	0
1830	Isoform Mitochondrial of Cysteine desulfurase, mitochondrial	IPI00295240	0.000	0.31207	2	0	0	0
1831	Isoform 1 of U4/U6 small nuclear ribonucleoprotein Prp31	IPI00292000	0.000	0.31207	0	0	0	2
1832	Putative uncharacterized protein WDR43	IPI00892938	0.000	0.31207	2	0	0	0
1833	Biotin--protein ligase	IPI00301907	0.000	0.31207	2	2	2	0
1834	Isoform 2 of Ribonuclease P protein subunit p40	IPI00332091	0.000	0.31207	2	0	0	0
1835	Nucleoside diphosphate-linked moiety X motif 19, mitochondrial	IPI00869107	0.000	0.31207	0	0	0	0
1836	cDNA, FLJ79450, highly similar to 3-ketoacyl-CoA thiolase, peroxisomal	IPI00011522	0.000	0.31207	0	2	0	0
1837	Isoform 1 of Interferon regulatory factor 2-binding protein 2	IPI00376199	0.000	0.31207	0	0	2	0
1838	Nucleus accumbens-associated protein 1	IPI00045207	0.000	0.31207	2	2	0	0
1839	Putative uncharacterized protein C9orf86	IPI00186586	0.000	0.31207	0	0	0	0
1840	S-adenosyl-L-methionine-dependent methyltransferase FTSJD2	IPI00166153	0.000	0.31207	0	0	0	0
1841	Isoform 3 of Sorting nexin-27	IPI00328097	0.000	0.31207	0	2	0	0
1842	Isoform 1 of Tuftelin-interacting protein 11	IPI00015924	0.000	0.31207	2	0	0	0
1843	Isoform 1 of Kinesin-like protein KIF15	IPI00024975	0.000	0.31207	2	2	0	0
1844	Biogenesis of lysosome-related organelles complex 1 subunit 1	IPI00020319	0.000	0.31207	2	0	0	0
1845	Ubiquitin-fold modifier 1	IPI00010207	0.000	0.31207	0	0	0	0
1846	HLA class I histocompatibility antigen, A-1 alpha chain	IPI00026569	0.000	0.31207	2	0	0	0
1847	Isoform 1 of DNA-directed RNA polymerase I subunit RPA49	IPI00251989	0.000	0.31207	0	0	2	2
1848	Translation initiation factor IF-2, mitochondrial	IPI00005039	0.000	0.31207	2	2	0	0
1849	Ribosomal protein 63, mitochondrial	IPI00031485	0.000	0.31207	0	2	2	2
1850	28S ribosomal protein S21, mitochondrial	IPI00014812	0.000	0.31207	0	0	2	0
1851	Mitochondrial import inner membrane translocase subunit Tim8 A	IPI00028376	0.000	0.31207	0	0	0	0
1852	Isoform Sp100-HMG of Nuclear autoantigen Sp-100	IPI00011675	0.000	0.31207	0	0	0	2
1853	Isoform Alpha of Caspase-6	IPI00023876	0.000	0.31207	0	0	0	0
1854	Isoform 2 of Ras association domain-containing protein 6	IPI00176707	0.000	0.31207	0	2	0	0
1855	Probable 7,8-dihydro-8-oxoguanine triphosphatase NUDT15	IPI00019487	0.000	0.31207	0	0	0	0
1856	Isoform 1 of Protein FAM115A	IPI00006050	0.000	0.31207	2	0	0	0
1857	Isoform 1 of Vesicle transport protein USE1	IPI00020515	0.000	0.31207	0	2	2	0
1858	Kinetochore protein Spc25	IPI00010219	0.000	0.31207	0	2	0	0
1859	DNA polymerase epsilon subunit 3	IPI00010141	0.000	0.31207	0	0	0	0
1860	Mothers against decapentaplegic homolog 5	IPI00017730	0.000	0.31207	0	0	2	0
1861	Arginase-2, mitochondrial	IPI00020332	0.000	0.31207	0	0	2	0
1862	cDNA FLJ58573, highly similar to Exonuclease 3'-5' domain-like-containing protein 2	IPI00465113	0.000	0.31207	0	0	2	0
1863	Serine/threonine-protein phosphatase 2A 56 kDa regulatory subunit alpha isoform	IPI00014978	0.000	0.31207	0	0	2	0
1864	Anaphase-promoting complex subunit 10	IPI00007088	0.000	0.31207	2	0	0	0
1865	Tripartite motif-containing protein 65	IPI00419451	0.000	0.31207	2	0	0	0
1866	UPF0552 protein C15orf38	IPI00074225	0.000	0.31207	0	2	0	2
1867	Isoform 1 of Mediator of RNA polymerase II transcription subunit 27	IPI00302652	0.000	0.31207	0	0	0	2
1868	Isoform 1 of Kelch domain-containing protein 4	IPI00152182	0.000	0.31207	2	0	0	0
1869	Isoform 1 of Multiple inositol polyphosphate phosphatase 1	IPI00293748	0.000	0.31207	0	0	2	0
1870	Isoform RMO1abc of Ras-associated and pleckstrin homology domains-containing protein 1	IPI00402234	0.000	0.31207	0	0	0	2
1871	WD repeat-containing protein 70	IPI00300060	0.000	0.31207	0	0	0	2
1872	Sorting nexin-5	IPI00295209	0.000	0.31207	0	0	0	0
1873	Isoform 1 of Phosphatidylinositol 3-kinase regulatory subunit alpha	IPI00021448	0.000	0.31207	0	0	0	2
1874	Protein SEC13 homolog	IPI00375370	0.000	0.31207	0	2	0	0
1875	Serine/threonine-protein kinase Chk1	IPI00023664	0.000	0.31207	0	0	0	0
1876	Methylmalonate-semialdehyde dehydrogenase [acylating], mitochondria	IPI00024990	0.000	0.31207	2	0	0	0
1877	DNA replication complex GINS protein PSF2	IPI00007146	0.000	0.31207	0	2	0	0
1878	NICE-4 protein (Fragment)	IPI00005416	0.000	0.31207	0	0	2	0
1879	cDNA: FLJ22686 fis, clone HSI10987	IPI00306532	0.000	0.31207	0	0	0	0
1880	Serine/threonine-protein kinase 38	IPI00027251	0.000	0.31207	0	2	0	0
1881	Delta-1-pyrroline-5-carboxylate dehydrogenase, mitochondrial	IPI00217871	0.000	0.31207	0	0	0	2
1882	Isoform 2 of Gamma-adducin	IPI00004408	0.000	0.31207	0	0	2	0
1883	Dolichyl-phosphate beta-glucosyltransferase	IPI00002506	0.000	0.31207	0	0	0	0
1884	FLJ00369 protein (Fragment)	IPI00166711	0.000	0.31207	0	0	0	2
1885	ADP-ribosylation factor-like protein 6	IPI00021685	0.000	0.31207	0	2	0	0

No.	Description	Accession number	STN[1]	p-Value[1]	Con_A[2]	Con_B[2]	SORA_A[2]	SORA_B[2]
1886	Isoform 1 of Hydroxysteroid dehydrogenase-like protein 2	IPI00414384	0.000	0.31207	2	0	0	0
1887	Isoform 1 of Peroxisomal membrane protein PEX14	IPI00025346	0.000	0.31207	0	0	0	0
1888	103 kDa protein	IPI00402008	0.000	0.31207	0	2	0	0
1889	Isoform 1 of Ribosome-recycling factor, mitochondrial	IPI00061108	0.000	0.31207	0	0	2	0
1890	TBC1 domain family member 7	IPI00009425	0.000	0.31207	0	2	0	0
1891	Protein AATF	IPI00302238	0.000	0.31207	2	2	0	0
1892	Isoform 1 of Presenilin-1	IPI00028077	0.000	0.31207	0	2	0	0
1893	Ras-related protein Rap-2b	IPI00018364	0.000	0.31207	0	0	0	2
1894	Mediator of RNA polymerase II transcription subunit 20	IPI00174852	0.000	0.31207	0	0	0	0
1895	Isoform 1 of Ribulose-phosphate 3-epimerase	IPI00335280	0.000	0.31207	0	2	0	0
1896	Isoform 1 of Alpha-globin transcription factor CP2	IPI00037599	0.000	0.31207	0	0	0	0
1897	Ras-related protein Rab-24	IPI00056496	0.000	0.31207	0	0	0	0
1898	Isoform 1 of Dermal papilla-derived protein 6	IPI00328185	0.000	0.31207	2	0	0	2
1899	Isoform 2 of cAMP-dependent protein kinase catalytic subunit beta	IPI00376119	0.000	0.31207	0	0	2	0
1900	Putative uncharacterized protein DKFZp686C1054	IPI00465054	0.000	0.31207	0	0	2	0
1901	3-oxoacyl-[acyl-carrier-protein] synthase, mitochondrial	IPI00016637	0.000	0.31207	2	0	0	0
1902	Putative uncharacterized protein DKFZp686G0859	IPI00470477	0.000	0.31207	2	2	0	0
1903	Isoform 2 of SAP30-binding protein	IPI00333699	0.000	0.31207	2	0	0	0
1904	Isoform 1 of Lysophospholipid acyltransferase LPCAT4	IPI00385128	0.000	0.31207	0	2	0	0
1905	Transmembrane protein 102	IPI00167764	0.000	0.31207	0	0	0	0
1906	Ubiquilin-4	IPI00024502	0.000	0.31207	2	0	0	0
1907	Isoform 2 of Monoacylglycerol lipase ABHD12	IPI00060569	0.000	0.31207	2	0	0	0
1908	Thyroid transcription factor 1-associated protein 26	IPI00329594	0.000	0.31207	0	0	0	0
1909	Isoform 3 of Poly [ADP-ribose] polymerase 16	IPI00297151	0.000	0.31207	0	0	0	2
1910	Isoform HERA-A of GTP-binding protein era homolog	IPI00026512	0.000	0.31207	0	0	0	0
1911	Isoform 1 of TraB domain-containing protein	IPI00008732	0.000	0.31207	0	0	0	2
1912	Isoform 1 of DNA replication complex GINS protein SLD5	IPI00031614	0.000	0.31207	0	0	0	2
1913	Isoform 1 of GPI transamidase component PIG-S	IPI00465308	0.000	0.31207	0	0	0	0
1914	Kelch repeat and BTB domain-containing protein 2	IPI00293363	0.000	0.31207	0	2	0	2
1915	Structural maintenance of chromosomes protein 5	IPI00413265	0.000	0.31207	0	0	0	0
1916	33 kDa protein	IPI00386323	0.000	0.31207	0	2	0	0
1917	Isoform 2 of Golgin subfamily A member 2	IPI00413895	0.000	0.31207	2	2	0	0
1918	Isoform 1 of DnaJ homolog subfamily C member 21	IPI00142716	0.000	0.31207	0	2	0	0
1919	Isoform 1 of AT-rich interactive domain-containing protein 2	IPI00470537	0.000	0.31207	0	0	0	0
1920	Isoform 1 of Splicing factor, arginine/serine-rich 12	IPI00103497	0.000	0.31207	0	0	0	0
1921	Glutamine-dependent NAD(+) synthetase	IPI00306689	0.000	0.31207	2	0	0	0
1922	Acyl-CoA-binding domain-containing protein 6	IPI00031680	0.000	0.31207	2	0	0	0
1923	KIAA1033 protein	IPI00298991	0.000	0.31207	0	0	0	2
1924	Isoform 1 of Zinc finger protein 638	IPI00470596	0.000	0.31207	0	0	0	0
1925	Digestive organ expansion factor homolog	IPI00004290	0.000	0.31207	2	0	0	0
1926	Isoform 1 of Constitutive coactivator of PPAR-gamma-like protein 2	IPI00394751	0.000	0.31207	0	0	0	0
1927	Ribonuclease P protein subunit p14	IPI00215966	0.000	0.31207	0	0	0	2
1928	Protein yippee-like 5	IPI00429538	0.000	0.31207	0	0	0	0
1929	Ras-related protein Rab-20	IPI00015839	0.000	0.31207	2	0	0	0
1930	Isoform 1 of Dehydrogenase/reductase SDR family member 11	IPI00034280	0.000	0.31207	0	0	0	0
1931	High mobility group protein B1	IPI00419258	0.000	0.31207	0	0	2	0
1932	MARCKS-related protein	IPI00641181	0.000	0.31207	0	0	0	0
1933	Trafficking protein particle complex subunit 1	IPI00009654	0.000	0.31207	2	0	0	0
1934	Isoform 1 of Ran guanine nucleotide release factor	IPI00025081	0.000	0.31207	2	0	0	0
1935	Transmembrane emp24 domain-containing protein 7	IPI00032825	0.000	0.31207	2	0	0	0
1936	Ras-related protein Rab-5B	IPI00017344	0.000	0.31207	0	0	2	0
1937	Isoform 2 of Transcription elongation factor A protein 1	IPI00218106	0.000	0.31207	0	0	0	0
1938	NudC domain-containing protein 3	IPI00238209	0.000	0.31207	2	0	0	0
1939	Isoform 1 of Septin-10	IPI00374970	0.000	0.31207	0	0	0	0
1940	cDNA FLJ55380, highly similar to Protein kinase C-binding protein 1	IPI00418316	0.000	0.31207	2	0	0	0
1941	cDNA FLJ44925 fis, clone BRAMY3014613	IPI00444788	0.000	0.31207	0	2	0	0
1942	Isoform 1 of Mitochondrial inner membrane protease subunit 2	IPI00045908	0.000	0.31207	2	0	0	0
1943	Putative uncharacterized protein PYCRL	IPI00604402	0.000	0.31207	2	2	0	0
1944	cDNA FLJ56452, highly similar to Echinoderm microtubule-associated protein-like 2	IPI00015944	0.000	0.31207	0	0	0	2
1945	Hydroxymethylglutaryl-CoA lyase, mitochondrial	IPI00293564	0.000	0.31207	2	0	0	0
1946	Beta-centractin	IPI00029469	0.000	0.31207	0	2	0	0
1947	Isoform 1 of Lysophosphatidic acid phosphatase type 6	IPI00099838	0.000	0.31207	2	0	0	0
1948	Protein FAM134C	IPI00254338	0.000	0.31207	2	0	0	0
1949	Methylcrotonoyl-CoA carboxylase subunit alpha, mitochondrial	IPI00024580	0.000	0.31207	0	0	0	2
1950	Isoform 1 of Glutaminase kidney isoform, mitochondrial	IPI00289159	0.000	0.31207	0	0	0	2
1951	Isoform 1 of Protein VPRBP	IPI00329528	0.000	0.31207	0	2	0	0
1952	Isoform 2 of Coiled-coil domain-containing protein 132	IPI00103148	0.000	0.31207	2	0	2	0
1953	Ubiquitin-conjugating enzyme E2 C	IPI00013002	0.000	0.31207	2	0	0	0
1954	Histidine triad nucleotide-binding protein 2, mitochondrial	IPI00000335	0.000	0.31207	2	0	0	0
1955	Isoform 1 of BTB/POZ domain-containing protein KCTD10	IPI00011209	0.000	0.31207	2	0	0	0
1956	glycogen [starch] synthase, muscle isoform 2	IPI00157144	0.000	0.31207	2	0	0	0
1957	Isoform 1 of NHL repeat-containing protein 2	IPI00301051	0.000	0.31207	0	2	0	0
1958	Aldose 1-epimerase	IPI00060200	0.000	0.31207	0	0	0	2
1959	Protein CTF18 homolog	IPI00178203	0.000	0.31207	0	2	0	0
1960	Probable glutamyl-tRNA(Gln) amidotransferase subunit B, mitochondrial	IPI00027228	0.000	0.31207	2	0	0	0
1961	Ubiquitin carboxyl-terminal hydrolase 8	IPI00030915	0.000	0.31207	0	0	0	2
1962	Isoform 1 of MAP kinase-activating death domain protein	IPI00103536	0.000	0.31207	2	0	0	0
1963	Protein Red	IPI00011875	0.000	0.31207	2	2	0	0
1964	Leucine zipper transcription factor-like protein 1	IPI00299465	0.000	0.31207	2	0	0	0
1965	Sodium-dependent multivitamin transporter	IPI00031822	0.000	0.31207	0	0	0	0
1966	Iron-sulfur cluster assembly 2 homolog, mitochondrial	IPI00376195	0.000	0.31207	0	2	0	0
1967	NF-kappa-B-activating protein	IPI00296934	0.000	0.31207	0	0	0	2
1968	Charged multivesicular body protein 4c	IPI00060414	0.000	0.31207	2	0	0	0
1969	Transcription factor MafG	IPI00007311	0.000	0.31207	0	0	0	0
1970	Isoform 1 of Nicotinamide mononucleotide adenylyltransferase 3	IPI00290687	0.000	0.31207	0	0	0	2
1971	Probable asparaginyl-tRNA synthetase, mitochondrial	IPI00101664	0.000	0.31207	0	0	0	2
1972	Isoform 2 of Insulin-like growth factor 2 mRNA-binding protein 3	IPI00165467	0.000	0.31207	0	0	0	0
1973	Isoform 1 of Homologous-pairing protein 2 homolog	IPI00009805	0.000	0.31207	0	0	0	0
1974	Isoform 2 of Sec1 family domain-containing protein 2	IPI00141564	0.000	0.31207	0	0	0	0
1975	HEAT repeat-containing protein 6	IPI00464999	0.000	0.31207	0	0	0	0
1976	Isoform 2 of ADP-ribosylation factor-binding protein GGA1	IPI00216337	0.000	0.31207	0	0	0	0
1977	Activating signal cointegrator 1	IPI00018840	0.000	0.31207	0	2	0	0
1978	Isoform 3 of Fanconi anemia group I protein	IPI00306518	0.000	0.31207	2	0	0	0
1979	cDNA FLJ55996, highly similar to Conserved oligomeric Golgi complex component 8	IPI00140201	0.000	0.31207	2	0	0	0
1980	Isoform 1 of ATPase family AAA domain-containing protein 5	IPI00102575	0.000	0.31207	0	2	0	0

No.	Description	Accession number	STN[1]	p-Value[1]	Con_A[2]	Con_B[2]	SORA_A[2]	SORA_B[2]
1981	Isoform 2 of Abnormal spindle-like microcephaly-associated protein	IPI00480042	0.000	0.31207	2	0	0	0
1982	Isoform 1 of NGFI-A-binding protein 2	IPI00019432	0.000	0.31207	0	2	0	0
1983	Immediate early response 3-interacting protein 1	IPI00007166	0.000	0.31207	2	0	0	0
1984	cDNA FLJ56350, highly similar to HESB-like domain-containing protein 2, mitochondrial	IPI00329615	0.000	0.31207	2	0	0	0
1985	Transcription initiation factor TFIID subunit 10	IPI00030364	0.000	0.31207	0	0	0	0
1986	CD2 antigen cytoplasmic tail-binding protein 2	IPI00006103	0.000	0.31207	2	0	0	0
1987	poly(A) polymerase beta	IPI00024525	0.000	0.31207	0	0	0	2
1988	Thioredoxin-like protein 4A	IPI00216338	0.000	0.31207	0	0	0	0
1989	Full-length cDNA clone CS0DI031YK16 of Placenta of Homo sapiens	IPI00384157	0.000	0.31207	0	0	0	0
1990	Isoform 3 of Syntenin-2	IPI00220218	0.000	0.31207	0	0	0	2
1991	Isoform 1 of Probable E3 ubiquitin-protein ligase HERC4	IPI00333067	0.000	0.31207	2	0	0	0
1992	Isoform 1 of Pericentriolar material 1 protein	IPI00006213	0.000	0.31207	2	0	0	0
1993	Something about silencing protein 10	IPI00006900	0.000	0.31207	2	0	0	0
1994	Protein S100-A9	IPI00027462	0.000	0.31207	2	0	0	0
1995	Isoform 2 of N-acetylserotonin O-methyltransferase-like protein	IPI00249080	0.000	0.31207	2	0	0	0
1996	Isoform 3 of Ral GTPase-activating protein subunit alpha-2	IPI00455174	0.000	0.31207	0	0	0	2
1997	Isoform 2 of Inositol hexakisphosphate and diphosphoinositol-pentakisphosphate kinase 2	IPI00178375	0.000	0.31207	0	0	2	0
1998	Uncharacterized protein C6orf125	IPI00031617	0.000	0.31207	0	2	0	0
1999	Acylphosphatase-2	IPI00216461	0.000	0.31207	2	0	0	0
2000	Isoform 2 of Tescalcin	IPI00791863	0.000	0.31207	0	2	0	0
2001	Mitogen-activated protein kinase 13	IPI00005741	0.000	0.31207	2	0	0	0
2002	Nuclear fragile X mental retardation-interacting protein 2	IPI00002349	0.000	0.31207	0	2	0	0
2003	Isoform 1 of Dynamin-1-like protein	IPI00146935	0.000	0.31207	0	0	0	0
2004	Putative uncharacterized protein DHX30	IPI00164906	0.000	0.31207	0	0	2	0
2005	Isoform GN-1L of Glycogenin-1	IPI00180386	0.000	0.31207	0	2	0	0
2006	cDNA FLJ12528 fis, clone NT2RM4000155, moderately similar to THREONYL-TRNA SYNTHETASE	IPI00018632	0.000	0.31207	2	0	0	0
2007	SAFB-like transcription modulator isoform b	IPI00019996	0.000	0.31207	0	0	0	2
2008	Phosphoribosyl pyrophosphate synthetase 1	IPI00552495	0.000	0.31207	0	0	0	2
2009	N-acylneuraminate-9-phosphatase	IPI00152196	0.000	0.31207	0	0	0	0
2010	Isoform 2 of Multiple inositol polyphosphate phosphatase 1	IPI00028553	0.000	0.31207	0	0	2	0
2011	Splicing factor, arginine/serine-rich 8	IPI00290094	0.000	0.31207	0	2	0	0
2012	Protein Mis18-beta	IPI00001999	0.000	0.31207	0	2	0	0
2013	Kinesin light chain 2	IPI00021634	0.000	0.31207	0	0	0	0
2014	Putative uncharacterized protein MTM1	IPI00639863	0.000	0.31207	0	0	0	2
2015	Plastin-2	IPI00010471	-0.071	0.28122	81	74	70	84
2016	Isoform 3 of Spectrin alpha chain, brain	IPI00843765	-0.080	0.28088	51	48	49	49
2017	Hypoxanthine-guanine phosphoribosyltransferase	IPI00218493	-0.081	0.28088	40	54	41	52
2018	Histone H2B type 2-E	IPI00003935	-0.082	0.28088	34	55	42	46
2019	Protein disulfide-isomerase A4	IPI00009904	-0.086	0.28073	41	36	37	39
2020	Estradiol 17-beta-dehydrogenase 12	IPI00007676	-0.091	0.28028	20	41	23	37
2021	Isoform 5 of Interleukin enhancer-binding factor 3	IPI00219330	-0.094	0.28001	28	26	26	27
2022	Isoform C1 of Heterogeneous nuclear ribonucleoproteins C1/C2	IPI00216592	-0.096	0.27994	23	28	25	25
2023	Annexin A5	IPI00329801	-0.096	0.27994	23	27	25	24
2024	Isoform 1 of Calcyclin-binding protein	IPI00395627	-0.098	0.27941	21	25	22	23
2025	Isoform 1 of Extended synaptotagmin-1	IPI00022143	-0.099	0.27929	20	25	22	22
2026	Glycogen phosphorylase, liver form	IPI00783313	-0.099	0.27918	23	21	21	22
2027	Proteasome subunit alpha type-2	IPI00219622	-0.101	0.27903	19	23	15	26
2028	cDNA FLJ75085, highly similar to Homo sapiens glutaminyl-tRNA synthetase (QARS), mRNA	IPI00026665	-0.102	0.27880	24	16	18	21
2029	Ras-related protein Rab-7a	IPI00016342	-0.102	0.27880	16	24	18	21
2030	Isoform 1 of 40S ribosomal protein S24	IPI00029750	-0.103	0.27869	18	21	16	22
2031	DNA replication licensing factor MCM2	IPI00184330	-0.104	0.27835	18	19	16	20
2032	EH domain-containing protein 4	IPI00005578	-0.106	0.27778	18	17	16	18
2033	Nucleosome assembly protein 1-like 1	IPI00023860	-0.108	0.27691	18	15	16	16
2034	Isoform Alpha-6X1X2B of Integrin alpha-6	IPI00010697	-0.109	0.27612	17	14	16	14
2035	Isoform 1 of Pyridoxal kinase	IPI00013004	-0.109	0.27612	11	20	12	18
2036	Isoform 1 of Far upstream element-binding protein 1	IPI00375441	-0.110	0.27585	14	16	14	15
2037	Isoform SERCA1B of Sarcoplasmic/endoplasmic reticulum calcium ATPase 1	IPI00024804	-0.110	0.27585	17	13	13	16
2038	Trifunctional enzyme subunit beta, mitochondrial	IPI00022793	-0.110	0.27585	15	15	14	15
2039	Delta(3,5)-Delta(2,4)-dienoyl-CoA isomerase, mitochondrial	IPI00011416	-0.111	0.27563	16	13	15	13
2040	protein arginine N-methyltransferase 5 isoform b	IPI00064328	-0.111	0.27563	11	18	12	16
2041	Isoform 1 of ATP-dependent RNA helicase DDX19B	IPI00008943	-0.116	0.27400	8	17	8	16
2042	Alcohol dehydrogenase [NADP+]	IPI00220271	-0.116	0.27400	14	11	12	12
2043	60S ribosomal protein L22	IPI00219153	-0.117	0.27336	8	16	14	9
2044	DCN1-like protein 5	IPI00165361	-0.117	0.27336	13	11	13	10
2045	Calponin-2	IPI00015262	-0.119	0.27291	13	10	12	10
2046	Peptidyl-prolyl cis-trans isomerase FKBP2	IPI00002535	-0.119	0.27291	10	13	9	13
2047	Isoform 1 of Serine/threonine-protein phosphatase 2A 65 kDa regulatory subunit A beta isoform	IPI00294178	-0.120	0.27241	10	12	10	11
2048	Protein flightless-1 homolog	IPI00031023	-0.120	0.27241	11	11	10	11
2049	Isoform 2 of Proteasome subunit alpha type-3	IPI00171199	-0.120	0.27241	11	11	10	11
2050	Stathmin	IPI00479997	-0.122	0.27128	8	13	8	12
2051	Nucleolar pre-ribosomal-associated protein 1	IPI00297241	-0.123	0.27068	10	10	7	12
2052	WD repeat-containing protein 36	IPI00169325	-0.123	0.27068	11	9	6	13
2053	DNA-(apurinic or apyrimidinic site) lyase	IPI00215911	-0.123	0.27068	12	8	10	9
2054	Eukaryotic translation initiation factor 3 subunit I	IPI00012795	-0.125	0.27011	9	10	10	8
2055	Isoform 1 of Core-binding factor subunit beta	IPI00016746	-0.125	0.27011	10	9	9	9
2056	Isoform 1 of Septin-2	IPI00014177	-0.125	0.27011	9	10	9	9
2057	Isoform Short of RNA-binding protein FUS	IPI00221354	-0.125	0.27011	10	9	9	9
2058	Dihydrolipoyllysine-residue acetyltransferase component of pyruvate dehydrogenase complex	IPI00021338	-0.125	0.27011	10	9	8	10
2059	Activated RNA polymerase II transcriptional coactivator p15	IPI00221222	-0.127	0.26947	7	11	7	10
2060	Isoform 4 of Serine/threonine-protein phosphatase 6 regulatory subunit 3	IPI00019540	-0.127	0.26947	9	9	9	8
2061	Splicing factor, arginine/serine-rich 2	IPI00005978	-0.127	0.26947	10	8	9	8
2062	Isoform 1 of Protein canopy homolog 2	IPI00443909	-0.127	0.26947	8	10	11	6
2063	Isoform 2 of Cat eye syndrome critical region protein 5	IPI00011511	-0.127	0.26947	10	8	8	9
2064	Isoform 1 of Glycerol-3-phosphate dehydrogenase, mitochondrial	IPI00017895	-0.127	0.26947	8	10	9	8
2065	Signal recognition particle receptor subunit alpha	IPI00385267	-0.129	0.26833	7	10	8	8
2066	Ribosome maturation protein SBDS	IPI00427330	-0.129	0.26833	6	11	5	11
2067	Copine-1	IPI00018452	-0.129	0.26833	9	8	8	8
2068	Thymidylate kinase	IPI00013862	-0.131	0.26750	8	8	7	8
2069	Isoform 2 of Serine/threonine-protein phosphatase PGAM5, mitochondrial	IPI00063242	-0.131	0.26750	7	9	5	10
2070	Signal recognition particle 72 kDa protein	IPI00215888	-0.131	0.26750	7	9	8	7
2071	Putative uncharacterized protein MDH1	IPI00915869	-0.131	0.26750	8	8	7	8
2072	Thioredoxin domain-containing protein 12	IPI00026328	-0.131	0.26750	9	7	8	7
2073	Isoform 1 of Enoyl-CoA hydratase domain-containing protein 1	IPI00302688	-0.131	0.26750	10	6	3	12
2074	GTP:AMP phosphotransferase mitochondrial	IPI00465256	-0.131	0.26750	9	7	7	8
2075	Isoform 2 of Transportin-3	IPI00395694	-0.133	0.26569	7	8	9	5

No.	Description	Accession number	STN[1]	p-Value[1]	Con_A[2]	Con_B[2]	SORA_A[2]	SORA_B[2]
2076	FKBP1A protein	IPI00413778	-0.133	0.26569	8	7	6	8
2077	Regulation of nuclear pre-mRNA domain-containing protein 1B	IPI00009659	-0.133	0.26569	7	8	6	8
2078	Isoform 1 of Gamma-glutamylcyclotransferase	IPI00031564	-0.133	0.26569	8	7	9	5
2079	Isoform 1 of Low molecular weight phosphotyrosine protein phosphatase	IPI00219861	-0.133	0.26569	7	8	6	8
2080	Isoform 1 of Host cell factor 1	IPI00019848	-0.136	0.26444	6	8	9	4
2081	Splicing factor, arginine/serine-rich 9	IPI00012340	-0.136	0.26444	9	5	7	6
2082	SDHA protein	IPI00217143	-0.136	0.26444	7	7	6	7
2083	NEDD8	IPI00020008	-0.136	0.26444	7	7	6	7
2084	Similar to Signal peptidase complex subunit 2	IPI00452747	-0.136	0.26444	6	8	6	7
2085	Exportin-T	IPI00306290	-0.136	0.26444	6	8	6	7
2086	Isoform 1 of Transcription elongation factor SPT6	IPI00784161	-0.136	0.26444	7	7	7	6
2087	Isoform 1 of RNA-binding protein Musashi homolog 2	IPI00073713	-0.136	0.26444	7	7	7	6
2088	Isoform 1 of KH domain-containing, RNA-binding, signal transduction-associated protein 1	IPI00008575	-0.139	0.26213	7	6	5	7
2089	Prostaglandin E synthase 3	IPI00015029	-0.139	0.26213	5	8	6	6
2090	Ribosome biogenesis protein WDR12	IPI00304232	-0.139	0.26213	7	6	7	5
2091	TRIP12 protein	IPI00032342	-0.139	0.26213	6	7	3	9
2092	Eukaryotic translation initiation factor 3 subunit K	IPI00033143	-0.139	0.26213	6	7	6	6
2093	Periodic tryptophan protein 2 homolog	IPI00300078	-0.142	0.26066	7	5	3	8
2094	DEAD (Asp-Glu-Ala-Asp) box polypeptide 39, isoform CRA_c	IPI00166874	-0.142	0.26066	4	8	5	6
2095	60S ribosomal protein L27a	IPI00456758	-0.142	0.26066	5	7	6	5
2096	Serine/threonine-protein phosphatase 2A 55 kDa regulatory subunit B alpha isoform	IPI00332511	-0.142	0.26066	8	4	7	4
2097	Similar to nonhistone chromosomal protein HMG-1	IPI00418184	-0.142	0.26066	5	7	6	5
2098	General transcription factor IIF subunit 2	IPI00477686	-0.142	0.26066	7	5	5	6
2099	Myosin regulatory light chain 12B	IPI00033494	-0.142	0.26066	6	6	5	6
2100	Isoform 1 of Coiled-coil domain-containing protein 47	IPI00024642	-0.142	0.26066	7	5	5	6
2101	Programmed cell death protein 5	IPI00023640	-0.142	0.26066	6	6	5	6
2102	Isoform 1 of LIM and SH3 domain protein 1	IPI00000861	-0.142	0.26066	6	6	6	5
2103	DNA polymerase delta subunit 2	IPI00025616	-0.142	0.26066	8	4	6	5
2104	ADP-ribosylation factor-like protein 3	IPI00003327	-0.142	0.26066	6	6	4	7
2105	Ubiquitin-conjugating enzyme E2 L3	IPI00021347	-0.145	0.25786	6	5	5	5
2106	DNA polymerase alpha catalytic subunit	IPI00220317	-0.145	0.25786	7	4	7	3
2107	Uncharacterized protein C17orf25	IPI00007102	-0.145	0.25786	3	8	5	5
2108	ATP-dependent Clp protease ATP-binding subunit clpX-like, mitochondrial	IPI00008728	-0.145	0.25786	4	7	5	5
2109	Isoform 1 of Ribose-phosphate pyrophosphokinase 2	IPI00219617	-0.145	0.25786	5	6	6	4
2110	Myosin-Ie	IPI00329672	-0.145	0.25786	7	4	5	5
2111	Isoform 1 of CLIP-associating protein 1	IPI00396279	-0.145	0.25786	6	5	6	4
2112	BAG family molecular chaperone regulator 2	IPI00000643	-0.145	0.25786	5	6	4	6
2113	tRNA methyltransferase 112 homolog	IPI00009010	-0.145	0.25786	6	5	5	5
2114	Isoform 2 of tRNA pseudouridine synthase A	IPI00001716	-0.145	0.25786	5	6	7	3
2115	Transmembrane protein 126A	IPI00031064	-0.145	0.25786	4	7	3	7
2116	Isoform 2 of Mitochondrial import inner membrane translocase subunit TIM50	IPI00418497	-0.145	0.25786	6	5	6	4
2117	U1 small nuclear ribonucleoprotein A	IPI00012382	-0.149	0.25571	3	7	4	5
2118	annexin A6 isoform 2	IPI00002459	-0.149	0.25571	6	4	5	4
2119	Importin subunit alpha-1	IPI00303292	-0.149	0.25571	6	4	4	5
2120	Isoform 2 of Histone deacetylase 2	IPI00289601	-0.149	0.25571	4	6	4	5
2121	Isoform 1 of Transmembrane protein 111	IPI00020472	-0.149	0.25571	5	5	4	5
2122	Isoform 2 of Ubiquitin thioesterase OTUB1	IPI00409750	-0.149	0.25571	4	6	5	4
2123	Heme-binding protein 1	IPI00148063	-0.149	0.25571	5	5	3	6
2124	Coiled-coil domain-containing protein 124	IPI00060627	-0.149	0.25571	4	6	4	5
2125	pyrroline-5-carboxylate reductase 1, mitochondrial isoform 2	IPI00376503	-0.149	0.25571	4	6	3	6
2126	28S ribosomal protein S28, mitochondrial	IPI00022276	-0.149	0.25571	6	4	3	6
2127	ADP-ribosylation factor-like protein 2	IPI00003326	-0.149	0.25571	4	6	5	4
2128	Isoform 2 of Protein-glutamine gamma-glutamyltransferase 2	IPI00218251	-0.149	0.25571	6	4	4	5
2129	Mitochondrial 28S ribosomal protein S2	IPI00006970	-0.154	0.25253	3	6	4	4
2130	Mitochondrial import receptor subunit TOM70	IPI00015602	-0.154	0.25253	7	2	4	4
2131	Isoform 1 of 1-phosphatidylinositol-4,5-bisphosphate phosphodiesterase gamma-1	IPI00016736	-0.154	0.25253	4	5	2	6
2132	Isoform 2 of 39S ribosomal protein L39, mitochondrial	IPI00084571	-0.154	0.25253	5	4	4	4
2133	CTP synthase 2	IPI00645702	-0.154	0.25253	4	5	4	4
2134	39S ribosomal protein L23, mitochondrial	IPI00293476	-0.154	0.25253	5	4	5	3
2135	Sorting nexin-9	IPI00001883	-0.154	0.25253	5	4	4	4
2136	RNA-binding motif protein, X-linked-like-2	IPI00004450	-0.154	0.25253	4	5	4	4
2137	Pyruvate dehydrogenase protein X component, mitochondrial	IPI00298423	-0.154	0.25253	4	5	3	5
2138	Metaxin-2	IPI00025717	-0.154	0.25253	5	4	3	5
2139	Ladinin-1	IPI00514234	-0.154	0.25253	4	5	4	4
2140	39S ribosomal protein L9, mitochondrial	IPI00307409	-0.154	0.25253	4	5	4	4
2141	4-hydroxyphenylpyruvate dioxygenase-like protein	IPI00063762	-0.154	0.25253	4	5	4	4
2142	Pyrroline-5-carboxylate reductase 2	IPI00470610	-0.154	0.25253	4	5	3	5
2143	Succinate dehydrogenase [ubiquinone] iron-sulfur subunit, mitochondrial	IPI00294911	-0.154	0.25253	5	4	3	5
2144	cDNA FLJ78497	IPI00289535	-0.154	0.25253	5	4	2	6
2145	22 kDa protein	IPI00219910	-0.154	0.25253	4	5	5	3
2146	Translation initiation factor eIF-2B subunit alpha	IPI00221300	-0.154	0.25253	0	7	4	4
2147	Isoform 1 of Huntingtin-interacting protein K	IPI00335001	-0.154	0.25253	2	7	0	6
2148	U6 snRNA-associated Sm-like protein LSm2	IPI00032460	-0.154	0.25253	4	5	3	5
2149	ADP/ATP translocase 1	IPI00022891	-0.159	0.24909	2	6	2	5
2150	Phosphatidylinositol-4-phosphate 3-kinase C2 domain-containing subunit alpha	IPI00002580	-0.159	0.24909	3	5	3	4
2151	Isoform 2 of AP-3 complex subunit delta-1	IPI00289608	-0.159	0.24909	4	4	3	4
2152	Probable ribosome biogenesis protein NEP1	IPI00025347	-0.159	0.24909	3	5	4	3
2153	cDNA FLJ56402, highly similar to Tripeptidyl-peptidase 1	IPI00298237	-0.159	0.24909	4	4	4	3
2154	Protein NipSnap homolog 3A	IPI00004845	-0.159	0.24909	3	5	2	5
2155	Chromobox protein homolog 5	IPI00024662	-0.159	0.24909	3	5	3	4
2156	Isoform 2 of Double-stranded RNA-specific adenosine deaminase	IPI00025057	-0.159	0.24909	4	4	3	4
2157	28S ribosomal protein S10, mitochondrial	IPI00061245	-0.159	0.24909	4	4	3	4
2158	Malectin	IPI00029046	-0.159	0.24909	2	6	2	5
2159	ATPase ASNA1	IPI00013466	-0.159	0.24909	4	4	3	4
2160	Developmentally-regulated GTP-binding protein 2	IPI00022697	-0.159	0.24909	6	2	4	3
2161	Endoplasmic reticulum resident protein 44	IPI00401264	-0.159	0.24909	5	3	4	3
2162	Coproporphyrinogen-III oxidase, mitochondrial	IPI00093057	-0.159	0.24909	4	4	3	4
2163	Isoform 1 of Metaxin-1	IPI00013678	-0.159	0.24909	6	2	2	5
2164	Ubiquitin-conjugating enzyme E2 O	IPI00783378	-0.159	0.24909	4	4	5	2
2165	programmed cell death 4 isoform 2	IPI00240675	-0.159	0.24909	4	4	2	5
2166	Ribosomal protein S6 kinase alpha-1	IPI00017305	-0.159	0.24909	6	2	3	4
2167	Isoform 1 of Splicing factor, arginine/serine-rich 15	IPI00181702	-0.159	0.24909	5	3	3	4
2168	BRO1 domain-containing protein BROX	IPI00065500	-0.159	0.24909	5	3	4	3
2169	Isoform 1 of RNA polymerase II-associated factor 1 homolog	IPI00300333	-0.159	0.24909	4	4	2	5
2170	Pyridoxine-5'-phosphate oxidase	IPI00018272	-0.159	0.24909	3	5	3	4

No.	Description	Accession number	STN[1]	p-Value[1]	Con_A[2]	Con_B[2]	SORA_A[2]	SORA_B[2]
2171	Guanine deaminase	IPI00644409	-0.159	0.24909	5	3	4	3
2172	G patch domain and KOW motifs-containing protein	IPI00024255	-0.159	0.24909	3	5	4	3
2173	DnaJ homolog subfamily C member 8	IPI00003438	-0.159	0.24909	5	3	3	4
2174	Succinate dehydrogenase assembly factor 2, mitochondrial	IPI00016443	-0.159	0.24909	3	5	3	4
2175	Poly [ADP-ribose] polymerase 1	IPI00449049	-0.159	0.24342	54	48	45	55
2176	Brefeldin A-inhibited guanine nucleotide-exchange protein 2	IPI00002186	-0.165	0.24331	3	4	2	4
2177	Keratin, type II cytoskeletal 5	IPI00009867	-0.165	0.24331	5	2	0	4
2178	DNA-directed RNA polymerase II subunit RPB3	IPI00018288	-0.165	0.24331	4	3	2	4
2179	Isoform C of Lethal(2) giant larvae protein homolog 2	IPI00465050	-0.165	0.24331	3	4	0	4
2180	Isoform 2C of Cytoplasmic dynein 1 intermediate chain 2	IPI00216348	-0.165	0.24331	4	3	4	2
2181	39S ribosomal protein L48, mitochondrial	IPI00295066	-0.165	0.24331	3	4	2	4
2182	Alpha-ketoglutarate dehydrogenase complex dihydrolipoyl succinyltransferase	IPI00033034	-0.165	0.24331	4	3	0	4
2183	Sphingosine-1-phosphate lyase 1	IPI00099463	-0.165	0.24331	4	3	4	2
2184	Putative myosin-XVB	IPI00786880	-0.165	0.24331	5	2	3	3
2185	Lamina-associated polypeptide 2, isoform alpha	IPI00216230	-0.165	0.24331	5	2	0	4
2186	Isoform 3 of Tyrosine-protein phosphatase non-receptor type 6	IPI00183046	-0.165	0.24331	3	4	4	2
2187	Coiled-coil domain-containing protein 6	IPI00000634	-0.165	0.24331	2	5	2	4
2188	Isoform 1 of Alpha-parvin	IPI00018963	-0.165	0.24331	3	4	3	3
2189	Isoform 1 of Translocon-associated protein subunit alpha	IPI00301021	-0.165	0.24331	5	0	0	4
2190	Xaa-Pro dipeptidase	IPI00257882	-0.165	0.24331	3	4	3	3
2191	UPF0600 protein C5orf51	IPI00374272	-0.165	0.24331	3	4	3	3
2192	39S ribosomal protein L50, mitochondrial	IPI00329036	-0.165	0.24331	2	5	3	3
2193	Isoform 3 of THO complex subunit 6 homolog	IPI00301252	-0.165	0.24331	4	3	3	3
2194	Isoform 1 of PDZ domain-containing protein 11	IPI00550841	-0.165	0.24331	3	4	4	2
2195	Peptidyl-prolyl cis-trans isomerase-like 4	IPI00642862	-0.165	0.24331	3	4	3	3
2196	Isoform 3 of Chitinase domain-containing protein 1	IPI00045536	-0.165	0.24331	5	2	2	4
2197	Protein FRG1	IPI00004655	-0.165	0.24331	3	4	2	4
2198	2-oxoisovalerate dehydrogenase subunit alpha, mitochondrial	IPI00025100	-0.165	0.24331	3	4	4	0
2199	Isoform 2 of Ubiquinol-cytochrome c reductase complex chaperone CBP3 homolog	IPI00219889	-0.165	0.24331	3	4	4	2
2200	N-alpha-acetyltransferase 20, NatB catalytic subunit	IPI00007174	-0.165	0.24331	3	4	3	3
2201	Protein S100-A11	IPI00013895	-0.165	0.24331	4	3	2	4
2202	Pterin-4-alpha-carbinolamine dehydratase	IPI00218568	-0.165	0.24331	3	4	3	3
2203	Uncharacterized protein C10orf58	IPI00296190	-0.165	0.24331	3	4	2	4
2204	Prohibitin	IPI00017334	-0.170	0.23893	32	48	33	45
2205	Putative uncharacterized protein CNOT1	IPI00032299	-0.173	0.23870	4	2	2	3
2206	39S ribosomal protein L15, mitochondrial	IPI00023086	-0.173	0.23870	3	3	3	2
2207	SPRY domain-containing protein 4	IPI00291643	-0.173	0.23870	4	2	0	3
2208	Isoform 1 of Acyl-CoA-binding protein	IPI00010182	-0.173	0.23870	3	3	3	0
2209	Ras-related protein Rap-2c	IPI00009607	-0.173	0.23870	4	2	2	3
2210	Cytokine-like nuclear factor n-pac, isoform CRA_a	IPI00000155	-0.173	0.23870	4	2	2	3
2211	RNA polymerase-associated protein CTR9 homolog	IPI00477468	-0.173	0.23870	3	3	2	3
2212	U3 small nucleolar RNA-associated protein 18 homolog	IPI00000733	-0.173	0.23870	2	4	0	3
2213	U3 small nucleolar RNA-associated protein 15 homolog	IPI00152708	-0.173	0.23870	2	4	3	2
2214	Retinol dehydrogenase 13	IPI00301204	-0.173	0.23870	3	3	3	2
2215	14 kDa phosphohistidine phosphatase	IPI00299977	-0.173	0.23870	3	3	2	3
2216	Mitochondrial ribosomal protein L21 isoform d	IPI00375677	-0.173	0.23870	3	3	0	3
2217	Isoform 1 of Nuclear-interacting partner of ALK	IPI00301421	-0.173	0.23870	2	4	0	3
2218	myosin regulatory light polypeptide 9 isoform b	IPI00030929	-0.173	0.23870	0	4	2	3
2219	Vacuolar fusion protein MON1 homolog B	IPI00848138	-0.173	0.23870	4	2	3	2
2220	Cell division protein kinase 7	IPI00000685	-0.173	0.23870	2	4	2	3
2221	Isoform 2 of Cytochrome P450 2S1	IPI00164018	-0.173	0.23870	0	4	0	3
2222	negative elongation factor A	IPI00394679	-0.173	0.23870	0	4	2	3
2223	cDNA FLJ60094, highly similar to F-actin capping protein subunit beta	IPI00218782	-0.173	0.23870	4	0	2	3
2224	Isoform 1 of Lysocardiolipin acyltransferase 1	IPI00419643	-0.173	0.23870	4	2	2	3
2225	Zinc finger protein ZPR1	IPI00025244	-0.173	0.23870	2	4	2	3
2226	UV excision repair protein RAD23 homolog A	IPI00008219	-0.173	0.23870	3	3	2	3
2227	Farnesyl pyrophosphate synthetase like-4 protein (Fragment)	IPI00382869	-0.173	0.23870	3	3	0	3
2228	YrdC domain-containing protein, mitochondrial	IPI00384180	-0.173	0.23870	0	4	3	0
2229	Dihydrofolate reductase	IPI00030357	-0.173	0.23870	4	2	3	2
2230	Isoform 3 of Transcription elongation factor SPT6	IPI00456683	-0.173	0.23870	3	3	0	3
2231	Thiopurine S-methyltransferase	IPI00019400	-0.173	0.23870	2	4	2	3
2232	OTU domain-containing protein 6B	IPI00182180	-0.173	0.23870	0	4	2	3
2233	Isoform 1 of HEAT repeat-containing protein 2	IPI00242630	-0.173	0.23870	3	3	2	3
2234	Isoform 1 of Hematological and neurological expressed 1-like protein	IPI00027397	-0.173	0.23870	3	3	2	3
2235	Isoform 1 of Protein kinase C and casein kinase substrate in neurons protein 2	IPI00027009	-0.173	0.23870	3	3	0	3
2236	Serine/threonine-protein kinase 10	IPI00304742	-0.173	0.23870	3	3	2	3
2237	Isoform 2 of U4/U6 small nuclear ribonucleoprotein Prp31	IPI00167198	-0.173	0.23870	3	3	2	3
2238	Isoform 1 of SAM domain and HD domain-containing protein 1	IPI00294739	-0.173	0.23870	3	3	3	2
2239	Isoform 1 of Peptidyl-prolyl cis-trans isomerase SDCCAG10	IPI00025174	-0.173	0.23870	2	4	2	3
2240	Alpha-taxilin	IPI00470779	-0.173	0.23870	3	3	0	3
2241	39S ribosomal protein L45, mitochondrial	IPI00185859	-0.173	0.23870	3	3	0	3
2242	cytochrome c oxidase subunit VIIa polypeptide 2 (liver) precursor	IPI00026570	-0.173	0.23870	3	3	3	0
2243	D-beta-hydroxybutyrate dehydrogenase, mitochondrial	IPI00025341	-0.173	0.23870	3	3	3	2
2244	Isoform 1 of ADP-ribosylation factor GTPase-activating protein 1	IPI00175169	-0.173	0.23870	3	3	2	3
2245	Isoform 3 of Tyrosine-protein kinase-like 7	IPI00168813	-0.173	0.23870	4	0	3	0
2246	V-type proton ATPase subunit d 1	IPI00034159	-0.173	0.23870	3	3	2	3
2247	Zinc finger CCHC-type and RNA-binding motif-containing protein 1	IPI00154614	-0.173	0.23870	3	3	0	3
2248	Isoform 1 of Beta-enolase	IPI00218474	-0.173	0.23870	4	2	3	2
2249	TBC1 domain family member 15 isoform 1	IPI00154645	-0.173	0.23870	3	3	3	2
2250	Ribonuclease UK114	IPI00005038	-0.173	0.23870	3	3	2	3
2251	Isoform 1 of RNA polymerase II-associated protein 3	IPI00002408	-0.173	0.23870	4	2	0	3
2252	Isoform Delta 6 of Calcium/calmodulin-dependent protein kinase type II subunit delta	IPI00172636	-0.173	0.23870	4	0	3	0
2253	Isoform 1 of Polyadenylate-binding protein 2	IPI00005792	-0.182	0.22146	3	2	0	2
2254	Isoform 1 of ER lumen protein retaining receptor 3	IPI00013872	-0.182	0.22146	0	3	0	2
2255	Mannose-P-dolichol utilization defect 1 protein	IPI00025292	-0.182	0.22146	2	3	2	0
2256	U2 small nuclear ribonucleoprotein B''	IPI00029267	-0.182	0.22146	2	3	0	0
2257	NADH-ubiquinone oxidoreductase chain 4	IPI00008495	-0.182	0.22146	3	0	0	0
2258	Solute carrier family 2, facilitated glucose transporter member 3	IPI00003909	-0.182	0.22146	3	0	0	0
2259	Isoform 3 of Rapamycin-insensitive companion of mTOR	IPI00166528	-0.182	0.22146	3	0	2	0
2260	Isoform 1 of Dual specificity mitogen-activated protein kinase kinase 3	IPI00218857	-0.182	0.22146	2	3	2	2
2261	cDNA FLJ56468, highly similar to Kynurenine--oxoglutarate transaminase 1	IPI00002523	-0.182	0.22146	0	3	0	0
2262	Isoform 1 of AP-2 complex subunit mu	IPI00022256	-0.182	0.22146	3	2	0	0
2263	Neurolysin, mitochondrial	IPI00010346	-0.182	0.22146	3	2	2	0
2264	Phosphoinositide 3-kinase regulatory subunit 4	IPI00024006	-0.182	0.22146	3	2	0	2
2265	Transmembrane emp24 domain-containing protein 9	IPI00023542	-0.182	0.22146	2	3	0	2

No.	Description	Accession number	STN[1]	p-Value[1]	Con_A[2]	Con_B[2]	SORA_A[2]	SORA_B[2]
2266	Isoform 2 of Integrator complex subunit 3	IPI00418336	-0.182	0.22146	0	3	0	2
2267	Acyl-CoA desaturase	IPI00299468	-0.182	0.22146	3	2	2	0
2268	Isoform 2 of DnaJ homolog subfamily A member 3, mitochondrial	IPI00179187	-0.182	0.22146	3	2	2	2
2269	Isoform 1 of RNA polymerase II-associated protein 1	IPI00402657	-0.182	0.22146	2	3	2	2
2270	Cathepsin B	IPI00295741	-0.182	0.22146	0	3	0	2
2271	Isoform 1 of Myotubularin-related protein 5	IPI00029446	-0.182	0.22146	3	2	0	0
2272	Isoform A of DnaJ homolog subfamily B member 6	IPI00024523	-0.182	0.22146	2	3	0	0
2273	DNA polymerase	IPI00744598	-0.182	0.22146	2	3	0	0
2274	Component of gems 4	IPI00027717	-0.182	0.22146	2	3	2	2
2275	Casein kinase II subunit beta	IPI00010865	-0.182	0.22146	3	0	2	0
2276	Isoform 2 of TIP41-like protein	IPI00641815	-0.182	0.22146	2	3	0	0
2277	Ubiquitin carboxyl-terminal hydrolase 10	IPI00291946	-0.182	0.22146	2	3	0	0
2278	Isoform 1 of Ras GTPase-activating protein 1	IPI00026262	-0.182	0.22146	3	2	2	2
2279	28S ribosomal protein S7, mitochondrial	IPI00006440	-0.182	0.22146	0	3	2	2
2280	RhoA activator C11orf59	IPI00016670	-0.182	0.22146	2	3	0	2
2281	B-cell receptor-associated protein 31	IPI00218200	-0.182	0.22146	0	3	2	2
2282	Isoform 1 of Solute carrier family 12 member 7	IPI00008616	-0.182	0.22146	0	3	0	0
2283	Vacuolar protein sorting-associated protein 26B	IPI00059264	-0.182	0.22146	2	3	0	0
2284	ATP-dependent RNA helicase DDX54 isoform 1	IPI00152510	-0.182	0.22146	3	0	0	2
2285	Isoform 1 of FAD synthase	IPI00220299	-0.182	0.22146	0	3	2	0
2286	Peflin	IPI00018235	-0.182	0.22146	3	2	0	2
2287	Mitotic spindle assembly checkpoint protein MAD2A	IPI00012369	-0.182	0.22146	2	3	2	2
2288	39S ribosomal protein L38, mitochondrial	IPI00783656	-0.182	0.22146	2	3	2	2
2289	39S ribosomal protein L40, mitochondrial	IPI00099871	-0.182	0.22146	3	0	2	2
2290	Isoform 1 of Uncharacterized protein KIAA0528	IPI00465142	-0.182	0.22146	3	2	2	0
2291	Vesicle-associated membrane protein 3	IPI00549343	-0.182	0.22146	3	2	0	2
2292	Isoform 2 of SWI/SNF-related matrix-associated actin-dependent regulator of chromatin subfamily A	IPI00008422	-0.182	0.22146	3	2	0	2
2293	NDUFB10 protein	IPI00074489	-0.182	0.22146	3	2	2	2
2294	28S ribosomal protein S16, mitochondrial	IPI00032872	-0.182	0.22146	3	2	2	2
2295	Ubiquitin-conjugating enzyme E2 G1	IPI00219783	-0.182	0.22146	3	2	2	2
2296	Isoform 2 of Nitrilase homolog 1	IPI00023779	-0.182	0.22146	0	3	0	0
2297	Vacuolar protein sorting-associated protein 4A	IPI00411356	-0.182	0.22146	2	3	0	0
2298	Isoform 2 of Arf-GAP with Rho-GAP domain, ANK repeat and PH domain-containing protein 1	IPI00220421	-0.182	0.22146	3	0	0	0
2299	Glioma tumor suppressor candidate region gene 2 protein	IPI00024567	-0.182	0.22146	2	3	2	2
2300	71 kDa protein	IPI00062599	-0.182	0.22146	2	3	0	0
2301	NADH dehydrogenase [ubiquinone] 1 alpha subcomplex subunit 6	IPI00419266	-0.182	0.22146	3	2	0	0
2302	Splicing factor 3B subunit 5	IPI00010404	-0.182	0.22146	0	3	2	2
2303	cDNA FLJ78567	IPI00043678	-0.182	0.22146	2	3	0	2
2304	Sterol O-acyltransferase 1	IPI00644020	-0.182	0.22146	3	0	0	0
2305	ADP-ribosylation factor GTPase-activating protein 2	IPI00297322	-0.182	0.22146	0	3	0	2
2306	U3 small nucleolar RNA-associated protein 6 homolog	IPI00020128	-0.182	0.22146	3	2	0	0
2307	Isoform 3 of Epithelial splicing regulatory protein 1	IPI00184262	-0.182	0.22146	0	3	2	2
2308	Aldo-keto reductase family 1 member C3	IPI00291483	-0.182	0.22146	3	2	0	0
2309	Beta-adrenergic receptor kinase 1	IPI00012497	-0.182	0.22146	2	3	0	0
2310	UPF0428 protein CXorf56	IPI00005055	-0.182	0.22146	3	2	0	2
2311	Putative uncharacterized protein	IPI00260769	-0.182	0.22146	3	2	2	0
2312	Isoform 1 of Regulator of microtubule dynamics protein 3	IPI00410079	-0.182	0.22146	3	0	0	0
2313	Isoform 1 of E3 ubiquitin-protein ligase BRE1B	IPI00162563	-0.182	0.22146	0	3	0	0
2314	Isoform 1 of Far upstream element-binding protein 3	IPI00377261	-0.182	0.22146	2	3	0	0
2315	Isoform 1 of Drebrin	IPI00003406	-0.182	0.22146	3	2	0	0
2316	NADH dehydrogenase [ubiquinone] 1 alpha subcomplex subunit 4	IPI00011770	-0.182	0.22146	3	0	0	0
2317	Mitochondrial fission 1 protein	IPI00007052	-0.182	0.22146	2	3	0	2
2318	Ras-related protein Rab-8B	IPI00024282	-0.182	0.22146	2	3	0	0
2319	Isoform 5 of Sigma non-opioid intracellular receptor 1	IPI00167206	-0.182	0.22146	3	0	2	2
2320	poly [ADP-ribose] polymerase 14	IPI00291215	-0.182	0.22146	3	0	0	2
2321	Pyridoxal phosphate phosphatase	IPI00025340	-0.182	0.22146	3	2	2	2
2322	39S ribosomal protein L11, mitochondrial	IPI00007001	-0.182	0.22146	3	2	0	0
2323	tRNA (guanine-N(7)-)-methyltransferase	IPI00290184	-0.182	0.22146	3	0	2	2
2324	LanC-like protein 2	IPI00032995	-0.182	0.22146	2	3	2	0
2325	Protein SGT1	IPI00027034	-0.182	0.22146	3	2	0	0
2326	Retinal rod rhodopsin-sensitive cGMP 3',5'-cyclic phosphodiesterase subunit delta	IPI00015161	-0.182	0.22146	2	3	2	2
2327	Protein FAM98A	IPI00174442	-0.182	0.22146	2	3	0	0
2328	Isoform 1 of Rhotekin	IPI00029834	-0.182	0.22146	3	2	0	0
2329	Ubiquitin-conjugating enzyme E2 R2	IPI00418603	-0.182	0.22146	3	0	0	2
2330	Pyruvate carboxylase, mitochondrial	IPI00299402	-0.182	0.22146	3	0	0	0
2331	Putative RNA-binding protein 16	IPI00829652	-0.182	0.22146	3	2	0	2
2332	Putative uncharacterized protein C3orf75	IPI00107155	-0.182	0.22146	3	0	0	0
2333	Probable ergosterol biosynthetic protein 28	IPI00007730	-0.182	0.22146	3	0	0	0
2334	Protein ETHE1, mitochondrial	IPI00003766	-0.182	0.22146	0	3	0	2
2335	Isoform 2 of Syntaxin-binding protein 1	IPI00046057	-0.182	0.22146	3	0	0	0
2336	Sulfhydryl oxidase 2	IPI00376394	-0.182	0.22146	3	2	0	0
2337	Dynactin subunit 4	IPI00550852	-0.182	0.22146	3	2	0	0
2338	Myosin-Ia	IPI00294386	-0.182	0.22146	3	2	2	2
2339	Isoform 5 of Protein transport protein Sec16A	IPI00031242	-0.182	0.22146	3	2	0	0
2340	Double-strand-break repair protein rad21 homolog	IPI00006715	-0.182	0.22146	3	2	0	0
2341	Isoform 1 of tRNA 2'-phosphotransferase 1	IPI00328580	-0.182	0.22146	3	0	0	0
2342	Isoform 2 of Oxidoreductase HTATIP2	IPI00383665	-0.182	0.22146	2	3	2	2
2343	Fumarylacetoacetase	IPI00031708	-0.182	0.22146	3	2	2	2
2344	Dynein, light chain, roadblock-type 1	IPI00412497	-0.182	0.22146	3	2	0	0
2345	COMM domain-containing protein 3	IPI00015773	-0.182	0.22146	2	3	2	2
2346	Isoform 1 of Phosphoribosyl pyrophosphate synthase-associated protein 1	IPI00291578	-0.182	0.22146	3	2	2	0
2347	GPN-loop GTPase 1 isoform a	IPI00027035	-0.182	0.22146	3	0	0	2
2348	Putative uncharacterized protein CASP2	IPI00018345	-0.182	0.22146	3	0	0	0
2349	Uncharacterized protein C19orf52	IPI00157215	-0.182	0.22146	2	3	2	0
2350	Interferon regulatory factor 3	IPI00291901	-0.182	0.22146	3	0	0	0
2351	Isoform 1 of 28S ribosomal protein S11, mitochondrial	IPI00010244	-0.182	0.22146	0	3	0	2
2352	Secernin-2	IPI00062266	-0.182	0.22146	2	3	2	0
2353	CCR4-NOT transcription complex subunit 8	IPI00295501	-0.182	0.22146	3	0	0	0
2354	Putative uncharacterized protein RBM12B	IPI00217626	-0.182	0.22146	3	0	0	0
2355	Isoform 1 of Pogo transposable element with ZNF domain	IPI00410717	-0.182	0.22146	3	2	0	0
2356	Isoform 1 of Putative deoxyribonuclease TATDN1	IPI00012463	-0.182	0.22146	3	0	0	2
2357	Isoform 2A of GTPase KRas	IPI00423568	-0.182	0.22146	0	3	0	0
2358	cDNA FLJ53160, highly similar to Zyxin	IPI00871311	-0.182	0.22146	2	3	2	0
2359	NADH dehydrogenase [ubiquinone] 1 subunit C2	IPI00029558	-0.182	0.22146	3	2	2	0

No.	Description	Accession number	STN[1]	p-Value[1]	Con_A[2]	Con_B[2]	SORA_A[2]	SORA_B[2]
2360	Probable histidyl-tRNA synthetase, mitochondrial	IPI00027445	-0.182	0.22146	3	2	0	2
2361	Isoform 1 of Transmembrane protein 85	IPI00009320	-0.182	0.22146	3	0	0	0
2362	Coiled-coil domain-containing protein 12	IPI00453463	-0.182	0.22146	0	3	0	2
2363	Complex I intermediate-associated protein 30, mitochondrial	IPI00032560	-0.182	0.22146	3	0	2	0
2364	Isoform 1 of Mammalian ependymin-related protein 1	IPI00259102	-0.182	0.22146	0	3	0	0
2365	Tyrosine-protein phosphatase non-receptor type 23	IPI00034006	-0.182	0.22146	2	3	0	2
2366	Enhancer of mRNA-decapping protein 3	IPI00018009	-0.182	0.22146	0	3	0	0
2367	Isoform 1 of Elongator complex protein 4	IPI00061376	-0.182	0.22146	3	0	0	0
2368	39S ribosomal protein L41, mitochondrial	IPI00217553	-0.182	0.22146	2	3	2	0
2369	cDNA FLJ56469, highly similar to Propionyl-CoA carboxylase alpha chain, mitochondrial	IPI00552419	-0.182	0.22146	3	0	0	2
2370	B-cell lymphoma/leukemia 10	IPI00022477	-0.182	0.22146	2	3	0	0
2371	Isoform 2 of WASH complex subunit FAM21C	IPI00456853	-0.182	0.22146	3	2	0	2
2372	Isoform 1 of Centromere protein M	IPI00031566	-0.182	0.22146	0	3	0	0
2373	Choline-phosphate cytidylyltransferase A	IPI00329338	-0.182	0.22146	3	0	0	0
2374	Creatine kinase B-type	IPI00022977	-0.182	0.22146	3	0	0	2
2375	Protein KTI12 homolog	IPI00061528	-0.182	0.22146	0	3	0	0
2376	Isoform 1 of Kynurenine--oxoglutarate transaminase 3	IPI00465373	-0.182	0.22146	3	2	0	0
2377	60 kDa protein	IPI00053288	-0.182	0.22146	0	3	2	2
2378	U4/U6.U5 small nuclear ribonucleoprotein 27 kDa protein	IPI00017289	-0.182	0.22146	2	3	2	2
2379	Succinate-semialdehyde dehydrogenase, mitochondrial	IPI00019888	-0.182	0.22146	3	2	0	0
2380	Y-box-binding protein 2	IPI00250153	-0.182	0.22146	3	0	2	0
2381	Syntaxin-6	IPI00013930	-0.182	0.22146	2	3	0	0
2382	Isoform 2 of Zinc finger CCCH domain-containing protein 18	IPI00293312	-0.182	0.22146	3	0	0	2
2383	28S ribosomal protein S18b, mitochondrial	IPI00022316	-0.182	0.22146	0	3	2	2
2384	Isoform 2 of Mitochondrial intermembrane space import and assembly protein 40	IPI00177428	-0.182	0.22146	3	2	0	0
2385	Isoform 2 of Ras-related protein Rab-4B	IPI00187143	-0.182	0.22146	2	3	0	2
2386	Isoform 1 of Vesicle-associated membrane protein 7	IPI00020887	-0.182	0.22146	0	3	0	2
2387	38 kDa protein	IPI00465230	-0.182	0.22146	3	0	0	0
2388	Isoform Long of Acidic fibroblast growth factor intracellular-binding protein	IPI00012443	-0.182	0.22146	0	3	0	2
2389	Isoform 1 of MYC-induced nuclear antigen	IPI00216737	-0.182	0.22146	3	2	0	0
2390	Isoform AGX2 of UDP-N-acetylhexosamine pyrophosphorylase	IPI00000684	-0.182	0.22146	0	3	2	0
2391	Acylphosphatase-1	IPI00221117	-0.182	0.22146	2	3	2	0
2392	Protein pelota homolog	IPI00106698	-0.182	0.22146	0	3	0	2
2393	Protein ariadne-2 homolog	IPI00007304	-0.182	0.22146	0	3	0	0
2394	Isoform 1 of Zinc finger protein 326	IPI00373877	-0.182	0.22146	3	0	0	0
2395	Protein unc-119 homolog B	IPI00414629	-0.182	0.22146	0	3	2	2
2396	Ubiquitin-conjugating enzyme E2 B	IPI00012060	-0.182	0.22146	3	0	0	0
2397	Isoform 1 of NADH-cytochrome b5 reductase 2	IPI00008234	-0.182	0.22146	0	3	0	2
2398	cDNA FLJ60939, highly similar to NAD-dependent deacetylase sirtuin-3, mitochondrial	IPI00183171	-0.182	0.22146	3	0	0	0
2399	Isoform 1 of Stromal membrane-associated protein 1	IPI00102096	-0.182	0.22146	3	0	0	0
2400	My002 protein	IPI00023584	-0.182	0.22146	3	2	0	0
2401	Isoform 2 of Mitochondrial ribonuclease P protein 3	IPI00004584	-0.182	0.22146	3	0	0	0
2402	Ubiquitin-conjugating enzyme E2 S	IPI00217949	-0.182	0.22146	3	0	0	0
2403	Isoform 3 of Protein DDI1 homolog 2	IPI00031618	-0.182	0.22146	3	0	0	0
2404	Isoform 3 of 5'-3' exoribonuclease 1	IPI00328115	-0.182	0.22146	3	0	0	0
2405	cDNA FLJ56439, highly similar to Pantothenate kinase 4	IPI00018946	-0.182	0.22146	3	0	0	0
2406	ADP-ribosylation factor 4	IPI00215918	-0.183	0.21095	25	36	30	29
2407	40S ribosomal protein S4, X isoform	IPI00217030	-0.186	0.21073	21	36	26	29
2408	26S protease regulatory subunit 6A	IPI00018398	-0.187	0.21073	29	27	25	29
2409	annexin A4	IPI00793199	-0.191	0.21020	24	28	27	23
2410	ATP-dependent RNA helicase DDX3X	IPI00215637	-0.192	0.21001	23	28	23	26
2411	ADP-sugar pyrophosphatase	IPI00296913	-0.200	0.20918	22	22	22	20
2412	Condensin complex subunit 1	IPI00299524	-0.203	0.20846	22	19	16	23
2413	Ras GTPase-activating protein-binding protein 1	IPI00012442	-0.208	0.20793	17	21	17	19
2414	Peptidyl-prolyl cis-trans isomerase FKBP3	IPI00024157	-0.209	0.20751	16	21	14	21
2415	Ezrin	IPI00843975	-0.216	0.20570	15	18	14	17
2416	Tubulin--tyrosine ligase-like protein 12	IPI00029048	-0.216	0.20570	16	17	13	18
2417	Sodium/potassium-transporting ATPase subunit beta-3	IPI00008167	-0.218	0.20559	17	15	13	17
2418	Nestin	IPI00010800	-0.220	0.20460	16	15	12	17
2419	Isoform Long of Eukaryotic translation initiation factor 4H	IPI00014263	-0.228	0.20287	11	16	9	16
2420	Isoform 2 of Protein disulfide-isomerase A6	IPI00299571	-0.231	0.20287	13	13	11	13
2421	Kinesin-1 heavy chain	IPI00012837	-0.233	0.20090	11	14	11	12
2422	Coronin-1B	IPI00007058	-0.236	0.20090	14	10	12	10
2423	Isoform 1 of Heterogeneous nuclear ribonucleoprotein H3	IPI00013877	-0.239	0.19946	10	13	8	13
2424	Isoform 1 of Poly(U)-binding-splicing factor PUF60	IPI00069750	-0.242	0.19931	12	10	9	11
2425	Glucosamine 6-phosphate N-acetyltransferase	IPI00061525	-0.242	0.19931	8	14	9	11
2426	Single-stranded DNA-binding protein, mitochondrial	IPI00029744	-0.242	0.19931	11	11	7	13
2427	cDNA FLJ51909, highly similar to Serine-threonine kinase receptor-associatedprotein	IPI00294536	-0.242	0.19931	13	9	8	12
2428	Rho-associated protein kinase 2	IPI00307155	-0.245	0.19704	11	10	9	10
2429	V-type proton ATPase subunit E 1	IPI00003856	-0.245	0.19704	11	10	8	11
2430	Thyroid hormone receptor-associated protein 3	IPI00104050	-0.248	0.19704	10	10	6	12
2431	Sideroflexin-1	IPI00009368	-0.248	0.19704	10	10	7	11
2432	Isoform 1 of Mitotic checkpoint protein BUB3	IPI00013468	-0.248	0.19704	10	10	8	10
2433	Galectin-3	IPI00465431	-0.252	0.19470	9	10	9	8
2434	Histone-binding protein RBBP7	IPI00395865	-0.252	0.19470	11	8	8	9
2435	DNA polymerase delta catalytic subunit	IPI00002894	-0.252	0.19470	9	10	8	9
2436	SAP domain-containing ribonucleoprotein	IPI00014938	-0.252	0.19470	8	11	9	8
2437	WD repeat-containing protein 61	IPI00019269	-0.256	0.19459	7	11	8	8
2438	Isocitrate dehydrogenase [NADP] cytoplasmic	IPI00027223	-0.256	0.19459	8	10	10	6
2439	NADH-ubiquinone oxidoreductase 75 kDa subunit	IPI00604664	-0.260	0.19092	8	9	8	7
2440	Alkyldihydroxyacetonephosphate synthase, peroxisomal	IPI00010349	-0.260	0.19092	8	9	6	9
2441	Isoform 1 of Replication factor C subunit 2	IPI00017412	-0.260	0.19092	8	9	6	9
2442	Nucleoporin 85	IPI00171542	-0.260	0.19092	10	7	7	8
2443	Signal recognition particle 14 kDa protein	IPI00293434	-0.260	0.19092	7	10	6	9
2444	Nuclear cap-binding protein subunit 1	IPI00019380	-0.264	0.19092	9	7	4	10
2445	Exportin-5	IPI00640703	-0.264	0.19092	7	9	5	9
2446	Ornithine aminotransferase, mitochondrial	IPI00022334	-0.264	0.19092	7	9	7	7
2447	Isocitrate dehydrogenase [NADP], mitochondrial	IPI00011107	-0.264	0.19092	7	9	9	5
2448	S-formylglutathione hydrolase	IPI00411706	-0.269	0.18616	7	8	7	6
2449	39S ribosomal protein L19, mitochondrial	IPI00027096	-0.269	0.18616	7	8	6	7
2450	60S ribosomal protein L36	IPI00216237	-0.269	0.18616	9	6	7	6
2451	Isoform 1 of Polyadenylate-binding protein 1	IPI00008524	-0.272	0.18616	34	29	30	30
2452	Isoform 2 of Nipped-B-like protein	IPI00026466	-0.274	0.18601	11	3	8	4
2453	Exportin-7	IPI00302458	-0.274	0.18601	8	6	7	5
2454	39S ribosomal protein L13, mitochondrial	IPI00022403	-0.274	0.18601	6	8	4	8

No.	Description	Accession number	STN[1]	p-Value[1]	Con_A[2]	Con_B[2]	SORA_A[2]	SORA_B[2]
2455	Isoform 2 of Bromodomain adjacent to zinc finger domain protein 1A	IPI00383565	-0.274	0.18601	8	6	6	6
2456	UPF0468 protein C16orf80	IPI00001655	-0.274	0.18601	6	8	3	9
2457	Isoform 2 of N-alpha-acetyltransferase 15, NatA auxiliary subunit	IPI00032158	-0.274	0.18601	8	6	5	7
2458	Paired amphipathic helix protein Sin3a	IPI00170596	-0.274	0.18601	9	5	6	6
2459	Isoform 2 of Guanine nucleotide-binding protein G(i) subunit alpha-2	IPI00217906	-0.274	0.18601	8	6	8	4
2460	Transmembrane protein 165	IPI00307572	-0.274	0.18601	6	8	6	6
2461	Coactosin-like protein	IPI00017704	-0.280	0.17962	4	9	3	8
2462	Spermidine synthase	IPI00292020	-0.280	0.17962	5	8	6	5
2463	Isoform 1 of Uridine-cytidine kinase 2	IPI00065671	-0.280	0.17962	8	5	7	4
2464	Tetratricopeptide repeat protein 37	IPI00005634	-0.280	0.17962	6	7	4	7
2465	Emerin	IPI00032003	-0.280	0.17962	6	7	6	5
2466	Phosphatidylinositol transfer protein alpha isoform	IPI00216048	-0.280	0.17962	6	7	5	6
2467	Dolichyl-diphosphooligosaccharide--protein glycosyltransferase subunit DAD1	IPI00009407	-0.280	0.17962	5	8	6	5
2468	Diablo homolog, mitochondrial precursor	IPI00008418	-0.280	0.17962	5	8	5	6
2469	Peptidyl-prolyl cis-trans isomerase H	IPI00007346	-0.280	0.17962	6	7	6	5
2470	dynactin subunit 2	IPI00220503	-0.280	0.17962	7	6	7	4
2471	28 kDa heat- and acid-stable phosphoprotein	IPI00013297	-0.280	0.17962	7	6	5	6
2472	Rab GDP dissociation inhibitor alpha	IPI00010154	-0.284	0.17951	28	26	23	28
2473	Small subunit processome component 20 homolog	IPI00004970	-0.285	0.17951	26	27	20	30
2474	Galectin-3-binding protein	IPI00023673	-0.287	0.17951	4	8	5	5
2475	Basic leucine zipper and W2 domain-containing protein 2	IPI00022305	-0.287	0.17951	6	6	6	4
2476	cDNA FLJ59367, highly similar to Adenylosuccinate lyase	IPI00026904	-0.287	0.17951	7	5	3	7
2477	Uncharacterized protein C19orf21	IPI00217121	-0.287	0.17951	6	6	7	3
2478	Isoform 1 of Polyadenylate-binding protein 4	IPI00012726	-0.287	0.17951	7	5	5	5
2479	UPF0553 protein C9orf64	IPI00170972	-0.287	0.17951	7	5	5	5
2480	TDP43	IPI00025815	-0.287	0.17951	5	7	4	6
2481	Cold-inducible RNA-binding protein	IPI00180954	-0.287	0.17951	5	7	6	4
2482	Isoform 2 of Ubiquilin-1	IPI00071180	-0.287	0.17951	7	5	4	6
2483	Eukaryotic translation initiation factor 5B	IPI00299254	-0.290	0.17153	24	26	21	26
2484	cDNA FLJ56285, highly similar to ADP-ribosylation factor-like protein 8B	IPI00018871	-0.294	0.17096	5	6	5	4
2485	Isoform 1 of Growth factor receptor-bound protein 2	IPI00021327	-0.294	0.17096	8	3	3	6
2486	DYNC1H1 protein	IPI00440177	-0.294	0.17096	7	4	6	3
2487	UPF0556 protein C19orf10	IPI00056357	-0.294	0.17096	5	6	2	7
2488	Isoform 1 of Mps one binder kinase activator-like 1B	IPI00301518	-0.294	0.17096	5	6	3	6
2489	Leucine-rich repeat-containing protein 47	IPI00170935	-0.294	0.17096	6	5	3	6
2490	Non-functional aryl hydrocarbon receptor interacting protein (Fragment)	IPI00925804	-0.294	0.17096	7	4	4	5
2491	26S proteasome non-ATPase regulatory subunit 10	IPI00003565	-0.294	0.17096	5	6	4	5
2492	Phosducin-like protein 3	IPI00031629	-0.294	0.17096	6	5	5	4
2493	Isoform 2 of Peptidyl-prolyl cis-trans isomerase NIMA-interacting 4	IPI00006658	-0.294	0.17096	5	6	7	0
2494	Isoform p27-L of 26S proteasome non-ATPase regulatory subunit 9	IPI00010860	-0.294	0.17096	6	5	4	5
2495	Threonyl-tRNA synthetase, cytoplasmic	IPI00329633	-0.300	0.17074	19	25	20	21
2496	ATP-dependent RNA helicase DHX29	IPI00217413	-0.303	0.17040	4	6	3	5
2497	WD40 repeat-containing protein SMU1	IPI00305833	-0.303	0.17040	5	5	0	6
2498	Eukaryotic peptide chain release factor GTP-binding subunit ERF3A	IPI00218829	-0.303	0.17040	6	4	4	4
2499	AP-1 complex subunit mu-1	IPI00032516	-0.303	0.17040	3	7	3	5
2500	Isoform 2 of Ubiquitin carboxyl-terminal hydrolase isozyme L5	IPI00219512	-0.303	0.17040	2	8	4	4
2501	ATP-binding cassette sub-family D member 1	IPI00291373	-0.303	0.17040	7	3	4	4
2502	Peroxisomal multifunctional enzyme type 2	IPI00019912	-0.303	0.17040	5	5	6	0
2503	U4/U6.U5 tri-snRNP-associated protein 1	IPI00021417	-0.303	0.17040	4	6	4	4
2504	Isoform 1 of U4/U6 small nuclear ribonucleoprotein Prp4	IPI00150269	-0.303	0.17040	5	5	4	4
2505	Isoform 1 of tRNA (adenine-N(1)-)-methyltransferase non-catalytic subunit TRM6	IPI00099311	-0.303	0.17040	5	5	3	5
2506	Heat shock 70 kDa protein 14	IPI00292499	-0.303	0.17040	5	5	4	4
2507	Isoform 2 of PERQ amino acid-rich with GYF domain-containing protein 2	IPI00647635	-0.303	0.17040	6	4	3	5
2508	MACRO domain-containing protein 1	IPI00155601	-0.303	0.17040	5	5	4	4
2509	Dual specificity protein phosphatase 3	IPI00018671	-0.303	0.17040	5	5	3	5
2510	Isoform Long of Spectrin beta chain, brain 1	IPI00005614	-0.305	0.16038	55	66	52	65
2511	TC4 protein	IPI00044779	-0.308	0.16019	14	26	15	22
2512	UDP-glucose 6-dehydrogenase	IPI00031420	-0.311	0.15996	16	23	18	18
2513	Isoform 1 of PC4 and SFRS1-interacting protein	IPI00028122	-0.313	0.15989	4	5	0	5
2514	Isoform 2 of Myosin-XVIIIa	IPI00334410	-0.313	0.15989	6	3	4	3
2515	Isoform Long of Cold shock domain-containing protein E1	IPI00470891	-0.313	0.15989	5	4	2	5
2516	60S acidic ribosomal protein P1	IPI00008527	-0.313	0.15989	2	7	0	5
2517	Cyclin-G-associated kinase	IPI00298949	-0.313	0.15989	7	2	3	4
2518	Mitochondrial import inner membrane translocase subunit Tim23	IPI00007309	-0.313	0.15989	4	5	4	3
2519	Isoform D of Constitutive coactivator of PPAR-gamma-like protein 1	IPI00039626	-0.313	0.15989	3	6	0	5
2520	Heat shock 70 kDa protein 4L	IPI00295485	-0.313	0.15989	4	5	0	5
2521	Translocation protein SEC63 homolog	IPI00218922	-0.313	0.15989	5	4	5	2
2522	Isoform 1 of Partner of Y14 and mago	IPI00305092	-0.313	0.15989	4	5	3	4
2523	Isoform 1 of Sorting nexin-12	IPI00438170	-0.313	0.15989	4	5	3	4
2524	Nuclear pore complex protein Nup153	IPI00292059	-0.313	0.15989	3	6	2	5
2525	REST corepressor 1	IPI00008531	-0.313	0.15989	4	5	4	3
2526	Tyrosine-protein kinase CSK	IPI00013212	-0.313	0.15989	4	5	5	2
2527	Thioredoxin-like protein 4B	IPI00016481	-0.313	0.15989	4	5	0	5
2528	Nitric oxide synthase-interacting protein	IPI00006408	-0.313	0.15989	4	5	4	3
2529	Isoform 1 of Melanoma-associated antigen D2	IPI00009542	-0.313	0.15989	4	5	3	4
2530	Trafficking protein particle complex subunit 3	IPI00004324	-0.313	0.15989	5	4	3	4
2531	Armadillo repeat-containing X-linked protein 3	IPI00009906	-0.313	0.15989	5	4	3	4
2532	Platelet-activating factor acetylhydrolase IB subunit gamma	IPI00014808	-0.324	0.15849	5	3	4	2
2533	Inosine triphosphate pyrophosphatase	IPI00018783	-0.324	0.15849	5	3	0	4
2534	PDZ and LIM domain protein 5	IPI00007935	-0.324	0.15849	5	3	4	2
2535	Serine/threonine-protein phosphatase 2A 56 kDa regulatory subunit epsilon isoform	IPI00002853	-0.324	0.15849	4	4	0	4
2536	Microsomal glutathione S-transferase 1	IPI00021805	-0.324	0.15849	3	5	0	4
2537	Mimitin, mitochondrial	IPI00031109	-0.324	0.15849	4	4	0	4
2538	Isoform 1 of Rho GTPase-activating protein 18	IPI00296353	-0.324	0.15849	2	6	0	4
2539	Isoform 1 of Aldehyde dehydrogenase family 16 member A1	IPI00217920	-0.324	0.15849	2	6	3	3
2540	Isoform 1 of U3 small nucleolar RNA-associated protein 14 homolog A	IPI00107113	-0.324	0.15849	4	4	2	4
2541	Isoform 1 of Methyl-CpG-binding domain protein 3	IPI00439194	-0.324	0.15849	4	4	3	3
2542	COMM domain-containing protein 2	IPI00456048	-0.324	0.15849	4	4	2	4
2543	Charged multivesicular body protein 7	IPI00395463	-0.324	0.15849	4	4	3	3
2544	Isoform Short of Ubiquitin fusion degradation protein 1 homolog	IPI00218292	-0.324	0.15849	5	3	2	4
2545	BRI3-binding protein	IPI00103599	-0.324	0.15849	6	0	4	0
2546	Deoxyhypusine hydroxylase	IPI00171856	-0.324	0.15849	4	4	2	4
2547	Ethanolamine kinase 1	IPI00030090	-0.324	0.15849	3	5	2	4
2548	Isoform 2 of Beta-catenin-like protein 1	IPI00472981	-0.324	0.15849	4	4	2	4
2549	V-type proton ATPase subunit F	IPI00004488	-0.324	0.15849	3	5	4	2

No.	Description	Accession number	STN[1]	p-Value[1]	Con_A[2]	Con_B[2]	SORA_A[2]	SORA_B[2]
2550	Uncharacterized protein C2orf47, mitochondrial	IPI00291751	-0.324	0.15849	5	3	0	4
2551	Isoform 1 of Creatine kinase U-type, mitochondrial	IPI00658109	-0.324	0.15849	4	4	4	2
2552	Isoform 1 of Craniofacial development protein 1	IPI00007306	-0.324	0.15849	4	4	2	4
2553	Ribonuclease H2 subunit A	IPI00290192	-0.324	0.15849	5	3	3	3
2554	cDNA FLJ56221, highly similar to YTH domain protein 3	IPI00396131	-0.324	0.15849	4	4	3	3
2555	Histidyl-tRNA synthetase, cytoplasmic	IPI00021808	-0.331	0.14299	18	13	14	14
2556	Isoform 1 of Coatomer subunit alpha	IPI00295857	-0.333	0.14261	50	37	37	46
2557	Peptidyl-prolyl cis-trans isomerase FKBP5	IPI00218775	-0.338	0.14231	3	4	3	0
2558	28S ribosomal protein S25, mitochondrial	IPI00013167	-0.338	0.14231	0	5	2	3
2559	TATA-binding protein-associated factor 172	IPI00024802	-0.338	0.14231	2	5	0	3
2560	Pumilio domain-containing protein KIAA0020	IPI00791325	-0.338	0.14231	4	3	0	3
2561	13kDa differentiation-associated protein variant (Fragment)	IPI00005966	-0.338	0.14231	3	4	3	2
2562	Syntaxin-binding protein 3	IPI00297626	-0.338	0.14231	5	0	2	3
2563	Isoform 1 of Protein timeless homolog	IPI00335541	-0.338	0.14231	2	5	3	0
2564	Isoform 3 of Sorting nexin-3	IPI00029740	-0.338	0.14231	4	3	2	3
2565	Small nuclear ribonucleoprotein E	IPI00029266	-0.338	0.14231	4	3	2	3
2566	Isoform 2 of Transcription elongation factor SPT6	IPI00430770	-0.338	0.14231	3	4	0	3
2567	Similar to Zinc finger CCCH domain-containing protein 15	IPI00000279	-0.338	0.14231	3	4	3	2
2568	[Pyruvate dehydrogenase [lipoamide]] kinase isozyme 3, mitochondrial	IPI00014849	-0.338	0.14231	3	4	0	3
2569	Isoform 1 of Ribonuclease H2 subunit C	IPI00382985	-0.338	0.14231	2	5	2	3
2570	Isoform Short of TATA-binding protein-associated factor 2N	IPI00020194	-0.338	0.14231	3	4	3	2
2571	Immunoglobulin-binding protein 1	IPI00019148	-0.338	0.14231	4	3	2	3
2572	Isoform 1 of Luc7-like protein 3	IPI00107745	-0.338	0.14231	4	3	3	2
2573	Glutamate--cysteine ligase regulatory subunit	IPI00010090	-0.338	0.14231	4	3	3	2
2574	General transcription factor IIF subunit 1	IPI00017450	-0.338	0.14231	3	4	3	2
2575	NEDD8-activating enzyme E1 catalytic subunit	IPI00328154	-0.338	0.14231	4	3	2	3
2576	Mitochondrial import receptor subunit TOM20 homolog	IPI00016676	-0.338	0.14231	0	5	0	3
2577	ATP-binding cassette sub-family E member 1	IPI00303207	-0.340	0.14231	41	40	35	42
2578	Importin subunit alpha-2	IPI00002214	-0.344	0.14133	12	15	12	12
2579	cytochrome b5 type B precursor	IPI00303954	-0.344	0.14133	12	15	14	10
2580	probable ubiquitin carboxyl-terminal hydrolase FAF-X isoform 4	IPI00003964	-0.352	0.14042	13	12	10	12
2581	FACT complex subunit SSRP1	IPI00005154	-0.352	0.14042	15	10	10	12
2582	Isoform 3 of Obg-like ATPase 1	IPI00216106	-0.352	0.14042	10	15	12	10
2583	Calcium-binding mitochondrial carrier protein Aralar2	IPI00007084	-0.352	0.14042	12	13	11	11
2584	Activating signal cointegrator 1 complex subunit 3	IPI00430472	-0.355	0.13989	0	4	2	0
2585	Growth hormone inducible transmembrane protein	IPI00549970	-0.355	0.13989	4	0	0	0
2586	cDNA FLJ12779 fis, clone NT2RP2001748	IPI00902799	-0.355	0.13989	3	3	2	2
2587	Isoform 1 of Protein furry homolog-like	IPI00739940	-0.355	0.13989	2	4	0	0
2588	Protein dpy-30 homolog	IPI00028109	-0.355	0.13989	2	4	0	0
2589	Isoform 1 of Cleft lip and palate transmembrane protein 1-like protein	IPI00151358	-0.355	0.13989	0	4	2	0
2590	24 kDa protein	IPI00397611	-0.355	0.13989	2	4	2	0
2591	Isoform 2 of Mediator of DNA damage checkpoint protein 1	IPI00470805	-0.355	0.13989	4	2	0	0
2592	Isoform 1 of Multidrug resistance-associated protein 4	IPI00006675	-0.355	0.13989	4	0	0	0
2593	Probable methyltransferase TARBP1	IPI00298447	-0.355	0.13989	4	2	0	0
2594	60S ribosomal protein L8	IPI00012772	-0.355	0.13989	0	4	0	0
2595	Myeloid-associated differentiation marker	IPI00102685	-0.355	0.13989	4	0	0	0
2596	RNA-binding protein PNO1	IPI00024524	-0.355	0.13989	3	3	2	2
2597	Isoform 1 of CDP-diacylglycerol--inositol 3-phosphatidyltransferase	IPI00645518	-0.355	0.13989	4	2	2	2
2598	Putative uncharacterized protein EIF4E2	IPI00556081	-0.355	0.13989	0	4	0	0
2599	Huntingtin	IPI00002335	-0.355	0.13989	2	4	0	0
2600	Isoform 1 of Fermitin family homolog 2	IPI00000856	-0.355	0.13989	4	2	2	0
2601	Isoform 1 of 39S ribosomal protein L47, mitochondrial	IPI00030820	-0.355	0.13989	3	3	2	0
2602	Branched-chain-amino-acid aminotransferase	IPI00181135	-0.355	0.13989	0	4	2	2
2603	Isoform B of AP-2 complex subunit alpha-1	IPI00256684	-0.355	0.13989	3	3	0	0
2604	Isoform 1 of CD109 antigen	IPI00152540	-0.355	0.13989	3	3	0	2
2605	UPF0760 protein C2orf29	IPI00014194	-0.355	0.13989	3	3	0	0
2606	Isoform 1 of Phosphatidate cytidylyltransferase 2	IPI00032150	-0.355	0.13989	4	2	0	2
2607	cDNA FLJ10824 fis, clone NT2RP4001086 (Fragment)	IPI00294810	-0.355	0.13989	3	3	2	2
2608	Vacuolar protein sorting-associated protein 33A	IPI00073179	-0.355	0.13989	3	3	0	0
2609	Isoform 1 of Clathrin coat assembly protein AP180	IPI00006612	-0.355	0.13989	2	4	2	2
2610	Isoform 3 of Cytosolic 5'-nucleotidase 3	IPI00100192	-0.355	0.13989	4	0	0	2
2611	DNA excision repair protein ERCC-6-like	IPI00552569	-0.355	0.13989	4	2	0	0
2612	Exosome complex exonuclease RRP42	IPI00014198	-0.355	0.13989	2	4	0	0
2613	N-alpha-acetyltransferase 38, NatC auxiliary subunit	IPI00219871	-0.355	0.13989	3	3	2	2
2614	Isoform 2 of Oxidation resistance protein 1	IPI00298348	-0.355	0.13989	3	3	0	0
2615	WD repeat-containing protein 82	IPI00152695	-0.355	0.13989	4	2	2	2
2616	Dihydroxyacetone phosphate acyltransferase	IPI00005677	-0.355	0.13989	3	3	0	0
2617	Nucleoside-triphosphatase C1orf57	IPI00031570	-0.355	0.13989	3	3	0	0
2618	Fatty acyl-CoA reductase 1	IPI00478838	-0.355	0.13989	0	4	0	0
2619	Isoform 2 of Ubiquitin-associated protein 2-like	IPI00029019	-0.355	0.13989	4	2	2	2
2620	Flotillin-1	IPI00027438	-0.355	0.13989	3	3	0	2
2621	Isoform 2 of mRNA cap guanine-N7 methyltransferase	IPI00410657	-0.355	0.13989	3	3	0	0
2622	Aldo-keto reductase family 1 member C2	IPI00005668	-0.355	0.13989	4	2	0	2
2623	Neudesin	IPI00002525	-0.355	0.13989	3	3	0	2
2624	Putative HLA class I histocompatibility antigen, alpha chain H	IPI00004672	-0.355	0.13989	4	0	0	2
2625	NADH dehydrogenase [ubiquinone] iron-sulfur protein 4, mitochondrial	IPI00011217	-0.355	0.13989	0	4	0	2
2626	U3 small nucleolar ribonucleoprotein protein MPP10	IPI00012149	-0.355	0.13989	4	2	0	0
2627	Calmodulin-regulated spectrin-associated protein 3	IPI00176702	-0.355	0.13989	4	2	0	2
2628	Isoform 2 of Phosphoenolpyruvate carboxykinase [GTP], mitochondrial	IPI00384116	-0.355	0.13989	3	3	2	2
2629	Putative uncharacterized protein DCP1A	IPI00164672	-0.355	0.13989	2	4	0	2
2630	Hydroxymethylglutaryl-CoA synthase, cytoplasmic	IPI00008475	-0.355	0.13989	3	3	0	0
2631	Isoform 2 of NAD-dependent deacetylase sirtuin-5	IPI00010331	-0.355	0.13989	0	4	2	2
2632	Isoform 1 of Transmembrane protein 87A	IPI00783698	-0.355	0.13989	0	4	0	0
2633	Isoform 2 of DNA-3-methyladenine glycosylase	IPI00218495	-0.355	0.13989	3	3	2	0
2634	cDNA FLJ43556 fis, clone PROST2018511, highly similar to Growth factor receptor-bound protein 7	IPI00448767	-0.355	0.13989	3	3	0	0
2635	Isoform 2 of Basic leucine zipper and W2 domain-containing protein 1	IPI00180128	-0.355	0.13989	4	2	2	2
2636	Adenylate kinase isoenzyme 6	IPI00032879	-0.355	0.13989	4	2	0	2
2637	Isoform 1 of Citron Rho-interacting kinase	IPI00022465	-0.355	0.13989	2	4	0	0
2638	Isoform 2 of 1-acyl-sn-glycerol-3-phosphate acyltransferase beta	IPI00221372	-0.355	0.13989	3	3	2	0
2639	Probable U3 small nucleolar RNA-associated protein 11	IPI00180454	-0.355	0.13989	0	4	0	0
2640	Uncharacterized protein C6orf130	IPI00184871	-0.355	0.13989	3	3	0	0
2641	ubiquitin-like with PHD and ring finger domains 1 isoform 2	IPI00797279	-0.355	0.13989	4	0	0	0
2642	60S ribosomal protein L35	IPI00412607	-0.355	0.13989	4	0	0	0
2643	Cysteine and glycine-rich protein 2	IPI00002824	-0.355	0.13989	4	2	0	0

No.	Description	Accession number	STN[1]	p-Value[1]	Con_A[2]	Con_B[2]	SORA_A[2]	SORA_B[2]
2644	C-terminal-binding protein 1	IPI00012835	-0.355	0.13989	4	0	0	0
2645	Serine/threonine-protein phosphatase 4 catalytic subunit	IPI00012833	-0.355	0.13989	4	0	0	0
2646	ADP-ribosylation factor 5	IPI00215919	-0.355	0.13989	0	4	0	0
2647	Phosphopantothenate--cysteine ligase	IPI00023987	-0.355	0.13989	3	3	2	2
2648	Putative uncharacterized protein PYCR2	IPI00335061	-0.355	0.13989	2	4	2	2
2649	Isoform 1 of Golgin subfamily A member 3	IPI00305267	-0.355	0.13989	3	3	0	0
2650	Maleylacetoacetate isomerase	IPI00013809	-0.355	0.13989	3	3	0	2
2651	DDB1- and CUL4-associated factor 7	IPI00006754	-0.355	0.13989	3	3	2	0
2652	Dehydrogenase/reductase SDR family member 7B	IPI00550165	-0.355	0.13989	3	3	0	2
2653	Isoform 3 of DnaJ homolog subfamily C member 11	IPI00333016	-0.355	0.13989	3	3	0	0
2654	Neighbor of COX4	IPI00005740	-0.355	0.13989	2	4	2	2
2655	Isoform 1 of Crooked neck-like protein 1	IPI00177437	-0.355	0.13989	2	4	0	0
2656	Sperm-associated antigen 7	IPI00006863	-0.355	0.13989	4	0	2	2
2657	Maspardin	IPI00010248	-0.355	0.13989	3	3	0	0
2658	Isoform 1 of Mannose-6-phosphate isomerase	IPI00219358	-0.355	0.13989	0	4	2	0
2659	Isoform 1 of SET domain-containing protein 3	IPI00165026	-0.355	0.13989	3	3	2	2
2660	Lanosterol synthase	IPI00009747	-0.355	0.13989	3	3	0	2
2661	114 kDa protein	IPI00166555	-0.355	0.13989	4	2	2	2
2662	Isoform 1 of Set1/Ash2 histone methyltransferase complex subunit ASH2	IPI00328658	-0.355	0.13989	3	3	2	2
2663	Transcriptional enhancer factor TEF-1	IPI00002901	-0.355	0.13989	3	3	2	2
2664	Isoform 1 of Nucleoporin p58/p45	IPI00107122	-0.355	0.13989	3	3	0	0
2665	arylacetamide deacetylase-like 1 isoform b	IPI00002230	-0.360	0.11835	11	12	7	13
2666	proteasome 26S non-ATPase subunit 8	IPI00010201	-0.360	0.11835	12	11	8	12
2667	Isochorismatase domain-containing protein 1	IPI00304082	-0.360	0.11835	10	13	9	11
2668	Isoform 1 of RNA-binding protein 39	IPI00163505	-0.360	0.11835	11	12	10	10
2669	Leucine-rich repeat-containing protein 59	IPI00396321	-0.365	0.11755	11	11	7	12
2670	Sorbitol dehydrogenase	IPI00216057	-0.365	0.11755	10	12	9	10
2671	Importin-7	IPI00007402	-0.365	0.11699	33	29	27	31
2672	poly(rC) binding protein 2 isoform b	IPI00012066	-0.365	0.11699	31	31	31	27
2673	Dual specificity mitogen-activated protein kinase kinase 2	IPI00003783	-0.370	0.11684	9	12	8	10
2674	26S protease regulatory subunit 7	IPI00021435	-0.370	0.11684	12	9	10	8
2675	Isoform 2 of SWI/SNF complex subunit SMARCC2	IPI00150057	-0.370	0.11684	11	10	8	10
2676	Asparaginyl-tRNA synthetase, cytoplasmic	IPI00306960	-0.370	0.11684	9	12	8	10
2677	Wolframin	IPI00008711	-0.375	0.11608	8	12	9	8
2678	CSNK2A1 protein	IPI00016613	-0.375	0.11608	11	9	10	7
2679	Insulin-degrading enzyme	IPI00220373	-0.375	0.11608	10	10	6	11
2680	Methylenetetrahydrofolate dehydrogenase (NADP+ dependent) 1-like	IPI00291646	-0.375	0.11608	10	10	6	11
2681	Isoform 1 of Vesicle-associated membrane protein-associated protein A	IPI00170692	-0.375	0.11608	10	10	7	10
2682	Ubiquitin carboxyl-terminal hydrolase isozyme L3	IPI00011250	-0.375	0.11608	11	9	8	9
2683	Isoform A1 of Tight junction protein ZO-2	IPI00003843	-0.380	0.11449	8	11	7	9
2684	Protein FAM49B	IPI00303318	-0.380	0.11449	9	10	8	8
2685	Proteasome subunit beta type-5	IPI00479306	-0.380	0.11449	8	11	9	7
2686	Signal peptidase complex catalytic subunit SEC11A	IPI00104128	-0.380	0.11449	9	10	8	8
2687	Isoform 1 of 60S ribosomal protein L12	IPI00024933	-0.384	0.11408	18	34	17	31
2688	rRNA 2'-O-methyltransferase fibrillarin	IPI00025039	-0.387	0.11370	9	9	9	6
2689	Vesicular integral-membrane protein VIP36	IPI00009950	-0.387	0.11370	8	10	5	10
2690	septin-9 isoform e	IPI00455033	-0.393	0.11294	8	9	6	8
2691	Pirin	IPI00012575	-0.393	0.11294	7	10	8	6
2692	Protein NipSnap homolog 2	IPI00016077	-0.393	0.11294	8	9	5	9
2693	Succinyl-CoA:3-ketoacid-coenzyme A transferase 1, mitochondrial	IPI00026516	-0.393	0.11294	8	9	6	8
2694	Structural maintenance of chromosomes protein 1A	IPI00291939	-0.397	0.11207	25	21	18	24
2695	Nascent polypeptide-associated complex subunit alpha	IPI00023748	-0.397	0.11207	23	23	19	23
2696	60S ribosomal protein L5	IPI00000494	-0.400	0.11162	5	11	4	9
2697	cDNA FLJ35809 fis, clone TESTI2006016, highly similar to Eukaryotic translation initiation factor 3	IPI00647650	-0.400	0.11162	10	6	7	6
2698	Dihydroorotate dehydrogenase, mitochondrial	IPI00024462	-0.400	0.11162	9	7	7	6
2699	Isoform 1 of BRCA2 and CDKN1A-interacting protein	IPI00002203	-0.400	0.11162	9	7	5	8
2700	Synaptic vesicle membrane protein VAT-1 homolog	IPI00156689	-0.400	0.11162	8	8	5	8
2701	Vimentin	IPI00418471	-0.404	0.11026	47	51	46	47
2702	Gamma-aminobutyric acid receptor-associated protein-like 2	IPI00026358	-0.408	0.10984	7	8	6	6
2703	N(G),N(G)-dimethylarginine dimethylaminohydrolase 2	IPI00000760	-0.408	0.10984	7	8	6	6
2704	Putative uncharacterized protein PSME2	IPI00384051	-0.410	0.10939	21	20	19	18
2705	Isoform E of Eukaryotic translation initiation factor 4 gamma 1	IPI00386533	-0.410	0.10939	22	19	19	18
2706	Isoform 1 of Heterogeneous nuclear ribonucleoprotein A3	IPI00419373	-0.410	0.10939	14	27	14	23
2707	Lamin-B1	IPI00217975	-0.413	0.10935	23	17	17	19
2708	Isoform 1 of Cytoskeleton-associated protein 5	IPI00028275	-0.416	0.10916	22	17	16	19
2709	Nucleolar protein 56	IPI00411937	-0.416	0.10916	19	20	17	18
2710	Condensin-2 complex subunit D3	IPI00747787	-0.416	0.10916	8	6	4	7
2711	Isoform 1 of General transcription factor 3C polypeptide 1	IPI00414482	-0.416	0.10916	9	5	4	7
2712	Deoxyribonucleoside 5'-monophosphate N-glycosidase	IPI00007926	-0.416	0.10916	6	8	4	7
2713	Proteasome subunit beta type-6	IPI00000811	-0.416	0.10916	6	8	5	6
2714	DnaJ homolog subfamily B member 1	IPI00015947	-0.416	0.10916	8	6	5	6
2715	Metastasis-associated protein MTA2	IPI00171798	-0.416	0.10916	7	7	4	7
2716	Isoform NELF-C of Negative elongation factor C/D	IPI00164949	-0.416	0.10916	8	6	5	6
2717	Carbonyl reductase [NADPH] 3	IPI00290462	-0.416	0.10916	7	7	4	7
2718	proteasome-associated protein ECM29 homolog	IPI00157790	-0.422	0.10739	19	18	17	16
2719	Isoform 1 of Keratin, type I cytoskeletal 13	IPI00009866	-0.422	0.10739	17	20	19	14
2720	Beta-hexosaminidase subunit beta	IPI00012585	-0.426	0.10701	4	9	6	4
2721	Protein VAC14 homolog	IPI00025160	-0.426	0.10701	6	7	5	5
2722	Importin subunit alpha-4	IPI00012578	-0.426	0.10701	7	6	6	4
2723	NEDD8-activating enzyme E1 regulatory subunit	IPI00018968	-0.426	0.10701	6	7	4	6
2724	sorting nexin-1 isoform c	IPI00183274	-0.426	0.10701	8	5	5	5
2725	ATP-dependent RNA helicase DDX50	IPI00031554	-0.426	0.10701	7	6	4	6
2726	regulator of chromosome condensation 1 isoform a	IPI00001661	-0.426	0.10701	7	6	5	5
2727	Pre-mRNA-splicing factor SPF27	IPI00025178	-0.426	0.10701	6	7	5	5
2728	cDNA FLJ59758, highly similar to S-methyl-5-thioadenosine phosphorylase	IPI00011876	-0.428	0.10595	18	17	15	16
2729	Phosphoglycerate kinase 1	IPI00169383	-0.433	0.10523	71	78	61	82
2730	Isoform 2 of Basigin	IPI00019906	-0.436	0.10516	15	18	11	18
2731	Isoform Beta-4C of Integrin beta-4	IPI00027422	-0.436	0.10516	40	34	29	40
2732	Aldose reductase	IPI00413641	-0.436	0.10516	7	5	4	5
2733	Isoform 2 of U1 small nuclear ribonucleoprotein 70 kDa	IPI00219483	-0.436	0.10516	5	7	5	4
2734	Probable rRNA-processing protein EBP2	IPI00745955	-0.436	0.10516	6	6	4	5
2735	COP9 signalosome complex subunit 3	IPI00025721	-0.436	0.10516	5	7	3	6
2736	Thioredoxin domain-containing protein 5	IPI00171438	-0.436	0.10516	5	7	4	5
2737	Isoform 1 of N-alpha-acetyltransferase 50, NatE catalytic subunit	IPI00018627	-0.436	0.10516	6	6	4	5

No.	Description	Accession number	STN[1]	p-Value[1]	Con_A[2]	Con_B[2]	SORA_A[2]	SORA_B[2]
2738	Isoform 1 of DDRGK domain-containing protein 1	IPI00028387	-0.436	0.10516	6	6	5	4
2739	Tropomodulin-3	IPI00005087	-0.436	0.10516	6	6	5	4
2740	AFG3-like protein 2	IPI00001091	-0.436	0.10516	6	6	6	3
2741	Proteasome 26S non-ATPase subunit 11 variant (Fragment)	IPI00105598	-0.439	0.10368	16	16	15	13
2742	ATP-dependent RNA helicase DDX18	IPI00301323	-0.443	0.10311	17	14	14	13
2743	Multifunctional protein ADE2	IPI00217223	-0.446	0.10311	28	40	29	34
2744	F-actin-capping protein subunit alpha-1	IPI00005969	-0.448	0.10300	15	15	12	14
2745	SUMO-activating enzyme subunit 2	IPI00023234	-0.448	0.10300	18	12	11	15
2746	Isoform Crk-II of Adapter molecule crk	IPI00004838	-0.448	0.10255	7	4	5	3
2747	Isoform 1 of Pleiotropic regulator 1	IPI00002624	-0.448	0.10255	7	4	4	4
2748	60S ribosomal protein L7-like 1	IPI00456940	-0.448	0.10255	4	7	5	3
2749	Tubulin-specific chaperone E	IPI00018402	-0.448	0.10255	7	4	4	4
2750	Diphosphoinositol polyphosphate phosphohydrolase 1	IPI00009148	-0.448	0.10255	3	8	3	5
2751	Putative deoxyribose-phosphate aldolase	IPI00219677	-0.448	0.10255	6	5	4	4
2752	Isoform 1 of Elongation factor Ts, mitochondrial	IPI00021016	-0.448	0.10255	5	6	4	4
2753	Mitochondrial import receptor subunit TOM22 homolog	IPI00024976	-0.448	0.10255	5	6	4	4
2754	Protein disulfide-isomerase A3	IPI00025252	-0.460	0.10077	45	74	51	62
2755	Peptidyl-prolyl cis-trans isomerase D	IPI00003927	-0.462	0.10077	6	4	4	3
2756	SWI/SNF complex subunit SMARCC1	IPI00234252	-0.462	0.10077	6	4	3	4
2757	DNA-directed RNA polymerases I, II, and III subunit RPABC1	IPI00291093	-0.462	0.10077	4	6	3	4
2758	NADH dehydrogenase [ubiquinone] flavoprotein 2, mitochondrial	IPI00291328	-0.462	0.10077	4	6	3	4
2759	Isoform 1 of Replication protein A 32 kDa subunit	IPI00013939	-0.462	0.10077	5	5	3	4
2760	Isoform 1 of Polymerase I and transcript release factor	IPI00176903	-0.462	0.10077	5	5	3	4
2761	Isoform 4 of Abhydrolase domain-containing protein 11	IPI00171152	-0.462	0.10077	5	5	3	4
2762	Isoform 1 of Chromosome-associated kinesin KIF4A	IPI00178150	-0.462	0.10077	6	4	3	4
2763	COP9 signalosome complex subunit 8	IPI00009480	-0.462	0.10077	4	6	3	4
2764	Ewing sarcoma breakpoint region 1 isoform 1	IPI00009841	-0.462	0.10077	7	3	4	3
2765	Isoform 1 of Oligoribonuclease, mitochondrial (Fragment)	IPI00032830	-0.462	0.10077	6	4	4	3
2766	UBX domain-containing protein 4	IPI00293946	-0.462	0.10077	6	4	3	4
2767	Dolichyl-diphosphooligosaccharide--protein glycosyltransferase subunit 1 precursor	IPI00025874	-0.466	0.09748	32	26	21	32
2768	cDNA FLJ40024 fis, clone STOMA2007745, highly similar to UBIQUITIN-ACTIVATING ENZYME E1	IPI00026119	-0.471	0.09627	50	59	50	53
2769	Dihydropyrimidinase-related protein 2	IPI00257508	-0.472	0.09627	14	11	11	10
2770	Seryl-tRNA synthetase, cytoplasmic	IPI00220637	-0.472	0.09627	12	13	10	11
2771	DnaJ homolog subfamily A member 2	IPI00032406	-0.472	0.09627	12	13	10	11
2772	Calcium-binding protein 39-like	IPI00026359	-0.472	0.09627	11	14	12	9
2773	26S proteasome non-ATPase regulatory subunit 5	IPI00002134	-0.472	0.09627	13	12	8	13
2774	Cytoplasmic dynein 1 heavy chain 1	IPI00456969	-0.474	0.09616	242	238	217	254
2775	T-complex protein 1 subunit epsilon	IPI00010720	-0.475	0.09616	54	52	45	55
2776	Isoform 1 of Elongation factor 1-delta	IPI00023048	-0.476	0.09616	22	32	21	28
2777	Isoform 1 of La-related protein 1	IPI00185919	-0.477	0.09593	12	12	9	11
2778	Isoform 1 of Putative helicase MOV-10	IPI00444452	-0.478	0.09469	4	5	0	4
2779	Isoform 1 of ATPase family AAA domain-containing protein 1	IPI00171445	-0.478	0.09469	5	4	2	4
2780	Isoform 1 of ATP synthase subunit d, mitochondrial	IPI00220487	-0.478	0.09469	4	5	4	2
2781	DNA-directed RNA polymerase I subunit RPA1	IPI00031960	-0.478	0.09469	4	5	0	4
2782	UV excision repair protein RAD23 homolog B	IPI00008223	-0.478	0.09469	5	4	3	3
2783	Visinin-like protein 1	IPI00216313	-0.478	0.09469	4	5	0	4
2784	Copine-3	IPI00024403	-0.478	0.09469	7	2	4	2
2785	Isoform 2 of Leucyl-cystinyl aminopeptidase	IPI00221240	-0.478	0.09469	6	3	2	4
2786	Full-length cDNA 5-PRIME end of clone CS0DJ009YL13 of T cells (Jurkat cell line) of Homo sapiens	IPI00384016	-0.478	0.09469	6	3	3	3
2787	Isoform Long of FAS-associated factor 1	IPI00070643	-0.478	0.09469	6	3	2	4
2788	YEATS domain-containing protein 4	IPI00008536	-0.478	0.09469	5	4	4	2
2789	Isoform 1 of Transcription intermediary factor 1-beta	IPI00438229	-0.481	0.09359	27	25	23	24
2790	Endoplasmic reticulum metallopeptidase 1	IPI00257903	-0.483	0.09359	13	10	9	10
2791	Translin-associated protein X	IPI00293350	-0.483	0.09359	10	13	10	9
2792	Heterogeneous nuclear ribonucleoprotein A0	IPI00011913	-0.483	0.09359	10	13	8	11
2793	Matrin-3	IPI00017297	-0.486	0.09348	25	25	22	23
2794	Phenylalanyl-tRNA synthetase alpha chain	IPI00031820	-0.490	0.09325	10	12	11	7
2795	Elongator complex protein 1	IPI00293735	-0.497	0.09143	12	9	11	6
2796	Isoform 1 of BH3-interacting domain death agonist	IPI00413587	-0.497	0.09143	11	10	8	9
2797	33 kDa protein	IPI00413108	-0.497	0.09143	13	8	8	9
2798	Isoform Beta of Nucleolar and coiled-body phosphoprotein 1	IPI00216654	-0.497	0.09140	5	3	3	2
2799	Isoform 3 of Nucleoporin NDC1	IPI00074330	-0.497	0.09140	6	0	2	3
2800	Isoform 1 of Calcineurin-like phosphoesterase domain-containing protein 1	IPI00305010	-0.497	0.09140	4	4	3	0
2801	Protein FAM98B	IPI00167572	-0.497	0.09140	4	4	2	3
2802	Isoform 4 of Nucleoporin NDC1	IPI00003455	-0.497	0.09140	3	5	2	3
2803	Isoform p26 of 7,8-dihydro-8-oxoguanine triphosphatase	IPI00004392	-0.497	0.09140	2	6	2	3
2804	Isoform 5 of Brain-specific angiogenesis inhibitor 1-associated protein 2	IPI00180292	-0.497	0.09140	4	4	0	3
2805	Methylosome subunit pICln	IPI00004795	-0.497	0.09140	4	4	2	3
2806	Isoform Rpn10A of 26S proteasome non-ATPase regulatory subunit 4	IPI00022694	-0.497	0.09140	3	5	2	3
2807	Toll-interacting protein	IPI00100154	-0.497	0.09140	4	4	2	3
2808	TAF6-like RNA polymerase II p300/CBP-associated factor-associated factor 65 kDa subunit 6L	IPI00007957	-0.497	0.09140	4	4	3	2
2809	JmjC domain-containing protein 7	IPI00382394	-0.497	0.09140	4	4	3	2
2810	Isoform 2 of Spliceosome RNA helicase BAT1	IPI00641829	-0.501	0.09113	21	24	22	18
2811	26S proteasome non-ATPase regulatory subunit 14	IPI00024821	-0.504	0.09106	11	9	9	7
2812	Early endosome antigen 1	IPI00329536	-0.504	0.09106	9	11	6	10
2813	perilipin-3 isoform 3	IPI00106668	-0.504	0.09106	10	10	9	7
2814	Tricarboxylate transport protein, mitochondrial	IPI00294159	-0.512	0.08856	7	12	7	8
2815	60S ribosomal protein L26-like 1	IPI00007144	-0.512	0.08856	9	10	6	9
2816	Isoform Non-muscle of Myosin light polypeptide 6	IPI00335168	-0.512	0.08856	6	13	8	7
2817	Isoform 1 of Nuclear pore complex protein Nup98-Nup96	IPI00006038	-0.512	0.08856	10	9	7	8
2818	cDNA FLJ77422	IPI00011268	-0.512	0.08856	8	11	6	9
2819	Isoform 1 of RNA-binding protein 8A	IPI00001757	-0.512	0.08856	9	10	7	8
2820	Histone H2A type 1-B/E	IPI00026272	-0.518	0.08803	0	38	0	33
2821	Isoform 1 of Structural maintenance of chromosomes protein 2	IPI00007927	-0.518	0.08803	19	21	14	21
2822	Isoform 1 of Importin-4	IPI00156374	-0.520	0.08781	11	7	8	6
2823	Histone acetyltransferase type B catalytic subunit	IPI00024719	-0.520	0.08781	9	9	7	7
2824	Isoform 1 of Nuclear pore complex protein Nup214	IPI00183294	-0.521	0.08508	3	4	2	0
2825	Protein phosphatase 1 regulatory subunit 14B	IPI00398922	-0.521	0.08508	5	0	0	0
2826	Protein of unknown function UPF0118 family protein	IPI00012429	-0.521	0.08508	5	0	0	0
2827	Putative uncharacterized protein NAPRT1	IPI00412498	-0.521	0.08508	5	2	0	0
2828	Isoform 1 of Neuroblastoma-amplified sequence	IPI00333913	-0.521	0.08508	4	3	0	0
2829	Isoform 2 of Ubiquitin carboxyl-terminal hydrolase 47	IPI00165528	-0.521	0.08508	3	4	0	2
2830	Keratin, type II cytoskeletal 6B	IPI00293665	-0.521	0.08508	2	5	0	0
2831	Claudin-1	IPI00000691	-0.521	0.08508	5	0	2	0
2832	Isoform 1 of Anaphase-promoting complex subunit 4	IPI00002551	-0.521	0.08508	5	0	2	2

No.	Description	Accession number	STN[1]	p-Value[1]	Con_A[2]	Con_B[2]	SORA_A[2]	SORA_B[2]
2833	Ubiquitin-conjugating enzyme E2 T	IPI00023087	-0.521	0.08508	3	4	0	0
2834	Isoform 1 of Dehydrogenase/reductase SDR family member 7	IPI00006957	-0.521	0.08508	3	4	2	2
2835	Isoform 1 of Thymocyte nuclear protein 1	IPI00383163	-0.521	0.08508	3	4	0	2
2836	Glycine cleavage system H protein, mitochondrial	IPI00011604	-0.521	0.08508	5	0	0	0
2837	Ribosome biogenesis protein BOP1	IPI00028955	-0.521	0.08508	3	4	2	0
2838	Isoform 2 of Lysocardiolipin acyltransferase 1	IPI00166225	-0.521	0.08508	5	2	2	2
2839	Bifunctional polynucleotide phosphatase/kinase	IPI00290684	-0.521	0.08508	3	4	2	0
2840	Isoform Long of Transformer-2 protein homolog alpha	IPI00013891	-0.521	0.08508	4	3	2	0
2841	Serine palmitoyltransferase 2	IPI00005751	-0.521	0.08508	3	4	0	0
2842	Isoform 1 of WD repeat-containing protein 74	IPI00018192	-0.521	0.08508	4	3	0	0
2843	Endonuclease/exonuclease/phosphatase family domain-containing protein 1	IPI00885036	-0.521	0.08508	4	3	0	0
2844	Isoform 3 of Exocyst complex component 7	IPI00103064	-0.521	0.08508	4	3	0	0
2845	Protein FAM83H	IPI00784320	-0.521	0.08508	5	0	0	0
2846	Zinc finger protein ubi-d4	IPI00023322	-0.521	0.08508	4	3	0	2
2847	Myotubularin	IPI00748788	-0.521	0.08508	5	2	0	0
2848	Isoform 1 of Serine protease HTRA2, mitochondrial	IPI00001663	-0.521	0.08508	5	0	2	2
2849	Actin-related protein 5	IPI00292787	-0.521	0.08508	4	3	0	0
2850	cDNA FLJ61658, highly similar to Transmembrane 9 superfamily protein member 1	IPI00101374	-0.521	0.08508	0	5	0	0
2851	Exocyst complex component 2	IPI00783559	-0.521	0.08508	4	3	0	0
2852	Isoform 1 of THO complex subunit 1	IPI00305374	-0.521	0.08508	4	3	0	0
2853	Isoform 2 of Apoptosis inhibitor 5	IPI00554742	-0.521	0.08316	20	19	14	20
2854	Isoform 1 of Acidic leucine-rich nuclear phosphoprotein 32 family member B	IPI00007423	-0.525	0.08304	18	20	15	18
2855	6-phosphogluconate dehydrogenase, decarboxylating	IPI00219525	-0.537	0.08267	38	30	25	37
2856	Sepiapterin reductase	IPI00017469	-0.539	0.08244	6	10	6	6
2857	Isoform 1 of Prolyl 4-hydroxylase subunit alpha-1	IPI00009923	-0.539	0.08244	8	8	7	5
2858	Phenylalanyl-tRNA synthetase beta chain	IPI00300074	-0.547	0.07957	16	17	11	17
2859	V-type proton ATPase subunit B, brain isoform	IPI00007812	-0.547	0.07957	16	17	12	16
2860	Isoform 2 of Inverted formin-2	IPI00876962	-0.550	0.07953	6	9	4	7
2861	Serine/threonine-protein kinase OSR1	IPI00010080	-0.550	0.07953	7	8	5	6
2862	tropomyosin alpha-3 chain isoform 1	IPI00183968	-0.550	0.07953	8	7	6	5
2863	Mitogen-activated protein kinase 1	IPI00003479	-0.550	0.07953	8	7	6	5
2864	Isoform 3 of PCI domain-containing protein 2	IPI00072541	-0.550	0.07953	8	7	6	5
2865	Histone H1.2	IPI00217465	-0.553	0.07919	22	39	29	26
2866	Isoform 1 of Protein-L-isoaspartate(D-aspartate) O-methyltransferase	IPI00411680	-0.557	0.07885	15	16	10	16
2867	Carbonyl reductase [NADPH] 1	IPI00295386	-0.557	0.07885	11	20	10	16
2868	Serpin H1	IPI00032140	-0.557	0.07885	15	16	13	13
2869	Transmembrane protein 33	IPI00299084	-0.562	0.07855	10	4	4	6
2870	Ubiquitin carboxyl-terminal hydrolase 24	IPI00902614	-0.562	0.07855	6	8	3	7
2871	Isoform 2 of COP9 signalosome complex subunit 2	IPI00018813	-0.562	0.07855	6	8	5	5
2872	Leucine-rich repeat and WD repeat-containing protein 1	IPI00069309	-0.562	0.07855	8	6	4	6
2873	NAD-dependent malic enzyme, mitochondrial	IPI00011201	-0.562	0.07855	8	6	5	5
2874	Transmembrane 9 superfamily member 2	IPI00018415	-0.562	0.07855	7	7	8	2
2875	Gem-associated protein 5	IPI00291783	-0.562	0.07855	7	7	5	5
2876	Isoform 1 of Annexin A7	IPI00002460	-0.562	0.07855	7	7	5	5
2877	Isoform 1 of Importin-5	IPI00793443	-0.564	0.07522	30	27	21	30
2878	Villin-1	IPI00218852	-0.572	0.07492	27	27	19	29
2879	transcription activator BRG1 isoform D	IPI00029822	-0.574	0.07492	15	13	14	9
2880	Ras-related protein Rab-10	IPI00016513	-0.575	0.07477	7	6	0	7
2881	Nucleolar complex protein 4 homolog	IPI00031661	-0.575	0.07477	7	6	5	4
2882	Putative uncharacterized protein INF2	IPI00872508	-0.575	0.07477	6	7	3	6
2883	39S ribosomal protein L1, mitochondrial	IPI00549381	-0.575	0.07477	5	8	5	4
2884	Peptidyl-tRNA hydrolase 2, mitochondrial	IPI00032903	-0.575	0.07477	9	4	4	5
2885	Isoform 1 of Protein POF1B	IPI00103242	-0.575	0.07477	7	6	4	5
2886	Aladin	IPI00024143	-0.591	0.07382	8	4	2	6
2887	Plakophilin-3	IPI00026952	-0.591	0.07382	5	7	5	3
2888	MKI67 FHA domain-interacting nucleolar phosphoprotein	IPI00154590	-0.591	0.07382	5	7	4	4
2889	FAS-associated factor 2	IPI00172656	-0.591	0.07382	7	5	3	5
2890	CCR4-NOT transcription complex subunit 7	IPI00006552	-0.591	0.07382	5	7	2	6
2891	Isoform 1 of C-terminal-binding protein 2	IPI00010120	-0.591	0.07382	7	5	0	6
2892	40S ribosomal protein S15	IPI00479058	-0.592	0.06864	22	26	20	22
2893	Isoform 1 of Heterogeneous nuclear ribonucleoprotein D-like	IPI00011274	-0.593	0.06838	11	14	8	12
2894	Isoform 2 of 4F2 cell-surface antigen heavy chain	IPI00027493	-0.595	0.06819	24	23	19	22
2895	Putative uncharacterized protein DKFZp686L20222	IPI00026689	-0.599	0.06819	18	28	20	20
2896	Isoform 1 of RRP12-like protein	IPI00101186	-0.601	0.06800	13	11	8	11
2897	Adenine phosphoribosyltransferase	IPI00218693	-0.601	0.06800	12	12	10	9
2898	Mitochondrial carrier homolog 2	IPI00003833	-0.603	0.06774	19	26	15	24
2899	Protein MEMO1	IPI00032426	-0.608	0.06732	5	6	4	3
2900	U4/U6.U5 tri-snRNP-associated protein 2	IPI00419844	-0.608	0.06732	6	5	4	3
2901	SWI/SNF related, matrix associated, actin dependent regulator of chromatin, subfamily a, member 1	IPI00216046	-0.608	0.06732	6	5	5	2
2902	nardilysin isoform a	IPI00243221	-0.608	0.06732	6	5	3	4
2903	Oxysterol-binding protein	IPI00163644	-0.608	0.06732	8	3	3	4
2904	Deoxycytidine kinase	IPI00020454	-0.608	0.06732	5	6	4	3
2905	Isoform 1 of Actin-like protein 6A	IPI00003627	-0.608	0.06732	7	4	4	3
2906	Isoform A of Uncharacterized protein C21orf70	IPI00027898	-0.608	0.06732	6	5	4	3
2907	Nucleoporin Nup43	IPI00742943	-0.608	0.06732	6	5	4	3
2908	Isoform 1 of Tropomyosin alpha-4 chain	IPI00010779	-0.608	0.06732	11	12	8	10
2909	Isoform 2 of Tropomyosin alpha-3 chain	IPI00218319	-0.608	0.06732	8	15	7	11
2910	Lamin-B2	IPI00009771	-0.608	0.06732	13	10	7	11
2911	Mitochondrial 2-oxoglutarate/malate carrier protein	IPI00219729	-0.615	0.06724	20	22	14	22
2912	Cytoplasmic dynein 1 light intermediate chain 2	IPI00011592	-0.617	0.06698	11	11	8	9
2913	Isoform 1 of Hydroxyacyl-coenzyme A dehydrogenase, mitochondrial	IPI00294398	-0.626	0.06656	10	11	8	8
2914	Isoform 1 of 3,2-trans-enoyl-CoA isomerase, mitochondrial	IPI00300567	-0.626	0.06656	10	11	6	10
2915	Isoform 1 of Large proline-rich protein BAT3	IPI00465128	-0.626	0.06656	10	11	6	10
2916	Heat shock 70 kDa protein 12A	IPI00011932	-0.626	0.06656	8	13	8	8
2917	Isoform F of Protein SON	IPI00000192	-0.628	0.06622	5	5	0	4
2918	Putative rRNA methyltransferase 3	IPI00217686	-0.628	0.06622	6	4	3	3
2919	60S ribosomal protein L17	IPI00413324	-0.628	0.06622	4	6	4	0
2920	Isoform 1 of tRNA guanosine-2'-O-methyltransferase TRM11 homolog	IPI00470606	-0.628	0.06622	5	5	3	3
2921	Dipeptidase 1	IPI00059476	-0.628	0.06622	4	6	2	4
2922	Mitochondrial import inner membrane translocase subunit Tim17-B	IPI00219833	-0.628	0.06622	6	4	0	4
2923	NADP-dependent malic enzyme	IPI00008215	-0.628	0.06622	5	5	4	2
2924	Mitochondrial chaperone BCS1	IPI00003985	-0.628	0.06622	5	5	2	4
2925	Charged multivesicular body protein 5	IPI00100796	-0.628	0.06622	5	5	3	3
2926	Glutamate-rich WD repeat-containing protein 1	IPI00027831	-0.628	0.06622	6	4	3	3

No.	Description	Accession number	STN[1]	p-Value[1]	Con_A[2]	Con_B[2]	SORA_A[2]	SORA_B[2]
2927	Isoform 1 of Acylglycerol kinase, mitochondrial	IPI00019353	-0.628	0.06622	6	4	3	3
2928	Ribonuclease inhibitor	IPI00550069	-0.628	0.06622	5	5	3	3
2929	Isoform 1 of Protein FAM65A	IPI00418799	-0.628	0.06622	7	3	0	4
2930	Isoform 1 of Protein NDRG3	IPI00005605	-0.628	0.06622	5	5	4	2
2931	Methylmalonyl-CoA mutase, mitochondrial	IPI00024934	-0.628	0.06622	3	7	4	0
2932	Nuclear pore complex protein Nup93	IPI00397904	-0.635	0.06169	12	8	5	10
2933	Isoform 1 of Cell division cycle and apoptosis regulator protein 1	IPI00217357	-0.635	0.06169	12	8	6	9
2934	cDNA FLJ60317, highly similar to Aminoacylase-1	IPI00009268	-0.635	0.06169	7	13	7	8
2935	Isoform Epsilon of Apoptosis regulator BAX	IPI00071059	-0.635	0.06169	10	10	8	7
2936	Isoform Complexed of Arginyl-tRNA synthetase, cytoplasmic	IPI00004860	-0.638	0.06161	20	17	13	18
2937	Calreticulin	IPI00020599	-0.644	0.06097	28	34	22	33
2938	Cytochrome c oxidase subunit 4 isoform 1, mitochondrial	IPI00006579	-0.645	0.06086	10	9	5	9
2939	protein ALO17 isoform 1	IPI00828098	-0.652	0.06048	3	6	3	2
2940	ribonucleoprotein PTB-binding 1	IPI00217661	-0.652	0.06048	4	5	0	3
2941	Isoform 1 of Dr1-associated corepressor	IPI00003084	-0.652	0.06048	3	6	3	0
2942	Uncharacterized protein KIAA0406	IPI00011702	-0.652	0.06048	6	3	0	3
2943	DNA ligase 1	IPI00219841	-0.652	0.06048	4	5	3	2
2944	cDNA FLJ56420, highly similar to Aspartyl aminopeptidase	IPI00015856	-0.652	0.06048	3	6	0	3
2945	Translational activator of cytochrome c oxidase 1	IPI00019903	-0.652	0.06048	5	4	0	3
2946	Isoform 1 of Beta-galactosidase	IPI00441344	-0.652	0.06048	3	6	0	3
2947	Synaptosomal-associated protein 29	IPI00032831	-0.652	0.06048	6	3	0	3
2948	Serum deprivation-response protein	IPI00005809	-0.652	0.06048	4	5	2	3
2949	Isoform 1 of ATP-dependent metalloprotease YME1L1	IPI00045946	-0.652	0.06048	5	4	2	3
2950	Actin-related protein 2/3 complex subunit 2	IPI00005161	-0.656	0.06006	8	10	6	7
2951	165 kDa protein	IPI00240812	-0.656	0.06006	11	7	6	7
2952	Isoform 1 of Voltage-dependent anion-selective channel protein 3	IPI00031804	-0.656	0.06006	8	10	5	8
2953	Pre-rRNA-processing protein TSR1 homolog	IPI00292894	-0.656	0.06006	7	11	4	9
2954	Alpha-soluble NSF attachment protein	IPI00009253	-0.656	0.06006	9	9	4	9
2955	Isoform 5 of Glycogen debranching enzyme	IPI00219065	-0.660	0.05957	17	16	12	15
2956	Cytochrome b-c1 complex subunit 2, mitochondrial	IPI00305383	-0.666	0.05938	14	18	12	14
2957	Vacuolar protein sorting-associated protein 35	IPI00018931	-0.666	0.05908	29	26	19	29
2958	Isoform 1 of Reticulon-4	IPI00021766	-0.668	0.05904	8	9	3	9
2959	cDNA FLJ56153, highly similar to Homo sapiens transforming growth factor beta regulator 4	IPI00329625	-0.668	0.05904	9	8	5	7
2960	Adenylosuccinate synthetase isozyme 2	IPI00026833	-0.668	0.05904	8	9	4	8
2961	Monocarboxylate transporter 4	IPI00006666	-0.668	0.05904	10	7	7	5
2962	Replication protein A 70 kDa DNA-binding subunit	IPI00020127	-0.681	0.05851	10	6	6	5
2963	COP9 signalosome complex subunit 5	IPI00009958	-0.681	0.05851	10	6	6	5
2964	Nuclear pore complex protein Nup107	IPI00028005	-0.681	0.05851	9	7	4	7
2965	DnaJ homolog subfamily C member 9	IPI00154975	-0.681	0.05851	7	9	6	5
2966	60S ribosomal protein L13a	IPI00304612	-0.682	0.05817	0	6	0	0
2967	Isoform 1 of Minor histocompatibility antigen H13	IPI00152441	-0.682	0.05817	6	0	2	2
2968	E3 ubiquitin-protein ligase HECTD1	IPI00328911	-0.682	0.05817	5	3	2	2
2969	NADH dehydrogenase [ubiquinone] 1 alpha subcomplex subunit 8	IPI00219034	-0.682	0.05817	3	5	2	0
2970	Isoform 1 of Disco-interacting protein 2 homolog B	IPI00465045	-0.682	0.05817	6	2	0	2
2971	Isoform 1 of KDEL motif-containing protein 2	IPI00143921	-0.682	0.05817	4	4	2	2
2972	Procollagen-lysine,2-oxoglutarate 5-dioxygenase 3	IPI00030255	-0.682	0.05817	3	5	0	0
2973	Prostaglandin E synthase 2	IPI00303568	-0.682	0.05817	4	4	0	0
2974	Isoform 1 of Cytochrome c oxidase assembly protein COX15 homolog	IPI00419869	-0.682	0.05817	3	5	0	0
2975	54 kDa protein	IPI00177890	-0.682	0.05817	4	4	0	0
2976	NADH dehydrogenase [ubiquinone] iron-sulfur protein 7, mitochondrial	IPI00307749	-0.682	0.05817	5	3	2	0
2977	Isoform 1 of Acetolactate synthase-like protein	IPI00554541	-0.682	0.05817	5	3	0	0
2978	Isoform 1 of Anaphase-promoting complex subunit 7	IPI00008248	-0.682	0.05817	4	4	2	2
2979	Isoform A of Peptidyl-prolyl cis-trans isomerase E	IPI00009316	-0.682	0.05817	6	0	2	0
2980	Carnitine O-palmitoyltransferase 2, mitochondrial	IPI00012912	-0.682	0.05817	5	3	0	0
2981	Putative uncharacterized protein ALB	IPI00022434	-0.682	0.05817	4	4	0	0
2982	Isoform 2 of Pinin	IPI00002649	-0.682	0.05817	5	3	2	0
2983	Isoform 1 of Dephospho-CoA kinase domain-containing protein	IPI00291417	-0.682	0.05817	4	4	0	0
2984	Neprilysin	IPI00247063	-0.682	0.05817	3	5	0	2
2985	Isoform 1 of Lymphoid-specific helicase	IPI00010590	-0.682	0.05817	3	5	0	0
2986	Probable ATP-dependent RNA helicase DDX20	IPI00005904	-0.682	0.05817	4	4	0	0
2987	Squalene synthase	IPI00020944	-0.682	0.05817	4	4	2	2
2988	Myosin-Id	IPI00329719	-0.682	0.05817	5	3	2	2
2989	Tetratricopeptide repeat protein 27	IPI00183938	-0.682	0.05817	6	0	0	2
2990	cDNA FLJ56180, highly similar to Negative elongation factor E	IPI00000858	-0.682	0.05817	5	3	2	2
2991	Huntingtin-interacting protein 1	IPI00782965	-0.682	0.05817	4	4	0	2
2992	Isoform 2 of 5'-3' exoribonuclease 1	IPI00657645	-0.682	0.05817	4	4	0	2
2993	82 kDa protein	IPI00719051	-0.682	0.05817	6	2	0	0
2994	Isoform 1 of Heterogeneous nuclear ribonucleoprotein M	IPI00171903	-0.682	0.05080	35	47	40	34
2995	6-phosphofructokinase type C	IPI00009790	-0.682	0.05080	38	44	32	42
2996	Collapsin response mediator protein 4 long variant	IPI00029111	-0.685	0.05069	14	15	12	11
2997	26S protease regulatory subunit 4	IPI00011126	-0.685	0.05069	12	17	9	14
2998	116 kDa U5 small nuclear ribonucleoprotein component	IPI00003519	-0.689	0.05069	25	24	19	23
2999	Inosine-5'-monophosphate dehydrogenase 2	IPI00291510	-0.693	0.04967	23	25	19	22
3000	Cleavage and polyadenylation specificity factor subunit 1	IPI00026219	-0.696	0.04955	7	8	6	4
3001	Isoform 1 of F-actin-capping protein subunit beta	IPI00026185	-0.696	0.04955	6	9	5	5
3002	Microsomal glutathione S-transferase 3	IPI00024266	-0.696	0.04955	8	7	6	4
3003	Protein of unknown function DUF410 family protein	IPI00419575	-0.696	0.04955	7	8	3	7
3004	Isoform 4 of E3 ubiquitin-protein ligase UBR4	IPI00640981	-0.697	0.04936	20	27	17	23
3005	Peptidyl-prolyl cis-trans isomerase FKBP4	IPI00219005	-0.702	0.04929	36	38	32	34
3006	SUMO-conjugating enzyme UBC9	IPI00032957	-0.712	0.04808	5	9	3	6
3007	ADP-ribosylation factor-like protein 1	IPI00219518	-0.712	0.04808	8	6	4	5
3008	Isoform 1 of Paraspeckle component 1	IPI00103525	-0.712	0.04808	7	7	5	4
3009	Isoform Cytoplasmic of Lysyl-tRNA synthetase	IPI00014238	-0.722	0.04687	32	35	28	31
3010	Rho GDP-dissociation inhibitor 1	IPI00003815	-0.725	0.04676	31	35	27	31
3011	26S protease regulatory subunit 8	IPI00023919	-0.725	0.04664	20	21	11	23
3012	Lactoylglutathione lyase	IPI00220766	-0.726	0.04661	11	13	8	10
3013	Mannosyl-oligosaccharide glucosidase	IPI00328170	-0.730	0.04559	7	6	5	3
3014	60S ribosomal protein L24	IPI00306332	-0.730	0.04559	5	8	3	5
3015	Isoform 1 of Tyrosine-protein kinase BAZ1B	IPI00069817	-0.730	0.04559	9	4	4	4
3016	Thioredoxin-related transmembrane protein 1	IPI00395887	-0.730	0.04559	8	5	4	4
3017	Isoform 1 of Serine/threonine-protein kinase 4	IPI00011488	-0.730	0.04559	5	8	6	2
3018	Isoform 5 of Thioredoxin reductase 1, cytoplasmic	IPI00554786	-0.730	0.04559	7	6	3	5
3019	Isoform 2 of Nuclear protein localization protein 4 homolog	IPI00001676	-0.736	0.04513	10	13	9	8
3020	Mitochondrial import receptor subunit TOM34	IPI00009946	-0.736	0.04513	12	11	8	9
3021	Nodal modulator 1	IPI00329352	-0.741	0.04487	22	16	14	17

No.	Description	Accession number	STN[1]	p-Value[1]	Con_A[2]	Con_B[2]	SORA_A[2]	SORA_B[2]
3022	transcriptional regulator ATRX isoform 2	IPI00220109	-0.751	0.04385	6	6	3	4
3023	magnesium transporter protein 1	IPI00301202	-0.751	0.04385	7	5	2	5
3024	Integrin-linked protein kinase	IPI00013219	-0.751	0.04385	6	6	3	4
3025	Isoform 1 of DNA-binding protein A	IPI00031801	-0.753	0.04377	15	21	13	16
3026	Isoform 1 of NADH-cytochrome b5 reductase 3	IPI00328415	-0.757	0.04377	8	13	7	8
3027	ATP synthase subunit O, mitochondrial	IPI00007611	-0.757	0.04377	10	11	7	8
3028	Replication factor C subunit 4	IPI00017381	-0.757	0.04377	11	10	7	8
3029	60S ribosomal protein L9	IPI00031691	-0.758	0.04377	43	43	39	38
3030	Isoform 1 of Heterogeneous nuclear ribonucleoprotein R	IPI00012074	-0.760	0.04377	25	31	24	24
3031	Eukaryotic translation initiation factor 5	IPI00022648	-0.769	0.04332	11	9	4	10
3032	Isoform 1 of Isocitrate dehydrogenase [NAD] subunit alpha, mitochondrial	IPI00030702	-0.773	0.04188	18	15	14	12
3033	Isoform 1 of Ataxin-2-like protein	IPI00456359	-0.775	0.04181	5	6	4	2
3034	Isoform A of Kinesin light chain 1	IPI00020096	-0.775	0.04181	7	4	4	2
3035	GTPase NRas	IPI00000005	-0.775	0.04181	7	4	0	4
3036	Eukaryotic translation initiation factor 4A, isoform 2, isoform CRA_b	IPI00030296	-0.775	0.04181	6	5	2	4
3037	Nucleolar protein 16	IPI00032849	-0.775	0.04181	5	6	3	3
3038	Transcription factor A, mitochondrial	IPI00020928	-0.781	0.04128	7	12	5	8
3039	S-adenosylmethionine synthase isoform type-2	IPI00010157	-0.781	0.04128	11	8	4	9
3040	40S ribosomal protein S3	IPI00011253	-0.785	0.04128	83	134	98	107
3041	Bifunctional aminoacyl-tRNA synthetase	IPI00013452	-0.785	0.04112	38	38	33	34
3042	3-hydroxyisobutyrate dehydrogenase, mitochondrial	IPI00013860	-0.795	0.04075	8	10	5	7
3043	Membrane-associated progesterone receptor component 1	IPI00220739	-0.795	0.04075	9	9	6	6
3044	cDNA FLJ60076, highly similar to ELAV-like protein 1	IPI00301936	-0.796	0.04014	14	16	11	12
3045	Plastin-1	IPI00032304	-0.799	0.04003	23	24	18	21
3046	cDNA FLJ54775, highly similar to Syntaxin-binding protein 2	IPI00019971	-0.803	0.03999	5	5	3	2
3047	Isoform CNPI of 2',3'-cyclic-nucleotide 3'-phosphodiesterase	IPI00220993	-0.803	0.03999	4	6	3	0
3048	Isoform 2 of Ubiquitin-conjugating enzyme E2 K	IPI00019894	-0.803	0.03999	5	5	0	3
3049	Farnesyltransferase, CAAX box, alpha, isoform CRA_a	IPI00026813	-0.803	0.03999	5	5	0	3
3050	Hsp90 co-chaperone Cdc37	IPI00013122	-0.803	0.03999	5	5	2	3
3051	ATP synthase subunit b, mitochondrial	IPI00029133	-0.804	0.03976	12	17	11	11
3052	Isoform 5 of Dynamin-1-like protein	IPI00037283	-0.809	0.03969	22	23	16	21
3053	mRNA turnover protein 4 homolog	IPI00106491	-0.811	0.03969	8	9	4	7
3054	Isoform p150 of Dynactin subunit 1	IPI00029485	-0.811	0.03969	10	7	6	5
3055	Isoform 2 of Tyrosine-protein phosphatase non-receptor type 11	IPI00298347	-0.811	0.03969	10	7	5	6
3056	Probable ATP-dependent RNA helicase DDX10	IPI00297900	-0.811	0.03969	8	9	6	5
3057	Cell division cycle 5-like protein	IPI00465294	-0.811	0.03969	9	8	5	6
3058	Isoform 2 of NSFL1 cofactor p47	IPI00022830	-0.811	0.03969	8	9	3	8
3059	Voltage-dependent anion-selective channel protein 1	IPI00216308	-0.820	0.03950	41	54	44	41
3060	Putative uncharacterized protein KIAA0664	IPI00024425	-0.822	0.03950	13	14	9	11
3061	Isoform Mitochondrial of Peroxiredoxin-5, mitochondrial	IPI00024915	-0.826	0.03946	20	22	16	18
3062	Transmembrane emp24 domain-containing protein 2	IPI00016608	-0.827	0.03942	6	10	6	4
3063	Isoform 1 of Exportin-2	IPI00022744	-0.831	0.03784	87	89	68	96
3064	probable E3 ubiquitin-protein ligase MYCBP2	IPI00289776	-0.832	0.03784	17	9	10	9
3065	U2 small nuclear ribonucleoprotein A'	IPI00297477	-0.832	0.03784	19	22	14	19
3066	Vitamin K epoxide reductase complex subunit 1-like protein 1	IPI00166079	-0.837	0.03780	0	7	0	0
3067	Isoform 1 of Peroxisomal membrane protein PEX16	IPI00006722	-0.837	0.03780	5	4	0	2
3068	NADH-ubiquinone oxidoreductase chain 2	IPI00007979	-0.837	0.03780	4	5	2	2
3069	Isoform 1 of HBS1-like protein	IPI00009070	-0.837	0.03780	2	7	2	2
3070	Unhealthy ribosome biogenesis protein 2 homolog	IPI00028980	-0.837	0.03780	5	4	0	2
3071	Glucosamine--fructose-6-phosphate aminotransferase [isomerizing] 2	IPI00216159	-0.837	0.03780	5	4	2	2
3072	Nucleoporin 54kDa variant (Fragment)	IPI00172580	-0.837	0.03780	6	3	2	2
3073	ANKHD1-EIF4EBP3 protein	IPI00217442	-0.837	0.03780	7	2	0	0
3074	Isoform 1 of Fanconi anemia group D2 protein	IPI00075081	-0.837	0.03780	6	3	0	0
3075	sideroflexin-3	IPI00793874	-0.837	0.03780	5	4	2	0
3076	Paladin	IPI00297212	-0.846	0.03689	8	7	4	5
3077	Isoform 1 of Protein strawberry notch homolog 1	IPI00023649	-0.846	0.03689	7	8	6	3
3078	Dipeptidyl peptidase 1	IPI00022810	-0.853	0.03655	12	12	9	8
3079	Nuclease-sensitive element-binding protein 1	IPI00031812	-0.865	0.03636	10	13	14	2
3080	28S ribosomal protein S23, mitochondrial	IPI00032881	-0.867	0.03610	7	7	5	3
3081	Isoform 1 of Transcription factor BTF3	IPI00221035	-0.872	0.03474	18	17	13	14
3082	NAD(P) transhydrogenase, mitochondrial	IPI00337541	-0.876	0.03470	32	20	17	26
3083	Isoform 1 of ATP-binding cassette sub-family B member 7, mitochondrial	IPI00306748	-0.877	0.03470	13	9	11	4
3084	Eukaryotic translation initiation factor 3 subunit C	IPI00016910	-0.880	0.03470	17	17	14	12
3085	Isoform 1 of UDP-glucose:glycoprotein glucosyltransferase 1	IPI00024466	-0.886	0.03436	26	24	19	22
3086	Isoform 5 of Protein polybromo-1	IPI00023097	-0.890	0.03432	5	8	5	2
3087	Isoform 1 of Interferon-inducible double stranded RNA-dependent protein kinase activator A	IPI00021167	-0.890	0.03432	7	6	3	4
3088	Ras suppressor protein 1	IPI00017256	-0.890	0.03432	5	8	5	2
3089	Dolichol-phosphate mannosyltransferase	IPI00022018	-0.890	0.03432	5	8	2	5
3090	Cell growth-regulating nucleolar protein	IPI00015838	-0.890	0.03432	6	7	4	3
3091	Isoform 1 of Wings apart-like protein homolog	IPI00375330	-0.890	0.03432	8	5	4	3
3092	Succinyl-CoA ligase [GDP-forming] subunit beta, mitochondrial	IPI00096066	-0.890	0.03432	7	6	4	3
3093	ERO1-like protein alpha	IPI00386755	-0.891	0.03432	12	9	8	6
3094	Aldehyde dehydrogenase family 1 member A3	IPI00026663	-0.891	0.03432	10	11	6	8
3095	NADH dehydrogenase [ubiquinone] iron-sulfur protein 2, mitochondrial	IPI00025239	-0.891	0.03432	9	12	7	7
3096	Interferon-induced, double-stranded RNA-activated protein kinase	IPI00019463	-0.891	0.03432	10	11	6	8
3097	Sodium/potassium-transporting ATPase subunit alpha-2	IPI00003021	-0.897	0.03425	18	14	9	15
3098	Ran GTPase-activating protein 1	IPI00294879	-0.905	0.03402	20	11	10	13
3099	Isoform Long of Trifunctional purine biosynthetic protein adenosine-3	IPI00025273	-0.914	0.03379	24	21	17	19
3100	Isoform 1 of Myb-binding protein 1A	IPI00005024	-0.915	0.03379	15	15	10	12
3101	Isoform 3 of Serine/threonine-protein kinase SMG1	IPI00183368	-0.918	0.03345	5	7	2	4
3102	Tubulin-folding cofactor B	IPI00293126	-0.918	0.03345	7	5	4	0
3103	Cell division protein kinase 5	IPI00023530	-0.918	0.03345	4	8	2	4
3104	Isoform 1 of Neurochondrin	IPI00549543	-0.918	0.03345	6	6	3	3
3105	Importin-9	IPI00185146	-0.921	0.03168	11	8	4	8
3106	Isoform Beta of Heat shock protein 105 kDa	IPI00218993	-0.931	0.03156	30	31	20	31
3107	Isoform 1 of Eukaryotic translation initiation factor 3 subunit B	IPI00396370	-0.940	0.03130	31	28	25	24
3108	Palmitoyl-protein thioesterase 1	IPI00002412	-0.950	0.03111	6	5	3	2
3109	Isoform 1 of Glomulin	IPI00074604	-0.950	0.03111	5	6	2	3
3110	WD repeat-containing protein 11	IPI00412224	-0.950	0.03111	4	7	0	3
3111	Isoform DPI of Desmoplakin	IPI00013933	-0.967	0.03016	38	37	26	38
3112	cDNA FLJ54957, highly similar to Transketolase	IPI00643920	-0.968	0.03016	46	56	40	50
3113	Histone deacetylase 1	IPI00013774	-0.969	0.03013	12	13	8	9
3114	Isoform 1 of Cirhin	IPI00239815	-0.978	0.03001	8	8	2	7
3115	Ribosome biogenesis protein BMS1 homolog	IPI00006099	-0.978	0.03001	7	9	3	6
3116	Calmodulin	IPI00075248	-0.978	0.03001	8	8	4	5

No.	Description	Accession number	STN[1]	p-Value[1]	Con_A[2]	Con_B[2]	SORA_A[2]	SORA_B[2]
3117	Nucleolar GTP-binding protein 1	IPI00385042	-0.982	0.03001	13	11	7	9
3118	Putative uncharacterized protein SPTAN1	IPI00745092	-0.982	0.03001	15	9	7	9
3119	Keratin, type I cytoskeletal 14	IPI00384444	-0.989	0.02918	3	7	0	2
3120	Kinesin-like protein KIF11	IPI00305289	-0.989	0.02918	6	4	0	2
3121	cDNA: FLJ22728 fis, clone HSI15617 (Fragment)	IPI00386139	-0.989	0.02918	7	3	0	2
3122	Isoform 1 of Probable DNA dC->dU-editing enzyme APOBEC-3B	IPI00005531	-0.989	0.02918	6	4	0	0
3123	Core histone macro-H2A.2	IPI00220994	-0.989	0.02918	6	4	2	2
3124	Transmembrane protein 14C	IPI00009346	-0.989	0.02918	6	4	0	0
3125	Apolipoprotein O-like	IPI00394809	-0.989	0.02918	6	4	2	2
3126	Conserved hypothetical protein	IPI00477526	-0.989	0.02918	5	5	0	0
3127	Isoform 1 of Telomere-associated protein RIF1	IPI00293845	-0.996	0.02601	11	12	8	7
3128	Tyrosyl-tRNA synthetase, mitochondrial	IPI00165092	-0.996	0.02601	11	12	9	6
3129	Thymidylate synthetase, isoform CRA_a	IPI00103732	-1.001	0.02585	7	8	3	5
3130	Probable ATP-dependent RNA helicase DDX6	IPI00030320	-1.004	0.02582	17	16	11	13
3131	Proteasome subunit beta type-4	IPI00555956	-1.004	0.02582	20	13	14	10
3132	Isoform 1 of Caprin-1	IPI00783872	-1.011	0.02574	15	7	7	7
3133	Coatomer subunit zeta-1	IPI00032851	-1.011	0.02574	11	11	6	8
3134	Isoform 1 of Polypyrimidine tract-binding protein 1	IPI00179964	-1.012	0.02487	48	68	50	53
3135	Phospholipase A-2-activating protein	IPI00218465	-1.014	0.02487	16	16	10	13
3136	Stress-induced-phosphoprotein 1	IPI00013894	-1.017	0.02480	30	33	28	24
3137	Bifunctional ATP-dependent dihydroxyacetone kinase/FAD-AMP lyase (cyclizing)	IPI00551024	-1.024	0.02468	15	16	11	11
3138	Probable methylthioribulose-1-phosphate dehydratase	IPI00549730	-1.028	0.02434	5	9	2	5
3139	SNW domain-containing protein 1	IPI00013830	-1.028	0.02434	8	6	4	3
3140	Acidic leucine-rich nuclear phosphoprotein 32 family member E	IPI00165393	-1.028	0.02434	8	6	2	5
3141	Ras-related protein Rab-18	IPI00014577	-1.028	0.02434	5	9	3	4
3142	ubiquitin and ribosomal protein S27a precursor	IPI00179330	-1.041	0.02393	37	42	30	37
3143	Elongation factor 1-beta	IPI00178440	-1.042	0.02393	17	41	21	26
3144	Glyoxylate reductase/hydroxypyruvate reductase	IPI00037448	-1.045	0.02381	9	11	8	4
3145	ATP-citrate synthase	IPI00021290	-1.048	0.02294	30	27	22	24
3146	Glutathione S-transferase kappa 1	IPI00219673	-1.056	0.02279	25	15	17	13
3147	Heat shock protein beta-1	IPI00025512	-1.058	0.02257	7	6	3	3
3148	Isoform 1 of Symplekin	IPI00023344	-1.064	0.02257	12	7	2	9
3149	Isoform 1 of DNA primase large subunit	IPI00027705	-1.064	0.02257	9	10	4	7
3150	Valyl-tRNA synthetase	IPI00000873	-1.071	0.02249	26	27	22	20
3151	Intron-binding protein aquarius	IPI00297572	-1.071	0.02249	14	13	7	11
3152	Vigilin	IPI00022228	-1.082	0.02226	20	17	12	15
3153	Serine/threonine-protein kinase mTOR	IPI00031410	-1.085	0.02223	13	13	6	11
3154	von Hippel-Lindau binding protein 1, isoform CRA_b	IPI00334159	-1.085	0.02223	11	15	7	10
3155	Isoform 1 of Squamous cell carcinoma antigen recognized by T-cells 3	IPI00006025	-1.085	0.02223	10	8	6	4
3156	Probable cysteinyl-tRNA synthetase, mitochondrial	IPI00336016	-1.085	0.02223	11	7	4	6
3157	40S ribosomal protein S3a	IPI00419880	-1.089	0.02124	27	23	19	20
3158	26S proteasome non-ATPase regulatory subunit 6	IPI00014151	-1.091	0.02124	21	15	13	13
3159	mRNA export factor	IPI00019733	-1.094	0.02105	6	6	0	3
3160	Isoform 1 of Fanconi anemia group I protein	IPI00019447	-1.101	0.02094	22	13	13	12
3161	Talin-1	IPI00298994	-1.104	0.02086	73	70	63	65
3162	Isoform 3 of Probable ATP-dependent RNA helicase DDX17	IPI00651653	-1.108	0.02068	9	8	4	5
3163	Isoform 1 of Fragile X mental retardation syndrome-related protein 1	IPI00016249	-1.114	0.02049	13	11	7	8
3164	Isoform Long of Double-stranded RNA-binding protein Staufen homolog 1	IPI00000001	-1.114	0.02049	12	12	6	9
3165	Heme oxygenase 2	IPI00026824	-1.134	0.02011	9	7	4	4
3166	Isoform 1 of Rab3 GTPase-activating protein non-catalytic subunit	IPI00554590	-1.134	0.02011	10	6	3	5
3167	Acyl-CoA dehydrogenase family member 9, mitochondrial	IPI00152981	-1.134	0.02011	9	7	3	5
3168	Isoform 2 of Vacuolar protein sorting-associated protein 13A	IPI00478586	-1.138	0.01966	6	5	0	2
3169	Isoform 1 of Histone-arginine methyltransferase CARM1	IPI00412880	-1.138	0.01966	5	6	0	2
3170	Isoform 2 of Transcription factor p65	IPI00219084	-1.138	0.01966	5	6	2	0
3171	MAGUK p55 subfamily member 6	IPI00303280	-1.138	0.01966	5	6	0	0
3172	ribonucleotide reductase M2 polypeptide isoform 1	IPI00011118	-1.138	0.01966	8	3	0	0
3173	Microtubule-associated protein RP/EB family member 1	IPI00017596	-1.145	0.01958	12	19	12	9
3174	Proteasome subunit beta type-2	IPI00028006	-1.145	0.01958	12	19	9	12
3175	Thioredoxin-dependent peroxide reductase, mitochondrial	IPI00024919	-1.152	0.01958	23	33	21	23
3176	Protein phosphatase 1G	IPI00006167	-1.157	0.01947	15	15	10	10
3177	Keratin, type I cytoskeletal 16	IPI00217963	-1.163	0.01916	8	7	3	4
3178	Serine/threonine-protein phosphatase 2A catalytic subunit alpha isoform	IPI00008380	-1.163	0.01916	6	9	2	5
3179	X-ray repair cross-complementing protein 6	IPI00644712	-1.165	0.01916	75	74	63	70
3180	Serine/threonine-protein kinase PAK 2	IPI00419979	-1.170	0.01909	15	14	10	9
3181	Aspartyl-tRNA synthetase, cytoplasmic	IPI00216951	-1.171	0.01909	34	36	32	25
3182	Isoform 2 of Microtubule-actin cross-linking factor 1, isoforms 1/2/3/5	IPI00256861	-1.188	0.01879	11	9	4	7
3183	Proline synthetase co-transcribed homolog (Bacterial), isoform CRA_b	IPI00016346	-1.188	0.01879	10	10	6	5
3184	Tumor protein, translationally-controlled 1	IPI00009943	-1.188	0.01879	10	10	5	6
3185	Alanyl-tRNA synthetase, cytoplasmic	IPI00027442	-1.190	0.01879	44	42	31	41
3186	Ribonucleoside-diphosphate reductase large subunit	IPI00013871	-1.196	0.01867	17	20	12	14
3187	40S ribosomal protein S20	IPI00012493	-1.196	0.01867	6	8	4	0
3188	Isoform 1 of Cytosolic acyl coenzyme A thioester hydrolase	IPI00010415	-1.199	0.01777	15	12	6	11
3189	Eukaryotic initiation factor 4A-III	IPI00009328	-1.200	0.01777	22	27	20	17
3190	cDNA FLJ54492, highly similar to Eukaryotic translation initiation factor 4B	IPI00012079	-1.207	0.01777	19	17	13	12
3191	Transferrin receptor protein 1	IPI00022462	-1.218	0.01727	14	21	6	18
3192	60S ribosomal protein L10a	IPI00412579	-1.229	0.01716	40	37	35	28
3193	DEAD (Asp-Glu-Ala-Asp) box polypeptide 39 transcript variant	IPI00062206	-1.231	0.01705	13	12	9	6
3194	Isoform 4 of Heterogeneous nuclear ribonucleoprotein A/B	IPI00106509	-1.231	0.01705	14	11	9	6
3195	Epoxide hydrolase 1	IPI00009896	-1.236	0.01705	5	8	3	2
3196	Retinoblastoma-associated protein	IPI00302829	-1.236	0.01705	6	7	3	2
3197	1-acyl-sn-glycerol-3-phosphate acyltransferase epsilon	IPI00028491	-1.236	0.01705	7	6	3	2
3198	Isoform 2 of Ubiquitin-associated domain-containing protein 2	IPI00007034	-1.236	0.01705	6	7	2	3
3199	Isoform Long of Deoxyhypusine synthase	IPI00026829	-1.236	0.01705	9	9	4	5
3200	Destrin	IPI00473014	-1.239	0.01705	25	33	22	23
3201	Isoform 2 of U5 small nuclear ribonucleoprotein 200 kDa helicase	IPI00168235	-1.248	0.01690	37	36	26	33
3202	Interleukin enhancer-binding factor 2	IPI00005198	-1.269	0.01629	25	44	23	32
3203	Isoform 1 of Apoptotic chromatin condensation inducer in the nucleus	IPI00007334	-1.283	0.01618	8	4	2	2
3204	Tubulin gamma-1 chain	IPI00295081	-1.296	0.01467	9	7	2	5
3205	Isoform 1 of DNA replication licensing factor MCM7	IPI00299904	-1.311	0.01451	12	9	5	6
3206	Isoform 2 of Isochorismatase domain-containing protein 2, mitochondrial	IPI00003031	-1.311	0.01451	12	9	5	6
3207	Transgelin-2	IPI00550363	-1.312	0.01451	14	14	8	9
3208	Isoform 1 of Deoxyuridine 5'-triphosphate nucleotidohydrolase, mitochondrial	IPI00013679	-1.323	0.01448	15	21	9	15
3209	regulator of differentiation 1 isoform 2	IPI00159072	-1.332	0.01436	6	9	2	4
3210	zinc finger protein 294	IPI00783835	-1.332	0.01436	5	10	2	4
3211	Isoform 1 of Mitochondrial inner membrane protein	IPI00009960	-1.335	0.01433	19	16	11	12

No.	Description	Accession number	STN[1]	p-Value[1]	Con_A[2]	Con_B[2]	SORA_A[2]	SORA_B[2]
3212	COP9 signalosome complex subunit 4	IPI00171844	-1.336	0.01433	10	10	4	6
3213	Proteasome subunit beta type-1	IPI00025019	-1.340	0.01406	22	23	13	19
3214	Splicing factor 3B subunit 1	IPI00026089	-1.345	0.01399	30	27	21	22
3215	Isoform 2 of Eukaryotic translation initiation factor 5A-1	IPI00376005	-1.346	0.01399	48	42	38	36
3216	Isoform 1 of Splicing factor, arginine/serine-rich 7	IPI00003377	-1.346	0.01399	12	14	8	7
3217	Eukaryotic translation initiation factor 3 subunit E	IPI00013068	-1.349	0.01395	17	27	12	19
3218	cDNA FLJ59211, highly similar to Glucosidase 2 subunit beta	IPI00026154	-1.362	0.01395	17	16	8	13
3219	Dolichyl-diphosphooligosaccharide--protein glycosyltransferase 48 kDa subunit	IPI00297084	-1.362	0.01395	16	17	12	9
3220	Translational activator GCN1	IPI00001159	-1.367	0.01395	85	72	58	80
3221	Guanine nucleotide-binding protein subunit beta-2-like 1	IPI00848226	-1.374	0.01383	31	53	25	43
3222	Nuclear pore complex protein Nup133	IPI00291200	-1.375	0.01383	7	7	3	2
3223	Condensin complex subunit 3	IPI00106495	-1.376	0.01383	17	15	11	9
3224	Isoform 1 of Clathrin heavy chain 1	IPI00024067	-1.378	0.01380	159	146	135	147
3225	Isoform 2 of Signal recognition particle 68 kDa protein	IPI00102936	-1.380	0.01368	23	18	15	13
3226	Eukaryotic initiation factor 4A-I	IPI00025491	-1.383	0.01365	108	108	92	103
3227	CTP synthase 1	IPI00290142	-1.384	0.01365	25	27	17	21
3228	Keratin, type I cytoskeletal 9	IPI00019359	-1.390	0.01361	80	68	56	73
3229	sister chromatid cohesion protein PDS5 homolog A isoform 2	IPI00303063	-1.391	0.01361	19	12	11	8
3230	GTP-binding protein SAR1a	IPI00015954	-1.393	0.01361	8	10	3	5
3231	Isoform 2 of Neutral alpha-glucosidase AB	IPI00011454	-1.401	0.01304	77	67	59	66
3232	Monocarboxylate transporter 1	IPI00024650	-1.407	0.01293	17	13	11	7
3233	Protein RRP5 homolog	IPI00400922	-1.429	0.01259	25	22	14	19
3234	Putative heat shock protein HSP 90-alpha A2	IPI00031523	-1.440	0.01232	115	145	93	144
3235	Isoform 1 of Nuclear pore complex protein Nup160	IPI00748807	-1.449	0.01210	21	24	12	19
3236	Isoform 1 of 2-oxoglutarate and iron-dependent oxygenase domain-containing protein 1	IPI00170429	-1.466	0.01210	9	7	4	0
3237	Protein tyrosine phosphatase-like protein PTPLAD1	IPI00008998	-1.484	0.01126	14	19	9	11
3238	Putative uncharacterized protein RPL17	IPI00394699	-1.488	0.01126	13	7	3	6
3239	Solute carrier family 4 sodium bicarbonate cotransporter member 7	IPI00021058	-1.500	0.01126	21	11	9	10
3240	Isoform 1 of Nuclear pore membrane glycoprotein 210	IPI00291755	-1.503	0.01126	12	13	5	8
3241	Ubiquitin carboxyl-terminal hydrolase 11	IPI00184533	-1.520	0.01100	12	7	6	2
3242	Coatomer subunit beta'	IPI00220219	-1.530	0.01092	28	31	20	23
3243	Isoform 3 of DNA topoisomerase 2-alpha	IPI00218753	-1.535	0.01073	16	14	8	9
3244	Isoform 2 of DNA replication licensing factor MCM7	IPI00219740	-1.535	0.01073	16	14	10	7
3245	vacuolar protein sorting-associated protein 13C isoform 2B	IPI00412216	-1.556	0.01062	11	7	0	5
3246	Isoform 2 of S-phase kinase-associated protein 1	IPI00172421	-1.561	0.01058	16	20	9	13
3247	PRMT3 protein (Fragment)	IPI00103026	-1.567	0.01055	8	6	2	2
3248	Histone H1.5	IPI00217468	-1.570	0.00968	13	31	15	14
3249	Dolichyl-diphosphooligosaccharide--protein glycosyltransferase subunit 2	IPI00028635	-1.572	0.00968	56	60	40	56
3250	Ras-related protein Rab-11B	IPI00020436	-1.573	0.00964	25	29	17	21
3251	Isoform 1 of Transformation/transcription domain-associated protein	IPI00069084	-1.576	0.00960	18	17	12	9
3252	Peptidyl-prolyl cis-trans isomerase B	IPI00646304	-1.581	0.00941	29	36	24	24
3253	epiplakin	IPI00010951	-1.592	0.00937	19	15	9	11
3254	baculoviral IAP repeat-containing protein 6	IPI00299635	-1.596	0.00926	13	14	7	7
3255	Isoform 1 of Spectrin beta chain, brain 2	IPI00012645	-1.598	0.00926	10	7	3	3
3256	WD repeat-containing protein 75	IPI00217240	-1.598	0.00926	10	7	2	4
3257	Isoform A of Lamin-A/C	IPI00021405	-1.601	0.00926	54	55	44	45
3258	Isoform 1 of DNA (cytosine-5)-methyltransferase 1	IPI00031519	-1.627	0.00915	15	17	7	11
3259	Isoform 1 of Serine/arginine repetitive matrix protein 2	IPI00782992	-1.640	0.00884	36	34	22	30
3260	Isoform 1 of Cleavage stimulation factor subunit 2	IPI00013256	-1.646	0.00881	10	10	6	2
3261	Isoform 1 of Splicing factor 3B subunit 3	IPI00300371	-1.652	0.00873	73	87	72	65
3262	Nuclear pore complex protein Nup205	IPI00783781	-1.655	0.00873	40	28	20	30
3263	Isoform 2 of Nucleoporin NUP188 homolog	IPI00385001	-1.701	0.00839	17	6	5	5
3264	60S ribosomal protein L3	IPI00550021	-1.728	0.00813	31	40	26	26
3265	Isoform 2 of Nucleosome-remodeling factor subunit BPTF	IPI00254408	-1.728	0.00813	9	9	2	4
3266	cDNA FLJ34068 fis, clone FCBBF3001918	IPI00168184	-1.731	0.00805	27	22	15	17
3267	Isoform A of Phosphate carrier protein, mitochondrial	IPI00022202	-1.736	0.00805	34	36	28	23
3268	T-complex protein 1 subunit gamma isoform b	IPI00290770	-1.765	0.00798	43	50	34	38
3269	WD repeat-containing protein 3	IPI00009471	-1.780	0.00779	10	7	0	3
3270	Ras-related protein Rap-1b	IPI00015148	-1.782	0.00779	23	22	16	12
3271	Leucine-rich PPR motif-containing protein, mitochondrial	IPI00783271	-1.793	0.00771	109	104	87	99
3272	Short heat shock protein 60 Hsp60s2	IPI00076042	-1.833	0.00745	21	38	24	16
3273	Kinetochore-associated protein 1	IPI00001458	-1.841	0.00737	9	7	2	0
3274	Isoform 1 of 5'(3')-deoxyribonucleotidase, cytosolic type	IPI00005573	-1.855	0.00714	11	12	4	5
3275	Isoform 1 of Chromodomain-helicase-DNA-binding protein 4	IPI00000846	-1.926	0.00658	41	41	27	33
3276	Isoform 1 of Ras-related protein Rab-1A	IPI00005719	-1.947	0.00643	45	47	37	32
3277	Heterogeneous nuclear ribonucleoprotein F	IPI00003881	-1.956	0.00643	33	34	19	27
3278	DnaJ homolog subfamily A member 1	IPI00012535	-1.972	0.00635	16	18	7	10
3279	Isoform 1 of ATPase family AAA domain-containing protein 2	IPI00170548	-1.984	0.00616	12	8	3	3
3280	Heterogeneous nuclear ribonucleoprotein L	IPI00027834	-1.990	0.00616	35	39	27	25
3281	Isoform 1 of Acetyl-CoA carboxylase 1	IPI00011569	-2.305	0.00416	31	24	11	21
3282	Isoform 1 of L-lactate dehydrogenase A chain	IPI00217966	-2.342	0.00397	170	202	163	168
3283	Midasin	IPI00167941	-2.399	0.00389	30	26	10	22
3284	Isoform 1 of Lipopolysaccharide-responsive and beige-like anchor protein	IPI00002255	-2.399	0.00389	34	22	15	17
3285	ATP-dependent RNA helicase A	IPI00844578	-2.430	0.00389	111	95	74	96
3286	Isoform 1 of Heat shock cognate 71 kDa protein	IPI00003865	-2.451	0.00386	120	145	111	115
3287	protein ELYS	IPI00170594	-2.490	0.00378	11	16	6	2
3288	Isoform Long of Antigen KI-67	IPI00004233	-2.601	0.00355	12	13	4	0
3289	T-complex protein 1 subunit beta	IPI00297779	-2.610	0.00355	83	95	67	74
3290	Heat shock 70 kDa protein 1A/1B	IPI00304925	-2.623	0.00352	40	38	20	29
3291	Glutathione S-transferase P	IPI00219757	-2.685	0.00340	112	119	95	95
3292	Isoform 5 of E3 ubiquitin-protein ligase UBR4	IPI00180305	-2.706	0.00340	33	31	13	23
3293	CAD protein	IPI00301263	-2.864	0.00310	90	81	66	65
3294	ADP/ATP translocase 2	IPI00007188	-2.967	0.00306	127	180	95	163
3295	TOB3	IPI00045921	-3.009	0.00299	17	16	4	5
3296	60S ribosomal protein L18	IPI00215719	-3.104	0.00272	24	36	9	20
3297	Carbonic anhydrase 1	IPI00215983	-3.122	0.00268	24	0	0	0
3298	Glyceraldehyde-3-phosphate dehydrogenase	IPI00219018	-3.498	0.00208	342	411	300	379
3299	Keratin, type I cytoskeletal 18	IPI00554788	-3.509	0.00204	421	567	378	530
3300	Keratin, type II cytoskeletal 2 epiderma	IPI00021304	-3.565	0.00204	105	99	76	76
3301	Elongation factor 1-alpha 2	IPI00014424	-4.036	0.00140	554	607	517	548
3302	Fatty acid synthase	IPI00026781	-4.172	0.00136	214	233	180	191
3303	Keratin, type I cytoskeletal 10	IPI00009865	-4.214	0.00129	147	147	96	130
3304	Isoform 1 of U5 small nuclear ribonucleoprotein 200 kDa helicase	IPI00420014	-4.405	0.00121	114	105	66	88
3305	Pre-mRNA-processing-splicing factor 8	IPI00007928	-4.567	0.00110	122	105	70	89
3306	Keratin, type II cytoskeletal 1	IPI00220327	-7.077	0.00042	350	430	279	352
