# Supplementary material for: Data supporting the identification of anti-metastatic drug and natural compound targets in isogenic colorectal cancer cells
Source: Data Brief. 2014 Nov 4;1:73–5. doi: 10.1016/j.dib.2014.10.005 (PMC4459770; doi:10.1016/j.dib.2014.10.005)
Supplement: Supplementary file 1 — Supplementary data [file mmc1.zip › CRC_Metastasis_DIB_Table04.pdf]

Table 4. List of proteins differentially expressed in SW620 by the treatment of 5-fluorouracil for 48hr. ([1]STN and p-value were acquired from PLGEM analysis, [2]Raw spectral counts from data compilation using ScaffoldTM software)

No.	Description	Accession number	STN[1]	p-Value[1]	Con_A[2]	Con_B[2]	5FU_A[2]	5FU_B[2]
1	Elongation factor 1-alpha 2	IPI00014424	7.927	0.00004	309	230	368	415
2	Cytoplasmic dynein 1 heavy chain 1	IPI00456969	5.255	0.00034	212	196	259	295
3	Isoform 1 of Myosin-9	IPI00019502	3.780	0.00078	206	169	240	236
4	Isoform 1 of DNA-dependent protein kinase catalytic subunit	IPI00296337	3.455	0.00115	159	129	184	189
5	Isoform 1 of Filamin-B	IPI00289334	3.096	0.00149	99	91	135	122
6	Neuroblast differentiation-associated protein AHNAK	IPI00021812	3.082	0.00153	68	80	104	106
7	Glyceraldehyde-3-phosphate dehydrogenase	IPI00219018	3.014	0.00160	231	115	230	194
8	Isoform 1 of Plectin-1	IPI00014898	2.936	0.00168	243	238	289	276
9	Isoform M1 of Pyruvate kinase isozymes M1/M2	IPI00220644	2.923	0.00168	171	98	179	160
10	Isoform Long of Spectrin beta chain, brain 1	IPI00005614	2.785	0.00190	57	52	77	83
11	Pre-mRNA-processing-splicing factor 8	IPI00007928	2.663	0.00223	95	71	102	119
12	Talin-1	IPI00298994	2.645	0.00231	59	33	73	65
13	Isoform 1 of Myosin-10	IPI00397526	2.512	0.00246	41	40	60	63
14	Isoform 3 of Spectrin alpha chain, brain	IPI00843765	2.437	0.00261	64	51	79	81
15	482 kDa protein	IPI00179298	2.347	0.00279	53	51	68	78
16	Isoform 1 of Clathrin heavy chain 1	IPI00024067	2.299	0.00294	124	105	134	147
17	ADP/ATP translocase 2	IPI00007188	2.298	0.00294	128	87	125	141
18	Isoform B1 of Heterogeneous nuclear ribonucleoproteins A2/B1	IPI00396378	2.140	0.00365	121	74	132	109
19	Isoform 1 of ATP-binding cassette sub-family D member 3	IPI00002372	2.138	0.00365	5	2	11	15
20	Isoform Long of Antigen KI-67	IPI00004233	2.115	0.00372	7	10	17	23
21	Keratin, type II cytoskeletal 2 epiderma	IPI00021304	2.010	0.00417	37	27	47	48
22	EH domain-containing protein 1	IPI00017184	1.959	0.00436	6	5	15	15
23	cDNA FLJ54957, highly similar to Transketolase	IPI00643920	1.940	0.00454	30	21	38	41
24	Non-POU domain-containing octamer-binding protein	IPI00304596	1.856	0.00503	39	29	47	50
25	Actin, aortic smooth muscle	IPI00008603	1.841	0.00521	31	39	54	45
26	DNA-directed RNA polymerase II subunit RPB1	IPI00031627	1.841	0.00521	13	13	23	25
27	Isoform 3 of DNA topoisomerase 2-alpha	IPI00218753	1.817	0.00562	13	10	21	23
28	Galectin-1	IPI00219219	1.768	0.00603	24	18	38	28
29	59 kDa protein	IPI00302925	1.756	0.00603	34	23	45	38
30	Isocitrate dehydrogenase 3, beta subunit isoform a precursor	IPI00304417	1.755	0.00603	7	0	12	13
31	Elongation factor 1-alpha	IPI00025447	1.738	0.00618	91	70	98	98
32	Protein disulfide-isomerase A3	IPI00025252	1.687	0.00655	29	21	39	35
33	Isoform 1 of Chromodomain-helicase-DNA-binding protein 4	IPI00000846	1.675	0.00659	42	35	50	54
34	Putative uncharacterized protein DKFZp686L20222	IPI00026689	1.668	0.00659	25	20	32	36
35	Kinesin-like protein KIF11	IPI00305289	1.658	0.00663	5	4	11	13
36	Isoform 2 of Nuclear mitotic apparatus protein 1	IPI00006196	1.652	0.00663	27	27	36	42
37	Ras GTPase-activating-like protein IQGAP1	IPI00009342	1.629	0.00685	63	45	70	67
38	Vimentin	IPI00418471	1.612	0.00693	37	31	48	45
39	ATP-dependent RNA helicase A	IPI00844578	1.598	0.00711	90	86	108	101
40	Vesicle transport protein GOT1B	IPI00007061	1.597	0.00715	2	0	8	8
41	Putative annexin A2-like protein	IPI00334627	1.564	0.00786	28	29	43	37
42	T-complex protein 1 subunit gamma isoform b	IPI00290770	1.554	0.00801	27	23	34	38
43	Isoform 1 of N-acylneuraminate cytidylyltransferase	IPI00303158	1.533	0.00823	3	2	8	9
44	Eukaryotic initiation factor 4A-I	IPI00025491	1.501	0.00856	75	52	81	74
45	Isoform 2 of Filamin-A	IPI00302592	1.500	0.00860	146	141	164	159
46	Phosphoglycerate kinase 1	IPI00169383	1.496	0.00860	70	44	72	69
47	Aladin	IPI00024143	1.483	0.00868	2	2	6	9
48	Tumor protein, translationally-controlled 1	IPI00009943	1.465	0.00916	8	12	18	18
49	Isoform A of Lamin-A/C	IPI00021405	1.450	0.00946	42	44	57	53
50	Thioredoxin domain-containing protein 17	IPI00646689	1.427	0.00961	4	6	11	12
51	Moesin	IPI00219365	1.405	0.00983	25	11	27	27
52	Isoform 1 of Transcription elongation factor SPT5	IPI00298058	1.390	0.01028	8	7	12	17
53	Peroxiredoxin-1	IPI00000874	1.382	0.01035	40	36	52	46
54	Proteasome subunit alpha type-5	IPI00291922	1.352	0.01139	14	20	28	23
55	Isoform 1 of Reticulon-4	IPI00021766	1.328	0.01177	8	2	13	9
56	Isoform 1 of Pyruvate dehydrogenase E1 component subunit beta, mitochondrial	IPI00003925	1.321	0.01225	17	13	21	25
57	Nuclear pore complex protein Nup205	IPI00783781	1.307	0.01232	27	20	33	32
58	rRNA 2'-O-methyltransferase fibrillarin	IPI00025039	1.299	0.01240	11	4	14	14
59	Isoform SERCA1B of Sarcoplasmic/endoplasmic reticulum calcium ATPase 1	IPI00024804	1.291	0.01270	6	0	9	10
60	T-complex protein 1 subunit beta	IPI00297779	1.291	0.01270	48	22	52	38
61	Vacuolar protein sorting-associated protein 26A	IPI00411426	1.279	0.01270	10	11	17	18
62	Serpin B6	IPI00413451	1.279	0.01270	8	8	16	13
63	Putative uncharacterized protein SPTAN1	IPI00745092	1.266	0.01311	16	12	18	25
64	Nucleolar protein 9	IPI00002902	1.248	0.01355	2	2	7	6
65	26S proteasome non-ATPase regulatory subunit 3	IPI00011603	1.239	0.01359	19	19	30	24
66	Very long-chain acyl-CoA synthetase	IPI00024787	1.228	0.01381	7	7	10	16
67	Fructose-bisphosphate aldolase A	IPI00465439	1.221	0.01400	19	13	28	19
68	Histone H4	IPI00453473	1.214	0.01400	81	97	93	110
69	Aspartate aminotransferase, mitochondrial	IPI00018206	1.214	0.01400	38	23	40	39
70	Leukocyte elastase inhibitor	IPI00027444	1.210	0.01404	14	12	19	21
71	Translational activator GCN1	IPI00001159	1.195	0.01430	69	38	61	67
72	Protein disulfide-isomerase	IPI00010796	1.189	0.01441	22	22	33	27
73	Glyoxylate reductase/hydroxypyruvate reductase	IPI00037448	1.188	0.01445	10	6	15	13
74	Isoform 2 of Extended synaptotagmin-2	IPI00409635	1.180	0.01527	9	13	18	17
75	60S ribosomal protein L21	IPI00247583	1.166	0.01527	13	10	15	21
76	Isoform 3 of Protein transport protein Sec31A	IPI00305152	1.154	0.01530	4	5	9	10
77	Nestin	IPI00010800	1.154	0.01530	15	16	21	24
78	Isoform 3 of Glutaminase kidney isoform, mitochondrial	IPI00215687	1.153	0.01538	10	8	15	15
79	T-complex protein 1 subunit alpha	IPI00290566	1.152	0.01564	43	30	46	45
80	Isoform 1 of OCIA domain-containing protein 1	IPI00016405	1.149	0.01564	2	4	8	7
81	Ubiquitin carboxyl-terminal hydrolase 7	IPI00003965	1.140	0.01571	13	12	17	21
82	Nucleoprotein TPR	IPI00742682	1.138	0.01571	30	33	40	40
83	Ras-related protein Rab-10	IPI00016513	1.133	0.01583	7	7	12	13
84	retinol-binding protein 1 isoform a	IPI00219718	1.133	0.01583	37	27	40	41
85	Coatomer subunit zeta-1	IPI00032851	1.126	0.01583	6	4	10	10
86	Myosin-Id	IPI00329719	1.126	0.01612	2	2	4	8
87	Actin, cytoplasmic 1	IPI00021439	1.124	0.01787	89	63	95	79
88	protein arginine N-methyltransferase 5 isoform b	IPI00064328	1.122	0.01787	12	8	16	16
89	annexin A4	IPI00793199	1.118	0.01817	25	19	28	31
90	Isoform F of Protein SON	IPI00000192	1.111	0.01825	2	5	9	7
91	Isoform SCPx of Non-specific lipid-transfer protein	IPI00026105	1.111	0.01825	3	4	7	9

No.	Description	Accession number	STN[1]	p-Value[1]	Con_A[2]	Con_B[2]	5FU_A[2]	5FU_B[2]
92	COP9 signalosome complex subunit 5	IPI00009958	1.111	0.01825	4	3	9	7
93	Isoform 3 of Ribosome-binding protein 1	IPI00215743	1.094	0.01839	14	8	20	14
94	T-complex protein 1 subunit delta	IPI00302927	1.091	0.01862	28	20	33	30
95	Isoform 1 of Nuclear pore complex protein Nup160	IPI00748807	1.079	0.01877	10	7	12	16
96	Splicing factor, arginine/serine-rich 9	IPI00012340	1.079	0.01877	8	9	11	17
97	Isoform 1 of Putative helicase MOV-10	IPI00444452	1.078	0.01884	7	5	9	13
98	ATP synthase subunit g, mitochondrial	IPI00027448	1.076	0.01906	2	3	5	8
99	Isoform 1 of Sodium-coupled neutral amino acid transporter 2	IPI00410034	1.076	0.01906	3	2	5	8
100	Putative uncharacterized protein NOP2	IPI00294891	1.063	0.01940	12	6	16	13
101	F-actin-capping protein subunit alpha-1	IPI00005969	1.058	0.01947	16	9	24	13
102	Isoform 2 of Triosephosphate isomerase	IPI00451401	1.055	0.01959	27	27	39	30
103	Isoform SERCA2A of Sarcoplasmic/endoplasmic reticulum calcium ATPase 2	IPI00177817	1.048	0.01959	8	11	14	16
104	Isoform 1 of 6-phosphofructokinase, liver type	IPI00332371	1.048	0.01959	10	9	12	18
105	A-kinase anchor protein 12 isoform 2	IPI00217683	1.048	0.01959	9	10	12	18
106	Isoform 1 of Vinculin	IPI00291175	1.043	0.01977	60	42	63	57
107	Isoform 1 of Acyl-CoA-binding protein	IPI00010182	1.035	0.02000	2	4	5	9
108	pyruvate dehydrogenase E1 alpha 1 isoform 2 precursor	IPI00306301	1.035	0.02000	0	4	7	7
109	Nodal modulator 1	IPI00329352	1.034	0.02074	9	11	14	17
110	Adenylate kinase isoenzyme 1	IPI00018342	1.020	0.02104	9	6	11	14
111	Splicing factor 3B subunit 1	IPI00026089	1.018	0.02104	33	28	39	37
112	32 kDa protein	IPI00176692	1.012	0.02108	83	51	90	63
113	Glucosamine-6-phosphate isomerase 1	IPI00009305	1.008	0.02115	8	14	15	18
114	Isoform 1 of Heterogeneous nuclear ribonucleoprotein D-like	IPI00011274	1.003	0.02115	9	7	14	12
115	Probable cysteinyl-tRNA synthetase, mitochondrial	IPI00336016	1.002	0.02152	2	2	7	4
116	Immature colon carcinoma transcript 1 protein	IPI00029114	1.002	0.02152	2	2	5	6
117	Brefeldin A-inhibited guanine nucleotide-exchange protein 2	IPI00002186	1.002	0.02152	0	0	5	6
118	cDNA FLJ56402, highly similar to Tripeptidyl-peptidase 1	IPI00298237	1.002	0.02152	2	0	7	4
119	cDNA FLJ56394, highly similar to N-acetylglucosamine kinase	IPI00296526	1.002	0.02152	2	2	6	5
120	Isoform 2 of Separin	IPI00017538	1.002	0.02152	0	0	6	5
121	Isoform 1 of Nuclear pore complex protein Nup214	IPI00183294	1.000	0.02163	2	5	5	10
122	treacle protein isoform a	IPI00165041	1.000	0.02163	3	4	8	7
123	ATP synthase subunit beta, mitochondrial	IPI00303476	0.998	0.02163	67	51	74	62
124	Isoform 3 of Core histone macro-H2A.1	IPI00059366	0.996	0.02163	16	7	18	16
125	Isoform 1 of Enoyl-CoA hydratase domain-containing protein 1	IPI00302688	0.987	0.02167	9	8	16	11
126	WD repeat-containing protein 36	IPI00169325	0.985	0.02171	12	12	18	17
127	Translin-associated protein X	IPI00293350	0.978	0.02171	6	6	11	10
128	Leucine-rich repeat and WD repeat-containing protein 1	IPI00069309	0.978	0.02171	4	8	11	10
129	FACT complex subunit SSRP1	IPI00005154	0.978	0.02171	7	5	10	11
130	ADP-sugar pyrophosphatase	IPI00296913	0.974	0.02171	13	12	17	19
131	Actin-related protein 2	IPI00005159	0.974	0.02171	12	13	18	18
132	Isoform 1 of Catenin beta-1	IPI00017292	0.973	0.02175	8	10	13	15
133	DEAD (Asp-Glu-Ala-Asp) box polypeptide 39 transcript variant	IPI00062206	0.969	0.02219	0	6	8	8
134	B-cell receptor-associated protein 31	IPI00218200	0.969	0.02219	5	3	10	6
135	Dihydropyrimidinase-related protein 2	IPI00257508	0.969	0.02219	4	4	7	9
136	Eukaryotic peptide chain release factor GTP-binding subunit ERF3A	IPI00218829	0.969	0.02219	2	6	7	9
137	Pyruvate carboxylase, mitochondrial	IPI00299402	0.969	0.02219	4	4	6	10
138	Ubiquitin-like modifier-activating enzyme 1	IPI00645078	0.957	0.02294	34	13	33	27
139	Isoform B of Arfaptin-1	IPI00021258	0.956	0.02305	2	3	6	6
140	Small nuclear ribonucleoprotein E	IPI00029266	0.942	0.02327	6	3	10	7
141	Superoxide dismutase [Mn], mitochondrial	IPI00022314	0.942	0.02327	5	4	10	7
142	High mobility group protein B2	IPI00219097	0.941	0.02331	6	8	13	10
143	Glucose-6-phosphate isomerase	IPI00027497	0.935	0.02335	22	17	24	27
144	Gamma-enolase	IPI00216171	0.934	0.02335	37	14	37	27
145	Intron-binding protein aquarius	IPI00297572	0.924	0.02353	7	8	12	12
146	Isoform 2 of Double-stranded RNA-specific adenosine deaminase	IPI00025057	0.918	0.02391	5	5	9	9
147	Ribosome maturation protein SBDS	IPI00427330	0.918	0.02391	5	5	8	10
148	Keratin, type I cytoskeletal 16	IPI00217963	0.918	0.02435	4	2	7	6
149	Alcohol dehydrogenase class-3	IPI00746777	0.918	0.02435	2	4	6	7
150	ATP-binding cassette sub-family D member 1	IPI00291373	0.918	0.02435	0	4	6	7
151	28S ribosomal protein S7, mitochondrial	IPI00006440	0.918	0.02435	3	3	5	8
152	Nuclear pore complex protein Nup50	IPI00026940	0.918	0.02435	3	3	7	6
153	TC4 protein	IPI00044779	0.918	0.02435	15	16	18	24
154	Thioredoxin-dependent peroxide reductase, mitochondrial	IPI00024919	0.910	0.02443	15	17	21	22
155	Thymidylate synthetase, isoform CRA_a	IPI00103732	0.909	0.02443	8	8	15	10
156	Isoform 1 of Glycerol-3-phosphate dehydrogenase, mitochondrial	IPI00017895	0.909	0.02443	9	7	11	14
157	Isoform 1 of Polypyrimidine tract-binding protein 1	IPI00179964	0.909	0.02443	32	11	23	32
158	Aspartyl-tRNA synthetase, cytoplasmic	IPI00216951	0.903	0.02446	32	41	44	43
159	Isoform 2 of Structural maintenance of chromosomes flexible hinge domain-containing protein 1	IPI00465022	0.900	0.02450	10	14	15	19
160	cDNA FLJ55574, highly similar to Calnexin	IPI00020984	0.897	0.02480	30	15	31	26
161	Isoform 1 of Electron transfer flavoprotein subunit beta	IPI00004902	0.894	0.02484	18	16	25	20
162	116 kDa U5 small nuclear ribonucleoprotein component	IPI00003519	0.891	0.02484	28	18	25	33
163	6-phosphofructokinase type C	IPI00009790	0.886	0.02487	34	27	33	41
164	Isoform 1 of POTE ankyrin domain family member E	IPI00479743	0.886	0.02491	0	5	7	7
165	Peroxisomal multifunctional enzyme type 2	IPI00019912	0.886	0.02491	5	2	5	9
166	SPRY domain-containing protein 4	IPI00291643	0.886	0.02491	4	3	7	7
167	Coatomer subunit beta'	IPI00220219	0.885	0.02491	25	22	28	31
168	Isoform Long of Sodium/potassium-transporting ATPase subunit alpha-1	IPI00006482	0.884	0.02495	52	46	52	61
169	Isoform DPI of Desmoplakin	IPI00013933	0.882	0.02495	33	29	35	40
170	10 kDa heat shock protein, mitochondrial	IPI00220362	0.881	0.02495	6	12	12	15
171	Activated RNA polymerase II transcriptional coactivator p15	IPI00221222	0.877	0.02528	5	7	11	9
172	Rho-associated protein kinase 2	IPI00307155	0.877	0.02528	8	4	9	11
173	Putative uncharacterized protein FUBP3	IPI00063245	0.874	0.02606	2	0	5	5
174	Isoform 2 of Actin-binding protein anillin	IPI00032958	0.874	0.02606	0	2	5	5
175	Protein transport protein Sec23A	IPI00017375	0.874	0.02606	2	0	4	6
176	Isoform 1 of Lysophospholipase-like protein 1	IPI00059762	0.874	0.02606	0	0	6	4
177	Serine/threonine-protein kinase 12	IPI00176642	0.874	0.02606	0	0	4	6
178	Serine/threonine-protein kinase mTOR	IPI00031410	0.868	0.02893	11	8	14	14
179	Keratin, type I cytoskeletal 10	IPI00009865	0.861	0.02919	73	58	70	77
180	Histidine triad nucleotide-binding protein 1	IPI00239077	0.859	0.02927	7	6	10	11
181	AP-1 complex subunit mu-1	IPI00032516	0.858	0.02927	6	0	9	6
182	Isoform 1 of Transcription elongation factor SPT6	IPI00784161	0.858	0.02927	5	3	9	6
183	Isoform 2 of Exosome complex exonuclease RRP44	IPI00183462	0.856	0.02930	11	9	15	14
184	Periplakin	IPI00298057	0.856	0.02930	12	8	13	16
185	Isoform 2 of Structural maintenance of chromosomes protein 4	IPI00328298	0.846	0.02945	18	12	19	21
186	Proteasome subunit beta type-1	IPI00025019	0.838	0.02983	19	12	21	20

No.	Description	Accession number	STN[1]	p-Value[1]	Con_A[2]	Con_B[2]	5FU_A[2]	5FU_B[2]
187	HMT1 hnRNP methyltransferase-like 2 isoform 1	IPI00018522	0.838	0.02983	13	18	18	23
188	Eukaryotic translation initiation factor 3 subunit C	IPI00016910	0.838	0.02983	17	14	22	19
189	Isoform 2 of Tyrosine-protein phosphatase non-receptor type 11	IPI00298347	0.833	0.02986	5	4	8	8
190	Isoform 1 of Peroxisomal acyl-coenzyme A oxidase 1	IPI00296907	0.833	0.02986	7	0	8	8
191	UPF0556 protein C19orf10	IPI00056357	0.833	0.02986	2	3	4	7
192	Ribose-phosphate pyrophosphokinase 3	IPI00218371	0.833	0.02986	3	0	5	6
193	Prenylcysteine oxidase 1	IPI00384280	0.833	0.02986	3	0	4	7
194	Deoxycytidine kinase	IPI00020454	0.833	0.02986	2	3	6	5
195	cDNA FLJ56184, highly similar to Proto-oncogene tyrosine-protein kinase LCK	IPI00394952	0.833	0.02986	3	2	6	5
196	Isoform 2 of Neuropathy target esterase	IPI00217600	0.833	0.02986	3	0	4	7
197	Tubulin beta-1 chain	IPI00006510	0.830	0.02990	16	16	21	21
198	Isoform 2 of Cytoplasmic FMR1-interacting protein 1	IPI00550212	0.828	0.02990	8	7	10	13
199	Eukaryotic translation initiation factor 2 subunit 1	IPI00219678	0.824	0.03001	34	26	37	35
200	Ubiquitin-conjugating enzyme E2 N	IPI00003949	0.814	0.03024	8	8	10	14
201	cDNA FLJ14048 fis, clone HEMBA1006650, weakly similar to ARP2/3 COMPLEX 20 KD SUBUNIT	IPI00386354	0.812	0.03072	6	4	7	10
202	E3 SUMO-protein ligase RanBP2	IPI00221325	0.804	0.03087	25	24	31	29
203	ATP synthase subunit b, mitochondrial	IPI00029133	0.800	0.03098	10	7	12	13
204	Isoform 1 of Phosphatidylinositol transfer protein beta isoform	IPI00334907	0.800	0.03098	9	8	11	14
205	Villin-1	IPI00218852	0.799	0.03098	24	26	30	31
206	Isoform 1 of Phosphatidylinositol-3,4,5-trisphosphate 5-phosphatase 1	IPI00329213	0.798	0.03098	4	2	6	6
207	myosin-IXb isoform 1	IPI00306933	0.798	0.03098	3	3	7	5
208	Isoform 6 of GTPase-activating protein and VPS9 domain-containing protein 1	IPI00292753	0.798	0.03098	2	4	6	6
209	Phosphatidylethanolamine-binding protein 1	IPI00219446	0.796	0.03243	19	18	22	25
210	Lamin-B receptor	IPI00292135	0.792	0.03258	9	0	9	9
211	Succinyl-CoA:3-ketoacid-coenzyme A transferase 1, mitochondrial	IPI00026516	0.792	0.03258	6	5	10	8
212	Isoform 1 of Clathrin heavy chain 2	IPI00022881	0.780	0.03344	40	32	45	39
213	Isoform 2 of Spliceosome RNA helicase BAT1	IPI00641829	0.778	0.03344	26	14	26	24
214	Eukaryotic translation initiation factor 3, subunit E interacting protein	IPI00465233	0.773	0.03362	25	16	28	23
215	Isoform 1 of UPF0424 protein C1orf128	IPI00015351	0.769	0.03362	3	4	7	6
216	Phosphatidylinositide phosphatase SAC1	IPI00022275	0.769	0.03362	2	5	6	7
217	Isoform 1 of Protein-glutamine gamma-glutamyltransferase 2	IPI00294578	0.769	0.03362	5	2	7	6
218	Isocitrate dehydrogenase [NAD] subunit gamma, mitochondrial	IPI00220150	0.769	0.03362	5	0	7	6
219	Neutral amino acid transporter B(0)	IPI00019472	0.764	0.03411	48	29	47	42
220	ribonucleotide reductase M2 polypeptide isoform 1	IPI00011118	0.758	0.03411	7	6	9	11
221	UPF0468 protein C16orf80	IPI00001655	0.758	0.03411	4	9	9	11
222	Protein tyrosine phosphatase-like protein PTPLAD1	IPI00008998	0.755	0.03444	13	8	14	15
223	Thyroid hormone receptor-associated protein 3	IPI00104050	0.755	0.03444	13	8	14	15
224	Isoform 2 of Proteasome subunit alpha type-3	IPI00171199	0.755	0.03444	9	12	16	13
225	Transaldolase	IPI00744692	0.752	0.03448	27	18	31	24
226	Single-stranded DNA-binding protein, mitochondrial	IPI00029744	0.746	0.03452	12	10	17	13
227	ADP/ATP translocase 3	IPI00291467	0.744	0.03470	0	6	0	12
228	L-xylulose reductase	IPI00448095	0.744	0.03470	4	4	7	7
229	Isoform 1 of Ataxin-2-like protein	IPI00456359	0.744	0.03470	6	2	7	7
230	Isoform 3 of UDP-N-acetylglucosamine--peptide N-acetylglucosaminyltransferase 110 kDa subunit	IPI00005780	0.744	0.03470	5	3	7	7
231	Isoform 2 of ATPase family AAA domain-containing protein 3A	IPI00295992	0.744	0.03470	6	2	7	7
232	Nuclear pore complex protein Nup133	IPI00291200	0.744	0.03537	8	6	10	11
233	Isoform 1 of Pyridoxal kinase	IPI00013004	0.744	0.03537	9	5	12	9
234	Ribose-phosphate pyrophosphokinase 1	IPI00219616	0.743	0.03552	2	2	2	7
235	Small nuclear ribonucleoprotein Sm D3	IPI00017964	0.743	0.03552	0	0	5	4
236	Isoform Long of ES1 protein homolog, mitochondrial	IPI00024913	0.743	0.03552	2	2	4	5
237	Scaffold attachment factor B1	IPI00300631	0.743	0.03552	2	0	6	3
238	Isoform A of GC-rich sequence DNA-binding factor homolog	IPI00001364	0.743	0.03552	2	0	4	5
239	Antigen peptide transporter 1	IPI00646625	0.743	0.03552	0	2	5	4
240	Isoform 3 of Yorkie homolog	IPI00009326	0.743	0.03552	2	0	5	4
241	NADH dehydrogenase [ubiquinone] iron-sulfur protein 4, mitochondrial	IPI00011217	0.743	0.03552	0	0	0	7
242	Peptidyl-prolyl cis-trans isomerase-like 1	IPI00007019	0.743	0.03552	0	0	5	4
243	Golgin subfamily A member 7	IPI00480022	0.743	0.03552	2	2	6	3
244	Isoform 1 of Proteasome subunit alpha type-7	IPI00024175	0.737	0.03563	18	16	23	20
245	E3 ubiquitin/ISG15 ligase TRIM25	IPI00029629	0.737	0.03563	15	19	24	19
246	Eukaryotic translation initiation factor 3 subunit A	IPI00029012	0.726	0.03612	40	29	44	36
247	Tubulin, beta	IPI00645452	0.724	0.03616	32	19	35	26
248	Pre-mRNA-processing factor 19	IPI00004968	0.723	0.03619	7	0	6	9
249	Nucleolar protein 56	IPI00411937	0.719	0.03623	15	10	15	18
250	Dolichyl-diphosphooligosaccharide--protein glycosyltransferase subunit STT3B	IPI00152377	0.717	0.03623	10	6	12	11
251	26S proteasome non-ATPase regulatory subunit 2	IPI00012268	0.717	0.03623	10	6	9	14
252	Adenosylhomocysteinase	IPI00012007	0.711	0.03653	33	41	46	39
253	Isoform 3 of Serine/threonine-protein kinase SMG1	IPI00183368	0.706	0.03657	0	3	2	8
254	histone deacetylase complex subunit SAP18	IPI00011698	0.706	0.03657	0	3	6	4
255	Isoform 1 of Ribonuclease H2 subunit C	IPI00382985	0.706	0.03657	3	2	5	5
256	Isoform 1 of Thymocyte nuclear protein 1	IPI00383163	0.706	0.03657	3	0	4	6
257	Isoform 1 of 182 kDa tankyrase-1-binding protein	IPI00304589	0.706	0.03657	3	0	6	4
258	Protein FAM50A	IPI00030098	0.706	0.03657	2	3	4	6
259	Isoform 1 of General transcription factor 3C polypeptide 1	IPI00414482	0.703	0.03675	3	7	6	10
260	DNA-directed RNA polymerase I subunit RPA1	IPI00031960	0.703	0.03675	5	5	9	7
261	Amidophosphoribosyltransferase	IPI00029534	0.703	0.03675	5	5	8	8
262	Isoform 1 of Mitochondrial antiviral-signaling protein	IPI00020719	0.703	0.03675	5	5	8	8
263	Calpain-2 catalytic subunit	IPI00289758	0.694	0.03850	10	8	11	14
264	Dolichyl-diphosphooligosaccharide--protein glycosyltransferase subunit 1 precursor	IPI00025874	0.693	0.03850	24	18	20	31
265	Isoform Short of Heterogeneous nuclear ribonucleoprotein U	IPI00479217	0.691	0.03850	48	33	47	45
266	proteasome-associated protein ECM29 homolog	IPI00157790	0.684	0.03854	15	4	12	14
267	Isoform 1 of Alpha-aminoadipic semialdehyde dehydrogenase	IPI00221234	0.684	0.03854	11	8	15	11
268	Programmed cell death protein 10	IPI00298558	0.684	0.03854	10	9	13	13
269	Galectin-3-binding protein	IPI00023673	0.676	0.03895	4	0	4	7
270	Isoform 1 of Spectrin beta chain, brain 2	IPI00012645	0.676	0.03895	3	3	7	4
271	Thimet oligopeptidase	IPI00549189	0.676	0.03895	2	4	4	7
272	WD repeat and HMG-box DNA-binding protein 1	IPI00411614	0.676	0.03895	3	3	4	7
273	Isoform 1 of Lysocardiolipin acyltransferase 1	IPI00419643	0.676	0.03895	2	4	5	6
274	Estradiol 17-beta-dehydrogenase 11	IPI00329598	0.670	0.03917	6	6	8	10
275	Isoform 1 of Insulin-like growth factor 2 mRNA-binding protein 2	IPI00179713	0.670	0.03917	6	6	8	10
276	Stathmin	IPI00479997	0.670	0.03917	8	4	9	9
277	CDGSH iron sulfur domain-containing protein 1	IPI00020510	0.670	0.03917	3	9	10	8
278	Ras-related protein Rab-7a	IPI00016342	0.670	0.04036	23	24	28	28
279	Isoform 1 of Heterogeneous nuclear ribonucleoprotein A3	IPI00419373	0.664	0.04074	21	12	20	21
280	Isoform 1 of Importin-5	IPI00793443	0.662	0.04074	31	18	32	26

No.	Description	Accession number	STN[1]	p-Value[1]	Con_A[2]	Con_B[2]	5FU_A[2]	5FU_B[2]
281	Mitogen-activated protein kinase 1	IPI00003479	0.650	0.04088	4	3	6	6
282	Replication factor C subunit 3	IPI00031521	0.650	0.04088	4	3	6	6
283	Isoform 1 of ATP-dependent RNA helicase DDX42	IPI00409671	0.643	0.04111	7	7	9	11
284	Phenylalanyl-tRNA synthetase alpha chain	IPI00031820	0.643	0.04111	8	6	9	11
285	Valyl-tRNA synthetase	IPI00000873	0.642	0.04226	23	14	24	21
286	Isoform 1 of F-actin-capping protein subunit beta	IPI00026185	0.640	0.04237	11	13	17	14
287	Eukaryotic initiation factor 4A-III	IPI00009328	0.637	0.04245	20	18	25	21
288	Isochorismatase domain-containing protein 1	IPI00304082	0.632	0.04249	11	14	18	14
289	Tyrosyl-tRNA synthetase, cytoplasmic	IPI00007074	0.632	0.04249	11	14	18	14
290	Calpain-1 catalytic subunit	IPI00011285	0.631	0.04263	10	5	11	10
291	Sialic acid synthase	IPI00147874	0.631	0.04263	9	6	9	12
292	145 kDa protein	IPI00218097	0.629	0.04263	6	2	6	7
293	NDUFB10 protein	IPI00074489	0.629	0.04263	3	5	8	5
294	Isoform 1 of BH3-interacting domain death agonist	IPI00413587	0.629	0.04263	3	5	7	6
295	Transmembrane protein 126A	IPI00031064	0.629	0.04263	4	4	9	4
296	MACRO domain-containing protein 1	IPI00155601	0.629	0.04263	4	4	6	7
297	Aldehyde dehydrogenase, mitochondrial	IPI00006663	0.625	0.04290	16	10	19	14
298	Electron transfer flavoprotein subunit alpha, mitochondrial	IPI00010810	0.619	0.04383	14	13	20	14
299	Nucleolar protein 58	IPI00006379	0.612	0.04416	15	13	19	16
300	DnaJ homolog subfamily C member 13	IPI00307259	0.612	0.04416	9	19	17	18
301	Stress-70 protein, mitochondrial	IPI00007765	0.610	0.04416	34	30	32	41
302	Isoform 1 of Proteasome activator complex subunit 4	IPI00005260	0.610	0.04427	4	5	7	7
303	Isoform A of Ras-related C3 botulinum toxin substrate 1	IPI00010271	0.610	0.04427	4	5	8	6
304	Selenide, water dikinase 1	IPI00029056	0.610	0.04427	7	2	9	5
305	L-aminoadipate-semialdehyde dehydrogenase-phosphopantetheinyl transferase	IPI00250297	0.610	0.04427	7	2	10	4
306	Peptidyl-prolyl cis-trans isomerase H	IPI00007346	0.610	0.04427	4	5	6	8
307	Eukaryotic translation initiation factor 3 subunit M	IPI00102069	0.609	0.04520	9	8	12	11
308	Lactoylglutathione lyase	IPI00220766	0.609	0.04520	10	7	12	11
309	Histone H2A type 1-B/E	IPI00026272	0.607	0.04520	0	0	0	6
310	Anaphase-promoting complex subunit 1	IPI00033907	0.607	0.04520	0	2	4	4
311	Activating signal cointegrator 1 complex subunit 3	IPI00430472	0.607	0.04520	0	2	6	2
312	CAAX prenyl protease 1 homolog	IPI00027180	0.607	0.04520	0	0	3	5
313	Putative uncharacterized protein KIAA0090	IPI00640734	0.607	0.04520	0	2	2	6
314	Small glutamine-rich tetratricopeptide repeat-containing protein alpha	IPI00013949	0.607	0.04520	2	2	3	5
315	DNA excision repair protein ERCC-6-like	IPI00552569	0.607	0.04520	0	2	4	4
316	Isoform 1 of Coiled-coil domain-containing protein 109A	IPI00171573	0.607	0.04520	2	0	3	5
317	cDNA FLJ56037, highly similar to Cullin-2	IPI00014311	0.607	0.04520	2	0	3	5
318	Heat shock protein beta-11	IPI00098827	0.607	0.04520	2	2	3	5
319	Isoform 1 of Ubiquitin carboxyl-terminal hydrolase 15	IPI00000728	0.607	0.04520	2	2	0	6
320	Kinesin-like protein KIF22	IPI00000769	0.607	0.04520	0	0	4	4
321	28S ribosomal protein S9, mitochondrial	IPI00641924	0.607	0.04520	2	0	5	3
322	Isoform 2 of Oxidation resistance protein 1	IPI00298348	0.607	0.04520	2	2	6	2
323	Isoform 1 of Regulator of nonsense transcripts 3B	IPI00023409	0.607	0.04520	2	2	5	3
324	Isoform 1 of Mammalian ependymin-related protein 1	IPI00259102	0.607	0.04520	2	0	5	3
325	cDNA FLJ60607, highly similar to Acyl-protein thioesterase 1	IPI00007321	0.607	0.04520	0	0	6	2
326	Isoform 2 of Protein-glutamine gamma-glutamyltransferase 2	IPI00218251	0.607	0.04520	0	0	0	6
327	Isoform 1 of YTH domain family protein 1	IPI00221345	0.607	0.04520	2	0	3	5
328	Kinesin-like protein KIFC1	IPI00306400	0.607	0.04520	0	0	5	3
329	Isoform 1 of Hematological and neurological expressed 1-like protein	IPI00027397	0.607	0.04520	2	0	4	4
330	Isoform 1 of WD repeat-containing protein 1	IPI00746165	0.606	0.05280	14	15	19	17
331	Dihydropteridine reductase	IPI00014439	0.599	0.05302	8	10	14	10
332	Flap endonuclease 1	IPI00026215	0.593	0.05366	4	6	7	8
333	Ribosomal L1 domain-containing protein 1	IPI00008708	0.593	0.05366	7	3	8	7
334	Glutathione S-transferase kappa 1	IPI00219673	0.588	0.05366	15	17	21	18
335	Small nuclear ribonucleoprotein Sm D1	IPI00302850	0.583	0.05399	16	17	19	21
336	Solute carrier family 2, facilitated glucose transporter member 1	IPI00220194	0.583	0.05399	17	16	22	18
337	DNA polymerase delta catalytic subunit	IPI00002894	0.582	0.05407	10	10	10	16
338	Beta-hexosaminidase subunit beta	IPI00012585	0.578	0.05459	5	6	7	9
339	Growth arrest and DNA damage-inducible proteins-interacting protein 1	IPI00552587	0.578	0.05459	5	6	10	6
340	Coiled-coil-helix-coiled-coil-helix domain-containing protein 3, mitochondrial	IPI00015833	0.578	0.05459	6	5	9	7
341	transcriptional regulator ATRX isoform 2	IPI00220109	0.576	0.05485	0	3	2	7
342	14 kDa protein	IPI00179589	0.576	0.05485	3	0	3	6
343	Isoform 1 of Nesprin-3	IPI00394994	0.576	0.05485	3	2	4	5
344	Isoform CSBP2 of Mitogen-activated protein kinase 14	IPI00002857	0.576	0.05485	2	3	5	4
345	Isoform 2 of Ribosomal RNA processing protein 1 homolog B	IPI00032374	0.576	0.05485	3	0	5	4
346	Isoform 2 of Calumenin	IPI00045396	0.576	0.05485	3	2	5	4
347	Isoform p27-L of 26S proteasome non-ATPase regulatory subunit 9	IPI00010860	0.576	0.05485	3	2	6	3
348	protein ELYS	IPI00170594	0.573	0.05485	9	12	14	13
349	Elongation factor 1-beta	IPI00178440	0.573	0.05485	10	11	15	12
350	GMP synthase [glutamine-hydrolyzing]	IPI00029079	0.573	0.05485	11	10	13	14
351	Isoform 1 of 14-3-3 protein sigma	IPI00013890	0.573	0.05485	10	11	15	12
352	Isoform 1 of General transcription factor II-I	IPI00054042	0.573	0.05485	22	13	19	23
353	E3 ubiquitin-protein ligase UBR5	IPI00026320	0.564	0.05533	7	5	8	9
354	Dolichyl-diphosphooligosaccharide--protein glycosyltransferase subunit STT3A	IPI00297492	0.564	0.05533	6	6	10	7
355	Isoform 1 of Tyrosine-protein kinase BAZ1B	IPI00069817	0.564	0.05533	7	5	9	8
356	Gamma-aminobutyric acid receptor-associated protein-like 2	IPI00026358	0.564	0.05533	6	6	9	8
357	Isoform 2 of U1 small nuclear ribonucleoprotein 70 kDa	IPI00219483	0.564	0.05533	6	6	9	8
358	PDZ domain-containing protein GIPC1	IPI00024705	0.564	0.05533	7	5	9	8
359	Metastasis-associated protein MTA2	IPI00171798	0.564	0.05533	7	5	9	8
360	Inosine-5'-monophosphate dehydrogenase 2	IPI00291510	0.564	0.05533	19	18	23	21
361	DNA damage-binding protein 1	IPI00293464	0.563	0.05537	39	44	51	41
362	UPF0568 protein C14orf166	IPI00006980	0.559	0.05541	14	9	17	12
363	Midasin	IPI00167941	0.555	0.05541	20	19	18	28
364	Isoform 2 of Microtubule-actin cross-linking factor 1, isoforms 1/2/3/5	IPI00256861	0.552	0.05559	5	8	7	11
365	cDNA FLJ56285, highly similar to ADP-ribosylation factor-like protein 8B	IPI00018871	0.552	0.05559	7	6	8	10
366	Isoform 2 of SWI/SNF complex subunit SMARCC2	IPI00150057	0.552	0.05559	10	3	9	9
367	Isoform 1 of Symplekin	IPI00023344	0.552	0.05559	8	5	9	9
368	SWI/SNF related, matrix associated, actin dependent regulator of chromatin, subfamily a, member 1	IPI00216046	0.552	0.05559	10	3	10	8
369	26S protease regulatory subunit 4	IPI00011126	0.552	0.05559	8	5	11	7
370	Importin-9	IPI00185146	0.552	0.05559	9	4	9	9
371	Acylamino-acid-releasing enzyme	IPI00337741	0.552	0.05559	8	5	11	7
372	Importin subunit alpha-2	IPI00002214	0.552	0.05600	13	11	18	12
373	V-type proton ATPase subunit B, brain isoform	IPI00007812	0.552	0.05600	13	11	15	15
374	Isoform 1 of UBX domain-containing protein 1	IPI00027378	0.550	0.05656	4	0	5	5

No.	Description	Accession number	STN[1]	p-Value[1]	Con_A[2]	Con_B[2]	5FU_A[2]	5FU_B[2]
375	39S ribosomal protein L24, mitochondrial	IPI00514506	0.550	0.05656	0	4	6	4
376	Isoform 1 of PC4 and SFRS1-interacting protein	IPI00028122	0.550	0.05656	4	2	4	6
377	Isoform 1 of SEC23-interacting protein	IPI00026969	0.550	0.05656	3	3	5	5
378	Isoform 3 of DNA repair protein RAD50	IPI00107531	0.550	0.05656	4	2	6	4
379	60S ribosomal protein L19	IPI00025329	0.550	0.05656	3	3	6	4
380	ANKHD1-EIF4EBP3 protein	IPI00217442	0.550	0.05656	4	2	5	5
381	Nucleoporin 54kDa variant (Fragment)	IPI00172580	0.550	0.05656	4	2	2	8
382	Synapse-associated protein 1	IPI00059242	0.550	0.05656	3	3	5	5
383	Protein FRG1	IPI00004655	0.550	0.05656	3	3	5	5
384	Mediator of RNA polymerase II transcription subunit 13	IPI00021388	0.550	0.05656	4	0	6	4
385	DKFZP586J0619 protein	IPI00740961	0.540	0.06088	8	6	8	11
386	Tubulin beta-2C chain	IPI00007752	0.534	0.06140	113	72	103	93
387	Isoform 1 of Isocitrate dehydrogenase [NAD] subunit alpha, mitochondrial	IPI00030702	0.533	0.06144	14	13	16	17
388	Isoform 1 of Splicing factor U2AF 65 kDa subunit	IPI00031556	0.533	0.06144	17	10	17	16
389	Echinoderm microtubule-associated protein-like 4	IPI00001466	0.530	0.06155	7	8	14	6
390	epiplakin	IPI00010951	0.530	0.06155	10	5	8	12
391	Ras-related protein Rab-18	IPI00014577	0.530	0.06155	7	8	12	8
392	Isoform 2 of Ubiquitin carboxyl-terminal hydrolase isozyme L5	IPI00219512	0.528	0.06189	4	3	7	4
393	Sphingosine-1-phosphate lyase 1	IPI00099463	0.528	0.06189	3	4	4	7
394	GTPase NRas	IPI00000005	0.528	0.06189	4	3	8	3
395	Isoform 2 of Ubiquilin-1	IPI00071180	0.528	0.06189	4	3	5	6
396	28S ribosomal protein S18b, mitochondrial	IPI00022316	0.528	0.06189	4	3	6	5
397	F-actin-capping protein subunit alpha-2	IPI00026182	0.528	0.06189	4	3	5	6
398	Isoform 1 of Transformation/transcription domain-associated protein	IPI00069084	0.527	0.06189	14	14	14	20
399	Radixin, isoform CRA_a	IPI00017367	0.522	0.06256	16	13	15	20
400	Isoform 1 of Regulator of nonsense transcripts 1	IPI00034049	0.522	0.06256	17	12	16	19
401	Proliferation-associated protein 2G4	IPI00299000	0.516	0.06274	18	12	20	16
402	cDNA FLJ56307, highly similar to Ubiquitin thioesterase protein OTUB1	IPI00000581	0.511	0.06311	14	17	19	18
403	Protein RRP5 homolog	IPI00400922	0.511	0.06311	25	26	28	30
404	Galectin-3	IPI00465431	0.511	0.06315	9	8	11	11
405	2-oxoglutarate dehydrogenase, mitochondrial	IPI00098902	0.511	0.06315	12	5	7	15
406	COP9 signalosome complex subunit 6	IPI00163230	0.510	0.06338	5	3	7	5
407	Nuclear pore complex protein Nup93	IPI00397904	0.510	0.06338	5	3	6	6
408	cDNA FLJ55177, highly similar to Ras-related protein Ral-B	IPI00004397	0.510	0.06338	5	3	8	4
409	Isoform B of Serine/threonine-protein kinase 24	IPI00002212	0.510	0.06338	5	3	6	6
410	Leucine-rich repeat-containing protein 47	IPI00170935	0.510	0.06338	5	3	6	6
411	Matrin-3	IPI00017297	0.508	0.06702	34	18	25	34
412	Ras-related protein Rab-2A	IPI00031169	0.507	0.06706	15	17	19	19
413	cDNA FLJ40024 fis, clone STOMA2007745, highly similar to UBIQUITIN-ACTIVATING ENZYME E1	IPI00026119	0.504	0.06732	50	32	48	42
414	Isoform Short of Glycylpeptide N-tetradecanoyltransferase 1	IPI00218830	0.503	0.06732	7	11	12	11
415	Isoform 1 of Host cell factor 1	IPI00019848	0.502	0.06732	15	18	19	20
416	Importin-7	IPI00007402	0.494	0.06769	38	19	28	36
417	General transcription factor IIF subunit 2	IPI00477686	0.494	0.06777	4	5	6	7
418	U4/U6.U5 tri-snRNP-associated protein 2	IPI00419844	0.494	0.06777	6	3	5	8
419	Isoform 1 of Uridine 5'-monophosphate synthase	IPI00003923	0.494	0.06777	6	3	5	8
420	Poly [ADP-ribose] polymerase 1	IPI00449049	0.489	0.06777	43	47	47	51
421	Nuclear migration protein nudC	IPI00550746	0.488	0.06777	12	8	15	10
422	Isoform 1 of Cullin-associated NEDD8-dissociated protein 1	IPI00100160	0.486	0.06777	38	22	31	36
423	Isoform 1 of Coiled-coil domain-containing protein 47	IPI00024642	0.480	0.06822	5	5	7	7
424	Developmentally-regulated GTP-binding protein 2	IPI00022697	0.480	0.06822	5	5	8	6
425	Putative high mobility group protein B3-like-1	IPI00006437	0.480	0.06822	6	4	8	6
426	Isoform 2 of Ubiquinol-cytochrome c reductase complex chaperone CBP3 homolog	IPI00219889	0.480	0.06822	4	6	5	9
427	cDNA FLJ78679, highly similar to Homo sapiens DEAD (Asp-Glu-Ala-Asp) box polypeptide 46 (DDX46)	IPI00329791	0.477	0.07116	22	17	23	22
428	Isoform 1 of Tryptophanyl-tRNA synthetase, cytoplasmic	IPI00295400	0.468	0.07131	13	10	13	15
429	Glutaredoxin-3	IPI00008552	0.467	0.07149	8	3	10	5
430	nardilysin isoform a	IPI00243221	0.467	0.07149	5	6	10	5
431	GTP:AMP phosphotransferase mitochondrial	IPI00465256	0.467	0.07149	6	5	7	8
432	ADP-ribosylation factor-like protein 2	IPI00003326	0.467	0.07149	6	5	7	8
433	Keratin, type II cytoskeletal 75	IPI00005859	0.467	0.07149	0	0	3	4
434	Cathepsin B	IPI00295741	0.467	0.07149	0	0	3	4
435	Mimitin, mitochondrial	IPI00031109	0.467	0.07149	2	0	3	4
436	Isoform 2 of Peptidyl-prolyl cis-trans isomerase NIMA-interacting 4	IPI00006658	0.467	0.07149	0	0	3	4
437	Golgin subfamily B member 1	IPI00004671	0.467	0.07149	0	0	3	4
438	Isoform 2C of Cytoplasmic dynein 1 intermediate chain 2	IPI00216348	0.467	0.07149	2	2	3	4
439	myosin regulatory light polypeptide 9 isoform b	IPI00030929	0.467	0.07149	2	2	4	3
440	Protein VAC14 homolog	IPI00025160	0.467	0.07149	2	0	2	5
441	Thymidine kinase, cytosolic	IPI00299214	0.467	0.07149	2	0	3	4
442	ESF1 homolog	IPI00024167	0.467	0.07149	2	2	3	4
443	Isoform 1 of Ubiquitin-protein ligase E3C	IPI00604464	0.467	0.07149	0	2	0	5
444	DNA-directed RNA polymerases I, II, and III subunit RPABC3	IPI00003309	0.467	0.07149	0	2	3	4
445	Methylosome protein 50	IPI00012202	0.467	0.07149	0	0	4	3
446	Sterol-4-alpha-carboxylate 3-dehydrogenase, decarboxylating	IPI00019407	0.467	0.07149	2	0	2	5
447	cDNA, FLJ96508, Homo sapiens SH3-domain GRB2-like 1 (SH3GL1), mRNA	IPI00019169	0.467	0.07149	0	2	3	4
448	Isoform 1 of UPF0557 protein C10orf119	IPI00478758	0.467	0.07149	2	2	3	4
449	Isoform 2 of Transcription elongation factor SPT6	IPI00430770	0.467	0.07149	2	0	5	2
450	D-dopachrome decarboxylase	IPI00293867	0.467	0.07149	2	2	3	4
451	Isoform 1 of OCIA domain-containing protein 2	IPI00555902	0.467	0.07149	2	0	5	0
452	PRA1 family protein 3	IPI00007426	0.467	0.07149	2	0	2	5
453	Mitochondrial import inner membrane translocase subunit Tim13	IPI00001589	0.467	0.07149	2	0	2	5
454	Isoform 1 of Protein furry homolog-like	IPI00739940	0.467	0.07149	0	2	3	4
455	cDNA FLJ55034	IPI00384122	0.467	0.07149	2	0	0	5
456	3'-5' exoribonuclease CSL4 homolog	IPI00032823	0.467	0.07149	0	0	5	2
457	Cysteine and glycine-rich protein 2	IPI00002824	0.467	0.07149	2	2	4	3
458	Serine/threonine-protein phosphatase 4 catalytic subunit	IPI00012833	0.467	0.07149	0	0	5	0
459	Putative uncharacterized protein RBM12B	IPI00217626	0.467	0.07149	0	0	4	3
460	Farnesyl pyrophosphate synthetase like-4 protein (Fragment)	IPI00382869	0.467	0.07149	2	2	3	4
461	cDNA FLJ40287 fis, clone TESTI2027909, highly similar to 5'-AMP-ACTIVATED PROTEIN KINASE	IPI00473047	0.467	0.07149	0	2	4	3
462	Isoform 1 of Death-inducer obliterator 1	IPI00249982	0.467	0.07149	0	0	3	4
463	Isoform Long of Ras-related protein Rab-27A	IPI00016381	0.467	0.07149	0	2	4	3
464	Isoform 1 of Polyglutamine-binding protein 1	IPI00024698	0.467	0.07149	0	0	4	3
465	Isoform 1 of Syntaxin-7	IPI00289876	0.467	0.07149	2	2	0	5
466	Vacuolar protein sorting-associated protein 37B	IPI00002926	0.467	0.07149	2	0	4	3
467	NADH-cytochrome b5 reductase 1	IPI00470674	0.467	0.07149	0	0	3	4
468	Calmodulin-regulated spectrin-associated protein 3	IPI00176702	0.467	0.07149	2	0	3	4

No.	Description	Accession number	STN[1]	p-Value[1]	Con_A[2]	Con_B[2]	5FU_A[2]	5FU_B[2]
469	Leucine-rich repeat-containing protein 57	IPI00470576	0.467	0.07149	0	0	5	2
470	Isoform 2 of Serine/threonine-protein kinase PAK 1	IPI00289746	0.467	0.07149	0	2	3	4
471	Choline-phosphate cytidylyltransferase A	IPI00329338	0.467	0.07149	0	0	4	3
472	Nuclear pore glycoprotein p62	IPI00293533	0.467	0.07149	2	0	3	4
473	Isoform 2 of Inner centromere protein	IPI00759472	0.467	0.07149	0	0	3	4
474	Isoleucyl-tRNA synthetase, cytoplasmic	IPI00644127	0.463	0.07335	25	18	21	28
475	Isoform 1 of Myoferlin	IPI00021048	0.459	0.07347	53	57	61	57
476	26S protease regulatory subunit 8	IPI00023919	0.457	0.07347	13	12	15	15
477	Phosphoserine phosphatase	IPI00019178	0.456	0.07350	6	6	7	9
478	Phospholipase A-2-activating protein	IPI00218465	0.446	0.07581	17	10	15	17
479	Bifunctional ATP-dependent dihydroxyacetone kinase/FAD-AMP lyase (cyclizing)	IPI00551024	0.446	0.07604	8	5	11	6
480	Aldose reductase	IPI00413641	0.441	0.07604	0	3	4	4
481	cDNA FLJ60317, highly similar to Aminoacylase-1	IPI00009268	0.441	0.07604	3	2	3	5
482	Isoform 1 of Citron Rho-interacting kinase	IPI00022465	0.441	0.07604	0	3	4	4
483	Isoform 1 of V-type proton ATPase subunit H	IPI00296191	0.441	0.07604	3	2	2	6
484	Isoform 1 of 2-oxoglutarate and iron-dependent oxygenase domain-containing protein 1	IPI00170429	0.441	0.07604	3	0	0	6
485	Isoform 2 of Suppressor of SWI4 1 homolog	IPI00219793	0.441	0.07604	0	3	5	3
486	Isoform 1 of L-2-hydroxyglutarate dehydrogenase, mitochondrial	IPI00016458	0.441	0.07604	0	3	4	4
487	Isoform 1 of KH domain-containing, RNA-binding, signal transduction-associated protein 1	IPI00008575	0.441	0.07604	3	0	4	4
488	tropomyosin alpha-3 chain isoform 1	IPI00183968	0.441	0.07604	0	3	4	4
489	cDNA FLJ56468, highly similar to Kynurenine--oxoglutarate transaminase 1	IPI00002523	0.441	0.07604	3	0	5	3
490	Isoform 3 of Cytosolic 5'-nucleotidase 3	IPI00100192	0.441	0.07604	3	0	5	3
491	Ribosomal RNA processing protein 1 homolog A	IPI00550766	0.441	0.07604	3	0	5	3
492	Isoform 1 of U4/U6 small nuclear ribonucleoprotein Prp4	IPI00150269	0.441	0.07604	2	3	5	3
493	Isoform 1 of EH domain-binding protein 1	IPI00178187	0.441	0.07604	2	3	4	4
494	Isoform 2 of Heme-binding protein 2	IPI00003799	0.441	0.07604	2	3	3	5
495	mRNA export factor	IPI00019733	0.441	0.07604	3	0	3	5
496	Calcium-binding protein p22	IPI00218924	0.441	0.07604	3	2	5	3
497	40S ribosomal protein S8	IPI00216587	0.437	0.07611	15	14	15	19
498	Isoform 1 of Nuclear pore complex protein Nup155	IPI00026625	0.437	0.07611	14	15	17	17
499	Isoform 3 of LIM domain only protein 7	IPI00291802	0.437	0.07611	16	13	16	18
500	Isoform 1 of U2-associated protein SR140	IPI00143753	0.436	0.07633	8	6	8	10
501	Protein NipSnap homolog 1	IPI00304435	0.436	0.07633	8	6	11	7
502	Signal recognition particle 14 kDa protein	IPI00293434	0.436	0.07633	8	6	10	8
503	Ubiquitin carboxyl-terminal hydrolase 24	IPI00902614	0.428	0.07827	8	7	11	8
504	Isoform 1 of Gamma-glutamylcyclotransferase	IPI00031564	0.428	0.07827	8	7	10	9
505	Nucleoporin 85	IPI00171542	0.428	0.07827	11	4	8	11
506	Small acidic protein	IPI00003419	0.428	0.07827	9	6	10	9
507	Putative pre-mRNA-splicing factor ATP-dependent RNA helicase DHX15	IPI00396435	0.427	0.07831	36	20	32	30
508	Phosphoribosylformylglycinamidine synthase	IPI00004534	0.424	0.07846	18	14	16	21
509	Hypoxia up-regulated protein 1	IPI00000877	0.422	0.07846	34	24	29	35
510	ATP-dependent DNA helicase Q1	IPI00178431	0.422	0.07846	29	29	34	30
511	Protein RCC2	IPI00465044	0.420	0.07853	4	0	7	2
512	Isoform 2 of Serrate RNA effector molecule homolog	IPI00220038	0.420	0.07853	4	2	4	5
513	Ras suppressor protein 1	IPI00017256	0.420	0.07853	3	3	4	5
514	DNA-directed RNA polymerase, mitochondrial precursor	IPI00298738	0.420	0.07853	2	4	0	7
515	Prefoldin subunit 4	IPI00015891	0.420	0.07853	4	2	7	2
516	Isoform 1 of tRNA (adenine-N(1)-)-methyltransferase non-catalytic subunit TRM6	IPI00099311	0.420	0.07853	4	2	3	6
517	cDNA FLJ61162, highly similar to Ras-related protein R-Ras2	IPI00012512	0.420	0.07853	3	3	4	5
518	28S ribosomal protein S21, mitochondrial	IPI00014812	0.420	0.07853	3	3	4	5
519	Serine/threonine-protein kinase N2	IPI00002804	0.420	0.07853	2	4	5	4
520	Copper chaperone for superoxide dismutase	IPI00021389	0.420	0.07853	2	4	4	5
521	Phosphomannomutase 2	IPI00006092	0.420	0.07853	3	3	5	4
522	Ubiquitin domain-containing protein UBFD1	IPI00005194	0.420	0.07853	2	4	4	5
523	Isoform 1 of Endophilin-B1	IPI00006558	0.420	0.07853	4	0	5	4
524	Isoform 1 of Serine/threonine-protein kinase 4	IPI00011488	0.420	0.07853	4	0	4	5
525	Isoform 1 of Transcription elongation regulator 1	IPI00247871	0.420	0.07898	7	9	9	11
526	Replication factor C subunit 4	IPI00017381	0.420	0.07898	11	5	11	9
527	T-complex protein 1 subunit zeta	IPI00027626	0.416	0.08073	48	13	33	34
528	Isoform 2 of Signal recognition particle 68 kDa protein	IPI00102936	0.405	0.08099	12	6	8	14
529	Condensin-2 complex subunit D3	IPI00747787	0.403	0.08188	5	2	6	4
530	Programmed cell death protein 5	IPI00023640	0.403	0.08188	4	3	5	5
531	28S ribosomal protein S25, mitochondrial	IPI00013167	0.403	0.08188	4	3	6	4
532	Dihydrofolate reductase	IPI00030357	0.403	0.08188	3	4	5	5
533	Sorting nexin-9	IPI00001883	0.403	0.08188	3	4	4	6
534	Isoform 1 of RNA-binding protein 4	IPI00003704	0.403	0.08188	4	3	6	4
535	Isoform 2 of Fumarylacetoacetate hydrolase domain-containing protein 1	IPI00440828	0.403	0.08188	4	3	4	6
536	Isoform Short of NADPH:adrenodoxin oxidoreductase, mitochondrial	IPI00026958	0.403	0.08188	4	3	4	6
537	similar to RAN binding protein 1	IPI00399212	0.403	0.08188	3	4	6	4
538	REST corepressor 1	IPI00008531	0.403	0.08188	2	5	5	5
539	Isoform 1 of Poly(U)-binding-splicing factor PUF60	IPI00069750	0.399	0.08434	12	7	9	14
540	cDNA FLJ51909, highly similar to Serine-threonine kinase receptor-associatedprotein	IPI00294536	0.399	0.08434	11	8	12	11
541	Isoform 1 of Core-binding factor subunit beta	IPI00016746	0.399	0.08434	11	8	12	11
542	probable ubiquitin carboxyl-terminal hydrolase FAF-X isoform 4	IPI00003964	0.393	0.08445	11	9	11	13
543	Ras-related protein Rab-14	IPI00291928	0.393	0.08445	10	10	14	10
544	Vesicle-trafficking protein SEC22b	IPI00006865	0.393	0.08445	11	9	12	12
545	Isoform 1 of AP-2 complex subunit mu	IPI00022256	0.388	0.08557	0	6	6	5
546	Mitochondrial chaperone BCS1	IPI00003985	0.388	0.08557	5	3	6	5
547	Isoform 1 of Pre-mRNA-processing factor 40 homolog A	IPI00337385	0.388	0.08557	5	3	5	6
548	Isoform 1 of HBS1-like protein	IPI00009070	0.388	0.08557	6	2	6	5
549	3-ketoacyl-CoA thiolase, mitochondrial	IPI00001539	0.388	0.08557	5	3	5	6
550	Isoform 1 of Prolyl 4-hydroxylase subunit alpha-1	IPI00009923	0.388	0.08557	3	5	6	5
551	Isoform 1 of Proteasome assembly chaperone 1	IPI00030770	0.388	0.08557	3	5	6	5
552	Acylphosphatase-1	IPI00221117	0.388	0.08557	3	5	6	5
553	Isoform 2 of Phosphoglucomutase-1	IPI00217872	0.388	0.08557	4	4	5	6
554	cDNA FLJ55482, highly similar to Annexin A11	IPI00414320	0.387	0.08579	13	8	16	9
555	Isoform 1 of Fanconi anemia group I protein	IPI00019447	0.382	0.08605	16	6	12	14
556	Isoform Beta-2 of DNA topoisomerase 2-beta	IPI00027280	0.382	0.08605	12	10	12	14
557	FACT complex subunit SPT16	IPI00026970	0.381	0.08672	39	42	46	41
558	Isoform alpha-enolase of Alpha-enolase	IPI00465248	0.380	0.08672	131	69	115	93
559	Ubiquitin carboxyl-terminal hydrolase isozyme L3	IPI00011250	0.377	0.08672	12	11	14	13
560	Isoform 1 of Surfeit locus protein 4	IPI00005737	0.376	0.08672	2	7	7	5
561	UPF0553 protein C9orf64	IPI00170972	0.376	0.08672	5	4	9	3
562	Isoform 1 of Roundabout homolog 1	IPI00219798	0.376	0.08672	5	4	5	7
563	cDNA FLJ53975, highly similar to Acetyl-CoA acetyltransferase, cytosolic	IPI00291419	0.376	0.08672	6	3	7	5

No.	Description	Accession number	STN[1]	p-Value[1]	Con_A[2]	Con_B[2]	5FU_A[2]	5FU_B[2]
564	Isoform 1 of N-alpha-acetyltransferase 25, NatB auxiliary subunit	IPI00025890	0.376	0.08672	7	2	4	8
565	Cytosolic purine 5'-nucleotidase	IPI00029054	0.376	0.08672	7	2	6	6
566	Isoform 1 of Rho GTPase-activating protein 18	IPI00296353	0.376	0.08672	4	5	6	6
567	Early endosome antigen 1	IPI00329536	0.372	0.08795	12	12	15	13
568	6-phosphogluconolactonase	IPI00029997	0.372	0.08795	15	9	15	13
569	Structural maintenance of chromosomes protein 3	IPI00219420	0.365	0.08832	30	22	30	27
570	40S ribosomal protein S20	IPI00012493	0.364	0.08832	5	5	7	6
571	Isoform 5 of Thioredoxin reductase 1, cytoplasmic	IPI00554786	0.364	0.08832	6	4	6	7
572	Isoform 1 of Heterogeneous nuclear ribonucleoprotein U-like protein 1	IPI00013070	0.364	0.08832	5	5	6	7
573	Protein FAM162A	IPI00023001	0.364	0.08832	5	5	9	4
574	Isoform Alpha of Signal transducer and activator of transcription 1-alpha/beta	IPI00030781	0.364	0.08832	8	2	6	7
575	pyrroline-5-carboxylate reductase 1, mitochondrial isoform 2	IPI00376503	0.364	0.08832	4	6	7	6
576	Twinfilin-2	IPI00550917	0.364	0.08832	6	4	7	6
577	Isoform 1 of Cleavage and polyadenylation specificity factor subunit 6	IPI00012998	0.364	0.08832	6	4	7	6
578	Heat shock-related 70 kDa protein 2	IPI00007702	0.364	0.08832	4	6	7	6
579	Sorting nexin-2	IPI00299095	0.364	0.08832	6	4	6	7
580	Histone H2A.V	IPI00018278	0.363	0.08877	14	12	16	14
581	Thioredoxin	IPI00216298	0.363	0.08877	13	13	14	16
582	Isoform 1 of Adenylyl cyclase-associated protein 1	IPI00008274	0.363	0.08877	16	10	17	13
583	Isoform 1 of Heterogeneous nuclear ribonucleoprotein D0	IPI00028888	0.363	0.08877	16	10	14	16
584	Isoform 2 of Liprin-beta-1	IPI00179172	0.359	0.08910	15	12	13	18
585	Isoform 3 of Mediator of RNA polymerase II transcription subunit 23	IPI00413272	0.355	0.08948	8	3	7	7
586	Isoform 1 of Cytoplasmic FMR1-interacting protein 1	IPI00644231	0.355	0.08948	8	3	5	9
587	Protein ERGIC-53	IPI00026530	0.355	0.08948	7	4	6	8
588	Exportin-1	IPI00298961	0.347	0.09093	37	24	29	37
589	Isoform 1 of Mitochondrial import receptor subunit TOM40 homolog	IPI00014053	0.346	0.09097	7	5	7	8
590	Isoform 1 of Pleiotropic regulator 1	IPI00002624	0.346	0.09097	6	6	10	5
591	Isoform 1 of Disco-interacting protein 2 homolog B	IPI00465045	0.346	0.09097	5	7	9	6
592	Isoform 1 of Importin-4	IPI00156374	0.346	0.09097	8	4	7	8
593	60S ribosomal protein L24	IPI00306332	0.346	0.09097	7	5	9	6
594	Non-functional aryl hydrocarbon receptor interacting protein (Fragment)	IPI00925804	0.346	0.09097	7	5	7	8
595	26S proteasome non-ATPase regulatory subunit 10	IPI00003565	0.346	0.09097	7	5	8	7
596	Cell division protein kinase 5	IPI00023530	0.346	0.09097	5	7	8	7
597	GrpE protein homolog 1, mitochondrial	IPI00029557	0.346	0.09097	5	7	8	7
598	Heterogeneous nuclear ribonucleoprotein C-like 1	IPI00027569	0.344	0.09130	19	12	20	15
599	Pre-mRNA-splicing factor ATP-dependent RNA helicase PRP16	IPI00294211	0.344	0.09130	19	12	17	18
600	Isoform 1 of 5'(3')-deoxyribonucleotidase, cytosolic type	IPI00005573	0.338	0.09171	7	6	7	9
601	Eukaryotic translation initiation factor 2 subunit 3	IPI00297982	0.338	0.09171	8	5	6	10
602	Heat shock 70 kDa protein 12A	IPI00011932	0.338	0.09171	8	5	6	10
603	MKI67 FHA domain-interacting nucleolar phosphoprotein	IPI00154590	0.338	0.09171	8	5	9	7
604	Isoform 2 of Protein disulfide-isomerase A6	IPI00299571	0.338	0.09171	9	4	7	9
605	Isoform 1 of Enhancer of mRNA-decapping protein 4	IPI00376317	0.334	0.09257	14	20	19	19
606	Small nuclear ribonucleoprotein Sm D2	IPI00017963	0.330	0.09275	8	6	10	7
607	Splicing factor 3A subunit 1	IPI00017451	0.330	0.09275	8	6	10	7
608	Isoform 1 of LETM1 and EF-hand domain-containing protein 1, mitochondrial	IPI00017592	0.330	0.09275	9	5	8	9
609	Trifunctional enzyme subunit alpha, mitochondrial	IPI00031522	0.326	0.09346	22	15	21	20
610	Isoform 1 of Hydroxyacyl-coenzyme A dehydrogenase, mitochondrial	IPI00294398	0.324	0.09346	7	8	10	8
611	Isoform Long of Long-chain-fatty-acid--CoA ligase 4	IPI00029737	0.324	0.09346	9	6	5	13
612	Peptidyl-prolyl cis-trans isomerase NIMA-interacting 1	IPI00013723	0.324	0.09346	8	7	8	10
613	Cytochrome c oxidase subunit 6C	IPI00015972	0.320	0.09436	0	0	3	3
614	Isoform Beta of Nucleolar and coiled-body phosphoprotein 1	IPI00216654	0.320	0.09436	0	0	3	3
615	Keratin, type II cytoskeletal 5	IPI00009867	0.320	0.09436	0	0	3	3
616	Mediator of RNA polymerase II transcription subunit 14	IPI00297191	0.320	0.09436	0	2	4	0
617	Mediator of RNA polymerase II transcription subunit 12	IPI00004068	0.320	0.09436	2	2	2	4
618	Isoform 7 of Protein BAT2-like 2	IPI00083708	0.320	0.09436	2	2	4	0
619	Isoform 1 of Phosphatidylinositol-3,4,5-trisphosphate 5-phosphatase 2	IPI00016932	0.320	0.09436	0	2	4	0
620	Junction plakoglobin	IPI00554711	0.320	0.09436	2	0	3	3
621	Isoform 1 of Helicase-like transcription factor	IPI00339381	0.320	0.09436	2	2	2	4
622	Probable RNA-binding protein 19	IPI00000686	0.320	0.09436	0	0	3	3
623	Glycylpeptide N-tetradecanoyltransferase 2	IPI00030223	0.320	0.09436	2	0	4	2
624	Retinol dehydrogenase 13	IPI00301204	0.320	0.09436	2	0	4	2
625	Isoform 1 of Fermitin family homolog 2	IPI00000856	0.320	0.09436	0	2	2	4
626	Isoform 1 of Huntingtin-interacting protein K	IPI00335001	0.320	0.09436	2	2	3	3
627	Isoform 1 of Protein fto	IPI00028277	0.320	0.09436	2	0	4	2
628	Heat shock protein beta (Fragment)	IPI00411633	0.320	0.09436	0	0	0	4
629	Ribosomal protein S6 kinase alpha-6	IPI00007123	0.320	0.09436	2	2	2	4
630	Vacuolar ATPase assembly integral membrane protein VMA21	IPI00146447	0.320	0.09436	0	2	4	0
631	Small nuclear ribonucleoprotein G	IPI00016572	0.320	0.09436	0	0	4	0
632	39S ribosomal protein L16, mitochondrial	IPI00000821	0.320	0.09436	2	0	3	3
633	Ubiquitin-conjugating enzyme E2 S	IPI00217949	0.320	0.09436	2	2	3	3
634	Ras-related C3 botulinum toxin substrate 2	IPI00010270	0.320	0.09436	0	0	4	0
635	Isoform 2 of 39S ribosomal protein L55, mitochondrial	IPI00419626	0.320	0.09436	0	0	0	4
636	Serine/threonine-protein kinase 38-like	IPI00237011	0.320	0.09436	2	0	4	0
637	Isoform 1 of RNA polymerase II-associated protein 1	IPI00402657	0.320	0.09436	2	0	3	3
638	NADH dehydrogenase [ubiquinone] 1 alpha subcomplex subunit 6	IPI00419266	0.320	0.09436	2	2	3	3
639	Endoplasmic reticulum resident protein 44	IPI00401264	0.320	0.09436	2	0	4	2
640	Ras-related protein Rab-5A	IPI00023510	0.320	0.09436	2	2	3	3
641	Isoform 2 of NudC domain-containing protein 1	IPI00306398	0.320	0.09436	2	0	4	0
642	Isoform 1 of Peroxisomal membrane protein PEX16	IPI00006722	0.320	0.09436	0	2	4	2
643	UPF0670 protein C8orf55	IPI00171421	0.320	0.09436	2	0	3	3
644	Isoform 1 of COP9 signalosome complex subunit 1	IPI00156282	0.320	0.09436	2	0	2	4
645	Vesicle-associated membrane protein 8	IPI00030911	0.320	0.09436	2	0	4	2
646	Isoform 2 of Diphosphoinositol polyphosphate phosphohydrolase 2	IPI00021408	0.320	0.09436	0	2	3	3
647	Similar to Ankyrin repeat and FYVE domain-containing protein 1	IPI00159899	0.320	0.09436	0	2	2	4
648	WD repeat domain 57 (U5 snRNP specific), isoform CRA_b	IPI00385642	0.320	0.09436	2	0	3	3
649	Profilin	IPI00107555	0.320	0.09436	0	0	4	0
650	Ras-related protein Ral-A	IPI00217519	0.320	0.09436	2	2	2	4
651	Stromal cell-derived factor 2	IPI00293167	0.320	0.09436	0	0	3	3
652	60S ribosomal protein L35	IPI00412607	0.320	0.09436	0	0	4	0
653	Isoform 1 of TIP41-like protein	IPI00745568	0.320	0.09436	0	0	4	0
654	Isoform 1 of DNA repair protein complementing XP-G cells	IPI00477535	0.320	0.09436	0	2	3	3
655	Histone H1x	IPI00021924	0.320	0.09436	0	2	2	4
656	Histidine triad nucleotide-binding protein 2, mitochondrial	IPI00000335	0.320	0.09436	2	0	3	3
657	Isoform 1 of Glucosamine-6-phosphate isomerase 2	IPI00550894	0.320	0.09436	0	0	3	3
658	Dolichyldiphosphatase 1	IPI00329410	0.320	0.09436	0	0	2	4

No.	Description	Accession number	STN[1]	p-Value[1]	Con_A[2]	Con_B[2]	5FU_A[2]	5FU_B[2]
659	OTU domain-containing protein 6B	IPI00182180	0.320	0.09436	2	2	3	3
660	Isoform 1 of Rab GTPase-binding effector protein 1	IPI00293009	0.320	0.09436	2	2	4	2
661	Uncharacterized protein C10orf58	IPI00296190	0.320	0.09436	0	0	3	3
662	Isoform DFF45 of DNA fragmentation factor subunit alpha (Fragment)	IPI00010882	0.320	0.09436	2	0	2	4
663	Isoform 2 of Protein diaphanous homolog 2	IPI00514075	0.320	0.09436	0	0	0	4
664	Fumarylacetoacetate hydrolase domain-containing protein 2A	IPI00329742	0.320	0.09436	2	2	2	4
665	Isoform 1 of Mortality factor 4-like protein 1	IPI00409675	0.320	0.09436	2	0	4	0
666	39S ribosomal protein L40, mitochondrial	IPI00099871	0.320	0.09436	0	0	4	2
667	PIH1 domain-containing protein 1	IPI00550995	0.320	0.09436	2	0	3	3
668	RAB4A, member RAS oncogene family variant	IPI00480056	0.320	0.09436	0	0	3	3
669	Xaa-Pro dipeptidase	IPI00257882	0.320	0.09436	2	0	4	0
670	ADP-ribosylation factor-like protein 8A	IPI00060031	0.320	0.09436	0	0	3	3
671	Putative uncharacterized protein DKFZp686H16220	IPI00552191	0.320	0.09436	0	2	2	4
672	Chloride intracellular channel protein 3	IPI00000692	0.320	0.09436	0	2	3	3
673	SNARE-associated protein Snapin	IPI00018331	0.320	0.09436	0	0	3	3
674	Protein FADD	IPI00011919	0.320	0.09436	0	2	4	2
675	Isoform 1 of Putative adenosylhomocysteinase 2	IPI00182938	0.320	0.09436	0	2	3	3
676	DDB1- and CUL4-associated factor 7	IPI00006754	0.320	0.09436	2	2	4	2
677	Targeting protein for Xklp2	IPI00008477	0.320	0.09436	0	0	3	3
678	Isoform 2 of Kinesin-like protein KIF2C	IPI00216113	0.320	0.09436	2	0	4	0
679	cDNA FLJ53160, highly similar to Zyxin	IPI00871311	0.320	0.09436	2	0	3	3
680	Isoform 1 of Pericentriolar material 1 protein	IPI00006213	0.320	0.09436	0	0	4	0
681	Isoform 1 of AF4/FMR2 family member 4	IPI00004344	0.320	0.09436	0	0	0	4
682	cDNA FLJ20242 fis, clone COLF6369	IPI00004901	0.320	0.09436	0	0	2	4
683	Inorganic pyrophosphatase	IPI00015018	0.319	0.11342	42	38	47	38
684	cDNA FLJ54492, highly similar to Eukaryotic translation initiation factor 4B	IPI00012079	0.318	0.11364	9	7	10	9
685	Histone deacetylase 1	IPI00013774	0.318	0.11364	8	8	10	9
686	Cleavage and polyadenylation specificity factor subunit 1	IPI00026219	0.312	0.11394	9	8	7	13
687	Isoform 1 of La-related protein 1	IPI00185919	0.312	0.11394	9	8	8	12
688	Proline synthetase co-transcribed homolog (Bacterial), isoform CRA_b	IPI00016346	0.312	0.11394	9	8	10	10
689	Ran GTPase-activating protein 1	IPI00294879	0.312	0.11394	7	10	11	9
690	T-complex protein 1 subunit eta	IPI00018465	0.309	0.11454	53	35	40	53
691	T-complex protein 1 subunit epsilon	IPI00010720	0.309	0.11454	27	17	26	22
692	Multifunctional protein ADE2	IPI00217223	0.307	0.11469	26	19	28	21
693	Isoform 1 of Myosin-Ib	IPI00376344	0.306	0.11469	11	7	8	13
694	Adenine phosphoribosyltransferase	IPI00218693	0.306	0.11469	10	8	11	10
695	Aldehyde dehydrogenase X, mitochondrial	IPI00103467	0.306	0.11469	8	10	11	10
696	Protein DEK	IPI00020021	0.301	0.11502	8	11	12	10
697	Isoform 1 of Transmembrane and coiled-coil domain-containing protein 1	IPI00026111	0.301	0.11539	3	0	5	0
698	magnesium transporter protein 1	IPI00301202	0.301	0.11539	3	0	2	5
699	Isoform 3 of HEAT repeat-containing protein 5B	IPI00333696	0.301	0.11539	3	2	3	4
700	Isoform 1 of Wings apart-like protein homolog	IPI00375330	0.301	0.11539	3	2	5	2
701	Isoform 1 of Splicing factor, arginine/serine-rich 15	IPI00181702	0.301	0.11539	3	0	2	5
702	Isoform A of Kinesin light chain 1	IPI00020096	0.301	0.11539	3	2	2	5
703	Isoform 1 of Liprin-alpha-1	IPI00163496	0.301	0.11539	3	0	3	4
704	Isoform 2 of cAMP-dependent protein kinase catalytic subunit alpha	IPI00217960	0.301	0.11539	3	0	5	2
705	Isoform 1 of 28S ribosomal protein S35, mitochondrial	IPI00073779	0.301	0.11539	0	3	2	5
706	Tetratricopeptide repeat protein 35	IPI00014149	0.301	0.11539	2	3	2	5
707	Lamina-associated polypeptide 2, isoform alpha	IPI00216230	0.301	0.11539	3	2	3	4
708	Isoform 1 of Actin-like protein 6A	IPI00003627	0.301	0.11539	3	0	5	0
709	Ras-related protein Rab-22A	IPI00007756	0.301	0.11539	3	0	4	3
710	Isoform 3 of Anamorsin	IPI00025333	0.301	0.11539	2	3	4	3
711	Isoform Long of FAS-associated factor 1	IPI00070643	0.301	0.11539	3	0	4	3
712	Isoform 1 of Oligoribonuclease, mitochondrial (Fragment)	IPI00032830	0.301	0.11539	2	3	3	4
713	Isoform 1 of Rab GTPase-activating protein 1	IPI00016702	0.301	0.11539	3	2	3	4
714	Complex I intermediate-associated protein 30, mitochondrial	IPI00032560	0.301	0.11539	2	3	3	4
715	Isoform 1 of Peptidyl-prolyl cis-trans isomerase SDCCAG10	IPI00025174	0.301	0.11539	2	3	4	3
716	PRKC apoptosis WT1 regulator protein	IPI00001871	0.301	0.11539	3	2	4	3
717	Isoform 1 of Drebrin	IPI00003406	0.301	0.11539	3	0	3	4
718	Isoform Short of Proteasome subunit alpha type-1	IPI00016832	0.297	0.11554	12	8	10	13
719	V-type proton ATPase subunit E 1	IPI00003856	0.297	0.11554	10	10	12	11
720	Isoleucyl-tRNA synthetase, mitochondrial	IPI00017283	0.288	0.11610	11	11	11	14
721	cDNA FLJ77422, highly similar to Homo sapiens RNA binding protein	IPI00011268	0.288	0.11610	12	10	11	14
722	Isoform 1 of Squamous cell carcinoma antigen recognized by T-cells 3	IPI00006025	0.288	0.11610	13	9	12	13
723	WD repeat-containing protein 18	IPI00032533	0.286	0.11632	4	2	2	6
724	Programmed cell death protein 6	IPI00025277	0.286	0.11632	3	3	3	5
725	ATPase ASNA1	IPI00013466	0.286	0.11632	4	2	4	4
726	Phosphoribosyl pyrophosphate synthase-associated protein 2	IPI00003168	0.286	0.11632	4	2	3	5
727	28S ribosomal protein S34, mitochondrial	IPI00169413	0.286	0.11632	4	0	4	4
728	tRNA (guanine-N(7)-)-methyltransferase	IPI00290184	0.286	0.11632	4	2	4	4
729	Isoform 1 of Bifunctional coenzyme A synthase	IPI00184821	0.286	0.11632	3	3	4	4
730	Isoform 2 of Golgi apparatus protein 1	IPI00414717	0.286	0.11632	3	3	4	4
731	Isoform 1 of Translation initiation factor eIF-2B subunit delta	IPI00005979	0.286	0.11632	2	4	5	3
732	Isoform 1 of Nicalin	IPI00470649	0.286	0.11632	3	3	5	3
733	Condensin complex subunit 2	IPI00299507	0.286	0.11632	4	0	6	0
734	Protein SCO1 homolog, mitochondrial	IPI00027233	0.286	0.11632	3	3	3	5
735	Putative uncharacterized protein DKFZp781K1356	IPI00412545	0.286	0.11632	4	0	5	3
736	Ketosamine-3-kinase	IPI00099986	0.286	0.11632	4	0	3	5
737	Isoform 1 of DDRGK domain-containing protein 1	IPI00028387	0.286	0.11632	4	0	5	3
738	Putative uncharacterized protein DKFZp761L1314 (Fragment)	IPI00183297	0.286	0.11632	3	3	4	4
739	Protein FAM98B	IPI00167572	0.286	0.11632	3	3	4	4
740	Isoform 2 of Nitrilase homolog 1	IPI00023779	0.286	0.11632	0	4	4	4
741	Isoform 1 of Nucleoside diphosphate kinase A	IPI00012048	0.285	0.12932	30	27	30	31
742	Heterogeneous nuclear ribonucleoprotein L	IPI00027834	0.285	0.12932	32	25	30	31
743	Protein mago nashi homolog 2	IPI00059292	0.284	0.12932	13	10	16	10
744	Isoform 1 of Nuclear autoantigenic sperm protein	IPI00179953	0.284	0.12932	15	8	14	12
745	Ribosome biogenesis protein BMS1 homolog	IPI00006099	0.281	0.12962	11	13	13	14
746	Isoform p150 of Dynactin subunit 1	IPI00029485	0.281	0.12962	15	9	14	13
747	Histone H1.5	IPI00217468	0.281	0.12962	12	12	15	12
748	Peroxiredoxin-4	IPI00011937	0.277	0.12965	13	12	14	14
749	Isoform 2 of Nucleoporin NUP188 homolog	IPI00385001	0.274	0.12988	13	13	14	15
750	Isoform 5 of E3 ubiquitin-protein ligase UBR4	IPI00180305	0.274	0.12988	18	8	14	15
751	Isoform 1 of Serine/threonine-protein phosphatase 2A 65 kDa regulatory subunit A beta isoform	IPI00294178	0.273	0.12988	5	2	5	4
752	twinfilin-1	IPI00183508	0.273	0.12988	5	2	0	7
753	Protein BUD31 homolog	IPI00013180	0.273	0.12988	3	4	3	6

No.	Description	Accession number	STN[1]	p-Value[1]	Con_A[2]	Con_B[2]	5FU_A[2]	5FU_B[2]
754	Isoform 3 of Fermitin family homolog 1	IPI00220602	0.273	0.12988	0	5	3	6
755	Negative elongation factor B	IPI00103483	0.273	0.12988	3	4	5	4
756	Isoform 1 of 60S ribosome subunit biogenesis protein NIP7 homolog	IPI00007175	0.273	0.12988	4	3	4	5
757	Macrophage-capping protein	IPI00027341	0.273	0.12988	5	2	6	3
758	Nucleolysin TIAR	IPI00005615	0.273	0.12988	4	3	6	3
759	Desmoglein-2	IPI00028931	0.273	0.12988	4	3	4	5
760	cDNA FLJ61629, highly similar to Clathrin interactor 1	IPI00291930	0.273	0.12988	4	3	4	5
761	Isoform 1 of Dehydrogenase/reductase SDR family member 7	IPI00006957	0.273	0.12988	4	3	4	5
762	39S ribosomal protein L18, mitochondrial	IPI00160421	0.273	0.12988	4	3	3	6
763	Microtubule-associated protein RP/EB family member 1	IPI00017596	0.271	0.12988	14	13	16	14
764	Methionyl-tRNA synthetase, cytoplasmic	IPI00008240	0.268	0.13018	22	6	14	17
765	60S ribosomal protein L22	IPI00219153	0.263	0.13051	4	4	4	6
766	Proteasome inhibitor PI31 subunit	IPI00009949	0.263	0.13051	4	4	6	4
767	WASH complex subunit strumpellin	IPI00029175	0.263	0.13051	4	4	3	7
768	Cyclin-G-associated kinase	IPI00298949	0.263	0.13051	4	4	3	7
769	Pterin-4-alpha-carbinolamine dehydratase	IPI00218568	0.263	0.13051	4	4	5	5
770	Isoform Membrane-bound of Catechol O-methyltransferase	IPI00011284	0.263	0.13051	3	5	6	4
771	Isoform 1 of Rab3 GTPase-activating protein catalytic subunit	IPI00014235	0.263	0.13051	5	3	4	6
772	40S ribosomal protein S25	IPI00012750	0.263	0.13051	4	4	5	5
773	Isoform 1 of Polyadenylate-binding protein 4	IPI00012726	0.263	0.13051	4	4	6	4
774	Metaxin-2	IPI00025717	0.263	0.13051	4	4	7	3
775	Isoform 2 of Leucyl-cystinyl aminopeptidase	IPI00221240	0.263	0.13051	6	0	4	6
776	Ribonucleases P/MRP protein subunit POP1	IPI00293331	0.263	0.13051	5	3	4	6
777	serine/threonine-protein phosphatase PP1-alpha catalytic subunit isoform 3	IPI00027423	0.263	0.13051	6	2	7	3
778	Sorting and assembly machinery component 50 homolog	IPI00412713	0.263	0.13051	5	3	5	5
779	Isoform 3 of Drebrin-like protein	IPI00101968	0.263	0.13051	4	4	5	5
780	Protein DJ-1	IPI00298547	0.262	0.13867	15	15	19	14
781	Isoform 2 of Voltage-dependent anion-selective channel protein 2	IPI00024145	0.262	0.13867	16	14	16	17
782	Isoform 2 of U5 small nuclear ribonucleoprotein 200 kDa helicase	IPI00168235	0.260	0.13878	37	39	38	42
783	TUBA1C protein	IPI00166768	0.258	0.13893	46	32	46	36
784	Isoform 1 of U5 small nuclear ribonucleoprotein 200 kDa helicase	IPI00420014	0.255	0.13911	88	73	76	90
785	Prostaglandin E synthase 3	IPI00015029	0.254	0.13930	4	5	9	2
786	Isoform 3 of Transcription elongation factor SPT6	IPI00456683	0.254	0.13930	6	3	6	5
787	Component of gems 4	IPI00027717	0.254	0.13930	5	4	4	7
788	Isoform 1 of Specifically androgen-regulated gene protein	IPI00028392	0.254	0.13930	6	3	7	4
789	Mitochondrial import receptor subunit TOM70	IPI00015602	0.254	0.13930	7	2	5	6
790	E3 ubiquitin-protein ligase HECTD1	IPI00328911	0.254	0.13930	4	5	5	6
791	DNA topoisomerase 1	IPI00413611	0.254	0.13930	7	0	7	4
792	7-dehydrocholesterol reductase	IPI00294501	0.254	0.13930	3	6	6	5
793	Core histone macro-H2A.2	IPI00220994	0.254	0.13930	4	5	6	5
794	Tyrosyl-tRNA synthetase, mitochondrial	IPI00165092	0.254	0.13930	7	0	8	3
795	NAD-dependent malic enzyme, mitochondrial	IPI00011201	0.254	0.13930	6	3	7	4
796	Isoform 1 of RuvB-like 1	IPI00021187	0.252	0.13930	20	14	20	17
797	DNA replication licensing factor MCM2	IPI00184330	0.252	0.13930	21	13	19	18
798	Small subunit processome component 20 homolog	IPI00004970	0.250	0.13952	24	11	18	20
799	Isoform 1 of Structural maintenance of chromosomes protein 2	IPI00007927	0.250	0.13952	19	16	20	18
800	Isoform 2 of Splicing factor 1	IPI00294627	0.246	0.13971	7	3	5	7
801	GTP-binding protein SAR1a	IPI00015954	0.246	0.13971	4	6	8	4
802	TDP43	IPI00025815	0.246	0.13971	8	2	9	3
803	Fructose-bisphosphate aldolase	IPI00418262	0.246	0.13971	8	2	7	5
804	U6 snRNA-associated Sm-like protein LSm4	IPI00294955	0.246	0.13971	3	7	6	6
805	Succinyl-CoA ligase [GDP-forming] subunit beta, mitochondrial	IPI00096066	0.246	0.13971	6	4	7	5
806	40S ribosomal protein S4, X isoform	IPI00217030	0.239	0.14548	21	19	23	20
807	WD repeat-containing protein 75	IPI00217240	0.239	0.14548	6	5	6	7
808	Cytochrome c oxidase subunit 5B, mitochondrial	IPI00021785	0.239	0.14548	5	6	6	7
809	Cytochrome b-c1 complex subunit Rieske, mitochondrial	IPI00026964	0.239	0.14548	5	6	6	7
810	Mortality factor 4-like protein 2	IPI00014174	0.239	0.14548	6	5	5	8
811	Isoform 1 of Ubiquitin-conjugating enzyme E2 variant 1	IPI00019599	0.239	0.14548	3	8	8	5
812	Structural maintenance of chromosomes protein 1A	IPI00291939	0.236	0.14559	22	20	25	20
813	Isoform 1 of Cytoskeleton-associated protein 5	IPI00028275	0.234	0.14559	23	20	21	25
814	Ubiquitin carboxyl-terminal hydrolase 11	IPI00184533	0.233	0.14574	7	5	7	7
815	Dual specificity mitogen-activated protein kinase kinase 2	IPI00003783	0.233	0.14574	9	3	5	9
816	Isoform 1 of Nck-associated protein 1	IPI00031982	0.233	0.14574	7	5	7	7
817	Isoform 1 of Apoptotic chromatin condensation inducer in the nucleus	IPI00007334	0.233	0.14574	6	6	8	6
818	Isoform 1 of Abhydrolase domain-containing protein 14B	IPI00063827	0.233	0.14574	6	6	7	7
819	Membrane-associated progesterone receptor component 1	IPI00220739	0.233	0.14574	6	6	7	7
820	Isoform 1 of E3 ubiquitin-protein ligase BRE1B	IPI00162563	0.233	0.14574	5	7	5	9
821	Inosine triphosphate pyrophosphatase	IPI00018783	0.228	0.14987	9	4	9	6
822	Asparaginyl-tRNA synthetase, cytoplasmic	IPI00306960	0.228	0.14987	8	5	9	6
823	22 kDa protein	IPI00219910	0.228	0.14987	6	7	8	7
824	Probable methylthioribulose-1-phosphate dehydratase	IPI00549730	0.228	0.14987	6	7	9	6
825	tropomyosin alpha-1 chain isoform 2	IPI00000230	0.228	0.14987	6	7	8	7
826	Dihydrolipoyllysine-residue acetyltransferase component of pyruvate dehydrogenase complex	IPI00021338	0.228	0.14987	7	6	7	8
827	26S protease regulatory subunit S10B	IPI00021926	0.223	0.15002	10	4	9	7
828	Isoform 1 of Fermitin family homolog 1	IPI00304754	0.223	0.15002	7	7	5	11
829	Tubulin-specific chaperone A	IPI00217236	0.223	0.15002	7	7	8	8
830	CCAAT/enhancer-binding protein zeta	IPI00306723	0.223	0.15002	9	5	7	9
831	Phosphomevalonate kinase	IPI00220648	0.223	0.15002	7	7	8	8
832	Isoform Short of Adenosine kinase	IPI00234368	0.218	0.15416	12	3	10	7
833	60S ribosomal protein L36	IPI00216237	0.218	0.15416	8	7	9	8
834	Isoform Long of Tight junction protein ZO-1	IPI00216219	0.218	0.15416	6	9	6	11
835	Sepiapterin reductase	IPI00017469	0.218	0.15416	10	5	10	7
836	Isoform 1 of 3-hydroxyacyl-CoA dehydrogenase type-2	IPI00017726	0.214	0.15430	32	25	35	25
837	Isoform 2 of Dedicator of cytokinesis protein 7	IPI00183572	0.214	0.15438	8	8	8	10
838	cDNA FLJ55599, highly similar to DNA replication licensing factor MCM3	IPI00013214	0.214	0.15438	11	5	9	9
839	Cytoplasmic dynein 1 light intermediate chain 1	IPI00007675	0.214	0.15438	10	6	9	9
840	Histidyl-tRNA synthetase, cytoplasmic	IPI00021808	0.214	0.15438	10	6	11	7
841	Leucine-rich PPR motif-containing protein, mitochondrial	IPI00783271	0.210	0.15691	91	56	74	77
842	Isoform 1 of Bcl-2-associated transcription factor 1	IPI00006079	0.210	0.15691	7	10	11	8
843	Isoform 1 of Cell division cycle and apoptosis regulator protein 1	IPI00217357	0.210	0.15691	10	7	10	9
844	Glycogen phosphorylase, brain form	IPI00004358	0.206	0.15695	14	4	10	10
845	Bifunctional aminoacyl-tRNA synthetase	IPI00013452	0.205	0.15874	36	29	37	31
846	Protein disulfide-isomerase A4	IPI00009904	0.205	0.15874	39	26	42	26
847	Isoform 1 of Serine/arginine repetitive matrix protein 2	IPI00782992	0.203	0.15874	30	37	35	35
848	Isoform 1 of Splicing factor, arginine/serine-rich 7	IPI00003377	0.203	0.15874	9	10	14	7

No.	Description	Accession number	STN[1]	p-Value[1]	Con_A[2]	Con_B[2]	5FU_A[2]	5FU_B[2]
849	40S ribosomal protein S9	IPI00221088	0.203	0.15874	11	8	10	11
850	Isoform 4 of Serine/threonine-protein phosphatase 6 regulatory subunit 3	IPI00019540	0.203	0.15874	10	9	10	11
851	Ras-related protein Rap-1b	IPI00015148	0.199	0.15877	12	8	16	6
852	Isoform 1 of Dipeptidyl peptidase 3	IPI00020672	0.199	0.15877	15	5	10	12
853	Phenylalanyl-tRNA synthetase beta chain	IPI00300074	0.199	0.15877	11	9	11	11
854	Enoyl-CoA hydratase, mitochondrial	IPI00024993	0.199	0.15877	11	9	13	9
855	Ras-related protein Rab-5C	IPI00016339	0.196	0.16019	10	11	15	8
856	Translin	IPI00018768	0.196	0.16019	11	10	10	13
857	Isoform 1 of Tropomyosin alpha-4 chain	IPI00010779	0.196	0.16019	13	8	11	12
858	Cytochrome c oxidase subunit 4 isoform 1, mitochondrial	IPI00006579	0.194	0.16030	13	9	14	10
859	Isoform 1 of Acidic leucine-rich nuclear phosphoprotein 32 family member B	IPI00007423	0.194	0.16030	12	10	12	12
860	Sideroflexin-1	IPI00009368	0.191	0.16149	14	9	14	11
861	Isoform 2 of Apoptosis inhibitor 5	IPI00554742	0.191	0.16149	14	9	11	14
862	Chromobox protein homolog 3	IPI00297579	0.191	0.16149	11	12	13	12
863	Elongation factor 2	IPI00186290	0.190	0.16149	55	28	38	48
864	Dolichyl-diphosphooligosaccharide--protein glycosyltransferase subunit 2	IPI00028635	0.190	0.16149	47	36	44	42
865	Vacuolar protein sorting-associated protein 35	IPI00018931	0.188	0.16149	17	7	13	13
866	Cytochrome b-c1 complex subunit 2, mitochondrial	IPI00305383	0.188	0.16149	14	10	13	13
867	Transcription factor A, mitochondrial	IPI00020928	0.188	0.16149	13	11	16	10
868	Isoform 1 of UTP--glucose-1-phosphate uridylyltransferase	IPI00329331	0.188	0.16149	15	9	14	12
869	Isoform 1 of Cytosol aminopeptidase	IPI00419237	0.188	0.16149	16	8	15	11
870	Ubiquitin carboxyl-terminal hydrolase 14	IPI00219913	0.186	0.16216	13	12	14	13
871	Isoform 2 of Splicing factor 3B subunit 3	IPI00179138	0.184	0.16220	8	18	15	13
872	Eukaryotic translation initiation factor 3 subunit I	IPI00012795	0.184	0.16220	17	9	15	13
873	Collapsin response mediator protein 4 long variant	IPI00029111	0.184	0.16220	13	13	13	15
874	Eukaryotic translation initiation factor 3 subunit E	IPI00013068	0.184	0.16220	16	10	15	13
875	Kinesin-1 heavy chain	IPI00012837	0.181	0.16268	14	13	15	14
876	Eukaryotic translation initiation factor 6	IPI00010105	0.181	0.16268	15	12	15	14
877	Lamin-B2	IPI00009771	0.181	0.16268	15	12	15	14
878	Isoform 1 of Vesicle-associated membrane protein-associated protein B/C	IPI00006211	0.179	0.16268	17	11	17	13
879	Mitochondrial 2-oxoglutarate/malate carrier protein	IPI00219729	0.179	0.16268	15	13	14	16
880	26S proteasome non-ATPase regulatory subunit 6	IPI00014151	0.177	0.16335	17	12	17	14
881	60S ribosomal protein L6	IPI00329389	0.176	0.16335	16	14	20	12
882	Isoform Long of Trifunctional purine biosynthetic protein adenosine-3	IPI00025273	0.174	0.16443	18	13	16	17
883	Coronin-1B	IPI00007058	0.174	0.16443	15	16	16	17
884	Isoform 1 of Apoptosis-inducing factor 1, mitochondrial	IPI00000690	0.170	0.16510	23	10	14	21
885	Isoform 1 of Cytosolic acyl coenzyme A thioester hydrolase	IPI00010415	0.170	0.16510	18	15	18	17
886	cDNA FLJ45706 fis, clone FEBRA2028457, highly similar to Nucleolin	IPI00444262	0.169	0.16510	22	12	19	17
887	ribonucleoprotein PTB-binding 1	IPI00217661	0.165	0.16577	2	0	3	0
888	Tax1-binding protein 3	IPI00005585	0.165	0.16577	0	0	3	0
889	Huntingtin	IPI00002335	0.165	0.16577	0	0	3	0
890	Putative uncharacterized protein CNOT1	IPI00032299	0.165	0.16577	2	2	0	3
891	Probable saccharopine dehydrogenase	IPI00329600	0.165	0.16577	2	0	3	2
892	Isoform 1 of Solute carrier family 12 member 4	IPI00021057	0.165	0.16577	2	2	2	3
893	DNA-directed RNA polymerase III subunit RPC1	IPI00024163	0.165	0.16577	2	0	3	2
894	cDNA FLJ45400 fis, clone BRHIP3028570	IPI00151888	0.165	0.16577	2	2	0	3
895	Aldo-keto reductase family 1 member C3	IPI00291483	0.165	0.16577	0	0	0	3
896	NADH dehydrogenase [ubiquinone] 1 beta subcomplex subunit 8, mitochondrial	IPI00028883	0.165	0.16577	0	0	3	0
897	AP-3 complex subunit beta-2	IPI00005793	0.165	0.16577	0	0	2	3
898	Isoform 1 of Fanconi anemia group D2 protein	IPI00075081	0.165	0.16577	0	0	0	3
899	Isoform 1 of Phosphatidylinositol glycan anchor biosynthesis class U protein	IPI00026044	0.165	0.16577	2	2	2	3
900	Isoamyl acetate-hydrolyzing esterase 1 homolog	IPI00419194	0.165	0.16577	2	2	3	2
901	LanC-like protein 1	IPI00005724	0.165	0.16577	2	2	2	3
902	Isoform 4 of Mitochondrial fission factor	IPI00024627	0.165	0.16577	2	0	3	2
903	Putative uncharacterized protein DOCK6	IPI00184772	0.165	0.16577	2	2	3	0
904	UPF0364 protein C6orf211	IPI00002270	0.165	0.16577	0	0	3	2
905	Putative uncharacterized protein ALB	IPI00022434	0.165	0.16577	2	0	2	3
906	Histone deacetylase 4	IPI00010088	0.165	0.16577	0	0	3	2
907	Sororin	IPI00061989	0.165	0.16577	0	2	3	2
908	Protein phosphatase 1 regulatory subunit 11	IPI00030355	0.165	0.16577	0	0	3	0
909	Ubiquitin-like protein 4A	IPI00005658	0.165	0.16577	0	2	0	3
910	Retinal rod rhodopsin-sensitive cGMP 3',5'-cyclic phosphodiesterase subunit delta	IPI00015161	0.165	0.16577	0	2	2	3
911	Dolichyl-phosphate beta-glucosyltransferase	IPI00002506	0.165	0.16577	0	2	3	0
912	Translation initiation factor eIF-2B subunit beta	IPI00028083	0.165	0.16577	2	2	0	3
913	synembryn-A	IPI00100106	0.165	0.16577	0	0	2	3
914	Isoform A of Probable cation-transporting ATPase 13A1	IPI00034277	0.165	0.16577	0	0	3	0
915	Isoform 1 of Melanoma inhibitory activity protein 3	IPI00455473	0.165	0.16577	0	0	3	0
916	Isoform 1 of Kinesin-like protein KIF21A	IPI00425404	0.165	0.16577	0	0	0	3
917	Isoform 1 of Transcription termination factor 2	IPI00290812	0.165	0.16577	0	0	0	3
918	Pre-mRNA cleavage complex 2 protein Pcf11	IPI00016387	0.165	0.16577	0	0	2	3
919	Isoform 1 of Uncharacterized protein C1orf77	IPI00300990	0.165	0.16577	0	2	2	3
920	Creatine kinase B-type	IPI00022977	0.165	0.16577	2	0	3	0
921	Dehydrogenase/reductase SDR family member 7B	IPI00550165	0.165	0.16577	2	0	2	3
922	NADP-dependent malic enzyme	IPI00008215	0.165	0.16577	2	0	3	2
923	Isoform Alpha-1 of Protein phosphatase 1A	IPI00020950	0.165	0.16577	2	2	2	3
924	Isoform 1 of KDEL motif-containing protein 2	IPI00143921	0.165	0.16577	0	0	3	0
925	Lipoma-preferred partner	IPI00023704	0.165	0.16577	0	2	3	2
926	Ferritin heavy chain	IPI00554521	0.165	0.16577	0	0	3	0
927	Isoform 2 of Ral GTPase-activating protein subunit alpha-1	IPI00456722	0.165	0.16577	2	0	3	2
928	Isoform 2 of Carbohydrate kinase domain-containing protein	IPI00645172	0.165	0.16577	0	0	3	2
929	Ribonuclease P protein subunit p38	IPI00019195	0.165	0.16577	0	0	2	3
930	Isoform 1 of IST1 homolog	IPI00024660	0.165	0.16577	2	0	0	3
931	Isoform 1 of Alpha-adducin	IPI00019901	0.165	0.16577	2	0	3	2
932	39S ribosomal protein L38, mitochondrial	IPI00783656	0.165	0.16577	0	0	0	3
933	Isoform Beta-1C of Integrin beta-1	IPI00217561	0.165	0.16577	2	0	3	0
934	Isoform 1 of Alanine aminotransferase 2	IPI00152432	0.165	0.16577	2	2	2	3
935	Fascin	IPI00163187	0.165	0.16577	2	0	0	3
936	cDNA FLJ10824 fis, clone NT2RP4001086 (Fragment)	IPI00294810	0.165	0.16577	0	2	2	3
937	Isoform 4 of Zinc finger protein 638	IPI00178953	0.165	0.16577	2	0	0	3
938	Isoform 2 of Oxidoreductase HTATIP2	IPI00383665	0.165	0.16577	2	2	3	2
939	Isoform 1 of Ubiquitin-conjugating enzyme E2 Z	IPI00011996	0.165	0.16577	2	2	3	0
940	U4/U6.U5 small nuclear ribonucleoprotein 27 kDa protein	IPI00017289	0.165	0.16577	2	0	2	3
941	EPS8L2 protein	IPI00414315	0.165	0.16577	2	2	0	3
942	Isoform 1 of MYC-induced nuclear antigen	IPI00216737	0.165	0.16577	2	0	3	0
943	Isoform 1 of Transcriptional repressor p66-alpha	IPI00410330	0.165	0.16577	0	2	2	3

No.	Description	Accession number	STN[1]	p-Value[1]	Con_A[2]	Con_B[2]	5FU_A[2]	5FU_B[2]
944	Isoform 1 of Regulator of microtubule dynamics protein 3	IPI00410079	0.165	0.16577	0	0	3	2
945	Isoform B of Smoothelin	IPI00024007	0.165	0.16577	0	0	0	3
946	39S ribosomal protein L14, mitochondrial	IPI00418290	0.165	0.16577	0	0	0	3
947	Isoform 1 of Ubiquitin carboxyl-terminal hydrolase 28	IPI00045496	0.165	0.16577	2	2	2	3
948	Isoform 2 of NAD-dependent deacetylase sirtuin-5	IPI00010331	0.165	0.16577	0	2	3	0
949	Synaptojanin-2-binding protein	IPI00299193	0.165	0.16577	2	2	3	0
950	U8 snoRNA-decapping enzyme	IPI00783497	0.165	0.16577	0	0	2	3
951	RNA-binding protein 7	IPI00001134	0.165	0.16577	2	0	3	0
952	Isoform 1 of Probable threonyl-tRNA synthetase 2, cytoplasmic	IPI00328082	0.165	0.16577	0	0	0	3
953	Similar to Protein SAAL1. Isoform 2	IPI00304935	0.165	0.16577	0	2	2	3
954	Isoform 1 of Protein CIP2A	IPI00154283	0.165	0.16577	0	0	0	3
955	Isoform 1 of Cell division cycle protein 23 homolog	IPI00005822	0.165	0.16577	0	0	3	2
956	Isoform 2 of Phosphoenolpyruvate carboxykinase [GTP], mitochondrial	IPI00384116	0.165	0.16577	2	2	3	2
957	Isoform 1 of Beta-enolase	IPI00218474	0.165	0.16577	2	2	3	0
958	Cytoplasmic aconitate hydratase	IPI00008485	0.165	0.16577	0	0	3	2
959	Putative transferase C1orf69, mitochondrial	IPI00145260	0.165	0.16577	0	0	3	0
960	Ribosomal RNA-processing protein 8	IPI00304932	0.165	0.16577	0	0	2	3
961	UDP-glucose 4-epimerase	IPI00553131	0.165	0.16577	2	2	0	3
962	AFG3-like protein 2	IPI00001091	0.165	0.16577	2	0	0	3
963	Coiled-coil domain-containing protein 86	IPI00012199	0.165	0.16577	0	0	0	3
964	My002 protein	IPI00023584	0.165	0.16577	2	0	3	2
965	ADP-ribosylation factor-like protein 6	IPI00021685	0.165	0.16577	2	0	2	3
966	Isoform 1 of RNA polymerase II subunit A C-terminal domain phosphatase SSU72	IPI00023556	0.165	0.16577	2	2	3	0
967	DUS1L protein (Fragment)	IPI00329754	0.165	0.16577	2	0	3	2
968	Isoform 2 of Arf-GAP domain and FG repeats-containing protein 1	IPI00304693	0.165	0.16577	0	0	3	2
969	SAFB-like transcription modulator isoform b	IPI00019996	0.165	0.16577	0	2	0	3
970	Isoform 2 of G-protein coupled receptor 56	IPI00397949	0.165	0.16577	0	0	0	3
971	Isoform 1 of H/ACA ribonucleoprotein complex subunit 1	IPI00302176	0.165	0.16577	2	2	3	0
972	Isoform 3 of Tyrosine-protein kinase Fyn	IPI00166845	0.165	0.16577	0	0	3	0
973	Major centromere autoantigen B	IPI00010388	0.165	0.16577	2	0	2	3
974	Melanoma-associated antigen G1	IPI00217104	0.165	0.16577	0	0	2	3
975	KIF1-binding protein	IPI00477355	0.165	0.16577	0	0	0	3
976	Exocyst complex component 8	IPI00028264	0.165	0.16577	0	0	0	3
977	Nuclear receptor coactivator 5	IPI00288941	0.165	0.16577	0	0	0	3
978	Microfibrillar-associated protein 1	IPI00022790	0.165	0.16577	0	0	2	3
979	Isoform 1 of Vacuolar protein sorting-associated protein 16 homolog	IPI00305438	0.165	0.16577	0	0	2	3
980	Isoform 5 of Brain-specific angiogenesis inhibitor 1-associated protein 2	IPI00180292	0.165	0.16577	0	0	3	2
981	Putative uncharacterized protein LCMT1	IPI00296370	0.165	0.16577	0	0	0	3
982	Protein kinase, AMP-activated, alpha 1 catalytic subunit, isoform CRA_b	IPI00061282	0.165	0.16577	0	0	3	0
983	Isoform 2 of 6-phosphofructo-2-kinase/fructose-2,6-biphosphatase 2	IPI00220808	0.165	0.16577	0	0	0	3
984	Aldose 1-epimerase	IPI00060200	0.165	0.16577	0	0	0	3
985	Proline-rich protein PRCC	IPI00294618	0.165	0.16577	0	0	3	0
986	cDNA FLJ44925 fis, clone BRAMY3014613, highly similar to Homo sapiens SH3-domain binding	IPI00444788	0.165	0.16577	2	0	3	0
987	Kinesin light chain 2	IPI00021634	0.165	0.16577	0	0	0	3
988	Isoform 1 of Interferon regulatory factor 2-binding protein 2	IPI00376199	0.165	0.16577	0	0	3	2
989	Integrin alpha-2	IPI00013744	0.165	0.16577	0	0	0	3
990	Stress-induced-phosphoprotein 1	IPI00013894	0.162	0.17642	20	19	24	17
991	Condensin complex subunit 1	IPI00299524	0.159	0.17661	24	17	20	23
992	zinc finger protein 294	IPI00783835	0.154	0.17687	3	2	4	2
993	Replication protein A 14 kDa subunit	IPI00017373	0.154	0.17687	2	3	4	2
994	Isoform 1 of Dual specificity mitogen-activated protein kinase kinase 3	IPI00218857	0.154	0.17687	3	0	4	2
995	Isoform 2 of NADH dehydrogenase [ubiquinone] flavoprotein 3, mitochondrial	IPI00291016	0.154	0.17687	3	2	3	3
996	Splicing factor 3B subunit 4	IPI00017339	0.154	0.17687	3	0	3	3
997	24 kDa protein	IPI00397611	0.154	0.17687	3	0	3	3
998	Isoform 2 of Isochorismatase domain-containing protein 2, mitochondrial	IPI00003031	0.154	0.17687	0	3	3	3
999	Pseudouridylate synthase 7 homolog	IPI00044761	0.154	0.17687	3	0	2	4
1000	Glutamate-rich WD repeat-containing protein 1	IPI00027831	0.154	0.17687	3	2	4	2
1001	Serine/threonine-protein phosphatase 2A 65 kDa regulatory subunit A alpha isoform	IPI00554737	0.154	0.17687	3	2	4	0
1002	Importin 5	IPI00639960	0.154	0.17687	0	3	3	3
1003	Major vault protein	IPI00000105	0.154	0.17687	3	0	2	4
1004	Isoform 1 of CUGBP Elav-like family member 1	IPI00034015	0.154	0.17687	2	3	4	2
1005	NADPH--cytochrome P450 reductase	IPI00470467	0.154	0.17687	3	2	3	3
1006	Isoform Alpha of Nuclear inhibitor of protein phosphatase 1	IPI00030383	0.154	0.17687	3	2	3	3
1007	Isoform SMN of Survival motor neuron protein	IPI00003394	0.154	0.17687	3	0	4	0
1008	Plakophilin-3	IPI00026952	0.154	0.17687	0	3	3	3
1009	Isoform 1 of Phosphoribosyl pyrophosphate synthase-associated protein 1	IPI00291578	0.154	0.17687	3	0	3	3
1010	Acetyl-coenzyme A synthetase, cytoplasmic	IPI00413730	0.154	0.17687	3	2	4	2
1011	Bis(5'-nucleosyl)-tetraphosphatase [asymmetrical]	IPI00221231	0.154	0.17687	3	0	2	4
1012	Peptidylprolyl isomerase domain and WD repeat-containing protein 1	IPI00149650	0.154	0.17687	3	0	4	0
1013	RNA 3'-terminal phosphate cyclase-like protein	IPI00294229	0.154	0.17687	3	2	4	2
1014	Huntingtin-interacting protein 1	IPI00782965	0.154	0.17687	3	2	2	4
1015	M-phase phosphoprotein 6	IPI00016074	0.154	0.17687	2	3	3	3
1016	Isoform 1 of Ran-binding protein 3	IPI00026337	0.154	0.17687	2	3	3	3
1017	LIM and cysteine-rich domains protein 1	IPI00303258	0.154	0.17687	3	0	4	2
1018	Isoform 1 of Uncharacterized protein KIAA0528	IPI00465142	0.154	0.17687	3	0	4	0
1019	Isoform 2 of Syntaxin-5	IPI00386786	0.154	0.17687	3	2	3	3
1020	Isoform 1 of Glomulin	IPI00074604	0.154	0.17687	3	0	4	2
1021	cDNA FLJ20475 fis, clone KAT07206	IPI00183065	0.154	0.17687	2	3	3	3
1022	La-related protein 7	IPI00294742	0.154	0.17687	2	3	3	3
1023	Isoform 1 of SET domain-containing protein 3	IPI00165026	0.154	0.17687	3	2	4	2
1024	Splicing factor, arginine/serine-rich 11	IPI00464952	0.154	0.17687	3	2	3	3
1025	Isoform 1 of Extended synaptotagmin-1	IPI00022143	0.154	0.18763	26	19	23	24
1026	60S acidic ribosomal protein P0	IPI00008530	0.149	0.18789	31	19	26	26
1027	Platelet-activating factor acetylhydrolase IB subunit gamma	IPI00014808	0.146	0.18800	3	3	5	2
1028	cohesin subunit SA-2 isoform a	IPI00470883	0.146	0.18800	4	0	2	5
1029	Talin-2	IPI00219299	0.146	0.18800	2	4	3	4
1030	Cell division cycle 5-like protein	IPI00465294	0.146	0.18800	4	0	2	5
1031	39S ribosomal protein L44, mitochondrial	IPI00009680	0.146	0.18800	3	3	2	5
1032	Isoform 1 of RNA-binding protein with serine-rich domain 1	IPI00033561	0.146	0.18800	3	3	5	2
1033	Pyridoxine-5'-phosphate oxidase	IPI00018272	0.146	0.18800	3	3	4	3
1034	Importin subunit alpha-1	IPI00303292	0.146	0.18800	4	2	3	4
1035	Isoform 1 of STE20-like serine/threonine-protein kinase	IPI00022827	0.146	0.18800	0	4	4	3
1036	Isoform 2 of Leucine-rich repeat flightless-interacting protein 1	IPI00006207	0.146	0.18800	3	3	3	4
1037	Pre-mRNA-splicing factor CWC22 homolog	IPI00177381	0.146	0.18800	2	4	3	4
1038	Glutamate--cysteine ligase catalytic subunit	IPI00215768	0.146	0.18800	3	3	3	4

No.	Description	Accession number	STN[1]	p-Value[1]	Con_A[2]	Con_B[2]	5FU_A[2]	5FU_B[2]
1039	Isoform 2 of Ubiquitin-conjugating enzyme E2 K	IPI00019894	0.146	0.18800	3	3	5	2
1040	Isoform 1 of Telomeric repeat-binding factor 2	IPI00024214	0.146	0.18800	4	2	5	0
1041	RNA-binding protein NOB1	IPI00022373	0.146	0.18800	4	0	4	3
1042	Vacuolar protein sorting-associated protein 4A	IPI00411356	0.146	0.18800	3	3	3	4
1043	Putative uncharacterized protein PYCR2	IPI00335061	0.146	0.18800	3	3	3	4
1044	Isoform 1 of Beta-galactosidase	IPI00441344	0.146	0.18800	3	3	4	3
1045	Heat shock 70 kDa protein 14	IPI00292499	0.146	0.18800	4	2	3	4
1046	Nuclear RNA export factor 1	IPI00033153	0.146	0.18800	4	2	4	3
1047	Putative uncharacterized protein ZNF326	IPI00337602	0.146	0.18800	2	4	3	4
1048	Annexin A5	IPI00329801	0.143	0.19184	30	27	33	26
1049	GTP-binding nuclear protein Ran	IPI00643041	0.143	0.19184	28	29	31	28
1050	Rho GDP-dissociation inhibitor 1	IPI00003815	0.142	0.19184	29	29	35	25
1051	Ribosome biogenesis protein BRX1 homolog	IPI00181728	0.139	0.19195	4	3	4	4
1052	Isoform 4 of Dipeptidyl peptidase 9	IPI00604483	0.139	0.19195	5	0	4	4
1053	Ribosomal protein L1	IPI00035167	0.139	0.19195	5	2	4	4
1054	Isoform 4 of Nucleoporin NDC1	IPI00003455	0.139	0.19195	3	4	0	6
1055	NADH-ubiquinone oxidoreductase chain 5	IPI00008511	0.139	0.19195	5	2	3	5
1056	Importin subunit alpha-4	IPI00012578	0.139	0.19195	5	2	4	4
1057	UDP-galactose-4-epimerase	IPI00030229	0.139	0.19195	4	3	5	3
1058	Probable ATP-dependent RNA helicase DDX23	IPI00006725	0.139	0.19195	4	3	4	4
1059	Copine-1	IPI00018452	0.139	0.19195	3	4	4	4
1060	Cation-independent mannose-6-phosphate receptor	IPI00289819	0.139	0.19195	4	3	5	3
1061	Isoform 3 of Centromere protein V	IPI00376481	0.139	0.19195	5	2	6	2
1062	Similar to nonhistone chromosomal protein HMG-1	IPI00418184	0.139	0.19195	4	3	3	5
1063	Isoform 1 of Serine/threonine-protein phosphatase 6 catalytic subunit	IPI00012970	0.139	0.19195	4	3	5	3
1064	Ewing sarcoma breakpoint region 1 isoform 1	IPI00009841	0.139	0.19195	4	3	5	3
1065	60 kDa heat shock protein, mitochondrial	IPI00784154	0.135	0.19567	145	95	121	122
1066	Isoform 1 of Large proline-rich protein BAT2	IPI00010700	0.134	0.19567	2	6	3	6
1067	Helicase SKI2W	IPI00414819	0.134	0.19567	6	2	5	4
1068	Tricarboxylate transport protein, mitochondrial	IPI00294159	0.134	0.19567	3	5	5	4
1069	Biliverdin reductase A	IPI00294158	0.134	0.19567	5	3	4	5
1070	Isoform 1 of Nucleolar protein 6	IPI00152890	0.134	0.19567	5	3	4	5
1071	Isoform 1 of ATP synthase subunit d, mitochondrial	IPI00220487	0.134	0.19567	3	5	6	3
1072	Importin-8	IPI00007401	0.134	0.19567	5	3	3	6
1073	Mannosyl-oligosaccharide glucosidase	IPI00328170	0.134	0.19567	4	4	5	4
1074	Isoform 1 of Apoptosis-inducing factor 2	IPI00013909	0.134	0.19567	4	4	5	4
1075	DNA polymerase subunit gamma-1	IPI00004317	0.134	0.19567	4	4	3	6
1076	Isoform 1 of Acyl-coenzyme A thioesterase 2, mitochondrial	IPI00220906	0.134	0.19567	6	2	4	5
1077	cDNA FLJ56357, highly similar to Homo sapiens apolipoprotein A-I binding protein (APOA1BP), mRNA	IPI00168479	0.134	0.19567	5	3	6	3
1078	ATP-dependent Clp protease ATP-binding subunit clpX-like, mitochondrial	IPI00008728	0.134	0.19567	6	2	5	4
1079	Serine/threonine-protein kinase OSR1	IPI00010080	0.134	0.19567	3	5	5	4
1080	Deoxyribonucleoside 5'-monophosphate N-glycosidase	IPI00007926	0.134	0.19567	4	4	6	3
1081	Ribonuclease H2 subunit A	IPI00290192	0.134	0.19567	4	4	3	6
1082	Dual specificity mitogen-activated protein kinase kinase 1	IPI00219604	0.134	0.19567	5	3	5	4
1083	Protein transport protein Sec23B	IPI00017376	0.134	0.19567	6	0	5	4
1084	Isoform 1 of Protein kinase C and casein kinase substrate in neurons protein 2	IPI00027009	0.134	0.19567	4	4	5	4
1085	Zinc finger protein ZPR1	IPI00025244	0.134	0.19567	4	4	5	4
1086	Alpha-taxilin	IPI00470779	0.134	0.19567	4	4	5	4
1087	Isoform C of Lethal(2) giant larvae protein homolog 2	IPI00465050	0.129	0.19917	5	4	5	5
1088	WD40 repeat-containing protein SMU1	IPI00305833	0.129	0.19917	3	6	4	6
1089	Phosphatidylinositol transfer protein alpha isoform	IPI00216048	0.129	0.19917	6	3	4	6
1090	Uncharacterized protein C7orf50	IPI00031651	0.129	0.19917	4	5	3	7
1091	Ras-related protein Rap-2c	IPI00009607	0.129	0.19917	5	4	6	4
1092	Isoform 1 of 39S ribosomal protein L47, mitochondrial	IPI00030820	0.129	0.19917	4	5	4	6
1093	Isoform 2 of Nipped-B-like protein	IPI00026466	0.129	0.19917	7	2	5	5
1094	NADH dehydrogenase [ubiquinone] 1 alpha subcomplex subunit 10, mitochondrial	IPI00029561	0.129	0.19917	5	4	5	5
1095	NADH dehydrogenase [ubiquinone] flavoprotein 2, mitochondrial	IPI00291328	0.129	0.19917	5	4	6	4
1096	Isoform 2 of Chromodomain-helicase-DNA-binding protein 2	IPI00023109	0.129	0.19917	5	4	5	5
1097	Nucleoporin NUP53	IPI00329650	0.129	0.19917	4	5	4	6
1098	Isoform 2 of Ubiquitin carboxyl-terminal hydrolase 47	IPI00165528	0.129	0.19917	4	5	6	4
1099	Importin 5	IPI00514205	0.129	0.19917	6	3	5	5
1100	SH3 domain-binding glutamic acid-rich-like protein	IPI00025318	0.129	0.19917	5	4	6	4
1101	Cell division protein kinase 6	IPI00023529	0.125	0.20193	5	5	6	5
1102	Vitamin K epoxide reductase complex subunit 1-like protein 1	IPI00166079	0.125	0.20193	5	5	5	6
1103	Ras-related protein Rab-1B	IPI00008964	0.125	0.20193	5	5	5	6
1104	Isoform Long of Double-stranded RNA-binding protein Staufen homolog 1	IPI00000001	0.125	0.20193	5	5	5	6
1105	Putative uncharacterized protein DKFZp313O211	IPI00552186	0.125	0.20193	3	7	7	4
1106	13kDa differentiation-associated protein variant (Fragment)	IPI00005966	0.125	0.20193	5	5	7	4
1107	Scaffold attachment factor B2	IPI00005648	0.125	0.20193	4	6	5	6
1108	U3 small nucleolar ribonucleoprotein protein IMP3	IPI00019488	0.125	0.20193	5	5	5	6
1109	1-phosphatidylinositol-4,5-bisphosphate phosphodiesterase beta-3	IPI00010400	0.125	0.20193	7	3	5	6
1110	28S ribosomal protein S23, mitochondrial	IPI00032881	0.125	0.20193	6	4	6	5
1111	Chromobox protein homolog 5	IPI00024662	0.121	0.20383	5	6	5	7
1112	Isoform 1 of Interferon-inducible double stranded RNA-dependent protein kinase activator A	IPI00021167	0.121	0.20383	7	4	8	4
1113	Hsc70-interacting protein	IPI00032826	0.121	0.20383	6	5	6	6
1114	cDNA FLJ56414, highly similar to Homo sapiens proline-, glutamic acid-, leucine-rich protein 1 (PELP1)	IPI00006702	0.121	0.20383	7	4	7	5
1115	WD repeat-containing protein 11	IPI00412224	0.121	0.20383	5	6	4	8
1116	Eukaryotic translation initiation factor 3 subunit K	IPI00033143	0.121	0.20383	7	4	7	5
1117	Seryl-tRNA synthetase, cytoplasmic	IPI00220637	0.121	0.20383	6	5	5	7
1118	Actin-related protein 2/3 complex subunit 2	IPI00005161	0.121	0.20383	6	5	4	8
1119	Pyrroline-5-carboxylate reductase 2	IPI00470610	0.121	0.20383	6	5	6	6
1120	Isoform 1 of Chromosome-associated kinesin KIF4A	IPI00178150	0.121	0.20383	5	6	5	7
1121	Isoform Long of Ubiquitin carboxyl-terminal hydrolase 5	IPI00024664	0.118	0.20565	9	3	5	8
1122	Isoform 2 of NSFL1 cofactor p47	IPI00022830	0.118	0.20565	7	5	7	6
1123	Telomeric repeat-binding factor 2-interacting protein 1	IPI00008961	0.118	0.20565	6	6	7	6
1124	Putative uncharacterized protein ZFR	IPI00748303	0.118	0.20565	9	3	5	8
1125	Isoform 1 of Medium-chain specific acyl-CoA dehydrogenase, mitochondrial	IPI00005040	0.118	0.20565	7	5	8	5
1126	Isoform 1 of Secretory carrier-associated membrane protein 3	IPI00306382	0.118	0.20565	6	6	5	8
1127	Vacuolar protein-sorting-associated protein 25	IPI00031655	0.115	0.20748	8	5	9	5
1128	Isoform 1 of RNA-binding protein 14	IPI00013174	0.115	0.20748	8	5	8	6
1129	SRA stem-loop-interacting RNA-binding protein, mitochondrial	IPI00009922	0.115	0.20748	4	9	7	7
1130	Isoleucyl-tRNA synthetase	IPI00514082	0.115	0.20748	9	4	7	7
1131	Transmembrane emp24 domain-containing protein 2	IPI00016608	0.112	0.20878	8	6	9	6

No.	Description	Accession number	STN[1]	p-Value[1]	Con_A[2]	Con_B[2]	5FU_A[2]	5FU_B[2]
1132	Putative uncharacterized protein ATP5J2	IPI00219291	0.112	0.20878	6	8	7	8
1133	Myosin-Ie	IPI00329672	0.112	0.20878	7	7	6	9
1134	Mitochondrial import inner membrane translocase subunit TIM44	IPI00306516	0.112	0.20878	7	7	8	7
1135	Crk-like protein	IPI00004839	0.112	0.20878	7	7	6	9
1136	Ribonuclease P protein subunit p30	IPI00019196	0.110	0.21068	7	8	9	7
1137	Isoform 1 of Methionine adenosyltransferase 2 subunit beta	IPI00002324	0.110	0.21068	9	6	7	9
1138	28S ribosomal protein S22, mitochondrial	IPI00013146	0.110	0.21068	8	7	6	10
1139	Isoform 1 of Ribose-phosphate pyrophosphokinase 2	IPI00219617	0.110	0.21068	9	6	9	7
1140	Exosome complex exonuclease RRP4	IPI00015905	0.108	0.21202	10	6	9	8
1141	Isoform 1 of 3,2-trans-enoyl-CoA isomerase, mitochondrial	IPI00300567	0.108	0.21202	8	8	7	10
1142	Cytochrome b-c1 complex subunit 1, mitochondrial	IPI00013847	0.108	0.21202	8	8	10	7
1143	Trifunctional enzyme subunit beta, mitochondrial	IPI00022793	0.108	0.21202	9	7	8	9
1144	cDNA FLJ56825, highly similar to WD repeat protein 57	IPI00006723	0.108	0.21202	9	7	9	8
1145	Sodium/potassium-transporting ATPase subunit beta-3	IPI00008167	0.108	0.21202	11	5	9	8
1146	Isoform 1 of Tensin-3	IPI00658152	0.106	0.21303	10	7	8	10
1147	TOB3	IPI00045921	0.106	0.21303	11	6	8	10
1148	cDNA FLJ35809 fis, clone TESTI2006016, highly similar to Eukaryotic translation initiation factor 3	IPI00647650	0.104	0.21370	10	8	8	11
1149	Vesicular integral-membrane protein VIP36	IPI00009950	0.104	0.21370	8	10	10	9
1150	Palmitoyl-protein thioesterase 1	IPI00002412	0.104	0.21370	9	9	12	7
1151	Isoform 1 of Voltage-dependent anion-selective channel protein 3	IPI00031804	0.102	0.21448	6	13	10	10
1152	DNA-directed RNA polymerase II subunit RPB2	IPI00027808	0.100	0.21492	10	10	11	10
1153	Ras GTPase-activating protein-binding protein 1	IPI00012442	0.100	0.21492	13	7	11	10
1154	Isoform 2 of 6-phosphofructokinase, muscle type	IPI00219585	0.100	0.21492	10	10	12	9
1155	Isoform 2 of Myosin-VI	IPI00008455	0.100	0.21492	9	11	15	6
1156	Thymidylate kinase	IPI00013862	0.100	0.21492	11	9	10	11
1157	Nucleolar pre-ribosomal-associated protein 1	IPI00297241	0.097	0.21608	12	10	11	12
1158	Proteasome subunit alpha type-4	IPI00299155	0.097	0.21608	13	9	10	13
1159	SWI/SNF-related matrix-associated actin-dependent regulator of chromatin subfamily A member 5	IPI00297211	0.096	0.21664	13	10	11	13
1160	Aconitate hydratase, mitochondrial	IPI00017855	0.096	0.21664	14	9	16	8
1161	Stomatin-like protein 2	IPI00334190	0.091	0.21753	19	8	17	11
1162	Condensin complex subunit 3	IPI00106495	0.091	0.21753	15	12	14	14
1163	Isoform 2 of Myosin-Ic	IPI00010418	0.090	0.21783	19	9	14	15
1164	Isoform Long of Splicing factor, proline- and glutamine-rich	IPI00010740	0.089	0.21805	17	12	16	14
1165	Isoform 2 of Annexin A2	IPI00418169	0.088	0.21824	17	13	16	15
1166	Nicotinamide phosphoribosyltransferase	IPI00018873	0.087	0.21880	18	13	15	17
1167	Hepatoma-derived growth factor	IPI00020956	0.086	0.21906	17	15	15	18
1168	Isoform 1 of UDP-glucose:glycoprotein glucosyltransferase 1	IPI00024466	0.084	0.21969	19	16	16	20
1169	Sodium/potassium-transporting ATPase subunit alpha-2	IPI00003021	0.080	0.22029	21	19	21	20
1170	Chloride intracellular channel protein 1	IPI00010896	0.080	0.22040	26	15	23	19
1171	Delta(3,5)-Delta(2,4)-dienoyl-CoA isomerase, mitochondrial	IPI00011416	0.080	0.22040	18	23	20	22
1172	Proteasome activator complex subunit 1	IPI00479722	0.079	0.22043	22	20	22	21
1173	40S ribosomal protein S14	IPI00026271	0.076	0.22070	27	20	25	23
1174	Isoform 1 of Keratin, type I cytoskeletal 13	IPI00009866	0.076	0.22073	33	15	30	19
1175	HEAT repeat-containing protein 1	IPI00024279	0.075	0.22081	29	21	28	23
1176	Peroxiredoxin-6	IPI00220301	0.000	0.22163	64	53	66	51
1177	Staphylococcal nuclease domain-containing protein 1	IPI00140420	0.000	0.22163	45	32	37	40
1178	NAD(P) transhydrogenase, mitochondrial	IPI00337541	0.000	0.22163	27	33	33	27
1179	60S ribosomal protein L15	IPI00470528	0.000	0.22163	22	18	16	24
1180	Isoform 1 of 26S proteasome non-ATPase regulatory subunit 1	IPI00299608	0.000	0.22163	24	10	14	20
1181	Isoform 1 of Nuclear pore membrane glycoprotein 210	IPI00291755	0.000	0.22163	21	19	20	20
1182	DNA replication licensing factor MCM5	IPI00018350	0.000	0.22163	19	24	22	21
1183	40S ribosomal protein S7	IPI00013415	0.000	0.22163	13	5	9	9
1184	Tripeptidyl-peptidase 2	IPI00020416	0.000	0.22163	24	15	17	22
1185	Estradiol 17-beta-dehydrogenase 12	IPI00007676	0.000	0.22163	23	16	22	17
1186	Short heat shock protein 60 Hsp60s2	IPI00076042	0.000	0.22163	10	5	8	7
1187	60S ribosomal protein L38	IPI00215790	0.000	0.22163	13	12	13	12
1188	Isoform 1 of Far upstream element-binding protein 1	IPI00375441	0.000	0.22163	22	9	15	16
1189	60S ribosomal protein L13a	IPI00304612	0.000	0.22163	0	0	2	0
1190	Isoform Gamma-1 of Serine/threonine-protein phosphatase PP1-gamma catalytic subunit	IPI00005705	0.000	0.22163	19	11	13	17
1191	Solute carrier family 4 sodium bicarbonate cotransporter member 7	IPI00021058	0.000	0.22163	17	13	16	14
1192	Lupus La protein	IPI00009032	0.000	0.22163	18	11	15	14
1193	60S acidic ribosomal protein P2	IPI00008529	0.000	0.22163	17	12	16	13
1194	arylacetamide deacetylase-like 1 isoform b	IPI00002230	0.000	0.22163	11	11	9	13
1195	Dihydropyrimidinase-like 2	IPI00106642	0.000	0.22163	13	6	9	10
1196	Isoform 1 of NADH-cytochrome b5 reductase 3	IPI00328415	0.000	0.22163	13	11	14	10
1197	Isoform 1 of Peripherin	IPI00013164	0.000	0.22163	17	10	13	14
1198	Wolframin	IPI00008711	0.000	0.22163	10	4	5	9
1199	Putative uncharacterized protein DKFZp451D234	IPI00031583	0.000	0.22163	17	13	14	16
1200	26S proteasome non-ATPase regulatory subunit 7	IPI00019927	0.000	0.22163	13	9	12	10
1201	Isoform 1 of Mitochondrial inner membrane protein	IPI00009960	0.000	0.22163	17	10	14	13
1202	Glutathione S-transferase omega-1	IPI00019755	0.000	0.22163	7	7	7	7
1203	Probable phosphoglycerate mutase 4	IPI00374975	0.000	0.22163	7	4	9	0
1204	Isocitrate dehydrogenase [NADP] cytoplasmic	IPI00027223	0.000	0.22163	13	13	14	12
1205	Splicing factor, arginine/serine-rich 2	IPI00005978	0.000	0.22163	13	11	15	9
1206	Protein transport protein Sec24C	IPI00024661	0.000	0.22163	10	4	5	9
1207	EH domain-containing protein 4	IPI00005578	0.000	0.22163	16	10	14	12
1208	Isoform 1 of Cullin-4B	IPI00179057	0.000	0.22163	14	6	7	13
1209	Transmembrane emp24 domain-containing protein 10	IPI00028055	0.000	0.22163	9	11	12	8
1210	165 kDa protein	IPI00240812	0.000	0.22163	7	9	9	7
1211	Isoform A1 of Tight junction protein ZO-2	IPI00003843	0.000	0.22163	10	11	10	11
1212	cDNA FLJ59211, highly similar to Glucosidase 2 subunit beta	IPI00026154	0.000	0.22163	17	10	13	14
1213	cysteinyl-tRNA synthetase, cytoplasmic isoform c	IPI00027443	0.000	0.22163	14	4	7	11
1214	Nuclear cap-binding protein subunit 1	IPI00019380	0.000	0.22163	7	3	4	6
1215	Isoform 1 of Polyadenylate-binding protein 2	IPI00005792	0.000	0.22163	0	0	2	0
1216	Isoform 1 of E3 ubiquitin-protein ligase RNF123	IPI00335085	0.000	0.22163	2	0	0	0
1217	NEDD8	IPI00020008	0.000	0.22163	6	2	4	4
1218	Proteasome subunit beta type-7	IPI00003217	0.000	0.22163	7	8	9	6
1219	Cytoplasmic dynein 1 light intermediate chain 2	IPI00011592	0.000	0.22163	7	5	6	6
1220	Src substrate cortactin	IPI00029601	0.000	0.22163	11	10	13	8
1221	26 kDa protein	IPI00219685	0.000	0.22163	8	6	8	6
1222	cDNA FLJ75085, highly similar to Homo sapiens glutaminyl-tRNA synthetase (QARS), mRNA	IPI00026665	0.000	0.22163	13	6	11	8
1223	Isoform 1 of Septin-2	IPI00014177	0.000	0.22163	9	4	7	6
1224	S-formylglutathione hydrolase	IPI00411706	0.000	0.22163	10	6	9	7
1225	Isoform 1 of RRP12-like protein	IPI00101186	0.000	0.22163	10	7	8	9

No.	Description	Accession number	STN[1]	p-Value[1]	Con_A[2]	Con_B[2]	5FU_A[2]	5FU_B[2]
1226	Ubiquitin-like modifier activating enzyme 1	IPI00552452	0.000	0.22163	10	6	9	7
1227	SUMO-activating enzyme subunit 2	IPI00023234	0.000	0.22163	11	4	5	10
1228	WD repeat-containing protein 3	IPI00009471	0.000	0.22163	4	2	4	2
1229	Insulin-like growth factor 2 mRNA-binding protein 1	IPI00008557	0.000	0.22163	13	9	12	10
1230	Pirin	IPI00012575	0.000	0.22163	6	4	5	5
1231	60S ribosomal protein L32	IPI00395998	0.000	0.22163	8	7	7	8
1232	LEM domain-containing protein 2	IPI00168336	0.000	0.22163	0	0	2	2
1233	Exportin-7	IPI00302458	0.000	0.22163	8	6	7	7
1234	Abhydrolase domain-containing protein 10, mitochondrial	IPI00020075	0.000	0.22163	9	6	8	7
1235	Inositol 1,4,5-trisphosphate receptor type 3	IPI00291607	0.000	0.22163	3	5	6	2
1236	Isoform 1 of Protein phosphatase 1 regulatory subunit 12A	IPI00183002	0.000	0.22163	10	4	7	7
1237	GTP cyclohydrolase 1 feedback regulatory protein	IPI00217253	0.000	0.22163	2	2	0	0
1238	Eukaryotic translation initiation factor 5	IPI00022648	0.000	0.22163	7	7	7	7
1239	Kinetochore-associated protein 1	IPI00001458	0.000	0.22163	0	0	2	2
1240	regulator of chromosome condensation 1 isoform a	IPI00001661	0.000	0.22163	6	8	7	7
1241	Isoform Long of Eukaryotic translation initiation factor 4H	IPI00014263	0.000	0.22163	7	5	6	6
1242	Isoform Long of Inositol 1,4,5-trisphosphate receptor type 2	IPI00031545	0.000	0.22163	5	0	2	5
1243	Tetratricopeptide repeat protein 37	IPI00005634	0.000	0.22163	8	4	7	5
1244	Isoform 1 of 39S ribosomal protein L4, mitochondrial	IPI00023334	0.000	0.22163	5	7	5	7
1245	Coactosin-like protein	IPI00017704	0.000	0.22163	4	0	4	0
1246	DCN1-like protein 5	IPI00165361	0.000	0.22163	7	7	9	5
1247	SUMO-conjugating enzyme UBC9	IPI00032957	0.000	0.22163	4	6	5	5
1248	Integrator complex subunit 2	IPI00477759	0.000	0.22163	2	2	0	0
1249	Eukaryotic translation initiation factor 2A	IPI00012462	0.000	0.22163	2	2	2	0
1250	Isoform 1 of Proteasome subunit beta type-8	IPI00000783	0.000	0.22163	7	8	9	6
1251	Succinate dehydrogenase [ubiquinone] iron-sulfur subunit, mitochondrial	IPI00294911	0.000	0.22163	7	8	8	7
1252	Tubulin beta-4 chain	IPI00023598	0.000	0.22163	0	0	0	2
1253	Isoform 1 of Trans-2,3-enoyl-CoA reductase	IPI00100656	0.000	0.22163	2	0	0	0
1254	Isoform 1 of Fragile X mental retardation syndrome-related protein 1	IPI00016249	0.000	0.22163	6	6	7	5
1255	Monocarboxylate transporter 1	IPI00024650	0.000	0.22163	0	2	0	0
1256	H/ACA ribonucleoprotein complex subunit 4	IPI00221394	0.000	0.22163	2	0	2	2
1257	ATP-dependent RNA helicase DHX29	IPI00217413	0.000	0.22163	5	3	5	3
1258	40S ribosomal protein S18	IPI00013296	0.000	0.22163	8	8	9	7
1259	Isoform 4 of Tubulin-specific chaperone D	IPI00030774	0.000	0.22163	5	6	5	6
1260	THO complex subunit 2	IPI00158615	0.000	0.22163	8	5	6	7
1261	Cullin-1	IPI00014310	0.000	0.22163	8	4	7	5
1262	Vesicle-fusing ATPase	IPI00006451	0.000	0.22163	4	0	3	3
1263	Isoform 1 of Methyl-CpG-binding domain protein 3	IPI00439194	0.000	0.22163	4	6	6	4
1264	Isoform 1 of Methylcrotonoyl-CoA carboxylase beta chain, mitochondrial	IPI00784044	0.000	0.22163	9	6	9	6
1265	60S ribosomal protein L13	IPI00465361	0.000	0.22163	2	0	0	0
1266	Isoform 2 of Mediator of DNA damage checkpoint protein 1	IPI00470805	0.000	0.22163	0	2	0	2
1267	Translational activator of cytochrome c oxidase 1	IPI00019903	0.000	0.22163	6	6	7	5
1268	Thiosulfate sulfurtransferase	IPI00216293	0.000	0.22163	5	5	3	7
1269	Coatomer subunit gamma-2	IPI00002557	0.000	0.22163	2	2	0	2
1270	cDNA FLJ54775, highly similar to Syntaxin-binding protein 2	IPI00019971	0.000	0.22163	7	2	3	6
1271	Transcription initiation factor IIB	IPI00022820	0.000	0.22163	9	0	4	7
1272	Isoform 1 of 1-phosphatidylinositol-4,5-bisphosphate phosphodiesterase gamma-1	IPI00016736	0.000	0.22163	7	3	5	5
1273	Isoform 2 of Condensin-2 complex subunit G2	IPI00396058	0.000	0.22163	0	2	0	0
1274	CDNA FLJ20030 fis, clone ADSU02156	IPI00014402	0.000	0.22163	0	8	2	8
1275	Adenylate kinase isoenzyme 6	IPI00032879	0.000	0.22163	3	5	4	4
1276	Isoform 1 of Glucosamine--fructose-6-phosphate aminotransferase [isomerizing] 1	IPI00217952	0.000	0.22163	5	4	3	6
1277	Isoform 1 of RNA-binding protein Musashi homolog 2	IPI00073713	0.000	0.22163	8	6	7	7
1278	Microsomal glutathione S-transferase 1	IPI00021805	0.000	0.22163	5	4	5	4
1279	Isoform 2 of Sacsin	IPI00784002	0.000	0.22163	0	2	0	2
1280	DNA mismatch repair protein Msh3	IPI00329605	0.000	0.22163	3	3	3	3
1281	Bifunctional methylenetetrahydrofolate dehydrogenase/cyclohydrolase, mitochondrial	IPI00011307	0.000	0.22163	4	3	4	3
1282	Aspartyl/asparaginyl beta-hydroxylase	IPI00294834	0.000	0.22163	4	4	3	5
1283	Phosphoinositide 3-kinase regulatory subunit 4	IPI00024006	0.000	0.22163	2	2	0	0
1284	Isoform 1 of Cleft lip and palate transmembrane protein 1-like protein	IPI00151358	0.000	0.22163	0	0	2	2
1285	Calcium-regulated heat stable protein 1	IPI00304409	0.000	0.22163	0	0	2	0
1286	Probable methyltransferase TARBP1	IPI00298447	0.000	0.22163	2	4	3	3
1287	Methyltransferase like 7B	IPI00090807	0.000	0.22163	4	4	4	4
1288	Isoform 1 of Cleavage stimulation factor subunit 2	IPI00013256	0.000	0.22163	4	2	4	2
1289	Isoform 2 of S-phase kinase-associated protein 1	IPI00172421	0.000	0.22163	2	3	2	3
1290	Putative uncharacterized protein ENSP00000382160	IPI00180956	0.000	0.22163	2	0	0	0
1291	Isoform 1 of Vacuolar protein sorting-associated protein 8 homolog	IPI00464985	0.000	0.22163	0	2	0	0
1292	Isoform 1 of Neuroblastoma-amplified sequence	IPI00333913	0.000	0.22163	0	2	2	0
1293	Synaptobrevin homolog YKT6	IPI00008569	0.000	0.22163	2	0	0	0
1294	Malectin	IPI00029046	0.000	0.22163	6	5	5	6
1295	Casein kinase II subunit beta	IPI00010865	0.000	0.22163	2	5	4	3
1296	SNW domain-containing protein 1	IPI00013830	0.000	0.22163	6	4	5	5
1297	Isoform 1 of Spermine synthase	IPI00005102	0.000	0.22163	7	2	6	3
1298	Isoform 1 of Elongation factor Ts, mitochondrial	IPI00021016	0.000	0.22163	5	4	5	4
1299	Heme-binding protein 1	IPI00148063	0.000	0.22163	3	4	4	3
1300	Mannose-P-dolichol utilization defect 1 protein	IPI00025292	0.000	0.22163	0	0	0	2
1301	Isoform 3 of Protein scribble homolog	IPI00410666	0.000	0.22163	3	3	0	4
1302	Probable fructose-2,6-bisphosphatase TIGAR	IPI00006907	0.000	0.22163	5	3	6	2
1303	39S ribosomal protein L53, mitochondrial	IPI00061531	0.000	0.22163	2	0	2	0
1304	Uncharacterized protein C2orf79	IPI00430803	0.000	0.22163	3	6	4	5
1305	Peptidyl-prolyl cis-trans isomerase FKBP11	IPI00009885	0.000	0.22163	2	0	0	0
1306	perilipin-3 isoform 3	IPI00106668	0.000	0.22163	7	6	7	6
1307	Chitobiosyldiphosphodolichol beta-mannosyltransferase	IPI00549761	0.000	0.22163	0	0	2	0
1308	Isoform 1 of Methylthioribose-1-phosphate isomerase	IPI00005948	0.000	0.22163	2	0	0	0
1309	Kinesin-like protein KIF13B	IPI00021753	0.000	0.22163	2	3	2	3
1310	DNA-directed RNA polymerase II subunit RPB3	IPI00018288	0.000	0.22163	5	3	4	4
1311	Adenylate kinase isoenzyme 4, mitochondrial	IPI00016568	0.000	0.22163	3	0	3	0
1312	Isoform 1 of CDP-diacylglycerol--inositol 3-phosphatidyltransferase	IPI00645518	0.000	0.22163	0	2	2	0
1313	Isoform 1 of Chromodomain-helicase-DNA-binding protein 1	IPI00297851	0.000	0.22163	3	0	3	2
1314	Mitochondrial ribonuclease P protein 1	IPI00099996	0.000	0.22163	5	2	5	2
1315	Protein tyrosine phosphatase type IVA 1	IPI00020164	0.000	0.22163	3	5	4	4
1316	UV excision repair protein RAD23 homolog A	IPI00008219	0.000	0.22163	0	2	0	2
1317	Isoform 2 of 1,2-dihydroxy-3-keto-5-methylthiopentene dioxygenase	IPI00470791	0.000	0.22163	3	2	2	3
1318	Isoform 1 of CLIP-associating protein 1	IPI00396279	0.000	0.22163	2	3	0	3
1319	Isoform 3 of Rapamycin-insensitive companion of mTOR	IPI00166528	0.000	0.22163	0	0	0	0
1320	Isoform Long of 60 kDa SS-A/Ro ribonucleoprotein	IPI00019450	0.000	0.22163	6	5	6	5

No.	Description	Accession number	STN[1]	p-Value[1]	Con_A[2]	Con_B[2]	5FU_A[2]	5FU_B[2]
1321	Coiled-coil domain-containing protein 124	IPI00060627	0.000	0.22163	0	3	3	0
1322	Isoform 1 of Protein transport protein Sec24A	IPI00873472	0.000	0.22163	2	0	2	2
1323	Isoform XLas-1 of Guanine nucleotide-binding protein G(s) subunit alpha isoforms XLas	IPI00095891	0.000	0.22163	3	0	2	3
1324	28 kDa heat- and acid-stable phosphoprotein	IPI00013297	0.000	0.22163	5	5	5	5
1325	YrdC domain-containing protein, mitochondrial	IPI00384180	0.000	0.22163	5	4	6	3
1326	Isoform 2 of Bromodomain adjacent to zinc finger domain protein 1A	IPI00383565	0.000	0.22163	3	4	0	5
1327	Splicing factor, arginine/serine-rich 4	IPI00000015	0.000	0.22163	0	0	0	0
1328	PDZ domain-containing protein 8	IPI00168698	0.000	0.22163	0	0	0	0
1329	Isoform 1 of Acetolactate synthase-like protein	IPI00554541	0.000	0.22163	0	0	0	2
1330	UPF0587 protein C1orf123	IPI00016605	0.000	0.22163	0	2	0	2
1331	Isoform 2 of Serine-protein kinase ATM	IPI00289986	0.000	0.22163	3	3	2	4
1332	39S ribosomal protein L49, mitochondrial	IPI00013195	0.000	0.22163	4	2	2	4
1333	Isoform 1 of Multidrug resistance-associated protein 4	IPI00006675	0.000	0.22163	0	0	0	0
1334	Isoform 1 of Caldesmon	IPI00014516	0.000	0.22163	3	2	2	3
1335	Eukaryotic translation initiation factor 1	IPI00015077	0.000	0.22163	3	3	4	2
1336	Peptidase M20 domain-containing protein 2	IPI00217852	0.000	0.22163	0	3	0	3
1337	Isocitrate dehydrogenase [NADP], mitochondrial	IPI00011107	0.000	0.22163	5	3	4	4
1338	NADH dehydrogenase [ubiquinone] 1 beta subcomplex subunit 7	IPI00219772	0.000	0.22163	0	0	0	0
1339	Isoform 1 of Deoxycytidylate deaminase	IPI00296863	0.000	0.22163	2	2	2	2
1340	Serine/threonine-protein phosphatase 1 regulatory subunit 10	IPI00298731	0.000	0.22163	5	2	3	4
1341	Isoform 1 of Probable E3 ubiquitin-protein ligase HERC4	IPI00333067	0.000	0.22163	3	0	3	2
1342	NADH dehydrogenase [ubiquinone] 1 beta subcomplex subunit 5, mitochondrial	IPI00013459	0.000	0.22163	5	4	6	3
1343	Isoform 1 of Metaxin-1	IPI00013678	0.000	0.22163	4	2	4	0
1344	cDNA FLJ54536, highly similar to Mitochondrial 28S ribosomal protein S27	IPI00022002	0.000	0.22163	4	0	4	2
1345	Calcium homeostasis endoplasmic reticulum protein	IPI00333010	0.000	0.22163	3	3	4	2
1346	39S ribosomal protein L48, mitochondrial	IPI00295066	0.000	0.22163	4	5	4	5
1347	RhoA activator C11orf59	IPI00016670	0.000	0.22163	2	0	0	0
1348	Toll-interacting protein	IPI00100154	0.000	0.22163	3	2	2	3
1349	N-alpha-acetyltransferase 20, NatB catalytic subunit	IPI00007174	0.000	0.22163	0	0	0	0
1350	1-acyl-sn-glycerol-3-phosphate acyltransferase epsilon	IPI00028491	0.000	0.22163	6	0	5	3
1351	Isoform 1 of Transmembrane protein 70, mitochondrial	IPI00106966	0.000	0.22163	0	2	0	0
1352	Transmembrane emp24 domain-containing protein 5	IPI00294472	0.000	0.22163	5	3	2	6
1353	Isoform 1 of Serine/threonine-protein phosphatase PGAM5, mitochondrial	IPI00788907	0.000	0.22163	0	0	0	0
1354	CTP synthase 2	IPI00645702	0.000	0.22163	5	0	2	5
1355	Isoform 1 of Partner of Y14 and mago	IPI00305092	0.000	0.22163	0	2	2	2
1356	Sperm-associated antigen 7	IPI00006863	0.000	0.22163	6	2	4	4
1357	Aminopeptidase B	IPI00642211	0.000	0.22163	2	5	3	4
1358	General transcription factor IIF subunit 1	IPI00017450	0.000	0.22163	6	6	6	6
1359	Vesicle-associated membrane protein 3	IPI00549343	0.000	0.22163	2	4	2	4
1360	Isoform 1 of DNA damage-binding protein 2	IPI00021518	0.000	0.22163	2	2	0	0
1361	Translocation protein SEC63 homolog	IPI00218922	0.000	0.22163	3	2	3	0
1362	Isoform 2 of Ubiquitin-associated domain-containing protein 2	IPI00007034	0.000	0.22163	2	0	2	2
1363	40S ribosomal protein S12	IPI00013917	0.000	0.22163	0	0	0	0
1364	Probable O-sialoglycoprotein endopeptidase	IPI00015809	0.000	0.22163	4	3	5	2
1365	Golgi phosphoprotein 3	IPI00005490	0.000	0.22163	0	0	2	0
1366	28S ribosomal protein S16, mitochondrial	IPI00032872	0.000	0.22163	0	2	0	0
1367	Eukaryotic translation initiation factor 3 subunit J	IPI00290461	0.000	0.22163	5	3	3	5
1368	Nucleoporin Nup43	IPI00742943	0.000	0.22163	5	4	5	4
1369	F-box only protein 2	IPI00007087	0.000	0.22163	3	2	3	2
1370	Isoform 1 of Caseinolytic peptidase B protein homolog	IPI00006615	0.000	0.22163	5	3	4	4
1371	High mobility group protein B3	IPI00217477	0.000	0.22163	6	6	5	7
1372	Isoform 1 of Ubiquitin carboxyl-terminal hydrolase 34	IPI00297593	0.000	0.22163	0	0	0	0
1373	Nucleolar protein 11	IPI00303813	0.000	0.22163	0	0	0	0
1374	DNA polymerase	IPI00744598	0.000	0.22163	0	0	0	0
1375	Isoform 5 of Protein transport protein Sec16A	IPI00031242	0.000	0.22163	2	2	0	2
1376	mesencephalic astrocyte-derived neurotrophic factor	IPI00328748	0.000	0.22163	0	0	2	2
1377	Mitogen-activated protein kinase scaffold protein 1	IPI00030919	0.000	0.22163	0	0	0	0
1378	Neudesin	IPI00002525	0.000	0.22163	0	0	0	2
1379	Isoform 1 of Apolipoprotein O	IPI00042580	0.000	0.22163	3	0	3	0
1380	Putative uncharacterized protein DKFZp686E2459	IPI00375731	0.000	0.22163	2	5	3	4
1381	ubiquitin-like protein fubi and ribosomal protein S30 precursor	IPI00019770	0.000	0.22163	2	2	2	2
1382	Isoform 1 of Ubiquitin conjugation factor E4 B	IPI00005715	0.000	0.22163	2	0	2	0
1383	Mitochondrial 28S ribosomal protein S2	IPI00006970	0.000	0.22163	0	0	2	2
1384	Charged multivesicular body protein 7	IPI00395463	0.000	0.22163	4	0	0	4
1385	Unhealthy ribosome biogenesis protein 2 homolog	IPI00028980	0.000	0.22163	2	3	0	3
1386	Isoform 1 of tRNA-nucleotidyltransferase 1, mitochondrial	IPI00289807	0.000	0.22163	5	3	3	5
1387	cDNA FLJ59712, highly similar to Golgi reassembly-stacking protein 2	IPI00743931	0.000	0.22163	5	4	4	5
1388	COP9 signalosome complex subunit 7a	IPI00301419	0.000	0.22163	4	5	5	4
1389	Isoform p26 of 7,8-dihydro-8-oxoguanine triphosphatase	IPI00004392	0.000	0.22163	3	4	3	4
1390	Isoform Alpha of Caveolin-1	IPI00009236	0.000	0.22163	2	0	2	0
1391	Isoform 1 of Anaphase-promoting complex subunit 4	IPI00002551	0.000	0.22163	2	0	0	0
1392	ATP synthase subunit delta, mitochondrial	IPI00024920	0.000	0.22163	2	0	0	0
1393	cDNA FLJ56047, highly similar to A kinase anchor protein 1, mitochondrial	IPI00022585	0.000	0.22163	0	0	0	0
1394	Phosphoglycerate mutase 2	IPI00218570	0.000	0.22163	3	3	4	2
1395	Isoform 2 of Pinin	IPI00002649	0.000	0.22163	4	2	3	3
1396	39S ribosomal protein L17, mitochondrial	IPI00172591	0.000	0.22163	2	2	2	0
1397	Uncharacterized protein C3orf26	IPI00031679	0.000	0.22163	3	2	2	3
1398	Uroporphyrinogen decarboxylase	IPI00301489	0.000	0.22163	2	0	0	0
1399	Molybdopterin synthase catalytic subunit	IPI00005218	0.000	0.22163	2	3	2	3
1400	Isoform 2 of U4/U6 small nuclear ribonucleoprotein Prp31	IPI00167198	0.000	0.22163	6	4	5	5
1401	NADH dehydrogenase [ubiquinone] 1 beta subcomplex subunit 9	IPI00255052	0.000	0.22163	0	0	0	0
1402	60S acidic ribosomal protein P1	IPI00008527	0.000	0.22163	0	0	0	0
1403	Isoform 1 of Adenylate kinase 2, mitochondrial	IPI00215901	0.000	0.22163	0	0	0	0
1404	Putative uncharacterized protein	IPI00010402	0.000	0.22163	0	2	2	2
1405	DNA polymerase delta subunit 2	IPI00025616	0.000	0.22163	4	3	4	3
1406	TP53-regulating kinase	IPI00290305	0.000	0.22163	3	2	3	2
1407	Short/branched chain specific acyl-CoA dehydrogenase, mitochondrial	IPI00024623	0.000	0.22163	2	0	2	2
1408	Epidermal growth factor receptor kinase substrate 8	IPI00290337	0.000	0.22163	5	0	5	0
1409	Tetratricopeptide repeat protein 9C	IPI00175096	0.000	0.22163	2	2	2	2
1410	Dimethyladenosine transferase 1, mitochondrial	IPI00291525	0.000	0.22163	0	0	2	2
1411	Isoform 1 of Protein LSM12 homolog	IPI00410324	0.000	0.22163	4	3	3	4
1412	Ras-related protein Rab-9A	IPI00016372	0.000	0.22163	4	4	4	4
1413	Isoform 1 of Phosphatidate cytidylyltransferase 2	IPI00032150	0.000	0.22163	0	0	0	2
1414	Isoform 1 of Solute carrier family 12 member 2	IPI00022649	0.000	0.22163	0	2	0	0
1415	Inner nuclear membrane protein Man1	IPI00032491	0.000	0.22163	2	0	0	2

No.	Description	Accession number	STN[1]	p-Value[1]	Con_A[2]	Con_B[2]	5FU_A[2]	5FU_B[2]
1416	NADH dehydrogenase [ubiquinone] 1 alpha subcomplex assembly factor 4	IPI00023064	0.000	0.22163	2	2	2	2
1417	Peroxisomal membrane protein PMP34	IPI00014440	0.000	0.22163	0	0	0	0
1418	RcDNAJ9 (Fragment)	IPI00014718	0.000	0.22163	3	3	3	3
1419	Epoxide hydrolase 1	IPI00009896	0.000	0.22163	2	2	2	0
1420	Protein phosphatase 1F	IPI00291412	0.000	0.22163	3	0	3	0
1421	cDNA FLJ53927, highly similar to Beta-hexosaminidase alpha chain	IPI00027851	0.000	0.22163	4	3	4	3
1422	Eukaryotic peptide chain release factor GTP-binding subunit ERF3B	IPI00642097	0.000	0.22163	2	0	0	0
1423	Isoform 1 of Autophagy-related protein 9A	IPI00383396	0.000	0.22163	0	0	0	2
1424	THO complex subunit 5 homolog	IPI00299417	0.000	0.22163	0	0	0	2
1425	Acyl-coenzyme A thioesterase 13	IPI00020530	0.000	0.22163	3	4	3	4
1426	60S ribosomal protein L35a	IPI00029731	0.000	0.22163	0	0	0	0
1427	39S ribosomal protein L41, mitochondrial	IPI00217553	0.000	0.22163	3	0	3	2
1428	Isoform 2 of RING finger protein 213	IPI00470478	0.000	0.22163	0	0	0	0
1429	Isoform 2 of WASH complex subunit 7	IPI00164930	0.000	0.22163	2	0	0	0
1430	U2 small nuclear ribonucleoprotein B''	IPI00029267	0.000	0.22163	0	2	0	0
1431	Isoform Long of Acidic fibroblast growth factor intracellular-binding protein	IPI00012443	0.000	0.22163	0	0	0	0
1432	Isoform 1 of Ral GTPase-activating protein subunit beta	IPI00409601	0.000	0.22163	0	0	2	0
1433	Isoform 1 of COP9 signalosome complex subunit 7b	IPI00009301	0.000	0.22163	2	4	3	3
1434	Isoform 1 of Integrator complex subunit 4	IPI00446765	0.000	0.22163	0	0	0	0
1435	Sodium/myo-inositol cotransporter	IPI00296311	0.000	0.22163	0	0	0	2
1436	Bystin	IPI00328987	0.000	0.22163	2	0	0	0
1437	YLP motif-containing protein 1	IPI00165434	0.000	0.22163	0	0	2	2
1438	YEATS domain-containing protein 4	IPI00008536	0.000	0.22163	2	2	0	2
1439	cDNA FLJ45232 fis, clone BRCAN2021718	IPI00170877	0.000	0.22163	3	2	3	0
1440	Isoform 1 of Transmembrane emp24 domain-containing protein 4	IPI00296259	0.000	0.22163	2	2	2	2
1441	Thioredoxin-like protein 4B	IPI00016481	0.000	0.22163	0	0	0	2
1442	Pumilio domain-containing protein C14orf21	IPI00216999	0.000	0.22163	2	2	0	0
1443	Cytosolic Fe-S cluster assembly factor NUBP2	IPI00644674	0.000	0.22163	0	2	0	0
1444	Cohesin subunit SA-1	IPI00025158	0.000	0.22163	4	2	3	3
1445	Vacuolar protein sorting-associated protein 4B	IPI00182728	0.000	0.22163	0	0	0	0
1446	Uncharacterized protein C2orf47, mitochondrial	IPI00291751	0.000	0.22163	0	0	2	0
1447	DNA-directed RNA polymerase II subunit RPB7	IPI00218895	0.000	0.22163	0	0	0	0
1448	Uncharacterized protein C11orf73	IPI00410091	0.000	0.22163	2	2	2	2
1449	60S ribosomal protein L31	IPI00026302	0.000	0.22163	0	0	2	0
1450	Isoform 3 of Nucleoporin NDC1	IPI00074330	0.000	0.22163	0	2	0	0
1451	Programmed cell death protein 2-like	IPI00031647	0.000	0.22163	0	0	2	0
1452	Cyclin-dependent kinases regulatory subunit 1	IPI00015104	0.000	0.22163	2	0	2	2
1453	Interferon-induced 17 kDa protein	IPI00375631	0.000	0.22163	0	4	2	4
1454	Pyridoxal phosphate phosphatase	IPI00025340	0.000	0.22163	4	4	4	4
1455	Dynein light chain Tctex-type 1	IPI00019495	0.000	0.22163	2	2	0	0
1456	Isoform 2 of Cytochrome b5	IPI00182933	0.000	0.22163	2	2	2	2
1457	COMM domain-containing protein 2	IPI00456048	0.000	0.22163	2	0	0	2
1458	Similar to Zinc finger CCCH domain-containing protein 15	IPI00000279	0.000	0.22163	3	4	5	2
1459	Immunoglobulin-binding protein 1	IPI00019148	0.000	0.22163	2	3	3	0
1460	COMM domain-containing protein 3	IPI00015773	0.000	0.22163	2	2	2	2
1461	Thioredoxin domain-containing protein 9	IPI00022386	0.000	0.22163	0	3	3	0
1462	Neighbor of COX4	IPI00005740	0.000	0.22163	2	3	0	3
1463	Probable ATP-dependent RNA helicase YTHDC2	IPI00010200	0.000	0.22163	0	2	0	2
1464	cDNA FLJ78567	IPI00043678	0.000	0.22163	0	3	3	0
1465	Isoform 1 of Mediator of RNA polymerase II transcription subunit 27	IPI00302652	0.000	0.22163	2	2	2	2
1466	Isoform 1 of Zinc finger protein 326	IPI00373877	0.000	0.22163	3	5	4	4
1467	TRMT61A protein (Fragment)	IPI00059718	0.000	0.22163	0	2	0	0
1468	Isoform 1 of Structural maintenance of chromosomes protein 4	IPI00411559	0.000	0.22163	0	0	2	0
1469	Isoform 1 of Mitogen-activated protein kinase kinase kinase kinase 4	IPI00006752	0.000	0.22163	0	0	0	2
1470	Proteasome assembly chaperone 3	IPI00031106	0.000	0.22163	0	2	2	2
1471	PNAS-117	IPI00020827	0.000	0.22163	0	0	0	0
1472	Tyrosine-protein phosphatase non-receptor type 23	IPI00034006	0.000	0.22163	0	0	2	0
1473	Ubiquitin-conjugating enzyme E2 G1	IPI00219783	0.000	0.22163	0	2	2	0
1474	N-acetylgalactosaminyltransferase 7	IPI00328391	0.000	0.22163	0	0	0	0
1475	Isoform 2 of Actin-related protein 2/3 complex subunit 5	IPI00007280	0.000	0.22163	4	2	3	3
1476	cDNA FLJ56277, highly similar to Toll-like receptor 9	IPI00219489	0.000	0.22163	0	0	0	2
1477	Gamma-soluble NSF attachment protein	IPI00293817	0.000	0.22163	4	3	4	3
1478	Isoform 1 of DAZ-associated protein 1	IPI00165230	0.000	0.22163	2	0	0	0
1479	Choline dehydrogenase, mitochondrial	IPI00168603	0.000	0.22163	2	0	0	0
1480	Phosphopantothenate--cysteine ligase	IPI00023987	0.000	0.22163	4	2	2	4
1481	Isoform 1 of Serine/threonine-protein kinase ATR	IPI00412298	0.000	0.22163	2	0	2	0
1482	39S ribosomal protein L20, mitochondrial	IPI00013706	0.000	0.22163	3	3	3	3
1483	Vacuolar protein sorting-associated protein 26B	IPI00059264	0.000	0.22163	0	0	2	0
1484	PRMT3 protein (Fragment)	IPI00103026	0.000	0.22163	2	0	2	2
1485	cDNA FLJ58308, highly similar to Alpha-1,2-mannosyltransferase ALG9	IPI00234857	0.000	0.22163	2	0	0	0
1486	Keratin, type II cytoskeletal 6B	IPI00293665	0.000	0.22163	0	0	0	0
1487	Cytochrome c oxidase subunit 1	IPI00464968	0.000	0.22163	0	2	0	0
1488	Isoform 1 of Origin recognition complex subunit 3	IPI00294402	0.000	0.22163	0	0	0	0
1489	Protein LLP homolog	IPI00031615	0.000	0.22163	2	2	2	0
1490	C-terminal-binding protein 1	IPI00012835	0.000	0.22163	4	0	4	0
1491	Haloacid dehalogenase-like hydrolase domain-containing protein 3	IPI00009931	0.000	0.22163	2	3	3	2
1492	Nucleoporin Nup37	IPI00171665	0.000	0.22163	3	2	3	2
1493	Exosome complex exonuclease RRP41	IPI00745613	0.000	0.22163	3	0	3	2
1494	39S ribosomal protein L28, mitochondrial	IPI00172594	0.000	0.22163	3	0	3	0
1495	E3 ubiquitin-protein ligase MARCH5	IPI00414168	0.000	0.22163	0	2	0	0
1496	Replication initiator 1	IPI00549171	0.000	0.22163	2	2	0	2
1497	NADH dehydrogenase [ubiquinone] 1 beta subcomplex subunit 3	IPI00219383	0.000	0.22163	2	0	0	0
1498	Protein FAM83H	IPI00784320	0.000	0.22163	2	2	0	2
1499	Isoform 1 of TRAF2 and NCK-interacting protein kinase	IPI00145805	0.000	0.22163	0	0	0	2
1500	Isoform 1 of Protein zwilch homolog	IPI00329679	0.000	0.22163	3	0	3	0
1501	ERBB2IP protein	IPI00438286	0.000	0.22163	2	2	0	2
1502	Uncharacterized protein C20orf4	IPI00166013	0.000	0.22163	3	2	2	3
1503	Tetratricopeptide repeat protein 19	IPI00170855	0.000	0.22163	2	2	0	0
1504	Protein CTF18 homolog	IPI00178203	0.000	0.22163	0	0	2	0
1505	Isoform 1 of Cullin-4A	IPI00419273	0.000	0.22163	3	2	2	3
1506	ATP synthase mitochondrial F1 complex assembly factor 2	IPI00296999	0.000	0.22163	3	3	3	3
1507	Isoform 2 of Uncharacterized protein C3orf63	IPI00745978	0.000	0.22163	0	0	2	2
1508	Isoform 2 of LisH domain and HEAT repeat-containing protein KIAA1468	IPI00023330	0.000	0.22163	0	0	2	2
1509	NEDD8-activating enzyme E1 catalytic subunit	IPI00328154	0.000	0.22163	4	3	4	3
1510	Isoform 2 of Low molecular weight phosphotyrosine protein phosphatase	IPI00218847	0.000	0.22163	4	3	4	3

No.	Description	Accession number	STN[1]	p-Value[1]	Con_A[2]	Con_B[2]	5FU_A[2]	5FU_B[2]
1511	RNA-binding motif protein, X-linked-like-2	IPI00004450	0.000	0.22163	0	0	2	0
1512	Isoform 1 of tRNA (guanine-N(7)-)-methyltransferase subunit WDR4	IPI00025718	0.000	0.22163	0	2	2	0
1513	Isoform 4 of Afadin	IPI00023461	0.000	0.22163	0	0	2	0
1514	Ubiquilin-4	IPI00024502	0.000	0.22163	2	0	2	2
1515	Isoform A of SWI/SNF-related matrix-associated actin-dependent regulator of chromatin subfamily B	IPI00029695	0.000	0.22163	2	0	0	2
1516	Ubiquitin carboxyl-terminal hydrolase 10	IPI00291946	0.000	0.22163	0	2	0	0
1517	Isoform 1 of FAD synthase	IPI00220299	0.000	0.22163	2	0	0	0
1518	Isoform 2 of Protein FAM36A	IPI00103057	0.000	0.22163	2	2	0	0
1519	Isoform 1 of HCLS1-associated protein X-1	IPI00010440	0.000	0.22163	0	0	2	0
1520	Retinol dehydrogenase 14	IPI00177940	0.000	0.22163	2	0	2	2
1521	Serine/threonine-protein kinase 10	IPI00304742	0.000	0.22163	2	2	0	2
1522	Coiled-coil domain-containing protein 58	IPI00046828	0.000	0.22163	2	2	2	0
1523	Isoform 1A of Myotubularin-related protein 1	IPI00220956	0.000	0.22163	2	0	0	0
1524	Transmembrane protein 214	IPI00477118	0.000	0.22163	0	0	0	2
1525	Isoform 1 of ADP-ribosylation factor-like protein 2-binding protein	IPI00015866	0.000	0.22163	3	2	3	0
1526	Histone chaperone ASF1B	IPI00041127	0.000	0.22163	2	2	0	0
1527	Isoform 1 of Mixed lineage kinase domain-like protein	IPI00180781	0.000	0.22163	3	3	3	3
1528	Isoform 1 of AT-rich interactive domain-containing protein 2	IPI00470537	0.000	0.22163	0	0	2	0
1529	DNA replication complex GINS protein PSF2	IPI00007146	0.000	0.22163	2	0	2	0
1530	Isoform 2 of RANBP2-like and GRIP domain-containing protein 5/6	IPI00100787	0.000	0.22163	0	0	0	2
1531	Sentrin-specific protease 3	IPI00171525	0.000	0.22163	0	2	2	2
1532	39S ribosomal protein L51, mitochondrial	IPI00607627	0.000	0.22163	0	2	0	0
1533	Isoform 2 of Mediator of RNA polymerase II transcription subunit 1	IPI00790747	0.000	0.22163	2	0	2	0
1534	Isoform 1 of Myotubularin-related protein 5	IPI00029446	0.000	0.22163	2	0	0	0
1535	Putative uncharacterized protein NAPRT1	IPI00412498	0.000	0.22163	3	2	3	0
1536	Histone chaperone ASF1A	IPI00292168	0.000	0.22163	0	2	0	0
1537	Myosin-Ia	IPI00294386	0.000	0.22163	4	0	0	4
1538	Ethanolamine kinase 1	IPI00030090	0.000	0.22163	2	0	0	0
1539	Isoform 1 of Integrator complex subunit 6	IPI00015922	0.000	0.22163	2	0	0	2
1540	ATP synthase mitochondrial F1 complex assembly factor 1	IPI00302673	0.000	0.22163	2	0	0	0
1541	Telomere length regulation protein TEL2 homolog	IPI00016868	0.000	0.22163	0	0	0	0
1542	Probable ATP-dependent RNA helicase DHX37	IPI00217630	0.000	0.22163	2	0	0	2
1543	UBX domain-containing protein 4	IPI00293946	0.000	0.22163	4	2	4	2
1544	Isoform 1 of Elongator complex protein 3	IPI00165477	0.000	0.22163	3	0	2	3
1545	28S ribosomal protein S18a, mitochondrial	IPI00018691	0.000	0.22163	0	0	0	0
1546	Isoform Alpha of E3 ubiquitin-protein ligase TRIM33	IPI00010252	0.000	0.22163	3	4	3	4
1547	Transcriptional repressor p66-beta	IPI00103554	0.000	0.22163	2	4	3	3
1548	Isoform 1 of Gamma-tubulin complex component 3	IPI00033516	0.000	0.22163	2	3	0	3
1549	Isoform 1 of DNA-directed RNA polymerases I and III subunit RPAC1	IPI00005179	0.000	0.22163	2	0	0	0
1550	UPF0534 protein C4orf43	IPI00019962	0.000	0.22163	2	2	2	2
1551	Isoform NELF-C of Negative elongation factor C/D	IPI00164949	0.000	0.22163	3	0	3	2
1552	Gamma-tubulin complex component 2	IPI00029705	0.000	0.22163	2	0	0	0
1553	32 kDa protein	IPI00399077	0.000	0.22163	0	0	0	0
1554	Isoform A of DnaJ homolog subfamily B member 6	IPI00024523	0.000	0.22163	2	0	0	0
1555	Isoform 1 of Elongator complex protein 2	IPI00015560	0.000	0.22163	0	0	0	0
1556	Protein naked cuticle homolog 1	IPI00056339	0.000	0.22163	2	2	0	0
1557	Isoform 1 of Hydroxyacylglutathione hydrolase, mitochondrial	IPI00003933	0.000	0.22163	0	0	2	0
1558	CD9 antigen	IPI00215997	0.000	0.22163	2	2	2	0
1559	Protein DERPC	IPI00171540	0.000	0.22163	0	0	2	0
1560	Tyrosine-protein kinase CSK	IPI00013212	0.000	0.22163	2	0	0	0
1561	Target of EGR1 protein 1	IPI00549516	0.000	0.22163	0	0	2	0
1562	Isoform Long of Transformer-2 protein homolog alpha	IPI00013891	0.000	0.22163	2	3	2	3
1563	Thiopurine S-methyltransferase	IPI00019400	0.000	0.22163	3	0	3	0
1564	Isoform 4 of Death-inducer obliterator 1	IPI00619921	0.000	0.22163	0	0	0	2
1565	Switch-associated protein 70	IPI00307200	0.000	0.22163	2	0	0	2
1566	Isoform 1 of Phosphotriesterase-related protein	IPI00100933	0.000	0.22163	0	0	0	0
1567	U3 small nucleolar RNA-interacting protein 2	IPI00217862	0.000	0.22163	2	0	0	2
1568	Isoform 1 of 28S ribosomal protein S11, mitochondrial	IPI00010244	0.000	0.22163	0	3	2	3
1569	Uncharacterized protein C1orf198	IPI00013912	0.000	0.22163	0	0	2	0
1570	Isoform Long of Ancient ubiquitous protein 1	IPI00001891	0.000	0.22163	3	2	3	0
1571	Casein kinase I isoform alpha-like	IPI00167096	0.000	0.22163	2	0	2	0
1572	Glutathione peroxidase 2	IPI00298176	0.000	0.22163	2	0	0	0
1573	Isoform HERA-A of GTP-binding protein era homolog	IPI00026512	0.000	0.22163	3	0	2	3
1574	UBX domain-containing protein 7	IPI00742124	0.000	0.22163	0	0	0	2
1575	Survival of motor neuron-related-splicing factor 30	IPI00025176	0.000	0.22163	3	2	3	2
1576	cDNA FLJ58333, highly similar to T-lymphokine-activated killer cell-originated protein kinase	IPI00306708	0.000	0.22163	2	0	2	2
1577	Isoform 5 of Sigma non-opioid intracellular receptor 1	IPI00167206	0.000	0.22163	3	2	0	3
1578	Ribonuclease P protein subunit p29	IPI00032791	0.000	0.22163	0	0	0	0
1579	Isoform 5 of Myosin-14	IPI00029818	0.000	0.22163	2	0	0	0
1580	39S ribosomal protein L11, mitochondrial	IPI00007001	0.000	0.22163	0	2	2	2
1581	Isoform 1 of Uracil-DNA glycosylase	IPI00006516	0.000	0.22163	2	0	0	0
1582	Serine/threonine-protein phosphatase 2A 56 kDa regulatory subunit epsilon isoform	IPI00002853	0.000	0.22163	2	0	0	0
1583	Isoform 1 of Cell division protein kinase 9	IPI00301923	0.000	0.22163	0	0	2	0
1584	Acylphosphatase-2	IPI00216461	0.000	0.22163	0	0	2	2
1585	Isoform 2 of Triple functional domain protein	IPI00479523	0.000	0.22163	0	0	0	2
1586	Ubiquitin carboxyl-terminal hydrolase 8	IPI00030915	0.000	0.22163	2	0	2	0
1587	Actin-related protein 2/3 complex subunit 1A	IPI00333068	0.000	0.22163	0	2	0	0
1588	Tyrosine-protein phosphatase non-receptor type 14	IPI00018914	0.000	0.22163	0	0	2	0
1589	Carboxymethylenebutenolidase homolog	IPI00383046	0.000	0.22163	2	0	0	0
1590	Isoform 1 of Remodeling and spacing factor 1	IPI00290652	0.000	0.22163	0	0	2	0
1591	Isoform 1 of Serine/threonine-protein phosphatase 2A 55 kDa regulatory subunit B beta isoform	IPI00020850	0.000	0.22163	2	0	0	0
1592	Isoform 1 of Disks large homolog 3	IPI00023343	0.000	0.22163	0	0	0	0
1593	Brefeldin A-inhibited guanine nucleotide-exchange protein 1	IPI00002188	0.000	0.22163	2	0	0	0
1594	Succinate dehydrogenase assembly factor 2, mitochondrial	IPI00016443	0.000	0.22163	2	0	0	0
1595	Isoform 1 of Protein IWS1 homolog	IPI00296432	0.000	0.22163	2	2	2	0
1596	Exosome complex exonuclease RRP43	IPI00552920	0.000	0.22163	3	2	3	0
1597	Coiled-coil-helix-coiled-coil-helix domain-containing protein 2, mitochondrial	IPI00007673	0.000	0.22163	0	0	0	0
1598	ATP-dependent RNA helicase SUPV3L1, mitochondrial	IPI00412404	0.000	0.22163	2	0	0	2
1599	Isoform 1 of Coiled-coil domain-containing protein 51	IPI00153023	0.000	0.22163	3	0	3	2
1600	Coiled-coil domain-containing protein 12	IPI00453463	0.000	0.22163	3	3	3	3
1601	Vacuolar protein sorting-associated protein 33A	IPI00073179	0.000	0.22163	0	0	0	2
1602	cDNA FLJ56420, highly similar to Aspartyl aminopeptidase	IPI00015856	0.000	0.22163	2	2	0	2
1603	Mitotic spindle assembly checkpoint protein MAD2A	IPI00012369	0.000	0.22163	0	2	2	2
1604	Sulfotransferase 1A1	IPI00300026	0.000	0.22163	0	2	0	2

No.	Description	Accession number	STN[1]	p-Value[1]	Con_A[2]	Con_B[2]	5FU_A[2]	5FU_B[2]
1605	18 kDa protein	IPI00741973	0.000	0.22163	2	2	2	0
1606	Isoform 2 of Vesicle-associated membrane protein 7	IPI00013236	0.000	0.22163	2	2	0	0
1607	Ethanolamine-phosphate cytidylyltransferase	IPI00015285	0.000	0.22163	2	2	0	0
1608	Isoform 2 of AT-rich interactive domain-containing protein 1A	IPI00642705	0.000	0.22163	0	0	0	2
1609	Glia maturation factor gamma	IPI00028414	0.000	0.22163	0	0	2	0
1610	Isoform 1 of Transmembrane protein 41B	IPI00555703	0.000	0.22163	0	2	0	0
1611	Isoform 2 of Torsin-1A-interacting protein 1	IPI00012280	0.000	0.22163	2	0	0	0
1612	Protein FAM50B	IPI00015912	0.000	0.22163	2	0	0	0
1613	NF-kappa-B-activating protein	IPI00296934	0.000	0.22163	0	2	2	2
1614	AP-3 complex subunit sigma-1	IPI00014624	0.000	0.22163	0	2	0	2
1615	Isoform 2 of Syntaxin-binding protein 1	IPI00046057	0.000	0.22163	0	2	0	2
1616	Isoform 3 of Sorting nexin-3	IPI00029740	0.000	0.22163	0	0	2	0
1617	Isoform 1 of Dephospho-CoA kinase domain-containing protein	IPI00291417	0.000	0.22163	2	2	0	0
1618	Aldo-keto reductase family 1 member C2	IPI00005668	0.000	0.22163	0	0	0	0
1619	Isoform 3 of Tyrosine-protein phosphatase non-receptor type 6	IPI00183046	0.000	0.22163	0	2	0	0
1620	BTB/POZ domain-containing protein KCTD14	IPI00181836	0.000	0.22163	0	0	0	0
1621	UPF0609 protein C4orf27	IPI00016532	0.000	0.22163	2	0	0	0
1622	Trafficking protein particle complex subunit 3	IPI00004324	0.000	0.22163	0	0	0	0
1623	Phosphatidylinositol-5-phosphate 4-kinase type-2 gamma	IPI00152303	0.000	0.22163	0	0	0	0
1624	importin subunit alpha-6	IPI00413214	0.000	0.22163	3	0	2	3
1625	Mitogen-activated protein kinase 13	IPI00005741	0.000	0.22163	2	0	0	2
1626	THO complex subunit 7 homolog	IPI00291131	0.000	0.22163	3	4	4	3
1627	Isoform 1 of Prostaglandin reductase 2	IPI00167515	0.000	0.22163	0	0	2	2
1628	RAC-alpha serine/threonine-protein kinase	IPI00012866	0.000	0.22163	2	0	2	0
1629	Isoform 1 of Discoidin, CUB and LCCL domain-containing protein 2	IPI00419836	0.000	0.22163	3	2	2	3
1630	Exportin-4	IPI00028357	0.000	0.22163	2	0	0	2
1631	GPN-loop GTPase 1 isoform a	IPI00027035	0.000	0.22163	0	0	2	0
1632	Methylmalonate-semialdehyde dehydrogenase [acylating], mitochondria	IPI00024990	0.000	0.22163	0	0	2	2
1633	Integrator complex subunit 5	IPI00304676	0.000	0.22163	2	0	0	2
1634	Poly(ADP-ribose) glycohydrolase ARH3	IPI00015865	0.000	0.22163	3	3	3	3
1635	2-oxoisovalerate dehydrogenase subunit alpha, mitochondrial	IPI00025100	0.000	0.22163	3	0	2	3
1636	39S ribosomal protein L3, mitochondrial	IPI00012353	0.000	0.22163	0	2	0	0
1637	Isoform 1 of Serine/threonine-protein kinase TAO1	IPI00002232	0.000	0.22163	0	0	2	0
1638	Insulin receptor substrate 2 insertion mutant (Fragment)	IPI00464978	0.000	0.22163	0	0	0	2
1639	Isoform 4 of Neurofibromin	IPI00220514	0.000	0.22163	0	2	0	0
1640	p53 and DNA damage-regulated protein 1	IPI00027887	0.000	0.22163	0	2	0	2
1641	Transmembrane protein 205	IPI00063130	0.000	0.22163	0	0	2	0
1642	Fatty acyl-CoA reductase 1	IPI00478838	0.000	0.22163	2	2	0	0
1643	Isoform Long of Protein HIRA	IPI00217560	0.000	0.22163	0	0	2	0
1644	Proto-oncogene tyrosine-protein kinase Yes	IPI00013981	0.000	0.22163	0	0	0	0
1645	Probable ATP-dependent RNA helicase DDX20	IPI00005904	0.000	0.22163	0	0	0	0
1646	Protein dpy-30 homolog	IPI00028109	0.000	0.22163	0	0	0	0
1647	Isoform 1 of Heterogeneous nuclear ribonucleoprotein L-like	IPI00103247	0.000	0.22163	0	0	0	0
1648	Activator of basal transcription 1	IPI00002938	0.000	0.22163	0	0	0	0
1649	TFIIH basal transcription factor complex helicase XPB subunit	IPI00747053	0.000	0.22163	0	2	0	2
1650	Mitochondrial glutamate carrier 1	IPI00003004	0.000	0.22163	0	2	2	2
1651	Major facilitator superfamily domain-containing protein 10	IPI00103940	0.000	0.22163	0	0	0	0
1652	Isoform 1 of Homologous-pairing protein 2 homolog	IPI00009805	0.000	0.22163	0	2	0	2
1653	Isoform 1 of Vacuolar-sorting protein SNF8	IPI00101524	0.000	0.22163	0	0	0	2
1654	TAF6-like RNA polymerase II p300/CBP-associated factor-associated factor 65 kDa subunit 6L	IPI00007957	0.000	0.22163	0	0	0	0
1655	Mitogen-activated protein kinase 3	IPI00018195	0.000	0.22163	2	0	2	2
1656	Serine/threonine-protein phosphatase 2A 56 kDa regulatory subunit alpha isoform	IPI00014978	0.000	0.22163	2	0	2	2
1657	NEDD8-conjugating enzyme Ubc12	IPI00022597	0.000	0.22163	3	0	3	0
1658	Protein CWC15 homolog	IPI00009009	0.000	0.22163	2	0	0	0
1659	WD repeat-containing protein 5	IPI00005492	0.000	0.22163	2	2	2	2
1660	Isoform Long of Autophagy protein 5	IPI00006800	0.000	0.22163	2	2	0	2
1661	Isoform 1 of Porphobilinogen deaminase	IPI00028160	0.000	0.22163	2	0	2	2
1662	Isoform 2 of Ras-related protein Rab-4B	IPI00187143	0.000	0.22163	2	0	0	2
1663	Isoform 1 of Protein unc-45 homolog A	IPI00072534	0.000	0.22163	0	0	0	2
1664	Biogenesis of lysosome-related organelles complex 1 subunit 1	IPI00020319	0.000	0.22163	0	0	0	0
1665	progesterone receptor membrane component 2	IPI00005202	0.000	0.22163	2	0	0	2
1666	Ubiquitin-conjugating enzyme E2 E1	IPI00021346	0.000	0.22163	2	0	0	0
1667	Isoform 1 of U4/U6 small nuclear ribonucleoprotein Prp3	IPI00005861	0.000	0.22163	0	2	0	0
1668	Isoform 1 of Far upstream element-binding protein 3	IPI00377261	0.000	0.22163	2	0	0	0
1669	Mitochondrial import inner membrane translocase subunit Tim8 A	IPI00028376	0.000	0.22163	0	0	2	2
1670	FUN14 domain-containing protein 2	IPI00171769	0.000	0.22163	0	0	0	0
1671	Phosphatidylserine synthase 1	IPI00010746	0.000	0.22163	0	0	2	0
1672	Isoform 1 of Nucleoporin p58/p45	IPI00107122	0.000	0.22163	0	0	0	2
1673	U6 snRNA-associated Sm-like protein LSm3	IPI00219229	0.000	0.22163	0	0	0	0
1674	Protein FAM98A	IPI00174442	0.000	0.22163	2	2	2	0
1675	cDNA FLJ61658, highly similar to Transmembrane 9 superfamily protein member 1	IPI00101374	0.000	0.22163	2	0	0	0
1676	Exosome complex exonuclease RRP46	IPI00015955	0.000	0.22163	2	2	2	2
1677	Rab-like protein 3	IPI00102897	0.000	0.22163	0	0	0	2
1678	Isoform 2 of Atlastin-2	IPI00007183	0.000	0.22163	0	0	0	2
1679	Ras-related protein Rab-32	IPI00014377	0.000	0.22163	2	0	0	0
1680	Serine/threonine-protein kinase 25	IPI00012093	0.000	0.22163	0	0	0	0
1681	NADH dehydrogenase [ubiquinone] iron-sulfur protein 5	IPI00220063	0.000	0.22163	2	0	2	0
1682	Isoform 2 of Protein PAT1 homolog 1	IPI00760958	0.000	0.22163	0	0	0	2
1683	Werner syndrome ATP-dependent helicase	IPI00029107	0.000	0.22163	2	0	0	0
1684	Probable tRNA(His) guanylyltransferase	IPI00016559	0.000	0.22163	2	0	0	0
1685	breast cancer anti-estrogen resistance protein 1 isoform 4	IPI00011998	0.000	0.22163	2	0	2	2
1686	FH1/FH2 domain-containing protein 1	IPI00001730	0.000	0.22163	0	0	0	0
1687	MAGUK p55 subfamily member 6	IPI00303280	0.000	0.22163	2	0	0	2
1688	Vacuolar fusion protein MON1 homolog B	IPI00848138	0.000	0.22163	0	0	0	0
1689	Uncharacterized protein C18orf19	IPI00290799	0.000	0.22163	0	0	0	0
1690	Isoform 3 of Shootin-1	IPI00448751	0.000	0.22163	3	0	2	3
1691	Pumilio homolog 1 (Drosophila), isoform CRA_c	IPI00032355	0.000	0.22163	3	0	2	3
1692	Transmembrane protein C9orf46	IPI00307547	0.000	0.22163	0	0	0	0
1693	Isoform 1 of Protein fat-free homolog	IPI00001710	0.000	0.22163	0	0	2	0
1694	Isoform 1 of Nucleolar protein 14	IPI00022613	0.000	0.22163	0	0	0	2
1695	Phenazine biosynthesis-like domain-containing protein	IPI00024896	0.000	0.22163	2	2	2	2
1696	Isoform 1 of Hepatocyte growth factor receptor	IPI00029273	0.000	0.22163	0	0	0	0
1697	Dual specificity protein phosphatase 3	IPI00018671	0.000	0.22163	3	3	3	3
1698	Sorcin	IPI00027175	0.000	0.22163	0	0	0	2
1699	cDNA FLJ61386, highly similar to Homo sapiens mitochondrial ribosomal protein L43 (MRPL43)	IPI00334579	0.000	0.22163	4	2	4	2

No.	Description	Accession number	STN[1]	p-Value[1]	Con_A[2]	Con_B[2]	5FU_A[2]	5FU_B[2]
1700	nucleoside diphosphate kinase type 6	IPI00218214	0.000	0.22163	2	2	2	2
1701	N-acylneuraminate-9-phosphatase	IPI00152196	0.000	0.22163	3	0	3	2
1702	28S ribosomal protein S15, mitochondrial	IPI00550037	0.000	0.22163	0	0	0	2
1703	Guanine nucleotide-binding protein-like 3-like protein	IPI00005132	0.000	0.22163	2	0	2	0
1704	Coiled-coil domain-containing protein 134	IPI00302674	0.000	0.22163	2	2	2	0
1705	Protein yippee-like 5	IPI00429538	0.000	0.22163	2	0	0	2
1706	Isoform RF1/RF2 of Retrotransposon-derived protein PEG10	IPI00022095	0.000	0.22163	2	0	0	0
1707	Isoform 1 of NHL repeat-containing protein 2	IPI00301051	0.000	0.22163	0	0	2	0
1708	Isoform 1 of Kinesin-like protein KIF23	IPI00291579	0.000	0.22163	0	0	0	2
1709	Isoform 2 of Gamma-adducin	IPI00004408	0.000	0.22163	0	2	0	0
1710	Isoform Alpha of Paxillin	IPI00220030	0.000	0.22163	0	2	0	2
1711	Isoform 1 of Alpha-parvin	IPI00018963	0.000	0.22163	0	0	0	0
1712	poly [ADP-ribose] polymerase 14	IPI00291215	0.000	0.22163	0	0	0	2
1713	Isoform 1 of TRM1-like protein	IPI00334914	0.000	0.22163	0	0	0	0
1714	DNA-directed RNA polymerase III subunit RPC2	IPI00301346	0.000	0.22163	0	0	0	2
1715	DNA repair protein RAD51 homolog 3	IPI00012829	0.000	0.22163	2	0	0	0
1716	F-box only protein 7	IPI00294567	0.000	0.22163	0	0	0	2
1717	Ubiquitin-conjugating enzyme E2 H	IPI00020965	0.000	0.22163	0	0	2	0
1718	Probable ergosterol biosynthetic protein 28	IPI00007730	0.000	0.22163	0	0	0	0
1719	Isoform GN-1L of Glycogenin-1	IPI00180386	0.000	0.22163	0	0	0	0
1720	119 kDa protein	IPI00297178	0.000	0.22163	2	2	0	2
1721	Isoform 1 of Protein argonaute-2	IPI00745433	0.000	0.22163	0	0	0	2
1722	Isoform 2 of Beta-catenin-like protein 1	IPI00472981	0.000	0.22163	2	0	0	0
1723	Protein phosphatase inhibitor 2	IPI00220402	0.000	0.22163	0	2	0	0
1724	COMM domain-containing protein 9	IPI00305212	0.000	0.22163	2	2	2	2
1725	Ras-related protein Rab-5B	IPI00017344	0.000	0.22163	2	0	2	0
1726	Phosphatidylinositol 4-kinase type 2-alpha	IPI00020124	0.000	0.22163	2	0	0	0
1727	cDNA FLJ52756, highly similar to RNA polymerase transcriptional regulation mediator	IPI00102495	0.000	0.22163	2	0	0	0
1728	U3 small nucleolar ribonucleoprotein protein IMP4	IPI00181116	0.000	0.22163	2	2	0	2
1729	Isoform 1 of DNA replication complex GINS protein SLD5	IPI00031614	0.000	0.22163	0	0	2	2
1730	FLJ00369 protein (Fragment)	IPI00166711	0.000	0.22163	0	0	0	2
1731	Histone acetyltransferase p300	IPI00020985	0.000	0.22163	0	0	0	0
1732	Isoform AGX2 of UDP-N-acetylhexosamine pyrophosphorylase	IPI00000684	0.000	0.22163	2	2	2	0
1733	Ras-related protein Rap-2b	IPI00018364	0.000	0.22163	2	2	2	2
1734	DNA polymerase epsilon subunit 3	IPI00010141	0.000	0.22163	0	0	0	0
1735	Hippocalcin-like protein 1	IPI00219344	0.000	0.22163	0	0	0	0
1736	cDNA FLJ31776 fis, clone NT2RI2008141, highly similar to CALUMENIN	IPI00789155	0.000	0.22163	2	0	0	0
1737	ubiquitin-like with PHD and ring finger domains 1 isoform 2	IPI00797279	0.000	0.22163	2	0	0	0
1738	Isoform 2 of Evolutionarily conserved signaling intermediate in Toll pathway, mitochondria	IPI00063188	0.000	0.22163	0	0	2	0
1739	Ras-related protein Rab-23	IPI00008034	0.000	0.22163	0	2	0	2
1740	Isoform 1 of Adaptin ear-binding coat-associated protein 2	IPI00018188	0.000	0.22163	0	0	0	0
1741	NADH-ubiquinone oxidoreductase chain 2	IPI00007979	0.000	0.22163	0	0	2	2
1742	Protoporphyrinogen oxidase	IPI00031357	0.000	0.22163	2	0	0	0
1743	Protein pelota homolog	IPI00106698	0.000	0.22163	0	0	2	0
1744	Zinc finger protein 828	IPI00064212	0.000	0.22163	0	0	2	2
1745	Ribosome biogenesis protein NSA2 homolog	IPI00007089	0.000	0.22163	2	2	2	0
1746	Actin-related protein 10	IPI00302690	0.000	0.22163	2	0	0	0
1747	Elongation of very long chain fatty acids protein 5	IPI00328307	0.000	0.22163	0	0	0	0
1748	Isoform 1 of ADP-ribosylation factor GTPase-activating protein 1	IPI00175169	0.000	0.22163	2	0	0	2
1749	Arfaptin-2	IPI00021257	0.000	0.22163	0	0	2	2
1750	Cyclin-dependent kinases regulatory subunit 2	IPI00015105	0.000	0.22163	2	0	0	0
1751	Isoform 1 of GPN-loop GTPase 3	IPI00470580	0.000	0.22163	2	2	2	0
1752	STE20/SPS1-related proline-alanine-rich protein kinase	IPI00004363	0.000	0.22163	0	0	0	0
1753	Isoform 4 of E3 ubiquitin-protein ligase UBR2	IPI00217407	0.000	0.22163	2	0	0	0
1754	Uncharacterized protein C6orf130	IPI00184871	0.000	0.22163	0	0	2	0
1755	Isoform 1 of Serine/threonine-protein kinase PAK 4	IPI00014068	0.000	0.22163	2	0	0	0
1756	Isoform 1 of Transmembrane protein 126B	IPI00020540	0.000	0.22163	0	2	2	0
1757	Isoform 2 of ATPase WRNIP1	IPI00102997	0.000	0.22163	0	0	2	0
1758	Isoform 1 of Serologically defined colon cancer antigen 1	IPI00301618	0.000	0.22163	0	2	0	0
1759	Large subunit GTPase 1 homolog	IPI00300094	0.000	0.22163	2	0	2	2
1760	Isoform 2 of Treacle protein	IPI00298696	0.000	0.22163	0	0	0	2
1761	Isoform 1 of Polyadenylate-binding protein-interacting protein 1	IPI00021466	0.000	0.22163	2	2	0	0
1762	Isoform 1 of Anaphase-promoting complex subunit 7	IPI00008248	0.000	0.22163	2	2	0	0
1763	Putative uncharacterized protein KIF20B	IPI00044751	0.000	0.22163	0	0	0	0
1764	Synaptogyrin-2	IPI00013946	0.000	0.22163	0	2	0	0
1765	Ufm1-specific protease 2	IPI00305303	0.000	0.22163	0	2	0	0
1766	Isoform 1 of Abl interactor 1	IPI00431025	0.000	0.22163	0	0	2	2
1767	Sulfide:quinone oxidoreductase, mitochondrial	IPI00009634	0.000	0.22163	2	2	2	0
1768	Isoform 1 of Putative splicing factor, arginine/serine-rich 14	IPI00158020	0.000	0.22163	0	2	0	2
1769	Serine/threonine-protein kinase MRCK beta	IPI00477763	0.000	0.22163	2	0	2	0
1770	WW domain-binding protein 11	IPI00170786	0.000	0.22163	2	0	0	0
1771	Isoform 1 of Probable aminopeptidase NPEPL1	IPI00100292	0.000	0.22163	0	0	2	0
1772	Thioredoxin-like protein 4A	IPI00216338	0.000	0.22163	2	0	2	2
1773	DCC-interacting protein 13-alpha	IPI00015836	0.000	0.22163	2	2	0	0
1774	UPF0428 protein CXorf56	IPI00005055	0.000	0.22163	2	2	2	0
1775	Isoform A of Protein CutA	IPI00034319	0.000	0.22163	0	2	2	0
1776	Isoform 1 of Carbonyl reductase family member 4	IPI00384297	0.000	0.22163	0	2	2	2
1777	Peflin	IPI00018235	0.000	0.22163	0	2	2	0
1778	Checkpoint protein HUS1	IPI00004712	0.000	0.22163	2	0	0	0
1779	Pyroglutamyl-peptidase 1	IPI00020539	0.000	0.22163	0	2	0	0
1780	[Pyruvate dehydrogenase [lipoamide]] kinase isozyme 3, mitochondrial	IPI00014849	0.000	0.22163	2	0	0	0
1781	Brain-specific angiogenesis inhibitor 1-associated protein 2-like protein 1	IPI00179326	0.000	0.22163	0	0	0	0
1782	Protein unc-119 homolog B	IPI00414629	0.000	0.22163	2	0	2	2
1783	Actin-related protein 2/3 complex subunit 5-like protein	IPI00414554	0.000	0.22163	0	0	2	0
1784	UPF0552 protein C15orf38	IPI00074225	0.000	0.22163	0	0	2	2
1785	CGG triplet repeat-binding protein 1	IPI00295585	0.000	0.22163	0	2	2	0
1786	Isoform 1 of NIF3-like protein 1	IPI00604624	0.000	0.22163	0	2	0	0
1787	Serum deprivation-response protein	IPI00005809	0.000	0.22163	3	0	2	3
1788	Isoform 2 of Arf-GAP with Rho-GAP domain, ANK repeat and PH domain-containing protein 1	IPI00220421	0.000	0.22163	2	2	0	0
1789	Torsin-1A-interacting protein 2	IPI00168878	0.000	0.22163	0	0	0	0
1790	Cytovillin 2 (Fragment)	IPI00384282	0.000	0.22163	0	0	0	0
1791	Caspase-3	IPI00292140	0.000	0.22163	2	2	0	0
1792	Isoform 2 of Septin-8	IPI00022082	0.000	0.22163	4	0	2	4
1793	Isoform 2 of Gamma-glutamylcyclotransferase	IPI00020301	0.000	0.22163	2	0	0	0
1794	CDGSH iron sulfur domain-containing protein 2	IPI00166865	0.000	0.22163	2	0	2	2

No.	Description	Accession number	STN[1]	p-Value[1]	Con_A[2]	Con_B[2]	5FU_A[2]	5FU_B[2]
1795	Ubiquitin protein ligase E3 component n-recognin 4	IPI00514902	0.000	0.22163	0	2	0	0
1796	Mitochondrial ornithine transporter 1	IPI00003389	0.000	0.22163	0	2	0	0
1797	DNA/RNA-binding protein KIN17	IPI00017580	0.000	0.22163	0	0	0	0
1798	Isoform 1 of GPI transamidase component PIG-T	IPI00100030	0.000	0.22163	0	0	0	2
1799	Probable leucyl-tRNA synthetase, mitochondrial	IPI00014213	0.000	0.22163	0	2	0	2
1800	Isoform 1 of Zinc phosphodiesterase ELAC protein 2	IPI00396627	0.000	0.22163	2	0	0	0
1801	28S ribosomal protein S30, mitochondrial	IPI00010278	0.000	0.22163	2	0	2	0
1802	Protein cappuccino homolog	IPI00020002	0.000	0.22163	0	2	0	0
1803	Transducin beta-like protein 2	IPI00000948	0.000	0.22163	0	2	2	2
1804	Secretory carrier-associated membrane protein 2	IPI00218850	0.000	0.22163	0	0	0	2
1805	Isoform 1 of Serine protease HTRA2, mitochondrial	IPI00001663	0.000	0.22163	2	0	2	0
1806	Proteasomal ubiquitin receptor ADRM1	IPI00033030	0.000	0.22163	2	0	2	2
1807	Isoform 2 of Epithelial splicing regulatory protein 2	IPI00034099	0.000	0.22163	0	2	0	0
1808	Isoform 3 of Sperm-specific antigen 2	IPI00386170	0.000	0.22163	0	2	2	0
1809	Isoform 2 of Double-stranded RNA-binding protein Staufen homolog 2	IPI00164481	0.000	0.22163	0	2	0	0
1810	cDNA FLJ56152, highly similar to Rho guanine nucleotide exchange factor 7	IPI00449906	0.000	0.22163	0	0	2	0
1811	Nuclear envelope pore membrane protein POM 121C	IPI00032358	0.000	0.22163	0	0	0	2
1812	Geranylgeranyl pyrophosphate synthase	IPI00032892	0.000	0.22163	0	0	0	2
1813	Isoform 1 of Casein kinase I isoform alpha	IPI00183400	0.000	0.22163	0	2	0	0
1814	PRA1 family protein 2	IPI00026994	0.000	0.22163	0	0	0	0
1815	Isoform 1 of Mitochondrial Rho GTPase 2	IPI00465059	0.000	0.22163	0	0	2	0
1816	Transcription factor MafG	IPI00007311	0.000	0.22163	0	0	0	2
1817	Phosphoglycolate phosphatase	IPI00177008	0.000	0.22163	0	0	2	0
1818	Isoform 1 of Splicing factor, arginine/serine-rich 12	IPI00103497	0.000	0.22163	2	0	0	2
1819	NADH dehydrogenase [ubiquinone] 1 alpha subcomplex assembly factor 3	IPI00399053	0.000	0.22163	0	0	0	2
1820	3-oxoacyl-[acyl-carrier-protein] synthase, mitochondrial	IPI00016637	0.000	0.22163	0	2	0	0
1821	Isoform 2 of Epimerase family protein SDR39U1	IPI00643286	0.000	0.22163	2	2	2	2
1822	Isoform 1 of Nicastrin	IPI00021983	0.000	0.22163	0	0	2	2
1823	Peptidyl-prolyl cis-trans isomerase-like 4	IPI00642862	0.000	0.22163	2	2	2	0
1824	Isoform 1 of RNA-binding protein 34	IPI00181617	0.000	0.22163	2	0	0	0
1825	Isoform 1 of Opioid growth factor receptor	IPI00021537	0.000	0.22163	0	0	0	2
1826	Isoform 1 of Tuftelin-interacting protein 11	IPI00015924	0.000	0.22163	2	0	0	0
1827	Putative uncharacterized protein TXNRD2	IPI00157820	0.000	0.22163	0	0	0	0
1828	Isoform 1 of Structural maintenance of chromosomes protein 6	IPI00154528	0.000	0.22163	2	0	0	0
1829	Hsp90 co-chaperone Cdc37	IPI00013122	0.000	0.22163	3	0	3	0
1830	Protein FAM118B	IPI00002240	0.000	0.22163	2	0	2	2
1831	Acyl-CoA-binding domain-containing protein 6	IPI00031680	0.000	0.22163	0	2	2	2
1832	Alpha-mannosidase 2	IPI00003802	0.000	0.22163	2	0	0	0
1833	Isoform 4 of tRNA dimethylallyltransferase, mitochondrial	IPI00164286	0.000	0.22163	0	2	2	0
1834	Atlastin-1	IPI00103530	0.000	0.22163	0	0	0	0
1835	Protein Red	IPI00011875	0.000	0.22163	0	0	0	2
1836	Ras-related protein Rab-20	IPI00015839	0.000	0.22163	0	0	0	0
1837	Isoform 1 of Dedicator of cytokinesis protein 9	IPI00216408	0.000	0.22163	0	0	0	2
1838	Pre-mRNA-splicing factor SYF1	IPI00163084	0.000	0.22163	0	0	0	2
1839	A-kinase anchor protein 8	IPI00014474	0.000	0.22163	0	0	0	0
1840	RNASEH2B protein	IPI00245135	0.000	0.22163	2	0	0	0
1841	Isoform Beta-1 of Protein phosphatase 1B	IPI00026612	0.000	0.22163	2	0	0	0
1842	Isoform 1 of Mediator of RNA polymerase II transcription subunit 16	IPI00037401	0.000	0.22163	0	0	0	0
1843	Isoform 2 of Exosome complex exonuclease RRP45	IPI00029697	0.000	0.22163	0	0	0	0
1844	Isoform 1 of Cleavage and polyadenylation specificity factor subunit 4	IPI00009137	0.000	0.22163	2	0	0	2
1845	CDC45-related protein	IPI00025695	0.000	0.22163	0	0	0	0
1846	39S ribosomal protein L54, mitochondrial	IPI00332157	0.000	0.22163	2	0	0	0
1847	Protein kinase C iota type	IPI00016639	0.000	0.22163	0	0	0	2
1848	Interferon-induced protein with tetratricopeptide repeats 5	IPI00012756	0.000	0.22163	0	0	0	2
1849	Isoform 1 of Protein LSM14 homolog B	IPI00032635	0.000	0.22163	2	2	0	0
1850	Isoform 1 of Pre-mRNA-splicing factor RBM22	IPI00019046	0.000	0.22163	2	2	0	2
1851	Isoform 2 of Inositol hexakisphosphate and diphosphoinositol-pentakisphosphate kinase 2	IPI00178375	0.000	0.22163	0	0	0	0
1852	Isoform 1 of Transmembrane protein 55B	IPI00030530	0.000	0.22163	0	0	2	2
1853	Isoform 1 of Solute carrier family 35 member F2	IPI00293362	0.000	0.22163	2	0	2	0
1854	cDNA FLJ10321 fis, clone NT2RM2000504, highly similar to Homo sapiens pitrilysin metallopeptidase	IPI00219613	0.000	0.22163	2	0	0	2
1855	General transcription factor 3C polypeptide 4	IPI00016725	0.000	0.22163	0	0	0	0
1856	Interferon regulatory factor 2-binding protein 1	IPI00645608	0.000	0.22163	0	0	0	0
1857	Isoform 1 of Regulation of nuclear pre-mRNA domain-containing protein 2	IPI00384541	0.000	0.22163	0	0	2	0
1858	Isoform 1 of Alpha-(1,6)-fucosyltransferase	IPI00004668	0.000	0.22163	0	0	2	0
1859	Isoform 2 of Ribonuclease P protein subunit p40	IPI00332091	0.000	0.22163	2	0	0	2
1860	Importin subunit alpha-3	IPI00299033	0.000	0.22163	0	2	2	0
1861	Isoform 1 of Mucosa-associated lymphoid tissue lymphoma translocation protein 1	IPI00009540	0.000	0.22163	2	0	0	0
1862	KRR1 small subunit processome component homolog	IPI00156032	0.000	0.22163	2	2	0	0
1863	Geminin	IPI00026309	0.000	0.22163	0	0	2	0
1864	MMP37-like protein, mitochondrial	IPI00060287	0.000	0.22163	0	0	2	2
1865	Isoform 3 of Ubiquitin-protein ligase E3C	IPI00411748	0.000	0.22163	2	2	2	0
1866	Putative RNA-binding protein 16	IPI00829652	0.000	0.22163	2	0	0	0
1867	Isoform 1 of Spastin	IPI00002707	0.000	0.22163	0	2	0	2
1868	Sulfhydryl oxidase 2	IPI00376394	0.000	0.22163	0	0	0	2
1869	Guanine nucleotide-binding protein G(i) subunit alpha-1	IPI00337415	0.000	0.22163	0	0	2	2
1870	Isoform 1 of Ubiquinone biosynthesis methyltransferase COQ5, mitochondrial	IPI00456965	0.000	0.22163	0	0	2	0
1871	Isoform 2 of Rho GTPase-activating protein 5	IPI00013988	0.000	0.22163	0	0	2	0
1872	NudC domain-containing protein 2	IPI00103142	0.000	0.22163	2	2	2	2
1873	1-phosphatidylinositol-4,5-bisphosphate phosphodiesterase delta-3	IPI00152701	0.000	0.22163	2	2	0	2
1874	Gamma-tubulin complex component 5	IPI00045492	0.000	0.22163	0	0	0	0
1875	Nucleoside diphosphate-linked moiety X motif 19, mitochondrial	IPI00869107	0.000	0.22163	0	0	0	0
1876	Proteasome assembly chaperone 2	IPI00644482	0.000	0.22163	0	0	0	0
1877	Isoform 2 of tRNA (adenine-N(1)-)-methyltransferase catalytic subunit TRMT61A	IPI00177856	0.000	0.22163	0	2	0	2
1878	Isoform 1 of Motile sperm domain-containing protein 2	IPI00169283	0.000	0.22163	2	0	0	0
1879	Isoform 3 of Protein LAS1 homolog	IPI00009917	0.000	0.22163	2	0	2	0
1880	ZW10 interactor	IPI00294008	0.000	0.22163	0	0	0	2
1881	Lysosome membrane protein 2	IPI00217766	0.000	0.22163	0	0	0	0
1882	MAP kinase-activated protein kinase 3	IPI00005777	0.000	0.22163	0	0	2	0
1883	Isoform A of Golgi SNAP receptor complex member 2	IPI00023135	0.000	0.22163	0	0	2	0
1884	5-formyltetrahydrofolate cyclo-ligase	IPI00220567	0.000	0.22163	0	2	0	0
1885	Isoform II of Ubiquitin-protein ligase E3A	IPI00011609	0.000	0.22163	2	0	2	0
1886	Splicing factor 45	IPI00176706	0.000	0.22163	2	0	0	0
1887	Dimethyladenosine transferase 2, mitochondrial	IPI00034069	0.000	0.22163	2	2	0	0
1888	Gamma-aminobutyric acid receptor-associated protein	IPI00027253	0.000	0.22163	2	2	0	0

No.	Description	Accession number	STN[1]	p-Value[1]	Con_A[2]	Con_B[2]	5FU_A[2]	5FU_B[2]
1889	Isoform 1 of Serine/threonine-protein phosphatase 4 regulatory subunit 3B	IPI00414323	0.000	0.22163	0	0	0	0
1890	Ras-related protein Rab-13	IPI00016373	0.000	0.22163	0	0	0	0
1891	Isoform 3 of DnaJ homolog subfamily C member 11	IPI00333016	0.000	0.22163	0	2	0	0
1892	Cytokine receptor-like factor 3	IPI00295387	0.000	0.22163	0	0	2	0
1893	Isoform 2 of Zinc finger CCCH-type antiviral protein 1	IPI00332936	0.000	0.22163	0	0	2	2
1894	NF-kappa-B-repressing factor	IPI00005675	0.000	0.22163	0	0	0	0
1895	Isoform 1 of Lysine-specific demethylase 5C	IPI00013185	0.000	0.22163	0	0	0	0
1896	Putative uncharacterized protein	IPI00027984	0.000	0.22163	0	0	0	0
1897	Cellular retinoic acid-binding protein 2	IPI00216088	0.000	0.22163	0	0	0	0
1898	Isoform 1 of Epidermal growth factor receptor kinase substrate 8-like protein 3	IPI00181833	0.000	0.22163	2	0	0	0
1899	Isoform 2 of DNA-directed RNA polymerase I subunit RPA2	IPI00026445	0.000	0.22163	2	0	0	0
1900	Isoform 2 of Lipolysis-stimulated lipoprotein receptor	IPI00328218	0.000	0.22163	2	0	0	2
1901	Angio-associated migratory cell protein	IPI00014481	0.000	0.22163	2	2	0	0
1902	Histidine triad nucleotide-binding protein 3	IPI00170924	0.000	0.22163	0	2	0	0
1903	Disintegrin and metalloproteinase domain-containing protein 10	IPI00013897	0.000	0.22163	2	0	2	0
1904	Isoform 1 of B-cell CLL/lymphoma 9-like protein	IPI00328798	0.000	0.22163	0	0	2	0
1905	COBW domain-containing protein 2	IPI00216734	0.000	0.22163	2	0	0	0
1906	Methionine aminopeptidase 2	IPI00033036	0.000	0.22163	2	0	2	0
1907	Isoform 1 of Citrate lyase subunit beta-like protein, mitochondrial	IPI00477957	0.000	0.22163	2	0	2	0
1908	Band 4.1-like protein 2	IPI00015973	0.000	0.22163	0	0	2	2
1909	25 kDa protein	IPI00010276	0.000	0.22163	0	0	0	2
1910	RNA methyltransferase-like protein 1	IPI00335589	0.000	0.22163	0	0	0	0
1911	37 kDa protein	IPI00032799	0.000	0.22163	2	0	0	0
1912	HLA class I histocompatibility antigen, B-7 alpha chain	IPI00004657	0.000	0.22163	2	2	0	2
1913	Acyl-coenzyme A thioesterase 8	IPI00298202	0.000	0.22163	2	0	0	0
1914	Isoform 3 of Tropomyosin beta chain	IPI00218820	0.000	0.22163	2	2	0	0
1915	Isoform 1 of Neuroguidin	IPI00000162	0.000	0.22163	0	0	0	2
1916	Uveal autoantigen with coiled-coil domains and ankyrin repeats	IPI00173359	0.000	0.22163	0	0	0	2
1917	Isoform 1 of Caspase-8	IPI00000149	0.000	0.22163	0	0	2	0
1918	Isoform 1 of CAP-Gly domain-containing linker protein 2	IPI00019642	0.000	0.22163	0	0	0	2
1919	Isoform 1 of Rho guanine nucleotide exchange factor 12	IPI00022164	0.000	0.22163	0	0	0	0
1920	Transcription initiation factor IIE subunit beta	IPI00019981	0.000	0.22163	0	0	0	0
1921	17 kDa protein	IPI00643390	0.000	0.22163	2	2	2	2
1922	Isoform 1 of Coiled-coil and C2 domain-containing protein 1A	IPI00302647	0.000	0.22163	2	0	2	0
1923	Isoform 1 of Endophilin-B2	IPI00024540	0.000	0.22163	0	0	0	0
1924	Isoform 1 of Pre-mRNA-splicing factor 38B	IPI00018098	0.000	0.22163	2	0	2	0
1925	Isoform 1 of Testin	IPI00024097	0.000	0.22163	2	0	2	0
1926	Solute carrier family 25 member 40	IPI00290827	0.000	0.22163	0	2	0	0
1927	Prefoldin subunit 2	IPI00006052	0.000	0.22163	2	2	0	0
1928	Isoform 1 of E3 ubiquitin-protein ligase Itchy homolog	IPI00061780	0.000	0.22163	2	0	0	0
1929	Isoform 3 of Exocyst complex component 7	IPI00103064	0.000	0.22163	0	0	0	2
1930	Isoform 1 of Trafficking protein particle complex subunit 2	IPI00005119	0.000	0.22163	0	0	0	2
1931	Reticulocalbin-2	IPI00029628	0.000	0.22163	2	0	0	0
1932	Isoform 1 of Kinetochore-associated protein DSN1 homolog	IPI00016580	0.000	0.22163	0	2	0	0
1933	Elongation factor G 2, mitochondrial precursor	IPI00071703	0.000	0.22163	2	0	0	0
1934	Origin recognition complex subunit 4	IPI00015164	0.000	0.22163	2	0	0	0
1935	Isoform Long of Metastasis-associated protein MTA1	IPI00012773	0.000	0.22163	2	0	0	0
1936	Deubiquitinating protein VCIP135	IPI00064162	0.000	0.22163	2	0	0	0
1937	Protein FAM91A1	IPI00152671	0.000	0.22163	2	0	2	0
1938	Transmembrane and coiled-coil domain-containing protein 7	IPI00034201	0.000	0.22163	2	0	0	0
1939	Isoform 2 of General transcription factor 3C polypeptide 5	IPI00411531	0.000	0.22163	0	0	2	0
1940	Armadillo repeat-containing protein 1	IPI00018260	0.000	0.22163	2	2	0	0
1941	Isoform 1 of Nuclear-interacting partner of ALK	IPI00301421	0.000	0.22163	0	0	2	0
1942	Isoform 1 of COMM domain-containing protein 7	IPI00743772	0.000	0.22163	0	0	0	0
1943	MRG-binding protein	IPI00019451	0.000	0.22163	2	0	0	0
1944	175 kDa protein	IPI00328318	0.000	0.22163	0	0	2	0
1945	Pentatricopeptide repeat-containing protein 1	IPI00171925	0.000	0.22163	0	0	0	2
1946	Trafficking protein particle complex subunit 5	IPI00177509	0.000	0.22163	0	0	2	2
1947	Cytochrome P450 monooxygenase	IPI00010218	0.000	0.22163	2	0	0	0
1948	Isoform 2 of Plakophilin-2	IPI00005264	0.000	0.22163	0	0	0	0
1949	cDNA FLJ56343, highly similar to Torsin A	IPI00413293	0.000	0.22163	2	0	0	0
1950	Isoform 3 of Guanine nucleotide exchange factor VAV2	IPI00004977	0.000	0.22163	0	0	0	2
1951	cDNA FLJ56221, highly similar to YTH domain protein 3	IPI00396131	0.000	0.22163	0	0	2	2
1952	69 kDa protein	IPI00018116	0.000	0.22163	0	2	2	0
1953	Isoform 2 of Diphthine synthase	IPI00006419	0.000	0.22163	0	0	0	2
1954	Translation initiation factor IF-2, mitochondrial	IPI00005039	0.000	0.22163	0	0	2	0
1955	Isoform 3 of Target of rapamycin complex subunit LST8	IPI00007182	0.000	0.22163	0	0	2	2
1956	Isoform 2 of LETM1 domain-containing protein 1	IPI00607602	0.000	0.22163	0	0	0	0
1957	Isoform 1 of Protein FAM65A	IPI00418799	0.000	0.22163	2	0	0	0
1958	Uncharacterized protein C8orf41	IPI00306207	0.000	0.22163	2	0	0	0
1959	Isoform 7 of Serine/threonine-protein kinase MARK2	IPI00290158	0.000	0.22163	2	0	0	0
1960	Cyclin B1	IPI00294696	0.000	0.22163	2	0	0	0
1961	Isoform 4 of Integrator complex subunit 4	IPI00419468	0.000	0.22163	0	0	0	0
1962	Isoform 1 of Acyl-CoA synthetase family member 3, mitochondrial	IPI00166395	0.000	0.22163	0	0	2	0
1963	Protein FAM96A	IPI00030985	0.000	0.22163	0	2	0	0
1964	alanyl-tRNA editing protein Aarsd1 isoform 1	IPI00748490	0.000	0.22163	2	2	2	2
1965	Protein HEXIM1	IPI00007941	0.000	0.22163	2	2	2	2
1966	Active regulator of SIRT1	IPI00219006	0.000	0.22163	2	2	2	2
1967	Geranylgeranyl transferase type-2 subunit alpha	IPI00022664	0.000	0.22163	0	0	0	0
1968	Isoform 1 of Long-chain-fatty-acid--CoA ligase 1	IPI00012728	0.000	0.22163	0	0	2	0
1969	Isoform 1 of PDZ and LIM domain protein 4	IPI00032206	0.000	0.22163	0	0	0	2
1970	Transmembrane emp24 domain-containing protein 7	IPI00032825	0.000	0.22163	0	0	0	0
1971	Isoform 1 of General transcription factor 3C polypeptide 3	IPI00015806	0.000	0.22163	0	0	2	0
1972	Isoform 1 of Dehydrogenase/reductase SDR family member 11	IPI00034280	0.000	0.22163	2	0	0	2
1973	Isoform 1 of MIF4G domain-containing protein	IPI00010240	0.000	0.22163	2	2	0	2
1974	Tryptophanyl-tRNA synthetase, mitochondrial	IPI00025050	0.000	0.22163	0	0	0	0
1975	peroxisomal 3,2-trans-enoyl-CoA isomerase isoform 1	IPI00419263	0.000	0.22163	2	2	0	2
1976	Isoform 1 of Cellular tumor antigen p53	IPI00025087	0.000	0.22163	2	2	0	2
1977	BRI3-binding protein	IPI00103599	0.000	0.22163	2	2	2	0
1978	Isoform 1 of Lipase maturation factor 2	IPI00385495	0.000	0.22163	2	0	0	0
1979	Multiple coagulation factor deficiency protein 2	IPI00328680	0.000	0.22163	0	0	0	2
1980	Isoform 2 of La-related protein 4	IPI00043638	0.000	0.22163	2	2	2	0
1981	Isoform 2 of Protein diaphanous homolog 3	IPI00655865	0.000	0.22163	0	0	2	0
1982	Isoform 1 of Rho-related GTP-binding protein RhoF	IPI00307458	0.000	0.22163	2	0	2	2
1983	Isoform 1 of Macoilin	IPI00296938	0.000	0.22163	2	0	0	0

No.	Description	Accession number	STN[1]	p-Value[1]	Con_A[2]	Con_B[2]	5FU_A[2]	5FU_B[2]
1984	Serine/threonine-protein kinase Nek7	IPI00152658	0.000	0.22163	0	2	0	0
1985	Formin-binding protein 4	IPI00170778	0.000	0.22163	0	2	2	2
1986	2'-5'-oligoadenylate synthase 3	IPI00002405	0.000	0.22163	0	0	0	0
1987	X-Pro aminopeptidase 1, soluble isoform 2	IPI00607814	0.000	0.22163	2	0	0	0
1988	Isoform 2 of SFRS2-interacting protein	IPI00181359	0.000	0.22163	0	2	0	2
1989	Zinc finger CCHC-type and RNA-binding motif-containing protein 1	IPI00154614	0.000	0.22163	0	0	0	0
1990	Interferon regulatory factor 3	IPI00291901	0.000	0.22163	2	0	0	0
1991	Syntaxin-4	IPI00029730	0.000	0.22163	0	0	2	0
1992	cDNA FLJ51219, highly similar to Homo sapiens mediator of RNA polymerase II transcription	IPI00305986	0.000	0.22163	0	0	0	0
1993	Inositol polyphosphate 1-phosphatase	IPI00027139	0.000	0.22163	0	0	0	0
1994	cDNA FLJ43556 fis, clone PROST2018511, highly similar to Growth factor receptor-bound protein 7	IPI00448767	0.000	0.22163	2	0	0	0
1995	Isoform 1 of Ribonucleoside-diphosphate reductase subunit M2 B	IPI00100213	0.000	0.22163	2	0	2	0
1996	Zinc finger CCCH domain-containing protein 4	IPI00187011	0.000	0.22163	2	0	0	0
1997	Isoform 2 of CDK5 regulatory subunit-associated protein 3	IPI00018780	0.000	0.22163	2	0	2	0
1998	kinesin-like 8 isoform c	IPI00061476	0.000	0.22163	0	2	0	0
1999	Probable ATP-dependent RNA helicase DDX49	IPI00003739	0.000	0.22163	0	2	0	0
2000	Isoform 2 of Choline-phosphate cytidylyltransferase B	IPI00001562	0.000	0.22163	2	0	0	0
2001	Isoform 1 of Protein diaphanous homolog 3	IPI00747250	0.000	0.22163	0	0	2	2
2002	Isoform 2 of Proto-oncogene tyrosine-protein kinase Src	IPI00328867	0.000	0.22163	0	0	0	0
2003	Isoform 1 of AP-1 complex subunit mu-2	IPI00002552	0.000	0.22163	0	0	0	2
2004	dehydrogenase/reductase SDR family member 4	IPI00106913	0.000	0.22163	0	0	0	2
2005	TATA box-binding protein-like protein 1	IPI00032911	0.000	0.22163	2	0	0	0
2006	Estradiol 17-beta-dehydrogenase 8	IPI00021890	0.000	0.22163	0	2	2	0
2007	Isoform 1 of N(2),N(2)-dimethylguanosine tRNA methyltransferase	IPI00020508	0.000	0.22163	2	0	0	0
2008	Transforming growth factor-beta receptor-associated protein 1	IPI00550891	0.000	0.22163	0	0	0	2
2009	Double-strand-break repair protein rad21 homolog	IPI00006715	0.000	0.22163	0	0	0	2
2010	Isoform 2 of N-acetylserotonin O-methyltransferase-like protein	IPI00249080	0.000	0.22163	2	0	0	0
2011	Hcp beta-lactamase-like protein C1orf163	IPI00303753	0.000	0.22163	2	2	0	0
2012	Delta-1-pyrroline-5-carboxylate dehydrogenase, mitochondrial	IPI00217871	0.000	0.22163	0	2	2	0
2013	Cell division cycle protein 123 homolog	IPI00005670	0.000	0.22163	0	0	0	0
2014	MORF4 family-associated protein 1	IPI00020915	0.000	0.22163	0	2	0	0
2015	Isoform B of Syntaxin-16	IPI00023149	0.000	0.22163	2	2	0	0
2016	Sorting nexin-4	IPI00029403	0.000	0.22163	0	0	0	0
2017	Isoform 2 of Hydroxysteroid dehydrogenase-like protein 2	IPI00031107	0.000	0.22163	0	0	2	2
2018	Mediator of RNA polymerase II transcription subunit 30	IPI00063213	0.000	0.22163	0	0	0	2
2019	Isoform 1 of Dynamin-1	IPI00413140	0.000	0.22163	0	0	2	0
2020	cDNA FLJ54848, highly similar to tRNA-splicing endonuclease subunit Sen34	IPI00451941	0.000	0.22163	2	2	0	0
2021	Secernin-2	IPI00062266	0.000	0.22163	0	2	0	0
2022	RAD50-interacting protein 1	IPI00072224	0.000	0.22163	0	0	0	2
2023	Isoform 1 of Multivesicular body subunit 12A	IPI00744702	0.000	0.22163	0	2	0	0
2024	CD2 antigen cytoplasmic tail-binding protein 2	IPI00006103	0.000	0.22163	0	2	2	0
2025	Galectin-7	IPI00219221	0.000	0.22163	0	0	2	2
2026	Nucleoside diphosphate kinase 3	IPI00012315	0.000	0.22163	0	0	2	0
2027	Isoform 2 of Cytochrome P450 2S1	IPI00164018	0.000	0.22163	0	2	0	0
2028	Protein MAK16 homolog	IPI00332428	0.000	0.22163	2	0	0	0
2029	Pyruvate dehydrogenase phosphatase regulatory subunit, mitochondrial	IPI00168407	0.000	0.22163	2	0	2	2
2030	WD repeat-containing protein 70	IPI00300060	0.000	0.22163	2	0	0	0
2031	Translation initiation factor eIF-2B subunit epsilon	IPI00011898	0.000	0.22163	0	0	0	2
2032	Isoform 3 of Zinc finger SWIM domain-containing protein KIAA0913	IPI00876841	0.000	0.22163	0	0	0	0
2033	CD2-associated protein	IPI00412771	0.000	0.22163	2	0	0	0
2034	High mobility group nucleosome-binding domain-containing protein 5	IPI00006157	0.000	0.22163	2	2	0	0
2035	Isoform 1 of RNA 3'-terminal phosphate cyclase	IPI00011726	0.000	0.22163	2	0	0	0
2036	Isoform 2 of Protein midA homolog, mitochondrial	IPI00375685	0.000	0.22163	0	0	0	0
2037	Isoform 1 of Ubiquitin-like modifier-activating enzyme 5	IPI00015736	0.000	0.22163	0	0	2	0
2038	Ubiquitin-conjugating enzyme E2 R2	IPI00418603	0.000	0.22163	0	2	0	0
2039	Isoform 1 of Vacuolar protein sorting-associated protein 18 homolog	IPI00001985	0.000	0.22163	0	0	0	0
2040	Short-chain specific acyl-CoA dehydrogenase, mitochondrial	IPI00027701	0.000	0.22163	0	0	0	2
2041	WD repeat-containing protein 81 isoform 1	IPI00917671	0.000	0.22163	0	0	2	0
2042	Rac GTPase-activating protein 1	IPI00152946	0.000	0.22163	0	0	2	2
2043	Isoform 1 of N-acylethanolamine-hydrolyzing acid amidase	IPI00024083	0.000	0.22163	0	0	2	0
2044	Ubiquinone biosynthesis protein COQ7 homolog	IPI00294073	0.000	0.22163	0	0	2	0
2045	Beta-adrenergic receptor kinase 1	IPI00012497	0.000	0.22163	2	0	0	0
2046	Isoform 1 of Protein FAM169A	IPI00737638	0.000	0.22163	2	0	0	0
2047	Wings apart-like homolog	IPI00103263	0.000	0.22163	2	0	2	2
2048	Fragile X mental retardation syndrome-related protein 2	IPI00016250	0.000	0.22163	0	0	0	0
2049	Selenoprotein O	IPI00031666	0.000	0.22163	2	0	0	0
2050	Phospholysine phosphohistidine inorganic pyrophosphate phosphatase	IPI00005474	0.000	0.22163	0	2	0	0
2051	GA-binding protein alpha chain	IPI00299413	0.000	0.22163	2	2	0	0
2052	Chromosome-associated kinesin KIF4B	IPI00175193	0.000	0.22163	2	0	0	0
2053	Isoform 2 of Abnormal spindle-like microcephaly-associated protein	IPI00480042	0.000	0.22163	0	0	0	0
2054	Isoform 1 of Ran guanine nucleotide release factor	IPI00025081	0.000	0.22163	2	0	0	0
2055	Syntaxin-6	IPI00013930	0.000	0.22163	2	0	0	0
2056	Isoform 1 of DnaJ homolog subfamily C member 21	IPI00142716	0.000	0.22163	2	0	0	0
2057	Isoform 2 of Golgin subfamily A member 2	IPI00413895	0.000	0.22163	2	0	0	0
2058	Protein AF1q	IPI00011421	0.000	0.22163	0	0	0	0
2059	AP-1 complex subunit sigma-2	IPI00922006	0.000	0.22163	0	0	2	2
2060	Isoform 1 of Non-homologous end-joining factor 1	IPI00549762	0.000	0.22163	0	0	0	0
2061	cDNA FLJ16404 fis, clone UTERU2008019, highly similar to Serine/threonine-protein kinase 3	IPI00411984	0.000	0.22163	2	0	0	2
2062	Isoform A of Methyl-CpG-binding protein 2	IPI00418234	0.000	0.22163	0	0	2	0
2063	Peroxisomal biogenesis factor 3	IPI00010232	0.000	0.22163	0	2	0	0
2064	Acidic leucine-rich nuclear phosphoprotein 32 family member A	IPI00025849	0.000	0.22163	2	0	0	0
2065	Trafficking protein particle complex subunit 1	IPI00009654	0.000	0.22163	2	0	0	0
2066	Isoform 1 of Upstream-binding protein 1	IPI00005018	0.000	0.22163	0	0	2	0
2067	Isoform Beta of LIM domain and actin-binding protein 1	IPI00008918	0.000	0.22163	0	0	2	2
2068	Isoform 1 of UPF0399 protein C6orf153	IPI00106638	0.000	0.22163	0	0	2	0
2069	Acyl-CoA-binding domain-containing protein 7	IPI00398874	0.000	0.22163	0	0	0	2
2070	Histone H2A type 1-H	IPI00081836	0.000	0.22163	0	2	0	2
2071	MIT domain-containing protein 1	IPI00103065	0.000	0.22163	0	0	0	0
2072	Isoform 1 of Kinesin-like protein KIF15	IPI00024975	0.000	0.22163	0	0	0	0
2073	Translocated promoter region	IPI00514531	0.000	0.22163	2	0	0	0
2074	Isoform 1 of Tumor susceptibility gene 101 protein	IPI00018434	0.000	0.22163	2	0	0	0
2075	Protein Njmu-R1	IPI00029473	0.000	0.22163	2	0	0	0
2076	Cation transport regulator-like protein 2	IPI00103047	0.000	0.22163	0	0	0	0
2077	Putative uncharacterized protein ZYX	IPI00924931	0.000	0.22163	0	0	0	0

No.	Description	Accession number	STN[1]	p-Value[1]	Con_A[2]	Con_B[2]	5FU_A[2]	5FU_B[2]
2078	Putative uncharacterized protein CTU2	IPI00418412	0.000	0.22163	0	0	0	0
2079	Isoform 1 of SOSS complex subunit B1	IPI00031633	0.000	0.22163	2	0	0	0
2080	Isoform 4 of Phosphorylase b kinase regulatory subunit beta	IPI00181893	0.000	0.22163	0	0	2	0
2081	Isoform 1 of CAP-Gly domain-containing linker protein 1	IPI00013455	0.000	0.22163	0	0	0	2
2082	Isoform 1 of PDZ and LIM domain protein 7	IPI00023122	0.000	0.22163	0	2	0	0
2083	Cellular retinoic acid-binding protein 1	IPI00219930	0.000	0.22163	0	0	0	0
2084	Isoform 7 of Rab GTPase-activating protein 1-like	IPI00006648	0.000	0.22163	2	0	0	0
2085	Arginase-2, mitochondrial	IPI00020332	0.000	0.22163	0	0	0	2
2086	Isoform 1 of Glutaminase kidney isoform, mitochondrial	IPI00289159	0.000	0.22163	2	0	0	0
2087	Catechol O-methyltransferase domain-containing protein 1	IPI00642041	0.000	0.22163	0	0	0	0
2088	Isoform 1 of Collagen type IV alpha-3-binding protein	IPI00024701	0.000	0.22163	2	0	0	0
2089	Isoform 1 of Microtubule-associated protein 4	IPI00396171	0.000	0.22163	2	0	0	2
2090	Isoform 2 of Alkylated DNA repair protein alkB homolog 5	IPI00413659	0.000	0.22163	0	0	0	0
2091	AT-rich interactive domain-containing protein 3A	IPI00018500	0.000	0.22163	2	2	0	0
2092	Glioma tumor suppressor candidate region gene 2 protein	IPI00024567	0.000	0.22163	0	2	0	0
2093	DDB1- and CUL4-associated factor 13	IPI00306642	0.000	0.22163	0	2	0	0
2094	Isoform 1 of Autophagy-related protein 7	IPI00007404	0.000	0.22163	2	0	0	0
2095	82 kDa protein	IPI00719051	0.000	0.22163	0	0	2	0
2096	Isoform 2 of Transportin-2	IPI00164417	0.000	0.22163	0	0	0	0
2097	DNA-directed RNA polymerase II subunit RPB4	IPI00007283	0.000	0.22163	0	0	0	2
2098	Thioredoxin-related transmembrane protein 4	IPI00100247	0.000	0.22163	0	0	0	0
2099	Wilm's tumour protein family protein	IPI00044748	0.000	0.22163	0	0	0	0
2100	Isoform 1 of Set1/Ash2 histone methyltransferase complex subunit ASH2	IPI00328658	0.000	0.22163	0	0	0	0
2101	Diphthamide biosynthesis protein 2	IPI00106549	0.000	0.22163	0	0	2	2
2102	Isoform 1 of Hepatocyte growth factor-regulated tyrosine kinase substrate	IPI00006176	0.000	0.22163	2	0	0	0
2103	TIM21-like protein, mitochondrial	IPI00306439	0.000	0.22163	0	0	0	0
2104	Serine/threonine-protein kinase Chk1	IPI00023664	0.000	0.22163	2	0	0	0
2105	Isoform 1 of Pre-mRNA-splicing factor 18	IPI00021146	0.000	0.22163	2	0	0	0
2106	Protein FAM3C	IPI00334282	0.000	0.22163	0	0	0	2
2107	Mevalonate kinase	IPI00010717	0.000	0.22163	2	0	0	0
2108	NEDD4-like E3 ubiquitin-protein ligase WWP2	IPI00013010	0.000	0.22163	0	2	0	0
2109	Isoform 1 of Uncharacterized protein C10orf46	IPI00410319	0.000	0.22163	2	0	0	0
2110	Putative uncharacterized protein DKFZp686C1054	IPI00465054	0.000	0.22163	0	0	0	0
2111	Isoform 1 of Proteasomal ATPase-associated factor 1	IPI00743862	0.000	0.22163	2	0	0	0
2112	Isoform 1 of Nucleotide-binding protein-like	IPI00384517	0.000	0.22163	0	2	0	0
2113	Putative uncharacterized protein MRPS24	IPI00000181	0.000	0.22163	0	0	2	0
2114	Probable asparaginyl-tRNA synthetase, mitochondrial	IPI00101664	0.000	0.22163	2	0	2	0
2115	Isoform 2 of Syntaxin-10	IPI00178839	0.000	0.22163	2	2	0	0
2116	Mitochondrial folate transporter/carrier	IPI00300886	0.000	0.22163	2	0	0	0
2117	Monoacylglycerol lipase ABHD6	IPI00107039	0.000	0.22163	2	0	0	0
2118	Protein ariadne-1 homolog	IPI00294943	0.000	0.22163	0	0	0	0
2119	Putative uncharacterized protein DKFZp686G0859	IPI00470477	0.000	0.22163	2	0	0	0
2120	3-ketoacyl-CoA thiolase, peroxisomal	IPI00012828	0.000	0.22163	2	0	0	0
2121	Isoform 1 of Dynamin-1-like protein	IPI00146935	0.000	0.22163	0	2	0	0
2122	UPF0361 protein C3orf37	IPI00024618	0.000	0.22163	2	0	0	0
2123	Isoform 2 of Pre-mRNA-splicing regulator WTAP	IPI00014150	0.000	0.22163	0	0	0	2
2124	Isoform 1 of Casein kinase I isoform delta	IPI00011102	0.000	0.22163	0	0	0	0
2125	Uncharacterized protein C18orf8	IPI00149964	0.000	0.22163	2	0	0	0
2126	Ran-binding protein 10	IPI00039864	0.000	0.22163	0	0	0	0
2127	Isoform 3 of Disabled homolog 2-interacting protein	IPI00045600	0.000	0.22163	0	0	2	0
2128	Isoform 1 of MAP kinase-activating death domain protein	IPI00103536	0.000	0.22163	0	2	0	0
2129	Threonine synthase-like 1	IPI00016287	0.000	0.22163	2	0	0	0
2130	Isoform 2 of ADP-ribosylation factor-binding protein GGA1	IPI00216337	0.000	0.22163	2	0	0	0
2131	cDNA FLJ56370, highly similar to Homo sapiens FK506 binding protein 8, 38kDa (FKBP8), mRNA	IPI00328161	0.000	0.22163	2	2	0	0
2132	NEDD4-binding protein 1	IPI00005667	0.000	0.22163	0	0	0	2
2133	Isoform 1 of Kinesin-like protein KIF16B	IPI00452246	0.000	0.22163	2	0	0	0
2134	Ubiquitin carboxyl-terminal hydrolase 3	IPI00002330	0.000	0.22163	0	0	2	0
2135	Isoform 1 of Epidermal growth factor receptor substrate 15	IPI00292134	0.000	0.22163	0	0	0	2
2136	Tetratricopeptide repeat protein 1	IPI00016912	0.000	0.22163	0	0	2	0
2137	Isoform 1 of 28S ribosomal protein S5, mitochondrial	IPI00169400	0.000	0.22163	0	0	0	0
2138	Isoform 1 of Dynamin-binding protein	IPI00174025	0.000	0.22163	0	0	2	2
2139	cDNA FLJ55996, highly similar to Conserved oligomeric Golgi complex component 8	IPI00140201	0.000	0.22163	0	2	0	0
2140	Isoform 2 of DnaJ homolog subfamily A member 4	IPI00465105	0.000	0.22163	2	0	0	0
2141	Isoform Mitochondrial of Malonyl-CoA decarboxylase, mitochondrial	IPI00000663	0.000	0.22163	0	0	0	0
2142	Putative uncharacterized protein CCNK	IPI00411579	0.000	0.22163	2	0	2	0
2143	Golgi SNAP receptor complex member 1	IPI00029447	0.000	0.22163	0	0	0	0
2144	Hydroxymethylglutaryl-CoA synthase, cytoplasmic	IPI00008475	0.000	0.22163	0	0	0	0
2145	Ras-related protein Rap-2a	IPI00019346	0.000	0.22163	0	0	0	0
2146	Vacuolar protein sorting-associated protein 45	IPI00090327	0.000	0.22163	0	0	2	0
2147	Calcium signal-modulating cyclophilin ligand	IPI00025729	0.000	0.22163	0	2	0	0
2148	Splicing factor, arginine/serine-rich 19	IPI00303343	0.000	0.22163	0	0	0	2
2149	Putative uncharacterized protein QTRTD1	IPI00074010	0.000	0.22163	2	2	0	0
2150	Neuron-specific calcium-binding protein hippocalcin	IPI00219103	0.000	0.22163	0	0	2	0
2151	cDNA FLJ56157, highly similar to Glucosylceramidase	IPI00021807	0.000	0.22163	0	0	0	2
2152	Isoform 1 of Vesicle-associated membrane protein 7	IPI00020887	0.000	0.22163	0	0	0	0
2153	Isoform 1 of NADH dehydrogenase [ubiquinone] 1 alpha subcomplex subunit 11	IPI00329301	0.000	0.22163	0	0	0	0
2154	Isoform 1 of SH3 domain-containing kinase-binding protein 1	IPI00294962	0.000	0.22163	0	0	0	0
2155	Protein Hook homolog 1	IPI00026305	0.000	0.22163	0	0	0	0
2156	Isoform 2 of Regulation of nuclear pre-mRNA domain-containing protein 1A	IPI00062336	0.000	0.22163	0	2	0	0
2157	Isoform 1 of Arginine/serine-rich coiled-coil protein 2	IPI00419791	0.000	0.22163	0	0	0	0
2158	Isoform 1 of UPF0489 protein C5orf22	IPI00019966	0.000	0.22163	0	0	0	2
2159	Isoform 2 of DnaJ homolog subfamily C member 2	IPI00455199	0.000	0.22163	0	0	2	0
2160	Hydroxymethylglutaryl-CoA lyase, mitochondrial	IPI00293564	0.000	0.22163	2	0	0	0
2161	Phosphatidylinositol-5-phosphate 4-kinase type-2 alpha	IPI00009688	0.000	0.22163	0	2	0	0
2162	Isoform 2 of DNA replication complex GINS protein PSF3	IPI00185097	0.000	0.22163	2	0	0	2
2163	Isoform 1 of Cell division cycle protein 16 homolog	IPI00022091	0.000	0.22163	2	0	0	0
2164	Syndecan-1	IPI00002441	0.000	0.22163	0	0	0	2
2165	Isoform 2 of RNA-binding protein 47	IPI00005042	0.000	0.22163	0	2	0	0
2166	Isoform 1 of NADH dehydrogenase [ubiquinone] flavoprotein 1, mitochondrial	IPI00028520	0.000	0.22163	2	0	0	0
2167	Transcriptional activator protein Pur-alpha	IPI00023591	0.000	0.22163	0	0	2	0
2168	Isoform 2 of Serine/threonine-protein kinase tousled-like 2	IPI00337659	0.000	0.22163	2	0	0	0
2169	arf-GAP with GTPase, ANK repeat and PH domain-containing protein 3 isoform a	IPI00163185	0.000	0.22163	2	0	0	0
2170	164 kDa protein	IPI00465246	0.000	0.22163	0	0	0	0
2171	Coiled-coil domain-containing protein 22	IPI00022265	0.000	0.22163	0	0	0	2
2172	DNA repair endonuclease XPF	IPI00219179	0.000	0.22163	0	0	0	0

No.	Description	Accession number	STN[1]	p-Value[1]	Con_A[2]	Con_B[2]	5FU_A[2]	5FU_B[2]
2173	Kinetochore protein Spc25	IPI00010219	0.000	0.22163	0	0	0	2
2174	WASH complex subunit CCDC53	IPI00032848	0.000	0.22163	0	0	0	2
2175	MORC family CW-type zinc finger 3	IPI00436705	0.000	0.22163	0	0	0	0
2176	Isoform 1 of Golgi-associated PDZ and coiled-coil motif-containing protein	IPI00186721	0.000	0.22163	0	0	0	0
2177	Putative heat shock 70 kDa protein 7	IPI00011134	0.000	0.22163	2	0	0	0
2178	Isoform 2 of SAP30-binding protein	IPI00333699	0.000	0.22163	2	2	0	0
2179	Isoform 1 of Transmembrane protein 163	IPI00152253	0.000	0.22163	0	2	0	0
2180	Isoform Alpha of DNA fragmentation factor subunit beta (Fragment)	IPI00008794	0.000	0.22163	0	0	0	0
2181	Notchless protein homolog 1	IPI00018196	0.000	0.22163	0	2	0	0
2182	Isoform 2 of Zinc finger C3H1 domain-containing protein	IPI00175146	0.000	0.22163	2	0	0	0
2183	Glycogen synthase kinase-3 alpha	IPI00292228	0.000	0.22163	2	0	0	0
2184	Coiled-coil domain-containing protein 137	IPI00401962	0.000	0.22163	2	2	0	0
2185	Beta-centractin	IPI00029469	0.000	0.22163	0	0	0	0
2186	DNA polymerase epsilon subunit 4	IPI00008436	0.000	0.22163	0	0	0	0
2187	Mitochondrial import inner membrane translocase subunit TIM14	IPI00304306	0.000	0.22163	0	0	0	0
2188	Isoform 1 of PHD finger protein 6	IPI00395568	0.000	0.22163	0	0	0	2
2189	Pre-mRNA-splicing factor SLU7	IPI00294435	0.000	0.22163	0	0	0	0
2190	Isoform 1 of Rho GTPase-activating protein 17	IPI00064767	0.000	0.22163	0	0	0	2
2191	Dual specificity protein phosphatase 14	IPI00013031	0.000	0.22163	0	0	0	2
2192	NFU1 iron-sulfur cluster scaffold homolog, mitochondrial isoform 1	IPI00160021	0.000	0.22163	0	0	0	2
2193	CCR4-NOT transcription complex subunit 8	IPI00295501	0.000	0.22163	0	0	2	0
2194	Isoform 1 of High mobility group protein 20A	IPI00018924	0.000	0.22163	0	0	2	0
2195	Isoform 1 of DNA-directed RNA polymerase I subunit RPA49	IPI00251989	0.000	0.22163	0	0	0	2
2196	Isoform B of Syntaxin-3	IPI00220099	0.000	0.22163	0	0	0	0
2197	Isoform 1 of Cell cycle checkpoint protein RAD1	IPI00003647	0.000	0.22163	0	0	0	0
2198	Aflatoxin B1 aldehyde reductase member 3	IPI00293721	0.000	0.22163	0	0	0	2
2199	Putative uncharacterized protein NMD3	IPI00101049	0.000	0.22163	0	0	0	0
2200	WD repeat-containing protein 13	IPI00016988	0.000	0.22163	0	2	0	0
2201	DNA methyltransferase 1-associated protein 1	IPI00219919	0.000	0.22163	0	0	2	0
2202	Ras-related protein Rab-43	IPI00329441	0.000	0.22163	0	0	0	0
2203	Zinc finger CCHC domain-containing protein 9	IPI00410284	0.000	0.22163	0	0	0	0
2204	Isoform 1 of Cysteine protease ATG4B	IPI00554649	0.000	0.22163	0	0	0	0
2205	RWD domain-containing protein 1	IPI00034010	0.000	0.22163	0	2	0	0
2206	Isoform L1 of Smoothelin	IPI00219460	0.000	0.22163	0	0	2	2
2207	Isoform 1 of Protein midA homolog, mitochondrial	IPI00412670	0.000	0.22163	2	0	0	0
2208	nuclear factor NF-kappa-B p100 subunit isoform b	IPI00807463	0.000	0.22163	0	0	0	2
2209	cDNA, FLJ79450, highly similar to 3-ketoacyl-CoA thiolase, peroxisomal	IPI00011522	0.000	0.22163	0	0	0	0
2210	Ubiquitin-associated protein 2	IPI00171127	0.000	0.22163	0	0	0	0
2211	KIAA1033 protein	IPI00298991	0.000	0.22163	0	0	0	0
2212	Alpha- and gamma-adaptin-binding protein p34	IPI00100193	0.000	0.22163	2	0	0	0
2213	Guanine nucleotide-binding protein subunit alpha-11	IPI00305551	0.000	0.22163	0	0	2	0
2214	cDNA FLJ54752, highly similar to Poly(rC)-binding protein 2	IPI00788837	0.000	0.22163	0	0	0	0
2215	Origin recognition complex subunit 6	IPI00001641	0.000	0.22163	0	2	0	0
2216	Isoform 1 of NGFI-A-binding protein 2	IPI00019432	0.000	0.22163	0	2	0	0
2217	Isoform 3 of ATPase family AAA domain-containing protein 3B	IPI00178879	0.000	0.22163	2	0	0	0
2218	Isoform 1 of mTERF domain-containing protein 1, mitochondrial	IPI00101163	0.000	0.22163	2	0	0	0
2219	Isoform Alpha' of Caspase-7	IPI00216675	0.000	0.22163	0	0	0	0
2220	Nuclear pore complex protein Nup88	IPI00001738	0.000	0.22163	0	0	0	0
2221	Syntaxin-17	IPI00012028	0.000	0.22163	2	0	0	0
2222	General transcription factor IIH subunit 1	IPI00030380	0.000	0.22163	0	0	0	0
2223	Isoform 1 of Quinone oxidoreductase PIG3	IPI00384643	0.000	0.22163	0	0	2	0
2224	Isoform 1 of Kinesin light chain 4	IPI00398812	0.000	0.22163	0	0	0	2
2225	Peptidyl-tRNA hydrolase family protein	IPI00048572	0.000	0.22163	0	0	0	2
2226	Uncharacterized protein C20orf29	IPI00019941	0.000	0.22163	2	0	0	0
2227	Keratin-81-like protein	IPI00008669	0.000	0.22163	0	0	0	2
2228	CTD small phosphatase-like protein (CTDSP-like)	IPI00024826	0.000	0.22163	0	0	0	0
2229	Isoform Beta of Zinc finger protein RFP	IPI00412657	0.000	0.22163	0	0	2	0
2230	Histone acetyltransferase MYST2	IPI00180764	0.000	0.22163	0	0	2	0
2231	Isoform 1 of Hematological and neurological expressed 1 protein	IPI00007764	0.000	0.22163	0	0	0	0
2232	Isoform 1 of Choline kinase alpha	IPI00409761	0.000	0.22163	0	0	0	0
2233	Isoform 1 of 5'-tyrosyl-DNA phosphodiesterase	IPI00009913	0.000	0.22163	0	2	0	0
2234	Isoform Alpha of Tripartite motif-containing protein 29	IPI00073096	0.000	0.22163	0	0	2	0
2235	Isoform 2 of Rab-3A-interacting protein	IPI00044842	0.000	0.22163	0	0	2	0
2236	Isoform 1 of CCR4-NOT transcription complex subunit 3	IPI00005015	0.000	0.22163	0	0	0	0
2237	interleukin-1 receptor-associated kinase 1 isoform 3	IPI00060149	0.000	0.22163	0	0	0	0
2238	Adenylyltransferase and sulfurtransferase MOCS3	IPI00004489	0.000	0.22163	2	0	0	0
2239	Isoform 3 of Protein FAM122B	IPI00152151	0.000	0.22163	0	0	2	0
2240	Isoform 2 of Fibronectin type-III domain-containing protein 3A	IPI00456630	0.000	0.22163	0	0	0	0
2241	Ribosyldihydronicotinamide dehydrogenase [quinone]	IPI00219129	0.000	0.22163	0	0	0	0
2242	cDNA FLJ55772, highly similar to Rab5 GDP/GTP exchange factor	IPI00004974	0.000	0.22163	2	0	0	0
2243	Isoform 1 of Splicing factor 3B subunit 3	IPI00300371	-0.050	0.36640	88	86	85	88
2244	L-lactate dehydrogenase B chain	IPI00219217	-0.051	0.36640	83	78	79	81
2245	Isoform 1 of Heterogeneous nuclear ribonucleoprotein M	IPI00171903	-0.063	0.36640	50	38	40	47
2246	ATP synthase subunit alpha, mitochondrial	IPI00440493	-0.064	0.36640	48	36	42	41
2247	N-acetyltransferase 10	IPI00300127	-0.070	0.36625	29	34	28	34
2248	C-1-tetrahydrofolate synthase, cytoplasmic	IPI00218342	-0.071	0.36625	36	25	28	32
2249	60S ribosomal protein L3	IPI00550021	-0.071	0.36614	36	23	29	29
2250	NCL protein	IPI00183526	-0.073	0.36599	38	18	24	31
2251	Glutamate dehydrogenase 1, mitochondrial	IPI00016801	-0.074	0.36595	31	22	26	26
2252	Probable ATP-dependent RNA helicase DDX5	IPI00017617	-0.075	0.36595	30	21	21	29
2253	ATP-citrate synthase	IPI00021290	-0.077	0.36558	26	20	18	27
2254	Isoform SM-B' of Small nuclear ribonucleoprotein-associated proteins B and B'	IPI00027285	-0.079	0.36558	24	20	20	23
2255	cDNA FLJ56389, highly similar to Elongation factor 1-gamma	IPI00000875	-0.080	0.36554	25	17	25	16
2256	Eukaryotic translation initiation factor 5B	IPI00299254	-0.082	0.36506	22	16	20	17
2257	Vigilin	IPI00022228	-0.084	0.36454	22	14	19	16
2258	Isoform 1 of Leukotriene A-4 hydrolase	IPI00219077	-0.086	0.36413	18	15	17	15
2259	Beta-actin-like protein 2	IPI00003269	-0.087	0.36394	19	13	18	13
2260	Proteasome subunit beta type-3	IPI00028004	-0.087	0.36394	16	16	19	12
2261	Rab GDP dissociation inhibitor alpha	IPI00010154	-0.088	0.36372	21	10	16	14
2262	UDP-glucose 6-dehydrogenase	IPI00031420	-0.088	0.36372	16	15	14	16
2263	Isoform 1 of ATP-dependent RNA helicase DDX19B	IPI00008943	-0.088	0.36372	14	17	14	16
2264	CTP synthase 1	IPI00290142	-0.089	0.36346	20	10	13	16
2265	Endoplasmic reticulum resident protein 29	IPI00024911	-0.089	0.36346	15	15	16	13
2266	3-hydroxyisobutyrate dehydrogenase, mitochondrial	IPI00013860	-0.091	0.36294	13	15	13	14
2267	Isoform 1 of Ubiquitin-like modifier-activating enzyme 6	IPI00023647	-0.092	0.36204	15	12	10	16

No.	Description	Accession number	STN[1]	p-Value[1]	Con_A[2]	Con_B[2]	5FU_A[2]	5FU_B[2]
2268	Isoform 1 of Kinectin	IPI00328753	-0.092	0.36204	16	11	13	13
2269	Annexin A1	IPI00218918	-0.092	0.36204	16	11	17	9
2270	ATP synthase subunit O, mitochondrial	IPI00007611	-0.092	0.36204	16	11	14	12
2271	Isoform 1 of CCR4-NOT transcription complex subunit 1	IPI00166010	-0.094	0.36174	15	11	15	10
2272	Probable ATP-dependent RNA helicase DDX6	IPI00030320	-0.095	0.36096	16	9	12	12
2273	Actin-related protein 2/3 complex subunit 4	IPI00554811	-0.096	0.36063	12	12	14	9
2274	Eukaryotic translation initiation factor 1A, Y-chromosomal	IPI00023004	-0.099	0.35985	15	7	14	7
2275	Ribonucleoside-diphosphate reductase large subunit	IPI00013871	-0.100	0.35903	14	7	11	9
2276	Dolichyl-diphosphooligosaccharide--protein glycosyltransferase 48 kDa subunit	IPI00297084	-0.100	0.35903	18	3	12	8
2277	29 kDa protein	IPI00453476	-0.102	0.35858	10	10	10	9
2278	Glucosamine 6-phosphate N-acetyltransferase	IPI00061525	-0.102	0.35858	10	10	8	11
2279	NADH-ubiquinone oxidoreductase 75 kDa subunit	IPI00604664	-0.102	0.35858	11	9	9	10
2280	Protein flightless-1 homolog	IPI00031023	-0.104	0.35802	11	8	9	9
2281	cDNA FLJ34068 fis, clone FCBBF3001918	IPI00168184	-0.106	0.35757	15	3	8	9
2282	Chloride intracellular channel protein 4	IPI00001960	-0.106	0.35757	9	9	9	8
2283	proteasome 26S non-ATPase subunit 8	IPI00010201	-0.106	0.35757	10	8	12	5
2284	Isoform 1 of Cirhin	IPI00239815	-0.106	0.35757	11	7	9	8
2285	Carbonyl reductase [NADPH] 1	IPI00295386	-0.106	0.35757	10	8	9	8
2286	Heme oxygenase 2	IPI00026824	-0.108	0.35657	11	6	7	9
2287	87 kDa protein	IPI00220365	-0.108	0.35657	7	10	6	10
2288	Isoform 2 of Suppressor of G2 allele of SKP1 homolog	IPI00791573	-0.108	0.35657	10	7	8	8
2289	Histone acetyltransferase type B catalytic subunit	IPI00024719	-0.108	0.35657	12	5	10	6
2290	14-3-3 protein eta	IPI00216319	-0.108	0.35657	10	7	9	7
2291	Coatomer subunit delta variant 2	IPI00298520	-0.110	0.35590	7	9	8	7
2292	ERO1-like protein alpha	IPI00386755	-0.110	0.35590	11	5	9	6
2293	Isoform 1 of LIM and SH3 domain protein 1	IPI00000861	-0.110	0.35590	7	9	8	7
2294	Diablo homolog, mitochondrial precursor	IPI00008418	-0.110	0.35590	8	8	9	6
2295	Insulin-degrading enzyme	IPI00220373	-0.112	0.35378	7	8	5	9
2296	Isoform 5 of Dynamin-1-like protein	IPI00037283	-0.112	0.35378	10	5	2	12
2297	Isoform 1 of Replication factor C subunit 2	IPI00017412	-0.112	0.35378	13	0	8	6
2298	Isoform 1AB of Catenin delta-1	IPI00182469	-0.112	0.35378	11	4	6	8
2299	Eukaryotic translation elongation factor 1 epsilon-1	IPI00003588	-0.112	0.35378	9	6	6	8
2300	E3 ubiquitin-protein ligase BRE1A	IPI00251559	-0.112	0.35378	7	8	6	8
2301	Putative ATP-dependent Clp protease proteolytic subunit, mitochondrial	IPI00003870	-0.112	0.35378	7	8	7	7
2302	Pre-mRNA-processing factor 6	IPI00305068	-0.112	0.35378	8	7	7	7
2303	Emerin	IPI00032003	-0.115	0.35244	8	6	6	7
2304	Isoform 1 of Nuclear pore complex protein Nup98-Nup96	IPI00006038	-0.115	0.35244	7	7	6	7
2305	Translocon-associated protein subunit delta precursor	IPI00019385	-0.115	0.35244	8	6	7	6
2306	Putative rRNA methyltransferase 3	IPI00217686	-0.115	0.35244	10	4	8	5
2307	Isoform Crk-II of Adapter molecule crk	IPI00004838	-0.115	0.35244	7	7	6	7
2308	Isoform 2 of Obg-like ATPase 1	IPI00216105	-0.115	0.35244	11	3	9	4
2309	S-adenosylmethionine synthase isoform type-2	IPI00010157	-0.115	0.35244	7	7	7	6
2310	Transmembrane protein 165	IPI00307572	-0.115	0.35244	8	6	7	6
2311	Isoform 1 of Serine/threonine-protein kinase WNK1	IPI00004472	-0.115	0.35244	9	5	5	8
2312	sideroflexin-3	IPI00793874	-0.115	0.35244	6	8	7	6
2313	Isoform 2 of Neutral alpha-glucosidase AB	IPI00011454	-0.118	0.35031	65	42	47	58
2314	40S ribosomal protein S6	IPI00021840	-0.118	0.35020	6	7	6	6
2315	33 kDa protein	IPI00413108	-0.118	0.35020	9	4	5	7
2316	Alpha-centractin	IPI00029468	-0.118	0.35020	9	4	6	6
2317	Protein NipSnap homolog 3A	IPI00004845	-0.118	0.35020	7	6	6	6
2318	Exocyst complex component 4	IPI00059279	-0.118	0.35020	5	8	6	6
2319	Quinone oxidoreductase	IPI00000792	-0.118	0.35020	8	5	8	4
2320	Isoform 3 of PCI domain-containing protein 2	IPI00072541	-0.118	0.35020	6	7	7	5
2321	RNA binding motif protein, X-linked-like 1	IPI00061178	-0.118	0.35020	8	5	6	6
2322	39S ribosomal protein L13, mitochondrial	IPI00022403	-0.118	0.35020	7	6	6	6
2323	Ornithine aminotransferase, mitochondrial	IPI00022334	-0.118	0.35020	7	6	6	6
2324	Calcium-binding mitochondrial carrier protein Aralar1	IPI00386271	-0.118	0.35020	8	5	5	7
2325	Isoform 1 of Vacuolar protein sorting-associated protein 29	IPI00170796	-0.121	0.34886	5	7	6	5
2326	1,4-alpha-glucan-branching enzyme	IPI00296635	-0.121	0.34886	8	4	4	7
2327	Aspartate aminotransferase, cytoplasmic	IPI00219029	-0.121	0.34886	8	4	8	3
2328	Isoform 1 of Enolase-phosphatase E1	IPI00038378	-0.121	0.34886	8	4	7	4
2329	39S ribosomal protein L15, mitochondrial	IPI00023086	-0.121	0.34886	5	7	5	6
2330	Parafibromin	IPI00300659	-0.121	0.34886	5	7	6	5
2331	Nuclear pore complex protein Nup153	IPI00292059	-0.121	0.34886	7	5	5	6
2332	Serine/threonine-protein phosphatase 2A 55 kDa regulatory subunit B alpha isoform	IPI00332511	-0.121	0.34886	5	7	6	5
2333	Isoform 1 of Retinol dehydrogenase 11	IPI00339384	-0.121	0.34886	5	7	7	4
2334	Isoform 1 of Nucleolar RNA helicase 2	IPI00015953	-0.122	0.34529	53	42	44	49
2335	Isoform 2 of Nucleosome-remodeling factor subunit BPTF	IPI00254408	-0.125	0.34529	3	8	4	6
2336	Isoform 2 of Myosin-XVIIIa	IPI00334410	-0.125	0.34529	6	5	4	6
2337	Isoform 1 of ATPase family AAA domain-containing protein 1	IPI00171445	-0.125	0.34529	5	6	7	3
2338	Isoform 2 of COP9 signalosome complex subunit 2	IPI00018813	-0.125	0.34529	7	4	7	3
2339	Cytochrome b-c1 complex subunit 7	IPI00220416	-0.125	0.34529	6	5	6	4
2340	Eukaryotic translation initiation factor 3 subunit G	IPI00290460	-0.125	0.34529	6	5	5	5
2341	COP9 signalosome complex subunit 3	IPI00025721	-0.125	0.34529	6	5	6	4
2342	Pre-mRNA branch site protein p14	IPI00032827	-0.125	0.34529	6	5	6	4
2343	EF-hand domain-containing protein D2	IPI00060181	-0.125	0.34529	6	5	6	4
2344	Polyribonucleotide nucleotidyltransferase 1, mitochondrial	IPI00744711	-0.125	0.34529	5	6	6	4
2345	Uncharacterized protein C20orf72	IPI00001287	-0.125	0.34529	5	6	5	5
2346	Isoform 2 of Serine/threonine-protein kinase PAK 3	IPI00027382	-0.125	0.34529	5	6	5	5
2347	Ras-related protein Rab-6B	IPI00016891	-0.125	0.34529	5	6	5	5
2348	Isoform 1 of Polymerase I and transcript release factor	IPI00176903	-0.125	0.34529	6	5	6	4
2349	Isoform 1 of Putative deoxyribonuclease TATDN1	IPI00012463	-0.125	0.34529	6	5	5	5
2350	DnaJ homolog subfamily B member 1	IPI00015947	-0.129	0.34335	7	3	6	3
2351	Isoform 1 of Replication protein A 32 kDa subunit	IPI00013939	-0.129	0.34335	5	5	4	5
2352	Isoform 2 of Calpastatin	IPI00220857	-0.129	0.34335	7	3	4	5
2353	Activity-dependent neuroprotector homeobox protein	IPI00022215	-0.129	0.34335	7	3	6	3
2354	Phosphoglucomutase-2	IPI00550364	-0.129	0.34335	5	5	7	2
2355	Peptidyl-tRNA hydrolase 2, mitochondrial	IPI00032903	-0.129	0.34335	6	4	5	4
2356	Isoform 2 of Ubiquitin-associated protein 2-like	IPI00029019	-0.129	0.34335	5	5	5	4
2357	Isoform 1 of Erlin-2	IPI00026942	-0.129	0.34335	7	3	5	4
2358	Acyl-CoA dehydrogenase family member 9, mitochondrial	IPI00152981	-0.129	0.34335	6	4	4	5
2359	Protein LYRIC	IPI00328715	-0.129	0.34335	6	4	6	3
2360	Nitric oxide synthase-interacting protein	IPI00006408	-0.129	0.34335	5	5	5	4
2361	Nucleolar protein 16	IPI00032849	-0.129	0.34335	4	6	6	3
2362	Tubulin-specific chaperone E	IPI00018402	-0.129	0.34335	7	3	5	4

No.	Description	Accession number	STN[1]	p-Value[1]	Con_A[2]	Con_B[2]	5FU_A[2]	5FU_B[2]
2363	Isoform 5 of Protein polybromo-1	IPI00023097	-0.134	0.33896	6	3	6	0
2364	Isoform 1 of Putative ATP-dependent RNA helicase DHX30	IPI00411733	-0.134	0.33896	5	4	3	5
2365	NADH dehydrogenase [ubiquinone] 1 beta subcomplex subunit 6	IPI00219385	-0.134	0.33896	6	3	4	4
2366	Rho-associated protein kinase 1	IPI00022542	-0.134	0.33896	5	4	3	5
2367	Isoform 2 of Nuclear protein localization protein 4 homolog	IPI00001676	-0.134	0.33896	5	4	6	2
2368	bifunctional protein NCOAT isoform b	IPI00181391	-0.134	0.33896	4	5	4	4
2369	Isoform CNPI of 2',3'-cyclic-nucleotide 3'-phosphodiesterase	IPI00220993	-0.134	0.33896	4	5	4	4
2370	Isoform 1 of Melanoma-associated antigen D2	IPI00009542	-0.134	0.33896	7	0	4	4
2371	Armadillo repeat-containing X-linked protein 3	IPI00009906	-0.134	0.33896	6	3	6	0
2372	Isoform 1 of Protein CDV3 homolog	IPI00014197	-0.134	0.33896	4	5	5	3
2373	Adenylosuccinate synthetase isozyme 2	IPI00026833	-0.134	0.33896	6	3	4	4
2374	Ubiquitin-conjugating enzyme E2 O	IPI00783378	-0.134	0.33896	4	5	4	4
2375	Isoform 1 of Replication factor C subunit 1	IPI00375358	-0.134	0.33896	4	5	4	4
2376	cDNA FLJ60124, highly similar to Mitochondrial dicarboxylate carrier	IPI00005537	-0.139	0.33639	3	5	5	2
2377	N-alpha-acetyltransferase 10, NatA catalytic subunit	IPI00013184	-0.139	0.33639	4	4	5	2
2378	DnaJ homolog subfamily B member 11	IPI00008454	-0.139	0.33639	6	2	4	3
2379	LDLR chaperone MESD	IPI00399089	-0.139	0.33639	4	4	5	0
2380	Isoform 2 of 39S ribosomal protein L39, mitochondrial	IPI00084571	-0.139	0.33639	3	5	3	4
2381	Exosome complex exonuclease MTR3	IPI00073602	-0.139	0.33639	3	5	3	4
2382	Isoform 1 of Probable ATP-dependent RNA helicase DHX36	IPI00027415	-0.139	0.33639	4	4	3	4
2383	Isoform A of Ras GTPase-activating protein-binding protein 2	IPI00009057	-0.139	0.33639	5	3	3	4
2384	cAMP-dependent protein kinase type I-alpha regulatory subunit	IPI00021831	-0.139	0.33639	4	4	4	3
2385	39S ribosomal protein L27, mitochondrial	IPI00009444	-0.139	0.33639	5	3	3	4
2386	Nucleolar complex protein 4 homolog	IPI00031661	-0.139	0.33639	5	3	0	5
2387	annexin A6 isoform 2	IPI00002459	-0.139	0.33639	6	2	4	3
2388	NIF3L1 isoform gamma	IPI00451429	-0.139	0.33639	4	4	5	2
2389	Isoform 1 of Protein NDRG3	IPI00005605	-0.139	0.33639	4	4	4	3
2390	ADP-ribosylation factor GTPase-activating protein 2	IPI00297322	-0.139	0.33639	3	5	4	3
2391	Carbonyl reductase [NADPH] 3	IPI00290462	-0.139	0.33639	4	4	3	4
2392	Isoform 1 of Secretory carrier-associated membrane protein 1	IPI00005129	-0.146	0.32842	5	2	2	4
2393	Dolichyl-diphosphooligosaccharide--protein glycosyltransferase subunit DAD1	IPI00009407	-0.146	0.32842	2	5	2	4
2394	Cleavage stimulation factor subunit 3	IPI00015195	-0.146	0.32842	5	2	3	3
2395	Isoform 2 of N-alpha-acetyltransferase 15, NatA auxiliary subunit	IPI00032158	-0.146	0.32842	4	3	4	2
2396	4-hydroxyphenylpyruvate dioxygenase-like protein	IPI00063762	-0.146	0.32842	5	2	4	0
2397	Isoform 1 of COMM domain-containing protein 4	IPI00413500	-0.146	0.32842	3	4	3	3
2398	Calcium-binding protein 39	IPI00032561	-0.146	0.32842	4	3	4	2
2399	rho GTPase-activating protein 4 isoform 1	IPI00328842	-0.146	0.32842	4	3	2	4
2400	14 kDa phosphohistidine phosphatase	IPI00299977	-0.146	0.32842	3	4	4	0
2401	cDNA FLJ78497	IPI00289535	-0.146	0.32842	4	3	2	4
2402	Osteoclast-stimulating factor 1	IPI00414836	-0.146	0.32842	3	4	3	3
2403	Isoform 1 of Putative methyltransferase METT10D	IPI00163391	-0.146	0.32842	5	0	0	4
2404	dCTP pyrophosphatase 1	IPI00012197	-0.146	0.32842	4	3	4	0
2405	Serine/threonine-protein phosphatase 5	IPI00019812	-0.146	0.32842	4	3	4	2
2406	TBC1 domain family member 15 isoform 1	IPI00154645	-0.146	0.32842	4	3	4	2
2407	HIV Tat-specific factor 1	IPI00013788	-0.146	0.32842	4	3	3	3
2408	Isoform GTBP-N of DNA mismatch repair protein Msh6	IPI00384456	-0.146	0.32842	4	3	2	4
2409	similar to unr-interacting protein	IPI00260209	-0.146	0.32842	4	3	4	2
2410	Isoform 1 of Pogo transposable element with ZNF domain	IPI00410717	-0.146	0.32842	5	0	3	3
2411	Glutathione S-transferase theta-1	IPI00741097	-0.146	0.32842	4	3	3	3
2412	inosine-5'-monophosphate dehydrogenase 1 isoform a	IPI00375527	-0.146	0.32842	2	5	3	3
2413	Isoform 3 of Myosin phosphatase Rho-interacting protein	IPI00166518	-0.146	0.32842	5	0	2	4
2414	Keratin-8-like protein 1	IPI00017870	-0.153	0.32447	26	22	20	26
2415	ATP-dependent RNA helicase DDX24	IPI00006987	-0.154	0.32443	4	2	3	2
2416	triosephosphate isomerase 1 isoform 2	IPI00465028	-0.154	0.32443	0	4	3	0
2417	Isoform 1 of Spermatid perinuclear RNA-binding protein	IPI00169430	-0.154	0.32443	2	4	3	2
2418	Adenylyl cyclase-associated protein	IPI00939159	-0.154	0.32443	4	2	3	0
2419	Putative uncharacterized protein EIF4E2	IPI00556081	-0.154	0.32443	2	4	2	3
2420	Mitochondrial import inner membrane translocase subunit Tim17-B	IPI00219833	-0.154	0.32443	0	4	3	0
2421	Cell differentiation protein RCD1 homolog	IPI00023101	-0.154	0.32443	3	3	3	2
2422	Putative HLA class I histocompatibility antigen, alpha chain H	IPI00004672	-0.154	0.32443	3	3	3	0
2423	Putative adenosylhomocysteinase 3	IPI00101645	-0.154	0.32443	0	4	2	3
2424	Isoform 2 of DnaJ homolog subfamily A member 3, mitochondrial	IPI00179187	-0.154	0.32443	3	3	3	0
2425	Branched-chain-amino-acid aminotransferase	IPI00181135	-0.154	0.32443	4	0	3	2
2426	Testis-expressed sequence 10 protein	IPI00549664	-0.154	0.32443	4	0	3	2
2427	Bifunctional 3'-phosphoadenosine 5'-phosphosulfate synthase 1	IPI00011619	-0.154	0.32443	3	3	0	3
2428	28S ribosomal protein S10, mitochondrial	IPI00061245	-0.154	0.32443	3	3	2	3
2429	Isoform 1 of Zinc finger protein 207	IPI00013457	-0.154	0.32443	4	2	3	2
2430	Protein ETHE1, mitochondrial	IPI00003766	-0.154	0.32443	4	2	3	2
2431	DnaJ homolog subfamily C member 3	IPI00006713	-0.154	0.32443	4	2	2	3
2432	V-type proton ATPase subunit G 1	IPI00025285	-0.154	0.32443	3	3	3	2
2433	39S ribosomal protein L2, mitochondrial	IPI00411816	-0.154	0.32443	3	3	2	3
2434	Isoform 1 of N-alpha-acetyltransferase 40, NatD catalytic subunit	IPI00328847	-0.154	0.32443	4	2	3	0
2435	Isoform 1 of Translation initiation factor eIF-2B subunit gamma	IPI00006504	-0.154	0.32443	4	0	3	0
2436	Putative uncharacterized protein DCP1A	IPI00164672	-0.154	0.32443	3	3	2	3
2437	Isoform 1 of Protein 4.1	IPI00003921	-0.154	0.32443	3	3	2	3
2438	Isoform 1 of Insulin-like growth factor 2 mRNA-binding protein 3	IPI00658000	-0.154	0.32443	4	0	3	2
2439	coatomer subunit epsilon isoform c	IPI00399319	-0.154	0.32443	4	0	3	2
2440	Uncharacterized protein C19orf52	IPI00157215	-0.154	0.32443	3	3	0	3
2441	Cell division protein kinase 3	IPI00023503	-0.154	0.32443	3	3	3	0
2442	cDNA FLJ56469, highly similar to Propionyl-CoA carboxylase alpha chain, mitochondrial	IPI00552419	-0.154	0.32443	2	4	3	2
2443	[Pyruvate dehydrogenase [acetyl-transferring]]-phosphatase 1, mitochondrial	IPI00218971	-0.154	0.32443	3	3	2	3
2444	Maleylacetoacetate isomerase	IPI00013809	-0.154	0.32443	0	4	0	3
2445	Isoform 3 of Keratin, type II cytoskeletal 80	IPI00375843	-0.154	0.32443	4	2	3	2
2446	Isoform SRP55-1 of Splicing factor, arginine/serine-rich 6	IPI00012345	-0.154	0.32443	4	2	3	2
2447	Isoform 1 of SAM domain and HD domain-containing protein 1	IPI00294739	-0.154	0.32443	4	2	2	3
2448	Enhancer of mRNA-decapping protein 3	IPI00018009	-0.154	0.32443	3	3	3	2
2449	Interleukin enhancer-binding factor 2	IPI00005198	-0.157	0.30254	28	17	23	20
2450	40S ribosomal protein S3	IPI00011253	-0.158	0.30254	70	81	76	72
2451	Heat shock protein HSP 90-beta	IPI00414676	-0.162	0.30150	77	62	71	65
2452	Isoform 1 of Myb-binding protein 1A	IPI00005024	-0.164	0.30105	25	14	15	22
2453	60S ribosomal protein L18a	IPI00026202	-0.165	0.30105	3	0	2	2
2454	Keratin, type I cytoskeletal 14	IPI00384444	-0.165	0.30105	2	3	2	2
2455	UPF0687 protein C20orf27	IPI00101095	-0.165	0.30105	3	2	0	0
2456	Isoform 1 of Serum paraoxonase/arylesterase 2	IPI00014958	-0.165	0.30105	3	0	2	0
2457	Adenosine deaminase	IPI00296441	-0.165	0.30105	0	3	0	0

No.	Description	Accession number	STN[1]	p-Value[1]	Con_A[2]	Con_B[2]	5FU_A[2]	5FU_B[2]
2458	Isoform 1 of Cytochrome c oxidase assembly protein COX15 homolog	IPI00419869	-0.165	0.30105	3	2	0	0
2459	Isoform IIA of Myc box-dependent-interacting protein 1	IPI00186966	-0.165	0.30105	3	2	2	2
2460	Ubiquitin-fold modifier-conjugating enzyme 1	IPI00294495	-0.165	0.30105	3	2	0	2
2461	Protein S100-A13	IPI00016179	-0.165	0.30105	2	3	2	2
2462	Golgi-specific brefeldin A-resistance guanine nucleotide exchange factor 1	IPI00021954	-0.165	0.30105	2	3	2	2
2463	40S ribosomal protein S11	IPI00025091	-0.165	0.30105	2	3	2	2
2464	Isoform 2 of Vacuolar protein sorting-associated protein 13A	IPI00478586	-0.165	0.30105	2	3	0	0
2465	U3 small nucleolar RNA-associated protein 15 homolog	IPI00152708	-0.165	0.30105	2	3	2	2
2466	Thioredoxin domain-containing protein 5	IPI00171438	-0.165	0.30105	3	0	0	0
2467	NADH dehydrogenase [ubiquinone] 1 alpha subcomplex subunit 8	IPI00219034	-0.165	0.30105	0	3	0	0
2468	N-alpha-acetyltransferase 38, NatC auxiliary subunit	IPI00219871	-0.165	0.30105	2	3	0	0
2469	Isoform 2 of Integrator complex subunit 3	IPI00418336	-0.165	0.30105	3	2	2	2
2470	E3 ubiquitin-protein ligase CBL	IPI00027269	-0.165	0.30105	3	2	0	0
2471	Isoform 2 of SWI/SNF-related matrix-associated actin-dependent regulator of chromatin subfamily A	IPI00008422	-0.165	0.30105	0	3	0	2
2472	Ribosome biogenesis protein BOP1	IPI00028955	-0.165	0.30105	2	3	0	2
2473	Splicing factor 3B subunit 5	IPI00010404	-0.165	0.30105	0	3	0	0
2474	Borealin	IPI00303099	-0.165	0.30105	3	2	2	0
2475	Exosome complex exonuclease RRP40	IPI00015956	-0.165	0.30105	3	2	0	2
2476	cDNA FLJ56280, highly similar to Endoplasmic reticulum-Golgi intermediate compartment protein 1	IPI00003635	-0.165	0.30105	2	3	2	2
2477	Isoform 1 of tRNA 2'-phosphotransferase 1	IPI00328580	-0.165	0.30105	3	0	2	0
2478	Syntaxin-binding protein 3	IPI00297626	-0.165	0.30105	0	3	0	0
2479	Transmembrane protein 11	IPI00012855	-0.165	0.30105	3	0	0	0
2480	Prolactin regulatory element-binding protein	IPI00033349	-0.165	0.30105	3	0	2	0
2481	SHC-transforming protein 1 isoform 3	IPI00021326	-0.165	0.30105	3	2	2	2
2482	Isoform 2 of Tether containing UBX domain for GLUT4	IPI00065276	-0.165	0.30105	2	3	2	2
2483	Isoform 1 of Dr1-associated corepressor	IPI00003084	-0.165	0.30105	3	0	2	0
2484	cDNA FLJ55543, highly similar to Phosphoacetylglucosamine mutase	IPI00030116	-0.165	0.30105	2	3	0	2
2485	Isoform 1 of Regulator of nonsense transcripts 2	IPI00300504	-0.165	0.30105	3	2	0	0
2486	Isoform 2 of Peptidyl-prolyl cis-trans isomerase-like 3	IPI00032473	-0.165	0.30105	3	2	2	2
2487	Beta-lactamase-like protein 2	IPI00006952	-0.165	0.30105	0	3	2	2
2488	RNA-binding protein 12	IPI00550308	-0.165	0.30105	3	2	2	2
2489	Isoform 1 of Serine/threonine-protein kinase N1	IPI00002803	-0.165	0.30105	3	2	2	2
2490	Isoform 1 of HEAT repeat-containing protein 3	IPI00100984	-0.165	0.30105	3	2	0	0
2491	V-type proton ATPase subunit D	IPI00001568	-0.165	0.30105	3	0	2	0
2492	LAG1 longevity assurance homolog 2	IPI00305304	-0.165	0.30105	3	0	0	0
2493	Eukaryotic translation initiation factor 4E	IPI00027485	-0.165	0.30105	0	3	0	2
2494	Isoform 2 of 5'-3' exoribonuclease 1	IPI00657645	-0.165	0.30105	2	3	0	0
2495	Kinetochore protein Spc24	IPI00168317	-0.165	0.30105	3	0	0	0
2496	Isoform 1 of Luc7-like protein 3	IPI00107745	-0.165	0.30105	3	2	2	0
2497	Tropomodulin-3	IPI00005087	-0.165	0.30105	3	0	2	2
2498	Protein KTI12 homolog	IPI00061528	-0.165	0.30105	3	2	2	2
2499	Origin recognition complex subunit 2	IPI00013216	-0.165	0.30105	3	2	0	0
2500	Transcription elongation factor B polypeptide 1	IPI00300341	-0.165	0.30105	3	0	2	0
2501	Isoform 1 of Chromodomain-helicase-DNA-binding protein 8	IPI00398992	-0.165	0.30105	2	3	2	2
2502	5'-nucleotidase domain-containing protein 1	IPI00177965	-0.165	0.30105	3	2	0	2
2503	cDNA FLJ56840, highly similar to Galactokinase	IPI00019383	-0.165	0.30105	0	3	2	0
2504	Isoform Long of Beta-glucuronidase	IPI00027745	-0.165	0.30105	3	0	0	2
2505	Isoform 1 of Protein syndesmos	IPI00031650	-0.165	0.30105	3	0	0	2
2506	Myeloid leukemia factor 2	IPI00023095	-0.165	0.30105	3	2	0	0
2507	Immediate early response 3-interacting protein 1	IPI00007166	-0.165	0.30105	2	3	2	0
2508	Cell division protein kinase 4	IPI00007811	-0.165	0.30105	2	3	0	0
2509	Ras-related protein Rap-1A	IPI00019345	-0.165	0.30105	3	0	0	0
2510	Ubiquilin-2	IPI00409659	-0.165	0.30105	3	0	2	2
2511	Isoform 1 of Calcium-binding mitochondrial carrier protein SCaMC-1	IPI00337494	-0.165	0.30105	3	2	0	0
2512	RNA-binding protein PNO1	IPI00024524	-0.165	0.30105	3	2	2	2
2513	Isoform 2 of Basic leucine zipper and W2 domain-containing protein 1	IPI00180128	-0.165	0.30105	3	0	0	0
2514	aldehyde dehydrogenase 9A1	IPI00479877	-0.165	0.30105	3	2	2	0
2515	Microtubule-associated protein 1S	IPI00296485	-0.165	0.30105	3	2	0	0
2516	Isoform 1 of HAUS augmin-like complex subunit 2	IPI00018198	-0.165	0.30105	3	0	0	0
2517	Cyclin-H	IPI00021305	-0.165	0.30105	3	2	2	0
2518	Myosin-11	IPI00020501	-0.165	0.30105	2	3	2	2
2519	Isoform 1 of HEAT repeat-containing protein 2	IPI00242630	-0.165	0.30105	3	0	2	0
2520	B-cell lymphoma/leukemia 10	IPI00022477	-0.165	0.30105	2	3	2	2
2521	Cysteine and glycine-rich protein 1	IPI00442073	-0.165	0.30105	3	2	2	2
2522	NHP2-like protein 1	IPI00026167	-0.165	0.30105	2	3	2	2
2523	E-cadherin	IPI00000513	-0.165	0.30105	3	2	2	2
2524	Probable U3 small nucleolar RNA-associated protein 11	IPI00180454	-0.165	0.30105	3	0	0	0
2525	Isoform 1 of C-terminal-binding protein 2	IPI00010120	-0.165	0.30105	3	0	0	2
2526	D-tyrosyl-tRNA(Tyr) deacylase 1	IPI00152692	-0.165	0.30105	0	3	0	0
2527	Gamma-taxilin	IPI00019994	-0.165	0.30105	3	2	0	0
2528	Isoform 1 of WD repeat-containing protein 74	IPI00018192	-0.165	0.30105	3	2	0	2
2529	Isoform 1 of tRNA guanosine-2'-O-methyltransferase TRM11 homolog	IPI00470606	-0.165	0.30105	3	2	2	0
2530	Isoform 1 of N6-adenosine-methyltransferase 70 kDa subunit	IPI00009755	-0.165	0.30105	2	3	2	2
2531	Prefoldin subunit 5	IPI00015361	-0.165	0.30105	0	3	2	0
2532	Glia maturation factor, beta	IPI00412987	-0.165	0.30105	3	0	2	0
2533	Isoform 1 of Lymphoid-specific helicase	IPI00010590	-0.165	0.30105	3	2	0	2
2534	U3 small nucleolar ribonucleoprotein protein MPP10	IPI00012149	-0.165	0.30105	3	2	2	2
2535	SCY1-like protein 2	IPI00396218	-0.165	0.30105	3	0	0	0
2536	Isoform 1 of Putative methyltransferase NSUN4	IPI00303944	-0.165	0.30105	0	3	2	2
2537	Mitochondrial intermediate peptidase	IPI00241860	-0.165	0.30105	3	2	0	0
2538	Isoform 1 of Putative ATP-dependent RNA helicase DHX57	IPI00168885	-0.165	0.30105	3	0	0	0
2539	Argininosuccinate synthase	IPI00020632	-0.165	0.30105	3	0	0	0
2540	Pleckstrin-2	IPI00009302	-0.165	0.30105	3	0	0	0
2541	Cell cycle regulator Mat89Bb homolog	IPI00550986	-0.165	0.30105	3	0	0	0
2542	Endoribonuclease Dicer	IPI00219036	-0.165	0.30105	3	0	0	0
2543	CDKN2A-interacting protein	IPI00020991	-0.165	0.30105	3	0	0	0
2544	U6 snRNA-associated Sm-like protein LSm1	IPI00004436	-0.165	0.30105	3	0	0	0
2545	Isoform 1 of Myosin-XVIIIa	IPI00760846	-0.165	0.30105	0	3	0	0
2546	HLA class I histocompatibility antigen, A-1 alpha chain	IPI00026569	-0.165	0.30105	0	3	0	0
2547	Pre-mRNA-splicing factor SYF2	IPI00022963	-0.165	0.30105	0	3	0	0
2548	Isoform 2 of Rho guanine nucleotide exchange factor 1	IPI00339379	-0.165	0.30105	3	0	0	0
2549	HSR1 protein	IPI00384745	-0.165	0.30105	3	0	2	0
2550	V-type proton ATPase subunit d 1	IPI00034159	-0.165	0.30105	3	2	2	0

No.	Description	Accession number	STN[1]	p-Value[1]	Con_A[2]	Con_B[2]	5FU_A[2]	5FU_B[2]
2551	S-adenosyl-L-methionine-dependent methyltransferase FTSJD2	IPI00166153	-0.165	0.30105	3	0	2	0
2552	Y-box-binding protein 2	IPI00250153	-0.165	0.30105	3	2	0	0
2553	Uncharacterized protein C21orf59	IPI00025710	-0.165	0.30105	3	2	0	0
2554	Syntaxin-12	IPI00329332	-0.165	0.30105	3	0	0	0
2555	Isoform 1 of Phosphoenolpyruvate carboxykinase [GTP], mitochondrial	IPI00797038	-0.165	0.30105	3	2	0	0
2556	Isoform 2 of mRNA cap guanine-N7 methyltransferase	IPI00410657	-0.165	0.30105	2	3	0	0
2557	Uridine diphosphate glucose pyrophosphatase	IPI00412878	-0.165	0.30105	2	3	0	0
2558	Zinc finger protein 622	IPI00056499	-0.165	0.30105	0	3	0	0
2559	Protein SEC13 homolog	IPI00375370	-0.165	0.30105	2	3	0	0
2560	U3 small nucleolar RNA-associated protein 6 homolog	IPI00020128	-0.165	0.30105	3	2	0	0
2561	Isoform I of Septin-6	IPI00216139	-0.165	0.30105	2	3	0	0
2562	Isoform 1 of Golgin subfamily A member 3	IPI00305267	-0.165	0.30105	3	2	0	2
2563	Methionyl-tRNA synthetase, mitochondrial	IPI00062839	-0.165	0.30105	3	0	2	0
2564	Protein SGT1	IPI00027034	-0.165	0.30105	3	0	0	0
2565	Isoform 1 of THO complex subunit 1	IPI00305374	-0.165	0.30105	3	0	0	0
2566	Aldehyde dehydrogenase, dimeric NADP-preferring	IPI00296183	-0.165	0.30105	2	3	0	2
2567	Isoform 1a of Oxysterol-binding protein-related protein 3	IPI00023555	-0.165	0.30105	3	0	0	2
2568	Isoform 1 of Cytosolic non-specific dipeptidase	IPI00177728	-0.165	0.30105	3	0	0	0
2569	Putative uncharacterized protein PYCRL	IPI00604402	-0.165	0.30105	0	3	0	0
2570	Isoform 1 of Uncharacterized protein CXorf38	IPI00152089	-0.165	0.30105	3	2	0	2
2571	Deoxynucleotidyltransferase terminal-interacting protein 2	IPI00290410	-0.165	0.30105	3	2	0	0
2572	Isoform 2 of Late secretory pathway protein AVL9 homolog	IPI00022042	-0.165	0.30105	2	3	0	0
2573	Isoform 2 of Integrator complex subunit 7	IPI00645022	-0.165	0.30105	3	0	0	0
2574	Isoform 1 of Multiple myeloma tumor-associated protein 2	IPI00293746	-0.165	0.30105	3	0	0	0
2575	Isoform 1 of Cytoskeleton-associated protein 4	IPI00141318	-0.165	0.30105	3	0	2	2
2576	Isoform 1 of Epsin-1	IPI00002495	-0.165	0.30105	3	0	0	2
2577	DNA polymerase beta	IPI00219538	-0.165	0.30105	2	3	0	0
2578	Isoform 2 of Insulin-like growth factor 2 mRNA-binding protein 3	IPI00165467	-0.165	0.30105	3	2	2	0
2579	Isoform Beta of DNA ligase 3	IPI00000156	-0.165	0.30105	3	2	0	0
2580	39S ribosomal protein L32, mitochondrial precursor	IPI00011077	-0.165	0.30105	3	2	0	0
2581	THO complex subunit 3	IPI00063729	-0.165	0.30105	3	0	0	0
2582	sorting nexin-6 isoform a	IPI00258833	-0.165	0.30105	3	0	0	0
2583	Digestive organ expansion factor homolog	IPI00004290	-0.165	0.30105	3	0	0	0
2584	71 kDa protein	IPI00062599	-0.165	0.30105	0	3	0	0
2585	WD repeat-containing protein 46	IPI00023126	-0.165	0.30105	3	0	0	0
2586	Isoform 1 of Tropomyosin beta chain	IPI00013991	-0.165	0.30105	0	3	0	0
2587	Coilin	IPI00006442	-0.165	0.30105	0	3	0	0
2588	SNF2 histone linker PHD RING helicase, isoform CRA_a	IPI00470627	-0.165	0.30105	0	3	0	0
2589	Isoform 6 of Ribosome-recycling factor, mitochondrial	IPI00030596	-0.165	0.30105	2	3	0	0
2590	Exocyst complex component 2	IPI00783559	-0.165	0.30105	3	0	0	0
2591	Isoform 1 of Surfeit locus protein 1	IPI00018034	-0.165	0.30105	3	0	0	0
2592	splicing factor 3B subunit 2	IPI00221106	-0.169	0.28951	19	17	17	17
2593	Isoform GTBP-alt of DNA mismatch repair protein Msh6	IPI00106847	-0.169	0.28951	20	16	16	18
2594	Isoform 1 of Nucleoside diphosphate kinase B	IPI00026260	-0.174	0.28817	19	14	14	17
2595	Proteasome subunit alpha type-6	IPI00029623	-0.174	0.28817	16	17	16	15
2596	Proteasome subunit beta type-2	IPI00028006	-0.177	0.28724	16	15	14	15
2597	DNA mismatch repair protein Msh2	IPI00017303	-0.181	0.28627	23	6	14	13
2598	Isoform 1 of Heterogeneous nuclear ribonucleoprotein K	IPI00216049	-0.182	0.28627	68	29	49	45
2599	Isoform 5 of Glycogen debranching enzyme	IPI00219065	-0.184	0.28627	15	13	12	14
2600	Putative uncharacterized protein NAP1L4	IPI00017763	-0.191	0.28392	16	9	12	11
2601	Isoform 1 of Mitotic checkpoint protein BUB3	IPI00013468	-0.191	0.28392	16	9	12	11
2602	Poly(rC)-binding protein 1	IPI00016610	-0.194	0.28381	14	10	11	11
2603	ubiquitin and ribosomal protein S27a precursor	IPI00179330	-0.195	0.28213	41	38	38	38
2604	cDNA FLJ59758, highly similar to S-methyl-5-thioadenosine phosphorylase	IPI00011876	-0.196	0.28210	11	12	11	10
2605	Protein NipSnap homolog 2	IPI00016077	-0.196	0.28210	11	12	13	8
2606	Isoform 1 of 60S ribosomal protein L12	IPI00024933	-0.199	0.28199	10	12	10	10
2607	26S proteasome non-ATPase regulatory subunit 13 isoform 2	IPI00375380	-0.199	0.28199	13	9	11	9
2608	Putative uncharacterized protein PSME2	IPI00384051	-0.199	0.28199	14	8	12	8
2609	Elongator complex protein 1	IPI00293735	-0.199	0.28199	10	12	11	9
2610	Serpin H1	IPI00032140	-0.199	0.28199	12	10	13	7
2611	Alanyl-tRNA synthetase, cytoplasmic	IPI00027442	-0.200	0.27983	47	27	39	32
2612	26S proteasome non-ATPase regulatory subunit 14	IPI00024821	-0.203	0.27971	14	7	10	9
2613	cDNA FLJ60076, highly similar to ELAV-like protein 1	IPI00301936	-0.203	0.27971	12	9	10	9
2614	Actin-related protein 3	IPI00028091	-0.203	0.27971	11	10	11	8
2615	Isoform Beta of Lamina-associated polypeptide 2, isoforms beta/gamma	IPI00030131	-0.210	0.27692	34	30	31	30
2616	Isoform 2 of Ubiquitin conjugation factor E4 A	IPI00028957	-0.210	0.27692	10	9	7	10
2617	Isoform 1 of Large proline-rich protein BAT3	IPI00465128	-0.210	0.27692	12	7	11	6
2618	Chromobox protein homolog 1	IPI00010320	-0.214	0.27662	10	8	8	8
2619	Isoform 1 of Growth factor receptor-bound protein 2	IPI00021327	-0.214	0.27662	9	9	9	7
2620	Prolyl endopeptidase	IPI00008164	-0.214	0.27662	12	6	9	7
2621	Isoform Short of RNA-binding protein FUS	IPI00221354	-0.214	0.27662	10	8	7	9
2622	DNA-(apurinic or apyrimidinic site) lyase	IPI00215911	-0.218	0.27327	11	6	7	8
2623	Protein MEMO1	IPI00032426	-0.218	0.27327	11	6	7	8
2624	Isoform 2 of DNA replication licensing factor MCM7	IPI00219740	-0.218	0.27327	11	6	7	8
2625	cDNA FLJ56425, highly similar to Very-long-chain specific acyl-CoAdehydrogenase, mitochondrial	IPI00028031	-0.222	0.27324	32	22	25	26
2626	Histone-binding protein RBBP7	IPI00395865	-0.223	0.27324	8	8	6	8
2627	Isoform 1 of Telomere-associated protein RIF1	IPI00293845	-0.223	0.27324	11	5	8	6
2628	Isoform 1 of RNA-binding protein 25	IPI00004273	-0.223	0.27324	8	8	7	7
2629	Reticulocalbin-1	IPI00015842	-0.223	0.27324	7	9	6	8
2630	Gamma-glutamyl hydrolase	IPI00023728	-0.228	0.26772	23	27	25	22
2631	Cytochrome c	IPI00465315	-0.228	0.26750	9	6	6	7
2632	Isoform 1 of ATP-binding cassette sub-family B member 7, mitochondrial	IPI00306748	-0.228	0.26750	6	9	5	8
2633	TRIP12 protein	IPI00032342	-0.228	0.26750	7	8	5	8
2634	Replication protein A 70 kDa DNA-binding subunit	IPI00020127	-0.228	0.26750	10	5	5	8
2635	cDNA FLJ55382, highly similar to Hsp70-binding protein 1	IPI00100748	-0.228	0.26750	9	6	7	6
2636	Isoform 1 of Low molecular weight phosphotyrosine protein phosphatase	IPI00219861	-0.228	0.26750	7	8	7	6
2637	Isoform 2 of Microtubule-associated protein 4	IPI00220113	-0.233	0.26739	6	8	6	6
2638	septin-9 isoform e	IPI00455033	-0.233	0.26739	8	6	6	6
2639	Dihydroorotate dehydrogenase, mitochondrial	IPI00024462	-0.233	0.26739	7	7	8	4
2640	Isoform 2 of Septin-11	IPI00019376	-0.233	0.26739	6	8	6	6
2641	Cold-inducible RNA-binding protein	IPI00180954	-0.233	0.26739	9	5	6	6
2642	Isoform 1 of 3'(2'),5'-bisphosphate nucleotidase 1	IPI00410214	-0.233	0.26739	10	4	7	5
2643	COP9 signalosome complex subunit 4	IPI00171844	-0.233	0.26739	9	5	6	6
2644	Putative heat shock protein HSP 90-alpha A2	IPI00031523	-0.235	0.26128	70	38	44	60
2645	Laminin receptor-like protein LAMRL5	IPI00411639	-0.236	0.26128	26	19	26	16

No.	Description	Accession number	STN[1]	p-Value[1]	Con_A[2]	Con_B[2]	5FU_A[2]	5FU_B[2]
2646	Heat shock protein 75 kDa, mitochondrial	IPI00030275	-0.236	0.26128	28	17	18	24
2647	SDHA protein	IPI00217143	-0.239	0.26098	7	6	5	6
2648	Isoform 1 of RNA-binding protein 8A	IPI00001757	-0.239	0.26098	7	6	6	5
2649	Isoform 1 of Protein canopy homolog 2	IPI00443909	-0.239	0.26098	6	7	5	6
2650	PNAS-139	IPI00000477	-0.239	0.26098	8	5	4	7
2651	Phosducin-like protein 3	IPI00031629	-0.239	0.26098	6	7	6	5
2652	40S ribosomal protein S21	IPI00017448	-0.239	0.26098	8	5	4	7
2653	cDNA FLJ61739, highly similar to Serine/arginine repetitive matrix protein 1	IPI00328293	-0.239	0.26098	6	7	6	5
2654	Isoform 1 of Ubiquitin-conjugating enzyme E2 K	IPI00021370	-0.239	0.26098	5	8	5	6
2655	Calponin-3	IPI00216682	-0.239	0.26098	7	6	6	5
2656	DNA polymerase alpha catalytic subunit	IPI00220317	-0.246	0.26087	6	6	5	5
2657	Oxysterol-binding protein	IPI00163644	-0.246	0.26087	7	5	5	5
2658	Tubulin beta-3 chain	IPI00013683	-0.246	0.26087	8	4	6	4
2659	Isoform 1 of Dynamin-2	IPI00033022	-0.246	0.26087	9	3	6	4
2660	24 kDa protein	IPI00398057	-0.246	0.26087	6	6	6	4
2661	Isoform 2 of Histone deacetylase 2	IPI00289601	-0.246	0.26087	7	5	5	5
2662	Signal recognition particle receptor subunit alpha	IPI00385267	-0.246	0.26087	8	4	5	5
2663	sorting nexin-1 isoform c	IPI00183274	-0.246	0.26087	6	6	3	7
2664	Eukaryotic translation initiation factor 4A, isoform 2, isoform CRA_b	IPI00030296	-0.246	0.26087	5	7	4	6
2665	Peptidyl-prolyl cis-trans isomerase A	IPI00419585	-0.251	0.25268	46	43	41	44
2666	Isoform 1 of Protein virilizer homolog	IPI00036742	-0.254	0.25253	7	4	3	6
2667	Signal peptidase complex catalytic subunit SEC11A	IPI00104128	-0.254	0.25253	6	5	5	4
2668	Isoform Short of Ubiquitin fusion degradation protein 1 homolog	IPI00218292	-0.254	0.25253	6	5	5	4
2669	NEDD8-activating enzyme E1 regulatory subunit	IPI00018968	-0.254	0.25253	5	6	5	4
2670	28S ribosomal protein S28, mitochondrial	IPI00022276	-0.254	0.25253	6	5	6	3
2671	Isoform 2 of PERQ amino acid-rich with GYF domain-containing protein 2	IPI00647635	-0.254	0.25253	7	4	4	5
2672	RuvB-like 2	IPI00009104	-0.254	0.25253	19	17	19	14
2673	Isoform Long of Glucose-6-phosphate 1-dehydrogenase	IPI00216008	-0.257	0.25182	20	15	17	15
2674	Peptidyl-prolyl cis-trans isomerase FKBP3	IPI00024157	-0.257	0.25182	17	18	15	17
2675	Isoform 1 of Rab3 GTPase-activating protein non-catalytic subunit	IPI00554590	-0.263	0.25115	5	5	5	3
2676	Thioredoxin-related transmembrane protein 1	IPI00395887	-0.263	0.25115	6	4	5	3
2677	RNA polymerase-associated protein CTR9 homolog	IPI00477468	-0.263	0.25115	6	4	5	3
2678	Probable rRNA-processing protein EBP2	IPI00745955	-0.263	0.25115	5	5	3	5
2679	cDNA FLJ56153, highly similar to Homo sapiens transforming growth factor beta regulator 4 (TBRG4)	IPI00329625	-0.263	0.25115	6	4	3	5
2680	Protein S100-A11	IPI00013895	-0.263	0.25115	3	7	0	6
2681	Isoform 1 of Neurochondrin	IPI00549543	-0.263	0.25115	5	5	3	5
2682	Isoform 1 of PDZ domain-containing protein 11	IPI00550841	-0.263	0.25115	5	5	3	5
2683	Isoform 1 of Ras-related protein Rab-6A	IPI00023526	-0.263	0.25115	5	5	4	4
2684	cDNA FLJ56180, highly similar to Negative elongation factor E	IPI00000858	-0.263	0.25115	6	4	4	4
2685	N(G),N(G)-dimethylarginine dimethylaminohydrolase 2	IPI00000760	-0.263	0.25115	5	5	4	4
2686	Isoform Long of Delta-1-pyrroline-5-carboxylate synthase	IPI00008982	-0.265	0.24024	18	14	12	17
2687	Isoform 1 of Carnitine O-palmitoyltransferase 1, liver isoform	IPI00032038	-0.271	0.23980	15	15	14	13
2688	NADH dehydrogenase [ubiquinone] 1 beta subcomplex subunit 4	IPI00220059	-0.273	0.23890	5	4	4	3
2689	Asparagine synthetase [glutamine-hydrolyzing]	IPI00554777	-0.273	0.23890	4	5	3	4
2690	Diphosphoinositol polyphosphate phosphohydrolase 1	IPI00009148	-0.273	0.23890	4	5	3	4
2691	Isoform Long of Tyrosine-protein kinase SYK	IPI00018597	-0.273	0.23890	5	4	4	3
2692	39S ribosomal protein L37, mitochondrial	IPI00162330	-0.273	0.23890	6	3	4	3
2693	Gem-associated protein 5	IPI00291783	-0.273	0.23890	6	3	3	4
2694	Isoform 2 of Mitochondrial import inner membrane translocase subunit TIM50	IPI00418497	-0.273	0.23890	4	5	4	3
2695	SWI/SNF complex subunit SMARCC1	IPI00234252	-0.273	0.23890	7	2	2	5
2696	Scavenger mRNA-decapping enzyme DcpS	IPI00335385	-0.273	0.23890	7	0	4	3
2697	CLASP2 protein	IPI00168165	-0.273	0.23890	4	5	3	4
2698	Isoform Heart of ATP synthase subunit gamma, mitochondrial	IPI00395769	-0.273	0.23890	4	5	4	3
2699	Isoform 3 of Chitinase domain-containing protein 1	IPI00045536	-0.273	0.23890	6	3	3	4
2700	Isoform B of AP-2 complex subunit alpha-1	IPI00256684	-0.273	0.23890	6	3	3	4
2701	Rho GDP-dissociation inhibitor 2	IPI00003817	-0.273	0.23890	4	5	5	2
2702	DnaJ homolog subfamily C member 7	IPI00329629	-0.273	0.23890	5	4	3	4
2703	Isoform 1 of Protein POF1B	IPI00103242	-0.273	0.23890	6	3	4	3
2704	Isoform 3 of Protein PRRC1	IPI00217053	-0.273	0.23890	6	3	5	2
2705	60S ribosomal protein L23	IPI00010153	-0.274	0.23890	16	13	12	14
2706	Endoplasmic reticulum metallopeptidase 1	IPI00257903	-0.281	0.23816	15	12	12	12
2707	U1 small nuclear ribonucleoprotein A	IPI00012382	-0.281	0.23816	14	13	11	13
2708	Actin-related protein 2/3 complex subunit 3	IPI00005162	-0.286	0.23704	4	4	4	0
2709	Tubulin gamma-1 chain	IPI00295081	-0.286	0.23704	6	2	4	2
2710	DEAH (Asp-Glu-Ala-His) box polypeptide 16	IPI00292510	-0.286	0.23704	4	4	2	4
2711	Isoform 1 of Protein-tyrosine phosphatase mitochondrial 1	IPI00174190	-0.286	0.23704	4	4	3	3
2712	Coiled-coil domain-containing protein 25	IPI00396174	-0.286	0.23704	3	5	2	4
2713	Isoform 1 of Elongation factor Tu GTP-binding domain-containing protein 1	IPI00293026	-0.286	0.23704	4	4	2	4
2714	V-type proton ATPase subunit C 1	IPI00007814	-0.286	0.23704	6	0	4	2
2715	Transmembrane protein 2	IPI00170706	-0.286	0.23704	5	3	3	3
2716	Aspartyl-tRNA synthetase, mitochondrial	IPI00100460	-0.286	0.23704	5	3	2	4
2717	39S ribosomal protein L23, mitochondrial	IPI00293476	-0.286	0.23704	5	3	3	3
2718	Astrocytic phosphoprotein PEA-15	IPI00014850	-0.286	0.23704	5	3	3	3
2719	Protein TFG	IPI00294619	-0.286	0.23704	5	3	4	2
2720	114 kDa protein	IPI00166555	-0.286	0.23704	3	5	4	0
2721	D-beta-hydroxybutyrate dehydrogenase, mitochondrial	IPI00025341	-0.286	0.23704	4	4	3	3
2722	Isoform 1 of Thyroid receptor-interacting protein 13	IPI00003505	-0.286	0.23704	3	5	2	4
2723	Density-regulated protein	IPI00306280	-0.286	0.23704	4	4	3	3
2724	28S ribosomal protein S31, mitochondrial	IPI00294242	-0.286	0.23704	4	4	2	4
2725	Isoform 1 of CD109 antigen	IPI00152540	-0.286	0.23704	5	3	3	3
2726	GDP-L-fucose synthase	IPI00014361	-0.286	0.23704	3	5	4	2
2727	2,4-dienoyl-CoA reductase, mitochondrial	IPI00003482	-0.286	0.23704	3	5	2	4
2728	Isoform 2 of Transcription elongation factor A protein 1	IPI00218106	-0.286	0.23704	4	4	3	3
2729	Transmembrane 9 superfamily member 4	IPI00021985	-0.286	0.23704	6	2	4	2
2730	Peptidyl-prolyl cis-trans isomerase F, mitochondrial	IPI00026519	-0.286	0.23704	4	4	3	3
2731	Isoform 1 of 2',5'-phosphodiesterase 12	IPI00174390	-0.286	0.23704	5	3	4	2
2732	Isoform 3 of THO complex subunit 6 homolog	IPI00301252	-0.286	0.23704	5	3	3	3
2733	Isoform 1 of Pentatricopeptide repeat-containing protein 3, mitochondrial	IPI00783302	-0.286	0.23704	6	2	2	4
2734	Isoform 2 of Valacyclovir hydrolase	IPI00003990	-0.286	0.23704	4	4	4	2
2735	Glutamate--cysteine ligase regulatory subunit	IPI00010090	-0.286	0.23704	5	3	3	3
2736	Leucyl-tRNA synthetase, cytoplasmic	IPI00103994	-0.286	0.21720	37	23	28	28
2737	transcription activator BRG1 isoform D	IPI00029822	-0.292	0.21679	12	12	10	11
2738	Isoform Epsilon of Apoptosis regulator BAX	IPI00071059	-0.292	0.21679	12	12	13	8
2739	Ribosome production factor 2 homolog	IPI00396329	-0.301	0.21571	4	3	3	0

No.	Description	Accession number	STN[1]	p-Value[1]	Con_A[2]	Con_B[2]	5FU_A[2]	5FU_B[2]
2740	Isoform 2 of Putative methyltransferase NSUN5	IPI00101659	-0.301	0.21571	3	4	3	2
2741	Isoform 2 of Isopentenyl-diphosphate Delta-isomerase 1	IPI00220014	-0.301	0.21571	5	2	0	3
2742	Serine/threonine-protein kinase VRK1	IPI00019640	-0.301	0.21571	4	3	3	2
2743	Uncharacterized protein C19orf21	IPI00217121	-0.301	0.21571	3	4	2	3
2744	Isoform 2 of Succinyl-CoA ligase [ADP-forming] subunit beta, mitochondrial	IPI00217232	-0.301	0.21571	5	2	3	2
2745	V-type proton ATPase subunit F	IPI00004488	-0.301	0.21571	4	3	3	2
2746	FAST kinase domain-containing protein 5	IPI00414973	-0.301	0.21571	3	4	2	3
2747	Peptidyl-prolyl cis-trans isomerase FKBP2	IPI00002535	-0.301	0.21571	5	2	0	3
2748	UV excision repair protein RAD23 homolog B	IPI00008223	-0.301	0.21571	3	4	3	2
2749	Coproporphyrinogen-III oxidase, mitochondrial	IPI00093057	-0.301	0.21571	5	2	3	0
2750	Isoform 1 of Protein phosphatase 1 regulatory subunit 7	IPI00033600	-0.301	0.21571	5	0	2	3
2751	Isoform 1 of Aldehyde dehydrogenase family 16 member A1	IPI00217920	-0.301	0.21571	4	3	2	3
2752	Isoform 3 of Epithelial splicing regulatory protein 1	IPI00184262	-0.301	0.21571	5	2	2	3
2753	WD repeat-containing protein 82	IPI00152695	-0.301	0.21571	4	3	3	2
2754	Isoform 1 of Rho guanine nucleotide exchange factor 2	IPI00291316	-0.301	0.21571	5	2	3	0
2755	54 kDa protein	IPI00177890	-0.301	0.21571	2	5	2	3
2756	dynactin subunit 2	IPI00220503	-0.301	0.21571	4	3	3	2
2757	Isoform 2 of DNA-3-methyladenine glycosylase	IPI00218495	-0.301	0.21571	3	4	2	3
2758	Isoform 1 of Acylglycerol kinase, mitochondrial	IPI00019353	-0.301	0.21571	5	2	3	2
2759	DCN1-like protein 1	IPI00291893	-0.301	0.21571	4	3	3	2
2760	Isoform 1 of RNA polymerase II-associated protein 3	IPI00002408	-0.301	0.21571	3	4	3	2
2761	lanosterol 14-alpha demethylase isoform 1	IPI00295772	-0.301	0.21571	5	0	2	3
2762	Methylosome subunit pICln	IPI00004795	-0.301	0.21571	4	3	3	0
2763	Charged multivesicular body protein 4b	IPI00025974	-0.301	0.21571	3	4	0	3
2764	Vacuolar protein sorting-associated protein VTA1 homolog	IPI00017160	-0.301	0.21571	4	3	3	2
2765	Guanine deaminase	IPI00644409	-0.301	0.21571	5	0	2	3
2766	Synaptosomal-associated protein 29	IPI00032831	-0.301	0.21571	4	3	3	0
2767	Isoform Non-muscle of Myosin light polypeptide 6	IPI00335168	-0.301	0.21571	13	9	10	9
2768	Calcium-binding mitochondrial carrier protein Aralar2	IPI00007084	-0.306	0.21474	14	7	9	9
2769	40S ribosomal protein S15a	IPI00221091	-0.312	0.21433	12	8	10	7
2770	Calpain small subunit 1	IPI00025084	-0.312	0.21433	11	9	11	6
2771	Mitochondrial import receptor subunit TOM34	IPI00009946	-0.312	0.21433	11	9	9	8
2772	V-type proton ATPase catalytic subunit A	IPI00007682	-0.312	0.21433	12	8	7	10
2773	Isoform 2 of 4F2 cell-surface antigen heavy chain	IPI00027493	-0.318	0.21291	13	6	7	9
2774	Sorbitol dehydrogenase	IPI00216057	-0.318	0.21291	9	10	8	8
2775	Isoform 2 of Titin	IPI00023283	-0.320	0.21209	0	4	0	0
2776	TATA-binding protein-associated factor 172	IPI00024802	-0.320	0.21209	4	2	0	2
2777	39S ribosomal protein L45, mitochondrial	IPI00185859	-0.320	0.21209	4	2	0	2
2778	Isoform 1 of Pescadillo homolog	IPI00003768	-0.320	0.21209	4	2	0	0
2779	Uncharacterized protein KIAA0406	IPI00011702	-0.320	0.21209	4	0	2	2
2780	programmed cell death 4 isoform 2	IPI00240675	-0.320	0.21209	2	4	2	2
2781	Visinin-like protein 1	IPI00216313	-0.320	0.21209	3	3	2	0
2782	GTP-binding protein Rheb	IPI00016669	-0.320	0.21209	4	2	0	0
2783	RER1 protein	IPI00005728	-0.320	0.21209	3	3	2	2
2784	Isoform SRP40-1 of Splicing factor, arginine/serine-rich 5	IPI00012341	-0.320	0.21209	4	2	2	0
2785	Exportin-T	IPI00306290	-0.320	0.21209	4	2	2	0
2786	Isoform 1 of DNA primase large subunit	IPI00027705	-0.320	0.21209	4	0	0	0
2787	Interferon-induced, double-stranded RNA-activated protein kinase	IPI00019463	-0.320	0.21209	4	2	0	0
2788	Isoform 1 of Exosome component 10	IPI00009464	-0.320	0.21209	2	4	2	0
2789	Nucleolar complex protein 3 homolog	IPI00102815	-0.320	0.21209	4	0	0	0
2790	Guanine nucleotide-binding protein G(I)/G(S)/G(T) subunit beta-2	IPI00003348	-0.320	0.21209	2	4	2	2
2791	Isoform A of Uncharacterized protein C21orf70	IPI00027898	-0.320	0.21209	0	4	2	2
2792	RNA-binding protein 28	IPI00304187	-0.320	0.21209	4	0	2	2
2793	Succinyl-CoA ligase [GDP-forming] subunit alpha, mitochondrial	IPI00872762	-0.320	0.21209	3	3	0	0
2794	cytochrome c oxidase subunit VIIa polypeptide 2 (liver) precursor	IPI00026570	-0.320	0.21209	2	4	2	2
2795	Isovaleryl-CoA dehydrogenase, mitochondrial	IPI00645805	-0.320	0.21209	4	2	0	2
2796	CCR4-NOT transcription complex subunit 7	IPI00006552	-0.320	0.21209	3	3	2	2
2797	Isoform 1 of GTP-binding protein 10	IPI00167638	-0.320	0.21209	4	2	2	2
2798	Transcription initiation factor TFIID subunit 2	IPI00328144	-0.320	0.21209	0	4	0	0
2799	Isoform 1 of Putative RNA-binding protein 15	IPI00102752	-0.320	0.21209	4	2	0	2
2800	G-rich sequence factor 1	IPI00478657	-0.320	0.21209	4	0	0	0
2801	Exportin-6	IPI00465296	-0.320	0.21209	3	3	2	0
2802	Isoform 1 of Creatine kinase U-type, mitochondrial	IPI00658109	-0.320	0.21209	4	0	2	2
2803	Protein FAM49A	IPI00006574	-0.320	0.21209	4	2	0	0
2804	Basic leucine zipper and W2 domain-containing protein 2	IPI00022305	-0.320	0.21209	4	0	0	0
2805	Isoform 2 of Glutaminase kidney isoform, mitochondrial	IPI00215685	-0.320	0.21209	4	0	2	0
2806	Putative uncharacterized protein C3orf75	IPI00107155	-0.320	0.21209	2	4	0	2
2807	C-Myc-binding protein	IPI00871174	-0.320	0.21209	0	4	0	0
2808	AP-3 complex subunit mu-1	IPI00032459	-0.320	0.21209	4	0	0	0
2809	Dihydroxyacetone phosphate acyltransferase	IPI00005677	-0.320	0.21209	4	2	0	2
2810	erlin-1	IPI00007940	-0.320	0.21209	4	0	2	2
2811	Ribonuclease inhibitor	IPI00550069	-0.320	0.21209	4	2	0	2
2812	Isoform 1 of Cullin-3	IPI00014312	-0.320	0.21209	3	3	2	2
2813	Cytokine-like nuclear factor n-pac, isoform CRA_a	IPI00000155	-0.320	0.21209	3	3	0	2
2814	Isoform 2 of Nucleolar protein 10	IPI00016494	-0.320	0.21209	4	0	2	0
2815	Leucine-rich repeat-containing protein 40	IPI00152998	-0.320	0.21209	3	3	0	0
2816	Exosome complex exonuclease RRP42	IPI00014198	-0.320	0.21209	3	3	2	2
2817	Isoform 1 of Autophagy-related protein 3	IPI00022254	-0.320	0.21209	2	4	0	2
2818	Isoform 1 of Mannose-6-phosphate isomerase	IPI00219358	-0.320	0.21209	3	3	2	2
2819	Neuronal protein	IPI00472058	-0.320	0.21209	3	3	0	0
2820	Autophagy-related protein 101	IPI00305296	-0.320	0.21209	3	3	0	0
2821	Pumilio domain-containing protein KIAA0020	IPI00791325	-0.320	0.21209	3	3	0	0
2822	Transcription factor BTF3 homolog 4	IPI00412792	-0.320	0.21209	0	4	0	0
2823	Isoform 2 of WASH complex subunit FAM21C	IPI00456853	-0.320	0.21209	3	3	0	0
2824	Isoform 1 of STIP1 homology and U box-containing protein 1	IPI00025156	-0.320	0.21209	3	3	2	2
2825	HDCMD34P	IPI00001672	-0.320	0.21209	4	0	2	0
2826	Aldehyde dehydrogenase family 1 member A3	IPI00026663	-0.320	0.21209	4	2	2	0
2827	Isoform 2 of Probable phospholipid-transporting ATPase IG	IPI00237446	-0.320	0.21209	0	4	0	0
2828	Glycerol-3-phosphate dehydrogenase 1-like protein	IPI00032959	-0.320	0.21209	3	3	0	0
2829	Vacuolar protein sorting-associated protein 28 homolog	IPI00007155	-0.320	0.21209	3	3	2	0
2830	Squalene synthase	IPI00020944	-0.320	0.21209	4	2	0	0
2831	Isoform 2 of Transcription factor p65	IPI00219084	-0.320	0.21209	3	3	0	2
2832	ATP-dependent RNA helicase DDX50	IPI00031554	-0.320	0.21209	4	2	2	2
2833	Ras-related protein Rab-24	IPI00056496	-0.320	0.21209	4	2	0	2
2834	Serine palmitoyltransferase 1	IPI00005745	-0.320	0.21209	4	2	0	0

No.	Description	Accession number	STN[1]	p-Value[1]	Con_A[2]	Con_B[2]	5FU_A[2]	5FU_B[2]
2835	Isoform 1 of Craniofacial development protein 1	IPI00007306	-0.320	0.21209	4	0	0	0
2836	JmjC domain-containing protein 7	IPI00382394	-0.320	0.21209	4	2	0	0
2837	NudC domain-containing protein 3	IPI00238209	-0.320	0.21209	4	0	0	0
2838	BRISC complex subunit Abro1	IPI00299517	-0.320	0.21209	3	3	0	2
2839	Zinc finger protein ubi-d4	IPI00023322	-0.320	0.21209	4	0	2	0
2840	Centromere protein H	IPI00009668	-0.320	0.21209	3	3	0	0
2841	Glutamine-dependent NAD(+) synthetase	IPI00306689	-0.320	0.21209	3	3	0	0
2842	Isoform 1 of Chaperone activity of bc1 complex-like, mitochondrial	IPI00176469	-0.320	0.21209	4	0	2	0
2843	Methylenetetrahydrofolate dehydrogenase (NADP+ dependent) 1-like	IPI00291646	-0.324	0.18860	10	8	7	8
2844	Isoform 1 of Ras-related protein Rab-1A	IPI00005719	-0.326	0.18692	42	38	39	36
2845	Glutathione S-transferase P	IPI00219757	-0.327	0.18692	74	63	73	58
2846	Isoform 1 of ATPase family AAA domain-containing protein 2	IPI00170548	-0.330	0.18655	8	9	7	7
2847	UMP-CMP kinase isoform a	IPI00219953	-0.330	0.18655	9	8	8	6
2848	U6 snRNA-associated Sm-like protein LSm2	IPI00032460	-0.330	0.18655	10	7	6	8
2849	ATP-binding cassette sub-family E member 1	IPI00303207	-0.336	0.18566	41	32	34	34
2850	Coatomer subunit beta	IPI00295851	-0.338	0.18566	27	10	14	19
2851	40S ribosomal protein S13	IPI00221089	-0.338	0.18566	7	9	8	5
2852	DYNC1H1 protein	IPI00440177	-0.338	0.18566	5	11	5	8
2853	CSNK2A1 protein	IPI00016613	-0.338	0.18566	8	8	6	7
2854	Synaptic vesicle membrane protein VAT-1 homolog	IPI00156689	-0.338	0.18566	10	6	7	6
2855	cDNA FLJ36192 fis, clone TESTI2027450, highly similar to Eukaryotic translation initiation factor 3	IPI00654777	-0.338	0.18566	11	5	7	6
2856	Serine/threonine-protein kinase PRP4 homolog	IPI00013721	-0.338	0.18566	10	6	4	9
2857	Isoform E of Eukaryotic translation initiation factor 4 gamma 1	IPI00386533	-0.341	0.18353	16	20	15	17
2858	Proteasome subunit beta type-4	IPI00555956	-0.344	0.18294	18	17	17	14
2859	Isoform C1 of Heterogeneous nuclear ribonucleoproteins C1/C2	IPI00216592	-0.346	0.18294	48	19	30	32
2860	Isoform 1 of Vesicle-associated membrane protein-associated protein A	IPI00170692	-0.346	0.18294	8	7	6	6
2861	Serine/threonine-protein kinase PAK 2	IPI00419979	-0.346	0.18294	8	7	5	7
2862	Dolichol-phosphate mannosyltransferase	IPI00022018	-0.346	0.18294	7	8	7	5
2863	coatomer subunit epsilon isoform b	IPI00399318	-0.346	0.18294	9	6	7	5
2864	DNA ligase 1	IPI00219841	-0.346	0.18294	9	6	5	7
2865	Isoform 1 of Protein phosphatase methylesterase 1	IPI00007694	-0.346	0.18294	8	7	6	6
2866	Isoform 1 of Protein SET	IPI00072377	-0.351	0.18108	21	12	17	12
2867	tRNA (cytosine-5-)-methyltransferase NSUN2	IPI00306369	-0.355	0.18104	9	5	4	7
2868	Isoform 2 of Cat eye syndrome critical region protein 5	IPI00011511	-0.355	0.18104	7	7	8	3
2869	Putative uncharacterized protein ENSP00000350479	IPI00069693	-0.355	0.18104	7	7	5	6
2870	Ladinin-1	IPI00514234	-0.355	0.18104	7	7	6	5
2871	Glycyl-tRNA synthetase	IPI00783097	-0.359	0.17862	19	12	16	11
2872	NADH dehydrogenase [ubiquinone] iron-sulfur protein 2, mitochondrial	IPI00025239	-0.364	0.17773	6	7	5	5
2873	Transgelin	IPI00216138	-0.364	0.17773	7	6	5	5
2874	Myosin regulatory light chain 12B	IPI00033494	-0.364	0.17773	9	4	7	3
2875	SAP domain-containing ribonucleoprotein	IPI00014938	-0.364	0.17773	7	6	5	5
2876	39S ribosomal protein L9, mitochondrial	IPI00307409	-0.364	0.17773	6	7	4	6
2877	Ras-related protein Rab-21	IPI00007755	-0.364	0.17773	7	6	5	5
2878	Tu translation elongation factor, mitochondrial precursor	IPI00027107	-0.367	0.17672	17	12	14	11
2879	Periodic tryptophan protein 2 homolog	IPI00300078	-0.367	0.17672	15	14	12	13
2880	Lamin-B1	IPI00217975	-0.367	0.17672	17	12	11	14
2881	Isoform 1 of Protein KIAA1967	IPI00182757	-0.372	0.17653	16	12	12	12
2882	Isoform 1 of Heterogeneous nuclear ribonucleoprotein R	IPI00012074	-0.374	0.17590	33	20	21	27
2883	Isoform 1 of Malignant T cell-amplified sequence 1	IPI00179026	-0.376	0.17590	7	5	5	4
2884	FKBP1A protein	IPI00413778	-0.376	0.17590	10	0	5	4
2885	Rho GTPase-activating protein 1	IPI00020567	-0.376	0.17590	9	3	5	4
2886	Isoform 1 of Acyl-coenzyme A thioesterase 9, mitochondrial	IPI00220710	-0.376	0.17590	6	6	6	3
2887	Isoform 1 of Armadillo repeat-containing protein 10	IPI00166394	-0.376	0.17590	5	7	7	0
2888	Isoform 1 of Protein timeless homolog	IPI00335541	-0.376	0.17590	5	7	4	5
2889	DNA-directed RNA polymerases I, II, and III subunit RPABC1	IPI00291093	-0.376	0.17590	6	6	4	5
2890	Synaptotagmin-1	IPI00009439	-0.376	0.17590	6	6	3	6
2891	Nascent polypeptide-associated complex subunit alpha	IPI00023748	-0.377	0.17277	14	13	13	10
2892	Keratin, type I cytoskeletal 18	IPI00554788	-0.380	0.17277	208	202	198	202
2893	Isoform 5 of Interleukin enhancer-binding factor 3	IPI00219330	-0.385	0.17125	25	24	22	22
2894	NAD(P)H dehydrogenase [quinone] 1	IPI00012069	-0.385	0.17117	44	40	42	36
2895	Histone H2B type 2-E	IPI00003935	-0.387	0.17102	49	34	42	35
2896	Alkyldihydroxyacetonephosphate synthase, peroxisomal	IPI00010349	-0.388	0.17084	7	4	4	4
2897	Rho-related GTP-binding protein RhoG	IPI00017342	-0.388	0.17084	7	4	5	3
2898	Putative uncharacterized protein	IPI00260769	-0.388	0.17084	5	6	2	6
2899	Niban-like protein 1	IPI00456750	-0.388	0.17084	8	3	4	4
2900	Tubulin-folding cofactor B	IPI00293126	-0.388	0.17084	8	3	5	3
2901	Developmentally-regulated GTP-binding protein 1	IPI00031836	-0.388	0.17084	6	5	4	4
2902	Isoform Rpn10A of 26S proteasome non-ATPase regulatory subunit 4	IPI00022694	-0.388	0.17084	7	4	5	3
2903	NADH dehydrogenase [ubiquinone] iron-sulfur protein 7, mitochondrial	IPI00307749	-0.388	0.17084	6	5	4	4
2904	Putative high mobility group protein 1-like 10	IPI00018755	-0.393	0.17020	10	14	9	11
2905	Exportin-5	IPI00640703	-0.393	0.17020	15	9	9	11
2906	40S ribosomal protein S19	IPI00215780	-0.393	0.17020	14	10	9	11
2907	NADH dehydrogenase [ubiquinone] iron-sulfur protein 3, mitochondrial	IPI00025796	-0.399	0.16801	12	11	13	6
2908	cytochrome b5 type B precursor	IPI00303954	-0.399	0.16801	11	12	10	9
2909	Isoform 1 of 26S protease regulatory subunit 6B	IPI00020042	-0.403	0.16756	7	3	4	3
2910	Peptidyl-prolyl cis-trans isomerase FKBP5	IPI00218775	-0.403	0.16756	6	4	4	3
2911	Transmembrane emp24 domain-containing protein 9	IPI00023542	-0.403	0.16756	4	6	5	2
2912	Ribose-5-phosphate isomerase	IPI00026513	-0.403	0.16756	4	6	3	4
2913	Cystatin-B	IPI00021828	-0.403	0.16756	8	2	4	3
2914	Isoform 1 of Dynamin-like 120 kDa protein, mitochondrial	IPI00006721	-0.403	0.16756	4	6	3	4
2915	COP9 signalosome complex subunit 8	IPI00009480	-0.403	0.16756	6	4	3	4
2916	Protein C20orf11	IPI00016634	-0.403	0.16756	5	5	4	3
2917	Probable ATP-dependent RNA helicase DDX52	IPI00032423	-0.403	0.16756	6	4	3	4
2918	Alcohol dehydrogenase [NADP+]	IPI00220271	-0.405	0.16287	12	10	11	7
2919	Isoform 3 of Adenylate kinase 2, mitochondrial	IPI00172460	-0.406	0.16123	24	18	23	14
2920	Fatty acid synthase	IPI00026781	-0.418	0.16052	166	140	146	150
2921	Dipeptidyl peptidase 1	IPI00022810	-0.420	0.15725	4	5	4	2
2922	Transmembrane protein 43	IPI00301280	-0.420	0.15725	5	4	4	2
2923	39S ribosomal protein L1, mitochondrial	IPI00549381	-0.420	0.15725	5	4	4	2
2924	Serine/threonine-protein phosphatase PP1-beta catalytic subunit	IPI00218236	-0.420	0.15725	7	0	4	0
2925	Translation initiation factor eIF-2B subunit alpha	IPI00221300	-0.420	0.15725	5	4	3	3
2926	PDZ and LIM domain protein 5	IPI00007935	-0.420	0.15725	5	4	3	3
2927	Isoform 1 of Pre-mRNA-splicing factor 38A	IPI00171390	-0.420	0.15725	6	3	0	4
2928	ADP-ribosylation factor 5	IPI00215919	-0.420	0.15725	5	4	4	2

No.	Description	Accession number	STN[1]	p-Value[1]	Con_A[2]	Con_B[2]	5FU_A[2]	5FU_B[2]
2929	Aldo-keto reductase family 1 member C1	IPI00029733	-0.420	0.15725	5	4	2	4
2930	Isoform 1 of Ras GTPase-activating protein 1	IPI00026262	-0.420	0.15725	6	3	0	4
2931	Golgi resident protein GCP60	IPI00009315	-0.420	0.15725	5	4	3	3
2932	poly(rC) binding protein 2 isoform b	IPI00012066	-0.422	0.15591	35	29	28	30
2933	Isoform 1 of Cysteine and histidine-rich domain-containing protein 1	IPI00015897	-0.432	0.15483	16	19	16	14
2934	NADH dehydrogenase [ubiquinone] iron-sulfur protein 8, mitochondrial	IPI00010845	-0.436	0.15475	10	8	9	5
2935	Regulation of nuclear pre-mRNA domain-containing protein 1B	IPI00009659	-0.436	0.15475	10	8	8	6
2936	Isoform 1 of 39S ribosomal protein L22, mitochondrial	IPI00414410	-0.436	0.15475	10	8	7	7
2937	Omega-amidase NIT2	IPI00549467	-0.436	0.15475	9	9	6	8
2938	GTP-binding protein SAR1b	IPI00002149	-0.441	0.15177	4	4	0	3
2939	Retinoblastoma-associated protein	IPI00302829	-0.441	0.15177	0	6	3	2
2940	Cytochrome c1, heme protein, mitochondrial	IPI00029264	-0.441	0.15177	3	5	3	2
2941	ATP-dependent RNA helicase DHX8	IPI00031508	-0.441	0.15177	4	4	3	0
2942	Heat shock 70 kDa protein 4L	IPI00295485	-0.441	0.15177	6	2	3	2
2943	Cytochrome c-type heme lyase	IPI00023406	-0.441	0.15177	4	4	0	3
2944	BRO1 domain-containing protein BROX	IPI00065500	-0.441	0.15177	5	3	3	2
2945	Isoform 1 of Voltage-gated potassium channel subunit beta-2	IPI00021088	-0.441	0.15177	6	2	2	3
2946	Signal peptidase complex subunit 3	IPI00300299	-0.441	0.15177	0	6	3	0
2947	G patch domain and KOW motifs-containing protein	IPI00024255	-0.441	0.15177	6	2	3	2
2948	Centromere/kinetochore protein zw10 homolog	IPI00011631	-0.441	0.15177	3	5	3	2
2949	Vasodilator-stimulated phosphoprotein	IPI00301058	-0.441	0.15177	5	3	3	2
2950	Coiled-coil domain-containing protein 6	IPI00000634	-0.441	0.15177	4	4	3	2
2951	Isoform 1 of Syntenin-1	IPI00299086	-0.441	0.15177	5	3	3	0
2952	HSPA5 protein	IPI00003362	-0.441	0.15177	49	40	41	41
2953	Isoform 1 of 5'-3' exoribonuclease 2	IPI00100151	-0.441	0.15177	21	12	12	16
2954	Cathepsin D	IPI00011229	-0.441	0.15177	17	16	15	13
2955	Isoform 1 of Transcription intermediary factor 1-beta	IPI00438229	-0.445	0.15174	26	29	24	25
2956	60S ribosomal protein L30	IPI00219156	-0.446	0.15174	8	9	5	8
2957	DNA replication licensing factor MCM6	IPI00031517	-0.446	0.15174	12	5	6	7
2958	DEAD (Asp-Glu-Ala-Asp) box polypeptide 39, isoform CRA_c	IPI00166874	-0.446	0.15174	8	9	7	6
2959	UPF0368 protein Cxorf26	IPI00107104	-0.446	0.15174	9	8	6	7
2960	Acetyl-CoA acetyltransferase, mitochondrial	IPI00030363	-0.446	0.15174	16	16	12	15
2961	Isoform 1 of Polyadenylate-binding protein 1	IPI00008524	-0.451	0.15095	32	21	21	26
2962	Casein kinase II subunit alpha'	IPI00020602	-0.456	0.15039	10	6	7	5
2963	von Hippel-Lindau binding protein 1, isoform CRA_b	IPI00334159	-0.456	0.15039	9	7	7	5
2964	Putative uncharacterized protein MDH1	IPI00915869	-0.456	0.15039	9	7	5	7
2965	Aminoacyl tRNA synthase complex-interacting multifunctional protein 1	IPI00006252	-0.456	0.15039	8	8	6	6
2966	Isoform 2 of Tropomyosin alpha-3 chain	IPI00218319	-0.456	0.15039	7	9	5	7
2967	Thioredoxin domain-containing protein 12	IPI00026328	-0.456	0.15039	7	9	6	6
2968	Catalase	IPI00465436	-0.456	0.15039	12	4	6	6
2969	protein ALO17 isoform 1	IPI00828098	-0.467	0.14544	3	4	2	0
2970	Solute carrier family 2, facilitated glucose transporter member 3	IPI00003909	-0.467	0.14544	2	5	2	2
2971	fatty acid desaturase 1	IPI00784651	-0.467	0.14544	0	5	0	0
2972	Neurolysin, mitochondrial	IPI00010346	-0.467	0.14544	5	2	0	2
2973	Isoform 4 of Uncharacterized protein KIAA0090	IPI00642244	-0.467	0.14544	5	2	2	2
2974	39S ribosomal protein L50, mitochondrial	IPI00329036	-0.467	0.14544	5	2	2	2
2975	Heterogeneous nuclear ribonucleoprotein H2	IPI00026230	-0.467	0.14544	5	0	2	0
2976	Cullin-5	IPI00216003	-0.467	0.14544	5	0	2	0
2977	Isoform 1 of Probable DNA dC->dU-editing enzyme APOBEC-3B	IPI00005531	-0.467	0.14544	5	0	0	0
2978	60S ribosomal protein L17	IPI00413324	-0.467	0.14544	4	3	2	2
2979	Procollagen-lysine,2-oxoglutarate 5-dioxygenase 3	IPI00030255	-0.467	0.14544	4	3	2	2
2980	Cell division protein kinase 7	IPI00000685	-0.467	0.14544	4	3	2	2
2981	Isoform 1 of Elongation factor G, mitochondrial	IPI00154473	-0.467	0.14544	5	0	2	0
2982	Calmodulin	IPI00075248	-0.467	0.14544	3	4	0	0
2983	Isoform 1 of 5'-nucleotidase domain-containing protein 3	IPI00465170	-0.467	0.14544	4	3	2	0
2984	Cleavage stimulation factor subunit 1	IPI00011528	-0.467	0.14544	4	3	2	2
2985	Perilipin-2	IPI00293307	-0.467	0.14544	4	3	0	0
2986	Myosin-If	IPI00218638	-0.467	0.14544	4	3	0	0
2987	Mitochondrial import inner membrane translocase subunit Tim16	IPI00218463	-0.467	0.14544	3	4	2	2
2988	cDNA: FLJ22728 fis, clone HSI15617 (Fragment)	IPI00386139	-0.467	0.14544	4	3	0	0
2989	Serpin B9	IPI00032139	-0.467	0.14544	4	3	2	0
2990	Protein kinase C and casein kinase substrate in neurons 3, isoform CRA_b	IPI00329572	-0.467	0.14544	4	3	2	0
2991	Conserved hypothetical protein	IPI00477526	-0.467	0.14544	2	5	2	2
2992	Transcription initiation factor TFIID subunit 9	IPI00002993	-0.467	0.14544	4	3	0	0
2993	Serine palmitoyltransferase 2	IPI00005751	-0.467	0.14544	5	0	2	0
2994	Charged multivesicular body protein 5	IPI00100796	-0.467	0.14544	4	3	0	0
2995	Isoform 2 of Protein SET	IPI00301311	-0.467	0.14544	4	3	0	0
2996	Serine/threonine-protein phosphatase 2A catalytic subunit alpha isoform	IPI00008380	-0.467	0.14332	10	5	7	4
2997	Microsomal glutathione S-transferase 3	IPI00024266	-0.467	0.14332	7	8	4	7
2998	Isoform 1 of N-alpha-acetyltransferase 50, NatE catalytic subunit	IPI00018627	-0.467	0.14332	9	6	6	5
2999	Heat shock 70 kDa protein 1A/1B	IPI00304925	-0.474	0.14276	27	19	20	20
3000	Isoform 1 of Filamin-C	IPI00178352	-0.474	0.14254	16	11	9	13
3001	Plastin-1	IPI00032304	-0.474	0.14254	15	12	11	11
3002	Isoform 1 of Adipocyte plasma membrane-associated protein	IPI00031131	-0.480	0.14213	10	4	6	4
3003	Isoform 1 of Hexokinase-1	IPI00018246	-0.480	0.14213	9	5	5	5
3004	Ephrin type-A receptor 2	IPI00021267	-0.481	0.13792	13	13	8	13
3005	Splicing factor 3A subunit 3	IPI00029764	-0.481	0.13792	15	11	8	13
3006	Keratin, type I cytoskeletal 19	IPI00479145	-0.487	0.13699	109	85	95	89
3007	Isoform Cytoplasmic of Lysyl-tRNA synthetase	IPI00014238	-0.493	0.13680	23	18	17	18
3008	60S ribosomal protein L26-like 1	IPI00007144	-0.494	0.13680	8	5	3	6
3009	Isoform 1 of Protein strawberry notch homolog 1	IPI00023649	-0.494	0.13680	7	6	5	4
3010	Pre-mRNA-splicing factor SPF27	IPI00025178	-0.494	0.13680	6	7	4	5
3011	Isoform 2 of Formin-like protein 1	IPI00025202	-0.494	0.13680	7	6	5	4
3012	Isoform 1 of Cleavage and polyadenylation specificity factor subunit 7	IPI00550821	-0.494	0.13680	10	3	4	5
3013	40S ribosomal protein S16	IPI00221092	-0.497	0.13613	18	22	18	16
3014	Protein phosphatase 1G	IPI00006167	-0.503	0.13584	12	11	9	9
3015	Keratin, type I cytoskeletal 9	IPI00019359	-0.504	0.13565	56	34	41	41
3016	Prohibitin-2	IPI00027252	-0.508	0.13505	30	29	27	25
3017	Barrier-to-autointegration factor	IPI00026087	-0.510	0.13505	10	0	5	3
3018	Isoform 1 of DNA replication licensing factor MCM7	IPI00299904	-0.510	0.13505	9	3	4	4
3019	Inositol monophosphatase 1	IPI00020906	-0.510	0.13505	6	6	6	2
3020	SUMO-activating enzyme subunit 1	IPI00033130	-0.510	0.13505	8	4	5	3
3021	cDNA FLJ14239 fis, clone NT2RP5003512, highly similar to Exportin-5	IPI00549861	-0.510	0.13505	7	5	3	5
3022	Regulator of microtubule dynamics protein 1	IPI00329696	-0.510	0.13505	6	6	5	3
3023	Isoform 3 of Serine/threonine-protein phosphatase 2A activator	IPI00217296	-0.510	0.13505	8	4	5	3

No.	Description	Accession number	STN[1]	p-Value[1]	Con_A[2]	Con_B[2]	5FU_A[2]	5FU_B[2]
3024	Paladin	IPI00297212	-0.510	0.13505	6	6	6	2
3025	Signal recognition particle 72 kDa protein	IPI00215888	-0.510	0.13505	7	5	4	4
3026	Isoform 1 of Paraspeckle component 1	IPI00103525	-0.510	0.13505	7	5	4	4
3027	Tubulin--tyrosine ligase-like protein 12	IPI00029048	-0.511	0.12854	15	7	8	9
3028	Isoform 1 of Catenin alpha-1	IPI00215948	-0.511	0.12854	14	8	5	12
3029	proteasome subunit beta type-5 isoform 3	IPI00383971	-0.520	0.12697	11	10	11	5
3030	Isoform Alpha-6X1X2B of Integrin alpha-6	IPI00010697	-0.527	0.12623	20	14	11	17
3031	Mitochondrial import receptor subunit TOM22 homolog	IPI00024976	-0.528	0.12560	6	5	4	3
3032	Isoform 2 of tRNA pseudouridine synthase A	IPI00001716	-0.528	0.12560	9	0	4	3
3033	Isoform 1 of Annexin A7	IPI00002460	-0.528	0.12560	6	5	4	3
3034	ATP-dependent RNA helicase DDX18	IPI00301323	-0.530	0.12560	15	5	8	7
3035	Putative uncharacterized protein INF2	IPI00872508	-0.530	0.12560	11	9	8	7
3036	Citrate synthase, mitochondrial	IPI00025366	-0.533	0.12437	22	11	16	11
3037	Isoform Mitochondrial of Glutathione reductase, mitochondrial	IPI00016862	-0.540	0.12295	9	10	5	9
3038	Isoform 1 of DNA-binding protein A	IPI00031801	-0.545	0.12280	15	16	12	13
3039	Epithelial cell adhesion molecule	IPI00296215	-0.550	0.12254	8	2	3	3
3040	Probable ATP-dependent RNA helicase DDX10	IPI00297900	-0.550	0.12254	6	4	3	3
3041	Isoform Delta-1 of Serine/threonine-protein phosphatase 2A 56 kDa regulatory subunit delta isoform	IPI00000030	-0.550	0.12254	8	0	0	4
3042	74 kDa protein	IPI00290439	-0.550	0.12254	6	4	4	2
3043	Pre-rRNA-processing protein TSR1 homolog	IPI00292894	-0.550	0.12254	6	4	4	2
3044	Isoform A of Peptidyl-prolyl cis-trans isomerase E	IPI00009316	-0.550	0.12254	5	5	3	3
3045	F-box-like/WD repeat-containing protein TBL1XR1	IPI00002922	-0.550	0.12254	5	5	0	4
3046	Isoform 2 of 3-hydroxyisobutyryl-CoA hydrolase, mitochondrial	IPI00377161	-0.550	0.12254	5	5	4	2
3047	probable E3 ubiquitin-protein ligase MYCBP2	IPI00289776	-0.552	0.11729	14	16	12	12
3048	KH-type splicing regulatory protein	IPI00479786	-0.552	0.11729	15	15	15	9
3049	Uncharacterized protein C17orf25	IPI00007102	-0.552	0.11595	9	9	8	5
3050	Isoform 1 of Transformer-2 protein homolog beta	IPI00301503	-0.552	0.11595	10	8	6	7
3051	3-mercaptopyruvate sulfurtransferase	IPI00165360	-0.564	0.11413	10	7	6	6
3052	Protein kinase, cAMP-dependent, regulatory, type II, alpha, isoform CRA_b	IPI00063234	-0.564	0.11413	10	7	6	6
3053	Isoform 1 of Elongation factor 1-delta	IPI00023048	-0.568	0.11301	21	22	18	18
3054	Isoform 1 of Protein diaphanous homolog 1	IPI00852685	-0.573	0.11294	19	8	7	14
3055	ADP-ribosylation factor 6	IPI00215920	-0.573	0.11294	14	13	10	11
3056	cDNA FLJ59571, highly similar to Eukaryotic translation initiation factor 4gamma 2	IPI00015952	-0.576	0.11294	7	2	3	2
3057	Isoform 2 of Inverted formin-2	IPI00876962	-0.576	0.11294	6	3	3	2
3058	Platelet-activating factor acetylhydrolase IB subunit beta	IPI00026546	-0.576	0.11294	6	3	3	0
3059	Isoform 1 of AP-3 complex subunit beta-1	IPI00021129	-0.576	0.11294	4	5	2	3
3060	Cell division protein kinase 2	IPI00031681	-0.576	0.11294	4	5	3	0
3061	DnaJ homolog subfamily C member 8	IPI00003438	-0.576	0.11294	5	4	3	2
3062	Carnitine O-palmitoyltransferase 2, mitochondrial	IPI00012912	-0.576	0.11294	5	4	0	3
3063	Isoform 2 of TIP41-like protein	IPI00641815	-0.576	0.11294	5	4	3	2
3064	Prostaglandin E synthase 2	IPI00303568	-0.576	0.11294	5	4	2	3
3065	Isoform 2 of Sorting nexin-3	IPI00216508	-0.576	0.11294	3	6	3	2
3066	Tubulin beta-2A chain	IPI00013475	-0.578	0.11294	10	6	6	5
3067	Isoform 3 of Probable ATP-dependent RNA helicase DDX17	IPI00651653	-0.578	0.11294	8	8	6	5
3068	Calcium-binding protein 39-like	IPI00026359	-0.578	0.11294	11	5	6	5
3069	Isoform 4 of Abhydrolase domain-containing protein 11	IPI00171152	-0.578	0.11294	7	9	8	3
3070	Heterogeneous nuclear ribonucleoprotein A0	IPI00011913	-0.578	0.11294	8	8	6	5
3071	Acidic leucine-rich nuclear phosphoprotein 32 family member E	IPI00165393	-0.578	0.11294	6	10	6	5
3072	Alpha-actinin-4	IPI00013808	-0.582	0.11100	80	72	67	74
3073	60S ribosomal protein L23a	IPI00021266	-0.588	0.11025	20	19	17	15
3074	sister chromatid cohesion protein PDS5 homolog A isoform 2	IPI00303063	-0.590	0.11018	13	12	11	8
3075	Isoform 3 of Splicing factor, arginine/serine-rich 13A	IPI00009071	-0.590	0.11018	14	11	13	6
3076	Eukaryotic translation initiation factor 5A-2	IPI00006935	-0.593	0.11018	7	8	4	6
3077	tRNA methyltransferase 112 homolog	IPI00009010	-0.593	0.11018	8	7	6	4
3078	UPF0160 protein MYG1, mitochondrial	IPI00029444	-0.593	0.11018	8	7	4	6
3079	60S ribosomal protein L27a	IPI00456758	-0.593	0.11018	8	7	6	4
3080	Protein of unknown function DUF410 family protein	IPI00419575	-0.593	0.11018	8	7	5	5
3081	Lon protease homolog, mitochondrial	IPI00005158	-0.594	0.10977	23	15	13	18
3082	Isoform 1 of Coatomer subunit alpha	IPI00295857	-0.595	0.10932	58	49	47	50
3083	THO complex subunit 4	IPI00328840	-0.606	0.10795	19	17	16	13
3084	Phosphatidylinositol-4-phosphate 3-kinase C2 domain-containing subunit alpha	IPI00002580	-0.607	0.10783	6	2	0	2
3085	Isoform 1 of Mps one binder kinase activator-like 1B	IPI00301518	-0.607	0.10783	5	3	2	2
3086	Putative RNA-binding protein 3	IPI00024320	-0.607	0.10783	4	4	0	0
3087	Isoform 1 of WD repeat-containing protein 44	IPI00444371	-0.607	0.10783	4	4	2	2
3088	Ubiquitin-conjugating enzyme E2 T	IPI00023087	-0.607	0.10783	4	4	2	2
3089	Ribosomal protein S6 kinase alpha-3	IPI00020898	-0.607	0.10783	4	4	2	2
3090	Isoform 1 of Uncharacterized methyltransferase WBSCR22	IPI00013810	-0.607	0.10783	5	3	2	0
3091	Isoform 2 of Phosphatidylinositol-binding clathrin assembly protein	IPI00216184	-0.607	0.10783	5	3	2	2
3092	Fumarylacetoacetate hydrolase domain-containing protein 2B	IPI00301994	-0.607	0.10783	3	5	2	0
3093	Isoform 2 of Guanine nucleotide-binding protein-like 3	IPI00003886	-0.607	0.10783	6	0	0	0
3094	Isoform Short of TATA-binding protein-associated factor 2N	IPI00020194	-0.607	0.10783	6	2	2	2
3095	Cell growth-regulating nucleolar protein	IPI00015838	-0.607	0.10783	6	2	2	2
3096	Guanine nucleotide-binding protein G(k) subunit alpha	IPI00220578	-0.607	0.10783	6	2	2	2
3097	Propionyl-CoA carboxylase beta chain, mitochondrial	IPI00007247	-0.607	0.10783	6	2	0	2
3098	cDNA FLJ60094, highly similar to F-actin capping protein subunit beta	IPI00218782	-0.607	0.10783	4	4	0	2
3099	Ribonuclease UK114	IPI00005038	-0.607	0.10783	3	5	2	2
3100	Dipeptidase 1	IPI00059476	-0.607	0.10783	5	3	0	0
3101	Isoform 1 of RNA polymerase II-associated factor 1 homolog	IPI00300333	-0.607	0.10783	5	3	2	2
3102	Programmed cell death 6-interacting protein	IPI00246058	-0.609	0.09763	14	9	10	7
3103	Isoform 2 of Guanine nucleotide-binding protein G(i) subunit alpha-2	IPI00217906	-0.609	0.09763	13	10	8	9
3104	Isoform 1 of Inorganic pyrophosphatase 2, mitochondrial	IPI00301109	-0.610	0.09748	8	6	5	4
3105	Isoform 1 of BRCA2 and CDKN1A-interacting protein	IPI00002203	-0.610	0.09748	12	0	5	4
3106	Peroxiredoxin-2	IPI00027350	-0.612	0.09503	19	16	17	11
3107	Isoform M2 of Pyruvate kinase isozymes M1/M2	IPI00479186	-0.614	0.09503	39	12	20	23
3108	Serine hydroxymethyltransferase, mitochondrial	IPI00002520	-0.614	0.09503	36	15	23	20
3109	Isoform 1 of Deoxyuridine 5'-triphosphate nucleotidohydrolase, mitochondrial	IPI00013679	-0.625	0.09216	18	15	15	11
3110	Pyruvate dehydrogenase protein X component, mitochondrial	IPI00298423	-0.629	0.09201	8	5	5	3
3111	Guanine nucleotide-binding protein subunit alpha-13	IPI00290928	-0.629	0.09201	6	7	3	5
3112	WD repeat-containing protein 61	IPI00019269	-0.629	0.09201	8	5	4	4
3113	Nucleolar GTP-binding protein 1	IPI00385042	-0.631	0.09160	12	9	7	8
3114	Spermidine synthase	IPI00292020	-0.631	0.09160	11	10	6	9
3115	ATP-dependent RNA helicase DDX3X	IPI00215637	-0.632	0.09160	20	12	11	14
3116	Nucleosome assembly protein 1-like 1	IPI00023860	-0.640	0.09093	18	13	12	12
3117	6-phosphogluconate dehydrogenase, decarboxylating	IPI00219525	-0.640	0.09093	18	13	16	8

No.	Description	Accession number	STN[1]	p-Value[1]	Con_A[2]	Con_B[2]	5FU_A[2]	5FU_B[2]
3118	Isoform 1 of 40S ribosomal protein S24	IPI00029750	-0.643	0.09074	13	7	9	5
3119	Isoform 2 of ATP-binding cassette sub-family F member 1	IPI00013495	-0.643	0.09074	12	8	7	7
3120	Long-chain-fatty-acid--CoA ligase 3	IPI00031397	-0.643	0.09074	7	13	7	7
3121	Isoform A1-B of Heterogeneous nuclear ribonucleoprotein A1	IPI00215965	-0.647	0.08836	56	28	43	31
3122	60S ribosomal protein L7	IPI00030179	-0.648	0.08836	14	16	12	11
3123	Proteasome subunit alpha type-2	IPI00219622	-0.648	0.08836	16	14	13	10
3124	Isoform 1 of Minor histocompatibility antigen H13	IPI00152441	-0.650	0.08825	7	5	3	4
3125	Isoform 1 of E3 UFM1-protein ligase 1	IPI00844000	-0.650	0.08825	7	5	4	3
3126	Transmembrane 9 superfamily member 2	IPI00018415	-0.650	0.08825	6	6	5	0
3127	Hypoxanthine-guanine phosphoribosyltransferase	IPI00218493	-0.654	0.08825	32	28	29	22
3128	CAD protein	IPI00301263	-0.655	0.08803	91	86	85	79
3129	Protein FAM49B	IPI00303318	-0.656	0.08803	9	10	6	7
3130	Glutathione synthetase	IPI00010706	-0.656	0.08803	12	7	8	5
3131	ADP-ribosylation factor-like protein 3	IPI00003327	-0.656	0.08803	9	10	7	6
3132	60S ribosomal protein L7a	IPI00299573	-0.658	0.08799	22	20	18	16
3133	ADP-ribosylation factor 4	IPI00215918	-0.664	0.08773	20	21	17	16
3134	Isoform 4 of Heterogeneous nuclear ribonucleoprotein A/B	IPI00106509	-0.670	0.08687	10	8	7	5
3135	cDNA FLJ59367, highly similar to Adenylosuccinate lyase	IPI00026904	-0.676	0.08445	9	0	4	2
3136	Deoxyhypusine hydroxylase	IPI00171856	-0.676	0.08445	6	5	4	0
3137	Isoform Long of Deoxyhypusine synthase	IPI00026829	-0.676	0.08445	9	2	4	0
3138	Isoform 2 of Lysine-specific histone demethylase 1A	IPI00217540	-0.676	0.08445	6	5	3	3
3139	Na(+)/H(+) exchange regulatory cofactor NHE-RF1	IPI00003527	-0.676	0.08445	7	4	4	0
3140	Isoform 2 of Tumor protein D54	IPI00221178	-0.676	0.08445	4	7	3	3
3141	Plastin-2	IPI00010471	-0.681	0.08385	63	33	39	46
3142	Isoform 1 of L-lactate dehydrogenase A chain	IPI00217966	-0.683	0.08356	80	43	60	51
3143	mRNA turnover protein 4 homolog	IPI00106491	-0.686	0.08315	8	9	5	6
3144	Isoform 2 of Basigin	IPI00019906	-0.689	0.08304	19	18	11	18
3145	Transgelin-2	IPI00550363	-0.689	0.08304	18	19	13	16
3146	Malate dehydrogenase	IPI00916111	-0.703	0.08233	6	10	5	5
3147	DnaJ homolog subfamily A member 2	IPI00032406	-0.703	0.08233	10	6	5	5
3148	Aflatoxin B1 aldehyde reductase member 2	IPI00305978	-0.703	0.08233	8	8	4	6
3149	Isoform 1 of Heterogeneous nuclear ribonucleoprotein Q	IPI00018140	-0.704	0.07942	40	26	27	29
3150	14-3-3 protein zeta/delta	IPI00021263	-0.705	0.07935	17	7	9	8
3151	Isoform Long of 14-3-3 protein beta/alpha	IPI00216318	-0.705	0.07935	15	9	11	6
3152	Proteasome subunit beta type-5	IPI00479306	-0.705	0.07935	12	12	9	8
3153	Uncharacterized protein KIAA1797	IPI00748360	-0.706	0.07890	4	6	3	2
3154	Apolipoprotein O-like	IPI00394809	-0.706	0.07890	4	6	2	3
3155	Isoform 2 of Serine/threonine-protein phosphatase PGAM5, mitochondrial	IPI00063242	-0.717	0.07797	11	12	7	9
3156	Isoform 1 of RNA-binding protein 39	IPI00163505	-0.717	0.07797	12	11	8	8
3157	Isoform Complexed of Arginyl-tRNA synthetase, cytoplasmic	IPI00004860	-0.719	0.07793	21	12	16	9
3158	Isoform 1 of Oxysterol-binding protein 1	IPI00024971	-0.723	0.07782	9	6	4	5
3159	28S ribosomal protein S26, mitochondrial	IPI00006606	-0.723	0.07782	6	9	3	6
3160	Isoform Mitochondrial of Fumarate hydratase, mitochondrial	IPI00296053	-0.730	0.07730	16	6	9	6
3161	Peptidyl-prolyl cis-trans isomerase B	IPI00646304	-0.733	0.07652	32	27	27	22
3162	60S ribosomal protein L10a	IPI00412579	-0.736	0.07633	20	11	12	11
3163	AP-1 complex subunit gamma-1 isoform a	IPI00293396	-0.743	0.07622	7	2	2	0
3164	Myeloid-associated differentiation marker	IPI00102685	-0.743	0.07622	6	3	0	2
3165	Ribosomal protein S6 kinase alpha-1	IPI00017305	-0.743	0.07622	6	3	2	2
3166	Isoform 3 of Protein VPRBP	IPI00181396	-0.743	0.07622	5	4	0	2
3167	Isoform 1 of Fatty aldehyde dehydrogenase	IPI00333619	-0.743	0.07622	5	4	0	2
3168	FAS-associated factor 2	IPI00172656	-0.743	0.07622	4	5	0	0
3169	Maspardin	IPI00010248	-0.743	0.07622	5	4	0	0
3170	Isoform 1 of Serine hydroxymethyltransferase, cytosolic	IPI00002519	-0.743	0.07622	6	3	0	0
3171	Isoform 1 of Transmembrane protein 111	IPI00020472	-0.743	0.07622	6	3	2	0
3172	Isoform 3 of Pre-mRNA 3'-end-processing factor FIP1	IPI00008449	-0.743	0.07622	7	0	2	2
3173	Nucleoside-triphosphatase C1orf57	IPI00031570	-0.743	0.07622	7	2	2	2
3174	Probable ATP-dependent RNA helicase DDX27	IPI00293078	-0.743	0.07622	6	3	0	0
3175	Activator of 90 kDa heat shock protein ATPase homolog 1	IPI00030706	-0.744	0.07496	16	5	10	4
3176	60S ribosomal protein L18	IPI00215719	-0.744	0.07496	12	9	6	8
3177	60S ribosomal protein L7-like 1	IPI00456940	-0.744	0.07469	7	7	4	4
3178	39S ribosomal protein L19, mitochondrial	IPI00027096	-0.744	0.07469	8	6	5	3
3179	Seryl-tRNA synthetase, mitochondrial	IPI00328361	-0.744	0.07469	7	7	3	5
3180	X-ray repair cross-complementing protein 5	IPI00220834	-0.746	0.07268	47	27	34	29
3181	Isoform 1 of Heat shock cognate 71 kDa protein	IPI00003865	-0.748	0.07257	78	71	65	70
3182	Isoform 1 of Eukaryotic translation initiation factor 3 subunit B	IPI00396370	-0.750	0.07257	26	15	13	19
3183	Cytochrome c oxidase subunit 5A, mitochondrial	IPI00025086	-0.758	0.07194	12	8	6	7
3184	Profilin-1	IPI00216691	-0.761	0.07157	114	97	99	96
3185	Keratin, type II cytoskeletal 8	IPI00554648	-0.764	0.07153	246	161	201	186
3186	Isoform 1 of Platelet-activating factor acetylhydrolase IB subunit alpha	IPI00218728	-0.766	0.07149	10	18	10	10
3187	Glycogen phosphorylase, liver form	IPI00783313	-0.766	0.07149	17	11	13	7
3188	Isoform 3 of Obg-like ATPase 1	IPI00216106	-0.769	0.07078	11	0	5	0
3189	Proteasome subunit beta type-6	IPI00000811	-0.769	0.07078	9	4	3	4
3190	Heat shock 70 kDa protein 4	IPI00002966	-0.771	0.07078	54	33	42	33
3191	ATP-dependent RNA helicase DDX1	IPI00293655	-0.775	0.07078	17	2	6	6
3192	D-3-phosphoglycerate dehydrogenase	IPI00011200	-0.775	0.07078	10	9	7	5
3193	Signal recognition particle receptor subunit beta	IPI00295098	-0.777	0.07064	16	11	10	9
3194	Prohibitin	IPI00017334	-0.777	0.07064	36	30	31	24
3195	Isoform 1 of Protein-L-isoaspartate(D-aspartate) O-methyltransferase	IPI00411680	-0.779	0.07049	18	19	14	14
3196	Isoform Beta-4C of Integrin beta-4	IPI00027422	-0.784	0.07038	27	22	15	24
3197	Endoplasmin	IPI00027230	-0.786	0.07038	42	22	27	26
3198	14-3-3 protein theta	IPI00018146	-0.788	0.07026	19	17	15	12
3199	Isoform Beta of Heat shock protein 105 kDa	IPI00218993	-0.790	0.06937	30	18	16	22
3200	Isoform 1 of 60S ribosomal protein L11	IPI00376798	-0.792	0.06926	8	10	4	7
3201	Transcription elongation factor B polypeptide 2	IPI00026670	-0.792	0.06926	11	7	6	5
3202	Isoform 2 of Transportin-3	IPI00395694	-0.798	0.06840	8	4	3	3
3203	Isoform 2 of AP-2 complex subunit alpha-2	IPI00016621	-0.798	0.06840	6	6	4	2
3204	Dihydrolipoyl dehydrogenase, mitochondrial	IPI00015911	-0.800	0.06583	12	13	9	8
3205	Guanine nucleotide-binding protein subunit beta-2-like 1	IPI00848226	-0.809	0.06553	28	31	25	23
3206	ADP/ATP translocase 1	IPI00022891	-0.812	0.06550	8	9	5	5
3207	BAG family molecular chaperone regulator 2	IPI00000643	-0.812	0.06550	8	9	5	5
3208	Heterogeneous nuclear ribonucleoprotein U-like protein 2	IPI00456887	-0.814	0.06442	20	13	10	14
3209	Isoform 1 of Transcription factor BTF3	IPI00221035	-0.824	0.06382	17	15	12	11
3210	Plastin-3	IPI00216694	-0.828	0.06330	13	10	8	7
3211	Actin-related protein 2/3 complex subunit 1B	IPI00005160	-0.833	0.06323	5	6	3	0
3212	ATP-binding cassette sub-family F member 2	IPI00005045	-0.833	0.06323	7	4	0	3

No.	Description	Accession number	STN[1]	p-Value[1]	Con_A[2]	Con_B[2]	5FU_A[2]	5FU_B[2]
3213	Mitochondrial-processing peptidase subunit alpha	IPI00166749	-0.833	0.06323	6	5	3	0
3214	Isoform 1 of Translocon-associated protein subunit alpha	IPI00301021	-0.833	0.06323	7	4	2	3
3215	Polymerase delta-interacting protein 2	IPI00165506	-0.833	0.06323	9	7	6	3
3216	Ubiquitin-conjugating enzyme E2 L3	IPI00021347	-0.833	0.06323	10	6	5	4
3217	Peptidyl-prolyl cis-trans isomerase FKBP4	IPI00219005	-0.841	0.06297	38	30	27	29
3218	Coatomer subunit gamma	IPI00783982	-0.843	0.06293	15	7	8	6
3219	Calreticulin	IPI00020599	-0.853	0.06192	31	20	22	18
3220	U4/U6.U5 tri-snRNP-associated protein 1	IPI00021417	-0.858	0.06177	10	5	4	4
3221	Isoform 1 of AP-2 complex subunit beta	IPI00784156	-0.858	0.06177	11	4	4	4
3222	Paired amphipathic helix protein Sin3a	IPI00170596	-0.859	0.06159	10	11	9	4
3223	14-3-3 protein gamma	IPI00220642	-0.859	0.06159	14	7	9	4
3224	Isoform 1 of Acetyl-CoA carboxylase 1	IPI00011569	-0.866	0.06081	22	27	14	24
3226	60S ribosomal protein L5	IPI00000494	-0.873	0.06058	22	26	21	16
3227	40S ribosomal protein S5	IPI00008433	-0.874	0.06055	8	0	2	0
3228	Isoform A of Nucleoporin SEH1	IPI00185533	-0.874	0.06055	5	5	0	2
3229	Isoform 1 of Disks large homolog 1	IPI00030351	-0.874	0.06055	6	4	2	2
3230	Isoform Long of Cold shock domain-containing protein E1	IPI00470891	-0.874	0.06055	7	3	2	2
3231	Putative myosin-XVB	IPI00786880	-0.874	0.06055	8	0	2	0
3232	cDNA FLJ38069 fis, clone CTONG2015434	IPI00029159	-0.874	0.06055	6	4	0	2
3233	51 kDa protein	IPI00033025	-0.874	0.06055	6	4	2	2
3234	Isoform 1 of Required for meiotic nuclear division protein 1 homolog	IPI00329591	-0.874	0.06055	5	5	2	2
3235	Neprilysin	IPI00247063	-0.874	0.06055	5	5	0	2
3236	Isoform 1 of SWI/SNF-related matrix-associated actin-dependent regulator of chromatin subfamily E	IPI00017669	-0.874	0.06055	5	5	2	2
3237	Alpha-soluble NSF attachment protein	IPI00009253	-0.877	0.05649	10	10	5	7
3238	Complement component 1 Q subcomponent-binding protein, mitochondrial	IPI00014230	-0.885	0.05425	29	30	23	24
3239	Thioredoxin-like protein 1	IPI00305692	-0.886	0.05425	5	9	4	3
3240	26S proteasome non-ATPase regulatory subunit 5	IPI00002134	-0.886	0.05425	7	7	2	5
3241	Putative deoxyribose-phosphate aldolase	IPI00219677	-0.886	0.05425	10	4	4	3
3242	Tyrosine-protein phosphatase non-receptor type 1	IPI00297261	-0.886	0.05425	5	9	3	4
3243	Bifunctional purine biosynthesis protein PURH	IPI00289499	-0.891	0.05395	38	20	24	22
3244	60S ribosomal protein L4	IPI00003918	-0.897	0.05377	11	8	6	5
3245	DnaJ homolog subfamily A member 1	IPI00012535	-0.918	0.05258	11	7	5	5
3246	40S ribosomal protein S17	IPI00221093	-0.924	0.05105	15	9	8	7
3247	Mitochondrial carrier homolog 2	IPI00003833	-0.933	0.05038	15	16	10	11
3248	Transitional endoplasmic reticulum ATPase	IPI00022774	-0.935	0.05027	33	18	18	21
3249	Phosphoserine aminotransferase	IPI00001734	-0.935	0.05027	23	17	17	12
3250	Putative uncharacterized protein RPL17	IPI00394699	-0.942	0.04997	8	9	4	5
3251	Nuclear pore complex protein Nup107	IPI00028005	-0.942	0.04997	11	6	4	5
3252	Mitochondrial import inner membrane translocase subunit Tim23	IPI00007309	-0.942	0.04997	8	9	5	4
3253	UPF0027 protein C22orf28	IPI00550689	-0.959	0.04882	15	14	11	8
3254	Cytochrome c oxidase subunit 2	IPI00017510	-0.959	0.04882	13	9	7	6
3255	ADP-ribosylation factor-like protein 1	IPI00219518	-0.969	0.04859	9	7	4	4
3256	Isoform 1 of Transportin-1	IPI00024364	-0.969	0.04859	11	5	6	2
3257	60S ribosomal protein L27	IPI00219155	-0.978	0.04654	8	13	7	5
3258	GDP-mannose 4,6 dehydratase	IPI00030207	-0.978	0.04654	13	8	6	6
3259	DNA replication licensing factor MCM4	IPI00018349	-0.985	0.04654	25	10	14	10
3260	Isoform Mitochondrial of Peroxiredoxin-5, mitochondrial	IPI00024915	-0.997	0.04632	24	19	16	15
3261	Heterogeneous nuclear ribonucleoprotein H	IPI00013881	-1.000	0.04628	8	7	3	4
3262	Isoform 1 of Histone-arginine methyltransferase CARM1	IPI00412880	-1.000	0.04628	8	7	2	5
3263	39S ribosomal protein L46, mitochondrial	IPI00023161	-1.000	0.04628	8	7	4	3
3264	Transducin beta-like protein 3	IPI00477971	-1.000	0.04628	10	10	6	5
3265	Heat shock protein beta-1	IPI00025512	-1.000	0.04628	10	10	6	5
3266	Isoform 1 of Caprin-1	IPI00783872	-1.000	0.04628	14	6	6	5
3267	Large neutral amino acids transporter small subunit 1	IPI00008986	-1.002	0.04625	2	9	0	2
3268	Peptidyl-prolyl cis-trans isomerase D	IPI00003927	-1.002	0.04625	7	4	2	2
3269	Isoform 1 of Sorting nexin-12	IPI00438170	-1.002	0.04625	6	5	2	2
3270	Puromycin-sensitive aminopeptidase	IPI00026216	-1.003	0.04602	16	10	7	9
3271	Threonyl-tRNA synthetase, cytoplasmic	IPI00329633	-1.003	0.04602	16	10	10	6
3272	U2 small nuclear ribonucleoprotein A'	IPI00297477	-1.006	0.04554	22	20	17	13
3273	26S protease regulatory subunit 6A	IPI00018398	-1.016	0.04535	22	19	19	10
3274	Proteasome 26S non-ATPase subunit 11 variant (Fragment)	IPI00105598	-1.021	0.04520	15	17	10	11
3275	Probable ATP-dependent RNA helicase DDX47	IPI00023972	-1.035	0.04461	8	6	4	2
3276	Isoform 3 of Oxidation resistance protein 1	IPI00166807	-1.035	0.04461	7	7	2	4
3277	Histone H1.2	IPI00217465	-1.049	0.04245	25	22	16	18
3278	Probable ribosome biogenesis protein NEP1	IPI00025347	-1.049	0.04245	10	8	4	5
3279	Lysophospholipid acyltransferase 5	IPI00306419	-1.049	0.04245	9	9	4	5
3280	40S ribosomal protein S10	IPI00008438	-1.061	0.04211	27	29	24	18
3281	Alpha-actinin-1	IPI00013508	-1.071	0.04178	130	70	89	89
3282	Isoform 1 of Calcyclin-binding protein	IPI00395627	-1.075	0.04155	29	25	22	18
3283	Probable dimethyladenosine transferase	IPI00004459	-1.076	0.04129	7	6	2	3
3284	cDNA FLJ54030, highly similar to Polymerase delta-interacting protein 3	IPI00440688	-1.076	0.04129	6	7	2	3
3285	Heterogeneous nuclear ribonucleoprotein F	IPI00003881	-1.078	0.04129	14	8	9	3
3286	Sec1 family domain-containing protein 1	IPI00165261	-1.078	0.04077	10	7	4	4
3287	Replication factor C subunit 5	IPI00031514	-1.079	0.04074	17	11	10	7
3288	Destrin	IPI00473014	-1.096	0.03980	17	10	10	6
3289	Signal recognition particle 54 kDa protein	IPI00009822	-1.096	0.03980	16	11	8	8
3290	Importin subunit beta-1	IPI00001639	-1.099	0.03969	36	15	18	19
3291	Isoform D of Constitutive coactivator of PPAR-gamma-like protein 1	IPI00039626	-1.111	0.03917	9	7	3	4
3292	Leucine-rich repeat-containing protein 59	IPI00396321	-1.122	0.03887	16	16	10	10
3293	Importin-11	IPI00301107	-1.126	0.03809	5	7	0	2
3294	Isoform 2 of Cytosolic non-specific dipeptidase	IPI00165579	-1.126	0.03809	7	5	0	2
3295	Ribosome biogenesis protein WDR12	IPI00304232	-1.126	0.03809	10	2	2	0
3296	sulfatase modifying factor 2 isoform b precursor	IPI00171412	-1.126	0.03809	9	3	2	0
3297	Voltage-dependent anion-selective channel protein 1	IPI00216308	-1.138	0.03444	50	44	36	40
3298	baculoviral IAP repeat-containing protein 6	IPI00299635	-1.149	0.03429	7	8	4	2
3299	Eukaryotic translation initiation factor 2 subunit 2	IPI00021728	-1.154	0.03366	11	8	4	5
3300	Ras-related protein Rab-11B	IPI00020436	-1.183	0.03347	29	22	17	19
3301	Isoform 1 of Calcineurin-like phosphoesterase domain-containing protein 1	IPI00305010	-1.194	0.03158	8	6	3	0
3302	Calponin-2	IPI00015262	-1.207	0.03117	15	12	9	6
3303	ADP-ribosylation factor 1	IPI00215914	-1.220	0.03098	73	65	56	60
3304	Ataxin-10	IPI00001636	-1.221	0.03098	12	5	5	2
3305	Superkiller viralicidic activity 2-like 2	IPI00647217	-1.228	0.03087	19	7	5	9
3306	Ezrin	IPI00843975	-1.228	0.03064	12	9	5	5
3307	Tubulin alpha-4A chain	IPI00007750	-1.229	0.03061	152	68	100	94

No.	Description	Accession number	STN[1]	p-Value[1]	Con_A[2]	Con_B[2]	5FU_A[2]	5FU_B[2]
3308	Aminoacyl tRNA synthase complex-interacting multifunctional protein 2	IPI00011916	-1.248	0.02994	6	7	2	2
3309	Isoform 2 of AP-3 complex subunit delta-1	IPI00289608	-1.248	0.02994	7	6	0	2
3310	Isoform 1 of Uridine-cytidine kinase 2	IPI00065671	-1.248	0.02994	6	7	2	2
3311	Isoform 3 of Tyrosine-protein kinase-like 7	IPI00168813	-1.248	0.02994	5	8	0	2
3312	DnaJ homolog subfamily C member 9	IPI00154975	-1.248	0.02994	6	7	2	2
3313	Isoform 2 of Eukaryotic translation initiation factor 5A-1	IPI00376005	-1.250	0.02990	18	7	6	7
3314	SERPINE1 mRNA binding protein 1, isoform CRA_d	IPI00410693	-1.258	0.02964	12	8	5	4
3315	26S proteasome non-ATPase regulatory subunit 12	IPI00185374	-1.260	0.02964	19	11	12	5
3316	Transmembrane protein 33	IPI00299084	-1.262	0.02964	8	8	4	0
3317	NADH dehydrogenase [ubiquinone] 1 alpha subcomplex subunit 9, mitochondrial	IPI00003968	-1.264	0.02919	20	16	14	8
3318	cDNA FLJ60299, highly similar to Rab GDP dissociation inhibitor beta	IPI00031461	-1.285	0.02837	32	26	24	17
3319	60S ribosomal protein L9	IPI00031691	-1.287	0.02837	26	23	17	16
3320	40S ribosomal protein S2	IPI00013485	-1.318	0.02722	23	16	14	10
3321	26S protease regulatory subunit 7	IPI00021435	-1.328	0.02681	14	8	4	6
3322	Isoform 1 of Heterogeneous nuclear ribonucleoprotein H3	IPI00013877	-1.345	0.02573	23	21	14	14
3323	Mitochondrial ribosomal protein L21 isoform d	IPI00375677	-1.366	0.02517	8	6	0	0
3324	Isoform 1 of U3 small nucleolar RNA-associated protein 14 homolog A	IPI00107113	-1.366	0.02517	7	7	2	0
3325	Flotillin-1	IPI00027438	-1.366	0.02517	8	6	0	0
3326	Proliferating cell nuclear antigen	IPI00021700	-1.367	0.02353	40	27	29	19
3327	Similar to Signal peptidase complex subunit 2	IPI00452747	-1.369	0.02353	15	15	9	7
3328	Transferrin receptor protein 1	IPI00022462	-1.413	0.02186	19	9	5	9
3329	Isoform 4 of E3 ubiquitin-protein ligase UBR4	IPI00640981	-1.417	0.02156	16	17	10	8
3330	28S ribosomal protein S29, mitochondrial	IPI00018120	-1.422	0.02145	9	7	2	3
3331	Monocarboxylate transporter 4	IPI00006666	-1.427	0.02141	10	13	5	5
3332	14-3-3 protein epsilon	IPI00000816	-1.430	0.02141	141	121	122	108
3333	Eukaryotic translation initiation factor 3 subunit D	IPI00006181	-1.435	0.02126	9	10	3	4
3334	Isoform 1 of Lipopolysaccharide-responsive and beige-like anchor protein	IPI00002255	-1.437	0.02119	31	28	24	16
3335	Eukaryotic peptide chain release factor subunit 1	IPI00429191	-1.438	0.02119	14	13	8	5
3336	Isoform 2 of Heat shock protein HSP 90-alpha	IPI00382470	-1.440	0.02119	122	112	102	101
3337	Histone-binding protein RBBP4	IPI00328319	-1.461	0.02096	12	10	7	2
3338	40S ribosomal protein S15	IPI00479058	-1.481	0.01959	24	17	11	13
3339	Isoform 1 of DNA (cytosine-5)-methyltransferase 1	IPI00031519	-1.503	0.01925	16	18	11	7
3340	Isoform 2 of Nucleophosmin	IPI00220740	-1.508	0.01918	65	46	47	39
3341	Isoform ASF-1 of Splicing factor, arginine/serine-rich 1	IPI00215884	-1.537	0.01851	34	40	27	25
3342	vacuolar protein sorting-associated protein 13C isoform 2B	IPI00412216	-1.597	0.01720	8	8	0	2
3343	Carbonic anhydrase 1	IPI00215983	-1.597	0.01720	14	0	0	0
3344	cDNA FLJ25678 fis, clone TST04067, highly similar to PURINE NUCLEOSIDE PHOSPHORYLASE	IPI00017672	-1.604	0.01601	42	33	25	27
3345	Isoform A of Phosphate carrier protein, mitochondrial	IPI00022202	-1.613	0.01590	29	29	19	18
3346	Cleavage and polyadenylation specificity factor subunit 5	IPI00646917	-1.658	0.01530	17	15	8	7
3347	Cofilin-1	IPI00012011	-1.669	0.01519	62	43	53	25
3348	40S ribosomal protein S3a	IPI00419880	-1.720	0.01441	23	11	10	6
3349	Putative uncharacterized protein KIAA0664	IPI00024425	-1.720	0.01441	18	16	9	7
3350	Malate dehydrogenase, mitochondrial	IPI00291006	-1.767	0.01374	52	48	37	35
3351	X-ray repair cross-complementing protein 6	IPI00644712	-1.774	0.01367	67	32	33	38
3352	Annexin A3	IPI00024095	-1.790	0.01333	32	32	21	19
3353	Isoform A of AP-1 complex subunit beta-1	IPI00328257	-1.811	0.01288	17	10	5	5
3354	Nuclease-sensitive element-binding protein 1	IPI00031812	-1.959	0.01005	17	13	8	3
3355	Isoform 1 of Exportin-2	IPI00022744	-1.961	0.00998	59	18	20	29
3356	Isoform 1 of Proteasome activator complex subunit 3	IPI00030243	-2.274	0.00704	27	28	13	14
3357	Keratin, type II cytoskeletal 1	IPI00220327	-3.378	0.00261	213	133	146	119
