# Supplementary material for: Data supporting the identification of anti-metastatic drug and natural compound targets in isogenic colorectal cancer cells
Source: Data Brief. 2014 Nov 4;1:73–5. doi: 10.1016/j.dib.2014.10.005 (PMC4459770; doi:10.1016/j.dib.2014.10.005)
Supplement: Supplementary file 1 — Supplementary data [file mmc1.zip › CRC_Metastasis_DIB_Table05.pdf]

Table 5. List of proteins differentially expressed in SW620 by the treatment of ginsenoside 20(S)-Rg3 for 48hr. ([1]STN and p-value were acquired from PLGEM analysis, [2]Raw spectral counts from data compilation using ScaffoldTM software)

| No. | Description | Accession number | STN[1] | p-Value[1] | Con_A[2] | Con_B[2] | RG3_A[2] | RG3_B[2] |
|---|---|---|---|---|---|---|---|---|
| 1 | Keratin, type II cytoskeletal 1 | IPI00220327 | 14.099 | 0.00000 | 198 | 213 | 438 | 386 |
| 2 | Keratin, type I cytoskeletal 9 | IPI00019359 | 8.523 | 0.00004 | 22 | 28 | 97 | 98 |
| 3 | Keratin, type I cytoskeletal 10 | IPI00009865 | 8.259 | 0.00004 | 36 | 55 | 128 | 122 |
| 4 | Keratin, type II cytoskeletal 8 | IPI00554648 | 6.953 | 0.00008 | 336 | 296 | 463 | 387 |
| 5 | Peptidyl-prolyl cis-trans isomerase A | IPI00419585 | 6.726 | 0.00008 | 97 | 126 | 197 | 184 |
| 6 | Isoform 1 of Nucleoside diphosphate kinase B | IPI00026260 | 6.358 | 0.00008 | 32 | 15 | 93 | 56 |
| 7 | Galectin-1 | IPI00219219 | 6.148 | 0.00013 | 0 | 0 | 35 | 33 |
| 8 | Isoform M1 of Pyruvate kinase isozymes M1/M2 | IPI00220644 | 5.229 | 0.00017 | 343 | 274 | 400 | 378 |
| 9 | Keratin, type II cytoskeletal 2 epiderma | IPI00021304 | 5.214 | 0.00017 | 28 | 18 | 66 | 61 |
| 10 | Isoform 2 of Eukaryotic translation initiation factor 5A-1 | IPI00376005 | 5.112 | 0.00017 | 75 | 50 | 129 | 97 |
| 11 | Tubulin alpha-4A chain | IPI00007750 | 4.704 | 0.00025 | 295 | 248 | 367 | 315 |
| 12 | ATP synthase subunit beta, mitochondrial | IPI00303476 | 4.685 | 0.00025 | 129 | 114 | 192 | 161 |
| 13 | Isoform 1 of Clathrin heavy chain 1 | IPI00024067 | 4.633 | 0.00029 | 198 | 222 | 259 | 288 |
| 14 | Elongation factor 1-alpha 2 | IPI00014424 | 4.592 | 0.00029 | 552 | 565 | 678 | 607 |
| 15 | Isoform B1 of Heterogeneous nuclear ribonucleoproteins A2/B1 | IPI00396378 | 4.543 | 0.00029 | 246 | 217 | 316 | 275 |
| 16 | Prohibitin-2 | IPI00027252 | 4.283 | 0.00038 | 69 | 65 | 121 | 98 |
| 17 | ADP/ATP translocase 2 | IPI00007188 | 3.641 | 0.00084 | 201 | 220 | 278 | 242 |
| 18 | Alpha-actinin-1 | IPI00013508 | 3.590 | 0.00088 | 166 | 118 | 197 | 174 |
| 19 | Glyceraldehyde-3-phosphate dehydrogenase | IPI00219018 | 3.450 | 0.00113 | 538 | 507 | 584 | 584 |
| 20 | 60S ribosomal protein L10a | IPI00412579 | 3.441 | 0.00113 | 22 | 11 | 35 | 45 |
| 21 | 60 kDa heat shock protein, mitochondrial | IPI00784154 | 3.384 | 0.00125 | 455 | 339 | 452 | 453 |
| 22 | probable E3 ubiquitin-protein ligase MYCBP2 | IPI00289776 | 3.349 | 0.00130 | 3 | 4 | 13 | 27 |
| 23 | Isoform 1 of Adenylyl cyclase-associated protein 1 | IPI00008274 | 3.333 | 0.00134 | 38 | 24 | 62 | 53 |
| 24 | Peroxiredoxin-6 | IPI00220301 | 3.124 | 0.00171 | 45 | 48 | 74 | 74 |
| 25 | Prohibitin | IPI00017334 | 3.052 | 0.00188 | 85 | 81 | 111 | 118 |
| 26 | Phosphoglycerate kinase 1 | IPI00169383 | 2.986 | 0.00197 | 130 | 93 | 171 | 119 |
| 27 | Protein disulfide-isomerase A3 | IPI00025252 | 2.912 | 0.00197 | 61 | 61 | 119 | 58 |
| 28 | Isoform 1 of Keratin, type I cytoskeletal 13 | IPI00009866 | 2.890 | 0.00201 | 14 | 20 | 36 | 37 |
| 29 | cDNA FLJ54957, highly similar to Transketolase | IPI00643920 | 2.871 | 0.00209 | 62 | 36 | 80 | 69 |
| 30 | Profilin-1 | IPI00216691 | 2.870 | 0.00209 | 142 | 142 | 187 | 166 |
| 31 | NAD(P) transhydrogenase, mitochondrial | IPI00337541 | 2.761 | 0.00255 | 63 | 59 | 90 | 84 |
| 32 | 60S ribosomal protein L23 | IPI00010153 | 2.758 | 0.00255 | 55 | 44 | 79 | 69 |
| 33 | 482 kDa protein | IPI00179298 | 2.747 | 0.00259 | 124 | 119 | 149 | 157 |
| 34 | Complement component 1 Q subcomponent-binding protein, mitochondrial | IPI00014230 | 2.747 | 0.00259 | 120 | 74 | 141 | 112 |
| 35 | Isoform 1 of Nucleoside diphosphate kinase A | IPI00012048 | 2.726 | 0.00263 | 60 | 29 | 86 | 50 |
| 36 | Estradiol 17-beta-dehydrogenase 12 | IPI00007676 | 2.602 | 0.00314 | 39 | 38 | 68 | 52 |
| 37 | Tubulin, beta | IPI00645452 | 2.548 | 0.00335 | 83 | 60 | 110 | 83 |
| 38 | Isoform 3 of Adenylate kinase 2, mitochondrial | IPI00172460 | 2.532 | 0.00339 | 26 | 18 | 38 | 42 |
| 39 | Dolichyl-diphosphooligosaccharide--protein glycosyltransferase subunit 2 | IPI00028635 | 2.472 | 0.00343 | 85 | 98 | 120 | 115 |
| 40 | Glucose-6-phosphate isomerase | IPI00027497 | 2.464 | 0.00347 | 55 | 40 | 71 | 67 |
| 41 | Isoform 1 of Heterogeneous nuclear ribonucleoprotein K | IPI00216049 | 2.431 | 0.00355 | 128 | 93 | 155 | 120 |
| 42 | Single-stranded DNA-binding protein, mitochondrial | IPI00029744 | 2.428 | 0.00360 | 27 | 15 | 48 | 28 |
| 43 | Isoform 4 of E3 ubiquitin-protein ligase UBR4 | IPI00640981 | 2.360 | 0.00389 | 17 | 12 | 24 | 35 |
| 44 | T-complex protein 1 subunit beta | IPI00297779 | 2.356 | 0.00389 | 126 | 77 | 146 | 108 |
| 45 | Isoform 2 of U5 small nuclear ribonucleoprotein 200 kDa helicase | IPI00168235 | 2.354 | 0.00397 | 44 | 43 | 65 | 62 |
| 46 | 40S ribosomal protein S5 | IPI00008433 | 2.287 | 0.00410 | 36 | 12 | 54 | 27 |
| 47 | Tubulin beta-2C chain | IPI00007752 | 2.270 | 0.00414 | 243 | 202 | 307 | 200 |
| 48 | Isoform 1 of Surfeit locus protein 4 | IPI00005737 | 2.227 | 0.00435 | 11 | 10 | 24 | 23 |
| 49 | Calreticulin | IPI00020599 | 2.227 | 0.00435 | 48 | 50 | 77 | 60 |
| 50 | Isoform 5 of Glycogen debranching enzyme | IPI00219065 | 2.222 | 0.00435 | 8 | 10 | 17 | 26 |
| 51 | Lupus La protein | IPI00009032 | 2.217 | 0.00435 | 59 | 50 | 83 | 66 |
| 52 | Keratin, type I cytoskeletal 16 | IPI00217963 | 2.197 | 0.00435 | 2 | 2 | 12 | 10 |
| 53 | Isoform 1 of Carnitine O-palmitoyltransferase 1, liver isoform | IPI00032038 | 2.183 | 0.00443 | 11 | 12 | 17 | 32 |
| 54 | 6-phosphogluconate dehydrogenase, decarboxylating | IPI00219525 | 2.172 | 0.00443 | 66 | 62 | 91 | 78 |
| 55 | Isoform 1 of 3-hydroxyacyl-CoA dehydrogenase type-2 | IPI00017726 | 2.162 | 0.00443 | 35 | 32 | 52 | 49 |
| 56 | Gamma-enolase | IPI00216171 | 2.136 | 0.00464 | 75 | 61 | 93 | 84 |
| 57 | Isoform 1 of Polypyrimidine tract-binding protein 1 | IPI00179964 | 2.112 | 0.00464 | 87 | 67 | 101 | 95 |
| 58 | Endoplasmic reticulum metallopeptidase 1 | IPI00257903 | 2.094 | 0.00473 | 2 | 2 | 5 | 16 |
| 59 | 14-3-3 protein zeta/delta | IPI00021263 | 2.073 | 0.00477 | 67 | 39 | 96 | 47 |
| 60 | Isoform 1 of Ubiquitin-conjugating enzyme E2 variant 1 | IPI00019599 | 2.072 | 0.00477 | 12 | 9 | 23 | 22 |
| 61 | Thioredoxin | IPI00216298 | 2.062 | 0.00477 | 8 | 10 | 23 | 18 |
| 62 | T-complex protein 1 subunit zeta | IPI00027626 | 2.044 | 0.00485 | 93 | 66 | 104 | 96 |
| 63 | T-complex protein 1 subunit delta | IPI00302927 | 2.036 | 0.00485 | 46 | 38 | 61 | 57 |
| 64 | Putative uncharacterized protein ATP5J2 | IPI00219291 | 2.032 | 0.00485 | 5 | 11 | 16 | 22 |
| 65 | Delta(3,5)-Delta(2,4)-dienoyl-CoA isomerase, mitochondrial | IPI00011416 | 2.026 | 0.00485 | 35 | 34 | 53 | 48 |
| 66 | Cofilin-1 | IPI00012011 | 1.991 | 0.00493 | 264 | 260 | 310 | 271 |
| 67 | ATP synthase subunit alpha, mitochondrial | IPI00440493 | 1.979 | 0.00506 | 86 | 78 | 117 | 87 |
| 68 | Malate dehydrogenase, mitochondrial | IPI00291006 | 1.954 | 0.00523 | 84 | 60 | 108 | 74 |
| 69 | Isoform 1 of L-lactate dehydrogenase A chain | IPI00217966 | 1.953 | 0.00527 | 287 | 240 | 306 | 277 |
| 70 | Dolichyl-diphosphooligosaccharide--protein glycosyltransferase subunit STT3B | IPI00152377 | 1.940 | 0.00531 | 14 | 14 | 21 | 31 |
| 71 | Estradiol 17-beta-dehydrogenase 11 | IPI00329598 | 1.935 | 0.00539 | 8 | 12 | 17 | 25 |
| 72 | Nodal modulator 1 | IPI00329352 | 1.919 | 0.00544 | 8 | 6 | 13 | 21 |
| 73 | Protein DJ-1 | IPI00298547 | 1.893 | 0.00577 | 16 | 15 | 23 | 32 |
| 74 | 2-oxoglutarate dehydrogenase, mitochondrial | IPI00098902 | 1.893 | 0.00581 | 7 | 5 | 14 | 17 |
| 75 | Histone H2B type 2-E | IPI00003935 | 1.886 | 0.00581 | 71 | 52 | 77 | 81 |
| 76 | Protein mago nashi homolog 2 | IPI00059292 | 1.875 | 0.00581 | 14 | 9 | 28 | 17 |
| 77 | Isoform 1 of Enhancer of mRNA-decapping protein 4 | IPI00376317 | 1.872 | 0.00581 | 4 | 6 | 12 | 16 |
| 78 | Serine/threonine-protein kinase mTOR | IPI00031410 | 1.859 | 0.00598 | 4 | 4 | 9 | 16 |
| 79 | cDNA FLJ40024 fis, clone STOMA2007745, highly similar to UBIQUITIN-ACTIVATING ENZYME E1 | IPI00026119 | 1.853 | 0.00602 | 73 | 58 | 90 | 76 |
| 80 | Translocon-associated protein subunit delta precursor | IPI00019385 | 1.835 | 0.00606 | 13 | 8 | 23 | 19 |
| 81 | Isoform 2 of Voltage-dependent anion-selective channel protein 2 | IPI00024145 | 1.825 | 0.00631 | 22 | 20 | 32 | 35 |
| 82 | Isoform 1 of Host cell factor 1 | IPI00019848 | 1.816 | 0.00640 | 2 | 3 | 10 | 10 |
| 83 | Lamin-B receptor | IPI00292135 | 1.813 | 0.00640 | 20 | 17 | 28 | 33 |
| 84 | Voltage-dependent anion-selective channel protein 1 | IPI00216308 | 1.799 | 0.00652 | 87 | 73 | 101 | 95 |
| 85 | Isoform 1 of Lipopolysaccharide-responsive and beige-like anchor protein | IPI00002255 | 1.774 | 0.00723 | 31 | 31 | 37 | 52 |
| 86 | TC4 protein | IPI00044779 | 1.754 | 0.00744 | 22 | 14 | 29 | 30 |
| 87 | retinol-binding protein 1 isoform a | IPI00219718 | 1.754 | 0.00744 | 25 | 24 | 42 | 32 |
| 88 | Isoform alpha-enolase of Alpha-enolase | IPI00465248 | 1.740 | 0.00749 | 370 | 315 | 398 | 341 |
| 89 | Isoform M2 of Pyruvate kinase isozymes M1/M2 | IPI00479186 | 1.740 | 0.00749 | 80 | 54 | 85 | 82 |
| 90 | Eukaryotic translation initiation factor 1A, Y-chromosomal | IPI00023004 | 1.719 | 0.00761 | 35 | 26 | 45 | 42 |
| 91 | Microsomal glutathione S-transferase 3 | IPI00024266 | 1.715 | 0.00761 | 15 | 13 | 20 | 29 |

| No. | Description | Accession number | STN[1] | p-Value[1] | Con_A[2] | Con_B[2] | RG3_A[2] | RG3_B[2] |
|---|---|---|---|---|---|---|---|---|
| 92 | N(G),N(G)-dimethylarginine dimethylaminohydrolase 2 | IPI00000760 | 1.709 | 0.00761 | 3 | 4 | 11 | 11 |
| 93 | Cleavage and polyadenylation specificity factor subunit 1 | IPI00026219 | 1.686 | 0.00799 | 4 | 6 | 7 | 19 |
| 94 | Isoform 1 of Protein SET | IPI00072377 | 1.677 | 0.00828 | 59 | 39 | 71 | 56 |
| 95 | Isoform Long of Inositol 1,4,5-trisphosphate receptor type 2 | IPI00031545 | 1.665 | 0.00845 | 56 | 57 | 58 | 85 |
| 96 | Plastin-2 | IPI00010471 | 1.623 | 0.00895 | 93 | 61 | 90 | 96 |
| 97 | Isoform Mitochondrial of Peroxiredoxin-5, mitochondrial | IPI00024915 | 1.611 | 0.00903 | 13 | 17 | 25 | 25 |
| 98 | Echinoderm microtubule-associated protein-like 4 | IPI00001466 | 1.609 | 0.00912 | 5 | 2 | 7 | 14 |
| 99 | SUMO-conjugating enzyme UBC9 | IPI00032957 | 1.609 | 0.00912 | 3 | 4 | 12 | 9 |
| 100 | Non-POU domain-containing octamer-binding protein | IPI00304596 | 1.599 | 0.00912 | 66 | 50 | 86 | 59 |
| 101 | Collapsin response mediator protein 4 long variant | IPI00029111 | 1.589 | 0.00937 | 22 | 16 | 28 | 31 |
| 102 | Heat shock 70 kDa protein 4 | IPI00002966 | 1.571 | 0.00962 | 36 | 38 | 41 | 58 |
| 103 | Inorganic pyrophosphatase | IPI00015018 | 1.560 | 0.01004 | 75 | 67 | 96 | 76 |
| 104 | Nucleolar protein 9 | IPI00002902 | 1.559 | 0.01004 | 2 | 2 | 3 | 13 |
| 105 | Isoform 1 of 26S proteasome non-ATPase regulatory subunit 1 | IPI00299608 | 1.549 | 0.01054 | 21 | 21 | 31 | 32 |
| 106 | Isoform 2 of AP-3 complex subunit delta-1 | IPI00289608 | 1.530 | 0.01071 | 4 | 5 | 7 | 16 |
| 107 | Thioredoxin domain-containing protein 12 | IPI00026328 | 1.530 | 0.01071 | 4 | 5 | 12 | 11 |
| 108 | Cytoplasmic dynein 1 heavy chain 1 | IPI00456969 | 1.529 | 0.01071 | 343 | 324 | 315 | 399 |
| 109 | Ubiquitin carboxyl-terminal hydrolase 14 | IPI00219913 | 1.528 | 0.01071 | 15 | 10 | 24 | 19 |
| 110 | Glutamate dehydrogenase 1, mitochondrial | IPI00016801 | 1.512 | 0.01087 | 22 | 24 | 35 | 32 |
| 111 | Isoform 2 of Neutral alpha-glucosidase AB | IPI00011454 | 1.510 | 0.01100 | 84 | 75 | 87 | 102 |
| 112 | SH3 domain-binding glutamic acid-rich-like protein | IPI00025318 | 1.507 | 0.01104 | 2 | 5 | 9 | 11 |
| 113 | 60S ribosomal protein L31 | IPI00026302 | 1.505 | 0.01104 | 9 | 4 | 18 | 10 |
| 114 | Tyrosyl-tRNA synthetase, cytoplasmic | IPI00007074 | 1.499 | 0.01117 | 18 | 9 | 22 | 23 |
| 115 | Isoform 1 of Alpha-aminoadipic semialdehyde dehydrogenase | IPI00221234 | 1.499 | 0.01117 | 13 | 14 | 21 | 24 |
| 116 | E3 ubiquitin-protein ligase UBR5 | IPI00026320 | 1.480 | 0.01146 | 7 | 7 | 12 | 17 |
| 117 | Small nuclear ribonucleoprotein E | IPI00029266 | 1.479 | 0.01146 | 8 | 15 | 23 | 17 |
| 118 | Prostaglandin E synthase 3 | IPI00015029 | 1.466 | 0.01163 | 23 | 13 | 26 | 29 |
| 119 | Endoplasmin | IPI00027230 | 1.453 | 0.01175 | 84 | 60 | 93 | 79 |
| 120 | Histidine triad nucleotide-binding protein 1 | IPI00239077 | 1.439 | 0.01209 | 6 | 6 | 14 | 12 |
| 121 | Dihydropyrimidinase-related protein 2 | IPI00257508 | 1.436 | 0.01238 | 18 | 14 | 26 | 24 |
| 122 | Small nuclear ribonucleoprotein Sm D1 | IPI00302850 | 1.429 | 0.01242 | 74 | 61 | 96 | 66 |
| 123 | HMT1 hnRNP methyltransferase-like 2 isoform 1 | IPI00018522 | 1.425 | 0.01246 | 16 | 17 | 25 | 26 |
| 124 | Cytochrome c oxidase subunit 4 isoform 1, mitochondrial | IPI00006579 | 1.425 | 0.01246 | 16 | 17 | 26 | 25 |
| 125 | Isoform 1 of Trans-2,3-enoyl-CoA reductase | IPI00100656 | 1.416 | 0.01250 | 9 | 8 | 13 | 19 |
| 126 | Glycogen phosphorylase, liver form | IPI00783313 | 1.398 | 0.01280 | 12 | 11 | 16 | 23 |
| 127 | Signal recognition particle 54 kDa protein | IPI00009822 | 1.397 | 0.01280 | 7 | 11 | 15 | 18 |
| 128 | Isoform 2 of ATPase family AAA domain-containing protein 3A | IPI00295992 | 1.390 | 0.01288 | 8 | 6 | 11 | 17 |
| 129 | Proteasome subunit beta type-4 | IPI00555956 | 1.390 | 0.01301 | 27 | 26 | 33 | 40 |
| 130 | Isoform 1 of ATPase family AAA domain-containing protein 1 | IPI00171445 | 1.389 | 0.01301 | 0 | 3 | 8 | 8 |
| 131 | Isoform 1 of U2-associated protein SR140 | IPI00143753 | 1.367 | 0.01359 | 3 | 5 | 7 | 13 |
| 132 | Phospholipase A-2-activating protein | IPI00218465 | 1.362 | 0.01368 | 11 | 9 | 17 | 18 |
| 133 | Cytochrome c | IPI00465315 | 1.346 | 0.01418 | 8 | 13 | 22 | 14 |
| 134 | Keratin, type I cytoskeletal 19 | IPI00479145 | 1.344 | 0.01418 | 150 | 113 | 179 | 115 |
| 135 | T-complex protein 1 subunit alpha | IPI00290566 | 1.341 | 0.01418 | 67 | 46 | 65 | 72 |
| 136 | Isoform 1 of Nuclear autoantigenic sperm protein | IPI00179953 | 1.340 | 0.01418 | 14 | 20 | 21 | 30 |
| 137 | Talin-1 | IPI00298994 | 1.336 | 0.01422 | 100 | 92 | 105 | 115 |
| 138 | Bifunctional aminoacyl-tRNA synthetase | IPI00013452 | 1.333 | 0.01430 | 49 | 51 | 57 | 66 |
| 139 | Keratin, type I cytoskeletal 14 | IPI00384444 | 1.332 | 0.01430 | 0 | 0 | 9 | 5 |
| 140 | 7-dehydrocholesterol reductase | IPI00294501 | 1.331 | 0.01598 | 10 | 12 | 18 | 19 |
| 141 | Isoform 1 of Filamin-B | IPI00289334 | 1.329 | 0.01606 | 51 | 50 | 57 | 67 |
| 142 | Isoform 1 of Tensin-3 | IPI00658152 | 1.329 | 0.01606 | 9 | 8 | 10 | 21 |
| 143 | 26 kDa protein | IPI00219685 | 1.322 | 0.01618 | 6 | 7 | 11 | 15 |
| 144 | 26S protease regulatory subunit 7 | IPI00021435 | 1.311 | 0.01635 | 11 | 7 | 15 | 17 |
| 145 | Eukaryotic translation initiation factor 5B | IPI00299254 | 1.310 | 0.01660 | 30 | 25 | 40 | 34 |
| 146 | Isoform 1 of WD repeat-containing protein 1 | IPI00746165 | 1.307 | 0.01664 | 13 | 17 | 23 | 23 |
| 147 | Isoform 1 of Oxysterol-binding protein 1 | IPI00024971 | 1.303 | 0.01664 | 5 | 5 | 9 | 13 |
| 148 | Isoform 1 of Pyruvate dehydrogenase E1 component subunit beta, mitochondrial | IPI00003925 | 1.302 | 0.01694 | 10 | 14 | 18 | 21 |
| 149 | Citrate synthase, mitochondrial | IPI00025366 | 1.287 | 0.01719 | 45 | 38 | 53 | 51 |
| 150 | Isoform 2 of Cytoplasmic FMR1-interacting protein 1 | IPI00550212 | 1.276 | 0.01761 | 4 | 7 | 11 | 12 |
| 151 | Dihydrolipoyl dehydrogenase, mitochondrial | IPI00015911 | 1.275 | 0.01761 | 16 | 17 | 25 | 24 |
| 152 | Isoform 1 of 6-phosphofructokinase, liver type | IPI00332371 | 1.264 | 0.01765 | 3 | 5 | 8 | 11 |
| 153 | Cytosolic purine 5'-nucleotidase | IPI00029054 | 1.251 | 0.01777 | 5 | 7 | 10 | 14 |
| 154 | AP-1 complex subunit gamma-1 isoform a | IPI00293396 | 1.241 | 0.01857 | 8 | 9 | 12 | 18 |
| 155 | Isoform 1 of Myosin-10 | IPI00397526 | 1.238 | 0.01869 | 62 | 67 | 71 | 81 |
| 156 | Putative heat shock protein HSP 90-alpha A2 | IPI00031523 | 1.234 | 0.01874 | 115 | 107 | 132 | 117 |
| 157 | Small nuclear ribonucleoprotein Sm D2 | IPI00017963 | 1.234 | 0.01874 | 16 | 7 | 22 | 15 |
| 158 | arylacetamide deacetylase-like 1 isoform b | IPI00002230 | 1.230 | 0.01903 | 14 | 16 | 17 | 28 |
| 159 | Isoform 1 of ATP-binding cassette sub-family D member 3 | IPI00002372 | 1.228 | 0.01911 | 8 | 5 | 9 | 16 |
| 160 | Beta-hexosaminidase subunit beta | IPI00012585 | 1.216 | 0.01945 | 2 | 0 | 7 | 6 |
| 161 | Sorting and assembly machinery component 50 homolog | IPI00412713 | 1.216 | 0.01945 | 2 | 0 | 6 | 7 |
| 162 | NAD(P)H dehydrogenase [quinone] 1 | IPI00012069 | 1.208 | 0.01957 | 68 | 53 | 80 | 63 |
| 163 | Isoform Beta of Lamina-associated polypeptide 2, isoforms beta/gamma | IPI00030131 | 1.208 | 0.01957 | 13 | 6 | 22 | 10 |
| 164 | ATP synthase subunit O, mitochondrial | IPI00007611 | 1.204 | 0.01974 | 3 | 7 | 9 | 12 |
| 165 | Isoform 2 of Myosin-Ic | IPI00010418 | 1.204 | 0.01974 | 4 | 6 | 9 | 12 |
| 166 | Vacuolar protein sorting-associated protein 35 | IPI00018931 | 1.204 | 0.01974 | 20 | 21 | 22 | 35 |
| 167 | Kinetochore-associated protein 1 | IPI00001458 | 1.194 | 0.02016 | 3 | 4 | 5 | 12 |
| 168 | Isoform 1 of Cell division cycle and apoptosis regulator protein 1 | IPI00217357 | 1.192 | 0.02016 | 11 | 9 | 15 | 18 |
| 169 | Glutathione S-transferase P | IPI00219757 | 1.192 | 0.02028 | 194 | 158 | 204 | 178 |
| 170 | Isoform Long of Sodium/potassium-transporting ATPase subunit alpha-1 | IPI00006482 | 1.187 | 0.02032 | 57 | 53 | 62 | 69 |
| 171 | Coatomer subunit beta' | IPI00220219 | 1.183 | 0.02032 | 33 | 33 | 42 | 42 |
| 172 | 3-mercaptopyruvate sulfurtransferase | IPI00165360 | 1.179 | 0.02041 | 7 | 4 | 12 | 10 |
| 173 | Isocitrate dehydrogenase [NADP] cytoplasmic | IPI00027223 | 1.179 | 0.02041 | 6 | 5 | 13 | 9 |
| 174 | Deoxyribonucleoside 5'-monophosphate N-glycosidase | IPI00007926 | 1.169 | 0.02066 | 10 | 6 | 12 | 16 |
| 175 | Isoform A of Nucleoporin SEH1 | IPI00185533 | 1.165 | 0.02083 | 2 | 3 | 6 | 8 |
| 176 | Stathmin | IPI00479997 | 1.164 | 0.02083 | 13 | 9 | 19 | 16 |
| 177 | Thiosulfate sulfurtransferase | IPI00216293 | 1.164 | 0.02083 | 10 | 12 | 14 | 21 |
| 178 | Trifunctional enzyme subunit alpha, mitochondrial | IPI00031522 | 1.163 | 0.02087 | 35 | 35 | 42 | 46 |
| 179 | cDNA FLJ53927, highly similar to Beta-hexosaminidase alpha chain | IPI00027851 | 1.161 | 0.02087 | 0 | 6 | 7 | 11 |
| 180 | Destrin | IPI00473014 | 1.154 | 0.02166 | 43 | 29 | 51 | 39 |
| 181 | protein arginine N-methyltransferase 5 isoform b | IPI00064328 | 1.151 | 0.02187 | 15 | 8 | 16 | 20 |
| 182 | proteasome 26S non-ATPase subunit 8 | IPI00010201 | 1.148 | 0.02196 | 22 | 17 | 30 | 24 |
| 183 | Dolichyl-diphosphooligosaccharide--protein glycosyltransferase 48 kDa subunit | IPI00297084 | 1.148 | 0.02196 | 16 | 23 | 29 | 25 |
| 184 | Leucine-rich PPR motif-containing protein, mitochondrial | IPI00783271 | 1.146 | 0.02196 | 102 | 91 | 103 | 114 |
| 185 | ATP-binding cassette sub-family E member 1 | IPI00303207 | 1.141 | 0.02200 | 29 | 21 | 32 | 34 |
| 186 | Activating signal cointegrator 1 complex subunit 3 | IPI00430472 | 1.134 | 0.02233 | 4 | 9 | 9 | 15 |

| No. | Description | Accession number | STN[1] | p-Value[1] | Con_A[2] | Con_B[2] | RG3_A[2] | RG3_B[2] |
|---|---|---|---|---|---|---|---|---|
| 187 | Eukaryotic translation initiation factor 5A-2 | IPI00006935 | 1.132 | 0.02258 | 35 | 6 | 37 | 19 |
| 188 | 26S proteasome non-ATPase regulatory subunit 3 | IPI00011603 | 1.132 | 0.02258 | 21 | 20 | 25 | 31 |
| 189 | Succinyl-CoA:3-ketoacid-coenzyme A transferase 1, mitochondrial | IPI00026516 | 1.124 | 0.02267 | 15 | 18 | 25 | 22 |
| 190 | Very long-chain acyl-CoA synthetase | IPI00024787 | 1.123 | 0.02267 | 0 | 4 | 5 | 10 |
| 191 | Integrator complex subunit 2 | IPI00477759 | 1.123 | 0.02267 | 4 | 0 | 5 | 10 |
| 192 | Lysophospholipid acyltransferase 5 | IPI00306419 | 1.123 | 0.02267 | 2 | 4 | 7 | 8 |
| 193 | 40S ribosomal protein S16 | IPI00221092 | 1.122 | 0.02267 | 35 | 18 | 38 | 31 |
| 194 | Ubiquitin carboxyl-terminal hydrolase isozyme L3 | IPI00011250 | 1.121 | 0.02267 | 12 | 7 | 17 | 14 |
| 195 | Transaldolase | IPI00744692 | 1.110 | 0.02283 | 33 | 35 | 45 | 40 |
| 196 | Isoform 1 of Pyridoxal kinase | IPI00013004 | 1.106 | 0.02292 | 19 | 16 | 27 | 22 |
| 197 | T-complex protein 1 subunit eta | IPI00018465 | 1.106 | 0.02292 | 69 | 71 | 87 | 74 |
| 198 | Growth hormone inducible transmembrane protein | IPI00549970 | 1.104 | 0.02296 | 2 | 8 | 12 | 8 |
| 199 | Staphylococcal nuclease domain-containing protein 1 | IPI00140420 | 1.104 | 0.02334 | 56 | 45 | 54 | 66 |
| 200 | Isoform 1 of Minor histocompatibility antigen H13 | IPI00152441 | 1.097 | 0.02346 | 2 | 0 | 6 | 6 |
| 201 | Cytochrome c oxidase subunit 6C | IPI00015972 | 1.097 | 0.02346 | 0 | 0 | 5 | 7 |
| 202 | Putative uncharacterized protein CNOT1 | IPI00032299 | 1.097 | 0.02346 | 0 | 0 | 4 | 8 |
| 203 | Isoform 2 of U1 small nuclear ribonucleoprotein 70 kDa | IPI00219483 | 1.097 | 0.02346 | 2 | 2 | 6 | 6 |
| 204 | Peptidyl-prolyl cis-trans isomerase FKBP2 | IPI00002535 | 1.097 | 0.02346 | 2 | 2 | 7 | 5 |
| 205 | Isoform 1 of Pleiotropic regulator 1 | IPI00002624 | 1.097 | 0.02346 | 0 | 0 | 5 | 7 |
| 206 | Trifunctional enzyme subunit beta, mitochondrial | IPI00022793 | 1.093 | 0.02518 | 11 | 10 | 18 | 15 |
| 207 | 60S ribosomal protein L7a | IPI00299573 | 1.089 | 0.02518 | 31 | 28 | 38 | 37 |
| 208 | Isoform 2 of Phosphoglucomutase-1 | IPI00217872 | 1.087 | 0.02518 | 4 | 3 | 9 | 7 |
| 209 | Quinone oxidoreductase | IPI00000792 | 1.087 | 0.02518 | 3 | 4 | 7 | 9 |
| 210 | 26S proteasome non-ATPase regulatory subunit 12 | IPI00185374 | 1.084 | 0.02522 | 26 | 22 | 32 | 31 |
| 211 | ATP-dependent DNA helicase Q1 | IPI00178431 | 1.084 | 0.02526 | 15 | 14 | 16 | 26 |
| 212 | Splicing factor, arginine/serine-rich 2 | IPI00005978 | 1.080 | 0.02526 | 7 | 4 | 14 | 7 |
| 213 | Heterogeneous nuclear ribonucleoprotein U-like protein 2 | IPI00456887 | 1.080 | 0.02526 | 13 | 9 | 16 | 18 |
| 214 | Nuclear cap-binding protein subunit 1 | IPI00019380 | 1.078 | 0.02543 | 7 | 9 | 10 | 17 |
| 215 | Protein NipSnap homolog 1 | IPI00304435 | 1.078 | 0.02543 | 8 | 8 | 13 | 14 |
| 216 | Annexin A5 | IPI00329801 | 1.075 | 0.02543 | 40 | 36 | 52 | 41 |
| 217 | Isoform Long of Splicing factor, proline- and glutamine-rich | IPI00010740 | 1.074 | 0.02543 | 24 | 15 | 33 | 20 |
| 218 | Annexin A3 | IPI00024095 | 1.072 | 0.02543 | 19 | 31 | 31 | 34 |
| 219 | Importin subunit beta-1 | IPI00001639 | 1.069 | 0.02547 | 50 | 44 | 54 | 58 |
| 220 | Dihydropyrimidinase-like 2 | IPI00106642 | 1.060 | 0.02559 | 25 | 27 | 32 | 35 |
| 221 | Thioredoxin domain-containing protein 17 | IPI00646689 | 1.059 | 0.02564 | 6 | 6 | 10 | 12 |
| 222 | Galectin-3-binding protein | IPI00023673 | 1.059 | 0.02564 | 5 | 7 | 10 | 12 |
| 223 | Isoform 1 of ER lumen protein retaining receptor 2 | IPI00018248 | 1.056 | 0.02568 | 11 | 13 | 19 | 17 |
| 224 | Vesicular integral-membrane protein VIP36 | IPI00009950 | 1.055 | 0.02568 | 3 | 5 | 8 | 9 |
| 225 | 3-hydroxyisobutyrate dehydrogenase, mitochondrial | IPI00013860 | 1.055 | 0.02568 | 4 | 4 | 9 | 8 |
| 226 | Isoform Gamma-1 of Serine/threonine-protein phosphatase PP1-gamma catalytic subunit | IPI00005705 | 1.055 | 0.02568 | 28 | 25 | 37 | 31 |
| 227 | Ubiquitin-conjugating enzyme E2 N | IPI00003949 | 1.053 | 0.02568 | 19 | 23 | 36 | 20 |
| 228 | Proteasome activator complex subunit 1 | IPI00479722 | 1.051 | 0.02568 | 39 | 28 | 49 | 34 |
| 229 | Isoform 1 of Calcium-binding mitochondrial carrier protein SCaMC-1 | IPI00337494 | 1.049 | 0.02572 | 2 | 3 | 6 | 7 |
| 230 | Secernin-1 | IPI00289862 | 1.049 | 0.02572 | 0 | 3 | 8 | 5 |
| 231 | Alanyl-tRNA synthetase, cytoplasmic | IPI00027442 | 1.048 | 0.02572 | 74 | 69 | 74 | 89 |
| 232 | Gamma-glutamyl hydrolase | IPI00023728 | 1.046 | 0.02576 | 20 | 23 | 31 | 26 |
| 233 | Heterogeneous nuclear ribonucleoprotein F | IPI00003881 | 1.041 | 0.02589 | 51 | 52 | 67 | 54 |
| 234 | DNA replication licensing factor MCM5 | IPI00018350 | 1.039 | 0.02597 | 31 | 25 | 24 | 47 |
| 235 | ATP-dependent RNA helicase DDX1 | IPI00293655 | 1.033 | 0.02618 | 25 | 32 | 33 | 39 |
| 236 | Isoform 1 of Coatomer subunit alpha | IPI00295857 | 1.029 | 0.02622 | 54 | 53 | 64 | 61 |
| 237 | Villin-1 | IPI00218852 | 1.023 | 0.02630 | 31 | 28 | 37 | 37 |
| 238 | Protein tyrosine phosphatase-like protein PTPLAD1 | IPI00008998 | 1.023 | 0.02630 | 16 | 20 | 18 | 31 |
| 239 | Aspartyl-tRNA synthetase, cytoplasmic | IPI00216951 | 1.015 | 0.02676 | 24 | 24 | 29 | 33 |
| 240 | Mitochondrial carrier homolog 2 | IPI00003833 | 1.015 | 0.02676 | 30 | 18 | 37 | 25 |
| 241 | Protein transport protein Sec23A | IPI00017375 | 1.010 | 0.02702 | 2 | 4 | 4 | 10 |
| 242 | vacuolar protein sorting-associated protein 13C isoform 2B | IPI00412216 | 1.008 | 0.02823 | 22 | 16 | 22 | 29 |
| 243 | Isoform 1 of Methylcrotonoyl-CoA carboxylase beta chain, mitochondrial | IPI00784044 | 1.005 | 0.02827 | 15 | 14 | 16 | 25 |
| 244 | Isoform 2 of Protein disulfide-isomerase A6 | IPI00299571 | 0.996 | 0.02840 | 14 | 16 | 17 | 25 |
| 245 | Electron transfer flavoprotein subunit alpha, mitochondrial | IPI00010810 | 0.983 | 0.02911 | 38 | 30 | 47 | 36 |
| 246 | Isoform 1 of 60S ribosomal protein L11 | IPI00376798 | 0.982 | 0.02911 | 35 | 19 | 41 | 27 |
| 247 | annexin A4 | IPI00793199 | 0.982 | 0.02911 | 33 | 21 | 38 | 30 |
| 248 | EH domain-containing protein 1 | IPI00017184 | 0.981 | 0.02915 | 5 | 6 | 9 | 11 |
| 249 | Isocitrate dehydrogenase 3, beta subunit isoform a precursor | IPI00304417 | 0.977 | 0.02940 | 0 | 5 | 7 | 8 |
| 250 | Transitional endoplasmic reticulum ATPase | IPI00022774 | 0.976 | 0.02940 | 56 | 50 | 57 | 66 |
| 251 | regulator of differentiation 1 isoform 2 | IPI00159072 | 0.975 | 0.02940 | 0 | 0 | 4 | 7 |
| 252 | Transmembrane protein 14C | IPI00009346 | 0.975 | 0.02940 | 0 | 0 | 5 | 6 |
| 253 | Serine/threonine-protein kinase OSR1 | IPI00010080 | 0.975 | 0.02940 | 2 | 2 | 7 | 4 |
| 254 | Testis-expressed sequence 10 protein | IPI00549664 | 0.975 | 0.02940 | 0 | 0 | 6 | 5 |
| 255 | cDNA FLJ55574, highly similar to Calnexin | IPI00020984 | 0.974 | 0.02978 | 63 | 44 | 69 | 55 |
| 256 | Ubiquitin-like modifier activating enzyme 1 | IPI00552452 | 0.973 | 0.02978 | 13 | 11 | 21 | 14 |
| 257 | L-lactate dehydrogenase B chain | IPI00219217 | 0.971 | 0.02982 | 211 | 171 | 228 | 179 |
| 258 | tRNA (cytosine-5-)-methyltransferase NSUN2 | IPI00306369 | 0.962 | 0.02998 | 12 | 13 | 18 | 18 |
| 259 | NADH dehydrogenase [ubiquinone] iron-sulfur protein 2, mitochondrial | IPI00025239 | 0.962 | 0.02998 | 9 | 16 | 15 | 21 |
| 260 | T-complex protein 1 subunit gamma isoform b | IPI00290770 | 0.953 | 0.03053 | 55 | 39 | 54 | 56 |
| 261 | Cytoplasmic dynein 1 light intermediate chain 1 | IPI00007675 | 0.948 | 0.03070 | 4 | 4 | 7 | 9 |
| 262 | Probable cysteinyl-tRNA synthetase, mitochondrial | IPI00336016 | 0.948 | 0.03070 | 5 | 3 | 8 | 8 |
| 263 | Putative pre-mRNA-splicing factor ATP-dependent RNA helicase DHX15 | IPI00396435 | 0.944 | 0.03141 | 41 | 21 | 34 | 42 |
| 264 | Isoform Heart of ATP synthase subunit gamma, mitochondrial | IPI00395769 | 0.943 | 0.03145 | 15 | 12 | 23 | 15 |
| 265 | CAD protein | IPI00301263 | 0.942 | 0.03145 | 106 | 98 | 116 | 108 |
| 266 | Rho-associated protein kinase 2 | IPI00307155 | 0.942 | 0.03145 | 5 | 8 | 11 | 11 |
| 267 | Isoform 1 of ATP-dependent RNA helicase DDX19B | IPI00008943 | 0.940 | 0.03153 | 15 | 22 | 21 | 28 |
| 268 | Leucyl-tRNA synthetase, cytoplasmic | IPI00103994 | 0.939 | 0.03153 | 23 | 26 | 26 | 36 |
| 269 | Actin, aortic smooth muscle | IPI00008603 | 0.937 | 0.03157 | 86 | 61 | 90 | 75 |
| 270 | Activated RNA polymerase II transcriptional coactivator p15 | IPI00221222 | 0.933 | 0.03166 | 18 | 10 | 29 | 10 |
| 271 | Mitochondrial import inner membrane translocase subunit TIM44 | IPI00306516 | 0.932 | 0.03229 | 2 | 3 | 5 | 7 |
| 272 | Tyrosine-protein phosphatase non-receptor type 23 | IPI00034006 | 0.932 | 0.03229 | 0 | 3 | 5 | 7 |
| 273 | cDNA FLJ25678 fis, clone TST04067, highly similar to PURINE NUCLEOSIDE PHOSPHORYLASE | IPI00017672 | 0.929 | 0.03233 | 25 | 26 | 33 | 31 |
| 274 | Isoform 1 of Voltage-dependent anion-selective channel protein 3 | IPI00031804 | 0.927 | 0.03233 | 21 | 18 | 23 | 28 |
| 275 | Isoform 1 of Fermitin family homolog 2 | IPI00000856 | 0.925 | 0.03233 | 9 | 5 | 10 | 13 |
| 276 | Isoform 1 of ATP-binding cassette sub-family B member 7, mitochondrial | IPI00306748 | 0.925 | 0.03233 | 8 | 6 | 8 | 15 |
| 277 | 2,4-dienoyl-CoA reductase, mitochondrial | IPI00003482 | 0.925 | 0.03233 | 7 | 7 | 12 | 11 |
| 278 | 60S ribosomal protein L6 | IPI00329389 | 0.919 | 0.03258 | 44 | 24 | 53 | 29 |
| 279 | Protein disulfide-isomerase | IPI00010796 | 0.918 | 0.03262 | 41 | 45 | 54 | 47 |
| 280 | Peptidyl-prolyl cis-trans isomerase FKBP4 | IPI00219005 | 0.918 | 0.03262 | 44 | 42 | 51 | 50 |
| 281 | L-xylulose reductase | IPI00448095 | 0.909 | 0.03304 | 10 | 5 | 15 | 9 |

| No. | Description | Accession number | STN[1] | p-Value[1] | Con_A[2] | Con_B[2] | RG3_A[2] | RG3_B[2] |
|---|---|---|---|---|---|---|---|---|
| 282 | Isoform Short of Proteasome subunit alpha type-1 | IPI00016832 | 0.908 | 0.03304 | 14 | 17 | 25 | 17 |
| 283 | Serine hydroxymethyltransferase, mitochondrial | IPI00002520 | 0.901 | 0.03329 | 39 | 34 | 45 | 42 |
| 284 | Isoform 1 of Hydroxyacyl-coenzyme A dehydrogenase, mitochondrial | IPI00294398 | 0.900 | 0.03329 | 11 | 21 | 18 | 25 |
| 285 | baculoviral IAP repeat-containing protein 6 | IPI00299635 | 0.900 | 0.03329 | 6 | 4 | 7 | 11 |
| 286 | lanosterol 14-alpha demethylase isoform 1 | IPI00295772 | 0.896 | 0.03417 | 3 | 3 | 5 | 8 |
| 287 | Membrane-associated progesterone receptor component 1 | IPI00220739 | 0.896 | 0.03417 | 3 | 3 | 9 | 4 |
| 288 | Transcription factor BTF3 homolog 4 | IPI00412792 | 0.896 | 0.03417 | 0 | 4 | 4 | 9 |
| 289 | Isoform 1 of Acetyl-CoA carboxylase 1 | IPI00011569 | 0.895 | 0.03442 | 31 | 27 | 28 | 43 |
| 290 | Keratin, type II cytoskeletal 75 | IPI00005859 | 0.894 | 0.03442 | 8 | 8 | 15 | 10 |
| 291 | magnesium transporter protein 1 | IPI00301202 | 0.894 | 0.03442 | 8 | 8 | 13 | 12 |
| 292 | F-actin-capping protein subunit alpha-1 | IPI00005969 | 0.893 | 0.03442 | 19 | 14 | 24 | 20 |
| 293 | Eukaryotic translation initiation factor 5 | IPI00022648 | 0.880 | 0.03496 | 7 | 10 | 12 | 14 |
| 294 | Ras GTPase-activating-like protein IQGAP1 | IPI00009342 | 0.878 | 0.03505 | 96 | 87 | 105 | 96 |
| 295 | Putative RNA-binding protein 3 | IPI00024320 | 0.870 | 0.03542 | 15 | 11 | 21 | 15 |
| 296 | Protein SCO1 homolog, mitochondrial | IPI00027233 | 0.865 | 0.03563 | 5 | 2 | 5 | 9 |
| 297 | Isoform 1 of Uncharacterized protein KIAA0528 | IPI00465142 | 0.865 | 0.03563 | 4 | 3 | 6 | 8 |
| 298 | perilipin-3 isoform 3 | IPI00106668 | 0.865 | 0.03563 | 0 | 5 | 6 | 8 |
| 299 | Isoform 1 of Nuclear pore membrane glycoprotein 210 | IPI00291755 | 0.864 | 0.03576 | 27 | 23 | 20 | 42 |
| 300 | Isoform 2 of Nucleophosmin | IPI00220740 | 0.864 | 0.03588 | 72 | 59 | 73 | 74 |
| 301 | Nucleoprotein TPR | IPI00742682 | 0.863 | 0.03588 | 38 | 28 | 33 | 46 |
| 302 | Isoform 1 of Abhydrolase domain-containing protein 14B | IPI00063827 | 0.861 | 0.03588 | 5 | 7 | 9 | 11 |
| 303 | Isoform 1 of Dynamin-2 | IPI00033022 | 0.861 | 0.03588 | 6 | 6 | 8 | 12 |
| 304 | Cystatin-B | IPI00021828 | 0.861 | 0.03588 | 5 | 7 | 8 | 12 |
| 305 | 60S ribosomal protein L27 | IPI00219155 | 0.861 | 0.03647 | 16 | 11 | 19 | 18 |
| 306 | Probable O-sialoglycoprotein endopeptidase | IPI00015809 | 0.851 | 0.03676 | 2 | 2 | 4 | 6 |
| 307 | Isoform 1 of CDP-diacylglycerol--inositol 3-phosphatidyltransferase | IPI00645518 | 0.851 | 0.03676 | 2 | 0 | 2 | 8 |
| 308 | HSR1 protein | IPI00384745 | 0.851 | 0.03676 | 2 | 2 | 5 | 5 |
| 309 | Isoform 1 of Serine/threonine-protein phosphatase 6 catalytic subunit | IPI00012970 | 0.851 | 0.03676 | 0 | 2 | 3 | 7 |
| 310 | 24-dehydrocholesterol reductase | IPI00016703 | 0.851 | 0.03676 | 0 | 0 | 0 | 8 |
| 311 | Myosin-11 | IPI00020501 | 0.851 | 0.03676 | 2 | 2 | 3 | 7 |
| 312 | Paired amphipathic helix protein Sin3a | IPI00170596 | 0.851 | 0.03676 | 0 | 0 | 3 | 7 |
| 313 | Calpain-1 catalytic subunit | IPI00011285 | 0.844 | 0.04224 | 15 | 14 | 17 | 22 |
| 314 | 59 kDa protein | IPI00302925 | 0.839 | 0.04228 | 53 | 40 | 70 | 37 |
| 315 | Transmembrane emp24 domain-containing protein 2 | IPI00016608 | 0.839 | 0.04228 | 3 | 5 | 8 | 7 |
| 316 | Mitochondrial glutamate carrier 1 | IPI00003004 | 0.839 | 0.04228 | 0 | 6 | 7 | 8 |
| 317 | Isoform 1 of 26S protease regulatory subunit 6B | IPI00020042 | 0.835 | 0.04307 | 15 | 27 | 22 | 31 |
| 318 | Acidic leucine-rich nuclear phosphoprotein 32 family member E | IPI00165393 | 0.833 | 0.04307 | 10 | 11 | 12 | 18 |
| 319 | 26S protease regulatory subunit 8 | IPI00023919 | 0.829 | 0.04312 | 14 | 17 | 23 | 18 |
| 320 | 6-phosphogluconolactonase | IPI00029997 | 0.829 | 0.04312 | 14 | 17 | 20 | 21 |
| 321 | Isoform A of Phosphate carrier protein, mitochondrial | IPI00022202 | 0.824 | 0.04379 | 32 | 27 | 45 | 26 |
| 322 | RuvB-like 2 | IPI00009104 | 0.816 | 0.04429 | 35 | 26 | 39 | 34 |
| 323 | Isoform 1AB of Catenin delta-1 | IPI00182469 | 0.816 | 0.04429 | 2 | 7 | 8 | 8 |
| 324 | Isoform 1 of Porphobilinogen deaminase | IPI00028160 | 0.816 | 0.04429 | 0 | 7 | 8 | 8 |
| 325 | 13kDa differentiation-associated protein variant (Fragment) | IPI00005966 | 0.816 | 0.04429 | 4 | 5 | 9 | 7 |
| 326 | UPF0556 protein C19orf10 | IPI00056357 | 0.816 | 0.04429 | 6 | 3 | 8 | 8 |
| 327 | Calmodulin | IPI00075248 | 0.816 | 0.04429 | 4 | 5 | 6 | 10 |
| 328 | 40S ribosomal protein S25 | IPI00012750 | 0.813 | 0.04429 | 15 | 8 | 16 | 16 |
| 329 | Aspartate aminotransferase, mitochondrial | IPI00018206 | 0.813 | 0.04433 | 40 | 41 | 47 | 47 |
| 330 | Protein flightless-1 homolog | IPI00031023 | 0.811 | 0.04437 | 3 | 2 | 2 | 9 |
| 331 | Flotillin-2 | IPI00789008 | 0.811 | 0.04437 | 2 | 3 | 3 | 8 |
| 332 | Isoform 2 of Triosephosphate isomerase | IPI00451401 | 0.810 | 0.04441 | 49 | 33 | 53 | 42 |
| 333 | Isoform 2 of Spliceosome RNA helicase BAT1 | IPI00641829 | 0.801 | 0.04466 | 15 | 20 | 25 | 20 |
| 334 | Isoform 1 of Transcription intermediary factor 1-beta | IPI00438229 | 0.801 | 0.04466 | 42 | 43 | 50 | 48 |
| 335 | Isoform 4 of Heterogeneous nuclear ribonucleoprotein A/B | IPI00106509 | 0.800 | 0.04466 | 6 | 10 | 10 | 14 |
| 336 | Isoform 1 of Ubiquitin-like modifier-activating enzyme 6 | IPI00023647 | 0.800 | 0.04466 | 8 | 8 | 14 | 10 |
| 337 | Isoform Epsilon of Apoptosis regulator BAX | IPI00071059 | 0.800 | 0.04466 | 8 | 8 | 11 | 13 |
| 338 | Thioredoxin-dependent peroxide reductase, mitochondrial | IPI00024919 | 0.798 | 0.04521 | 33 | 33 | 45 | 33 |
| 339 | Vacuolar protein sorting-associated protein 26A | IPI00411426 | 0.795 | 0.04529 | 4 | 6 | 7 | 10 |
| 340 | Isoform 1 of Spermine synthase | IPI00005102 | 0.795 | 0.04529 | 5 | 5 | 6 | 11 |
| 341 | 60S ribosomal protein L18 | IPI00215719 | 0.795 | 0.04554 | 18 | 18 | 27 | 19 |
| 342 | Cytochrome b-c1 complex subunit 2, mitochondrial | IPI00305383 | 0.795 | 0.04588 | 10 | 15 | 15 | 19 |
| 343 | Ribosome maturation protein SBDS | IPI00427330 | 0.788 | 0.04596 | 8 | 9 | 8 | 17 |
| 344 | Isoform 1 of Low molecular weight phosphotyrosine protein phosphatase | IPI00219861 | 0.788 | 0.04596 | 9 | 8 | 15 | 10 |
| 345 | ATP synthase subunit b, mitochondrial | IPI00029133 | 0.781 | 0.04604 | 33 | 20 | 34 | 30 |
| 346 | Cell division protein kinase 5 | IPI00023530 | 0.779 | 0.04617 | 2 | 4 | 6 | 6 |
| 347 | Isoform 1 of BRCA2 and CDKN1A-interacting protein | IPI00002203 | 0.779 | 0.04617 | 2 | 4 | 4 | 8 |
| 348 | Dihydrolipoyllysine-residue acetyltransferase component of pyruvate dehydrogenase complex | IPI00021338 | 0.778 | 0.04784 | 12 | 15 | 14 | 22 |
| 349 | Protein BUD31 homolog | IPI00013180 | 0.777 | 0.04788 | 4 | 7 | 6 | 12 |
| 350 | 60S ribosomal protein L30 | IPI00219156 | 0.777 | 0.04788 | 5 | 6 | 9 | 9 |
| 351 | Omega-amidase NIT2 | IPI00549467 | 0.777 | 0.04788 | 7 | 4 | 10 | 8 |
| 352 | Cold-inducible RNA-binding protein | IPI00180954 | 0.776 | 0.04805 | 9 | 9 | 14 | 12 |
| 353 | Ubiquitin carboxyl-terminal hydrolase 24 | IPI00902614 | 0.765 | 0.04876 | 10 | 9 | 12 | 15 |
| 354 | Isoform 1 of Reticulon-4 | IPI00021766 | 0.760 | 0.04880 | 7 | 5 | 13 | 6 |
| 355 | DEAD (Asp-Glu-Ala-Asp) box polypeptide 39, isoform CRA_c | IPI00166874 | 0.751 | 0.04951 | 3 | 4 | 6 | 7 |
| 356 | Sterol-4-alpha-carboxylate 3-dehydrogenase, decarboxylating | IPI00019407 | 0.751 | 0.04951 | 2 | 5 | 4 | 9 |
| 357 | Non-functional aryl hydrocarbon receptor interacting protein (Fragment) | IPI00925804 | 0.751 | 0.04951 | 3 | 4 | 8 | 5 |
| 358 | Glucosamine-6-phosphate isomerase 1 | IPI00009305 | 0.745 | 0.04968 | 7 | 14 | 16 | 13 |
| 359 | Isoform 2 of Serine/threonine-protein phosphatase PGAM5, mitochondrial | IPI00063242 | 0.745 | 0.04968 | 14 | 7 | 18 | 11 |
| 360 | Rho GTPase-activating protein 1 | IPI00020567 | 0.744 | 0.04968 | 6 | 7 | 10 | 10 |
| 361 | Isoform 1 of Cullin-4B | IPI00179057 | 0.744 | 0.04968 | 8 | 5 | 6 | 14 |
| 362 | Peptidyl-prolyl cis-trans isomerase NIMA-interacting 1 | IPI00013723 | 0.744 | 0.04968 | 6 | 7 | 8 | 12 |
| 363 | Isoform 1 of Protein canopy homolog 2 | IPI00443909 | 0.744 | 0.04968 | 7 | 6 | 10 | 10 |
| 364 | Isoform 1 of Sorting nexin-12 | IPI00438170 | 0.744 | 0.04968 | 6 | 7 | 12 | 8 |
| 365 | Catalase | IPI00465436 | 0.744 | 0.04968 | 6 | 7 | 11 | 9 |
| 366 | Isoform 1 of Transcription factor BTF3 | IPI00221035 | 0.742 | 0.04981 | 16 | 16 | 19 | 22 |
| 367 | Alpha-actinin-4 | IPI00013808 | 0.742 | 0.04981 | 62 | 48 | 64 | 59 |
| 368 | Inosine-5'-monophosphate dehydrogenase 2 | IPI00291510 | 0.742 | 0.04981 | 21 | 25 | 30 | 26 |
| 369 | D-3-phosphoglycerate dehydrogenase | IPI00011200 | 0.735 | 0.04993 | 11 | 11 | 16 | 14 |
| 370 | Peroxiredoxin-2 | IPI00027350 | 0.733 | 0.05027 | 30 | 18 | 35 | 23 |
| 371 | Isoform 2 of Nuclear protein localization protein 4 homolog | IPI00001676 | 0.730 | 0.05027 | 8 | 6 | 11 | 10 |
| 372 | Isocitrate dehydrogenase [NADP], mitochondrial | IPI00011107 | 0.730 | 0.05027 | 5 | 9 | 11 | 10 |
| 373 | Fascin | IPI00163187 | 0.728 | 0.05064 | 5 | 3 | 6 | 8 |
| 374 | Isoform 1 of Peroxisomal acyl-coenzyme A oxidase 1 | IPI00296907 | 0.728 | 0.05064 | 2 | 6 | 7 | 7 |
| 375 | U6 snRNA-associated Sm-like protein LSm3 | IPI00219229 | 0.728 | 0.05064 | 6 | 0 | 6 | 8 |
| 376 | cDNA FLJ55034 | IPI00384122 | 0.728 | 0.05064 | 3 | 5 | 5 | 9 |

| No. | Description | Accession number | STN[1] | p-Value[1] | Con_A[2] | Con_B[2] | RG3_A[2] | RG3_B[2] |
|---|---|---|---|---|---|---|---|---|
| 377 | Alpha-centractin | IPI00029468 | 0.726 | 0.05299 | 12 | 11 | 16 | 15 |
| 378 | Isoform 1 of RuvB-like 1 | IPI00021187 | 0.724 | 0.05299 | 25 | 25 | 32 | 28 |
| 379 | Puromycin-sensitive aminopeptidase | IPI00026216 | 0.724 | 0.05311 | 22 | 13 | 26 | 18 |
| 380 | Vesicle transport protein GOT1B | IPI00007061 | 0.722 | 0.05315 | 2 | 0 | 5 | 4 |
| 381 | Isoform 1 of Multidrug resistance-associated protein 4 | IPI00006675 | 0.722 | 0.05315 | 0 | 0 | 2 | 7 |
| 382 | Monocarboxylate transporter 1 | IPI00024650 | 0.722 | 0.05315 | 2 | 0 | 7 | 2 |
| 383 | Isoform 1 of V-type proton ATPase subunit H | IPI00296191 | 0.722 | 0.05315 | 2 | 2 | 3 | 6 |
| 384 | Isoform 2 of Ubiquitin conjugation factor E4 A | IPI00028957 | 0.722 | 0.05315 | 2 | 0 | 2 | 7 |
| 385 | Tetratricopeptide repeat protein 37 | IPI00005634 | 0.722 | 0.05315 | 2 | 0 | 4 | 5 |
| 386 | Isoform 1 of Retinol dehydrogenase 11 | IPI00339384 | 0.722 | 0.05315 | 2 | 2 | 7 | 0 |
| 387 | Isoform Delta-1 of Serine/threonine-protein phosphatase 2A 56 kDa regulatory subunit delta isoform | IPI00000030 | 0.722 | 0.05315 | 0 | 0 | 3 | 6 |
| 388 | Claudin-1 | IPI00000691 | 0.722 | 0.05315 | 0 | 0 | 6 | 3 |
| 389 | NudC domain-containing protein 2 | IPI00103142 | 0.722 | 0.05315 | 2 | 0 | 6 | 3 |
| 390 | Protein TFG | IPI00294619 | 0.722 | 0.05315 | 0 | 2 | 4 | 5 |
| 391 | Isoform 2 of WASH complex subunit 7 | IPI00164930 | 0.722 | 0.05315 | 2 | 2 | 2 | 7 |
| 392 | Putative uncharacterized protein TXNRD2 | IPI00157820 | 0.722 | 0.05315 | 0 | 2 | 5 | 4 |
| 393 | Thioredoxin-interacting protein | IPI00007956 | 0.722 | 0.05315 | 0 | 0 | 0 | 7 |
| 394 | Isoform 1 of Nuclear pore complex protein Nup214 | IPI00183294 | 0.722 | 0.05315 | 0 | 0 | 3 | 6 |
| 395 | Isoform 1 of Beta-galactosidase | IPI00441344 | 0.722 | 0.05315 | 0 | 0 | 4 | 5 |
| 396 | Isoform 1 of Probable aminopeptidase NPEPL1 | IPI00100292 | 0.722 | 0.05315 | 0 | 0 | 4 | 5 |
| 397 | Isoform 1 of Apolipoprotein O | IPI00042580 | 0.718 | 0.05537 | 14 | 10 | 23 | 9 |
| 398 | Isoform 2 of Microtubule-associated protein 4 | IPI00220113 | 0.717 | 0.05596 | 7 | 8 | 9 | 13 |
| 399 | Aldose reductase | IPI00413641 | 0.707 | 0.05642 | 4 | 5 | 9 | 6 |
| 400 | 39S ribosomal protein L1, mitochondrial | IPI00549381 | 0.707 | 0.05642 | 2 | 7 | 7 | 8 |
| 401 | Isoform 1 of Paraspeckle component 1 | IPI00103525 | 0.707 | 0.05642 | 2 | 7 | 6 | 9 |
| 402 | DnaJ homolog subfamily C member 8 | IPI00003438 | 0.707 | 0.05642 | 5 | 4 | 6 | 9 |
| 403 | 28S ribosomal protein S28, mitochondrial | IPI00022276 | 0.707 | 0.05642 | 0 | 7 | 4 | 11 |
| 404 | Isoform 1 of E3 UFM1-protein ligase 1 | IPI00844000 | 0.705 | 0.05646 | 7 | 9 | 10 | 13 |
| 405 | cytochrome b5 type B precursor | IPI00303954 | 0.702 | 0.05675 | 14 | 12 | 20 | 14 |
| 406 | Isoform 1 of Protein diaphanous homolog 1 | IPI00852685 | 0.702 | 0.05708 | 23 | 16 | 21 | 27 |
| 407 | Ubiquitin carboxyl-terminal hydrolase 7 | IPI00003965 | 0.702 | 0.05708 | 20 | 19 | 23 | 25 |
| 408 | NADH dehydrogenase [ubiquinone] 1 alpha subcomplex subunit 9, mitochondrial | IPI00003968 | 0.702 | 0.05708 | 16 | 23 | 23 | 25 |
| 409 | 10 kDa heat shock protein, mitochondrial | IPI00220362 | 0.694 | 0.05750 | 9 | 8 | 11 | 13 |
| 410 | Isoform 2 of Inverted formin-2 | IPI00876962 | 0.694 | 0.05750 | 7 | 10 | 13 | 11 |
| 411 | Eukaryotic translation elongation factor 1 epsilon-1 | IPI00003588 | 0.694 | 0.05750 | 9 | 8 | 15 | 9 |
| 412 | Proteasome subunit beta type-3 | IPI00028004 | 0.692 | 0.05759 | 22 | 19 | 28 | 22 |
| 413 | Inosine triphosphate pyrophosphatase | IPI00018783 | 0.689 | 0.05763 | 5 | 5 | 10 | 6 |
| 414 | 29 kDa protein | IPI00453476 | 0.688 | 0.05959 | 16 | 12 | 15 | 21 |
| 415 | Isoform 1 of Cleavage and polyadenylation specificity factor subunit 6 | IPI00012998 | 0.688 | 0.05959 | 16 | 12 | 23 | 13 |
| 416 | Isoform 3 of Fermitin family homolog 1 | IPI00220602 | 0.687 | 0.05993 | 0 | 3 | 8 | 0 |
| 417 | WD repeat-containing protein 11 | IPI00412224 | 0.687 | 0.05993 | 3 | 2 | 4 | 6 |
| 418 | AFG3-like protein 2 | IPI00001091 | 0.687 | 0.05993 | 2 | 3 | 7 | 3 |
| 419 | Pyruvate dehydrogenase protein X component, mitochondrial | IPI00298423 | 0.687 | 0.05993 | 2 | 3 | 3 | 7 |
| 420 | Casein kinase II subunit beta | IPI00010865 | 0.687 | 0.05993 | 3 | 2 | 5 | 5 |
| 421 | Keratin, type II cytoskeletal 5 | IPI00009867 | 0.687 | 0.05993 | 3 | 0 | 8 | 2 |
| 422 | Ras-related protein Rab-9A | IPI00016372 | 0.687 | 0.05993 | 3 | 0 | 5 | 5 |
| 423 | 2-oxoisovalerate dehydrogenase subunit alpha, mitochondrial | IPI00025100 | 0.687 | 0.05993 | 3 | 2 | 4 | 6 |
| 424 | Isoform Non-muscle of Myosin light polypeptide 6 | IPI00335168 | 0.683 | 0.06010 | 10 | 8 | 14 | 11 |
| 425 | cDNA FLJ59367, highly similar to Adenylosuccinate lyase | IPI00026904 | 0.683 | 0.06010 | 8 | 10 | 13 | 12 |
| 426 | Isochorismatase domain-containing protein 1 | IPI00304082 | 0.683 | 0.06010 | 9 | 9 | 13 | 12 |
| 427 | Mitochondrial fission 1 protein | IPI00007052 | 0.683 | 0.06010 | 9 | 9 | 14 | 11 |
| 428 | 60S ribosomal protein L36 | IPI00216237 | 0.681 | 0.06035 | 18 | 11 | 20 | 17 |
| 429 | Isoform 1 of Solute carrier family 12 member 2 | IPI00022649 | 0.675 | 0.06043 | 19 | 11 | 19 | 19 |
| 430 | Phenylalanyl-tRNA synthetase beta chain | IPI00300074 | 0.675 | 0.06043 | 17 | 13 | 15 | 23 |
| 431 | cysteinyl-tRNA synthetase, cytoplasmic isoform c | IPI00027443 | 0.675 | 0.06043 | 14 | 16 | 20 | 18 |
| 432 | Dipeptidyl peptidase 1 | IPI00022810 | 0.674 | 0.06064 | 17 | 28 | 25 | 29 |
| 433 | Isoform 1 of Phosphatidylinositol transfer protein beta isoform | IPI00334907 | 0.672 | 0.06093 | 7 | 4 | 8 | 9 |
| 434 | Isoform 1 of 39S ribosomal protein L4, mitochondrial | IPI00023334 | 0.672 | 0.06093 | 0 | 9 | 6 | 11 |
| 435 | Isoform 2 of Serrate RNA effector molecule homolog | IPI00220038 | 0.672 | 0.06093 | 6 | 5 | 6 | 11 |
| 436 | Isoform 1 of Bifunctional coenzyme A synthase | IPI00184821 | 0.672 | 0.06093 | 5 | 6 | 5 | 12 |
| 437 | Eukaryotic translation initiation factor 2 subunit 2 | IPI00021728 | 0.672 | 0.06093 | 4 | 7 | 6 | 11 |
| 438 | 60S ribosomal protein L3 | IPI00550021 | 0.671 | 0.06093 | 39 | 50 | 47 | 53 |
| 439 | E3 ubiquitin/ISG15 ligase TRIM25 | IPI00029629 | 0.669 | 0.06097 | 13 | 18 | 21 | 18 |
| 440 | Isoform 1 of Chromodomain-helicase-DNA-binding protein 4 | IPI00000846 | 0.663 | 0.06131 | 60 | 63 | 62 | 73 |
| 441 | Isoform 1 of Leukotriene A-4 hydrolase | IPI00219077 | 0.661 | 0.06164 | 27 | 21 | 27 | 30 |
| 442 | Long-chain-fatty-acid--CoA ligase 3 | IPI00031397 | 0.659 | 0.06164 | 4 | 0 | 4 | 7 |
| 443 | Sphingosine-1-phosphate lyase 1 | IPI00099463 | 0.659 | 0.06164 | 3 | 3 | 5 | 6 |
| 444 | Isoform NELF-C of Negative elongation factor C/D | IPI00164949 | 0.659 | 0.06164 | 0 | 4 | 5 | 6 |
| 445 | Dehydrogenase/reductase SDR family member 7B | IPI00550165 | 0.659 | 0.06164 | 0 | 4 | 5 | 6 |
| 446 | Programmed cell death protein 10 | IPI00298558 | 0.657 | 0.06206 | 5 | 7 | 10 | 8 |
| 447 | Isoform 1 of AP-2 complex subunit beta | IPI00784156 | 0.655 | 0.06332 | 12 | 9 | 14 | 14 |
| 448 | 60S ribosomal protein L15 | IPI00470528 | 0.654 | 0.06340 | 45 | 26 | 44 | 37 |
| 449 | Mitochondrial 2-oxoglutarate/malate carrier protein | IPI00219729 | 0.653 | 0.06340 | 23 | 27 | 30 | 29 |
| 450 | Isoform 1 of Protein-L-isoaspartate(D-aspartate) O-methyltransferase | IPI00411680 | 0.647 | 0.06373 | 11 | 11 | 14 | 15 |
| 451 | Mitochondrial ribosomal protein L21 isoform d | IPI00375677 | 0.644 | 0.06390 | 7 | 6 | 10 | 9 |
| 452 | Serine/threonine-protein phosphatase 2A catalytic subunit alpha isoform | IPI00008380 | 0.644 | 0.06390 | 3 | 10 | 7 | 12 |
| 453 | GTP:AMP phosphotransferase mitochondrial | IPI00465256 | 0.644 | 0.06390 | 6 | 7 | 10 | 9 |
| 454 | V-type proton ATPase catalytic subunit A | IPI00007682 | 0.641 | 0.06390 | 20 | 16 | 19 | 25 |
| 455 | Asparaginyl-tRNA synthetase, cytoplasmic | IPI00306960 | 0.639 | 0.06428 | 11 | 12 | 12 | 18 |
| 456 | Isoform 1 of General transcription factor II-I | IPI00054042 | 0.636 | 0.06457 | 28 | 27 | 30 | 34 |
| 457 | 1,4-alpha-glucan-branching enzyme | IPI00296635 | 0.635 | 0.06457 | 4 | 3 | 8 | 4 |
| 458 | Fructose-bisphosphate aldolase | IPI00418262 | 0.635 | 0.06457 | 0 | 5 | 6 | 6 |
| 459 | 3-ketoacyl-CoA thiolase, mitochondrial | IPI00001539 | 0.635 | 0.06457 | 2 | 5 | 5 | 7 |
| 460 | NADH dehydrogenase [ubiquinone] 1 beta subcomplex subunit 4 | IPI00220059 | 0.635 | 0.06457 | 3 | 4 | 5 | 7 |
| 461 | Eukaryotic translation initiation factor 2 subunit 1 | IPI00219678 | 0.631 | 0.06470 | 9 | 15 | 17 | 14 |
| 462 | U1 small nuclear ribonucleoprotein A | IPI00012382 | 0.631 | 0.06470 | 10 | 14 | 12 | 19 |
| 463 | FKBP1A protein | IPI00413778 | 0.631 | 0.06482 | 6 | 8 | 12 | 8 |
| 464 | Isoform 1 of Hexokinase-1 | IPI00018246 | 0.631 | 0.06482 | 9 | 5 | 10 | 10 |
| 465 | Succinyl-CoA ligase [GDP-forming] subunit beta, mitochondrial | IPI00096066 | 0.631 | 0.06482 | 3 | 11 | 4 | 16 |
| 466 | Proteasome subunit beta type-2 | IPI00028006 | 0.626 | 0.06599 | 23 | 16 | 26 | 21 |
| 467 | Isoform 1 of Isocitrate dehydrogenase [NAD] subunit alpha, mitochondrial | IPI00030702 | 0.624 | 0.06608 | 15 | 10 | 19 | 13 |
| 468 | Acetyl-CoA acetyltransferase, mitochondrial | IPI00030363 | 0.616 | 0.06645 | 34 | 27 | 41 | 29 |
| 469 | Isoform 1 of Serine hydroxymethyltransferase, cytosolic | IPI00002519 | 0.614 | 0.06658 | 4 | 4 | 7 | 6 |
| 470 | Tubulin beta-3 chain | IPI00013683 | 0.614 | 0.06658 | 2 | 6 | 7 | 6 |

| No. | Description | Accession number | STN[1] | p-Value[1] | Con_A[2] | Con_B[2] | RG3_A[2] | RG3_B[2] |
|---|---|---|---|---|---|---|---|---|
| 471 | Ethanolamine-phosphate cytidylyltransferase | IPI00015285 | 0.614 | 0.06658 | 4 | 4 | 8 | 5 |
| 472 | Diablo homolog, mitochondrial precursor | IPI00008418 | 0.614 | 0.06658 | 0 | 6 | 8 | 5 |
| 473 | Heat shock 70 kDa protein 4L | IPI00295485 | 0.614 | 0.06658 | 4 | 4 | 6 | 7 |
| 474 | pyruvate dehydrogenase E1 alpha 1 isoform 2 precursor | IPI00306301 | 0.614 | 0.06658 | 3 | 5 | 6 | 7 |
| 475 | Isoform D of Constitutive coactivator of PPAR-gamma-like protein 1 | IPI00039626 | 0.614 | 0.06658 | 4 | 4 | 4 | 9 |
| 476 | Flotillin-1 | IPI00027438 | 0.614 | 0.06658 | 2 | 6 | 7 | 6 |
| 477 | Aldo-keto reductase family 1 member C2 | IPI00005668 | 0.614 | 0.06658 | 4 | 4 | 8 | 5 |
| 478 | Putative uncharacterized protein DKFZp781K1356 | IPI00412545 | 0.614 | 0.06658 | 3 | 5 | 6 | 7 |
| 479 | Protein NipSnap homolog 2 | IPI00016077 | 0.611 | 0.06779 | 11 | 16 | 17 | 17 |
| 480 | Putative uncharacterized protein MDH1 | IPI00915869 | 0.611 | 0.06779 | 14 | 13 | 18 | 16 |
| 481 | Laminin receptor-like protein LAMRL5 | IPI00411639 | 0.611 | 0.06787 | 36 | 27 | 45 | 27 |
| 482 | Phenylalanyl-tRNA synthetase alpha chain | IPI00031820 | 0.609 | 0.06796 | 6 | 10 | 11 | 11 |
| 483 | Importin-9 | IPI00185146 | 0.609 | 0.06796 | 11 | 5 | 10 | 12 |
| 484 | Isoform 1 of Inorganic pyrophosphatase 2, mitochondrial | IPI00301109 | 0.609 | 0.06796 | 11 | 5 | 11 | 11 |
| 485 | 40S ribosomal protein S14 | IPI00026271 | 0.609 | 0.06875 | 31 | 12 | 32 | 19 |
| 486 | Phosphatidylethanolamine-binding protein 1 | IPI00219446 | 0.608 | 0.06875 | 36 | 28 | 44 | 29 |
| 487 | Stomatin-like protein 2 | IPI00334190 | 0.605 | 0.06875 | 13 | 15 | 19 | 16 |
| 488 | Lon protease homolog, mitochondrial | IPI00005158 | 0.605 | 0.06888 | 23 | 21 | 21 | 31 |
| 489 | PNAS-139 | IPI00000477 | 0.599 | 0.06909 | 9 | 8 | 15 | 8 |
| 490 | Transducin beta-like protein 3 | IPI00477971 | 0.599 | 0.06909 | 8 | 9 | 10 | 13 |
| 491 | Isoleucyl-tRNA synthetase, mitochondrial | IPI00017283 | 0.599 | 0.06909 | 7 | 10 | 13 | 10 |
| 492 | Isoform Long of Glucose-6-phosphate 1-dehydrogenase | IPI00216008 | 0.599 | 0.06909 | 13 | 16 | 23 | 13 |
| 493 | Programmed cell death 6-interacting protein | IPI00246058 | 0.597 | 0.06917 | 23 | 23 | 27 | 27 |
| 494 | Isoform 1 of Acyl-coenzyme A thioesterase 2, mitochondrial | IPI00220906 | 0.596 | 0.06938 | 4 | 5 | 6 | 8 |
| 495 | COP9 signalosome complex subunit 5 | IPI00009958 | 0.596 | 0.06938 | 4 | 5 | 6 | 8 |
| 496 | Isoform 2 of Dedicator of cytokinesis protein 7 | IPI00183572 | 0.596 | 0.06938 | 5 | 4 | 8 | 6 |
| 497 | dynactin subunit 2 | IPI00220503 | 0.596 | 0.06938 | 4 | 5 | 6 | 8 |
| 498 | Heat shock 70 kDa protein 14 | IPI00292499 | 0.596 | 0.06938 | 4 | 5 | 8 | 6 |
| 499 | Isoform 1 of Cytosol aminopeptidase | IPI00419237 | 0.593 | 0.07017 | 14 | 16 | 18 | 19 |
| 500 | Isoform A1-B of Heterogeneous nuclear ribonucleoprotein A1 | IPI00215965 | 0.592 | 0.07030 | 76 | 59 | 78 | 68 |
| 501 | Isoform 1 of Dr1-associated corepressor | IPI00003084 | 0.590 | 0.07034 | 0 | 0 | 6 | 0 |
| 502 | NADH-ubiquinone oxidoreductase chain 1 | IPI00007961 | 0.590 | 0.07034 | 0 | 0 | 2 | 6 |
| 503 | General transcription factor 3C polypeptide 4 | IPI00016725 | 0.590 | 0.07034 | 0 | 2 | 3 | 5 |
| 504 | SF3A2 protein (Fragment) | IPI00017341 | 0.590 | 0.07034 | 0 | 0 | 4 | 4 |
| 505 | SNARE-associated protein Snapin | IPI00018331 | 0.590 | 0.07034 | 0 | 2 | 3 | 5 |
| 506 | Uncharacterized protein C3orf26 | IPI00031679 | 0.590 | 0.07034 | 0 | 0 | 5 | 3 |
| 507 | Isoform 1 of Serine/threonine-protein kinase WNK1 | IPI00004472 | 0.590 | 0.07034 | 0 | 2 | 5 | 3 |
| 508 | Transmembrane 9 superfamily member 4 | IPI00021985 | 0.590 | 0.07034 | 0 | 0 | 0 | 6 |
| 509 | 1-acyl-sn-glycerol-3-phosphate acyltransferase epsilon | IPI00028491 | 0.590 | 0.07034 | 2 | 2 | 6 | 2 |
| 510 | Isoform 1 of Regulator of microtubule dynamics protein 3 | IPI00410079 | 0.590 | 0.07034 | 0 | 0 | 3 | 5 |
| 511 | Transmembrane protein 109 | IPI00031697 | 0.590 | 0.07034 | 0 | 0 | 5 | 3 |
| 512 | Isoform 1 of FAD synthase | IPI00220299 | 0.590 | 0.07034 | 2 | 2 | 4 | 4 |
| 513 | Isoform 4 of Dipeptidyl peptidase 9 | IPI00604483 | 0.590 | 0.07034 | 0 | 0 | 2 | 6 |
| 514 | Seryl-tRNA synthetase, mitochondrial | IPI00328361 | 0.590 | 0.07034 | 0 | 2 | 3 | 5 |
| 515 | Leucine-rich repeat and WD repeat-containing protein 1 | IPI00069309 | 0.590 | 0.07034 | 2 | 2 | 0 | 6 |
| 516 | Probable asparaginyl-tRNA synthetase, mitochondrial | IPI00101664 | 0.590 | 0.07034 | 2 | 0 | 4 | 4 |
| 517 | cDNA FLJ10824 fis, clone NT2RP4001086 (Fragment) | IPI00294810 | 0.590 | 0.07034 | 0 | 0 | 3 | 5 |
| 518 | Proteasome subunit beta type-7 | IPI00003217 | 0.590 | 0.08059 | 7 | 11 | 10 | 14 |
| 519 | Regulation of nuclear pre-mRNA domain-containing protein 1B | IPI00009659 | 0.590 | 0.08059 | 8 | 10 | 10 | 14 |
| 520 | Rho GDP-dissociation inhibitor 1 | IPI00003815 | 0.587 | 0.08159 | 46 | 26 | 43 | 38 |
| 521 | Uncharacterized protein C17orf25 | IPI00007102 | 0.582 | 0.08201 | 17 | 15 | 19 | 20 |
| 522 | Isoform 1 of 5'-3' exoribonuclease 2 | IPI00100151 | 0.582 | 0.08201 | 18 | 14 | 13 | 26 |
| 523 | Ubiquitin-conjugating enzyme E2 L3 | IPI00021347 | 0.581 | 0.08226 | 12 | 7 | 15 | 10 |
| 524 | Pre-mRNA-splicing factor ATP-dependent RNA helicase PRP16 | IPI00294211 | 0.581 | 0.08226 | 10 | 9 | 13 | 12 |
| 525 | Proteasome inhibitor PI31 subunit | IPI00009949 | 0.581 | 0.08226 | 9 | 10 | 10 | 15 |
| 526 | V-type proton ATPase subunit E 1 | IPI00003856 | 0.581 | 0.08226 | 6 | 13 | 11 | 14 |
| 527 | Ribosomal protein S6 kinase alpha-3 | IPI00020898 | 0.580 | 0.08226 | 4 | 6 | 8 | 7 |
| 528 | pyrroline-5-carboxylate reductase 1, mitochondrial isoform 2 | IPI00376503 | 0.580 | 0.08226 | 5 | 5 | 8 | 7 |
| 529 | Tyrosyl-tRNA synthetase, mitochondrial | IPI00165092 | 0.580 | 0.08226 | 3 | 7 | 6 | 9 |
| 530 | Isoform 1 of Pre-mRNA-processing factor 40 homolog A | IPI00337385 | 0.580 | 0.08226 | 5 | 5 | 5 | 10 |
| 531 | Isoform 1 of Rab3 GTPase-activating protein non-catalytic subunit | IPI00554590 | 0.580 | 0.08226 | 6 | 4 | 7 | 8 |
| 532 | Isoform 2 of Extended synaptotagmin-2 | IPI00409635 | 0.580 | 0.08226 | 3 | 7 | 2 | 13 |
| 533 | Tu translation elongation factor, mitochondrial precursor | IPI00027107 | 0.579 | 0.08268 | 30 | 21 | 34 | 25 |
| 534 | Enoyl-CoA hydratase, mitochondrial | IPI00024993 | 0.577 | 0.08280 | 20 | 13 | 22 | 18 |
| 535 | Isoform 1 of UTP--glucose-1-phosphate uridylyltransferase | IPI00329331 | 0.573 | 0.08326 | 7 | 13 | 10 | 16 |
| 536 | 40S ribosomal protein S3a | IPI00419880 | 0.567 | 0.08460 | 46 | 35 | 47 | 43 |
| 537 | Isoform SERCA1B of Sarcoplasmic/endoplasmic reticulum calcium ATPase 1 | IPI00024804 | 0.566 | 0.08460 | 4 | 7 | 4 | 12 |
| 538 | Isoform 1 of Phosphatidate cytidylyltransferase 2 | IPI00032150 | 0.566 | 0.08460 | 6 | 5 | 6 | 10 |
| 539 | S-formylglutathione hydrolase | IPI00411706 | 0.566 | 0.08460 | 5 | 6 | 8 | 8 |
| 540 | Cytochrome c oxidase subunit 5A, mitochondrial | IPI00025086 | 0.566 | 0.08460 | 6 | 5 | 8 | 8 |
| 541 | Acyl-CoA dehydrogenase family member 9, mitochondrial | IPI00152981 | 0.566 | 0.08460 | 5 | 6 | 8 | 8 |
| 542 | Putative ATP-dependent Clp protease proteolytic subunit, mitochondrial | IPI00003870 | 0.566 | 0.08460 | 7 | 4 | 10 | 6 |
| 543 | Coatomer subunit zeta-1 | IPI00032851 | 0.565 | 0.08477 | 12 | 9 | 12 | 15 |
| 544 | Protein FAM49B | IPI00303318 | 0.565 | 0.08477 | 11 | 10 | 12 | 15 |
| 545 | Histone deacetylase 1 | IPI00013774 | 0.565 | 0.08477 | 12 | 9 | 12 | 15 |
| 546 | Methionyl-tRNA synthetase, cytoplasmic | IPI00008240 | 0.563 | 0.08485 | 21 | 15 | 23 | 20 |
| 547 | Tubulin gamma-1 chain | IPI00295081 | 0.560 | 0.08506 | 2 | 3 | 6 | 3 |
| 548 | Isoform 1 of HEAT repeat-containing protein 2 | IPI00242630 | 0.560 | 0.08506 | 0 | 3 | 0 | 7 |
| 549 | Cytokine-like nuclear factor n-pac, isoform CRA_a | IPI00000155 | 0.560 | 0.08506 | 0 | 3 | 4 | 5 |
| 550 | Hydroxymethylglutaryl-CoA lyase, mitochondrial | IPI00293564 | 0.560 | 0.08506 | 0 | 3 | 4 | 5 |
| 551 | importin subunit alpha-6 | IPI00413214 | 0.560 | 0.08506 | 3 | 0 | 4 | 5 |
| 552 | Isoform 1 of Gamma-tubulin complex component 3 | IPI00033516 | 0.560 | 0.08506 | 2 | 3 | 3 | 6 |
| 553 | cDNA FLJ40287 fis, clone TESTI2027909 | IPI00473047 | 0.560 | 0.08506 | 0 | 3 | 0 | 7 |
| 554 | Dolichyl-diphosphooligosaccharide--protein glycosyltransferase subunit STT3A | IPI00297492 | 0.558 | 0.08527 | 10 | 12 | 14 | 14 |
| 555 | Serine/threonine-protein kinase PAK 2 | IPI00419979 | 0.558 | 0.08527 | 12 | 10 | 14 | 14 |
| 556 | Transforming protein RhoA | IPI00478231 | 0.553 | 0.08569 | 10 | 0 | 10 | 7 |
| 557 | Cytochrome b-c1 complex subunit 1, mitochondrial | IPI00013847 | 0.553 | 0.08569 | 5 | 7 | 8 | 9 |
| 558 | Isoform Long of ES1 protein homolog, mitochondrial | IPI00024913 | 0.553 | 0.08569 | 5 | 7 | 7 | 10 |
| 559 | CCR4-NOT transcription complex subunit 7 | IPI00006552 | 0.553 | 0.08569 | 6 | 6 | 6 | 11 |
| 560 | Elongation factor 1-alpha | IPI00025447 | 0.552 | 0.08581 | 152 | 139 | 144 | 160 |
| 561 | Nicotinamide phosphoribosyltransferase | IPI00018873 | 0.551 | 0.08586 | 6 | 17 | 12 | 17 |
| 562 | WD repeat-containing protein 36 | IPI00169325 | 0.551 | 0.08586 | 14 | 9 | 12 | 17 |
| 563 | SUMO-activating enzyme subunit 1 | IPI00033130 | 0.551 | 0.08586 | 11 | 12 | 15 | 14 |
| 564 | Isoform A of AP-1 complex subunit beta-1 | IPI00328257 | 0.550 | 0.08586 | 19 | 20 | 22 | 24 |
| 565 | Isoform 1 of Nuclear pore complex protein Nup160 | IPI00748807 | 0.546 | 0.08607 | 17 | 23 | 19 | 28 |

| No. | Description | Accession number | STN[1] | p-Value[1] | Con_A[2] | Con_B[2] | RG3_A[2] | RG3_B[2] |
|---|---|---|---|---|---|---|---|---|
| 566 | SERPINE1 mRNA binding protein 1, isoform CRA_d | IPI00410693 | 0.544 | 0.08607 | 15 | 9 | 18 | 12 |
| 567 | U2 small nuclear ribonucleoprotein A' | IPI00297477 | 0.542 | 0.08678 | 19 | 22 | 23 | 25 |
| 568 | Isoform 2 of AP-2 complex subunit alpha-2 | IPI00016621 | 0.541 | 0.08686 | 4 | 9 | 7 | 11 |
| 569 | Isoform B of Serine/threonine-protein kinase 24 | IPI00002212 | 0.541 | 0.08686 | 5 | 8 | 9 | 9 |
| 570 | Palmitoyl-protein thioesterase 1 | IPI00002412 | 0.541 | 0.08686 | 7 | 6 | 6 | 12 |
| 571 | Isoform 1 of Annexin A7 | IPI00002460 | 0.541 | 0.08686 | 7 | 6 | 8 | 10 |
| 572 | cDNA FLJ56307, highly similar to Ubiquitin thioesterase protein OTUB1 | IPI00000581 | 0.538 | 0.08724 | 13 | 12 | 18 | 13 |
| 573 | 6-phosphofructokinase type C | IPI00009790 | 0.538 | 0.08724 | 11 | 14 | 11 | 20 |
| 574 | Isoleucyl-tRNA synthetase | IPI00514082 | 0.536 | 0.08728 | 3 | 3 | 5 | 5 |
| 575 | 39S ribosomal protein L38, mitochondrial | IPI00783656 | 0.536 | 0.08728 | 2 | 4 | 4 | 6 |
| 576 | Peptidase M20 domain-containing protein 2 | IPI00217852 | 0.536 | 0.08728 | 4 | 0 | 6 | 4 |
| 577 | Methyltransferase like 7B | IPI00090807 | 0.536 | 0.08728 | 3 | 3 | 4 | 6 |
| 578 | Aldo-keto reductase family 1 member C1 | IPI00029733 | 0.536 | 0.08728 | 0 | 4 | 5 | 5 |
| 579 | Isoform 2 of CDK5 regulatory subunit-associated protein 3 | IPI00018780 | 0.536 | 0.08728 | 4 | 0 | 3 | 7 |
| 580 | ATP synthase subunit g, mitochondrial | IPI00027448 | 0.536 | 0.08728 | 0 | 4 | 3 | 7 |
| 581 | Emerin | IPI00032003 | 0.536 | 0.08728 | 2 | 4 | 6 | 4 |
| 582 | Isoform 1 of UPF0598 protein C8orf82 | IPI00166638 | 0.536 | 0.08728 | 2 | 4 | 4 | 6 |
| 583 | Isoform 2 of Transcription elongation factor A protein 1 | IPI00218106 | 0.536 | 0.08728 | 3 | 3 | 6 | 4 |
| 584 | Isoform 2 of tRNA pseudouridine synthase A | IPI00001716 | 0.536 | 0.08728 | 0 | 4 | 5 | 5 |
| 585 | Isoform 2 of NADH dehydrogenase [ubiquinone] flavoprotein 3, mitochondrial | IPI00291016 | 0.536 | 0.08728 | 4 | 0 | 7 | 3 |
| 586 | 40S ribosomal protein S10 | IPI00008438 | 0.535 | 0.09217 | 66 | 72 | 75 | 73 |
| 587 | Isoform 1 of Serine/arginine repetitive matrix protein 2 | IPI00782992 | 0.534 | 0.09225 | 32 | 35 | 36 | 39 |
| 588 | cDNA FLJ75085, highly similar to Homo sapiens glutaminyl-tRNA synthetase (QARS), mRNA | IPI00026665 | 0.530 | 0.09297 | 8 | 6 | 11 | 8 |
| 589 | Isoform Long of Long-chain-fatty-acid--CoA ligase 4 | IPI00029737 | 0.530 | 0.09297 | 7 | 7 | 8 | 11 |
| 590 | ADP-ribosylation factor-like protein 3 | IPI00003327 | 0.530 | 0.09297 | 7 | 7 | 10 | 9 |
| 591 | probable ubiquitin carboxyl-terminal hydrolase FAF-X isoform 4 | IPI00003964 | 0.526 | 0.09338 | 10 | 17 | 14 | 19 |
| 592 | Isoform 1 of Dipeptidyl peptidase 3 | IPI00020672 | 0.526 | 0.09338 | 12 | 15 | 13 | 20 |
| 593 | DNA-directed RNA polymerase II subunit RPB2 | IPI00027808 | 0.526 | 0.09338 | 15 | 12 | 17 | 16 |
| 594 | 60S ribosomal protein L9 | IPI00031691 | 0.523 | 0.09338 | 35 | 37 | 37 | 43 |
| 595 | Adenosylhomocysteinase | IPI00012007 | 0.523 | 0.09338 | 45 | 27 | 49 | 31 |
| 596 | 60S ribosomal protein L21 | IPI00247583 | 0.521 | 0.09347 | 17 | 11 | 17 | 17 |
| 597 | UPF0027 protein C22orf28 | IPI00550689 | 0.521 | 0.09347 | 12 | 16 | 21 | 13 |
| 598 | Isoform 1 of Protein phosphatase 1 regulatory subunit 7 | IPI00033600 | 0.521 | 0.09418 | 4 | 11 | 9 | 11 |
| 599 | Isoform Crk-II of Adapter molecule crk | IPI00004838 | 0.515 | 0.09451 | 3 | 4 | 4 | 7 |
| 600 | COP9 signalosome complex subunit 7a | IPI00301419 | 0.515 | 0.09451 | 3 | 4 | 7 | 4 |
| 601 | TRMT61A protein (Fragment) | IPI00059718 | 0.515 | 0.09451 | 3 | 4 | 3 | 8 |
| 602 | Signal recognition particle receptor subunit alpha | IPI00385267 | 0.515 | 0.09451 | 4 | 3 | 4 | 7 |
| 603 | Nucleolar complex protein 3 homolog | IPI00102815 | 0.515 | 0.09451 | 4 | 3 | 5 | 6 |
| 604 | Calcium-binding mitochondrial carrier protein Aralar1 | IPI00386271 | 0.515 | 0.09451 | 0 | 5 | 3 | 8 |
| 605 | Isoform 2 of Cytosolic non-specific dipeptidase | IPI00165579 | 0.511 | 0.09456 | 5 | 11 | 5 | 16 |
| 606 | ATP-dependent RNA helicase DHX29 | IPI00217413 | 0.511 | 0.09456 | 6 | 10 | 10 | 11 |
| 607 | cDNA FLJ56389, highly similar to Elongation factor 1-gamma | IPI00000875 | 0.506 | 0.09518 | 64 | 54 | 71 | 56 |
| 608 | Isoform 1 of Plectin-1 | IPI00014898 | 0.501 | 0.09581 | 156 | 152 | 147 | 173 |
| 609 | ADP/ATP translocase 1 | IPI00022891 | 0.498 | 0.09585 | 3 | 5 | 5 | 7 |
| 610 | U6 snRNA-associated Sm-like protein LSm7 | IPI00007163 | 0.498 | 0.09585 | 4 | 4 | 6 | 6 |
| 611 | 33 kDa protein | IPI00413108 | 0.498 | 0.09585 | 3 | 5 | 6 | 6 |
| 612 | RNA-binding protein NOB1 | IPI00022373 | 0.498 | 0.09585 | 3 | 5 | 5 | 7 |
| 613 | Isoform 1 of Acyl-coenzyme A thioesterase 9, mitochondrial | IPI00220710 | 0.498 | 0.09585 | 3 | 5 | 7 | 5 |
| 614 | Mitochondrial import inner membrane translocase subunit Tim17-B | IPI00219833 | 0.498 | 0.09585 | 3 | 5 | 6 | 6 |
| 615 | Isoform 3 of Ester hydrolase C11orf54 | IPI00061507 | 0.498 | 0.09585 | 3 | 5 | 5 | 7 |
| 616 | F-box-like/WD repeat-containing protein TBL1XR1 | IPI00002922 | 0.498 | 0.09585 | 4 | 4 | 8 | 4 |
| 617 | Cytoplasmic dynein 1 light intermediate chain 2 | IPI00011592 | 0.495 | 0.10070 | 9 | 9 | 12 | 11 |
| 618 | Heat shock protein HSP 90-beta | IPI00414676 | 0.494 | 0.10120 | 151 | 93 | 153 | 102 |
| 619 | Ribose-phosphate pyrophosphokinase 1 | IPI00219616 | 0.488 | 0.10166 | 19 | 16 | 23 | 18 |
| 620 | Isoform 1 of Cleavage and polyadenylation specificity factor subunit 7 | IPI00550821 | 0.487 | 0.10166 | 7 | 12 | 14 | 10 |
| 621 | Intron-binding protein aquarius | IPI00297572 | 0.487 | 0.10166 | 8 | 11 | 8 | 16 |
| 622 | Kinesin-1 heavy chain | IPI00012837 | 0.487 | 0.10166 | 10 | 9 | 11 | 13 |
| 623 | 60S ribosomal protein L22 | IPI00219153 | 0.484 | 0.10200 | 28 | 8 | 32 | 10 |
| 624 | Isoform 1 of Armadillo repeat-containing protein 10 | IPI00166394 | 0.483 | 0.10258 | 2 | 7 | 4 | 9 |
| 625 | Isoform 2 of Ubiquitin-associated domain-containing protein 2 | IPI00007034 | 0.483 | 0.10258 | 5 | 4 | 7 | 6 |
| 626 | Aflatoxin B1 aldehyde reductase member 2 | IPI00305978 | 0.483 | 0.10258 | 3 | 6 | 5 | 8 |
| 627 | WASH complex subunit strumpellin | IPI00029175 | 0.483 | 0.10258 | 7 | 0 | 9 | 4 |
| 628 | Isoform 1 of Gamma-glutamylcyclotransferase | IPI00031564 | 0.483 | 0.10258 | 3 | 6 | 9 | 4 |
| 629 | Mitochondrial import receptor subunit TOM70 | IPI00015602 | 0.483 | 0.10258 | 3 | 6 | 6 | 7 |
| 630 | sister chromatid cohesion protein PDS5 homolog A isoform 2 | IPI00303063 | 0.480 | 0.10267 | 11 | 9 | 13 | 12 |
| 631 | Isoform Mitochondrial of Fumarate hydratase, mitochondrial | IPI00296053 | 0.480 | 0.10292 | 20 | 17 | 21 | 22 |
| 632 | Isoform 1 of STE20-like serine/threonine-protein kinase | IPI00022827 | 0.469 | 0.10371 | 5 | 5 | 6 | 8 |
| 633 | Isoform 3 of DNA repair protein RAD50 | IPI00107531 | 0.469 | 0.10371 | 6 | 4 | 6 | 8 |
| 634 | Isoform 1 of F-actin-capping protein subunit beta | IPI00026185 | 0.469 | 0.10698 | 18 | 22 | 25 | 21 |
| 635 | cAMP-dependent protein kinase type I-alpha regulatory subunit | IPI00021831 | 0.468 | 0.10744 | 8 | 14 | 13 | 14 |
| 636 | cDNA FLJ55482, highly similar to Annexin A11 | IPI00414320 | 0.468 | 0.10744 | 11 | 11 | 14 | 13 |
| 637 | Aconitate hydratase, mitochondrial | IPI00017855 | 0.468 | 0.10744 | 7 | 15 | 16 | 11 |
| 638 | cDNA FLJ55599, highly similar to DNA replication licensing factor MCM3 | IPI00013214 | 0.466 | 0.10769 | 24 | 17 | 21 | 26 |
| 639 | 40S ribosomal protein S15 | IPI00479058 | 0.462 | 0.10790 | 66 | 42 | 73 | 43 |
| 640 | Copine-1 | IPI00018452 | 0.462 | 0.10794 | 12 | 11 | 12 | 16 |
| 641 | Dolichyl-diphosphooligosaccharide--protein glycosyltransferase subunit 1 precursor | IPI00025874 | 0.460 | 0.10823 | 36 | 35 | 32 | 46 |
| 642 | cDNA FLJ60299, highly similar to Rab GDP dissociation inhibitor beta | IPI00031461 | 0.460 | 0.10823 | 60 | 50 | 64 | 54 |
| 643 | Isoform 1 of Calcineurin-like phosphoesterase domain-containing protein 1 | IPI00305010 | 0.458 | 0.10823 | 5 | 6 | 7 | 8 |
| 644 | Isoform Long of 14-3-3 protein beta/alpha | IPI00216318 | 0.455 | 0.10865 | 46 | 28 | 47 | 34 |
| 645 | Macrophage migration inhibitory factor | IPI00293276 | 0.453 | 0.10865 | 0 | 2 | 2 | 5 |
| 646 | U6 snRNA-associated Sm-like protein LSm1 | IPI00004436 | 0.453 | 0.10865 | 2 | 2 | 0 | 5 |
| 647 | Isoform 1 of Dual specificity mitogen-activated protein kinase kinase 3 | IPI00218857 | 0.453 | 0.10865 | 2 | 2 | 5 | 0 |
| 648 | Isoform 1 of CLIP-associating protein 1 | IPI00396279 | 0.453 | 0.10865 | 0 | 2 | 2 | 5 |
| 649 | Isoform 1 of Thymocyte nuclear protein 1 | IPI00383163 | 0.453 | 0.10865 | 0 | 0 | 3 | 4 |
| 650 | Isoform 2 of Peptidyl-prolyl cis-trans isomerase NIMA-interacting 4 | IPI00006658 | 0.453 | 0.10865 | 2 | 0 | 4 | 3 |
| 651 | Cyclin-G-associated kinase | IPI00298949 | 0.453 | 0.10865 | 0 | 2 | 0 | 5 |
| 652 | Isoform 2 of Integrator complex subunit 3 | IPI00418336 | 0.453 | 0.10865 | 0 | 0 | 3 | 4 |
| 653 | cDNA, FLJ79450, highly similar to 3-ketoacyl-CoA thiolase, peroxisomal | IPI00011522 | 0.453 | 0.10865 | 0 | 0 | 4 | 3 |
| 654 | Isoform 1 of Elongation factor Tu GTP-binding domain-containing protein 1 | IPI00293026 | 0.453 | 0.10865 | 0 | 2 | 4 | 3 |
| 655 | 145 kDa protein | IPI00218097 | 0.453 | 0.10865 | 0 | 2 | 4 | 3 |
| 656 | Ubiquitin-conjugating enzyme E2 B | IPI00012060 | 0.453 | 0.10865 | 0 | 0 | 0 | 5 |
| 657 | 28S ribosomal protein S18b, mitochondrial | IPI00022316 | 0.453 | 0.10865 | 2 | 0 | 4 | 3 |
| 658 | Elongator complex protein 1 | IPI00293735 | 0.453 | 0.10865 | 2 | 0 | 2 | 5 |
| 659 | Transcription initiation factor IIE subunit beta | IPI00019981 | 0.453 | 0.10865 | 0 | 0 | 4 | 3 |
| 660 | HDCMD34P | IPI00001672 | 0.453 | 0.10865 | 2 | 2 | 4 | 3 |

| No. | Description | Accession number | STN[1] | p-Value[1] | Con_A[2] | Con_B[2] | RG3_A[2] | RG3_B[2] |
|---|---|---|---|---|---|---|---|---|
| 661 | Isoform 3 of Protein VPRBP | IPI00181396 | 0.453 | 0.10865 | 0 | 2 | 4 | 3 |
| 662 | Uncharacterized protein KIAA1797 | IPI00748360 | 0.453 | 0.10865 | 2 | 2 | 3 | 4 |
| 663 | DNA-directed RNA polymerase, mitochondrial precursor | IPI00298738 | 0.453 | 0.10865 | 2 | 0 | 0 | 5 |
| 664 | Phosphatidylserine synthase 1 | IPI00010746 | 0.453 | 0.10865 | 2 | 0 | 2 | 5 |
| 665 | Cullin-5 | IPI00216003 | 0.453 | 0.10865 | 0 | 0 | 2 | 5 |
| 666 | Isoform 1 of Telomeric repeat-binding factor 2 | IPI00024214 | 0.453 | 0.10865 | 0 | 2 | 0 | 5 |
| 667 | RER1 protein | IPI00005728 | 0.453 | 0.10865 | 2 | 0 | 2 | 5 |
| 668 | Isoform 1 of Translation initiation factor eIF-2B subunit gamma | IPI00006504 | 0.453 | 0.10865 | 0 | 0 | 4 | 3 |
| 669 | Short/branched chain specific acyl-CoA dehydrogenase, mitochondrial | IPI00024623 | 0.453 | 0.10865 | 0 | 0 | 3 | 4 |
| 670 | Glia maturation factor, beta | IPI00412987 | 0.453 | 0.10865 | 0 | 0 | 3 | 4 |
| 671 | Retinal rod rhodopsin-sensitive cGMP 3',5'-cyclic phosphodiesterase subunit delta | IPI00015161 | 0.453 | 0.10865 | 2 | 0 | 3 | 4 |
| 672 | Sedoheptulokinase | IPI00005914 | 0.453 | 0.10865 | 0 | 0 | 4 | 3 |
| 673 | Myeloid-associated differentiation marker | IPI00102685 | 0.453 | 0.10865 | 0 | 0 | 0 | 5 |
| 674 | Isoform 1 of Ubiquitin-conjugating enzyme E2 Z | IPI00011996 | 0.453 | 0.10865 | 0 | 0 | 4 | 3 |
| 675 | Isoform 1 of SEC23-interacting protein | IPI00026969 | 0.453 | 0.10865 | 2 | 2 | 0 | 5 |
| 676 | Isocitrate dehydrogenase [NAD] subunit gamma, mitochondrial | IPI00220150 | 0.453 | 0.10865 | 0 | 0 | 3 | 4 |
| 677 | cDNA FLJ59712, highly similar to Golgi reassembly-stacking protein 2 | IPI00743931 | 0.453 | 0.10865 | 0 | 2 | 4 | 3 |
| 678 | ANKHD1-EIF4EBP3 protein | IPI00217442 | 0.453 | 0.10865 | 2 | 2 | 2 | 5 |
| 679 | Phosphomannomutase 2 | IPI00006092 | 0.453 | 0.10865 | 2 | 2 | 5 | 2 |
| 680 | Pre-mRNA-splicing factor CWC22 homolog | IPI00177381 | 0.453 | 0.10865 | 0 | 0 | 0 | 5 |
| 681 | Isoform 1 of Deoxycytidylate deaminase | IPI00296863 | 0.453 | 0.10865 | 0 | 2 | 0 | 5 |
| 682 | Bis(5'-nucleosyl)-tetraphosphatase [asymmetrical] | IPI00221231 | 0.453 | 0.10865 | 0 | 0 | 4 | 3 |
| 683 | Isoform 2 of Leucine-rich repeat flightless-interacting protein 1 | IPI00006207 | 0.453 | 0.10865 | 2 | 0 | 3 | 4 |
| 684 | Isoform 1 of Rab GTPase-activating protein 1 | IPI00016702 | 0.453 | 0.10865 | 0 | 2 | 2 | 5 |
| 685 | Macrophage-capping protein | IPI00027341 | 0.453 | 0.10865 | 2 | 0 | 4 | 3 |
| 686 | Isoform 1 of Apoptosis-inducing factor 2 | IPI00013909 | 0.453 | 0.10865 | 0 | 2 | 0 | 5 |
| 687 | Isoform 3 of Protein PRRC1 | IPI00217053 | 0.453 | 0.10865 | 0 | 0 | 0 | 5 |
| 688 | Isoform Long of Beta-glucuronidase | IPI00027745 | 0.453 | 0.10865 | 2 | 0 | 0 | 5 |
| 689 | OTU domain-containing protein 6B | IPI00182180 | 0.453 | 0.10865 | 0 | 2 | 3 | 4 |
| 690 | Hepatoma-derived growth factor | IPI00020956 | 0.451 | 0.11471 | 12 | 13 | 17 | 13 |
| 691 | Hypoxanthine-guanine phosphoribosyltransferase | IPI00218493 | 0.449 | 0.11509 | 45 | 32 | 48 | 36 |
| 692 | Succinate dehydrogenase [ubiquinone] iron-sulfur subunit, mitochondrial | IPI00294911 | 0.447 | 0.11509 | 6 | 6 | 11 | 5 |
| 693 | 40S ribosomal protein S18 | IPI00013296 | 0.439 | 0.11793 | 47 | 36 | 51 | 39 |
| 694 | Protein RCC2 | IPI00465044 | 0.437 | 0.11810 | 6 | 7 | 4 | 13 |
| 695 | Peptidyl-prolyl cis-trans isomerase H | IPI00007346 | 0.437 | 0.11810 | 6 | 7 | 7 | 10 |
| 696 | Mitochondrial import receptor subunit TOM22 homolog | IPI00024976 | 0.432 | 0.11835 | 16 | 13 | 17 | 17 |
| 697 | Junction plakoglobin | IPI00554711 | 0.429 | 0.11860 | 3 | 2 | 3 | 5 |
| 698 | Isoform GTBP-N of DNA mismatch repair protein Msh6 | IPI00384456 | 0.429 | 0.11860 | 2 | 3 | 4 | 4 |
| 699 | Putative uncharacterized protein | IPI00260769 | 0.429 | 0.11860 | 3 | 0 | 6 | 0 |
| 700 | Vacuolar protein sorting-associated protein 33A | IPI00073179 | 0.429 | 0.11860 | 2 | 3 | 0 | 6 |
| 701 | NADH dehydrogenase [ubiquinone] 1 beta subcomplex subunit 5, mitochondrial | IPI00013459 | 0.429 | 0.11860 | 0 | 3 | 0 | 6 |
| 702 | Isoform 2 of 6-phosphofructokinase, muscle type | IPI00219585 | 0.429 | 0.11860 | 3 | 2 | 3 | 5 |
| 703 | NADH dehydrogenase [ubiquinone] 1 beta subcomplex subunit 8, mitochondrial | IPI00028883 | 0.429 | 0.11860 | 0 | 3 | 0 | 6 |
| 704 | TATA-binding protein-associated factor 172 | IPI00024802 | 0.429 | 0.11860 | 0 | 3 | 0 | 6 |
| 705 | Cation-independent mannose-6-phosphate receptor | IPI00289819 | 0.429 | 0.11860 | 3 | 2 | 3 | 5 |
| 706 | Isoform 1 of 28S ribosomal protein S11, mitochondrial | IPI00010244 | 0.429 | 0.11860 | 3 | 2 | 6 | 0 |
| 707 | Dehydrogenase/reductase SDR family member on chromosome X | IPI00166860 | 0.429 | 0.11860 | 3 | 0 | 3 | 5 |
| 708 | Sulfide:quinone oxidoreductase, mitochondrial | IPI00009634 | 0.429 | 0.11860 | 2 | 3 | 4 | 4 |
| 709 | Density-regulated protein | IPI00306280 | 0.429 | 0.11860 | 0 | 3 | 2 | 6 |
| 710 | Putative deoxyribose-phosphate aldolase | IPI00219677 | 0.429 | 0.11860 | 3 | 2 | 4 | 4 |
| 711 | Isoform 1 of Retinoid-inducible serine carboxypeptidase | IPI00012426 | 0.429 | 0.11860 | 3 | 0 | 3 | 5 |
| 712 | Isoform 1a of Oxysterol-binding protein-related protein 3 | IPI00023555 | 0.429 | 0.11860 | 3 | 2 | 3 | 5 |
| 713 | Isoform 1 of LIM and SH3 domain protein 1 | IPI00000861 | 0.428 | 0.11860 | 7 | 7 | 10 | 8 |
| 714 | Sialic acid synthase | IPI00147874 | 0.428 | 0.11860 | 7 | 7 | 9 | 9 |
| 715 | Isoform 1 of Platelet-activating factor acetylhydrolase IB subunit alpha | IPI00218728 | 0.427 | 0.12061 | 14 | 16 | 23 | 12 |
| 716 | Phosphoribosylformylglycinamidine synthase | IPI00004534 | 0.425 | 0.12082 | 28 | 28 | 26 | 36 |
| 717 | histone deacetylase complex subunit SAP18 | IPI00011698 | 0.420 | 0.12099 | 5 | 10 | 6 | 13 |
| 718 | Chloride intracellular channel protein 4 | IPI00001960 | 0.420 | 0.12099 | 6 | 9 | 11 | 8 |
| 719 | Signal peptidase complex catalytic subunit SEC11A | IPI00104128 | 0.420 | 0.12099 | 6 | 9 | 6 | 13 |
| 720 | Cytochrome b-c1 complex subunit Rieske, mitochondrial | IPI00026964 | 0.420 | 0.12099 | 6 | 9 | 9 | 10 |
| 721 | Isoform 1 of Malignant T cell-amplified sequence 1 | IPI00179026 | 0.419 | 0.12099 | 13 | 19 | 19 | 18 |
| 722 | Putative uncharacterized protein PSME2 | IPI00384051 | 0.419 | 0.12099 | 18 | 14 | 20 | 17 |
| 723 | Eukaryotic translation initiation factor 2 subunit 3 | IPI00297982 | 0.412 | 0.12140 | 8 | 8 | 12 | 8 |
| 724 | Isoform 2 of Isochorismatase domain-containing protein 2, mitochondrial | IPI00003031 | 0.412 | 0.12140 | 7 | 9 | 8 | 12 |
| 725 | Tripeptidyl-peptidase 2 | IPI00020416 | 0.412 | 0.12341 | 18 | 16 | 17 | 22 |
| 726 | Coronin-1B | IPI00007058 | 0.409 | 0.12362 | 4 | 2 | 4 | 5 |
| 727 | DNA polymerase alpha catalytic subunit | IPI00220317 | 0.409 | 0.12362 | 3 | 3 | 4 | 5 |
| 728 | Transmembrane protein 43 | IPI00301280 | 0.409 | 0.12362 | 4 | 0 | 3 | 6 |
| 729 | HIV Tat-specific factor 1 | IPI00013788 | 0.409 | 0.12362 | 2 | 4 | 5 | 4 |
| 730 | DNA polymerase | IPI00744598 | 0.409 | 0.12362 | 3 | 3 | 3 | 6 |
| 731 | Interferon-induced 17 kDa protein | IPI00375631 | 0.409 | 0.12362 | 2 | 4 | 5 | 4 |
| 732 | Histone H1x | IPI00021924 | 0.409 | 0.12362 | 3 | 3 | 6 | 3 |
| 733 | Choline-phosphate cytidylyltransferase A | IPI00329338 | 0.409 | 0.12362 | 0 | 4 | 3 | 6 |
| 734 | Methylmalonyl-CoA mutase, mitochondrial | IPI00024934 | 0.409 | 0.12362 | 4 | 0 | 4 | 5 |
| 735 | Isoform 4 of Abhydrolase domain-containing protein 11 | IPI00171152 | 0.409 | 0.12362 | 3 | 3 | 4 | 5 |
| 736 | Isoform 1 of Lysocardiolipin acyltransferase 1 | IPI00419643 | 0.409 | 0.12362 | 2 | 4 | 4 | 5 |
| 737 | DnaJ homolog subfamily C member 7 | IPI00329629 | 0.409 | 0.12362 | 2 | 4 | 4 | 5 |
| 738 | Isoform 1 of RNA-binding protein 14 | IPI00013174 | 0.409 | 0.12362 | 4 | 0 | 7 | 0 |
| 739 | Charged multivesicular body protein 5 | IPI00100796 | 0.409 | 0.12362 | 3 | 3 | 5 | 4 |
| 740 | MACRO domain-containing protein 1 | IPI00155601 | 0.409 | 0.12362 | 3 | 3 | 4 | 5 |
| 741 | Myosin-Ie | IPI00329672 | 0.409 | 0.12362 | 2 | 4 | 4 | 5 |
| 742 | D-dopachrome decarboxylase | IPI00293867 | 0.409 | 0.12362 | 0 | 4 | 4 | 5 |
| 743 | Squalene synthase | IPI00020944 | 0.409 | 0.12362 | 4 | 0 | 4 | 5 |
| 744 | Gamma-aminobutyric acid receptor-associated protein-like 2 | IPI00026358 | 0.405 | 0.12621 | 9 | 8 | 13 | 8 |
| 745 | Isoform 1 of Tropomyosin alpha-4 chain | IPI00010779 | 0.405 | 0.12621 | 7 | 10 | 10 | 11 |
| 746 | Glycogen phosphorylase, brain form | IPI00004358 | 0.402 | 0.12659 | 16 | 21 | 18 | 24 |
| 747 | DNA polymerase delta catalytic subunit | IPI00002894 | 0.399 | 0.12671 | 10 | 8 | 7 | 15 |
| 748 | Isoform 2 of Nucleoporin NUP188 homolog | IPI00385001 | 0.393 | 0.12809 | 4 | 3 | 2 | 8 |
| 749 | Guanine nucleotide-binding protein G(k) subunit alpha | IPI00220578 | 0.393 | 0.12809 | 3 | 4 | 5 | 5 |
| 750 | Lamina-associated polypeptide 2, isoform alpha | IPI00216230 | 0.393 | 0.12809 | 0 | 5 | 5 | 5 |
| 751 | Putative uncharacterized protein ZFR | IPI00748303 | 0.393 | 0.12809 | 4 | 3 | 3 | 7 |
| 752 | Sec1 family domain-containing protein 1 | IPI00165261 | 0.393 | 0.12809 | 5 | 2 | 4 | 6 |
| 753 | Peptidyl-prolyl cis-trans isomerase FKBP5 | IPI00218775 | 0.393 | 0.12809 | 2 | 5 | 4 | 6 |
| 754 | Small acidic protein | IPI00003419 | 0.393 | 0.12809 | 5 | 0 | 6 | 4 |
| 755 | 28S ribosomal protein S9, mitochondrial | IPI00641924 | 0.393 | 0.12809 | 3 | 4 | 4 | 6 |

| No. | Description | Accession number | STN[1] | p-Value[1] | Con_A[2] | Con_B[2] | RG3_A[2] | RG3_B[2] |
|---|---|---|---|---|---|---|---|---|
| 756 | SPRY domain-containing protein 4 | IPI00291643 | 0.393 | 0.12809 | 0 | 5 | 5 | 5 |
| 757 | Tropomodulin-3 | IPI00005087 | 0.393 | 0.12809 | 3 | 4 | 6 | 4 |
| 758 | Isoform 1 of Septin-2 | IPI00014177 | 0.393 | 0.13090 | 10 | 9 | 11 | 12 |
| 759 | 14-3-3 protein eta | IPI00216319 | 0.393 | 0.13090 | 14 | 5 | 18 | 5 |
| 760 | 26S protease regulatory subunit 4 | IPI00011126 | 0.393 | 0.13090 | 15 | 25 | 20 | 25 |
| 761 | Isoform 1 of Acidic leucine-rich nuclear phosphoprotein 32 family member B | IPI00007423 | 0.387 | 0.13119 | 23 | 19 | 23 | 24 |
| 762 | Carbonyl reductase [NADPH] 1 | IPI00295386 | 0.387 | 0.13119 | 8 | 12 | 11 | 13 |
| 763 | Glutathione S-transferase kappa 1 | IPI00219673 | 0.382 | 0.13278 | 24 | 20 | 26 | 23 |
| 764 | Aminoacyl tRNA synthase complex-interacting multifunctional protein 2 | IPI00011916 | 0.381 | 0.13278 | 12 | 9 | 13 | 12 |
| 765 | Splicing factor 3A subunit 1 | IPI00017451 | 0.381 | 0.13278 | 11 | 10 | 12 | 13 |
| 766 | Actin, cytoplasmic 1 | IPI00021439 | 0.381 | 0.13278 | 190 | 224 | 220 | 204 |
| 767 | Isoform 1 of Protein virilizer homolog | IPI00036742 | 0.379 | 0.13290 | 4 | 4 | 4 | 7 |
| 768 | Cytochrome c1, heme protein, mitochondrial | IPI00029264 | 0.379 | 0.13290 | 4 | 4 | 5 | 6 |
| 769 | Isoform 2 of Epimerase family protein SDR39U1 | IPI00643286 | 0.379 | 0.13290 | 0 | 6 | 3 | 8 |
| 770 | Isoform 1 of Ribonuclease H2 subunit C | IPI00382985 | 0.379 | 0.13290 | 4 | 4 | 6 | 5 |
| 771 | Dolichyl-diphosphooligosaccharide--protein glycosyltransferase subunit DAD1 | IPI00009407 | 0.379 | 0.13290 | 0 | 6 | 3 | 8 |
| 772 | Tetratricopeptide repeat protein 35 | IPI00014149 | 0.379 | 0.13290 | 4 | 4 | 5 | 6 |
| 773 | UBX domain-containing protein 4 | IPI00293946 | 0.379 | 0.13290 | 3 | 5 | 5 | 6 |
| 774 | Serpin H1 | IPI00032140 | 0.374 | 0.13629 | 18 | 29 | 24 | 28 |
| 775 | Proteasome subunit beta type-1 | IPI00025019 | 0.372 | 0.13629 | 24 | 24 | 24 | 29 |
| 776 | Actin-related protein 3 | IPI00028091 | 0.371 | 0.13629 | 10 | 13 | 14 | 13 |
| 777 | Ornithine aminotransferase, mitochondrial | IPI00022334 | 0.371 | 0.13629 | 8 | 15 | 13 | 14 |
| 778 | Small nuclear ribonucleoprotein Sm D3 | IPI00017964 | 0.367 | 0.13658 | 3 | 6 | 8 | 4 |
| 779 | Isoform 1 of Vacuolar protein sorting-associated protein 29 | IPI00170796 | 0.367 | 0.13658 | 4 | 5 | 8 | 4 |
| 780 | Isoform Short of Glycylpeptide N-tetradecanoyltransferase 1 | IPI00218830 | 0.367 | 0.13658 | 0 | 7 | 4 | 8 |
| 781 | Succinyl-CoA ligase [GDP-forming] subunit alpha, mitochondrial | IPI00872762 | 0.367 | 0.13658 | 4 | 5 | 6 | 6 |
| 782 | DnaJ homolog subfamily C member 13 | IPI00307259 | 0.367 | 0.13658 | 4 | 5 | 8 | 4 |
| 783 | DNA damage-binding protein 1 | IPI00293464 | 0.363 | 0.13893 | 53 | 41 | 44 | 56 |
| 784 | Isoform 1 of Poly(U)-binding-splicing factor PUF60 | IPI00069750 | 0.363 | 0.13901 | 10 | 15 | 14 | 15 |
| 785 | Glutathione synthetase | IPI00010706 | 0.359 | 0.13901 | 24 | 30 | 24 | 35 |
| 786 | Early endosome antigen 1 | IPI00329536 | 0.358 | 0.13901 | 12 | 14 | 13 | 17 |
| 787 | Dual specificity mitogen-activated protein kinase kinase 2 | IPI00003783 | 0.356 | 0.13989 | 5 | 5 | 8 | 5 |
| 788 | 39S ribosomal protein L46, mitochondrial | IPI00023161 | 0.356 | 0.13989 | 4 | 6 | 6 | 7 |
| 789 | 165 kDa protein | IPI00240812 | 0.356 | 0.13989 | 5 | 5 | 7 | 6 |
| 790 | 60S ribosomal protein L19 | IPI00025329 | 0.356 | 0.13989 | 7 | 3 | 7 | 6 |
| 791 | Lysosomal alpha-glucosidase | IPI00293088 | 0.356 | 0.13989 | 4 | 6 | 5 | 8 |
| 792 | Isoform 1 of 2',5'-phosphodiesterase 12 | IPI00174390 | 0.356 | 0.13989 | 5 | 5 | 3 | 10 |
| 793 | Isoform 1 of Acylglycerol kinase, mitochondrial | IPI00019353 | 0.356 | 0.13989 | 6 | 4 | 7 | 6 |
| 794 | Telomeric repeat-binding factor 2-interacting protein 1 | IPI00008961 | 0.356 | 0.13989 | 4 | 6 | 8 | 5 |
| 795 | Exportin-T | IPI00306290 | 0.356 | 0.13989 | 4 | 6 | 5 | 8 |
| 796 | Isoform 1 of Cullin-associated NEDD8-dissociated protein 1 | IPI00100160 | 0.355 | 0.14164 | 49 | 52 | 52 | 55 |
| 797 | Isoform 1 of NADH-cytochrome b5 reductase 3 | IPI00328415 | 0.351 | 0.14169 | 12 | 16 | 13 | 19 |
| 798 | Keratin-8-like protein 1 | IPI00017870 | 0.349 | 0.14294 | 39 | 20 | 42 | 22 |
| 799 | Probable methylthioribulose-1-phosphate dehydratase | IPI00549730 | 0.347 | 0.14311 | 5 | 6 | 6 | 8 |
| 800 | cDNA FLJ55586, highly similar to MMS19-like protein | IPI00154451 | 0.347 | 0.14311 | 6 | 5 | 9 | 5 |
| 801 | Importin subunit alpha-4 | IPI00012578 | 0.347 | 0.14311 | 7 | 4 | 6 | 8 |
| 802 | Isoform 1 of Proteasome activator complex subunit 4 | IPI00005260 | 0.347 | 0.14311 | 7 | 4 | 4 | 10 |
| 803 | Prenylcysteine oxidase 1 | IPI00384280 | 0.347 | 0.14311 | 4 | 7 | 8 | 6 |
| 804 | cDNA FLJ53975, highly similar to Acetyl-CoA acetyltransferase, cytosolic | IPI00291419 | 0.347 | 0.14311 | 4 | 7 | 6 | 8 |
| 805 | High mobility group protein B2 | IPI00219097 | 0.347 | 0.14311 | 6 | 5 | 9 | 5 |
| 806 | 28S ribosomal protein S10, mitochondrial | IPI00061245 | 0.347 | 0.14311 | 4 | 7 | 6 | 8 |
| 807 | Metaxin-2 | IPI00025717 | 0.347 | 0.14311 | 6 | 5 | 8 | 6 |
| 808 | Signal recognition particle 14 kDa protein | IPI00293434 | 0.347 | 0.14311 | 5 | 6 | 6 | 8 |
| 809 | Rho-related GTP-binding protein RhoG | IPI00017342 | 0.347 | 0.14311 | 4 | 7 | 7 | 7 |
| 810 | Isoform 1 of 3,2-trans-enoyl-CoA isomerase, mitochondrial | IPI00300567 | 0.347 | 0.14453 | 17 | 12 | 20 | 13 |
| 811 | Isoform 5 of Interleukin enhancer-binding factor 3 | IPI00219330 | 0.344 | 0.14457 | 34 | 28 | 34 | 33 |
| 812 | Stress-induced-phosphoprotein 1 | IPI00013894 | 0.344 | 0.14457 | 37 | 25 | 37 | 30 |
| 813 | Galectin-3 | IPI00465431 | 0.344 | 0.14457 | 17 | 13 | 20 | 14 |
| 814 | Isoform DPI of Desmoplakin | IPI00013933 | 0.343 | 0.14499 | 60 | 53 | 53 | 66 |
| 815 | 60S acidic ribosomal protein P0 | IPI00008530 | 0.341 | 0.14499 | 37 | 27 | 37 | 32 |
| 816 | Superkiller viralicidic activity 2-like 2 | IPI00647217 | 0.340 | 0.14512 | 16 | 15 | 14 | 21 |
| 817 | UDP-glucose 6-dehydrogenase | IPI00031420 | 0.340 | 0.14512 | 12 | 19 | 16 | 19 |
| 818 | Tubulin beta-1 chain | IPI00006510 | 0.339 | 0.14512 | 30 | 35 | 33 | 37 |
| 819 | Isoform 1 of Elongation factor Ts, mitochondrial | IPI00021016 | 0.339 | 0.14516 | 4 | 8 | 6 | 9 |
| 820 | Cleavage stimulation factor subunit 3 | IPI00015195 | 0.339 | 0.14516 | 7 | 5 | 7 | 8 |
| 821 | Isoform 1 of Ubiquitin-conjugating enzyme E2 K | IPI00021370 | 0.339 | 0.14516 | 5 | 7 | 9 | 6 |
| 822 | Abhydrolase domain-containing protein 10, mitochondrial | IPI00020075 | 0.337 | 0.14616 | 14 | 18 | 18 | 18 |
| 823 | Isoform A of Lamin-A/C | IPI00021405 | 0.335 | 0.14712 | 48 | 20 | 39 | 34 |
| 824 | Aspartate aminotransferase, cytoplasmic | IPI00219029 | 0.334 | 0.14712 | 16 | 17 | 15 | 22 |
| 825 | Argininosuccinate synthase | IPI00020632 | 0.331 | 0.14721 | 5 | 8 | 8 | 8 |
| 826 | Src substrate cortactin | IPI00029601 | 0.331 | 0.14721 | 7 | 6 | 10 | 6 |
| 827 | PRA1 family protein 3 | IPI00007426 | 0.331 | 0.14721 | 6 | 7 | 6 | 10 |
| 828 | Isoform Long of 60 kDa SS-A/Ro ribonucleoprotein | IPI00019450 | 0.331 | 0.14721 | 5 | 8 | 7 | 9 |
| 829 | proteasome-associated protein ECM29 homolog | IPI00157790 | 0.331 | 0.14808 | 18 | 16 | 16 | 22 |
| 830 | Heterogeneous nuclear ribonucleoprotein H | IPI00013881 | 0.326 | 0.14842 | 40 | 34 | 45 | 34 |
| 831 | Coactosin-like protein | IPI00017704 | 0.324 | 0.14909 | 7 | 7 | 7 | 10 |
| 832 | Isoform 1 of Replication protein A 32 kDa subunit | IPI00013939 | 0.324 | 0.14909 | 8 | 6 | 11 | 6 |
| 833 | Lactoylglutathione lyase | IPI00220766 | 0.323 | 0.15009 | 22 | 15 | 20 | 21 |
| 834 | rRNA 2'-O-methyltransferase fibrillarin | IPI00025039 | 0.320 | 0.15018 | 18 | 20 | 23 | 19 |
| 835 | Pre-mRNA-processing-splicing factor 8 | IPI00007928 | 0.319 | 0.15034 | 183 | 176 | 170 | 197 |
| 836 | cDNA FLJ45706 fis, clone FEBRA2028457, highly similar to Nucleolin | IPI00444262 | 0.318 | 0.15034 | 44 | 36 | 50 | 35 |
| 837 | Isoform 1 of ATP-dependent RNA helicase DDX42 | IPI00409671 | 0.318 | 0.15034 | 7 | 8 | 11 | 7 |
| 838 | Tubulin-specific chaperone A | IPI00217236 | 0.318 | 0.15034 | 8 | 7 | 12 | 6 |
| 839 | Transmembrane protein 126A | IPI00031064 | 0.318 | 0.15034 | 7 | 8 | 10 | 8 |
| 840 | KH-type splicing regulatory protein | IPI00479786 | 0.315 | 0.15089 | 24 | 16 | 25 | 19 |
| 841 | Phosphoglycerate mutase 2 | IPI00218570 | 0.312 | 0.15118 | 10 | 6 | 12 | 7 |
| 842 | Putative uncharacterized protein SPTAN1 | IPI00745092 | 0.311 | 0.15168 | 21 | 21 | 22 | 24 |
| 843 | NADH-ubiquinone oxidoreductase chain 4 | IPI00008495 | 0.310 | 0.15222 | 2 | 0 | 2 | 4 |
| 844 | cDNA FLJ59739, highly similar to Protein transport protein Sec61 subunit alpha isoform 1 | IPI00218466 | 0.310 | 0.15222 | 0 | 0 | 0 | 4 |
| 845 | CDNA FLJ20030 fis, clone ADSU02156 | IPI00014402 | 0.310 | 0.15222 | 2 | 2 | 4 | 0 |
| 846 | Isoform 3 of Nucleoporin NDC1 | IPI00074330 | 0.310 | 0.15222 | 0 | 2 | 3 | 3 |
| 847 | YLP motif-containing protein 1 | IPI00165434 | 0.310 | 0.15222 | 0 | 2 | 0 | 4 |
| 848 | Isoform 1 of Mps one binder kinase activator-like 1B | IPI00301518 | 0.310 | 0.15222 | 0 | 0 | 3 | 3 |
| 849 | DNA mismatch repair protein Msh3 | IPI00329605 | 0.310 | 0.15222 | 2 | 2 | 0 | 4 |
| 850 | Deoxycytidine kinase | IPI00020454 | 0.310 | 0.15222 | 2 | 0 | 3 | 3 |

| No. | Description | Accession number | STN[1] | p-Value[1] | Con_A[2] | Con_B[2] | RG3_A[2] | RG3_B[2] |
|---|---|---|---|---|---|---|---|---|
| 851 | Programmed cell death protein 6 | IPI00025277 | 0.310 | 0.15222 | 0 | 0 | 0 | 4 |
| 852 | Isoform 2 of Double-stranded RNA-specific adenosine deaminase | IPI00025057 | 0.310 | 0.15222 | 2 | 2 | 0 | 4 |
| 853 | Isoform 1 of Ataxin-2-like protein | IPI00456359 | 0.310 | 0.15222 | 0 | 0 | 2 | 4 |
| 854 | Isoform 1 of Acetolactate synthase-like protein | IPI00554541 | 0.310 | 0.15222 | 0 | 2 | 2 | 4 |
| 855 | LanC-like protein 2 | IPI00032995 | 0.310 | 0.15222 | 2 | 0 | 2 | 4 |
| 856 | 26S proteasome non-ATPase regulatory subunit 10 | IPI00003565 | 0.310 | 0.15222 | 0 | 2 | 0 | 4 |
| 857 | Putative uncharacterized protein THADA | IPI00412647 | 0.310 | 0.15222 | 2 | 0 | 2 | 4 |
| 858 | Choline dehydrogenase, mitochondrial | IPI00168603 | 0.310 | 0.15222 | 2 | 0 | 4 | 0 |
| 859 | Isoform 1 of Protein transport protein Sec24A | IPI00873472 | 0.310 | 0.15222 | 0 | 0 | 4 | 0 |
| 860 | Isoform 1 of Protein tyrosine phosphatase type IVA 2 | IPI00020191 | 0.310 | 0.15222 | 2 | 0 | 4 | 0 |
| 861 | Small glutamine-rich tetratricopeptide repeat-containing protein alpha | IPI00013949 | 0.310 | 0.15222 | 0 | 0 | 4 | 2 |
| 862 | Isoform 2 of Choline-phosphate cytidylyltransferase B | IPI00001562 | 0.310 | 0.15222 | 2 | 2 | 2 | 4 |
| 863 | ER lumen protein retaining receptor 1 | IPI00028116 | 0.310 | 0.15222 | 0 | 0 | 3 | 3 |
| 864 | Isoform SCPx of Non-specific lipid-transfer protein | IPI00026105 | 0.310 | 0.15222 | 0 | 0 | 3 | 3 |
| 865 | Amidophosphoribosyltransferase | IPI00029534 | 0.310 | 0.15222 | 2 | 2 | 2 | 4 |
| 866 | Glutamate--cysteine ligase catalytic subunit | IPI00215768 | 0.310 | 0.15222 | 2 | 0 | 4 | 2 |
| 867 | Isoform 1 of Acyl-CoA-binding protein | IPI00010182 | 0.310 | 0.15222 | 0 | 0 | 4 | 0 |
| 868 | Isoform 1 of Integrator complex subunit 4 | IPI00446765 | 0.310 | 0.15222 | 0 | 0 | 2 | 4 |
| 869 | Synaptotagmin-1 | IPI00009439 | 0.310 | 0.15222 | 0 | 0 | 2 | 4 |
| 870 | Translocated promoter region | IPI00514531 | 0.310 | 0.15222 | 2 | 0 | 2 | 4 |
| 871 | rho GTPase-activating protein 4 isoform 1 | IPI00328842 | 0.310 | 0.15222 | 0 | 0 | 3 | 3 |
| 872 | 60S ribosomal protein L34 | IPI00219160 | 0.310 | 0.15222 | 0 | 0 | 4 | 0 |
| 873 | Isoform 1 of Ubiquitin carboxyl-terminal hydrolase 15 | IPI00000728 | 0.310 | 0.15222 | 0 | 2 | 2 | 4 |
| 874 | Acetyl-coenzyme A synthetase, cytoplasmic | IPI00413730 | 0.310 | 0.15222 | 0 | 2 | 3 | 3 |
| 875 | Heat shock-related 70 kDa protein 2 | IPI00007702 | 0.310 | 0.15222 | 0 | 2 | 2 | 4 |
| 876 | Isoform 2 of Peroxisomal acyl-coenzyme A oxidase 1 | IPI00477729 | 0.310 | 0.15222 | 0 | 0 | 3 | 3 |
| 877 | NADH-ubiquinone oxidoreductase chain 5 | IPI00008511 | 0.310 | 0.15222 | 2 | 0 | 3 | 3 |
| 878 | Isoform A of Peptidyl-prolyl cis-trans isomerase E | IPI00009316 | 0.310 | 0.15222 | 0 | 2 | 3 | 3 |
| 879 | Huntingtin-interacting protein 1 | IPI00782965 | 0.310 | 0.15222 | 2 | 2 | 3 | 3 |
| 880 | cDNA FLJ56420, highly similar to Aspartyl aminopeptidase | IPI00015856 | 0.310 | 0.15222 | 0 | 0 | 0 | 4 |
| 881 | STE20/SPS1-related proline-alanine-rich protein kinase | IPI00004363 | 0.310 | 0.15222 | 0 | 0 | 0 | 4 |
| 882 | Nucleoside diphosphate-linked moiety X motif 19, mitochondrial | IPI00869107 | 0.310 | 0.15222 | 0 | 2 | 0 | 4 |
| 883 | Isoform 2 of Membrane magnesium transporter 1 | IPI00166785 | 0.310 | 0.15222 | 0 | 0 | 2 | 4 |
| 884 | Isoform 1 of PDZ domain-containing protein 11 | IPI00550841 | 0.310 | 0.15222 | 2 | 2 | 0 | 4 |
| 885 | NEDD8-activating enzyme E1 catalytic subunit | IPI00328154 | 0.310 | 0.15222 | 2 | 2 | 2 | 4 |
| 886 | Isoform 1 of Rho GTPase-activating protein 18 | IPI00296353 | 0.310 | 0.15222 | 2 | 0 | 4 | 2 |
| 887 | U4/U6.U5 tri-snRNP-associated protein 1 | IPI00021417 | 0.310 | 0.15222 | 2 | 2 | 4 | 2 |
| 888 | Isoform CSBP2 of Mitogen-activated protein kinase 14 | IPI00002857 | 0.310 | 0.15222 | 0 | 2 | 0 | 4 |
| 889 | Isoform 1 of Mitochondrial antiviral-signaling protein | IPI00020719 | 0.310 | 0.15222 | 2 | 2 | 3 | 3 |
| 890 | COP9 signalosome complex subunit 8 | IPI00009480 | 0.310 | 0.15222 | 0 | 2 | 3 | 3 |
| 891 | erlin-1 | IPI00007940 | 0.310 | 0.15222 | 0 | 0 | 3 | 3 |
| 892 | Isoform 1 of N-alpha-acetyltransferase 40, NatD catalytic subunit | IPI00328847 | 0.310 | 0.15222 | 2 | 0 | 4 | 0 |
| 893 | Isoform 1 of Tumor suppressor p53-binding protein 1 | IPI00029778 | 0.310 | 0.15222 | 0 | 0 | 2 | 4 |
| 894 | Nuclear pore complex protein Nup50 | IPI00026940 | 0.310 | 0.15222 | 2 | 2 | 2 | 4 |
| 895 | Isoform Alpha of Nuclear inhibitor of protein phosphatase 1 | IPI00030383 | 0.310 | 0.15222 | 2 | 0 | 4 | 2 |
| 896 | cDNA FLJ60094, highly similar to F-actin capping protein subunit beta | IPI00218782 | 0.310 | 0.15222 | 0 | 0 | 4 | 2 |
| 897 | Isoform 4 of Phosphorylase b kinase regulatory subunit beta | IPI00181893 | 0.310 | 0.15222 | 0 | 0 | 0 | 4 |
| 898 | Epidermal growth factor receptor kinase substrate 8 | IPI00290337 | 0.310 | 0.15222 | 0 | 2 | 4 | 2 |
| 899 | 28S ribosomal protein S21, mitochondrial | IPI00014812 | 0.310 | 0.15222 | 2 | 0 | 4 | 0 |
| 900 | Uncharacterized protein C19orf52 | IPI00157215 | 0.310 | 0.15222 | 2 | 2 | 2 | 4 |
| 901 | Atlastin-1 | IPI00103530 | 0.310 | 0.15222 | 0 | 0 | 0 | 4 |
| 902 | Isoform 2 of Carbohydrate kinase domain-containing protein | IPI00645172 | 0.310 | 0.15222 | 2 | 0 | 4 | 0 |
| 903 | cDNA FLJ78567 | IPI00043678 | 0.310 | 0.15222 | 0 | 0 | 2 | 4 |
| 904 | Ribonuclease UK114 | IPI00005038 | 0.310 | 0.15222 | 0 | 2 | 3 | 3 |
| 905 | Delta-1-pyrroline-5-carboxylate dehydrogenase, mitochondrial | IPI00217871 | 0.310 | 0.15222 | 0 | 0 | 2 | 4 |
| 906 | Isoform 1 of THO complex subunit 1 | IPI00305374 | 0.310 | 0.15222 | 0 | 2 | 0 | 4 |
| 907 | Tryptophanyl-tRNA synthetase, mitochondrial | IPI00025050 | 0.310 | 0.15222 | 0 | 0 | 4 | 0 |
| 908 | Lysophosphatidylcholine acyltransferase 1 | IPI00171626 | 0.310 | 0.15222 | 0 | 0 | 3 | 3 |
| 909 | Sorting nexin-9 | IPI00001883 | 0.310 | 0.15222 | 0 | 0 | 0 | 4 |
| 910 | Switch-associated protein 70 | IPI00307200 | 0.310 | 0.15222 | 2 | 2 | 3 | 3 |
| 911 | Isoform 1 of Nucleobindin-2 | IPI00009123 | 0.310 | 0.15222 | 2 | 0 | 4 | 2 |
| 912 | Isoform 1 of Ubiquinone biosynthesis methyltransferase COQ5, mitochondrial | IPI00456965 | 0.310 | 0.15222 | 2 | 0 | 4 | 2 |
| 913 | Histone deacetylase 4 | IPI00010088 | 0.310 | 0.15222 | 0 | 0 | 0 | 4 |
| 914 | Isoform 1 of Cullin-3 | IPI00014312 | 0.310 | 0.15222 | 0 | 2 | 0 | 4 |
| 915 | Probable 2-oxoglutarate dehydrogenase E1 component DHKTD1, mitochondrial | IPI00063408 | 0.310 | 0.15222 | 0 | 0 | 0 | 4 |
| 916 | Hexokinase-3 | IPI00005118 | 0.310 | 0.15222 | 0 | 0 | 0 | 4 |
| 917 | Isoform 1 of Protein disulfide-isomerase TMX3 | IPI00064193 | 0.310 | 0.15222 | 0 | 0 | 0 | 4 |
| 918 | 39S ribosomal protein L32, mitochondrial precursor | IPI00011077 | 0.310 | 0.15222 | 0 | 2 | 3 | 3 |
| 919 | Methyltransferase-like protein 7A | IPI00022300 | 0.310 | 0.15222 | 0 | 0 | 0 | 4 |
| 920 | sideroflexin-3 | IPI00793874 | 0.310 | 0.15222 | 0 | 2 | 4 | 2 |
| 921 | Isoform 2 of Nitrilase homolog 1 | IPI00023779 | 0.310 | 0.15222 | 0 | 0 | 0 | 4 |
| 922 | Isoform 1 of L-2-hydroxyglutarate dehydrogenase, mitochondrial | IPI00016458 | 0.310 | 0.15222 | 0 | 0 | 4 | 0 |
| 923 | Nucleoporin NUP53 | IPI00329650 | 0.310 | 0.15222 | 2 | 0 | 0 | 4 |
| 924 | Isoform 1 of Protein VPRBP | IPI00329528 | 0.310 | 0.15222 | 0 | 0 | 4 | 0 |
| 925 | Isoform 1 of Nucleotide-binding protein-like | IPI00384517 | 0.310 | 0.15222 | 0 | 2 | 0 | 4 |
| 926 | Isoform 3 of DnaJ homolog subfamily C member 11 | IPI00333016 | 0.310 | 0.15222 | 2 | 2 | 0 | 4 |
| 927 | cDNA FLJ56414, highly similar to Homo sapiens proline-, glutamic acid-, leucine-rich protein 1 (PELP1) | IPI00006702 | 0.306 | 0.17870 | 10 | 7 | 6 | 14 |
| 928 | Translation initiation factor eIF-2B subunit alpha | IPI00221300 | 0.306 | 0.17870 | 10 | 7 | 10 | 10 |
| 929 | Isoform 1 of Medium-chain specific acyl-CoA dehydrogenase, mitochondrial | IPI00005040 | 0.306 | 0.17870 | 7 | 10 | 10 | 10 |
| 930 | Poly(rC)-binding protein 1 | IPI00016610 | 0.301 | 0.17983 | 13 | 5 | 13 | 8 |
| 931 | Isoform 2 of Signal recognition particle 68 kDa protein | IPI00102936 | 0.301 | 0.17983 | 10 | 8 | 12 | 9 |
| 932 | von Hippel-Lindau binding protein 1, isoform CRA_b | IPI00334159 | 0.301 | 0.17983 | 11 | 7 | 11 | 10 |
| 933 | Calpain-2 catalytic subunit | IPI00289758 | 0.301 | 0.17983 | 9 | 9 | 8 | 13 |
| 934 | Isoform 2 of TIP41-like protein | IPI00641815 | 0.292 | 0.18129 | 0 | 3 | 2 | 5 |
| 935 | Isoform 4 of Tubulin-specific chaperone D | IPI00030774 | 0.292 | 0.18129 | 0 | 3 | 0 | 5 |
| 936 | Brefeldin A-inhibited guanine nucleotide-exchange protein 2 | IPI00002186 | 0.292 | 0.18129 | 3 | 0 | 4 | 3 |
| 937 | Visinin-like protein 1 | IPI00216313 | 0.292 | 0.18129 | 0 | 3 | 0 | 5 |
| 938 | bifunctional protein NCOAT isoform b | IPI00181391 | 0.292 | 0.18129 | 3 | 0 | 3 | 4 |
| 939 | Oxysterol-binding protein | IPI00163644 | 0.292 | 0.18129 | 3 | 0 | 0 | 5 |
| 940 | Thiopurine S-methyltransferase | IPI00019400 | 0.292 | 0.18129 | 2 | 3 | 3 | 4 |
| 941 | Fumarylacetoacetate hydrolase domain-containing protein 2B | IPI00301994 | 0.292 | 0.18129 | 0 | 3 | 0 | 5 |
| 942 | Retinol dehydrogenase 13 | IPI00301204 | 0.292 | 0.18129 | 3 | 0 | 5 | 0 |
| 943 | Isoform 1 of Coiled-coil domain-containing protein 109A | IPI00171573 | 0.292 | 0.18129 | 3 | 2 | 3 | 4 |
| 944 | Isoform 3 of Protein scribble homolog | IPI00410666 | 0.292 | 0.18129 | 2 | 3 | 3 | 4 |

| No. | Description | Accession number | STN[1] | p-Value[1] | Con_A[2] | Con_B[2] | RG3_A[2] | RG3_B[2] |
|---|---|---|---|---|---|---|---|---|
| 945 | Methylosome protein 50 | IPI00012202 | 0.292 | 0.18129 | 0 | 3 | 3 | 4 |
| 946 | Isoform 1 of ADP-ribosylation factor-like protein 2-binding protein | IPI00015866 | 0.292 | 0.18129 | 3 | 0 | 4 | 3 |
| 947 | Calcium-binding protein 39 | IPI00032561 | 0.292 | 0.18129 | 2 | 3 | 4 | 3 |
| 948 | 39S ribosomal protein L50, mitochondrial | IPI00329036 | 0.292 | 0.18129 | 0 | 3 | 4 | 3 |
| 949 | 39S ribosomal protein L45, mitochondrial | IPI00185859 | 0.292 | 0.18129 | 0 | 3 | 3 | 4 |
| 950 | General transcription factor IIF subunit 2 | IPI00477686 | 0.292 | 0.18129 | 3 | 0 | 5 | 0 |
| 951 | Isoform 1 of Magnesium-dependent phosphatase 1 | IPI00337556 | 0.292 | 0.18129 | 3 | 0 | 5 | 0 |
| 952 | 28S ribosomal protein S30, mitochondrial | IPI00010278 | 0.292 | 0.18129 | 2 | 3 | 4 | 3 |
| 953 | Isoform Beta-1C of Integrin beta-1 | IPI00217561 | 0.292 | 0.18129 | 2 | 3 | 3 | 4 |
| 954 | Isoform 1 of Ras GTPase-activating protein 1 | IPI00026262 | 0.292 | 0.18129 | 2 | 3 | 3 | 4 |
| 955 | Vacuolar protein sorting-associated protein VTA1 homolog | IPI00017160 | 0.292 | 0.18129 | 0 | 3 | 4 | 3 |
| 956 | Isoform 1 of Cytosolic non-specific dipeptidase | IPI00177728 | 0.292 | 0.18129 | 3 | 2 | 4 | 3 |
| 957 | Isoform 1 of Syntaxin-7 | IPI00289876 | 0.292 | 0.18129 | 2 | 3 | 3 | 4 |
| 958 | Actin-related protein 2/3 complex subunit 5-like protein | IPI00414554 | 0.292 | 0.18129 | 3 | 2 | 2 | 5 |
| 959 | cDNA FLJ56184, highly similar to Proto-oncogene tyrosine-protein kinase LCK | IPI00394952 | 0.292 | 0.18129 | 2 | 3 | 2 | 5 |
| 960 | Isoform 2 of cAMP-dependent protein kinase catalytic subunit beta | IPI00376119 | 0.292 | 0.18129 | 3 | 0 | 4 | 3 |
| 961 | 12 kDa protein | IPI00176698 | 0.292 | 0.18129 | 2 | 3 | 3 | 4 |
| 962 | Phosphopantothenate--cysteine ligase | IPI00023987 | 0.292 | 0.18129 | 0 | 3 | 3 | 4 |
| 963 | Isoform 1 of RNA polymerase II-associated factor 1 homolog | IPI00300333 | 0.292 | 0.18129 | 2 | 3 | 5 | 2 |
| 964 | Isoform 1 of Fatty aldehyde dehydrogenase | IPI00333619 | 0.292 | 0.18129 | 3 | 0 | 5 | 0 |
| 965 | NCL protein | IPI00183526 | 0.292 | 0.18229 | 100 | 91 | 99 | 98 |
| 966 | Isoleucyl-tRNA synthetase, cytoplasmic | IPI00644127 | 0.289 | 0.18246 | 25 | 28 | 27 | 30 |
| 967 | TUBA1C protein | IPI00166768 | 0.288 | 0.18250 | 81 | 117 | 88 | 116 |
| 968 | Isoform 2 of Exosome complex exonuclease RRP44 | IPI00183462 | 0.288 | 0.18250 | 9 | 12 | 10 | 14 |
| 969 | Isoform 1 of Mitochondrial import receptor subunit TOM40 homolog | IPI00014053 | 0.280 | 0.18372 | 13 | 10 | 12 | 14 |
| 970 | Protein DEK | IPI00020021 | 0.280 | 0.18372 | 13 | 10 | 12 | 14 |
| 971 | Ataxin-10 | IPI00001636 | 0.280 | 0.18372 | 9 | 14 | 12 | 14 |
| 972 | 60S ribosomal protein L13a | IPI00304612 | 0.278 | 0.18430 | 0 | 4 | 5 | 3 |
| 973 | Isoform 3 of Protein transport protein Sec31A | IPI00305152 | 0.278 | 0.18430 | 3 | 3 | 2 | 6 |
| 974 | Putative uncharacterized protein FUBP3 | IPI00063245 | 0.278 | 0.18430 | 4 | 0 | 2 | 6 |
| 975 | Isoform 1 of Nicalin | IPI00470649 | 0.278 | 0.18430 | 4 | 0 | 5 | 3 |
| 976 | Endoplasmic reticulum resident protein 44 | IPI00401264 | 0.278 | 0.18430 | 2 | 4 | 0 | 6 |
| 977 | Isoform 2 of Hydroxysteroid dehydrogenase-like protein 2 | IPI00031107 | 0.278 | 0.18430 | 3 | 3 | 3 | 5 |
| 978 | Isoform 1 of tRNA-nucleotidyltransferase 1, mitochondrial | IPI00289807 | 0.278 | 0.18430 | 2 | 4 | 4 | 4 |
| 979 | N-alpha-acetyltransferase 20, NatB catalytic subunit | IPI00007174 | 0.278 | 0.18430 | 0 | 4 | 0 | 6 |
| 980 | 40S ribosomal protein S21 | IPI00017448 | 0.278 | 0.18430 | 4 | 2 | 6 | 0 |
| 981 | cDNA FLJ61386, highly similar to Homo sapiens mitochondrial ribosomal protein L43 (MRPL43) | IPI00334579 | 0.278 | 0.18430 | 0 | 4 | 2 | 6 |
| 982 | cDNA FLJ60124, highly similar to Mitochondrial dicarboxylate carrier | IPI00005537 | 0.277 | 0.19869 | 10 | 14 | 13 | 14 |
| 983 | 40S ribosomal protein S20 | IPI00012493 | 0.273 | 0.19957 | 17 | 8 | 20 | 8 |
| 984 | cDNA FLJ59571, highly similar to Eukaryotic translation initiation factor 4gamma 2 | IPI00015952 | 0.266 | 0.20061 | 3 | 4 | 4 | 5 |
| 985 | ribonucleoprotein PTB-binding 1 | IPI00217661 | 0.266 | 0.20061 | 2 | 5 | 4 | 5 |
| 986 | Peroxisomal multifunctional enzyme type 2 | IPI00019912 | 0.266 | 0.20061 | 2 | 5 | 3 | 6 |
| 987 | B-cell receptor-associated protein 31 | IPI00218200 | 0.266 | 0.20061 | 4 | 3 | 6 | 3 |
| 988 | Isoform 2 of Low molecular weight phosphotyrosine protein phosphatase | IPI00218847 | 0.266 | 0.20061 | 4 | 3 | 5 | 4 |
| 989 | Isoform 1 of Ribose-phosphate pyrophosphokinase 2 | IPI00219617 | 0.266 | 0.20061 | 3 | 4 | 4 | 5 |
| 990 | 60S ribosomal protein L8 | IPI00012772 | 0.266 | 0.20061 | 5 | 0 | 7 | 0 |
| 991 | Mitochondrial import receptor subunit TOM20 homolog | IPI00016676 | 0.266 | 0.20061 | 0 | 5 | 0 | 7 |
| 992 | cDNA FLJ56357, highly similar to Homo sapiens apolipoprotein A-I binding protein (APOA1BP), mRNA | IPI00168479 | 0.266 | 0.20061 | 4 | 3 | 4 | 5 |
| 993 | Putative uncharacterized protein NAPRT1 | IPI00412498 | 0.266 | 0.20061 | 0 | 5 | 0 | 7 |
| 994 | Isoform 1 of Protein strawberry notch homolog 1 | IPI00023649 | 0.266 | 0.20061 | 3 | 4 | 2 | 7 |
| 995 | Isoform 1 of Alpha-parvin | IPI00018963 | 0.266 | 0.20061 | 4 | 3 | 4 | 5 |
| 996 | Synaptic vesicle membrane protein VAT-1 homolog | IPI00156689 | 0.266 | 0.20061 | 3 | 4 | 4 | 5 |
| 997 | Nuclear pore glycoprotein p62 | IPI00293533 | 0.266 | 0.20061 | 4 | 3 | 5 | 4 |
| 998 | WD repeat-containing protein 61 | IPI00019269 | 0.264 | 0.20074 | 17 | 11 | 16 | 15 |
| 999 | Isoform Long of Ubiquitin carboxyl-terminal hydrolase 5 | IPI00024664 | 0.262 | 0.20132 | 15 | 14 | 13 | 19 |
| 1000 | Sideroflexin-1 | IPI00009368 | 0.262 | 0.20132 | 16 | 13 | 15 | 17 |
| 1001 | Proteasome subunit beta type-5 | IPI00479306 | 0.262 | 0.20132 | 14 | 15 | 17 | 15 |
| 1002 | Rab GDP dissociation inhibitor alpha | IPI00010154 | 0.260 | 0.20153 | 37 | 38 | 42 | 37 |
| 1003 | DnaJ homolog subfamily B member 11 | IPI00008454 | 0.257 | 0.20174 | 3 | 5 | 5 | 5 |
| 1004 | Isoform 1 of Protein fto | IPI00028277 | 0.257 | 0.20174 | 0 | 6 | 5 | 5 |
| 1005 | Parafibromin | IPI00300659 | 0.257 | 0.20174 | 5 | 3 | 4 | 6 |
| 1006 | Isoform 2 of 1,2-dihydroxy-3-keto-5-methylthiopentene dioxygenase | IPI00470791 | 0.257 | 0.20174 | 5 | 3 | 4 | 6 |
| 1007 | 39S ribosomal protein L13, mitochondrial | IPI00022403 | 0.257 | 0.20174 | 4 | 4 | 3 | 7 |
| 1008 | Isoform 1 of Fermitin family homolog 1 | IPI00304754 | 0.257 | 0.20174 | 4 | 4 | 2 | 8 |
| 1009 | Thioredoxin-related transmembrane protein 1 | IPI00395887 | 0.257 | 0.20174 | 5 | 3 | 7 | 3 |
| 1010 | Cytochrome c-type heme lyase | IPI00023406 | 0.257 | 0.20174 | 4 | 4 | 4 | 6 |
| 1011 | SRA stem-loop-interacting RNA-binding protein, mitochondrial | IPI00009922 | 0.257 | 0.20174 | 4 | 4 | 6 | 4 |
| 1012 | Isoform 1 of Protein LSM12 homolog | IPI00410324 | 0.257 | 0.20174 | 4 | 4 | 5 | 5 |
| 1013 | Isoform 1 of Uncharacterized methyltransferase WBSCR22 | IPI00013810 | 0.257 | 0.20174 | 4 | 4 | 5 | 5 |
| 1014 | Superoxide dismutase [Cu-Zn] | IPI00218733 | 0.257 | 0.20174 | 4 | 4 | 6 | 4 |
| 1015 | Regulator of microtubule dynamics protein 1 | IPI00329696 | 0.257 | 0.20174 | 2 | 6 | 5 | 5 |
| 1016 | Tyrosine-protein kinase CSK | IPI00013212 | 0.257 | 0.20174 | 3 | 5 | 3 | 7 |
| 1017 | Heterogeneous nuclear ribonucleoprotein C-like 1 | IPI00027569 | 0.256 | 0.21169 | 39 | 40 | 40 | 43 |
| 1018 | Probable ATP-dependent RNA helicase DDX6 | IPI00030320 | 0.254 | 0.21169 | 12 | 20 | 18 | 17 |
| 1019 | Isoform 1 of Mitochondrial inner membrane protein | IPI00009960 | 0.252 | 0.21215 | 19 | 14 | 21 | 15 |
| 1020 | splicing factor 3B subunit 2 | IPI00221106 | 0.252 | 0.21215 | 18 | 15 | 21 | 15 |
| 1021 | Ribosome biogenesis protein BRX1 homolog | IPI00181728 | 0.248 | 0.21236 | 4 | 5 | 5 | 6 |
| 1022 | Importin-11 | IPI00301107 | 0.248 | 0.21236 | 5 | 4 | 3 | 8 |
| 1023 | Bleomycin hydrolase | IPI00219575 | 0.248 | 0.21236 | 3 | 6 | 6 | 5 |
| 1024 | Cytochrome c oxidase subunit 5B, mitochondrial | IPI00021785 | 0.248 | 0.21236 | 4 | 5 | 6 | 5 |
| 1025 | Cytoplasmic aconitate hydratase | IPI00008485 | 0.248 | 0.21236 | 4 | 5 | 5 | 6 |
| 1026 | Ribonuclease H2 subunit A | IPI00290192 | 0.248 | 0.21236 | 4 | 5 | 6 | 5 |
| 1027 | 26S proteasome non-ATPase regulatory subunit 6 | IPI00014151 | 0.245 | 0.21253 | 13 | 23 | 18 | 21 |
| 1028 | Vitamin K epoxide reductase complex subunit 1-like protein 1 | IPI00166079 | 0.241 | 0.21357 | 0 | 8 | 10 | 0 |
| 1029 | 60S ribosomal protein L35 | IPI00412607 | 0.241 | 0.21357 | 7 | 3 | 7 | 5 |
| 1030 | Myosin regulatory light chain 12B | IPI00033494 | 0.241 | 0.21357 | 4 | 6 | 4 | 8 |
| 1031 | 39S ribosomal protein L49, mitochondrial | IPI00013195 | 0.241 | 0.21357 | 5 | 5 | 5 | 7 |
| 1032 | Heme oxygenase 2 | IPI00026824 | 0.241 | 0.21357 | 5 | 5 | 4 | 8 |
| 1033 | Isoform 1 of RNA-binding protein Musashi homolog 2 | IPI00073713 | 0.241 | 0.21357 | 2 | 8 | 3 | 9 |
| 1034 | ATP-dependent Clp protease ATP-binding subunit clpX-like, mitochondrial | IPI00008728 | 0.241 | 0.21357 | 6 | 4 | 6 | 6 |
| 1035 | Uncharacterized protein C7orf50 | IPI00031651 | 0.241 | 0.21357 | 4 | 6 | 8 | 4 |
| 1036 | Isoform 1 of Protein phosphatase 1 regulatory subunit 12A | IPI00183002 | 0.241 | 0.21357 | 5 | 5 | 4 | 8 |
| 1037 | Growth arrest and DNA damage-inducible proteins-interacting protein 1 | IPI00552587 | 0.241 | 0.21357 | 4 | 6 | 4 | 8 |
| 1038 | 39S ribosomal protein L44, mitochondrial | IPI00009680 | 0.241 | 0.21357 | 4 | 6 | 6 | 6 |

| No. | Description | Accession number | STN[1] | p-Value[1] | Con_A[2] | Con_B[2] | RG3_A[2] | RG3_B[2] |
|---|---|---|---|---|---|---|---|---|
| 1039 | Eukaryotic translation initiation factor 3 subunit M | IPI00102069 | 0.237 | 0.22102 | 20 | 20 | 28 | 15 |
| 1040 | Isovaleryl-CoA dehydrogenase, mitochondrial | IPI00645805 | 0.234 | 0.22144 | 5 | 6 | 5 | 8 |
| 1041 | Cell growth-regulating nucleolar protein | IPI00015838 | 0.234 | 0.22144 | 2 | 9 | 7 | 6 |
| 1042 | Alcohol dehydrogenase [NADP+] | IPI00220271 | 0.234 | 0.22144 | 4 | 7 | 8 | 5 |
| 1043 | Cullin-1 | IPI00014310 | 0.234 | 0.22144 | 4 | 7 | 5 | 8 |
| 1044 | Isoform Membrane-bound of Catechol O-methyltransferase | IPI00011284 | 0.234 | 0.22144 | 5 | 6 | 5 | 8 |
| 1045 | cDNA FLJ54536, highly similar to Mitochondrial 28S ribosomal protein S27 | IPI00022002 | 0.234 | 0.22144 | 5 | 6 | 6 | 7 |
| 1046 | Isoform 1 of 3'(2'),5'-bisphosphate nucleotidase 1 | IPI00410214 | 0.234 | 0.22144 | 4 | 7 | 5 | 8 |
| 1047 | Serine/threonine-protein phosphatase PP1-beta catalytic subunit | IPI00218236 | 0.234 | 0.22144 | 5 | 6 | 8 | 5 |
| 1048 | Isoform 2 of Apoptosis inhibitor 5 | IPI00554742 | 0.229 | 0.22177 | 24 | 21 | 24 | 24 |
| 1049 | Isoform 2 of COP9 signalosome complex subunit 2 | IPI00018813 | 0.228 | 0.22190 | 6 | 6 | 7 | 7 |
| 1050 | Protein MEMO1 | IPI00032426 | 0.228 | 0.22190 | 4 | 8 | 4 | 10 |
| 1051 | Isoform A of Ras-related C3 botulinum toxin substrate 1 | IPI00010271 | 0.228 | 0.22190 | 6 | 6 | 8 | 6 |
| 1052 | Isoform 1 of 39S ribosomal protein L22, mitochondrial | IPI00414410 | 0.228 | 0.22190 | 7 | 5 | 8 | 6 |
| 1053 | Isoform Rpn10A of 26S proteasome non-ATPase regulatory subunit 4 | IPI00022694 | 0.228 | 0.22190 | 5 | 7 | 8 | 6 |
| 1054 | Isoform 1 of Splicing factor U2AF 65 kDa subunit | IPI00031556 | 0.227 | 0.22595 | 20 | 26 | 19 | 30 |
| 1055 | Glutathione S-transferase omega-1 | IPI00019755 | 0.223 | 0.22625 | 6 | 7 | 9 | 6 |
| 1056 | Ras suppressor protein 1 | IPI00017256 | 0.223 | 0.22625 | 6 | 7 | 7 | 8 |
| 1057 | Mitogen-activated protein kinase 1 | IPI00003479 | 0.223 | 0.22625 | 5 | 8 | 8 | 7 |
| 1058 | cDNA FLJ59211, highly similar to Glucosidase 2 subunit beta | IPI00026154 | 0.223 | 0.22625 | 20 | 29 | 21 | 31 |
| 1059 | Isoform p150 of Dynactin subunit 1 | IPI00029485 | 0.218 | 0.22654 | 7 | 7 | 6 | 10 |
| 1060 | Actin-related protein 2/3 complex subunit 3 | IPI00005162 | 0.218 | 0.22654 | 0 | 12 | 6 | 10 |
| 1061 | Mannosyl-oligosaccharide glucosidase | IPI00328170 | 0.214 | 0.22993 | 4 | 11 | 7 | 10 |
| 1062 | V-type proton ATPase subunit D | IPI00001568 | 0.214 | 0.22993 | 5 | 10 | 9 | 8 |
| 1063 | Phosphatidylinositol transfer protein alpha isoform | IPI00216048 | 0.214 | 0.22993 | 7 | 8 | 7 | 10 |
| 1064 | Signal recognition particle 72 kDa protein | IPI00215888 | 0.210 | 0.23005 | 7 | 9 | 9 | 9 |
| 1065 | Isoform 1 of LETM1 and EF-hand domain-containing protein 1, mitochondrial | IPI00017592 | 0.210 | 0.23005 | 8 | 8 | 8 | 10 |
| 1066 | Eukaryotic translation initiation factor 3, subunit E interacting protein | IPI00465233 | 0.207 | 0.23344 | 31 | 31 | 33 | 32 |
| 1067 | Isoform 1 of General transcription factor 3C polypeptide 1 | IPI00414482 | 0.206 | 0.23356 | 8 | 9 | 9 | 10 |
| 1068 | Isoform 1 of Squamous cell carcinoma antigen recognized by T-cells 3 | IPI00006025 | 0.206 | 0.23356 | 8 | 9 | 8 | 11 |
| 1069 | Isoform 1 of SAM domain and HD domain-containing protein 1 | IPI00294739 | 0.206 | 0.23356 | 9 | 8 | 11 | 8 |
| 1070 | Dihydropteridine reductase | IPI00014439 | 0.206 | 0.23356 | 8 | 9 | 9 | 10 |
| 1071 | Isoform 1 of Methionine adenosyltransferase 2 subunit beta | IPI00002324 | 0.202 | 0.23428 | 10 | 8 | 12 | 8 |
| 1072 | Isoform 1 of KH domain-containing, RNA-binding, signal transduction-associated protein 1 | IPI00008575 | 0.199 | 0.23595 | 10 | 9 | 12 | 9 |
| 1073 | Glutaredoxin-3 | IPI00008552 | 0.196 | 0.23612 | 11 | 9 | 13 | 9 |
| 1074 | septin-9 isoform e | IPI00455033 | 0.196 | 0.23612 | 13 | 7 | 14 | 8 |
| 1075 | Eukaryotic translation initiation factor 3 subunit I | IPI00012795 | 0.196 | 0.23612 | 10 | 10 | 11 | 11 |
| 1076 | 40S ribosomal protein S15a | IPI00221091 | 0.195 | 0.23816 | 43 | 33 | 39 | 40 |
| 1077 | UPF0468 protein C16orf80 | IPI00001655 | 0.193 | 0.23829 | 10 | 11 | 14 | 9 |
| 1078 | coatomer subunit epsilon isoform b | IPI00399318 | 0.191 | 0.23846 | 11 | 11 | 12 | 12 |
| 1079 | Thymidylate kinase | IPI00013862 | 0.188 | 0.24038 | 10 | 13 | 13 | 12 |
| 1080 | poly(rC) binding protein 2 isoform b | IPI00012066 | 0.187 | 0.24038 | 47 | 40 | 50 | 40 |
| 1081 | COP9 signalosome complex subunit 3 | IPI00025721 | 0.186 | 0.24038 | 14 | 10 | 14 | 12 |
| 1082 | Isoform 1 of Ras-related protein Rab-1A | IPI00005719 | 0.182 | 0.24189 | 53 | 41 | 48 | 49 |
| 1083 | Putative uncharacterized protein RPL17 | IPI00394699 | 0.181 | 0.24193 | 15 | 11 | 15 | 13 |
| 1084 | Isoform 2 of Proteasome subunit alpha type-3 | IPI00171199 | 0.181 | 0.24193 | 14 | 12 | 18 | 10 |
| 1085 | T-complex protein 1 subunit epsilon | IPI00010720 | 0.179 | 0.24272 | 65 | 34 | 56 | 46 |
| 1086 | Isoform 1 of Cysteine and histidine-rich domain-containing protein 1 | IPI00015897 | 0.179 | 0.24272 | 13 | 14 | 16 | 13 |
| 1087 | Isoform Short of RNA-binding protein FUS | IPI00221354 | 0.179 | 0.24272 | 21 | 6 | 18 | 11 |
| 1088 | Isoform 1 of Protein KIAA1967 | IPI00182757 | 0.179 | 0.24272 | 14 | 13 | 16 | 13 |
| 1089 | Chromobox protein homolog 3 | IPI00297579 | 0.177 | 0.24277 | 14 | 14 | 18 | 12 |
| 1090 | Ras-related protein Rab-2A | IPI00031169 | 0.177 | 0.24277 | 11 | 17 | 11 | 19 |
| 1091 | GDP-mannose 4,6 dehydratase | IPI00030207 | 0.173 | 0.24419 | 15 | 15 | 19 | 13 |
| 1092 | cDNA FLJ60076, highly similar to ELAV-like protein 1 | IPI00301936 | 0.173 | 0.24419 | 14 | 16 | 16 | 16 |
| 1093 | ATP-dependent RNA helicase A | IPI00844578 | 0.172 | 0.24511 | 142 | 141 | 131 | 156 |
| 1094 | Neuroblast differentiation-associated protein AHNAK | IPI00021812 | 0.172 | 0.24511 | 16 | 15 | 13 | 20 |
| 1095 | Isoform 2 of Splicing factor 3B subunit 3 | IPI00179138 | 0.170 | 0.24511 | 15 | 17 | 16 | 18 |
| 1096 | 26S proteasome non-ATPase regulatory subunit 13 isoform 2 | IPI00375380 | 0.168 | 0.24607 | 20 | 13 | 20 | 15 |
| 1097 | Ras-related protein Rab-10 | IPI00016513 | 0.167 | 0.24615 | 28 | 6 | 28 | 8 |
| 1098 | ADP-sugar pyrophosphatase | IPI00296913 | 0.164 | 0.24665 | 17 | 19 | 20 | 18 |
| 1099 | Isoform 1 of Far upstream element-binding protein 1 | IPI00375441 | 0.164 | 0.24665 | 23 | 13 | 23 | 15 |
| 1100 | Proteasome subunit alpha type-4 | IPI00299155 | 0.161 | 0.24711 | 18 | 20 | 21 | 19 |
| 1101 | cDNA FLJ36192 fis, clone TESTI2027450, highly similar to Eukaryotic translation initiation factor 3 | IPI00654777 | 0.160 | 0.24749 | 24 | 15 | 27 | 14 |
| 1102 | NADH dehydrogenase [ubiquinone] 1 beta subcomplex subunit 6 | IPI00219385 | 0.160 | 0.24749 | 0 | 0 | 2 | 3 |
| 1103 | Isoform 1 of Neuroblastoma-amplified sequence | IPI00333913 | 0.160 | 0.24749 | 0 | 0 | 0 | 3 |
| 1104 | Putative uncharacterized protein HERC1 | IPI00022479 | 0.160 | 0.24749 | 0 | 0 | 3 | 2 |
| 1105 | Isoform 1 of Serine/threonine-protein phosphatase PGAM5, mitochondrial | IPI00788907 | 0.160 | 0.24749 | 0 | 0 | 3 | 0 |
| 1106 | Isoform 1 of AP-2 complex subunit mu | IPI00022256 | 0.160 | 0.24749 | 0 | 0 | 0 | 3 |
| 1107 | Pseudouridylate synthase 7 homolog | IPI00044761 | 0.160 | 0.24749 | 2 | 0 | 2 | 3 |
| 1108 | Phosphatidylinositol-4-phosphate 3-kinase C2 domain-containing subunit alpha | IPI00002580 | 0.160 | 0.24749 | 0 | 0 | 3 | 0 |
| 1109 | Isoform 1 of Transcription elongation regulator 1 | IPI00247871 | 0.160 | 0.24749 | 2 | 0 | 3 | 2 |
| 1110 | NADH dehydrogenase [ubiquinone] 1 beta subcomplex subunit 9 | IPI00255052 | 0.160 | 0.24749 | 0 | 2 | 3 | 0 |
| 1111 | Isoform 1 of COP9 signalosome complex subunit 1 | IPI00156282 | 0.160 | 0.24749 | 0 | 0 | 3 | 2 |
| 1112 | Isoform 1 of OCIA domain-containing protein 1 | IPI00016405 | 0.160 | 0.24749 | 0 | 2 | 0 | 3 |
| 1113 | Isoform 1 of Phosphatidylinositol glycan anchor biosynthesis class U protein | IPI00026044 | 0.160 | 0.24749 | 0 | 0 | 0 | 3 |
| 1114 | FAST kinase domain-containing protein 5 | IPI00414973 | 0.160 | 0.24749 | 0 | 0 | 0 | 3 |
| 1115 | Isoform 1 of Dynamin-like 120 kDa protein, mitochondrial | IPI00006721 | 0.160 | 0.24749 | 2 | 2 | 0 | 3 |
| 1116 | zinc finger protein 294 | IPI00783835 | 0.160 | 0.24749 | 2 | 0 | 0 | 3 |
| 1117 | 28S ribosomal protein S16, mitochondrial | IPI00032872 | 0.160 | 0.24749 | 0 | 0 | 2 | 3 |
| 1118 | Isoform 1 of Serine/threonine-protein kinase 4 | IPI00011488 | 0.160 | 0.24749 | 0 | 2 | 2 | 3 |
| 1119 | mRNA export factor | IPI00019733 | 0.160 | 0.24749 | 0 | 0 | 2 | 3 |
| 1120 | Importin subunit alpha-1 | IPI00303292 | 0.160 | 0.24749 | 0 | 2 | 2 | 3 |
| 1121 | Phosphatidylinositide phosphatase SAC1 | IPI00022275 | 0.160 | 0.24749 | 0 | 0 | 2 | 3 |
| 1122 | Isoform 2 of Serine-protein kinase ATM | IPI00289986 | 0.160 | 0.24749 | 2 | 0 | 2 | 3 |
| 1123 | Isoform 1 of Interferon-inducible double stranded RNA-dependent protein kinase activator A | IPI00021167 | 0.160 | 0.24749 | 0 | 2 | 3 | 0 |
| 1124 | Isoform 1 of Peroxisomal membrane protein PEX16 | IPI00006722 | 0.160 | 0.24749 | 0 | 0 | 0 | 3 |
| 1125 | Transmembrane 7 superfamily member 3 | IPI00009797 | 0.160 | 0.24749 | 0 | 0 | 0 | 3 |
| 1126 | Isoform 2 of Peptidyl-prolyl cis-trans isomerase-like 3 | IPI00032473 | 0.160 | 0.24749 | 0 | 2 | 2 | 3 |
| 1127 | AP-3 complex subunit beta-2 | IPI00005793 | 0.160 | 0.24749 | 0 | 0 | 0 | 3 |
| 1128 | cytochrome c oxidase subunit VIIa polypeptide 2 (liver) precursor | IPI00026570 | 0.160 | 0.24749 | 2 | 0 | 3 | 0 |
| 1129 | treacle protein isoform a | IPI00165041 | 0.160 | 0.24749 | 0 | 0 | 2 | 3 |
| 1130 | Sorting nexin-2 | IPI00299095 | 0.160 | 0.24749 | 0 | 2 | 0 | 3 |
| 1131 | CLASP2 protein | IPI00168165 | 0.160 | 0.24749 | 0 | 0 | 2 | 3 |
| 1132 | Band 4.1-like protein 2 | IPI00015973 | 0.160 | 0.24749 | 2 | 2 | 2 | 3 |

| No. | Description | Accession number | STN[1] | p-Value[1] | Con_A[2] | Con_B[2] | RG3_A[2] | RG3_B[2] |
|---|---|---|---|---|---|---|---|---|
| 1133 | Protein ERGIC-53 | IPI00026530 | 0.160 | 0.24749 | 2 | 0 | 0 | 3 |
| 1134 | Coproporphyrinogen-III oxidase, mitochondrial | IPI00093057 | 0.160 | 0.24749 | 2 | 0 | 0 | 3 |
| 1135 | Isoform 1 of IST1 homolog | IPI00024660 | 0.160 | 0.24749 | 2 | 0 | 3 | 2 |
| 1136 | Calcium homeostasis endoplasmic reticulum protein | IPI00333010 | 0.160 | 0.24749 | 2 | 0 | 3 | 0 |
| 1137 | 39S ribosomal protein L24, mitochondrial | IPI00514506 | 0.160 | 0.24749 | 0 | 2 | 2 | 3 |
| 1138 | Isoform 3 of Guanine nucleotide exchange factor VAV2 | IPI00004977 | 0.160 | 0.24749 | 2 | 0 | 3 | 2 |
| 1139 | Isoform 3 of THO complex subunit 6 homolog | IPI00301252 | 0.160 | 0.24749 | 2 | 0 | 2 | 3 |
| 1140 | UPF0670 protein C8orf55 | IPI00171421 | 0.160 | 0.24749 | 0 | 0 | 0 | 3 |
| 1141 | cDNA FLJ12779 fis, clone NT2RP2001748 | IPI00902799 | 0.160 | 0.24749 | 0 | 0 | 3 | 0 |
| 1142 | Transmembrane protein 2 | IPI00170706 | 0.160 | 0.24749 | 0 | 0 | 3 | 0 |
| 1143 | Signal peptidase complex catalytic subunit SEC11C | IPI00219436 | 0.160 | 0.24749 | 0 | 2 | 2 | 3 |
| 1144 | E3 ubiquitin-protein ligase RNF181 | IPI00292354 | 0.160 | 0.24749 | 0 | 0 | 0 | 3 |
| 1145 | Isoform 2 of GPI ethanolamine phosphate transferase 3 | IPI00181620 | 0.160 | 0.24749 | 0 | 0 | 3 | 2 |
| 1146 | Isoform 2 of PERQ amino acid-rich with GYF domain-containing protein 2 | IPI00647635 | 0.160 | 0.24749 | 2 | 2 | 0 | 3 |
| 1147 | Isoform 3 of Cytosolic 5'-nucleotidase 3 | IPI00100192 | 0.160 | 0.24749 | 0 | 2 | 3 | 2 |
| 1148 | Copper chaperone for superoxide dismutase | IPI00021389 | 0.160 | 0.24749 | 0 | 0 | 2 | 3 |
| 1149 | Isoform Long of Acidic fibroblast growth factor intracellular-binding protein | IPI00012443 | 0.160 | 0.24749 | 0 | 0 | 0 | 3 |
| 1150 | Guanine nucleotide-binding protein subunit alpha-14 | IPI00000695 | 0.160 | 0.24749 | 0 | 0 | 3 | 0 |
| 1151 | Isoform 5 of Protein prune homolog | IPI00028177 | 0.160 | 0.24749 | 0 | 0 | 0 | 3 |
| 1152 | tRNA-splicing endonuclease subunit Sen15 | IPI00450071 | 0.160 | 0.24749 | 0 | 0 | 0 | 3 |
| 1153 | Pre-mRNA-splicing factor SYF1 | IPI00163084 | 0.160 | 0.24749 | 2 | 0 | 0 | 3 |
| 1154 | sulfatase modifying factor 2 isoform b precursor | IPI00171412 | 0.160 | 0.24749 | 2 | 0 | 3 | 0 |
| 1155 | Isoform 3 of Transcription elongation factor SPT6 | IPI00456683 | 0.160 | 0.24749 | 0 | 0 | 0 | 3 |
| 1156 | Prefoldin subunit 2 | IPI00006052 | 0.160 | 0.24749 | 0 | 0 | 0 | 3 |
| 1157 | Isoform 1 of Mixed lineage kinase domain-like protein | IPI00180781 | 0.160 | 0.24749 | 2 | 2 | 2 | 3 |
| 1158 | Isoform Long of Transformer-2 protein homolog alpha | IPI00013891 | 0.160 | 0.24749 | 2 | 2 | 0 | 3 |
| 1159 | Brefeldin A-inhibited guanine nucleotide-exchange protein 1 | IPI00002188 | 0.160 | 0.24749 | 0 | 0 | 2 | 3 |
| 1160 | Isoform 1 of Serine protease HTRA2, mitochondrial | IPI00001663 | 0.160 | 0.24749 | 0 | 2 | 2 | 3 |
| 1161 | cDNA FLJ61162, highly similar to Ras-related protein R-Ras2 | IPI00012512 | 0.160 | 0.24749 | 0 | 0 | 2 | 3 |
| 1162 | Dual specificity protein phosphatase 3 | IPI00018671 | 0.160 | 0.24749 | 2 | 2 | 2 | 3 |
| 1163 | Putative uncharacterized protein DOCK6 | IPI00184772 | 0.160 | 0.24749 | 0 | 0 | 0 | 3 |
| 1164 | Ras-related protein Rab-5B | IPI00017344 | 0.160 | 0.24749 | 0 | 0 | 0 | 3 |
| 1165 | DEAH (Asp-Glu-Ala-His) box polypeptide 16 | IPI00292510 | 0.160 | 0.24749 | 2 | 2 | 0 | 3 |
| 1166 | Isoform 1 of Alpha-adducin | IPI00019901 | 0.160 | 0.24749 | 0 | 2 | 0 | 3 |
| 1167 | Isoform A of Uncharacterized protein C21orf70 | IPI00027898 | 0.160 | 0.24749 | 2 | 2 | 3 | 2 |
| 1168 | Retinol dehydrogenase 14 | IPI00177940 | 0.160 | 0.24749 | 0 | 0 | 0 | 3 |
| 1169 | Protein AATF | IPI00302238 | 0.160 | 0.24749 | 0 | 2 | 0 | 3 |
| 1170 | 54 kDa protein | IPI00177890 | 0.160 | 0.24749 | 0 | 0 | 3 | 2 |
| 1171 | Zinc finger CCHC domain-containing protein 3 | IPI00011550 | 0.160 | 0.24749 | 0 | 0 | 2 | 3 |
| 1172 | Guanine nucleotide-binding protein G(i) subunit alpha-1 | IPI00337415 | 0.160 | 0.24749 | 2 | 0 | 0 | 3 |
| 1173 | Nucleoporin 54kDa variant (Fragment) | IPI00172580 | 0.160 | 0.24749 | 0 | 2 | 3 | 2 |
| 1174 | Exosome complex exonuclease RRP46 | IPI00015955 | 0.160 | 0.24749 | 0 | 0 | 3 | 0 |
| 1175 | Isoform 2 of ATPase WRNIP1 | IPI00102997 | 0.160 | 0.24749 | 0 | 0 | 0 | 3 |
| 1176 | Isoform 1 of Metaxin-1 | IPI00013678 | 0.160 | 0.24749 | 0 | 2 | 2 | 3 |
| 1177 | Integrin beta-5 | IPI00029741 | 0.160 | 0.24749 | 0 | 0 | 3 | 0 |
| 1178 | dehydrogenase/reductase SDR family member 4 | IPI00106913 | 0.160 | 0.24749 | 0 | 2 | 0 | 3 |
| 1179 | DNA-directed RNA polymerase III subunit RPC1 | IPI00024163 | 0.160 | 0.24749 | 0 | 0 | 0 | 3 |
| 1180 | cDNA FLJ54710, highly similar to Target of Myb protein 1 | IPI00023191 | 0.160 | 0.24749 | 0 | 0 | 0 | 3 |
| 1181 | Armadillo repeat-containing protein 6 | IPI00020196 | 0.160 | 0.24749 | 0 | 2 | 0 | 3 |
| 1182 | Isoform 3 of Serine/threonine-protein kinase SMG1 | IPI00183368 | 0.160 | 0.24749 | 0 | 0 | 0 | 3 |
| 1183 | Ribonuclease P protein subunit p20 | IPI00027142 | 0.160 | 0.24749 | 0 | 0 | 3 | 0 |
| 1184 | Isoform 1 of Protein dpy-19 homolog 1 | IPI00007461 | 0.160 | 0.24749 | 0 | 0 | 2 | 3 |
| 1185 | Polymerase delta interacting protein 46 | IPI00429180 | 0.160 | 0.24749 | 2 | 2 | 3 | 2 |
| 1186 | Protein S100-A16 | IPI00062120 | 0.160 | 0.24749 | 0 | 0 | 0 | 3 |
| 1187 | alpha-methylacyl-CoA racemase isoform 3 | IPI00005918 | 0.160 | 0.24749 | 0 | 2 | 3 | 2 |
| 1188 | Casein kinase I isoform alpha-like | IPI00167096 | 0.160 | 0.24749 | 0 | 0 | 3 | 0 |
| 1189 | Transcription initiation factor IIB | IPI00022820 | 0.160 | 0.24749 | 0 | 0 | 3 | 2 |
| 1190 | Methylcrotonoyl-CoA carboxylase subunit alpha, mitochondrial | IPI00024580 | 0.160 | 0.24749 | 0 | 0 | 0 | 3 |
| 1191 | Isoform 1 of Malonyl-CoA-acyl carrier protein transacylase, mitochondrial | IPI00023359 | 0.160 | 0.24749 | 0 | 0 | 0 | 3 |
| 1192 | Apolipoprotein O-like | IPI00394809 | 0.160 | 0.24749 | 2 | 0 | 3 | 0 |
| 1193 | Isoform 1 of Polymerase I and transcript release factor | IPI00176903 | 0.160 | 0.24749 | 2 | 0 | 3 | 0 |
| 1194 | Ubiquitin-conjugating enzyme E2 R2 | IPI00418603 | 0.160 | 0.24749 | 2 | 0 | 2 | 3 |
| 1195 | Isoform 4 of E3 ubiquitin-protein ligase UBR2 | IPI00217407 | 0.160 | 0.24749 | 0 | 0 | 0 | 3 |
| 1196 | Profilin | IPI00107555 | 0.160 | 0.24749 | 0 | 2 | 0 | 3 |
| 1197 | Isoform 1 of Glutaminase kidney isoform, mitochondrial | IPI00289159 | 0.160 | 0.24749 | 0 | 0 | 3 | 0 |
| 1198 | Isoform 1 of Wings apart-like protein homolog | IPI00375330 | 0.160 | 0.24749 | 0 | 0 | 0 | 3 |
| 1199 | V-type proton ATPase subunit G 1 | IPI00025285 | 0.160 | 0.24749 | 2 | 0 | 3 | 0 |
| 1200 | Isoform II of Ubiquitin-protein ligase E3A | IPI00011609 | 0.160 | 0.24749 | 2 | 0 | 2 | 3 |
| 1201 | Isoform 1 of Autophagy-related protein 9A | IPI00383396 | 0.160 | 0.24749 | 0 | 0 | 0 | 3 |
| 1202 | Isoform 2 of Serine/threonine-protein kinase PAK 1 | IPI00289746 | 0.160 | 0.24749 | 0 | 0 | 2 | 3 |
| 1203 | Isoform 1 of Dynamin-1 | IPI00413140 | 0.160 | 0.24749 | 0 | 0 | 2 | 3 |
| 1204 | cDNA FLJ56343, highly similar to Torsin A | IPI00413293 | 0.160 | 0.24749 | 0 | 0 | 3 | 0 |
| 1205 | cDNA FLJ54836 | IPI00063160 | 0.160 | 0.24749 | 0 | 0 | 0 | 3 |
| 1206 | Isoform 2 of V-type proton ATPase 116 kDa subunit a isoform 1 | IPI00743576 | 0.160 | 0.24749 | 0 | 0 | 0 | 3 |
| 1207 | mesencephalic astrocyte-derived neurotrophic factor | IPI00328748 | 0.160 | 0.24749 | 0 | 0 | 0 | 3 |
| 1208 | Isoform 1 of Ubiquinone biosynthesis protein COQ9, mitochondrial | IPI00470631 | 0.160 | 0.24749 | 0 | 0 | 0 | 3 |
| 1209 | WD repeat-containing protein 81 isoform 1 | IPI00917671 | 0.160 | 0.24749 | 0 | 0 | 0 | 3 |
| 1210 | Propionyl-CoA carboxylase beta chain, mitochondrial | IPI00007247 | 0.160 | 0.24749 | 0 | 0 | 2 | 3 |
| 1211 | FLJ00369 protein (Fragment) | IPI00166711 | 0.160 | 0.24749 | 0 | 0 | 3 | 0 |
| 1212 | Exportin-6 | IPI00465296 | 0.160 | 0.24749 | 0 | 0 | 0 | 3 |
| 1213 | cDNA FLJ12528 fis, clone NT2RM4000155, moderately similar to THREONYL-TRNA SYNTHETASE | IPI00018632 | 0.160 | 0.24749 | 0 | 2 | 0 | 3 |
| 1214 | Methylmalonate-semialdehyde dehydrogenase [acylating], mitochondria | IPI00024990 | 0.160 | 0.24749 | 0 | 0 | 0 | 3 |
| 1215 | Isoform B of Arfaptin-1 | IPI00021258 | 0.160 | 0.24749 | 0 | 0 | 0 | 3 |
| 1216 | Isoform 2 of Ribonuclease P protein subunit p40 | IPI00332091 | 0.160 | 0.24749 | 0 | 0 | 0 | 3 |
| 1217 | Isoform 1 of Ubiquitin-like modifier-activating enzyme 5 | IPI00015736 | 0.160 | 0.24749 | 0 | 0 | 3 | 2 |
| 1218 | Threonine synthase-like 1 | IPI00016287 | 0.160 | 0.24749 | 2 | 0 | 0 | 3 |
| 1219 | Putative uncharacterized protein DKFZp686E2459 | IPI00375731 | 0.160 | 0.24749 | 0 | 2 | 3 | 2 |
| 1220 | Translation initiation factor eIF-2B subunit beta | IPI00028083 | 0.160 | 0.24749 | 0 | 0 | 3 | 0 |
| 1221 | peroxisomal 3,2-trans-enoyl-CoA isomerase isoform 1 | IPI00419263 | 0.160 | 0.24749 | 0 | 0 | 0 | 3 |
| 1222 | Mediator of RNA polymerase II transcription subunit 13 | IPI00021388 | 0.160 | 0.24749 | 0 | 0 | 2 | 3 |
| 1223 | Isoform 1 of 28S ribosomal protein S5, mitochondrial | IPI00169400 | 0.160 | 0.24749 | 0 | 2 | 0 | 3 |
| 1224 | Exocyst complex component 2 | IPI00783559 | 0.160 | 0.24749 | 0 | 0 | 3 | 2 |
| 1225 | RNA polymerase-associated protein CTR9 homolog | IPI00477468 | 0.160 | 0.24749 | 0 | 0 | 0 | 3 |
| 1226 | Isoform 2 of WASH complex subunit FAM21C | IPI00456853 | 0.160 | 0.24749 | 0 | 0 | 3 | 0 |
| 1227 | Vasodilator-stimulated phosphoprotein | IPI00301058 | 0.160 | 0.24749 | 0 | 0 | 3 | 0 |

| No. | Description | Accession number | STN[1] | p-Value[1] | Con_A[2] | Con_B[2] | RG3_A[2] | RG3_B[2] |
|---|---|---|---|---|---|---|---|---|
| 1228 | Isoform 1 of Transmembrane protein 55B | IPI00030530 | 0.160 | 0.24749 | 0 | 2 | 0 | 3 |
| 1229 | Lipoamide acyltransferase component of branched-chain alpha-keto acid dehydrogenase complex | IPI00003944 | 0.160 | 0.24749 | 0 | 2 | 0 | 3 |
| 1230 | Interferon regulatory factor 2-binding protein 1 | IPI00645608 | 0.160 | 0.24749 | 0 | 0 | 0 | 3 |
| 1231 | Methylosome subunit pICln | IPI00004795 | 0.160 | 0.24749 | 0 | 0 | 0 | 3 |
| 1232 | RhoA activator C11orf59 | IPI00016670 | 0.160 | 0.24749 | 2 | 2 | 0 | 3 |
| 1233 | Laminin subunit beta-1 | IPI00013976 | 0.160 | 0.24749 | 0 | 0 | 0 | 3 |
| 1234 | 39S ribosomal protein L41, mitochondrial | IPI00217553 | 0.160 | 0.24749 | 0 | 0 | 0 | 3 |
| 1235 | Putative transferase C1orf69, mitochondrial | IPI00145260 | 0.160 | 0.24749 | 0 | 0 | 3 | 0 |
| 1236 | Isoform 1 of RNA polymerase II-associated protein 1 | IPI00402657 | 0.160 | 0.24749 | 0 | 0 | 0 | 3 |
| 1237 | Guanine nucleotide exchange factor MSS4 | IPI00023939 | 0.160 | 0.24749 | 0 | 0 | 0 | 3 |
| 1238 | Acyl-coenzyme A thioesterase 8 | IPI00298202 | 0.160 | 0.24749 | 0 | 0 | 0 | 3 |
| 1239 | Isoform 1 of RNA polymerase-associated protein LEO1 | IPI00103090 | 0.160 | 0.24749 | 0 | 0 | 0 | 3 |
| 1240 | Putative uncharacterized protein DKFZp686G0859 | IPI00470477 | 0.160 | 0.24749 | 0 | 0 | 0 | 3 |
| 1241 | Secernin-2 | IPI00062266 | 0.160 | 0.24749 | 0 | 0 | 0 | 3 |
| 1242 | Isoform 1 of UPF0557 protein C10orf119 | IPI00478758 | 0.160 | 0.24749 | 0 | 0 | 3 | 0 |
| 1243 | Isoform 2 of NAD-dependent deacetylase sirtuin-5 | IPI00010331 | 0.160 | 0.24749 | 0 | 0 | 3 | 0 |
| 1244 | ATP synthase subunit delta, mitochondrial | IPI00024920 | 0.160 | 0.24749 | 0 | 0 | 3 | 0 |
| 1245 | Isoform Mitochondrial of Cysteine desulfurase, mitochondrial | IPI00295240 | 0.160 | 0.24749 | 0 | 0 | 0 | 3 |
| 1246 | Proteasome assembly chaperone 4 | IPI00895892 | 0.160 | 0.24749 | 0 | 0 | 0 | 3 |
| 1247 | Succinate-semialdehyde dehydrogenase, mitochondrial | IPI00019888 | 0.160 | 0.24749 | 0 | 0 | 0 | 3 |
| 1248 | Isoform 2 of Uncharacterized protein C3orf21 | IPI00165665 | 0.160 | 0.24749 | 0 | 0 | 0 | 3 |
| 1249 | NHP2-like protein 1 | IPI00026167 | 0.160 | 0.24749 | 2 | 0 | 3 | 0 |
| 1250 | Isoform 1 of Multivesicular body subunit 12A | IPI00744702 | 0.160 | 0.24749 | 0 | 0 | 0 | 3 |
| 1251 | Isoform Alpha of Caspase-6 | IPI00023876 | 0.160 | 0.24749 | 0 | 0 | 0 | 3 |
| 1252 | Gem-associated protein 7 | IPI00003027 | 0.160 | 0.24749 | 0 | 0 | 0 | 3 |
| 1253 | Isoform 6 of Ribosome-recycling factor, mitochondrial | IPI00030596 | 0.160 | 0.24749 | 0 | 2 | 0 | 3 |
| 1254 | Isoform 2 of Torsin-1A-interacting protein 1 | IPI00012280 | 0.160 | 0.24749 | 0 | 0 | 0 | 3 |
| 1255 | Microtubule-associated protein 1S | IPI00296485 | 0.160 | 0.24749 | 0 | 0 | 0 | 3 |
| 1256 | Isoform 2 of SWI/SNF complex subunit SMARCC2 | IPI00150057 | 0.159 | 0.26644 | 22 | 18 | 26 | 16 |
| 1257 | Plastin-3 | IPI00216694 | 0.158 | 0.26669 | 18 | 23 | 16 | 27 |
| 1258 | Isoform Long of Trifunctional purine biosynthetic protein adenosine-3 | IPI00025273 | 0.155 | 0.26681 | 19 | 24 | 15 | 30 |
| 1259 | Isoform 1 of Cytosolic acyl coenzyme A thioester hydrolase | IPI00010415 | 0.153 | 0.26710 | 25 | 20 | 24 | 23 |
| 1260 | 26S protease regulatory subunit 6A | IPI00018398 | 0.151 | 0.26719 | 19 | 28 | 25 | 24 |
| 1261 | fatty acid desaturase 1 | IPI00784651 | 0.150 | 0.26723 | 3 | 0 | 3 | 3 |
| 1262 | Ribosomal protein S27 | IPI00514399 | 0.150 | 0.26723 | 3 | 2 | 4 | 2 |
| 1263 | Fatty acid-binding protein, epidermal | IPI00007797 | 0.150 | 0.26723 | 0 | 3 | 0 | 4 |
| 1264 | Protein VAC14 homolog | IPI00025160 | 0.150 | 0.26723 | 3 | 0 | 2 | 4 |
| 1265 | Ras-related C3 botulinum toxin substrate 2 | IPI00010270 | 0.150 | 0.26723 | 3 | 0 | 3 | 3 |
| 1266 | Isoform 2 of Triple functional domain protein | IPI00479523 | 0.150 | 0.26723 | 3 | 2 | 0 | 4 |
| 1267 | ATPase ASNA1 | IPI00013466 | 0.150 | 0.26723 | 2 | 3 | 0 | 4 |
| 1268 | Isoform 1 of Zinc finger protein 207 | IPI00013457 | 0.150 | 0.26723 | 3 | 2 | 3 | 3 |
| 1269 | Protein tyrosine phosphatase type IVA 1 | IPI00020164 | 0.150 | 0.26723 | 2 | 3 | 2 | 4 |
| 1270 | cDNA FLJ60317, highly similar to Aminoacylase-1 | IPI00009268 | 0.150 | 0.26723 | 3 | 2 | 3 | 3 |
| 1271 | Trafficking protein particle complex subunit 3 | IPI00004324 | 0.150 | 0.26723 | 0 | 3 | 0 | 4 |
| 1272 | Aspartyl-tRNA synthetase, mitochondrial | IPI00100460 | 0.150 | 0.26723 | 0 | 3 | 2 | 4 |
| 1273 | Isoform 1 of Splicing factor, arginine/serine-rich 15 | IPI00181702 | 0.150 | 0.26723 | 0 | 3 | 2 | 4 |
| 1274 | Replication protein A 14 kDa subunit | IPI00017373 | 0.150 | 0.26723 | 2 | 3 | 0 | 4 |
| 1275 | Isoform 2 of Protein FAM36A | IPI00103057 | 0.150 | 0.26723 | 3 | 0 | 4 | 0 |
| 1276 | Isoform 1 of Exosome component 10 | IPI00009464 | 0.150 | 0.26723 | 2 | 3 | 3 | 3 |
| 1277 | Probable histidyl-tRNA synthetase, mitochondrial | IPI00027445 | 0.150 | 0.26723 | 0 | 3 | 0 | 4 |
| 1278 | 14 kDa phosphohistidine phosphatase | IPI00299977 | 0.150 | 0.26723 | 2 | 3 | 3 | 3 |
| 1279 | Golgin subfamily B member 1 | IPI00004671 | 0.150 | 0.26723 | 3 | 2 | 4 | 0 |
| 1280 | Isoform 1 of E3 ubiquitin-protein ligase BRE1B | IPI00162563 | 0.150 | 0.26723 | 2 | 3 | 3 | 3 |
| 1281 | Isoform 1 of ATP synthase subunit d, mitochondrial | IPI00220487 | 0.150 | 0.26723 | 0 | 3 | 2 | 4 |
| 1282 | Toll-interacting protein | IPI00100154 | 0.150 | 0.26723 | 3 | 0 | 3 | 3 |
| 1283 | 39S ribosomal protein L14, mitochondrial | IPI00418290 | 0.150 | 0.26723 | 0 | 3 | 3 | 3 |
| 1284 | Isoform 1 of Melanoma-associated antigen D2 | IPI00009542 | 0.150 | 0.26723 | 3 | 0 | 3 | 3 |
| 1285 | U6 snRNA-associated Sm-like protein LSm2 | IPI00032460 | 0.150 | 0.26723 | 3 | 0 | 3 | 3 |
| 1286 | Transmembrane protein C3orf1 | IPI00299387 | 0.150 | 0.26723 | 0 | 3 | 3 | 3 |
| 1287 | Mimitin, mitochondrial | IPI00031109 | 0.150 | 0.26723 | 0 | 3 | 3 | 3 |
| 1288 | Isoform 3 of Exocyst complex component 7 | IPI00103064 | 0.150 | 0.26723 | 3 | 0 | 2 | 4 |
| 1289 | Protein FRG1 | IPI00004655 | 0.150 | 0.26723 | 0 | 3 | 3 | 3 |
| 1290 | 39S ribosomal protein L53, mitochondrial | IPI00061531 | 0.150 | 0.26723 | 3 | 0 | 4 | 2 |
| 1291 | Isoform 1 of Far upstream element-binding protein 3 | IPI00377261 | 0.150 | 0.26723 | 3 | 0 | 4 | 0 |
| 1292 | Isoform 1 of Transmembrane protein 85 | IPI00009320 | 0.150 | 0.26723 | 0 | 3 | 0 | 4 |
| 1293 | Ketosamine-3-kinase | IPI00099986 | 0.150 | 0.26723 | 0 | 3 | 0 | 4 |
| 1294 | Thioredoxin-like protein 4A | IPI00216338 | 0.150 | 0.26723 | 0 | 3 | 0 | 4 |
| 1295 | Golgin subfamily A member 7 | IPI00480022 | 0.150 | 0.26723 | 0 | 3 | 3 | 3 |
| 1296 | Isoform 1 of NADH dehydrogenase [ubiquinone] flavoprotein 1, mitochondrial | IPI00028520 | 0.150 | 0.26723 | 0 | 3 | 0 | 4 |
| 1297 | Valyl-tRNA synthetase | IPI00000873 | 0.146 | 0.27806 | 26 | 27 | 30 | 25 |
| 1298 | Isoform 1 of Myoferlin | IPI00021048 | 0.146 | 0.27806 | 30 | 23 | 24 | 31 |
| 1299 | Isoform 1 of Elongation factor 1-delta | IPI00023048 | 0.145 | 0.27806 | 32 | 22 | 34 | 22 |
| 1300 | 40S ribosomal protein S13 | IPI00221089 | 0.143 | 0.27819 | 37 | 19 | 38 | 20 |
| 1301 | Isoform 1 of CCR4-NOT transcription complex subunit 1 | IPI00166010 | 0.142 | 0.27827 | 4 | 2 | 3 | 4 |
| 1302 | Isoform 1 of Nuclear pore complex protein Nup98-Nup96 | IPI00006038 | 0.142 | 0.27827 | 3 | 3 | 3 | 4 |
| 1303 | cDNA FLJ55475 | IPI00306017 | 0.142 | 0.27827 | 0 | 4 | 3 | 4 |
| 1304 | 39S ribosomal protein L40, mitochondrial | IPI00099871 | 0.142 | 0.27827 | 2 | 4 | 3 | 4 |
| 1305 | Isoform F of Protein SON | IPI00000192 | 0.142 | 0.27827 | 4 | 2 | 2 | 5 |
| 1306 | Ubiquitin-conjugating enzyme E2 G1 | IPI00219783 | 0.142 | 0.27827 | 3 | 3 | 4 | 3 |
| 1307 | Isoform 1 of Nck-associated protein 1 | IPI00031982 | 0.142 | 0.27827 | 0 | 4 | 3 | 4 |
| 1308 | Glycylpeptide N-tetradecanoyltransferase 2 | IPI00030223 | 0.142 | 0.27827 | 0 | 4 | 3 | 4 |
| 1309 | Scaffold attachment factor B2 | IPI00005648 | 0.142 | 0.27827 | 2 | 4 | 4 | 3 |
| 1310 | NDUFB10 protein | IPI00074489 | 0.142 | 0.27827 | 4 | 0 | 4 | 3 |
| 1311 | [Pyruvate dehydrogenase [lipoamide]] kinase isozyme 3, mitochondrial | IPI00014849 | 0.142 | 0.27827 | 0 | 4 | 0 | 5 |
| 1312 | Isoform 1 of COMM domain-containing protein 4 | IPI00413500 | 0.142 | 0.27827 | 0 | 4 | 3 | 4 |
| 1313 | Immunoglobulin-binding protein 1 | IPI00019148 | 0.142 | 0.27827 | 3 | 3 | 2 | 5 |
| 1314 | Isoform 3 of Sorting nexin-3 | IPI00029740 | 0.142 | 0.27827 | 0 | 4 | 3 | 4 |
| 1315 | U8 snoRNA-decapping enzyme | IPI00783497 | 0.142 | 0.27827 | 2 | 4 | 0 | 5 |
| 1316 | Peptidyl-tRNA hydrolase 2, mitochondrial | IPI00032903 | 0.142 | 0.27827 | 0 | 4 | 2 | 5 |
| 1317 | Glutathione S-transferase theta-1 | IPI00741097 | 0.142 | 0.27827 | 2 | 4 | 3 | 4 |
| 1318 | CDGSH iron sulfur domain-containing protein 2 | IPI00166865 | 0.142 | 0.27827 | 0 | 4 | 2 | 5 |
| 1319 | NADH dehydrogenase [ubiquinone] 1 alpha subcomplex assembly factor 3 | IPI00399053 | 0.142 | 0.27827 | 4 | 0 | 5 | 0 |
| 1320 | 60S ribosomal protein L7 | IPI00030179 | 0.140 | 0.28350 | 36 | 24 | 35 | 27 |
| 1321 | Isoform 1 of Proteasome subunit alpha type-7 | IPI00024175 | 0.139 | 0.28362 | 40 | 21 | 35 | 28 |
| 1322 | Isoform A1 of Tight junction protein ZO-2 | IPI00003843 | 0.136 | 0.28421 | 2 | 5 | 6 | 2 |

| No. | Description | Accession number | STN[1] | p-Value[1] | Con_A[2] | Con_B[2] | RG3_A[2] | RG3_B[2] |
|---|---|---|---|---|---|---|---|---|
| 1323 | GTP-binding protein Rheb | IPI00016669 | 0.136 | 0.28421 | 2 | 5 | 0 | 6 |
| 1324 | cDNA FLJ14239 fis, clone NT2RP5003512, highly similar to Exportin-5 | IPI00549861 | 0.136 | 0.28421 | 4 | 3 | 3 | 5 |
| 1325 | Pyruvate carboxylase, mitochondrial | IPI00299402 | 0.136 | 0.28421 | 3 | 4 | 4 | 4 |
| 1326 | G-rich sequence factor 1 | IPI00478657 | 0.136 | 0.28421 | 3 | 4 | 4 | 4 |
| 1327 | Transcription elongation factor B polypeptide 2 | IPI00026670 | 0.136 | 0.28421 | 4 | 3 | 5 | 3 |
| 1328 | Histidine triad nucleotide-binding protein 2, mitochondrial | IPI00000335 | 0.136 | 0.28421 | 2 | 5 | 3 | 5 |
| 1329 | Selenide, water dikinase 1 | IPI00029056 | 0.136 | 0.28421 | 2 | 5 | 4 | 4 |
| 1330 | Lysosomal Pro-X carboxypeptidase | IPI00001593 | 0.136 | 0.28421 | 3 | 4 | 2 | 6 |
| 1331 | Catechol O-methyltransferase domain-containing protein 1 | IPI00642041 | 0.136 | 0.28421 | 0 | 5 | 0 | 6 |
| 1332 | Ewing sarcoma breakpoint region 1 isoform 1 | IPI00009841 | 0.136 | 0.28421 | 4 | 3 | 4 | 4 |
| 1333 | Isoform 4 of Nucleoporin NDC1 | IPI00003455 | 0.136 | 0.28421 | 4 | 3 | 4 | 4 |
| 1334 | Acyl-coenzyme A thioesterase 13 | IPI00020530 | 0.136 | 0.28421 | 3 | 4 | 3 | 5 |
| 1335 | V-type proton ATPase subunit F | IPI00004488 | 0.136 | 0.28421 | 3 | 4 | 3 | 5 |
| 1336 | Ras-related protein Rap-2c | IPI00009607 | 0.136 | 0.28421 | 2 | 5 | 2 | 6 |
| 1337 | 22 kDa protein | IPI00219910 | 0.136 | 0.28421 | 4 | 3 | 4 | 4 |
| 1338 | Ubiquitin domain-containing protein UBFD1 | IPI00005194 | 0.136 | 0.28421 | 4 | 3 | 4 | 4 |
| 1339 | Isoform 1 of Metallo-beta-lactamase domain-containing protein 2 | IPI00293336 | 0.136 | 0.28421 | 5 | 0 | 5 | 3 |
| 1340 | Isoform 1 of Myb-binding protein 1A | IPI00005024 | 0.134 | 0.28789 | 34 | 35 | 32 | 39 |
| 1341 | Isoform 1 of Apoptosis-inducing factor 1, mitochondrial | IPI00000690 | 0.132 | 0.28835 | 47 | 26 | 37 | 38 |
| 1342 | Anaphase-promoting complex subunit 1 | IPI00033907 | 0.131 | 0.28860 | 3 | 5 | 2 | 7 |
| 1343 | Isoform 1 of Cleavage stimulation factor subunit 2 | IPI00013256 | 0.131 | 0.28860 | 4 | 4 | 0 | 7 |
| 1344 | Isoform 1 of Polyadenylate-binding protein 4 | IPI00012726 | 0.131 | 0.28860 | 4 | 4 | 4 | 5 |
| 1345 | Mitochondrial import inner membrane translocase subunit Tim23 | IPI00007309 | 0.131 | 0.28860 | 3 | 5 | 3 | 6 |
| 1346 | ubiquitin-like protein fubi and ribosomal protein S30 precursor | IPI00019770 | 0.131 | 0.28860 | 3 | 5 | 4 | 5 |
| 1347 | Pyridoxine-5'-phosphate oxidase | IPI00018272 | 0.131 | 0.28860 | 5 | 3 | 5 | 4 |
| 1348 | Immature colon carcinoma transcript 1 protein | IPI00029114 | 0.131 | 0.28860 | 5 | 3 | 6 | 3 |
| 1349 | 39S ribosomal protein L20, mitochondrial | IPI00013706 | 0.131 | 0.28860 | 4 | 4 | 4 | 5 |
| 1350 | Nuclear RNA export factor 1 | IPI00033153 | 0.131 | 0.28860 | 2 | 6 | 5 | 4 |
| 1351 | Signal peptidase complex subunit 3 | IPI00300299 | 0.131 | 0.28860 | 5 | 3 | 5 | 4 |
| 1352 | Eukaryotic peptide chain release factor GTP-binding subunit ERF3A | IPI00218829 | 0.126 | 0.29353 | 7 | 0 | 7 | 3 |
| 1353 | U6 snRNA-associated Sm-like protein LSm4 | IPI00294955 | 0.126 | 0.29353 | 4 | 5 | 6 | 4 |
| 1354 | 39S ribosomal protein L23, mitochondrial | IPI00293476 | 0.126 | 0.29353 | 4 | 5 | 6 | 4 |
| 1355 | Serine/threonine-protein phosphatase 5 | IPI00019812 | 0.126 | 0.29353 | 2 | 7 | 4 | 6 |
| 1356 | Integrin-linked protein kinase | IPI00013219 | 0.126 | 0.29353 | 4 | 5 | 5 | 5 |
| 1357 | Copine-3 | IPI00024403 | 0.126 | 0.29353 | 5 | 4 | 6 | 4 |
| 1358 | Isoform 1 of TP53RK-binding protein | IPI00301432 | 0.126 | 0.29353 | 3 | 6 | 4 | 6 |
| 1359 | Isoform 1 of Syntenin-1 | IPI00299086 | 0.126 | 0.29353 | 2 | 7 | 4 | 6 |
| 1360 | Pre-rRNA-processing protein TSR1 homolog | IPI00292894 | 0.126 | 0.29353 | 3 | 6 | 4 | 6 |
| 1361 | Bifunctional purine biosynthesis protein PURH | IPI00289499 | 0.125 | 0.29567 | 49 | 37 | 42 | 46 |
| 1362 | ADP-ribosylation factor 1 | IPI00215914 | 0.124 | 0.29575 | 163 | 160 | 155 | 171 |
| 1363 | Platelet-activating factor acetylhydrolase IB subunit gamma | IPI00014808 | 0.122 | 0.29579 | 5 | 5 | 6 | 5 |
| 1364 | Cell division cycle 5-like protein | IPI00465294 | 0.122 | 0.29579 | 5 | 5 | 4 | 7 |
| 1365 | Isoform 2 of Serine/threonine-protein kinase PAK 3 | IPI00027382 | 0.122 | 0.29579 | 4 | 6 | 5 | 6 |
| 1366 | Isoform 1 of BAG family molecular chaperone regulator 5 | IPI00007731 | 0.122 | 0.29579 | 0 | 8 | 5 | 6 |
| 1367 | Acylamino-acid-releasing enzyme | IPI00337741 | 0.119 | 0.29977 | 6 | 5 | 8 | 4 |
| 1368 | Isoform 2 of Histone deacetylase 2 | IPI00289601 | 0.119 | 0.29977 | 5 | 6 | 8 | 4 |
| 1369 | Biliverdin reductase A | IPI00294158 | 0.119 | 0.29977 | 6 | 5 | 5 | 7 |
| 1370 | DNA-directed RNA polymerases I, II, and III subunit RPABC3 | IPI00003309 | 0.119 | 0.29977 | 5 | 6 | 5 | 7 |
| 1371 | NADH dehydrogenase [ubiquinone] iron-sulfur protein 4, mitochondrial | IPI00011217 | 0.116 | 0.30211 | 5 | 7 | 5 | 8 |
| 1372 | Isoform 2 of Isopentenyl-diphosphate Delta-isomerase 1 | IPI00220014 | 0.116 | 0.30211 | 5 | 7 | 7 | 6 |
| 1373 | UPF0553 protein C9orf64 | IPI00170972 | 0.116 | 0.30211 | 4 | 8 | 7 | 6 |
| 1374 | CSNK2A1 protein | IPI00016613 | 0.113 | 0.30432 | 3 | 10 | 3 | 11 |
| 1375 | Isoform 1 of Enolase-phosphatase E1 | IPI00038378 | 0.113 | 0.30432 | 8 | 5 | 9 | 5 |
| 1376 | Splicing factor, arginine/serine-rich 9 | IPI00012340 | 0.113 | 0.30432 | 7 | 6 | 6 | 8 |
| 1377 | Phosphoribosyl pyrophosphate synthase-associated protein 2 | IPI00003168 | 0.113 | 0.30432 | 2 | 11 | 6 | 8 |
| 1378 | Isoform 2 of Ubiquitin thioesterase OTUB1 | IPI00409750 | 0.113 | 0.30432 | 5 | 8 | 8 | 6 |
| 1379 | RNA binding motif protein, X-linked-like 1 | IPI00061178 | 0.113 | 0.30432 | 8 | 5 | 9 | 5 |
| 1380 | 51 kDa protein | IPI00033025 | 0.110 | 0.30562 | 7 | 7 | 6 | 9 |
| 1381 | Protein NipSnap homolog 3A | IPI00004845 | 0.110 | 0.30562 | 5 | 9 | 9 | 6 |
| 1382 | Isoform Mitochondrial of Glutathione reductase, mitochondrial | IPI00016862 | 0.108 | 0.30688 | 10 | 5 | 7 | 9 |
| 1383 | Isoform 4 of Serine/threonine-protein phosphatase 6 regulatory subunit 3 | IPI00019540 | 0.108 | 0.30688 | 7 | 8 | 7 | 9 |
| 1384 | 39S ribosomal protein L19, mitochondrial | IPI00027096 | 0.108 | 0.30688 | 6 | 9 | 6 | 10 |
| 1385 | Isoform 2 of Tropomyosin alpha-3 chain | IPI00218319 | 0.106 | 0.30746 | 9 | 7 | 9 | 8 |
| 1386 | Paladin | IPI00297212 | 0.106 | 0.30746 | 8 | 8 | 9 | 8 |
| 1387 | myosin regulatory light polypeptide 9 isoform b | IPI00030929 | 0.106 | 0.30746 | 7 | 9 | 10 | 7 |
| 1388 | Ribosome biogenesis protein WDR12 | IPI00304232 | 0.106 | 0.30746 | 8 | 8 | 8 | 9 |
| 1389 | Transcription factor A, mitochondrial | IPI00020928 | 0.104 | 0.30909 | 5 | 12 | 8 | 10 |
| 1390 | SWI/SNF complex subunit SMARCC1 | IPI00234252 | 0.104 | 0.30909 | 8 | 9 | 9 | 9 |
| 1391 | Neprilysin | IPI00247063 | 0.104 | 0.30909 | 10 | 7 | 6 | 12 |
| 1392 | Protein kinase, cAMP-dependent, regulatory, type II, alpha, isoform CRA_b | IPI00063234 | 0.104 | 0.30909 | 8 | 9 | 10 | 8 |
| 1393 | Malectin | IPI00029046 | 0.102 | 0.31026 | 9 | 9 | 8 | 11 |
| 1394 | 14-3-3 protein epsilon | IPI00000816 | 0.102 | 0.31102 | 324 | 274 | 340 | 261 |
| 1395 | Dolichol-phosphate mannosyltransferase | IPI00022018 | 0.100 | 0.31102 | 9 | 10 | 14 | 6 |
| 1396 | Similar to nonhistone chromosomal protein HMG-1 | IPI00418184 | 0.100 | 0.31102 | 13 | 6 | 14 | 6 |
| 1397 | NADH dehydrogenase [ubiquinone] iron-sulfur protein 8, mitochondrial | IPI00010845 | 0.099 | 0.31156 | 8 | 12 | 12 | 9 |
| 1398 | cDNA FLJ55177, highly similar to Ras-related protein Ral-B | IPI00004397 | 0.099 | 0.31156 | 9 | 11 | 10 | 11 |
| 1399 | Putative uncharacterized protein ENSP00000350479 | IPI00069693 | 0.097 | 0.31248 | 16 | 5 | 18 | 4 |
| 1400 | Isoform 1 of 5'(3')-deoxyribonucleotidase, cytosolic type | IPI00005573 | 0.097 | 0.31248 | 11 | 10 | 11 | 11 |
| 1401 | Protein of unknown function DUF410 family protein | IPI00419575 | 0.096 | 0.31323 | 10 | 12 | 11 | 12 |
| 1402 | Wolframin | IPI00008711 | 0.095 | 0.31424 | 11 | 12 | 12 | 12 |
| 1403 | Isoform 1 of Nuclear pore complex protein Nup155 | IPI00026625 | 0.095 | 0.31424 | 10 | 13 | 10 | 14 |
| 1404 | Vesicle-trafficking protein SEC22b | IPI00006865 | 0.095 | 0.31424 | 9 | 14 | 12 | 12 |
| 1405 | 60S ribosomal protein L27a | IPI00456758 | 0.093 | 0.31478 | 13 | 11 | 16 | 9 |
| 1406 | Prolyl endopeptidase | IPI00008164 | 0.092 | 0.31516 | 12 | 13 | 11 | 15 |
| 1407 | Isoform 1 of Structural maintenance of chromosomes protein 2 | IPI00007927 | 0.090 | 0.31570 | 14 | 13 | 13 | 15 |
| 1408 | DNA mismatch repair protein Msh2 | IPI00017303 | 0.090 | 0.31570 | 12 | 15 | 12 | 16 |
| 1409 | Tubulin--tyrosine ligase-like protein 12 | IPI00029048 | 0.088 | 0.31616 | 13 | 16 | 11 | 19 |
| 1410 | cDNA FLJ77422, highly similar to Homo sapiens RNA binding protein | IPI00011268 | 0.087 | 0.31645 | 16 | 14 | 17 | 14 |
| 1411 | Cytochrome c oxidase subunit 2 | IPI00017510 | 0.083 | 0.31783 | 17 | 18 | 21 | 15 |
| 1412 | Isoform 2 of Annexin A2 | IPI00418169 | 0.083 | 0.31783 | 21 | 14 | 17 | 19 |
| 1413 | Putative uncharacterized protein NAP1L4 | IPI00017763 | 0.082 | 0.31800 | 22 | 15 | 21 | 17 |
| 1414 | ADP-ribosylation factor 6 | IPI00215920 | 0.082 | 0.31800 | 20 | 17 | 19 | 19 |
| 1415 | Isoform 5 of Dynamin-1-like protein | IPI00037283 | 0.078 | 0.31875 | 16 | 26 | 19 | 24 |
| 1416 | ADP-ribosylation factor 4 | IPI00215918 | 0.077 | 0.31875 | 26 | 18 | 29 | 16 |
| 1417 | 26S proteasome non-ATPase regulatory subunit 2 | IPI00012268 | 0.076 | 0.31896 | 21 | 25 | 27 | 20 |

| No. | Description | Accession number | STN[1] | p-Value[1] | Con_A[2] | Con_B[2] | RG3_A[2] | RG3_B[2] |
|---|---|---|---|---|---|---|---|---|
| 1418 | Proteasome subunit alpha type-2 | IPI00219622 | 0.071 | 0.31980 | 29 | 28 | 35 | 23 |
| 1419 | 40S ribosomal protein S17 | IPI00221093 | 0.064 | 0.32160 | 49 | 31 | 49 | 32 |
| 1420 | Isoform 1 of Vinculin | IPI00291175 | 0.061 | 0.32214 | 46 | 49 | 46 | 50 |
| 1421 | C-1-tetrahydrofolate synthase, cytoplasmic | IPI00218342 | 0.058 | 0.32260 | 57 | 52 | 53 | 57 |
| 1422 | Splicing factor 3B subunit 1 | IPI00026089 | 0.000 | 0.32285 | 39 | 42 | 36 | 45 |
| 1423 | Hypoxia up-regulated protein 1 | IPI00000877 | 0.000 | 0.32285 | 37 | 40 | 37 | 40 |
| 1424 | THO complex subunit 4 | IPI00328840 | 0.000 | 0.32285 | 51 | 30 | 51 | 30 |
| 1425 | ATP-citrate synthase | IPI00021290 | 0.000 | 0.32285 | 41 | 37 | 38 | 40 |
| 1426 | Radixin, isoform CRA_a | IPI00017367 | 0.000 | 0.32285 | 34 | 23 | 30 | 27 |
| 1427 | Multifunctional protein ADE2 | IPI00217223 | 0.000 | 0.32285 | 34 | 31 | 30 | 35 |
| 1428 | Tubulin beta-2A chain | IPI00013475 | 0.000 | 0.32285 | 22 | 18 | 19 | 21 |
| 1429 | Leukocyte elastase inhibitor | IPI00027444 | 0.000 | 0.32285 | 22 | 25 | 26 | 21 |
| 1430 | Isoform Short of Adenosine kinase | IPI00234368 | 0.000 | 0.32285 | 16 | 14 | 16 | 14 |
| 1431 | Isoform Complexed of Arginyl-tRNA synthetase, cytoplasmic | IPI00004860 | 0.000 | 0.32285 | 18 | 25 | 21 | 22 |
| 1432 | 26S proteasome non-ATPase regulatory subunit 7 | IPI00019927 | 0.000 | 0.32285 | 18 | 21 | 23 | 16 |
| 1433 | Adenylate kinase isoenzyme 1 | IPI00018342 | 0.000 | 0.32285 | 18 | 22 | 20 | 20 |
| 1434 | Flap endonuclease 1 | IPI00026215 | 0.000 | 0.32285 | 11 | 14 | 11 | 14 |
| 1435 | DNA-directed RNA polymerase II subunit RPB1 | IPI00031627 | 0.000 | 0.32285 | 18 | 16 | 10 | 24 |
| 1436 | Tricarboxylate transport protein, mitochondrial | IPI00294159 | 0.000 | 0.32285 | 14 | 15 | 19 | 10 |
| 1437 | ADP-ribosylation factor-like protein 1 | IPI00219518 | 0.000 | 0.32285 | 9 | 13 | 9 | 13 |
| 1438 | NADH-ubiquinone oxidoreductase 75 kDa subunit | IPI00604664 | 0.000 | 0.32285 | 15 | 11 | 13 | 13 |
| 1439 | Peptidyl-prolyl cis-trans isomerase FKBP11 | IPI00009885 | 0.000 | 0.32285 | 15 | 8 | 12 | 11 |
| 1440 | Isoform Beta-4C of Integrin beta-4 | IPI00027422 | 0.000 | 0.32285 | 17 | 16 | 20 | 13 |
| 1441 | Putative high mobility group protein 1-like 10 | IPI00018755 | 0.000 | 0.32285 | 15 | 5 | 15 | 5 |
| 1442 | Isoform 1 of Tryptophanyl-tRNA synthetase, cytoplasmic | IPI00295400 | 0.000 | 0.32285 | 11 | 19 | 13 | 17 |
| 1443 | Pre-mRNA-processing factor 19 | IPI00004968 | 0.000 | 0.32285 | 13 | 11 | 10 | 14 |
| 1444 | Mitochondrial 28S ribosomal protein S2 | IPI00006970 | 0.000 | 0.32285 | 12 | 0 | 12 | 0 |
| 1445 | Platelet-activating factor acetylhydrolase IB subunit beta | IPI00026546 | 0.000 | 0.32285 | 11 | 11 | 10 | 12 |
| 1446 | Coatomer subunit gamma | IPI00783982 | 0.000 | 0.32285 | 13 | 11 | 11 | 13 |
| 1447 | S-adenosylmethionine synthase isoform type-2 | IPI00010157 | 0.000 | 0.32285 | 10 | 13 | 10 | 13 |
| 1448 | Adenine phosphoribosyltransferase | IPI00218693 | 0.000 | 0.32285 | 9 | 14 | 10 | 13 |
| 1449 | Insulin-like growth factor 2 mRNA-binding protein 1 | IPI00008557 | 0.000 | 0.32285 | 11 | 13 | 14 | 10 |
| 1450 | Protein transport protein Sec24C | IPI00024661 | 0.000 | 0.32285 | 11 | 7 | 7 | 11 |
| 1451 | Golgi phosphoprotein 3 | IPI00005490 | 0.000 | 0.32285 | 11 | 11 | 10 | 12 |
| 1452 | Transmembrane protein 33 | IPI00299084 | 0.000 | 0.32285 | 4 | 0 | 0 | 4 |
| 1453 | cDNA FLJ56153, highly similar to Homo sapiens transforming growth factor beta regulator 4 (TBRG4) | IPI00329625 | 0.000 | 0.32285 | 8 | 11 | 10 | 9 |
| 1454 | Nuclear transport factor 2 | IPI00009901 | 0.000 | 0.32285 | 0 | 0 | 0 | 0 |
| 1455 | Spermidine synthase | IPI00292020 | 0.000 | 0.32285 | 9 | 12 | 8 | 13 |
| 1456 | Vacuolar protein-sorting-associated protein 25 | IPI00031655 | 0.000 | 0.32285 | 6 | 13 | 9 | 10 |
| 1457 | Isoform 2 of Basigin | IPI00019906 | 0.000 | 0.32285 | 10 | 10 | 13 | 7 |
| 1458 | Isoform 1 of Caprin-1 | IPI00783872 | 0.000 | 0.32285 | 7 | 9 | 7 | 9 |
| 1459 | Isoform 1 of DNA-binding protein A | IPI00031801 | 0.000 | 0.32285 | 9 | 9 | 11 | 7 |
| 1460 | AP-1 complex subunit mu-1 | IPI00032516 | 0.000 | 0.32285 | 6 | 9 | 5 | 10 |
| 1461 | Protein naked cuticle homolog 1 | IPI00056339 | 0.000 | 0.32285 | 7 | 8 | 5 | 10 |
| 1462 | Prostaglandin E synthase 2 | IPI00303568 | 0.000 | 0.32285 | 7 | 9 | 5 | 11 |
| 1463 | Calpain small subunit 1 | IPI00025084 | 0.000 | 0.32285 | 8 | 11 | 7 | 12 |
| 1464 | Isoform 1 of Methylthioribose-1-phosphate isomerase | IPI00005948 | 0.000 | 0.32285 | 5 | 6 | 6 | 5 |
| 1465 | Isoform 1 of Protein unc-45 homolog A | IPI00072534 | 0.000 | 0.32285 | 6 | 12 | 9 | 9 |
| 1466 | Proteasome subunit beta type-6 | IPI00000811 | 0.000 | 0.32285 | 10 | 4 | 8 | 6 |
| 1467 | Isoform 2 of Tyrosine-protein phosphatase non-receptor type 11 | IPI00298347 | 0.000 | 0.32285 | 7 | 4 | 3 | 8 |
| 1468 | Aldehyde dehydrogenase X, mitochondrial | IPI00103467 | 0.000 | 0.32285 | 7 | 11 | 8 | 10 |
| 1469 | cDNA FLJ14048 fis, clone HEMBA1006650, weakly similar to ARP2/3 COMPLEX 20 KD SUBUNIT | IPI00386354 | 0.000 | 0.32285 | 11 | 8 | 11 | 8 |
| 1470 | Isoform 1 of Coiled-coil domain-containing protein 47 | IPI00024642 | 0.000 | 0.32285 | 6 | 7 | 6 | 7 |
| 1471 | Nucleolar GTP-binding protein 1 | IPI00385042 | 0.000 | 0.32285 | 7 | 5 | 4 | 8 |
| 1472 | Isoform 1 of Fragile X mental retardation syndrome-related protein 1 | IPI00016249 | 0.000 | 0.32285 | 3 | 7 | 4 | 6 |
| 1473 | Isoform 1 of Serine/threonine-protein phosphatase 2A 65 kDa regulatory subunit A beta isoform | IPI00294178 | 0.000 | 0.32285 | 7 | 6 | 5 | 8 |
| 1474 | transcriptional regulator ATRX isoform 2 | IPI00220109 | 0.000 | 0.32285 | 0 | 3 | 0 | 3 |
| 1475 | Importin 5 | IPI00514205 | 0.000 | 0.32285 | 7 | 7 | 7 | 7 |
| 1476 | Xaa-Pro dipeptidase | IPI00257882 | 0.000 | 0.32285 | 2 | 7 | 4 | 5 |
| 1477 | 24 kDa protein | IPI00398057 | 0.000 | 0.32285 | 0 | 0 | 0 | 0 |
| 1478 | Isoform 2 of Ubiquitin-conjugating enzyme E2 K | IPI00019894 | 0.000 | 0.32285 | 7 | 5 | 5 | 7 |
| 1479 | Dolichyldiphosphatase 1 | IPI00329410 | 0.000 | 0.32285 | 0 | 2 | 2 | 2 |
| 1480 | Probable ribosome biogenesis protein NEP1 | IPI00025347 | 0.000 | 0.32285 | 6 | 9 | 6 | 9 |
| 1481 | cohesin subunit SA-2 isoform a | IPI00470883 | 0.000 | 0.32285 | 5 | 4 | 3 | 6 |
| 1482 | Casein kinase II subunit alpha' | IPI00020602 | 0.000 | 0.32285 | 5 | 7 | 5 | 7 |
| 1483 | proteasome subunit beta type-5 isoform 3 | IPI00383971 | 0.000 | 0.32285 | 13 | 5 | 14 | 4 |
| 1484 | Isoform 1 of Growth factor receptor-bound protein 2 | IPI00021327 | 0.000 | 0.32285 | 7 | 4 | 5 | 6 |
| 1485 | Exportin-7 | IPI00302458 | 0.000 | 0.32285 | 6 | 4 | 3 | 7 |
| 1486 | Isoform 1 of Methyl-CpG-binding domain protein 3 | IPI00439194 | 0.000 | 0.32285 | 6 | 8 | 7 | 7 |
| 1487 | L-aminoadipate-semialdehyde dehydrogenase-phosphopantetheinyl transferase | IPI00250297 | 0.000 | 0.32285 | 5 | 8 | 5 | 8 |
| 1488 | F-actin-capping protein subunit alpha-2 | IPI00026182 | 0.000 | 0.32285 | 6 | 8 | 7 | 7 |
| 1489 | Mannose-P-dolichol utilization defect 1 protein | IPI00025292 | 0.000 | 0.32285 | 0 | 0 | 0 | 0 |
| 1490 | Uroporphyrinogen decarboxylase | IPI00301489 | 0.000 | 0.32285 | 0 | 0 | 0 | 0 |
| 1491 | Tyrosine-protein phosphatase non-receptor type 1 | IPI00297261 | 0.000 | 0.32285 | 6 | 5 | 6 | 5 |
| 1492 | Condensin-2 complex subunit D3 | IPI00747787 | 0.000 | 0.32285 | 4 | 5 | 3 | 6 |
| 1493 | Ribosomal protein L1 | IPI00035167 | 0.000 | 0.32285 | 5 | 5 | 5 | 5 |
| 1494 | synembryn-A | IPI00100106 | 0.000 | 0.32285 | 4 | 7 | 5 | 6 |
| 1495 | UPF0160 protein MYG1, mitochondrial | IPI00029444 | 0.000 | 0.32285 | 5 | 7 | 6 | 6 |
| 1496 | Pirin | IPI00012575 | 0.000 | 0.32285 | 9 | 4 | 7 | 6 |
| 1497 | Eukaryotic translation initiation factor 4E | IPI00027485 | 0.000 | 0.32285 | 0 | 0 | 0 | 0 |
| 1498 | Talin-2 | IPI00219299 | 0.000 | 0.32285 | 8 | 4 | 7 | 5 |
| 1499 | Isoform 3 of Serine/threonine-protein phosphatase 2A activator | IPI00217296 | 0.000 | 0.32285 | 3 | 8 | 0 | 9 |
| 1500 | Isoform 1 of Oligoribonuclease, mitochondrial (Fragment) | IPI00032830 | 0.000 | 0.32285 | 4 | 5 | 3 | 6 |
| 1501 | DNA-directed RNA polymerases I, II, and III subunit RPABC1 | IPI00291093 | 0.000 | 0.32285 | 5 | 6 | 4 | 7 |
| 1502 | Eukaryotic translation initiation factor 3 subunit J | IPI00290461 | 0.000 | 0.32285 | 6 | 6 | 6 | 6 |
| 1503 | Pre-mRNA branch site protein p14 | IPI00032827 | 0.000 | 0.32285 | 8 | 11 | 10 | 9 |
| 1504 | Isoform 1 of Large proline-rich protein BAT2 | IPI00010700 | 0.000 | 0.32285 | 3 | 3 | 2 | 4 |
| 1505 | Synaptobrevin homolog YKT6 | IPI00008569 | 0.000 | 0.32285 | 2 | 2 | 0 | 2 |
| 1506 | Similar to Signal peptidase complex subunit 2 | IPI00452747 | 0.000 | 0.32285 | 6 | 6 | 4 | 8 |
| 1507 | 39S ribosomal protein L17, mitochondrial | IPI00172591 | 0.000 | 0.32285 | 4 | 6 | 5 | 5 |
| 1508 | Actin-related protein 2/3 complex subunit 1B | IPI00005160 | 0.000 | 0.32285 | 2 | 2 | 2 | 2 |
| 1509 | 4-hydroxyphenylpyruvate dioxygenase-like protein | IPI00063762 | 0.000 | 0.32285 | 0 | 7 | 3 | 6 |
| 1510 | Isoform 1 of DDRGK domain-containing protein 1 | IPI00028387 | 0.000 | 0.32285 | 7 | 4 | 9 | 0 |
| 1511 | Large neutral amino acids transporter small subunit 1 | IPI00008986 | 0.000 | 0.32285 | 2 | 2 | 0 | 0 |

| No. | Description | Accession number | STN[1] | p-Value[1] | Con_A[2] | Con_B[2] | RG3_A[2] | RG3_B[2] |
|---|---|---|---|---|---|---|---|---|
| 1512 | Isoform 2 of Diphosphoinositol polyphosphate phosphohydrolase 2 | IPI00021408 | 0.000 | 0.32285 | 3 | 8 | 3 | 8 |
| 1513 | Carbonyl reductase [NADPH] 3 | IPI00290462 | 0.000 | 0.32285 | 6 | 3 | 5 | 4 |
| 1514 | Isoform 1 of Heterogeneous nuclear ribonucleoprotein U-like protein 1 | IPI00013070 | 0.000 | 0.32285 | 6 | 5 | 6 | 5 |
| 1515 | Isoform 1 of Transcription elongation factor SPT5 | IPI00298058 | 0.000 | 0.32285 | 4 | 3 | 3 | 4 |
| 1516 | 28S ribosomal protein S31, mitochondrial | IPI00294242 | 0.000 | 0.32285 | 3 | 7 | 4 | 6 |
| 1517 | WD repeat-containing protein 82 | IPI00152695 | 0.000 | 0.32285 | 4 | 7 | 5 | 6 |
| 1518 | 8 kDa protein | IPI00160382 | 0.000 | 0.32285 | 0 | 0 | 2 | 0 |
| 1519 | Isoform 1 of 60S ribosome subunit biogenesis protein NIP7 homolog | IPI00007175 | 0.000 | 0.32285 | 3 | 0 | 3 | 0 |
| 1520 | Cell differentiation protein RCD1 homolog | IPI00023101 | 0.000 | 0.32285 | 4 | 5 | 3 | 6 |
| 1521 | Isoform 1 of Huntingtin-interacting protein K | IPI00335001 | 0.000 | 0.32285 | 4 | 8 | 4 | 8 |
| 1522 | Phosphoglucomutase-2 | IPI00550364 | 0.000 | 0.32285 | 3 | 3 | 0 | 4 |
| 1523 | Bystin | IPI00328987 | 0.000 | 0.32285 | 2 | 3 | 2 | 3 |
| 1524 | Metastasis-associated protein MTA2 | IPI00171798 | 0.000 | 0.32285 | 5 | 6 | 4 | 7 |
| 1525 | Glyoxylate reductase/hydroxypyruvate reductase | IPI00037448 | 0.000 | 0.32285 | 5 | 6 | 5 | 6 |
| 1526 | cDNA FLJ56402, highly similar to Tripeptidyl-peptidase 1 | IPI00298237 | 0.000 | 0.32285 | 0 | 5 | 3 | 4 |
| 1527 | Crk-like protein | IPI00004839 | 0.000 | 0.32285 | 4 | 5 | 4 | 5 |
| 1528 | Hydroxymethylglutaryl-CoA synthase, cytoplasmic | IPI00008475 | 0.000 | 0.32285 | 4 | 7 | 5 | 6 |
| 1529 | Isoform 1 of Serum paraoxonase/arylesterase 2 | IPI00014958 | 0.000 | 0.32285 | 0 | 0 | 0 | 2 |
| 1530 | 28S ribosomal protein S25, mitochondrial | IPI00013167 | 0.000 | 0.32285 | 4 | 4 | 4 | 4 |
| 1531 | TP53-regulating kinase | IPI00290305 | 0.000 | 0.32285 | 5 | 4 | 5 | 4 |
| 1532 | Isoform p26 of 7,8-dihydro-8-oxoguanine triphosphatase | IPI00004392 | 0.000 | 0.32285 | 2 | 6 | 2 | 6 |
| 1533 | Rho-associated protein kinase 1 | IPI00022542 | 0.000 | 0.32285 | 4 | 4 | 5 | 3 |
| 1534 | Dihydroorotate dehydrogenase, mitochondrial | IPI00024462 | 0.000 | 0.32285 | 3 | 7 | 5 | 5 |
| 1535 | Epoxide hydrolase 1 | IPI00009896 | 0.000 | 0.32285 | 3 | 3 | 0 | 4 |
| 1536 | nardilysin isoform a | IPI00243221 | 0.000 | 0.32285 | 6 | 4 | 6 | 4 |
| 1537 | similar to RAN binding protein 1 | IPI00399212 | 0.000 | 0.32285 | 5 | 3 | 6 | 2 |
| 1538 | Tubulin-folding cofactor B | IPI00293126 | 0.000 | 0.32285 | 3 | 3 | 2 | 4 |
| 1539 | Similar to Protein SAAL1. Isoform 2 | IPI00304935 | 0.000 | 0.32285 | 2 | 4 | 3 | 3 |
| 1540 | Isoform 1 of Ubiquitin carboxyl-terminal hydrolase 34 | IPI00297593 | 0.000 | 0.32285 | 0 | 0 | 0 | 2 |
| 1541 | Isoform Short of TATA-binding protein-associated factor 2N | IPI00020194 | 0.000 | 0.32285 | 5 | 0 | 4 | 3 |
| 1542 | Coiled-coil-helix-coiled-coil-helix domain-containing protein 3, mitochondrial | IPI00015833 | 0.000 | 0.32285 | 9 | 4 | 8 | 5 |
| 1543 | Isoform 1 of Histone-arginine methyltransferase CARM1 | IPI00412880 | 0.000 | 0.32285 | 0 | 0 | 0 | 2 |
| 1544 | Retinoblastoma-associated protein | IPI00302829 | 0.000 | 0.32285 | 2 | 4 | 2 | 4 |
| 1545 | Solute carrier family 4 sodium bicarbonate cotransporter member 7 | IPI00021058 | 0.000 | 0.32285 | 0 | 0 | 0 | 0 |
| 1546 | Epithelial cell adhesion molecule | IPI00296215 | 0.000 | 0.32285 | 4 | 0 | 3 | 3 |
| 1547 | Scavenger mRNA-decapping enzyme DcpS | IPI00335385 | 0.000 | 0.32285 | 3 | 5 | 4 | 4 |
| 1548 | Putative uncharacterized protein ALB | IPI00022434 | 0.000 | 0.32285 | 5 | 5 | 5 | 5 |
| 1549 | Isoform 2 of Putative methyltransferase NSUN5 | IPI00101659 | 0.000 | 0.32285 | 3 | 6 | 5 | 4 |
| 1550 | Mitochondrial import receptor subunit TOM7 homolog | IPI00000980 | 0.000 | 0.32285 | 0 | 0 | 0 | 0 |
| 1551 | Developmentally-regulated GTP-binding protein 2 | IPI00022697 | 0.000 | 0.32285 | 3 | 7 | 5 | 5 |
| 1552 | Isoform 4 of Uncharacterized protein KIAA0090 | IPI00642244 | 0.000 | 0.32285 | 2 | 2 | 2 | 2 |
| 1553 | Hexokinase-2 | IPI00102864 | 0.000 | 0.32285 | 3 | 2 | 2 | 3 |
| 1554 | Vacuolar protein sorting-associated protein 4A | IPI00411356 | 0.000 | 0.32285 | 2 | 2 | 0 | 0 |
| 1555 | RNA-binding protein 28 | IPI00304187 | 0.000 | 0.32285 | 4 | 5 | 4 | 5 |
| 1556 | Isoform 3 of Tyrosine-protein phosphatase non-receptor type 6 | IPI00183046 | 0.000 | 0.32285 | 3 | 5 | 3 | 5 |
| 1557 | Isoform 1 of POTE ankyrin domain family member E | IPI00479743 | 0.000 | 0.32285 | 0 | 5 | 0 | 5 |
| 1558 | Isoform Long of FAS-associated factor 1 | IPI00070643 | 0.000 | 0.32285 | 5 | 3 | 3 | 5 |
| 1559 | RNA methyltransferase-like protein 1 | IPI00335589 | 0.000 | 0.32285 | 0 | 0 | 2 | 0 |
| 1560 | Isoform 3 of Rapamycin-insensitive companion of mTOR | IPI00166528 | 0.000 | 0.32285 | 4 | 0 | 4 | 0 |
| 1561 | Putative uncharacterized protein PYCR2 | IPI00335061 | 0.000 | 0.32285 | 5 | 5 | 5 | 5 |
| 1562 | Ras-related protein Rab-5A | IPI00023510 | 0.000 | 0.32285 | 0 | 5 | 3 | 4 |
| 1563 | Aldo-keto reductase family 1 member C3 | IPI00291483 | 0.000 | 0.32285 | 2 | 5 | 0 | 5 |
| 1564 | Isoform 1 of C-terminal-binding protein 2 | IPI00010120 | 0.000 | 0.32285 | 5 | 3 | 5 | 3 |
| 1565 | Mortality factor 4-like protein 2 | IPI00014174 | 0.000 | 0.32285 | 0 | 0 | 0 | 2 |
| 1566 | Isoform 1 of Putative helicase MOV-10 | IPI00444452 | 0.000 | 0.32285 | 0 | 0 | 0 | 2 |
| 1567 | Ubiquitin-conjugating enzyme E2 A | IPI00746451 | 0.000 | 0.32285 | 0 | 0 | 2 | 2 |
| 1568 | Splicing factor, arginine/serine-rich 11 | IPI00464952 | 0.000 | 0.32285 | 2 | 3 | 2 | 3 |
| 1569 | Serine/threonine-protein kinase PRP4 homolog | IPI00013721 | 0.000 | 0.32285 | 4 | 4 | 3 | 5 |
| 1570 | ATP-binding cassette sub-family F member 2 | IPI00005045 | 0.000 | 0.32285 | 5 | 2 | 4 | 3 |
| 1571 | Transmembrane 9 superfamily member 2 | IPI00018415 | 0.000 | 0.32285 | 4 | 0 | 2 | 4 |
| 1572 | Isoform A of DnaJ homolog subfamily B member 6 | IPI00024523 | 0.000 | 0.32285 | 2 | 2 | 0 | 2 |
| 1573 | Probable fructose-2,6-bisphosphatase TIGAR | IPI00006907 | 0.000 | 0.32285 | 2 | 0 | 2 | 0 |
| 1574 | Isoform 2 of Tether containing UBX domain for GLUT4 | IPI00065276 | 0.000 | 0.32285 | 5 | 3 | 4 | 4 |
| 1575 | BAG family molecular chaperone regulator 2 | IPI00000643 | 0.000 | 0.32285 | 0 | 2 | 0 | 2 |
| 1576 | GTPase NRas | IPI00000005 | 0.000 | 0.32285 | 3 | 3 | 0 | 4 |
| 1577 | Isoform 1 of Solute carrier family 12 member 7 | IPI00008616 | 0.000 | 0.32285 | 2 | 0 | 0 | 0 |
| 1578 | WD40 repeat-containing protein SMU1 | IPI00305833 | 0.000 | 0.32285 | 2 | 2 | 0 | 2 |
| 1579 | Isoform 1 of Proteasome assembly chaperone 1 | IPI00030770 | 0.000 | 0.32285 | 0 | 2 | 0 | 2 |
| 1580 | Thioredoxin-like protein 1 | IPI00305692 | 0.000 | 0.32285 | 2 | 2 | 0 | 0 |
| 1581 | Dual specificity mitogen-activated protein kinase kinase 1 | IPI00219604 | 0.000 | 0.32285 | 0 | 3 | 0 | 3 |
| 1582 | U3 small nucleolar ribonucleoprotein protein IMP3 | IPI00019488 | 0.000 | 0.32285 | 4 | 3 | 2 | 5 |
| 1583 | Deoxyhypusine hydroxylase | IPI00171856 | 0.000 | 0.32285 | 4 | 0 | 3 | 3 |
| 1584 | Isoform 1 of HBS1-like protein | IPI00009070 | 0.000 | 0.32285 | 2 | 4 | 2 | 4 |
| 1585 | Cation-dependent mannose-6-phosphate receptor | IPI00025049 | 0.000 | 0.32285 | 2 | 2 | 2 | 0 |
| 1586 | Isoform 1 of HEAT repeat-containing protein 3 | IPI00100984 | 0.000 | 0.32285 | 0 | 7 | 3 | 6 |
| 1587 | Isoform Del-701 of Signal transducer and activator of transcription 3 | IPI00306436 | 0.000 | 0.32285 | 0 | 0 | 0 | 0 |
| 1588 | Isoform 1 of Transmembrane protein 111 | IPI00020472 | 0.000 | 0.32285 | 3 | 4 | 3 | 4 |
| 1589 | Isoform 1 of N-acylneuraminate cytidylyltransferase | IPI00303158 | 0.000 | 0.32285 | 2 | 2 | 2 | 2 |
| 1590 | Phosphatidylinositol-5-phosphate 4-kinase type-2 gamma | IPI00152303 | 0.000 | 0.32285 | 0 | 0 | 0 | 0 |
| 1591 | 39S ribosomal protein L37, mitochondrial | IPI00162330 | 0.000 | 0.32285 | 2 | 3 | 3 | 2 |
| 1592 | 60S ribosomal protein L17 | IPI00413324 | 0.000 | 0.32285 | 4 | 3 | 5 | 0 |
| 1593 | Isoform 2 of Mitochondrial import inner membrane translocase subunit TIM50 | IPI00418497 | 0.000 | 0.32285 | 3 | 6 | 2 | 7 |
| 1594 | NEDD8-conjugating enzyme Ubc12 | IPI00022597 | 0.000 | 0.32285 | 3 | 0 | 3 | 0 |
| 1595 | Histone chaperone ASF1B | IPI00041127 | 0.000 | 0.32285 | 0 | 0 | 0 | 0 |
| 1596 | N-acetylgalactosaminyltransferase 7 | IPI00328391 | 0.000 | 0.32285 | 0 | 0 | 0 | 2 |
| 1597 | cDNA FLJ45232 fis, clone BRCAN2021718 | IPI00170877 | 0.000 | 0.32285 | 2 | 0 | 0 | 0 |
| 1598 | Ras-related protein Rab-35 | IPI00300096 | 0.000 | 0.32285 | 0 | 2 | 0 | 2 |
| 1599 | Isoform 1 of Zinc finger MYM-type protein 3 | IPI00029484 | 0.000 | 0.32285 | 0 | 0 | 2 | 2 |
| 1600 | Isoform 2 of Sacsin | IPI00784002 | 0.000 | 0.32285 | 0 | 0 | 0 | 2 |
| 1601 | Isoform 4 of Afadin | IPI00023461 | 0.000 | 0.32285 | 0 | 3 | 2 | 3 |
| 1602 | cDNA FLJ38069 fis, clone CTONG2015434 | IPI00029159 | 0.000 | 0.32285 | 2 | 3 | 3 | 2 |
| 1603 | Isoform 2 of Ubiquilin-1 | IPI00071180 | 0.000 | 0.32285 | 3 | 0 | 3 | 2 |
| 1604 | Pyridoxal phosphate phosphatase | IPI00025340 | 0.000 | 0.32285 | 2 | 5 | 2 | 5 |
| 1605 | programmed cell death 4 isoform 2 | IPI00240675 | 0.000 | 0.32285 | 4 | 4 | 5 | 3 |
| 1606 | 28S ribosomal protein S7, mitochondrial | IPI00006440 | 0.000 | 0.32285 | 3 | 4 | 4 | 3 |

| No. | Description | Accession number | STN[1] | p-Value[1] | Con_A[2] | Con_B[2] | RG3_A[2] | RG3_B[2] |
|---|---|---|---|---|---|---|---|---|
| 1607 | GrpE protein homolog 1, mitochondrial | IPI00029557 | 0.000 | 0.32285 | 0 | 3 | 2 | 3 |
| 1608 | Probable ergosterol biosynthetic protein 28 | IPI00007730 | 0.000 | 0.32285 | 3 | 3 | 2 | 4 |
| 1609 | Isoform 1 of Chromodomain-helicase-DNA-binding protein 8 | IPI00398992 | 0.000 | 0.32285 | 0 | 2 | 0 | 0 |
| 1610 | cDNA: FLJ22728 fis, clone HSI15617 (Fragment) | IPI00386139 | 0.000 | 0.32285 | 4 | 3 | 4 | 3 |
| 1611 | Exosome complex exonuclease RRP43 | IPI00552920 | 0.000 | 0.32285 | 0 | 0 | 0 | 2 |
| 1612 | Neuronal protein | IPI00472058 | 0.000 | 0.32285 | 0 | 0 | 0 | 0 |
| 1613 | Importin-8 | IPI00007401 | 0.000 | 0.32285 | 3 | 0 | 2 | 3 |
| 1614 | Cytochrome b-c1 complex subunit 7 | IPI00220416 | 0.000 | 0.32285 | 5 | 2 | 4 | 3 |
| 1615 | High mobility group protein B3 | IPI00217477 | 0.000 | 0.32285 | 4 | 5 | 4 | 5 |
| 1616 | Protein phosphatase 1F | IPI00291412 | 0.000 | 0.32285 | 0 | 3 | 2 | 3 |
| 1617 | Isoform 2 of Nucleosome-remodeling factor subunit BPTF | IPI00254408 | 0.000 | 0.32285 | 2 | 2 | 2 | 0 |
| 1618 | Isoform 2 of DnaJ homolog subfamily A member 3, mitochondrial | IPI00179187 | 0.000 | 0.32285 | 3 | 0 | 2 | 3 |
| 1619 | Isoform 1 of Ral GTPase-activating protein subunit beta | IPI00409601 | 0.000 | 0.32285 | 0 | 0 | 0 | 2 |
| 1620 | DNA polymerase alpha subunit B | IPI00290272 | 0.000 | 0.32285 | 2 | 2 | 0 | 0 |
| 1621 | Polymerase delta-interacting protein 2 | IPI00165506 | 0.000 | 0.32285 | 4 | 4 | 6 | 2 |
| 1622 | Similar to Zinc finger CCCH domain-containing protein 15 | IPI00000279 | 0.000 | 0.32285 | 3 | 3 | 0 | 4 |
| 1623 | Isoform 2 of Ubiquitin carboxyl-terminal hydrolase 47 | IPI00165528 | 0.000 | 0.32285 | 2 | 3 | 3 | 2 |
| 1624 | Exocyst complex component 4 | IPI00059279 | 0.000 | 0.32285 | 2 | 3 | 2 | 3 |
| 1625 | Protoporphyrinogen oxidase | IPI00031357 | 0.000 | 0.32285 | 0 | 0 | 0 | 0 |
| 1626 | Major centromere autoantigen B | IPI00010388 | 0.000 | 0.32285 | 4 | 3 | 3 | 4 |
| 1627 | Isoform 1 of HCLS1-associated protein X-1 | IPI00010440 | 0.000 | 0.32285 | 2 | 0 | 0 | 2 |
| 1628 | Isoform 2 of 39S ribosomal protein L55, mitochondrial | IPI00419626 | 0.000 | 0.32285 | 0 | 0 | 0 | 0 |
| 1629 | E3 ubiquitin-protein ligase BRE1A | IPI00251559 | 0.000 | 0.32285 | 5 | 2 | 5 | 0 |
| 1630 | cDNA FLJ56152, highly similar to Rho guanine nucleotide exchange factor 7 | IPI00449906 | 0.000 | 0.32285 | 2 | 5 | 3 | 4 |
| 1631 | Isoform Short of NADPH:adrenodoxin oxidoreductase, mitochondrial | IPI00026958 | 0.000 | 0.32285 | 2 | 0 | 2 | 0 |
| 1632 | Isoform 1 of Craniofacial development protein 1 | IPI00007306 | 0.000 | 0.32285 | 2 | 5 | 3 | 4 |
| 1633 | 39S ribosomal protein L18, mitochondrial | IPI00160421 | 0.000 | 0.32285 | 0 | 5 | 4 | 3 |
| 1634 | Probable methyltransferase TARBP1 | IPI00298447 | 0.000 | 0.32285 | 0 | 2 | 0 | 0 |
| 1635 | Isoform 1 of RNA-binding protein 4 | IPI00003704 | 0.000 | 0.32285 | 3 | 2 | 3 | 0 |
| 1636 | Isoform 1 of Secretory carrier-associated membrane protein 1 | IPI00005129 | 0.000 | 0.32285 | 2 | 0 | 2 | 0 |
| 1637 | Coiled-coil domain-containing protein 6 | IPI00000634 | 0.000 | 0.32285 | 3 | 3 | 3 | 3 |
| 1638 | D-tyrosyl-tRNA(Tyr) deacylase 1 | IPI00152692 | 0.000 | 0.32285 | 3 | 4 | 3 | 4 |
| 1639 | cDNA FLJ60939, highly similar to NAD-dependent deacetylase sirtuin-3, mitochondrial | IPI00183171 | 0.000 | 0.32285 | 0 | 3 | 0 | 3 |
| 1640 | cDNA, FLJ96841 | IPI00099995 | 0.000 | 0.32285 | 0 | 0 | 0 | 2 |
| 1641 | Isoform 1 of Vacuolar protein sorting-associated protein 8 homolog | IPI00464985 | 0.000 | 0.32285 | 2 | 0 | 2 | 0 |
| 1642 | Isoform Short of Ubiquitin fusion degradation protein 1 homolog | IPI00218292 | 0.000 | 0.32285 | 2 | 2 | 0 | 0 |
| 1643 | Isoform 1 of Protein fat-free homolog | IPI00001710 | 0.000 | 0.32285 | 0 | 0 | 0 | 2 |
| 1644 | Vacuolar protein sorting-associated protein 4B | IPI00182728 | 0.000 | 0.32285 | 0 | 2 | 2 | 2 |
| 1645 | 71 kDa protein | IPI00062599 | 0.000 | 0.32285 | 2 | 3 | 0 | 3 |
| 1646 | Cysteine and glycine-rich protein 1 | IPI00442073 | 0.000 | 0.32285 | 2 | 2 | 2 | 0 |
| 1647 | Isoform 2 of Pinin | IPI00002649 | 0.000 | 0.32285 | 0 | 3 | 0 | 3 |
| 1648 | Telomere length regulation protein TEL2 homolog | IPI00016868 | 0.000 | 0.32285 | 0 | 3 | 0 | 3 |
| 1649 | Procollagen-lysine,2-oxoglutarate 5-dioxygenase 3 | IPI00030255 | 0.000 | 0.32285 | 0 | 2 | 0 | 2 |
| 1650 | 5-formyltetrahydrofolate cyclo-ligase | IPI00220567 | 0.000 | 0.32285 | 3 | 4 | 3 | 4 |
| 1651 | Protein | IPI00892529 | 0.000 | 0.32285 | 2 | 0 | 0 | 2 |
| 1652 | Exosome complex exonuclease RRP40 | IPI00015956 | 0.000 | 0.32285 | 0 | 0 | 2 | 2 |
| 1653 | RWD domain-containing protein 1 | IPI00034010 | 0.000 | 0.32285 | 2 | 2 | 2 | 0 |
| 1654 | Ethanolamine kinase 1 | IPI00030090 | 0.000 | 0.32285 | 0 | 0 | 2 | 0 |
| 1655 | Death domain-containing protein CRADD | IPI00020364 | 0.000 | 0.32285 | 0 | 0 | 0 | 0 |
| 1656 | Isoform 3 of LIM domain only protein 7 | IPI00291802 | 0.000 | 0.32285 | 0 | 2 | 2 | 2 |
| 1657 | Ras-related protein Rab-8B | IPI00024282 | 0.000 | 0.32285 | 0 | 3 | 0 | 3 |
| 1658 | Component of gems 4 | IPI00027717 | 0.000 | 0.32285 | 0 | 0 | 0 | 2 |
| 1659 | Mediator of RNA polymerase II transcription subunit 14 | IPI00297191 | 0.000 | 0.32285 | 0 | 0 | 0 | 2 |
| 1660 | Condensin complex subunit 2 | IPI00299507 | 0.000 | 0.32285 | 0 | 0 | 0 | 0 |
| 1661 | Legumain | IPI00293303 | 0.000 | 0.32285 | 0 | 0 | 0 | 0 |
| 1662 | Isoform 3 of Anamorsin | IPI00025333 | 0.000 | 0.32285 | 0 | 0 | 0 | 0 |
| 1663 | Isoform 1 of Dehydrogenase/reductase SDR family member 7 | IPI00006957 | 0.000 | 0.32285 | 0 | 3 | 2 | 3 |
| 1664 | Farnesyltransferase, CAAX box, alpha, isoform CRA_a | IPI00026813 | 0.000 | 0.32285 | 0 | 0 | 0 | 0 |
| 1665 | Gem-associated protein 5 | IPI00291783 | 0.000 | 0.32285 | 3 | 0 | 3 | 0 |
| 1666 | Alcohol dehydrogenase class-3 | IPI00746777 | 0.000 | 0.32285 | 0 | 0 | 2 | 2 |
| 1667 | Isoform 1 of Presenilin-1 | IPI00028077 | 0.000 | 0.32285 | 0 | 3 | 2 | 3 |
| 1668 | Isoform 2 of Gamma-glutamylcyclotransferase | IPI00020301 | 0.000 | 0.32285 | 0 | 4 | 2 | 4 |
| 1669 | WD repeat domain 57 (U5 snRNP specific), isoform CRA_b | IPI00385642 | 0.000 | 0.32285 | 0 | 4 | 0 | 4 |
| 1670 | Isoform 1 of Cell division cycle protein 23 homolog | IPI00005822 | 0.000 | 0.32285 | 2 | 2 | 2 | 2 |
| 1671 | DCC-interacting protein 13-alpha | IPI00015836 | 0.000 | 0.32285 | 0 | 0 | 0 | 0 |
| 1672 | Transmembrane protein 11 | IPI00012855 | 0.000 | 0.32285 | 2 | 0 | 0 | 0 |
| 1673 | PDZ domain-containing protein GIPC1 | IPI00024705 | 0.000 | 0.32285 | 0 | 2 | 2 | 0 |
| 1674 | Farnesyl pyrophosphate synthetase like-4 protein (Fragment) | IPI00382869 | 0.000 | 0.32285 | 2 | 0 | 0 | 0 |
| 1675 | Werner syndrome ATP-dependent helicase | IPI00029107 | 0.000 | 0.32285 | 3 | 2 | 0 | 3 |
| 1676 | Isoform HERA-A of GTP-binding protein era homolog | IPI00026512 | 0.000 | 0.32285 | 0 | 2 | 2 | 0 |
| 1677 | L-lactate dehydrogenase A-like 6A | IPI00148061 | 0.000 | 0.32285 | 0 | 2 | 2 | 0 |
| 1678 | Vacuolar protein sorting-associated protein 26B | IPI00059264 | 0.000 | 0.32285 | 2 | 2 | 0 | 0 |
| 1679 | D-beta-hydroxybutyrate dehydrogenase, mitochondrial | IPI00025341 | 0.000 | 0.32285 | 0 | 0 | 2 | 2 |
| 1680 | Derlin-1 | IPI00013271 | 0.000 | 0.32285 | 0 | 2 | 2 | 0 |
| 1681 | Pre-mRNA cleavage complex 2 protein Pcf11 | IPI00016387 | 0.000 | 0.32285 | 0 | 0 | 2 | 0 |
| 1682 | Huntingtin | IPI00002335 | 0.000 | 0.32285 | 0 | 2 | 0 | 0 |
| 1683 | General transcription factor IIH subunit 4 | IPI00016839 | 0.000 | 0.32285 | 2 | 3 | 0 | 3 |
| 1684 | Isoform 2 of DnaJ homolog subfamily C member 2 | IPI00455199 | 0.000 | 0.32285 | 0 | 3 | 2 | 3 |
| 1685 | Proteasome assembly chaperone 2 | IPI00644482 | 0.000 | 0.32285 | 2 | 0 | 0 | 0 |
| 1686 | Translation initiation factor eIF-2B subunit epsilon | IPI00011898 | 0.000 | 0.32285 | 0 | 0 | 0 | 0 |
| 1687 | Isoform 1 of U4/U6 small nuclear ribonucleoprotein Prp4 | IPI00150269 | 0.000 | 0.32285 | 2 | 2 | 0 | 2 |
| 1688 | Isoform 1 of Protein dopey-2 | IPI00294653 | 0.000 | 0.32285 | 0 | 0 | 0 | 0 |
| 1689 | Protein FAM50A | IPI00030098 | 0.000 | 0.32285 | 2 | 2 | 0 | 2 |
| 1690 | Cell division protein kinase 3 | IPI00023503 | 0.000 | 0.32285 | 0 | 2 | 0 | 0 |
| 1691 | Mitochondrial ornithine transporter 1 | IPI00003389 | 0.000 | 0.32285 | 2 | 2 | 0 | 2 |
| 1692 | Isoform 1 of Pre-mRNA-splicing factor RBM22 | IPI00019046 | 0.000 | 0.32285 | 0 | 3 | 3 | 2 |
| 1693 | Isoform 5 of Serine/threonine-protein phosphatase 4 regulatory subunit 3A | IPI00017290 | 0.000 | 0.32285 | 0 | 3 | 0 | 3 |
| 1694 | Isoform 1 of AP-3 complex subunit beta-1 | IPI00021129 | 0.000 | 0.32285 | 4 | 2 | 4 | 0 |
| 1695 | cDNA FLJ56394, highly similar to N-acetylglucosamine kinase | IPI00296526 | 0.000 | 0.32285 | 0 | 0 | 2 | 2 |
| 1696 | Carboxypeptidase D | IPI00027078 | 0.000 | 0.32285 | 2 | 2 | 0 | 0 |
| 1697 | Protein FAM49A | IPI00006574 | 0.000 | 0.32285 | 0 | 0 | 0 | 0 |
| 1698 | Trafficking protein particle complex subunit 4 | IPI00007691 | 0.000 | 0.32285 | 0 | 2 | 0 | 2 |
| 1699 | Isoform 1 of Protein CDV3 homolog | IPI00014197 | 0.000 | 0.32285 | 0 | 0 | 2 | 0 |
| 1700 | twinfilin-1 | IPI00183508 | 0.000 | 0.32285 | 2 | 2 | 2 | 0 |
| 1701 | ATP-binding cassette sub-family D member 1 | IPI00291373 | 0.000 | 0.32285 | 0 | 0 | 0 | 0 |

| No. | Description | Accession number | STN[1] | p-Value[1] | Con_A[2] | Con_B[2] | RG3_A[2] | RG3_B[2] |
|---|---|---|---|---|---|---|---|---|
| 1702 | Prefoldin subunit 4 | IPI00015891 | 0.000 | 0.32285 | 4 | 0 | 4 | 0 |
| 1703 | Caspase-3 | IPI00292140 | 0.000 | 0.32285 | 0 | 2 | 0 | 2 |
| 1704 | Activity-dependent neuroprotector homeobox protein | IPI00022215 | 0.000 | 0.32285 | 0 | 2 | 2 | 2 |
| 1705 | Isoform 2 of Condensin-2 complex subunit G2 | IPI00396058 | 0.000 | 0.32285 | 0 | 0 | 0 | 0 |
| 1706 | Armadillo repeat-containing X-linked protein 3 | IPI00009906 | 0.000 | 0.32285 | 0 | 0 | 2 | 2 |
| 1707 | Probable ATP-dependent RNA helicase YTHDC2 | IPI00010200 | 0.000 | 0.32285 | 0 | 0 | 0 | 0 |
| 1708 | Isoform 2 of Transcription elongation factor SPT6 | IPI00430770 | 0.000 | 0.32285 | 2 | 0 | 2 | 2 |
| 1709 | Myosin-Id | IPI00329719 | 0.000 | 0.32285 | 0 | 2 | 0 | 0 |
| 1710 | Isoform 1 of TIP41-like protein | IPI00745568 | 0.000 | 0.32285 | 0 | 2 | 0 | 0 |
| 1711 | Ephrin type-A receptor 2 | IPI00021267 | 0.000 | 0.32285 | 2 | 2 | 0 | 0 |
| 1712 | Peptidyl-prolyl cis-trans isomerase F, mitochondrial | IPI00026519 | 0.000 | 0.32285 | 0 | 3 | 0 | 3 |
| 1713 | NADH dehydrogenase [ubiquinone] 1 alpha subcomplex assembly factor 4 | IPI00023064 | 0.000 | 0.32285 | 0 | 0 | 2 | 2 |
| 1714 | Isoform 1 of 28S ribosomal protein S35, mitochondrial | IPI00073779 | 0.000 | 0.32285 | 0 | 2 | 0 | 0 |
| 1715 | E3 ubiquitin-protein ligase MARCH5 | IPI00414168 | 0.000 | 0.32285 | 0 | 0 | 0 | 0 |
| 1716 | Chitobiosyldiphosphodolichol beta-mannosyltransferase | IPI00549761 | 0.000 | 0.32285 | 0 | 0 | 2 | 0 |
| 1717 | Isoform XLas-1 of Guanine nucleotide-binding protein G(s) subunit alpha isoforms XLas | IPI00095891 | 0.000 | 0.32285 | 2 | 0 | 0 | 0 |
| 1718 | Acidic leucine-rich nuclear phosphoprotein 32 family member A | IPI00025849 | 0.000 | 0.32285 | 0 | 0 | 2 | 0 |
| 1719 | Isoform 1 of Leucine-rich repeat-containing protein 16A | IPI00014843 | 0.000 | 0.32285 | 2 | 0 | 0 | 0 |
| 1720 | Isoform 1 of Lysine-specific demethylase 3B | IPI00298935 | 0.000 | 0.32285 | 0 | 0 | 0 | 0 |
| 1721 | Biogenesis of lysosome-related organelles complex 1 subunit 1 | IPI00020319 | 0.000 | 0.32285 | 3 | 2 | 3 | 2 |
| 1722 | Isoform 2 of Valacyclovir hydrolase | IPI00003990 | 0.000 | 0.32285 | 0 | 2 | 2 | 2 |
| 1723 | MAGUK p55 subfamily member 6 | IPI00303280 | 0.000 | 0.32285 | 0 | 0 | 0 | 0 |
| 1724 | DNA-directed RNA polymerase II subunit RPB7 | IPI00218895 | 0.000 | 0.32285 | 2 | 0 | 2 | 0 |
| 1725 | 39S ribosomal protein L11, mitochondrial | IPI00007001 | 0.000 | 0.32285 | 0 | 3 | 0 | 3 |
| 1726 | UPF0554 protein C2orf43 | IPI00030257 | 0.000 | 0.32285 | 0 | 2 | 0 | 0 |
| 1727 | Isoform 1 of Lysophosphatidic acid phosphatase type 6 | IPI00099838 | 0.000 | 0.32285 | 0 | 0 | 0 | 2 |
| 1728 | Isoform 1 of Ligatin | IPI00013160 | 0.000 | 0.32285 | 3 | 0 | 0 | 3 |
| 1729 | Gamma-tubulin complex component 2 | IPI00029705 | 0.000 | 0.32285 | 0 | 2 | 2 | 0 |
| 1730 | Isoform 1 of Nucleolar protein of 40 kDa | IPI00099810 | 0.000 | 0.32285 | 0 | 0 | 2 | 0 |
| 1731 | Isoform 1 of Nuclear-interacting partner of ALK | IPI00301421 | 0.000 | 0.32285 | 2 | 0 | 0 | 0 |
| 1732 | cDNA FLJ61629, highly similar to Clathrin interactor 1 | IPI00291930 | 0.000 | 0.32285 | 0 | 2 | 0 | 0 |
| 1733 | Isoform 1 of Ubiquitin carboxyl-terminal hydrolase 19 | IPI00016589 | 0.000 | 0.32285 | 0 | 0 | 2 | 0 |
| 1734 | Isoform 1 of Cytochrome c oxidase assembly protein COX15 homolog | IPI00419869 | 0.000 | 0.32285 | 2 | 0 | 0 | 0 |
| 1735 | RNA 3'-terminal phosphate cyclase-like protein | IPI00294229 | 0.000 | 0.32285 | 0 | 3 | 0 | 3 |
| 1736 | Isoform 1 of Translation initiation factor eIF-2B subunit delta | IPI00005979 | 0.000 | 0.32285 | 3 | 0 | 0 | 3 |
| 1737 | Isoform 1 of Alanine aminotransferase 2 | IPI00152432 | 0.000 | 0.32285 | 2 | 0 | 0 | 2 |
| 1738 | poly [ADP-ribose] polymerase 14 | IPI00291215 | 0.000 | 0.32285 | 0 | 2 | 0 | 2 |
| 1739 | Leucine-rich repeat-containing protein 40 | IPI00152998 | 0.000 | 0.32285 | 0 | 0 | 0 | 0 |
| 1740 | cDNA FLJ12662 fis, clone NT2RM4002205, moderately similar to ELONGATION FACTOR G | IPI00026321 | 0.000 | 0.32285 | 0 | 2 | 0 | 0 |
| 1741 | Isoform 1 of Creatine kinase U-type, mitochondrial | IPI00658109 | 0.000 | 0.32285 | 0 | 2 | 2 | 2 |
| 1742 | Similar to Ankyrin repeat and FYVE domain-containing protein 1 | IPI00159899 | 0.000 | 0.32285 | 0 | 0 | 0 | 2 |
| 1743 | Isoform 1 of Kinesin-like protein KIF1B | IPI00029011 | 0.000 | 0.32285 | 2 | 0 | 0 | 2 |
| 1744 | Small nuclear ribonucleoprotein F | IPI00220528 | 0.000 | 0.32285 | 2 | 3 | 0 | 3 |
| 1745 | Isoform 2 of Liprin-beta-1 | IPI00179172 | 0.000 | 0.32285 | 0 | 0 | 2 | 2 |
| 1746 | similar to unr-interacting protein | IPI00260209 | 0.000 | 0.32285 | 2 | 0 | 0 | 0 |
| 1747 | Isoform 1 of Protein IWS1 homolog | IPI00296432 | 0.000 | 0.32285 | 2 | 0 | 0 | 2 |
| 1748 | NADH dehydrogenase [ubiquinone] 1 beta subcomplex subunit 7 | IPI00219772 | 0.000 | 0.32285 | 2 | 2 | 0 | 0 |
| 1749 | Isoform 1 of N-alpha-acetyltransferase 25, NatB auxiliary subunit | IPI00025890 | 0.000 | 0.32285 | 0 | 0 | 0 | 0 |
| 1750 | Putative uncharacterized protein DKFZp686C1054 | IPI00465054 | 0.000 | 0.32285 | 2 | 2 | 2 | 0 |
| 1751 | Progressive ankylosis protein homolog | IPI00215869 | 0.000 | 0.32285 | 0 | 0 | 0 | 2 |
| 1752 | Isoform 1 of Abl interactor 1 | IPI00431025 | 0.000 | 0.32285 | 0 | 0 | 0 | 2 |
| 1753 | Isoform 2 of Tumor protein D54 | IPI00221178 | 0.000 | 0.32285 | 2 | 0 | 0 | 2 |
| 1754 | Putative uncharacterized protein KIAA0090 | IPI00640734 | 0.000 | 0.32285 | 0 | 0 | 0 | 2 |
| 1755 | Isoform 1 of Protein kinase C and casein kinase substrate in neurons protein 2 | IPI00027009 | 0.000 | 0.32285 | 0 | 0 | 2 | 0 |
| 1756 | Isoform 2 of Ribosomal RNA processing protein 1 homolog B | IPI00032374 | 0.000 | 0.32285 | 0 | 2 | 0 | 2 |
| 1757 | Isoform 1 of Peroxisomal proliferator-activated receptor A-interacting complex 285 kDa proteir | IPI00249304 | 0.000 | 0.32285 | 0 | 0 | 0 | 2 |
| 1758 | Exosome complex exonuclease RRP42 | IPI00014198 | 0.000 | 0.32285 | 0 | 0 | 2 | 0 |
| 1759 | cDNA FLJ59722, highly similar to Retinoblastoma-binding protein 5 | IPI00478230 | 0.000 | 0.32285 | 2 | 0 | 0 | 0 |
| 1760 | Heterochromatin protein 1, binding protein 3 | IPI00640417 | 0.000 | 0.32285 | 2 | 0 | 2 | 0 |
| 1761 | DUS1L protein (Fragment) | IPI00329754 | 0.000 | 0.32285 | 0 | 0 | 2 | 0 |
| 1762 | Isoform 1 of Lysine-specific demethylase 5C | IPI00013185 | 0.000 | 0.32285 | 0 | 0 | 0 | 2 |
| 1763 | Cytochrome P450 monooxygenase | IPI00010218 | 0.000 | 0.32285 | 0 | 2 | 0 | 0 |
| 1764 | 39S ribosomal protein L15, mitochondrial | IPI00023086 | 0.000 | 0.32285 | 0 | 0 | 2 | 0 |
| 1765 | Isoform 1 of Microtubule-associated protein RP/EB family member 2 | IPI00003420 | 0.000 | 0.32285 | 0 | 0 | 2 | 2 |
| 1766 | Isoform 1 of TRM1-like protein | IPI00334914 | 0.000 | 0.32285 | 0 | 2 | 2 | 2 |
| 1767 | Dimethyladenosine transferase 1, mitochondrial | IPI00291525 | 0.000 | 0.32285 | 0 | 2 | 0 | 2 |
| 1768 | Dolichyl-phosphate beta-glucosyltransferase | IPI00002506 | 0.000 | 0.32285 | 0 | 0 | 0 | 2 |
| 1769 | Cyclin-H | IPI00021305 | 0.000 | 0.32285 | 0 | 2 | 0 | 0 |
| 1770 | Isoform DFF45 of DNA fragmentation factor subunit alpha (Fragment) | IPI00010882 | 0.000 | 0.32285 | 0 | 0 | 0 | 0 |
| 1771 | Thioredoxin-related transmembrane protein 4 | IPI00100247 | 0.000 | 0.32285 | 2 | 0 | 0 | 0 |
| 1772 | Eukaryotic translation initiation factor 4A, isoform 2, isoform CRA_b | IPI00030296 | 0.000 | 0.32285 | 2 | 0 | 2 | 2 |
| 1773 | LIM and cysteine-rich domains protein 1 | IPI00303258 | 0.000 | 0.32285 | 0 | 2 | 2 | 2 |
| 1774 | Isoform 1 of Ubiquitin conjugation factor E4 B | IPI00005715 | 0.000 | 0.32285 | 0 | 2 | 2 | 0 |
| 1775 | NEDD4-like E3 ubiquitin-protein ligase WWP2 | IPI00013010 | 0.000 | 0.32285 | 0 | 2 | 2 | 2 |
| 1776 | Interferon regulatory factor 3 | IPI00291901 | 0.000 | 0.32285 | 2 | 3 | 3 | 0 |
| 1777 | Charged multivesicular body protein 2a | IPI00004416 | 0.000 | 0.32285 | 0 | 0 | 0 | 0 |
| 1778 | Vesicle-associated membrane protein 3 | IPI00549343 | 0.000 | 0.32285 | 2 | 2 | 0 | 2 |
| 1779 | Mitochondrial import inner membrane translocase subunit Tim13 | IPI00001589 | 0.000 | 0.32285 | 0 | 2 | 0 | 0 |
| 1780 | Isoform 2 of Transcription factor p65 | IPI00219084 | 0.000 | 0.32285 | 2 | 0 | 2 | 2 |
| 1781 | cDNA FLJ60607, highly similar to Acyl-protein thioesterase 1 | IPI00007321 | 0.000 | 0.32285 | 0 | 0 | 0 | 2 |
| 1782 | Protein FAM50B | IPI00015912 | 0.000 | 0.32285 | 0 | 2 | 0 | 0 |
| 1783 | DNA primase small subunit | IPI00027704 | 0.000 | 0.32285 | 0 | 0 | 0 | 0 |
| 1784 | cDNA FLJ45400 fis, clone BRHIP3028570 | IPI00151888 | 0.000 | 0.32285 | 2 | 0 | 0 | 0 |
| 1785 | WD repeat-containing protein 33 | IPI00106567 | 0.000 | 0.32285 | 0 | 0 | 0 | 0 |
| 1786 | KDEL motif-containing protein 1 | IPI00005270 | 0.000 | 0.32285 | 0 | 3 | 0 | 3 |
| 1787 | Ribonucleases P/MRP protein subunit POP1 | IPI00293331 | 0.000 | 0.32285 | 3 | 0 | 3 | 0 |
| 1788 | cDNA FLJ56280, highly similar to Endoplasmic reticulum-Golgi intermediate compartment protein 1 | IPI00003635 | 0.000 | 0.32285 | 0 | 2 | 0 | 2 |
| 1789 | Tetraspanin-6 | IPI00013449 | 0.000 | 0.32285 | 0 | 0 | 0 | 2 |
| 1790 | Isoform 1 of Zinc transporter ZIP14 | IPI00014236 | 0.000 | 0.32285 | 2 | 0 | 0 | 0 |
| 1791 | Histone acetyltransferase p300 | IPI00020985 | 0.000 | 0.32285 | 0 | 0 | 2 | 0 |
| 1792 | Cathepsin B | IPI00295741 | 0.000 | 0.32285 | 0 | 0 | 2 | 0 |
| 1793 | Isoform 1 of ARF GTPase-activating protein GIT1 | IPI00384861 | 0.000 | 0.32285 | 0 | 0 | 0 | 0 |
| 1794 | Isoform Long of Metastasis-associated protein MTA1 | IPI00012773 | 0.000 | 0.32285 | 2 | 0 | 2 | 0 |
| 1795 | Isoform 4 of Protein LAS1 homolog | IPI00152781 | 0.000 | 0.32285 | 2 | 2 | 2 | 0 |

| No. | Description | Accession number | STN[1] | p-Value[1] | Con_A[2] | Con_B[2] | RG3_A[2] | RG3_B[2] |
|---|---|---|---|---|---|---|---|---|
| 1796 | X-Pro aminopeptidase 1, soluble isoform 2 | IPI00607814 | 0.000 | 0.32285 | 0 | 0 | 2 | 0 |
| 1797 | PDXDC1 protein | IPI00329208 | 0.000 | 0.32285 | 2 | 0 | 0 | 0 |
| 1798 | Astrocytic phosphoprotein PEA-15 | IPI00014850 | 0.000 | 0.32285 | 0 | 2 | 0 | 0 |
| 1799 | Aspartyl/asparaginyl beta-hydroxylase | IPI00294834 | 0.000 | 0.32285 | 0 | 0 | 0 | 2 |
| 1800 | Plexin B2 | IPI00852623 | 0.000 | 0.32285 | 0 | 0 | 2 | 0 |
| 1801 | Uncharacterized protein C11orf73 | IPI00410091 | 0.000 | 0.32285 | 0 | 2 | 0 | 0 |
| 1802 | Protein unc-119 homolog B | IPI00414629 | 0.000 | 0.32285 | 0 | 2 | 2 | 2 |
| 1803 | Nucleolar complex protein 2 homolog | IPI00411886 | 0.000 | 0.32285 | 0 | 2 | 2 | 0 |
| 1804 | Isoform 1 of Protein syndesmos | IPI00031650 | 0.000 | 0.32285 | 0 | 2 | 0 | 0 |
| 1805 | Probable DNA dC->dU-editing enzyme APOBEC-3C | IPI00555878 | 0.000 | 0.32285 | 0 | 0 | 0 | 0 |
| 1806 | Isoform 1 of RNA 3'-terminal phosphate cyclase | IPI00011726 | 0.000 | 0.32285 | 0 | 3 | 2 | 3 |
| 1807 | Isoform 1 of Lysophospholipase-like protein 1 | IPI00059762 | 0.000 | 0.32285 | 0 | 2 | 2 | 2 |
| 1808 | Isoform 1 of Casein kinase I isoform alpha | IPI00183400 | 0.000 | 0.32285 | 2 | 0 | 2 | 0 |
| 1809 | WD repeat and HMG-box DNA-binding protein 1 | IPI00411614 | 0.000 | 0.32285 | 2 | 0 | 0 | 0 |
| 1810 | Ubiquitin-conjugating enzyme E2 T | IPI00023087 | 0.000 | 0.32285 | 2 | 0 | 0 | 0 |
| 1811 | Ubiquitin carboxyl-terminal hydrolase 13 | IPI00024401 | 0.000 | 0.32285 | 2 | 2 | 0 | 0 |
| 1812 | Isoform IIA of Myc box-dependent-interacting protein 1 | IPI00186966 | 0.000 | 0.32285 | 0 | 2 | 0 | 0 |
| 1813 | Receptor expression-enhancing protein 5 | IPI00024670 | 0.000 | 0.32285 | 0 | 0 | 0 | 2 |
| 1814 | Isoform 1 of Anaphase-promoting complex subunit 7 | IPI00008248 | 0.000 | 0.32285 | 0 | 2 | 0 | 0 |
| 1815 | 164 kDa protein | IPI00465246 | 0.000 | 0.32285 | 2 | 2 | 0 | 0 |
| 1816 | Isoform 1 of Myotubularin-related protein 5 | IPI00029446 | 0.000 | 0.32285 | 2 | 0 | 0 | 0 |
| 1817 | Isoform 1 of Putative ATP-dependent RNA helicase DHX57 | IPI00168885 | 0.000 | 0.32285 | 0 | 0 | 0 | 0 |
| 1818 | Isoform 1 of Caldesmon | IPI00014516 | 0.000 | 0.32285 | 4 | 0 | 4 | 0 |
| 1819 | Sentrin-specific protease 3 | IPI00171525 | 0.000 | 0.32285 | 2 | 0 | 0 | 0 |
| 1820 | Rhomboid domain-containing protein 2 | IPI00010255 | 0.000 | 0.32285 | 0 | 0 | 0 | 0 |
| 1821 | dCTP pyrophosphatase 1 | IPI00012197 | 0.000 | 0.32285 | 0 | 0 | 0 | 0 |
| 1822 | Branched-chain-amino-acid aminotransferase | IPI00181135 | 0.000 | 0.32285 | 0 | 2 | 0 | 0 |
| 1823 | Cytosolic Fe-S cluster assembly factor NUBP2 | IPI00644674 | 0.000 | 0.32285 | 0 | 2 | 0 | 2 |
| 1824 | Isoform 2 of Chromodomain-helicase-DNA-binding protein 2 | IPI00023109 | 0.000 | 0.32285 | 0 | 0 | 0 | 0 |
| 1825 | Isoform 1 of Cellular tumor antigen p53 | IPI00025087 | 0.000 | 0.32285 | 0 | 0 | 2 | 0 |
| 1826 | Sperm-associated antigen 7 | IPI00006863 | 0.000 | 0.32285 | 2 | 2 | 0 | 2 |
| 1827 | Isoform LAMP-2A of Lysosome-associated membrane glycoprotein 2 | IPI00009030 | 0.000 | 0.32285 | 0 | 0 | 0 | 2 |
| 1828 | Isoform UBF1 of Nucleolar transcription factor 1 | IPI00014533 | 0.000 | 0.32285 | 0 | 2 | 0 | 0 |
| 1829 | Isoform 1 of Integral membrane protein 2C | IPI00016014 | 0.000 | 0.32285 | 0 | 2 | 0 | 0 |
| 1830 | Glutamate--cysteine ligase regulatory subunit | IPI00010090 | 0.000 | 0.32285 | 0 | 2 | 2 | 2 |
| 1831 | Ubiquitin-like domain-containing CTD phosphatase 1 | IPI00291669 | 0.000 | 0.32285 | 0 | 0 | 0 | 2 |
| 1832 | Putative uncharacterized protein CASP2 | IPI00018345 | 0.000 | 0.32285 | 2 | 0 | 0 | 0 |
| 1833 | Isoform 1 of Vacuolar protein sorting-associated protein 16 homolog | IPI00305438 | 0.000 | 0.32285 | 2 | 0 | 0 | 2 |
| 1834 | Keratin, type II cytoskeletal 6B | IPI00293665 | 0.000 | 0.32285 | 2 | 2 | 2 | 2 |
| 1835 | Myosin-Ia | IPI00294386 | 0.000 | 0.32285 | 2 | 2 | 2 | 0 |
| 1836 | Cohesin subunit SA-1 | IPI00025158 | 0.000 | 0.32285 | 0 | 0 | 2 | 2 |
| 1837 | Isoform 1 of Mannose-1-phosphate guanyltransferase alpha | IPI00101782 | 0.000 | 0.32285 | 0 | 0 | 0 | 2 |
| 1838 | Isoform 1 of Zinc finger FYVE domain-containing protein 16 | IPI00424460 | 0.000 | 0.32285 | 0 | 0 | 0 | 2 |
| 1839 | Cob(I)yrinic acid a,c-diamide adenosyltransferase, mitochondrial | IPI00029665 | 0.000 | 0.32285 | 0 | 0 | 2 | 2 |
| 1840 | N(G),N(G)-dimethylarginine dimethylaminohydrolase 1 | IPI00220342 | 0.000 | 0.32285 | 0 | 0 | 2 | 2 |
| 1841 | Isoform Mitochondrial of Phospholipid hydroperoxide glutathione peroxidase, mitochondrial | IPI00304814 | 0.000 | 0.32285 | 2 | 0 | 0 | 0 |
| 1842 | Isoform 1 of Polyglutamine-binding protein 1 | IPI00024698 | 0.000 | 0.32285 | 0 | 2 | 0 | 0 |
| 1843 | Isoform 1 of Centromere protein M | IPI00031566 | 0.000 | 0.32285 | 0 | 0 | 0 | 0 |
| 1844 | Isoform A of SWI/SNF-related matrix-associated actin-dependent regulator of chromatin subfamily B | IPI00029695 | 0.000 | 0.32285 | 2 | 2 | 2 | 0 |
| 1845 | Isoform 1 of Acetoacetyl-CoA synthetase | IPI00217272 | 0.000 | 0.32285 | 0 | 0 | 0 | 2 |
| 1846 | Methionine aminopeptidase 2 | IPI00033036 | 0.000 | 0.32285 | 2 | 2 | 2 | 0 |
| 1847 | cDNA FLJ55543, highly similar to Phosphoacetylglucosamine mutase | IPI00030116 | 0.000 | 0.32285 | 0 | 0 | 0 | 2 |
| 1848 | Isoform 1 of Cell division protein kinase 12 | IPI00021175 | 0.000 | 0.32285 | 0 | 0 | 0 | 2 |
| 1849 | Phosphoglycolate phosphatase | IPI00177008 | 0.000 | 0.32285 | 0 | 0 | 0 | 2 |
| 1850 | Isoform 1 of Vacuolar-sorting protein SNF8 | IPI00101524 | 0.000 | 0.32285 | 0 | 3 | 0 | 3 |
| 1851 | Isoform 1 of Serine/threonine-protein phosphatase 4 regulatory subunit 3A | IPI00217013 | 0.000 | 0.32285 | 0 | 2 | 0 | 0 |
| 1852 | Isoform 1 of Rab3 GTPase-activating protein catalytic subunit | IPI00014235 | 0.000 | 0.32285 | 0 | 2 | 2 | 2 |
| 1853 | U3 small nucleolar RNA-associated protein 18 homolog | IPI00000733 | 0.000 | 0.32285 | 0 | 0 | 0 | 2 |
| 1854 | Pumilio domain-containing protein KIAA0020 | IPI00791325 | 0.000 | 0.32285 | 2 | 2 | 0 | 0 |
| 1855 | Isoform 1 of Protein SDA1 homolog | IPI00018240 | 0.000 | 0.32285 | 0 | 2 | 0 | 0 |
| 1856 | Isoform 1 of Multiple inositol polyphosphate phosphatase 1 | IPI00293748 | 0.000 | 0.32285 | 0 | 2 | 0 | 0 |
| 1857 | Conserved hypothetical protein | IPI00477526 | 0.000 | 0.32285 | 0 | 2 | 0 | 0 |
| 1858 | Isoform 2 of Transportin-2 | IPI00164417 | 0.000 | 0.32285 | 0 | 2 | 0 | 2 |
| 1859 | Isoform A of Protein CutA | IPI00034319 | 0.000 | 0.32285 | 0 | 2 | 0 | 0 |
| 1860 | Elongation factor G 2, mitochondrial precursor | IPI00071703 | 0.000 | 0.32285 | 0 | 0 | 2 | 0 |
| 1861 | S-adenosyl-L-methionine-dependent methyltransferase FTSJD2 | IPI00166153 | 0.000 | 0.32285 | 0 | 0 | 0 | 2 |
| 1862 | Isoform 1 of AP-1 complex subunit sigma-1A | IPI00152898 | 0.000 | 0.32285 | 0 | 2 | 0 | 0 |
| 1863 | Isoform 2 of Heme-binding protein 2 | IPI00003799 | 0.000 | 0.32285 | 0 | 0 | 0 | 0 |
| 1864 | Isoform 2 of Neurobeachin-like protein 2 | IPI00852973 | 0.000 | 0.32285 | 0 | 0 | 0 | 0 |
| 1865 | HLA class I histocompatibility antigen, B-7 alpha chain | IPI00004657 | 0.000 | 0.32285 | 0 | 2 | 0 | 2 |
| 1866 | Dynactin subunit 4 | IPI00550852 | 0.000 | 0.32285 | 2 | 0 | 2 | 0 |
| 1867 | Replication factor C subunit 3 | IPI00031521 | 0.000 | 0.32285 | 0 | 0 | 0 | 2 |
| 1868 | GDP-L-fucose synthase | IPI00014361 | 0.000 | 0.32285 | 0 | 0 | 2 | 0 |
| 1869 | UPF0600 protein C5orf51 | IPI00374272 | 0.000 | 0.32285 | 0 | 0 | 0 | 2 |
| 1870 | sorting nexin-6 isoform a | IPI00258833 | 0.000 | 0.32285 | 2 | 0 | 2 | 2 |
| 1871 | Isoform 2 of Pre-mRNA-splicing factor ISY1 homolog | IPI00063673 | 0.000 | 0.32285 | 0 | 0 | 0 | 2 |
| 1872 | Zinc finger protein ubi-d4 | IPI00023322 | 0.000 | 0.32285 | 2 | 2 | 0 | 0 |
| 1873 | Isoform 2 of Plakophilin-2 | IPI00005264 | 0.000 | 0.32285 | 0 | 0 | 2 | 0 |
| 1874 | Isoform 1 of Coiled-coil domain-containing protein 51 | IPI00153023 | 0.000 | 0.32285 | 0 | 2 | 0 | 0 |
| 1875 | Isoform 1 of Hydroxyacylglutathione hydrolase, mitochondrial | IPI00003933 | 0.000 | 0.32285 | 2 | 0 | 0 | 0 |
| 1876 | Reticulocalbin-2 | IPI00029628 | 0.000 | 0.32285 | 2 | 2 | 0 | 2 |
| 1877 | Protein pelota homolog | IPI00106698 | 0.000 | 0.32285 | 0 | 2 | 2 | 2 |
| 1878 | Isoform 1 of WD repeat-containing protein 26 | IPI00414197 | 0.000 | 0.32285 | 0 | 0 | 0 | 0 |
| 1879 | Isoform 1 of Mannose-6-phosphate isomerase | IPI00219358 | 0.000 | 0.32285 | 0 | 2 | 0 | 2 |
| 1880 | Isoform I of Septin-6 | IPI00216139 | 0.000 | 0.32285 | 0 | 0 | 2 | 0 |
| 1881 | Beta-centractin | IPI00029469 | 0.000 | 0.32285 | 0 | 2 | 0 | 2 |
| 1882 | Isoform 1 of AP-1 complex subunit mu-2 | IPI00002552 | 0.000 | 0.32285 | 0 | 0 | 2 | 0 |
| 1883 | RAB4A, member RAS oncogene family variant | IPI00480056 | 0.000 | 0.32285 | 0 | 0 | 0 | 2 |
| 1884 | Nucleoporin Nup43 | IPI00742943 | 0.000 | 0.32285 | 2 | 0 | 2 | 0 |
| 1885 | Isoform 1 of SAC3 domain-containing protein 1 | IPI00854724 | 0.000 | 0.32285 | 0 | 2 | 0 | 0 |
| 1886 | Transcription initiation factor TFIID subunit 9 | IPI00002993 | 0.000 | 0.32285 | 0 | 0 | 0 | 0 |
| 1887 | Isoform 1 of Uncharacterized protein C1orf93 | IPI00152199 | 0.000 | 0.32285 | 0 | 0 | 2 | 0 |
| 1888 | Isoform 2 of VIP36-like protein | IPI00218337 | 0.000 | 0.32285 | 0 | 0 | 2 | 2 |
| 1889 | Isoform 1 of Set1/Ash2 histone methyltransferase complex subunit ASH2 | IPI00328658 | 0.000 | 0.32285 | 0 | 0 | 2 | 0 |

| No. | Description | Accession number | STN[1] | p-Value[1] | Con_A[2] | Con_B[2] | RG3_A[2] | RG3_B[2] |
|---|---|---|---|---|---|---|---|---|
| 1890 | Isoform 1 of ADP-ribosylation factor GTPase-activating protein 1 | IPI00175169 | 0.000 | 0.32285 | 3 | 0 | 3 | 0 |
| 1891 | Isoform 1 of tRNA guanosine-2'-O-methyltransferase TRM11 homolog | IPI00470606 | 0.000 | 0.32285 | 2 | 2 | 0 | 0 |
| 1892 | RNA-binding protein 12 | IPI00550308 | 0.000 | 0.32285 | 0 | 0 | 0 | 2 |
| 1893 | 39S ribosomal protein L16, mitochondrial | IPI00000821 | 0.000 | 0.32285 | 0 | 0 | 2 | 0 |
| 1894 | UV excision repair protein RAD23 homolog A | IPI00008219 | 0.000 | 0.32285 | 0 | 0 | 0 | 2 |
| 1895 | Isoform 1 of Testin | IPI00024097 | 0.000 | 0.32285 | 0 | 2 | 2 | 2 |
| 1896 | U3 small nucleolar ribonucleoprotein protein MPP10 | IPI00012149 | 0.000 | 0.32285 | 0 | 2 | 0 | 0 |
| 1897 | Ribosomal RNA-processing protein 8 | IPI00304932 | 0.000 | 0.32285 | 0 | 2 | 0 | 0 |
| 1898 | Isoform 1 of Probable Xaa-Pro aminopeptidase 3 | IPI00550192 | 0.000 | 0.32285 | 0 | 0 | 0 | 2 |
| 1899 | Cyclin B1 | IPI00294696 | 0.000 | 0.32285 | 0 | 2 | 0 | 0 |
| 1900 | NADH dehydrogenase [ubiquinone] iron-sulfur protein 7, mitochondrial | IPI00307749 | 0.000 | 0.32285 | 0 | 0 | 2 | 0 |
| 1901 | Agmatinase, mitochondrial | IPI00305360 | 0.000 | 0.32285 | 0 | 0 | 2 | 0 |
| 1902 | Ribosomal RNA processing protein 1 homolog A | IPI00550766 | 0.000 | 0.32285 | 2 | 0 | 0 | 0 |
| 1903 | PIH1 domain-containing protein 1 | IPI00550995 | 0.000 | 0.32285 | 0 | 0 | 0 | 2 |
| 1904 | Isoform 1 of Leucine-rich repeat flightless-interacting protein 2 | IPI00007277 | 0.000 | 0.32285 | 2 | 0 | 2 | 0 |
| 1905 | 37 kDa protein | IPI00032799 | 0.000 | 0.32285 | 0 | 0 | 0 | 0 |
| 1906 | Cleavage and polyadenylation specificity factor subunit 3 | IPI00007818 | 0.000 | 0.32285 | 0 | 0 | 2 | 0 |
| 1907 | cDNA FLJ53160, highly similar to Zyxin | IPI00871311 | 0.000 | 0.32285 | 2 | 0 | 2 | 2 |
| 1908 | Ladinin-1 | IPI00514234 | 0.000 | 0.32285 | 0 | 0 | 0 | 0 |
| 1909 | Zinc finger protein 622 | IPI00056499 | 0.000 | 0.32285 | 2 | 2 | 0 | 0 |
| 1910 | DNA replication complex GINS protein PSF2 | IPI00007146 | 0.000 | 0.32285 | 0 | 0 | 2 | 0 |
| 1911 | Isoform 2 of Polyhomeotic-like protein 2 | IPI00419684 | 0.000 | 0.32285 | 0 | 0 | 2 | 0 |
| 1912 | ATP-dependent RNA helicase DHX8 | IPI00031508 | 0.000 | 0.32285 | 0 | 2 | 0 | 0 |
| 1913 | Sortilin-related receptor | IPI00022608 | 0.000 | 0.32285 | 0 | 0 | 0 | 0 |
| 1914 | Isoform 1 of Mammalian ependymin-related protein 1 | IPI00259102 | 0.000 | 0.32285 | 0 | 2 | 0 | 2 |
| 1915 | Calcium-binding protein p22 | IPI00218924 | 0.000 | 0.32285 | 0 | 0 | 0 | 0 |
| 1916 | cDNA FLJ54848, highly similar to tRNA-splicing endonuclease subunit Sen34 | IPI00451941 | 0.000 | 0.32285 | 0 | 0 | 2 | 0 |
| 1917 | Isoform 1 of H/ACA ribonucleoprotein complex subunit 1 | IPI00302176 | 0.000 | 0.32285 | 0 | 2 | 0 | 0 |
| 1918 | UDP-glucose 4-epimerase | IPI00553131 | 0.000 | 0.32285 | 0 | 2 | 0 | 0 |
| 1919 | Coiled-coil domain-containing protein 134 | IPI00302674 | 0.000 | 0.32285 | 0 | 0 | 0 | 0 |
| 1920 | Alpha-galactosidase A | IPI00025869 | 0.000 | 0.32285 | 0 | 0 | 0 | 0 |
| 1921 | Isoform 1 of Histone-lysine N-methyltransferase MLL | IPI00009286 | 0.000 | 0.32285 | 0 | 0 | 0 | 0 |
| 1922 | Isoform 1 of Phosphatidylinositol 4-kinase alpha | IPI00070943 | 0.000 | 0.32285 | 0 | 0 | 0 | 0 |
| 1923 | Isoform 1 of GPI transamidase component PIG-S | IPI00465308 | 0.000 | 0.32285 | 2 | 0 | 0 | 0 |
| 1924 | Isoform 3 of Shootin-1 | IPI00448751 | 0.000 | 0.32285 | 0 | 0 | 0 | 2 |
| 1925 | Vacuolar protein sorting-associated protein 28 homolog | IPI00007155 | 0.000 | 0.32285 | 0 | 0 | 0 | 2 |
| 1926 | ATP-binding domain-containing protein 4 | IPI00063121 | 0.000 | 0.32285 | 2 | 0 | 0 | 2 |
| 1927 | Phosphatidylinositol 4-kinase type 2-alpha | IPI00020124 | 0.000 | 0.32285 | 0 | 0 | 0 | 2 |
| 1928 | Probable E3 ubiquitin-protein ligase HERC2 | IPI00005826 | 0.000 | 0.32285 | 0 | 0 | 0 | 2 |
| 1929 | Perilipin-2 | IPI00293307 | 0.000 | 0.32285 | 0 | 0 | 2 | 0 |
| 1930 | Probable ATP-dependent RNA helicase DDX28 | IPI00020050 | 0.000 | 0.32285 | 0 | 0 | 0 | 0 |
| 1931 | Isoform 2 of Arf-GAP with Rho-GAP domain, ANK repeat and PH domain-containing protein 1 | IPI00220421 | 0.000 | 0.32285 | 0 | 0 | 2 | 0 |
| 1932 | ATP-dependent RNA helicase DDX54 isoform 1 | IPI00152510 | 0.000 | 0.32285 | 0 | 0 | 0 | 0 |
| 1933 | Isoform 1 of Lymphoid-specific helicase | IPI00010590 | 0.000 | 0.32285 | 2 | 0 | 0 | 0 |
| 1934 | Isoform 2 of Dephospho-CoA kinase domain-containing protein | IPI00015737 | 0.000 | 0.32285 | 0 | 0 | 0 | 0 |
| 1935 | Protein kinase C iota type | IPI00016639 | 0.000 | 0.32285 | 0 | 0 | 0 | 0 |
| 1936 | Isoform 1 of GPN-loop GTPase 3 | IPI00470580 | 0.000 | 0.32285 | 2 | 0 | 2 | 0 |
| 1937 | Isoform 1 of Polyadenylate-binding protein-interacting protein 1 | IPI00021466 | 0.000 | 0.32285 | 0 | 0 | 2 | 0 |
| 1938 | Isoform 2 of Oxidation resistance protein 1 | IPI00298348 | 0.000 | 0.32285 | 0 | 2 | 0 | 0 |
| 1939 | Origin recognition complex subunit 4 | IPI00015164 | 0.000 | 0.32285 | 0 | 0 | 0 | 2 |
| 1940 | Prolactin regulatory element-binding protein | IPI00033349 | 0.000 | 0.32285 | 0 | 0 | 0 | 2 |
| 1941 | AP-1 complex subunit sigma-2 | IPI00922006 | 0.000 | 0.32285 | 2 | 0 | 0 | 0 |
| 1942 | Isoform 1 of Mediator of RNA polymerase II transcription subunit 16 | IPI00037401 | 0.000 | 0.32285 | 0 | 0 | 0 | 2 |
| 1943 | Isoform 3 of Parkinson disease 7 domain-containing protein 1 | IPI00167976 | 0.000 | 0.32285 | 0 | 2 | 0 | 2 |
| 1944 | Charged multivesicular body protein 3 | IPI00100673 | 0.000 | 0.32285 | 0 | 2 | 0 | 0 |
| 1945 | Isoform 2 of UPF0465 protein C5orf33 | IPI00431405 | 0.000 | 0.32285 | 2 | 0 | 2 | 0 |
| 1946 | 28S ribosomal protein S14, mitochondrial | IPI00005050 | 0.000 | 0.32285 | 2 | 0 | 2 | 0 |
| 1947 | Isoform 1 of Trafficking protein particle complex subunit 2-like protein | IPI00007819 | 0.000 | 0.32285 | 0 | 0 | 0 | 2 |
| 1948 | Tetratricopeptide repeat protein 1 | IPI00016912 | 0.000 | 0.32285 | 2 | 0 | 0 | 0 |
| 1949 | Isoform 1 of Melanoma inhibitory activity protein 3 | IPI00455473 | 0.000 | 0.32285 | 0 | 0 | 0 | 0 |
| 1950 | Isoform 1 of Quinone oxidoreductase PIG3 | IPI00384643 | 0.000 | 0.32285 | 0 | 0 | 2 | 0 |
| 1951 | Isoform 1 of Regulator of nonsense transcripts 3B | IPI00023409 | 0.000 | 0.32285 | 0 | 0 | 2 | 0 |
| 1952 | Isoform 1 of NEDD8-conjugating enzyme UBE2F | IPI00056432 | 0.000 | 0.32285 | 0 | 0 | 0 | 0 |
| 1953 | Isoform 2 of N-acetylserotonin O-methyltransferase-like protein | IPI00249080 | 0.000 | 0.32285 | 2 | 2 | 0 | 0 |
| 1954 | Maleylacetoacetate isomerase | IPI00013809 | 0.000 | 0.32285 | 0 | 0 | 0 | 0 |
| 1955 | Isoform 1 of Endophilin-B1 | IPI00006558 | 0.000 | 0.32285 | 0 | 0 | 0 | 2 |
| 1956 | Proline-rich protein PRCC | IPI00294618 | 0.000 | 0.32285 | 0 | 0 | 2 | 2 |
| 1957 | cDNA FLJ54752, highly similar to Poly(rC)-binding protein 2 | IPI00788837 | 0.000 | 0.32285 | 0 | 0 | 0 | 0 |
| 1958 | Isoform 1 of Rho guanine nucleotide exchange factor 12 | IPI00022164 | 0.000 | 0.32285 | 0 | 2 | 0 | 0 |
| 1959 | cDNA: FLJ22221 fis, clone HRC01651 | IPI00184854 | 0.000 | 0.32285 | 0 | 0 | 0 | 0 |
| 1960 | Isoform 1 of Pogo transposable element with ZNF domain | IPI00410717 | 0.000 | 0.32285 | 2 | 0 | 2 | 0 |
| 1961 | Isoform 3 of Poly [ADP-ribose] polymerase 16 | IPI00297151 | 0.000 | 0.32285 | 0 | 0 | 0 | 0 |
| 1962 | Isoform 2 of Proto-oncogene tyrosine-protein kinase Src | IPI00328867 | 0.000 | 0.32285 | 0 | 0 | 0 | 2 |
| 1963 | Isoform 1 of Golgin subfamily A member 3 | IPI00305267 | 0.000 | 0.32285 | 0 | 0 | 0 | 0 |
| 1964 | PNAS-117 | IPI00020827 | 0.000 | 0.32285 | 0 | 0 | 0 | 2 |
| 1965 | Isoform 1 of NHL repeat-containing protein 2 | IPI00301051 | 0.000 | 0.32285 | 0 | 2 | 0 | 0 |
| 1966 | DNA excision repair protein ERCC-6-like | IPI00552569 | 0.000 | 0.32285 | 0 | 0 | 0 | 0 |
| 1967 | cDNA FLJ56157, highly similar to Glucosylceramidase | IPI00021807 | 0.000 | 0.32285 | 0 | 0 | 0 | 2 |
| 1968 | Isoform 3 of Tyrosine-protein kinase Fyn | IPI00166845 | 0.000 | 0.32285 | 0 | 0 | 0 | 2 |
| 1969 | KIAA1033 protein | IPI00298991 | 0.000 | 0.32285 | 0 | 0 | 2 | 0 |
| 1970 | Maspardin | IPI00010248 | 0.000 | 0.32285 | 0 | 0 | 0 | 2 |
| 1971 | Putative uncharacterized protein ZNF326 | IPI00337602 | 0.000 | 0.32285 | 2 | 0 | 0 | 0 |
| 1972 | GDH/6PGL endoplasmic bifunctional protein | IPI00607861 | 0.000 | 0.32285 | 0 | 0 | 2 | 0 |
| 1973 | Isoform 1 of YTH domain family protein 1 | IPI00221345 | 0.000 | 0.32285 | 0 | 2 | 0 | 0 |
| 1974 | WD repeat-containing protein 70 | IPI00300060 | 0.000 | 0.32285 | 2 | 0 | 0 | 0 |
| 1975 | Procollagen galactosyltransferase 1 | IPI00168262 | 0.000 | 0.32285 | 0 | 0 | 0 | 0 |
| 1976 | cDNA FLJ54030, highly similar to Polymerase delta-interacting protein 3 | IPI00440688 | 0.000 | 0.32285 | 0 | 0 | 2 | 0 |
| 1977 | Isoform 1 of Protein zwilch homolog | IPI00329679 | 0.000 | 0.32285 | 0 | 0 | 0 | 0 |
| 1978 | Proteolipid protein 2 | IPI00030362 | 0.000 | 0.32285 | 0 | 0 | 2 | 0 |
| 1979 | Isoform 1 of Copine-7 | IPI00002657 | 0.000 | 0.32285 | 0 | 0 | 0 | 0 |
| 1980 | Isoform 3 of F-box only protein 22 | IPI00169168 | 0.000 | 0.32285 | 0 | 0 | 0 | 0 |
| 1981 | Isoform 1 of Interferon regulatory factor 2-binding protein 2 | IPI00376199 | 0.000 | 0.32285 | 0 | 0 | 0 | 2 |
| 1982 | Protein FAM96B | IPI00007024 | 0.000 | 0.32285 | 0 | 0 | 2 | 0 |
| 1983 | Mitochondrial import inner membrane translocase subunit Tim8 A | IPI00028376 | 0.000 | 0.32285 | 0 | 0 | 2 | 0 |
| 1984 | Acylphosphatase-1 | IPI00221117 | 0.000 | 0.32285 | 0 | 0 | 0 | 0 |

| No. | Description | Accession number | STN[1] | p-Value[1] | Con_A[2] | Con_B[2] | RG3_A[2] | RG3_B[2] |
|---|---|---|---|---|---|---|---|---|
| 1985 | Ubiquitin protein ligase E3 component n-recognin 4 | IPI00514902 | 0.000 | 0.32285 | 0 | 2 | 0 | 0 |
| 1986 | Neighbor of COX4 | IPI00005740 | 0.000 | 0.32285 | 0 | 0 | 0 | 0 |
| 1987 | Isoform 1 of Transcriptional repressor p66-alpha | IPI00410330 | 0.000 | 0.32285 | 0 | 2 | 2 | 0 |
| 1988 | tRNA (guanine-N(1)-)-methyltransferase | IPI00455268 | 0.000 | 0.32285 | 0 | 2 | 2 | 0 |
| 1989 | Isoform 4 of Inhibitor of nuclear factor kappa-B kinase-interacting protein | IPI00043598 | 0.000 | 0.32285 | 0 | 0 | 2 | 0 |
| 1990 | Microfibrillar-associated protein 1 | IPI00022790 | 0.000 | 0.32285 | 0 | 0 | 0 | 0 |
| 1991 | Isoform 1 of Ribonucleoside-diphosphate reductase subunit M2 B | IPI00100213 | 0.000 | 0.32285 | 0 | 0 | 0 | 2 |
| 1992 | Isoform 2 of mRNA cap guanine-N7 methyltransferase | IPI00410657 | 0.000 | 0.32285 | 0 | 0 | 2 | 0 |
| 1993 | Fumarylacetoacetase | IPI00031708 | 0.000 | 0.32285 | 2 | 0 | 0 | 0 |
| 1994 | Serine/threonine-protein kinase 12 | IPI00176642 | 0.000 | 0.32285 | 0 | 2 | 0 | 0 |
| 1995 | NF-kappa-B-repressing factor | IPI00005675 | 0.000 | 0.32285 | 0 | 0 | 2 | 2 |
| 1996 | Isoform 2 of DNA-directed RNA polymerase I subunit RPA2 | IPI00026445 | 0.000 | 0.32285 | 0 | 0 | 0 | 0 |
| 1997 | Vacuolar protein sorting-associated protein 33B | IPI00032905 | 0.000 | 0.32285 | 0 | 0 | 0 | 0 |
| 1998 | Kinesin-like protein KIFC1 | IPI00306400 | 0.000 | 0.32285 | 0 | 2 | 0 | 0 |
| 1999 | Calcineurin subunit B type 1 | IPI00027464 | 0.000 | 0.32285 | 0 | 0 | 0 | 2 |
| 2000 | Proteasome subunit beta type-10 | IPI00027933 | 0.000 | 0.32285 | 0 | 2 | 0 | 0 |
| 2001 | NADH dehydrogenase [ubiquinone] iron-sulfur protein 5 | IPI00220063 | 0.000 | 0.32285 | 0 | 0 | 0 | 2 |
| 2002 | tyrosine-protein phosphatase non-receptor type 2 | IPI00106928 | 0.000 | 0.32285 | 0 | 2 | 0 | 2 |
| 2003 | Isoform 2 of Integrator complex subunit 7 | IPI00645022 | 0.000 | 0.32285 | 0 | 0 | 2 | 0 |
| 2004 | EPS8L2 protein | IPI00414315 | 0.000 | 0.32285 | 0 | 0 | 0 | 2 |
| 2005 | Isoform 2 of DNA-3-methyladenine glycosylase | IPI00218495 | 0.000 | 0.32285 | 0 | 2 | 0 | 2 |
| 2006 | U4/U6.U5 small nuclear ribonucleoprotein 27 kDa protein | IPI00017289 | 0.000 | 0.32285 | 0 | 0 | 0 | 0 |
| 2007 | Isoform Long of Glutaryl-CoA dehydrogenase, mitochondrial | IPI00024317 | 0.000 | 0.32285 | 0 | 0 | 0 | 2 |
| 2008 | MMP37-like protein, mitochondrial | IPI00060287 | 0.000 | 0.32285 | 0 | 0 | 2 | 0 |
| 2009 | Isoform 2 of Multiple inositol polyphosphate phosphatase 1 | IPI00028553 | 0.000 | 0.32285 | 0 | 0 | 2 | 0 |
| 2010 | Isoform 1 of Splicing factor, arginine/serine-rich 12 | IPI00103497 | 0.000 | 0.32285 | 0 | 2 | 0 | 2 |
| 2011 | NADP-dependent malic enzyme, mitochondrial | IPI00003970 | 0.000 | 0.32285 | 0 | 0 | 0 | 2 |
| 2012 | Isoform 1 of Lysosomal acid lipase/cholesteryl ester hydrolase | IPI00007207 | 0.000 | 0.32285 | 0 | 2 | 0 | 0 |
| 2013 | Importin subunit alpha-3 | IPI00299033 | 0.000 | 0.32285 | 2 | 0 | 0 | 0 |
| 2014 | Metastasis-associated in colon cancer protein 1 | IPI00376087 | 0.000 | 0.32285 | 0 | 2 | 0 | 0 |
| 2015 | Isoform 3 of Formin-binding protein 1-like | IPI00015580 | 0.000 | 0.32285 | 2 | 2 | 0 | 0 |
| 2016 | Ribonuclease P protein subunit p38 | IPI00019195 | 0.000 | 0.32285 | 0 | 0 | 2 | 2 |
| 2017 | Mitochondrial intermediate peptidase | IPI00241860 | 0.000 | 0.32285 | 0 | 0 | 0 | 2 |
| 2018 | 69 kDa protein | IPI00172590 | 0.000 | 0.32285 | 0 | 2 | 0 | 0 |
| 2019 | 28S ribosomal protein S15, mitochondrial | IPI00550037 | 0.000 | 0.32285 | 0 | 0 | 0 | 2 |
| 2020 | Isoform 2 of Glutaminase kidney isoform, mitochondrial | IPI00215685 | 0.000 | 0.32285 | 0 | 0 | 0 | 0 |
| 2021 | Ubiquinone biosynthesis protein COQ7 homolog | IPI00294073 | 0.000 | 0.32285 | 0 | 0 | 2 | 2 |
| 2022 | Isoform 1 of Hydrocephalus-inducing protein homolog | IPI00647188 | 0.000 | 0.32285 | 0 | 0 | 0 | 0 |
| 2023 | C-Myc-binding protein | IPI00871174 | 0.000 | 0.32285 | 2 | 0 | 0 | 0 |
| 2024 | Glutamine-dependent NAD(+) synthetase | IPI00306689 | 0.000 | 0.32285 | 2 | 2 | 2 | 0 |
| 2025 | Isoform 5 of Methyltransferase-like protein 13 | IPI00384061 | 0.000 | 0.32285 | 0 | 2 | 2 | 2 |
| 2026 | Fumarylacetoacetate hydrolase domain-containing protein 2A | IPI00329742 | 0.000 | 0.32285 | 0 | 2 | 0 | 0 |
| 2027 | cDNA FLJ56469, highly similar to Propionyl-CoA carboxylase alpha chain, mitochondrial | IPI00552419 | 0.000 | 0.32285 | 0 | 0 | 2 | 2 |
| 2028 | B-cell lymphoma/leukemia 10 | IPI00022477 | 0.000 | 0.32285 | 0 | 0 | 0 | 2 |
| 2029 | Cell division cycle protein 123 homolog | IPI00005670 | 0.000 | 0.32285 | 0 | 2 | 0 | 0 |
| 2030 | Isoform 3 of Sigma non-opioid intracellular receptor 1 | IPI00004267 | 0.000 | 0.32285 | 2 | 0 | 0 | 0 |
| 2031 | Isoform 1 of Terminal uridylyltransferase 4 | IPI00289861 | 0.000 | 0.32285 | 0 | 0 | 0 | 2 |
| 2032 | Transducin beta-like protein 2 | IPI00000948 | 0.000 | 0.32285 | 3 | 0 | 3 | 0 |
| 2033 | Isoform 1 of Zinc finger CCCH-type antiviral protein 1-like | IPI00062866 | 0.000 | 0.32285 | 0 | 0 | 0 | 0 |
| 2034 | Argininosuccinate lyase | IPI00220267 | 0.000 | 0.32285 | 0 | 0 | 0 | 0 |
| 2035 | Guanine nucleotide-binding protein subunit alpha-11 | IPI00305551 | 0.000 | 0.32285 | 0 | 2 | 0 | 0 |
| 2036 | Isoform Long of Vesicle transport through interaction with t-SNAREs homolog 1B | IPI00063784 | 0.000 | 0.32285 | 0 | 2 | 0 | 0 |
| 2037 | Diphosphomevalonate decarboxylase | IPI00022745 | 0.000 | 0.32285 | 0 | 2 | 0 | 0 |
| 2038 | Isoform 2 of Septin-8 | IPI00022082 | 0.000 | 0.32285 | 2 | 0 | 0 | 0 |
| 2039 | Isoform 1 of ATP-binding cassette sub-family F member 3 | IPI00465160 | 0.000 | 0.32285 | 0 | 2 | 0 | 0 |
| 2040 | Nucleoside diphosphate kinase 3 | IPI00012315 | 0.000 | 0.32285 | 0 | 2 | 0 | 0 |
| 2041 | V-type proton ATPase subunit d 1 | IPI00034159 | 0.000 | 0.32285 | 0 | 2 | 0 | 0 |
| 2042 | DDB1- and CUL4-associated factor 7 | IPI00006754 | 0.000 | 0.32285 | 0 | 0 | 2 | 0 |
| 2043 | MAP kinase-activated protein kinase 3 | IPI00005777 | 0.000 | 0.32285 | 0 | 0 | 2 | 0 |
| 2044 | Peptidyl-tRNA hydrolase family protein | IPI00048572 | 0.000 | 0.32285 | 0 | 0 | 0 | 2 |
| 2045 | Isoform 1 of Stromal membrane-associated protein 1 | IPI00102096 | 0.000 | 0.32285 | 0 | 0 | 0 | 0 |
| 2046 | GA-binding protein alpha chain | IPI00299413 | 0.000 | 0.32285 | 0 | 0 | 0 | 2 |
| 2047 | Isoform 1 of Ras-related GTP-binding protein B | IPI00010317 | 0.000 | 0.32285 | 0 | 2 | 2 | 0 |
| 2048 | Isoform Long of Deoxyhypusine synthase | IPI00026829 | 0.000 | 0.32285 | 0 | 2 | 0 | 0 |
| 2049 | cDNA FLJ31776 fis, clone NT2RI2008141, highly similar to CALUMENIN | IPI00789155 | 0.000 | 0.32285 | 2 | 0 | 0 | 0 |
| 2050 | Isoform 2 of Protein PAT1 homolog 1 | IPI00760958 | 0.000 | 0.32285 | 0 | 2 | 0 | 2 |
| 2051 | CTP synthase II | IPI00553127 | 0.000 | 0.32285 | 0 | 0 | 0 | 0 |
| 2052 | Isoform 1 of Tuftelin-interacting protein 11 | IPI00015924 | 0.000 | 0.32285 | 0 | 0 | 2 | 0 |
| 2053 | Isoform 1 of TraB domain-containing protein | IPI00008732 | 0.000 | 0.32285 | 0 | 0 | 0 | 0 |
| 2054 | Isoform 1 of Microtubule-associated protein 4 | IPI00396171 | 0.000 | 0.32285 | 0 | 0 | 0 | 2 |
| 2055 | Isoform LYN A of Tyrosine-protein kinase Lyn | IPI00298625 | 0.000 | 0.32285 | 0 | 2 | 0 | 0 |
| 2056 | Putative heat shock 70 kDa protein 7 | IPI00011134 | 0.000 | 0.32285 | 0 | 0 | 2 | 0 |
| 2057 | Cyclin-dependent kinases regulatory subunit 1 | IPI00015104 | 0.000 | 0.32285 | 0 | 0 | 2 | 0 |
| 2058 | Large subunit GTPase 1 homolog | IPI00300094 | 0.000 | 0.32285 | 0 | 2 | 0 | 0 |
| 2059 | Borealin | IPI00303099 | 0.000 | 0.32285 | 0 | 0 | 0 | 0 |
| 2060 | RNA polymerase-associated protein RTF1 homolog | IPI00303832 | 0.000 | 0.32285 | 0 | 0 | 2 | 0 |
| 2061 | Isoform 1 of 182 kDa tankyrase-1-binding protein | IPI00304589 | 0.000 | 0.32285 | 0 | 0 | 0 | 0 |
| 2062 | Isoform 1 of Metal transporter CNNM3 | IPI00168565 | 0.000 | 0.32285 | 0 | 2 | 0 | 0 |
| 2063 | Renin receptor | IPI00168884 | 0.000 | 0.32285 | 0 | 0 | 0 | 0 |
| 2064 | Isoform 3 of Mediator of RNA polymerase II transcription subunit 23 | IPI00413272 | 0.000 | 0.32285 | 0 | 0 | 0 | 0 |
| 2065 | DNA replication complex GINS protein PSF1 | IPI00032387 | 0.000 | 0.32285 | 2 | 0 | 0 | 0 |
| 2066 | Cystatin-C | IPI00032293 | 0.000 | 0.32285 | 0 | 0 | 2 | 0 |
| 2067 | Serine/threonine-protein kinase 10 | IPI00304742 | 0.000 | 0.32285 | 0 | 0 | 0 | 0 |
| 2068 | Uncharacterized protein C9orf114 | IPI00844014 | 0.000 | 0.32285 | 0 | 0 | 0 | 0 |
| 2069 | Transcription elongation factor B polypeptide 1 | IPI00300341 | 0.000 | 0.32285 | 0 | 0 | 2 | 0 |
| 2070 | Ras-related protein Rap-2b | IPI00018364 | 0.000 | 0.32285 | 0 | 0 | 2 | 0 |
| 2071 | Endonuclease/exonuclease/phosphatase family domain-containing protein 1 | IPI00885036 | 0.000 | 0.32285 | 0 | 0 | 0 | 0 |
| 2072 | Transmembrane emp24 domain-containing protein 1 | IPI00009976 | 0.000 | 0.32285 | 0 | 0 | 0 | 2 |
| 2073 | Putative GTP-binding protein RAY-like variant (Fragment) | IPI00411514 | 0.000 | 0.32285 | 0 | 2 | 0 | 0 |
| 2074 | Myristoylated alanine-rich C-kinase substrate | IPI00219301 | 0.000 | 0.32285 | 0 | 0 | 2 | 0 |
| 2075 | NudC domain-containing protein 3 | IPI00238209 | 0.000 | 0.32285 | 0 | 0 | 0 | 0 |
| 2076 | Ubiquilin-2 | IPI00409659 | 0.000 | 0.32285 | 0 | 0 | 0 | 2 |
| 2077 | cDNA FLJ61658, highly similar to Transmembrane 9 superfamily protein member 1 | IPI00101374 | 0.000 | 0.32285 | 0 | 2 | 0 | 0 |
| 2078 | Isoform 2 of Oxidoreductase HTATIP2 | IPI00383665 | 0.000 | 0.32285 | 0 | 0 | 0 | 0 |
| 2079 | Isoform 1 of Beta-enolase | IPI00218474 | 0.000 | 0.32285 | 0 | 0 | 0 | 2 |

| No. | Description | Accession number | STN[1] | p-Value[1] | Con_A[2] | Con_B[2] | RG3_A[2] | RG3_B[2] |
|---|---|---|---|---|---|---|---|---|
| 2080 | Isoform 1 of Alpha-globin transcription factor CP2 | IPI00037599 | 0.000 | 0.32285 | 0 | 0 | 2 | 0 |
| 2081 | Origin recognition complex subunit 6 | IPI00001641 | 0.000 | 0.32285 | 0 | 2 | 0 | 0 |
| 2082 | Isoform 1 of Pre-mRNA-splicing factor 38B | IPI00018098 | 0.000 | 0.32285 | 0 | 2 | 0 | 0 |
| 2083 | Isoform 1 of Tumor protein D52 | IPI00619958 | 0.000 | 0.32285 | 0 | 2 | 0 | 0 |
| 2084 | Aldose 1-epimerase | IPI00060200 | 0.000 | 0.32285 | 0 | 0 | 2 | 0 |
| 2085 | SCY1-like protein 2 | IPI00396218 | 0.000 | 0.32285 | 0 | 0 | 0 | 0 |
| 2086 | Isoform 1 of Adaptin ear-binding coat-associated protein 2 | IPI00018188 | 0.000 | 0.32285 | 0 | 0 | 0 | 2 |
| 2087 | Isoform 4 of Mitochondrial fission factor | IPI00024627 | 0.000 | 0.32285 | 0 | 0 | 2 | 0 |
| 2088 | cAMP-dependent protein kinase type I-beta regulatory subunit | IPI00554488 | 0.000 | 0.32285 | 0 | 0 | 0 | 2 |
| 2089 | Synaptojanin-2-binding protein | IPI00299193 | 0.000 | 0.32285 | 0 | 0 | 0 | 0 |
| 2090 | Isoform 1 of Kinesin-like protein KIF15 | IPI00024975 | 0.000 | 0.32285 | 0 | 0 | 0 | 0 |
| 2091 | serine/threonine-protein phosphatase 2B catalytic subunit beta isoform isoform a | IPI00027809 | 0.000 | 0.32285 | 0 | 0 | 0 | 0 |
| 2092 | My002 protein | IPI00023584 | 0.000 | 0.32285 | 0 | 0 | 2 | 2 |
| 2093 | Isoform 3 of Protein LAS1 homolog | IPI00009917 | 0.000 | 0.32285 | 0 | 2 | 0 | 0 |
| 2094 | Alpha-taxilin | IPI00470779 | 0.000 | 0.32285 | 2 | 0 | 0 | 0 |
| 2095 | Isoform 1 of Transmembrane protein 163 | IPI00152253 | 0.000 | 0.32285 | 2 | 0 | 2 | 0 |
| 2096 | Isoform 1 of GPI transamidase component PIG-T | IPI00100030 | 0.000 | 0.32285 | 0 | 2 | 0 | 0 |
| 2097 | Isoform 1 of Serine/threonine-protein kinase N1 | IPI00002803 | 0.000 | 0.32285 | 0 | 0 | 0 | 0 |
| 2098 | Digestive organ expansion factor homolog | IPI00004290 | 0.000 | 0.32285 | 0 | 2 | 0 | 0 |
| 2099 | Protein LTV1 homolog | IPI00153032 | 0.000 | 0.32285 | 2 | 0 | 0 | 0 |
| 2100 | Isoform 1 of Heterogeneous nuclear ribonucleoprotein L-like | IPI00103247 | 0.000 | 0.32285 | 0 | 0 | 2 | 0 |
| 2101 | USP48 protein | IPI00328815 | 0.000 | 0.32285 | 0 | 0 | 2 | 0 |
| 2102 | Serine/threonine-protein kinase Nek9 | IPI00301609 | 0.000 | 0.32285 | 0 | 0 | 2 | 0 |
| 2103 | Protein FAM114A2 | IPI00329662 | 0.000 | 0.32285 | 0 | 2 | 0 | 0 |
| 2104 | Patatin-like phospholipase domain-containing protein 4 | IPI00013218 | 0.000 | 0.32285 | 0 | 3 | 0 | 3 |
| 2105 | Isoform 1 of Caspase-8 | IPI00000149 | 0.000 | 0.32285 | 0 | 2 | 0 | 0 |
| 2106 | Uridine diphosphate glucose pyrophosphatase | IPI00412878 | 0.000 | 0.32285 | 0 | 2 | 0 | 0 |
| 2107 | RRP15-like protein | IPI00007004 | 0.000 | 0.32285 | 0 | 0 | 0 | 0 |
| 2108 | Isoform 1 of UPF0489 protein C5orf22 | IPI00019966 | 0.000 | 0.32285 | 0 | 2 | 0 | 0 |
| 2109 | 39S ribosomal protein L54, mitochondrial | IPI00332157 | 0.000 | 0.32285 | 0 | 0 | 0 | 2 |
| 2110 | Isoform 1 of Lariat debranching enzyme | IPI00305545 | 0.000 | 0.32285 | 2 | 0 | 0 | 0 |
| 2111 | Isoform 4 of Calcium/calmodulin-dependent protein kinase type II subunit gamma | IPI00172450 | 0.000 | 0.32285 | 0 | 0 | 0 | 0 |
| 2112 | Isoform 2 of Rap guanine nucleotide exchange factor 6 | IPI00291839 | 0.000 | 0.32285 | 0 | 0 | 0 | 2 |
| 2113 | Protein DPCD | IPI00063962 | 0.000 | 0.32285 | 2 | 0 | 0 | 0 |
| 2114 | Isoform 1 of Transmembrane protein 192 | IPI00855873 | 0.000 | 0.32285 | 0 | 2 | 0 | 0 |
| 2115 | Queuine tRNA-ribosyltransferase | IPI00215974 | 0.000 | 0.32285 | 0 | 0 | 0 | 0 |
| 2116 | Gamma-soluble NSF attachment protein | IPI00293817 | 0.000 | 0.32285 | 0 | 0 | 0 | 2 |
| 2117 | cDNA FLJ35172 fis, clone PLACE6013232 | IPI00385785 | 0.000 | 0.32285 | 0 | 0 | 2 | 0 |
| 2118 | MIT domain-containing protein 1 | IPI00103065 | 0.000 | 0.32285 | 0 | 2 | 0 | 0 |
| 2119 | Pre-mRNA-splicing factor SYF2 | IPI00022963 | 0.000 | 0.32285 | 0 | 0 | 0 | 0 |
| 2120 | GPN-loop GTPase 1 isoform a | IPI00027035 | 0.000 | 0.32285 | 0 | 0 | 0 | 0 |
| 2121 | Pyruvate dehydrogenase phosphatase regulatory subunit, mitochondrial | IPI00168407 | 0.000 | 0.32285 | 0 | 0 | 0 | 0 |
| 2122 | Isoform 2 of Transducin-like enhancer protein 3 | IPI00177938 | 0.000 | 0.32285 | 0 | 0 | 0 | 0 |
| 2123 | Arfaptin-2 | IPI00021257 | 0.000 | 0.32285 | 0 | 0 | 0 | 0 |
| 2124 | Isoform 1 of Serine/threonine-protein phosphatase 6 regulatory ankyrin repeat subunit A | IPI00477505 | 0.000 | 0.32285 | 0 | 0 | 0 | 0 |
| 2125 | cDNA FLJ56176, highly similar to Poly(A) polymerase alpha | IPI00384028 | 0.000 | 0.32285 | 0 | 0 | 0 | 2 |
| 2126 | F-box only protein 7 | IPI00294567 | 0.000 | 0.32285 | 2 | 0 | 0 | 0 |
| 2127 | UPF0480 protein C15orf24 | IPI00024551 | 0.000 | 0.32285 | 2 | 0 | 0 | 0 |
| 2128 | Isoform 3 of Epithelial splicing regulatory protein 1 | IPI00184262 | 0.000 | 0.32285 | 0 | 2 | 2 | 0 |
| 2129 | NEDD8 ultimate buster 1 | IPI00157365 | 0.000 | 0.32285 | 0 | 0 | 0 | 0 |
| 2130 | Putative uncharacterized protein QTRTD1 | IPI00074010 | 0.000 | 0.32285 | 0 | 0 | 0 | 2 |
| 2131 | Isoform 1 of Peptidyl-prolyl cis-trans isomerase SDCCAG10 | IPI00025174 | 0.000 | 0.32285 | 0 | 0 | 0 | 2 |
| 2132 | Isoform 1 of Liprin-alpha-1 | IPI00163496 | 0.000 | 0.32285 | 0 | 2 | 0 | 0 |
| 2133 | Isoform GN-1L of Glycogenin-1 | IPI00180386 | 0.000 | 0.32285 | 0 | 0 | 2 | 0 |
| 2134 | Iron-sulfur cluster assembly 2 homolog, mitochondrial | IPI00376195 | 0.000 | 0.32285 | 0 | 0 | 0 | 2 |
| 2135 | 1-acyl-sn-glycerol-3-phosphate acyltransferase alpha | IPI00019141 | 0.000 | 0.32285 | 0 | 2 | 0 | 0 |
| 2136 | TIM21-like protein, mitochondrial | IPI00306439 | 0.000 | 0.32285 | 0 | 2 | 0 | 0 |
| 2137 | Isoform 1 of RNA-binding protein 34 | IPI00181617 | 0.000 | 0.32285 | 0 | 2 | 0 | 0 |
| 2138 | Isoform 2 of Bifunctional arginine demethylase and lysyl-hydroxylase JMJD6 | IPI00375496 | 0.000 | 0.32285 | 2 | 0 | 0 | 0 |
| 2139 | Protein kinase C and casein kinase substrate in neurons 3, isoform CRA_b | IPI00329572 | 0.000 | 0.32285 | 0 | 0 | 0 | 0 |
| 2140 | Dimethyladenosine transferase 2, mitochondrial | IPI00034069 | 0.000 | 0.32285 | 0 | 2 | 0 | 0 |
| 2141 | Isoform 3 of Protein DDI1 homolog 2 | IPI00031618 | 0.000 | 0.32285 | 0 | 2 | 0 | 0 |
| 2142 | Serpin B9 | IPI00032139 | 0.000 | 0.32285 | 0 | 2 | 0 | 0 |
| 2143 | Isoform 2 of LisH domain and HEAT repeat-containing protein KIAA1468 | IPI00023330 | 0.000 | 0.32285 | 0 | 0 | 0 | 0 |
| 2144 | Torsin A interacting protein 1 | IPI00644766 | 0.000 | 0.32285 | 0 | 2 | 0 | 0 |
| 2145 | Isoform 1 of Vesicle-associated membrane protein 7 | IPI00020887 | 0.000 | 0.32285 | 0 | 2 | 0 | 0 |
| 2146 | Isoform 1 of Signal recognition particle 68 kDa protein | IPI00168388 | 0.000 | 0.32285 | 2 | 0 | 2 | 0 |
| 2147 | Isoform 1 of Putative methyltransferase METT10D | IPI00163391 | 0.000 | 0.32285 | 0 | 0 | 0 | 2 |
| 2148 | Isoform 1 of Regulation of nuclear pre-mRNA domain-containing protein 2 | IPI00384541 | 0.000 | 0.32285 | 0 | 0 | 0 | 0 |
| 2149 | Isoform Alpha of Paxillin | IPI00220030 | 0.000 | 0.32285 | 0 | 0 | 2 | 0 |
| 2150 | Putative uncharacterized protein WDR43 | IPI00892938 | 0.000 | 0.32285 | 0 | 0 | 0 | 0 |
| 2151 | Isoform 1 of tRNA 2'-phosphotransferase 1 | IPI00328580 | 0.000 | 0.32285 | 0 | 2 | 0 | 0 |
| 2152 | Isoform 1 of Chaperone activity of bc1 complex-like, mitochondrial | IPI00176469 | 0.000 | 0.32285 | 0 | 0 | 0 | 2 |
| 2153 | Isoform 1 of Prostaglandin reductase 2 | IPI00167515 | 0.000 | 0.32285 | 0 | 0 | 0 | 2 |
| 2154 | Isoform 1 of Ribulose-phosphate 3-epimerase | IPI00335280 | 0.000 | 0.32285 | 0 | 0 | 0 | 2 |
| 2155 | Angio-associated migratory cell protein | IPI00014481 | 0.000 | 0.32285 | 0 | 0 | 0 | 0 |
| 2156 | Isoform 1 of Eukaryotic initiation factor 4A-II | IPI00328328 | 0.000 | 0.32285 | 0 | 0 | 0 | 2 |
| 2157 | Protein disulfide-isomerase A5 | IPI00031479 | 0.000 | 0.32285 | 0 | 0 | 0 | 0 |
| 2158 | Inactive hydroxysteroid dehydrogenase-like protein 1 | IPI00171459 | 0.000 | 0.32285 | 0 | 0 | 0 | 2 |
| 2159 | CD2 antigen cytoplasmic tail-binding protein 2 | IPI00006103 | 0.000 | 0.32285 | 0 | 0 | 0 | 0 |
| 2160 | Bifunctional polynucleotide phosphatase/kinase | IPI00290684 | 0.000 | 0.32285 | 0 | 2 | 0 | 0 |
| 2161 | Probable arginyl-tRNA synthetase, mitochondrial | IPI00549566 | 0.000 | 0.32285 | 0 | 2 | 0 | 0 |
| 2162 | Isoform 1 of CTD small phosphatase-like protein 2 | IPI00033054 | 0.000 | 0.32285 | 0 | 2 | 0 | 0 |
| 2163 | General transcription factor IIF subunit 1 | IPI00017450 | 0.000 | 0.32285 | 2 | 0 | 0 | 0 |
| 2164 | Isoform 2 of Uncharacterized protein C3orf63 | IPI00745978 | 0.000 | 0.32285 | 0 | 0 | 0 | 2 |
| 2165 | Isoform 1 of Casein kinase I isoform delta | IPI00011102 | 0.000 | 0.32285 | 2 | 0 | 0 | 0 |
| 2166 | Leucine zipper transcription factor-like protein 1 | IPI00299465 | 0.000 | 0.32285 | 0 | 0 | 0 | 2 |
| 2167 | Polypeptide N-acetylgalactosaminyltransferase 2 | IPI00004669 | 0.000 | 0.32285 | 0 | 2 | 0 | 0 |
| 2168 | Low-density lipoprotein receptor | IPI00000070 | 0.000 | 0.32285 | 2 | 0 | 0 | 0 |
| 2169 | 25 kDa protein | IPI00010276 | 0.000 | 0.32285 | 0 | 0 | 0 | 0 |
| 2170 | cDNA FLJ56443, highly similar to Putative ATP-dependent RNA helicase DHX33 | IPI00302860 | 0.000 | 0.32285 | 0 | 0 | 0 | 0 |
| 2171 | Isoform 1 of Elongator complex protein 2 | IPI00015560 | 0.000 | 0.32285 | 0 | 0 | 0 | 0 |
| 2172 | STAM-binding protein | IPI00007943 | 0.000 | 0.32285 | 0 | 0 | 0 | 2 |
| 2173 | Isoform 2 of Caspase-2 | IPI00216410 | 0.000 | 0.32285 | 0 | 0 | 0 | 0 |
| 2174 | Isoform 2 of SAP30-binding protein | IPI00333699 | 0.000 | 0.32285 | 0 | 0 | 0 | 0 |

| No. | Description | Accession number | STN[1] | p-Value[1] | Con_A[2] | Con_B[2] | RG3_A[2] | RG3_B[2] |
|---|---|---|---|---|---|---|---|---|
| 2175 | Guanine nucleotide-binding protein subunit alpha-12 | IPI00328744 | 0.000 | 0.32285 | 0 | 0 | 0 | 0 |
| 2176 | cDNA FLJ61478, highly similar to Succinate dehydrogenase (ubiquinone) flavoprotein subunit | IPI00305166 | 0.000 | 0.32285 | 2 | 0 | 0 | 0 |
| 2177 | Isoform 2 of Ubiquitin-associated protein 2-like | IPI00029019 | 0.000 | 0.32285 | 0 | 0 | 0 | 2 |
| 2178 | Isoform 3 of Target of rapamycin complex subunit LST8 | IPI00007182 | 0.000 | 0.32285 | 0 | 0 | 2 | 0 |
| 2179 | Thiamine-triphosphatase | IPI00013621 | 0.000 | 0.32285 | 0 | 0 | 0 | 0 |
| 2180 | Isoform 3 of Centromere protein V | IPI00376481 | 0.000 | 0.32285 | 0 | 2 | 0 | 0 |
| 2181 | EF-hand domain-containing family member A1 | IPI00640276 | 0.000 | 0.32285 | 0 | 2 | 0 | 0 |
| 2182 | Golgin subfamily A member 1 | IPI00031115 | 0.000 | 0.32285 | 0 | 0 | 0 | 2 |
| 2183 | Isoform 1 of U5 small nuclear ribonucleoprotein 200 kDa helicase | IPI00420014 | -0.042 | 0.30412 | 157 | 144 | 121 | 179 |
| 2184 | 40S ribosomal protein S7 | IPI00013415 | -0.066 | 0.30253 | 38 | 37 | 44 | 30 |
| 2185 | Peptidyl-prolyl cis-trans isomerase B | IPI00646304 | -0.066 | 0.30240 | 47 | 26 | 44 | 28 |
| 2186 | Isoform 1 of 60S ribosomal protein L12 | IPI00024933 | -0.069 | 0.30177 | 31 | 32 | 29 | 33 |
| 2187 | Microtubule-associated protein RP/EB family member 1 | IPI00017596 | -0.075 | 0.30064 | 31 | 19 | 32 | 17 |
| 2188 | 40S ribosomal protein S8 | IPI00216587 | -0.077 | 0.30035 | 23 | 22 | 21 | 23 |
| 2189 | Isoform 1 of Catenin beta-1 | IPI00017292 | -0.079 | 0.30027 | 24 | 18 | 27 | 14 |
| 2190 | Proteasome subunit alpha type-5 | IPI00291922 | -0.080 | 0.30002 | 23 | 17 | 22 | 17 |
| 2191 | Barrier-to-autointegration factor | IPI00026087 | -0.081 | 0.29964 | 22 | 17 | 23 | 15 |
| 2192 | V-type proton ATPase subunit B, brain isoform | IPI00007812 | -0.082 | 0.29947 | 16 | 22 | 16 | 21 |
| 2193 | Isoform 1 of Heterogeneous nuclear ribonucleoprotein D-like | IPI00011274 | -0.083 | 0.29926 | 15 | 21 | 19 | 16 |
| 2194 | Isoform 1 of Importin-4 | IPI00156374 | -0.083 | 0.29926 | 20 | 16 | 19 | 16 |
| 2195 | Sodium/potassium-transporting ATPase subunit beta-3 | IPI00008167 | -0.085 | 0.29910 | 14 | 19 | 15 | 17 |
| 2196 | Ribosomal L1 domain-containing protein 1 | IPI00008708 | -0.087 | 0.29826 | 20 | 11 | 14 | 16 |
| 2197 | 26S proteasome non-ATPase regulatory subunit 14 | IPI00024821 | -0.089 | 0.29742 | 11 | 18 | 12 | 16 |
| 2198 | Isoform 1 of RNA-binding protein 39 | IPI00163505 | -0.090 | 0.29713 | 15 | 13 | 13 | 14 |
| 2199 | Isoform 1 of Transportin-1 | IPI00024364 | -0.092 | 0.29655 | 14 | 12 | 10 | 15 |
| 2200 | Nuclear migration protein nudC | IPI00550746 | -0.093 | 0.29604 | 14 | 11 | 12 | 12 |
| 2201 | Eukaryotic translation initiation factor 3 subunit K | IPI00033143 | -0.093 | 0.29604 | 13 | 12 | 13 | 11 |
| 2202 | Isoform 1 of Adipocyte plasma membrane-associated protein | IPI00031131 | -0.095 | 0.29575 | 12 | 12 | 11 | 12 |
| 2203 | Sorbitol dehydrogenase | IPI00216057 | -0.095 | 0.29575 | 13 | 11 | 11 | 12 |
| 2204 | Actin-related protein 2/3 complex subunit 2 | IPI00005161 | -0.095 | 0.29575 | 11 | 13 | 8 | 15 |
| 2205 | protein ALO17 isoform 1 | IPI00828098 | -0.096 | 0.29512 | 10 | 13 | 5 | 17 |
| 2206 | Proline synthetase co-transcribed homolog (Bacterial), isoform CRA_b | IPI00016346 | -0.096 | 0.29512 | 9 | 14 | 9 | 13 |
| 2207 | Serpin B6 | IPI00413451 | -0.096 | 0.29512 | 11 | 12 | 11 | 11 |
| 2208 | Hsc70-interacting protein | IPI00032826 | -0.096 | 0.29512 | 13 | 10 | 13 | 9 |
| 2209 | Isoform 1 of Clathrin heavy chain 2 | IPI00022881 | -0.097 | 0.29458 | 96 | 98 | 90 | 102 |
| 2210 | Isoform 1 of RRP12-like protein | IPI00101186 | -0.099 | 0.29358 | 10 | 11 | 8 | 12 |
| 2211 | Nuclease-sensitive element-binding protein 1 | IPI00031812 | -0.099 | 0.29358 | 13 | 8 | 14 | 6 |
| 2212 | Condensin complex subunit 3 | IPI00106495 | -0.099 | 0.29358 | 10 | 11 | 11 | 9 |
| 2213 | Seryl-tRNA synthetase, cytoplasmic | IPI00220637 | -0.099 | 0.29358 | 9 | 12 | 6 | 14 |
| 2214 | Isoform 2 of Cat eye syndrome critical region protein 5 | IPI00011511 | -0.099 | 0.29358 | 9 | 12 | 11 | 9 |
| 2215 | Isoform 1 of OCIA domain-containing protein 2 | IPI00555902 | -0.100 | 0.29299 | 18 | 0 | 17 | 0 |
| 2216 | Isoform C1 of Heterogeneous nuclear ribonucleoproteins C1/C2 | IPI00216592 | -0.102 | 0.29211 | 82 | 86 | 86 | 80 |
| 2217 | cDNA FLJ51909, highly similar to Serine-threonine kinase receptor-associatedprotein | IPI00294536 | -0.102 | 0.29211 | 10 | 9 | 12 | 6 |
| 2218 | tropomyosin alpha-1 chain isoform 2 | IPI00000230 | -0.102 | 0.29211 | 12 | 7 | 12 | 6 |
| 2219 | Replication factor C subunit 4 | IPI00017381 | -0.102 | 0.29211 | 9 | 10 | 9 | 9 |
| 2220 | Isoform 1 of Splicing factor, arginine/serine-rich 7 | IPI00003377 | -0.104 | 0.29157 | 11 | 7 | 9 | 8 |
| 2221 | Eukaryotic translation initiation factor 3 subunit D | IPI00006181 | -0.104 | 0.29157 | 10 | 8 | 8 | 9 |
| 2222 | UPF0368 protein Cxorf26 | IPI00107104 | -0.104 | 0.29157 | 8 | 10 | 7 | 10 |
| 2223 | 60S ribosomal protein L26-like 1 | IPI00007144 | -0.106 | 0.28969 | 9 | 8 | 9 | 7 |
| 2224 | Isoform 1 of Vesicle-associated membrane protein-associated protein B/C | IPI00006211 | -0.106 | 0.28969 | 8 | 9 | 9 | 7 |
| 2225 | Isoform 1 of Vesicle-associated membrane protein-associated protein A | IPI00170692 | -0.106 | 0.28969 | 10 | 7 | 9 | 7 |
| 2226 | Isoform 2 of NSFL1 cofactor p47 | IPI00022830 | -0.106 | 0.28969 | 9 | 8 | 9 | 7 |
| 2227 | Isoform 1 of Spermatid perinuclear RNA-binding protein | IPI00169430 | -0.106 | 0.28969 | 9 | 8 | 9 | 7 |
| 2228 | 60S ribosomal protein L18a | IPI00026202 | -0.108 | 0.28852 | 9 | 7 | 10 | 5 |
| 2229 | Isoform 1 of RNA-binding protein 8A | IPI00001757 | -0.108 | 0.28852 | 8 | 8 | 8 | 7 |
| 2230 | Calponin-2 | IPI00015262 | -0.108 | 0.28852 | 9 | 7 | 8 | 7 |
| 2231 | Interferon-induced, double-stranded RNA-activated protein kinase | IPI00019463 | -0.108 | 0.28852 | 8 | 8 | 8 | 7 |
| 2232 | Isoform 2 of Transportin-3 | IPI00395694 | -0.110 | 0.28797 | 6 | 9 | 6 | 8 |
| 2233 | NADH dehydrogenase [ubiquinone] 1 alpha subcomplex subunit 10, mitochondrial | IPI00029561 | -0.110 | 0.28797 | 0 | 13 | 5 | 9 |
| 2234 | Bifunctional methylenetetrahydrofolate dehydrogenase/cyclohydrolase, mitochondrial | IPI00011307 | -0.113 | 0.28743 | 5 | 9 | 5 | 8 |
| 2235 | tRNA methyltransferase 112 homolog | IPI00009010 | -0.113 | 0.28743 | 7 | 7 | 7 | 6 |
| 2236 | Isoform 1 of Transmembrane and coiled-coil domain-containing protein 1 | IPI00026111 | -0.116 | 0.28563 | 7 | 6 | 6 | 6 |
| 2237 | Isoform 1 of Probable threonyl-tRNA synthetase 2, cytoplasmic | IPI00328082 | -0.116 | 0.28563 | 4 | 9 | 4 | 8 |
| 2238 | Ribonuclease P protein subunit p30 | IPI00019196 | -0.116 | 0.28563 | 5 | 8 | 5 | 7 |
| 2239 | Isoform 3 of PCI domain-containing protein 2 | IPI00072541 | -0.116 | 0.28563 | 6 | 7 | 5 | 7 |
| 2240 | Isoform 5 of Thioredoxin reductase 1, cytoplasmic | IPI00554786 | -0.116 | 0.28563 | 4 | 9 | 6 | 6 |
| 2241 | Isoform 1 of Insulin-like growth factor 2 mRNA-binding protein 2 | IPI00179713 | -0.116 | 0.28563 | 7 | 6 | 6 | 6 |
| 2242 | Isoform Alpha-6X1X2B of Integrin alpha-6 | IPI00010697 | -0.116 | 0.28563 | 5 | 8 | 5 | 7 |
| 2243 | Calcium-binding protein 39-like | IPI00026359 | -0.116 | 0.28563 | 5 | 8 | 7 | 5 |
| 2244 | Isoform SERCA2A of Sarcoplasmic/endoplasmic reticulum calcium ATPase 2 | IPI00177817 | -0.119 | 0.28408 | 8 | 4 | 4 | 7 |
| 2245 | Eukaryotic translation initiation factor 3 subunit G | IPI00290460 | -0.119 | 0.28408 | 7 | 5 | 6 | 5 |
| 2246 | Malate dehydrogenase | IPI00916111 | -0.119 | 0.28408 | 8 | 4 | 7 | 4 |
| 2247 | DNA-directed RNA polymerase II subunit RPB3 | IPI00018288 | -0.119 | 0.28408 | 6 | 6 | 5 | 6 |
| 2248 | Isoform 1 of CUGBP Elav-like family member 1 | IPI00034015 | -0.119 | 0.28408 | 8 | 4 | 7 | 4 |
| 2249 | Twinfilin-2 | IPI00550917 | -0.119 | 0.28408 | 6 | 6 | 5 | 6 |
| 2250 | Isoform 2 of Ubiquinol-cytochrome c reductase complex chaperone CBP3 homolog | IPI00219889 | -0.119 | 0.28408 | 5 | 7 | 5 | 6 |
| 2251 | Isoform 2 of Lysine-specific histone demethylase 1A | IPI00217540 | -0.119 | 0.28408 | 6 | 6 | 3 | 8 |
| 2252 | Hsp90 co-chaperone Cdc37 | IPI00013122 | -0.119 | 0.28408 | 7 | 5 | 5 | 6 |
| 2253 | Neurolysin, mitochondrial | IPI00010346 | -0.122 | 0.28137 | 4 | 7 | 3 | 7 |
| 2254 | 24 kDa protein | IPI00397611 | -0.122 | 0.28137 | 6 | 5 | 6 | 4 |
| 2255 | N-alpha-acetyltransferase 38, NatC auxiliary subunit | IPI00219871 | -0.122 | 0.28137 | 5 | 6 | 5 | 5 |
| 2256 | CDGSH iron sulfur domain-containing protein 1 | IPI00020510 | -0.122 | 0.28137 | 5 | 6 | 4 | 6 |
| 2257 | Isoform 2 of cAMP-dependent protein kinase catalytic subunit alpha | IPI00217960 | -0.122 | 0.28137 | 6 | 5 | 5 | 5 |
| 2258 | Splicing factor, arginine/serine-rich 3 | IPI00010204 | -0.122 | 0.28137 | 6 | 5 | 6 | 4 |
| 2259 | Probable ATP-dependent RNA helicase DDX27 | IPI00293078 | -0.122 | 0.28137 | 4 | 7 | 4 | 6 |
| 2260 | Putative uncharacterized protein DKFZp451D234 | IPI00031583 | -0.126 | 0.27898 | 4 | 6 | 4 | 5 |
| 2261 | Diphosphoinositol polyphosphate phosphohydrolase 1 | IPI00009148 | -0.126 | 0.27898 | 6 | 4 | 2 | 7 |
| 2262 | myosin-IXb isoform 1 | IPI00306933 | -0.126 | 0.27898 | 5 | 5 | 4 | 5 |
| 2263 | Isoform 1 of Transcription elongation factor SPT6 | IPI00784161 | -0.126 | 0.27898 | 6 | 4 | 4 | 5 |
| 2264 | Peroxisomal membrane protein 2 | IPI00221002 | -0.126 | 0.27898 | 6 | 4 | 4 | 5 |
| 2265 | SWI/SNF related, matrix associated, actin dependent regulator of chromatin | IPI00216046 | -0.126 | 0.27898 | 5 | 5 | 5 | 4 |
| 2266 | Coiled-coil domain-containing protein 124 | IPI00060627 | -0.126 | 0.27898 | 4 | 6 | 4 | 5 |
| 2267 | Putative uncharacterized protein EIF4E2 | IPI00556081 | -0.126 | 0.27898 | 5 | 5 | 4 | 5 |
| 2268 | Isoform 1 of UBX domain-containing protein 1 | IPI00027378 | -0.126 | 0.27898 | 4 | 6 | 5 | 4 |
| 2269 | Isoform 2 of Fumarylacetoacetate hydrolase domain-containing protein 1 | IPI00440828 | -0.126 | 0.27898 | 4 | 6 | 4 | 5 |

| No. | Description | Accession number | STN[1] | p-Value[1] | Con_A[2] | Con_B[2] | RG3_A[2] | RG3_B[2] |
|---|---|---|---|---|---|---|---|---|
| 2270 | Phosducin-like protein 3 | IPI00031629 | -0.126 | 0.27898 | 3 | 7 | 4 | 5 |
| 2271 | DNA polymerase delta subunit 2 | IPI00025616 | -0.126 | 0.27898 | 4 | 6 | 4 | 5 |
| 2272 | Isoform ASF-1 of Splicing factor, arginine/serine-rich 1 | IPI00215884 | -0.127 | 0.27685 | 47 | 38 | 42 | 41 |
| 2273 | Lamin-B1 | IPI00217975 | -0.130 | 0.27672 | 44 | 34 | 38 | 38 |
| 2274 | Isoform 1 of COP9 signalosome complex subunit 7b | IPI00009301 | -0.131 | 0.27664 | 6 | 3 | 4 | 4 |
| 2275 | Serine/threonine-protein phosphatase 2A 56 kDa regulatory subunit epsilon isoform | IPI00002853 | -0.131 | 0.27664 | 5 | 4 | 3 | 5 |
| 2276 | COP9 signalosome complex subunit 6 | IPI00163230 | -0.131 | 0.27664 | 4 | 5 | 3 | 5 |
| 2277 | Isoform 1 of KDEL motif-containing protein 2 | IPI00143921 | -0.131 | 0.27664 | 4 | 5 | 3 | 5 |
| 2278 | Serine/threonine-protein phosphatase 2A 65 kDa regulatory subunit A alpha isoform | IPI00554737 | -0.131 | 0.27664 | 4 | 5 | 3 | 5 |
| 2279 | Serine/threonine-protein phosphatase 2A 55 kDa regulatory subunit B alpha isoform | IPI00332511 | -0.131 | 0.27664 | 3 | 6 | 4 | 4 |
| 2280 | Basic leucine zipper and W2 domain-containing protein 2 | IPI00022305 | -0.131 | 0.27664 | 5 | 4 | 4 | 4 |
| 2281 | Probable rRNA-processing protein EBP2 | IPI00745955 | -0.131 | 0.27664 | 5 | 4 | 5 | 3 |
| 2282 | Ras-related protein Ral-A | IPI00217519 | -0.131 | 0.27664 | 4 | 5 | 3 | 5 |
| 2283 | Isoform 3 of Drebrin-like protein | IPI00101968 | -0.131 | 0.27664 | 2 | 7 | 4 | 4 |
| 2284 | UPF0760 protein C2orf29 | IPI00014194 | -0.131 | 0.27664 | 5 | 4 | 3 | 5 |
| 2285 | REST corepressor 1 | IPI00008531 | -0.131 | 0.27664 | 4 | 5 | 4 | 4 |
| 2286 | Calcium-regulated heat stable protein 1 | IPI00304409 | -0.136 | 0.27396 | 4 | 4 | 4 | 3 |
| 2287 | 28S ribosomal protein S22, mitochondrial | IPI00013146 | -0.136 | 0.27396 | 5 | 3 | 5 | 2 |
| 2288 | Monocarboxylate transporter 4 | IPI00006666 | -0.136 | 0.27396 | 6 | 2 | 2 | 5 |
| 2289 | TRIP12 protein | IPI00032342 | -0.136 | 0.27396 | 5 | 3 | 3 | 4 |
| 2290 | Isoform Long of Cold shock domain-containing protein E1 | IPI00470891 | -0.136 | 0.27396 | 5 | 3 | 5 | 2 |
| 2291 | Isoform 1 of Kinectin | IPI00328753 | -0.136 | 0.27396 | 5 | 3 | 4 | 3 |
| 2292 | Isoform 1 of Origin recognition complex subunit 3 | IPI00294402 | -0.136 | 0.27396 | 3 | 5 | 3 | 4 |
| 2293 | NADH dehydrogenase [ubiquinone] flavoprotein 2, mitochondrial | IPI00291328 | -0.136 | 0.27396 | 2 | 6 | 3 | 4 |
| 2294 | Survival of motor neuron-related-splicing factor 30 | IPI00025176 | -0.136 | 0.27396 | 3 | 5 | 3 | 4 |
| 2295 | UDP-galactose-4-epimerase | IPI00030229 | -0.136 | 0.27396 | 4 | 4 | 4 | 3 |
| 2296 | Uncharacterized protein C10orf58 | IPI00296190 | -0.136 | 0.27396 | 0 | 6 | 0 | 5 |
| 2297 | Isoform 1 of Deoxyuridine 5'-triphosphate nucleotidohydrolase, mitochondrial | IPI00013679 | -0.138 | 0.26882 | 34 | 31 | 32 | 31 |
| 2298 | Helicase SKI2W | IPI00414819 | -0.142 | 0.26823 | 4 | 3 | 4 | 0 |
| 2299 | mortality factor 4 | IPI00001955 | -0.142 | 0.26823 | 3 | 4 | 3 | 3 |
| 2300 | Charged multivesicular body protein 7 | IPI00395463 | -0.142 | 0.26823 | 0 | 5 | 2 | 4 |
| 2301 | 28S ribosomal protein S23, mitochondrial | IPI00032881 | -0.142 | 0.26823 | 0 | 5 | 2 | 4 |
| 2302 | Isoform 2C of Cytoplasmic dynein 1 intermediate chain 2 | IPI00216348 | -0.142 | 0.26823 | 5 | 0 | 2 | 4 |
| 2303 | Adenylyl cyclase-associated protein | IPI00939159 | -0.142 | 0.26823 | 4 | 3 | 2 | 4 |
| 2304 | Ribosomal protein S6 kinase alpha-1 | IPI00017305 | -0.142 | 0.26823 | 0 | 5 | 0 | 4 |
| 2305 | Isoform 2 of U4/U6 small nuclear ribonucleoprotein Prp31 | IPI00167198 | -0.142 | 0.26823 | 4 | 3 | 2 | 4 |
| 2306 | Protein LYRIC | IPI00328715 | -0.142 | 0.26823 | 5 | 2 | 2 | 4 |
| 2307 | Protein transport protein Sec23B | IPI00017376 | -0.142 | 0.26823 | 4 | 3 | 3 | 3 |
| 2308 | tRNA (guanine-N(7)-)-methyltransferase | IPI00290184 | -0.142 | 0.26823 | 0 | 5 | 0 | 4 |
| 2309 | Synapse-associated protein 1 | IPI00059242 | -0.142 | 0.26823 | 4 | 3 | 3 | 3 |
| 2310 | Pyruvate dehydrogenase E1 component subunit alpha, testis-specific form, mitochondrial | IPI00024087 | -0.142 | 0.26823 | 3 | 4 | 4 | 0 |
| 2311 | Isoform 2 of Succinyl-CoA ligase [ADP-forming] subunit beta, mitochondrial | IPI00217232 | -0.142 | 0.26823 | 3 | 4 | 0 | 4 |
| 2312 | Isoform 1 of Erlin-2 | IPI00026942 | -0.142 | 0.26823 | 0 | 5 | 2 | 4 |
| 2313 | Heat shock 70 kDa protein 12A | IPI00011932 | -0.142 | 0.26823 | 0 | 5 | 2 | 4 |
| 2314 | serine/threonine-protein phosphatase PP1-alpha catalytic subunit isoform 3 | IPI00027423 | -0.142 | 0.26823 | 4 | 3 | 4 | 2 |
| 2315 | ATP synthase mitochondrial F1 complex assembly factor 2 | IPI00296999 | -0.142 | 0.26823 | 0 | 5 | 0 | 4 |
| 2316 | Splicing factor 45 | IPI00176706 | -0.142 | 0.26823 | 5 | 0 | 4 | 0 |
| 2317 | Isoform 1 of Phosphoribosyl pyrophosphate synthase-associated protein 1 | IPI00291578 | -0.142 | 0.26823 | 0 | 5 | 2 | 4 |
| 2318 | AP-3 complex subunit mu-1 | IPI00032459 | -0.142 | 0.26823 | 0 | 5 | 2 | 4 |
| 2319 | 40S ribosomal protein S6 | IPI00021840 | -0.145 | 0.26460 | 29 | 27 | 34 | 20 |
| 2320 | Peptidyl-prolyl cis-trans isomerase FKBP3 | IPI00024157 | -0.147 | 0.26401 | 28 | 25 | 30 | 21 |
| 2321 | CAAX prenyl protease 1 homolog | IPI00027180 | -0.150 | 0.26388 | 4 | 0 | 2 | 3 |
| 2322 | Mitochondrial-processing peptidase subunit alpha | IPI00166749 | -0.150 | 0.26388 | 3 | 3 | 3 | 2 |
| 2323 | NADH dehydrogenase [ubiquinone] 1 alpha subcomplex subunit 8 | IPI00219034 | -0.150 | 0.26388 | 4 | 2 | 3 | 0 |
| 2324 | EF-hand domain-containing protein D2 | IPI00060181 | -0.150 | 0.26388 | 0 | 4 | 3 | 0 |
| 2325 | Isoform 2 of Myosin-XVIIIa | IPI00334410 | -0.150 | 0.26388 | 3 | 3 | 0 | 3 |
| 2326 | Mitochondrial 18 kDa protein | IPI00784376 | -0.150 | 0.26388 | 0 | 4 | 0 | 3 |
| 2327 | Probable dimethyladenosine transferase | IPI00004459 | -0.150 | 0.26388 | 3 | 3 | 2 | 3 |
| 2328 | Isoform 1 of Fanconi anemia group D2 protein | IPI00075081 | -0.150 | 0.26388 | 3 | 3 | 2 | 3 |
| 2329 | Succinate dehydrogenase assembly factor 2, mitochondrial | IPI00016443 | -0.150 | 0.26388 | 4 | 0 | 3 | 0 |
| 2330 | Serine/threonine-protein phosphatase 1 regulatory subunit 10 | IPI00298731 | -0.150 | 0.26388 | 3 | 3 | 2 | 3 |
| 2331 | DCN1-like protein 1 | IPI00291893 | -0.150 | 0.26388 | 3 | 3 | 3 | 0 |
| 2332 | WW domain-binding protein 11 | IPI00170786 | -0.150 | 0.26388 | 3 | 3 | 0 | 3 |
| 2333 | Isoform 2 of Sorting nexin-3 | IPI00216508 | -0.150 | 0.26388 | 4 | 0 | 3 | 0 |
| 2334 | Protein LLP homolog | IPI00031615 | -0.150 | 0.26388 | 0 | 4 | 0 | 3 |
| 2335 | Nitric oxide synthase-interacting protein | IPI00006408 | -0.150 | 0.26388 | 4 | 0 | 2 | 3 |
| 2336 | Thioredoxin-like protein 4B | IPI00016481 | -0.150 | 0.26388 | 3 | 3 | 2 | 3 |
| 2337 | KIF1-binding protein | IPI00477355 | -0.150 | 0.26388 | 2 | 4 | 0 | 3 |
| 2338 | Ribosome biogenesis protein BOP1 | IPI00028955 | -0.150 | 0.26388 | 3 | 3 | 2 | 3 |
| 2339 | Ribosome biogenesis regulatory protein homolog | IPI00014253 | -0.150 | 0.26388 | 0 | 4 | 0 | 3 |
| 2340 | Translocation protein SEC63 homolog | IPI00218922 | -0.150 | 0.26388 | 0 | 4 | 0 | 3 |
| 2341 | Isoform 1 of Trafficking protein particle complex subunit 2 | IPI00005119 | -0.150 | 0.26388 | 0 | 4 | 0 | 3 |
| 2342 | cDNA FLJ56840, highly similar to Galactokinase | IPI00019383 | -0.150 | 0.26388 | 0 | 4 | 0 | 3 |
| 2343 | 114 kDa protein | IPI00166555 | -0.150 | 0.26388 | 3 | 3 | 3 | 0 |
| 2344 | Glutathione peroxidase 2 | IPI00298176 | -0.150 | 0.26388 | 2 | 4 | 0 | 3 |
| 2345 | Isoform 1 of Serine/threonine-protein phosphatase 4 regulatory subunit 3B | IPI00414323 | -0.150 | 0.26388 | 0 | 4 | 0 | 3 |
| 2346 | Active regulator of SIRT1 | IPI00219006 | -0.150 | 0.26388 | 2 | 4 | 2 | 3 |
| 2347 | Isoform 1 of WD repeat-containing protein 74 | IPI00018192 | -0.150 | 0.26388 | 3 | 3 | 3 | 0 |
| 2348 | G patch domain and KOW motifs-containing protein | IPI00024255 | -0.150 | 0.26388 | 0 | 4 | 2 | 3 |
| 2349 | Stress-70 protein, mitochondrial | IPI00007765 | -0.153 | 0.25226 | 92 | 76 | 96 | 69 |
| 2350 | 18 kDa protein | IPI00797709 | -0.160 | 0.25109 | 0 | 3 | 0 | 0 |
| 2351 | Isoform Long of Antigen KI-67 | IPI00004233 | -0.160 | 0.25109 | 3 | 2 | 0 | 0 |
| 2352 | Eukaryotic translation initiation factor 2A | IPI00012462 | -0.160 | 0.25109 | 3 | 0 | 2 | 0 |
| 2353 | Isoform A of Kinesin light chain 1 | IPI00020096 | -0.160 | 0.25109 | 0 | 3 | 0 | 0 |
| 2354 | HLA class I histocompatibility antigen, A-1 alpha chain | IPI00026569 | -0.160 | 0.25109 | 0 | 3 | 0 | 0 |
| 2355 | NADH dehydrogenase [ubiquinone] 1 alpha subcomplex subunit 6 | IPI00419266 | -0.160 | 0.25109 | 3 | 2 | 0 | 0 |
| 2356 | Ribose-5-phosphate isomerase | IPI00026513 | -0.160 | 0.25109 | 0 | 3 | 2 | 2 |
| 2357 | ATP-dependent RNA helicase DDX24 | IPI00006987 | -0.160 | 0.25109 | 2 | 3 | 0 | 2 |
| 2358 | Isoform 1 of tRNA (adenine-N(1)-)-methyltransferase non-catalytic subunit TRM6 | IPI00099311 | -0.160 | 0.25109 | 2 | 3 | 2 | 0 |
| 2359 | EF-hand domain-containing protein D1 | IPI00031091 | -0.160 | 0.25109 | 3 | 0 | 0 | 0 |
| 2360 | 28S ribosomal protein S18a, mitochondrial | IPI00018691 | -0.160 | 0.25109 | 0 | 3 | 0 | 2 |
| 2361 | Isoform 1 of Zinc phosphodiesterase ELAC protein 2 | IPI00396627 | -0.160 | 0.25109 | 3 | 2 | 0 | 2 |
| 2362 | Na(+)/H(+) exchange regulatory cofactor NHE-RF1 | IPI00003527 | -0.160 | 0.25109 | 0 | 3 | 0 | 0 |
| 2363 | DnaJ homolog subfamily C member 3 | IPI00006713 | -0.160 | 0.25109 | 3 | 2 | 0 | 0 |
| 2364 | Isoform 1 of Polyadenylate-binding protein 2 | IPI00005792 | -0.160 | 0.25109 | 2 | 3 | 2 | 2 |

| No. | Description | Accession number | STN[1] | p-Value[1] | Con_A[2] | Con_B[2] | RG3_A[2] | RG3_B[2] |
|---|---|---|---|---|---|---|---|---|
| 2365 | Hepatoma-derived growth factor-related protein 3 | IPI00007063 | -0.160 | 0.25109 | 3 | 0 | 0 | 0 |
| 2366 | Uncharacterized protein C7orf30 | IPI00061492 | -0.160 | 0.25109 | 0 | 3 | 0 | 0 |
| 2367 | Isoform 2 of Actin-related protein 2/3 complex subunit 5 | IPI00007280 | -0.160 | 0.25109 | 3 | 2 | 0 | 0 |
| 2368 | Serine/threonine-protein phosphatase 4 catalytic subunit | IPI00012833 | -0.160 | 0.25109 | 3 | 0 | 0 | 0 |
| 2369 | ribonucleotide reductase M2 polypeptide isoform 1 | IPI00011118 | -0.160 | 0.25109 | 3 | 2 | 0 | 0 |
| 2370 | Isoform 1 of Pre-mRNA-splicing factor 38A | IPI00171390 | -0.160 | 0.25109 | 0 | 3 | 2 | 0 |
| 2371 | Synaptogyrin-2 | IPI00013946 | -0.160 | 0.25109 | 3 | 0 | 2 | 0 |
| 2372 | Tubulin alpha-1A chain | IPI00180675 | -0.160 | 0.25109 | 0 | 3 | 0 | 0 |
| 2373 | Isoform 2 of Ubiquitin carboxyl-terminal hydrolase isozyme L5 | IPI00219512 | -0.160 | 0.25109 | 0 | 3 | 2 | 0 |
| 2374 | Importin 5 | IPI00639960 | -0.160 | 0.25109 | 3 | 0 | 0 | 0 |
| 2375 | Cleavage stimulation factor subunit 1 | IPI00011528 | -0.160 | 0.25109 | 0 | 3 | 2 | 2 |
| 2376 | H/ACA ribonucleoprotein complex subunit 2 | IPI00041325 | -0.160 | 0.25109 | 3 | 2 | 0 | 0 |
| 2377 | telomerase-binding protein EST1A isoform 2 | IPI00014252 | -0.160 | 0.25109 | 2 | 3 | 2 | 2 |
| 2378 | Isoform 3 of Pre-mRNA 3'-end-processing factor FIP1 | IPI00008449 | -0.160 | 0.25109 | 3 | 2 | 0 | 2 |
| 2379 | Isoform 1 of Protein 4.1 | IPI00003921 | -0.160 | 0.25109 | 3 | 0 | 2 | 0 |
| 2380 | Nucleoside-triphosphatase C1orf57 | IPI00031570 | -0.160 | 0.25109 | 0 | 3 | 2 | 2 |
| 2381 | Pumilio domain-containing protein C14orf21 | IPI00216999 | -0.160 | 0.25109 | 2 | 3 | 0 | 2 |
| 2382 | Coiled-coil domain-containing protein 25 | IPI00396174 | -0.160 | 0.25109 | 0 | 3 | 0 | 0 |
| 2383 | WD repeat-containing protein 5 | IPI00005492 | -0.160 | 0.25109 | 2 | 3 | 0 | 2 |
| 2384 | NIF3L1 isoform gamma | IPI00451429 | -0.160 | 0.25109 | 2 | 3 | 0 | 2 |
| 2385 | Keratin, type I cytoskeletal 17 | IPI00450768 | -0.160 | 0.25109 | 3 | 0 | 0 | 0 |
| 2386 | Isoform 1 of Protein-tyrosine phosphatase mitochondrial 1 | IPI00174190 | -0.160 | 0.25109 | 3 | 0 | 0 | 2 |
| 2387 | Isoform B of AP-2 complex subunit alpha-1 | IPI00256684 | -0.160 | 0.25109 | 3 | 2 | 2 | 2 |
| 2388 | Isoform AGX2 of UDP-N-acetylhexosamine pyrophosphorylase | IPI00000684 | -0.160 | 0.25109 | 0 | 3 | 0 | 0 |
| 2389 | A-kinase anchor protein 8 | IPI00014474 | -0.160 | 0.25109 | 0 | 3 | 0 | 0 |
| 2390 | Ribonuclease inhibitor | IPI00550069 | -0.160 | 0.25109 | 3 | 2 | 2 | 2 |
| 2391 | Isoform 1 of Putative RNA-binding protein 15 | IPI00102752 | -0.160 | 0.25109 | 0 | 3 | 0 | 0 |
| 2392 | Beta-adrenergic receptor kinase 1 | IPI00012497 | -0.160 | 0.25109 | 0 | 3 | 0 | 0 |
| 2393 | Dynein, light chain, roadblock-type 1 | IPI00412497 | -0.160 | 0.25109 | 0 | 3 | 0 | 0 |
| 2394 | ubiquitin-like with PHD and ring finger domains 1 isoform 2 | IPI00797279 | -0.160 | 0.25109 | 3 | 2 | 0 | 0 |
| 2395 | Charged multivesicular body protein 4b | IPI00025974 | -0.160 | 0.25109 | 3 | 0 | 0 | 0 |
| 2396 | Probable ATP-dependent RNA helicase DDX52 | IPI00032423 | -0.160 | 0.25109 | 0 | 3 | 0 | 2 |
| 2397 | Probable tRNA(His) guanylyltransferase | IPI00016559 | -0.160 | 0.25109 | 3 | 2 | 0 | 0 |
| 2398 | Isoform 2 of NudC domain-containing protein 1 | IPI00306398 | -0.160 | 0.25109 | 0 | 3 | 0 | 0 |
| 2399 | Guanine nucleotide-binding protein G(q) subunit alpha | IPI00288947 | -0.160 | 0.25109 | 0 | 3 | 2 | 2 |
| 2400 | Desmoglein-2 | IPI00028931 | -0.160 | 0.25109 | 3 | 0 | 0 | 0 |
| 2401 | TATA box-binding protein-like protein 1 | IPI00032911 | -0.160 | 0.25109 | 0 | 3 | 2 | 2 |
| 2402 | DNA-directed RNA polymerase II subunit RPB4 | IPI00007283 | -0.160 | 0.25109 | 2 | 3 | 0 | 2 |
| 2403 | LanC-like protein 1 | IPI00005724 | -0.160 | 0.25109 | 0 | 3 | 0 | 0 |
| 2404 | Transcriptional activator protein Pur-alpha | IPI00023591 | -0.160 | 0.25109 | 0 | 3 | 0 | 0 |
| 2405 | COMM domain-containing protein 3 | IPI00015773 | -0.160 | 0.25109 | 3 | 0 | 2 | 0 |
| 2406 | Cysteine and glycine-rich protein 2 | IPI00002824 | -0.160 | 0.25109 | 3 | 0 | 2 | 0 |
| 2407 | coatomer subunit epsilon isoform c | IPI00399319 | -0.160 | 0.25109 | 0 | 3 | 0 | 0 |
| 2408 | serine/threonine-protein kinase MST4 isoform 3 | IPI00182383 | -0.160 | 0.25109 | 3 | 0 | 0 | 0 |
| 2409 | Dipeptidase 1 | IPI00059476 | -0.160 | 0.25109 | 0 | 3 | 2 | 2 |
| 2410 | RAC-alpha serine/threonine-protein kinase | IPI00012866 | -0.160 | 0.25109 | 3 | 2 | 0 | 0 |
| 2411 | Isoform 2 of Syntaxin-binding protein 1 | IPI00046057 | -0.160 | 0.25109 | 0 | 3 | 0 | 0 |
| 2412 | Nucleoporin Nup37 | IPI00171665 | -0.160 | 0.25109 | 0 | 3 | 0 | 2 |
| 2413 | Interferon-induced protein with tetratricopeptide repeats 5 | IPI00012756 | -0.160 | 0.25109 | 0 | 3 | 0 | 2 |
| 2414 | UPF0552 protein C15orf38 | IPI00074225 | -0.160 | 0.25109 | 3 | 0 | 2 | 0 |
| 2415 | Isoform 1 of Kinesin-like protein KIF2A | IPI00010368 | -0.160 | 0.25109 | 3 | 2 | 2 | 2 |
| 2416 | Estradiol 17-beta-dehydrogenase 8 | IPI00021890 | -0.160 | 0.25109 | 3 | 0 | 2 | 2 |
| 2417 | Sorting nexin-5 | IPI00295209 | -0.160 | 0.25109 | 0 | 3 | 2 | 0 |
| 2418 | Ubiquitin-conjugating enzyme E2 variant 2 | IPI00019600 | -0.160 | 0.25109 | 3 | 2 | 2 | 0 |
| 2419 | Endonuclease G, mitochondrial | IPI00290614 | -0.160 | 0.25109 | 0 | 3 | 0 | 0 |
| 2420 | Integrator complex subunit 9 | IPI00290514 | -0.160 | 0.25109 | 2 | 3 | 0 | 2 |
| 2421 | Uncharacterized protein C20orf29 | IPI00019941 | -0.160 | 0.25109 | 0 | 3 | 0 | 2 |
| 2422 | FtsJ methyltransferase domain-containing protein 1 | IPI00334846 | -0.160 | 0.25109 | 0 | 3 | 0 | 0 |
| 2423 | Isoform 1 of Gephyrin | IPI00016006 | -0.160 | 0.25109 | 0 | 3 | 0 | 0 |
| 2424 | 3'-5' exoribonuclease CSL4 homolog | IPI00032823 | -0.160 | 0.25109 | 3 | 0 | 2 | 0 |
| 2425 | 15 kDa protein | IPI00000186 | -0.160 | 0.25109 | 0 | 3 | 0 | 0 |
| 2426 | Isoform 2 of Myosin-VI | IPI00008455 | -0.160 | 0.25109 | 0 | 3 | 0 | 2 |
| 2427 | Putative uncharacterized protein LCMT1 | IPI00296370 | -0.160 | 0.25109 | 0 | 3 | 0 | 0 |
| 2428 | Isoform 1 of F-box only protein 4 | IPI00073357 | -0.160 | 0.25109 | 0 | 3 | 0 | 0 |
| 2429 | Annexin A1 | IPI00218918 | -0.160 | 0.25109 | 0 | 3 | 0 | 0 |
| 2430 | Zinc finger CCHC-type and RNA-binding motif-containing protein 1 | IPI00154614 | -0.160 | 0.25109 | 0 | 3 | 0 | 0 |
| 2431 | Trafficking protein particle complex subunit 5 | IPI00177509 | -0.160 | 0.25109 | 0 | 3 | 0 | 0 |
| 2432 | TBC1 domain family member 15 isoform 1 | IPI00154645 | -0.160 | 0.25109 | 0 | 3 | 0 | 2 |
| 2433 | WD repeat-containing protein 46 | IPI00023126 | -0.160 | 0.25109 | 0 | 3 | 0 | 2 |
| 2434 | Methionyl-tRNA synthetase, mitochondrial | IPI00062839 | -0.160 | 0.25109 | 0 | 3 | 0 | 0 |
| 2435 | Histone acetyltransferase MYST2 | IPI00180764 | -0.160 | 0.25109 | 2 | 3 | 0 | 0 |
| 2436 | Probable ATP-dependent RNA helicase DDX49 | IPI00003739 | -0.160 | 0.25109 | 3 | 0 | 0 | 0 |
| 2437 | THUMP domain-containing protein 3 | IPI00306127 | -0.160 | 0.25109 | 0 | 3 | 0 | 0 |
| 2438 | cDNA FLJ11251 fis, clone PLACE1008813 | IPI00010953 | -0.160 | 0.25109 | 0 | 3 | 0 | 0 |
| 2439 | Eukaryotic peptide chain release factor subunit 1 | IPI00429191 | -0.160 | 0.24097 | 20 | 21 | 17 | 22 |
| 2440 | Cleavage and polyadenylation specificity factor subunit 5 | IPI00646917 | -0.163 | 0.24038 | 18 | 21 | 19 | 18 |
| 2441 | Isoform 1 of Heterogeneous nuclear ribonucleoprotein H3 | IPI00013877 | -0.164 | 0.24038 | 13 | 25 | 17 | 19 |
| 2442 | Peroxiredoxin-4 | IPI00011937 | -0.164 | 0.24038 | 20 | 18 | 18 | 18 |
| 2443 | Isoform 1 of DNA replication licensing factor MCM7 | IPI00299904 | -0.167 | 0.23980 | 22 | 14 | 17 | 17 |
| 2444 | transcription activator BRG1 isoform D | IPI00029822 | -0.170 | 0.23896 | 19 | 15 | 13 | 19 |
| 2445 | Histone-binding protein RBBP7 | IPI00395865 | -0.179 | 0.23612 | 15 | 14 | 13 | 14 |
| 2446 | Chloride intracellular channel protein 1 | IPI00010896 | -0.181 | 0.23607 | 54 | 46 | 54 | 43 |
| 2447 | mRNA turnover protein 4 homolog | IPI00106491 | -0.181 | 0.23607 | 12 | 16 | 13 | 13 |
| 2448 | Isoform 1 of La-related protein 1 | IPI00185919 | -0.183 | 0.23520 | 10 | 17 | 12 | 13 |
| 2449 | Proteasome 26S non-ATPase subunit 11 variant (Fragment) | IPI00105598 | -0.183 | 0.23520 | 11 | 16 | 9 | 16 |
| 2450 | Translin-associated protein X | IPI00293350 | -0.188 | 0.23377 | 16 | 9 | 15 | 8 |
| 2451 | Isoform 1 of Glucosamine--fructose-6-phosphate aminotransferase [isomerizing] 1 | IPI00217952 | -0.191 | 0.23377 | 11 | 13 | 10 | 12 |
| 2452 | 60S ribosomal protein L38 | IPI00215790 | -0.193 | 0.23218 | 11 | 12 | 10 | 11 |
| 2453 | DNA-(apurinic or apyrimidinic site) lyase | IPI00215911 | -0.196 | 0.23214 | 10 | 12 | 10 | 10 |
| 2454 | Ran GTPase-activating protein 1 | IPI00294879 | -0.196 | 0.23214 | 12 | 10 | 12 | 8 |
| 2455 | Ras-related protein Rab-14 | IPI00291928 | -0.196 | 0.23214 | 11 | 11 | 9 | 11 |
| 2456 | Alpha-soluble NSF attachment protein | IPI00009253 | -0.196 | 0.23214 | 9 | 13 | 9 | 11 |
| 2457 | Isoform 2 of Structural maintenance of chromosomes flexible hinge domain-containing protein 1 | IPI00465022 | -0.199 | 0.23005 | 11 | 10 | 9 | 10 |
| 2458 | Transmembrane protein 165 | IPI00307572 | -0.202 | 0.23001 | 13 | 7 | 11 | 7 |
| 2459 | Probable ATP-dependent RNA helicase DDX23 | IPI00006725 | -0.202 | 0.23001 | 8 | 12 | 9 | 9 |

| No. | Description | Accession number | STN[1] | p-Value[1] | Con_A[2] | Con_B[2] | RG3_A[2] | RG3_B[2] |
|---|---|---|---|---|---|---|---|---|
| 2460 | Isoform 3 of Obg-like ATPase 1 | IPI00216106 | -0.206 | 0.22729 | 12 | 7 | 10 | 7 |
| 2461 | Isoform Long of Double-stranded RNA-binding protein Staufen homolog 1 | IPI00000001 | -0.210 | 0.22717 | 8 | 10 | 9 | 7 |
| 2462 | Isoform 1 of Large proline-rich protein BAT3 | IPI00465128 | -0.210 | 0.22717 | 11 | 7 | 7 | 9 |
| 2463 | Isoform 1 of DNA primase large subunit | IPI00027705 | -0.210 | 0.22717 | 8 | 10 | 6 | 10 |
| 2464 | COP9 signalosome complex subunit 4 | IPI00171844 | -0.210 | 0.22717 | 9 | 9 | 7 | 9 |
| 2465 | Isoform 1 of Fanconi anemia group I protein | IPI00019447 | -0.214 | 0.22386 | 7 | 10 | 0 | 13 |
| 2466 | Nucleoporin 85 | IPI00171542 | -0.214 | 0.22386 | 13 | 4 | 8 | 7 |
| 2467 | 39S ribosomal protein L28, mitochondrial | IPI00172594 | -0.214 | 0.22386 | 8 | 9 | 6 | 9 |
| 2468 | Isoform Alpha of Signal transducer and activator of transcription 1-alpha/beta | IPI00030781 | -0.214 | 0.22386 | 7 | 10 | 4 | 11 |
| 2469 | Polyribonucleotide nucleotidyltransferase 1, mitochondrial | IPI00744711 | -0.214 | 0.22386 | 9 | 8 | 7 | 8 |
| 2470 | Nuclear pore complex protein Nup133 | IPI00291200 | -0.218 | 0.22332 | 7 | 9 | 8 | 6 |
| 2471 | Guanine nucleotide-binding protein subunit alpha-13 | IPI00290928 | -0.218 | 0.22332 | 6 | 10 | 5 | 9 |
| 2472 | Pyrroline-5-carboxylate reductase 2 | IPI00470610 | -0.218 | 0.22332 | 7 | 9 | 6 | 8 |
| 2473 | Ubiquitin-like modifier-activating enzyme 1 | IPI00645078 | -0.222 | 0.22027 | 66 | 63 | 64 | 61 |
| 2474 | Isoform A of Ras GTPase-activating protein-binding protein 2 | IPI00009057 | -0.223 | 0.22010 | 7 | 8 | 6 | 7 |
| 2475 | Isoform 1 of N-alpha-acetyltransferase 50, NatE catalytic subunit | IPI00018627 | -0.223 | 0.22010 | 6 | 9 | 5 | 8 |
| 2476 | regulator of chromosome condensation 1 isoform a | IPI00001661 | -0.223 | 0.22010 | 8 | 7 | 6 | 7 |
| 2477 | Splicing factor 3B subunit 4 | IPI00017339 | -0.228 | 0.21951 | 6 | 8 | 6 | 6 |
| 2478 | UV excision repair protein RAD23 homolog B | IPI00008223 | -0.228 | 0.21951 | 5 | 9 | 7 | 5 |
| 2479 | Isoform 1 of 1-phosphatidylinositol-4,5-bisphosphate phosphodiesterase gamma-1 | IPI00016736 | -0.228 | 0.21951 | 8 | 6 | 6 | 6 |
| 2480 | Phosphoserine phosphatase | IPI00019178 | -0.228 | 0.21951 | 9 | 5 | 6 | 6 |
| 2481 | Nascent polypeptide-associated complex subunit alpha | IPI00023748 | -0.232 | 0.21495 | 25 | 21 | 20 | 23 |
| 2482 | NEDD8 | IPI00020008 | -0.234 | 0.21475 | 5 | 8 | 6 | 5 |
| 2483 | DKFZP586J0619 protein | IPI00740961 | -0.234 | 0.21475 | 7 | 6 | 6 | 5 |
| 2484 | Isoform 1 of Luc7-like protein 3 | IPI00107745 | -0.234 | 0.21475 | 6 | 7 | 5 | 6 |
| 2485 | Bifunctional ATP-dependent dihydroxyacetone kinase/FAD-AMP lyase (cyclizing) | IPI00551024 | -0.237 | 0.21437 | 20 | 23 | 16 | 24 |
| 2486 | Isoform 1 of BH3-interacting domain death agonist | IPI00413587 | -0.241 | 0.21412 | 6 | 6 | 4 | 6 |
| 2487 | WD repeat-containing protein 75 | IPI00217240 | -0.241 | 0.21412 | 8 | 4 | 4 | 6 |
| 2488 | Niban-like protein 1 | IPI00456750 | -0.241 | 0.21412 | 5 | 7 | 3 | 7 |
| 2489 | Isoform 2 of Septin-11 | IPI00019376 | -0.241 | 0.21412 | 6 | 6 | 6 | 4 |
| 2490 | Calponin-3 | IPI00216682 | -0.241 | 0.21412 | 5 | 7 | 2 | 8 |
| 2491 | Glucosamine--fructose-6-phosphate aminotransferase [isomerizing] 2 | IPI00216159 | -0.241 | 0.21412 | 7 | 5 | 5 | 5 |
| 2492 | Isoform 5 of Sigma non-opioid intracellular receptor 1 | IPI00167206 | -0.241 | 0.21412 | 5 | 7 | 4 | 6 |
| 2493 | GMP synthase [glutamine-hydrolyzing] | IPI00029079 | -0.243 | 0.20839 | 24 | 16 | 20 | 17 |
| 2494 | Isoform Long of Spectrin beta chain, brain 1 | IPI00005614 | -0.244 | 0.20785 | 103 | 92 | 104 | 86 |
| 2495 | Interleukin enhancer-binding factor 2 | IPI00005198 | -0.245 | 0.20764 | 50 | 45 | 48 | 43 |
| 2496 | Isoform 2 of Splicing factor 1 | IPI00294627 | -0.247 | 0.20764 | 20 | 18 | 11 | 24 |
| 2497 | triosephosphate isomerase 1 isoform 2 | IPI00465028 | -0.248 | 0.20730 | 7 | 4 | 7 | 0 |
| 2498 | Isoform 2 of Suppressor of G2 allele of SKP1 homolog | IPI00791573 | -0.248 | 0.20730 | 6 | 5 | 3 | 6 |
| 2499 | Ubiquitin-conjugating enzyme E2 O | IPI00783378 | -0.248 | 0.20730 | 7 | 4 | 5 | 4 |
| 2500 | 39S ribosomal protein L48, mitochondrial | IPI00295066 | -0.248 | 0.20730 | 6 | 5 | 5 | 4 |
| 2501 | Isoform 1 of 39S ribosomal protein L47, mitochondrial | IPI00030820 | -0.248 | 0.20730 | 5 | 6 | 3 | 6 |
| 2502 | LDLR chaperone MESD | IPI00399089 | -0.248 | 0.20730 | 4 | 7 | 4 | 5 |
| 2503 | Isoform 1 of DAZ-associated protein 1 | IPI00165230 | -0.248 | 0.20730 | 3 | 8 | 3 | 6 |
| 2504 | cDNA FLJ78497 | IPI00289535 | -0.248 | 0.20730 | 5 | 6 | 4 | 5 |
| 2505 | Isoform 2 of Beta-catenin-like protein 1 | IPI00472981 | -0.248 | 0.20730 | 7 | 4 | 7 | 0 |
| 2506 | Peptidylprolyl isomerase domain and WD repeat-containing protein 1 | IPI00149650 | -0.248 | 0.20730 | 4 | 7 | 3 | 6 |
| 2507 | Isoform 1 of Filamin-C | IPI00178352 | -0.249 | 0.20730 | 18 | 19 | 22 | 12 |
| 2508 | Replication factor C subunit 5 | IPI00031514 | -0.252 | 0.20705 | 16 | 20 | 16 | 17 |
| 2509 | Isoform 2 of Obg-like ATPase 1 | IPI00216105 | -0.256 | 0.20651 | 16 | 18 | 17 | 14 |
| 2510 | BRI3-binding protein | IPI00103599 | -0.257 | 0.20651 | 5 | 5 | 4 | 4 |
| 2511 | CCAAT/enhancer-binding protein zeta | IPI00306723 | -0.257 | 0.20651 | 5 | 5 | 3 | 5 |
| 2512 | Isoform 1 of Prolyl 4-hydroxylase subunit alpha-1 | IPI00009923 | -0.257 | 0.20651 | 3 | 7 | 3 | 5 |
| 2513 | Isoform 1 of Pentatricopeptide repeat-containing protein 3, mitochondrial | IPI00783302 | -0.257 | 0.20651 | 4 | 6 | 4 | 4 |
| 2514 | Isoform 1 of Phosphoenolpyruvate carboxykinase [GTP], mitochondrial | IPI00797038 | -0.257 | 0.20651 | 4 | 6 | 5 | 3 |
| 2515 | Isoform Long of Tyrosine-protein kinase SYK | IPI00018597 | -0.257 | 0.20651 | 7 | 3 | 3 | 5 |
| 2516 | Isoform 2 of Guanine nucleotide-binding protein G(i) subunit alpha-2 | IPI00217906 | -0.259 | 0.19952 | 10 | 23 | 14 | 16 |
| 2517 | 26S protease regulatory subunit S10B | IPI00021926 | -0.262 | 0.19923 | 14 | 18 | 15 | 14 |
| 2518 | UPF0687 protein C20orf27 | IPI00101095 | -0.266 | 0.19856 | 4 | 5 | 2 | 5 |
| 2519 | C-terminal-binding protein 1 | IPI00012835 | -0.266 | 0.19856 | 3 | 6 | 2 | 5 |
| 2520 | Ferritin heavy chain | IPI00554521 | -0.266 | 0.19856 | 4 | 5 | 3 | 4 |
| 2521 | cDNA FLJ61739, highly similar to Serine/arginine repetitive matrix protein 1 | IPI00328293 | -0.266 | 0.19856 | 6 | 3 | 5 | 0 |
| 2522 | Translational activator of cytochrome c oxidase 1 | IPI00019903 | -0.266 | 0.19856 | 4 | 5 | 2 | 5 |
| 2523 | Isoform 1 of Nucleoredoxin | IPI00304267 | -0.266 | 0.19856 | 3 | 6 | 2 | 5 |
| 2524 | 39S ribosomal protein L9, mitochondrial | IPI00307409 | -0.266 | 0.19856 | 4 | 5 | 4 | 3 |
| 2525 | RNA-binding motif protein, X-linked-like-2 | IPI00004450 | -0.266 | 0.19856 | 5 | 4 | 2 | 5 |
| 2526 | Isoform 2 of 3-hydroxyisobutyryl-CoA hydrolase, mitochondrial | IPI00377161 | -0.266 | 0.19856 | 5 | 4 | 3 | 4 |
| 2527 | Peflin | IPI00018235 | -0.266 | 0.19856 | 5 | 4 | 3 | 4 |
| 2528 | Isoform 2 of S-phase kinase-associated protein 1 | IPI00172421 | -0.267 | 0.19856 | 16 | 14 | 12 | 15 |
| 2529 | Isoform Cytoplasmic of Lysyl-tRNA synthetase | IPI00014238 | -0.268 | 0.19819 | 40 | 32 | 33 | 35 |
| 2530 | Isoform 1 of Proteasome activator complex subunit 3 | IPI00030243 | -0.270 | 0.19810 | 12 | 17 | 12 | 14 |
| 2531 | 60S ribosomal protein L5 | IPI00000494 | -0.272 | 0.19785 | 37 | 32 | 46 | 19 |
| 2532 | Elongation factor 2 | IPI00186290 | -0.273 | 0.19785 | 132 | 111 | 125 | 112 |
| 2533 | Signal recognition particle receptor subunit beta | IPI00295098 | -0.273 | 0.19785 | 14 | 14 | 17 | 8 |
| 2534 | 40S ribosomal protein S2 | IPI00013485 | -0.275 | 0.19772 | 79 | 54 | 79 | 49 |
| 2535 | Isoform 1 of Sodium-coupled neutral amino acid transporter 2 | IPI00410034 | -0.277 | 0.19768 | 12 | 15 | 12 | 12 |
| 2536 | Translational activator GCN1 | IPI00001159 | -0.277 | 0.19731 | 114 | 117 | 106 | 119 |
| 2537 | Nuclear pore complex protein Nup93 | IPI00397904 | -0.278 | 0.19726 | 6 | 2 | 3 | 3 |
| 2538 | Isoform SRP40-1 of Splicing factor, arginine/serine-rich 5 | IPI00012341 | -0.278 | 0.19726 | 3 | 5 | 2 | 4 |
| 2539 | Isoform 1 of Cullin-4A | IPI00419273 | -0.278 | 0.19726 | 5 | 3 | 4 | 0 |
| 2540 | Ribosome production factor 2 homolog | IPI00396329 | -0.278 | 0.19726 | 3 | 5 | 0 | 4 |
| 2541 | Putative uncharacterized protein DKFZp313O211 | IPI00552186 | -0.278 | 0.19726 | 6 | 0 | 4 | 0 |
| 2542 | Carnitine O-palmitoyltransferase 2, mitochondrial | IPI00012912 | -0.278 | 0.19726 | 3 | 5 | 2 | 4 |
| 2543 | Isoform 2 of Basic leucine zipper and W2 domain-containing protein 1 | IPI00180128 | -0.278 | 0.19726 | 3 | 5 | 3 | 3 |
| 2544 | Cleavage and polyadenylation specificity factor subunit 2 | IPI00419531 | -0.278 | 0.19726 | 3 | 5 | 0 | 4 |
| 2545 | AP-3 complex subunit sigma-1 | IPI00014624 | -0.278 | 0.19726 | 3 | 5 | 0 | 4 |
| 2546 | Programmed cell death protein 5 | IPI00023640 | -0.278 | 0.19726 | 4 | 4 | 3 | 3 |
| 2547 | Tumor protein, translationally-controlled 1 | IPI00009943 | -0.280 | 0.18493 | 12 | 14 | 11 | 12 |
| 2548 | SDHA protein | IPI00217143 | -0.284 | 0.18397 | 18 | 7 | 17 | 5 |
| 2549 | Probable ATP-dependent RNA helicase DDX47 | IPI00023972 | -0.284 | 0.18397 | 9 | 16 | 10 | 12 |
| 2550 | Methylenetetrahydrofolate dehydrogenase (NADP+ dependent) 1-like | IPI00291646 | -0.284 | 0.18397 | 13 | 12 | 11 | 11 |
| 2551 | THO complex subunit 2 | IPI00158615 | -0.288 | 0.18351 | 12 | 12 | 9 | 12 |
| 2552 | Vesicle-fusing ATPase | IPI00006451 | -0.292 | 0.18221 | 12 | 11 | 12 | 8 |
| 2553 | Transgelin-2 | IPI00550363 | -0.292 | 0.18221 | 11 | 12 | 10 | 10 |
| 2554 | Nucleolar complex protein 4 homolog | IPI00031661 | -0.292 | 0.18171 | 0 | 5 | 2 | 3 |

| No. | Description | Accession number | STN[1] | p-Value[1] | Con_A[2] | Con_B[2] | RG3_A[2] | RG3_B[2] |
|---|---|---|---|---|---|---|---|---|
| 2555 | Mitochondrial ribonuclease P protein 1 | IPI00099996 | -0.292 | 0.18171 | 2 | 5 | 3 | 2 |
| 2556 | Protein C20orf11 | IPI00016634 | -0.292 | 0.18171 | 0 | 5 | 0 | 3 |
| 2557 | Uncharacterized protein KIAA0406 | IPI00011702 | -0.292 | 0.18171 | 4 | 3 | 0 | 3 |
| 2558 | V-type proton ATPase subunit C 1 | IPI00007814 | -0.292 | 0.18171 | 4 | 3 | 0 | 3 |
| 2559 | Cytovillin 2 (Fragment) | IPI00384282 | -0.292 | 0.18171 | 5 | 2 | 0 | 3 |
| 2560 | Isoform 1 of SWI/SNF-related matrix-associated actin-dependent regulator of chromatin subfamily E | IPI00017669 | -0.292 | 0.18171 | 4 | 3 | 3 | 0 |
| 2561 | Scaffold attachment factor B1 | IPI00300631 | -0.292 | 0.18171 | 3 | 4 | 3 | 0 |
| 2562 | Isoform 1 of Probable ATP-dependent RNA helicase DHX36 | IPI00027415 | -0.292 | 0.18171 | 3 | 4 | 3 | 2 |
| 2563 | Isoform 1 of Adenylate kinase 2, mitochondrial | IPI00215901 | -0.297 | 0.18125 | 11 | 11 | 11 | 8 |
| 2564 | Isoform 3 of Glutaminase kidney isoform, mitochondrial | IPI00215687 | -0.297 | 0.18125 | 12 | 10 | 9 | 10 |
| 2565 | 14-3-3 protein gamma | IPI00220642 | -0.298 | 0.18087 | 30 | 22 | 28 | 20 |
| 2566 | GTP-binding nuclear protein Ran | IPI00643041 | -0.304 | 0.17962 | 57 | 41 | 48 | 45 |
| 2567 | Histone acetyltransferase type B catalytic subunit | IPI00024719 | -0.306 | 0.17916 | 10 | 10 | 8 | 9 |
| 2568 | Isoform 1 of Replication factor C subunit 2 | IPI00017412 | -0.306 | 0.17916 | 8 | 12 | 9 | 8 |
| 2569 | FAS-associated factor 2 | IPI00172656 | -0.306 | 0.17916 | 11 | 9 | 8 | 9 |
| 2570 | Coatomer subunit gamma-2 | IPI00002557 | -0.310 | 0.17849 | 4 | 2 | 2 | 2 |
| 2571 | Isoform 1 of Pescadillo homolog | IPI00003768 | -0.310 | 0.17849 | 0 | 4 | 0 | 2 |
| 2572 | Protein dpy-30 homolog | IPI00028109 | -0.310 | 0.17849 | 2 | 4 | 0 | 0 |
| 2573 | Putative uncharacterized protein INF2 | IPI00872508 | -0.310 | 0.17849 | 2 | 4 | 2 | 2 |
| 2574 | Farnesyl pyrophosphate synthase | IPI00914566 | -0.310 | 0.17849 | 0 | 4 | 0 | 0 |
| 2575 | WD repeat-containing protein 3 | IPI00009471 | -0.310 | 0.17849 | 4 | 0 | 0 | 2 |
| 2576 | Isoform 1 of Telomere-associated protein RIF1 | IPI00293845 | -0.310 | 0.17849 | 0 | 4 | 0 | 0 |
| 2577 | 28S ribosomal protein S34, mitochondrial | IPI00169413 | -0.310 | 0.17849 | 0 | 4 | 2 | 2 |
| 2578 | Full-length cDNA 5-PRIME end of clone CS0DJ009YL13 of T cells (Jurkat cell line) of Homo sapiens | IPI00384016 | -0.310 | 0.17849 | 4 | 0 | 0 | 0 |
| 2579 | Syntaxin-binding protein 3 | IPI00297626 | -0.310 | 0.17849 | 4 | 0 | 0 | 2 |
| 2580 | Isoform 2 of Mediator of DNA damage checkpoint protein 1 | IPI00470805 | -0.310 | 0.17849 | 4 | 0 | 0 | 0 |
| 2581 | U3 small nucleolar RNA-associated protein 15 homolog | IPI00152708 | -0.310 | 0.17849 | 0 | 4 | 0 | 2 |
| 2582 | Isoamyl acetate-hydrolyzing esterase 1 homolog | IPI00419194 | -0.310 | 0.17849 | 4 | 0 | 2 | 0 |
| 2583 | Periplakin | IPI00298057 | -0.310 | 0.17849 | 3 | 3 | 2 | 2 |
| 2584 | Aminopeptidase B | IPI00642211 | -0.310 | 0.17849 | 4 | 2 | 0 | 0 |
| 2585 | Peptidyl-prolyl cis-trans isomerase-like 1 | IPI00007019 | -0.310 | 0.17849 | 2 | 4 | 0 | 0 |
| 2586 | Isoform 1 of GTP-binding protein 10 | IPI00167638 | -0.310 | 0.17849 | 0 | 4 | 2 | 0 |
| 2587 | Isoform 2 of Phosphoenolpyruvate carboxykinase [GTP], mitochondrial | IPI00384116 | -0.310 | 0.17849 | 2 | 4 | 2 | 0 |
| 2588 | Phosphomevalonate kinase | IPI00220648 | -0.310 | 0.17849 | 3 | 3 | 0 | 2 |
| 2589 | Mitochondrial import inner membrane translocase subunit Tim16 | IPI00218463 | -0.310 | 0.17849 | 0 | 4 | 0 | 0 |
| 2590 | Isoform 1 of Transmembrane emp24 domain-containing protein 4 | IPI00296259 | -0.310 | 0.17849 | 3 | 3 | 2 | 2 |
| 2591 | Replication initiator 1 | IPI00549171 | -0.310 | 0.17849 | 3 | 3 | 0 | 0 |
| 2592 | Isoform 1 of Insulin-like growth factor 2 mRNA-binding protein 3 | IPI00658000 | -0.310 | 0.17849 | 0 | 4 | 0 | 2 |
| 2593 | Isoform 1 of UPF0424 protein C1orf128 | IPI00015351 | -0.310 | 0.17849 | 0 | 4 | 2 | 0 |
| 2594 | 74 kDa protein | IPI00290439 | -0.310 | 0.17849 | 0 | 4 | 0 | 0 |
| 2595 | PRMT3 protein (Fragment) | IPI00103026 | -0.310 | 0.17849 | 2 | 4 | 0 | 0 |
| 2596 | Isoform 3 of Nuclear transcription factor Y subunit gamma | IPI00071697 | -0.310 | 0.17849 | 0 | 4 | 0 | 0 |
| 2597 | 1-phosphatidylinositol-4,5-bisphosphate phosphodiesterase beta-3 | IPI00010400 | -0.310 | 0.17849 | 4 | 2 | 0 | 2 |
| 2598 | Fatty acyl-CoA reductase 1 | IPI00478838 | -0.310 | 0.17849 | 4 | 2 | 0 | 2 |
| 2599 | YEATS domain-containing protein 4 | IPI00008536 | -0.310 | 0.17849 | 3 | 3 | 2 | 2 |
| 2600 | Chromosome-associated kinesin KIF4B | IPI00175193 | -0.310 | 0.17849 | 2 | 4 | 0 | 0 |
| 2601 | DNA ligase 1 | IPI00219841 | -0.310 | 0.17849 | 3 | 3 | 0 | 0 |
| 2602 | Carbonic anhydrase 1 | IPI00215983 | -0.310 | 0.17849 | 0 | 4 | 0 | 0 |
| 2603 | Isoform 1 of Transcription elongation factor A protein 1 | IPI00333215 | -0.310 | 0.17849 | 3 | 3 | 2 | 2 |
| 2604 | Isoform 1 of ATP-dependent metalloprotease YME1L1 | IPI00045946 | -0.310 | 0.17849 | 2 | 4 | 2 | 0 |
| 2605 | Ras-related protein Rab-22A | IPI00007756 | -0.310 | 0.17849 | 0 | 4 | 0 | 2 |
| 2606 | Ribosome biogenesis protein NSA2 homolog | IPI00007089 | -0.310 | 0.17849 | 0 | 4 | 0 | 2 |
| 2607 | U3 small nucleolar RNA-associated protein 6 homolog | IPI00020128 | -0.310 | 0.17849 | 3 | 3 | 0 | 0 |
| 2608 | Protein UXT | IPI00002646 | -0.310 | 0.17849 | 0 | 4 | 0 | 2 |
| 2609 | Isoform 1 of Dynamin-3 | IPI00221332 | -0.310 | 0.17849 | 0 | 4 | 2 | 2 |
| 2610 | WD repeat-containing protein 43 | IPI00937477 | -0.310 | 0.17849 | 0 | 4 | 0 | 0 |
| 2611 | UPF0444 transmembrane protein C12orf23 | IPI00184546 | -0.310 | 0.17849 | 0 | 4 | 0 | 0 |
| 2612 | Isoform 1 of Zinc finger protein 326 | IPI00373877 | -0.310 | 0.17849 | 4 | 0 | 0 | 0 |
| 2613 | 60S ribosomal protein L32 | IPI00395998 | -0.312 | 0.15913 | 11 | 8 | 9 | 7 |
| 2614 | Reticulocalbin-1 | IPI00015842 | -0.312 | 0.15913 | 9 | 10 | 11 | 5 |
| 2615 | Exosome complex exonuclease RRP4 | IPI00015905 | -0.312 | 0.15913 | 7 | 12 | 8 | 8 |
| 2616 | Elongation factor 1-beta | IPI00178440 | -0.313 | 0.15883 | 24 | 21 | 22 | 19 |
| 2617 | Eukaryotic translation initiation factor 3 subunit A | IPI00029012 | -0.316 | 0.15825 | 41 | 46 | 40 | 42 |
| 2618 | Isoform 2 of Microtubule-actin cross-linking factor 1, isoforms 1/2/3/5 | IPI00256861 | -0.318 | 0.15821 | 10 | 8 | 4 | 11 |
| 2619 | EH domain-containing protein 4 | IPI00005578 | -0.318 | 0.15821 | 9 | 9 | 8 | 7 |
| 2620 | Vigilin | IPI00022228 | -0.323 | 0.15766 | 22 | 19 | 19 | 18 |
| 2621 | Isoform 1 of Catenin alpha-1 | IPI00215948 | -0.324 | 0.15766 | 9 | 8 | 6 | 8 |
| 2622 | Exportin-5 | IPI00640703 | -0.324 | 0.15766 | 8 | 9 | 7 | 7 |
| 2623 | Leucine-rich repeat-containing protein 47 | IPI00170935 | -0.324 | 0.15766 | 10 | 7 | 6 | 8 |
| 2624 | Isoform 1 of Apoptotic chromatin condensation inducer in the nucleus | IPI00007334 | -0.324 | 0.15766 | 11 | 6 | 7 | 7 |
| 2625 | 32 kDa protein | IPI00176692 | -0.324 | 0.15712 | 171 | 177 | 145 | 195 |
| 2626 | Translin | IPI00018768 | -0.328 | 0.15628 | 18 | 21 | 15 | 20 |
| 2627 | Putative uncharacterized protein DKFZp686L20222 | IPI00026689 | -0.329 | 0.15628 | 43 | 34 | 38 | 34 |
| 2628 | Histidyl-tRNA synthetase, cytoplasmic | IPI00021808 | -0.331 | 0.15624 | 19 | 19 | 19 | 15 |
| 2629 | SAP domain-containing ribonucleoprotein | IPI00014938 | -0.331 | 0.15599 | 11 | 5 | 8 | 5 |
| 2630 | Ribosome biogenesis protein BMS1 homolog | IPI00006099 | -0.331 | 0.15599 | 9 | 7 | 6 | 7 |
| 2631 | Inositol monophosphatase 1 | IPI00020906 | -0.331 | 0.15599 | 8 | 8 | 8 | 5 |
| 2632 | Isoform 1 of Actin-like protein 6A | IPI00003627 | -0.331 | 0.15599 | 6 | 10 | 6 | 7 |
| 2633 | Uncharacterized protein C2orf47, mitochondrial | IPI00291751 | -0.331 | 0.15599 | 7 | 9 | 7 | 6 |
| 2634 | Probable phosphoglycerate mutase 4 | IPI00374975 | -0.334 | 0.15490 | 24 | 13 | 22 | 11 |
| 2635 | Isoform 1 of Regulator of nonsense transcripts 1 | IPI00034049 | -0.334 | 0.15490 | 19 | 18 | 14 | 19 |
| 2636 | Isoform 1 of Putative ATP-dependent RNA helicase DHX30 | IPI00411733 | -0.339 | 0.15377 | 10 | 5 | 2 | 10 |
| 2637 | Ras-related protein Rab-18 | IPI00014577 | -0.339 | 0.15377 | 5 | 10 | 5 | 7 |
| 2638 | Actin-related protein 2/3 complex subunit 4 | IPI00554811 | -0.346 | 0.15281 | 37 | 29 | 36 | 25 |
| 2639 | Isoform 3 of Probable ATP-dependent RNA helicase DDX17 | IPI00651653 | -0.347 | 0.15268 | 16 | 17 | 16 | 13 |
| 2640 | Isoform 1 of Bcl-2-associated transcription factor 1 | IPI00006079 | -0.347 | 0.15268 | 9 | 5 | 6 | 5 |
| 2641 | Isoform 1 of Peripherin | IPI00013164 | -0.347 | 0.15268 | 8 | 6 | 5 | 6 |
| 2642 | DnaJ homolog subfamily B member 1 | IPI00015947 | -0.347 | 0.15268 | 7 | 7 | 5 | 6 |
| 2643 | Putative high mobility group protein B3-like-1 | IPI00006437 | -0.347 | 0.15268 | 9 | 5 | 7 | 4 |
| 2644 | Lamin-B2 | IPI00009771 | -0.351 | 0.15059 | 24 | 8 | 17 | 11 |
| 2645 | Ras-related protein Rab-5C | IPI00016339 | -0.354 | 0.14946 | 15 | 16 | 14 | 13 |
| 2646 | GTP-binding protein SAR1b | IPI00002149 | -0.356 | 0.14942 | 4 | 9 | 4 | 6 |
| 2647 | Isoform 2 of Nipped-B-like protein | IPI00026466 | -0.356 | 0.14942 | 7 | 6 | 7 | 3 |
| 2648 | Probable saccharopine dehydrogenase | IPI00329600 | -0.356 | 0.14942 | 8 | 5 | 6 | 4 |

| No. | Description | Accession number | STN[1] | p-Value[1] | Con_A[2] | Con_B[2] | RG3_A[2] | RG3_B[2] |
|---|---|---|---|---|---|---|---|---|
| 2649 | Peroxisomal membrane protein PMP34 | IPI00014440 | -0.356 | 0.14942 | 7 | 6 | 5 | 5 |
| 2650 | Putative rRNA methyltransferase 3 | IPI00217686 | -0.367 | 0.14620 | 8 | 4 | 5 | 4 |
| 2651 | Isoform 3 of Splicing factor, arginine/serine-rich 13A | IPI00009071 | -0.371 | 0.14503 | 12 | 15 | 10 | 13 |
| 2652 | Putative annexin A2-like protein | IPI00334627 | -0.372 | 0.14503 | 82 | 67 | 83 | 59 |
| 2653 | Sepiapterin reductase | IPI00017469 | -0.376 | 0.14495 | 10 | 16 | 9 | 13 |
| 2654 | Isoform 2 of Leucyl-cystinyl aminopeptidase | IPI00221240 | -0.379 | 0.14382 | 6 | 5 | 4 | 4 |
| 2655 | Isoform 1 of RNA-binding protein with serine-rich domain 1 | IPI00033561 | -0.379 | 0.14382 | 2 | 9 | 2 | 6 |
| 2656 | CTP synthase 2 | IPI00645702 | -0.379 | 0.14382 | 5 | 6 | 3 | 5 |
| 2657 | cDNA FLJ56425, highly similar to Very-long-chain specific acyl-CoAdehydrogenase, mitochondrial | IPI00028031 | -0.379 | 0.14240 | 27 | 23 | 24 | 21 |
| 2658 | Isoform 1 of Heterogeneous nuclear ribonucleoprotein R | IPI00012074 | -0.381 | 0.14202 | 49 | 37 | 37 | 43 |
| 2659 | Isoform 1 of Electron transfer flavoprotein subunit beta | IPI00004902 | -0.390 | 0.14098 | 27 | 19 | 27 | 14 |
| 2660 | Plastin-1 | IPI00032304 | -0.390 | 0.14098 | 24 | 22 | 21 | 20 |
| 2661 | DNA replication licensing factor MCM4 | IPI00018349 | -0.393 | 0.14068 | 24 | 21 | 19 | 21 |
| 2662 | DnaJ homolog subfamily A member 2 | IPI00032406 | -0.393 | 0.14056 | 10 | 13 | 8 | 11 |
| 2663 | Isoform 1 of Core-binding factor subunit beta | IPI00016746 | -0.393 | 0.14056 | 10 | 13 | 10 | 9 |
| 2664 | Ribosomal protein S6 kinase alpha-6 | IPI00007123 | -0.393 | 0.14056 | 7 | 3 | 3 | 4 |
| 2665 | Isoform 1 of Cleft lip and palate transmembrane protein 1-like proteir | IPI00151358 | -0.393 | 0.14056 | 3 | 7 | 2 | 5 |
| 2666 | Isoform 1 of Partner of Y14 and mago | IPI00305092 | -0.393 | 0.14056 | 4 | 6 | 3 | 4 |
| 2667 | MKI67 FHA domain-interacting nucleolar phosphoprotein | IPI00154590 | -0.393 | 0.14056 | 4 | 6 | 2 | 5 |
| 2668 | Isoform 1 of DNA-directed RNA polymerases I and III subunit RPAC1 | IPI00005179 | -0.393 | 0.14056 | 4 | 6 | 4 | 3 |
| 2669 | ATP-dependent RNA helicase SUPV3L1, mitochondrial | IPI00412404 | -0.393 | 0.14056 | 5 | 5 | 5 | 2 |
| 2670 | Pre-mRNA-processing factor 6 | IPI00305068 | -0.393 | 0.14056 | 4 | 6 | 3 | 4 |
| 2671 | Exosome complex exonuclease MTR3 | IPI00073602 | -0.393 | 0.14056 | 4 | 6 | 4 | 3 |
| 2672 | Nucleolar protein 16 | IPI00032849 | -0.393 | 0.14056 | 4 | 6 | 4 | 3 |
| 2673 | Moesin | IPI00219365 | -0.394 | 0.13817 | 46 | 32 | 39 | 33 |
| 2674 | Coatomer subunit beta | IPI00295851 | -0.396 | 0.13817 | 25 | 19 | 22 | 17 |
| 2675 | Isoform 1 of Glycerol-3-phosphate dehydrogenase, mitochondrial | IPI00017895 | -0.399 | 0.13780 | 11 | 11 | 6 | 12 |
| 2676 | Actin-related protein 2 | IPI00005159 | -0.402 | 0.13625 | 21 | 21 | 18 | 19 |
| 2677 | Ras-related protein Rab-7a | IPI00016342 | -0.402 | 0.13612 | 38 | 35 | 32 | 35 |
| 2678 | UMP-CMP kinase isoform a | IPI00219953 | -0.405 | 0.13608 | 23 | 18 | 23 | 13 |
| 2679 | Heterogeneous nuclear ribonucleoprotein A0 | IPI00011913 | -0.405 | 0.13608 | 22 | 19 | 20 | 16 |
| 2680 | Isoform 1 of 14-3-3 protein sigma | IPI00013890 | -0.405 | 0.13596 | 10 | 11 | 7 | 10 |
| 2681 | Isoform 2 of ATP-binding cassette sub-family F member 1 | IPI00013495 | -0.405 | 0.13596 | 10 | 11 | 9 | 8 |
| 2682 | 60S ribosomal protein L24 | IPI00306332 | -0.405 | 0.13596 | 12 | 9 | 9 | 8 |
| 2683 | TOB3 | IPI00045921 | -0.405 | 0.13596 | 13 | 8 | 12 | 5 |
| 2684 | GTP-binding protein SAR1a | IPI00015954 | -0.405 | 0.13596 | 10 | 11 | 6 | 11 |
| 2685 | ADP-ribosylation factor-like protein 2 | IPI00003326 | -0.405 | 0.13596 | 11 | 10 | 9 | 8 |
| 2686 | Tubulin-specific chaperone E | IPI00018402 | -0.409 | 0.13550 | 3 | 6 | 2 | 4 |
| 2687 | Centromere/kinetochore protein zw10 homolog | IPI00011631 | -0.409 | 0.13550 | 3 | 6 | 3 | 3 |
| 2688 | Osteoclast-stimulating factor 1 | IPI00414836 | -0.409 | 0.13550 | 7 | 0 | 3 | 3 |
| 2689 | Microsomal glutathione S-transferase 1 | IPI00021805 | -0.409 | 0.13550 | 3 | 6 | 0 | 4 |
| 2690 | Isoform 1 of RNA polymerase II-associated protein 3 | IPI00002408 | -0.409 | 0.13550 | 4 | 5 | 4 | 0 |
| 2691 | Isoform 1 of Putative deoxyribonuclease TATDN1 | IPI00012463 | -0.409 | 0.13550 | 3 | 6 | 3 | 3 |
| 2692 | DYNC1H1 protein | IPI00440177 | -0.412 | 0.13412 | 10 | 10 | 9 | 7 |
| 2693 | cDNA FLJ56285, highly similar to ADP-ribosylation factor-like protein 8B | IPI00018871 | -0.412 | 0.13412 | 10 | 10 | 7 | 9 |
| 2694 | cDNA FLJ78679, highly similar to Homo sapiens DEAD (Asp-Glu-Ala-Asp) box polypeptide 46 (DDX46) | IPI00329791 | -0.416 | 0.13261 | 19 | 19 | 15 | 18 |
| 2695 | 60S ribosomal protein L23a | IPI00021266 | -0.418 | 0.13223 | 36 | 29 | 39 | 20 |
| 2696 | Insulin-degrading enzyme | IPI00220373 | -0.428 | 0.13161 | 8 | 10 | 7 | 7 |
| 2697 | Superoxide dismutase [Mn], mitochondrial | IPI00022314 | -0.428 | 0.13161 | 6 | 12 | 7 | 7 |
| 2698 | Prefoldin subunit 5 | IPI00015361 | -0.429 | 0.12964 | 4 | 4 | 2 | 3 |
| 2699 | Isoform 1 of Neurochondrin | IPI00549543 | -0.429 | 0.12964 | 3 | 5 | 3 | 2 |
| 2700 | Pre-mRNA-splicing factor SPF27 | IPI00025178 | -0.429 | 0.12964 | 4 | 4 | 3 | 0 |
| 2701 | 28 kDa heat- and acid-stable phosphoprotein | IPI00013297 | -0.429 | 0.12964 | 5 | 3 | 3 | 0 |
| 2702 | Histone-binding protein RBBP4 | IPI00328319 | -0.432 | 0.12960 | 16 | 18 | 12 | 17 |
| 2703 | Isoform Beta of Heat shock protein 105 kDa | IPI00218993 | -0.432 | 0.12960 | 18 | 16 | 11 | 18 |
| 2704 | Eukaryotic translation initiation factor 6 | IPI00010105 | -0.436 | 0.12918 | 20 | 13 | 17 | 11 |
| 2705 | Thyroid hormone receptor-associated protein 3 | IPI00104050 | -0.437 | 0.12885 | 8 | 9 | 7 | 6 |
| 2706 | Protein FAM98B | IPI00167572 | -0.437 | 0.12885 | 5 | 12 | 5 | 8 |
| 2707 | NADPH--cytochrome P450 reductase | IPI00470467 | -0.437 | 0.12885 | 8 | 9 | 5 | 8 |
| 2708 | Isoform 1 of Extended synaptotagmin-1 | IPI00022143 | -0.439 | 0.12868 | 30 | 26 | 22 | 28 |
| 2709 | Leucine-rich repeat-containing protein 59 | IPI00396321 | -0.451 | 0.12529 | 10 | 20 | 11 | 14 |
| 2710 | Proliferation-associated protein 2G4 | IPI00299000 | -0.451 | 0.12504 | 47 | 36 | 43 | 33 |
| 2711 | L antigen family member 3 | IPI00032314 | -0.453 | 0.12492 | 0 | 5 | 2 | 0 |
| 2712 | Isoform 1 of Chromodomain-helicase-DNA-binding protein 1 | IPI00297851 | -0.453 | 0.12492 | 4 | 3 | 0 | 0 |
| 2713 | Isoform 1 of Nucleolar protein 6 | IPI00152890 | -0.453 | 0.12492 | 4 | 3 | 2 | 2 |
| 2714 | Isoform 1 of Glomulin | IPI00074604 | -0.453 | 0.12492 | 2 | 5 | 0 | 0 |
| 2715 | Thioredoxin domain-containing protein 5 | IPI00171438 | -0.453 | 0.12492 | 3 | 4 | 2 | 2 |
| 2716 | Mitotic spindle assembly checkpoint protein MAD2A | IPI00012369 | -0.453 | 0.12492 | 4 | 3 | 2 | 2 |
| 2717 | Splicing factor, arginine/serine-rich 4 | IPI00000015 | -0.453 | 0.12492 | 2 | 5 | 2 | 0 |
| 2718 | RNA-binding protein PNO1 | IPI00024524 | -0.453 | 0.12492 | 5 | 2 | 0 | 2 |
| 2719 | Isoform 1 of Caseinolytic peptidase B protein homolog | IPI00006615 | -0.453 | 0.12492 | 4 | 3 | 0 | 0 |
| 2720 | Serine/threonine-protein kinase 38-like | IPI00237011 | -0.453 | 0.12492 | 3 | 4 | 0 | 0 |
| 2721 | Protein FAM162A | IPI00023001 | -0.453 | 0.12492 | 0 | 5 | 2 | 2 |
| 2722 | NEDD8-activating enzyme E1 regulatory subunit | IPI00018968 | -0.453 | 0.12492 | 0 | 5 | 0 | 0 |
| 2723 | N-alpha-acetyltransferase 10, NatA catalytic subunit | IPI00013184 | -0.453 | 0.12492 | 5 | 0 | 0 | 0 |
| 2724 | Serine/threonine-protein kinase VRK1 | IPI00019640 | -0.453 | 0.12492 | 3 | 4 | 0 | 0 |
| 2725 | Isoform 1 of Protein phosphatase methylesterase 1 | IPI00007694 | -0.453 | 0.12492 | 0 | 5 | 2 | 0 |
| 2726 | Cell division protein kinase 7 | IPI00000685 | -0.453 | 0.12492 | 2 | 5 | 2 | 2 |
| 2727 | M-phase phosphoprotein 6 | IPI00016074 | -0.453 | 0.12492 | 3 | 4 | 2 | 0 |
| 2728 | Nucleolar protein 11 | IPI00303813 | -0.453 | 0.12492 | 4 | 3 | 0 | 0 |
| 2729 | Ubiquilin-4 | IPI00024502 | -0.453 | 0.12492 | 4 | 3 | 2 | 2 |
| 2730 | Nuclear receptor coactivator 5 | IPI00288941 | -0.453 | 0.12492 | 4 | 3 | 0 | 0 |
| 2731 | Serine palmitoyltransferase 1 | IPI00005745 | -0.453 | 0.12492 | 3 | 4 | 2 | 2 |
| 2732 | Neudesin | IPI00002525 | -0.453 | 0.12492 | 5 | 2 | 0 | 0 |
| 2733 | NADP-dependent malic enzyme | IPI00008215 | -0.453 | 0.12492 | 3 | 4 | 0 | 0 |
| 2734 | Isoform SRP55-1 of Splicing factor, arginine/serine-rich 6 | IPI00012345 | -0.453 | 0.12492 | 3 | 4 | 0 | 0 |
| 2735 | Protein SGT1 | IPI00027034 | -0.453 | 0.12492 | 3 | 4 | 0 | 0 |
| 2736 | Cell division protein kinase 2 | IPI00031681 | -0.453 | 0.12492 | 3 | 4 | 0 | 2 |
| 2737 | Transmembrane emp24 domain-containing protein 7 | IPI00032825 | -0.453 | 0.12492 | 4 | 3 | 2 | 0 |
| 2738 | Nucleolysin TIAR | IPI00005615 | -0.453 | 0.12492 | 5 | 0 | 0 | 0 |
| 2739 | Unhealthy ribosome biogenesis protein 2 homolog | IPI00028980 | -0.453 | 0.12492 | 5 | 0 | 0 | 0 |
| 2740 | Ras-related protein Rab-13 | IPI00016373 | -0.453 | 0.12492 | 0 | 5 | 0 | 0 |
| 2741 | cDNA FLJ59758, highly similar to S-methyl-5-thioadenosine phosphorylase | IPI00011876 | -0.453 | 0.12211 | 25 | 26 | 22 | 23 |
| 2742 | Structural maintenance of chromosomes protein 1A | IPI00291939 | -0.456 | 0.12199 | 25 | 25 | 21 | 23 |

| No. | Description | Accession number | STN[1] | p-Value[1] | Con_A[2] | Con_B[2] | RG3_A[2] | RG3_B[2] |
|---|---|---|---|---|---|---|---|---|
| 2743 | 87 kDa protein | IPI00220365 | -0.458 | 0.12170 | 8 | 7 | 6 | 5 |
| 2744 | Developmentally-regulated GTP-binding protein 1 | IPI00031836 | -0.458 | 0.12170 | 8 | 7 | 6 | 5 |
| 2745 | Isoform 5 of E3 ubiquitin-protein ligase UBR4 | IPI00180305 | -0.469 | 0.12069 | 21 | 25 | 11 | 29 |
| 2746 | Peptidyl-prolyl cis-trans isomerase D | IPI00003927 | -0.469 | 0.12032 | 8 | 6 | 5 | 5 |
| 2747 | 28S ribosomal protein S26, mitochondrial | IPI00006606 | -0.469 | 0.12032 | 6 | 8 | 5 | 5 |
| 2748 | Isoform 1 of Thyroid receptor-interacting protein 13 | IPI00003505 | -0.469 | 0.12032 | 5 | 9 | 4 | 6 |
| 2749 | 26S proteasome non-ATPase regulatory subunit 5 | IPI00002134 | -0.474 | 0.11722 | 12 | 14 | 11 | 10 |
| 2750 | Fructose-bisphosphate aldolase A | IPI00465439 | -0.477 | 0.11613 | 34 | 36 | 31 | 32 |
| 2751 | cDNA FLJ55988, highly similar to RNA-binding protein Luc7-like 2 | IPI00006932 | -0.483 | 0.11580 | 10 | 3 | 7 | 2 |
| 2752 | Isoform 1 of Elongation factor G, mitochondrial | IPI00154473 | -0.483 | 0.11580 | 6 | 7 | 3 | 6 |
| 2753 | DNA topoisomerase 1 | IPI00413611 | -0.483 | 0.11580 | 7 | 6 | 5 | 4 |
| 2754 | sorting nexin-1 isoform c | IPI00183274 | -0.483 | 0.11580 | 6 | 7 | 4 | 5 |
| 2755 | Isoform 1 of Uridine 5'-monophosphate synthase | IPI00003923 | -0.483 | 0.11580 | 6 | 7 | 3 | 6 |
| 2756 | Ras-related protein Rab-21 | IPI00007755 | -0.483 | 0.11580 | 8 | 5 | 4 | 5 |
| 2757 | 116 kDa U5 small nuclear ribonucleoprotein component | IPI00003519 | -0.496 | 0.11388 | 43 | 51 | 41 | 45 |
| 2758 | Isoform 2 of Titin | IPI00023283 | -0.498 | 0.11371 | 4 | 8 | 5 | 3 |
| 2759 | H/ACA ribonucleoprotein complex subunit 4 | IPI00221394 | -0.498 | 0.11371 | 6 | 6 | 6 | 2 |
| 2760 | Isoform 2 of 39S ribosomal protein L39, mitochondrial | IPI00084571 | -0.498 | 0.11371 | 6 | 6 | 5 | 3 |
| 2761 | Isoform 1 of 2-oxoglutarate and iron-dependent oxygenase domain-containing protein 1 | IPI00170429 | -0.498 | 0.11371 | 7 | 5 | 0 | 6 |
| 2762 | aldehyde dehydrogenase 9A1 | IPI00479877 | -0.498 | 0.11371 | 6 | 6 | 6 | 0 |
| 2763 | Isoform 1 of Myosin-Ib | IPI00376344 | -0.503 | 0.10936 | 9 | 13 | 6 | 11 |
| 2764 | Isoform 1 of Enoyl-CoA hydratase domain-containing protein 1 | IPI00302688 | -0.503 | 0.10936 | 10 | 12 | 6 | 11 |
| 2765 | Isoform 1 of Cytoplasmic FMR1-interacting protein 1 | IPI00644231 | -0.503 | 0.10936 | 10 | 12 | 6 | 11 |
| 2766 | 40S ribosomal protein S19 | IPI00215780 | -0.506 | 0.10915 | 21 | 16 | 14 | 17 |
| 2767 | cDNA FLJ35809 fis, clone TESTI2006016, highly similar to Eukaryotic translation initiation factor 3 | IPI00647650 | -0.515 | 0.10844 | 7 | 4 | 5 | 2 |
| 2768 | NAD-dependent malic enzyme, mitochondrial | IPI00011201 | -0.515 | 0.10844 | 6 | 5 | 3 | 4 |
| 2769 | Isoform 1 of Dephospho-CoA kinase domain-containing protein | IPI00291417 | -0.515 | 0.10844 | 8 | 3 | 5 | 0 |
| 2770 | cDNA FLJ56825, highly similar to WD repeat protein 57 | IPI00006723 | -0.515 | 0.10844 | 5 | 6 | 3 | 4 |
| 2771 | Heterogeneous nuclear ribonucleoprotein H2 | IPI00026230 | -0.530 | 0.10681 | 9 | 10 | 5 | 9 |
| 2772 | ERO1-like protein alpha | IPI00386755 | -0.530 | 0.10681 | 10 | 9 | 8 | 6 |
| 2773 | Transmembrane emp24 domain-containing protein 9 | IPI00023542 | -0.536 | 0.10580 | 5 | 5 | 0 | 4 |
| 2774 | Golgi-specific brefeldin A-resistance guanine nucleotide exchange factor 1 | IPI00021954 | -0.536 | 0.10580 | 4 | 6 | 3 | 3 |
| 2775 | COMM domain-containing protein 2 | IPI00456048 | -0.536 | 0.10580 | 6 | 4 | 0 | 4 |
| 2776 | Isoform CNPI of 2',3'-cyclic-nucleotide 3'-phosphodiesterase | IPI00220993 | -0.536 | 0.10580 | 6 | 4 | 3 | 3 |
| 2777 | Heat shock protein beta-11 | IPI00098827 | -0.536 | 0.10580 | 5 | 5 | 3 | 3 |
| 2778 | 5'-nucleotidase domain-containing protein 1 | IPI00177965 | -0.536 | 0.10580 | 3 | 7 | 4 | 2 |
| 2779 | BRO1 domain-containing protein BROX | IPI00065500 | -0.536 | 0.10580 | 4 | 6 | 4 | 2 |
| 2780 | Splicing factor 3A subunit 3 | IPI00029764 | -0.538 | 0.10217 | 14 | 17 | 11 | 14 |
| 2781 | 28S ribosomal protein S29, mitochondrial | IPI00018120 | -0.541 | 0.10208 | 6 | 12 | 5 | 8 |
| 2782 | Proliferating cell nuclear antigen | IPI00021700 | -0.542 | 0.10141 | 69 | 73 | 62 | 70 |
| 2783 | Nucleosome assembly protein 1-like 1 | IPI00023860 | -0.550 | 0.10066 | 25 | 21 | 18 | 21 |
| 2784 | TDP43 | IPI00025815 | -0.553 | 0.10020 | 9 | 8 | 7 | 5 |
| 2785 | cDNA FLJ54775, highly similar to Syntaxin-binding protein 2 | IPI00019971 | -0.560 | 0.09911 | 5 | 4 | 2 | 3 |
| 2786 | Isoform 1 of Drebrin | IPI00003406 | -0.560 | 0.09911 | 6 | 3 | 3 | 2 |
| 2787 | ATP-dependent RNA helicase DDX50 | IPI00031554 | -0.560 | 0.09911 | 2 | 7 | 0 | 3 |
| 2788 | Adenylosuccinate synthetase isozyme 2 | IPI00026833 | -0.580 | 0.09594 | 4 | 11 | 5 | 5 |
| 2789 | Mitochondrial import receptor subunit TOM34 | IPI00009946 | -0.580 | 0.09594 | 7 | 8 | 8 | 2 |
| 2790 | Isoform 1 of Mitotic checkpoint protein BUB3 | IPI00013468 | -0.581 | 0.09568 | 13 | 12 | 8 | 11 |
| 2791 | 14-3-3 protein theta | IPI00018146 | -0.582 | 0.09552 | 33 | 25 | 25 | 25 |
| 2792 | Isoform 1 of Exportin-2 | IPI00022744 | -0.587 | 0.09502 | 106 | 90 | 84 | 100 |
| 2793 | tropomyosin alpha-3 chain isoform 1 | IPI00183968 | -0.590 | 0.09502 | 14 | 10 | 10 | 8 |
| 2794 | U2 small nuclear ribonucleoprotein B'' | IPI00029267 | -0.590 | 0.09405 | 0 | 6 | 0 | 0 |
| 2795 | Probable ATP-dependent RNA helicase DDX10 | IPI00297900 | -0.590 | 0.09405 | 4 | 4 | 2 | 2 |
| 2796 | Isoform 2 of Calumenin | IPI00045396 | -0.590 | 0.09405 | 3 | 5 | 2 | 0 |
| 2797 | Uncharacterized protein C18orf19 | IPI00290799 | -0.590 | 0.09405 | 6 | 0 | 0 | 0 |
| 2798 | Ubiquitin carboxyl-terminal hydrolase 11 | IPI00184533 | -0.590 | 0.09405 | 4 | 4 | 0 | 0 |
| 2799 | Lanosterol synthase | IPI00009747 | -0.590 | 0.09405 | 3 | 5 | 2 | 2 |
| 2800 | Isoform 1 of HAUS augmin-like complex subunit 2 | IPI00018198 | -0.590 | 0.09405 | 3 | 5 | 0 | 0 |
| 2801 | ESF1 homolog | IPI00024167 | -0.590 | 0.09405 | 6 | 0 | 2 | 0 |
| 2802 | 40S ribosomal protein S11 | IPI00025091 | -0.596 | 0.08623 | 9 | 5 | 5 | 4 |
| 2803 | U4/U6.U5 tri-snRNP-associated protein 2 | IPI00419844 | -0.596 | 0.08623 | 7 | 7 | 2 | 7 |
| 2804 | Isoform Long of Tight junction protein ZO-1 | IPI00216219 | -0.596 | 0.08623 | 8 | 6 | 4 | 5 |
| 2805 | NADH dehydrogenase [ubiquinone] iron-sulfur protein 3, mitochondrial | IPI00025796 | -0.599 | 0.08544 | 19 | 17 | 13 | 16 |
| 2806 | Glucosamine 6-phosphate N-acetyltransferase | IPI00061525 | -0.599 | 0.08531 | 12 | 11 | 8 | 9 |
| 2807 | Isoform Short of Heterogeneous nuclear ribonucleoprotein U | IPI00479217 | -0.608 | 0.08456 | 93 | 83 | 82 | 82 |
| 2808 | Isoform 1 of Transformation/transcription domain-associated protein | IPI00069084 | -0.609 | 0.08456 | 11 | 11 | 7 | 9 |
| 2809 | Isoform Long of Delta-1-pyrroline-5-carboxylate synthase | IPI00008982 | -0.611 | 0.08351 | 53 | 46 | 42 | 47 |
| 2810 | 60S ribosomal protein L4 | IPI00003918 | -0.613 | 0.08351 | 29 | 21 | 27 | 15 |
| 2811 | Isoform 2 of Filamin-A | IPI00302592 | -0.614 | 0.08310 | 142 | 134 | 142 | 120 |
| 2812 | Isoform 5 of Protein polybromo-1 | IPI00023097 | -0.614 | 0.08310 | 8 | 5 | 4 | 4 |
| 2813 | Heat shock 70 kDa protein 1A/1B | IPI00304925 | -0.626 | 0.08151 | 33 | 34 | 29 | 29 |
| 2814 | Isoform 1 of Heterogeneous nuclear ribonucleoprotein D0 | IPI00028888 | -0.626 | 0.08151 | 32 | 35 | 32 | 26 |
| 2815 | Isoform 3 of Chitinase domain-containing protein 1 | IPI00045536 | -0.631 | 0.08121 | 8 | 12 | 6 | 8 |
| 2816 | DNA-directed RNA polymerase I subunit RPA1 | IPI00031960 | -0.635 | 0.08013 | 7 | 5 | 3 | 4 |
| 2817 | Guanine nucleotide-binding protein G(I)/G(S)/G(T) subunit beta-2 | IPI00003348 | -0.635 | 0.08013 | 6 | 6 | 5 | 2 |
| 2818 | Dihydrofolate reductase | IPI00030357 | -0.635 | 0.08013 | 2 | 10 | 2 | 5 |
| 2819 | UPF0568 protein C14orf166 | IPI00006980 | -0.636 | 0.08000 | 25 | 20 | 21 | 16 |
| 2820 | Threonyl-tRNA synthetase, cytoplasmic | IPI00329633 | -0.639 | 0.08000 | 32 | 31 | 25 | 29 |
| 2821 | Isoform SM-B' of Small nuclear ribonucleoprotein-associated proteins B and B' | IPI00027285 | -0.641 | 0.07946 | 24 | 20 | 18 | 18 |
| 2822 | Isoform 1 of Calcyclin-binding protein | IPI00395627 | -0.642 | 0.07887 | 31 | 31 | 26 | 27 |
| 2823 | 40S ribosomal protein S4, X isoform | IPI00217030 | -0.643 | 0.07887 | 47 | 38 | 38 | 37 |
| 2824 | cDNA FLJ54492, highly similar to Eukaryotic translation initiation factor 4B | IPI00012079 | -0.647 | 0.07866 | 13 | 16 | 12 | 10 |
| 2825 | Guanine nucleotide-binding protein subunit beta-2-like 1 | IPI00848226 | -0.649 | 0.07858 | 72 | 72 | 58 | 74 |
| 2826 | Isoform 1 of 40S ribosomal protein S24 | IPI00029750 | -0.651 | 0.07829 | 53 | 29 | 42 | 30 |
| 2827 | Cell division protein kinase 6 | IPI00023529 | -0.657 | 0.07804 | 10 | 8 | 8 | 4 |
| 2828 | Ras-related protein Rab-6B | IPI00016891 | -0.657 | 0.07804 | 6 | 12 | 5 | 7 |
| 2829 | Aminoacyl tRNA synthase complex-interacting multifunctional protein 1 | IPI00006252 | -0.659 | 0.07737 | 5 | 6 | 3 | 3 |
| 2830 | ATP-dependent RNA helicase DDX18 | IPI00301323 | -0.663 | 0.07707 | 26 | 14 | 18 | 14 |
| 2831 | Isoform 1 of Heterogeneous nuclear ribonucleoprotein Q | IPI00018140 | -0.667 | 0.07666 | 55 | 47 | 48 | 43 |
| 2832 | Phosphoserine aminotransferase | IPI00001734 | -0.669 | 0.07649 | 18 | 21 | 14 | 17 |
| 2833 | Thimet oligopeptidase | IPI00549189 | -0.672 | 0.07645 | 9 | 8 | 4 | 7 |
| 2834 | Alkyldihydroxyacetonephosphate synthase, peroxisomal | IPI00010349 | -0.672 | 0.07645 | 7 | 10 | 5 | 6 |
| 2835 | Isoform 1 of Heterogeneous nuclear ribonucleoprotein A3 | IPI00419373 | -0.674 | 0.07590 | 28 | 26 | 23 | 22 |
| 2836 | Eukaryotic initiation factor 4A-III | IPI00009328 | -0.678 | 0.07544 | 27 | 26 | 25 | 19 |

| No. | Description | Accession number | STN[1] | p-Value[1] | Con_A[2] | Con_B[2] | RG3_A[2] | RG3_B[2] |
|---|---|---|---|---|---|---|---|---|
| 2837 | Putative uncharacterized protein NOP2 | IPI00294891 | -0.683 | 0.07544 | 16 | 9 | 11 | 7 |
| 2838 | Heat shock protein beta-1 | IPI00025512 | -0.683 | 0.07528 | 35 | 36 | 30 | 31 |
| 2839 | Exportin-1 | IPI00298961 | -0.686 | 0.07498 | 58 | 64 | 43 | 67 |
| 2840 | Isoform 1 of Symplekin | IPI00023344 | -0.687 | 0.07490 | 8 | 2 | 3 | 2 |
| 2841 | Transmembrane emp24 domain-containing protein 5 | IPI00294472 | -0.687 | 0.07490 | 5 | 5 | 2 | 3 |
| 2842 | Glutamate-rich WD repeat-containing protein 1 | IPI00027831 | -0.687 | 0.07490 | 0 | 8 | 0 | 3 |
| 2843 | SNW domain-containing protein 1 | IPI00013830 | -0.687 | 0.07490 | 5 | 5 | 3 | 2 |
| 2844 | Isoform 1 of Ras-related protein Rab-6A | IPI00023526 | -0.694 | 0.07272 | 11 | 13 | 9 | 8 |
| 2845 | Heterogeneous nuclear ribonucleoprotein L | IPI00027834 | -0.701 | 0.07243 | 52 | 36 | 50 | 27 |
| 2846 | Ras-related protein Rab-1B | IPI00008964 | -0.707 | 0.07143 | 9 | 6 | 4 | 5 |
| 2847 | Coatomer subunit delta variant 2 | IPI00298520 | -0.707 | 0.07143 | 4 | 11 | 3 | 6 |
| 2848 | Isoform GTBP-alt of DNA mismatch repair protein Msh6 | IPI00106847 | -0.713 | 0.07114 | 23 | 23 | 18 | 19 |
| 2849 | Nucleolar pre-ribosomal-associated protein 1 | IPI00297241 | -0.722 | 0.07009 | 6 | 3 | 0 | 0 |
| 2850 | cDNA FLJ50992, highly similar to Coronin-1C | IPI00798401 | -0.722 | 0.07009 | 0 | 7 | 0 | 2 |
| 2851 | Isoform 1 of PC4 and SFRS1-interacting protein | IPI00028122 | -0.722 | 0.07009 | 3 | 6 | 0 | 2 |
| 2852 | Heme-binding protein 1 | IPI00148063 | -0.722 | 0.07009 | 2 | 7 | 0 | 2 |
| 2853 | DnaJ homolog subfamily C member 9 | IPI00154975 | -0.722 | 0.07009 | 4 | 5 | 0 | 0 |
| 2854 | Putative uncharacterized protein KIAA0664 | IPI00024425 | -0.726 | 0.06904 | 16 | 15 | 12 | 11 |
| 2855 | Aladin | IPI00024143 | -0.728 | 0.06896 | 5 | 9 | 4 | 4 |
| 2856 | Isoform 1 of Translocon-associated protein subunit alpha | IPI00301021 | -0.728 | 0.06896 | 6 | 8 | 3 | 5 |
| 2857 | HEAT repeat-containing protein 1 | IPI00024279 | -0.736 | 0.06595 | 22 | 20 | 10 | 23 |
| 2858 | Thymidylate synthetase, isoform CRA_a | IPI00103732 | -0.751 | 0.06490 | 7 | 6 | 4 | 3 |
| 2859 | Nuclear pore complex protein Nup107 | IPI00028005 | -0.751 | 0.06490 | 5 | 8 | 0 | 5 |
| 2860 | Isoform 2 of DNA replication licensing factor MCM7 | IPI00219740 | -0.754 | 0.06490 | 15 | 13 | 9 | 11 |
| 2861 | Transmembrane emp24 domain-containing protein 10 | IPI00028055 | -0.757 | 0.06436 | 35 | 35 | 31 | 28 |
| 2862 | DEAD (Asp-Glu-Ala-Asp) box polypeptide 39 transcript variant | IPI00062206 | -0.760 | 0.06436 | 9 | 10 | 6 | 6 |
| 2863 | Ribonucleoside-diphosphate reductase large subunit | IPI00013871 | -0.760 | 0.06436 | 11 | 8 | 5 | 7 |
| 2864 | Isoform 3 of Spectrin alpha chain, brain | IPI00843765 | -0.765 | 0.06403 | 87 | 87 | 84 | 75 |
| 2865 | Aldehyde dehydrogenase, mitochondrial | IPI00006663 | -0.769 | 0.06382 | 30 | 37 | 33 | 23 |
| 2866 | Zinc finger protein ZPR1 | IPI00025244 | -0.779 | 0.06252 | 6 | 6 | 3 | 3 |
| 2867 | Endoplasmic reticulum resident protein 29 | IPI00024911 | -0.801 | 0.05968 | 18 | 27 | 17 | 18 |
| 2868 | Isoform 1 of Transformer-2 protein homolog beta | IPI00301503 | -0.804 | 0.05955 | 20 | 13 | 18 | 6 |
| 2869 | Isoform 1 of Uridine-cytidine kinase 2 | IPI00065671 | -0.811 | 0.05901 | 5 | 6 | 2 | 3 |
| 2870 | Thymidine kinase, cytosolic | IPI00299214 | -0.811 | 0.05901 | 4 | 7 | 0 | 3 |
| 2871 | Isoform 2 of Golgi apparatus protein 1 | IPI00414717 | -0.811 | 0.05901 | 6 | 5 | 0 | 3 |
| 2872 | Isoform 1 of Chromosome-associated kinesin KIF4A | IPI00178150 | -0.811 | 0.05901 | 4 | 7 | 2 | 3 |
| 2873 | ADP-ribosylation factor 5 | IPI00215919 | -0.811 | 0.05901 | 9 | 2 | 3 | 0 |
| 2874 | Isoform Beta of Nucleolar and coiled-body phosphoprotein 1 | IPI00216654 | -0.816 | 0.05867 | 7 | 9 | 5 | 4 |
| 2875 | Peroxiredoxin-1 | IPI00000874 | -0.822 | 0.05826 | 81 | 59 | 70 | 55 |
| 2876 | Isoform 1 of Spectrin beta chain, brain 2 | IPI00012645 | -0.828 | 0.05800 | 13 | 9 | 9 | 5 |
| 2877 | Isoform 2 of Structural maintenance of chromosomes protein 4 | IPI00328298 | -0.829 | 0.05742 | 25 | 29 | 19 | 24 |
| 2878 | Isoform 1 of Cytoskeleton-associated protein 5 | IPI00028275 | -0.836 | 0.05725 | 21 | 19 | 9 | 21 |
| 2879 | SWI/SNF-related matrix-associated actin-dependent regulator of chromatin subfamily A member 5 | IPI00297211 | -0.844 | 0.05679 | 12 | 9 | 6 | 7 |
| 2880 | Isoform 1 of RNA-binding protein 25 | IPI00004273 | -0.844 | 0.05679 | 12 | 9 | 7 | 6 |
| 2881 | 32 kDa protein | IPI00399077 | -0.851 | 0.05667 | 5 | 5 | 0 | 0 |
| 2882 | Negative elongation factor B | IPI00103483 | -0.851 | 0.05667 | 4 | 6 | 0 | 2 |
| 2883 | Isoform 1 of Crooked neck-like protein 1 | IPI00177437 | -0.851 | 0.05667 | 5 | 5 | 0 | 0 |
| 2884 | RcDNAJ9 (Fragment) | IPI00014718 | -0.851 | 0.05667 | 4 | 6 | 0 | 0 |
| 2885 | cDNA FLJ34068 fis, clone FCBBF3001918 | IPI00168184 | -0.852 | 0.05248 | 21 | 29 | 18 | 21 |
| 2886 | Protein RRP5 homolog | IPI00400922 | -0.861 | 0.05228 | 20 | 17 | 11 | 16 |
| 2887 | Replication protein A 70 kDa DNA-binding subunit | IPI00020127 | -0.867 | 0.05131 | 12 | 15 | 6 | 12 |
| 2888 | Ras GTPase-activating protein-binding protein 1 | IPI00012442 | -0.874 | 0.05089 | 28 | 32 | 23 | 25 |
| 2889 | Vimentin | IPI00418471 | -0.878 | 0.05048 | 37 | 38 | 32 | 30 |
| 2890 | 60S acidic ribosomal protein P2 | IPI00008529 | -0.879 | 0.05048 | 16 | 19 | 14 | 11 |
| 2891 | 40S ribosomal protein S9 | IPI00221088 | -0.880 | 0.05039 | 30 | 29 | 26 | 21 |
| 2892 | CTP synthase 1 | IPI00290142 | -0.902 | 0.04813 | 34 | 21 | 19 | 24 |
| 2893 | Isoform 1 of UDP-glucose:glycoprotein glucosyltransferase 1 | IPI00024466 | -0.908 | 0.04797 | 42 | 43 | 36 | 35 |
| 2894 | Protein phosphatase 1G | IPI00006167 | -0.925 | 0.04688 | 10 | 13 | 7 | 7 |
| 2895 | Periodic tryptophan protein 2 homolog | IPI00300078 | -0.932 | 0.04675 | 13 | 17 | 8 | 12 |
| 2896 | 40S ribosomal protein S3 | IPI00011253 | -0.945 | 0.04563 | 149 | 109 | 139 | 98 |
| 2897 | Keratin, type I cytoskeletal 18 | IPI00554788 | -0.948 | 0.04471 | 375 | 319 | 334 | 331 |
| 2898 | Matrin-3 | IPI00017297 | -0.968 | 0.04349 | 52 | 53 | 48 | 41 |
| 2899 | epiplakin | IPI00010951 | -0.975 | 0.04337 | 5 | 6 | 2 | 2 |
| 2900 | Isoform 1 of ATPase family AAA domain-containing protein 2 | IPI00170548 | -0.975 | 0.04337 | 6 | 5 | 0 | 0 |
| 2901 | Isoform 2 of N-alpha-acetyltransferase 15, NatA auxiliary subunit | IPI00032158 | -0.975 | 0.04337 | 5 | 6 | 2 | 2 |
| 2902 | Isoform 1 of Tyrosine-protein kinase BAZ1B | IPI00069817 | -0.981 | 0.04291 | 9 | 11 | 6 | 5 |
| 2903 | Ras-related protein Rap-1b | IPI00015148 | -0.994 | 0.04228 | 26 | 27 | 22 | 18 |
| 2904 | Eukaryotic translation initiation factor 3 subunit C | IPI00016910 | -0.995 | 0.04224 | 18 | 15 | 10 | 12 |
| 2905 | Protein S100-A6 | IPI00027463 | -1.003 | 0.04186 | 13 | 6 | 4 | 6 |
| 2906 | Nestin | IPI00010800 | -1.007 | 0.04174 | 15 | 17 | 9 | 12 |
| 2907 | SUMO-activating enzyme subunit 2 | IPI00023234 | -1.008 | 0.04161 | 22 | 29 | 22 | 16 |
| 2908 | protein ELYS | IPI00170594 | -1.010 | 0.04161 | 7 | 7 | 0 | 4 |
| 2909 | DCN1-like protein 5 | IPI00165361 | -1.033 | 0.03948 | 14 | 16 | 7 | 12 |
| 2910 | Eukaryotic translation initiation factor 3 subunit E | IPI00013068 | -1.039 | 0.03939 | 25 | 22 | 15 | 19 |
| 2911 | Isoform 1 of Aldehyde dehydrogenase family 16 member A1 | IPI00217920 | -1.049 | 0.03919 | 4 | 9 | 0 | 3 |
| 2912 | Neutral amino acid transporter B(0) | IPI00019472 | -1.050 | 0.03910 | 77 | 85 | 69 | 73 |
| 2913 | Chromobox protein homolog 5 | IPI00024662 | -1.087 | 0.03793 | 9 | 7 | 4 | 3 |
| 2914 | Activator of 90 kDa heat shock protein ATPase homolog 1 | IPI00030706 | -1.094 | 0.03793 | 18 | 23 | 9 | 19 |
| 2915 | Isoform 2 of Guanine nucleotide-binding protein-like 3 | IPI00003886 | -1.097 | 0.03781 | 6 | 6 | 2 | 2 |
| 2916 | Isoform 1 of Heterogeneous nuclear ribonucleoprotein M | IPI00171903 | -1.103 | 0.03647 | 94 | 93 | 88 | 77 |
| 2917 | Nucleolar protein 58 | IPI00006379 | -1.104 | 0.03647 | 31 | 29 | 24 | 21 |
| 2918 | Isoform Long of Eukaryotic translation initiation factor 4H | IPI00014263 | -1.115 | 0.03580 | 26 | 22 | 18 | 16 |
| 2919 | Nuclear pore complex protein Nup205 | IPI00783781 | -1.122 | 0.03546 | 41 | 28 | 26 | 27 |
| 2920 | Ribose-phosphate pyrophosphokinase 3 | IPI00218371 | -1.123 | 0.03546 | 9 | 6 | 0 | 4 |
| 2921 | 60S ribosomal protein L7-like 1 | IPI00456940 | -1.123 | 0.03546 | 7 | 8 | 0 | 4 |
| 2922 | Isoform 1 of Polyadenylate-binding protein 1 | IPI00008524 | -1.124 | 0.03546 | 48 | 34 | 32 | 33 |
| 2923 | Chromobox protein homolog 1 | IPI00010320 | -1.134 | 0.03517 | 10 | 14 | 6 | 7 |
| 2924 | Isoform 1 of Eukaryotic translation initiation factor 3 subunit B | IPI00396370 | -1.134 | 0.03517 | 39 | 28 | 29 | 22 |
| 2925 | Isoform 1 of Splicing factor 3B subunit 3 | IPI00300371 | -1.138 | 0.03488 | 115 | 107 | 90 | 108 |
| 2926 | Isoform E of Eukaryotic translation initiation factor 4 gamma 1 | IPI00386533 | -1.164 | 0.03341 | 14 | 21 | 11 | 11 |
| 2927 | Isoform 3 of Ribosome-binding protein 1 | IPI00215743 | -1.169 | 0.03333 | 12 | 16 | 8 | 8 |
| 2928 | DNA replication licensing factor MCM6 | IPI00031517 | -1.204 | 0.03153 | 31 | 26 | 23 | 18 |
| 2929 | Glycyl-tRNA synthetase | IPI00783097 | -1.209 | 0.03128 | 39 | 28 | 23 | 27 |
| 2930 | Isoform 1 of Cirhin | IPI00239815 | -1.216 | 0.03128 | 5 | 8 | 2 | 2 |
| 2931 | Isoform 1 of SET domain-containing protein 3 | IPI00165026 | -1.216 | 0.03128 | 5 | 8 | 0 | 0 |

| No. | Description | Accession number | STN[1] | p-Value[1] | Con_A[2] | Con_B[2] | RG3_A[2] | RG3_B[2] |
|---|---|---|---|---|---|---|---|---|
| 2932 | Ras-related protein Rab-11B | IPI00020436 | -1.230 | 0.03065 | 22 | 23 | 17 | 13 |
| 2933 | Probable ATP-dependent RNA helicase DDX5 | IPI00017617 | -1.257 | 0.02919 | 70 | 41 | 48 | 42 |
| 2934 | Calcium-binding mitochondrial carrier protein Aralar2 | IPI00007084 | -1.259 | 0.02919 | 26 | 34 | 20 | 23 |
| 2935 | Importin subunit alpha-2 | IPI00002214 | -1.278 | 0.02860 | 20 | 14 | 9 | 11 |
| 2936 | Small subunit processome component 20 homolog | IPI00004970 | -1.289 | 0.02806 | 20 | 20 | 11 | 14 |
| 2937 | Proteasome subunit alpha type-6 | IPI00029623 | -1.311 | 0.02693 | 26 | 28 | 21 | 16 |
| 2938 | Isoform 2 of 4F2 cell-surface antigen heavy chain | IPI00027493 | -1.324 | 0.02668 | 33 | 39 | 25 | 28 |
| 2939 | Core histone macro-H2A.2 | IPI00220994 | -1.332 | 0.02601 | 6 | 8 | 2 | 0 |
| 2940 | Short heat shock protein 60 Hsp60s2 | IPI00076042 | -1.355 | 0.02430 | 58 | 32 | 51 | 18 |
| 2941 | DNA replication licensing factor MCM2 | IPI00184330 | -1.359 | 0.02392 | 33 | 34 | 26 | 22 |
| 2942 | Condensin complex subunit 1 | IPI00299524 | -1.375 | 0.02346 | 31 | 34 | 26 | 20 |
| 2943 | 40S ribosomal protein S23 | IPI00218606 | -1.389 | 0.02300 | 14 | 0 | 3 | 2 |
| 2944 | DnaJ homolog subfamily A member 1 | IPI00012535 | -1.398 | 0.02279 | 20 | 19 | 11 | 12 |
| 2945 | Beta-actin-like protein 2 | IPI00003269 | -1.400 | 0.02267 | 51 | 56 | 40 | 44 |
| 2946 | FACT complex subunit SSRP1 | IPI00005154 | -1.401 | 0.02250 | 13 | 10 | 4 | 6 |
| 2947 | Ezrin | IPI00843975 | -1.436 | 0.02179 | 38 | 29 | 30 | 17 |
| 2948 | E3 SUMO-protein ligase RanBP2 | IPI00221325 | -1.449 | 0.02095 | 22 | 20 | 8 | 17 |
| 2949 | Eukaryotic initiation factor 4A-I | IPI00025491 | -1.495 | 0.02003 | 138 | 98 | 115 | 89 |
| 2950 | Heat shock protein 75 kDa, mitochondrial | IPI00030275 | -1.507 | 0.01966 | 41 | 36 | 27 | 28 |
| 2951 | Asparagine synthetase [glutamine-hydrolyzing] | IPI00554777 | -1.530 | 0.01890 | 11 | 12 | 4 | 5 |
| 2952 | Cathepsin D | IPI00011229 | -1.543 | 0.01865 | 15 | 27 | 7 | 17 |
| 2953 | HSPA5 protein | IPI00003362 | -1.554 | 0.01832 | 107 | 67 | 81 | 63 |
| 2954 | Sodium/potassium-transporting ATPase subunit alpha-2 | IPI00003021 | -1.562 | 0.01794 | 31 | 23 | 18 | 16 |
| 2955 | Histone H1.5 | IPI00217468 | -1.674 | 0.01526 | 27 | 13 | 12 | 9 |
| 2956 | Isoform 1 of Nucleolar RNA helicase 2 | IPI00015953 | -1.717 | 0.01397 | 62 | 57 | 42 | 48 |
| 2957 | N-acetyltransferase 10 | IPI00300127 | -1.720 | 0.01397 | 25 | 30 | 11 | 22 |
| 2958 | Isoform 1 of Myosin-9 | IPI00019502 | -1.729 | 0.01388 | 379 | 375 | 377 | 323 |
| 2959 | Isoform 1 of DNA (cytosine-5)-methyltransferase 1 | IPI00031519 | -1.795 | 0.01225 | 20 | 19 | 10 | 9 |
| 2960 | Isoform 1 of Heat shock cognate 71 kDa protein | IPI00003865 | -1.864 | 0.01108 | 269 | 150 | 209 | 162 |
| 2961 | Transferrin receptor protein 1 | IPI00022462 | -1.865 | 0.01104 | 27 | 24 | 15 | 13 |
| 2962 | Isoform Beta-2 of DNA topoisomerase 2-beta | IPI00027280 | -1.910 | 0.01037 | 15 | 12 | 2 | 7 |
| 2963 | ubiquitin and ribosomal protein S27a precursor | IPI00179330 | -1.937 | 0.01008 | 73 | 62 | 48 | 53 |
| 2964 | Nucleolar protein 56 | IPI00411937 | -1.953 | 0.00987 | 23 | 23 | 12 | 11 |
| 2965 | Midasin | IPI00167941 | -1.981 | 0.00953 | 23 | 17 | 3 | 15 |
| 2966 | Protein disulfide-isomerase A4 | IPI00009904 | -2.030 | 0.00907 | 54 | 65 | 42 | 43 |
| 2967 | 60S ribosomal protein L13 | IPI00465361 | -2.094 | 0.00874 | 13 | 8 | 0 | 0 |
| 2968 | Isoform 1 of Importin-5 | IPI00793443 | -2.110 | 0.00849 | 42 | 49 | 30 | 29 |
| 2969 | Importin-7 | IPI00007402 | -2.173 | 0.00807 | 53 | 55 | 33 | 40 |
| 2970 | Kinesin-like protein KIF11 | IPI00305289 | -2.197 | 0.00807 | 11 | 11 | 0 | 0 |
| 2971 | ATP-dependent RNA helicase DDX3X | IPI00215637 | -2.242 | 0.00786 | 55 | 30 | 29 | 23 |
| 2972 | Solute carrier family 2, facilitated glucose transporter member 1 | IPI00220194 | -2.282 | 0.00761 | 51 | 60 | 41 | 33 |
| 2973 | Isoform 2 of Heat shock protein HSP 90-alpha | IPI00382470 | -2.370 | 0.00719 | 253 | 172 | 200 | 164 |
| 2974 | Histone H1.2 | IPI00217465 | -2.431 | 0.00665 | 41 | 19 | 21 | 8 |
| 2975 | Isoform 1 of DNA-dependent protein kinase catalytic subunit | IPI00296337 | -2.456 | 0.00661 | 277 | 254 | 217 | 246 |
| 2976 | Histone H3.2 | IPI00171611 | -2.585 | 0.00602 | 26 | 23 | 10 | 9 |
| 2977 | X-ray repair cross-complementing protein 6 | IPI00644712 | -2.629 | 0.00577 | 130 | 91 | 95 | 72 |
| 2978 | FACT complex subunit SPT16 | IPI00026970 | -2.715 | 0.00531 | 50 | 58 | 26 | 39 |
| 2979 | Fatty acid synthase | IPI00026781 | -2.834 | 0.00414 | 375 | 383 | 359 | 311 |
| 2980 | Poly [ADP-ribose] polymerase 1 | IPI00449049 | -3.024 | 0.00381 | 42 | 33 | 15 | 19 |
| 2981 | X-ray repair cross-complementing protein 5 | IPI00220834 | -3.161 | 0.00326 | 124 | 94 | 83 | 71 |
| 2982 | Isoform 2 of Nuclear mitotic apparatus protein 1 | IPI00006196 | -3.229 | 0.00314 | 45 | 37 | 17 | 20 |
| 2983 | Structural maintenance of chromosomes protein 3 | IPI00219420 | -3.266 | 0.00305 | 33 | 56 | 15 | 27 |
| 2984 | Isoform 3 of Core histone macro-H2A.1 | IPI00059366 | -3.807 | 0.00134 | 30 | 31 | 6 | 10 |
| 2986 | Isoform 3 of DNA topoisomerase 2-alpha | IPI00218753 | -5.055 | 0.00033 | 25 | 29 | 0 | 2 |
| 2987 | Histone H2A.V | IPI00018278 | -7.056 | 0.00017 | 81 | 113 | 37 | 32 |
| 2988 | Histone H2A type 1-B/E | IPI00026272 | -8.785 | 0.00008 | 53 | 52 | 0 | 0 |
| 2989 | Histone H4 | IPI00453473 | -10.302 | 0.00000 | 279 | 315 | 146 | 173 |
