# Supplementary material for: Data supporting the identification of anti-metastatic drug and natural compound targets in isogenic colorectal cancer cells
Source: Data Brief. 2014 Nov 4;1:73–5. doi: 10.1016/j.dib.2014.10.005 (PMC4459770; doi:10.1016/j.dib.2014.10.005)
Supplement: Supplementary file 1 — Supplementary data [file mmc1.zip › CRC_Metastasis_DIB_Table06.pdf]

Table 6. List of proteins differentially expressed in SW620 by the treatment of curcumin for 48hr. ([1]STN and p-value were acquired from PLGEM analysis, [2]Raw spectral counts from data compilation using ScaffoldTM software)

No.	Description	Accession number	STN[1]	p-Value[1]	Con_A[2]	Con_B[2]	CUR_A[2]	CUR_B[2]
1	Neuroblast differentiation-associated protein AHNAK	IPI00021812	13.556	0.00000	28	33	98	106
2	Isoform 1 of Plectin-1	IPI00014898	11.639	0.00000	201	188	309	284
3	NAD(P)H dehydrogenase [quinone] 1	IPI00012069	7.511	0.00009	33	33	68	72
4	Cytoplasmic dynein 1 heavy chain 1	IPI00456969	5.811	0.00028	294	281	353	334
5	Isoform 1 of Serine/arginine repetitive matrix protein 2	IPI00782992	5.369	0.00028	32	34	60	57
6	Alanyl-tRNA synthetase, cytoplasmic	IPI00027442	4.478	0.00043	50	52	82	68
7	Isoleucyl-tRNA synthetase, cytoplasmic	IPI00644127	4.173	0.00057	17	18	34	33
8	ADP-ribosylation factor 1	IPI00215914	3.573	0.00080	96	113	121	136
9	UDP-glucose 6-dehydrogenase	IPI00031420	3.447	0.00090	17	14	27	29
10	Isoform 1 of Voltage-dependent anion-selective channel protein 3	IPI00031804	3.424	0.00099	22	24	33	41
11	Tubulin beta-2C chain	IPI00007752	3.418	0.00099	145	192	189	202
12	Voltage-dependent anion-selective channel protein 1	IPI00216308	3.273	0.00118	46	52	66	66
13	U1 small nuclear ribonucleoprotein A	IPI00012382	3.266	0.00118	10	11	21	21
14	Putative heat shock protein HSP 90-alpha A2	IPI00031523	3.259	0.00118	147	146	174	168
15	Isoform 1 of 26S proteasome non-ATPase regulatory subunit 1	IPI00299608	3.141	0.00151	28	27	42	40
16	Cofilin-1	IPI00012011	2.991	0.00180	163	152	180	181
17	6-phosphofructokinase type C	IPI00009790	2.879	0.00203	12	8	20	18
18	Isoform Long of Long-chain-fatty-acid--CoA ligase 4	IPI00029737	2.879	0.00203	10	10	17	21
19	probable E3 ubiquitin-protein ligase MYCBP2	IPI00289776	2.812	0.00217	9	9	16	19
20	Calpain-2 catalytic subunit	IPI00289758	2.792	0.00217	8	7	15	16
21	Nucleoprotein TPR	IPI00742682	2.769	0.00222	30	44	49	51
22	Histone H2A.V	IPI00018278	2.730	0.00236	94	109	107	132
23	Transitional endoplasmic reticulum ATPase	IPI00022774	2.702	0.00250	54	46	69	59
24	Quinone oxidoreductase	IPI00000792	2.662	0.00283	1	2	6	7
25	protein ALO17 isoform 1	IPI00828098	2.635	0.00283	9	6	15	15
26	Annexin A1	IPI00218918	2.626	0.00293	0	1	8	3
27	Moesin	IPI00219365	2.599	0.00298	36	35	47	48
28	Methionyl-tRNA synthetase, cytoplasmic	IPI00008240	2.598	0.00298	14	15	24	23
29	Isoform 2 of Microtubule-associated protein 4	IPI00220113	2.570	0.00335	6	4	12	11
30	Aspartate aminotransferase, mitochondrial	IPI00018206	2.519	0.00354	33	36	45	47
31	Isoform 2 of 6-phosphofructokinase, muscle type	IPI00219585	2.517	0.00354	2	2	7	7
32	Isoform 2 of Heat shock protein HSP 90-alpha	IPI00382470	2.502	0.00373	183	199	207	216
33	Isoform 1 of Enhancer of mRNA-decapping protein 4	IPI00376317	2.459	0.00373	10	14	21	19
34	Peroxiredoxin-6	IPI00220301	2.456	0.00373	61	73	79	83
35	Annexin A5	IPI00329801	2.443	0.00373	39	37	53	46
36	Isoform 1 of RRP12-like protein	IPI00101186	2.439	0.00373	11	9	14	21
37	Isoform 1 of Cullin-4B	IPI00179057	2.401	0.00378	3	2	8	7
38	Keratin, type I cytoskeletal 9	IPI00019359	2.371	0.00406	75	59	83	78
39	26S proteasome non-ATPase regulatory subunit 2	IPI00012268	2.351	0.00416	20	14	24	27
40	Insulin-degrading enzyme	IPI00220373	2.305	0.00435	2	4	9	7
41	lanosterol 14-alpha demethylase isoform 1	IPI00295772	2.302	0.00472	2	2	6	7
42	ATP-dependent DNA helicase Q1	IPI00178431	2.262	0.00482	17	15	29	19
43	ADP/ATP translocase 3	IPI00291467	2.226	0.00487	80	82	101	88
44	Beta-hexosaminidase subunit beta	IPI00012585	2.224	0.00487	3	4	9	8
45	cysteinyl-tRNA synthetase, cytoplasmic isoform c	IPI00027443	2.203	0.00496	8	10	18	13
46	Phosphatidylinositol-4-phosphate 3-kinase C2 domain-containing subunit alpha	IPI00002580	2.193	0.00510	3	2	7	7
47	Isoform 1 of Pre-mRNA-processing factor 40 homolog A	IPI00337385	2.193	0.00510	4	1	7	7
48	Transmembrane emp24 domain-containing protein 10	IPI00028055	2.180	0.00520	25	27	36	34
49	probable ubiquitin carboxyl-terminal hydrolase FAF-X isoform 4	IPI00003964	2.170	0.00520	9	10	17	15
50	Paired amphipathic helix protein Sin3a	IPI00170596	2.162	0.00520	5	6	11	11
51	Isoform 1 of 1-phosphatidylinositol-4,5-bisphosphate phosphodiesterase gamma-1	IPI00016736	2.153	0.00529	3	5	10	8
52	Isoform 1 of Cytoskeleton-associated protein 5	IPI00028275	2.150	0.00562	18	13	25	21
53	Serine hydroxymethyltransferase, mitochondrial	IPI00002520	2.131	0.00576	25	22	32	32
54	Profilin-1	IPI00216691	2.118	0.00576	139	140	162	148
55	Chloride intracellular channel protein 1	IPI00010896	2.117	0.00576	25	23	35	30
56	E3 SUMO-protein ligase RanBP2	IPI00221325	2.115	0.00576	19	21	31	25
57	Proteasome subunit beta type-4	IPI00555956	2.115	0.00576	19	21	27	29
58	Prohibitin	IPI00017334	2.065	0.00624	71	73	85	83
59	Isoform 2 of Microtubule-actin cross-linking factor 1, isoforms 1/2/3/5	IPI00256861	2.034	0.00633	5	5	11	9
60	Malate dehydrogenase, mitochondrial	IPI00291006	2.028	0.00638	51	52	62	62
61	Helicase SKI2W	IPI00414819	2.027	0.00642	4	3	7	9
62	Isoform 1 of Glycerol-3-phosphate dehydrogenase, mitochondrial	IPI00017895	2.016	0.00642	9	10	15	16
63	Radixin, isoform CRA_a	IPI00017367	2.005	0.00642	30	27	36	38
64	Protein transport protein Sec23A	IPI00017375	1.984	0.00652	5	6	12	9
65	Isoform 1 of Kinectin	IPI00328753	1.981	0.00652	1	4	8	5
66	Probable dimethyladenosine transferase	IPI00004459	1.981	0.00652	2	3	7	6
67	Dehydrogenase/reductase SDR family member 7B	IPI00550165	1.976	0.00652	2	0	5	5
68	Serine/threonine-protein kinase PRP4 homolog	IPI00013721	1.976	0.00652	2	1	7	3
69	Cathepsin B	IPI00295741	1.976	0.00652	2	1	5	5
70	proteasome-associated protein ECM29 homolog	IPI00157790	1.974	0.00652	19	22	29	27
71	Protein disulfide-isomerase A4	IPI00009904	1.953	0.00652	51	49	61	59
72	Stress-70 protein, mitochondrial	IPI00007765	1.916	0.00694	73	86	96	86
73	Isocitrate dehydrogenase [NADP] cytoplasmic	IPI00027223	1.897	0.00709	4	2	6	8
74	22 kDa protein	IPI00219910	1.897	0.00709	3	3	8	6
75	Delta(3,5)-Delta(2,4)-dienoyl-CoA isomerase, mitochondrial	IPI00011416	1.892	0.00742	27	30	37	36
76	Isoform 1 of ATP synthase subunit d, mitochondrial	IPI00220487	1.884	0.00742	0	1	4	4
77	HEAT repeat-containing protein 1	IPI00024279	1.868	0.00841	36	48	50	52
78	Isoform 2 of Myosin-Ic	IPI00010418	1.858	0.00855	7	7	12	12
79	NADP-dependent malic enzyme	IPI00008215	1.857	0.00855	3	1	7	4
80	Isoform A of Phosphate carrier protein, mitochondrial	IPI00022202	1.843	0.00869	25	26	29	37
81	Leucyl-tRNA synthetase, cytoplasmic	IPI00103994	1.843	0.00869	24	27	32	34
82	Isoform 2 of Nucleosome-remodeling factor subunit BPTF	IPI00254408	1.826	0.00879	4	3	7	8
83	Bifunctional aminoacyl-tRNA synthetase	IPI00013452	1.820	0.00883	47	44	57	52
84	Src substrate cortactin	IPI00029601	1.803	0.00883	4	7	7	13
85	DYNC1H1 protein	IPI00440177	1.764	0.00921	3	5	11	5
86	Isoform 1 of CLIP-associating protein 1	IPI00396279	1.764	0.00921	2	6	8	8
87	Elongator complex protein 1	IPI00293735	1.764	0.00921	2	6	8	8
88	2,4-dienoyl-CoA reductase, mitochondrial	IPI00003482	1.764	0.00921	4	4	7	9
89	Peptidylprolyl isomerase domain and WD repeat-containing protein 1	IPI00149650	1.763	0.00950	2	3	6	6
90	Isoform A of Lamin-A/C	IPI00021405	1.746	0.00964	37	36	48	41
91	vacuolar protein sorting-associated protein 13C isoform 2B	IPI00412216	1.736	0.00973	20	20	27	26

No.	Description	Accession number	STN[1]	p-Value[1]	Con_A[2]	Con_B[2]	CUR_A[2]	CUR_B[2]
92	Putative uncharacterized protein DOCK6	IPI00184772	1.733	0.00973	0	2	6	3
93	sideroflexin-3	IPI00793874	1.733	0.00973	1	2	5	4
94	Isoform 1 of Lipopolysaccharide-responsive and beige-like anchor protein	IPI00002255	1.724	0.00983	54	53	61	64
95	165 kDa protein	IPI00240812	1.722	0.00983	7	6	12	10
96	Isoform 1 of CDP-diacylglycerol--inositol 3-phosphatidyltransferase	IPI00645518	1.722	0.00983	5	8	13	9
97	Neurolysin, mitochondrial	IPI00010346	1.710	0.01006	4	5	8	9
98	Glycogen phosphorylase, liver form	IPI00783313	1.694	0.01025	12	14	20	17
99	Endoplasmin	IPI00027230	1.680	0.01049	64	71	80	74
100	E3 ubiquitin-protein ligase UBR5	IPI00026320	1.678	0.01049	19	16	23	24
101	Cullin-1	IPI00014310	1.662	0.01068	6	4	9	9
102	D-3-phosphoglycerate dehydrogenase	IPI00011200	1.652	0.01105	11	10	13	18
103	Peroxiredoxin-1	IPI00000874	1.626	0.01139	52	56	67	58
104	Isocitrate dehydrogenase 3, beta subunit isoform a precursor	IPI00304417	1.624	0.01139	3	1	6	4
105	Claudin-1	IPI00000691	1.624	0.01139	2	2	7	3
106	Protein NipSnap homolog 1	IPI00304435	1.622	0.01294	8	8	14	11
107	Serine/threonine-protein phosphatase 2A 55 kDa regulatory subunit B alpha isoform	IPI00332511	1.617	0.01299	1	1	4	3
108	Cullin-5	IPI00216003	1.617	0.01299	1	1	4	3
109	Tetratricopeptide repeat protein 35	IPI00014149	1.617	0.01299	1	1	4	3
110	Isoform 1 of Serine/threonine-protein kinase WNK1	IPI00004472	1.617	0.01299	0	0	2	5
111	Integrin-linked protein kinase	IPI00013219	1.617	0.01299	0	1	1	6
112	cDNA FLJ56840, highly similar to Galactokinase	IPI00019383	1.617	0.01299	1	1	3	4
113	Putative uncharacterized protein MDH1	IPI00915869	1.607	0.01327	11	12	18	15
114	Glycyl-tRNA synthetase	IPI00783097	1.607	0.01327	26	25	35	29
115	Gamma-glutamyl hydrolase	IPI00023728	1.596	0.01351	19	22	26	27
116	Isoform 1AB of Catenin delta-1	IPI00182469	1.564	0.01394	5	3	6	9
117	Staphylococcal nuclease domain-containing protein 1	IPI00140420	1.553	0.01413	45	41	45	56
118	TRIP12 protein	IPI00032342	1.545	0.01436	7	6	10	11
119	Isoform 1 of Peripherin	IPI00013164	1.545	0.01436	7	6	8	13
120	26S protease regulatory subunit 4	IPI00011126	1.544	0.01436	17	18	24	22
121	Aspartate aminotransferase, cytoplasmic	IPI00219029	1.544	0.01436	17	18	23	23
122	Insulin-like growth factor 2 mRNA-binding protein 1	IPI00008557	1.542	0.01436	8	11	14	14
123	Isoform 1 of Hexokinase-1	IPI00018246	1.538	0.01436	2	3	6	5
124	ADP-sugar pyrophosphatase	IPI00296913	1.530	0.01469	14	13	19	18
125	Vesicle-fusing ATPase	IPI00006451	1.515	0.01512	5	4	8	8
126	26 kDa protein	IPI00219685	1.515	0.01512	4	5	8	8
127	Isoform 1 of Phosphoenolpyruvate carboxykinase [GTP], mitochondrial	IPI00797038	1.515	0.01512	5	4	8	8
128	Isoform 1 of LETM1 and EF-hand domain-containing protein 1, mitochondrial	IPI00017592	1.496	0.01620	12	9	15	15
129	Isoform 1 of N-acylneuraminate cytidylyltransferase	IPI00303158	1.481	0.01630	1	2	4	4
130	Isoform 1 of Protein-tyrosine phosphatase mitochondrial 1	IPI00174190	1.481	0.01630	2	1	4	4
131	Myosin-Ie	IPI00329672	1.481	0.01630	1	2	3	5
132	Retinol dehydrogenase 13	IPI00301204	1.481	0.01630	1	2	4	4
133	Lysosomal alpha-glucosidase	IPI00293088	1.481	0.01630	2	1	4	4
134	Isoleucyl-tRNA synthetase, mitochondrial	IPI00017283	1.471	0.01644	3	7	9	8
135	Isoform 1 of COP9 signalosome complex subunit 7b	IPI00009301	1.471	0.01644	5	5	8	9
136	cDNA FLJ56357, highly similar to Homo sapiens apolipoprotein A-I binding protein (APOA1BP), mRNA	IPI00168479	1.471	0.01644	5	5	9	8
137	Isoform 1 of Uridine 5'-monophosphate synthase	IPI00003923	1.469	0.01668	2	4	7	5
138	Exportin-7	IPI00302458	1.469	0.01668	3	3	8	4
139	Heat shock 70 kDa protein 12A	IPI00011932	1.469	0.01668	2	4	6	6
140	Complement component 1 Q subcomponent-binding protein, mitochondrial	IPI00014230	1.449	0.01847	92	116	107	120
141	Isochorismatase domain-containing protein 1	IPI00304082	1.436	0.01861	10	14	14	19
142	baculoviral IAP repeat-containing protein 6	IPI00299635	1.432	0.01861	5	6	11	7
143	cDNA FLJ55177, highly similar to Ras-related protein Ral-B	IPI00004397	1.428	0.01866	8	9	13	12
144	Isoform 1 of Nuclear pore complex protein Nup214	IPI00183294	1.410	0.01894	4	3	6	7
145	Mitochondrial-processing peptidase subunit alpha	IPI00166749	1.410	0.01894	3	4	6	7
146	Isoform 2 of Golgi apparatus protein 1	IPI00414717	1.410	0.01894	4	3	7	6
147	Eukaryotic translation initiation factor 5B	IPI00299254	1.402	0.01909	31	30	40	33
148	Isoform 1 of Symplekin	IPI00023344	1.397	0.01913	5	7	9	10
149	Glutathione S-transferase omega-1	IPI00019755	1.397	0.01913	6	6	11	8
150	NADPH--cytochrome P450 reductase	IPI00470467	1.397	0.01913	6	6	10	9
151	Ezrin	IPI00843975	1.386	0.01932	26	23	29	31
152	Lactoylglutathione lyase	IPI00220766	1.384	0.01932	14	13	19	17
153	Isoform 1 of Transcription elongation factor SPT5	IPI00298058	1.383	0.01932	2	2	6	3
154	Putative uncharacterized protein DKFZp451D234	IPI00031583	1.383	0.01932	2	2	5	4
155	Isoform 2C of Cytoplasmic dynein 1 intermediate chain 2	IPI00216348	1.383	0.01932	2	2	4	5
156	Nuclear pore complex protein Nup153	IPI00292059	1.383	0.01932	1	3	5	4
157	Isoform 1 of Tryptophanyl-tRNA synthetase, cytoplasmic	IPI00295400	1.368	0.01989	14	14	17	20
158	Glutathione S-transferase P	IPI00219757	1.362	0.02027	141	146	157	150
159	COP9 signalosome complex subunit 5	IPI00009958	1.359	0.02027	4	4	7	7
160	Ras-related protein Rap-2c	IPI00009607	1.359	0.02027	5	3	7	7
161	Fascin	IPI00163187	1.359	0.02093	9	11	13	15
162	Rab GDP dissociation inhibitor alpha	IPI00010154	1.350	0.02154	26	27	32	32
163	Isoform 1 of Myoferlin	IPI00021048	1.342	0.02154	29	25	33	32
164	Isoform 1 of Heterogeneous nuclear ribonucleoprotein M	IPI00171903	1.340	0.02154	66	67	74	74
165	Serine/threonine-protein kinase MRCK beta	IPI00477763	1.338	0.02154	1	0	3	3
166	Microtubule-associated protein 1B	IPI00008868	1.338	0.02154	0	0	5	1
167	Isoform 1 of THO complex subunit 1	IPI00305374	1.338	0.02154	1	0	4	2
168	A-kinase anchor protein 12 isoform 2	IPI00217683	1.338	0.02154	0	0	3	3
169	GTP-binding protein 1	IPI00010463	1.338	0.02154	1	0	3	3
170	Isoform 1 of Clathrin coat assembly protein AP180	IPI00006612	1.338	0.02154	1	1	3	3
171	Isoform 1 of Mammalian ependymin-related protein 1	IPI00259102	1.338	0.02154	1	0	3	3
172	Proteasome subunit alpha type-6	IPI00029623	1.325	0.02702	16	15	22	18
173	Isoform 1 of Mitochondrial inner membrane protein	IPI00009960	1.319	0.02707	10	12	15	15
174	Isoform 1 of Host cell factor 1	IPI00019848	1.315	0.02721	6	3	7	8
175	Ribosome biogenesis protein WDR12	IPI00304232	1.315	0.02721	3	6	7	8
176	Isoform 2 of SWI/SNF complex subunit SMARCC2	IPI00150057	1.311	0.02735	15	17	19	22
177	Carbonyl reductase [NADPH] 1	IPI00295386	1.308	0.02745	7	8	11	11
178	Heterogeneous nuclear ribonucleoprotein H2	IPI00026230	1.307	0.02745	2	3	3	7
179	Aspartyl-tRNA synthetase, mitochondrial	IPI00100460	1.307	0.02745	2	3	3	7
180	Glutamate--cysteine ligase regulatory subunit	IPI00010090	1.307	0.02745	3	2	6	4
181	SPRY domain-containing protein 4	IPI00291643	1.307	0.02745	3	2	4	6
182	cDNA FLJ56402, highly similar to Tripeptidyl-peptidase 1	IPI00298237	1.307	0.02745	2	3	3	7
183	Ubiquilin-2	IPI00409659	1.307	0.02745	3	2	5	5
184	Tubulin beta-1 chain	IPI00006510	1.281	0.02801	22	25	29	28
185	Isoform 1 of Dynamin-2	IPI00033022	1.276	0.02806	6	4	10	6

No.	Description	Accession number	STN[1]	p-Value[1]	Con_A[2]	Con_B[2]	CUR_A[2]	CUR_B[2]
186	X-ray repair cross-complementing protein 6	IPI00644712	1.263	0.02943	117	108	123	119
187	Ubiquitin carboxyl-terminal hydrolase 7	IPI00003965	1.252	0.02981	12	14	17	17
188	Isoform 1 of Protein diaphanous homolog 1	IPI00852685	1.251	0.03000	20	17	26	20
189	Protein MEMO1	IPI00032426	1.246	0.03005	3	3	7	4
190	Trifunctional enzyme subunit beta, mitochondrial	IPI00022793	1.241	0.03033	5	6	8	9
191	Isoform 5 of E3 ubiquitin-protein ligase UBR4	IPI00180305	1.237	0.03047	8	10	14	11
192	Isoform 1 of 5'-3' exoribonuclease 2	IPI00100151	1.237	0.03047	10	8	11	14
193	Isoform Long of Glucose-6-phosphate 1-dehydrogenase	IPI00216008	1.237	0.03061	13	14	16	19
194	Isoform 3 of Probable ATP-dependent RNA helicase DDX17	IPI00651653	1.237	0.03061	13	14	19	16
195	HSR1 protein	IPI00384745	1.219	0.03094	2	0	2	5
196	bifunctional protein NCOAT isoform b	IPI00181391	1.219	0.03094	2	1	3	4
197	Isoform 2 of Phosphatidylinositol-binding clathrin assembly protein	IPI00216184	1.219	0.03094	1	2	4	3
198	Choline-phosphate cytidylyltransferase A	IPI00329338	1.219	0.03094	1	2	3	4
199	Isoform 1 of Rho GTPase-activating protein 18	IPI00296353	1.219	0.03094	1	2	5	2
200	Aconitate hydratase, mitochondrial	IPI00017855	1.210	0.03104	7	5	10	8
201	Prostaglandin E synthase 2	IPI00303568	1.210	0.03104	5	7	9	9
202	Proteasome subunit beta type-7	IPI00003217	1.209	0.03231	16	13	17	20
203	Isoform 1 of Dipeptidyl peptidase 3	IPI00020672	1.209	0.03231	13	16	16	21
204	26S proteasome non-ATPase regulatory subunit 12	IPI00185374	1.209	0.03231	28	28	33	33
205	Isoform 1 of Apoptosis-inducing factor 1, mitochondrial	IPI00000690	1.204	0.03269	47	49	53	55
206	Signal recognition particle receptor subunit beta	IPI00295098	1.197	0.03269	11	9	13	14
207	Eukaryotic peptide chain release factor GTP-binding subunit ERF3A	IPI00218829	1.194	0.03279	3	4	7	5
208	Isoform 1 of RNA polymerase II-associated protein 3	IPI00002408	1.194	0.03279	4	3	5	7
209	Isoform 1 of Nucleolar protein 6	IPI00152890	1.194	0.03279	4	3	7	5
210	Isoform 1 of Origin recognition complex subunit 3	IPI00294402	1.194	0.03279	4	3	6	6
211	Isoform 1 of Melanoma-associated antigen D2	IPI00009542	1.194	0.03279	4	3	6	6
212	Trifunctional enzyme subunit alpha, mitochondrial	IPI00031522	1.181	0.03321	41	38	45	45
213	Leucine-rich repeat-containing protein 59	IPI00396321	1.171	0.03321	16	16	20	20
214	Isoform 2 of Voltage-dependent anion-selective channel protein 2	IPI00024145	1.157	0.03364	23	24	27	29
215	Isoform 3 of Obg-like ATPase 1	IPI00216106	1.155	0.03364	7	7	9	11
216	Isoform SERCA2A of Sarcoplasmic/endoplasmic reticulum calcium ATPase 2	IPI00177817	1.150	0.03430	5	3	8	5
217	GTPase NRas	IPI00000005	1.150	0.03430	4	4	7	6
218	Aflatoxin B1 aldehyde reductase member 2	IPI00305978	1.150	0.03430	4	4	7	6
219	Histone H2A type 1-B/E	IPI00026272	1.134	0.03519	107	108	114	116
220	Tubulin beta-3 chain	IPI00013683	1.134	0.03519	2	2	1	7
221	Protein VAC14 homolog	IPI00025160	1.134	0.03519	3	1	3	5
222	Isoform A1 of Tight junction protein ZO-2	IPI00003843	1.134	0.03519	1	3	3	5
223	Protein transport protein Sec23B	IPI00017376	1.134	0.03519	1	3	4	4
224	Isoform 1 of RNA polymerase II-associated protein 1	IPI00402657	1.134	0.03519	1	3	4	4
225	poly [ADP-ribose] polymerase 14	IPI00291215	1.134	0.03519	2	2	4	4
226	Isoform 2 of Chromodomain-helicase-DNA-binding protein 2	IPI00023109	1.134	0.03519	2	2	4	4
227	Isoform 1 of Coiled-coil domain-containing protein 109A	IPI00171573	1.134	0.03519	2	2	5	3
228	tRNA (guanine-N(7)-)-methyltransferase	IPI00290184	1.134	0.03519	2	2	4	4
229	Mitochondrial ribonuclease P protein 1	IPI00099996	1.134	0.03519	1	3	4	4
230	cDNA FLJ12528 fis, clone NT2RM4000155, moderately similar to THREONYL-TRNA SYNTHETASE	IPI00018632	1.134	0.03519	2	2	4	4
231	Methylmalonyl-CoA mutase, mitochondrial	IPI00024934	1.134	0.03519	2	2	4	4
232	Isoform 1 of Splicing factor, arginine/serine-rich 12	IPI00103497	1.134	0.03519	2	2	3	5
233	Isoform 1 of Transportin-1	IPI00024364	1.130	0.03921	13	11	16	15
234	Seryl-tRNA synthetase, cytoplasmic	IPI00220637	1.130	0.03921	11	13	16	15
235	Isoform 1 of Cleavage and polyadenylation specificity factor subunit 7	IPI00550821	1.116	0.03959	11	14	16	16
236	HSPA5 protein	IPI00003362	1.112	0.03964	96	91	101	100
237	Aspartyl-tRNA synthetase, cytoplasmic	IPI00216951	1.107	0.04058	18	20	24	22
238	Heat shock 70 kDa protein 1A/1B	IPI00304925	1.090	0.04077	40	36	43	43
239	60S ribosomal protein L24	IPI00306332	1.088	0.04105	10	7	13	10
240	Isoform 1 of Nuclear pore complex protein Nup98-Nup96	IPI00006038	1.077	0.04134	5	5	8	7
241	cDNA FLJ53927, highly similar to Beta-hexosaminidase alpha chain	IPI00027851	1.077	0.04134	7	3	9	6
242	Isoform 1 of ATP-binding cassette sub-family B member 7, mitochondrial	IPI00306748	1.069	0.04162	13	5	12	12
243	Isoform Mitochondrial of Glutathione reductase, mitochondrial	IPI00016862	1.069	0.04162	9	9	9	15
244	Isoform 2 of Vacuolar protein sorting-associated protein 13A	IPI00478586	1.068	0.04242	1	4	6	3
245	145 kDa protein	IPI00218097	1.068	0.04242	3	2	5	4
246	FAST kinase domain-containing protein 5	IPI00414973	1.068	0.04242	2	3	5	4
247	Ribose-5-phosphate isomerase	IPI00026513	1.068	0.04242	2	3	7	2
248	Phosphoglucomutase-2	IPI00550364	1.068	0.04242	2	3	4	5
249	Isoform 1 of Thymocyte nuclear protein 1	IPI00383163	1.068	0.04242	3	2	5	4
250	Isoform 1 of Putative deoxyribonuclease TATDN1	IPI00012463	1.068	0.04242	2	3	5	4
251	Isoform 1 of Filamin-B	IPI00289334	1.055	0.04247	59	51	60	61
252	retinol-binding protein 1 isoform a	IPI00219718	1.052	0.04247	15	15	20	17
253	Protein tyrosine phosphatase-like protein PTPLAD1	IPI00008998	1.047	0.04247	22	23	28	25
254	Isoform Long of Tight junction protein ZO-1	IPI00216219	1.047	0.04247	6	5	9	7
255	Isoform 1 of Vesicle-associated membrane protein-associated protein A	IPI00170692	1.047	0.04247	5	6	9	7
256	Pirin	IPI00012575	1.047	0.04247	6	5	9	7
257	Rho-related GTP-binding protein RhoC	IPI00027434	1.044	0.04280	1	1	2	3
258	U6 snRNA-associated Sm-like protein LSm1	IPI00004436	1.044	0.04280	1	1	2	3
259	ERBB2IP protein	IPI00438286	1.044	0.04280	1	1	2	3
260	Prenylcysteine oxidase 1	IPI00384280	1.044	0.04280	1	1	2	3
261	ATP-dependent RNA helicase SUPV3L1, mitochondrial	IPI00412404	1.044	0.04280	0	0	2	3
262	Isoform 1 of Beta-galactosidase	IPI00441344	1.044	0.04280	1	0	3	2
263	NADH dehydrogenase [ubiquinone] 1 beta subcomplex subunit 5, mitochondrial	IPI00013459	1.044	0.04280	1	1	2	3
264	Isoform 1 of Cullin-3	IPI00014312	1.044	0.04280	1	1	3	2
265	Ras-related protein Rab-9A	IPI00016372	1.044	0.04280	1	1	3	2
266	Isoform 2 of NADH dehydrogenase [ubiquinone] flavoprotein 3, mitochondrial	IPI00291016	1.044	0.04280	1	1	3	2
267	Plakophilin-3	IPI00026952	1.044	0.04280	0	1	3	2
268	Isoform 3 of Mitochondrial Rho GTPase 1	IPI00217536	1.044	0.04280	1	0	3	2
269	Isoform 2 of Liprin-beta-1	IPI00179172	1.044	0.04280	1	0	4	1
270	Isoform 1 of Putative methyltransferase METT10D	IPI00163391	1.044	0.04280	1	0	2	3
271	cDNA FLJ55508, highly similar to Sad1/unc-84-like protein 2	IPI00295940	1.044	0.04280	1	1	2	3
272	Protein UXT	IPI00002646	1.044	0.04280	1	0	3	2
273	Isoform 4 of Abhydrolase domain-containing protein 11	IPI00171152	1.044	0.04280	1	1	3	2
274	Myotubularin	IPI00748788	1.044	0.04280	1	1	3	2
275	114 kDa protein	IPI00166555	1.044	0.04280	1	1	3	2
276	Isoform 2 of ATP-binding cassette sub-family B member 6, mitochondrial	IPI00065486	1.044	0.04280	0	0	3	2
277	Retinal rod rhodopsin-sensitive cGMP 3',5'-cyclic phosphodiesterase subunit delta	IPI00015161	1.044	0.04280	1	1	3	2
278	Isoform 1 of Gelsolin	IPI00026314	1.044	0.04280	0	0	3	2
279	Patatin-like phospholipase domain-containing protein 4	IPI00013218	1.044	0.04280	1	1	3	2
280	Isoform 1 of Transmembrane protein 55B	IPI00030530	1.044	0.04280	1	0	3	2

No.	Description	Accession number	STN[1]	p-Value[1]	Con_A[2]	Con_B[2]	CUR_A[2]	CUR_B[2]
281	Sideroflexin-1	IPI00009368	1.034	0.04370	12	8	13	13
282	Glutamate dehydrogenase 1, mitochondrial	IPI00016801	1.032	0.04408	23	24	26	29
283	ATP synthase subunit alpha, mitochondrial	IPI00440493	1.022	0.04417	77	77	82	84
284	Isoform 1 of DNA-dependent protein kinase catalytic subunit	IPI00296337	1.021	0.04460	236	247	255	246
285	Transmembrane emp24 domain-containing protein 2	IPI00016608	1.020	0.04460	5	7	8	9
286	Isoform 1 of Oxysterol-binding protein 1	IPI00024971	1.020	0.04460	6	6	10	7
287	Isoform 1 of Oligoribonuclease, mitochondrial (Fragment)	IPI00032830	1.020	0.04460	7	5	10	7
288	Aldo-keto reductase family 1 member C2	IPI00005668	1.016	0.04474	4	2	5	5
289	Small nuclear ribonucleoprotein Sm D3	IPI00017964	1.016	0.04474	3	3	5	5
290	TATA box-binding protein-like protein 1	IPI00032911	1.016	0.04474	3	3	5	5
291	ER lumen protein retaining receptor 1	IPI00028116	1.016	0.04474	3	3	5	5
292	L antigen family member 3	IPI00032314	1.016	0.04474	3	3	4	6
293	cDNA FLJ56152, highly similar to Rho guanine nucleotide exchange factor 7	IPI00449906	1.016	0.04474	4	2	4	6
294	Cytovillin 2 (Fragment)	IPI00384282	1.016	0.04474	4	2	5	5
295	Uncharacterized protein KIAA1797	IPI00748360	1.016	0.04474	3	3	5	5
296	Uncharacterized protein C17orf25	IPI00007102	1.003	0.04932	11	11	15	13
297	Abhydrolase domain-containing protein 10, mitochondrial	IPI00020075	1.003	0.04932	12	10	14	14
298	Prohibitin-2	IPI00027252	1.002	0.04993	84	79	87	88
299	Isoform 4 of E3 ubiquitin-protein ligase UBR4	IPI00640981	1.000	0.05012	20	15	22	20
300	Very long-chain acyl-CoA synthetase	IPI00024787	0.995	0.05012	8	5	9	9
301	N(G),N(G)-dimethylarginine dimethylaminohydrolase 2	IPI00000760	0.995	0.05012	7	6	10	8
302	26S protease regulatory subunit 6A	IPI00018398	0.988	0.05041	10	13	16	13
303	Destrin	IPI00473014	0.973	0.05097	21	17	19	26
304	proteasome 26S non-ATPase subunit 8	IPI00010201	0.973	0.05097	17	21	22	23
305	Proteasome subunit alpha type-2	IPI00219622	0.973	0.05097	28	28	29	35
306	24 kDa protein	IPI00398057	0.972	0.05097	6	8	9	10
307	Isoform 1 of Enoyl-CoA hydratase domain-containing protein 1	IPI00302688	0.972	0.05097	9	5	8	11
308	Lysophospholipid acyltransferase 5	IPI00306419	0.972	0.05097	7	7	9	10
309	Isoform 3 of Serine/threonine-protein kinase SMG1	IPI00183368	0.972	0.05097	5	2	5	6
310	cDNA FLJ59712, highly similar to Golgi reassembly-stacking protein 2	IPI00743931	0.972	0.05097	2	5	7	4
311	Protein ERGIC-53	IPI00026530	0.972	0.05097	3	4	5	6
312	pyruvate dehydrogenase E1 alpha 1 isoform 2 precursor	IPI00306301	0.972	0.05097	4	3	6	5
313	Isoform 1 of Clathrin heavy chain 2	IPI00022881	0.964	0.05097	95	87	97	97
314	Isoform 1 of Hydroxyacyl-coenzyme A dehydrogenase, mitochondrial	IPI00294398	0.962	0.05097	11	14	13	18
315	Signal recognition particle 54 kDa protein	IPI00009822	0.962	0.05097	11	14	15	16
316	40S ribosomal protein S17	IPI00221093	0.956	0.05111	20	20	23	24
317	Nuclear pore complex protein Nup205	IPI00783781	0.954	0.05116	41	42	50	42
318	Phenylalanyl-tRNA synthetase alpha chain	IPI00031820	0.951	0.05116	6	9	13	7
319	Protein transport protein Sec24C	IPI00024661	0.951	0.05116	7	8	11	9
320	Isoform Alpha of Signal transducer and activator of transcription 1-alpha/beta	IPI00030781	0.951	0.05116	9	6	12	8
321	Keratin, type II cytoskeletal 2 epiderma	IPI00021304	0.948	0.05197	58	56	63	61
322	Isoform Delta-1 of Serine/threonine-protein phosphatase 2A 56 kDa regulatory subunit delta isoform	IPI00000030	0.944	0.05197	2	1	3	3
323	Calcium-binding protein 39-like	IPI00026359	0.944	0.05197	2	1	2	4
324	D-tyrosyl-tRNA(Tyr) deacylase 1	IPI00152692	0.944	0.05197	2	1	3	3
325	Pre-mRNA-processing factor 6	IPI00305068	0.944	0.05197	2	1	2	4
326	Isoform 1 of Lysine-specific demethylase 5C	IPI00013185	0.944	0.05197	1	2	3	3
327	Isoform 1 of Translation initiation factor eIF-2B subunit gamma	IPI00006504	0.944	0.05197	2	1	4	2
328	N-alpha-acetyltransferase 20, NatB catalytic subunit	IPI00007174	0.944	0.05197	0	2	3	3
329	Uncharacterized protein C10orf58	IPI00296190	0.944	0.05197	1	2	3	3
330	26S proteasome non-ATPase regulatory subunit 14	IPI00024821	0.938	0.05215	14	13	16	17
331	Isoform 1 of PC4 and SFRS1-interacting protein	IPI00028122	0.934	0.05215	3	5	4	8
332	Isoform 1 of Transcription elongation regulator 1	IPI00247871	0.934	0.05215	5	3	6	6
333	Glucosamine--fructose-6-phosphate aminotransferase [isomerizing] 2	IPI00216159	0.934	0.05215	4	4	7	5
334	Isoform 1 of Filamin-C	IPI00178352	0.927	0.05551	14	14	16	18
335	Lon protease homolog, mitochondrial	IPI00005158	0.916	0.05626	13	16	18	17
336	Threonyl-tRNA synthetase, cytoplasmic	IPI00329633	0.915	0.05626	36	31	38	37
337	Isoform 1 of Malignant T cell-amplified sequence 1	IPI00179026	0.906	0.05655	16	14	18	18
338	10 kDa heat shock protein, mitochondrial	IPI00220362	0.902	0.05683	4	5	4	9
339	Huntingtin	IPI00002335	0.902	0.05683	4	5	5	8
340	Transmembrane protein 126A	IPI00031064	0.902	0.05683	4	5	7	6
341	Inositol monophosphatase 1	IPI00020906	0.902	0.05683	7	2	7	6
342	Cytoplasmic aconitate hydratase	IPI00008485	0.902	0.05683	4	5	6	7
343	Aldo-keto reductase family 1 member C3	IPI00291483	0.902	0.05683	5	4	5	8
344	Isoform 1 of Protein phosphatase 1 regulatory subunit 12A	IPI00183002	0.902	0.05683	4	5	7	6
345	Asparagine synthetase [glutamine-hydrolyzing]	IPI00554777	0.898	0.05693	9	9	11	12
346	coatomer subunit epsilon isoform b	IPI00399318	0.898	0.05693	7	11	11	12
347	Isoform 1 of Electron transfer flavoprotein subunit beta	IPI00004902	0.896	0.05693	14	17	17	20
348	UMP-CMP kinase isoform a	IPI00219953	0.896	0.05693	17	14	19	18
349	Hypoxia up-regulated protein 1	IPI00000877	0.893	0.05693	35	37	40	40
350	Transcription factor A, mitochondrial	IPI00020928	0.882	0.05745	9	10	13	11
351	Protein FAM49B	IPI00303318	0.882	0.05745	8	11	13	11
352	Electron transfer flavoprotein subunit alpha, mitochondrial	IPI00010810	0.881	0.05745	26	25	28	30
353	Ubiquitin carboxyl-terminal hydrolase 14	IPI00219913	0.878	0.05745	14	19	19	20
354	Isoform 2 of TIP41-like protein	IPI00641815	0.874	0.05759	2	2	4	3
355	Periplakin	IPI00298057	0.874	0.05759	1	3	4	3
356	Transmembrane protein 2	IPI00170706	0.874	0.05759	3	1	3	4
357	Protein TFG	IPI00294619	0.874	0.05759	2	2	4	3
358	Isoform 1 of Acylglycerol kinase, mitochondrial	IPI00019353	0.874	0.05759	2	2	3	4
359	Isoform 2 of Bromodomain adjacent to zinc finger domain protein 2A	IPI00296388	0.874	0.05759	3	1	3	4
360	Ras-related protein Rap-2b	IPI00018364	0.874	0.05759	3	1	3	4
361	ADP-ribosylation factor-like protein 8A	IPI00060031	0.874	0.05759	2	2	3	4
362	U2 small nuclear ribonucleoprotein B''	IPI00029267	0.873	0.05886	4	6	6	8
363	Isoform 2 of Cytosolic non-specific dipeptidase	IPI00165579	0.873	0.05886	4	6	6	8
364	Mannosyl-oligosaccharide glucosidase	IPI00328170	0.873	0.05886	5	5	9	5
365	Isoform 5 of Thioredoxin reductase 1, cytoplasmic	IPI00554786	0.873	0.05886	5	5	8	6
366	Isoform 1 of Cytoplasmic FMR1-interacting protein 1	IPI00644231	0.873	0.05886	6	4	8	6
367	Acylamino-acid-releasing enzyme	IPI00337741	0.873	0.05886	5	5	7	7
368	Diablo homolog, mitochondrial precursor	IPI00008418	0.873	0.05886	5	5	6	8
369	3-ketoacyl-CoA thiolase, mitochondrial	IPI00001539	0.873	0.05886	5	5	8	6
370	Peptidyl-prolyl cis-trans isomerase H	IPI00007346	0.873	0.05886	6	4	7	7
371	Glucosamine 6-phosphate N-acetyltransferase	IPI00061525	0.854	0.06316	11	10	12	14
372	26S proteasome non-ATPase regulatory subunit 5	IPI00002134	0.854	0.06316	10	11	11	15
373	Isoform A of AP-1 complex subunit beta-1	IPI00328257	0.853	0.06344	20	16	20	22
374	Isoform Cytoplasmic of Lysyl-tRNA synthetase	IPI00014238	0.850	0.06359	40	43	41	50

No.	Description	Accession number	STN[1]	p-Value[1]	Con_A[2]	Con_B[2]	CUR_A[2]	CUR_B[2]
375	Nuclear pore complex protein Nup133	IPI00291200	0.848	0.06359	8	3	7	8
376	Isoleucyl-tRNA synthetase	IPI00514082	0.848	0.06359	5	6	7	8
377	cDNA FLJ75085, highly similar to Homo sapiens glutaminyl-tRNA synthetase (QARS), mRNA	IPI00026665	0.848	0.06359	5	6	9	6
378	Calcium-binding mitochondrial carrier protein Aralar1	IPI00386271	0.848	0.06359	5	6	7	8
379	WD repeat-containing protein 36	IPI00169325	0.830	0.06430	18	21	20	25
380	Vesicular integral-membrane protein VIP36	IPI00009950	0.825	0.06444	6	6	8	8
381	Probable ATP-dependent RNA helicase DDX23	IPI00006725	0.825	0.06444	4	8	9	7
382	Putative ATP-dependent Clp protease proteolytic subunit, mitochondrial	IPI00003870	0.825	0.06444	5	7	8	8
383	Isoform Complexed of Arginyl-tRNA synthetase, cytoplasmic	IPI00004860	0.823	0.06704	20	20	23	23
384	Ras-related protein Rap-1b	IPI00015148	0.823	0.06704	20	20	21	25
385	COP9 signalosome complex subunit 6	IPI00163230	0.820	0.06732	2	3	4	4
386	Transcription initiation factor IIE subunit beta	IPI00019981	0.820	0.06732	3	2	4	4
387	Isoform 1 of Pogo transposable element with ZNF domain	IPI00410717	0.820	0.06732	2	3	3	5
388	Isoform 1 of Alanine aminotransferase 2	IPI00152432	0.820	0.06732	3	2	4	4
389	Microsomal glutathione S-transferase 1	IPI00021805	0.820	0.06732	3	2	4	4
390	Isoform 1 of Proteasome assembly chaperone 1	IPI00030770	0.820	0.06732	3	2	5	3
391	Huntingtin-interacting protein 1	IPI00782965	0.820	0.06732	3	2	5	3
392	Isoform 1 of CD109 antigen	IPI00152540	0.820	0.06732	2	3	4	4
393	Dehydrogenase/reductase SDR family member on chromosome X	IPI00166860	0.820	0.06732	3	2	5	3
394	Major centromere autoantigen B	IPI00010388	0.820	0.06732	3	2	5	3
395	RNA 3'-terminal phosphate cyclase-like protein	IPI00294229	0.820	0.06732	2	3	4	4
396	Putative uncharacterized protein NOP2	IPI00294891	0.818	0.06803	12	12	14	15
397	Splicing factor 3A subunit 1	IPI00017451	0.818	0.06803	12	12	14	15
398	X-ray repair cross-complementing protein 5	IPI00220834	0.808	0.06864	88	92	100	90
399	Probable phosphoglycerate mutase 4	IPI00374975	0.807	0.06869	12	13	16	14
400	Isoform E of Eukaryotic translation initiation factor 4 gamma 1	IPI00386533	0.807	0.06869	12	13	15	15
401	Proteasome 26S non-ATPase subunit 11 variant (Fragment)	IPI00105598	0.807	0.06869	12	13	16	14
402	Succinyl-CoA:3-ketoacid-coenzyme A transferase 1, mitochondrial	IPI00026516	0.807	0.06869	13	12	16	14
403	Echinoderm microtubule-associated protein-like 4	IPI00001466	0.805	0.06874	6	7	7	10
404	cDNA FLJ53975, highly similar to Acetyl-CoA acetyltransferase, cytosolic	IPI00291419	0.805	0.06874	7	6	8	9
405	Isoform Long of ES1 protein homolog, mitochondrial	IPI00024913	0.805	0.06874	5	8	8	9
406	GTP:AMP phosphotransferase mitochondrial	IPI00465256	0.805	0.06874	7	6	9	8
407	Isoform Beta of Lamina-associated polypeptide 2, isoforms beta/gamma	IPI00030131	0.796	0.06944	13	13	16	15
408	N-acetyltransferase 10	IPI00300127	0.791	0.06968	25	20	25	26
409	Thioredoxin-dependent peroxide reductase, mitochondrial	IPI00024919	0.791	0.06968	23	22	26	25
410	Copine-1	IPI00018452	0.786	0.06973	12	15	16	16
411	Isoform 1 of Protein virilizer homolog	IPI00036742	0.786	0.06992	6	8	10	8
412	Isoform 2 of Splicing factor 1	IPI00294627	0.779	0.07275	23	24	27	26
413	CLASP2 protein	IPI00168165	0.778	0.07275	3	3	5	4
414	Peptidyl-tRNA hydrolase 2, mitochondrial	IPI00032903	0.778	0.07275	3	3	5	4
415	cDNA FLJ55034	IPI00384122	0.778	0.07275	3	3	4	5
416	28S ribosomal protein S31, mitochondrial	IPI00294242	0.778	0.07275	4	2	5	4
417	Isoform p26 of 7,8-dihydro-8-oxoguanine triphosphatase	IPI00004392	0.778	0.07275	3	3	5	4
418	ribonucleoprotein PTB-binding 1	IPI00217661	0.769	0.07516	7	8	9	10
419	Uncharacterized protein C2orf47, mitochondrial	IPI00291751	0.769	0.07516	9	6	9	10
420	3-mercaptopyruvate sulfurtransferase	IPI00165360	0.769	0.07516	9	6	9	10
421	Leukocyte elastase inhibitor	IPI00027444	0.759	0.07563	16	14	17	18
422	Lamin-B receptor	IPI00292135	0.758	0.07563	27	24	26	31
423	Isoform 2 of Exosome complex exonuclease RRP44	IPI00183462	0.753	0.07601	7	9	10	10
424	Asparaginyl-tRNA synthetase, cytoplasmic	IPI00306960	0.751	0.07781	16	15	18	18
425	SF3A2 protein (Fragment)	IPI00017341	0.742	0.07866	5	2	5	5
426	Core histone macro-H2A.2	IPI00220994	0.742	0.07866	2	5	3	7
427	Protein LYRIC	IPI00328715	0.742	0.07866	3	4	7	3
428	Heat shock 70 kDa protein 14	IPI00292499	0.742	0.07866	4	3	5	5
429	Cystatin-B	IPI00021828	0.742	0.07866	4	3	5	5
430	Isoform 1 of Spermatid perinuclear RNA-binding protein	IPI00169430	0.742	0.07866	4	3	4	6
431	Thioredoxin-interacting protein	IPI00007956	0.742	0.07866	2	5	6	4
432	Actin-related protein 2/3 complex subunit 2	IPI00005161	0.738	0.07984	8	9	9	12
433	Probable O-sialoglycoprotein endopeptidase	IPI00015809	0.729	0.08022	1	1	2	2
434	Isoform 1 of FAD synthase	IPI00220299	0.729	0.08022	1	0	3	1
435	cDNA FLJ61739, highly similar to Serine/arginine repetitive matrix protein 1	IPI00328293	0.729	0.08022	1	1	1	3
436	Golgin subfamily B member 1	IPI00004671	0.729	0.08022	1	1	2	2
437	Isoform 2 of Vacuolar protein sorting-associated protein 13D	IPI00783859	0.729	0.08022	0	0	1	3
438	Bifunctional 3'-phosphoadenosine 5'-phosphosulfate synthase 1	IPI00011619	0.729	0.08022	0	1	2	2
439	cDNA FLJ45232 fis, clone BRCAN2021718	IPI00170877	0.729	0.08022	0	1	2	2
440	Isoform 2 of Tumor protein D54	IPI00221178	0.729	0.08022	0	1	2	2
441	Isoform 1 of Serum paraoxonase/arylesterase 2	IPI00014958	0.729	0.08022	1	1	2	2
442	Conserved hypothetical protein	IPI00477526	0.729	0.08022	1	1	2	2
443	Probable cysteinyl-tRNA synthetase, mitochondrial	IPI00336016	0.729	0.08022	0	1	2	2
444	Ribonucleases P/MRP protein subunit POP1	IPI00293331	0.729	0.08022	0	0	3	0
445	Isoform 1 of U3 small nucleolar RNA-associated protein 14 homolog A	IPI00107113	0.729	0.08022	0	1	0	3
446	Probable asparaginyl-tRNA synthetase, mitochondrial	IPI00101664	0.729	0.08022	1	1	1	3
447	Isoform 1 of Heterogeneous nuclear ribonucleoprotein L-like	IPI00103247	0.729	0.08022	1	1	1	3
448	Isoform 1 of WD repeat-containing protein 26	IPI00414197	0.729	0.08022	0	1	2	2
449	Cell division cycle protein 123 homolog	IPI00005670	0.729	0.08022	1	0	2	2
450	Isoform 2 of Carbohydrate kinase domain-containing protein	IPI00645172	0.729	0.08022	0	0	2	2
451	Isoform 2 of Myosin-XVIIIa	IPI00334410	0.729	0.08022	1	0	2	2
452	UPF0428 protein CXorf56	IPI00005055	0.729	0.08022	1	1	1	3
453	Exocyst complex component 4	IPI00059279	0.729	0.08022	0	0	1	3
454	EPS8L2 protein	IPI00414315	0.729	0.08022	1	0	3	1
455	WASH complex subunit strumpellin	IPI00029175	0.729	0.08022	0	0	3	1
456	Procollagen galactosyltransferase 1	IPI00168262	0.729	0.08022	0	1	1	3
457	Nucleolar complex protein 2 homolog	IPI00411886	0.729	0.08022	0	0	3	1
458	cDNA FLJ54030, highly similar to Polymerase delta-interacting protein 3	IPI00440688	0.729	0.08022	1	0	3	1
459	Agrin	IPI00374563	0.729	0.08022	0	0	2	2
460	Gamma-soluble NSF attachment protein	IPI00293817	0.729	0.08022	1	1	2	2
461	Isoform 1 of Caldesmon	IPI00014516	0.729	0.08022	0	1	2	2
462	Protein Red	IPI00011875	0.729	0.08022	0	1	2	2
463	Isoform 3 of Tyrosine-protein kinase-like 7	IPI00168813	0.729	0.08022	0	1	3	1
464	Isoform 1 of NHL repeat-containing protein 2	IPI00301051	0.729	0.08022	0	1	2	2
465	Isoform Long of Ancient ubiquitous protein 1	IPI00001891	0.729	0.08022	1	1	2	2
466	Isoform 2 of WASH complex subunit FAM21C	IPI00456853	0.729	0.08022	1	1	3	1
467	Mitochondrial fission 1 protein	IPI00007052	0.729	0.08022	1	1	2	2
468	Putative uncharacterized protein DKFZp686C1054	IPI00465054	0.729	0.08022	0	1	2	2
469	Threonine synthase-like 1	IPI00016287	0.729	0.08022	1	1	2	2

No.	Description	Accession number	STN[1]	p-Value[1]	Con_A[2]	Con_B[2]	CUR_A[2]	CUR_B[2]
470	Isoform 1 of Adaptin ear-binding coat-associated protein 2	IPI00018188	0.729	0.08022	1	1	2	2
471	Macrophage-capping protein	IPI00027341	0.729	0.08022	0	0	2	2
472	Derlin-2	IPI00304264	0.729	0.08022	1	1	3	1
473	Isoform 2 of DNA-3-methyladenine glycosylase	IPI00218495	0.729	0.08022	0	1	2	2
474	La-related protein 7	IPI00294742	0.729	0.08022	0	0	2	2
475	NF-kappa-B-repressing factor	IPI00005675	0.729	0.08022	1	1	2	2
476	Isoform 5 of Methyltransferase-like protein 13	IPI00384061	0.729	0.08022	1	1	2	2
477	Isoform 2 of Rho guanine nucleotide exchange factor 1	IPI00339379	0.729	0.08022	0	0	3	0
478	Sulfotransferase 1A1	IPI00300026	0.729	0.08022	0	0	0	3
479	Vesicle-associated membrane protein 3	IPI00549343	0.729	0.08022	1	1	3	1
480	Coiled-coil domain-containing protein 58	IPI00046828	0.729	0.08022	0	0	1	3
481	Zinc finger, MYM-type 6, isoform CRA_a	IPI00168438	0.729	0.08022	0	1	2	2
482	Golgi resident protein GCP60	IPI00009315	0.729	0.08022	0	0	1	3
483	Hermansky-Pudlak syndrome 6 protein	IPI00015505	0.729	0.08022	0	0	2	2
484	TC4 protein	IPI00044779	0.728	0.10010	17	17	15	24
485	transcription activator BRG1 isoform D	IPI00029822	0.728	0.10010	20	14	21	18
486	cDNA FLJ56285, highly similar to ADP-ribosylation factor-like protein 8B	IPI00018871	0.724	0.10058	9	9	11	11
487	Isoform 2 of Signal recognition particle 68 kDa protein	IPI00102936	0.724	0.10058	11	7	11	11
488	Isoform 1 of Nuclear pore membrane glycoprotein 210	IPI00291755	0.721	0.10223	29	30	33	32
489	Heat shock 70 kDa protein 4	IPI00002966	0.714	0.10261	48	46	51	50
490	Isoform 2 of Dedicator of cytokinesis protein 7	IPI00183572	0.712	0.10261	3	5	6	5
491	YLP motif-containing protein 1	IPI00165434	0.712	0.10261	4	4	6	5
492	Biliverdin reductase A	IPI00294158	0.712	0.10261	4	4	6	5
493	Translational activator of cytochrome c oxidase 1	IPI00019903	0.712	0.10261	5	3	5	6
494	Coronin-1B	IPI00007058	0.712	0.10261	4	4	6	5
495	U6 snRNA-associated Sm-like protein LSm4	IPI00294955	0.712	0.10261	4	4	5	6
496	Isoform 2 of Phosphoenolpyruvate carboxykinase [GTP], mitochondrial	IPI00384116	0.712	0.10261	4	4	6	5
497	Isoform 2 of Splicing factor, arginine/serine-rich 12	IPI00375462	0.712	0.10261	4	4	6	5
498	Putative pre-mRNA-splicing factor ATP-dependent RNA helicase DHX15	IPI00396435	0.712	0.10303	28	33	36	31
499	Signal recognition particle 72 kDa protein	IPI00215888	0.712	0.10303	11	8	11	12
500	Calpain-1 catalytic subunit	IPI00011285	0.707	0.10332	17	20	23	19
501	L-lactate dehydrogenase B chain	IPI00219217	0.704	0.10341	163	184	164	194
502	Hypoxanthine-guanine phosphoribosyltransferase	IPI00218493	0.701	0.10369	33	31	37	33
503	Peptidyl-prolyl cis-trans isomerase FKBP11	IPI00009885	0.700	0.10384	9	11	12	12
504	2-oxoglutarate dehydrogenase, mitochondrial	IPI00098902	0.700	0.10384	10	10	14	10
505	Alpha-centractin	IPI00029468	0.700	0.10384	9	11	11	13
506	Translocon-associated protein subunit delta precursor	IPI00019385	0.700	0.10384	10	10	12	12
507	Isoform Mitochondrial of Fumarate hydratase, mitochondrial	IPI00296053	0.700	0.10384	10	10	13	11
508	Splicing factor 3B subunit 1	IPI00026089	0.689	0.10582	55	49	51	60
509	annexin A4	IPI00793199	0.689	0.10582	20	20	23	22
510	Spermidine synthase	IPI00292020	0.688	0.10582	11	10	12	13
511	Mitochondrial import receptor subunit TOM70	IPI00015602	0.687	0.10582	5	4	4	8
512	Thyroid hormone receptor-associated protein 3	IPI00104050	0.687	0.10582	5	4	6	6
513	UPF0160 protein MYG1, mitochondrial	IPI00029444	0.687	0.10582	4	5	7	5
514	Protein C20orf11	IPI00016634	0.687	0.10582	5	4	6	6
515	Isoform 1 of Polyadenylate-binding protein 4	IPI00012726	0.687	0.10582	6	3	5	7
516	Aldo-keto reductase family 1 member C1	IPI00029733	0.687	0.10582	4	5	8	4
517	3-hydroxyisobutyrate dehydrogenase, mitochondrial	IPI00013860	0.687	0.10582	4	5	7	5
518	14-3-3 protein epsilon	IPI00000816	0.683	0.10676	233	248	236	257
519	Exportin-1	IPI00298961	0.682	0.10681	46	61	60	54
520	DNA-directed RNA polymerase II subunit RPB2	IPI00027808	0.678	0.10691	12	10	12	14
521	Tripeptidyl-peptidase 2	IPI00020416	0.672	0.10752	22	21	23	25
522	THO complex subunit 2	IPI00158615	0.668	0.10757	12	11	13	14
523	ADP-ribosylation factor 6	IPI00215920	0.668	0.10757	12	11	13	14
524	Long-chain-fatty-acid--CoA ligase 3	IPI00031397	0.664	0.10799	5	5	4	9
525	Importin-11	IPI00301107	0.664	0.10799	6	4	5	8
526	FAS-associated factor 2	IPI00172656	0.664	0.10799	5	5	5	8
527	Isoform 2 of ATP-binding cassette sub-family F member 1	IPI00013495	0.664	0.10799	6	4	7	6
528	Isoform 1 of tRNA (adenine-N(1)-)-methyltransferase non-catalytic subunit TRM6	IPI00099311	0.664	0.10799	4	6	5	8
529	Isoform 2 of PERQ amino acid-rich with GYF domain-containing protein 2	IPI00647635	0.664	0.10799	3	7	6	7
530	Paladin	IPI00297212	0.664	0.10799	4	6	7	6
531	Isoform 1 of Armadillo repeat-containing protein 10	IPI00166394	0.664	0.10799	5	5	6	7
532	Isoform Long of FAS-associated factor 1	IPI00070643	0.664	0.10799	6	4	5	8
533	Rho GTPase-activating protein 1	IPI00020567	0.653	0.11054	2	1	3	2
534	Isoform 1 of Dynamin-like 120 kDa protein, mitochondrial	IPI00006721	0.653	0.11054	1	2	2	3
535	Band 4.1-like protein 2	IPI00015973	0.653	0.11054	2	1	2	3
536	Epoxide hydrolase 1	IPI00009896	0.653	0.11054	2	1	3	2
537	Isoform 1 of Mixed lineage kinase domain-like protein	IPI00180781	0.653	0.11054	1	2	3	2
538	Thioredoxin-like protein 4A	IPI00216338	0.653	0.11054	2	0	2	3
539	Epidermal growth factor receptor kinase substrate 8	IPI00290337	0.653	0.11054	0	2	3	2
540	Isoform 1 of Dehydrogenase/reductase SDR family member 7	IPI00006957	0.653	0.11054	1	2	2	3
541	Uncharacterized protein C3orf26	IPI00031679	0.653	0.11054	1	2	3	2
542	mesencephalic astrocyte-derived neurotrophic factor	IPI00328748	0.653	0.11054	0	2	2	3
543	Heterochromatin protein 1, binding protein 3	IPI00640417	0.653	0.11054	1	2	2	3
544	Lanosterol synthase	IPI00009747	0.653	0.11054	1	2	2	3
545	Isoform 1 of 182 kDa tankyrase-1-binding protein	IPI00304589	0.653	0.11054	2	1	2	3
546	Isoform 1 of Serine protease HTRA2, mitochondrial	IPI00001663	0.653	0.11054	1	2	2	3
547	Isoform 1 of Dynamin-1	IPI00413140	0.653	0.11054	2	1	2	3
548	Glutathione synthetase	IPI00010706	0.652	0.11054	25	22	27	25
549	Cleavage stimulation factor subunit 3	IPI00015195	0.644	0.11083	6	5	8	6
550	Nuclear pore complex protein Nup93	IPI00397904	0.644	0.11083	4	7	7	7
551	Cell division cycle 5-like protein	IPI00465294	0.644	0.11083	3	8	4	10
552	Ribonuclease P protein subunit p30	IPI00019196	0.644	0.11083	5	6	7	7
553	Isoform 1 of Insulin-like growth factor 2 mRNA-binding protein 3	IPI00658000	0.644	0.11083	6	5	7	7
554	Mitochondrial import inner membrane translocase subunit Tim16	IPI00218463	0.644	0.11083	5	6	8	6
555	Structural maintenance of chromosomes protein 1A	IPI00291939	0.633	0.11234	26	25	30	26
556	Isoform 1 of Isocitrate dehydrogenase [NAD] subunit alpha, mitochondrial	IPI00030702	0.633	0.11234	24	27	28	28
557	V-type proton ATPase subunit B, brain isoform	IPI00007812	0.633	0.11234	16	11	15	16
558	cDNA FLJ56425, highly similar to Very-long-chain specific acyl-CoAdehydrogenase, mitochondrial	IPI00028031	0.629	0.11248	28	24	26	31
559	Isoform 1 of Transmembrane and coiled-coil domain-containing protein 1	IPI00026111	0.626	0.11286	6	6	7	8
560	GTP-binding protein SAR1b	IPI00002149	0.626	0.11286	7	5	8	7
561	Translocated promoter region	IPI00514531	0.626	0.11286	7	5	8	7
562	UBX domain-containing protein 4	IPI00293946	0.626	0.11286	6	6	8	7
563	WD repeat-containing protein 61	IPI00019269	0.625	0.11428	14	14	17	15
564	Sepiapterin reductase	IPI00017469	0.618	0.11513	14	15	16	17

No.	Description	Accession number	STN[1]	p-Value[1]	Con_A[2]	Con_B[2]	CUR_A[2]	CUR_B[2]
565	Phosphoribosylformylglycinamidine synthase	IPI00004534	0.617	0.11527	29	26	33	27
566	FACT complex subunit SSRP1	IPI00005154	0.611	0.11602	16	14	17	17
567	Prolyl endopeptidase	IPI00008164	0.611	0.11602	17	13	18	16
568	Isoform 1 of Cell division cycle and apoptosis regulator protein 1	IPI00217357	0.610	0.11683	6	7	5	11
569	Vesicle transport protein GOT1B	IPI00007061	0.610	0.11683	7	6	7	9
570	Alcohol dehydrogenase [NADP+]	IPI00220271	0.610	0.11683	6	7	8	8
571	Dolichyldiphosphatase 1	IPI00329410	0.610	0.11683	8	5	7	9
572	Isoform 1 of E3 UFM1-protein ligase 1	IPI00844000	0.610	0.11683	5	8	8	8
573	ADP-ribosylation factor-like protein 2	IPI00003326	0.610	0.11683	6	7	8	8
574	Phosphoserine phosphatase	IPI00019178	0.610	0.11683	6	7	9	7
575	Fructose-bisphosphate aldolase	IPI00418262	0.610	0.11683	7	6	8	8
576	Desmoglein-2	IPI00028931	0.600	0.11777	3	1	3	3
577	Isoform 2 of Integrator complex subunit 3	IPI00418336	0.600	0.11777	2	2	3	3
578	24-dehydrocholesterol reductase	IPI00016703	0.600	0.11777	2	2	4	2
579	Isoform 1 of Pentatricopeptide repeat-containing protein 3, mitochondrial	IPI00783302	0.600	0.11777	2	2	3	3
580	Protein FAM162A	IPI00023001	0.600	0.11777	2	2	2	4
581	G patch domain and KOW motifs-containing protein	IPI00024255	0.600	0.11777	2	2	3	3
582	Talin-2	IPI00219299	0.600	0.11777	2	2	2	4
583	Isoform 1 of Uncharacterized protein KIAA0528	IPI00465142	0.600	0.11777	2	2	3	3
584	Isoform 1 of Nicalin	IPI00470649	0.600	0.11777	2	2	2	4
585	Alpha-ketoglutarate dehydrogenase complex dihydrolipoyl succinyltransferase	IPI00033034	0.600	0.11777	2	2	3	3
586	Isoform 1 of Signal transducing adapter molecule 1	IPI00020178	0.600	0.11777	2	2	3	3
587	Isoform 1 of Ligatin	IPI00013160	0.600	0.11777	1	3	3	3
588	Ras-related protein Rab-32	IPI00014377	0.600	0.11777	2	2	3	3
589	Isoform 1 of Lariat debranching enzyme	IPI00305545	0.600	0.11777	2	2	3	3
590	Isoform 5 of Myosin-14	IPI00029818	0.596	0.13497	6	8	8	9
591	Isoform 1 of RNA-binding protein 25	IPI00004273	0.596	0.13497	6	8	9	8
592	TRMT61A protein (Fragment)	IPI00059718	0.596	0.13497	7	7	10	7
593	Eukaryotic translation initiation factor 3 subunit J	IPI00290461	0.596	0.13497	7	7	9	8
594	ATP-dependent RNA helicase DDX1	IPI00293655	0.592	0.13544	29	33	31	36
595	cDNA FLJ59758, highly similar to S-methyl-5-thioadenosine phosphorylase	IPI00011876	0.591	0.13549	17	16	18	19
596	14-3-3 protein gamma	IPI00220642	0.585	0.13558	17	17	18	20
597	Nuclear cap-binding protein subunit 1	IPI00019380	0.582	0.13634	6	9	9	9
598	Proteasome subunit beta type-5	IPI00479306	0.579	0.13719	18	17	18	21
599	histone deacetylase complex subunit SAP18	IPI00011698	0.570	0.13846	10	6	9	10
600	Midasin	IPI00167941	0.568	0.13889	17	20	23	18
601	Small subunit processome component 20 homolog	IPI00004970	0.563	0.13894	20	18	22	20
602	1,4-alpha-glucan-branching enzyme	IPI00296635	0.561	0.13974	2	3	3	4
603	Glutamate--cysteine ligase catalytic subunit	IPI00215768	0.561	0.13974	2	3	4	3
604	Tetratricopeptide repeat protein 37	IPI00005634	0.561	0.13974	2	3	5	2
605	negative elongation factor A	IPI00394679	0.561	0.13974	2	3	4	3
606	Isoform 1 of Apoptosis-inducing factor 2	IPI00013909	0.561	0.13974	3	2	4	3
607	Sorting nexin-9	IPI00001883	0.561	0.13974	2	3	3	4
608	Isoform 2 of Nitrilase homolog 1	IPI00023779	0.561	0.13974	3	2	4	3
609	Actin-related protein 2/3 complex subunit 3	IPI00005162	0.559	0.13974	8	9	9	11
610	Tubulin--tyrosine ligase-like protein 12	IPI00029048	0.548	0.14101	21	20	22	23
611	Isoform ASF-1 of Splicing factor, arginine/serine-rich 1	IPI00215884	0.544	0.14182	40	39	40	44
612	Isoform 1 of RuvB-like 1	IPI00021187	0.540	0.14187	23	20	25	22
613	Transmembrane emp24 domain-containing protein 9	IPI00023542	0.530	0.14267	4	2	5	3
614	Aminoacyl tRNA synthase complex-interacting multifunctional protein 1	IPI00006252	0.530	0.14267	3	3	4	4
615	DNA-directed RNA polymerase III subunit RPC1	IPI00024163	0.530	0.14267	3	3	4	4
616	Integrator complex subunit 2	IPI00477759	0.530	0.14267	3	3	4	4
617	Transmembrane emp24 domain-containing protein 5	IPI00294472	0.530	0.14267	3	3	3	5
618	Signal recognition particle receptor subunit alpha	IPI00385267	0.530	0.14267	4	2	4	4
619	magnesium transporter protein 1	IPI00301202	0.530	0.14267	2	4	3	5
620	Sorting and assembly machinery component 50 homolog	IPI00412713	0.530	0.14267	3	3	4	4
621	Isoform A of Peptidyl-prolyl cis-trans isomerase E	IPI00009316	0.530	0.14267	2	4	4	4
622	Mimitin, mitochondrial	IPI00031109	0.530	0.14267	3	3	4	4
623	Isoform 1 of Serine/threonine-protein phosphatase 2A 55 kDa regulatory subunit B beta isoform	IPI00020850	0.530	0.14267	3	3	4	4
624	Translation initiation factor eIF-2B subunit epsilon	IPI00011898	0.530	0.14267	3	3	4	4
625	Protein	IPI00892529	0.530	0.14267	1	5	4	4
626	Isoform 2 of Choline-phosphate cytidylyltransferase B	IPI00001562	0.530	0.14267	2	4	4	4
627	NADH dehydrogenase [ubiquinone] iron-sulfur protein 4, mitochondrial	IPI00011217	0.530	0.14267	3	3	4	4
628	Prefoldin subunit 2	IPI00006052	0.530	0.14267	3	3	4	4
629	cDNA FLJ59739, highly similar to Protein transport protein Sec61 subunit alpha isoform 1	IPI00218466	0.529	0.15637	9	11	12	11
630	NADH-ubiquinone oxidoreductase 75 kDa subunit	IPI00604664	0.529	0.15637	8	12	10	13
631	Cleavage and polyadenylation specificity factor subunit 1	IPI00026219	0.520	0.15698	10	11	13	11
632	Proteasome subunit beta type-1	IPI00025019	0.508	0.15887	26	25	26	29
633	S-formylglutathione hydrolase	IPI00411706	0.505	0.15920	3	4	3	6
634	Isoform 1 of Neuroblastoma-amplified sequence	IPI00333913	0.505	0.15920	6	1	4	5
635	RNA-binding protein 28	IPI00304187	0.505	0.15920	3	4	5	4
636	Carnitine O-palmitoyltransferase 2, mitochondrial	IPI00012912	0.505	0.15920	3	4	4	5
637	Twinfilin-2	IPI00550917	0.505	0.15920	4	3	5	4
638	Isoform 1 of Lysocardiolipin acyltransferase 1	IPI00419643	0.505	0.15920	4	3	4	5
639	Isoform 1 of Low molecular weight phosphotyrosine protein phosphatase	IPI00219861	0.505	0.15920	3	4	5	4
640	Thioredoxin	IPI00216298	0.504	0.15920	12	11	13	13
641	Protein RRP5 homolog	IPI00400922	0.492	0.16010	25	31	30	30
642	Annexin A3	IPI00024095	0.489	0.16048	27	30	29	32
643	Heat shock protein 75 kDa, mitochondrial	IPI00030275	0.487	0.16048	56	52	55	58
644	Aldose reductase	IPI00413641	0.484	0.16081	4	4	5	5
645	Activating signal cointegrator 1 complex subunit 3	IPI00430472	0.484	0.16081	4	4	7	3
646	Isoform 1 of Rab3 GTPase-activating protein non-catalytic subunit	IPI00554590	0.484	0.16081	4	4	4	6
647	Isoform 1 of Cell division cycle protein 23 homolog	IPI00005822	0.484	0.16081	5	3	6	4
648	Isoform 3 of Protein PRRC1	IPI00217053	0.484	0.16081	3	5	5	5
649	cDNA FLJ56184, highly similar to Proto-oncogene tyrosine-protein kinase LCK	IPI00394952	0.484	0.16081	5	3	5	5
650	U3 small nucleolar ribonucleoprotein protein IMP3	IPI00019488	0.484	0.16081	4	4	5	5
651	Carbonyl reductase [NADPH] 3	IPI00290462	0.484	0.16081	4	4	4	6
652	Isoform 1 of Apolipoprotein O	IPI00042580	0.484	0.16081	5	3	5	5
653	ADP-ribosylation factor 4	IPI00215918	0.484	0.17153	12	14	13	16
654	Isoform 1 of Proteasome activator complex subunit 4	IPI00005260	0.477	0.17205	13	14	18	12
655	SUMO-activating enzyme subunit 2	IPI00023234	0.472	0.17281	13	15	14	17
656	Phosphoserine aminotransferase	IPI00001734	0.466	0.17361	16	13	16	16
657	Microsomal glutathione S-transferase 3	IPI00024266	0.466	0.17361	13	16	17	15
658	Galectin-1	IPI00219219	0.466	0.17361	14	15	14	18
659	Nucleolar GTP-binding protein 1	IPI00385042	0.465	0.17404	4	5	6	5

No.	Description	Accession number	STN[1]	p-Value[1]	Con_A[2]	Con_B[2]	CUR_A[2]	CUR_B[2]
660	Peroxisomal multifunctional enzyme type 2	IPI00019912	0.465	0.17404	5	4	5	6
661	28S ribosomal protein S25, mitochondrial	IPI00013167	0.465	0.17404	5	4	5	6
662	Myeloid-associated differentiation marker	IPI00102685	0.465	0.17404	5	4	6	5
663	Phosphatidylethanolamine-binding protein 1	IPI00219446	0.465	0.17404	36	30	35	35
664	Isoform 2 of Nuclear protein localization protein 4 homolog	IPI00001676	0.450	0.17517	15	17	19	16
665	Isoform 1 of Cysteine and histidine-rich domain-containing protein 1	IPI00015897	0.450	0.17531	5	5	6	6
666	Isoform 2 of Lysine-specific histone demethylase 1A	IPI00217540	0.450	0.17531	4	6	5	7
667	Isoform 1 of Sorting nexin-12	IPI00438170	0.450	0.17531	5	5	7	5
668	Isoform 2 of U1 small nuclear ribonucleoprotein 70 kDa	IPI00219483	0.450	0.17531	4	6	7	5
669	Isoform 1 of Porphobilinogen deaminase	IPI00028160	0.450	0.17531	5	5	6	6
670	Xaa-Pro dipeptidase	IPI00257882	0.450	0.17531	5	5	4	8
671	Signal peptidase complex subunit 3	IPI00300299	0.450	0.17531	5	5	4	8
672	cDNA FLJ54492, highly similar to Eukaryotic translation initiation factor 4B	IPI00012079	0.445	0.18490	16	17	17	19
673	Isoform 1 of ATP-dependent RNA helicase DDX42	IPI00409671	0.436	0.18622	8	3	8	5
674	Ras suppressor protein 1	IPI00017256	0.436	0.18622	3	8	5	8
675	Isoform 2 of Beta-catenin-like protein 1	IPI00472981	0.436	0.18622	6	5	6	7
676	Mitochondrial glutamate carrier 1	IPI00003004	0.436	0.18622	6	5	6	7
677	Isoform 1 of ATPase family AAA domain-containing protein 1	IPI00171445	0.436	0.18622	5	6	6	7
678	Heat shock 70 kDa protein 4L	IPI00295485	0.436	0.18622	6	5	8	5
679	Proteasome subunit beta type-2	IPI00028006	0.424	0.18698	20	18	21	20
680	Dolichyl-diphosphooligosaccharide--protein glycosyltransferase subunit STT3A	IPI00297492	0.423	0.18731	5	7	7	7
681	Isoform A of Nucleoporin SEH1	IPI00185533	0.423	0.18731	7	5	9	5
682	Isoform 1 of Serine hydroxymethyltransferase, cytosolic	IPI00002519	0.423	0.18731	5	7	7	7
683	Isoform 2 of Transportin-3	IPI00395694	0.412	0.19619	6	7	6	9
684	Splicing factor, arginine/serine-rich 2	IPI00005978	0.412	0.19619	8	5	8	7
685	Superoxide dismutase [Mn], mitochondrial	IPI00022314	0.412	0.19619	8	5	7	8
686	Isoform 1 of Squamous cell carcinoma antigen recognized by T-cells 3	IPI00006025	0.412	0.19619	9	4	7	8
687	Nuclear RNA export factor 1	IPI00033153	0.412	0.19619	6	7	9	6
688	39S ribosomal protein L48, mitochondrial	IPI00295066	0.412	0.19619	6	7	9	6
689	Isoform 1 of Pescadillo homolog	IPI00003768	0.402	0.19704	7	7	6	10
690	Isoform 1 of Probable threonyl-tRNA synthetase 2, cytoplasmic	IPI00328082	0.402	0.19704	7	7	9	7
691	Translation initiation factor eIF-2B subunit alpha	IPI00221300	0.402	0.19704	7	7	8	8
692	CDGSH iron sulfur domain-containing protein 1	IPI00020510	0.402	0.19704	6	8	8	8
693	Programmed cell death protein 10	IPI00298558	0.402	0.19704	8	6	9	7
694	Isoform 1 of Protein canopy homolog 2	IPI00443909	0.402	0.19704	7	7	8	8
695	cDNA FLJ55574, highly similar to Calnexin	IPI00020984	0.394	0.20257	55	50	52	57
696	Protein flightless-1 homolog	IPI00031023	0.392	0.20300	6	9	7	10
697	Eukaryotic translation elongation factor 1 epsilon-1	IPI00003588	0.392	0.20300	8	7	8	9
698	Isoform 1 of Abhydrolase domain-containing protein 14B	IPI00063827	0.392	0.20300	6	9	9	8
699	Isoform Short of Glycylpeptide N-tetradecanoyltransferase 1	IPI00218830	0.392	0.20300	8	7	9	8
700	DNA-directed RNA polymerase, mitochondrial precursor	IPI00298738	0.386	0.20356	1	0	2	1
701	Isoform 1 of Zinc finger ZZ-type and EF-hand domain-containing protein 1	IPI00385631	0.386	0.20356	1	1	2	1
702	1-phosphatidylinositol-4,5-bisphosphate phosphodiesterase beta-3	IPI00010400	0.386	0.20356	1	1	1	2
703	Mediator of RNA polymerase II transcription subunit 14	IPI00297191	0.386	0.20356	0	0	2	0
704	coatomer subunit epsilon isoform c	IPI00399319	0.386	0.20356	1	0	2	0
705	Phosphoinositide 3-kinase regulatory subunit 4	IPI00024006	0.386	0.20356	0	0	2	1
706	Beta-adrenergic receptor kinase 1	IPI00012497	0.386	0.20356	1	1	2	1
707	Isoform 1 of Disco-interacting protein 2 homolog B	IPI00465045	0.386	0.20356	1	1	1	2
708	Protein NipSnap homolog 3A	IPI00004845	0.386	0.20356	1	0	1	2
709	Isoform A of GC-rich sequence DNA-binding factor homolog	IPI00001364	0.386	0.20356	0	0	2	1
710	E3 ubiquitin-protein ligase HECTD1	IPI00328911	0.386	0.20356	0	0	1	2
711	Isoform 1 of Long-chain-fatty-acid--CoA ligase 1	IPI00012728	0.386	0.20356	0	0	2	0
712	Vacuolar protein sorting-associated protein 4B	IPI00182728	0.386	0.20356	1	1	1	2
713	Isoform 1 of Protein furry homolog-like	IPI00739940	0.386	0.20356	0	0	1	2
714	ATPase ASNA1	IPI00013466	0.386	0.20356	1	0	2	1
715	Microtubule-associated protein 1S	IPI00296485	0.386	0.20356	0	0	1	2
716	Isoform 2 of Pinin	IPI00002649	0.386	0.20356	0	1	0	2
717	RNA polymerase-associated protein CTR9 homolog	IPI00477468	0.386	0.20356	0	1	2	1
718	Isoform 1 of Protein SDA1 homolog	IPI00018240	0.386	0.20356	1	0	2	1
719	Poly(A)-specific ribonuclease PARN	IPI00294744	0.386	0.20356	0	1	2	0
720	Thioredoxin-like protein 4B	IPI00016481	0.386	0.20356	0	0	2	0
721	46 kDa protein	IPI00641706	0.386	0.20356	0	0	0	2
722	Mitochondrial ornithine transporter 1	IPI00003389	0.386	0.20356	0	1	2	0
723	Isoform 1 of Acyl-coenzyme A thioesterase 9, mitochondrial	IPI00220710	0.386	0.20356	0	1	2	1
724	Zinc finger protein 622	IPI00056499	0.386	0.20356	0	1	1	2
725	Ephrin type-A receptor 2	IPI00021267	0.386	0.20356	1	1	1	2
726	UBX domain-containing protein 7	IPI00742124	0.386	0.20356	1	0	2	1
727	Isoform 1 of TP53RK-binding protein	IPI00301432	0.386	0.20356	0	0	1	2
728	UPF0444 transmembrane protein C12orf23	IPI00184546	0.386	0.20356	1	1	1	2
729	Translation initiation factor IF-2, mitochondrial	IPI00005039	0.386	0.20356	0	0	1	2
730	Isoform 1 of SWI/SNF-related matrix-associated actin-dependent regulator of chromatin subfamily E	IPI00017669	0.386	0.20356	1	1	2	1
731	Isoform 1 of GTP-binding protein 10	IPI00167638	0.386	0.20356	1	1	2	1
732	Thiopurine S-methyltransferase	IPI00019400	0.386	0.20356	1	1	1	2
733	Isoform 1 of Polyglutamine-binding protein 1	IPI00024698	0.386	0.20356	1	1	1	2
734	Isoform 1 of Ras GTPase-activating protein 1	IPI00026262	0.386	0.20356	0	0	1	2
735	Isoform 1 of Liprin-alpha-1	IPI00163496	0.386	0.20356	0	1	1	2
736	Isoform 2 of ATPase WRNIP1	IPI00102997	0.386	0.20356	1	0	1	2
737	Dynactin subunit 4	IPI00550852	0.386	0.20356	1	1	1	2
738	Coproporphyrinogen-III oxidase, mitochondrial	IPI00093057	0.386	0.20356	0	1	2	1
739	Mitochondrial folate transporter/carrier	IPI00300886	0.386	0.20356	1	1	2	1
740	Isoform 1 of Nucleotide-binding protein-like	IPI00384517	0.386	0.20356	0	1	2	1
741	Isoform 1 of Helicase-like transcription factor	IPI00339381	0.386	0.20356	0	1	1	2
742	cDNA FLJ56420, highly similar to Aspartyl aminopeptidase	IPI00015856	0.386	0.20356	0	1	1	2
743	Isoform 2 of Protein transport protein Sec24A	IPI00178798	0.386	0.20356	0	0	0	2
744	Isoform 1 of Nucleolar protein 7	IPI00007729	0.386	0.20356	1	0	1	2
745	Rho guanine nucleotide exchange factor 11	IPI00157442	0.386	0.20356	0	0	0	2
746	Glypican-6	IPI00001755	0.386	0.20356	1	0	2	1
747	Glucose 1,6-bisphosphate synthase	IPI00173346	0.386	0.20356	0	0	1	2
748	Transcriptional activator protein Pur-alpha	IPI00023591	0.386	0.20356	1	1	1	2
749	UPF0554 protein C2orf43	IPI00030257	0.386	0.20356	0	0	2	1
750	74 kDa protein	IPI00290439	0.386	0.20356	0	0	0	2
751	Isoform 1 of Rab3 GTPase-activating protein catalytic subunit	IPI00014235	0.386	0.20356	1	0	2	1
752	Isoform 1 of tRNA 2'-phosphotransferase 1	IPI00328580	0.386	0.20356	0	1	1	2
753	Isoform Beta of DNA ligase 3	IPI00000156	0.386	0.20356	0	0	2	0

No.	Description	Accession number	STN[1]	p-Value[1]	Con_A[2]	Con_B[2]	CUR_A[2]	CUR_B[2]
754	Replication factor C subunit 3	IPI00031521	0.386	0.20356	1	1	0	2
755	Isoform 1 of Epidermal growth factor receptor kinase substrate 8-like protein 3	IPI00181833	0.386	0.20356	1	1	2	0
756	Isoform 1 of Abl interactor 1	IPI00431025	0.386	0.20356	1	1	2	1
757	U3 small nucleolar ribonucleoprotein protein MPP10	IPI00012149	0.386	0.20356	0	1	1	2
758	Isoform 2 of Epithelial splicing regulatory protein 2	IPI00034099	0.386	0.20356	1	0	2	1
759	Isoform 1 of YTH domain family protein 2	IPI00306043	0.386	0.20356	0	0	1	2
760	Isoform 3 of Sorting nexin-3	IPI00029740	0.386	0.20356	1	0	1	2
761	Tetratricopeptide repeat protein 1	IPI00016912	0.386	0.20356	1	1	2	1
762	Isoform 2 of Receptor-type tyrosine-protein phosphatase alpha	IPI00221067	0.386	0.20356	0	1	1	2
763	X-prolyl aminopeptidase (Aminopeptidase P) 1, soluble	IPI00514564	0.386	0.20356	1	1	1	2
764	Trafficking protein particle complex subunit 5	IPI00177509	0.386	0.20356	1	0	2	0
765	Isoform 4 of Dipeptidyl peptidase 9	IPI00604483	0.386	0.20356	1	0	2	0
766	Propionyl-CoA carboxylase beta chain, mitochondrial	IPI00007247	0.386	0.20356	1	0	2	1
767	Glia maturation factor, beta	IPI00412987	0.386	0.20356	0	1	1	2
768	Isocitrate dehydrogenase [NAD] subunit gamma, mitochondrial	IPI00220150	0.386	0.20356	1	0	2	1
769	Switch-associated protein 70	IPI00307200	0.386	0.20356	0	1	1	2
770	sorting nexin-6 isoform a	IPI00258833	0.386	0.20356	1	1	2	0
771	DNA-directed RNA polymerase II subunit RPB4	IPI00007283	0.386	0.20356	0	0	2	1
772	NEDD8-activating enzyme E1 catalytic subunit	IPI00328154	0.386	0.20356	1	1	2	1
773	GA-binding protein alpha chain	IPI00299413	0.386	0.20356	1	1	1	2
774	Protein pelota homolog	IPI00106698	0.386	0.20356	1	1	2	1
775	Isoform 1 of Rab GTPase-activating protein 1	IPI00016702	0.386	0.20356	1	0	2	0
776	Isoform 1 of Creatine kinase U-type, mitochondrial	IPI00658109	0.386	0.20356	1	1	1	2
777	Isoform 2 of tRNA pseudouridine synthase A	IPI00001716	0.386	0.20356	1	1	1	2
778	Isoform 1 of Long-chain-fatty-acid--CoA ligase 5	IPI00008037	0.386	0.20356	1	1	2	1
779	Maspardin	IPI00010248	0.386	0.20356	0	1	0	2
780	2-oxoisovalerate dehydrogenase subunit alpha, mitochondrial	IPI00025100	0.386	0.20356	1	1	2	0
781	Wiskott-Aldrich syndrome protein family member 1	IPI00022007	0.386	0.20356	0	0	2	1
782	2-aminoethanethiol dioxygenase	IPI00045939	0.386	0.20356	0	0	2	1
783	Armadillo repeat-containing X-linked protein 3	IPI00009906	0.386	0.20356	0	0	1	2
784	Isoform 1 of Metallo-beta-lactamase domain-containing protein 2	IPI00293336	0.386	0.20356	1	0	2	1
785	Serine/threonine-protein kinase TBK1	IPI00293613	0.386	0.20356	0	0	2	0
786	Isoform 1 of Methyl-CpG-binding domain protein 2	IPI00434623	0.386	0.20356	0	0	2	1
787	Estradiol 17-beta-dehydrogenase 8	IPI00021890	0.386	0.20356	0	1	2	1
788	FLJ00369 protein (Fragment)	IPI00166711	0.386	0.20356	0	0	2	0
789	Notchless protein homolog 1	IPI00018196	0.386	0.20356	1	0	1	2
790	Mitochondrial intermediate peptidase	IPI00241860	0.386	0.20356	0	0	2	0
791	Isoform Short of Adenosine kinase	IPI00234368	0.384	0.21920	9	7	10	8
792	Proteasome inhibitor PI31 subunit	IPI00009949	0.384	0.21920	9	7	9	9
793	Ribosome maturation protein SBDS	IPI00427330	0.384	0.21920	9	7	11	7
794	39S ribosomal protein L13, mitochondrial	IPI00022403	0.384	0.21920	8	8	9	9
795	Thiosulfate sulfurtransferase	IPI00216293	0.384	0.21920	7	9	8	10
796	Polyribonucleotide nucleotidyltransferase 1, mitochondrial	IPI00744711	0.384	0.21920	8	8	8	10
797	Cytochrome b-c1 complex subunit Rieske, mitochondrial	IPI00026964	0.384	0.21920	7	9	7	11
798	Isoform 1 of Insulin-like growth factor 2 mRNA-binding protein 2	IPI00179713	0.384	0.21920	8	8	10	8
799	Isoform Beta-2 of DNA topoisomerase 2-beta	IPI00027280	0.380	0.22298	31	21	29	26
800	Eukaryotic translation initiation factor 1A, Y-chromosomal	IPI00023004	0.380	0.22298	23	29	26	29
801	Tricarboxylate transport protein, mitochondrial	IPI00294159	0.376	0.22340	9	8	10	9
802	Fructose-bisphosphate aldolase A	IPI00465439	0.370	0.22388	29	27	28	31
803	Nodal modulator 1	IPI00329352	0.369	0.22388	10	8	9	11
804	Acidic leucine-rich nuclear phosphoprotein 32 family member E	IPI00165393	0.369	0.22388	10	8	11	9
805	von Hippel-Lindau binding protein 1, isoform CRA_b	IPI00334159	0.369	0.22388	10	8	11	9
806	Estradiol 17-beta-dehydrogenase 12	IPI00007676	0.363	0.22761	29	30	32	30
807	sister chromatid cohesion protein PDS5 homolog A isoform 2	IPI00303063	0.362	0.22780	11	8	11	10
808	Puromycin-sensitive aminopeptidase	IPI00026216	0.362	0.22780	10	9	9	12
809	AP-1 complex subunit gamma-1 isoform a	IPI00293396	0.355	0.22836	7	13	10	12
810	Isoform 1 of Adenylate kinase 2, mitochondrial	IPI00215901	0.355	0.22836	11	9	11	11
811	UPF0468 protein C16orf80	IPI00001655	0.355	0.22836	11	9	12	10
812	Growth hormone inducible transmembrane protein	IPI00549970	0.350	0.23172	13	8	9	14
813	Actin-related protein 3	IPI00028091	0.350	0.23172	12	9	13	10
814	tropomyosin alpha-3 chain isoform 1	IPI00183968	0.350	0.23172	11	10	11	12
815	Eukaryotic translation initiation factor 5	IPI00022648	0.344	0.23210	10	12	12	12
816	Isoform 1 of UDP-glucose:glycoprotein glucosyltransferase 1	IPI00024466	0.344	0.23413	38	31	36	36
817	Similar to Protein SAAL1. Isoform 2	IPI00304935	0.341	0.23422	0	2	3	0
818	SNARE-associated protein Snapin	IPI00018331	0.341	0.23422	1	2	2	2
819	Mitochondrial 18 kDa protein	IPI00784376	0.341	0.23422	2	1	1	3
820	39S ribosomal protein L45, mitochondrial	IPI00185859	0.341	0.23422	1	2	0	3
821	mRNA export factor	IPI00019733	0.341	0.23422	1	2	2	2
822	28S ribosomal protein S18a, mitochondrial	IPI00018691	0.341	0.23422	0	2	2	2
823	Isoform 1 of Exosome component 10	IPI00009464	0.341	0.23422	2	0	2	2
824	cytochrome c oxidase subunit VIIa polypeptide 2 (liver) precursor	IPI00026570	0.341	0.23422	1	2	2	2
825	TAF6-like RNA polymerase II p300/CBP-associated factor-associated factor 65 kDa subunit 6L	IPI00007957	0.341	0.23422	0	2	2	2
826	Isoform 1 of Aldehyde dehydrogenase family 16 member A1	IPI00217920	0.341	0.23422	1	2	3	1
827	60S ribosomal protein L35a	IPI00029731	0.341	0.23422	2	1	2	2
828	Isoform 2 of Septin-11	IPI00019376	0.341	0.23422	1	2	1	3
829	Isoform II of Ubiquitin-protein ligase E3A	IPI00011609	0.341	0.23422	1	2	1	3
830	Myosin-Id	IPI00329719	0.341	0.23422	0	2	0	3
831	Isoform 1 of Zinc finger MYM-type protein 3	IPI00029484	0.341	0.23422	2	1	0	3
832	Procollagen-lysine,2-oxoglutarate 5-dioxygenase 3	IPI00030255	0.341	0.23422	2	1	3	1
833	ADP-ribosylation factor-like protein 6	IPI00021685	0.341	0.23422	1	2	3	1
834	Isoform 1 of TRM1-like protein	IPI00334914	0.341	0.23422	1	2	2	2
835	ATP synthase subunit g, mitochondrial	IPI00027448	0.341	0.23422	1	2	2	2
836	Charged multivesicular body protein 5	IPI00100796	0.341	0.23422	2	1	1	3
837	Alcohol dehydrogenase class-3	IPI00746777	0.341	0.23422	0	2	2	2
838	Sentrin-specific protease 3	IPI00171525	0.341	0.23422	2	1	2	2
839	Scaffold attachment factor B1	IPI00300631	0.341	0.23422	2	1	2	2
840	Isoform 1 of Caseinolytic peptidase B protein homolog	IPI00006615	0.341	0.23422	2	0	3	0
841	Phosphomannomutase 2	IPI00006092	0.341	0.23422	2	1	2	2
842	WD repeat-containing protein 81 isoform 1	IPI00917671	0.341	0.23422	1	2	3	0
843	Isoform 1 of Rho guanine nucleotide exchange factor 12	IPI00022164	0.341	0.23422	2	0	2	2
844	Nucleoporin NUP53	IPI00329650	0.341	0.23422	2	1	1	3
845	Phosphopantothenate--cysteine ligase	IPI00023987	0.341	0.23422	1	2	2	2
846	Isoform 1 of Kinesin-like protein KIF2A	IPI00010368	0.341	0.23422	1	2	2	2
847	28S ribosomal protein S9, mitochondrial	IPI00641924	0.341	0.23422	1	2	2	2
848	Ubiquitin-conjugating enzyme E2 R2	IPI00418603	0.341	0.23422	2	1	2	2

No.	Description	Accession number	STN[1]	p-Value[1]	Con_A[2]	Con_B[2]	CUR_A[2]	CUR_B[2]
849	Isoform 2 of Valacyclovir hydrolase	IPI00003990	0.341	0.23422	2	1	0	3
850	Isoform 1a of Oxysterol-binding protein-related protein 3	IPI00023555	0.341	0.23422	2	1	2	2
851	Isoform 1 of Cullin-associated NEDD8-dissociated protein 1	IPI00100160	0.335	0.24395	37	37	39	38
852	Ubiquitin carboxyl-terminal hydrolase isozyme L3	IPI00011250	0.334	0.24395	14	10	15	11
853	Nicotinamide phosphoribosyltransferase	IPI00018873	0.334	0.24395	12	12	12	14
854	Isoform 1 of RNA-binding protein 8A	IPI00001757	0.334	0.24395	12	12	15	11
855	Isoform p150 of Dynactin subunit 1	IPI00029485	0.334	0.24395	12	12	13	13
856	Small nuclear ribonucleoprotein Sm D2	IPI00017963	0.334	0.24395	12	12	12	14
857	Isoform 2 of Structural maintenance of chromosomes flexible hinge domain-containing protein 1	IPI00465022	0.325	0.24622	15	11	14	14
858	cDNA FLJ56307, highly similar to Ubiquitin thioesterase protein OTUB1	IPI00000581	0.312	0.24995	15	14	16	15
859	Putative uncharacterized protein CNOT1	IPI00032299	0.311	0.25024	3	1	3	2
860	51 kDa protein	IPI00033025	0.311	0.25024	2	2	3	2
861	Isoform 1 of Partner of Y14 and mago	IPI00305092	0.311	0.25024	3	0	2	3
862	Isoform 4 of Afadin	IPI00023461	0.311	0.25024	2	2	3	2
863	Isoform 1 of Integrator complex subunit 4	IPI00446765	0.311	0.25024	1	3	1	4
864	Bystin	IPI00328987	0.311	0.25024	2	2	3	2
865	Isoform 1 of Ubiquitin carboxyl-terminal hydrolase 15	IPI00000728	0.311	0.25024	2	2	2	3
866	28S ribosomal protein S28, mitochondrial	IPI00022276	0.311	0.25024	3	1	3	2
867	Isoform Long of Deoxyhypusine synthase	IPI00026829	0.311	0.25024	2	2	3	2
868	Isoform 2 of Myosin-VI	IPI00008455	0.311	0.25024	1	3	1	4
869	Proline-rich protein PRCC	IPI00294618	0.311	0.25024	2	2	2	3
870	OTU domain-containing protein 6B	IPI00182180	0.311	0.25024	2	2	3	2
871	Isoform 2 of Oxidoreductase HTATIP2	IPI00383665	0.311	0.25024	2	2	2	3
872	Isoform 1 of C-terminal-binding protein 2	IPI00010120	0.311	0.25024	2	2	2	3
873	Gem-associated protein 7	IPI00003027	0.311	0.25024	1	3	3	2
874	Cytochrome c oxidase subunit 5B, mitochondrial	IPI00021785	0.311	0.25024	2	2	2	3
875	aldehyde dehydrogenase 9A1	IPI00479877	0.311	0.25024	0	3	2	3
876	Isoform 1 of Far upstream element-binding protein 1	IPI00375441	0.305	0.26167	14	17	17	16
877	Isoform 1 of Vinculin	IPI00291175	0.305	0.26167	46	51	51	49
878	Isoform 1 of Protein-L-isoaspartate(D-aspartate) O-methyltransferase	IPI00411680	0.302	0.26167	16	16	18	16
879	Importin subunit beta-1	IPI00001639	0.296	0.26280	49	56	52	56
880	Dihydrolipoyl dehydrogenase, mitochondrial	IPI00015911	0.292	0.26375	16	19	17	20
881	Isoform 4 of Heterogeneous nuclear ribonucleoprotein A/B	IPI00106509	0.290	0.26379	18	18	19	19
882	RNA-binding protein PNO1	IPI00024524	0.289	0.26502	2	3	4	2
883	Isoform 1 of Ribose-phosphate pyrophosphokinase 2	IPI00219617	0.289	0.26502	3	2	3	3
884	Guanine nucleotide-binding protein G(k) subunit alpha	IPI00220578	0.289	0.26502	2	3	4	2
885	cDNA FLJ35809 fis, clone TESTI2006016, highly similar to Eukaryotic translation initiation factor 3	IPI00647650	0.289	0.26502	2	3	4	2
886	Endoplasmic reticulum resident protein 44	IPI00401264	0.289	0.26502	2	3	4	2
887	Tubulin-folding cofactor B	IPI00293126	0.289	0.26502	3	2	2	4
888	Sorting nexin-2	IPI00299095	0.289	0.26502	2	3	3	3
889	Transmembrane protein 43	IPI00301280	0.289	0.26502	3	2	3	3
890	Isoform 2 of Transcription elongation factor A protein 1	IPI00218106	0.289	0.26502	3	2	3	3
891	Isoform 1 of Peroxisomal membrane protein PEX16	IPI00006722	0.289	0.26502	3	2	3	3
892	Aminopeptidase B	IPI00642211	0.289	0.26502	3	2	3	3
893	Isoform 3 of Nucleoporin NDC1	IPI00074330	0.289	0.26502	2	3	4	2
894	Isoform 2 of WASH complex subunit 7	IPI00164930	0.289	0.26502	1	4	3	3
895	Nuclear pore complex protein Nup107	IPI00028005	0.289	0.26502	3	2	4	2
896	Tyrosine-protein phosphatase non-receptor type 23	IPI00034006	0.289	0.26502	2	3	3	3
897	U3 small nucleolar RNA-associated protein 6 homolog	IPI00020128	0.289	0.26502	2	3	2	4
898	cDNA FLJ56370, highly similar to Homo sapiens FK506 binding protein 8, 38kDa (FKBP8), mRNA	IPI00328161	0.289	0.26502	2	3	2	4
899	Survival of motor neuron-related-splicing factor 30	IPI00025176	0.289	0.26502	2	3	3	3
900	Sulfide:quinone oxidoreductase, mitochondrial	IPI00009634	0.289	0.26502	2	3	3	3
901	Ras-related protein Rab-22A	IPI00007756	0.289	0.26502	3	2	3	3
902	Exosome complex exonuclease RRP42	IPI00014198	0.289	0.26502	2	3	3	3
903	Isoform 1 of Cytosolic non-specific dipeptidase	IPI00177728	0.289	0.26502	2	3	3	3
904	Isoform 1 of Secretory carrier-associated membrane protein 3	IPI00306382	0.289	0.26502	3	2	4	2
905	Isoform 1 of Surfeit locus protein 4	IPI00005737	0.287	0.27159	23	14	21	18
906	Phenylalanyl-tRNA synthetase beta chain	IPI00300074	0.279	0.27286	21	19	21	21
907	cDNA FLJ54957, highly similar to Transketolase	IPI00643920	0.277	0.27405	65	62	66	64
908	Keratin, type II cytoskeletal 5	IPI00009867	0.272	0.27504	4	2	3	4
909	Niban-like protein 1	IPI00456750	0.272	0.27504	3	3	2	5
910	Isoform Del-701 of Signal transducer and activator of transcription 3	IPI00306436	0.272	0.27504	3	3	3	4
911	Isoform 1 of HEAT repeat-containing protein 2	IPI00242630	0.272	0.27504	2	4	3	4
912	DKFZP586J0619 protein	IPI00740961	0.272	0.27504	2	4	2	5
913	ATP-binding cassette sub-family F member 2	IPI00005045	0.272	0.27504	3	3	4	3
914	DNA-directed RNA polymerases I, II, and III subunit RPABC3	IPI00003309	0.272	0.27504	2	4	4	3
915	TATA-binding protein-associated factor 172	IPI00024802	0.272	0.27504	4	2	4	3
916	Histidine triad nucleotide-binding protein 2, mitochondrial	IPI00000335	0.272	0.27504	4	2	4	3
917	Isoform 1 of Microtubule-associated protein 4	IPI00396171	0.272	0.27504	2	4	3	4
918	perilipin-3 isoform 3	IPI00106668	0.272	0.27504	1	5	3	4
919	ATP synthase mitochondrial F1 complex assembly factor 2	IPI00296999	0.272	0.27504	3	3	5	2
920	Isoform 3 of Centromere protein V	IPI00376481	0.272	0.27504	4	2	4	3
921	Cytochrome b-c1 complex subunit 7	IPI00220416	0.272	0.27504	3	3	4	3
922	Hydroxymethylglutaryl-CoA lyase, mitochondrial	IPI00293564	0.272	0.27504	3	3	4	3
923	Isoform 2 of Annexin A2	IPI00418169	0.265	0.28297	25	21	23	25
924	Isoform 1 of V-type proton ATPase subunit H	IPI00296191	0.258	0.28411	4	3	4	4
925	Cell growth-regulating nucleolar protein	IPI00015838	0.258	0.28411	3	4	4	4
926	B-cell receptor-associated protein 31	IPI00218200	0.258	0.28411	4	3	3	5
927	Isoform 1 of Wings apart-like protein homolog	IPI00375330	0.258	0.28411	3	4	4	4
928	Mitochondrial import inner membrane translocase subunit Tim23	IPI00007309	0.258	0.28411	3	4	3	5
929	pyrroline-5-carboxylate reductase 1, mitochondrial isoform 2	IPI00376503	0.258	0.28411	4	3	4	4
930	Kinesin-like protein KIF11	IPI00305289	0.258	0.28411	4	3	3	5
931	Heme-binding protein 1	IPI00148063	0.258	0.28411	3	4	4	4
932	Isoform 1 of SET domain-containing protein 3	IPI00165026	0.258	0.28411	4	3	3	5
933	Isoform 3 of Serine/threonine-protein phosphatase 2A activator	IPI00217296	0.258	0.28411	2	5	5	3
934	Copine-3	IPI00024403	0.258	0.28411	4	3	4	4
935	U6 snRNA-associated Sm-like protein LSm3	IPI00219229	0.258	0.28411	4	3	3	5
936	Isoform 1 of Syntenin-1	IPI00299086	0.258	0.28411	3	4	4	4
937	serine/threonine-protein phosphatase PP1-alpha catalytic subunit isoform 3	IPI00027423	0.258	0.28411	4	3	5	3
938	Isoform 1 of Dephospho-CoA kinase domain-containing protein	IPI00291417	0.258	0.28411	3	4	3	5
939	Isoform 1 of Mitochondrial antiviral-signaling protein	IPI00020719	0.258	0.28411	3	4	4	4
940	Isoform 3 of THO complex subunit 6 homolog	IPI00301252	0.258	0.28411	4	3	3	5
941	cDNA FLJ61629, highly similar to Clathrin interactor 1	IPI00291930	0.258	0.28411	4	3	2	6
942	Putative annexin A2-like protein	IPI00334627	0.251	0.28982	75	91	75	94
943	40S ribosomal protein S11	IPI00025091	0.247	0.29067	3	5	5	4

No.	Description	Accession number	STN[1]	p-Value[1]	Con_A[2]	Con_B[2]	CUR_A[2]	CUR_B[2]
944	Isoform 2 of Ubiquitin-conjugating enzyme E2 K	IPI00019894	0.247	0.29067	5	3	5	4
945	General transcription factor 3C polypeptide 4	IPI00016725	0.247	0.29067	3	5	5	4
946	Mitogen-activated protein kinase 1	IPI00003479	0.247	0.29067	3	5	4	5
947	Isoform 2 of Suppressor of G2 allele of SKP1 homolog	IPI00791573	0.247	0.29067	3	5	4	5
948	CDGSH iron sulfur domain-containing protein 2	IPI00166865	0.247	0.29067	4	4	5	4
949	Isoform 2 of Ubiquilin-1	IPI00071180	0.247	0.29067	6	2	6	3
950	Isoform 3 of Ester hydrolase C11orf54	IPI00061507	0.247	0.29067	4	4	4	5
951	Sialic acid synthase	IPI00147874	0.247	0.29067	4	4	5	4
952	AFG3-like protein 2	IPI00001091	0.247	0.29067	5	3	4	5
953	Protein FAM98B	IPI00167572	0.247	0.29067	5	3	4	5
954	dynactin subunit 2	IPI00220503	0.247	0.29067	4	4	5	4
955	Histone H1x	IPI00021924	0.247	0.29067	3	5	4	5
956	Putative uncharacterized protein MYO7B	IPI00738806	0.237	0.29734	2	7	4	6
957	Nucleolar complex protein 3 homolog	IPI00102815	0.237	0.29734	3	6	7	3
958	Calcium-regulated heat stable protein 1	IPI00304409	0.237	0.29734	4	5	5	5
959	Solute carrier family 4 sodium bicarbonate cotransporter member 7	IPI00021058	0.237	0.29734	6	3	6	4
960	Scaffold attachment factor B2	IPI00005648	0.237	0.29734	5	4	5	5
961	Chromobox protein homolog 5	IPI00024662	0.237	0.29734	4	5	5	5
962	Immunoglobulin-binding protein 1	IPI00019148	0.237	0.29734	7	2	5	5
963	Probable ATP-dependent RNA helicase DDX52	IPI00032423	0.237	0.29734	4	5	5	5
964	UPF0556 protein C19orf10	IPI00056357	0.237	0.29734	5	4	6	4
965	Sphingosine-1-phosphate lyase 1	IPI00099463	0.237	0.29734	6	3	5	5
966	60S ribosomal protein L30	IPI00219156	0.237	0.29734	4	5	5	5
967	Isoform 1 of Alpha-parvin	IPI00018963	0.237	0.29734	4	5	5	5
968	Growth arrest and DNA damage-inducible proteins-interacting protein 1	IPI00552587	0.237	0.29734	5	4	5	5
969	TP53-regulating kinase	IPI00290305	0.237	0.29734	5	4	5	5
970	Ras-related protein Ral-A	IPI00217519	0.237	0.29734	4	5	5	5
971	Isoform 1 of Calcyclin-binding protein	IPI00395627	0.229	0.30159	35	35	36	36
972	Isoform 1 of Gamma-glutamylcyclotransferase	IPI00031564	0.228	0.30211	7	3	6	5
973	Methyltransferase like 7B	IPI00090807	0.228	0.30211	5	5	5	6
974	DNA-directed RNA polymerase II subunit RPB3	IPI00018288	0.228	0.30211	5	5	6	5
975	Isoform 1 of Coiled-coil domain-containing protein 47	IPI00024642	0.228	0.30211	6	4	7	4
976	Isoform 4 of Nucleoporin NDC1	IPI00003455	0.228	0.30211	4	6	6	5
977	Eukaryotic translation initiation factor 2 subunit 2	IPI00021728	0.228	0.30211	6	4	6	5
978	Signal recognition particle 14 kDa protein	IPI00293434	0.228	0.30211	5	5	6	5
979	Isoform 2 of Diphosphoinositol polyphosphate phosphohydrolase 2	IPI00021408	0.228	0.30211	5	5	7	4
980	Isoform 1 of Ras-related protein Rab-6A	IPI00023526	0.228	0.30211	5	5	6	5
981	Ras-related protein Rab-7a	IPI00016342	0.227	0.30721	37	34	38	35
982	Heat shock protein beta (Fragment)	IPI00411633	0.224	0.30745	35	39	38	38
983	Isoform 1 of Enolase-phosphatase E1	IPI00038378	0.221	0.30778	5	6	6	6
984	Isoform 1 of Chromodomain-helicase-DNA-binding protein 1	IPI00297851	0.221	0.30778	6	5	6	6
985	cDNA FLJ50992, highly similar to Coronin-1C	IPI00798401	0.221	0.30778	7	4	4	8
986	Cyclin-G-associated kinase	IPI00298949	0.221	0.30778	7	4	4	8
987	Isoform 1 of N-alpha-acetyltransferase 50, NatE catalytic subunit	IPI00018627	0.221	0.30778	6	5	7	5
988	Isoform 1 of Calcineurin-like phosphoesterase domain-containing protein 1	IPI00305010	0.221	0.30778	5	6	6	6
989	septin-9 isoform e	IPI00455033	0.221	0.30778	8	3	4	8
990	Isoform 1 of Protein LSM12 homolog	IPI00410324	0.221	0.30778	5	6	5	7
991	cDNA FLJ40287 fis, clone TESTI2027909, highly similar to 5'-AMP-ACTIVATED PROTEIN KINASE	IPI00473047	0.221	0.30778	6	5	7	5
992	Sec1 family domain-containing protein 1	IPI00165261	0.214	0.31132	7	5	7	6
993	sorting nexin-1 isoform c	IPI00183274	0.214	0.31132	6	6	7	6
994	UV excision repair protein RAD23 homolog B	IPI00008223	0.214	0.31132	8	4	6	7
995	Isoform 1 of Spermine synthase	IPI00005102	0.214	0.31132	5	7	6	7
996	Isoform 1 of Methyl-CpG-binding domain protein 3	IPI00439194	0.214	0.31132	6	6	7	6
997	Mitochondrial 28S ribosomal protein S2	IPI00006970	0.209	0.31661	9	4	7	7
998	DNA topoisomerase 1	IPI00413611	0.209	0.31661	4	9	7	7
999	Isoform 1 of Septin-2	IPI00014177	0.209	0.31661	6	7	7	7
1000	V-type proton ATPase subunit D	IPI00001568	0.209	0.31661	6	7	7	7
1001	N-alpha-acetyltransferase 38, NatC auxiliary subunit	IPI00219871	0.209	0.31661	6	7	8	6
1002	L-xylulose reductase	IPI00448095	0.203	0.31978	8	6	8	7
1003	Parafibromin	IPI00300659	0.203	0.31978	7	7	8	7
1004	60S ribosomal protein L7-like 1	IPI00456940	0.203	0.31978	6	8	8	7
1005	Vitamin K epoxide reductase complex subunit 1-like protein 1	IPI00166079	0.203	0.31978	6	8	7	8
1006	Isoform 1 of Adenylyl cyclase-associated protein 1	IPI00008274	0.199	0.32303	52	51	53	52
1007	NADH-ubiquinone oxidoreductase chain 1	IPI00007961	0.198	0.32308	9	6	7	9
1008	60S ribosomal protein L19	IPI00025329	0.198	0.32308	8	7	9	7
1009	cDNA FLJ14048 fis, clone HEMBA1006650, weakly similar to ARP2/3 COMPLEX 20 KD SUBUNIT	IPI00386354	0.198	0.32308	6	9	7	9
1010	Malate dehydrogenase	IPI00916111	0.198	0.32308	8	7	8	8
1011	Histone-binding protein RBBP7	IPI00395865	0.194	0.32445	9	7	8	9
1012	Ribosome biogenesis protein BRX1 homolog	IPI00181728	0.194	0.32445	8	8	9	8
1013	60S ribosomal protein L26-like 1	IPI00007144	0.194	0.32445	9	7	8	9
1014	Isoform 1 of Large proline-rich protein BAT3	IPI00465128	0.194	0.32445	11	5	8	9
1015	26S protease regulatory subunit 7	IPI00021435	0.194	0.32445	9	7	9	8
1016	EH domain-containing protein 4	IPI00005578	0.194	0.32445	9	7	8	9
1017	Galectin-3-binding protein	IPI00023673	0.194	0.32445	8	8	9	8
1018	Proline synthetase co-transcribed homolog (Bacterial), isoform CRA_b	IPI00016346	0.190	0.32592	10	7	9	9
1019	UPF0368 protein Cxorf26	IPI00107104	0.190	0.32592	7	10	9	9
1020	Phospholipase A-2-activating protein	IPI00218465	0.190	0.32592	8	9	9	9
1021	Isoform 1 of RNA-binding protein Musashi homolog 2	IPI00073713	0.190	0.32592	10	7	9	9
1022	Thioredoxin domain-containing protein 17	IPI00646689	0.186	0.32705	7	11	8	11
1023	Protein DEK	IPI00020021	0.186	0.32705	10	8	10	9
1024	Isoform 4 of Serine/threonine-protein phosphatase 6 regulatory subunit 3	IPI00019540	0.186	0.32705	10	8	9	10
1025	Alpha-actinin-4	IPI00013808	0.185	0.32875	67	60	67	62
1026	U4/U6.U5 tri-snRNP-associated protein 2	IPI00419844	0.183	0.32875	11	8	8	12
1027	Isoform 1 of Vesicle-associated membrane protein-associated protein B/C	IPI00006211	0.183	0.32875	11	8	9	11
1028	Inorganic pyrophosphatase	IPI00015018	0.182	0.32993	68	65	69	66
1029	Isoform 1 of 39S ribosomal protein L4, mitochondrial	IPI00023334	0.179	0.32998	10	10	10	11
1030	26S protease regulatory subunit S10B	IPI00021926	0.176	0.33163	9	12	11	11
1031	TOB3	IPI00045921	0.176	0.33163	10	11	12	10
1032	Ras-related protein Rab-14	IPI00291928	0.173	0.33281	11	11	12	11
1033	Stathmin	IPI00479997	0.173	0.33281	12	10	12	11
1034	Isoform 1 of U2-associated protein SR140	IPI00143753	0.171	0.33343	12	11	13	11
1035	Nuclear migration protein nudC	IPI00550746	0.171	0.33343	12	11	13	11
1036	Isoform 1 of Reticulon-4	IPI00021766	0.168	0.33437	13	11	12	13
1037	Mitochondrial import receptor subunit TOM22 homolog	IPI00024976	0.168	0.33437	14	10	15	10
1038	Isoform 3 of Ribosome-binding protein 1	IPI00215743	0.166	0.33522	14	11	12	14

No.	Description	Accession number	STN[1]	p-Value[1]	Con_A[2]	Con_B[2]	CUR_A[2]	CUR_B[2]
1039	Isoform 1 of Myosin-Ib	IPI00376344	0.166	0.33522	13	12	12	14
1040	Activated RNA polymerase II transcriptional coactivator p15	IPI00221222	0.163	0.33584	14	12	14	13
1041	Sorbitol dehydrogenase	IPI00216057	0.163	0.33584	14	12	13	14
1042	Isoform Short of Proteasome subunit alpha type-1	IPI00016832	0.161	0.33640	12	15	13	15
1043	Isoform 1 of Glucosamine--fructose-6-phosphate aminotransferase [isomerizing] 1	IPI00217952	0.161	0.33640	15	12	13	15
1044	Isoform 1 of NADH-cytochrome b5 reductase 3	IPI00328415	0.157	0.33844	16	13	15	15
1045	26S proteasome non-ATPase regulatory subunit 13 isoform 2	IPI00375380	0.155	0.33929	19	11	15	16
1046	26S proteasome non-ATPase regulatory subunit 7	IPI00019927	0.153	0.33929	13	18	14	18
1047	Endoplasmic reticulum resident protein 29	IPI00024911	0.152	0.33962	17	15	16	17
1048	Histidyl-tRNA synthetase, cytoplasmic	IPI00021808	0.152	0.33962	16	16	19	14
1049	Ras-related protein Rab-2A	IPI00031169	0.152	0.33962	14	18	17	16
1050	NADH dehydrogenase [ubiquinone] iron-sulfur protein 3, mitochondrial	IPI00025796	0.150	0.33995	18	15	18	16
1051	ATP synthase subunit beta, mitochondrial	IPI00303476	0.149	0.34023	117	115	117	117
1052	Isoform 1 of WD repeat-containing protein 1	IPI00746165	0.148	0.34033	16	18	18	17
1053	Ubiquitin-conjugating enzyme E2 N	IPI00003949	0.148	0.34033	16	18	16	19
1054	KH-type splicing regulatory protein	IPI00479786	0.148	0.34033	17	17	17	18
1055	6-phosphogluconolactonase	IPI00029997	0.147	0.34103	19	16	17	19
1056	Glycogen phosphorylase, brain form	IPI00004358	0.145	0.34136	19	17	19	18
1057	Vigilin	IPI00022228	0.144	0.34193	19	18	22	16
1058	DNA-directed RNA polymerase II subunit RPB1	IPI00031627	0.137	0.34358	21	22	20	24
1059	Proteasome subunit alpha type-5	IPI00291922	0.137	0.34358	22	21	21	23
1060	Isoform 1 of Proteasome subunit alpha type-7	IPI00024175	0.134	0.34406	21	24	23	23
1061	Isoform 1 of Heterogeneous nuclear ribonucleoprotein A3	IPI00419373	0.118	0.34784	31	34	31	35
1062	Isoform 3 of DNA topoisomerase 2-alpha	IPI00218753	0.113	0.34840	37	36	42	32
1063	Peptidyl-prolyl cis-trans isomerase B	IPI00646304	0.110	0.34892	39	40	39	41
1064	Ubiquitin-like modifier-activating enzyme 1	IPI00645078	0.100	0.35053	50	52	54	49
1065	Phosphoglycerate kinase 1	IPI00169383	0.090	0.35100	72	65	67	71
1066	Isoform 3 of Spectrin alpha chain, brain	IPI00843765	0.088	0.35105	74	74	82	67
1067	Isoform 1 of Myb-binding protein 1A	IPI00005024	0.000	0.35223	40	37	39	38
1068	Putative uncharacterized protein RPL17	IPI00394699	0.000	0.35223	17	12	14	15
1069	Structural maintenance of chromosomes protein 3	IPI00219420	0.000	0.35223	26	31	32	25
1070	Protein DJ-1	IPI00298547	0.000	0.35223	27	25	24	28
1071	Mitochondrial carrier homolog 2	IPI00003833	0.000	0.35223	27	23	23	27
1072	Eukaryotic translation initiation factor 5A-2	IPI00006935	0.000	0.35223	17	21	19	19
1073	Keratin, type II cytoskeletal 75	IPI00005859	0.000	0.35223	18	19	18	19
1074	U2 small nuclear ribonucleoprotein A'	IPI00297477	0.000	0.35223	20	26	18	28
1075	Isoform 1 of Splicing factor, arginine/serine-rich 7	IPI00003377	0.000	0.35223	19	18	18	19
1076	Isoform 3 of Adenylate kinase 2, mitochondrial	IPI00172460	0.000	0.35223	18	18	18	18
1077	Isoform 1 of La-related protein 1	IPI00185919	0.000	0.35223	14	11	12	13
1078	Isoform 1 of Nuclear autoantigenic sperm protein	IPI00179953	0.000	0.35223	14	19	16	17
1079	Isoform SM-B' of Small nuclear ribonucleoprotein-associated proteins B and B'	IPI00027285	0.000	0.35223	15	15	15	15
1080	UPF0568 protein C14orf166	IPI00006980	0.000	0.35223	19	20	22	17
1081	Isoform 1 of 26S protease regulatory subunit 6B	IPI00020042	0.000	0.35223	15	13	9	19
1082	Isoform 1 of Mitochondrial import receptor subunit TOM40 homolog	IPI00014053	0.000	0.35223	6	8	5	9
1083	protein arginine N-methyltransferase 5 isoform b	IPI00064328	0.000	0.35223	16	15	16	15
1084	Isoform 1 of F-actin-capping protein subunit beta	IPI00026185	0.000	0.35223	16	15	17	14
1085	Histidine triad nucleotide-binding protein 1	IPI00239077	0.000	0.35223	4	8	6	6
1086	Cytochrome c	IPI00465315	0.000	0.35223	12	12	13	11
1087	Isoform 1 of Cytosol aminopeptidase	IPI00419237	0.000	0.35223	18	15	15	18
1088	Stomatin-like protein 2	IPI00334190	0.000	0.35223	14	17	17	14
1089	Isoform 1 of Core-binding factor subunit beta	IPI00016746	0.000	0.35223	14	9	14	9
1090	Cytoplasmic dynein 1 light intermediate chain 2	IPI00011592	0.000	0.35223	12	9	10	11
1091	60S ribosomal protein L21	IPI00247583	0.000	0.35223	13	12	12	13
1092	Isoform 1 of Alpha-aminoadipic semialdehyde dehydrogenase	IPI00221234	0.000	0.35223	9	10	9	10
1093	Isoform 2 of Nipped-B-like protein	IPI00026466	0.000	0.35223	8	9	10	7
1094	40S ribosomal protein S25	IPI00012750	0.000	0.35223	11	7	11	7
1095	Isoform 2 of ATPase family AAA domain-containing protein 3A	IPI00295992	0.000	0.35223	18	15	19	14
1096	Early endosome antigen 1	IPI00329536	0.000	0.35223	12	11	12	11
1097	Junction plakoglobin	IPI00554711	0.000	0.35223	6	9	8	7
1098	Cytoplasmic dynein 1 light intermediate chain 1	IPI00007675	0.000	0.35223	8	10	10	8
1099	60S ribosomal protein L38	IPI00215790	0.000	0.35223	11	11	11	11
1100	NADH dehydrogenase [ubiquinone] iron-sulfur protein 8, mitochondrial	IPI00010845	0.000	0.35223	10	11	11	10
1101	Proteasome subunit beta type-6	IPI00000811	0.000	0.35223	7	6	7	6
1102	cDNA FLJ55482, highly similar to Annexin A11	IPI00414320	0.000	0.35223	13	11	13	11
1103	Platelet-activating factor acetylhydrolase IB subunit gamma	IPI00014808	0.000	0.35223	5	4	4	5
1104	Ribosomal protein S6 kinase alpha-6	IPI00007123	0.000	0.35223	11	8	11	8
1105	Dolichol-phosphate mannosyltransferase	IPI00022018	0.000	0.35223	10	9	11	8
1106	Importin 5	IPI00514205	0.000	0.35223	7	11	8	10
1107	Isoform 2 of AP-2 complex subunit alpha-2	IPI00016621	0.000	0.35223	6	4	6	4
1108	Inosine triphosphate pyrophosphatase	IPI00018783	0.000	0.35223	6	10	8	8
1109	Isoform 2 of Cytoplasmic FMR1-interacting protein 1	IPI00550212	0.000	0.35223	9	10	10	9
1110	DnaJ homolog subfamily C member 13	IPI00307259	0.000	0.35223	9	7	7	9
1111	Kinesin-1 heavy chain	IPI00012837	0.000	0.35223	7	8	8	7
1112	Isoform 1 of 6-phosphofructokinase, liver type	IPI00332371	0.000	0.35223	5	7	7	5
1113	Similar to Signal peptidase complex subunit 2	IPI00452747	0.000	0.35223	9	8	9	8
1114	MKI67 FHA domain-interacting nucleolar phosphoprotein	IPI00154590	0.000	0.35223	9	8	9	8
1115	Isoform 2 of Extended synaptotagmin-2	IPI00409635	0.000	0.35223	7	7	7	7
1116	Isoform 2 of Serine/threonine-protein phosphatase PGAM5, mitochondrial	IPI00063242	0.000	0.35223	7	6	5	8
1117	RAP1, GTP-GDP dissociation stimulator 1 isoform 6	IPI00424869	0.000	0.35223	1	1	1	0
1118	epiplakin	IPI00010951	0.000	0.35223	5	7	4	8
1119	Isoform 1 of Bcl-2-associated transcription factor 1	IPI00006079	0.000	0.35223	2	5	2	5
1120	Isoform 1 of SAM domain and HD domain-containing protein 1	IPI00294739	0.000	0.35223	6	9	9	6
1121	Histone H1.0	IPI00550239	0.000	0.35223	9	9	10	8
1122	V-type proton ATPase subunit E 1	IPI00003856	0.000	0.35223	9	9	8	10
1123	Isoform 1 of Inorganic pyrophosphatase 2, mitochondrial	IPI00301109	0.000	0.35223	11	12	12	11
1124	Exportin-T	IPI00306290	0.000	0.35223	6	7	8	5
1125	FKBP1A protein	IPI00413778	0.000	0.35223	7	7	6	8
1126	Exportin-5	IPI00640703	0.000	0.35223	3	5	3	5
1127	Isoform B of Serine/threonine-protein kinase 24	IPI00002212	0.000	0.35223	7	7	8	6
1128	Isoform 1 of STE20-like serine/threonine-protein kinase	IPI00022827	0.000	0.35223	7	7	7	7
1129	Metaxin-2	IPI00025717	0.000	0.35223	5	6	5	6
1130	Putative uncharacterized protein THADA	IPI00412647	0.000	0.35223	3	4	4	3
1131	Isoform 1 of Growth factor receptor-bound protein 2	IPI00021327	0.000	0.35223	4	5	5	4
1132	Isoform Long of 60 kDa SS-A/Ro ribonucleoprotein	IPI00019450	0.000	0.35223	7	7	7	7
1133	proteasome subunit beta type-5 isoform 3	IPI00383971	0.000	0.35223	9	10	9	10

No.	Description	Accession number	STN[1]	p-Value[1]	Con_A[2]	Con_B[2]	CUR_A[2]	CUR_B[2]
1134	Coactosin-like protein	IPI00017704	0.000	0.35223	8	6	8	6
1135	Ribose-phosphate pyrophosphokinase 3	IPI00218371	0.000	0.35223	7	5	6	6
1136	AP-1 complex subunit mu-1	IPI00032516	0.000	0.35223	7	5	7	5
1137	Heat shock protein beta-11	IPI00098827	0.000	0.35223	5	2	4	3
1138	Nucleolar protein 11	IPI00303813	0.000	0.35223	4	5	6	3
1139	Isoform 2 of NSFL1 cofactor p47	IPI00022830	0.000	0.35223	7	6	6	7
1140	Isoform 1 of Putative ATP-dependent RNA helicase DHX57	IPI00168885	0.000	0.35223	2	2	2	2
1141	treacle protein isoform a	IPI00165041	0.000	0.35223	2	2	2	2
1142	Syntaxin-binding protein 3	IPI00297626	0.000	0.35223	3	5	3	5
1143	tropomyosin alpha-1 chain isoform 2	IPI00000230	0.000	0.35223	10	11	11	10
1144	Myosin-11	IPI00020501	0.000	0.35223	5	6	5	6
1145	DNA-directed RNA polymerases I, II, and III subunit RPABC1	IPI00291093	0.000	0.35223	4	5	4	5
1146	Isoform 1 of Zinc phosphodiesterase ELAC protein 2	IPI00396627	0.000	0.35223	1	1	0	1
1147	Thimet oligopeptidase	IPI00549189	0.000	0.35223	5	6	6	5
1148	zinc finger protein 294	IPI00783835	0.000	0.35223	4	3	4	3
1149	Isoform 2 of COP9 signalosome complex subunit 2	IPI00018813	0.000	0.35223	5	4	5	4
1150	Isoform 1 of Bifunctional coenzyme A synthase	IPI00184821	0.000	0.35223	4	4	5	3
1151	Pyruvate dehydrogenase protein X component, mitochondrial	IPI00298423	0.000	0.35223	6	3	5	4
1152	Isoform 1 of 39S ribosomal protein L22, mitochondrial	IPI00414410	0.000	0.35223	7	7	7	7
1153	Adenylyl cyclase-associated protein	IPI00939159	0.000	0.35223	4	3	5	2
1154	Glyoxylate reductase/hydroxypyruvate reductase	IPI00037448	0.000	0.35223	6	6	6	6
1155	Leucine-rich repeat and WD repeat-containing protein 1	IPI00069309	0.000	0.35223	6	3	4	5
1156	SWI/SNF related, matrix associated, actin dependent regulator of chromatin	IPI00216046	0.000	0.35223	5	3	4	4
1157	Succinyl-CoA ligase [GDP-forming] subunit alpha, mitochondrial	IPI00872762	0.000	0.35223	6	5	5	6
1158	Monocarboxylate transporter 4	IPI00006666	0.000	0.35223	4	6	6	4
1159	Isoform 2 of U4/U6 small nuclear ribonucleoprotein Prp31	IPI00167198	0.000	0.35223	5	4	4	5
1160	Protein of unknown function DUF410 family protein	IPI00419575	0.000	0.35223	6	6	7	5
1161	Cleavage and polyadenylation specificity factor subunit 2	IPI00419531	0.000	0.35223	3	5	4	4
1162	Serine/threonine-protein phosphatase 2A 65 kDa regulatory subunit A alpha isoform	IPI00554737	0.000	0.35223	5	2	4	3
1163	Protein BUD31 homolog	IPI00013180	0.000	0.35223	5	5	6	4
1164	Isoform 1 of Ubiquitin-conjugating enzyme E2 K	IPI00021370	0.000	0.35223	7	6	6	7
1165	Protein kinase, cAMP-dependent, regulatory, type II, alpha, isoform CRA_b	IPI00063234	0.000	0.35223	2	2	2	2
1166	Mitochondrial ribosomal protein L21 isoform d	IPI00375677	0.000	0.35223	5	2	4	3
1167	Isoform 1 of Ataxin-2-like protein	IPI00456359	0.000	0.35223	4	4	3	5
1168	cDNA FLJ38069 fis, clone CTONG2015434, highly similar to DOUBLE-STRAND BREAK REPAIR PROTEIN	IPI00029159	0.000	0.35223	3	4	3	4
1169	BRI3-binding protein	IPI00103599	0.000	0.35223	6	6	7	5
1170	DnaJ homolog subfamily C member 8	IPI00003438	0.000	0.35223	6	4	4	6
1171	Isoform 1 of Histone-arginine methyltransferase CARM1	IPI00412880	0.000	0.35223	3	4	3	4
1172	Isoform 1 of BRCA2 and CDKN1A-interacting protein	IPI00002203	0.000	0.35223	5	6	5	6
1173	ATP-dependent RNA helicase DDX24	IPI00006987	0.000	0.35223	2	3	2	3
1174	Cytochrome c1, heme protein, mitochondrial	IPI00029264	0.000	0.35223	1	3	1	3
1175	Isoform 1 of Vacuolar protein sorting-associated protein 29	IPI00170796	0.000	0.35223	3	3	3	3
1176	Osteoclast-stimulating factor 1	IPI00414836	0.000	0.35223	3	2	3	2
1177	Ribosomal protein S6 kinase alpha-1	IPI00017305	0.000	0.35223	4	4	4	4
1178	NEDD8-activating enzyme E1 regulatory subunit	IPI00018968	0.000	0.35223	1	6	4	3
1179	U3 small nucleolar RNA-associated protein 15 homolog	IPI00152708	0.000	0.35223	3	5	4	4
1180	Nucleoside-triphosphatase C1orf57	IPI00031570	0.000	0.35223	3	4	3	4
1181	60S ribosomal protein L35	IPI00412607	0.000	0.35223	3	3	3	3
1182	18 kDa protein	IPI00797709	0.000	0.35223	3	5	5	3
1183	Programmed cell death protein 5	IPI00023640	0.000	0.35223	6	6	6	6
1184	4-hydroxyphenylpyruvate dioxygenase-like protein	IPI00063762	0.000	0.35223	3	4	5	2
1185	Isoform 2 of Mitochondrial import inner membrane translocase subunit TIM50	IPI00418497	0.000	0.35223	7	6	6	7
1186	Splicing factor U2AF 35 kDa subunit	IPI00005613	0.000	0.35223	4	3	4	3
1187	Isoform 3 of Pre-mRNA 3'-end-processing factor FIP1	IPI00008449	0.000	0.35223	4	3	4	3
1188	Isoform 1 of Tropomyosin alpha-4 chain	IPI00010779	0.000	0.35223	5	7	5	7
1189	EF-hand domain-containing protein D2	IPI00060181	0.000	0.35223	4	4	3	5
1190	Nuclear pore glycoprotein p62	IPI00293533	0.000	0.35223	6	6	5	7
1191	Isoform 1 of Mps one binder kinase activator-like 1B	IPI00301518	0.000	0.35223	1	3	3	1
1192	Putative uncharacterized protein EIF4E2	IPI00556081	0.000	0.35223	4	3	3	4
1193	60S ribosomal protein L31	IPI00026302	0.000	0.35223	2	4	2	4
1194	Rho-related GTP-binding protein RhoG	IPI00017342	0.000	0.35223	5	5	5	5
1195	Phosphoribosyl pyrophosphate synthase-associated protein 2	IPI00003168	0.000	0.35223	4	3	4	3
1196	Regulator of microtubule dynamics protein 1	IPI00329696	0.000	0.35223	3	5	5	3
1197	28S ribosomal protein S16, mitochondrial	IPI00032872	0.000	0.35223	1	1	1	1
1198	Pyruvate carboxylase, mitochondrial	IPI00299402	0.000	0.35223	2	0	2	1
1199	Isoform 1 of Myotubularin-related protein 5	IPI00029446	0.000	0.35223	1	2	2	1
1200	Isoform 1 of Drebrin	IPI00003406	0.000	0.35223	3	1	2	2
1201	Cytochrome b-c1 complex subunit 1, mitochondrial	IPI00013847	0.000	0.35223	3	3	2	4
1202	Isoform 2 of Ubiquitin conjugation factor E4 A	IPI00028957	0.000	0.35223	2	3	3	2
1203	Isoform 4 of Tubulin-specific chaperone D	IPI00030774	0.000	0.35223	2	1	1	2
1204	Atlastin-1	IPI00103530	0.000	0.35223	2	3	3	2
1205	Isoform 4 of E3 ubiquitin-protein ligase UBR2	IPI00217407	0.000	0.35223	4	3	3	4
1206	Isoform 1 of Anaphase-promoting complex subunit 7	IPI00008248	0.000	0.35223	3	3	4	2
1207	Glutamate-rich WD repeat-containing protein 1	IPI00027831	0.000	0.35223	4	3	3	4
1208	5'-nucleotidase domain-containing protein 1	IPI00177965	0.000	0.35223	1	3	2	2
1209	Ribonuclease H2 subunit A	IPI00290192	0.000	0.35223	5	3	3	5
1210	13kDa differentiation-associated protein variant (Fragment)	IPI00005966	0.000	0.35223	4	4	4	4
1211	Probable ATP-dependent RNA helicase YTHDC2	IPI00010200	0.000	0.35223	0	1	1	0
1212	Isoform 1 of Serine/threonine-protein phosphatase 6 catalytic subunit	IPI00012970	0.000	0.35223	3	4	3	4
1213	Serine/threonine-protein phosphatase 5	IPI00019812	0.000	0.35223	2	5	4	3
1214	RNA-binding protein NOB1	IPI00022373	0.000	0.35223	1	3	2	2
1215	Ubiquitin carboxyl-terminal hydrolase 11	IPI00184533	0.000	0.35223	2	3	3	2
1216	NADH-ubiquinone oxidoreductase chain 5	IPI00008511	0.000	0.35223	4	4	4	4
1217	Deoxycytidine kinase	IPI00020454	0.000	0.35223	1	1	1	1
1218	Zinc finger protein ZPR1	IPI00025244	0.000	0.35223	5	3	4	4
1219	39S ribosomal protein L50, mitochondrial	IPI00329036	0.000	0.35223	4	3	4	3
1220	Hepatoma-derived growth factor-related protein 3	IPI00007063	0.000	0.35223	2	2	3	1
1221	Phosducin-like protein 3	IPI00031629	0.000	0.35223	5	2	3	4
1222	Isoform 3 of Protein scribble homolog	IPI00410666	0.000	0.35223	2	2	2	2
1223	Isoform 2 of Peptidyl-prolyl cis-trans isomerase NIMA-interacting 4	IPI00006658	0.000	0.35223	4	3	2	5
1224	SRA stem-loop-interacting RNA-binding protein, mitochondrial	IPI00009922	0.000	0.35223	3	3	3	3
1225	Isoform D of Constitutive coactivator of PPAR-gamma-like protein 1	IPI00039626	0.000	0.35223	3	5	5	3
1226	Isoform 1 of Acetolactate synthase-like protein	IPI00554541	0.000	0.35223	3	3	3	3
1227	NEDD8-conjugating enzyme Ubc12	IPI00022597	0.000	0.35223	3	2	1	4

No.	Description	Accession number	STN[1]	p-Value[1]	Con_A[2]	Con_B[2]	CUR_A[2]	CUR_B[2]
1228	Peptidyl-prolyl cis-trans isomerase FKBP2	IPI00002535	0.000	0.35223	2	4	3	3
1229	Isoform 1 of Uncharacterized methyltransferase WBSCR22	IPI00013810	0.000	0.35223	4	4	4	4
1230	Isoform 1 of SEC23-interacting protein	IPI00026969	0.000	0.35223	2	3	1	4
1231	28S ribosomal protein S10, mitochondrial	IPI00061245	0.000	0.35223	4	5	4	5
1232	Leucine-rich repeat-containing protein 40	IPI00152998	0.000	0.35223	2	3	3	2
1233	Isoform UBF1 of Nucleolar transcription factor 1	IPI00014533	0.000	0.35223	0	1	0	1
1234	Cell division protein kinase 2	IPI00031681	0.000	0.35223	2	3	2	3
1235	Condensin complex subunit 2	IPI00299507	0.000	0.35223	1	0	0	1
1236	60S ribosomal protein L8	IPI00012772	0.000	0.35223	1	1	0	1
1237	ATP-dependent RNA helicase DDX50	IPI00031554	0.000	0.35223	3	1	1	3
1238	Isoform 3 of Drebrin-like protein	IPI00101968	0.000	0.35223	3	1	2	2
1239	39S ribosomal protein L9, mitochondrial	IPI00307409	0.000	0.35223	3	2	2	3
1240	Exosome complex exonuclease RRP40	IPI00015956	0.000	0.35223	2	2	1	3
1241	Isoform 2 of Putative methyltransferase NSUN5	IPI00101659	0.000	0.35223	3	2	2	3
1242	Isoform A of Kinesin light chain 1	IPI00020096	0.000	0.35223	2	0	1	2
1243	Probable ATP-dependent RNA helicase DHX37	IPI00217630	0.000	0.35223	2	2	1	3
1244	Isoform 1 of Tumor suppressor p53-binding protein 1	IPI00029778	0.000	0.35223	1	2	2	1
1245	Isoform B of AP-2 complex subunit alpha-1	IPI00256684	0.000	0.35223	0	3	3	0
1246	Isoform 1 of Probable ATP-dependent RNA helicase DHX36	IPI00027415	0.000	0.35223	2	3	3	2
1247	Isoform 2 of Fumarylacetoacetate hydrolase domain-containing protein 1	IPI00440828	0.000	0.35223	2	2	2	2
1248	Putative uncharacterized protein DKFZp686H16220	IPI00552191	0.000	0.35223	2	1	1	2
1249	Ubiquitin carboxyl-terminal hydrolase 13	IPI00024401	0.000	0.35223	2	1	2	1
1250	Isoform 3 of Transcription elongation factor SPT6	IPI00456683	0.000	0.35223	2	2	2	2
1251	Pyridoxal phosphate phosphatase	IPI00025340	0.000	0.35223	3	3	2	4
1252	UPF0670 protein C8orf55	IPI00171421	0.000	0.35223	1	2	0	2
1253	DNA ligase 1	IPI00219841	0.000	0.35223	2	2	2	2
1254	60S ribosomal protein L17	IPI00413324	0.000	0.35223	2	3	2	3
1255	Isoform 3 of Protein VPRBP	IPI00181396	0.000	0.35223	1	2	2	1
1256	Toll-interacting protein	IPI00100154	0.000	0.35223	3	3	3	3
1257	Isoform 1 of UPF0424 protein C1orf128	IPI00015351	0.000	0.35223	1	0	1	1
1258	28S ribosomal protein S18b, mitochondrial	IPI00022316	0.000	0.35223	1	3	2	2
1259	Isoform 1 of ATP-binding cassette sub-family F member 3	IPI00465160	0.000	0.35223	2	2	2	2
1260	Isoform 2 of Torsin-1A-interacting protein 1	IPI00012280	0.000	0.35223	3	3	3	3
1261	Isoform 2 of Membrane magnesium transporter 1	IPI00166785	0.000	0.35223	2	1	1	2
1262	39S ribosomal protein L17, mitochondrial	IPI00172591	0.000	0.35223	3	3	3	3
1263	Selenide, water dikinase 1	IPI00029056	0.000	0.35223	3	2	3	2
1264	Isoform 1 of Nck-associated protein 1	IPI00031982	0.000	0.35223	3	1	2	2
1265	DnaJ homolog subfamily C member 7	IPI00329629	0.000	0.35223	3	3	3	3
1266	Isoform 3 of Yorkie homolog	IPI00009326	0.000	0.35223	0	1	1	0
1267	Isoform 1 of Neurochondrin	IPI00549543	0.000	0.35223	2	3	2	3
1268	twinfilin-1	IPI00183508	0.000	0.35223	3	3	4	2
1269	ESF1 homolog	IPI00024167	0.000	0.35223	2	2	3	1
1270	Guanine nucleotide-binding protein subunit alpha-13	IPI00290928	0.000	0.35223	10	5	8	7
1271	Isoform 1 of Ral GTPase-activating protein subunit beta	IPI00409601	0.000	0.35223	0	1	0	1
1272	Isoform 1 of Translation initiation factor eIF-2B subunit delta	IPI00005979	0.000	0.35223	3	2	3	2
1273	Ras-related protein Rab-13	IPI00016373	0.000	0.35223	4	4	4	4
1274	Isoform 2 of Peptidyl-prolyl cis-trans isomerase-like 3	IPI00032473	0.000	0.35223	1	2	2	1
1275	Isoform 1 of AP-3 complex subunit beta-1	IPI00021129	0.000	0.35223	2	0	2	1
1276	PRMT3 protein (Fragment)	IPI00103026	0.000	0.35223	2	2	3	1
1277	Isoform 2 of Ubiquitin carboxyl-terminal hydrolase isozyme L5	IPI00219512	0.000	0.35223	2	2	1	3
1278	Isoform 1 of HCLS1-associated protein X-1	IPI00010440	0.000	0.35223	0	1	1	1
1279	Isoform 1 of 28S ribosomal protein S35, mitochondrial	IPI00073779	0.000	0.35223	1	2	1	2
1280	5-formyltetrahydrofolate cyclo-ligase	IPI00220567	0.000	0.35223	2	3	2	3
1281	Isoform 3 of Guanine nucleotide exchange factor VAV2	IPI00004977	0.000	0.35223	2	2	2	2
1282	D-dopachrome decarboxylase	IPI00293867	0.000	0.35223	4	3	4	3
1283	WD repeat domain 57 (U5 snRNP specific), isoform CRA_b	IPI00385642	0.000	0.35223	0	3	2	2
1284	Isoform A of 5'-AMP-activated protein kinase subunit gamma-2	IPI00005367	0.000	0.35223	1	0	1	1
1285	Isoform 2 of Low molecular weight phosphotyrosine protein phosphatase	IPI00218847	0.000	0.35223	3	4	4	3
1286	39S ribosomal protein L40, mitochondrial	IPI00099871	0.000	0.35223	1	2	0	2
1287	Isoform 2 of Syntaxin-binding protein 1	IPI00046057	0.000	0.35223	0	2	0	2
1288	Ribosome biogenesis protein NSA2 homolog	IPI00007089	0.000	0.35223	3	3	3	3
1289	Rho-associated protein kinase 1	IPI00022542	0.000	0.35223	0	1	1	1
1290	cDNA FLJ78497	IPI00289535	0.000	0.35223	1	3	2	2
1291	Isoform Long of Transcription intermediary factor 1-alpha	IPI00005184	0.000	0.35223	1	1	1	1
1292	Isoform 1 of Pleiotropic regulator 1	IPI00002624	0.000	0.35223	2	2	3	0
1293	cDNA FLJ10824 fis, clone NT2RP4001086 (Fragment)	IPI00294810	0.000	0.35223	2	2	2	2
1294	KIF1-binding protein	IPI00477355	0.000	0.35223	1	3	2	2
1295	Ribosome biogenesis regulatory protein homolog	IPI00014253	0.000	0.35223	3	3	2	4
1296	Isoform 1 of N-alpha-acetyltransferase 25, NatB auxiliary subunit	IPI00025890	0.000	0.35223	2	2	2	2
1297	Emerin	IPI00032003	0.000	0.35223	3	3	3	3
1298	LAG1 longevity assurance homolog 2	IPI00305304	0.000	0.35223	1	2	2	0
1299	General transcription factor IIF subunit 2	IPI00477686	0.000	0.35223	1	1	1	1
1300	Retinol dehydrogenase 14	IPI00177940	0.000	0.35223	3	2	2	3
1301	RWD domain-containing protein 1	IPI00034010	0.000	0.35223	1	1	1	0
1302	Protein phosphatase 1F	IPI00291412	0.000	0.35223	1	1	1	1
1303	Isoform 1 of E3 ubiquitin-protein ligase BRE1B	IPI00162563	0.000	0.35223	1	2	2	1
1304	Isoform 1 of Nucleolar protein 14	IPI00022613	0.000	0.35223	1	1	1	1
1305	39S ribosomal protein L18, mitochondrial	IPI00160421	0.000	0.35223	4	3	4	3
1306	Isoform 1 of Protein timeless homolog	IPI00335541	0.000	0.35223	0	0	0	0
1307	Dihydroorotate dehydrogenase, mitochondrial	IPI00024462	0.000	0.35223	1	3	2	2
1308	Ubiquitin domain-containing protein UBFD1	IPI00005194	0.000	0.35223	3	2	3	2
1309	Isoform 1 of Transcription elongation factor A protein 1	IPI00333215	0.000	0.35223	2	3	3	2
1310	Heat shock-related 70 kDa protein 2	IPI00007702	0.000	0.35223	3	2	3	2
1311	Isoform 1 of Disks large homolog 1	IPI00030351	0.000	0.35223	1	2	1	2
1312	Small nuclear ribonucleoprotein F	IPI00220528	0.000	0.35223	1	2	1	2
1313	Cysteine and glycine-rich protein 1	IPI00442073	0.000	0.35223	1	2	1	2
1314	Gigaxonin	IPI00022758	0.000	0.35223	0	2	1	2
1315	Isoform 1 of Regulation of nuclear pre-mRNA domain-containing protein 2	IPI00384541	0.000	0.35223	1	1	0	0
1316	Isoform 1 of Rhotekin	IPI00029834	0.000	0.35223	2	2	1	3
1317	Heme oxygenase 1	IPI00215893	0.000	0.35223	0	0	0	0
1318	Protein AATF	IPI00302238	0.000	0.35223	2	2	2	2
1319	Replication protein A 14 kDa subunit	IPI00017373	0.000	0.35223	1	3	1	3
1320	Isoform 1 of Protein POF1B	IPI00103242	0.000	0.35223	2	2	2	2
1321	Nucleoside diphosphate-linked moiety X motif 19, mitochondrial	IPI00869107	0.000	0.35223	1	0	1	1
1322	39S ribosomal protein L15, mitochondrial	IPI00023086	0.000	0.35223	2	1	1	2

No.	Description	Accession number	STN[1]	p-Value[1]	Con_A[2]	Con_B[2]	CUR_A[2]	CUR_B[2]
1323	Peptidase M20 domain-containing protein 2	IPI00217852	0.000	0.35223	0	1	0	0
1324	Cell division protein kinase 3	IPI00023503	0.000	0.35223	2	1	2	0
1325	Isoform 1 of 28S ribosomal protein S5, mitochondrial	IPI00169400	0.000	0.35223	3	2	3	2
1326	NDUFB10 protein	IPI00074489	0.000	0.35223	1	2	2	0
1327	Caspase-3	IPI00292140	0.000	0.35223	2	1	1	2
1328	Putative transferase C1orf69, mitochondrial	IPI00145260	0.000	0.35223	1	2	2	0
1329	Acetyl-coenzyme A synthetase, cytoplasmic	IPI00413730	0.000	0.35223	1	2	2	1
1330	Isoform 2 of Transducin-like enhancer protein 3	IPI00177938	0.000	0.35223	1	2	1	2
1331	Cyclin-H	IPI00021305	0.000	0.35223	2	2	2	2
1332	Active regulator of SIRT1	IPI00219006	0.000	0.35223	3	3	3	3
1333	GTP-binding protein GUF1 homolog	IPI00296563	0.000	0.35223	1	1	0	1
1334	Pre-mRNA-splicing factor SYF1	IPI00163084	0.000	0.35223	1	1	1	0
1335	Isoform 3 of Cytosolic 5'-nucleotidase 3	IPI00100192	0.000	0.35223	1	1	1	1
1336	Protein FAM50A	IPI00030098	0.000	0.35223	2	2	3	1
1337	Ribonuclease UK114	IPI00005038	0.000	0.35223	2	3	3	2
1338	Tryptophanyl-tRNA synthetase, mitochondrial	IPI00025050	0.000	0.35223	0	2	2	1
1339	Isoform 2A of GTPase KRas	IPI00423568	0.000	0.35223	0	1	1	1
1340	H/ACA ribonucleoprotein complex subunit 2	IPI00041325	0.000	0.35223	1	1	1	0
1341	Proteasome maturation protein	IPI00006377	0.000	0.35223	2	3	2	3
1342	Serine/threonine-protein phosphatase 2A 56 kDa regulatory subunit alpha isoform	IPI00014978	0.000	0.35223	1	1	1	0
1343	Myosin-Ia	IPI00294386	0.000	0.35223	2	1	2	0
1344	15 kDa protein	IPI00000186	0.000	0.35223	2	2	2	2
1345	NADH dehydrogenase [ubiquinone] 1 beta subcomplex subunit 8, mitochondrial	IPI00028883	0.000	0.35223	2	2	2	2
1346	TBC1 domain family member 15 isoform 1	IPI00154645	0.000	0.35223	0	1	0	1
1347	Synaptosomal-associated protein 29	IPI00032831	0.000	0.35223	2	1	2	1
1348	Isoform 1 of Syntaxin-7	IPI00289876	0.000	0.35223	2	2	3	1
1349	Keratin, type II cytoskeletal 6B	IPI00293665	0.000	0.35223	1	1	0	0
1350	Charged multivesicular body protein 4b	IPI00025974	0.000	0.35223	2	1	2	1
1351	N-alpha-acetyltransferase 10, NatA catalytic subunit	IPI00013184	0.000	0.35223	2	2	3	1
1352	D-beta-hydroxybutyrate dehydrogenase, mitochondrial	IPI00025341	0.000	0.35223	1	2	2	1
1353	Guanine nucleotide-binding protein G(q) subunit alpha	IPI00288947	0.000	0.35223	2	0	1	2
1354	cDNA FLJ56176, highly similar to Poly(A) polymerase alpha	IPI00384028	0.000	0.35223	2	1	2	1
1355	Isoform 1 of Torsin-3A	IPI00301631	0.000	0.35223	0	1	0	0
1356	Digestive organ expansion factor homolog	IPI00004290	0.000	0.35223	1	2	1	2
1357	Isoform 1 of Erlin-2	IPI00026942	0.000	0.35223	2	1	0	2
1358	Cyclin B1	IPI00294696	0.000	0.35223	1	0	0	1
1359	inosine-5'-monophosphate dehydrogenase 1 isoform a	IPI00375527	0.000	0.35223	0	1	1	1
1360	Isoform 1 of Regulator of nonsense transcripts 3B	IPI00023409	0.000	0.35223	1	3	3	1
1361	Transcription factor BTF3 homolog 4	IPI00412792	0.000	0.35223	2	1	2	1
1362	alanyl-tRNA editing protein Aarsd1 isoform 1	IPI00748490	0.000	0.35223	1	2	1	2
1363	RNA methyltransferase-like protein 1	IPI00335589	0.000	0.35223	1	1	0	0
1364	Isoform 1 of Mannose-1-phosphate guanyltransferase alpha	IPI00101782	0.000	0.35223	1	0	1	1
1365	Signal peptidase complex catalytic subunit SEC11C	IPI00219436	0.000	0.35223	2	1	2	1
1366	3'-5' exoribonuclease CSL4 homolog	IPI00032823	0.000	0.35223	2	2	2	2
1367	Isoform 2 of Protein PAT1 homolog 1	IPI00760958	0.000	0.35223	1	1	1	1
1368	Isoform CSBP2 of Mitogen-activated protein kinase 14	IPI00002857	0.000	0.35223	2	1	0	2
1369	Short/branched chain specific acyl-CoA dehydrogenase, mitochondrial	IPI00024623	0.000	0.35223	1	3	3	1
1370	Ubiquitin-conjugating enzyme E2 B	IPI00012060	0.000	0.35223	0	1	0	0
1371	WD repeat-containing protein 46	IPI00023126	0.000	0.35223	2	2	2	2
1372	Isoform 3 of Exocyst complex component 7	IPI00103064	0.000	0.35223	1	2	1	2
1373	Isoform 1 of Craniofacial development protein 1	IPI00007306	0.000	0.35223	1	1	1	1
1374	cDNA FLJ54848, highly similar to tRNA-splicing endonuclease subunit Sen34	IPI00451941	0.000	0.35223	1	1	0	1
1375	Torsin A interacting protein 1	IPI00644766	0.000	0.35223	1	2	2	1
1376	Putative uncharacterized protein DKFZp686E2459	IPI00375731	0.000	0.35223	1	1	0	1
1377	V-type proton ATPase subunit C 1	IPI00007814	0.000	0.35223	0	1	0	0
1378	ubiquitin-like with PHD and ring finger domains 1 isoform 2	IPI00797279	0.000	0.35223	1	1	0	0
1379	Origin recognition complex subunit 6	IPI00001641	0.000	0.35223	1	1	0	0
1380	Isoform 1 of Testin	IPI00024097	0.000	0.35223	0	0	1	1
1381	Ubiquitin-conjugating enzyme E2 variant 2	IPI00019600	0.000	0.35223	1	2	1	2
1382	Trafficking protein particle complex subunit 3	IPI00004324	0.000	0.35223	1	1	1	1
1383	Protein KTI12 homolog	IPI00061528	0.000	0.35223	0	0	1	0
1384	Isoform 1 of Protein FAM115A	IPI00006050	0.000	0.35223	0	1	0	0
1385	NADH dehydrogenase [ubiquinone] 1 beta subcomplex subunit 7	IPI00219772	0.000	0.35223	2	2	2	2
1386	Isoform 1 of Endophilin-B1	IPI00006558	0.000	0.35223	1	1	1	1
1387	Isoform 2 of VIP36-like protein	IPI00218337	0.000	0.35223	0	0	1	0
1388	Seryl-tRNA synthetase, mitochondrial	IPI00328361	0.000	0.35223	1	1	0	1
1389	Isoform 1 of CTD small phosphatase-like protein 2	IPI00033054	0.000	0.35223	1	0	1	0
1390	Isoform 1 of Kinesin-like protein KIF1B	IPI00029011	0.000	0.35223	1	0	1	0
1391	Actin-related protein 5	IPI00292787	0.000	0.35223	2	1	2	0
1392	Targeting protein for Xklp2	IPI00008477	0.000	0.35223	0	0	0	0
1393	Isoform 2 of Syntaxin-5	IPI00386786	0.000	0.35223	1	0	0	0
1394	Protein tyrosine phosphatase type IVA 1	IPI00020164	0.000	0.35223	1	0	0	1
1395	Isoform 1 of N-alpha-acetyltransferase 40, NatD catalytic subunit	IPI00328847	0.000	0.35223	1	0	0	0
1396	Isoform 6 of Terminal uridylyltransferase 7	IPI00336000	0.000	0.35223	0	1	0	1
1397	DEAH (Asp-Glu-Ala-His) box polypeptide 16	IPI00292510	0.000	0.35223	1	1	1	0
1398	Queuine tRNA-ribosyltransferase	IPI00215974	0.000	0.35223	1	2	1	2
1399	Borealin	IPI00303099	0.000	0.35223	1	1	0	0
1400	Isoform 1 of Microtubule-associated protein RP/EB family member 2	IPI00003420	0.000	0.35223	1	1	1	1
1401	Prolactin regulatory element-binding protein	IPI00033349	0.000	0.35223	1	1	1	1
1402	Alpha- and gamma-adaptin-binding protein p34	IPI00100193	0.000	0.35223	0	1	1	0
1403	cDNA FLJ56221, highly similar to YTH domain protein 3	IPI00396131	0.000	0.35223	1	1	1	0
1404	Isoform 1 of Protein kinase C and casein kinase substrate in neurons protein 2	IPI00027009	0.000	0.35223	1	1	1	1
1405	WD repeat-containing protein 70	IPI00300060	0.000	0.35223	1	1	0	1
1406	BTB/POZ domain-containing protein KCTD14	IPI00181836	0.000	0.35223	1	1	1	0
1407	Isoform 1 of Anaphase-promoting complex subunit 5	IPI00008247	0.000	0.35223	0	0	0	0
1408	Profilin	IPI00107555	0.000	0.35223	0	0	0	1
1409	Isoform 1 of Set1/Ash2 histone methyltransferase complex subunit ASH2	IPI00328658	0.000	0.35223	2	1	1	2
1410	Isoform 2 of Kinesin-like protein KIF2C	IPI00216113	0.000	0.35223	0	0	0	0
1411	KRR1 small subunit processome component homolog	IPI00156032	0.000	0.35223	0	0	0	0
1412	Autocrine motility factor receptor, isoform 2	IPI00423874	0.000	0.35223	0	1	1	1
1413	cDNA FLJ60094, highly similar to F-actin capping protein subunit beta	IPI00218782	0.000	0.35223	2	0	2	0
1414	Coiled-coil domain-containing protein 137	IPI00401962	0.000	0.35223	0	1	0	0
1415	Iron-sulfur cluster assembly 2 homolog, mitochondrial	IPI00376195	0.000	0.35223	1	1	1	1
1416	Full-length cDNA clone CS0DI031YK16 of Placenta of Homo sapiens	IPI00384157	0.000	0.35223	0	2	2	0
1417	TBC1 domain family member 7	IPI00009425	0.000	0.35223	0	0	1	1

No.	Description	Accession number	STN[1]	p-Value[1]	Con_A[2]	Con_B[2]	CUR_A[2]	CUR_B[2]
1418	Isoform 1 of Glycerol kinase	IPI00027424	0.000	0.35223	1	0	1	1
1419	Peptidyl-prolyl cis-trans isomerase A	IPI00419585	-0.089	0.35894	69	72	72	68
1420	Isoform B1 of Heterogeneous nuclear ribonucleoproteins A2/B1	IPI00396378	-0.109	0.35738	263	291	267	285
1421	Stress-induced-phosphoprotein 1	IPI00013894	-0.111	0.35681	39	38	39	37
1422	60S ribosomal protein L18	IPI00215719	-0.120	0.35530	21	41	28	33
1423	CTP synthase 1	IPI00290142	-0.125	0.35421	27	29	28	27
1424	Plastin-3	IPI00216694	-0.125	0.35421	29	27	28	27
1425	Mitochondrial 2-oxoglutarate/malate carrier protein	IPI00219729	-0.126	0.35336	28	27	28	26
1426	Isoform 2 of Spliceosome RNA helicase BAT1	IPI00641829	-0.127	0.35303	27	27	26	27
1427	Proteasome subunit beta type-3	IPI00028004	-0.128	0.35303	23	30	26	26
1428	Translin	IPI00018768	-0.131	0.35195	23	26	24	24
1429	Putative uncharacterized protein SPTAN1	IPI00745092	-0.140	0.34921	21	20	22	18
1430	Isoform 1 of Poly(U)-binding-splicing factor PUF60	IPI00069750	-0.140	0.34921	22	19	21	19
1431	Acetyl-CoA acetyltransferase, mitochondrial	IPI00030363	-0.141	0.34892	20	20	20	19
1432	Programmed cell death 6-interacting protein	IPI00246058	-0.144	0.34883	20	18	19	18
1433	Putative uncharacterized protein NAP1L4	IPI00017763	-0.145	0.34854	18	19	19	17
1434	Glutathione S-transferase kappa 1	IPI00219673	-0.147	0.34826	16	20	18	17
1435	Single-stranded DNA-binding protein, mitochondrial	IPI00029744	-0.147	0.34826	20	16	20	15
1436	ATP synthase subunit b, mitochondrial	IPI00029133	-0.147	0.34826	19	17	19	16
1437	Glucosamine-6-phosphate isomerase 1	IPI00009305	-0.153	0.34637	16	16	16	15
1438	Adenylate kinase isoenzyme 1	IPI00018342	-0.153	0.34637	16	16	17	14
1439	Actin-related protein 2	IPI00005159	-0.153	0.34637	15	17	17	14
1440	tRNA (cytosine-5-)-methyltransferase NSUN2	IPI00306369	-0.157	0.34533	17	13	16	13
1441	26S proteasome non-ATPase regulatory subunit 6	IPI00014151	-0.157	0.34533	17	13	14	15
1442	Protein mago nashi homolog 2	IPI00059292	-0.159	0.34486	15	14	15	13
1443	Ras GTPase-activating-like protein IQGAP1	IPI00009342	-0.160	0.34425	95	96	91	98
1444	Isoform 1 of ER lumen protein retaining receptor 2	IPI00018248	-0.166	0.34311	14	12	10	15
1445	Ubiquitin-like modifier activating enzyme 1	IPI00552452	-0.166	0.34311	14	12	13	12
1446	Eukaryotic translation initiation factor 2 subunit 3	IPI00297982	-0.168	0.34226	14	11	13	11
1447	Coatomer subunit zeta-1	IPI00032851	-0.168	0.34226	13	12	13	11
1448	Enoyl-CoA hydratase, mitochondrial	IPI00024993	-0.173	0.33976	12	11	12	10
1449	Isoform 1 of Nuclear pore complex protein Nup155	IPI00026625	-0.179	0.33683	12	9	9	11
1450	Isoform 1 of LIM and SH3 domain protein 1	IPI00000861	-0.179	0.33683	11	10	11	9
1451	Isoform 1 of Ubiquitin-conjugating enzyme E2 variant 1	IPI00019599	-0.179	0.33683	11	10	11	9
1452	cDNA FLJ40024 fis, clone STOMA2007745, highly similar to UBIQUITIN-ACTIVATING ENZYME E1	IPI00026119	-0.181	0.33570	66	70	64	70
1453	Isoform 1 of Fragile X mental retardation syndrome-related protein 1	IPI00016249	-0.183	0.33565	9	11	11	8
1454	ATP-dependent RNA helicase DHX29	IPI00217413	-0.183	0.33565	10	10	9	10
1455	Eukaryotic peptide chain release factor subunit 1	IPI00429191	-0.183	0.33565	10	10	10	9
1456	F-actin-capping protein subunit alpha-2	IPI00026182	-0.183	0.33565	10	10	8	11
1457	Exosome complex exonuclease RRP4	IPI00015905	-0.186	0.33409	10	9	11	7
1458	Isoform 1 of Ubiquitin-like modifier-activating enzyme 6	IPI00023647	-0.186	0.33409	11	8	9	9
1459	Isoform 1 of 2',5'-phosphodiesterase 12	IPI00174390	-0.186	0.33409	10	9	9	9
1460	Isoform 2 of Proteasome subunit alpha type-3	IPI00171199	-0.190	0.33281	8	10	9	8
1461	Cytochrome c oxidase subunit 4 isoform 1, mitochondrial	IPI00006579	-0.190	0.33281	10	8	8	9
1462	Isoform 1 of Paraspeckle component 1	IPI00103525	-0.190	0.33281	9	9	10	7
1463	Isoform 2 of Ubiquitin-associated domain-containing protein 2	IPI00007034	-0.190	0.33281	9	9	9	8
1464	Isoform 2 of Cat eye syndrome critical region protein 5	IPI00011511	-0.190	0.33281	9	9	9	8
1465	DCN1-like protein 5	IPI00165361	-0.194	0.33078	5	12	8	8
1466	Isoform 1 of Protein unc-45 homolog A	IPI00072534	-0.194	0.33078	10	7	8	8
1467	Gamma-aminobutyric acid receptor-associated protein-like 2	IPI00026358	-0.194	0.33078	9	8	8	8
1468	Interferon-induced, double-stranded RNA-activated protein kinase	IPI00019463	-0.194	0.33078	9	8	8	8
1469	Leucine-rich repeat-containing protein 47	IPI00170935	-0.194	0.33078	9	8	8	8
1470	Pre-mRNA branch site protein p14	IPI00032827	-0.194	0.33078	9	8	9	7
1471	Ras-related protein Rab-6B	IPI00016891	-0.198	0.32941	8	8	8	7
1472	Thioredoxin domain-containing protein 12	IPI00026328	-0.198	0.32941	8	8	8	7
1473	Chloride intracellular channel protein 4	IPI00001960	-0.203	0.32729	9	6	9	5
1474	Isoform 1 of Phosphatidylinositol transfer protein beta isoform	IPI00334907	-0.203	0.32729	7	8	7	7
1475	Transforming protein RhoA	IPI00478231	-0.203	0.32729	8	7	7	7
1476	Hsc70-interacting protein	IPI00032826	-0.203	0.32729	7	8	8	6
1477	Isoform F of Protein SON	IPI00000192	-0.209	0.32535	7	7	8	5
1478	Isoform SERCA1B of Sarcoplasmic/endoplasmic reticulum calcium ATPase 1	IPI00024804	-0.209	0.32535	8	6	6	7
1479	NADH dehydrogenase [ubiquinone] 1 alpha subcomplex subunit 10, mitochondrial	IPI00029561	-0.209	0.32535	9	5	5	8
1480	Isoform 2 of Epimerase family protein SDR39U1	IPI00643286	-0.209	0.32535	7	7	7	6
1481	Isoform 2 of Phosphoglucomutase-1	IPI00217872	-0.209	0.32535	7	7	8	5
1482	Isoform NELF-C of Negative elongation factor C/D	IPI00164949	-0.209	0.32535	8	6	8	5
1483	Isoform 1 of Elongation factor G, mitochondrial	IPI00154473	-0.209	0.32535	7	7	7	6
1484	Serpin B6	IPI00413451	-0.209	0.32535	7	7	8	5
1485	Tubulin-specific chaperone A	IPI00217236	-0.209	0.32535	6	8	6	7
1486	60S acidic ribosomal protein P1	IPI00008527	-0.209	0.32535	7	7	6	7
1487	PNAS-139	IPI00000477	-0.214	0.32190	6	7	5	7
1488	Signal peptidase complex catalytic subunit SEC11A	IPI00104128	-0.214	0.32190	6	7	7	5
1489	Probable ATP-dependent RNA helicase DDX10	IPI00297900	-0.214	0.32190	6	7	5	7
1490	Isoform 1 of Heterogeneous nuclear ribonucleoprotein U-like protein 1	IPI00013070	-0.214	0.32190	8	5	7	5
1491	Phosphoglycerate mutase 2	IPI00218570	-0.214	0.32190	6	7	6	6
1492	Isoform 2 of Serrate RNA effector molecule homolog	IPI00220038	-0.214	0.32190	6	7	6	6
1493	Isoform 3 of PCI domain-containing protein 2	IPI00072541	-0.214	0.32190	6	7	7	5
1494	DnaJ homolog subfamily B member 1	IPI00015947	-0.214	0.32190	6	7	6	6
1495	Developmentally-regulated GTP-binding protein 1	IPI00031836	-0.214	0.32190	7	6	6	6
1496	Phosphatidylinositol transfer protein alpha isoform	IPI00216048	-0.214	0.32190	8	5	5	7
1497	Histone H3.2	IPI00171611	-0.221	0.31897	6	6	6	5
1498	H/ACA ribonucleoprotein complex subunit 4	IPI00221394	-0.221	0.31897	6	6	7	4
1499	Isoform 1 of 60S ribosome subunit biogenesis protein NIP7 homolog	IPI00007175	-0.221	0.31897	5	7	4	7
1500	cohesin subunit SA-2 isoform a	IPI00470883	-0.221	0.31897	5	7	5	6
1501	39S ribosomal protein L28, mitochondrial	IPI00172594	-0.221	0.31897	6	6	6	5
1502	Putative uncharacterized protein ZFR	IPI00748303	-0.221	0.31897	6	6	5	6
1503	Isoform 3 of DNA repair protein RAD50	IPI00107531	-0.221	0.31897	6	6	3	8
1504	DnaJ homolog subfamily C member 9	IPI00154975	-0.221	0.31897	5	7	5	6
1505	Protein FRG1	IPI00004655	-0.221	0.31897	6	6	5	6
1506	Isoform 1 of RNA-binding protein 14	IPI00013174	-0.221	0.31897	5	7	6	5
1507	Translational activator GCN1	IPI00001159	-0.226	0.31458	113	112	104	118
1508	Eukaryotic translation initiation factor 3 subunit G	IPI00290460	-0.228	0.31458	5	6	6	4
1509	Isoform 2 of Isopentenyl-diphosphate Delta-isomerase 1	IPI00220014	-0.228	0.31458	5	6	6	4
1510	Catalase	IPI00465436	-0.228	0.31458	5	6	4	6
1511	Isoform 2 of CDK5 regulatory subunit-associated protein 3	IPI00018780	-0.228	0.31458	6	5	5	5
1512	Isoform 2 of cAMP-dependent protein kinase catalytic subunit alpha	IPI00217960	-0.228	0.31458	7	4	6	4

No.	Description	Accession number	STN[1]	p-Value[1]	Con_A[2]	Con_B[2]	CUR_A[2]	CUR_B[2]
1513	Succinate dehydrogenase [ubiquinone] iron-sulfur subunit, mitochondrial	IPI00294911	-0.228	0.31458	6	5	5	5
1514	Putative high mobility group protein B3-like-1	IPI00006437	-0.228	0.31458	5	6	5	5
1515	Transmembrane protein 14C	IPI00009346	-0.237	0.31037	6	4	4	5
1516	Ribosomal protein L1	IPI00035167	-0.237	0.31037	6	4	5	4
1517	Tubulin-specific chaperone E	IPI00018402	-0.237	0.31037	5	5	4	5
1518	Putative rRNA methyltransferase 3	IPI00217686	-0.237	0.31037	5	5	4	5
1519	Ras-related protein Rab-5A	IPI00023510	-0.237	0.31037	6	4	4	5
1520	Isoform 1 of OCIA domain-containing protein 2	IPI00555902	-0.237	0.31037	5	5	6	3
1521	synembryn-A	IPI00100106	-0.237	0.31037	4	6	4	5
1522	Crk-like protein	IPI00004839	-0.237	0.31037	5	5	4	5
1523	Ribosome biogenesis protein BOP1	IPI00028955	-0.237	0.31037	6	4	6	3
1524	CTP synthase 2	IPI00645702	-0.237	0.31037	6	4	4	5
1525	Isoform 1 of Huntingtin-interacting protein K	IPI00335001	-0.237	0.31037	5	5	3	6
1526	Transmembrane 9 superfamily member 4	IPI00021985	-0.237	0.31037	5	5	5	4
1527	WD repeat-containing protein 82	IPI00152695	-0.237	0.31037	6	4	5	4
1528	Glycylpeptide N-tetradecanoyltransferase 2	IPI00030223	-0.237	0.31037	5	5	4	5
1529	Isoform Rpn10A of 26S proteasome non-ATPase regulatory subunit 4	IPI00022694	-0.237	0.31037	5	5	4	5
1530	Isoform 1 of DDRGK domain-containing protein 1	IPI00028387	-0.237	0.31037	5	5	5	4
1531	Isoform Beta of Heat shock protein 105 kDa	IPI00218993	-0.246	0.30362	29	30	30	27
1532	Isoform 1 of Methylthioribose-1-phosphate isomerase	IPI00005948	-0.247	0.30362	5	4	3	5
1533	39S ribosomal protein L1, mitochondrial	IPI00549381	-0.247	0.30362	3	6	4	4
1534	CCAAT/enhancer-binding protein zeta	IPI00306723	-0.247	0.30362	3	6	6	2
1535	Isoform Alpha-6X1X2B of Integrin alpha-6	IPI00010697	-0.247	0.30362	4	5	4	4
1536	Isoform 3 of Chitinase domain-containing protein 1	IPI00045536	-0.247	0.30362	5	4	3	5
1537	Probable fructose-2,6-bisphosphatase TIGAR	IPI00006907	-0.247	0.30362	5	4	4	4
1538	Mitochondrial import inner membrane translocase subunit TIM44	IPI00306516	-0.247	0.30362	3	6	5	3
1539	Isoform 1 of Medium-chain specific acyl-CoA dehydrogenase, mitochondrial	IPI00005040	-0.247	0.30362	5	4	5	3
1540	Isoform 1 of Peroxisomal acyl-coenzyme A oxidase 1	IPI00296907	-0.247	0.30362	5	4	4	4
1541	Ewing sarcoma breakpoint region 1 isoform 1	IPI00009841	-0.247	0.30362	4	5	4	4
1542	ubiquitin-like protein fubi and ribosomal protein S30 precursor	IPI00019770	-0.247	0.30362	5	4	4	4
1543	Isoform 2 of Protein SET	IPI00301311	-0.247	0.30362	6	3	5	3
1544	Uncharacterized protein C7orf50	IPI00031651	-0.247	0.30362	4	5	4	4
1545	REST corepressor 1	IPI00008531	-0.247	0.30362	5	4	4	4
1546	Isoform 2 of Apoptosis inhibitor 5	IPI00554742	-0.252	0.29823	30	25	28	25
1547	Talin-1	IPI00298994	-0.258	0.29795	83	75	82	73
1548	Isoform 2 of Titin	IPI00023283	-0.258	0.29734	2	6	3	4
1549	COMM domain-containing protein 2	IPI00456048	-0.258	0.29734	5	3	3	4
1550	Testis-expressed sequence 10 protein	IPI00549664	-0.258	0.29734	4	4	4	3
1551	Ran-binding protein 6	IPI00514622	-0.258	0.29734	4	4	3	4
1552	Isoform 2 of Ubiquitin thioesterase OTUB1	IPI00409750	-0.258	0.29734	5	3	3	4
1553	Similar to Zinc finger CCCH domain-containing protein 15	IPI00000279	-0.258	0.29734	4	4	4	3
1554	Cell division protein kinase 5	IPI00023530	-0.258	0.29734	3	5	3	4
1555	Hsp90 co-chaperone Cdc37	IPI00013122	-0.258	0.29734	4	4	3	4
1556	cDNA FLJ56825, highly similar to WD repeat protein 57	IPI00006723	-0.258	0.29734	5	3	5	2
1557	F-box-like/WD repeat-containing protein TBL1XR1	IPI00002922	-0.258	0.29734	5	3	4	3
1558	Isoform 1 of 3'(2'),5'-bisphosphate nucleotidase 1	IPI00410214	-0.258	0.29734	4	4	4	3
1559	Coiled-coil-helix-coiled-coil-helix domain-containing protein 3, mitochondrial	IPI00015833	-0.258	0.29734	3	5	3	4
1560	C-terminal-binding protein 1	IPI00012835	-0.258	0.29734	4	4	4	3
1561	Serine/threonine-protein phosphatase 4 catalytic subunit	IPI00012833	-0.258	0.29734	5	3	3	4
1562	Ribosomal L1 domain-containing protein 1	IPI00008708	-0.265	0.28912	20	28	23	23
1563	14-3-3 protein theta	IPI00018146	-0.265	0.28912	24	24	23	23
1564	Isoform 1 of Cleavage and polyadenylation specificity factor subunit 6	IPI00012998	-0.270	0.28874	25	21	24	20
1565	Isoform 2 of Double-stranded RNA-specific adenosine deaminase	IPI00025057	-0.272	0.28831	5	2	3	3
1566	Pumilio domain-containing protein C14orf21	IPI00216999	-0.272	0.28831	3	4	4	2
1567	mortality factor 4	IPI00001955	-0.272	0.28831	3	4	3	3
1568	DCN1-like protein 1	IPI00291893	-0.272	0.28831	4	3	4	2
1569	Isoform 1 of DAZ-associated protein 1	IPI00165230	-0.272	0.28831	4	3	3	3
1570	Full-length cDNA 5-PRIME end of clone CS0DJ009YL13 of T cells (Jurkat cell line) of Homo sapiens	IPI00384016	-0.272	0.28831	4	3	3	3
1571	Ubiquitin-conjugating enzyme E2 G1	IPI00219783	-0.272	0.28831	3	4	3	3
1572	Oxysterol-binding protein	IPI00163644	-0.272	0.28831	2	5	3	3
1573	Isoform 1 of Transmembrane protein 111	IPI00020472	-0.272	0.28831	4	3	3	3
1574	Nitric oxide synthase-interacting protein	IPI00006408	-0.272	0.28831	3	4	4	2
1575	Isoform 2 of Leucine-rich repeat flightless-interacting protein 1	IPI00006207	-0.272	0.28831	4	3	4	2
1576	Isoform 2 of DnaJ homolog subfamily C member 2	IPI00455199	-0.272	0.28831	3	4	3	3
1577	Flotillin-1	IPI00027438	-0.272	0.28831	3	4	3	3
1578	NADH dehydrogenase [ubiquinone] 1 beta subcomplex subunit 4	IPI00220059	-0.272	0.28831	3	4	3	3
1579	Phosphomevalonate kinase	IPI00220648	-0.272	0.28831	3	4	3	3
1580	Serine/threonine-protein phosphatase PP1-beta catalytic subunit	IPI00218236	-0.272	0.28831	4	3	4	2
1581	cDNA FLJ61162, highly similar to Ras-related protein R-Ras2	IPI00012512	-0.272	0.28831	4	3	3	3
1582	28 kDa heat- and acid-stable phosphoprotein	IPI00013297	-0.272	0.28831	5	2	3	3
1583	Isoform 1 of Deoxycytidylate deaminase	IPI00296863	-0.272	0.28831	3	4	3	3
1584	Isoform 1 of Zinc finger protein 207	IPI00013457	-0.272	0.28831	3	4	3	3
1585	cDNA FLJ54710, highly similar to Target of Myb protein 1	IPI00023191	-0.272	0.28831	5	2	3	3
1586	cDNA FLJ56343, highly similar to Torsin A	IPI00413293	-0.272	0.28831	3	4	4	2
1587	Isoform 1 of Transmembrane protein 85	IPI00009320	-0.272	0.28831	3	4	3	3
1588	F-actin-capping protein subunit alpha-1	IPI00005969	-0.279	0.28212	26	16	20	20
1589	Ribose-phosphate pyrophosphokinase 1	IPI00219616	-0.279	0.28212	21	21	18	22
1590	Isoform Mitochondrial of Peroxiredoxin-5, mitochondrial	IPI00024915	-0.284	0.28104	21	19	18	20
1591	Beta-actin-like protein 2	IPI00003269	-0.284	0.28000	65	56	64	54
1592	Aldehyde dehydrogenase, mitochondrial	IPI00006663	-0.287	0.27986	19	20	18	19
1593	Adenine phosphoribosyltransferase	IPI00218693	-0.287	0.27986	21	18	16	21
1594	Heme oxygenase 2	IPI00026824	-0.289	0.27981	2	4	4	1
1595	Negative elongation factor B	IPI00103483	-0.289	0.27981	4	2	3	2
1596	Isoform 5 of Sigma non-opioid intracellular receptor 1	IPI00167206	-0.289	0.27981	3	3	2	3
1597	Isoform 2 of N-alpha-acetyltransferase 15, NatA auxiliary subunit	IPI00032158	-0.289	0.27981	2	4	4	1
1598	Isoform 1 of Lysine-specific demethylase 3B	IPI00298935	-0.289	0.27981	2	4	1	4
1599	Peroxisomal membrane protein 2	IPI00221002	-0.289	0.27981	3	3	3	2
1600	AP-3 complex subunit sigma-1	IPI00014624	-0.289	0.27981	4	2	3	2
1601	Telomeric repeat-binding factor 2-interacting protein 1	IPI00008961	-0.289	0.27981	3	3	2	3
1602	Vacuolar protein sorting-associated protein VTA1 homolog	IPI00017160	-0.289	0.27981	4	2	3	2
1603	39S ribosomal protein L37, mitochondrial	IPI00162330	-0.289	0.27981	3	3	3	2
1604	Nuclear pore complex protein Nup50	IPI00026940	-0.289	0.27981	3	3	2	3
1605	nardilysin isoform a	IPI00243221	-0.289	0.27981	5	1	2	3
1606	Isoform 1 of Terminal uridylyltransferase 4	IPI00289861	-0.289	0.27981	3	3	3	2
1607	NADH dehydrogenase [ubiquinone] iron-sulfur protein 7, mitochondrial	IPI00307749	-0.289	0.27981	3	3	3	2

No.	Description	Accession number	STN[1]	p-Value[1]	Con_A[2]	Con_B[2]	CUR_A[2]	CUR_B[2]
1608	Acidic leucine-rich nuclear phosphoprotein 32 family member C	IPI00018262	-0.289	0.27981	3	3	2	3
1609	Epithelial cell adhesion molecule	IPI00296215	-0.289	0.27981	3	3	3	2
1610	39S ribosomal protein L11, mitochondrial	IPI00007001	-0.289	0.27981	2	4	3	2
1611	UPF0600 protein C5orf51	IPI00374272	-0.289	0.27981	4	2	2	3
1612	Isoform 1 of Casein kinase I isoform alpha	IPI00183400	-0.289	0.27981	4	2	3	2
1613	NudC domain-containing protein 2	IPI00103142	-0.289	0.27981	3	3	3	2
1614	Ubiquilin-4	IPI00024502	-0.289	0.27981	3	3	3	2
1615	Peroxiredoxin-4	IPI00011937	-0.290	0.27017	20	18	20	16
1616	Tubulin beta-2A chain	IPI00013475	-0.292	0.26880	18	19	19	16
1617	Peptidyl-prolyl cis-trans isomerase FKBP3	IPI00024157	-0.295	0.26875	19	17	15	19
1618	60S ribosomal protein L4	IPI00003918	-0.299	0.26762	18	17	17	16
1619	40S ribosomal protein S13	IPI00221089	-0.302	0.26748	17	17	16	16
1620	Translin-associated protein X	IPI00293350	-0.309	0.26630	16	16	16	14
1621	Eukaryotic translation initiation factor 6	IPI00010105	-0.309	0.26630	16	16	14	16
1622	Matrin-3	IPI00017297	-0.309	0.26474	48	48	49	44
1623	Tubulin, beta	IPI00645452	-0.310	0.26460	51	44	41	51
1624	Coatomer subunit gamma-2	IPI00002557	-0.311	0.26460	2	3	3	1
1625	Isoform 2 of 1,2-dihydroxy-3-keto-5-methylthiopentene dioxygenase	IPI00470791	-0.311	0.26460	2	3	1	3
1626	Hexokinase-2	IPI00102864	-0.311	0.26460	2	3	3	1
1627	39S ribosomal protein L38, mitochondrial	IPI00783656	-0.311	0.26460	4	1	2	2
1628	DnaJ homolog subfamily B member 11	IPI00008454	-0.311	0.26460	3	2	2	2
1629	Carboxypeptidase D	IPI00027078	-0.311	0.26460	3	2	2	2
1630	Isoform 1 of Replication factor C subunit 1	IPI00375358	-0.311	0.26460	3	2	2	2
1631	Coiled-coil domain-containing protein 124	IPI00060627	-0.311	0.26460	4	1	2	2
1632	WW domain-binding protein 11	IPI00170786	-0.311	0.26460	2	3	3	1
1633	Isoform SMN of Survival motor neuron protein	IPI00003394	-0.311	0.26460	3	2	2	2
1634	WD40 repeat-containing protein SMU1	IPI00305833	-0.311	0.26460	3	2	3	1
1635	Isoform 2 of Calumenin	IPI00045396	-0.311	0.26460	3	2	2	2
1636	M-phase phosphoprotein 6	IPI00016074	-0.311	0.26460	3	2	2	2
1637	Isoform 1 of Transcriptional repressor p66-alpha	IPI00410330	-0.311	0.26460	3	2	1	3
1638	C-Myc-binding protein	IPI00871174	-0.311	0.26460	1	4	2	2
1639	Farnesyl pyrophosphate synthetase like-4 protein (Fragment)	IPI00382869	-0.311	0.26460	3	2	3	0
1640	Exosome complex exonuclease MTR3	IPI00073602	-0.311	0.26460	2	3	2	2
1641	Isoform 1 of Trafficking protein particle complex subunit 2	IPI00005119	-0.311	0.26460	3	2	3	1
1642	Hexokinase-3	IPI00005118	-0.311	0.26460	2	3	2	2
1643	Isoform 1 of UPF0557 protein C10orf119	IPI00478758	-0.311	0.26460	3	2	2	2
1644	erlin-1	IPI00007940	-0.311	0.26460	2	3	2	2
1645	Uncharacterized protein C7orf30	IPI00061492	-0.311	0.26460	2	3	2	2
1646	Delta-1-pyrroline-5-carboxylate dehydrogenase, mitochondrial	IPI00217871	-0.311	0.26460	2	3	2	2
1647	37 kDa protein	IPI00032799	-0.311	0.26460	3	2	2	2
1648	Synaptojanin-2-binding protein	IPI00299193	-0.311	0.26460	2	3	2	2
1649	Acyl-coenzyme A thioesterase 13	IPI00020530	-0.311	0.26460	2	3	1	3
1650	Isoform Long of Delta-1-pyrroline-5-carboxylate synthase	IPI00008982	-0.313	0.25779	48	45	48	42
1651	splicing factor 3B subunit 2	IPI00221106	-0.316	0.25761	14	16	15	13
1652	C-1-tetrahydrofolate synthase, cytoplasmic	IPI00218342	-0.317	0.25619	47	43	46	41
1653	Protein disulfide-isomerase	IPI00010796	-0.319	0.25609	46	42	42	43
1654	Ubiquitin carboxyl-terminal hydrolase 24	IPI00902614	-0.325	0.25581	11	17	12	14
1655	Transaldolase	IPI00744692	-0.331	0.25326	39	41	40	37
1656	29 kDa protein	IPI00453476	-0.334	0.25307	14	12	12	12
1657	Aminoacyl tRNA synthase complex-interacting multifunctional protein 2	IPI00011916	-0.339	0.25109	12	13	12	11
1658	Small nuclear ribonucleoprotein E	IPI00029266	-0.339	0.25109	14	11	10	13
1659	Isoform C1 of Heterogeneous nuclear ribonucleoproteins C1/C2	IPI00216592	-0.339	0.25109	88	76	77	83
1660	NADH dehydrogenase [ubiquinone] 1 alpha subcomplex subunit 4	IPI00011770	-0.341	0.25090	2	2	2	1
1661	Isoform 1 of Large proline-rich protein BAT2	IPI00010700	-0.341	0.25090	3	1	0	2
1662	Isovaleryl-CoA dehydrogenase, mitochondrial	IPI00645805	-0.341	0.25090	3	1	2	1
1663	Isoform 1 of Glomulin	IPI00074604	-0.341	0.25090	2	2	2	1
1664	28S ribosomal protein S34, mitochondrial	IPI00169413	-0.341	0.25090	2	2	2	1
1665	Isoform 1 of Protein transport protein Sec24A	IPI00873472	-0.341	0.25090	2	2	2	1
1666	Isoform 3 of HEAT repeat-containing protein 5B	IPI00333696	-0.341	0.25090	3	1	0	2
1667	Lysosomal Pro-X carboxypeptidase	IPI00001593	-0.341	0.25090	3	1	2	1
1668	UPF0587 protein C1orf123	IPI00016605	-0.341	0.25090	2	2	2	1
1669	Isoform 2 of Ubiquitin carboxyl-terminal hydrolase 47	IPI00165528	-0.341	0.25090	2	2	1	2
1670	Isoform 6 of GTPase-activating protein and VPS9 domain-containing protein 1	IPI00292753	-0.341	0.25090	2	2	1	2
1671	GrpE protein homolog 1, mitochondrial	IPI00029557	-0.341	0.25090	2	2	2	1
1672	Ubiquitin carboxyl-terminal hydrolase 10	IPI00291946	-0.341	0.25090	0	3	0	2
1673	Isoform SRP55-1 of Splicing factor, arginine/serine-rich 6	IPI00012345	-0.341	0.25090	1	3	2	1
1674	Isoform 1 of Leucine-rich repeat-containing protein 16A	IPI00014843	-0.341	0.25090	2	2	2	0
1675	Keratin, type I cytoskeletal 14	IPI00384444	-0.341	0.25090	2	2	1	2
1676	Translocation protein SEC63 homolog	IPI00218922	-0.341	0.25090	3	1	0	2
1677	cDNA FLJ60939, highly similar to NAD-dependent deacetylase sirtuin-3, mitochondrial	IPI00183171	-0.341	0.25090	2	2	0	2
1678	Activator of basal transcription 1	IPI00002938	-0.341	0.25090	3	1	1	2
1679	Beta-centractin	IPI00029469	-0.341	0.25090	2	2	1	2
1680	SH3 domain-binding glutamic acid-rich-like protein 2	IPI00412272	-0.341	0.25090	2	2	1	2
1681	cDNA FLJ55484, highly similar to ATP-dependent RNA helicase DDX39	IPI00644431	-0.341	0.25090	2	2	2	1
1682	RNA-binding protein 12	IPI00550308	-0.341	0.25090	1	3	0	2
1683	Translocating chain-associated membrane protein 1	IPI00219111	-0.341	0.25090	3	1	2	1
1684	PDZ domain-containing protein GIPC1	IPI00024705	-0.341	0.25090	2	2	2	1
1685	Cytosolic Fe-S cluster assembly factor NUBP2	IPI00644674	-0.341	0.25090	0	3	0	2
1686	Isoform 2 of Ubiquitin-associated protein 2-like	IPI00029019	-0.341	0.25090	2	2	1	2
1687	Ribonuclease inhibitor	IPI00550069	-0.341	0.25090	2	2	1	2
1688	Isoform 1 of RNA polymerase-associated protein LEO1	IPI00103090	-0.341	0.25090	2	2	2	0
1689	Isoform 1 of WD repeat-containing protein 74	IPI00018192	-0.341	0.25090	2	2	2	1
1690	Dipeptidase 1	IPI00059476	-0.341	0.25090	2	2	2	1
1691	Methylmalonate-semialdehyde dehydrogenase [acylating], mitochondria	IPI00024990	-0.341	0.25090	2	2	2	1
1692	UDP-galactose-4-epimerase	IPI00030229	-0.341	0.25090	2	2	2	1
1693	Glutathione S-transferase theta-1	IPI00741097	-0.341	0.25090	2	2	1	2
1694	Nuclease-sensitive element-binding protein 1	IPI00031812	-0.344	0.23762	10	14	9	13
1695	Isoform 1 of Leukotriene A-4 hydrolase	IPI00219077	-0.349	0.23413	35	34	34	32
1696	Isoform Epsilon of Apoptosis regulator BAX	IPI00071059	-0.350	0.23408	12	11	10	11
1697	GTP-binding nuclear protein Ran	IPI00643041	-0.351	0.23399	34	34	32	33
1698	cDNA FLJ78679, highly similar to Homo sapiens DEAD (Asp-Glu-Ala-Asp) box polypeptide 46 (DDX46)	IPI00329791	-0.355	0.23328	11	11	11	9
1699	Methylenetetrahydrofolate dehydrogenase (NADP+ dependent) 1-like	IPI00291646	-0.355	0.23328	11	11	12	8
1700	Isoform 5 of Interleukin enhancer-binding factor 3	IPI00219330	-0.359	0.22921	33	31	33	28
1701	Galectin-3	IPI00465431	-0.362	0.22879	12	9	11	8

No.	Description	Accession number	STN[1]	p-Value[1]	Con_A[2]	Con_B[2]	CUR_A[2]	CUR_B[2]
1702	Golgi phosphoprotein 3	IPI00005490	-0.362	0.22879	11	10	11	8
1703	ATP synthase subunit O, mitochondrial	IPI00007611	-0.362	0.22879	12	9	10	9
1704	26S proteasome non-ATPase regulatory subunit 3	IPI00011603	-0.373	0.22411	32	26	27	28
1705	Ubiquitin-conjugating enzyme E2 L3	IPI00021347	-0.376	0.22369	9	10	10	7
1706	Isoform 1 of UTP--glucose-1-phosphate uridylyltransferase	IPI00329331	-0.384	0.22293	8	10	8	8
1707	39S ribosomal protein L46, mitochondrial	IPI00023161	-0.384	0.22293	9	9	8	8
1708	Diphosphoinositol polyphosphate phosphohydrolase 1	IPI00009148	-0.384	0.22293	10	8	8	8
1709	Isoform 1 of Platelet-activating factor acetylhydrolase IB subunit alpha	IPI00218728	-0.384	0.22293	10	8	8	8
1710	cDNA FLJ55988, highly similar to RNA-binding protein Luc7-like 2	IPI00006932	-0.386	0.21830	1	2	0	1
1711	N-acetylgalactosaminyltransferase 7	IPI00328391	-0.386	0.21830	1	2	1	1
1712	Brefeldin A-inhibited guanine nucleotide-exchange protein 2	IPI00002186	-0.386	0.21830	2	1	0	1
1713	Calmodulin-regulated spectrin-associated protein 3	IPI00176702	-0.386	0.21830	2	0	0	1
1714	Acetyl-coenzyme A transporter 1	IPI00006205	-0.386	0.21830	1	2	1	1
1715	Isoform 2 of Triple functional domain protein	IPI00479523	-0.386	0.21830	2	1	1	1
1716	Isoform 1 of Protein phosphatase methylesterase 1	IPI00007694	-0.386	0.21830	1	2	1	1
1717	Isoform 2 of Serine-protein kinase ATM	IPI00289986	-0.386	0.21830	1	2	1	0
1718	Ubiquitin-conjugating enzyme E2 T	IPI00023087	-0.386	0.21830	1	2	0	1
1719	Dedicator of cytokinesis protein 1	IPI00015286	-0.386	0.21830	2	1	0	1
1720	Bleomycin hydrolase	IPI00219575	-0.386	0.21830	2	1	1	1
1721	Lipoamide acyltransferase component of branched-chain alpha-keto acid dehydrogenase complex	IPI00003944	-0.386	0.21830	1	2	1	1
1722	Isoform Long of Beta-glucuronidase	IPI00027745	-0.386	0.21830	0	2	1	1
1723	DmX-like protein 1	IPI00294728	-0.386	0.21830	0	2	0	1
1724	Isoform 1 of Ubiquitin conjugation factor E4 B	IPI00005715	-0.386	0.21830	1	2	1	0
1725	Isoform 1 of Serine/threonine-protein kinase 4	IPI00011488	-0.386	0.21830	1	2	1	1
1726	Isoform 1 of Pre-mRNA-splicing factor RBM22	IPI00019046	-0.386	0.21830	2	0	1	1
1727	cDNA FLJ12662 fis, clone NT2RM4002205, moderately similar to ELONGATION FACTOR G	IPI00026321	-0.386	0.21830	0	2	1	1
1728	Isoform 1 of Elongator complex protein 3	IPI00165477	-0.386	0.21830	2	1	0	0
1729	Isoform 1 of ATP-dependent metalloprotease YME1L1	IPI00045946	-0.386	0.21830	1	2	1	1
1730	Vacuolar protein sorting-associated protein 45	IPI00090327	-0.386	0.21830	0	2	1	1
1731	Beta-lactamase-like protein 2	IPI00006952	-0.386	0.21830	2	1	1	0
1732	Isoform 1 of Hematological and neurological expressed 1 protein	IPI00007764	-0.386	0.21830	0	2	0	1
1733	Isoform 1 of Carnitine O-acetyltransferase	IPI00016457	-0.386	0.21830	2	0	0	1
1734	Isoform 1 of Poly [ADP-ribose] polymerase 2	IPI00026497	-0.386	0.21830	2	1	1	1
1735	B-cell lymphoma/leukemia 10	IPI00022477	-0.386	0.21830	2	0	1	1
1736	Isoform XLas-1 of Guanine nucleotide-binding protein G(s) subunit alpha isoforms XLas	IPI00095891	-0.386	0.21830	2	0	1	1
1737	Sulfhydryl oxidase 2	IPI00376394	-0.386	0.21830	2	1	1	1
1738	Guanine nucleotide-binding protein-like 3-like protein	IPI00005132	-0.386	0.21830	0	2	1	0
1739	Isoform A of Uncharacterized protein C21orf70	IPI00027898	-0.386	0.21830	1	2	1	1
1740	Isoform 1 of Axin interactor, dorsalization-associated protein	IPI00303602	-0.386	0.21830	1	2	1	1
1741	Chromosome-associated kinesin KIF4B	IPI00175193	-0.386	0.21830	1	2	0	0
1742	Proteasome assembly chaperone 4	IPI00895892	-0.386	0.21830	2	1	1	1
1743	Interferon-related developmental regulator 2, isoform CRA_b	IPI00395667	-0.386	0.21830	0	2	1	1
1744	cDNA FLJ60317, highly similar to Aminoacylase-1	IPI00009268	-0.386	0.21830	2	1	1	0
1745	Isoform 4 of Mitochondrial fission factor	IPI00024627	-0.386	0.21830	0	2	1	1
1746	Isoform 2 of Arf-GAP with Rho-GAP domain, ANK repeat and PH domain-containing protein 1	IPI00220421	-0.386	0.21830	0	2	1	1
1747	Probable ATP-dependent RNA helicase DDX28	IPI00020050	-0.386	0.21830	2	1	1	1
1748	Nuclear receptor coactivator 5	IPI00288941	-0.386	0.21830	2	1	0	1
1749	Casein kinase I isoform alpha-like	IPI00167096	-0.386	0.21830	2	1	0	1
1750	COMM domain-containing protein 9	IPI00305212	-0.386	0.21830	1	2	0	0
1751	Probable ATP-dependent RNA helicase DDX41	IPI00007208	-0.386	0.21830	2	0	1	1
1752	Importin subunit alpha-3	IPI00299033	-0.386	0.21830	2	0	1	1
1753	KDEL motif-containing protein 1	IPI00005270	-0.386	0.21830	0	2	1	1
1754	Isoform 1 of NADH dehydrogenase [ubiquinone] 1 alpha subcomplex subunit 11	IPI00329301	-0.386	0.21830	1	2	1	1
1755	Isoform 3 of Protein MON2 homolog	IPI00183974	-0.386	0.21830	2	1	1	0
1756	Isoform 1 of ADP-ribosylation factor-like protein 2-binding protein	IPI00015866	-0.386	0.21830	2	1	1	1
1757	Isoform 1 of General transcription factor 3C polypeptide 3	IPI00015806	-0.386	0.21830	1	2	0	0
1758	Vacuolar protein sorting-associated protein 33B	IPI00032905	-0.386	0.21830	2	1	1	1
1759	dehydrogenase/reductase SDR family member 4	IPI00106913	-0.386	0.21830	1	2	0	1
1760	Isoform 1 of Mesoderm-specific transcript homolog protein	IPI00298947	-0.386	0.21830	1	2	0	0
1761	dCTP pyrophosphatase 1	IPI00012197	-0.386	0.21830	1	2	1	1
1762	Calmodulin	IPI00075248	-0.386	0.21830	2	1	1	1
1763	Isoform 1 of Neuroguidin	IPI00000162	-0.386	0.21830	1	2	1	0
1764	F-box only protein 7	IPI00294567	-0.386	0.21830	2	0	0	0
1765	cDNA FLJ53160, highly similar to Zyxin	IPI00871311	-0.386	0.21830	1	2	1	1
1766	Bis(5'-nucleosyl)-tetraphosphatase [asymmetrical]	IPI00221231	-0.386	0.21830	1	2	1	1
1767	Polymerase delta interacting protein 46	IPI00429180	-0.386	0.21830	2	1	0	1
1768	Isoform 1 of Leucine-rich repeat protein SHOC-2	IPI00298928	-0.386	0.21830	2	0	0	1
1769	Isoform 1 of Trafficking protein particle complex subunit 2-like protein	IPI00007819	-0.386	0.21830	2	1	1	1
1770	cDNA FLJ56443, highly similar to Putative ATP-dependent RNA helicase DHX33	IPI00302860	-0.386	0.21830	2	1	0	1
1771	Isoform 1 of Serine/threonine-protein phosphatase 4 regulatory subunit 3A	IPI00217013	-0.386	0.21830	2	0	0	1
1772	Isoform 1 of Lymphoid-specific helicase	IPI00010590	-0.386	0.21830	0	2	0	0
1773	Arginase-2, mitochondrial	IPI00020332	-0.386	0.21830	2	1	1	1
1774	MORF4 family-associated protein 1	IPI00020915	-0.386	0.21830	0	2	0	0
1775	Uncharacterized protein C4orf14	IPI00385928	-0.386	0.21830	2	1	1	0
1776	Isoform 1 of Transmembrane emp24 domain-containing protein 4	IPI00296259	-0.386	0.21830	0	2	1	1
1777	Endonuclease G, mitochondrial	IPI00290614	-0.386	0.21830	2	1	0	1
1778	Nuclear receptor-binding protein	IPI00604756	-0.386	0.21830	1	2	0	0
1779	USP48 protein	IPI00328815	-0.386	0.21830	1	2	0	0
1780	Isoform 2 of Uncharacterized protein C3orf21	IPI00165665	-0.386	0.21830	1	2	1	1
1781	Isoform 1 of Vacuolar-sorting protein SNF8	IPI00101524	-0.386	0.21830	2	1	1	1
1782	Biogenesis of lysosome-related organelles complex 1 subunit 1	IPI00020319	-0.386	0.21830	2	1	1	1
1783	Serine palmitoyltransferase 2	IPI00005751	-0.386	0.21830	0	2	0	1
1784	Ubiquitin-associated protein 2	IPI00171127	-0.386	0.21830	1	2	0	0
1785	Isoform 1 of Kinesin-like protein KIF15	IPI00024975	-0.386	0.21830	1	2	0	0
1786	Isoform 1 of Peptidyl-prolyl cis-trans isomerase-like 2	IPI00003824	-0.386	0.21830	0	2	0	0
1787	Translation initiation factor eIF-2B subunit beta	IPI00028083	-0.386	0.21830	2	0	1	1
1788	Isoform 6 of Ribosome-recycling factor, mitochondrial	IPI00030596	-0.386	0.21830	0	2	1	1
1789	Isoform Long of Glutaryl-CoA dehydrogenase, mitochondrial	IPI00024317	-0.386	0.21830	2	0	0	0
1790	Protein farnesyltransferase subunit beta	IPI00026817	-0.386	0.21830	2	0	0	0
1791	Isoform 2 of AP-3 complex subunit delta-1	IPI00289608	-0.392	0.20796	9	8	7	8
1792	28S ribosomal protein S29, mitochondrial	IPI00018120	-0.392	0.20796	9	8	7	8
1793	Isoform 1 of Putative ATP-dependent RNA helicase DHX30	IPI00411733	-0.402	0.20640	7	9	8	6
1794	Isoform 1 of Replication protein A 32 kDa subunit	IPI00013939	-0.402	0.20640	9	7	7	7
1795	Isoform 1 of Phosphatidate cytidylyltransferase 2	IPI00032150	-0.402	0.20640	7	9	7	7
1796	Pyrroline-5-carboxylate reductase 2	IPI00470610	-0.402	0.20640	8	8	8	6

No.	Description	Accession number	STN[1]	p-Value[1]	Con_A[2]	Con_B[2]	CUR_A[2]	CUR_B[2]
1797	Isoform 1 of Pyridoxal kinase	IPI00013004	-0.403	0.20238	27	20	20	24
1798	Heterogeneous nuclear ribonucleoprotein A0	IPI00011913	-0.410	0.20163	23	22	22	20
1799	Wolframin	IPI00008711	-0.412	0.20148	7	8	6	7
1800	28S ribosomal protein S26, mitochondrial	IPI00006606	-0.412	0.20148	7	8	8	5
1801	Aldehyde dehydrogenase X, mitochondrial	IPI00103467	-0.412	0.20148	7	8	7	6
1802	RNA-binding motif protein, X-linked-like-2	IPI00004450	-0.412	0.20148	8	7	6	7
1803	Heterogeneous nuclear ribonucleoprotein U-like protein 2	IPI00456887	-0.417	0.20096	21	22	20	20
1804	Eukaryotic translation initiation factor 3 subunit A	IPI00029012	-0.418	0.20087	46	47	39	50
1805	Isoform 1 of Ras-related protein Rab-1A	IPI00005719	-0.421	0.20044	44	47	42	45
1806	60S ribosomal protein L18a	IPI00026202	-0.423	0.20044	6	8	5	7
1807	cDNA FLJ59571, highly similar to Eukaryotic translation initiation factor 4gamma 2	IPI00015952	-0.423	0.20044	5	9	7	5
1808	Isoform A of Ras GTPase-activating protein-binding protein 2	IPI00009057	-0.423	0.20044	8	6	6	6
1809	Mitochondrial import receptor subunit TOM34	IPI00009946	-0.423	0.20044	6	8	7	5
1810	Malectin	IPI00029046	-0.423	0.20044	6	8	5	7
1811	Isoform 3 of LIM domain only protein 7	IPI00291802	-0.423	0.20044	4	10	7	5
1812	Histone H2B type 2-E	IPI00003935	-0.426	0.19355	124	143	124	137
1813	Isoform 5 of Dynamin-1-like protein	IPI00037283	-0.432	0.19284	17	22	19	17
1814	Isoform 2 of Nucleoporin NUP188 homolog	IPI00385001	-0.436	0.19260	4	9	6	5
1815	Isoform 2 of Mediator of DNA damage checkpoint protein 1	IPI00470805	-0.436	0.19260	6	7	6	5
1816	Cytosolic purine 5'-nucleotidase	IPI00029054	-0.436	0.19260	8	5	6	5
1817	Aladin	IPI00024143	-0.436	0.19260	6	7	5	6
1818	importin subunit alpha-6	IPI00413214	-0.436	0.19260	9	4	6	5
1819	DEAD (Asp-Glu-Ala-Asp) box polypeptide 39, isoform CRA_c	IPI00166874	-0.436	0.19260	7	6	6	5
1820	39S ribosomal protein L49, mitochondrial	IPI00013195	-0.436	0.19260	7	6	5	6
1821	Periodic tryptophan protein 2 homolog	IPI00300078	-0.436	0.19260	17	21	18	17
1822	Proteasome subunit alpha type-4	IPI00299155	-0.445	0.19142	20	16	18	15
1823	ubiquitin and ribosomal protein S27a precursor	IPI00179330	-0.449	0.19081	66	75	68	68
1824	87 kDa protein	IPI00220365	-0.450	0.19081	7	5	5	5
1825	24 kDa protein	IPI00397611	-0.450	0.19081	5	7	5	5
1826	Guanine nucleotide-binding protein G(I)/G(S)/G(T) subunit beta-2	IPI00003348	-0.450	0.19081	6	6	5	5
1827	SAP domain-containing ribonucleoprotein	IPI00014938	-0.450	0.19081	7	5	4	6
1828	Splicing factor, arginine/serine-rich 3	IPI00010204	-0.450	0.19081	5	7	5	5
1829	Omega-amidase NIT2	IPI00549467	-0.450	0.19081	6	6	5	5
1830	Ras-related protein Rab-21	IPI00007755	-0.450	0.19081	6	6	5	5
1831	Isoform 1 of Prolyl 4-hydroxylase subunit alpha-1	IPI00009923	-0.450	0.19081	6	6	6	4
1832	Isoform 1 of 3,2-trans-enoyl-CoA isomerase, mitochondrial	IPI00300567	-0.450	0.19081	6	6	5	5
1833	SUMO-conjugating enzyme UBC9	IPI00032957	-0.450	0.19081	7	5	5	5
1834	Lamin-B2	IPI00009771	-0.450	0.18193	20	15	17	15
1835	Actin, aortic smooth muscle	IPI00008603	-0.461	0.18065	64	67	65	61
1836	DEAD (Asp-Glu-Ala-Asp) box polypeptide 39 transcript variant	IPI00062206	-0.465	0.18046	7	4	4	5
1837	Isoform 1 of Spectrin beta chain, brain 2	IPI00012645	-0.465	0.18046	8	3	5	4
1838	40S ribosomal protein S20	IPI00012493	-0.465	0.18046	6	5	3	6
1839	Isoform 1 of CUGBP Elav-like family member 1	IPI00034015	-0.465	0.18046	6	5	5	4
1840	Centromere/kinetochore protein zw10 homolog	IPI00011631	-0.465	0.18046	5	6	5	4
1841	LDLR chaperone MESD	IPI00399089	-0.465	0.18046	5	6	5	4
1842	Isoform 2 of Basigin	IPI00019906	-0.465	0.18046	6	5	5	4
1843	Reticulocalbin-1	IPI00015842	-0.465	0.18046	6	5	5	4
1844	Cation-independent mannose-6-phosphate receptor	IPI00289819	-0.465	0.18046	5	6	4	5
1845	Programmed cell death protein 6	IPI00025277	-0.465	0.18046	6	5	4	5
1846	Vacuolar protein sorting-associated protein 26A	IPI00411426	-0.465	0.18046	2	9	5	4
1847	CCR4-NOT transcription complex subunit 7	IPI00006552	-0.465	0.18046	6	5	4	5
1848	NADH dehydrogenase [ubiquinone] flavoprotein 2, mitochondrial	IPI00291328	-0.465	0.18046	6	5	4	5
1849	Isoform 2 of Transcription elongation factor SPT6	IPI00430770	-0.465	0.18046	6	5	4	5
1850	1-acyl-sn-glycerol-3-phosphate acyltransferase epsilon	IPI00028491	-0.465	0.18046	6	5	4	5
1851	Isoform 1 of Importin-4	IPI00156374	-0.477	0.17829	17	13	14	13
1852	Isoform Heart of ATP synthase subunit gamma, mitochondrial	IPI00395769	-0.484	0.17758	14	15	12	14
1853	transcriptional regulator ATRX isoform 2	IPI00220109	-0.484	0.17715	5	5	3	5
1854	Ubiquitin-conjugating enzyme E2 O	IPI00783378	-0.484	0.17715	4	6	3	5
1855	Ribosome production factor 2 homolog	IPI00396329	-0.484	0.17715	4	6	4	4
1856	Isoform 1 of DNA-directed RNA polymerases I and III subunit RPAC1	IPI00005179	-0.484	0.17715	5	5	4	4
1857	SNW domain-containing protein 1	IPI00013830	-0.484	0.17715	6	4	4	4
1858	Ras-related protein Rab-5B	IPI00017344	-0.484	0.17715	5	5	4	4
1859	HDCMD34P	IPI00001672	-0.484	0.17715	6	4	5	3
1860	Isoform 1 of Heterogeneous nuclear ribonucleoprotein H3	IPI00013877	-0.492	0.16445	31	29	30	26
1861	Isoform 1 of KH domain-containing, RNA-binding, signal transduction-associated protein 1	IPI00008575	-0.497	0.16445	13	14	11	13
1862	Isoform 1 of Protein KIAA1967	IPI00182757	-0.504	0.16341	14	12	12	11
1863	Rho-associated protein kinase 2	IPI00307155	-0.505	0.16341	4	5	3	4
1864	BAG family molecular chaperone regulator 2	IPI00000643	-0.505	0.16341	5	4	3	4
1865	Derlin-1	IPI00013271	-0.505	0.16341	4	5	4	3
1866	Eukaryotic translation initiation factor 2A	IPI00012462	-0.505	0.16341	4	5	3	4
1867	Cell differentiation protein RCD1 homolog	IPI00023101	-0.505	0.16341	3	6	3	4
1868	Peptidyl-prolyl cis-trans isomerase FKBP5	IPI00218775	-0.505	0.16341	3	6	4	3
1869	U3 small nucleolar RNA-associated protein 18 homolog	IPI00000733	-0.505	0.16341	4	5	4	3
1870	Tyrosyl-tRNA synthetase, mitochondrial	IPI00165092	-0.505	0.16341	5	4	4	3
1871	TDP43	IPI00025815	-0.505	0.16341	5	4	4	3
1872	Isoform 3 of Tyrosine-protein phosphatase non-receptor type 6	IPI00183046	-0.505	0.16341	4	5	4	3
1873	Isoform 1 of UBX domain-containing protein 1	IPI00027378	-0.505	0.16341	5	4	3	4
1874	Isoform 1 of Annexin A7	IPI00002460	-0.505	0.16341	5	4	4	3
1875	Uroporphyrinogen decarboxylase	IPI00301489	-0.505	0.16341	4	5	5	2
1876	Multifunctional protein ADE2	IPI00217223	-0.508	0.16303	28	27	28	23
1877	Adenosylhomocysteinase	IPI00012007	-0.519	0.16204	23	29	24	24
1878	Serine/threonine-protein phosphatase 2A catalytic subunit alpha isoform	IPI00008380	-0.529	0.16043	11	12	11	9
1879	cDNA FLJ14239 fis, clone NT2RP5003512, highly similar to Exportin-5	IPI00549861	-0.530	0.16034	5	3	4	2
1880	Protein SCO1 homolog, mitochondrial	IPI00027233	-0.530	0.16034	4	4	2	4
1881	39S ribosomal protein L19, mitochondrial	IPI00027096	-0.530	0.16034	5	3	2	4
1882	Unhealthy ribosome biogenesis protein 2 homolog	IPI00028980	-0.530	0.16034	4	4	4	2
1883	Density-regulated protein	IPI00306280	-0.530	0.16034	3	5	2	4
1884	Cytochrome c oxidase assembly protein COX11, mitochondrial	IPI00295394	-0.530	0.16034	3	5	3	3
1885	Isoform 1 of Putative helicase MOV-10	IPI00444452	-0.530	0.16034	4	4	3	3
1886	Synaptic vesicle membrane protein VAT-1 homolog	IPI00156689	-0.530	0.16034	4	4	3	3
1887	Putative uncharacterized protein DKFZp781K1356	IPI00412545	-0.530	0.16034	5	3	2	4
1888	Isoform 1 of UPF0598 protein C8orf82	IPI00166638	-0.530	0.16034	5	3	2	4
1889	Isoform 1 of Metaxin-1	IPI00013678	-0.530	0.16034	3	5	3	3
1890	WD repeat-containing protein 5	IPI00005492	-0.530	0.16034	4	4	4	2
1891	Isoform 2 of Gamma-glutamylcyclotransferase	IPI00020301	-0.530	0.16034	5	3	2	4

No.	Description	Accession number	STN[1]	p-Value[1]	Con_A[2]	Con_B[2]	CUR_A[2]	CUR_B[2]
1892	Isoform 2 of Serine/threonine-protein kinase PAK 1	IPI00289746	-0.530	0.16034	4	4	3	3
1893	Mitochondrial import inner membrane translocase subunit Tim17-B	IPI00219833	-0.530	0.16034	4	4	3	3
1894	39S ribosomal protein L41, mitochondrial	IPI00217553	-0.530	0.16034	4	4	3	3
1895	Reticulocalbin-2	IPI00029628	-0.530	0.16034	5	3	3	3
1896	Platelet-activating factor acetylhydrolase IB subunit beta	IPI00026546	-0.538	0.14621	12	10	9	10
1897	Isoform 1 of Pyruvate dehydrogenase E1 component subunit beta, mitochondrial	IPI00003925	-0.538	0.14621	11	11	9	10
1898	cDNA FLJ56414, highly similar to Homo sapiens proline-, glutamic acid-, leucine-rich protein 1	IPI00006702	-0.548	0.14494	11	10	11	7
1899	Prostaglandin E synthase 3	IPI00015029	-0.548	0.14494	12	9	11	7
1900	Isoform 1 of BH3-interacting domain death agonist	IPI00413587	-0.548	0.14494	11	10	10	8
1901	GMP synthase [glutamine-hydrolyzing]	IPI00029079	-0.553	0.14432	24	20	20	20
1902	Isoform Long of Trifunctional purine biosynthetic protein adenosine-3	IPI00025273	-0.558	0.14371	22	21	20	19
1903	Splicing factor, arginine/serine-rich 9	IPI00012340	-0.559	0.14371	11	9	10	7
1904	Isoform 1 of 5'(3')-deoxyribonucleotidase, cytosolic type	IPI00005573	-0.559	0.14371	11	9	9	8
1905	Isoform 1 of Nucleoside diphosphate kinase A	IPI00012048	-0.559	0.14286	39	39	36	37
1906	Isoform Gamma-1 of Serine/threonine-protein phosphatase PP1-gamma catalytic subunit	IPI00005705	-0.559	0.14286	37	41	34	39
1907	Isoform 1 of Cytochrome c oxidase assembly protein COX15 homolog	IPI00419869	-0.561	0.14286	4	3	2	3
1908	Isoform 2 of DnaJ homolog subfamily A member 3, mitochondrial	IPI00179187	-0.561	0.14286	4	3	3	2
1909	DnaJ homolog subfamily C member 3	IPI00006713	-0.561	0.14286	2	5	2	3
1910	Uncharacterized protein KIAA0406	IPI00011702	-0.561	0.14286	3	4	3	2
1911	Isoform 3 of Protein transport protein Sec31A	IPI00305152	-0.561	0.14286	4	3	2	3
1912	Isoform 1 of U4/U6 small nuclear ribonucleoprotein Prp4	IPI00150269	-0.561	0.14286	4	3	2	3
1913	Exosome complex exonuclease RRP43	IPI00552920	-0.561	0.14286	3	4	3	2
1914	Scavenger mRNA-decapping enzyme DcpS	IPI00335385	-0.561	0.14286	4	3	4	1
1915	Isoform 1 of Crooked neck-like protein 1	IPI00177437	-0.561	0.14286	5	2	3	2
1916	Isoform 2 of Ubiquinol-cytochrome c reductase complex chaperone CBP3 homolog	IPI00219889	-0.561	0.14286	3	4	2	3
1917	Isoform 1 of Interferon regulatory factor 2-binding protein 2	IPI00376199	-0.561	0.14286	3	4	2	3
1918	Lupus La protein	IPI00009032	-0.562	0.14286	39	38	29	43
1919	Peroxiredoxin-2	IPI00027350	-0.568	0.14191	23	18	21	16
1920	Isoform 1 of Serine/threonine-protein phosphatase 2A 65 kDa regulatory subunit A beta isoform	IPI00294178	-0.570	0.14191	11	8	9	7
1921	Vacuolar protein-sorting-associated protein 25	IPI00031655	-0.570	0.14191	10	9	9	7
1922	Ornithine aminotransferase, mitochondrial	IPI00022334	-0.570	0.14191	9	10	8	8
1923	Eukaryotic translation initiation factor 3 subunit I	IPI00012795	-0.570	0.14191	9	10	8	8
1924	Barrier-to-autointegration factor	IPI00026087	-0.570	0.14191	10	9	8	8
1925	Putative nascent polypeptide-associated complex subunit alpha-like protein	IPI00012479	-0.582	0.13964	9	9	8	7
1926	Vesicle-trafficking protein SEC22b	IPI00006865	-0.582	0.13964	10	8	9	6
1927	PRA1 family protein 3	IPI00007426	-0.582	0.13964	9	9	8	7
1928	NADH dehydrogenase [ubiquinone] 1 alpha subcomplex subunit 9, mitochondrial	IPI00003968	-0.585	0.13837	18	20	17	17
1929	Transgelin-2	IPI00550363	-0.585	0.13837	20	18	18	16
1930	Isoform DPI of Desmoplakin	IPI00013933	-0.587	0.13766	81	90	82	82
1931	EH domain-containing protein 1	IPI00017184	-0.596	0.13733	10	7	7	7
1932	Metastasis-associated protein MTA2	IPI00171798	-0.596	0.13733	7	10	8	6
1933	Isoform A of Ras-related C3 botulinum toxin substrate 1	IPI00010271	-0.596	0.13733	9	8	7	7
1934	Ras-related C3 botulinum toxin substrate 2	IPI00010270	-0.596	0.13733	6	11	7	7
1935	DNA polymerase delta catalytic subunit	IPI00002894	-0.596	0.13733	10	7	8	6
1936	7-dehydrocholesterol reductase	IPI00294501	-0.597	0.13695	18	18	17	15
1937	60S ribosomal protein L3	IPI00550021	-0.600	0.13596	57	49	58	42
1938	Isoform 1 of Adipocyte plasma membrane-associated protein	IPI00031131	-0.600	0.13596	3	3	2	2
1939	Basic leucine zipper and W2 domain-containing protein 2	IPI00022305	-0.600	0.13596	3	3	3	1
1940	Isoform 1 of Cullin-4A	IPI00419273	-0.600	0.13596	3	3	3	1
1941	Protein LLP homolog	IPI00031615	-0.600	0.13596	3	3	2	2
1942	Putative high mobility group protein 1-like 10	IPI00018755	-0.600	0.13596	4	2	1	3
1943	cDNA, FLJ79450, highly similar to 3-ketoacyl-CoA thiolase, peroxisomal	IPI00011522	-0.600	0.13596	2	4	2	2
1944	Isoform 1 of Acyl-coenzyme A thioesterase 2, mitochondrial	IPI00220906	-0.600	0.13596	3	3	1	3
1945	programmed cell death 4 isoform 2	IPI00240675	-0.600	0.13596	3	3	1	3
1946	Eukaryotic translation initiation factor 1	IPI00015077	-0.600	0.13596	3	3	2	2
1947	Methylosome protein 50	IPI00012202	-0.600	0.13596	3	3	2	2
1948	39S ribosomal protein L14, mitochondrial	IPI00418290	-0.600	0.13596	3	3	2	2
1949	STE20/SPS1-related proline-alanine-rich protein kinase	IPI00004363	-0.600	0.13596	3	3	2	2
1950	Importin 5	IPI00639960	-0.600	0.13596	4	2	2	2
1951	COMM domain-containing protein 3	IPI00015773	-0.600	0.13596	3	3	1	3
1952	GDP-L-fucose synthase	IPI00014361	-0.600	0.13596	2	4	3	1
1953	Isoform 1 of U4/U6 small nuclear ribonucleoprotein Prp31	IPI00292000	-0.600	0.13596	2	4	1	3
1954	Isoform 1 of Phosphoribosyl pyrophosphate synthase-associated protein 1	IPI00291578	-0.600	0.13596	3	3	2	2
1955	Succinate dehydrogenase assembly factor 2, mitochondrial	IPI00016443	-0.600	0.13596	3	3	2	2
1956	Putative uncharacterized protein PYCR2	IPI00335061	-0.600	0.13596	3	3	3	1
1957	Peflin	IPI00018235	-0.600	0.13596	3	3	2	2
1958	Squalene synthase	IPI00020944	-0.600	0.13596	4	2	3	1
1959	Lamina-associated polypeptide 2, isoform alpha	IPI00216230	-0.610	0.11952	9	7	6	7
1960	Isoform 1 of 2-oxoglutarate and iron-dependent oxygenase domain-containing protein 1	IPI00170429	-0.610	0.11952	8	8	7	6
1961	Isoform 1 of Replication factor C subunit 2	IPI00017412	-0.610	0.11952	7	9	6	7
1962	Isoform 1 of RNA-binding protein with serine-rich domain 1	IPI00033561	-0.610	0.11952	9	7	6	7
1963	NCL protein	IPI00183526	-0.612	0.11730	112	107	103	108
1964	Rho GDP-dissociation inhibitor 1	IPI00003815	-0.613	0.11730	31	30	26	30
1965	Isoform 1 of 40S ribosomal protein S24	IPI00029750	-0.617	0.11706	29	31	28	27
1966	Pre-mRNA-processing factor 19	IPI00004968	-0.625	0.11673	16	16	15	13
1967	Ras-related protein Rab-10	IPI00016513	-0.625	0.11673	17	15	14	14
1968	Protein NipSnap homolog 2	IPI00016077	-0.625	0.11673	15	17	13	15
1969	60S ribosomal protein L27	IPI00219155	-0.626	0.11541	7	8	7	5
1970	NEDD8	IPI00020008	-0.626	0.11541	9	6	6	6
1971	Cytochrome c oxidase subunit 5A, mitochondrial	IPI00025086	-0.626	0.11541	7	8	5	7
1972	Mannose-P-dolichol utilization defect 1 protein	IPI00025292	-0.626	0.11541	6	9	7	5
1973	ADP-ribosylation factor-like protein 3	IPI00003327	-0.626	0.11541	9	6	6	6
1974	SH3 domain-binding glutamic acid-rich-like protein	IPI00025318	-0.626	0.11541	8	7	5	7
1975	Hepatoma-derived growth factor	IPI00020956	-0.641	0.11366	15	15	14	12
1976	60S ribosomal protein L13a	IPI00304612	-0.644	0.11253	5	9	5	6
1977	Palmitoyl-protein thioesterase 1	IPI00002412	-0.644	0.11253	7	7	6	5
1978	Peptidyl-prolyl cis-trans isomerase NIMA-interacting 1	IPI00013723	-0.644	0.11253	6	8	5	6
1979	Isoform 2 of Leucyl-cystinyl aminopeptidase	IPI00221240	-0.644	0.11253	7	7	6	5
1980	Isoform 1 of Actin-like protein 6A	IPI00003627	-0.644	0.11253	7	7	5	6
1981	Protein phosphatase 1 regulatory subunit 14B	IPI00398922	-0.644	0.11253	8	6	6	5
1982	Membrane-associated progesterone receptor component 1	IPI00220739	-0.644	0.11253	7	7	6	5
1983	Isoform 2 of Nucleophosmin	IPI00220740	-0.650	0.11154	97	89	89	89
1984	Nucleolar complex protein 4 homolog	IPI00031661	-0.653	0.11149	3	2	1	2
1985	Splicing factor 3B subunit 4	IPI00017339	-0.653	0.11149	3	2	2	0
1986	Isoform 2 of 39S ribosomal protein L39, mitochondrial	IPI00084571	-0.653	0.11149	2	3	2	1

No.	Description	Accession number	STN[1]	p-Value[1]	Con_A[2]	Con_B[2]	CUR_A[2]	CUR_B[2]
1987	Flotillin-2	IPI00789008	-0.653	0.11149	4	1	2	0
1988	Isoform 1 of Retinol dehydrogenase 11	IPI00339384	-0.653	0.11149	3	2	1	2
1989	Component of gems 4	IPI00027717	-0.653	0.11149	3	2	1	2
1990	Isoform AGX2 of UDP-N-acetylhexosamine pyrophosphorylase	IPI00000684	-0.653	0.11149	3	2	2	1
1991	Isoform 4 of Uncharacterized protein KIAA0090	IPI00642244	-0.653	0.11149	4	1	2	1
1992	Isoform Long of Metastasis-associated protein MTA1	IPI00012773	-0.653	0.11149	2	3	1	2
1993	NADH dehydrogenase [ubiquinone] 1 alpha subcomplex subunit 6	IPI00419266	-0.653	0.11149	3	2	1	2
1994	Isoform 2 of Actin-related protein 2/3 complex subunit 5	IPI00007280	-0.653	0.11149	2	3	1	2
1995	Isoform SRP40-1 of Splicing factor, arginine/serine-rich 5	IPI00012341	-0.653	0.11149	3	2	2	1
1996	cDNA FLJ61386, highly similar to Homo sapiens mitochondrial ribosomal protein L43 (MRPL43)	IPI00334579	-0.653	0.11149	3	2	2	1
1997	Importin-8	IPI00007401	-0.653	0.11149	3	2	2	1
1998	Isoform 2 of Pre-mRNA-splicing factor ISY1 homolog	IPI00063673	-0.653	0.11149	3	2	0	2
1999	Nucleolysin TIAR	IPI00005615	-0.653	0.11149	3	2	2	1
2000	NEDD4-like E3 ubiquitin-protein ligase WWP2	IPI00013010	-0.653	0.11149	2	3	1	2
2001	Dual specificity protein phosphatase 3	IPI00018671	-0.653	0.11149	2	3	2	1
2002	[Pyruvate dehydrogenase [lipoamide]] kinase isozyme 3, mitochondrial	IPI00014849	-0.653	0.11149	3	2	0	2
2003	Syndecan-1	IPI00002441	-0.653	0.11149	1	4	2	1
2004	Golgin subfamily A member 7	IPI00480022	-0.653	0.11149	3	2	2	1
2005	Isoform 1 of Beta-1-syntrophin	IPI00026059	-0.653	0.11149	3	2	2	0
2006	Pterin-4-alpha-carbinolamine dehydratase	IPI00218568	-0.653	0.11149	2	3	2	1
2007	PCTP-like protein	IPI00186008	-0.653	0.11149	2	3	0	2
2008	Isoform 1 of Nuclear pore complex protein Nup160	IPI00748807	-0.656	0.11130	25	26	22	24
2009	Mortality factor 4-like protein 2	IPI00014174	-0.664	0.10946	6	7	6	4
2010	myosin-IXb isoform 1	IPI00306933	-0.664	0.10946	6	7	5	5
2011	Isoform 1 of Apoptotic chromatin condensation inducer in the nucleus	IPI00007334	-0.664	0.10946	6	7	4	6
2012	Phosphatidylserine synthase 1	IPI00010746	-0.664	0.10946	6	7	5	5
2013	similar to RAN binding protein 1	IPI00399212	-0.664	0.10946	6	7	5	5
2014	Nucleosome assembly protein 1-like 1	IPI00023860	-0.667	0.10828	25	24	24	20
2015	26S protease regulatory subunit 8	IPI00023919	-0.668	0.10823	15	12	14	9
2016	Protein RCC2	IPI00465044	-0.678	0.10795	14	12	12	10
2017	Cathepsin D	IPI00011229	-0.678	0.10795	12	14	11	11
2018	Dipeptidyl peptidase 1	IPI00022810	-0.683	0.10710	23	23	19	22
2019	Ribosomal protein S6 kinase alpha-3	IPI00020898	-0.687	0.10676	6	6	5	4
2020	Cytochrome c-type heme lyase	IPI00023406	-0.687	0.10676	5	7	4	5
2021	Peroxisomal membrane protein PMP34	IPI00014440	-0.687	0.10676	6	6	4	5
2022	Putative uncharacterized protein NAPRT1	IPI00412498	-0.687	0.10676	6	6	5	4
2023	Probable methylthioribulose-1-phosphate dehydratase	IPI00549730	-0.687	0.10676	6	6	6	3
2024	Nucleolar pre-ribosomal-associated protein 1	IPI00297241	-0.688	0.10558	13	12	10	11
2025	Inosine-5'-monophosphate dehydrogenase 2	IPI00291510	-0.689	0.10558	21	24	20	20
2026	Regulation of nuclear pre-mRNA domain-containing protein 1B	IPI00009659	-0.700	0.10497	11	13	11	9
2027	RuvB-like 2	IPI00009104	-0.712	0.10204	34	33	27	34
2028	Peptidyl-prolyl cis-trans isomerase D	IPI00003927	-0.712	0.10204	6	5	6	2
2029	Isoform 1 of Protein phosphatase 1 regulatory subunit 7	IPI00033600	-0.712	0.10204	5	6	4	4
2030	Pyridoxine-5'-phosphate oxidase	IPI00018272	-0.712	0.10204	5	6	3	5
2031	39S ribosomal protein L20, mitochondrial	IPI00013706	-0.712	0.10204	6	5	4	4
2032	Dolichyl-diphosphooligosaccharide--protein glycosyltransferase subunit DAD1	IPI00009407	-0.712	0.10204	5	6	3	5
2033	Tyrosyl-tRNA synthetase, cytoplasmic	IPI00007074	-0.714	0.10166	20	21	18	18
2034	Isoform Short of Heterogeneous nuclear ribonucleoprotein U	IPI00479217	-0.721	0.10077	96	98	99	86
2035	Probable ribosome biogenesis protein NEP1	IPI00025347	-0.724	0.10072	12	10	10	8
2036	cDNA FLJ36192 fis, clone TESTI2027450, highly similar to Eukaryotic translation initiation factor 3	IPI00654777	-0.724	0.10072	12	10	9	9
2037	Heterogeneous nuclear ribonucleoprotein L	IPI00027834	-0.724	0.09892	46	51	47	43
2038	Isoform 1 of Minor histocompatibility antigen H13	IPI00152441	-0.729	0.09845	2	2	0	1
2039	Isoform 5 of Protein polybromo-1	IPI00023097	-0.729	0.09845	1	3	0	0
2040	Casein kinase II subunit beta	IPI00010865	-0.729	0.09845	2	2	1	0
2041	Isoform 1 of Chromosome-associated kinesin KIF4A	IPI00178150	-0.729	0.09845	3	1	1	0
2042	Cytochrome c oxidase subunit 6A1, mitochondrial	IPI00021793	-0.729	0.09845	2	2	0	0
2043	ANKHD1-EIF4EBP3 protein	IPI00217442	-0.729	0.09845	1	3	1	1
2044	Isoform 1 of Mitogen-activated protein kinase kinase kinase kinase 4	IPI00006752	-0.729	0.09845	3	0	0	1
2045	ATP-dependent RNA helicase DDX54 isoform 1	IPI00152510	-0.729	0.09845	2	2	1	1
2046	Isoform 1 of Serine/threonine-protein phosphatase PGAM5, mitochondrial	IPI00788907	-0.729	0.09845	2	2	1	1
2047	Isoform 1 of Solute carrier family 12 member 7	IPI00008616	-0.729	0.09845	3	1	0	1
2048	High mobility group protein B2	IPI00219097	-0.729	0.09845	2	2	0	0
2049	Isoform Short of Ubiquitin fusion degradation protein 1 homolog	IPI00218292	-0.729	0.09845	2	2	1	1
2050	Isoform 1 of GPI transamidase component PIG-S	IPI00465308	-0.729	0.09845	0	3	1	1
2051	Isoform 1 of OCIA domain-containing protein 1	IPI00016405	-0.729	0.09845	1	3	1	1
2052	THUMP domain-containing protein 3	IPI00306127	-0.729	0.09845	3	1	1	1
2053	Exosome complex exonuclease RRP46	IPI00015955	-0.729	0.09845	2	2	1	1
2054	Uncharacterized protein C20orf4	IPI00166013	-0.729	0.09845	2	2	0	1
2055	Putative uncharacterized protein WDR43	IPI00892938	-0.729	0.09845	2	2	1	0
2056	Isoform 1 of Kinesin-like protein KIF21A	IPI00425404	-0.729	0.09845	3	1	1	1
2057	YEATS domain-containing protein 4	IPI00008536	-0.729	0.09845	2	2	1	1
2058	Putative myosin-XVB	IPI00786880	-0.729	0.09845	2	2	0	0
2059	DnaJ homolog subfamily C member 17	IPI00018798	-0.729	0.09845	1	3	1	1
2060	Ubiquitin-conjugating enzyme E2 S	IPI00217949	-0.729	0.09845	2	2	0	0
2061	Nucleoporin Nup43	IPI00742943	-0.729	0.09845	2	2	1	1
2062	Isoform 1 of Malonyl-CoA-acyl carrier protein transacylase, mitochondrial	IPI00023359	-0.729	0.09845	1	3	1	1
2063	Isoform 1 of Collagen type IV alpha-3-binding protein	IPI00024701	-0.729	0.09845	2	2	1	1
2064	Polyadenylate-binding protein-interacting protein 2	IPI00304483	-0.729	0.09845	0	3	1	0
2065	Isoform 1 of Stromal membrane-associated protein 1	IPI00102096	-0.729	0.09845	2	2	1	1
2066	Isoform Alpha of Nuclear inhibitor of protein phosphatase 1	IPI00030383	-0.729	0.09845	3	1	1	1
2067	Isoform 1 of Copine-7	IPI00002657	-0.729	0.09845	2	2	1	0
2068	cDNA FLJ35172 fis, clone PLACE6013232	IPI00385785	-0.729	0.09845	2	2	1	1
2069	PIH1 domain-containing protein 1	IPI00550995	-0.729	0.09845	2	2	0	1
2070	Isoform 2 of Suppressor of SWI4 1 homolog	IPI00219793	-0.729	0.09845	2	2	0	0
2071	Cleavage stimulation factor subunit 1	IPI00011528	-0.729	0.09845	3	1	0	0
2072	Isoform 3 of F-box only protein 22	IPI00169168	-0.729	0.09845	2	2	1	0
2073	Uncharacterized protein C20orf29	IPI00019941	-0.729	0.09845	2	2	0	1
2074	RhoA activator C11orf59	IPI00016670	-0.729	0.09845	0	3	1	1
2075	V-type proton ATPase subunit d 1	IPI00034159	-0.729	0.09845	3	1	1	1
2076	Prefoldin subunit 6	IPI00005657	-0.729	0.09845	2	2	0	0
2077	TIM21-like protein, mitochondrial	IPI00306439	-0.729	0.09845	2	2	1	1
2078	Isoform Long of Spectrin beta chain, brain 1	IPI00005614	-0.730	0.08125	96	92	92	87
2079	Nestin	IPI00010800	-0.735	0.08097	17	21	16	17
2080	SUMO-activating enzyme subunit 1	IPI00033130	-0.738	0.08097	10	11	9	8
2081	Coatomer subunit delta variant 2	IPI00298520	-0.738	0.08097	11	10	7	10

No.	Description	Accession number	STN[1]	p-Value[1]	Con_A[2]	Con_B[2]	CUR_A[2]	CUR_B[2]
2082	cDNA FLJ59211, highly similar to Glucosidase 2 subunit beta	IPI00026154	-0.738	0.08092	32	29	28	27
2083	NAD-dependent malic enzyme, mitochondrial	IPI00011201	-0.742	0.08092	4	6	4	3
2084	Protein dpy-30 homolog	IPI00028109	-0.742	0.08092	4	6	4	3
2085	Serine/threonine-protein kinase 38-like	IPI00237011	-0.742	0.08092	4	6	3	4
2086	Gem-associated protein 5	IPI00291783	-0.742	0.08092	6	4	5	2
2087	Dual specificity mitogen-activated protein kinase kinase 2	IPI00003783	-0.742	0.08092	5	5	2	5
2088	Isoform 1 of Elongation factor Ts, mitochondrial	IPI00021016	-0.742	0.08092	7	3	4	3
2089	Adenylosuccinate synthetase isozyme 2	IPI00026833	-0.742	0.08092	5	5	5	2
2090	Nucleolar protein 16	IPI00032849	-0.742	0.08092	5	5	3	4
2091	Isoform 1 of Phosphatidylinositol glycan anchor biosynthesis class U protein	IPI00026044	-0.742	0.08092	5	5	3	4
2092	Isoform 1 of Transcription elongation factor SPT6	IPI00784161	-0.742	0.08092	5	5	4	3
2093	Calcium-binding protein 39	IPI00032561	-0.742	0.08092	5	5	3	4
2094	Thioredoxin-related transmembrane protein 1	IPI00395887	-0.742	0.08092	4	6	3	4
2095	Cell division protein kinase 7	IPI00000685	-0.742	0.08092	5	5	4	3
2096	Isoform 1 of RNA-binding protein 39	IPI00163505	-0.743	0.07880	18	19	16	16
2097	CAAX prenyl protease 1 homolog	IPI00027180	-0.753	0.07757	12	8	8	8
2098	COP9 signalosome complex subunit 3	IPI00025721	-0.753	0.07757	11	9	8	8
2099	Isoform 2 of Guanine nucleotide-binding protein G(i) subunit alpha-2	IPI00217906	-0.759	0.07559	17	18	12	18
2100	UPF0687 protein C20orf27	IPI00101095	-0.769	0.07483	10	9	7	8
2101	Dihydropteridine reductase	IPI00014439	-0.769	0.07483	10	9	6	9
2102	Isoform 1 of CCR4-NOT transcription complex subunit 1	IPI00166010	-0.778	0.07426	4	5	4	2
2103	Inositol 1,4,5-trisphosphate receptor type 3	IPI00291607	-0.778	0.07426	3	6	3	3
2104	Isoform GTBP-N of DNA mismatch repair protein Msh6	IPI00384456	-0.778	0.07426	6	3	4	2
2105	GTP-binding protein Rheb	IPI00016669	-0.778	0.07426	4	5	3	3
2106	cDNA FLJ54536, highly similar to Mitochondrial 28S ribosomal protein S27	IPI00022002	-0.778	0.07426	5	4	3	3
2107	Isoform 1 of Gamma-tubulin complex component 3	IPI00033516	-0.778	0.07426	3	6	4	2
2108	Isoform Long of Transformer-2 protein homolog alpha	IPI00013891	-0.778	0.07426	4	5	3	3
2109	cytochrome b5 type B precursor	IPI00303954	-0.786	0.07285	9	9	8	6
2110	Isoform 2 of Isochorismatase domain-containing protein 2, mitochondrial	IPI00003031	-0.786	0.07285	9	9	9	5
2111	Alpha-soluble NSF attachment protein	IPI00009253	-0.786	0.07285	11	7	9	5
2112	ADP/ATP translocase 1	IPI00022891	-0.786	0.07285	9	9	7	7
2113	Vimentin	IPI00418471	-0.787	0.07025	39	39	34	37
2114	Isoform 2 of Inverted formin-2	IPI00876962	-0.796	0.06992	18	13	12	14
2115	Eukaryotic translation initiation factor 2 subunit 1	IPI00219678	-0.797	0.06959	22	28	20	24
2116	Condensin complex subunit 3	IPI00106495	-0.805	0.06878	9	8	9	4
2117	60S ribosomal protein L36	IPI00216237	-0.805	0.06878	8	9	6	7
2118	Isoform 2 of Serine/threonine-protein kinase PAK 3	IPI00027382	-0.805	0.06878	8	9	7	6
2119	60S ribosomal protein L32	IPI00395998	-0.805	0.06878	9	8	7	6
2120	Isoform 1 of Nucleoside diphosphate kinase B	IPI00026260	-0.815	0.06831	35	36	32	32
2121	Actin-related protein 2/3 complex subunit 4	IPI00554811	-0.816	0.06831	23	24	21	20
2122	rRNA 2'-O-methyltransferase fibrillarin	IPI00025039	-0.816	0.06831	22	25	20	21
2123	mRNA turnover protein 4 homolog	IPI00106491	-0.818	0.06831	14	15	12	12
2124	Cold-inducible RNA-binding protein	IPI00180954	-0.818	0.06831	16	13	12	12
2125	DNA mismatch repair protein Msh3	IPI00329605	-0.820	0.06817	3	5	3	2
2126	Amidophosphoribosyltransferase	IPI00029534	-0.820	0.06817	5	3	3	2
2127	COP9 signalosome complex subunit 8	IPI00009480	-0.820	0.06817	4	4	3	2
2128	Isoform 2 of Tether containing UBX domain for GLUT4	IPI00065276	-0.820	0.06817	3	5	3	2
2129	Pre-mRNA-splicing factor SPF27	IPI00025178	-0.820	0.06817	4	4	3	2
2130	Developmentally-regulated GTP-binding protein 2	IPI00022697	-0.820	0.06817	5	3	4	1
2131	Isoform 1 of AP-2 complex subunit beta	IPI00784156	-0.825	0.06656	7	9	7	5
2132	L-aminoadipate-semialdehyde dehydrogenase-phosphopantetheinyl transferase	IPI00250297	-0.825	0.06656	8	8	5	7
2133	Importin subunit alpha-1	IPI00303292	-0.825	0.06656	9	7	6	6
2134	Isoform Long of Eukaryotic translation initiation factor 4H	IPI00014263	-0.829	0.06458	15	13	12	11
2135	ADP-ribosylation factor-like protein 1	IPI00219518	-0.829	0.06458	16	12	12	11
2136	Bifunctional purine biosynthesis protein PURH	IPI00289499	-0.830	0.06444	46	51	44	45
2137	ADP/ATP translocase 2	IPI00007188	-0.842	0.06392	234	191	217	194
2138	CSNK2A1 protein	IPI00016613	-0.848	0.06359	7	8	6	5
2139	Ras-related protein Rab-1B	IPI00008964	-0.848	0.06359	6	9	6	5
2140	Isoform 1 of Translocon-associated protein subunit alpha	IPI00301021	-0.848	0.06359	7	8	5	6
2141	ATP-dependent RNA helicase DDX18	IPI00301323	-0.848	0.06359	34	30	29	28
2142	Nucleolar protein 56	IPI00411937	-0.848	0.06359	31	33	31	26
2143	Alpha-actinin-1	IPI00013508	-0.849	0.06359	172	168	177	150
2144	Eukaryotic translation initiation factor 3 subunit M	IPI00102069	-0.853	0.06359	20	22	18	18
2145	Vacuolar protein sorting-associated protein 35	IPI00018931	-0.853	0.06344	28	35	29	27
2146	SERPINE1 mRNA binding protein 1, isoform CRA_d	IPI00410693	-0.854	0.06330	14	12	8	13
2147	Isoform 1 of Trans-2,3-enoyl-CoA reductase	IPI00100656	-0.868	0.06307	12	13	10	10
2148	Activator of 90 kDa heat shock protein ATPase homolog 1	IPI00030706	-0.869	0.06293	19	21	18	16
2149	Isoform Long of Antigen KI-67	IPI00004233	-0.873	0.06189	7	7	7	3
2150	SWI/SNF-related matrix-associated actin-dependent regulator of chromatin subfamily A member 5	IPI00297211	-0.873	0.06189	6	8	5	5
2151	Isoform 1 of Splicing factor, arginine/serine-rich 15	IPI00181702	-0.873	0.06189	6	8	5	5
2152	Isoform 1 of Luc7-like protein 3	IPI00107745	-0.873	0.06189	7	7	6	4
2153	Coiled-coil domain-containing protein 6	IPI00000634	-0.873	0.06189	7	7	5	5
2154	myosin regulatory light polypeptide 9 isoform b	IPI00030929	-0.873	0.06189	7	7	6	4
2155	Golgi-specific brefeldin A-resistance guanine nucleotide exchange factor 1	IPI00021954	-0.874	0.05919	3	4	1	3
2156	Isoform 1 of Protein fto	IPI00028277	-0.874	0.05919	2	5	3	1
2157	telomerase-binding protein EST1A isoform 2	IPI00014252	-0.874	0.05919	5	2	2	2
2158	Ladinin-1	IPI00514234	-0.874	0.05919	3	4	2	2
2159	cDNA FLJ54775, highly similar to Syntaxin-binding protein 2	IPI00019971	-0.874	0.05919	4	3	2	2
2160	Isoform 1 of Calcium-binding mitochondrial carrier protein SCaMC-1	IPI00337494	-0.874	0.05919	4	3	2	2
2161	Polymerase delta-interacting protein 2	IPI00165506	-0.874	0.05919	4	3	3	1
2162	Isoform 2 of Basic leucine zipper and W2 domain-containing protein 1	IPI00180128	-0.874	0.05919	3	4	2	2
2163	Phosphoglycolate phosphatase	IPI00177008	-0.874	0.05919	3	4	2	2
2164	cDNA FLJ56389, highly similar to Elongation factor 1-gamma	IPI00000875	-0.875	0.05806	31	28	23	29
2165	Transmembrane protein 33	IPI00299084	-0.882	0.05801	12	12	9	10
2166	WD repeat-containing protein 75	IPI00217240	-0.887	0.05792	21	17	17	15
2167	Tu translation elongation factor, mitochondrial precursor	IPI00027107	-0.887	0.05792	19	19	15	17
2168	Isoform 1 of Catenin beta-1	IPI00017292	-0.896	0.05735	21	16	16	15
2169	Dolichyl-diphosphooligosaccharide--protein glycosyltransferase subunit STT3B	IPI00152377	-0.896	0.05735	20	17	17	14
2170	Eukaryotic translation initiation factor 3 subunit C	IPI00016910	-0.898	0.05735	12	11	10	8
2171	GDP-mannose 4,6 dehydratase	IPI00030207	-0.898	0.05735	12	11	12	6
2172	Ras-related protein Rab-35	IPI00300096	-0.898	0.05735	12	11	9	9
2173	cDNA FLJ55586, highly similar to MMS19-like protein	IPI00154451	-0.902	0.05726	6	7	5	4
2174	Myosin regulatory light chain 12B	IPI00033494	-0.902	0.05726	6	7	5	4
2175	U8 snoRNA-decapping enzyme	IPI00783497	-0.902	0.05726	7	6	5	4
2176	Isoform 1 of Heterogeneous nuclear ribonucleoprotein D-like	IPI00011274	-0.906	0.05726	28	26	23	24

No.	Description	Accession number	STN[1]	p-Value[1]	Con_A[2]	Con_B[2]	CUR_A[2]	CUR_B[2]
2177	Isoform 1 of Myosin-10	IPI00397526	-0.908	0.05678	95	85	83	86
2178	Isoform 3 of Core histone macro-H2A.1	IPI00059366	-0.910	0.05678	32	44	34	34
2179	Isoform 1 of Heterogeneous nuclear ribonucleoprotein R	IPI00012074	-0.911	0.05678	51	53	48	47
2180	Isoform 1 of Keratin, type I cytoskeletal 13	IPI00009866	-0.912	0.05678	28	25	21	25
2181	V-type proton ATPase catalytic subunit A	IPI00007682	-0.912	0.05678	28	25	24	22
2182	60S ribosomal protein L13	IPI00465361	-0.914	0.05674	11	11	8	9
2183	DNA-(apurinic or apyrimidinic site) lyase	IPI00215911	-0.914	0.05674	11	11	9	8
2184	Isoform 2 of Guanine nucleotide-binding protein-like 3	IPI00003886	-0.914	0.05674	12	10	7	10
2185	60S ribosomal protein L22	IPI00219153	-0.914	0.05674	12	10	9	8
2186	Isoform 1 of Mitotic checkpoint protein BUB3	IPI00013468	-0.914	0.05674	10	12	10	7
2187	Flap endonuclease 1	IPI00026215	-0.932	0.05570	9	12	10	6
2188	Isoform Short of TATA-binding protein-associated factor 2N	IPI00020194	-0.934	0.05565	6	6	4	4
2189	cDNA FLJ25678 fis, clone TST04067, highly similar to PURINE NUCLEOSIDE PHOSPHORYLASE	IPI00017672	-0.934	0.05239	36	35	30	33
2190	60S ribosomal protein L10a	IPI00412579	-0.935	0.05239	46	51	43	45
2191	Dolichyl-diphosphooligosaccharide--protein glycosyltransferase subunit 1 precursor	IPI00025874	-0.935	0.05239	48	49	43	45
2192	Isoform 1 of Polyadenylate-binding protein 2	IPI00005792	-0.944	0.05215	3	3	1	2
2193	Serine/threonine-protein phosphatase 1 regulatory subunit 10	IPI00298731	-0.944	0.05215	3	3	1	2
2194	Nucleolar protein 9	IPI00002902	-0.944	0.05215	3	3	2	1
2195	Nucleoporin 54kDa variant (Fragment)	IPI00172580	-0.944	0.05215	2	4	2	1
2196	Isoform 1 of Protein tyrosine phosphatase type IVA 2	IPI00020191	-0.944	0.05215	4	2	2	1
2197	Isoform 1 of PDZ domain-containing protein 11	IPI00550841	-0.944	0.05215	4	2	1	2
2198	Methionine aminopeptidase 2	IPI00033036	-0.944	0.05215	2	4	2	0
2199	Na(+)/H(+) exchange regulatory cofactor NHE-RF1	IPI00003527	-0.944	0.05215	3	3	1	2
2200	Mitochondrial chaperone BCS1	IPI00003985	-0.944	0.05215	3	3	2	0
2201	Ketosamine-3-kinase	IPI00099986	-0.944	0.05215	4	2	1	2
2202	Isoform 1 of HEAT repeat-containing protein 3	IPI00100984	-0.944	0.05215	3	3	1	2
2203	Acidic leucine-rich nuclear phosphoprotein 32 family member A	IPI00025849	-0.944	0.05215	4	2	1	2
2204	Isoform 1 of Probable aminopeptidase NPEPL1	IPI00100292	-0.944	0.05215	4	2	2	1
2205	Isoform A of Protein CutA	IPI00034319	-0.944	0.05215	3	3	1	2
2206	Calcium-binding protein p22	IPI00218924	-0.944	0.05215	3	3	2	1
2207	HLA class I histocompatibility antigen, B-7 alpha chain	IPI00004657	-0.944	0.05215	2	4	2	1
2208	Protein kinase C iota type	IPI00016639	-0.944	0.05215	3	3	1	2
2209	40S ribosomal protein S9	IPI00221088	-0.948	0.05215	23	25	22	19
2210	Replication protein A 70 kDa DNA-binding subunit	IPI00020127	-0.950	0.05215	18	14	15	11
2211	Isoform 1 of Polyadenylate-binding protein 1	IPI00008524	-0.950	0.05135	42	51	40	44
2212	cDNA FLJ56153, highly similar to Homo sapiens transforming growth factor beta regulator 4 (TBRG4)	IPI00329625	-0.951	0.05130	10	10	8	7
2213	Poly(rC)-binding protein 1	IPI00016610	-0.951	0.05130	13	7	8	7
2214	Thymidylate kinase	IPI00013862	-0.951	0.05130	9	11	9	6
2215	cDNA FLJ59367, highly similar to Adenylosuccinate lyase	IPI00026904	-0.951	0.05130	11	9	9	6
2216	Laminin receptor-like protein LAMRL5	IPI00411639	-0.955	0.05102	32	35	32	27
2217	Short heat shock protein 60 Hsp60s2	IPI00076042	-0.962	0.05078	14	17	12	13
2218	Cytochrome b-c1 complex subunit 2, mitochondrial	IPI00305383	-0.962	0.05078	18	13	13	12
2219	GTP-binding protein SAR1a	IPI00015954	-0.962	0.05078	16	15	14	11
2220	Isoform 2 of Protein disulfide-isomerase A6	IPI00299571	-0.962	0.05078	17	14	13	12
2221	Plastin-1	IPI00032304	-0.964	0.05078	22	24	19	20
2222	NAD(P) transhydrogenase, mitochondrial	IPI00337541	-0.966	0.05078	44	45	46	34
2223	Isoform 1 of 3-hydroxyacyl-CoA dehydrogenase type-2	IPI00017726	-0.966	0.05078	39	50	40	40
2224	cDNA FLJ45706 fis, clone FEBRA2028457, highly similar to Nucleolin	IPI00444262	-0.970	0.05078	60	57	54	53
2225	V-type proton ATPase subunit F	IPI00004488	-0.972	0.05078	5	6	3	4
2226	Isoform A of DnaJ homolog subfamily B member 6	IPI00024523	-0.972	0.05078	5	6	4	3
2227	Serine/threonine-protein kinase mTOR	IPI00031410	-0.972	0.05078	10	9	7	7
2228	Keratin-8-like protein 1	IPI00017870	-0.975	0.05064	42	45	43	35
2229	Cytochrome c oxidase subunit 2	IPI00017510	-0.975	0.05064	16	14	11	13
2230	cDNA FLJ60076, highly similar to ELAV-like protein 1	IPI00301936	-0.981	0.05022	22	22	19	18
2231	60S ribosomal protein L23a	IPI00021266	-0.988	0.05022	43	41	37	38
2232	Histone-binding protein RBBP4	IPI00328319	-0.988	0.05022	14	15	12	11
2233	Putative uncharacterized protein PSME2	IPI00384051	-0.988	0.05022	16	13	13	10
2234	Cytokine-like nuclear factor n-pac, isoform CRA_a	IPI00000155	-0.995	0.05012	9	9	7	6
2235	Probable ATP-dependent RNA helicase DDX47	IPI00023972	-1.003	0.04951	14	14	9	13
2236	Chromobox protein homolog 3	IPI00297579	-1.009	0.04885	21	20	18	16
2237	Serine/threonine-protein kinase OSR1	IPI00010080	-1.016	0.04875	5	5	4	2
2238	Isoform 1 of Ribonuclease H2 subunit C	IPI00382985	-1.016	0.04875	4	6	3	3
2239	Isoform 1 of Nuclear-interacting partner of ALK	IPI00301421	-1.016	0.04875	5	5	4	2
2240	Isoform 1 of Protein zwilch homolog	IPI00329679	-1.016	0.04875	5	5	2	4
2241	Acyl-CoA dehydrogenase family member 9, mitochondrial	IPI00152981	-1.016	0.04875	6	4	4	2
2242	ATP-dependent RNA helicase DDX3X	IPI00215637	-1.017	0.04422	37	41	33	36
2243	Proteasome activator complex subunit 1	IPI00479722	-1.017	0.04422	30	27	28	21
2244	Heterogeneous nuclear ribonucleoprotein C-like 1	IPI00027569	-1.018	0.04422	76	57	65	57
2245	Coatomer subunit beta	IPI00295851	-1.019	0.04422	22	18	18	15
2246	Isoform Crk-II of Adapter molecule crk	IPI00004838	-1.020	0.04422	8	9	6	6
2247	Pre-rRNA-processing protein TSR1 homolog	IPI00292894	-1.020	0.04422	8	9	6	6
2248	Heat shock protein HSP 90-beta	IPI00414676	-1.028	0.04365	127	121	111	123
2249	Calcium-binding mitochondrial carrier protein Aralar2	IPI00007084	-1.032	0.04365	28	27	27	20
2250	Coatomer subunit gamma	IPI00783982	-1.034	0.04360	11	15	11	9
2251	Ribosome biogenesis protein BMS1 homolog	IPI00006099	-1.034	0.04360	14	12	10	10
2252	Isoform 1 of Structural maintenance of chromosomes protein 2	IPI00007927	-1.034	0.04360	14	12	9	11
2253	60S ribosomal protein L7a	IPI00299573	-1.037	0.04337	40	34	33	32
2254	Isoform 3 of Splicing factor, arginine/serine-rich 13A	IPI00009071	-1.040	0.04327	18	20	15	16
2255	Monocarboxylate transporter 1	IPI00024650	-1.044	0.04327	3	2	0	1
2256	DNA polymerase alpha subunit B	IPI00290272	-1.044	0.04327	3	2	1	0
2257	Isoform 2 of Condensin-2 complex subunit G2	IPI00396058	-1.044	0.04327	2	3	0	0
2258	NADH dehydrogenase [ubiquinone] 1 beta subcomplex subunit 9	IPI00255052	-1.044	0.04327	3	2	1	1
2259	Isoform 1 of Protein dpy-19 homolog 1	IPI00007461	-1.044	0.04327	3	2	1	1
2260	Coiled-coil domain-containing protein 134	IPI00302674	-1.044	0.04327	3	2	1	1
2261	Isoform 2 of RNA-binding protein 47	IPI00005042	-1.044	0.04327	2	3	1	1
2262	Fatty acyl-CoA reductase 1	IPI00478838	-1.044	0.04327	2	3	0	0
2263	Phosphatidylinositol-5-phosphate 4-kinase type-2 gamma	IPI00152303	-1.044	0.04327	4	1	1	1
2264	Zinc finger protein ubi-d4	IPI00023322	-1.044	0.04327	3	2	1	1
2265	Actin-related protein 2/3 complex subunit 5-like protein	IPI00414554	-1.044	0.04327	2	3	1	0
2266	Isoform 1 of Ubiquitin-protein ligase E3C	IPI00604464	-1.044	0.04327	3	2	1	0
2267	DNA replication complex GINS protein PSF2	IPI00007146	-1.044	0.04327	4	1	1	0
2268	interferon alpha responsive protein	IPI00007096	-1.044	0.04327	2	3	1	1
2269	Isoform Long of Tyrosine-protein kinase SYK	IPI00018597	-1.044	0.04327	4	1	1	0
2270	Uncharacterized protein C20orf72	IPI00001287	-1.044	0.04327	2	3	0	0

No.	Description	Accession number	STN[1]	p-Value[1]	Con_A[2]	Con_B[2]	CUR_A[2]	CUR_B[2]
2271	Isoform 1 of Stimulated by retinoic acid gene 6 protein homolog	IPI00465247	-1.044	0.04327	1	4	0	0
2272	Cob(I)yrinic acid a,c-diamide adenosyltransferase, mitochondrial	IPI00029665	-1.044	0.04327	2	3	1	0
2273	Putative uncharacterized protein INF2	IPI00872508	-1.047	0.04247	7	9	6	5
2274	Putative uncharacterized protein FUBP3	IPI00063245	-1.047	0.04247	9	7	6	5
2275	Dolichyl-diphosphooligosaccharide--protein glycosyltransferase subunit 2	IPI00028635	-1.050	0.04190	96	96	91	88
2276	Isoform Beta of Nucleolar and coiled-body phosphoprotein 1	IPI00216654	-1.051	0.04190	9	16	8	11
2277	Isoform 2 of S-phase kinase-associated protein 1	IPI00172421	-1.052	0.04190	13	24	15	15
2278	40S ribosomal protein S5	IPI00008433	-1.060	0.04190	33	37	29	32
2279	Microtubule-associated protein RP/EB family member 1	IPI00017596	-1.063	0.04186	18	18	14	15
2280	Isocitrate dehydrogenase [NADP], mitochondrial	IPI00011107	-1.068	0.04186	5	4	1	4
2281	26S proteasome non-ATPase regulatory subunit 10	IPI00003565	-1.068	0.04186	3	6	4	1
2282	Charged multivesicular body protein 7	IPI00395463	-1.068	0.04186	4	5	3	2
2283	Tropomodulin-3	IPI00005087	-1.068	0.04186	5	4	2	3
2284	Probable rRNA-processing protein EBP2	IPI00745955	-1.068	0.04186	5	4	2	3
2285	Isoform 1 of HBS1-like protein	IPI00009070	-1.068	0.04186	5	4	3	2
2286	PDXDC1 protein	IPI00329208	-1.068	0.04186	4	5	3	2
2287	Visinin-like protein 1	IPI00216313	-1.077	0.04063	7	8	5	5
2288	Valyl-tRNA synthetase	IPI00000873	-1.085	0.04053	35	31	29	28
2289	Importin subunit alpha-4	IPI00012578	-1.088	0.04049	12	11	8	9
2290	60S ribosomal protein L15	IPI00470528	-1.104	0.04006	33	30	26	28
2291	Ras-related protein Rab-18	IPI00014577	-1.109	0.04001	12	10	8	8
2292	RcDNAJ9 (Fragment)	IPI00014718	-1.111	0.03907	5	9	5	4
2293	High mobility group protein B3	IPI00217477	-1.111	0.03907	7	7	4	5
2294	Villin-1	IPI00218852	-1.111	0.03878	32	30	27	26
2295	Intron-binding protein aquarius	IPI00297572	-1.116	0.03878	18	14	13	12
2296	Isoform 1 of General transcription factor II-I	IPI00054042	-1.118	0.03878	28	33	25	27
2297	Dynein light chain 1, cytoplasmic	IPI00019329	-1.134	0.03855	4	4	2	2
2298	DNA polymerase	IPI00744598	-1.134	0.03855	5	3	1	3
2299	Vacuolar protein sorting-associated protein 33A	IPI00073179	-1.134	0.03855	4	4	2	2
2300	Keratin, type I cytoskeletal 24	IPI00004550	-1.134	0.03855	4	4	3	1
2301	Nucleoporin Nup37	IPI00171665	-1.134	0.03855	3	5	1	3
2302	Isoform 1 of COMM domain-containing protein 4	IPI00413500	-1.134	0.03855	4	4	3	1
2303	Adenylate kinase isoenzyme 6	IPI00032879	-1.134	0.03855	4	4	2	2
2304	Isoform 1 of Fermitin family homolog 1	IPI00304754	-1.134	0.03855	3	5	2	2
2305	BRO1 domain-containing protein BROX	IPI00065500	-1.134	0.03855	4	4	1	3
2306	Isoform 1 of YTH domain family protein 1	IPI00221345	-1.134	0.03855	5	3	3	1
2307	Isoform 1 of Protein syndesmos	IPI00031650	-1.134	0.03855	4	4	2	2
2308	ADP-ribosylation factor 5	IPI00215919	-1.134	0.03855	5	3	3	0
2309	T-complex protein 1 subunit alpha	IPI00290566	-1.135	0.03472	79	77	72	71
2310	Isoform 1 of Methylcrotonoyl-CoA carboxylase beta chain, mitochondrial	IPI00784044	-1.138	0.03472	19	24	20	15
2311	TUBA1C protein	IPI00166768	-1.144	0.03458	110	115	102	108
2312	Isoform 1 of Acidic leucine-rich nuclear phosphoprotein 32 family member B	IPI00007423	-1.146	0.03458	16	14	12	11
2313	Isoform 1 of Protein strawberry notch homolog 1	IPI00023649	-1.150	0.03411	5	8	2	6
2314	60S ribosomal protein L5	IPI00000494	-1.150	0.03411	5	8	4	4
2315	Isoform Long of Double-stranded RNA-binding protein Staufen homolog 1	IPI00000001	-1.150	0.03411	6	7	4	4
2316	COP9 signalosome complex subunit 7a	IPI00301419	-1.150	0.03411	5	8	4	4
2317	S-adenosylmethionine synthase isoform type-2	IPI00010157	-1.155	0.03387	11	9	9	5
2318	Isoform 2 of Triosephosphate isomerase	IPI00451401	-1.156	0.03312	43	31	29	35
2319	60S acidic ribosomal protein P2	IPI00008529	-1.162	0.03312	14	15	13	9
2320	Dihydropyrimidinase-like 2	IPI00106642	-1.165	0.03293	28	27	22	24
2321	Solute carrier family 2, facilitated glucose transporter member 1	IPI00220194	-1.171	0.03283	20	20	16	16
2322	Elongation factor 1-beta	IPI00178440	-1.171	0.03283	18	22	17	15
2323	Isoform 1 of Extended synaptotagmin-1	IPI00022143	-1.174	0.03250	28	26	24	21
2324	Nucleoporin 85	IPI00171542	-1.179	0.03250	15	13	11	10
2325	Bifunctional methylenetetrahydrofolate dehydrogenase/cyclohydrolase, mitochondrial	IPI00011307	-1.181	0.03250	10	9	6	7
2326	60S ribosomal protein L6	IPI00329389	-1.181	0.03250	45	45	41	38
2327	Plastin-2	IPI00010471	-1.186	0.03250	101	103	95	94
2328	Serine/threonine-protein phosphatase 2A 56 kDa regulatory subunit epsilon isoform	IPI00002853	-1.194	0.03227	6	6	3	4
2329	Isoform Long of Cold shock domain-containing protein E1	IPI00470891	-1.194	0.03227	5	7	4	3
2330	Isoform 2 of Tropomyosin alpha-3 chain	IPI00218319	-1.194	0.03227	5	7	3	4
2331	COP9 signalosome complex subunit 4	IPI00171844	-1.194	0.03227	7	5	4	3
2332	Poly [ADP-ribose] polymerase 1	IPI00449049	-1.196	0.03203	86	80	83	69
2333	Isoform 1 of Regulator of nonsense transcripts 1	IPI00034049	-1.209	0.03170	21	16	13	16
2334	Splicing factor 3A subunit 3	IPI00029764	-1.209	0.03170	20	17	15	14
2335	cDNA FLJ77422, highly similar to Homo sapiens RNA binding protein	IPI00011268	-1.209	0.03170	17	20	16	13
2336	Isoform Non-muscle of Myosin light polypeptide 6	IPI00335168	-1.210	0.03170	8	10	6	6
2337	33 kDa protein	IPI00413108	-1.210	0.03170	10	8	7	5
2338	Isoform 2 of Nuclear mitotic apparatus protein 1	IPI00006196	-1.213	0.03038	48	36	39	34
2339	Isoform 1 of General transcription factor 3C polypeptide 1	IPI00414482	-1.219	0.03033	3	4	1	2
2340	Splicing factor, arginine/serine-rich 11	IPI00464952	-1.219	0.03033	5	2	1	2
2341	Tubulin gamma-1 chain	IPI00295081	-1.219	0.03033	5	2	1	2
2342	Pseudouridylate synthase 7 homolog	IPI00044761	-1.219	0.03033	3	4	1	2
2343	U4/U6.U5 tri-snRNP-associated protein 1	IPI00021417	-1.219	0.03033	4	3	2	1
2344	Sterol-4-alpha-carboxylate 3-dehydrogenase, decarboxylating	IPI00019407	-1.219	0.03033	3	4	2	0
2345	WD repeat-containing protein 11	IPI00412224	-1.219	0.03033	5	2	2	1
2346	39S ribosomal protein L24, mitochondrial	IPI00514506	-1.219	0.03033	4	3	1	2
2347	60S ribosomal protein L23	IPI00010153	-1.231	0.02990	37	44	32	38
2348	cDNA FLJ34068 fis, clone FCBBF3001918	IPI00168184	-1.231	0.02990	33	30	27	26
2349	Isoform 2 of DNA replication licensing factor MCM7	IPI00219740	-1.237	0.02990	13	12	9	9
2350	40S ribosomal protein S16	IPI00221092	-1.241	0.02967	25	22	20	18
2351	40S ribosomal protein S3a	IPI00419880	-1.243	0.02967	39	40	33	35
2352	Non-functional aryl hydrocarbon receptor interacting protein (Fragment)	IPI00925804	-1.246	0.02967	7	4	5	1
2353	Probable methyltransferase TARBP1	IPI00298447	-1.246	0.02967	5	6	2	4
2354	Lysophosphatidylcholine acyltransferase 1	IPI00171626	-1.246	0.02967	5	6	3	3
2355	Similar to nonhistone chromosomal protein HMG-1	IPI00418184	-1.246	0.02967	6	5	3	3
2356	Putative deoxyribose-phosphate aldolase	IPI00219677	-1.246	0.02967	4	7	3	3
2357	Ras-related protein Rab-11B	IPI00020436	-1.252	0.02924	18	16	12	14
2358	Heterogeneous nuclear ribonucleoprotein H	IPI00013881	-1.255	0.02877	42	35	27	39
2359	Serine/threonine-protein kinase PAK 2	IPI00419979	-1.259	0.02872	9	15	10	7
2360	Isoform 2 of Eukaryotic translation initiation factor 5A-1	IPI00376005	-1.265	0.02834	47	48	44	39
2361	59 kDa protein	IPI00302925	-1.270	0.02830	45	49	41	41
2362	Thymidylate synthetase, isoform CRA_a	IPI00103732	-1.276	0.02816	7	9	5	5
2363	Isoform 2 of Histone deacetylase 2	IPI00289601	-1.276	0.02816	8	8	5	5
2364	14-3-3 protein eta	IPI00216319	-1.276	0.02816	8	8	5	5
2365	tRNA methyltransferase 112 homolog	IPI00009010	-1.276	0.02816	7	9	6	4

No.	Description	Accession number	STN[1]	p-Value[1]	Con_A[2]	Con_B[2]	CUR_A[2]	CUR_B[2]
2366	Actin, cytoplasmic 1	IPI00021439	-1.280	0.02688	215	199	203	190
2367	cDNA FLJ60124, highly similar to Mitochondrial dicarboxylate carrier	IPI00005537	-1.283	0.02688	11	12	8	8
2368	40S ribosomal protein S18	IPI00013296	-1.284	0.02688	18	14	10	14
2369	Serpin H1	IPI00032140	-1.284	0.02688	16	16	9	15
2370	Eukaryotic translation initiation factor 3 subunit K	IPI00033143	-1.299	0.02622	22	20	17	16
2371	THO complex subunit 4	IPI00328840	-1.300	0.02622	27	28	23	22
2372	RER1 protein	IPI00005728	-1.307	0.02603	6	4	2	3
2373	protein ELYS	IPI00170594	-1.308	0.02603	10	12	7	8
2374	60S ribosomal protein L27a	IPI00456758	-1.308	0.02603	11	11	7	8
2375	Glutaredoxin-3	IPI00008552	-1.308	0.02603	11	11	8	7
2376	ATP-citrate synthase	IPI00021290	-1.315	0.02594	42	44	40	34
2377	regulator of chromosome condensation 1 isoform a	IPI00001661	-1.315	0.02575	9	6	5	4
2378	Endoplasmic reticulum metallopeptidase 1	IPI00257903	-1.315	0.02575	7	8	5	4
2379	Isoform 3 of Fermitin family homolog 1	IPI00220602	-1.315	0.02575	7	8	5	4
2380	Isoform 1 of Carnitine O-palmitoyltransferase 1, liver isoform	IPI00032038	-1.319	0.02575	15	15	11	11
2381	SDHA protein	IPI00217143	-1.319	0.02575	15	15	13	9
2382	Isoform 2 of 4F2 cell-surface antigen heavy chain	IPI00027493	-1.326	0.02556	35	32	28	28
2383	Condensin-2 complex subunit D3	IPI00747787	-1.335	0.02523	10	11	7	7
2384	regulator of differentiation 1 isoform 2	IPI00159072	-1.335	0.02523	11	10	6	8
2385	Isoform 1 of ATPase family AAA domain-containing protein 2	IPI00170548	-1.338	0.02523	2	4	1	1
2386	Isoform 1 of Serine/threonine-protein kinase ATR	IPI00412298	-1.338	0.02523	2	4	1	1
2387	Isoform 1 of Vacuolar protein sorting-associated protein 8 homolog	IPI00464985	-1.338	0.02523	4	2	1	1
2388	39S ribosomal protein L23, mitochondrial	IPI00293476	-1.338	0.02523	3	3	1	1
2389	Origin recognition complex subunit 4	IPI00015164	-1.338	0.02523	2	4	1	1
2390	AP-3 complex subunit mu-1	IPI00032459	-1.338	0.02523	3	3	0	1
2391	Protein FAM98A	IPI00174442	-1.338	0.02523	3	3	1	0
2392	Isoform 2 of Glutaminase kidney isoform, mitochondrial	IPI00215685	-1.338	0.02523	4	2	0	0
2393	Isoform Beta-4C of Integrin beta-4	IPI00027422	-1.339	0.02064	16	13	10	11
2394	Putative uncharacterized protein ATP5J2	IPI00219291	-1.339	0.02064	14	15	11	10
2395	T-complex protein 1 subunit gamma isoform b	IPI00290770	-1.345	0.02064	62	60	53	55
2396	T-complex protein 1 subunit zeta	IPI00027626	-1.347	0.02064	101	103	89	98
2397	Peptidyl-prolyl cis-trans isomerase FKBP4	IPI00219005	-1.349	0.02064	63	58	56	51
2398	Isoform Short of RNA-binding protein FUS	IPI00221354	-1.359	0.02017	12	16	10	10
2399	Isoform 1 of 14-3-3 protein sigma	IPI00013890	-1.359	0.01984	6	8	3	5
2400	Isoform 1 of DNA-binding protein A	IPI00031801	-1.359	0.01984	7	7	4	4
2401	DNA-directed RNA polymerase II subunit RPB7	IPI00218895	-1.359	0.01984	8	6	4	4
2402	Probable ATP-dependent RNA helicase DDX27	IPI00293078	-1.359	0.01984	7	7	4	4
2403	Transducin beta-like protein 3	IPI00477971	-1.380	0.01852	12	15	9	10
2404	Putative RNA-binding protein 3	IPI00024320	-1.380	0.01852	15	12	9	10
2405	WD repeat-containing protein 3	IPI00009471	-1.383	0.01852	4	5	1	3
2406	Protein S100-A6	IPI00027463	-1.383	0.01852	4	5	3	1
2407	Ethanolamine-phosphate cytidylyltransferase	IPI00015285	-1.383	0.01852	4	5	2	2
2408	40S ribosomal protein S14	IPI00026271	-1.384	0.01838	19	17	13	14
2409	ERO1-like protein alpha	IPI00386755	-1.397	0.01809	9	10	6	6
2410	Isoform 2 of Filamin-A	IPI00302592	-1.399	0.01800	153	133	137	129
2411	Dolichyl-diphosphooligosaccharide--protein glycosyltransferase 48 kDa subunit	IPI00297084	-1.401	0.01795	19	16	13	13
2412	Glucose-6-phosphate isomerase	IPI00027497	-1.405	0.01776	59	50	48	47
2413	Isoform 1 of Heterogeneous nuclear ribonucleoprotein D0	IPI00028888	-1.405	0.01776	29	29	24	23
2414	NADH-ubiquinone oxidoreductase chain 4	IPI00008495	-1.410	0.01776	7	6	3	4
2415	UPF0553 protein C9orf64	IPI00170972	-1.410	0.01776	7	6	4	3
2416	Cell division protein kinase 6	IPI00023529	-1.410	0.01776	7	6	4	3
2417	NADH dehydrogenase [ubiquinone] iron-sulfur protein 2, mitochondrial	IPI00025239	-1.410	0.01776	8	5	2	5
2418	Protein phosphatase 1G	IPI00006167	-1.418	0.01767	18	16	13	12
2419	Isoform 1 of Cleavage stimulation factor subunit 2	IPI00013256	-1.432	0.01743	9	9	7	4
2420	cDNA FLJ51909, highly similar to Serine-threonine kinase receptor-associatedprotein	IPI00294536	-1.432	0.01743	9	9	4	7
2421	Isoform 1 of Cleft lip and palate transmembrane protein 1-like protein	IPI00151358	-1.432	0.01743	11	7	5	6
2422	Isoform M2 of Pyruvate kinase isozymes M1/M2	IPI00479186	-1.434	0.01743	117	116	110	104
2423	Isoform Long of Ubiquitin carboxyl-terminal hydrolase 5	IPI00024664	-1.436	0.01743	16	17	11	13
2424	Replication factor C subunit 5	IPI00031514	-1.436	0.01743	17	16	9	15
2425	Superkiller viralicidic activity 2-like 2	IPI00647217	-1.436	0.01743	22	21	19	14
2426	Isoform 1 of L-lactate dehydrogenase A chain	IPI00217966	-1.438	0.01743	249	237	218	243
2427	FACT complex subunit SPT16	IPI00026970	-1.441	0.01743	89	82	79	75
2428	T-complex protein 1 subunit epsilon	IPI00010720	-1.447	0.01724	50	51	42	45
2429	Cleavage and polyadenylation specificity factor subunit 5	IPI00646917	-1.451	0.01724	21	21	15	17
2430	arylacetamide deacetylase-like 1 isoform b	IPI00002230	-1.451	0.01724	20	22	15	17
2431	Dihydrolipoyllysine-residue acetyltransferase component of pyruvate dehydrogenase complex	IPI00021338	-1.465	0.01663	20	21	15	16
2432	U6 snRNA-associated Sm-like protein LSm7	IPI00007163	-1.469	0.01653	6	6	3	3
2433	Keratin, type I cytoskeletal 16	IPI00217963	-1.469	0.01653	6	6	3	3
2434	Lamin-B1	IPI00217975	-1.470	0.01531	49	48	43	40
2435	Isoform 1 of Catenin alpha-1	IPI00215948	-1.471	0.01526	8	9	6	4
2436	Succinyl-CoA ligase [GDP-forming] subunit beta, mitochondrial	IPI00096066	-1.471	0.01526	11	6	4	6
2437	Isoform 1 of Transformation/transcription domain-associated protein	IPI00069084	-1.480	0.01512	18	22	17	13
2438	DNA-directed RNA polymerase I subunit RPA1	IPI00031960	-1.481	0.01493	4	4	2	1
2439	28S ribosomal protein S22, mitochondrial	IPI00013146	-1.481	0.01493	4	4	1	2
2440	28S ribosomal protein S7, mitochondrial	IPI00006440	-1.481	0.01493	3	5	1	2
2441	Eukaryotic translation initiation factor 3 subunit D	IPI00006181	-1.496	0.01483	14	16	10	11
2442	60S ribosomal protein L7	IPI00030179	-1.496	0.01483	17	22	15	14
2443	40S ribosomal protein S4, X isoform	IPI00217030	-1.504	0.01455	24	25	16	22
2444	Isoform 1 of Deoxyuridine 5'-triphosphate nucleotidohydrolase, mitochondrial	IPI00013679	-1.504	0.01455	26	23	20	18
2445	Histone deacetylase 1	IPI00013774	-1.512	0.01455	9	13	7	7
2446	Isoform 1 of Methionine adenosyltransferase 2 subunit beta	IPI00002324	-1.512	0.01455	12	10	7	7
2447	Putative uncharacterized protein KIAA0664	IPI00024425	-1.513	0.01403	18	20	14	14
2448	Collapsin response mediator protein 4 long variant	IPI00029111	-1.517	0.01384	24	24	22	15
2449	T-complex protein 1 subunit eta	IPI00018465	-1.523	0.01379	101	98	85	95
2450	Heterogeneous nuclear ribonucleoprotein F	IPI00003881	-1.527	0.01379	41	47	36	38
2451	Isoform alpha-enolase of Alpha-enolase	IPI00465248	-1.538	0.01370	271	281	218	306
2452	Isoform 1 of Fermitin family homolog 2	IPI00000856	-1.538	0.01361	5	6	2	3
2453	Isoform 1 of Fanconi anemia group D2 protein	IPI00075081	-1.538	0.01361	5	6	3	2
2454	Tyrosine-protein phosphatase non-receptor type 1	IPI00297261	-1.538	0.01361	5	6	0	4
2455	14-3-3 protein zeta/delta	IPI00021263	-1.542	0.01361	61	61	52	54
2456	Sodium/potassium-transporting ATPase subunit beta-3	IPI00008167	-1.542	0.01361	16	12	9	10
2457	Importin-9	IPI00185146	-1.545	0.01361	11	10	7	6
2458	Calpain small subunit 1	IPI00025084	-1.545	0.01361	10	11	6	7
2459	Tumor protein, translationally-controlled 1	IPI00009943	-1.548	0.01361	18	18	14	12
2460	Importin-7	IPI00007402	-1.563	0.01304	41	42	36	33

No.	Description	Accession number	STN[1]	p-Value[1]	Con_A[2]	Con_B[2]	CUR_A[2]	CUR_B[2]
2461	Casein kinase II subunit alpha'	IPI00020602	-1.564	0.01304	8	7	6	2
2462	Heat shock protein beta-1	IPI00025512	-1.577	0.01304	33	34	26	28
2463	Isoform 2 of Tyrosine-protein phosphatase non-receptor type 11	IPI00298347	-1.580	0.01304	9	11	6	6
2464	482 kDa protein	IPI00179298	-1.582	0.01285	78	78	68	70
2465	Isoform 1 of Clathrin heavy chain 1	IPI00024067	-1.588	0.01261	235	224	216	216
2466	Ras-related protein Rab-5C	IPI00016339	-1.589	0.01261	22	21	15	17
2467	40S ribosomal protein S19	IPI00215780	-1.604	0.01261	20	22	16	15
2468	Isoform 2 of Cleft lip and palate transmembrane protein 1	IPI00107357	-1.617	0.01228	3	4	1	1
2469	Dihydrofolate reductase	IPI00030357	-1.617	0.01228	5	2	0	0
2470	ribonucleotide reductase M2 polypeptide isoform 1	IPI00011118	-1.617	0.01228	3	4	0	0
2471	Protein naked cuticle homolog 1	IPI00056339	-1.617	0.01228	3	4	1	1
2472	Isoform 2 of 3-hydroxyisobutyryl-CoA hydrolase, mitochondrial	IPI00377161	-1.617	0.01228	3	4	1	1
2473	cDNA FLJ54752, highly similar to Poly(rC)-binding protein 2	IPI00788837	-1.617	0.01228	3	4	1	1
2474	ATP-dependent Clp protease ATP-binding subunit clpX-like, mitochondrial	IPI00008728	-1.619	0.01209	10	9	7	4
2475	cAMP-dependent protein kinase type I-alpha regulatory subunit	IPI00021831	-1.619	0.01209	8	11	5	6
2476	Replication factor C subunit 4	IPI00017381	-1.619	0.01209	9	10	6	5
2477	Synaptobrevin homolog YKT6	IPI00008569	-1.620	0.01209	6	8	4	3
2478	Deoxyhypusine hydroxylase	IPI00171856	-1.620	0.01209	6	8	4	3
2479	Isoform 1 of Fanconi anemia group I protein	IPI00019447	-1.622	0.01209	12	13	7	9
2480	Isoform 1 of Telomeric repeat-binding factor 2	IPI00024214	-1.624	0.01209	5	5	2	2
2481	Estradiol 17-beta-dehydrogenase 11	IPI00329598	-1.638	0.01002	23	17	14	15
2482	40S ribosomal protein S6	IPI00021840	-1.638	0.01002	20	20	15	14
2483	Isoform 1 of Heterogeneous nuclear ribonucleoprotein Q	IPI00018140	-1.646	0.01002	58	63	51	53
2484	Isoform 1 of Protein SET	IPI00072377	-1.648	0.01002	40	47	36	36
2485	Isoform 1 of Splicing factor U2AF 65 kDa subunit	IPI00031556	-1.649	0.01002	22	27	19	18
2486	Anaphase-promoting complex subunit 1	IPI00033907	-1.662	0.01002	12	6	5	5
2487	Calponin-2	IPI00015262	-1.662	0.01002	8	10	6	4
2488	Thymidine kinase, cytosolic	IPI00299214	-1.685	0.00954	6	7	4	2
2489	Kinetochore-associated protein 1	IPI00001458	-1.685	0.00954	7	6	3	3
2490	Calponin-3	IPI00216682	-1.685	0.00954	5	8	3	3
2491	Isoform CNPI of 2',3'-cyclic-nucleotide 3'-phosphodiesterase	IPI00220993	-1.685	0.00954	6	7	2	4
2492	Ran GTPase-activating protein 1	IPI00294879	-1.686	0.00954	10	13	6	8
2493	40S ribosomal protein S2	IPI00013485	-1.697	0.00926	59	53	50	45
2494	Eukaryotic initiation factor 4A-III	IPI00009328	-1.697	0.00926	29	27	23	20
2495	Isoform 2 of Structural maintenance of chromosomes protein 4	IPI00328298	-1.697	0.00926	31	25	21	22
2496	Nucleolar protein 58	IPI00006379	-1.714	0.00912	33	33	29	23
2497	Small nuclear ribonucleoprotein Sm D1	IPI00302850	-1.721	0.00893	55	53	40	51
2498	UPF0027 protein C22orf28	IPI00550689	-1.722	0.00893	11	11	6	7
2499	Alkyldihydroxyacetonephosphate synthase, peroxisomal	IPI00010349	-1.722	0.00893	12	10	7	6
2500	Neprilysin	IPI00247063	-1.733	0.00869	4	5	0	2
2501	Protein SGT1	IPI00027034	-1.733	0.00869	4	5	1	2
2502	Prefoldin subunit 5	IPI00015361	-1.733	0.00869	5	4	2	1
2503	Isoform 1 of Thyroid receptor-interacting protein 13	IPI00003505	-1.733	0.00869	5	4	1	2
2504	Isoform 1 of DNA replication licensing factor MCM7	IPI00299904	-1.758	0.00855	18	16	12	11
2505	DnaJ homolog subfamily A member 2	IPI00032406	-1.758	0.00855	14	13	6	11
2506	Isoform 1 of Cirhin	IPI00239815	-1.761	0.00855	11	10	6	6
2507	Isoform 1 of Proteasome activator complex subunit 3	IPI00030243	-1.764	0.00827	25	26	21	17
2508	Isoform 1 of ATP-dependent RNA helicase DDX19B	IPI00008943	-1.778	0.00827	21	20	15	14
2509	Isoform 2 of Obg-like ATPase 1	IPI00216105	-1.803	0.00784	10	10	6	5
2510	Importin subunit alpha-2	IPI00002214	-1.806	0.00784	15	17	13	8
2511	39S ribosomal protein L44, mitochondrial	IPI00009680	-1.826	0.00784	8	7	3	4
2512	Isoform 2 of Splicing factor 3B subunit 3	IPI00179138	-1.838	0.00784	19	19	12	14
2513	Isoform 1 of Polypyrimidine tract-binding protein 1	IPI00179964	-1.854	0.00779	89	87	82	72
2514	Hydroxymethylglutaryl-CoA synthase, cytoplasmic	IPI00008475	-1.857	0.00779	6	5	2	2
2515	60S acidic ribosomal protein P0	IPI00008530	-1.861	0.00775	38	38	29	31
2516	Isoform 2 of Neutral alpha-glucosidase AB	IPI00011454	-1.881	0.00751	79	71	61	68
2517	Small glutamine-rich tetratricopeptide repeat-containing protein alpha	IPI00013949	-1.884	0.00751	4	4	1	1
2518	Transcription initiation factor IIB	IPI00022820	-1.884	0.00751	3	5	1	1
2519	Histone H1.2	IPI00217465	-1.885	0.00647	42	44	35	34
2520	28S ribosomal protein S23, mitochondrial	IPI00032881	-1.897	0.00647	6	8	0	5
2521	Deoxyribonucleoside 5'-monophosphate N-glycosidase	IPI00007926	-1.902	0.00605	10	8	5	4
2522	40S ribosomal protein S15a	IPI00221091	-1.904	0.00605	36	25	21	25
2523	Eukaryotic translation initiation factor 3 subunit E	IPI00013068	-1.907	0.00605	17	18	11	12
2524	40S ribosomal protein S15	IPI00479058	-1.913	0.00605	40	43	32	34
2525	Nascent polypeptide-associated complex subunit alpha	IPI00023748	-1.917	0.00605	27	33	24	21
2526	Pre-mRNA-splicing factor ATP-dependent RNA helicase PRP16	IPI00294211	-1.932	0.00600	17	17	11	11
2527	Chromobox protein homolog 1	IPI00010320	-1.956	0.00572	46	45	36	37
2528	DNA mismatch repair protein Msh2	IPI00017303	-1.958	0.00572	17	16	12	9
2529	E3 ubiquitin/ISG15 ligase TRIM25	IPI00029629	-1.961	0.00572	9	8	4	4
2530	Isoform 1 of KDEL motif-containing protein 2	IPI00143921	-1.976	0.00572	4	6	2	0
2531	Neutral amino acid transporter B(0)	IPI00019472	-1.979	0.00572	74	75	62	65
2532	protein transport protein Sec61 subunit alpha isoform 2 isoform b	IPI00182313	-1.981	0.00572	6	7	1	4
2533	Isoform 1 of Telomere-associated protein RIF1	IPI00293845	-1.981	0.00572	5	8	3	2
2534	ATP-dependent RNA helicase A	IPI00844578	-1.991	0.00567	187	178	171	163
2535	DNA damage-binding protein 1	IPI00293464	-2.028	0.00557	35	37	29	26
2536	cDNA FLJ55599, highly similar to DNA replication licensing factor MCM3	IPI00013214	-2.056	0.00543	23	20	14	15
2537	T-complex protein 1 subunit delta	IPI00302927	-2.075	0.00543	54	63	51	45
2538	71 kDa protein	IPI00062599	-2.083	0.00543	6	6	2	2
2539	Bifunctional ATP-dependent dihydroxyacetone kinase/FAD-AMP lyase (cyclizing)	IPI00551024	-2.091	0.00510	24	25	16	18
2540	Coatomer subunit beta'	IPI00220219	-2.115	0.00510	29	27	21	19
2541	Ataxin-10	IPI00001636	-2.115	0.00505	14	14	5	11
2542	Isoform 1 of Myosin-9	IPI00019502	-2.116	0.00505	380	370	352	355
2543	Isoform 1 of Transformer-2 protein homolog beta	IPI00301503	-2.129	0.00505	25	22	15	17
2544	Isoform 1 of Uridine-cytidine kinase 2	IPI00065671	-2.140	0.00496	5	4	1	1
2545	Ribosomal protein S27	IPI00514399	-2.140	0.00496	5	4	0	1
2546	Isoform 1 of Far upstream element-binding protein 3	IPI00377261	-2.140	0.00496	5	4	1	1
2547	Isoform Long of Splicing factor, proline- and glutamine-rich	IPI00010740	-2.148	0.00491	24	30	20	18
2548	Leucine-rich PPR motif-containing protein, mitochondrial	IPI00783271	-2.159	0.00449	117	108	98	99
2549	Isoform 3 of Glutaminase kidney isoform, mitochondrial	IPI00215687	-2.162	0.00449	12	10	6	5
2550	DNA replication licensing factor MCM2	IPI00184330	-2.166	0.00449	29	24	17	20
2551	Probable ATP-dependent RNA helicase DDX5	IPI00017617	-2.178	0.00449	61	56	48	47
2552	Transmembrane 9 superfamily member 2	IPI00018415	-2.193	0.00444	7	7	4	1
2553	40S ribosomal protein S3	IPI00011253	-2.208	0.00430	86	107	65	101
2554	cDNA: FLJ22728 fis, clone HSI15617 (Fragment)	IPI00386139	-2.211	0.00430	5	6	1	2
2555	Isoform 1 of Elongation factor 1-delta	IPI00023048	-2.249	0.00416	34	31	26	21

No.	Description	Accession number	STN[1]	p-Value[1]	Con_A[2]	Con_B[2]	CUR_A[2]	CUR_B[2]
2556	Isoform 5 of Glycogen debranching enzyme	IPI00219065	-2.262	0.00416	24	24	15	17
2557	Isoform 1 of Tyrosine-protein kinase BAZ1B	IPI00069817	-2.276	0.00416	12	17	8	8
2558	60 kDa heat shock protein, mitochondrial	IPI00784154	-2.276	0.00416	404	427	380	403
2559	40S ribosomal protein S7	IPI00013415	-2.280	0.00416	44	38	30	32
2560	Isoform 1 of Caprin-1	IPI00783872	-2.285	0.00416	12	12	7	5
2561	SWI/SNF complex subunit SMARCC1	IPI00234252	-2.285	0.00416	12	12	5	7
2562	Gamma-enolase	IPI00216171	-2.286	0.00406	72	58	42	64
2563	Probable ATP-dependent RNA helicase DDX6	IPI00030320	-2.323	0.00354	17	16	8	11
2564	Isoform GTBP-alt of DNA mismatch repair protein Msh6	IPI00106847	-2.331	0.00354	26	26	15	20
2565	Histone acetyltransferase type B catalytic subunit	IPI00024719	-2.342	0.00354	9	10	4	4
2566	40S ribosomal protein S8	IPI00216587	-2.345	0.00354	22	37	16	25
2567	Isoform 1 of Transcription intermediary factor 1-beta	IPI00438229	-2.348	0.00350	53	56	46	40
2568	Isoform 1 of ATP-binding cassette sub-family D member 3	IPI00002372	-2.375	0.00345	17	20	10	12
2569	Keratin, type II cytoskeletal 1	IPI00220327	-2.378	0.00345	333	326	303	310
2570	cDNA FLJ60299, highly similar to Rab GDP dissociation inhibitor beta	IPI00031461	-2.392	0.00326	45	48	33	38
2571	Putative uncharacterized protein ENSP00000350479	IPI00069693	-2.394	0.00326	18	31	10	22
2572	Isoform Long of 14-3-3 protein beta/alpha	IPI00216318	-2.395	0.00317	37	36	28	25
2573	116 kDa U5 small nuclear ribonucleoprotein component	IPI00003519	-2.423	0.00307	52	49	41	37
2574	Isoform 1 of Tensin-3	IPI00658152	-2.450	0.00307	31	30	22	20
2575	Isoform 1 of DNA primase large subunit	IPI00027705	-2.457	0.00307	9	12	5	4
2576	Isoform 1 of 60S ribosomal protein L12	IPI00024933	-2.469	0.00307	36	32	19	29
2577	Ras GTPase-activating protein-binding protein 1	IPI00012442	-2.486	0.00307	30	29	20	20
2578	Dihydropyrimidinase-related protein 2	IPI00257508	-2.515	0.00307	22	22	15	12
2579	Histone H1.5	IPI00217468	-2.518	0.00283	31	34	22	23
2580	Isoform 1 of Coatomer subunit alpha	IPI00295857	-2.523	0.00274	50	52	37	41
2581	Interleukin enhancer-binding factor 2	IPI00005198	-2.593	0.00217	63	54	41	50
2582	DNA polymerase alpha catalytic subunit	IPI00220317	-2.604	0.00217	6	10	3	2
2583	60S ribosomal protein L9	IPI00031691	-2.635	0.00198	51	41	36	32
2584	Guanine nucleotide-binding protein subunit beta-2-like 1	IPI00848226	-2.642	0.00198	75	73	60	59
2585	Mitotic spindle assembly checkpoint protein MAD2A	IPI00012369	-2.662	0.00198	6	7	2	0
2586	Sodium/potassium-transporting ATPase subunit alpha-2	IPI00003021	-2.698	0.00175	21	17	12	9
2587	DNA replication licensing factor MCM4	IPI00018349	-2.702	0.00175	14	19	10	7
2588	HMT1 hnRNP methyltransferase-like 2 isoform 1	IPI00018522	-2.710	0.00170	32	30	24	17
2589	Transmembrane protein 165	IPI00307572	-2.726	0.00161	8	7	3	1
2590	ATP-binding cassette sub-family E member 1	IPI00303207	-2.764	0.00146	46	45	33	33
2591	Eukaryotic translation initiation factor 3, subunit E interacting protein	IPI00465233	-2.765	0.00146	40	34	26	25
2592	Condensin complex subunit 1	IPI00299524	-2.772	0.00146	33	33	21	23
2593	Citrate synthase, mitochondrial	IPI00025366	-2.786	0.00137	21	25	10	17
2594	Isoform 1 of Exportin-2	IPI00022744	-2.831	0.00137	105	113	88	94
2595	Retinoblastoma-associated protein	IPI00302829	-2.858	0.00137	6	6	1	1
2596	Isoform 1 of Zinc transporter ZIP14	IPI00014236	-2.858	0.00137	6	6	1	0
2597	Isoform 1 of Transcription factor BTF3	IPI00221035	-2.862	0.00123	28	27	19	15
2598	Isoform 1 of Eukaryotic translation initiation factor 3 subunit B	IPI00396370	-2.872	0.00123	31	30	17	22
2599	Glyceraldehyde-3-phosphate dehydrogenase	IPI00219018	-2.873	0.00123	657	679	611	653
2600	Isoform 1 of Heat shock cognate 71 kDa protein	IPI00003865	-2.912	0.00113	215	218	196	189
2601	Isoform 1 of Chromodomain-helicase-DNA-binding protein 4	IPI00000846	-3.004	0.00109	70	69	54	53
2602	Proliferation-associated protein 2G4	IPI00299000	-3.013	0.00109	26	29	15	18
2603	CAD protein	IPI00301263	-3.029	0.00109	121	121	95	107
2604	6-phosphogluconate dehydrogenase, decarboxylating	IPI00219525	-3.049	0.00104	37	43	21	33
2605	DNA replication licensing factor MCM6	IPI00031517	-3.087	0.00094	20	22	10	12
2606	Ribonucleoside-diphosphate reductase large subunit	IPI00013871	-3.101	0.00094	15	18	8	7
2607	40S ribosomal protein S10	IPI00008438	-3.140	0.00094	46	79	42	51
2608	Elongation factor 1-alpha	IPI00025447	-3.150	0.00090	127	122	101	106
2609	Calreticulin	IPI00020599	-3.191	0.00085	61	69	54	43
2610	Isoform 1 of Heterogeneous nuclear ribonucleoprotein K	IPI00216049	-3.194	0.00085	146	142	118	125
2611	Isoform 1 of Importin-5	IPI00793443	-3.199	0.00085	48	38	32	26
2612	Keratin, type I cytoskeletal 19	IPI00479145	-3.267	0.00076	134	138	108	119
2613	DNA replication licensing factor MCM5	IPI00018350	-3.351	0.00066	24	25	14	12
2614	Non-POU domain-containing octamer-binding protein	IPI00304596	-3.355	0.00066	84	70	61	56
2615	DnaJ homolog subfamily A member 1	IPI00012535	-3.403	0.00066	19	16	4	11
2616	Protein disulfide-isomerase A3	IPI00025252	-3.412	0.00066	94	98	79	72
2617	Proliferating cell nuclear antigen	IPI00021700	-3.490	0.00043	66	56	45	42
2618	Isoform 1 of Cytosolic acyl coenzyme A thioester hydrolase	IPI00010415	-3.501	0.00043	23	22	13	9
2619	Putative uncharacterized protein DKFZp686L20222	IPI00026689	-3.514	0.00043	36	34	22	20
2620	Isoform 2 of U5 small nuclear ribonucleoprotein 200 kDa helicase	IPI00168235	-3.604	0.00043	62	68	46	47
2621	Histone H4	IPI00453473	-3.660	0.00043	369	387	334	348
2622	Tubulin alpha-4A chain	IPI00007750	-3.871	0.00033	259	263	222	232
2623	Isoform Long of Sodium/potassium-transporting ATPase subunit alpha-1	IPI00006482	-3.919	0.00033	48	46	29	30
2624	Isoform 1 of Acetyl-CoA carboxylase 1	IPI00011569	-3.999	0.00033	33	30	17	16
2625	Isoform 1 of Nucleolar RNA helicase 2	IPI00015953	-4.108	0.00033	68	56	43	40
2626	Isoform A1-B of Heterogeneous nuclear ribonucleoprotein A1	IPI00215965	-4.172	0.00033	101	115	86	78
2627	Keratin, type I cytoskeletal 10	IPI00009865	-4.187	0.00033	171	165	131	143
2628	poly(rC) binding protein 2 isoform b	IPI00012066	-4.264	0.00033	45	39	25	23
2629	Keratin, type II cytoskeletal 8	IPI00554648	-4.401	0.00028	507	508	447	469
2630	Isoform 1 of Splicing factor 3B subunit 3	IPI00300371	-4.444	0.00028	107	107	77	82
2631	Isoform 1 of DNA (cytosine-5)-methyltransferase 1	IPI00031519	-4.512	0.00028	25	22	10	8
2632	Isoform 1 of 60S ribosomal protein L11	IPI00376798	-4.571	0.00028	55	55	36	31
2633	Elongation factor 2	IPI00186290	-4.605	0.00028	150	162	128	118
2634	T-complex protein 1 subunit beta	IPI00297779	-4.624	0.00028	154	143	112	120
2635	Transferrin receptor protein 1	IPI00022462	-4.708	0.00028	28	33	13	14
2636	32 kDa protein	IPI00176692	-4.983	0.00024	293	296	234	264
2637	Isoform M1 of Pyruvate kinase isozymes M1/M2	IPI00220644	-5.217	0.00014	371	336	304	301
2638	Pre-mRNA-processing-splicing factor 8	IPI00007928	-5.342	0.00014	168	176	134	131
2639	Isoform 1 of Solute carrier family 12 member 2	IPI00022649	-5.462	0.00014	25	23	7	7
2640	Isoform 1 of U5 small nuclear ribonucleoprotein 200 kDa helicase	IPI00420014	-5.995	0.00014	214	202	157	164
2641	Isoform Long of Inositol 1,4,5-trisphosphate receptor type 2	IPI00031545	-6.170	0.00009	99	97	64	60
2642	Isoform 1 of Sodium-coupled neutral amino acid transporter 2	IPI00410034	-6.539	0.00005	17	17	0	2
2643	Keratin, type I cytoskeletal 18	IPI00554788	-6.805	0.00005	256	335	176	292
2644	Elongation factor 1-alpha 2	IPI00014424	-10.554	0.00000	419	437	301	339
2645	Eukaryotic initiation factor 4A-I	IPI00025491	-10.766	0.00000	127	140	61	71
2646	Fatty acid synthase	IPI00026781	-11.210	0.00000	367	359	258	254
