# Supplementary material for: Data supporting the identification of anti-metastatic drug and natural compound targets in isogenic colorectal cancer cells
Source: Data Brief. 2014 Nov 4;1:73–5. doi: 10.1016/j.dib.2014.10.005 (PMC4459770; doi:10.1016/j.dib.2014.10.005)
Supplement: Supplementary file 1 — Supplementary data [file mmc1.zip › CRC_Metastasis_DIB_Table07.pdf]

Table 7. List of proteins differentially expressed in SW620 by the treatment of luteolin for 48hr. ([1]STN and p-value were acquired from PLGEM analysis, [2]Raw spectral counts from data compilation using Scaffold[TM] software)

| No. | Description | Accession number | STN[1] | p-Value[1] | Con_A[2] | Con_B[2] | LUTEO_A[2] | LUTEO_B[2] |
|---|---|---|---|---|---|---|---|---|
| 1 | Keratin, type I cytoskeletal 10 | IPI00009865 | 24.593 | 0.00000 | 32 | 25 | 226 | 195 |
| 2 | Keratin, type II cytoskeletal 2 epiderma | IPI00021304 | 14.025 | 0.00000 | 19 | 14 | 102 | 87 |
| 3 | Keratin, type II cytoskeletal 1 | IPI00220327 | 13.206 | 0.00000 | 215 | 204 | 368 | 352 |
| 4 | Keratin, type I cytoskeletal 9 | IPI00019359 | 9.684 | 0.00000 | 28 | 18 | 83 | 70 |
| 5 | Endoplasmin | IPI00027230 | 5.599 | 0.00000 | 62 | 68 | 114 | 95 |
| 6 | Isoform 1 of Nucleolar RNA helicase 2 | IPI00015953 | 5.200 | 0.00000 | 33 | 33 | 60 | 64 |
| 7 | ADP/ATP translocase 2 | IPI00007188 | 4.937 | 0.00000 | 118 | 109 | 139 | 172 |
| 8 | Alanyl-tRNA synthetase, cytoplasmic | IPI00027442 | 4.436 | 0.00005 | 42 | 34 | 65 | 62 |
| 9 | ATP-citrate synthase | IPI00021290 | 4.393 | 0.00005 | 25 | 29 | 50 | 49 |
| 10 | Keratin, type II cytoskeletal 75 | IPI00005859 | 4.364 | 0.00005 | 13 | 12 | 28 | 32 |
| 11 | 60 kDa heat shock protein, mitochondrial | IPI00784154 | 3.907 | 0.00005 | 233 | 250 | 282 | 288 |
| 12 | cDNA FLJ40024 fis, clone STOMA2007745, highly similar to UBIQUITIN-ACTIVATING ENZYME E1 | IPI00026119 | 3.891 | 0.00005 | 38 | 37 | 58 | 61 |
| 13 | Dolichyl-diphosphooligosaccharide--protein glycosyltransferase subunit 1 precursor | IPI00025874 | 3.882 | 0.00005 | 28 | 25 | 44 | 48 |
| 14 | Cytoplasmic dynein 1 heavy chain 1 | IPI00456969 | 3.842 | 0.00014 | 84 | 94 | 117 | 120 |
| 15 | 59 kDa protein | IPI00302925 | 3.826 | 0.00014 | 35 | 39 | 58 | 59 |
| 16 | ADP-ribosylation factor 1 | IPI00215914 | 3.763 | 0.00014 | 87 | 85 | 109 | 120 |
| 17 | Dolichyl-diphosphooligosaccharide--protein glycosyltransferase subunit 2 | IPI00028635 | 3.631 | 0.00014 | 38 | 38 | 63 | 54 |
| 18 | Exportin-1 | IPI00298961 | 3.611 | 0.00014 | 35 | 37 | 54 | 58 |
| 19 | Protein disulfide-isomerase | IPI00010796 | 3.595 | 0.00014 | 34 | 39 | 61 | 52 |
| 20 | Transmembrane emp24 domain-containing protein 10 | IPI00028055 | 3.349 | 0.00033 | 27 | 30 | 46 | 45 |
| 21 | Importin subunit beta-1 | IPI00001639 | 3.176 | 0.00047 | 48 | 51 | 70 | 68 |
| 22 | Isoform 1 of 5'-3' exoribonuclease 2 | IPI00100151 | 3.135 | 0.00052 | 9 | 10 | 21 | 20 |
| 23 | Staphylococcal nuclease domain-containing protein 1 | IPI00140420 | 3.066 | 0.00052 | 29 | 34 | 45 | 50 |
| 24 | Transferrin receptor protein 1 | IPI00022462 | 3.051 | 0.00052 | 31 | 33 | 46 | 50 |
| 25 | Phosphoserine aminotransferase | IPI00001734 | 3.009 | 0.00052 | 12 | 10 | 26 | 18 |
| 26 | Prenylcysteine oxidase 1 | IPI00384280 | 2.912 | 0.00057 | 1 | 1 | 7 | 6 |
| 27 | Protein disulfide-isomerase A4 | IPI00009904 | 2.868 | 0.00066 | 46 | 53 | 68 | 66 |
| 28 | Isoform Short of Heterogeneous nuclear ribonucleoprotein U | IPI00479217 | 2.845 | 0.00066 | 60 | 58 | 86 | 69 |
| 29 | Ubiquitin-like modifier-activating enzyme 1 | IPI00645078 | 2.835 | 0.00066 | 42 | 45 | 61 | 59 |
| 30 | Leucine-rich PPR motif-containing protein, mitochondrial | IPI00783271 | 2.824 | 0.00066 | 118 | 99 | 137 | 126 |
| 31 | Isoform 2 of Exosome complex exonuclease RRP44 | IPI00183462 | 2.738 | 0.00066 | 5 | 4 | 11 | 13 |
| 32 | cDNA FLJ25678 fis, clone TST04067, highly similar to PURINE NUCLEOSIDE PHOSPHORYLASE | IPI00017672 | 2.720 | 0.00066 | 20 | 20 | 31 | 33 |
| 33 | C-1-tetrahydrofolate synthase, cytoplasmic | IPI00218342 | 2.694 | 0.00090 | 35 | 29 | 46 | 46 |
| 34 | Isoform 4 of E3 ubiquitin-protein ligase UBR4 | IPI00640981 | 2.675 | 0.00090 | 3 | 3 | 9 | 10 |
| 35 | Ras-related protein Rab-10 | IPI00016513 | 2.587 | 0.00090 | 11 | 9 | 22 | 16 |
| 36 | Isoform 1 of Importin-4 | IPI00156374 | 2.573 | 0.00090 | 14 | 10 | 28 | 15 |
| 37 | Isoform 1 of DNA-dependent protein kinase catalytic subunit | IPI00296337 | 2.561 | 0.00090 | 76 | 83 | 102 | 94 |
| 38 | Translational activator GCN1 | IPI00001159 | 2.536 | 0.00100 | 88 | 100 | 113 | 114 |
| 39 | Putative uncharacterized protein NOP2 | IPI00294891 | 2.496 | 0.00109 | 4 | 4 | 10 | 11 |
| 40 | Isoform 1 of Rab3 GTPase-activating protein non-catalytic subunit | IPI00554590 | 2.491 | 0.00109 | 1 | 0 | 7 | 4 |
| 41 | Importin-7 | IPI00007402 | 2.483 | 0.00109 | 27 | 26 | 42 | 35 |
| 42 | Isoform 1 of Clathrin heavy chain 2 | IPI00022881 | 2.434 | 0.00109 | 52 | 43 | 63 | 61 |
| 43 | Coatomer subunit gamma | IPI00783982 | 2.357 | 0.00147 | 12 | 11 | 20 | 20 |
| 44 | cDNA FLJ59739, highly similar to Protein transport protein Sec61 subunit alpha isoform 1 | IPI00218466 | 2.327 | 0.00161 | 9 | 11 | 20 | 16 |
| 45 | Isoform Long of Glucose-6-phosphate 1-dehydrogenase | IPI00216008 | 2.326 | 0.00161 | 12 | 12 | 23 | 18 |
| 46 | Isoform 1 of Myosin-9 | IPI00019502 | 2.325 | 0.00161 | 222 | 235 | 260 | 247 |
| 47 | Signal recognition particle receptor subunit alpha | IPI00385267 | 2.302 | 0.00171 | 1 | 2 | 7 | 5 |
| 48 | ATP synthase subunit alpha, mitochondrial | IPI00440493 | 2.289 | 0.00180 | 81 | 79 | 100 | 93 |
| 49 | Poly [ADP-ribose] polymerase 1 | IPI00449049 | 2.284 | 0.00180 | 38 | 38 | 50 | 51 |
| 50 | Anaphase-promoting complex subunit 1 | IPI00033907 | 2.270 | 0.00185 | 1 | 1 | 0 | 9 |
| 51 | Isoform 1 of Lipopolysaccharide-responsive and beige-like anchor protein | IPI00002255 | 2.270 | 0.00185 | 1 | 0 | 5 | 5 |
| 52 | Ephrin type-A receptor 2 | IPI00021267 | 2.270 | 0.00185 | 1 | 1 | 5 | 5 |
| 53 | Isoform 1 of Ras-related protein Rab-1A | IPI00005719 | 2.234 | 0.00233 | 27 | 23 | 37 | 34 |
| 54 | Protein disulfide-isomerase A3 | IPI00025252 | 2.228 | 0.00233 | 62 | 60 | 80 | 71 |
| 55 | cDNA FLJ55574, highly similar to Calnexin | IPI00020984 | 2.221 | 0.00233 | 58 | 54 | 75 | 65 |
| 56 | Isoform 1 of Tryptophanyl-tRNA synthetase, cytoplasmic | IPI00295400 | 2.195 | 0.00261 | 13 | 7 | 18 | 17 |
| 57 | HEAT repeat-containing protein 1 | IPI00024279 | 2.140 | 0.00275 | 17 | 26 | 30 | 32 |
| 58 | proteasome 26S non-ATPase subunit 8 | IPI00010201 | 2.103 | 0.00285 | 8 | 7 | 15 | 13 |
| 59 | Structural maintenance of chromosomes protein 3 | IPI00219420 | 2.101 | 0.00285 | 26 | 13 | 29 | 28 |
| 60 | Isoform 1 of Hexokinase-1 | IPI00018246 | 2.088 | 0.00285 | 6 | 6 | 13 | 11 |
| 61 | Stress-70 protein, mitochondrial | IPI00007765 | 2.063 | 0.00290 | 77 | 74 | 97 | 83 |
| 62 | Isoform 5 of E3 ubiquitin-protein ligase UBR4 | IPI00180305 | 2.051 | 0.00299 | 2 | 3 | 9 | 5 |
| 63 | Periodic tryptophan protein 2 homolog | IPI00300078 | 2.051 | 0.00299 | 3 | 2 | 7 | 7 |
| 64 | Cleavage and polyadenylation specificity factor subunit 1 | IPI00026219 | 2.051 | 0.00299 | 2 | 3 | 5 | 9 |
| 65 | Isoform 4 of Tubulin-specific chaperone D | IPI00030774 | 2.041 | 0.00299 | 0 | 1 | 4 | 5 |
| 66 | Cullin-5 | IPI00216003 | 2.041 | 0.00299 | 1 | 1 | 4 | 5 |
| 67 | Signal recognition particle receptor subunit beta | IPI00295098 | 2.031 | 0.00299 | 12 | 9 | 15 | 20 |
| 68 | DNA replication licensing factor MCM2 | IPI00184330 | 2.027 | 0.00299 | 18 | 19 | 28 | 26 |
| 69 | Isoform 1 of ATP-binding cassette sub-family D member 3 | IPI00002372 | 2.026 | 0.00299 | 8 | 9 | 14 | 16 |
| 70 | Importin-9 | IPI00185146 | 1.990 | 0.00309 | 2 | 6 | 10 | 8 |
| 71 | HSPA5 protein | IPI00003362 | 1.985 | 0.00337 | 77 | 76 | 88 | 93 |
| 72 | Heat shock 70 kDa protein 4 | IPI00002966 | 1.981 | 0.00337 | 26 | 28 | 40 | 33 |
| 73 | ATP synthase subunit b, mitochondrial | IPI00029133 | 1.974 | 0.00337 | 12 | 11 | 18 | 19 |
| 74 | Plastin-2 | IPI00010471 | 1.963 | 0.00337 | 88 | 85 | 106 | 96 |
| 75 | Asparagine synthetase [glutamine-hydrolyzing] | IPI00554777 | 1.960 | 0.00337 | 3 | 3 | 10 | 5 |
| 76 | Isoform 1 of Surfeit locus protein 4 | IPI00005737 | 1.960 | 0.00337 | 4 | 2 | 8 | 7 |
| 77 | Isoform SERCA2A of Sarcoplasmic/endoplasmic reticulum calcium ATPase 2 | IPI00177817 | 1.959 | 0.00337 | 2 | 2 | 5 | 7 |
| 78 | Similar to Protein SAAL1. Isoform 2 | IPI00304935 | 1.959 | 0.00337 | 2 | 2 | 8 | 4 |
| 79 | Isoform Long of Trifunctional purine biosynthetic protein adenosine-3 | IPI00025273 | 1.932 | 0.00408 | 14 | 16 | 26 | 19 |
| 80 | Isoleucyl-tRNA synthetase, cytoplasmic | IPI00644127 | 1.898 | 0.00432 | 22 | 23 | 37 | 25 |
| 81 | Alpha-actinin-1 | IPI00013508 | 1.884 | 0.00432 | 138 | 133 | 155 | 149 |
| 82 | Isoform 2 of Leucyl-cystinyl aminopeptidase | IPI00221240 | 1.882 | 0.00432 | 3 | 4 | 8 | 8 |
| 83 | Isoform 1 of Gamma-tubulin complex component 3 | IPI00033516 | 1.873 | 0.00432 | 1 | 2 | 6 | 4 |
| 84 | Isoform 1 of Pre-mRNA-processing factor 40 homolog A | IPI00337385 | 1.855 | 0.00446 | 2 | 3 | 8 | 5 |
| 85 | Bifunctional purine biosynthesis protein PURH | IPI00289499 | 1.855 | 0.00446 | 36 | 39 | 47 | 48 |
| 86 | N-acetyltransferase 10 | IPI00300127 | 1.851 | 0.00446 | 10 | 8 | 14 | 16 |
| 87 | Asparaginyl-tRNA synthetase, cytoplasmic | IPI00306960 | 1.844 | 0.00461 | 12 | 11 | 20 | 16 |
| 88 | 40S ribosomal protein S9 | IPI00221088 | 1.837 | 0.00461 | 18 | 17 | 26 | 24 |
| 89 | NCL protein | IPI00183526 | 1.812 | 0.00465 | 89 | 89 | 106 | 99 |
| 90 | E3 ubiquitin-protein ligase UBR5 | IPI00026320 | 1.803 | 0.00465 | 0 | 0 | 4 | 4 |
| 91 | Isoform 1 of Ubiquitin carboxyl-terminal hydrolase 15 | IPI00000728 | 1.803 | 0.00465 | 1 | 1 | 4 | 4 |

| No. | Description | Accession number | STN[1] | p-Value[1] | Con_A[2] | Con_B[2] | LUTEO_A[2] | LUTEO_B[2] |
|---|---|---|---|---|---|---|---|---|
| 92 | Isoform 1 of CDP-diacylglycerol--inositol 3-phosphatidyltransferase | IPI00645518 | 1.803 | 0.00465 | 1 | 1 | 4 | 4 |
| 93 | Isoform 1 of Cleavage and polyadenylation specificity factor subunit 6 | IPI00012998 | 1.793 | 0.00617 | 18 | 13 | 24 | 21 |
| 94 | Isoform 1 of Oxysterol-binding protein 1 | IPI00024971 | 1.756 | 0.00726 | 5 | 4 | 11 | 7 |
| 95 | Heat shock 70 kDa protein 4L | IPI00295485 | 1.751 | 0.00736 | 1 | 3 | 5 | 6 |
| 96 | Hypoxia up-regulated protein 1 | IPI00000877 | 1.750 | 0.00736 | 40 | 37 | 48 | 48 |
| 97 | Lon protease homolog, mitochondrial | IPI00005158 | 1.730 | 0.00760 | 17 | 11 | 22 | 19 |
| 98 | Isoform 1 of Nuclear pore complex protein Nup98-Nup96 | IPI00006038 | 1.698 | 0.00764 | 4 | 3 | 9 | 6 |
| 99 | Isoform 1 of Polypyrimidine tract-binding protein 1 | IPI00179964 | 1.668 | 0.00788 | 74 | 72 | 86 | 83 |
| 100 | Isoform 2 of Myosin-Ic | IPI00010418 | 1.656 | 0.00793 | 7 | 4 | 11 | 9 |
| 101 | WD repeat-containing protein 36 | IPI00169325 | 1.654 | 0.00793 | 1 | 4 | 7 | 5 |
| 102 | Cell growth-regulating nucleolar protein | IPI00015838 | 1.654 | 0.00793 | 3 | 2 | 5 | 7 |
| 103 | Isoform 1 of Acetolactate synthase-like protein | IPI00554541 | 1.648 | 0.00793 | 2 | 1 | 7 | 2 |
| 104 | Splicing factor 3B subunit 1 | IPI00026089 | 1.617 | 0.00869 | 39 | 44 | 51 | 50 |
| 105 | Alpha-actinin-4 | IPI00013808 | 1.611 | 0.00869 | 57 | 54 | 69 | 62 |
| 106 | cDNA FLJ45706 fis, clone FEBRA2028457, highly similar to Nucleolin | IPI00444262 | 1.604 | 0.00874 | 44 | 54 | 62 | 55 |
| 107 | Estradiol 17-beta-dehydrogenase 12 | IPI00007676 | 1.587 | 0.00893 | 15 | 14 | 20 | 21 |
| 108 | Isoform 2 of 6-phosphofructokinase, muscle type | IPI00219585 | 1.581 | 0.00897 | 5 | 4 | 11 | 6 |
| 109 | Isoform 2 of 4F2 cell-surface antigen heavy chain | IPI00027493 | 1.580 | 0.00897 | 39 | 37 | 49 | 44 |
| 110 | Dolichol-phosphate mannosyltransferase | IPI00022018 | 1.575 | 0.00907 | 3 | 3 | 7 | 6 |
| 111 | arylacetamide deacetylase-like 1 isoform b | IPI00002230 | 1.575 | 0.00907 | 3 | 3 | 8 | 5 |
| 112 | Glycogen phosphorylase, brain form | IPI00004358 | 1.569 | 0.00907 | 14 | 16 | 20 | 22 |
| 113 | Aspartate aminotransferase, mitochondrial | IPI00018206 | 1.568 | 0.00912 | 25 | 21 | 28 | 32 |
| 114 | Isoform Long of Delta-1-pyrroline-5-carboxylate synthase | IPI00008982 | 1.565 | 0.00926 | 41 | 37 | 46 | 49 |
| 115 | Isoform 2 of Neutral alpha-glucosidase AB | IPI00011454 | 1.565 | 0.00926 | 52 | 53 | 69 | 55 |
| 116 | Inosine-5'-monophosphate dehydrogenase 2 | IPI00291510 | 1.556 | 0.00926 | 23 | 24 | 33 | 28 |
| 117 | Dolichyl-diphosphooligosaccharide--protein glycosyltransferase subunit STT3A | IPI00297492 | 1.553 | 0.00926 | 0 | 1 | 3 | 4 |
| 118 | TATA-binding protein-associated factor 172 | IPI00024802 | 1.553 | 0.00926 | 1 | 0 | 3 | 4 |
| 119 | Isoform 1 of Peroxisomal membrane protein PEX16 | IPI00006722 | 1.553 | 0.00926 | 0 | 0 | 3 | 4 |
| 120 | Derlin-1 | IPI00013271 | 1.553 | 0.00926 | 1 | 0 | 2 | 5 |
| 121 | Ribosomal RNA-processing protein 8 | IPI00304932 | 1.553 | 0.00926 | 1 | 1 | 5 | 2 |
| 122 | cDNA FLJ60939, highly similar to NAD-dependent deacetylase sirtuin-3, mitochondrial | IPI00183171 | 1.553 | 0.00926 | 1 | 1 | 5 | 2 |
| 123 | Signal peptidase complex catalytic subunit SEC11C | IPI00219436 | 1.553 | 0.00926 | 0 | 1 | 5 | 2 |
| 124 | Protein UXT | IPI00002646 | 1.553 | 0.00926 | 1 | 1 | 3 | 4 |
| 125 | Isoform 1 of Beta-2-syntrophin | IPI00009505 | 1.553 | 0.00926 | 0 | 0 | 4 | 3 |
| 126 | Protein transport protein Sec24C | IPI00024661 | 1.539 | 0.00949 | 7 | 7 | 11 | 12 |
| 127 | Lactoylglutathione lyase | IPI00220766 | 1.539 | 0.00949 | 7 | 7 | 13 | 10 |
| 128 | Insulin-like growth factor 2 mRNA-binding protein 1 | IPI00008557 | 1.539 | 0.00949 | 8 | 11 | 12 | 17 |
| 129 | Isoform 1 of Regulator of microtubule dynamics protein 3 | IPI00410079 | 1.535 | 0.00959 | 2 | 2 | 5 | 5 |
| 130 | Isoform 1 of 26S proteasome non-ATPase regulatory subunit 1 | IPI00299608 | 1.533 | 0.01154 | 23 | 26 | 33 | 30 |
| 131 | 116 kDa U5 small nuclear ribonucleoprotein component | IPI00003519 | 1.525 | 0.01206 | 31 | 29 | 36 | 39 |
| 132 | Glycogen phosphorylase, liver form | IPI00783313 | 1.517 | 0.01215 | 12 | 14 | 19 | 18 |
| 133 | ATP-dependent RNA helicase A | IPI00844578 | 1.508 | 0.01215 | 105 | 109 | 126 | 112 |
| 134 | Isoform 1 of Transcription elongation factor SPT6 | IPI00784161 | 1.489 | 0.01215 | 4 | 7 | 10 | 9 |
| 135 | Cytochrome b-c1 complex subunit Rieske, mitochondrial | IPI00026964 | 1.489 | 0.01215 | 7 | 4 | 9 | 10 |
| 136 | Isoform Complexed of Arginyl-tRNA synthetase, cytoplasmic | IPI00004860 | 1.489 | 0.01215 | 20 | 15 | 22 | 25 |
| 137 | Puromycin-sensitive aminopeptidase | IPI00026216 | 1.480 | 0.01215 | 15 | 13 | 18 | 21 |
| 138 | Isoform 2 of Protein disulfide-isomerase A6 | IPI00299571 | 1.475 | 0.01220 | 7 | 9 | 13 | 12 |
| 139 | Glucose-6-phosphate isomerase | IPI00027497 | 1.466 | 0.01253 | 35 | 32 | 42 | 40 |
| 140 | Transmembrane emp24 domain-containing protein 9 | IPI00023542 | 1.452 | 0.01272 | 4 | 4 | 9 | 6 |
| 141 | Probable cysteinyl-tRNA synthetase, mitochondrial | IPI00336016 | 1.450 | 0.01272 | 6 | 6 | 8 | 12 |
| 142 | Isoform 5 of Thioredoxin reductase 1, cytoplasmic | IPI00554786 | 1.450 | 0.01272 | 6 | 6 | 9 | 11 |
| 143 | Cleavage stimulation factor subunit 3 | IPI00015195 | 1.447 | 0.01301 | 9 | 8 | 13 | 13 |
| 144 | Peptidyl-prolyl cis-trans isomerase FKBP3 | IPI00024157 | 1.447 | 0.01301 | 11 | 6 | 14 | 12 |
| 145 | Isoform 1 of Splicing factor U2AF 65 kDa subunit | IPI00031556 | 1.445 | 0.01301 | 15 | 15 | 22 | 19 |
| 146 | Thioredoxin-dependent peroxide reductase, mitochondrial | IPI00024919 | 1.445 | 0.01301 | 15 | 15 | 19 | 22 |
| 147 | Replication protein A 70 kDa DNA-binding subunit | IPI00020127 | 1.445 | 0.01301 | 11 | 12 | 15 | 18 |
| 148 | Cofilin-1 | IPI00012011 | 1.433 | 0.01301 | 137 | 135 | 147 | 150 |
| 149 | Insulin-degrading enzyme | IPI00220373 | 1.414 | 0.01358 | 8 | 5 | 10 | 11 |
| 150 | UDP-glucose 6-dehydrogenase | IPI00031420 | 1.414 | 0.01358 | 18 | 14 | 23 | 20 |
| 151 | Cell division protein kinase 5 | IPI00023530 | 1.412 | 0.01358 | 2 | 1 | 4 | 4 |
| 152 | Isoform 1 of Spermatid perinuclear RNA-binding protein | IPI00169430 | 1.412 | 0.01358 | 2 | 1 | 2 | 6 |
| 153 | cDNA FLJ55475 | IPI00306017 | 1.412 | 0.01358 | 2 | 1 | 6 | 2 |
| 154 | Gigaxonin | IPI00022758 | 1.412 | 0.01358 | 2 | 1 | 3 | 5 |
| 155 | Proteasome subunit alpha type-5 | IPI00291922 | 1.402 | 0.01358 | 5 | 4 | 7 | 9 |
| 156 | Isoform C1 of Heterogeneous nuclear ribonucleoproteins C1/C2 | IPI00216592 | 1.385 | 0.01382 | 55 | 54 | 66 | 60 |
| 157 | Isoform 1 of Dynamin-2 | IPI00033022 | 1.381 | 0.01391 | 6 | 8 | 13 | 9 |
| 158 | 26S proteasome non-ATPase regulatory subunit 2 | IPI00012268 | 1.371 | 0.01609 | 18 | 17 | 28 | 18 |
| 159 | Isoform 1 of ATPase family AAA domain-containing protein 1 | IPI00171445 | 1.358 | 0.01609 | 6 | 4 | 9 | 8 |
| 160 | 6-phosphofructokinase type C | IPI00009790 | 1.353 | 0.01609 | 14 | 14 | 20 | 18 |
| 161 | Isoform 1 of Exportin-2 | IPI00022744 | 1.332 | 0.01633 | 82 | 80 | 96 | 85 |
| 162 | TC4 protein | IPI00044779 | 1.329 | 0.01633 | 11 | 11 | 14 | 17 |
| 163 | Isoform 3 of Glutaminase kidney isoform, mitochondrial | IPI00215687 | 1.318 | 0.01690 | 5 | 6 | 10 | 8 |
| 164 | Protein transport protein Sec23B | IPI00017376 | 1.315 | 0.01690 | 3 | 4 | 8 | 5 |
| 165 | Isoform 2 of U1 small nuclear ribonucleoprotein 70 kDa | IPI00219483 | 1.311 | 0.01704 | 2 | 2 | 4 | 5 |
| 166 | B-cell receptor-associated protein 31 | IPI00218200 | 1.311 | 0.01704 | 2 | 2 | 3 | 6 |
| 167 | Testis-expressed sequence 10 protein | IPI00549664 | 1.311 | 0.01704 | 2 | 2 | 6 | 3 |
| 168 | WD repeat-containing protein 75 | IPI00217240 | 1.311 | 0.01704 | 3 | 1 | 4 | 5 |
| 169 | Dehydrogenase/reductase SDR family member 7B | IPI00550165 | 1.311 | 0.01704 | 2 | 2 | 4 | 5 |
| 170 | Translational activator of cytochrome c oxidase 1 | IPI00019903 | 1.311 | 0.01704 | 2 | 2 | 5 | 4 |
| 171 | Isoform 1 of Ribose-phosphate pyrophosphokinase 2 | IPI00219617 | 1.311 | 0.01704 | 2 | 2 | 5 | 4 |
| 172 | Putative uncharacterized protein ENSP00000350479 | IPI00069693 | 1.297 | 0.01723 | 9 | 8 | 9 | 16 |
| 173 | Lamin-B2 | IPI00009771 | 1.292 | 0.01723 | 17 | 15 | 23 | 19 |
| 174 | Lysosomal Pro-X carboxypeptidase | IPI00001593 | 1.290 | 0.01738 | 0 | 1 | 5 | 0 |
| 175 | Isoform 1 of Erlin-2 | IPI00026942 | 1.290 | 0.01738 | 1 | 1 | 4 | 2 |
| 176 | Isoform 1 of Probable Xaa-Pro aminopeptidase 3 | IPI00550192 | 1.290 | 0.01738 | 1 | 1 | 3 | 3 |
| 177 | Uncharacterized protein C4orf14 | IPI00385928 | 1.290 | 0.01738 | 1 | 1 | 4 | 2 |
| 178 | NEDD8 ultimate buster 1 Isoform 2 | IPI00157365 | 1.290 | 0.01738 | 0 | 0 | 4 | 2 |
| 179 | M-phase phosphoprotein 6 | IPI00016074 | 1.290 | 0.01738 | 1 | 0 | 3 | 3 |
| 180 | Peroxisomal membrane protein PMP34 | IPI00014440 | 1.290 | 0.01738 | 1 | 0 | 3 | 3 |
| 181 | ER lumen protein retaining receptor 1 | IPI00028116 | 1.290 | 0.01738 | 0 | 1 | 4 | 2 |
| 182 | Protein jagunal homolog 1 | IPI00329025 | 1.290 | 0.01738 | 1 | 0 | 5 | 1 |
| 183 | Isoform 1 of Metallo-beta-lactamase domain-containing protein 2 | IPI00293336 | 1.290 | 0.01738 | 0 | 1 | 3 | 3 |
| 184 | Complement component 1 Q subcomponent-binding protein, mitochondrial | IPI00014230 | 1.281 | 0.02312 | 49 | 48 | 55 | 57 |
| 185 | Aspartyl-tRNA synthetase, cytoplasmic | IPI00216951 | 1.278 | 0.02312 | 17 | 16 | 25 | 18 |
| 186 | sister chromatid cohesion protein PDS5 homolog A isoform 2 | IPI00303063 | 1.273 | 0.02321 | 8 | 10 | 14 | 12 |

| No. | Description | Accession number | STN[1] | p-Value[1] | Con_A[2] | Con_B[2] | LUTEO_A[2] | LUTEO_B[2] |
|---|---|---|---|---|---|---|---|---|
| 187 | Nucleoprotein TPR | IPI00742682 | 1.265 | 0.02374 | 16 | 18 | 20 | 24 |
| 188 | Isoform 1 of Ubiquitin-like modifier-activating enzyme 6 | IPI00023647 | 1.264 | 0.02388 | 5 | 3 | 9 | 5 |
| 189 | Uncharacterized protein KIAA0406 | IPI00011702 | 1.264 | 0.02388 | 3 | 5 | 6 | 8 |
| 190 | 60S ribosomal protein L18 | IPI00215719 | 1.256 | 0.02492 | 14 | 12 | 13 | 22 |
| 191 | Adenylate kinase isoenzyme 1 | IPI00018342 | 1.250 | 0.02492 | 6 | 7 | 11 | 9 |
| 192 | Leucine-rich repeat-containing protein 47 | IPI00170935 | 1.250 | 0.02492 | 7 | 6 | 10 | 10 |
| 193 | Matrin-3 | IPI00017297 | 1.238 | 0.02502 | 31 | 28 | 34 | 37 |
| 194 | DnaJ homolog subfamily C member 3 | IPI00006713 | 1.232 | 0.02511 | 3 | 2 | 7 | 3 |
| 195 | ATP-dependent RNA helicase DHX29 | IPI00217413 | 1.232 | 0.02511 | 4 | 1 | 4 | 6 |
| 196 | Isoform 1 of Armadillo repeat-containing protein 10 | IPI00166394 | 1.232 | 0.02511 | 3 | 2 | 6 | 4 |
| 197 | Alcohol dehydrogenase [NADP+] | IPI00220271 | 1.221 | 0.02573 | 6 | 8 | 11 | 10 |
| 198 | Methylenetetrahydrofolate dehydrogenase (NADP+ dependent) 1-like | IPI00291646 | 1.219 | 0.02573 | 4 | 5 | 9 | 6 |
| 199 | Glucosamine-6-phosphate isomerase 1 | IPI00009305 | 1.219 | 0.02573 | 4 | 5 | 6 | 9 |
| 200 | Ras-related protein Rab-21 | IPI00007755 | 1.219 | 0.02573 | 6 | 3 | 7 | 8 |
| 201 | Ornithine aminotransferase, mitochondrial | IPI00022334 | 1.193 | 0.02606 | 9 | 6 | 12 | 10 |
| 202 | Coatomer subunit beta' | IPI00220219 | 1.190 | 0.02611 | 12 | 10 | 19 | 11 |
| 203 | Calcium-binding mitochondrial carrier protein Aralar2 | IPI00007084 | 1.183 | 0.02659 | 28 | 25 | 32 | 32 |
| 204 | Mitochondrial carrier homolog 2 | IPI00003833 | 1.182 | 0.02659 | 16 | 15 | 23 | 17 |
| 205 | Histone H3.2 | IPI00171611 | 1.179 | 0.02659 | 5 | 5 | 8 | 8 |
| 206 | Peroxisomal multifunctional enzyme type 2 | IPI00019912 | 1.179 | 0.02659 | 4 | 6 | 8 | 8 |
| 207 | DNA replication licensing factor MCM6 | IPI00031517 | 1.174 | 0.02787 | 20 | 22 | 26 | 26 |
| 208 | Ras-related protein Rab-2A | IPI00031169 | 1.172 | 0.02787 | 12 | 11 | 13 | 18 |
| 209 | Phosphoglycerate kinase 1 | IPI00169383 | 1.170 | 0.02787 | 44 | 41 | 46 | 52 |
| 210 | Isoform 1 of Cirhin | IPI00239815 | 1.169 | 0.02787 | 4 | 2 | 5 | 6 |
| 211 | Chromobox protein homolog 5 | IPI00024662 | 1.169 | 0.02787 | 2 | 4 | 6 | 5 |
| 212 | F-box-like/WD repeat-containing protein TBL1XR1 | IPI00002922 | 1.169 | 0.02787 | 5 | 1 | 6 | 5 |
| 213 | NADH-ubiquinone oxidoreductase chain 4 | IPI00008495 | 1.166 | 0.02801 | 1 | 2 | 4 | 3 |
| 214 | 26 kDa protein | IPI00219685 | 1.166 | 0.02801 | 1 | 2 | 4 | 3 |
| 215 | Protein phosphatase 1F | IPI00291412 | 1.166 | 0.02801 | 2 | 1 | 4 | 3 |
| 216 | Nuclear pore complex protein Nup107 | IPI00028005 | 1.166 | 0.02801 | 1 | 2 | 3 | 4 |
| 217 | 39S ribosomal protein L46, mitochondrial | IPI00023161 | 1.166 | 0.02801 | 2 | 1 | 2 | 5 |
| 218 | U3 small nucleolar RNA-associated protein 18 homolog | IPI00000733 | 1.166 | 0.02801 | 2 | 1 | 4 | 3 |
| 219 | Very long-chain acyl-CoA synthetase | IPI00024787 | 1.166 | 0.02801 | 1 | 2 | 3 | 4 |
| 220 | U3 small nucleolar RNA-associated protein 6 homolog | IPI00020128 | 1.166 | 0.02801 | 2 | 1 | 4 | 3 |
| 221 | Cation-independent mannose-6-phosphate receptor | IPI00289819 | 1.166 | 0.02801 | 1 | 2 | 3 | 4 |
| 222 | Isoform Beta of Heat shock protein 105 kDa | IPI00218993 | 1.154 | 0.02829 | 24 | 20 | 30 | 24 |
| 223 | Elongation factor 2 | IPI00186290 | 1.151 | 0.02839 | 131 | 107 | 139 | 118 |
| 224 | Isoform 2 of AP-3 complex subunit delta-1 | IPI00289608 | 1.145 | 0.02863 | 9 | 8 | 13 | 11 |
| 225 | Transmembrane protein 33 | IPI00299084 | 1.144 | 0.02863 | 6 | 5 | 9 | 8 |
| 226 | Isoform 3 of Obg-like ATPase 1 | IPI00216106 | 1.144 | 0.02863 | 5 | 6 | 9 | 8 |
| 227 | Peptidyl-prolyl cis-trans isomerase B | IPI00646304 | 1.119 | 0.02934 | 24 | 24 | 29 | 29 |
| 228 | Isoform 1 of Alanine aminotransferase 2 | IPI00152432 | 1.116 | 0.02948 | 4 | 3 | 5 | 7 |
| 229 | Ubiquitin carboxyl-terminal hydrolase 11 | IPI00184533 | 1.116 | 0.02948 | 5 | 2 | 7 | 5 |
| 230 | Inositol monophosphatase 1 | IPI00020906 | 1.116 | 0.02948 | 4 | 3 | 7 | 5 |
| 231 | Methionine aminopeptidase 2 | IPI00033036 | 1.116 | 0.02948 | 4 | 3 | 7 | 5 |
| 232 | Proline synthetase co-transcribed homolog (Bacterial), isoform CRA_b | IPI00016346 | 1.112 | 0.02972 | 7 | 5 | 8 | 10 |
| 233 | ADP-sugar pyrophosphatase | IPI00296913 | 1.108 | 0.03048 | 12 | 15 | 18 | 17 |
| 234 | Proteasome subunit beta type-5 | IPI00479306 | 1.103 | 0.03048 | 11 | 8 | 16 | 10 |
| 235 | Plastin-1 | IPI00032304 | 1.094 | 0.03048 | 14 | 14 | 20 | 16 |
| 236 | Stress-induced-phosphoprotein 1 | IPI00013894 | 1.094 | 0.03067 | 29 | 22 | 33 | 28 |
| 237 | Isoform 4 of Serine/threonine-protein phosphatase 6 regulatory subunit 3 | IPI00019540 | 1.084 | 0.03076 | 8 | 5 | 10 | 9 |
| 238 | Cytoplasmic dynein 1 light intermediate chain 2 | IPI00011592 | 1.084 | 0.03076 | 5 | 8 | 9 | 10 |
| 239 | Isoform 1 of AP-2 complex subunit beta | IPI00784156 | 1.083 | 0.03076 | 12 | 8 | 16 | 11 |
| 240 | Endoplasmic reticulum resident protein 29 | IPI00024911 | 1.083 | 0.03076 | 11 | 9 | 14 | 13 |
| 241 | 60S ribosomal protein L3 | IPI00550021 | 1.081 | 0.03081 | 14 | 15 | 17 | 20 |
| 242 | Isoform 1 of Bcl-2-associated transcription factor 1 | IPI00006079 | 1.077 | 0.03081 | 3 | 1 | 5 | 3 |
| 243 | Huntingtin-interacting protein 1 | IPI00782965 | 1.077 | 0.03081 | 2 | 2 | 3 | 5 |
| 244 | NAD-dependent malic enzyme, mitochondrial | IPI00011201 | 1.070 | 0.03537 | 3 | 5 | 6 | 7 |
| 245 | Isoform 2 of Serrate RNA effector molecule homolog | IPI00220038 | 1.070 | 0.03537 | 4 | 4 | 7 | 6 |
| 246 | Cytochrome c oxidase subunit 4 isoform 1, mitochondrial | IPI00006579 | 1.070 | 0.03537 | 4 | 4 | 7 | 6 |
| 247 | Pre-rRNA-processing protein TSR1 homolog | IPI00292894 | 1.070 | 0.03537 | 6 | 2 | 7 | 6 |
| 248 | 60S acidic ribosomal protein P1 | IPI00008527 | 1.070 | 0.03537 | 3 | 5 | 7 | 6 |
| 249 | Acyl-CoA dehydrogenase family member 9, mitochondrial | IPI00152981 | 1.070 | 0.03537 | 4 | 4 | 7 | 6 |
| 250 | Mitochondrial 2-oxoglutarate/malate carrier protein | IPI00219729 | 1.068 | 0.03580 | 16 | 14 | 20 | 18 |
| 251 | cDNA FLJ55482, highly similar to Annexin A11 | IPI00414320 | 1.065 | 0.03589 | 12 | 9 | 14 | 14 |
| 252 | Isoform 1 of Leukotriene A-4 hydrolase | IPI00219077 | 1.034 | 0.03712 | 24 | 21 | 28 | 26 |
| 253 | Methionyl-tRNA synthetase, cytoplasmic | IPI00008240 | 1.033 | 0.03712 | 18 | 15 | 21 | 20 |
| 254 | Similar to Signal peptidase complex subunit 2 | IPI00452747 | 1.031 | 0.03712 | 6 | 3 | 7 | 7 |
| 255 | Isoform 1 of UDP-glucose:glycoprotein glucosyltransferase 1 | IPI00024466 | 1.025 | 0.03727 | 31 | 30 | 39 | 32 |
| 256 | tRNA (cytosine-5-)-methyltransferase NSUN2 | IPI00306369 | 1.017 | 0.03760 | 14 | 10 | 15 | 16 |
| 257 | ADP/ATP translocase 3 | IPI00291467 | 1.016 | 0.03760 | 81 | 69 | 88 | 76 |
| 258 | cDNA FLJ56153, highly similar to Homo sapiens transforming growth factor beta regulator 4 | IPI00329625 | 1.011 | 0.03769 | 7 | 9 | 11 | 11 |
| 259 | Isoform 3 of HEAT repeat-containing protein 5B | IPI00333696 | 1.011 | 0.03807 | 1 | 0 | 3 | 2 |
| 260 | protein ELYS | IPI00170594 | 1.011 | 0.03807 | 1 | 0 | 2 | 3 |
| 261 | Isoform 2 of Tumor protein D54 | IPI00221178 | 1.011 | 0.03807 | 0 | 1 | 3 | 2 |
| 262 | Component of gems 4 | IPI00027717 | 1.011 | 0.03807 | 1 | 1 | 3 | 2 |
| 263 | Similar to Ankyrin repeat and FYVE domain-containing protein 1 | IPI00159899 | 1.011 | 0.03807 | 1 | 1 | 1 | 4 |
| 264 | negative elongation factor A | IPI00394679 | 1.011 | 0.03807 | 1 | 1 | 2 | 3 |
| 265 | Isoform 1 of Putative helicase MOV-10 | IPI00444452 | 1.011 | 0.03807 | 1 | 0 | 2 | 3 |
| 266 | Transmembrane 9 superfamily member 4 | IPI00021985 | 1.011 | 0.03807 | 0 | 1 | 3 | 2 |
| 267 | Lysosome-associated membrane glycoprotein 1 | IPI00884105 | 1.011 | 0.03807 | 1 | 1 | 3 | 2 |
| 268 | Putative uncharacterized protein KIAA0090 | IPI00640734 | 1.011 | 0.03807 | 0 | 0 | 3 | 2 |
| 269 | Isoform 2 of Transportin-2 | IPI00164417 | 1.011 | 0.03807 | 0 | 0 | 2 | 3 |
| 270 | Phosphatidylinositide phosphatase SAC1 | IPI00022275 | 1.011 | 0.03807 | 1 | 1 | 4 | 0 |
| 271 | ATP-dependent RNA helicase DDX55 | IPI00185361 | 1.011 | 0.03807 | 1 | 1 | 3 | 2 |
| 272 | Sentrin-specific protease 3 | IPI00171525 | 1.011 | 0.03807 | 1 | 1 | 3 | 2 |
| 273 | Proteolipid protein 2 | IPI00030362 | 1.011 | 0.03807 | 0 | 0 | 3 | 2 |
| 274 | V-type proton ATPase subunit C 1 | IPI00007814 | 1.009 | 0.03964 | 2 | 3 | 5 | 4 |
| 275 | ATP-dependent RNA helicase SUPV3L1, mitochondrial | IPI00412404 | 1.009 | 0.03964 | 2 | 3 | 5 | 4 |
| 276 | Isoform 2 of Cytosolic non-specific dipeptidase | IPI00165579 | 1.009 | 0.03964 | 2 | 3 | 5 | 4 |
| 277 | 1-phosphatidylinositol-4,5-bisphosphate phosphodiesterase beta-3 | IPI00010400 | 1.009 | 0.03964 | 3 | 2 | 5 | 4 |
| 278 | Isoform AGX2 of UDP-N-acetylhexosamine pyrophosphorylase | IPI00000684 | 1.009 | 0.03964 | 2 | 3 | 4 | 5 |
| 279 | Isoform 1 of Nuclear pore membrane glycoprotein 210 | IPI00291755 | 1.003 | 0.03969 | 11 | 14 | 14 | 18 |
| 280 | DNA mismatch repair protein Msh2 | IPI00017303 | 1.003 | 0.03969 | 14 | 11 | 18 | 14 |
| 281 | ERO1-like protein alpha | IPI00386755 | 0.997 | 0.03983 | 5 | 5 | 8 | 7 |

| No. | Description | Accession number | STN[1] | p-Value[1] | Con_A[2] | Con_B[2] | LUTEO_A[2] | LUTEO_B[2] |
|---|---|---|---|---|---|---|---|---|
| 282 | Midasin | IPI00167941 | 0.997 | 0.03983 | 1 | 9 | 6 | 9 |
| 283 | GTP-binding protein SAR1b | IPI00002149 | 0.997 | 0.03983 | 6 | 4 | 9 | 6 |
| 284 | Isoleucyl-tRNA synthetase | IPI00514082 | 0.997 | 0.03983 | 6 | 4 | 6 | 9 |
| 285 | Isoform 1 of Apoptosis-inducing factor 1, mitochondrial | IPI00000690 | 0.995 | 0.03997 | 25 | 25 | 32 | 27 |
| 286 | NAD(P) transhydrogenase, mitochondrial | IPI00337541 | 0.976 | 0.04059 | 11 | 16 | 19 | 15 |
| 287 | 14-3-3 protein gamma | IPI00220642 | 0.976 | 0.04059 | 16 | 11 | 17 | 17 |
| 288 | Programmed cell death 6-interacting protein | IPI00246058 | 0.972 | 0.04069 | 21 | 18 | 24 | 23 |
| 289 | ADP-ribosylation factor 4 | IPI00215918 | 0.971 | 0.04069 | 8 | 10 | 12 | 12 |
| 290 | Mannosyl-oligosaccharide glucosidase | IPI00328170 | 0.966 | 0.04149 | 6 | 5 | 11 | 5 |
| 291 | Carbonyl reductase [NADPH] 1 | IPI00295386 | 0.966 | 0.04149 | 6 | 5 | 7 | 9 |
| 292 | Transitional endoplasmic reticulum ATPase | IPI00022774 | 0.957 | 0.04187 | 49 | 45 | 61 | 44 |
| 293 | Neutral amino acid transporter B(0) | IPI00019472 | 0.957 | 0.04187 | 44 | 50 | 62 | 43 |
| 294 | Isoform Beta of Nucleolar and coiled-body phosphoprotein 1 | IPI00216654 | 0.955 | 0.04187 | 3 | 3 | 6 | 4 |
| 295 | Chloride intracellular channel protein 4 | IPI00001960 | 0.955 | 0.04187 | 3 | 3 | 7 | 3 |
| 296 | Isoform 2 of Ubiquitin conjugation factor E4 A | IPI00028957 | 0.955 | 0.04187 | 3 | 3 | 6 | 4 |
| 297 | Prostaglandin E synthase 2 | IPI00303568 | 0.955 | 0.04187 | 2 | 4 | 5 | 5 |
| 298 | AFG3-like protein 2 | IPI00001091 | 0.955 | 0.04187 | 2 | 4 | 6 | 4 |
| 299 | Pirin | IPI00012575 | 0.955 | 0.04187 | 2 | 4 | 4 | 6 |
| 300 | DCN1-like protein 1 | IPI00291893 | 0.955 | 0.04187 | 4 | 2 | 5 | 5 |
| 301 | D-3-phosphoglycerate dehydrogenase | IPI00011200 | 0.938 | 0.04719 | 7 | 5 | 8 | 9 |
| 302 | Isoform 2 of Voltage-dependent anion-selective channel protein 2 | IPI00024145 | 0.931 | 0.04838 | 20 | 24 | 28 | 24 |
| 303 | Tyrosyl-tRNA synthetase, cytoplasmic | IPI00007074 | 0.929 | 0.04842 | 17 | 14 | 22 | 16 |
| 304 | Prohibitin | IPI00017334 | 0.918 | 0.04842 | 40 | 42 | 42 | 50 |
| 305 | Isoform SERCA1B of Sarcoplasmic/endoplasmic reticulum calcium ATPase 1 | IPI00024804 | 0.913 | 0.04861 | 5 | 8 | 8 | 10 |
| 306 | Platelet-activating factor acetylhydrolase IB subunit gamma | IPI00014808 | 0.910 | 0.04880 | 4 | 3 | 6 | 5 |
| 307 | Importin-11 | IPI00301107 | 0.910 | 0.04880 | 3 | 4 | 6 | 5 |
| 308 | Isoform 5 of Myosin-14 | IPI00029818 | 0.910 | 0.04880 | 3 | 4 | 5 | 6 |
| 309 | Cell division cycle 5-like protein | IPI00465294 | 0.910 | 0.04880 | 5 | 2 | 7 | 4 |
| 310 | Tubulin-specific chaperone E | IPI00018402 | 0.910 | 0.04880 | 5 | 2 | 5 | 6 |
| 311 | UPF0556 protein C19orf10 | IPI00056357 | 0.910 | 0.04880 | 3 | 4 | 6 | 5 |
| 312 | Mitochondrial glutamate carrier 1 | IPI00003004 | 0.910 | 0.04880 | 4 | 3 | 6 | 5 |
| 313 | Activating signal cointegrator 1 complex subunit 3 | IPI00430472 | 0.906 | 0.04885 | 1 | 2 | 2 | 4 |
| 314 | Isoform 1 of COP9 signalosome complex subunit 1 | IPI00156282 | 0.906 | 0.04885 | 2 | 0 | 2 | 4 |
| 315 | Isoform 1 of Alkylated DNA repair protein alkB homolog 4 | IPI00033770 | 0.906 | 0.04885 | 2 | 0 | 4 | 2 |
| 316 | Isoform 3 of Protein scribble homolog | IPI00410666 | 0.906 | 0.04885 | 1 | 2 | 3 | 3 |
| 317 | Coiled-coil domain-containing protein 6 | IPI00000634 | 0.906 | 0.04885 | 1 | 2 | 2 | 4 |
| 318 | Copine-3 | IPI00024403 | 0.906 | 0.04885 | 1 | 2 | 5 | 0 |
| 319 | Ubiquitin domain-containing protein UBFD1 | IPI00005194 | 0.906 | 0.04885 | 2 | 1 | 3 | 3 |
| 320 | Isoform 1 of YTH domain family protein 1 | IPI00221345 | 0.906 | 0.04885 | 2 | 1 | 3 | 3 |
| 321 | Isoform 3 of DnaJ homolog subfamily C member 11 | IPI00333016 | 0.906 | 0.04885 | 1 | 2 | 4 | 2 |
| 322 | Isoform 2 of Glutaminase kidney isoform, mitochondrial | IPI00215685 | 0.906 | 0.04885 | 2 | 1 | 3 | 3 |
| 323 | Isoform 1 of Ubiquitin-protein ligase E3C | IPI00604464 | 0.906 | 0.04885 | 2 | 0 | 3 | 3 |
| 324 | Isoform 1 of Splicing factor 3B subunit 3 | IPI00300371 | 0.905 | 0.04885 | 49 | 60 | 56 | 64 |
| 325 | Succinyl-CoA:3-ketoacid-coenzyme A transferase 1, mitochondrial | IPI00026516 | 0.905 | 0.04890 | 11 | 11 | 16 | 12 |
| 326 | Isoform Long of 14-3-3 protein beta/alpha | IPI00216318 | 0.898 | 0.04975 | 18 | 16 | 25 | 16 |
| 327 | Isoform 1 of Cytoskeleton-associated protein 5 | IPI00028275 | 0.891 | 0.04994 | 7 | 7 | 8 | 11 |
| 328 | Isoform 1 of 6-phosphofructokinase, liver type | IPI00332371 | 0.891 | 0.04994 | 8 | 6 | 9 | 10 |
| 329 | Isoform 1 of 2',5'-phosphodiesterase 12 | IPI00174390 | 0.891 | 0.04994 | 6 | 8 | 9 | 10 |
| 330 | Isoform 1 of 3,2-trans-enoyl-CoA isomerase, mitochondrial | IPI00300567 | 0.871 | 0.05080 | 4 | 4 | 7 | 5 |
| 331 | Putative uncharacterized protein DKFZp451D234 | IPI00031583 | 0.871 | 0.05080 | 4 | 4 | 6 | 6 |
| 332 | Nucleolar complex protein 3 homolog | IPI00102815 | 0.871 | 0.05080 | 5 | 3 | 8 | 4 |
| 333 | Heat shock protein 75 kDa, mitochondrial | IPI00030275 | 0.852 | 0.05521 | 37 | 39 | 45 | 40 |
| 334 | Isoform 1 of Putative ATP-dependent RNA helicase DHX30 | IPI00411733 | 0.842 | 0.05526 | 13 | 14 | 18 | 15 |
| 335 | U2 small nuclear ribonucleoprotein A' | IPI00297477 | 0.842 | 0.05526 | 16 | 11 | 20 | 13 |
| 336 | Pre-mRNA-processing-splicing factor 8 | IPI00007928 | 0.840 | 0.05531 | 79 | 87 | 91 | 87 |
| 337 | 2-oxoglutarate dehydrogenase, mitochondrial | IPI00098902 | 0.838 | 0.05540 | 5 | 4 | 8 | 5 |
| 338 | Lamin-B receptor | IPI00292135 | 0.833 | 0.05545 | 8 | 9 | 12 | 10 |
| 339 | Isoform 1 of Nck-associated protein 1 | IPI00031982 | 0.832 | 0.05559 | 2 | 2 | 3 | 4 |
| 340 | Isoform 1 of AP-3 complex subunit beta-1 | IPI00021129 | 0.832 | 0.05559 | 2 | 2 | 4 | 3 |
| 341 | DnaJ homolog subfamily B member 11 | IPI00008454 | 0.832 | 0.05559 | 2 | 2 | 2 | 5 |
| 342 | Similar to Zinc finger CCCH domain-containing protein 15 | IPI00000279 | 0.832 | 0.05559 | 2 | 2 | 3 | 4 |
| 343 | treacle protein isoform a | IPI00165041 | 0.832 | 0.05559 | 2 | 2 | 3 | 4 |
| 344 | Isoform 2 of Epimerase family protein SDR39U1 | IPI00643286 | 0.832 | 0.05559 | 2 | 2 | 3 | 4 |
| 345 | Isoform 3 of Shootin-1 | IPI00448751 | 0.832 | 0.05559 | 3 | 1 | 5 | 2 |
| 346 | Isoform 2 of tRNA pseudouridine synthase A | IPI00001716 | 0.832 | 0.05559 | 2 | 2 | 3 | 4 |
| 347 | Serine palmitoyltransferase 2 | IPI00005751 | 0.832 | 0.05559 | 1 | 3 | 3 | 4 |
| 348 | Zinc finger, MYM-type 6, isoform CRA_a | IPI00168438 | 0.832 | 0.05559 | 3 | 1 | 4 | 3 |
| 349 | Isoform 2 of Apoptosis inhibitor 5 | IPI00554742 | 0.811 | 0.05773 | 21 | 24 | 27 | 25 |
| 350 | Protein DJ-1 | IPI00298547 | 0.810 | 0.05778 | 13 | 17 | 19 | 17 |
| 351 | Isoform 1 of Bifunctional coenzyme A synthase | IPI00184821 | 0.809 | 0.05830 | 6 | 4 | 7 | 7 |
| 352 | Isoform 1 of Trans-2,3-enoyl-CoA reductase | IPI00100656 | 0.809 | 0.05830 | 6 | 4 | 7 | 7 |
| 353 | Isoform 1 of Transportin-1 | IPI00024364 | 0.801 | 0.06186 | 15 | 16 | 21 | 16 |
| 354 | Isoform Long of Splicing factor, proline- and glutamine-rich | IPI00010740 | 0.798 | 0.06191 | 24 | 23 | 28 | 26 |
| 355 | Condensin complex subunit 3 | IPI00106495 | 0.786 | 0.06238 | 11 | 9 | 12 | 13 |
| 356 | cDNA FLJ55586, highly similar to MMS19-like protein | IPI00154451 | 0.786 | 0.06238 | 11 | 9 | 16 | 9 |
| 357 | Isoform 1 of tRNA (adenine-N(1)-)-methyltransferase non-catalytic subunit TRM6 | IPI00099311 | 0.783 | 0.06243 | 5 | 6 | 9 | 6 |
| 358 | GTP:AMP phosphotransferase mitochondrial | IPI00465256 | 0.783 | 0.06243 | 5 | 6 | 6 | 9 |
| 359 | 60S ribosomal protein L13a | IPI00304612 | 0.777 | 0.06262 | 3 | 2 | 3 | 5 |
| 360 | DEAD (Asp-Glu-Ala-Asp) box polypeptide 39 transcript variant | IPI00062206 | 0.777 | 0.06262 | 3 | 2 | 3 | 5 |
| 361 | Isoform 1 of Protein transport protein Sec24A | IPI00873472 | 0.777 | 0.06262 | 3 | 2 | 4 | 4 |
| 362 | Cytochrome b-c1 complex subunit 1, mitochondrial | IPI00013847 | 0.777 | 0.06262 | 3 | 2 | 4 | 4 |
| 363 | Keratin, type II cytoskeletal 5 | IPI00009867 | 0.777 | 0.06262 | 3 | 2 | 5 | 3 |
| 364 | RNA-binding protein 28 | IPI00304187 | 0.777 | 0.06262 | 3 | 2 | 4 | 4 |
| 365 | Dihydroorotate dehydrogenase, mitochondrial | IPI00024462 | 0.777 | 0.06262 | 2 | 3 | 5 | 3 |
| 366 | Sialic acid synthase | IPI00147874 | 0.777 | 0.06262 | 2 | 3 | 3 | 5 |
| 367 | 114 kDa protein | IPI00166555 | 0.777 | 0.06262 | 3 | 2 | 4 | 4 |
| 368 | Isoform 1 of ER lumen protein retaining receptor 2 | IPI00018248 | 0.774 | 0.06366 | 18 | 16 | 19 | 21 |
| 369 | 60S ribosomal protein L7a | IPI00299573 | 0.774 | 0.06366 | 16 | 18 | 20 | 20 |
| 370 | KH-type splicing regulatory protein | IPI00479786 | 0.766 | 0.06423 | 18 | 17 | 23 | 18 |
| 371 | Hypoxanthine-guanine phosphoribosyltransferase | IPI00218493 | 0.763 | 0.06423 | 25 | 28 | 30 | 30 |
| 372 | Kinesin-like protein KIF11 | IPI00305289 | 0.760 | 0.06423 | 7 | 5 | 8 | 8 |
| 373 | Isoform 1 of Deoxyuridine 5'-triphosphate nucleotidohydrolase, mitochondrial | IPI00013679 | 0.758 | 0.06689 | 20 | 16 | 26 | 16 |
| 374 | Ras-related protein Rab-7a | IPI00016342 | 0.758 | 0.06756 | 30 | 24 | 32 | 29 |
| 375 | Small nuclear ribonucleoprotein Sm D2 | IPI00017963 | 0.740 | 0.06789 | 8 | 5 | 8 | 9 |
| 376 | Ras-related protein Rab-18 | IPI00014577 | 0.740 | 0.06789 | 8 | 5 | 8 | 9 |

| No. | Description | Accession number | STN[1] | p-Value[1] | Con_A[2] | Con_B[2] | LUTEO_A[2] | LUTEO_B[2] |
|---|---|---|---|---|---|---|---|---|
| 377 | Importin subunit alpha-2 | IPI00002214 | 0.737 | 0.06789 | 13 | 11 | 14 | 15 |
| 378 | Protein MEMO1 | IPI00032426 | 0.733 | 0.06789 | 3 | 3 | 4 | 5 |
| 379 | Isoform 1 of KDEL motif-containing protein 2 | IPI00143921 | 0.733 | 0.06789 | 2 | 4 | 3 | 6 |
| 380 | Isoform 1 of Insulin-like growth factor 2 mRNA-binding protein 3 | IPI00658000 | 0.733 | 0.06789 | 2 | 4 | 4 | 5 |
| 381 | Syntaxin-binding protein 3 | IPI00297626 | 0.733 | 0.06789 | 3 | 3 | 6 | 3 |
| 382 | Phosphoserine phosphatase | IPI00019178 | 0.733 | 0.06789 | 3 | 3 | 5 | 4 |
| 383 | Putative rRNA methyltransferase 3 | IPI00217686 | 0.733 | 0.06789 | 2 | 4 | 5 | 4 |
| 384 | Vacuolar protein sorting-associated protein 33A | IPI00073179 | 0.733 | 0.06789 | 3 | 3 | 5 | 4 |
| 385 | Isoform 1 of Pyridoxal kinase | IPI00013004 | 0.726 | 0.06964 | 14 | 11 | 15 | 15 |
| 386 | cDNA FLJ59211, highly similar to Glucosidase 2 subunit beta | IPI00026154 | 0.722 | 0.06988 | 23 | 18 | 25 | 22 |
| 387 | Isoform 1 of Cell division cycle and apoptosis regulator protein 1 | IPI00217357 | 0.721 | 0.06988 | 7 | 7 | 9 | 9 |
| 388 | cDNA FLJ54957, highly similar to Transketolase | IPI00643920 | 0.715 | 0.07278 | 44 | 45 | 45 | 52 |
| 389 | Ubiquitin carboxyl-terminal hydrolase 10 | IPI00291946 | 0.709 | 0.07292 | 0 | 1 | 2 | 2 |
| 390 | Non-functional aryl hydrocarbon receptor interacting protein (Fragment) | IPI00925804 | 0.709 | 0.07292 | 0 | 1 | 3 | 0 |
| 391 | Lysosomal alpha-glucosidase | IPI00293088 | 0.709 | 0.07292 | 1 | 1 | 3 | 1 |
| 392 | Dolichyldiphosphatase 1 | IPI00329410 | 0.709 | 0.07292 | 1 | 0 | 3 | 1 |
| 393 | Isoform A of Kinesin light chain 1 | IPI00020096 | 0.709 | 0.07292 | 1 | 1 | 3 | 1 |
| 394 | Isoform 2 of Ribosomal RNA processing protein 1 homolog B | IPI00032374 | 0.709 | 0.07292 | 1 | 1 | 3 | 1 |
| 395 | Heterochromatin protein 1, binding protein 3 | IPI00640417 | 0.709 | 0.07292 | 1 | 1 | 2 | 2 |
| 396 | Ribonucleases P/MRP protein subunit POP1 | IPI00293331 | 0.709 | 0.07292 | 0 | 1 | 3 | 0 |
| 397 | 60S ribosomal protein L35a | IPI00029731 | 0.709 | 0.07292 | 1 | 1 | 2 | 2 |
| 398 | Beta-lactamase-like protein 2 | IPI00006952 | 0.709 | 0.07292 | 1 | 1 | 2 | 2 |
| 399 | Protein tyrosine phosphatase type IVA 1 | IPI00020164 | 0.709 | 0.07292 | 1 | 1 | 0 | 3 |
| 400 | COMM domain-containing protein 2 | IPI00456048 | 0.709 | 0.07292 | 1 | 1 | 2 | 2 |
| 401 | Replication initiator 1 | IPI00549171 | 0.709 | 0.07292 | 1 | 0 | 2 | 2 |
| 402 | NADP-dependent malic enzyme | IPI00008215 | 0.709 | 0.07292 | 1 | 1 | 2 | 2 |
| 403 | Epoxide hydrolase 1 | IPI00009896 | 0.709 | 0.07292 | 1 | 1 | 2 | 2 |
| 404 | DNA repair protein XRCC1 | IPI00002564 | 0.709 | 0.07292 | 1 | 1 | 3 | 1 |
| 405 | Isoform 1 of N-alpha-acetyltransferase 25, NatB auxiliary subunit | IPI00025890 | 0.709 | 0.07292 | 1 | 1 | 3 | 0 |
| 406 | SCY1-like protein 2 | IPI00396218 | 0.709 | 0.07292 | 0 | 0 | 2 | 2 |
| 407 | Laminin subunit beta-1 | IPI00013976 | 0.709 | 0.07292 | 1 | 0 | 0 | 3 |
| 408 | Enhancer of mRNA-decapping protein 3 | IPI00018009 | 0.709 | 0.07292 | 1 | 0 | 1 | 3 |
| 409 | Isoform 1 of Protein disulfide-isomerase TMX3 | IPI00064193 | 0.709 | 0.07292 | 1 | 1 | 3 | 1 |
| 410 | Zinc finger protein 622 | IPI00056499 | 0.709 | 0.07292 | 1 | 1 | 2 | 2 |
| 411 | Phosphopantothenate--cysteine ligase | IPI00023987 | 0.709 | 0.07292 | 0 | 0 | 2 | 2 |
| 412 | Ribosomal protein S27 | IPI00514399 | 0.709 | 0.07292 | 1 | 1 | 2 | 2 |
| 413 | Isoform 2 of ATPase WRNIP1 | IPI00102997 | 0.709 | 0.07292 | 0 | 0 | 2 | 2 |
| 414 | PDXDC1 protein | IPI00329208 | 0.709 | 0.07292 | 1 | 0 | 2 | 2 |
| 415 | Propionyl-CoA carboxylase beta chain, mitochondrial | IPI00007247 | 0.709 | 0.07292 | 1 | 1 | 3 | 0 |
| 416 | erlin-1 | IPI00007940 | 0.709 | 0.07292 | 1 | 1 | 2 | 2 |
| 417 | Isoform 2 of Kinesin-like protein KIF2C | IPI00216113 | 0.709 | 0.07292 | 0 | 0 | 2 | 2 |
| 418 | Growth/differentiation factor 15 | IPI00306543 | 0.709 | 0.07292 | 0 | 0 | 2 | 2 |
| 419 | Isoform 1a of Oxysterol-binding protein-related protein 3 | IPI00023555 | 0.709 | 0.07292 | 0 | 0 | 1 | 3 |
| 420 | Isoform 1 of Acylglycerol kinase, mitochondrial | IPI00019353 | 0.709 | 0.07292 | 0 | 1 | 2 | 2 |
| 421 | Isoform 1 of Neuroguidin | IPI00000162 | 0.709 | 0.07292 | 1 | 0 | 3 | 0 |
| 422 | Annexin A1 | IPI00218918 | 0.709 | 0.07292 | 0 | 1 | 3 | 1 |
| 423 | Pyruvate dehydrogenase phosphatase regulatory subunit, mitochondrial | IPI00168407 | 0.709 | 0.07292 | 1 | 1 | 3 | 1 |
| 424 | Isoform 1 of Acyl-coenzyme A thioesterase 9, mitochondrial | IPI00220710 | 0.709 | 0.07292 | 1 | 1 | 1 | 3 |
| 425 | Isoform 2 of Endoplasmic reticulum aminopeptidase 1 | IPI00165949 | 0.709 | 0.07292 | 1 | 0 | 2 | 2 |
| 426 | USP48 protein | IPI00328815 | 0.709 | 0.07292 | 0 | 1 | 3 | 0 |
| 427 | Isoform 1 of General transcription factor 3C polypeptide 3 | IPI00015806 | 0.709 | 0.07292 | 1 | 0 | 2 | 2 |
| 428 | Isoform 2 of Dephospho-CoA kinase domain-containing protein | IPI00015737 | 0.709 | 0.07292 | 0 | 1 | 2 | 2 |
| 429 | Choline/ethanolaminephosphotransferase 1 | IPI00005775 | 0.709 | 0.07292 | 1 | 0 | 2 | 2 |
| 430 | Ubiquitin-conjugating enzyme E2 variant 2 | IPI00019600 | 0.709 | 0.07292 | 1 | 1 | 2 | 2 |
| 431 | Armadillo repeat-containing X-linked protein 3 | IPI00009906 | 0.709 | 0.07292 | 0 | 0 | 1 | 3 |
| 432 | Probable ATP-dependent RNA helicase DDX20 | IPI00005904 | 0.709 | 0.07292 | 0 | 0 | 3 | 0 |
| 433 | Isoform 1 of Core-binding factor subunit beta | IPI00016746 | 0.703 | 0.09832 | 8 | 7 | 11 | 8 |
| 434 | Isoform 1 of Vesicle-associated membrane protein-associated protein B/C | IPI00006211 | 0.703 | 0.09832 | 8 | 7 | 9 | 10 |
| 435 | Cystatin-B | IPI00021828 | 0.696 | 0.09856 | 4 | 3 | 5 | 5 |
| 436 | 33 kDa protein | IPI00413108 | 0.696 | 0.09856 | 3 | 4 | 5 | 5 |
| 437 | Isoform 1 of SET domain-containing protein 3 | IPI00165026 | 0.696 | 0.09856 | 4 | 3 | 6 | 4 |
| 438 | Splicing factor, arginine/serine-rich 4 | IPI00000015 | 0.696 | 0.09856 | 2 | 5 | 4 | 6 |
| 439 | tRNA methyltransferase 112 homolog | IPI00009010 | 0.696 | 0.09856 | 2 | 5 | 6 | 4 |
| 440 | Heme-binding protein 1 | IPI00148063 | 0.696 | 0.09856 | 2 | 5 | 5 | 5 |
| 441 | 60S ribosomal protein L15 | IPI00470528 | 0.688 | 0.10027 | 14 | 15 | 18 | 16 |
| 442 | Dihydrolipoyllysine-residue acetyltransferase component of pyruvate dehydrogenase complex | IPI00021338 | 0.688 | 0.10027 | 17 | 12 | 16 | 18 |
| 443 | 60S ribosomal protein L24 | IPI00306332 | 0.687 | 0.10060 | 9 | 7 | 11 | 9 |
| 444 | Coatomer subunit beta | IPI00295851 | 0.686 | 0.10193 | 24 | 23 | 33 | 20 |
| 445 | Carnitine O-palmitoyltransferase 2, mitochondrial | IPI00012912 | 0.666 | 0.10278 | 3 | 5 | 8 | 3 |
| 446 | Proteasome inhibitor PI31 subunit | IPI00009949 | 0.666 | 0.10278 | 4 | 4 | 7 | 4 |
| 447 | Mannose-P-dolichol utilization defect 1 protein | IPI00025292 | 0.666 | 0.10278 | 5 | 3 | 5 | 6 |
| 448 | Isoform 1 of Protein zwilch homolog | IPI00329679 | 0.666 | 0.10278 | 4 | 4 | 6 | 5 |
| 449 | Ribose-phosphate pyrophosphokinase 3 | IPI00218371 | 0.666 | 0.10278 | 4 | 4 | 5 | 6 |
| 450 | cDNA FLJ14048 fis, clone HEMBA1006650, weakly similar to ARP2/3 COMPLEX 20 KD SUBUNIT | IPI00386354 | 0.666 | 0.10278 | 5 | 3 | 6 | 5 |
| 451 | Zinc finger protein ZPR1 | IPI00025244 | 0.666 | 0.10278 | 4 | 4 | 8 | 3 |
| 452 | F-actin-capping protein subunit alpha-1 | IPI00005969 | 0.659 | 0.10364 | 11 | 7 | 12 | 10 |
| 453 | Stomatin-like protein 2 | IPI00334190 | 0.659 | 0.10364 | 8 | 10 | 13 | 9 |
| 454 | Isoform 1 of Probable threonyl-tRNA synthetase 2, cytoplasmic | IPI00328082 | 0.646 | 0.10630 | 10 | 9 | 13 | 10 |
| 455 | synembryn-A | IPI00100106 | 0.639 | 0.10667 | 5 | 4 | 8 | 4 |
| 456 | Vesicle transport protein GOT1B | IPI00007061 | 0.639 | 0.10667 | 5 | 4 | 4 | 8 |
| 457 | Chromobox protein homolog 3 | IPI00297579 | 0.634 | 0.10786 | 9 | 11 | 10 | 14 |
| 458 | Lysophosphatidylcholine acyltransferase 1 | IPI00171626 | 0.629 | 0.10924 | 2 | 1 | 4 | 1 |
| 459 | COP9 signalosome complex subunit 6 | IPI00163230 | 0.629 | 0.10924 | 1 | 2 | 2 | 3 |
| 460 | Sterol-4-alpha-carboxylate 3-dehydrogenase, decarboxylating | IPI00019407 | 0.629 | 0.10924 | 1 | 2 | 2 | 3 |
| 461 | Probable ATP-dependent RNA helicase DDX10 | IPI00297900 | 0.629 | 0.10924 | 1 | 2 | 2 | 3 |
| 462 | Keratin, type I cytoskeletal 24 | IPI00004550 | 0.629 | 0.10924 | 0 | 2 | 4 | 1 |
| 463 | PDZ and LIM domain protein 5 | IPI00007935 | 0.629 | 0.10924 | 1 | 2 | 2 | 3 |
| 464 | Isoform 1 of Histone-arginine methyltransferase CARM1 | IPI00412880 | 0.629 | 0.10924 | 1 | 2 | 3 | 2 |
| 465 | Isoform 1 of Beta-galactosidase | IPI00441344 | 0.629 | 0.10924 | 1 | 2 | 4 | 0 |
| 466 | Isoform 1 of 5'-nucleotidase domain-containing protein 3 | IPI00465170 | 0.629 | 0.10924 | 2 | 1 | 2 | 3 |
| 467 | Isoform 1 of Transmembrane emp24 domain-containing protein 4 | IPI00296259 | 0.629 | 0.10924 | 1 | 2 | 3 | 2 |
| 468 | C-terminal-binding protein 1 | IPI00012835 | 0.629 | 0.10924 | 2 | 1 | 3 | 2 |
| 469 | KIAA1033 protein | IPI00298991 | 0.629 | 0.10924 | 1 | 2 | 3 | 2 |
| 470 | Pyridoxal phosphate phosphatase | IPI00025340 | 0.629 | 0.10924 | 2 | 1 | 2 | 3 |
| 471 | Coiled-coil domain-containing protein 134 | IPI00302674 | 0.629 | 0.10924 | 2 | 1 | 3 | 2 |

| No. | Description | Accession number | STN[1] | p-Value[1] | Con_A[2] | Con_B[2] | LUTEO_A[2] | LUTEO_B[2] |
|---|---|---|---|---|---|---|---|---|
| 472 | Isoform 2 of Haloacid dehalogenase-like hydrolase domain-containing protein 2 | IPI00644472 | 0.629 | 0.10924 | 2 | 1 | 3 | 2 |
| 473 | Isoform 1 of ATP-binding cassette sub-family B member 7, mitochondrial | IPI00306748 | 0.629 | 0.10924 | 1 | 2 | 4 | 0 |
| 474 | Protein AATF | IPI00302238 | 0.629 | 0.10924 | 1 | 2 | 4 | 1 |
| 475 | Myosin-Ia | IPI00294386 | 0.629 | 0.10924 | 0 | 2 | 2 | 3 |
| 476 | cDNA FLJ55484, highly similar to ATP-dependent RNA helicase DDX39 | IPI00644431 | 0.629 | 0.10924 | 2 | 1 | 2 | 3 |
| 477 | Isoform A of AP-1 complex subunit beta-1 | IPI00328257 | 0.628 | 0.10924 | 17 | 20 | 21 | 21 |
| 478 | NAD(P)H dehydrogenase [quinone] 1 | IPI00012069 | 0.626 | 0.10933 | 31 | 29 | 33 | 33 |
| 479 | DNA topoisomerase 1 | IPI00413611 | 0.623 | 0.10933 | 12 | 9 | 13 | 12 |
| 480 | Isoform 1 of Cytosolic acyl coenzyme A thioester hydrolase | IPI00010415 | 0.623 | 0.10933 | 11 | 10 | 13 | 12 |
| 481 | Isoleucyl-tRNA synthetase, mitochondrial | IPI00017283 | 0.616 | 0.10981 | 5 | 5 | 5 | 8 |
| 482 | Isoform 1 of Protein fto | IPI00028277 | 0.616 | 0.10981 | 5 | 5 | 8 | 5 |
| 483 | 24 kDa protein | IPI00398057 | 0.616 | 0.10981 | 6 | 4 | 7 | 6 |
| 484 | SDHA protein | IPI00217143 | 0.613 | 0.11109 | 10 | 12 | 13 | 13 |
| 485 | Heat shock protein beta (Fragment) | IPI00411633 | 0.607 | 0.11266 | 32 | 33 | 36 | 35 |
| 486 | 40S ribosomal protein S3a | IPI00419880 | 0.600 | 0.11299 | 50 | 50 | 61 | 46 |
| 487 | DnaJ homolog subfamily C member 13 | IPI00307259 | 0.596 | 0.11299 | 4 | 7 | 5 | 9 |
| 488 | Heat shock 70 kDa protein 14 | IPI00292499 | 0.596 | 0.11299 | 6 | 5 | 7 | 7 |
| 489 | Isoform 1 of NADH-cytochrome b5 reductase 3 | IPI00328415 | 0.594 | 0.11365 | 12 | 12 | 15 | 13 |
| 490 | cDNA FLJ59571, highly similar to Eukaryotic translation initiation factor 4gamma 2 | IPI00015952 | 0.578 | 0.11522 | 4 | 8 | 8 | 7 |
| 491 | cDNA FLJ56414, highly similar to Homo sapiens proline-, glutamic acid-, leucine-rich protein 1 | IPI00006702 | 0.578 | 0.11522 | 5 | 7 | 8 | 7 |
| 492 | Mitochondrial import receptor subunit TOM70 | IPI00015602 | 0.578 | 0.11522 | 6 | 6 | 8 | 7 |
| 493 | Abhydrolase domain-containing protein 10, mitochondrial | IPI00020075 | 0.578 | 0.11522 | 6 | 6 | 7 | 8 |
| 494 | Kinesin-1 heavy chain | IPI00012837 | 0.578 | 0.11522 | 7 | 5 | 8 | 7 |
| 495 | Isoform Long of Double-stranded RNA-binding protein Staufen homolog 1 | IPI00000001 | 0.578 | 0.11522 | 5 | 7 | 8 | 7 |
| 496 | Citrate synthase, mitochondrial | IPI00025366 | 0.576 | 0.11593 | 14 | 12 | 17 | 13 |
| 497 | Telomere length regulation protein TEL2 homolog | IPI00016868 | 0.574 | 0.11679 | 3 | 0 | 3 | 3 |
| 498 | zinc finger protein 294 | IPI00783835 | 0.574 | 0.11679 | 0 | 3 | 1 | 5 |
| 499 | probable E3 ubiquitin-protein ligase MYCBP2 | IPI00289776 | 0.574 | 0.11679 | 2 | 2 | 0 | 5 |
| 500 | Isoform 1 of Growth factor receptor-bound protein 2 | IPI00021327 | 0.574 | 0.11679 | 3 | 1 | 4 | 2 |
| 501 | Wolframin | IPI00008711 | 0.574 | 0.11679 | 2 | 2 | 3 | 3 |
| 502 | WW domain-binding protein 11 | IPI00170786 | 0.574 | 0.11679 | 1 | 3 | 5 | 1 |
| 503 | Isoform 1 of NHL repeat-containing protein 2 | IPI00301051 | 0.574 | 0.11679 | 3 | 1 | 4 | 2 |
| 504 | Thioredoxin domain-containing protein 12 | IPI00026328 | 0.574 | 0.11679 | 3 | 1 | 3 | 3 |
| 505 | Isoform 1 of Alpha-adducin | IPI00019901 | 0.574 | 0.11679 | 1 | 3 | 3 | 3 |
| 506 | Protein FAM162A | IPI00023001 | 0.574 | 0.11679 | 2 | 2 | 4 | 2 |
| 507 | Omega-amidase NIT2 | IPI00549467 | 0.574 | 0.11679 | 2 | 2 | 3 | 3 |
| 508 | Sorting nexin-4 | IPI00029403 | 0.574 | 0.11679 | 1 | 3 | 4 | 2 |
| 509 | Aldo-keto reductase family 1 member C2 | IPI00005668 | 0.574 | 0.11679 | 3 | 1 | 2 | 4 |
| 510 | Scaffold attachment factor B1 | IPI00300631 | 0.574 | 0.11679 | 2 | 2 | 3 | 3 |
| 511 | U3 small nucleolar ribonucleoprotein protein IMP3 | IPI00019488 | 0.574 | 0.11679 | 2 | 2 | 3 | 3 |
| 512 | Isoform 1 of Phosphatidate cytidylyltransferase 2 | IPI00032150 | 0.574 | 0.11679 | 3 | 1 | 2 | 4 |
| 513 | Translocated promoter region | IPI00514531 | 0.574 | 0.11679 | 2 | 2 | 3 | 3 |
| 514 | cDNA FLJ56389, highly similar to Elongation factor 1-gamma | IPI00000875 | 0.570 | 0.13535 | 26 | 22 | 28 | 25 |
| 515 | Isoform 1 of UTP--glucose-1-phosphate uridylyltransferase | IPI00329331 | 0.568 | 0.13549 | 11 | 16 | 16 | 15 |
| 516 | Neurolysin, mitochondrial | IPI00010346 | 0.562 | 0.13563 | 4 | 9 | 7 | 9 |
| 517 | Isoform Mitochondrial of Fumarate hydratase, mitochondrial | IPI00296053 | 0.562 | 0.13563 | 7 | 6 | 7 | 9 |
| 518 | Putative pre-mRNA-splicing factor ATP-dependent RNA helicase DHX15 | IPI00396435 | 0.561 | 0.13668 | 25 | 25 | 29 | 26 |
| 519 | T-complex protein 1 subunit zeta | IPI00027626 | 0.554 | 0.13772 | 64 | 59 | 63 | 67 |
| 520 | ATP-dependent DNA helicase Q1 | IPI00178431 | 0.553 | 0.13777 | 14 | 15 | 18 | 15 |
| 521 | Phosphatidylethanolamine-binding protein 1 | IPI00219446 | 0.553 | 0.13777 | 17 | 12 | 15 | 18 |
| 522 | Nuclear pore complex protein Nup93 | IPI00397904 | 0.547 | 0.13806 | 7 | 7 | 10 | 7 |
| 523 | Importin subunit alpha-4 | IPI00012578 | 0.547 | 0.13806 | 6 | 8 | 8 | 9 |
| 524 | 482 kDa protein | IPI00179298 | 0.543 | 0.13948 | 46 | 41 | 50 | 43 |
| 525 | Mitochondrial 28S ribosomal protein S2 | IPI00006970 | 0.533 | 0.14005 | 8 | 7 | 8 | 10 |
| 526 | Splicing factor 3A subunit 3 | IPI00029764 | 0.533 | 0.14057 | 17 | 15 | 18 | 18 |
| 527 | Isoform 2 of Syntaxin-binding protein 1 | IPI00046057 | 0.533 | 0.14133 | 3 | 2 | 4 | 3 |
| 528 | 39S ribosomal protein L28, mitochondrial | IPI00172594 | 0.533 | 0.14133 | 2 | 3 | 3 | 4 |
| 529 | Isoform A of Ras-related C3 botulinum toxin substrate 1 | IPI00010271 | 0.533 | 0.14133 | 3 | 2 | 4 | 3 |
| 530 | 28S ribosomal protein S28, mitochondrial | IPI00022276 | 0.533 | 0.14133 | 1 | 4 | 4 | 3 |
| 531 | LDLR chaperone MESD | IPI00399089 | 0.533 | 0.14133 | 3 | 2 | 4 | 3 |
| 532 | Isoform 1 of Exosome component 10 | IPI00009464 | 0.533 | 0.14133 | 2 | 3 | 3 | 4 |
| 533 | U6 snRNA-associated Sm-like protein LSm7 | IPI00007163 | 0.533 | 0.14133 | 3 | 2 | 3 | 4 |
| 534 | 60S ribosomal protein L17 | IPI00413324 | 0.533 | 0.14133 | 2 | 3 | 3 | 4 |
| 535 | Putative uncharacterized protein KIAA0664 | IPI00024425 | 0.527 | 0.14171 | 18 | 15 | 17 | 20 |
| 536 | Collapsin response mediator protein 4 long variant | IPI00029111 | 0.527 | 0.14171 | 16 | 17 | 19 | 18 |
| 537 | Protein tyrosine phosphatase-like protein PTPLAD1 | IPI00008998 | 0.521 | 0.14238 | 8 | 8 | 7 | 12 |
| 538 | Isoform 1 of Adenylate kinase 2, mitochondrial | IPI00215901 | 0.521 | 0.14238 | 7 | 9 | 9 | 10 |
| 539 | Isoform 1 of Carnitine O-palmitoyltransferase 1, liver isoform | IPI00032038 | 0.521 | 0.14238 | 9 | 7 | 10 | 9 |
| 540 | Isoform 1 of Methylcrotonoyl-CoA carboxylase beta chain, mitochondrial | IPI00784044 | 0.510 | 0.14285 | 17 | 19 | 22 | 18 |
| 541 | Thymidine kinase, cytosolic | IPI00299214 | 0.501 | 0.14394 | 2 | 4 | 2 | 6 |
| 542 | Isoform Short of TATA-binding protein-associated factor 2N | IPI00020194 | 0.501 | 0.14394 | 2 | 4 | 5 | 3 |
| 543 | Isoform 1 of Coiled-coil domain-containing protein 109A | IPI00171573 | 0.501 | 0.14394 | 3 | 3 | 4 | 4 |
| 544 | Sorting nexin-2 | IPI00299095 | 0.501 | 0.14394 | 4 | 2 | 4 | 4 |
| 545 | Quinone oxidoreductase | IPI00000792 | 0.501 | 0.14394 | 3 | 3 | 3 | 5 |
| 546 | Calcium-binding protein 39 | IPI00032561 | 0.501 | 0.14394 | 4 | 2 | 3 | 5 |
| 547 | Adenylosuccinate synthetase isozyme 2 | IPI00026833 | 0.501 | 0.14394 | 3 | 3 | 4 | 4 |
| 548 | Oxysterol-binding protein | IPI00163644 | 0.501 | 0.14394 | 3 | 3 | 4 | 4 |
| 549 | Isoform CNPI of 2',3'-cyclic-nucleotide 3'-phosphodiesterase | IPI00220993 | 0.501 | 0.14394 | 4 | 2 | 4 | 4 |
| 550 | Polyribonucleotide nucleotidyltransferase 1, mitochondrial | IPI00744711 | 0.499 | 0.15752 | 8 | 10 | 13 | 8 |
| 551 | FACT complex subunit SPT16 | IPI00026970 | 0.476 | 0.16027 | 34 | 43 | 40 | 42 |
| 552 | Isoform 2 of AP-2 complex subunit alpha-2 | IPI00016621 | 0.475 | 0.16027 | 5 | 2 | 7 | 2 |
| 553 | Isoform 1 of 60S ribosome subunit biogenesis protein NIP7 homolog | IPI00007175 | 0.475 | 0.16027 | 6 | 1 | 4 | 5 |
| 554 | WASH complex subunit strumpellin | IPI00029175 | 0.475 | 0.16027 | 3 | 4 | 4 | 5 |
| 555 | Isoform 1 of Replication protein A 32 kDa subunit | IPI00013939 | 0.475 | 0.16027 | 4 | 3 | 5 | 4 |
| 556 | Protein NipSnap homolog 1 | IPI00304435 | 0.475 | 0.16027 | 3 | 4 | 4 | 5 |
| 557 | Tetratricopeptide repeat protein 35 | IPI00014149 | 0.475 | 0.16027 | 2 | 5 | 5 | 4 |
| 558 | L antigen family member 3 | IPI00032314 | 0.475 | 0.16027 | 4 | 3 | 5 | 4 |
| 559 | NADH dehydrogenase [ubiquinone] 1 alpha subcomplex subunit 10, mitochondrial | IPI00029561 | 0.475 | 0.16027 | 6 | 1 | 5 | 4 |
| 560 | Isoform 1 of STE20-like serine/threonine-protein kinase | IPI00022827 | 0.475 | 0.16027 | 3 | 4 | 4 | 5 |
| 561 | Transmembrane emp24 domain-containing protein 5 | IPI00294472 | 0.475 | 0.16027 | 5 | 2 | 6 | 3 |
| 562 | Transcription factor A, mitochondrial | IPI00020928 | 0.475 | 0.16027 | 4 | 3 | 5 | 4 |
| 563 | Eukaryotic translation initiation factor 4E | IPI00027485 | 0.475 | 0.16027 | 4 | 3 | 5 | 4 |
| 564 | Isoform Long of Transformer-2 protein homolog alpha | IPI00013891 | 0.475 | 0.16027 | 3 | 4 | 4 | 5 |
| 565 | serine/threonine-protein phosphatase PP1-alpha catalytic subunit isoform 3 | IPI00027423 | 0.475 | 0.16027 | 4 | 3 | 5 | 4 |
| 566 | Isoform A of Phosphate carrier protein, mitochondrial | IPI00022202 | 0.473 | 0.16056 | 25 | 19 | 25 | 23 |

| No. | Description | Accession number | STN[1] | p-Value[1] | Con_A[2] | Con_B[2] | LUTEO_A[2] | LUTEO_B[2] |
|---|---|---|---|---|---|---|---|---|
| 567 | Isoform 1 of ATP-dependent RNA helicase DDX19B | IPI00008943 | 0.471 | 0.16113 | 9 | 12 | 13 | 11 |
| 568 | cysteinyl-tRNA synthetase, cytoplasmic isoform c | IPI00027443 | 0.471 | 0.16113 | 13 | 8 | 14 | 10 |
| 569 | Nuclear pore complex protein Nup205 | IPI00783781 | 0.466 | 0.16175 | 38 | 43 | 50 | 36 |
| 570 | Pre-mRNA-processing factor 19 | IPI00004968 | 0.463 | 0.16208 | 12 | 10 | 13 | 12 |
| 571 | Isoform 1 of Symplekin | IPI00023344 | 0.463 | 0.16208 | 12 | 10 | 12 | 13 |
| 572 | Isoform 1 of Poly(U)-binding-splicing factor PUF60 | IPI00069750 | 0.463 | 0.16208 | 11 | 11 | 13 | 12 |
| 573 | Sideroflexin-1 | IPI00009368 | 0.463 | 0.16208 | 9 | 13 | 14 | 11 |
| 574 | Superkiller viralicidic activity 2-like 2 | IPI00647217 | 0.456 | 0.16336 | 12 | 11 | 11 | 15 |
| 575 | Isoform 2 of Dedicator of cytokinesis protein 7 | IPI00183572 | 0.453 | 0.16398 | 3 | 5 | 7 | 3 |
| 576 | Aminoacyl tRNA synthase complex-interacting multifunctional protein 1 | IPI00006252 | 0.453 | 0.16398 | 4 | 4 | 4 | 6 |
| 577 | Isoform 1 of Cleavage stimulation factor subunit 2 | IPI00013256 | 0.453 | 0.16398 | 5 | 3 | 5 | 5 |
| 578 | Isoform 1 of CCR4-NOT transcription complex subunit 1 | IPI00166010 | 0.453 | 0.16398 | 3 | 5 | 4 | 6 |
| 579 | Isoform 2 of Beta-catenin-like protein 1 | IPI00472981 | 0.453 | 0.16398 | 4 | 4 | 5 | 5 |
| 580 | Solute carrier family 4 sodium bicarbonate cotransporter member 7 | IPI00021058 | 0.453 | 0.16398 | 4 | 4 | 7 | 3 |
| 581 | Isoform 1 of Ras-related protein Rab-6A | IPI00023526 | 0.453 | 0.16398 | 3 | 5 | 5 | 5 |
| 582 | Isoform 2 of Ubiquinol-cytochrome c reductase complex chaperone CBP3 homolog | IPI00219889 | 0.453 | 0.16398 | 4 | 4 | 5 | 5 |
| 583 | Isoform 2 of Mitochondrial import inner membrane translocase subunit TIM50 | IPI00418497 | 0.453 | 0.16398 | 4 | 4 | 4 | 6 |
| 584 | 14-3-3 protein epsilon | IPI00000816 | 0.447 | 0.17395 | 147 | 153 | 152 | 156 |
| 585 | Nucleolar protein 11 | IPI00303813 | 0.434 | 0.17509 | 5 | 4 | 7 | 4 |
| 586 | CTP synthase 2 | IPI00645702 | 0.434 | 0.17509 | 4 | 5 | 6 | 5 |
| 587 | Isoform 1 of Translocon-associated protein subunit alpha | IPI00301021 | 0.434 | 0.17509 | 3 | 6 | 7 | 4 |
| 588 | TRMT61A protein (Fragment) | IPI00059718 | 0.434 | 0.17509 | 5 | 4 | 5 | 6 |
| 589 | Isoform 1 of Vesicle-associated membrane protein-associated protein A | IPI00170692 | 0.434 | 0.17509 | 5 | 4 | 5 | 6 |
| 590 | Transaldolase | IPI00744692 | 0.428 | 0.17528 | 31 | 26 | 24 | 37 |
| 591 | Eukaryotic translation initiation factor 1A, Y-chromosomal | IPI00023004 | 0.423 | 0.17547 | 14 | 14 | 15 | 16 |
| 592 | Malectin | IPI00029046 | 0.418 | 0.17585 | 6 | 4 | 4 | 8 |
| 593 | Peroxisomal membrane protein 2 | IPI00221002 | 0.418 | 0.17585 | 5 | 5 | 6 | 6 |
| 594 | Ubiquitin carboxyl-terminal hydrolase 14 | IPI00219913 | 0.418 | 0.18468 | 17 | 12 | 17 | 15 |
| 595 | Non-POU domain-containing octamer-binding protein | IPI00304596 | 0.417 | 0.18487 | 55 | 53 | 62 | 51 |
| 596 | Isoform 1 of Importin-5 | IPI00793443 | 0.407 | 0.18543 | 34 | 31 | 32 | 37 |
| 597 | GTP-binding nuclear protein Ran | IPI00643041 | 0.407 | 0.18543 | 16 | 15 | 15 | 19 |
| 598 | Ras-related protein Rab-1B | IPI00008964 | 0.404 | 0.18567 | 5 | 6 | 5 | 8 |
| 599 | Gamma-aminobutyric acid receptor-associated protein-like 2 | IPI00026358 | 0.404 | 0.18567 | 5 | 6 | 7 | 6 |
| 600 | Isoform A of Ras GTPase-activating protein-binding protein 2 | IPI00009057 | 0.404 | 0.18567 | 6 | 5 | 8 | 5 |
| 601 | cDNA FLJ54492, highly similar to Eukaryotic translation initiation factor 4B | IPI00012079 | 0.402 | 0.18567 | 17 | 15 | 16 | 19 |
| 602 | T-complex protein 1 subunit delta | IPI00302927 | 0.391 | 0.18662 | 40 | 32 | 43 | 33 |
| 603 | Cytoplasmic aconitate hydratase | IPI00008485 | 0.391 | 0.18662 | 7 | 5 | 7 | 7 |
| 604 | Tubulin-specific chaperone A | IPI00217236 | 0.391 | 0.18662 | 7 | 5 | 7 | 7 |
| 605 | Ras-related protein Rab-6B | IPI00016891 | 0.391 | 0.18662 | 7 | 5 | 8 | 6 |
| 606 | Isoform Long of Ubiquitin carboxyl-terminal hydrolase 5 | IPI00024664 | 0.389 | 0.19260 | 18 | 17 | 19 | 19 |
| 607 | Short heat shock protein 60 Hsp60s2 | IPI00076042 | 0.385 | 0.19322 | 31 | 44 | 32 | 47 |
| 608 | Vimentin | IPI00418471 | 0.385 | 0.19322 | 37 | 38 | 42 | 37 |
| 609 | Isoform 2 of Serine/threonine-protein phosphatase PGAM5, mitochondrial | IPI00063242 | 0.380 | 0.19412 | 8 | 5 | 8 | 7 |
| 610 | 60S ribosomal protein L22 | IPI00219153 | 0.380 | 0.19412 | 7 | 6 | 8 | 7 |
| 611 | Transmembrane protein 126A | IPI00031064 | 0.380 | 0.19412 | 6 | 7 | 9 | 6 |
| 612 | Isoform 1 of Long-chain-fatty-acid--CoA ligase 1 | IPI00012728 | 0.378 | 0.19412 | 0 | 1 | 2 | 0 |
| 613 | Isoform 1 of Minor histocompatibility antigen H13 | IPI00152441 | 0.378 | 0.19412 | 0 | 0 | 2 | 1 |
| 614 | Vacuolar protein sorting-associated protein 4B | IPI00182728 | 0.378 | 0.19412 | 1 | 1 | 2 | 0 |
| 615 | ATP-dependent RNA helicase DDX24 | IPI00006987 | 0.378 | 0.19412 | 0 | 0 | 1 | 2 |
| 616 | coatomer subunit epsilon isoform c | IPI00399319 | 0.378 | 0.19412 | 0 | 1 | 2 | 1 |
| 617 | Endoplasmic reticulum resident protein 44 | IPI00401264 | 0.378 | 0.19412 | 1 | 1 | 1 | 2 |
| 618 | Kinetochore-associated protein 1 | IPI00001458 | 0.378 | 0.19412 | 0 | 0 | 1 | 2 |
| 619 | 28S ribosomal protein S7, mitochondrial | IPI00006440 | 0.378 | 0.19412 | 1 | 0 | 2 | 1 |
| 620 | Isoform 1 of Mesoderm-specific transcript homolog protein | IPI00298947 | 0.378 | 0.19412 | 1 | 1 | 2 | 1 |
| 621 | SNARE-associated protein Snapin | IPI00018331 | 0.378 | 0.19412 | 1 | 1 | 2 | 1 |
| 622 | Isoform 1 of Telomeric repeat-binding factor 2 | IPI00024214 | 0.378 | 0.19412 | 0 | 1 | 2 | 1 |
| 623 | Isoform B of AP-2 complex subunit alpha-1 | IPI00256684 | 0.378 | 0.19412 | 0 | 0 | 1 | 2 |
| 624 | Golgi phosphoprotein 3-like | IPI00012313 | 0.378 | 0.19412 | 1 | 1 | 0 | 2 |
| 625 | Pre-mRNA-splicing factor CWC22 homolog | IPI00177381 | 0.378 | 0.19412 | 1 | 1 | 1 | 2 |
| 626 | WD repeat-containing protein 11 | IPI00412224 | 0.378 | 0.19412 | 0 | 1 | 1 | 2 |
| 627 | Peptidyl-prolyl cis-trans isomerase F, mitochondrial | IPI00026519 | 0.378 | 0.19412 | 1 | 0 | 2 | 1 |
| 628 | Isoform 1 of TBC1 domain family member 9B | IPI00307257 | 0.378 | 0.19412 | 1 | 1 | 2 | 1 |
| 629 | Transmembrane protein 11 | IPI00012855 | 0.378 | 0.19412 | 1 | 1 | 0 | 2 |
| 630 | Isoform 1 of Caldesmon | IPI00014516 | 0.378 | 0.19412 | 1 | 1 | 2 | 1 |
| 631 | Isoform 2 of Calpastatin | IPI00220857 | 0.378 | 0.19412 | 1 | 0 | 0 | 2 |
| 632 | Isoform 1 of Regulator of nonsense transcripts 2 | IPI00300504 | 0.378 | 0.19412 | 1 | 1 | 2 | 1 |
| 633 | Glutamate--cysteine ligase regulatory subunit | IPI00010090 | 0.378 | 0.19412 | 0 | 1 | 2 | 0 |
| 634 | Isoform 2 of Basic leucine zipper and W2 domain-containing protein 1 | IPI00180128 | 0.378 | 0.19412 | 1 | 1 | 1 | 2 |
| 635 | Isoform 1 of L-2-hydroxyglutarate dehydrogenase, mitochondrial | IPI00016458 | 0.378 | 0.19412 | 1 | 1 | 2 | 0 |
| 636 | NF-kappa-B-repressing factor | IPI00005675 | 0.378 | 0.19412 | 1 | 1 | 1 | 2 |
| 637 | AP-3 complex subunit mu-1 | IPI00032459 | 0.378 | 0.19412 | 1 | 1 | 2 | 1 |
| 638 | Myosin-Id | IPI00329719 | 0.378 | 0.19412 | 1 | 1 | 2 | 0 |
| 639 | Isoform 2 of Gamma-adducin | IPI00004408 | 0.378 | 0.19412 | 1 | 1 | 2 | 0 |
| 640 | KDEL motif-containing protein 1 | IPI00005270 | 0.378 | 0.19412 | 1 | 1 | 2 | 1 |
| 641 | Ras-related protein Rab-9A | IPI00016372 | 0.378 | 0.19412 | 1 | 0 | 1 | 2 |
| 642 | Isoform 1 of Malonyl-CoA-acyl carrier protein transacylase, mitochondrial | IPI00023359 | 0.378 | 0.19412 | 0 | 1 | 1 | 2 |
| 643 | Pre-mRNA-splicing factor SYF1 | IPI00163084 | 0.378 | 0.19412 | 1 | 1 | 0 | 2 |
| 644 | Dolichyl-phosphate beta-glucosyltransferase | IPI00002506 | 0.378 | 0.19412 | 1 | 0 | 0 | 2 |
| 645 | Acetyl-coenzyme A synthetase, cytoplasmic | IPI00413730 | 0.378 | 0.19412 | 1 | 1 | 1 | 2 |
| 646 | Isoform 1 of Cell division protein kinase 9 | IPI00301923 | 0.378 | 0.19412 | 0 | 0 | 1 | 2 |
| 647 | 204 kDa protein | IPI00158296 | 0.378 | 0.19412 | 0 | 0 | 2 | 1 |
| 648 | Isoform 1 of GH3 domain-containing protein | IPI00427501 | 0.378 | 0.19412 | 0 | 0 | 1 | 2 |
| 649 | Isoform 1 of TP53RK-binding protein | IPI00301432 | 0.378 | 0.19412 | 1 | 0 | 2 | 1 |
| 650 | Aspartyl/asparaginyl beta-hydroxylase | IPI00294834 | 0.378 | 0.19412 | 0 | 1 | 2 | 1 |
| 651 | Isoform 1 of Elongation factor Tu GTP-binding domain-containing protein 1 | IPI00293026 | 0.378 | 0.19412 | 0 | 1 | 2 | 1 |
| 652 | Uncharacterized protein C20orf72 | IPI00001287 | 0.378 | 0.19412 | 1 | 1 | 2 | 1 |
| 653 | cDNA FLJ56443, highly similar to Putative ATP-dependent RNA helicase DHX33 | IPI00302860 | 0.378 | 0.19412 | 1 | 0 | 2 | 1 |
| 654 | X-prolyl aminopeptidase (Aminopeptidase P) 1, soluble | IPI00514564 | 0.378 | 0.19412 | 1 | 1 | 2 | 1 |
| 655 | TATA box-binding protein-like protein 1 | IPI00032911 | 0.378 | 0.19412 | 1 | 1 | 2 | 1 |
| 656 | Isoform 1 of Rab3 GTPase-activating protein catalytic subunit | IPI00014235 | 0.378 | 0.19412 | 1 | 1 | 2 | 1 |
| 657 | Isoform 7 of Serine/threonine-protein kinase MARK2 | IPI00290158 | 0.378 | 0.19412 | 1 | 0 | 2 | 0 |
| 658 | UPF0554 protein C2orf43 | IPI00030257 | 0.378 | 0.19412 | 0 | 0 | 2 | 1 |
| 659 | cDNA FLJ45232 fis, clone BRCAN2021718 | IPI00170877 | 0.378 | 0.19412 | 1 | 0 | 0 | 2 |
| 660 | Isoform 1 of WD repeat-containing protein 74 | IPI00018192 | 0.378 | 0.19412 | 1 | 1 | 1 | 2 |
| 661 | Golgin subfamily A member 7 | IPI00480022 | 0.378 | 0.19412 | 1 | 1 | 0 | 2 |

| No. | Description | Accession number | STN[1] | p-Value[1] | Con_A[2] | Con_B[2] | LUTEO_A[2] | LUTEO_B[2] |
|---|---|---|---|---|---|---|---|---|
| 662 | Dolichyl-diphosphooligosaccharide--protein glycosyltransferase subunit DAD1 | IPI00009407 | 0.378 | 0.19412 | 1 | 1 | 2 | 1 |
| 663 | Zinc finger CCCH domain-containing protein 11A | IPI00328306 | 0.378 | 0.19412 | 0 | 0 | 2 | 1 |
| 664 | Nucleoporin NUP53 | IPI00329650 | 0.378 | 0.19412 | 1 | 1 | 2 | 1 |
| 665 | Profilin | IPI00107555 | 0.378 | 0.19412 | 1 | 1 | 2 | 1 |
| 666 | Isoform 1 of DnaJ homolog subfamily C member 10 | IPI00293260 | 0.378 | 0.19412 | 0 | 1 | 2 | 0 |
| 667 | Isoform 1 of Cohesin loading complex subunit SCC4 homolog | IPI00000656 | 0.378 | 0.19412 | 1 | 0 | 1 | 2 |
| 668 | Ras-related protein Rab-32 | IPI00014377 | 0.378 | 0.19412 | 1 | 1 | 2 | 1 |
| 669 | Isoform 2 of Plakophilin-2 | IPI00005264 | 0.378 | 0.19412 | 0 | 0 | 1 | 2 |
| 670 | Vacuolar protein sorting-associated protein 33B | IPI00032905 | 0.378 | 0.19412 | 1 | 1 | 2 | 1 |
| 671 | Isoform 2 of N-acetylserotonin O-methyltransferase-like protein | IPI00249080 | 0.378 | 0.19412 | 1 | 1 | 2 | 1 |
| 672 | Beclin-1 | IPI00748342 | 0.378 | 0.19412 | 1 | 1 | 1 | 2 |
| 673 | Rhomboid domain-containing protein 2 | IPI00010255 | 0.378 | 0.19412 | 1 | 0 | 2 | 1 |
| 674 | Methylcrotonoyl-CoA carboxylase subunit alpha, mitochondrial | IPI00024580 | 0.378 | 0.19412 | 1 | 1 | 2 | 0 |
| 675 | Neighbor of COX4 | IPI00005740 | 0.378 | 0.19412 | 0 | 0 | 2 | 0 |
| 676 | Isoform 1 of Dehydrogenase/reductase SDR family member 7 | IPI00006957 | 0.378 | 0.19412 | 1 | 0 | 2 | 1 |
| 677 | Sperm-associated antigen 7 | IPI00006863 | 0.378 | 0.19412 | 0 | 1 | 0 | 2 |
| 678 | Isoform 1 of Vacuolar protein sorting-associated protein 16 homolog | IPI00305438 | 0.378 | 0.19412 | 1 | 1 | 2 | 1 |
| 679 | Isoform 1 of Zinc finger CCCH-type antiviral protein 1 | IPI00410067 | 0.378 | 0.19412 | 1 | 0 | 2 | 1 |
| 680 | Isoform 4 of Phosphorylase b kinase regulatory subunit beta | IPI00181893 | 0.378 | 0.19412 | 1 | 0 | 2 | 1 |
| 681 | Isoform 2 of Protein transport protein Sec24A | IPI00178798 | 0.378 | 0.19412 | 0 | 0 | 1 | 2 |
| 682 | MORF4 family-associated protein 1 | IPI00020915 | 0.378 | 0.19412 | 1 | 1 | 0 | 2 |
| 683 | Protein disulfide-isomerase A5 | IPI00031479 | 0.378 | 0.19412 | 0 | 0 | 2 | 1 |
| 684 | Coiled-coil domain-containing protein 25 | IPI00396174 | 0.378 | 0.19412 | 0 | 0 | 0 | 2 |
| 685 | Isoform 3 of Protein DDI1 homolog 2 | IPI00031618 | 0.378 | 0.19412 | 0 | 1 | 0 | 2 |
| 686 | Prolactin regulatory element-binding protein | IPI00033349 | 0.378 | 0.19412 | 1 | 1 | 2 | 0 |
| 687 | FtsJ methyltransferase domain-containing protein 1 | IPI00334846 | 0.378 | 0.19412 | 0 | 1 | 0 | 2 |
| 688 | cDNA, FLJ79184, highly similar to Procollagen-lysine, 2-oxoglutarate 5-dioxygenase 1 | IPI00027192 | 0.378 | 0.19412 | 0 | 0 | 1 | 2 |
| 689 | Acylphosphatase-1 | IPI00221117 | 0.378 | 0.19412 | 1 | 1 | 2 | 0 |
| 690 | Isoform 1 of Serine protease HTRA2, mitochondrial | IPI00001663 | 0.378 | 0.19412 | 1 | 0 | 1 | 2 |
| 691 | General transcription factor IIF subunit 2 | IPI00477686 | 0.378 | 0.19412 | 0 | 0 | 2 | 1 |
| 692 | Diphosphomevalonate decarboxylase | IPI00022745 | 0.378 | 0.19412 | 1 | 1 | 2 | 1 |
| 693 | Isoform 1 of Polyglutamine-binding protein 1 | IPI00024698 | 0.378 | 0.19412 | 0 | 1 | 2 | 1 |
| 694 | Endonuclease/exonuclease/phosphatase family domain-containing protein 1 | IPI00885036 | 0.378 | 0.19412 | 1 | 0 | 2 | 0 |
| 695 | Isoform 2 of General transcription factor 3C polypeptide 5 | IPI00411531 | 0.378 | 0.19412 | 1 | 0 | 2 | 1 |
| 696 | Isoform 2 of Suppressor of SWI4 1 homolog | IPI00219793 | 0.378 | 0.19412 | 0 | 1 | 1 | 2 |
| 697 | Ubiquitin-conjugating enzyme E2 S | IPI00217949 | 0.378 | 0.19412 | 1 | 0 | 2 | 0 |
| 698 | Isoform 5 of Methyltransferase-like protein 13 | IPI00384061 | 0.378 | 0.19412 | 0 | 0 | 2 | 1 |
| 699 | Isoform 1 of Oxysterol-binding protein-related protein 2 | IPI00014137 | 0.378 | 0.19412 | 0 | 0 | 2 | 0 |
| 700 | 1-acyl-sn-glycerol-3-phosphate acyltransferase epsilon | IPI00028491 | 0.378 | 0.19412 | 0 | 0 | 2 | 1 |
| 701 | Isoform L1 of Smoothelin | IPI00219460 | 0.378 | 0.19412 | 0 | 0 | 1 | 2 |
| 702 | Ras-related protein Rab-39B | IPI00060801 | 0.378 | 0.19412 | 0 | 0 | 0 | 2 |
| 703 | Isoform 1 of DNA repair protein RAD50 | IPI00305282 | 0.378 | 0.19412 | 0 | 0 | 2 | 0 |
| 704 | Solute carrier family 2, facilitated glucose transporter member 1 | IPI00220194 | 0.377 | 0.21563 | 19 | 19 | 22 | 19 |
| 705 | Gamma-glutamyl hydrolase | IPI00023728 | 0.373 | 0.21582 | 19 | 20 | 22 | 20 |
| 706 | Acetyl-CoA acetyltransferase, mitochondrial | IPI00030363 | 0.373 | 0.21582 | 19 | 20 | 21 | 21 |
| 707 | CSNK2A1 protein | IPI00016613 | 0.369 | 0.21653 | 6 | 8 | 8 | 8 |
| 708 | Isoform 1 of Malignant T cell-amplified sequence 1 | IPI00179026 | 0.369 | 0.21653 | 8 | 6 | 10 | 6 |
| 709 | ATP-dependent RNA helicase DDX50 | IPI00031554 | 0.369 | 0.21653 | 6 | 8 | 10 | 6 |
| 710 | TOB3 | IPI00045921 | 0.369 | 0.21653 | 7 | 7 | 8 | 8 |
| 711 | Isoform 1 of Squamous cell carcinoma antigen recognized by T-cells 3 | IPI00006025 | 0.369 | 0.21653 | 10 | 4 | 10 | 6 |
| 712 | Isoform 1 of 1-phosphatidylinositol-4,5-bisphosphate phosphodiesterase gamma-1 | IPI00016736 | 0.369 | 0.21653 | 4 | 10 | 8 | 8 |
| 713 | V-type proton ATPase catalytic subunit A | IPI00007682 | 0.366 | 0.22128 | 24 | 17 | 26 | 18 |
| 714 | Isoform 1 of Protein KIAA1967 | IPI00182757 | 0.360 | 0.22185 | 7 | 8 | 9 | 8 |
| 715 | L-xylulose reductase | IPI00448095 | 0.360 | 0.22185 | 6 | 9 | 9 | 8 |
| 716 | Peptidyl-prolyl cis-trans isomerase FKBP11 | IPI00009885 | 0.360 | 0.22185 | 7 | 8 | 10 | 7 |
| 717 | Ubiquitin-like modifier activating enzyme 1 | IPI00552452 | 0.360 | 0.22185 | 6 | 9 | 11 | 6 |
| 718 | Sorbitol dehydrogenase | IPI00216057 | 0.360 | 0.22185 | 9 | 6 | 8 | 9 |
| 719 | cDNA FLJ56425, highly similar to Very-long-chain specific acyl-CoAdehydrogenase, mitochondrial | IPI00028031 | 0.353 | 0.22223 | 21 | 24 | 25 | 23 |
| 720 | 60S ribosomal protein L21 | IPI00247583 | 0.351 | 0.22261 | 7 | 9 | 9 | 9 |
| 721 | X-ray repair cross-complementing protein 5 | IPI00220834 | 0.347 | 0.22769 | 91 | 82 | 92 | 86 |
| 722 | Small nuclear ribonucleoprotein Sm D1 | IPI00302850 | 0.344 | 0.22769 | 23 | 25 | 27 | 24 |
| 723 | probable ubiquitin carboxyl-terminal hydrolase FAF-X isoform 4 | IPI00003964 | 0.343 | 0.22783 | 7 | 10 | 11 | 8 |
| 724 | Translocon-associated protein subunit delta precursor | IPI00019385 | 0.343 | 0.22783 | 8 | 9 | 10 | 9 |
| 725 | Isoform Long of Sodium/potassium-transporting ATPase subunit alpha-1 | IPI00006482 | 0.337 | 0.22849 | 25 | 26 | 27 | 27 |
| 726 | 40S ribosomal protein S19 | IPI00215780 | 0.336 | 0.22859 | 11 | 7 | 11 | 9 |
| 727 | Enoyl-CoA hydratase, mitochondrial | IPI00024993 | 0.336 | 0.22859 | 9 | 9 | 9 | 11 |
| 728 | Proteasome 26S non-ATPase subunit 11 variant (Fragment) | IPI00105598 | 0.336 | 0.22859 | 10 | 8 | 10 | 10 |
| 729 | Isoform 2 of Double-stranded RNA-specific adenosine deaminase | IPI00025057 | 0.330 | 0.23148 | 1 | 2 | 2 | 2 |
| 730 | Reticulocalbin-1 | IPI00015842 | 0.330 | 0.23148 | 1 | 2 | 1 | 3 |
| 731 | Putative uncharacterized protein DKFZp686E2459 | IPI00375731 | 0.330 | 0.23148 | 2 | 0 | 2 | 2 |
| 732 | DNA polymerase | IPI00744598 | 0.330 | 0.23148 | 1 | 2 | 2 | 2 |
| 733 | Isoform 1 of BH3-interacting domain death agonist | IPI00413587 | 0.330 | 0.23148 | 1 | 2 | 2 | 2 |
| 734 | PRMT3 protein (Fragment) | IPI00103026 | 0.330 | 0.23148 | 2 | 1 | 3 | 0 |
| 735 | Isoform 1 of Putative splicing factor, arginine/serine-rich 14 | IPI00158020 | 0.330 | 0.23148 | 1 | 2 | 2 | 2 |
| 736 | cDNA FLJ52741, highly similar to Mus musculus adaptor-related protein complex 1 | IPI00009244 | 0.330 | 0.23148 | 2 | 1 | 2 | 2 |
| 737 | Isoform 1 of Protein phosphatase methylesterase 1 | IPI00007694 | 0.330 | 0.23148 | 0 | 2 | 3 | 1 |
| 738 | Retinol dehydrogenase 14 | IPI00177940 | 0.330 | 0.23148 | 1 | 2 | 2 | 2 |
| 739 | Isoform 2 of WASH complex subunit 7 | IPI00164930 | 0.330 | 0.23148 | 1 | 2 | 3 | 1 |
| 740 | ATPase ASNA1 | IPI00013466 | 0.330 | 0.23148 | 0 | 2 | 3 | 1 |
| 741 | 39S ribosomal protein L40, mitochondrial | IPI00099871 | 0.330 | 0.23148 | 2 | 1 | 1 | 3 |
| 742 | Argininosuccinate synthase | IPI00020632 | 0.330 | 0.23148 | 0 | 2 | 2 | 2 |
| 743 | Heat shock protein beta-11 | IPI00098827 | 0.330 | 0.23148 | 2 | 1 | 2 | 2 |
| 744 | Isoform 1 of Putative RNA-binding protein 15 | IPI00102752 | 0.330 | 0.23148 | 1 | 2 | 2 | 2 |
| 745 | U6 snRNA-associated Sm-like protein LSm1 | IPI00004436 | 0.330 | 0.23148 | 0 | 2 | 3 | 1 |
| 746 | Cation-dependent mannose-6-phosphate receptor | IPI00025049 | 0.330 | 0.23148 | 2 | 1 | 1 | 3 |
| 747 | cDNA FLJ56343, highly similar to Torsin A | IPI00413293 | 0.330 | 0.23148 | 1 | 2 | 3 | 1 |
| 748 | D-dopachrome decarboxylase | IPI00293867 | 0.330 | 0.23148 | 1 | 2 | 3 | 1 |
| 749 | GDP-L-fucose synthase | IPI00014361 | 0.330 | 0.23148 | 2 | 0 | 2 | 2 |
| 750 | Ribonuclease inhibitor | IPI00550069 | 0.330 | 0.23148 | 2 | 1 | 2 | 2 |
| 751 | 5'-nucleotidase domain-containing protein 1 | IPI00177965 | 0.330 | 0.23148 | 1 | 2 | 2 | 2 |
| 752 | Isoform 1 of Origin recognition complex subunit 3 | IPI00294402 | 0.330 | 0.23148 | 1 | 2 | 0 | 3 |
| 753 | Lysophospholipid acyltransferase 5 | IPI00306419 | 0.330 | 0.23148 | 1 | 2 | 2 | 2 |
| 754 | Isoform 5 of Brain-specific angiogenesis inhibitor 1-associated protein 2 | IPI00180292 | 0.330 | 0.23148 | 1 | 2 | 2 | 2 |
| 755 | Isoform 1 of Metaxin-1 | IPI00013678 | 0.330 | 0.23148 | 1 | 2 | 2 | 2 |
| 756 | Isoform 1 of Mammalian ependymin-related protein 1 | IPI00259102 | 0.330 | 0.23148 | 2 | 0 | 3 | 1 |

| No. | Description | Accession number | STN[1] | p-Value[1] | Con_A[2] | Con_B[2] | LUTEO_A[2] | LUTEO_B[2] |
|---|---|---|---|---|---|---|---|---|
| 757 | Importin subunit alpha-3 | IPI00299033 | 0.330 | 0.23148 | 2 | 1 | 3 | 0 |
| 758 | Isoform 1 of H/ACA ribonucleoprotein complex subunit 1 | IPI00302176 | 0.330 | 0.23148 | 1 | 2 | 2 | 2 |
| 759 | Vacuolar protein sorting-associated protein VTA1 homolog | IPI00017160 | 0.330 | 0.23148 | 2 | 1 | 2 | 2 |
| 760 | Isoform 1 of Coronin-7 | IPI00027996 | 0.330 | 0.23148 | 1 | 2 | 2 | 2 |
| 761 | Isoform 1 of Apoptosis-inducing factor 2 | IPI00013909 | 0.330 | 0.23148 | 1 | 2 | 3 | 1 |
| 762 | Trafficking protein particle complex subunit 5 | IPI00177509 | 0.330 | 0.23148 | 2 | 1 | 2 | 2 |
| 763 | Isoform II of Ubiquitin-protein ligase E3A | IPI00011609 | 0.330 | 0.23148 | 1 | 2 | 1 | 3 |
| 764 | Isoform 1 of HAUS augmin-like complex subunit 2 | IPI00018198 | 0.330 | 0.23148 | 1 | 2 | 2 | 2 |
| 765 | NADH-cytochrome b5 reductase 1 | IPI00470674 | 0.330 | 0.23148 | 2 | 1 | 3 | 1 |
| 766 | Isoform 2 of Hydroxysteroid dehydrogenase-like protein 2 | IPI00031107 | 0.330 | 0.23148 | 0 | 2 | 3 | 1 |
| 767 | Proline-rich protein PRCC | IPI00294618 | 0.330 | 0.23148 | 2 | 1 | 3 | 0 |
| 768 | Uncharacterized protein C7orf30 | IPI00061492 | 0.330 | 0.23148 | 2 | 1 | 2 | 2 |
| 769 | Nuclear pore complex protein Nup88 | IPI00001738 | 0.330 | 0.23148 | 2 | 0 | 2 | 2 |
| 770 | Isoform 2 of Rho guanine nucleotide exchange factor 1 | IPI00339379 | 0.330 | 0.23148 | 0 | 2 | 2 | 2 |
| 771 | Isoform 2 of 5'-AMP-activated protein kinase catalytic subunit alpha-1 | IPI00410287 | 0.330 | 0.23148 | 1 | 2 | 2 | 2 |
| 772 | Calcium-binding protein p22 | IPI00218924 | 0.330 | 0.23148 | 2 | 1 | 3 | 1 |
| 773 | TGF-beta-activated kinase 1 and MAP3K7-binding protein 1 | IPI00019459 | 0.330 | 0.23148 | 1 | 2 | 3 | 1 |
| 774 | Fumarylacetoacetate hydrolase domain-containing protein 2A | IPI00329742 | 0.330 | 0.23148 | 0 | 2 | 2 | 2 |
| 775 | Eukaryotic translation initiation factor 3 subunit D | IPI00006181 | 0.323 | 0.24506 | 10 | 10 | 12 | 10 |
| 776 | Sodium/potassium-transporting ATPase subunit alpha-2 | IPI00003021 | 0.311 | 0.24739 | 14 | 8 | 13 | 11 |
| 777 | Isoform 1 of RNA-binding protein 8A | IPI00001757 | 0.306 | 0.24934 | 12 | 11 | 14 | 11 |
| 778 | Isoform 1 of RRP12-like protein | IPI00101186 | 0.306 | 0.24934 | 11 | 12 | 14 | 11 |
| 779 | Isoform 1 of Elongation factor Ts, mitochondrial | IPI00021016 | 0.298 | 0.25076 | 3 | 1 | 1 | 4 |
| 780 | Isoform 2 of TIP41-like protein | IPI00641815 | 0.298 | 0.25076 | 2 | 2 | 1 | 4 |
| 781 | Isoform 1 of Protein fat-free homolog | IPI00001710 | 0.298 | 0.25076 | 2 | 2 | 3 | 2 |
| 782 | cDNA FLJ61739, highly similar to Serine/arginine repetitive matrix protein 1 | IPI00328293 | 0.298 | 0.25076 | 2 | 2 | 2 | 3 |
| 783 | Isoform 1 of Tenascin | IPI00031008 | 0.298 | 0.25076 | 1 | 3 | 3 | 2 |
| 784 | Isoform 1 of RNA-binding protein 4 | IPI00003704 | 0.298 | 0.25076 | 3 | 1 | 2 | 3 |
| 785 | Protein NipSnap homolog 3A | IPI00004845 | 0.298 | 0.25076 | 0 | 3 | 4 | 1 |
| 786 | Glutamate--cysteine ligase catalytic subunit | IPI00215768 | 0.298 | 0.25076 | 3 | 1 | 4 | 1 |
| 787 | General transcription factor 3C polypeptide 4 | IPI00016725 | 0.298 | 0.25076 | 1 | 3 | 3 | 2 |
| 788 | GTPase NRas | IPI00000005 | 0.298 | 0.25076 | 3 | 1 | 3 | 2 |
| 789 | Probable ATP-dependent RNA helicase DDX28 | IPI00020050 | 0.298 | 0.25076 | 2 | 2 | 4 | 0 |
| 790 | Isoform 1 of U3 small nucleolar RNA-associated protein 14 homolog A | IPI00107113 | 0.298 | 0.25076 | 3 | 1 | 3 | 2 |
| 791 | Isoform 1 of U4/U6 small nuclear ribonucleoprotein Prp31 | IPI00292000 | 0.298 | 0.25076 | 2 | 2 | 2 | 3 |
| 792 | Mitotic spindle assembly checkpoint protein MAD2A | IPI00012369 | 0.298 | 0.25076 | 2 | 2 | 3 | 2 |
| 793 | Density-regulated protein | IPI00306280 | 0.298 | 0.25076 | 3 | 1 | 3 | 2 |
| 794 | Alpha-mannosidase 2 | IPI00003802 | 0.298 | 0.25076 | 2 | 2 | 2 | 3 |
| 795 | 22 kDa protein | IPI00219910 | 0.298 | 0.25076 | 3 | 1 | 4 | 1 |
| 796 | Transcription initiation factor IIB | IPI00022820 | 0.298 | 0.25076 | 3 | 1 | 2 | 3 |
| 797 | 3-ketoacyl-CoA thiolase, peroxisomal | IPI00012828 | 0.298 | 0.25076 | 2 | 2 | 3 | 2 |
| 798 | Isoform 1 of Transmembrane protein 85 | IPI00009320 | 0.298 | 0.25076 | 2 | 2 | 2 | 3 |
| 799 | Reticulocalbin-2 | IPI00029628 | 0.298 | 0.25076 | 1 | 3 | 3 | 2 |
| 800 | Synaptic vesicle membrane protein VAT-1 homolog | IPI00156689 | 0.298 | 0.25076 | 3 | 1 | 2 | 3 |
| 801 | GPI-anchor transamidase | IPI00022543 | 0.298 | 0.25076 | 2 | 2 | 2 | 3 |
| 802 | Isoform 4 of Protein LAS1 homolog | IPI00152781 | 0.298 | 0.25076 | 2 | 2 | 3 | 2 |
| 803 | General vesicular transport factor p115 | IPI00941161 | 0.298 | 0.25076 | 2 | 2 | 3 | 2 |
| 804 | Serine/threonine-protein kinase 10 | IPI00304742 | 0.298 | 0.25076 | 2 | 2 | 2 | 3 |
| 805 | Isoform E of Eukaryotic translation initiation factor 4 gamma 1 | IPI00386533 | 0.297 | 0.26073 | 11 | 14 | 13 | 14 |
| 806 | Protein phosphatase 1G | IPI00006167 | 0.292 | 0.26092 | 13 | 13 | 13 | 15 |
| 807 | Isoform 3 of Probable ATP-dependent RNA helicase DDX17 | IPI00651653 | 0.288 | 0.26277 | 15 | 12 | 16 | 13 |
| 808 | Prolyl endopeptidase | IPI00008164 | 0.288 | 0.26277 | 15 | 12 | 15 | 14 |
| 809 | 40S ribosomal protein S13 | IPI00221089 | 0.284 | 0.26291 | 13 | 15 | 15 | 15 |
| 810 | Fructose-bisphosphate aldolase A | IPI00465439 | 0.283 | 0.26472 | 52 | 28 | 45 | 38 |
| 811 | Histidyl-tRNA synthetase, cytoplasmic | IPI00021808 | 0.280 | 0.26486 | 16 | 13 | 15 | 16 |
| 812 | Isoform 1 of Heterogeneous nuclear ribonucleoprotein Q | IPI00018140 | 0.277 | 0.26510 | 42 | 42 | 48 | 39 |
| 813 | Nuclear migration protein nudC | IPI00550746 | 0.277 | 0.26524 | 16 | 14 | 17 | 15 |
| 814 | Isoform 1 of Zinc phosphodiesterase ELAC protein 2 | IPI00396627 | 0.275 | 0.26700 | 2 | 3 | 3 | 3 |
| 815 | 60S ribosomal protein L7-like 1 | IPI00456940 | 0.275 | 0.26700 | 3 | 2 | 4 | 2 |
| 816 | Isoform D of Constitutive coactivator of PPAR-gamma-like protein 1 | IPI00039626 | 0.275 | 0.26700 | 4 | 1 | 4 | 2 |
| 817 | Isoform 1 of Thyroid receptor-interacting protein 13 | IPI00003505 | 0.275 | 0.26700 | 3 | 2 | 2 | 4 |
| 818 | FAS-associated factor 2 | IPI00172656 | 0.275 | 0.26700 | 3 | 2 | 3 | 3 |
| 819 | 60S ribosomal protein L35 | IPI00412607 | 0.275 | 0.26700 | 2 | 3 | 3 | 3 |
| 820 | Isoform 1 of HCLS1-associated protein X-1 | IPI00010440 | 0.275 | 0.26700 | 2 | 3 | 4 | 2 |
| 821 | 28S ribosomal protein S23, mitochondrial | IPI00032881 | 0.275 | 0.26700 | 1 | 4 | 3 | 3 |
| 822 | Exportin-7 | IPI00302458 | 0.275 | 0.26700 | 2 | 3 | 3 | 3 |
| 823 | Neprilysin | IPI00247063 | 0.275 | 0.26700 | 1 | 4 | 4 | 2 |
| 824 | FKBP1A protein | IPI00413778 | 0.275 | 0.26700 | 1 | 4 | 3 | 3 |
| 825 | U6 snRNA-associated Sm-like protein LSm3 | IPI00219229 | 0.275 | 0.26700 | 2 | 3 | 3 | 3 |
| 826 | Importin-8 | IPI00007401 | 0.275 | 0.26700 | 2 | 3 | 4 | 2 |
| 827 | Nuclear pore complex protein Nup50 | IPI00026940 | 0.275 | 0.26700 | 3 | 2 | 4 | 2 |
| 828 | cDNA FLJ56037, highly similar to Cullin-2 | IPI00014311 | 0.275 | 0.26700 | 2 | 3 | 4 | 2 |
| 829 | Isoform 1 of Reticulon-4 | IPI00021766 | 0.275 | 0.26700 | 3 | 2 | 4 | 2 |
| 830 | Isoform 1 of RNA polymerase II-associated protein 1 | IPI00402657 | 0.275 | 0.26700 | 3 | 2 | 3 | 3 |
| 831 | 39S ribosomal protein L14, mitochondrial | IPI00418290 | 0.275 | 0.26700 | 3 | 2 | 4 | 2 |
| 832 | Toll-interacting protein | IPI00100154 | 0.275 | 0.26700 | 4 | 1 | 3 | 3 |
| 833 | Uncharacterized protein C7orf50 | IPI00031651 | 0.275 | 0.26700 | 3 | 2 | 4 | 2 |
| 834 | Isoform 1 of Dephospho-CoA kinase domain-containing protein | IPI00291417 | 0.275 | 0.26700 | 3 | 2 | 3 | 3 |
| 835 | Isoform 1 of Deoxycytidylate deaminase | IPI00296863 | 0.275 | 0.26700 | 3 | 2 | 3 | 3 |
| 836 | Large neutral amino acids transporter small subunit 1 | IPI00008986 | 0.275 | 0.26700 | 2 | 3 | 4 | 2 |
| 837 | Isoform 1 of SEC23-interacting protein | IPI00026969 | 0.275 | 0.26700 | 2 | 3 | 3 | 3 |
| 838 | Transmembrane protein 109 | IPI00031697 | 0.275 | 0.26700 | 3 | 2 | 3 | 3 |
| 839 | ADP-ribosylation factor 5 | IPI00215919 | 0.275 | 0.26700 | 4 | 1 | 3 | 3 |
| 840 | REST corepressor 1 | IPI00008531 | 0.275 | 0.26700 | 2 | 3 | 5 | 0 |
| 841 | 40S ribosomal protein S11 | IPI00025091 | 0.257 | 0.27782 | 3 | 3 | 3 | 4 |
| 842 | Isoform 1 of 2-oxoglutarate and iron-dependent oxygenase domain-containing protein 1 | IPI00170429 | 0.257 | 0.27782 | 3 | 3 | 4 | 3 |
| 843 | Splicing factor U2AF 35 kDa subunit | IPI00005613 | 0.257 | 0.27782 | 4 | 2 | 4 | 3 |
| 844 | 145 kDa protein | IPI00218097 | 0.257 | 0.27782 | 3 | 3 | 3 | 4 |
| 845 | Isoform 1 of Methylthioribose-1-phosphate isomerase | IPI00005948 | 0.257 | 0.27782 | 3 | 3 | 4 | 3 |
| 846 | Splicing factor, arginine/serine-rich 3 | IPI00010204 | 0.257 | 0.27782 | 4 | 2 | 3 | 4 |
| 847 | Glyoxylate reductase/hydroxypyruvate reductase | IPI00037448 | 0.257 | 0.27782 | 3 | 3 | 3 | 4 |
| 848 | Isoform 1 of GPI transamidase component PIG-S | IPI00465308 | 0.257 | 0.27782 | 3 | 3 | 3 | 4 |
| 849 | Aflatoxin B1 aldehyde reductase member 2 | IPI00305978 | 0.257 | 0.27782 | 3 | 3 | 3 | 4 |
| 850 | Negative elongation factor B | IPI00103483 | 0.257 | 0.27782 | 3 | 3 | 4 | 3 |
| 851 | Receptor accessory protein 6, isoform CRA_a | IPI00646963 | 0.257 | 0.27782 | 3 | 3 | 4 | 3 |

| No. | Description | Accession number | STN[1] | p-Value[1] | Con_A[2] | Con_B[2] | LUTEO_A[2] | LUTEO_B[2] |
|---|---|---|---|---|---|---|---|---|
| 852 | Isoform 3 of CCR4-NOT transcription complex subunit 10 | IPI00396314 | 0.257 | 0.27782 | 4 | 2 | 3 | 4 |
| 853 | Isoform 2 of Phosphoenolpyruvate carboxykinase [GTP], mitochondrial | IPI00384116 | 0.257 | 0.27782 | 3 | 3 | 3 | 4 |
| 854 | Peptidyl-prolyl cis-trans isomerase FKBP2 | IPI00002535 | 0.257 | 0.27782 | 2 | 4 | 4 | 3 |
| 855 | High mobility group protein B3 | IPI00217477 | 0.257 | 0.27782 | 3 | 3 | 4 | 3 |
| 856 | similar to RAN binding protein 1 | IPI00399212 | 0.257 | 0.27782 | 3 | 3 | 4 | 3 |
| 857 | CCR4-NOT transcription complex subunit 7 | IPI00006552 | 0.257 | 0.27782 | 3 | 3 | 4 | 3 |
| 858 | Phospholipase A-2-activating protein | IPI00218465 | 0.257 | 0.27782 | 4 | 2 | 3 | 4 |
| 859 | Isoform 2 of Low molecular weight phosphotyrosine protein phosphatase | IPI00218847 | 0.257 | 0.27782 | 3 | 3 | 4 | 3 |
| 860 | BRO1 domain-containing protein BROX | IPI00065500 | 0.257 | 0.27782 | 3 | 3 | 4 | 3 |
| 861 | Nucleoporin Nup37 | IPI00171665 | 0.257 | 0.27782 | 3 | 3 | 4 | 3 |
| 862 | CAD protein | IPI00301263 | 0.257 | 0.28423 | 49 | 53 | 52 | 53 |
| 863 | Voltage-dependent anion-selective channel protein 1 | IPI00216308 | 0.255 | 0.28428 | 53 | 51 | 52 | 55 |
| 864 | Isoform 1 of Nuclear pore complex protein Nup160 | IPI00748807 | 0.250 | 0.28470 | 19 | 20 | 20 | 21 |
| 865 | Rho GTPase-activating protein 1 | IPI00020567 | 0.243 | 0.28570 | 3 | 4 | 4 | 4 |
| 866 | Isovaleryl-CoA dehydrogenase, mitochondrial | IPI00645805 | 0.243 | 0.28570 | 3 | 4 | 5 | 3 |
| 867 | Isoform 2 of Phosphoglucomutase-1 | IPI00217872 | 0.243 | 0.28570 | 4 | 3 | 4 | 4 |
| 868 | Myosin-11 | IPI00020501 | 0.243 | 0.28570 | 4 | 3 | 4 | 4 |
| 869 | Catechol O-methyltransferase domain-containing protein 1 | IPI00642041 | 0.243 | 0.28570 | 5 | 2 | 5 | 3 |
| 870 | ADP/ATP translocase 1 | IPI00022891 | 0.243 | 0.28570 | 4 | 3 | 4 | 4 |
| 871 | Myosin regulatory light chain 12B | IPI00033494 | 0.243 | 0.28570 | 4 | 3 | 5 | 3 |
| 872 | DEAD (Asp-Glu-Ala-Asp) box polypeptide 39, isoform CRA_c | IPI00166874 | 0.243 | 0.28570 | 3 | 4 | 4 | 4 |
| 873 | Ribosome biogenesis protein WDR12 | IPI00304232 | 0.243 | 0.28570 | 5 | 2 | 2 | 6 |
| 874 | Hsp90 co-chaperone Cdc37 | IPI00013122 | 0.243 | 0.28570 | 4 | 3 | 4 | 4 |
| 875 | DNA damage-binding protein 1 | IPI00293464 | 0.243 | 0.29040 | 22 | 20 | 24 | 20 |
| 876 | Glutamate dehydrogenase 1, mitochondrial | IPI00016801 | 0.239 | 0.29083 | 24 | 20 | 23 | 23 |
| 877 | 60S ribosomal protein L18a | IPI00026202 | 0.231 | 0.29268 | 4 | 4 | 5 | 4 |
| 878 | 28S ribosomal protein S29, mitochondrial | IPI00018120 | 0.231 | 0.29268 | 5 | 3 | 5 | 4 |
| 879 | Isocitrate dehydrogenase 3, beta subunit isoform a precursor | IPI00304417 | 0.231 | 0.29268 | 2 | 6 | 5 | 4 |
| 880 | 40S ribosomal protein S20 | IPI00012493 | 0.231 | 0.29268 | 3 | 5 | 4 | 5 |
| 881 | Isoform 3 of Chitinase domain-containing protein 1 | IPI00045536 | 0.231 | 0.29268 | 3 | 5 | 6 | 3 |
| 882 | COP9 signalosome complex subunit 5 | IPI00009958 | 0.231 | 0.29268 | 5 | 3 | 6 | 3 |
| 883 | Metaxin-2 | IPI00025717 | 0.231 | 0.29268 | 4 | 4 | 7 | 2 |
| 884 | Myosin-Ie | IPI00329672 | 0.231 | 0.29268 | 3 | 5 | 5 | 4 |
| 885 | Isoform 1 of Phosphoenolpyruvate carboxykinase [GTP], mitochondrial | IPI00797038 | 0.231 | 0.29268 | 4 | 4 | 5 | 4 |
| 886 | Isoform 1 of Phosphatidylinositol transfer protein beta isoform | IPI00334907 | 0.221 | 0.29776 | 4 | 5 | 5 | 5 |
| 887 | 60S ribosomal protein L27 | IPI00219155 | 0.221 | 0.29776 | 6 | 3 | 6 | 4 |
| 888 | Ribosome maturation protein SBDS | IPI00427330 | 0.221 | 0.29776 | 5 | 4 | 4 | 6 |
| 889 | Isoform Long of 60 kDa SS-A/Ro ribonucleoprotein | IPI00019450 | 0.221 | 0.29776 | 4 | 5 | 5 | 5 |
| 890 | Isoform 1 of Spermine synthase | IPI00005102 | 0.221 | 0.29776 | 4 | 5 | 4 | 6 |
| 891 | Guanine nucleotide-binding protein G(I)/G(S)/G(T) subunit beta-2 | IPI00003348 | 0.221 | 0.29776 | 4 | 5 | 5 | 5 |
| 892 | Coiled-coil-helix-coiled-coil-helix domain-containing protein 3, mitochondrial | IPI00015833 | 0.221 | 0.29776 | 5 | 4 | 5 | 5 |
| 893 | Mitochondrial import inner membrane translocase subunit Tim16 | IPI00218463 | 0.221 | 0.29776 | 4 | 5 | 6 | 4 |
| 894 | Succinate dehydrogenase assembly factor 2, mitochondrial | IPI00016443 | 0.221 | 0.29776 | 5 | 4 | 5 | 5 |
| 895 | Eukaryotic translation initiation factor 3, subunit E interacting protein | IPI00465233 | 0.220 | 0.30094 | 26 | 28 | 29 | 27 |
| 896 | Isoform 1 of U5 small nuclear ribonucleoprotein 200 kDa helicase | IPI00420014 | 0.220 | 0.30118 | 77 | 75 | 76 | 79 |
| 897 | Isoform Long of Cold shock domain-containing protein E1 | IPI00470891 | 0.213 | 0.30160 | 5 | 5 | 7 | 4 |
| 898 | Isoform 1 of Coiled-coil domain-containing protein 47 | IPI00024642 | 0.213 | 0.30160 | 6 | 4 | 8 | 3 |
| 899 | 60S ribosomal protein L13 | IPI00465361 | 0.213 | 0.30160 | 4 | 6 | 5 | 6 |
| 900 | Isoform 1 of Ubiquitin-conjugating enzyme E2 K | IPI00021370 | 0.213 | 0.30160 | 5 | 5 | 5 | 6 |
| 901 | Programmed cell death protein 5 | IPI00023640 | 0.213 | 0.30160 | 6 | 4 | 6 | 5 |
| 902 | Isoform 1 of Oligoribonuclease, mitochondrial (Fragment) | IPI00032830 | 0.213 | 0.30160 | 5 | 5 | 6 | 5 |
| 903 | Glycyl-tRNA synthetase | IPI00783097 | 0.209 | 0.30668 | 30 | 32 | 34 | 30 |
| 904 | Proteasome subunit beta type-7 | IPI00003217 | 0.205 | 0.30711 | 6 | 5 | 8 | 4 |
| 905 | Transmembrane emp24 domain-containing protein 2 | IPI00016608 | 0.205 | 0.30711 | 6 | 5 | 6 | 6 |
| 906 | pyrroline-5-carboxylate reductase 1, mitochondrial isoform 2 | IPI00376503 | 0.205 | 0.30711 | 6 | 5 | 6 | 6 |
| 907 | COP9 signalosome complex subunit 3 | IPI00025721 | 0.205 | 0.30711 | 3 | 8 | 7 | 5 |
| 908 | Isoform Long of Long-chain-fatty-acid--CoA ligase 4 | IPI00029737 | 0.205 | 0.30711 | 6 | 5 | 6 | 6 |
| 909 | L-aminoadipate-semialdehyde dehydrogenase-phosphopantetheinyl transferase | IPI00250297 | 0.205 | 0.30711 | 5 | 6 | 6 | 6 |
| 910 | RNA-binding motif protein, X-linked-like-2 | IPI00004450 | 0.205 | 0.30711 | 6 | 5 | 6 | 6 |
| 911 | Isoform 1 of Porphobilinogen deaminase | IPI00028160 | 0.205 | 0.30711 | 5 | 6 | 7 | 5 |
| 912 | Uncharacterized protein C2orf47, mitochondrial | IPI00291751 | 0.205 | 0.30711 | 5 | 6 | 6 | 6 |
| 913 | DnaJ homolog subfamily A member 1 | IPI00012535 | 0.198 | 0.31006 | 5 | 7 | 6 | 7 |
| 914 | Isoform 3 of Drebrin-like protein | IPI00101968 | 0.198 | 0.31006 | 5 | 7 | 9 | 4 |
| 915 | Isoform 1 of Coatomer subunit alpha | IPI00295857 | 0.198 | 0.31233 | 39 | 32 | 36 | 37 |
| 916 | Coatomer subunit gamma-2 | IPI00002557 | 0.192 | 0.31305 | 6 | 7 | 7 | 7 |
| 917 | Glucosamine 6-phosphate N-acetyltransferase | IPI00061525 | 0.187 | 0.31466 | 9 | 5 | 8 | 7 |
| 918 | Eukaryotic peptide chain release factor subunit 1 | IPI00429191 | 0.187 | 0.31466 | 8 | 6 | 7 | 8 |
| 919 | COP9 signalosome complex subunit 7a | IPI00301419 | 0.187 | 0.31466 | 9 | 5 | 9 | 6 |
| 920 | Ataxin-10 | IPI00001636 | 0.187 | 0.31466 | 7 | 7 | 7 | 8 |
| 921 | 7-dehydrocholesterol reductase | IPI00294501 | 0.187 | 0.31466 | 6 | 8 | 8 | 7 |
| 922 | Isoform 1 of Septin-2 | IPI00014177 | 0.182 | 0.31656 | 8 | 7 | 9 | 7 |
| 923 | Isoform p150 of Dynactin subunit 1 | IPI00029485 | 0.182 | 0.31656 | 9 | 6 | 8 | 8 |
| 924 | Ribonuclease P protein subunit p30 | IPI00019196 | 0.182 | 0.31656 | 7 | 8 | 9 | 7 |
| 925 | Isoform 1 of Transcription intermediary factor 1-beta | IPI00438229 | 0.179 | 0.31784 | 48 | 44 | 52 | 42 |
| 926 | Nucleolar pre-ribosomal-associated protein 1 | IPI00297241 | 0.178 | 0.31808 | 7 | 9 | 10 | 7 |
| 927 | Isoform 1 of 5'(3')-deoxyribonucleotidase, cytosolic type | IPI00005573 | 0.178 | 0.31808 | 8 | 8 | 9 | 8 |
| 928 | Malate dehydrogenase, mitochondrial | IPI00291006 | 0.176 | 0.32097 | 50 | 45 | 54 | 43 |
| 929 | Alkyldihydroxyacetonephosphate synthase, peroxisomal | IPI00010349 | 0.174 | 0.32107 | 8 | 9 | 9 | 9 |
| 930 | Isoform 1 of 26S protease regulatory subunit 6B | IPI00020042 | 0.174 | 0.32107 | 7 | 10 | 10 | 8 |
| 931 | Ran GTPase-activating protein 1 | IPI00294879 | 0.174 | 0.32107 | 9 | 8 | 10 | 8 |
| 932 | Isoform 1 of WD repeat-containing protein 1 | IPI00746165 | 0.170 | 0.32268 | 10 | 8 | 11 | 8 |
| 933 | Putative uncharacterized protein RPL17 | IPI00394699 | 0.166 | 0.32397 | 9 | 10 | 12 | 8 |
| 934 | 40S ribosomal protein S14 | IPI00026271 | 0.166 | 0.32397 | 9 | 10 | 11 | 9 |
| 935 | 40S ribosomal protein S25 | IPI00012750 | 0.166 | 0.32397 | 9 | 10 | 10 | 10 |
| 936 | Isoform 3 of Adenylate kinase 2, mitochondrial | IPI00172460 | 0.163 | 0.32482 | 9 | 11 | 11 | 10 |
| 937 | Putative uncharacterized protein MDH1 | IPI00915869 | 0.163 | 0.32482 | 10 | 10 | 11 | 10 |
| 938 | mRNA turnover protein 4 homolog | IPI00106491 | 0.163 | 0.32482 | 12 | 8 | 12 | 9 |
| 939 | Isoform 1 of Calcyclin-binding protein | IPI00395627 | 0.160 | 0.32563 | 12 | 9 | 12 | 10 |
| 940 | Intron-binding protein aquarius | IPI00297572 | 0.160 | 0.32563 | 12 | 9 | 14 | 8 |
| 941 | Proteasome subunit alpha type-4 | IPI00299155 | 0.157 | 0.32605 | 11 | 11 | 12 | 11 |
| 942 | UPF0568 protein C14orf166 | IPI00006980 | 0.154 | 0.32662 | 12 | 11 | 15 | 9 |
| 943 | Isoform 1 of Glucosamine--fructose-6-phosphate aminotransferase [isomerizing] 1 | IPI00217952 | 0.154 | 0.32662 | 13 | 10 | 13 | 11 |
| 944 | Isoform SM-B' of Small nuclear ribonucleoprotein-associated proteins B and B' | IPI00027285 | 0.154 | 0.32662 | 14 | 9 | 14 | 10 |
| 945 | Ras-related protein Rap-1b | IPI00015148 | 0.152 | 0.32729 | 12 | 12 | 12 | 13 |
| 946 | Importin 5 | IPI00514205 | 0.152 | 0.32729 | 13 | 11 | 14 | 11 |

| No. | Description | Accession number | STN[1] | p-Value[1] | Con_A[2] | Con_B[2] | LUTEO_A[2] | LUTEO_B[2] |
|---|---|---|---|---|---|---|---|---|
| 947 | cDNA FLJ75085, highly similar to Homo sapiens glutaminyl-tRNA synthetase (QARS), mRNA | IPI00026665 | 0.149 | 0.32786 | 11 | 14 | 13 | 13 |
| 948 | Cullin-1 | IPI00014310 | 0.149 | 0.32786 | 11 | 14 | 12 | 14 |
| 949 | Proteasome subunit beta type-2 | IPI00028006 | 0.147 | 0.32833 | 14 | 12 | 13 | 14 |
| 950 | cDNA FLJ59758, highly similar to S-methyl-5-thioadenosine phosphorylase | IPI00011876 | 0.145 | 0.32914 | 14 | 13 | 15 | 13 |
| 951 | 40S ribosomal protein S18 | IPI00013296 | 0.141 | 0.33047 | 17 | 12 | 17 | 13 |
| 952 | Dihydropyrimidinase-related protein 2 | IPI00257508 | 0.141 | 0.33047 | 17 | 12 | 16 | 14 |
| 953 | Proteasome subunit alpha type-6 | IPI00029623 | 0.139 | 0.33080 | 15 | 15 | 14 | 17 |
| 954 | Isoform 1 of Electron transfer flavoprotein subunit beta | IPI00004902 | 0.137 | 0.33151 | 15 | 16 | 17 | 15 |
| 955 | Dolichyl-diphosphooligosaccharide--protein glycosyltransferase 48 kDa subunit | IPI00297084 | 0.137 | 0.33151 | 15 | 16 | 20 | 12 |
| 956 | Isoform 1 of Isocitrate dehydrogenase [NAD] subunit alpha, mitochondrial | IPI00030702 | 0.136 | 0.33213 | 16 | 16 | 16 | 17 |
| 957 | Bifunctional ATP-dependent dihydroxyacetone kinase/FAD-AMP lyase (cyclizing) | IPI00551024 | 0.134 | 0.33275 | 13 | 20 | 21 | 13 |
| 958 | Proteasome subunit beta type-1 | IPI00025019 | 0.124 | 0.33474 | 22 | 18 | 21 | 20 |
| 959 | ATP-binding cassette sub-family E member 1 | IPI00303207 | 0.122 | 0.33555 | 21 | 21 | 25 | 18 |
| 960 | Lupus La protein | IPI00009032 | 0.121 | 0.33560 | 20 | 23 | 21 | 23 |
| 961 | Heat shock 70 kDa protein 1A/1B | IPI00304925 | 0.120 | 0.33560 | 21 | 23 | 23 | 22 |
| 962 | 60S ribosomal protein L9 | IPI00031691 | 0.117 | 0.33617 | 24 | 23 | 27 | 21 |
| 963 | Structural maintenance of chromosomes protein 1A | IPI00291939 | 0.117 | 0.33617 | 24 | 23 | 23 | 25 |
| 964 | Annexin A3 | IPI00024095 | 0.115 | 0.33693 | 26 | 23 | 25 | 25 |
| 965 | 6-phosphogluconate dehydrogenase, decarboxylating | IPI00219525 | 0.108 | 0.33759 | 30 | 27 | 30 | 28 |
| 966 | Threonyl-tRNA synthetase, cytoplasmic | IPI00329633 | 0.097 | 0.33882 | 39 | 36 | 40 | 36 |
| 967 | Isoform 1 of Myosin-10 | IPI00397526 | 0.082 | 0.34020 | 55 | 61 | 58 | 59 |
| 968 | Putative heat shock protein HSP 90-alpha A2 | IPI00031523 | 0.061 | 0.34063 | 123 | 121 | 133 | 112 |
| 969 | Elongation factor 1-alpha | IPI00025447 | 0.000 | 0.34115 | 45 | 51 | 48 | 48 |
| 970 | Rho GDP-dissociation inhibitor 1 | IPI00003815 | 0.000 | 0.34115 | 21 | 23 | 22 | 22 |
| 971 | Nucleolar protein 56 | IPI00411937 | 0.000 | 0.34115 | 30 | 30 | 29 | 31 |
| 972 | DNA replication licensing factor MCM4 | IPI00018349 | 0.000 | 0.34115 | 27 | 20 | 28 | 19 |
| 973 | Destrin | IPI00473014 | 0.000 | 0.34115 | 22 | 16 | 18 | 20 |
| 974 | 40S ribosomal protein S15 | IPI00479058 | 0.000 | 0.34115 | 17 | 22 | 19 | 20 |
| 975 | Isoform 1 of Proteasome subunit alpha type-7 | IPI00024175 | 0.000 | 0.34115 | 18 | 19 | 18 | 19 |
| 976 | Peroxiredoxin-2 | IPI00027350 | 0.000 | 0.34115 | 15 | 17 | 17 | 15 |
| 977 | Isoform 1 of Mitochondrial import receptor subunit TOM40 homolog | IPI00014053 | 0.000 | 0.34115 | 6 | 7 | 7 | 6 |
| 978 | Isoform 1 of Proteasome activator complex subunit 3 | IPI00030243 | 0.000 | 0.34115 | 18 | 15 | 17 | 16 |
| 979 | Isoform Long of Inositol 1,4,5-trisphosphate receptor type 2 | IPI00031545 | 0.000 | 0.34115 | 11 | 13 | 10 | 14 |
| 980 | 26S proteasome non-ATPase regulatory subunit 7 | IPI00019927 | 0.000 | 0.34115 | 13 | 12 | 13 | 12 |
| 981 | Translin-associated protein X | IPI00293350 | 0.000 | 0.34115 | 18 | 15 | 17 | 16 |
| 982 | Eukaryotic translation initiation factor 2 subunit 1 | IPI00219678 | 0.000 | 0.34115 | 15 | 17 | 13 | 19 |
| 983 | Seryl-tRNA synthetase, cytoplasmic | IPI00220637 | 0.000 | 0.34115 | 13 | 12 | 14 | 11 |
| 984 | AP-1 complex subunit gamma-1 isoform a | IPI00293396 | 0.000 | 0.34115 | 10 | 9 | 11 | 8 |
| 985 | Delta(3,5)-Delta(2,4)-dienoyl-CoA isomerase, mitochondrial | IPI00011416 | 0.000 | 0.34115 | 15 | 13 | 16 | 12 |
| 986 | Isoform 2 of Nuclear protein localization protein 4 homolog | IPI00001676 | 0.000 | 0.34115 | 15 | 16 | 19 | 12 |
| 987 | Ras-related protein Rab-5C | IPI00016339 | 0.000 | 0.34115 | 14 | 14 | 16 | 12 |
| 988 | rRNA 2'-O-methyltransferase fibrillarin | IPI00025039 | 0.000 | 0.34115 | 9 | 4 | 5 | 8 |
| 989 | DNA-directed RNA polymerase I subunit RPA1 | IPI00031960 | 0.000 | 0.34115 | 7 | 9 | 10 | 6 |
| 990 | Isoform 2 of Triosephosphate isomerase | IPI00451401 | 0.000 | 0.34115 | 15 | 15 | 16 | 14 |
| 991 | Isoform 1 of Voltage-dependent anion-selective channel protein 3 | IPI00031804 | 0.000 | 0.34115 | 12 | 12 | 13 | 11 |
| 992 | Phenylalanyl-tRNA synthetase alpha chain | IPI00031820 | 0.000 | 0.34115 | 10 | 6 | 9 | 7 |
| 993 | Isoform 1 of E3 UFM1-protein ligase 1 | IPI00844000 | 0.000 | 0.34115 | 7 | 9 | 8 | 8 |
| 994 | Coatomer subunit delta variant 2 | IPI00298520 | 0.000 | 0.34115 | 9 | 7 | 7 | 9 |
| 995 | Nucleolar complex protein 4 homolog | IPI00031661 | 0.000 | 0.34115 | 5 | 4 | 3 | 6 |
| 996 | DNA-(apurinic or apyrimidinic site) lyase | IPI00215911 | 0.000 | 0.34115 | 11 | 10 | 11 | 10 |
| 997 | Long-chain-fatty-acid--CoA ligase 3 | IPI00031397 | 0.000 | 0.34115 | 7 | 4 | 5 | 6 |
| 998 | Copine-1 | IPI00018452 | 0.000 | 0.34115 | 9 | 9 | 9 | 9 |
| 999 | Casein kinase II subunit alpha' | IPI00020602 | 0.000 | 0.34115 | 10 | 8 | 9 | 9 |
| 1000 | 40S ribosomal protein S6 | IPI00021840 | 0.000 | 0.34115 | 10 | 11 | 10 | 11 |
| 1001 | Uroporphyrinogen decarboxylase | IPI00301489 | 0.000 | 0.34115 | 2 | 2 | 3 | 1 |
| 1002 | regulator of differentiation 1 isoform 2 | IPI00159072 | 0.000 | 0.34115 | 4 | 2 | 2 | 4 |
| 1003 | Protein transport protein Sec23A | IPI00017375 | 0.000 | 0.34115 | 9 | 9 | 9 | 9 |
| 1004 | Junction plakoglobin | IPI00554711 | 0.000 | 0.34115 | 4 | 6 | 7 | 3 |
| 1005 | Replication factor C subunit 5 | IPI00031514 | 0.000 | 0.34115 | 11 | 8 | 9 | 10 |
| 1006 | Ubiquitin-conjugating enzyme E2 O | IPI00783378 | 0.000 | 0.34115 | 3 | 5 | 4 | 4 |
| 1007 | cDNA FLJ56285, highly similar to ADP-ribosylation factor-like protein 8B | IPI00018871 | 0.000 | 0.34115 | 3 | 5 | 3 | 5 |
| 1008 | Isoform 1 of Elongation factor G, mitochondrial | IPI00154473 | 0.000 | 0.34115 | 9 | 7 | 7 | 9 |
| 1009 | H/ACA ribonucleoprotein complex subunit 4 | IPI00221394 | 0.000 | 0.34115 | 5 | 5 | 6 | 4 |
| 1010 | tropomyosin alpha-3 chain isoform 1 | IPI00183968 | 0.000 | 0.34115 | 10 | 6 | 9 | 7 |
| 1011 | Ribosome biogenesis protein BMS1 homolog | IPI00006099 | 0.000 | 0.34115 | 6 | 4 | 5 | 5 |
| 1012 | Putative uncharacterized protein CNOT1 | IPI00032299 | 0.000 | 0.34115 | 2 | 4 | 4 | 2 |
| 1013 | Coactosin-like protein | IPI00017704 | 0.000 | 0.34115 | 10 | 7 | 9 | 8 |
| 1014 | Isoform 1 of Luc7-like protein 3 | IPI00107745 | 0.000 | 0.34115 | 8 | 9 | 7 | 10 |
| 1015 | UV excision repair protein RAD23 homolog B | IPI00008223 | 0.000 | 0.34115 | 6 | 7 | 7 | 6 |
| 1016 | Hepatoma-derived growth factor | IPI00020956 | 0.000 | 0.34115 | 8 | 8 | 8 | 8 |
| 1017 | sorting nexin-1 isoform c | IPI00183274 | 0.000 | 0.34115 | 9 | 7 | 8 | 8 |
| 1018 | 26S proteasome non-ATPase regulatory subunit 14 | IPI00024821 | 0.000 | 0.34115 | 6 | 4 | 5 | 5 |
| 1019 | V-type proton ATPase subunit E 1 | IPI00003856 | 0.000 | 0.34115 | 7 | 9 | 8 | 8 |
| 1020 | Platelet-activating factor acetylhydrolase IB subunit beta | IPI00026546 | 0.000 | 0.34115 | 6 | 8 | 7 | 7 |
| 1021 | Parafibromin | IPI00300659 | 0.000 | 0.34115 | 4 | 5 | 5 | 4 |
| 1022 | cDNA FLJ56152, highly similar to Rho guanine nucleotide exchange factor 7 | IPI00449906 | 0.000 | 0.34115 | 3 | 6 | 5 | 4 |
| 1023 | 60S ribosomal protein L27a | IPI00456758 | 0.000 | 0.34115 | 6 | 5 | 6 | 5 |
| 1024 | Isoform 1 of Enolase-phosphatase E1 | IPI00038378 | 0.000 | 0.34115 | 3 | 3 | 3 | 3 |
| 1025 | Isoform Non-muscle of Myosin light polypeptide 6 | IPI00335168 | 0.000 | 0.34115 | 4 | 4 | 4 | 4 |
| 1026 | DnaJ homolog subfamily A member 2 | IPI00032406 | 0.000 | 0.34115 | 6 | 5 | 6 | 5 |
| 1027 | Putative nascent polypeptide-associated complex subunit alpha-like protein | IPI00012479 | 0.000 | 0.34115 | 7 | 6 | 6 | 7 |
| 1028 | Isoform 2 of ATP-binding cassette sub-family F member 1 | IPI00013495 | 0.000 | 0.34115 | 5 | 6 | 4 | 7 |
| 1029 | Isoform GTBP-N of DNA mismatch repair protein Msh6 | IPI00384456 | 0.000 | 0.34115 | 4 | 6 | 4 | 6 |
| 1030 | NADH dehydrogenase [ubiquinone] iron-sulfur protein 8, mitochondrial | IPI00010845 | 0.000 | 0.34115 | 4 | 5 | 6 | 3 |
| 1031 | Isoform 1 of Gamma-glutamylcyclotransferase | IPI00031564 | 0.000 | 0.34115 | 8 | 6 | 8 | 6 |
| 1032 | DNA-directed RNA polymerases I, II, and III subunit RPABC1 | IPI00291093 | 0.000 | 0.34115 | 5 | 4 | 4 | 5 |
| 1033 | Synaptobrevin homolog YKT6 | IPI00008569 | 0.000 | 0.34115 | 5 | 1 | 4 | 2 |
| 1034 | Trifunctional enzyme subunit beta, mitochondrial | IPI00022793 | 0.000 | 0.34115 | 6 | 4 | 4 | 6 |
| 1035 | transcriptional regulator ATRX isoform 2 | IPI00220109 | 0.000 | 0.34115 | 2 | 1 | 2 | 1 |
| 1036 | CD2-associated protein | IPI00412771 | 0.000 | 0.34115 | 1 | 3 | 3 | 1 |
| 1037 | Isoform Crk-II of Adapter molecule crk | IPI00004838 | 0.000 | 0.34115 | 6 | 5 | 8 | 3 |
| 1038 | Rho-related GTP-binding protein RhoG | IPI00017342 | 0.000 | 0.34115 | 6 | 4 | 6 | 4 |
| 1039 | Uncharacterized protein C18orf19 | IPI00290799 | 0.000 | 0.34115 | 6 | 6 | 6 | 6 |
| 1040 | Peptidyl-prolyl cis-trans isomerase H | IPI00007346 | 0.000 | 0.34115 | 7 | 6 | 9 | 4 |
| 1041 | Desmoglein-2 | IPI00028931 | 0.000 | 0.34115 | 6 | 4 | 3 | 7 |

| No. | Description | Accession number | STN[1] | p-Value[1] | Con_A[2] | Con_B[2] | LUTEO_A[2] | LUTEO_B[2] |
|---|---|---|---|---|---|---|---|---|
| 1042 | E3 ubiquitin/ISG15 ligase TRIM25 | IPI00029629 | 0.000 | 0.34115 | 4 | 5 | 5 | 4 |
| 1043 | Pumilio domain-containing protein C14orf21 | IPI00216999 | 0.000 | 0.34115 | 5 | 3 | 4 | 4 |
| 1044 | Isoform 1 of Sorting nexin-12 | IPI00438170 | 0.000 | 0.34115 | 3 | 4 | 5 | 2 |
| 1045 | Transmembrane protein 14C | IPI00009346 | 0.000 | 0.34115 | 2 | 5 | 4 | 3 |
| 1046 | Splicing factor, arginine/serine-rich 2 | IPI00005978 | 0.000 | 0.34115 | 4 | 5 | 3 | 6 |
| 1047 | Xaa-Pro dipeptidase | IPI00257882 | 0.000 | 0.34115 | 3 | 5 | 4 | 4 |
| 1048 | Charged multivesicular body protein 7 | IPI00395463 | 0.000 | 0.34115 | 3 | 4 | 2 | 5 |
| 1049 | Isoform 1 of Serine/threonine-protein phosphatase 2A 65 kDa regulatory subunit A beta isoform | IPI00294178 | 0.000 | 0.34115 | 3 | 3 | 3 | 3 |
| 1050 | Prostaglandin E synthase 3 | IPI00015029 | 0.000 | 0.34115 | 5 | 4 | 4 | 5 |
| 1051 | 60S ribosomal protein L5 | IPI00000494 | 0.000 | 0.34115 | 2 | 5 | 4 | 3 |
| 1052 | Vesicular integral-membrane protein VIP36 | IPI00009950 | 0.000 | 0.34115 | 4 | 5 | 5 | 4 |
| 1053 | GTP-binding protein SAR1a | IPI00015954 | 0.000 | 0.34115 | 7 | 5 | 5 | 7 |
| 1054 | Exportin-T | IPI00306290 | 0.000 | 0.34115 | 4 | 3 | 4 | 3 |
| 1055 | Serine/threonine-protein kinase OSR1 | IPI00010080 | 0.000 | 0.34115 | 4 | 1 | 4 | 1 |
| 1056 | RNA-binding protein PNO1 | IPI00024524 | 0.000 | 0.34115 | 2 | 3 | 2 | 3 |
| 1057 | Isoform 2 of Tropomyosin alpha-3 chain | IPI00218319 | 0.000 | 0.34115 | 4 | 5 | 5 | 4 |
| 1058 | Isoform 1 of Serine/threonine-protein kinase ATR | IPI00412298 | 0.000 | 0.34115 | 1 | 2 | 2 | 1 |
| 1059 | Basic leucine zipper and W2 domain-containing protein 2 | IPI00022305 | 0.000 | 0.34115 | 2 | 2 | 3 | 1 |
| 1060 | Probable methyltransferase TARBP1 | IPI00298447 | 0.000 | 0.34115 | 3 | 2 | 2 | 3 |
| 1061 | SUMO-conjugating enzyme UBC9 | IPI00032957 | 0.000 | 0.34115 | 4 | 4 | 5 | 3 |
| 1062 | Exportin-6 | IPI00465296 | 0.000 | 0.34115 | 2 | 0 | 2 | 0 |
| 1063 | Isoform 1 of Splicing factor, arginine/serine-rich 15 | IPI00181702 | 0.000 | 0.34115 | 5 | 5 | 5 | 5 |
| 1064 | cDNA FLJ40287 fis, clone TESTI2027909, highly similar to 5'-AMP-ACTIVATED PROTEIN KINASE | IPI00473047 | 0.000 | 0.34115 | 4 | 3 | 3 | 4 |
| 1065 | Rho-related GTP-binding protein RhoB | IPI00000041 | 0.000 | 0.34115 | 6 | 5 | 8 | 3 |
| 1066 | RcDNAJ9 (Fragment) | IPI00014718 | 0.000 | 0.34115 | 5 | 5 | 6 | 4 |
| 1067 | Isoform 2 of Phosphatidylinositol-binding clathrin assembly protein | IPI00216184 | 0.000 | 0.34115 | 4 | 4 | 5 | 3 |
| 1068 | Eukaryotic translation initiation factor 3 subunit J | IPI00290461 | 0.000 | 0.34115 | 6 | 4 | 6 | 4 |
| 1069 | Transmembrane protein 43 | IPI00301280 | 0.000 | 0.34115 | 2 | 0 | 0 | 2 |
| 1070 | Isoform 1 of Large proline-rich protein BAT2 | IPI00010700 | 0.000 | 0.34115 | 1 | 0 | 0 | 0 |
| 1071 | Transcription elongation factor B polypeptide 2 | IPI00026670 | 0.000 | 0.34115 | 5 | 3 | 3 | 5 |
| 1072 | Isoform 1 of Scavenger receptor class B member 1 | IPI00177968 | 0.000 | 0.34115 | 1 | 3 | 0 | 3 |
| 1073 | cDNA FLJ54536, highly similar to Mitochondrial 28S ribosomal protein S27 | IPI00022002 | 0.000 | 0.34115 | 5 | 5 | 6 | 4 |
| 1074 | Ribose-5-phosphate isomerase | IPI00026513 | 0.000 | 0.34115 | 3 | 4 | 4 | 3 |
| 1075 | Pseudouridylate synthase 7 homolog | IPI00044761 | 0.000 | 0.34115 | 1 | 2 | 2 | 1 |
| 1076 | Bifunctional 3'-phosphoadenosine 5'-phosphosulfate synthase 1 | IPI00011619 | 0.000 | 0.34115 | 2 | 3 | 1 | 4 |
| 1077 | Serine/threonine-protein kinase PRP4 homolog | IPI00013721 | 0.000 | 0.34115 | 2 | 3 | 2 | 3 |
| 1078 | GTP-binding protein Rheb | IPI00016669 | 0.000 | 0.34115 | 3 | 3 | 3 | 3 |
| 1079 | Isoform 2 of N-alpha-acetyltransferase 15, NatA auxiliary subunit | IPI00032158 | 0.000 | 0.34115 | 3 | 2 | 2 | 3 |
| 1080 | Isoform 1 of Lysine-specific demethylase 3B | IPI00298935 | 0.000 | 0.34115 | 0 | 3 | 1 | 3 |
| 1081 | Casein kinase II subunit beta | IPI00010865 | 0.000 | 0.34115 | 4 | 3 | 3 | 4 |
| 1082 | RNA-binding protein NOB1 | IPI00022373 | 0.000 | 0.34115 | 2 | 3 | 1 | 4 |
| 1083 | EF-hand domain-containing protein D2 | IPI00060181 | 0.000 | 0.34115 | 4 | 2 | 2 | 4 |
| 1084 | Transducin beta-like protein 3 | IPI00477971 | 0.000 | 0.34115 | 3 | 3 | 3 | 3 |
| 1085 | Mitochondrial 18 kDa protein | IPI00784376 | 0.000 | 0.34115 | 3 | 0 | 3 | 1 |
| 1086 | Isoform p26 of 7,8-dihydro-8-oxoguanine triphosphatase | IPI00004392 | 0.000 | 0.34115 | 2 | 3 | 3 | 2 |
| 1087 | DNA-directed RNA polymerases I, II, and III subunit RPABC3 | IPI00003309 | 0.000 | 0.34115 | 4 | 2 | 5 | 0 |
| 1088 | Cell division protein kinase 2 | IPI00031681 | 0.000 | 0.34115 | 2 | 3 | 3 | 2 |
| 1089 | Aspartyl-tRNA synthetase, mitochondrial | IPI00100460 | 0.000 | 0.34115 | 2 | 1 | 2 | 1 |
| 1090 | Isoform 2 of DnaJ homolog subfamily A member 3, mitochondrial | IPI00179187 | 0.000 | 0.34115 | 3 | 2 | 2 | 3 |
| 1091 | DNA polymerase alpha subunit B | IPI00290272 | 0.000 | 0.34115 | 1 | 1 | 1 | 0 |
| 1092 | 39S ribosomal protein L45, mitochondrial | IPI00185859 | 0.000 | 0.34115 | 0 | 0 | 1 | 1 |
| 1093 | 28S ribosomal protein S26, mitochondrial | IPI00006606 | 0.000 | 0.34115 | 3 | 3 | 3 | 3 |
| 1094 | Isoform 2 of Liprin-beta-1 | IPI00179172 | 0.000 | 0.34115 | 4 | 3 | 3 | 4 |
| 1095 | Isoform 1 of Chromodomain-helicase-DNA-binding protein 1 | IPI00297851 | 0.000 | 0.34115 | 3 | 3 | 2 | 4 |
| 1096 | Isoform 2 of COP9 signalosome complex subunit 2 | IPI00018813 | 0.000 | 0.34115 | 1 | 2 | 2 | 1 |
| 1097 | Ras-related protein Rab-5A | IPI00023510 | 0.000 | 0.34115 | 3 | 2 | 3 | 2 |
| 1098 | cDNA FLJ55988, highly similar to RNA-binding protein Luc7-like 2 | IPI00006932 | 0.000 | 0.34115 | 2 | 1 | 2 | 1 |
| 1099 | Isoform 1 of Medium-chain specific acyl-CoA dehydrogenase, mitochondrial | IPI00005040 | 0.000 | 0.34115 | 2 | 3 | 1 | 4 |
| 1100 | Peptidyl-tRNA hydrolase 2, mitochondrial | IPI00032903 | 0.000 | 0.34115 | 5 | 4 | 5 | 4 |
| 1101 | 60S ribosomal protein L30 | IPI00219156 | 0.000 | 0.34115 | 5 | 3 | 4 | 4 |
| 1102 | Exosome complex exonuclease RRP40 | IPI00015956 | 0.000 | 0.34115 | 3 | 1 | 2 | 2 |
| 1103 | Pre-mRNA-processing factor 6 | IPI00305068 | 0.000 | 0.34115 | 3 | 3 | 4 | 2 |
| 1104 | DnaJ homolog subfamily C member 7 | IPI00329629 | 0.000 | 0.34115 | 5 | 3 | 5 | 3 |
| 1105 | Isoform 1 of Far upstream element-binding protein 3 | IPI00377261 | 0.000 | 0.34115 | 1 | 3 | 1 | 3 |
| 1106 | mRNA export factor | IPI00019733 | 0.000 | 0.34115 | 2 | 0 | 2 | 1 |
| 1107 | Cytochrome c1, heme protein, mitochondrial | IPI00029264 | 0.000 | 0.34115 | 0 | 1 | 0 | 1 |
| 1108 | Carbonyl reductase [NADPH] 3 | IPI00290462 | 0.000 | 0.34115 | 5 | 3 | 3 | 5 |
| 1109 | Adenylyl cyclase-associated protein | IPI00939159 | 0.000 | 0.34115 | 2 | 2 | 2 | 2 |
| 1110 | Isoform HERA-A of GTP-binding protein era homolog | IPI00026512 | 0.000 | 0.34115 | 3 | 3 | 3 | 3 |
| 1111 | Hsc70-interacting protein | IPI00032826 | 0.000 | 0.34115 | 4 | 3 | 4 | 3 |
| 1112 | Ribosome production factor 2 homolog | IPI00396329 | 0.000 | 0.34115 | 2 | 1 | 2 | 1 |
| 1113 | DNA-directed RNA polymerase II subunit RPB7 | IPI00218895 | 0.000 | 0.34115 | 2 | 2 | 2 | 2 |
| 1114 | Cell division protein kinase 3 | IPI00023503 | 0.000 | 0.34115 | 2 | 3 | 3 | 2 |
| 1115 | 39S ribosomal protein L37, mitochondrial | IPI00162330 | 0.000 | 0.34115 | 2 | 2 | 2 | 2 |
| 1116 | LanC-like protein 1 | IPI00005724 | 0.000 | 0.34115 | 3 | 2 | 3 | 2 |
| 1117 | Isoform 3 of Tyrosine-protein kinase Lck | IPI00515097 | 0.000 | 0.34115 | 2 | 2 | 3 | 1 |
| 1118 | Pyridoxine-5'-phosphate oxidase | IPI00018272 | 0.000 | 0.34115 | 3 | 3 | 3 | 3 |
| 1119 | GrpE protein homolog 1, mitochondrial | IPI00029557 | 0.000 | 0.34115 | 2 | 2 | 2 | 2 |
| 1120 | Isoform 1 of Phosphoribosyl pyrophosphate synthase-associated protein 1 | IPI00291578 | 0.000 | 0.34115 | 3 | 4 | 4 | 3 |
| 1121 | Putative uncharacterized protein DOCK6 | IPI00184772 | 0.000 | 0.34115 | 0 | 2 | 2 | 1 |
| 1122 | Isoform 2 of Actin-binding protein anillin | IPI00032958 | 0.000 | 0.34115 | 1 | 0 | 0 | 0 |
| 1123 | Ras-related protein Ral-A | IPI00217519 | 0.000 | 0.34115 | 5 | 2 | 4 | 3 |
| 1124 | TP53-regulating kinase | IPI00290305 | 0.000 | 0.34115 | 3 | 2 | 2 | 3 |
| 1125 | Dipeptidase 1 | IPI00059476 | 0.000 | 0.34115 | 3 | 1 | 3 | 0 |
| 1126 | Heat shock 70 kDa protein 12A | IPI00011932 | 0.000 | 0.34115 | 2 | 2 | 3 | 0 |
| 1127 | WD40 repeat-containing protein SMU1 | IPI00305833 | 0.000 | 0.34115 | 2 | 2 | 2 | 2 |
| 1128 | Coiled-coil domain-containing protein 124 | IPI00060627 | 0.000 | 0.34115 | 3 | 2 | 3 | 2 |
| 1129 | Isoform 1 of Protein LSM12 homolog | IPI00410324 | 0.000 | 0.34115 | 3 | 4 | 4 | 3 |
| 1130 | Tyrosine-protein phosphatase non-receptor type 23 | IPI00034006 | 0.000 | 0.34115 | 1 | 1 | 1 | 1 |
| 1131 | Isoform 2 of Leucine-rich repeat flightless-interacting protein 1 | IPI00006207 | 0.000 | 0.34115 | 3 | 2 | 2 | 3 |
| 1132 | Transmembrane protein 2 | IPI00170706 | 0.000 | 0.34115 | 1 | 2 | 1 | 2 |
| 1133 | Isoform 2 of 3-hydroxyisobutyryl-CoA hydrolase, mitochondrial | IPI00377161 | 0.000 | 0.34115 | 2 | 5 | 6 | 1 |
| 1134 | Phosphomannomutase 2 | IPI00006092 | 0.000 | 0.34115 | 3 | 3 | 2 | 4 |
| 1135 | Regulator of microtubule dynamics protein 1 | IPI00329696 | 0.000 | 0.34115 | 3 | 3 | 4 | 2 |
| 1136 | Putative uncharacterized protein PYCR2 | IPI00335061 | 0.000 | 0.34115 | 4 | 3 | 4 | 3 |

| No. | Description | Accession number | STN[1] | p-Value[1] | Con_A[2] | Con_B[2] | LUTEO_A[2] | LUTEO_B[2] |
|---|---|---|---|---|---|---|---|---|
| 1137 | Isoform 2 of Transcription elongation factor SPT6 | IPI00430770 | 0.000 | 0.34115 | 2 | 2 | 3 | 1 |
| 1138 | RNA-binding protein 12 | IPI00550308 | 0.000 | 0.34115 | 2 | 1 | 2 | 0 |
| 1139 | Methyltransferase like 7B | IPI00090807 | 0.000 | 0.34115 | 2 | 2 | 3 | 0 |
| 1140 | WD repeat-containing protein 5 | IPI00005492 | 0.000 | 0.34115 | 3 | 4 | 4 | 3 |
| 1141 | Isoform 1 of Probable ATP-dependent RNA helicase DHX36 | IPI00027415 | 0.000 | 0.34115 | 3 | 2 | 3 | 2 |
| 1142 | Isoform 2 of NudC domain-containing protein 1 | IPI00306398 | 0.000 | 0.34115 | 1 | 1 | 1 | 0 |
| 1143 | Bystin | IPI00328987 | 0.000 | 0.34115 | 1 | 1 | 1 | 1 |
| 1144 | Putative uncharacterized protein ATP5J2 | IPI00219291 | 0.000 | 0.34115 | 3 | 3 | 3 | 3 |
| 1145 | Isoform 1 of Ubiquitin carboxyl-terminal hydrolase 34 | IPI00297593 | 0.000 | 0.34115 | 1 | 2 | 2 | 0 |
| 1146 | Trafficking protein particle complex subunit 3 | IPI00004324 | 0.000 | 0.34115 | 2 | 2 | 2 | 2 |
| 1147 | Flotillin-1 | IPI00027438 | 0.000 | 0.34115 | 3 | 3 | 2 | 4 |
| 1148 | RWD domain-containing protein 1 | IPI00034010 | 0.000 | 0.34115 | 2 | 1 | 2 | 1 |
| 1149 | Coproporphyrinogen-III oxidase, mitochondrial | IPI00093057 | 0.000 | 0.34115 | 1 | 3 | 2 | 2 |
| 1150 | TBC1 domain family member 15 isoform 1 | IPI00154645 | 0.000 | 0.34115 | 2 | 2 | 2 | 2 |
| 1151 | Isoform 2 of Transcription elongation factor A protein 1 | IPI00218106 | 0.000 | 0.34115 | 0 | 2 | 0 | 2 |
| 1152 | Isoform 4 of Uncharacterized protein KIAA0090 | IPI00642244 | 0.000 | 0.34115 | 2 | 2 | 3 | 1 |
| 1153 | Thioredoxin-like protein 4B | IPI00016481 | 0.000 | 0.34115 | 3 | 0 | 3 | 1 |
| 1154 | Putative uncharacterized protein DCP1A | IPI00164672 | 0.000 | 0.34115 | 2 | 1 | 1 | 2 |
| 1155 | Isoform 1 of Lariat debranching enzyme | IPI00305545 | 0.000 | 0.34115 | 2 | 2 | 1 | 3 |
| 1156 | Elongation factor G 2, mitochondrial precursor | IPI00071703 | 0.000 | 0.34115 | 2 | 2 | 2 | 2 |
| 1157 | Vacuolar protein sorting-associated protein 45 | IPI00090327 | 0.000 | 0.34115 | 1 | 2 | 0 | 2 |
| 1158 | Carboxymethylenebutenolidase homolog | IPI00383046 | 0.000 | 0.34115 | 1 | 0 | 0 | 1 |
| 1159 | Immunity-related GTPase family Q protein | IPI00103925 | 0.000 | 0.34115 | 1 | 2 | 2 | 1 |
| 1160 | Probable saccharopine dehydrogenase | IPI00329600 | 0.000 | 0.34115 | 0 | 0 | 1 | 1 |
| 1161 | Isoform 1 of Rho guanine nucleotide exchange factor 2 | IPI00291316 | 0.000 | 0.34115 | 1 | 0 | 1 | 1 |
| 1162 | RNA 3'-terminal phosphate cyclase-like protein | IPI00294229 | 0.000 | 0.34115 | 3 | 3 | 2 | 4 |
| 1163 | Probable ATP-dependent RNA helicase DDX41 | IPI00007208 | 0.000 | 0.34115 | 0 | 1 | 1 | 1 |
| 1164 | Synaptogyrin-2 | IPI00013946 | 0.000 | 0.34115 | 2 | 1 | 0 | 2 |
| 1165 | Isoform 3 of Pre-mRNA 3'-end-processing factor FIP1 | IPI00008449 | 0.000 | 0.34115 | 1 | 0 | 1 | 1 |
| 1166 | NEDD8-activating enzyme E1 regulatory subunit | IPI00018968 | 0.000 | 0.34115 | 2 | 4 | 4 | 2 |
| 1167 | Isoform 1 of Signal transducing adapter molecule 1 | IPI00020178 | 0.000 | 0.34115 | 2 | 1 | 2 | 1 |
| 1168 | Splicing factor, arginine/serine-rich 11 | IPI00464952 | 0.000 | 0.34115 | 2 | 3 | 2 | 3 |
| 1169 | Retinol dehydrogenase 13 | IPI00301204 | 0.000 | 0.34115 | 3 | 2 | 3 | 2 |
| 1170 | Isoform 1 of FAD synthase | IPI00220299 | 0.000 | 0.34115 | 1 | 1 | 1 | 1 |
| 1171 | tRNA-dihydrouridine synthase 2-like | IPI00015804 | 0.000 | 0.34115 | 1 | 0 | 1 | 1 |
| 1172 | U4/U6.U5 tri-snRNP-associated protein 1 | IPI00021417 | 0.000 | 0.34115 | 3 | 2 | 3 | 2 |
| 1173 | Uncharacterized protein C20orf4 | IPI00166013 | 0.000 | 0.34115 | 3 | 3 | 3 | 3 |
| 1174 | Succinate dehydrogenase [ubiquinone] iron-sulfur subunit, mitochondrial | IPI00294911 | 0.000 | 0.34115 | 3 | 2 | 3 | 2 |
| 1175 | Isoform 2 of Ubiquilin-1 | IPI00071180 | 0.000 | 0.34115 | 1 | 2 | 2 | 1 |
| 1176 | Isoform Mitochondrial of Phospholipid hydroperoxide glutathione peroxidase, mitochondrial | IPI00304814 | 0.000 | 0.34115 | 2 | 2 | 2 | 2 |
| 1177 | Isoform 1 of Kinesin-like protein KIF21A | IPI00425404 | 0.000 | 0.34115 | 2 | 1 | 2 | 0 |
| 1178 | 18 kDa protein | IPI00797709 | 0.000 | 0.34115 | 2 | 0 | 2 | 1 |
| 1179 | Isoform 3 of THO complex subunit 6 homolog | IPI00301252 | 0.000 | 0.34115 | 2 | 2 | 3 | 1 |
| 1180 | Isoform 2 of Chromodomain-helicase-DNA-binding protein 2 | IPI00023109 | 0.000 | 0.34115 | 2 | 2 | 2 | 2 |
| 1181 | Isoform 3 of Sorting nexin-3 | IPI00029740 | 0.000 | 0.34115 | 1 | 2 | 2 | 0 |
| 1182 | Exosome complex exonuclease MTR3 | IPI00073602 | 0.000 | 0.34115 | 2 | 1 | 2 | 1 |
| 1183 | Isoform 1 of Protein FAM115A | IPI00006050 | 0.000 | 0.34115 | 0 | 0 | 0 | 1 |
| 1184 | cDNA FLJ56840, highly similar to Galactokinase | IPI00019383 | 0.000 | 0.34115 | 1 | 3 | 2 | 2 |
| 1185 | Isoform 1 of CTD small phosphatase-like protein 2 | IPI00033054 | 0.000 | 0.34115 | 2 | 2 | 2 | 2 |
| 1186 | Receptor expression-enhancing protein 5 | IPI00024670 | 0.000 | 0.34115 | 3 | 1 | 2 | 2 |
| 1187 | Acidic leucine-rich nuclear phosphoprotein 32 family member C | IPI00018262 | 0.000 | 0.34115 | 2 | 3 | 2 | 3 |
| 1188 | Isoform LAMP-2A of Lysosome-associated membrane glycoprotein 2 | IPI00009030 | 0.000 | 0.34115 | 1 | 1 | 1 | 1 |
| 1189 | GTP-binding protein 1 | IPI00010463 | 0.000 | 0.34115 | 2 | 2 | 2 | 2 |
| 1190 | Isoform 2 of Exosome complex exonuclease RRP45 | IPI00029697 | 0.000 | 0.34115 | 3 | 2 | 3 | 2 |
| 1191 | STE20/SPS1-related proline-alanine-rich protein kinase | IPI00004363 | 0.000 | 0.34115 | 1 | 3 | 3 | 1 |
| 1192 | GA-binding protein alpha chain | IPI00299413 | 0.000 | 0.34115 | 2 | 1 | 2 | 0 |
| 1193 | Ras-related protein Rab-8B | IPI00024282 | 0.000 | 0.34115 | 2 | 2 | 2 | 2 |
| 1194 | Periplakin | IPI00298057 | 0.000 | 0.34115 | 0 | 0 | 0 | 1 |
| 1195 | Cytochrome c oxidase subunit 5B, mitochondrial | IPI00021785 | 0.000 | 0.34115 | 2 | 2 | 3 | 1 |
| 1196 | Cytochrome b-c1 complex subunit 7 | IPI00220416 | 0.000 | 0.34115 | 2 | 2 | 2 | 2 |
| 1197 | AP-1 complex subunit sigma-2 | IPI00922006 | 0.000 | 0.34115 | 2 | 2 | 3 | 1 |
| 1198 | Mitochondrial chaperone BCS1 | IPI00003985 | 0.000 | 0.34115 | 2 | 1 | 1 | 2 |
| 1199 | Isoform SMN of Survival motor neuron protein | IPI00003394 | 0.000 | 0.34115 | 2 | 1 | 2 | 1 |
| 1200 | Trimeric intracellular cation channel type B | IPI00018237 | 0.000 | 0.34115 | 1 | 1 | 0 | 1 |
| 1201 | Calcium homeostasis endoplasmic reticulum protein | IPI00333010 | 0.000 | 0.34115 | 1 | 1 | 1 | 1 |
| 1202 | Isoform Beta of DNA ligase 3 | IPI00000156 | 0.000 | 0.34115 | 1 | 2 | 1 | 2 |
| 1203 | Isoform 1 of Vacuolar protein sorting-associated protein 18 homolog | IPI00001985 | 0.000 | 0.34115 | 0 | 0 | 0 | 1 |
| 1204 | Isoform 1 of Putative methyltransferase METT10D | IPI00163391 | 0.000 | 0.34115 | 1 | 0 | 0 | 1 |
| 1205 | Isoform 1 of ATP synthase subunit d, mitochondrial | IPI00220487 | 0.000 | 0.34115 | 2 | 2 | 2 | 2 |
| 1206 | Coiled-coil domain-containing protein 86 | IPI00012199 | 0.000 | 0.34115 | 0 | 0 | 1 | 0 |
| 1207 | Isoform 1 of Platelet-activating factor acetylhydrolase IB subunit alpha | IPI00218728 | 0.000 | 0.34115 | 2 | 2 | 0 | 3 |
| 1208 | Isoform 2 of Arf-GAP with Rho-GAP domain, ANK repeat and PH domain-containing protein 1 | IPI00220421 | 0.000 | 0.34115 | 1 | 1 | 1 | 1 |
| 1209 | cDNA: FLJ22221 fis, clone HRC01651 | IPI00184854 | 0.000 | 0.34115 | 0 | 0 | 1 | 0 |
| 1210 | Small nuclear ribonucleoprotein F | IPI00220528 | 0.000 | 0.34115 | 2 | 3 | 2 | 3 |
| 1211 | Isoform 1 of Trafficking protein particle complex subunit 2 | IPI00005119 | 0.000 | 0.34115 | 0 | 2 | 2 | 1 |
| 1212 | THUMP domain-containing protein 3 | IPI00306127 | 0.000 | 0.34115 | 2 | 0 | 1 | 2 |
| 1213 | CDKN2A-interacting protein | IPI00020991 | 0.000 | 0.34115 | 0 | 0 | 1 | 1 |
| 1214 | Procollagen galactosyltransferase 1 | IPI00168262 | 0.000 | 0.34115 | 1 | 1 | 0 | 0 |
| 1215 | Dynactin subunit 4 | IPI00550852 | 0.000 | 0.34115 | 1 | 2 | 2 | 0 |
| 1216 | Alpha-taxilin | IPI00470779 | 0.000 | 0.34115 | 1 | 1 | 0 | 1 |
| 1217 | CDGSH iron sulfur domain-containing protein 1 | IPI00020510 | 0.000 | 0.34115 | 3 | 2 | 3 | 2 |
| 1218 | Isoform 1 of Partner of Y14 and mago | IPI00305092 | 0.000 | 0.34115 | 0 | 2 | 1 | 2 |
| 1219 | Isoform 1 of Zinc finger protein 207 | IPI00013457 | 0.000 | 0.34115 | 0 | 2 | 0 | 2 |
| 1220 | 39S ribosomal protein L27, mitochondrial | IPI00009444 | 0.000 | 0.34115 | 1 | 1 | 1 | 1 |
| 1221 | Armadillo repeat-containing protein 1 | IPI00018260 | 0.000 | 0.34115 | 2 | 2 | 3 | 1 |
| 1222 | NEDD4-like E3 ubiquitin-protein ligase WWP2 | IPI00013010 | 0.000 | 0.34115 | 3 | 2 | 2 | 3 |
| 1223 | Cyclin B1 | IPI00294696 | 0.000 | 0.34115 | 0 | 0 | 1 | 1 |
| 1224 | Exosome complex exonuclease RRP46 | IPI00015955 | 0.000 | 0.34115 | 2 | 2 | 2 | 2 |
| 1225 | Isoform 1 of Annexin A7 | IPI00002460 | 0.000 | 0.34115 | 2 | 0 | 1 | 2 |
| 1226 | Isoform 1 of Golgin subfamily A member 3 | IPI00305267 | 0.000 | 0.34115 | 1 | 1 | 0 | 0 |
| 1227 | Isoform 1 of FAD-dependent oxidoreductase domain-containing protein 1 | IPI00549357 | 0.000 | 0.34115 | 1 | 1 | 0 | 0 |
| 1228 | 67 kDa protein | IPI00010365 | 0.000 | 0.34115 | 1 | 1 | 1 | 1 |
| 1229 | Isoform A of GC-rich sequence DNA-binding factor homolog | IPI00001364 | 0.000 | 0.34115 | 0 | 0 | 0 | 0 |
| 1230 | Maspardin | IPI00010248 | 0.000 | 0.34115 | 1 | 1 | 1 | 1 |
| 1231 | Isoform 1 of Poly(A) RNA polymerase, mitochondrial | IPI00153051 | 0.000 | 0.34115 | 1 | 1 | 1 | 1 |

| No. | Description | Accession number | STN[1] | p-Value[1] | Con_A[2] | Con_B[2] | LUTEO_A[2] | LUTEO_B[2] |
|---|---|---|---|---|---|---|---|---|
| 1232 | Isoform Membrane-bound of Catechol O-methyltransferase | IPI00011284 | 0.000 | 0.34115 | 0 | 0 | 1 | 0 |
| 1233 | Isoform 2 of Tether containing UBX domain for GLUT4 | IPI00065276 | 0.000 | 0.34115 | 1 | 1 | 1 | 1 |
| 1234 | Nuclear receptor coactivator 5 | IPI00288941 | 0.000 | 0.34115 | 1 | 1 | 1 | 0 |
| 1235 | Isoform 1 of Serine/threonine-protein phosphatase 4 regulatory subunit 1 | IPI00411730 | 0.000 | 0.34115 | 0 | 1 | 0 | 0 |
| 1236 | cytochrome c oxidase subunit VIIa polypeptide 2 (liver) precursor | IPI00026570 | 0.000 | 0.34115 | 2 | 2 | 2 | 2 |
| 1237 | Isoform A of Protein CutA | IPI00034319 | 0.000 | 0.34115 | 0 | 1 | 0 | 1 |
| 1238 | Probable ergosterol biosynthetic protein 28 | IPI00007730 | 0.000 | 0.34115 | 2 | 1 | 2 | 1 |
| 1239 | dehydrogenase/reductase SDR family member 4 | IPI00106913 | 0.000 | 0.34115 | 1 | 1 | 1 | 1 |
| 1240 | Active regulator of SIRT1 | IPI00219006 | 0.000 | 0.34115 | 2 | 2 | 2 | 2 |
| 1241 | Isoform 3 of Cytosolic 5'-nucleotidase 3 | IPI00100192 | 0.000 | 0.34115 | 1 | 1 | 1 | 0 |
| 1242 | Cell cycle regulator Mat89Bb homolog | IPI00550986 | 0.000 | 0.34115 | 1 | 2 | 2 | 1 |
| 1243 | Isoform 1 of E3 ubiquitin-protein ligase Itchy homolog | IPI00061780 | 0.000 | 0.34115 | 1 | 2 | 2 | 0 |
| 1244 | Sororin | IPI00061989 | 0.000 | 0.34115 | 0 | 0 | 0 | 0 |
| 1245 | 3'-5' exoribonuclease CSL4 homolog | IPI00032823 | 0.000 | 0.34115 | 2 | 2 | 2 | 2 |
| 1246 | Isoform 3 of Centromere protein V | IPI00376481 | 0.000 | 0.34115 | 2 | 2 | 2 | 2 |
| 1247 | Target of EGR1 protein 1 | IPI00549516 | 0.000 | 0.34115 | 1 | 2 | 1 | 2 |
| 1248 | Isoform 1 of COMM domain-containing protein 4 | IPI00413500 | 0.000 | 0.34115 | 1 | 0 | 0 | 0 |
| 1249 | Serine/threonine-protein kinase Nek7 | IPI00152658 | 0.000 | 0.34115 | 2 | 1 | 2 | 0 |
| 1250 | Isoform 2 of Protein PAT1 homolog 1 | IPI00760958 | 0.000 | 0.34115 | 0 | 1 | 1 | 1 |
| 1251 | Secernin-1 | IPI00289862 | 0.000 | 0.34115 | 2 | 2 | 2 | 2 |
| 1252 | Golgi resident protein GCP60 | IPI00009315 | 0.000 | 0.34115 | 3 | 0 | 2 | 2 |
| 1253 | Ribonuclease UK114 | IPI00005038 | 0.000 | 0.34115 | 2 | 2 | 3 | 1 |
| 1254 | Sedoheptulokinase | IPI00005914 | 0.000 | 0.34115 | 2 | 2 | 2 | 2 |
| 1255 | cDNA FLJ56370, highly similar to Homo sapiens FK506 binding protein 8, 38kDa (FKBP8), mRNA | IPI00328161 | 0.000 | 0.34115 | 1 | 2 | 1 | 2 |
| 1256 | Protein KTI12 homolog | IPI00061528 | 0.000 | 0.34115 | 1 | 2 | 2 | 1 |
| 1257 | NHP2-like protein 1 | IPI00026167 | 0.000 | 0.34115 | 2 | 2 | 2 | 2 |
| 1258 | Mitochondrial import inner membrane translocase subunit Tim13 | IPI00001589 | 0.000 | 0.34115 | 2 | 1 | 2 | 1 |
| 1259 | Isoform 3 of Fermitin family homolog 1 | IPI00220602 | 0.000 | 0.34115 | 0 | 3 | 3 | 1 |
| 1260 | Isoform 1 of Collagen type IV alpha-3-binding protein | IPI00024701 | 0.000 | 0.34115 | 1 | 1 | 0 | 0 |
| 1261 | Vacuolar fusion protein MON1 homolog B | IPI00848138 | 0.000 | 0.34115 | 1 | 1 | 1 | 1 |
| 1262 | DDB1- and CUL4-associated factor 7 | IPI00006754 | 0.000 | 0.34115 | 1 | 2 | 2 | 1 |
| 1263 | Isoform 1 of Transmembrane protein 126B | IPI00020540 | 0.000 | 0.34115 | 1 | 1 | 0 | 1 |
| 1264 | Branched-chain-amino-acid aminotransferase | IPI00181135 | 0.000 | 0.34115 | 1 | 2 | 2 | 1 |
| 1265 | Isoform 1 of Stromal membrane-associated protein 1 | IPI00102096 | 0.000 | 0.34115 | 1 | 2 | 1 | 2 |
| 1266 | Nucleolar protein 9 | IPI00002902 | 0.000 | 0.34115 | 1 | 0 | 0 | 0 |
| 1267 | Guanine nucleotide-binding protein G(q) subunit alpha | IPI00288947 | 0.000 | 0.34115 | 2 | 1 | 1 | 2 |
| 1268 | Isoform Rpn10A of 26S proteasome non-ATPase regulatory subunit 4 | IPI00022694 | 0.000 | 0.34115 | 1 | 1 | 0 | 1 |
| 1269 | Biogenesis of lysosome-related organelles complex 1 subunit 1 | IPI00020319 | 0.000 | 0.34115 | 1 | 2 | 2 | 0 |
| 1270 | Ras-related protein Rab-20 | IPI00015839 | 0.000 | 0.34115 | 3 | 0 | 3 | 0 |
| 1271 | Beta-centractin | IPI00029469 | 0.000 | 0.34115 | 1 | 1 | 1 | 1 |
| 1272 | HIV Tat-specific factor 1 | IPI00013788 | 0.000 | 0.34115 | 1 | 1 | 1 | 0 |
| 1273 | Isoform Alpha of Caspase-6 | IPI00023876 | 0.000 | 0.34115 | 1 | 1 | 0 | 1 |
| 1274 | COMM domain-containing protein 3 | IPI00015773 | 0.000 | 0.34115 | 1 | 1 | 1 | 1 |
| 1275 | Protein FAM98B | IPI00167572 | 0.000 | 0.34115 | 1 | 2 | 2 | 1 |
| 1276 | Ubiquitin-conjugating enzyme E2 R2 | IPI00418603 | 0.000 | 0.34115 | 2 | 1 | 1 | 2 |
| 1277 | CDC45-related protein | IPI00025695 | 0.000 | 0.34115 | 1 | 1 | 0 | 1 |
| 1278 | Sideroflexin-2 | IPI00043564 | 0.000 | 0.34115 | 1 | 0 | 1 | 1 |
| 1279 | Isoform 1 of CWF19-like protein 1 | IPI00101600 | 0.000 | 0.34115 | 0 | 1 | 0 | 0 |
| 1280 | Isoform 1 of Alpha-globin transcription factor CP2 | IPI00037599 | 0.000 | 0.34115 | 0 | 3 | 2 | 2 |
| 1281 | NADH-ubiquinone oxidoreductase chain 5 | IPI00008511 | 0.000 | 0.34115 | 0 | 2 | 0 | 2 |
| 1282 | Isoform 2 of Gamma-glutamylcyclotransferase | IPI00020301 | 0.000 | 0.34115 | 2 | 1 | 2 | 1 |
| 1283 | Galactosylgalactosylxylosylprotein 3-beta-glucuronosyltransferase 3 | IPI00304331 | 0.000 | 0.34115 | 1 | 2 | 2 | 1 |
| 1284 | DNA repair protein RAD51 homolog 3 | IPI00012829 | 0.000 | 0.34115 | 0 | 1 | 0 | 1 |
| 1285 | Isoform 5 of Protein transport protein Sec16A | IPI00031242 | 0.000 | 0.34115 | 0 | 0 | 1 | 1 |
| 1286 | Isoform 1 of Centrosomal protein of 170 kDa | IPI00186194 | 0.000 | 0.34115 | 0 | 0 | 0 | 0 |
| 1287 | Isoform 1 of Mediator of RNA polymerase II transcription subunit 27 | IPI00302652 | 0.000 | 0.34115 | 1 | 1 | 0 | 0 |
| 1288 | Myotubularin | IPI00748788 | 0.000 | 0.34115 | 1 | 0 | 0 | 0 |
| 1289 | UDP-galactose-4-epimerase | IPI00030229 | 0.000 | 0.34115 | 1 | 1 | 1 | 1 |
| 1290 | WD repeat-containing protein 70 | IPI00300060 | 0.000 | 0.34115 | 1 | 1 | 1 | 1 |
| 1291 | Isoform 1 of Pre-mRNA-splicing factor 38A | IPI00171390 | 0.000 | 0.34115 | 1 | 1 | 1 | 1 |
| 1292 | PIH1 domain-containing protein 1 | IPI00550995 | 0.000 | 0.34115 | 2 | 1 | 2 | 0 |
| 1293 | Ras-related protein Rab-24 | IPI00056496 | 0.000 | 0.34115 | 1 | 0 | 1 | 1 |
| 1294 | Isoform 1 of Glycerophosphodiester phosphodiesterase domain-containing protein 1 | IPI00298535 | 0.000 | 0.34115 | 0 | 0 | 0 | 0 |
| 1295 | Protein phosphatase 1 regulatory subunit 11 | IPI00030355 | 0.000 | 0.34115 | 1 | 1 | 1 | 1 |
| 1296 | Isoform 1 of SOSS complex subunit B1 | IPI00031633 | 0.000 | 0.34115 | 0 | 0 | 0 | 0 |
| 1297 | Isoform 1 of Interleukin enhancer-binding factor 3 | IPI00298788 | 0.000 | 0.34115 | 1 | 1 | 1 | 1 |
| 1298 | Isoform 1 of Craniofacial development protein 1 | IPI00007306 | 0.000 | 0.34115 | 0 | 1 | 0 | 0 |
| 1299 | Isoform 2 of Oxidation resistance protein 1 | IPI00298348 | 0.000 | 0.34115 | 0 | 1 | 0 | 0 |
| 1300 | My002 protein | IPI00023584 | 0.000 | 0.34115 | 1 | 0 | 0 | 1 |
| 1301 | Translation initiation factor eIF-2B subunit beta | IPI00028083 | 0.000 | 0.34115 | 0 | 0 | 1 | 1 |
| 1302 | tRNA pseudouridine synthase 3 | IPI00306260 | 0.000 | 0.34115 | 0 | 1 | 0 | 0 |
| 1303 | Macrophage-capping protein | IPI00027341 | 0.000 | 0.34115 | 0 | 1 | 1 | 0 |
| 1304 | Glyoxylate reductase/hydroxypyruvate reductase, isoform CRA_c | IPI00026486 | 0.000 | 0.34115 | 1 | 0 | 0 | 1 |
| 1305 | Annexin A5 | IPI00329801 | -0.092 | 0.37894 | 50 | 38 | 41 | 46 |
| 1306 | Isoform alpha-enolase of Alpha-enolase | IPI00465248 | -0.094 | 0.37889 | 237 | 233 | 240 | 228 |
| 1307 | Leucyl-tRNA synthetase, cytoplasmic | IPI00103994 | -0.107 | 0.37794 | 29 | 30 | 31 | 27 |
| 1308 | 40S ribosomal protein S5 | IPI00008433 | -0.111 | 0.37728 | 27 | 27 | 28 | 25 |
| 1309 | 40S ribosomal protein S2 | IPI00013485 | -0.113 | 0.37690 | 24 | 28 | 26 | 25 |
| 1310 | Isoform 1 of 40S ribosomal protein S24 | IPI00029750 | -0.122 | 0.37438 | 24 | 19 | 23 | 19 |
| 1311 | Isoform 1 of Myoferlin | IPI00021048 | -0.126 | 0.37396 | 15 | 25 | 19 | 20 |
| 1312 | V-type proton ATPase subunit B, brain isoform | IPI00007812 | -0.128 | 0.37381 | 18 | 20 | 15 | 22 |
| 1313 | 60S ribosomal protein L6 | IPI00329389 | -0.131 | 0.37334 | 21 | 15 | 20 | 15 |
| 1314 | 26S protease regulatory subunit 4 | IPI00011126 | -0.133 | 0.37267 | 19 | 16 | 15 | 19 |
| 1315 | Isoform 2 of Annexin A2 | IPI00418169 | -0.134 | 0.37239 | 17 | 17 | 15 | 18 |
| 1316 | Tu translation elongation factor, mitochondrial precursor | IPI00027107 | -0.136 | 0.37187 | 17 | 16 | 17 | 15 |
| 1317 | Phenylalanyl-tRNA synthetase beta chain | IPI00300074 | -0.136 | 0.37187 | 18 | 15 | 17 | 15 |
| 1318 | Translin | IPI00018768 | -0.136 | 0.37187 | 15 | 18 | 17 | 15 |
| 1319 | Isoform 4 of Heterogeneous nuclear ribonucleoprotein A/B | IPI00106509 | -0.137 | 0.37153 | 16 | 16 | 18 | 13 |
| 1320 | Multifunctional protein ADE2 | IPI00217223 | -0.137 | 0.37153 | 15 | 17 | 16 | 15 |
| 1321 | Proteasome subunit beta type-3 | IPI00028004 | -0.139 | 0.37039 | 16 | 15 | 19 | 11 |
| 1322 | 40S ribosomal protein S7 | IPI00013415 | -0.141 | 0.36959 | 14 | 16 | 15 | 14 |
| 1323 | Isoform Mitochondrial of Peroxiredoxin-5, mitochondrial | IPI00024915 | -0.143 | 0.36945 | 13 | 16 | 14 | 14 |
| 1324 | Leucine-rich repeat-containing protein 59 | IPI00396321 | -0.145 | 0.36873 | 16 | 12 | 15 | 12 |
| 1325 | Isoform 2 of Spliceosome RNA helicase BAT1 | IPI00641829 | -0.147 | 0.36793 | 13 | 14 | 14 | 12 |
| 1326 | Nucleoporin 85 | IPI00171542 | -0.154 | 0.36612 | 13 | 11 | 12 | 11 |

| No. | Description | Accession number | STN[1] | p-Value[1] | Con_A[2] | Con_B[2] | LUTEO_A[2] | LUTEO_B[2] |
|---|---|---|---|---|---|---|---|---|
| 1327 | Aspartate aminotransferase, cytoplasmic | IPI00219029 | -0.157 | 0.36470 | 11 | 12 | 12 | 10 |
| 1328 | Exosome complex exonuclease RRP4 | IPI00015905 | -0.157 | 0.36470 | 13 | 10 | 10 | 12 |
| 1329 | Proliferation-associated protein 2G4 | IPI00299000 | -0.160 | 0.36403 | 10 | 12 | 10 | 11 |
| 1330 | Condensin-2 complex subunit D3 | IPI00747787 | -0.160 | 0.36403 | 9 | 13 | 12 | 9 |
| 1331 | Microsomal glutathione S-transferase 3 | IPI00024266 | -0.160 | 0.36403 | 11 | 11 | 11 | 10 |
| 1332 | Gamma-enolase | IPI00216171 | -0.160 | 0.36332 | 65 | 58 | 61 | 60 |
| 1333 | Isoform 1 of Enhancer of mRNA-decapping protein 4 | IPI00376317 | -0.163 | 0.36313 | 9 | 12 | 9 | 11 |
| 1334 | Nuclear cap-binding protein subunit 1 | IPI00019380 | -0.163 | 0.36313 | 12 | 9 | 8 | 12 |
| 1335 | Isoform 2 of Cytoplasmic FMR1-interacting protein 1 | IPI00550212 | -0.163 | 0.36313 | 11 | 10 | 10 | 10 |
| 1336 | Sodium/potassium-transporting ATPase subunit beta-3 | IPI00008167 | -0.163 | 0.36313 | 11 | 10 | 11 | 9 |
| 1337 | Isoform 1 of Glycerol-3-phosphate dehydrogenase, mitochondrial | IPI00017895 | -0.166 | 0.36266 | 11 | 9 | 11 | 8 |
| 1338 | Fascin | IPI00163187 | -0.166 | 0.36266 | 12 | 8 | 9 | 10 |
| 1339 | Tricarboxylate transport protein, mitochondrial | IPI00294159 | -0.170 | 0.36076 | 10 | 9 | 10 | 8 |
| 1340 | Flap endonuclease 1 | IPI00026215 | -0.174 | 0.35967 | 7 | 11 | 7 | 10 |
| 1341 | 29 kDa protein | IPI00453476 | -0.174 | 0.35967 | 10 | 8 | 9 | 8 |
| 1342 | Isoform 1 of RNA-binding protein 25 | IPI00004273 | -0.174 | 0.35967 | 8 | 10 | 9 | 8 |
| 1343 | Isoform 1 of Enoyl-CoA hydratase domain-containing protein 1 | IPI00302688 | -0.174 | 0.35967 | 12 | 6 | 8 | 9 |
| 1344 | 6-phosphogluconolactonase | IPI00029997 | -0.174 | 0.35967 | 10 | 8 | 10 | 7 |
| 1345 | Protein FAM49B | IPI00303318 | -0.178 | 0.35781 | 9 | 8 | 9 | 7 |
| 1346 | 26S protease regulatory subunit 8 | IPI00023919 | -0.182 | 0.35615 | 10 | 6 | 8 | 7 |
| 1347 | Isoform 1 of Mitotic checkpoint protein BUB3 | IPI00013468 | -0.182 | 0.35615 | 9 | 7 | 7 | 8 |
| 1348 | Replication factor C subunit 4 | IPI00017381 | -0.182 | 0.35615 | 8 | 8 | 7 | 8 |
| 1349 | 32 kDa protein | IPI00399077 | -0.182 | 0.35615 | 9 | 7 | 6 | 9 |
| 1350 | DCN1-like protein 5 | IPI00165361 | -0.187 | 0.35435 | 7 | 8 | 8 | 6 |
| 1351 | Biliverdin reductase A | IPI00294158 | -0.187 | 0.35435 | 8 | 7 | 6 | 8 |
| 1352 | Ubiquitin carboxyl-terminal hydrolase 24 | IPI00902614 | -0.192 | 0.35302 | 5 | 9 | 5 | 8 |
| 1353 | 165 kDa protein | IPI00240812 | -0.192 | 0.35302 | 6 | 8 | 6 | 7 |
| 1354 | Isoform Short of RNA-binding protein FUS | IPI00221354 | -0.192 | 0.35302 | 8 | 6 | 7 | 6 |
| 1355 | CCAAT/enhancer-binding protein zeta | IPI00306723 | -0.192 | 0.35302 | 8 | 6 | 8 | 5 |
| 1356 | Translation initiation factor eIF-2B subunit alpha | IPI00221300 | -0.192 | 0.35302 | 7 | 7 | 6 | 7 |
| 1357 | Cytochrome c | IPI00465315 | -0.192 | 0.35302 | 9 | 5 | 7 | 6 |
| 1358 | Isoform NELF-C of Negative elongation factor C/D | IPI00164949 | -0.192 | 0.35302 | 7 | 7 | 6 | 7 |
| 1359 | ADP-ribosylation factor 6 | IPI00215920 | -0.192 | 0.35302 | 8 | 6 | 7 | 6 |
| 1360 | Pyrroline-5-carboxylate reductase 2 | IPI00470610 | -0.192 | 0.35302 | 8 | 6 | 8 | 5 |
| 1361 | T-complex protein 1 subunit gamma isoform b | IPI00290770 | -0.194 | 0.35084 | 38 | 39 | 38 | 37 |
| 1362 | EH domain-containing protein 1 | IPI00017184 | -0.198 | 0.35060 | 7 | 6 | 6 | 6 |
| 1363 | Nodal modulator 1 | IPI00329352 | -0.198 | 0.35060 | 7 | 6 | 6 | 6 |
| 1364 | Isoform 2 of Guanine nucleotide-binding protein-like 3 | IPI00003886 | -0.198 | 0.35060 | 6 | 7 | 6 | 6 |
| 1365 | BRI3-binding protein | IPI00103599 | -0.198 | 0.35060 | 8 | 5 | 6 | 6 |
| 1366 | Acidic leucine-rich nuclear phosphoprotein 32 family member E | IPI00165393 | -0.198 | 0.35060 | 6 | 7 | 6 | 6 |
| 1367 | Isoform 1 of DDRGK domain-containing protein 1 | IPI00028387 | -0.198 | 0.35060 | 7 | 6 | 6 | 6 |
| 1368 | Ribosome biogenesis protein BRX1 homolog | IPI00181728 | -0.205 | 0.34898 | 6 | 6 | 4 | 7 |
| 1369 | Signal peptidase complex catalytic subunit SEC11A | IPI00104128 | -0.205 | 0.34898 | 7 | 5 | 7 | 4 |
| 1370 | Core histone macro-H2A.2 | IPI00220994 | -0.205 | 0.34898 | 6 | 6 | 5 | 6 |
| 1371 | Isoform 1 of Transmembrane and coiled-coil domain-containing protein 1 | IPI00026111 | -0.205 | 0.34898 | 7 | 5 | 6 | 5 |
| 1372 | 3-ketoacyl-CoA thiolase, mitochondrial | IPI00001539 | -0.205 | 0.34898 | 4 | 8 | 5 | 6 |
| 1373 | Isoform 2 of Obg-like ATPase 1 | IPI00216105 | -0.205 | 0.34898 | 5 | 7 | 7 | 4 |
| 1374 | V-type proton ATPase subunit D | IPI00001568 | -0.205 | 0.34898 | 6 | 6 | 5 | 6 |
| 1375 | Isoform 2 of Mediator of DNA damage checkpoint protein 1 | IPI00470805 | -0.213 | 0.34481 | 7 | 4 | 5 | 5 |
| 1376 | Phosphoribosyl pyrophosphate synthase-associated protein 2 | IPI00003168 | -0.213 | 0.34481 | 5 | 6 | 4 | 6 |
| 1377 | Centromere/kinetochore protein zw10 homolog | IPI00011631 | -0.213 | 0.34481 | 5 | 6 | 5 | 5 |
| 1378 | 60S ribosomal protein L36 | IPI00216237 | -0.213 | 0.34481 | 5 | 6 | 6 | 4 |
| 1379 | Developmentally-regulated GTP-binding protein 2 | IPI00022697 | -0.213 | 0.34481 | 6 | 5 | 4 | 6 |
| 1380 | Mitochondrial import receptor subunit TOM34 | IPI00009946 | -0.213 | 0.34481 | 5 | 6 | 5 | 5 |
| 1381 | U3 small nucleolar RNA-associated protein 15 homolog | IPI00152708 | -0.213 | 0.34481 | 5 | 6 | 6 | 4 |
| 1382 | Isoform 1 of N-alpha-acetyltransferase 50, NatE catalytic subunit | IPI00018627 | -0.221 | 0.34144 | 5 | 5 | 6 | 3 |
| 1383 | cAMP-dependent protein kinase type I-alpha regulatory subunit | IPI00021831 | -0.221 | 0.34144 | 6 | 4 | 5 | 4 |
| 1384 | Isoform 1 of Protein phosphatase 1 regulatory subunit 7 | IPI00033600 | -0.221 | 0.34144 | 5 | 5 | 4 | 5 |
| 1385 | Isoform 2 of Myosin-VI | IPI00008455 | -0.221 | 0.34144 | 5 | 5 | 5 | 4 |
| 1386 | Isoform 2 of Isopentenyl-diphosphate Delta-isomerase 1 | IPI00220014 | -0.221 | 0.34144 | 6 | 4 | 5 | 4 |
| 1387 | MKI67 FHA domain-interacting nucleolar phosphoprotein | IPI00154590 | -0.221 | 0.34144 | 5 | 5 | 5 | 4 |
| 1388 | pyruvate dehydrogenase E1 alpha 1 isoform 2 precursor | IPI00306301 | -0.221 | 0.34144 | 4 | 6 | 5 | 4 |
| 1389 | cDNA FLJ56825, highly similar to WD repeat protein 57 | IPI00006723 | -0.221 | 0.34144 | 5 | 5 | 4 | 5 |
| 1390 | Translation initiation factor eIF-2B subunit epsilon | IPI00011898 | -0.221 | 0.34144 | 5 | 5 | 5 | 4 |
| 1391 | Paired amphipathic helix protein Sin3a | IPI00170596 | -0.221 | 0.34144 | 6 | 4 | 6 | 3 |
| 1392 | Isoform 2 of Serine/threonine-protein kinase PAK 3 | IPI00027382 | -0.221 | 0.34144 | 4 | 6 | 6 | 3 |
| 1393 | Glycylpeptide N-tetradecanoyltransferase 2 | IPI00030223 | -0.221 | 0.34144 | 5 | 5 | 3 | 6 |
| 1394 | Deoxyribonucleoside 5'-monophosphate N-glycosidase | IPI00007926 | -0.221 | 0.34144 | 5 | 5 | 5 | 4 |
| 1395 | Fructose-bisphosphate aldolase | IPI00418262 | -0.221 | 0.34144 | 5 | 5 | 5 | 4 |
| 1396 | Ras suppressor protein 1 | IPI00017256 | -0.231 | 0.33488 | 3 | 6 | 2 | 6 |
| 1397 | Catalase | IPI00465436 | -0.231 | 0.33488 | 5 | 4 | 6 | 2 |
| 1398 | Isoform 1 of Fanconi anemia group D2 protein | IPI00075081 | -0.231 | 0.33488 | 5 | 4 | 4 | 4 |
| 1399 | SWI/SNF related, matrix associated, actin dependent regulator of chromatin | IPI00216046 | -0.231 | 0.33488 | 5 | 4 | 5 | 3 |
| 1400 | Translocation protein SEC63 homolog | IPI00218922 | -0.231 | 0.33488 | 4 | 5 | 4 | 4 |
| 1401 | Microsomal glutathione S-transferase 1 | IPI00021805 | -0.231 | 0.33488 | 5 | 4 | 5 | 3 |
| 1402 | Importin subunit alpha-1 | IPI00303292 | -0.231 | 0.33488 | 3 | 6 | 5 | 3 |
| 1403 | Succinyl-CoA ligase [GDP-forming] subunit beta, mitochondrial | IPI00096066 | -0.231 | 0.33488 | 4 | 5 | 5 | 3 |
| 1404 | Isoform 2 of U4/U6 small nuclear ribonucleoprotein Prp31 | IPI00167198 | -0.231 | 0.33488 | 6 | 3 | 4 | 4 |
| 1405 | WD repeat-containing protein 82 | IPI00152695 | -0.231 | 0.33488 | 5 | 4 | 5 | 3 |
| 1406 | 60S ribosomal protein L19 | IPI00025329 | -0.231 | 0.33488 | 5 | 4 | 4 | 4 |
| 1407 | cDNA FLJ55599, highly similar to DNA replication licensing factor MCM3 | IPI00013214 | -0.236 | 0.33071 | 24 | 23 | 27 | 18 |
| 1408 | Isoform 1 of Nucleoside diphosphate kinase A | IPI00012048 | -0.241 | 0.33037 | 23 | 22 | 20 | 23 |
| 1409 | SUMO-activating enzyme subunit 2 | IPI00023234 | -0.243 | 0.33037 | 22 | 22 | 25 | 17 |
| 1410 | DNA ligase 1 | IPI00219841 | -0.243 | 0.33014 | 2 | 6 | 5 | 2 |
| 1411 | Deoxyhypusine hydroxylase | IPI00171856 | -0.243 | 0.33014 | 4 | 4 | 5 | 2 |
| 1412 | Cytochrome c oxidase subunit 5A, mitochondrial | IPI00025086 | -0.243 | 0.33014 | 5 | 3 | 3 | 4 |
| 1413 | Isoform 1 of Pentatricopeptide repeat-containing protein 3, mitochondrial | IPI00783302 | -0.243 | 0.33014 | 3 | 5 | 3 | 4 |
| 1414 | Hexokinase-2 | IPI00102864 | -0.243 | 0.33014 | 4 | 4 | 4 | 3 |
| 1415 | NEDD8-conjugating enzyme Ubc12 | IPI00022597 | -0.243 | 0.33014 | 4 | 4 | 4 | 3 |
| 1416 | Isoform 1 of Serine/threonine-protein phosphatase 6 catalytic subunit | IPI00012970 | -0.243 | 0.33014 | 5 | 3 | 4 | 3 |
| 1417 | Twinfilin-2 | IPI00550917 | -0.243 | 0.33014 | 4 | 4 | 4 | 3 |
| 1418 | DEAH (Asp-Glu-Ala-His) box polypeptide 16 | IPI00292510 | -0.243 | 0.33014 | 4 | 4 | 4 | 3 |
| 1419 | cDNA FLJ61629, highly similar to Clathrin interactor 1 | IPI00291930 | -0.243 | 0.33014 | 5 | 3 | 4 | 3 |
| 1420 | Ras-related protein Rab-35 | IPI00300096 | -0.243 | 0.33014 | 6 | 2 | 4 | 3 |
| 1421 | Signal peptidase complex subunit 3 | IPI00300299 | -0.243 | 0.33014 | 5 | 3 | 4 | 3 |

| No. | Description | Accession number | STN[1] | p-Value[1] | Con_A[2] | Con_B[2] | LUTEO_A[2] | LUTEO_B[2] |
|---|---|---|---|---|---|---|---|---|
| 1422 | Isoform A of DnaJ homolog subfamily B member 6 | IPI00024523 | -0.243 | 0.33014 | 4 | 4 | 3 | 4 |
| 1423 | cDNA FLJ56402, highly similar to Tripeptidyl-peptidase 1 | IPI00298237 | -0.243 | 0.33014 | 5 | 3 | 4 | 3 |
| 1424 | Plastin-3 | IPI00216694 | -0.247 | 0.32216 | 21 | 21 | 20 | 20 |
| 1425 | Probable ATP-dependent RNA helicase DDX6 | IPI00030320 | -0.250 | 0.32116 | 20 | 21 | 20 | 19 |
| 1426 | Mitochondrial import inner membrane translocase subunit TIM44 | IPI00306516 | -0.257 | 0.32055 | 3 | 4 | 3 | 3 |
| 1427 | Probable rRNA-processing protein EBP2 | IPI00745955 | -0.257 | 0.32055 | 5 | 2 | 3 | 3 |
| 1428 | Isoform 1 of OCIA domain-containing protein 1 | IPI00016405 | -0.257 | 0.32055 | 4 | 3 | 3 | 3 |
| 1429 | Isoform 1 of Methyl-CpG-binding domain protein 3 | IPI00439194 | -0.257 | 0.32055 | 4 | 3 | 3 | 3 |
| 1430 | Cell division protein kinase 7 | IPI00000685 | -0.257 | 0.32055 | 3 | 4 | 3 | 3 |
| 1431 | Cytochrome c-type heme lyase | IPI00023406 | -0.257 | 0.32055 | 5 | 2 | 5 | 1 |
| 1432 | Isoform 1 of Transmembrane protein 111 | IPI00020472 | -0.257 | 0.32055 | 4 | 3 | 4 | 2 |
| 1433 | Isoform 1 of 3'(2'),5'-bisphosphate nucleotidase 1 | IPI00410214 | -0.257 | 0.32055 | 3 | 4 | 3 | 3 |
| 1434 | Polymerase delta-interacting protein 2 | IPI00165506 | -0.257 | 0.32055 | 4 | 3 | 3 | 3 |
| 1435 | ATP synthase mitochondrial F1 complex assembly factor 2 | IPI00296999 | -0.257 | 0.32055 | 4 | 3 | 3 | 3 |
| 1436 | V-type proton ATPase subunit F | IPI00004488 | -0.257 | 0.32055 | 4 | 3 | 2 | 4 |
| 1437 | Exosome complex exonuclease RRP42 | IPI00014198 | -0.257 | 0.32055 | 4 | 3 | 5 | 1 |
| 1438 | Isoform 1 of DNA-directed RNA polymerases I and III subunit RPAC1 | IPI00005179 | -0.257 | 0.32055 | 2 | 5 | 4 | 2 |
| 1439 | 39S ribosomal protein L20, mitochondrial | IPI00013706 | -0.257 | 0.32055 | 4 | 3 | 4 | 2 |
| 1440 | Similar to nonhistone chromosomal protein HMG-1 | IPI00418184 | -0.257 | 0.32055 | 4 | 3 | 3 | 3 |
| 1441 | Isoform Beta of Lamina-associated polypeptide 2, isoforms beta/gamma | IPI00030131 | -0.258 | 0.31580 | 19 | 19 | 21 | 15 |
| 1442 | Cleavage and polyadenylation specificity factor subunit 5 | IPI00646917 | -0.264 | 0.31513 | 18 | 18 | 16 | 18 |
| 1443 | Isoform 1 of Cysteine and histidine-rich domain-containing protein 1 | IPI00015897 | -0.275 | 0.31243 | 3 | 3 | 1 | 4 |
| 1444 | Bifunctional methylenetetrahydrofolate dehydrogenase/cyclohydrolase, mitochondrial | IPI00011307 | -0.275 | 0.31243 | 2 | 4 | 2 | 3 |
| 1445 | cDNA FLJ54775, highly similar to Syntaxin-binding protein 2 | IPI00019971 | -0.275 | 0.31243 | 5 | 1 | 3 | 2 |
| 1446 | Tubulin gamma-1 chain | IPI00295081 | -0.275 | 0.31243 | 3 | 3 | 2 | 3 |
| 1447 | Isoform 3 of Transcription elongation factor SPT6 | IPI00456683 | -0.275 | 0.31243 | 1 | 5 | 2 | 3 |
| 1448 | Calcium-regulated heat stable protein 1 | IPI00304409 | -0.275 | 0.31243 | 3 | 3 | 2 | 3 |
| 1449 | F-actin-capping protein subunit alpha-2 | IPI00026182 | -0.275 | 0.31243 | 2 | 4 | 3 | 2 |
| 1450 | BAG family molecular chaperone regulator 2 | IPI00000643 | -0.275 | 0.31243 | 4 | 2 | 2 | 3 |
| 1451 | Isoform 1 of E3 ubiquitin-protein ligase BRE1B | IPI00162563 | -0.275 | 0.31243 | 5 | 1 | 1 | 4 |
| 1452 | Isoform 3 of Serine/threonine-protein phosphatase 2A activator | IPI00217296 | -0.275 | 0.31243 | 4 | 2 | 3 | 2 |
| 1453 | von Hippel-Lindau binding protein 1, isoform CRA_b | IPI00334159 | -0.275 | 0.31243 | 2 | 4 | 2 | 3 |
| 1454 | Isoform 1 of COP9 signalosome complex subunit 7b | IPI00009301 | -0.275 | 0.31243 | 2 | 4 | 4 | 1 |
| 1455 | Isoform 2 of Ubiquitin carboxyl-terminal hydrolase 47 | IPI00165528 | -0.275 | 0.31243 | 3 | 3 | 3 | 2 |
| 1456 | Putative uncharacterized protein EIF4E2 | IPI00556081 | -0.275 | 0.31243 | 5 | 1 | 4 | 1 |
| 1457 | tRNA (guanine-N(7)-)-methyltransferase | IPI00290184 | -0.275 | 0.31243 | 2 | 4 | 3 | 2 |
| 1458 | G patch domain and KOW motifs-containing protein | IPI00024255 | -0.275 | 0.31243 | 2 | 4 | 2 | 3 |
| 1459 | Nucleoporin 54kDa variant (Fragment) | IPI00172580 | -0.275 | 0.31243 | 3 | 3 | 2 | 3 |
| 1460 | U6 snRNA-associated Sm-like protein LSm4 | IPI00294955 | -0.275 | 0.31243 | 4 | 2 | 3 | 2 |
| 1461 | Isoform 1 of RNA-binding protein with serine-rich domain 1 | IPI00033561 | -0.275 | 0.31243 | 4 | 2 | 3 | 2 |
| 1462 | Transcriptional activator protein Pur-alpha | IPI00023591 | -0.275 | 0.31243 | 2 | 4 | 3 | 2 |
| 1463 | Nucleoporin Nup43 | IPI00742943 | -0.275 | 0.31243 | 2 | 4 | 3 | 2 |
| 1464 | Heterogeneous nuclear ribonucleoprotein U-like protein 2 | IPI00456887 | -0.280 | 0.29776 | 16 | 15 | 17 | 12 |
| 1465 | 60S ribosomal protein L7 | IPI00030179 | -0.292 | 0.29548 | 14 | 14 | 15 | 11 |
| 1466 | Leukocyte elastase inhibitor | IPI00027444 | -0.292 | 0.29548 | 15 | 13 | 15 | 11 |
| 1467 | Isochorismatase domain-containing protein 1 | IPI00304082 | -0.297 | 0.29353 | 13 | 14 | 14 | 11 |
| 1468 | Isoform 1 of Cell division cycle protein 23 homolog | IPI00005822 | -0.298 | 0.29353 | 3 | 2 | 3 | 1 |
| 1469 | NADH dehydrogenase [ubiquinone] 1 alpha subcomplex subunit 8 | IPI00219034 | -0.298 | 0.29353 | 2 | 3 | 2 | 2 |
| 1470 | Isoform 3 of Tyrosine-protein phosphatase non-receptor type 6 | IPI00183046 | -0.298 | 0.29353 | 2 | 3 | 1 | 3 |
| 1471 | Proteasome subunit beta type-6 | IPI00000811 | -0.298 | 0.29353 | 3 | 2 | 2 | 2 |
| 1472 | 2,4-dienoyl-CoA reductase, mitochondrial | IPI00003482 | -0.298 | 0.29353 | 3 | 2 | 1 | 3 |
| 1473 | Isoform 1 of Proteasome assembly chaperone 1 | IPI00030770 | -0.298 | 0.29353 | 3 | 2 | 1 | 3 |
| 1474 | Osteoclast-stimulating factor 1 | IPI00414836 | -0.298 | 0.29353 | 3 | 2 | 3 | 1 |
| 1475 | Ubiquitin-conjugating enzyme E2 G1 | IPI00219783 | -0.298 | 0.29353 | 3 | 2 | 2 | 2 |
| 1476 | Isoform 1 of Serine/threonine-protein kinase 4 | IPI00011488 | -0.298 | 0.29353 | 3 | 2 | 2 | 2 |
| 1477 | AP-3 complex subunit sigma-1 | IPI00014624 | -0.298 | 0.29353 | 3 | 2 | 2 | 2 |
| 1478 | Isoform 3 of Guanine nucleotide exchange factor VAV2 | IPI00004977 | -0.298 | 0.29353 | 2 | 3 | 2 | 2 |
| 1479 | NADH dehydrogenase [ubiquinone] 1 beta subcomplex subunit 5, mitochondrial | IPI00013459 | -0.298 | 0.29353 | 3 | 2 | 2 | 2 |
| 1480 | ubiquitin-like protein fubi and ribosomal protein S30 precursor | IPI00019770 | -0.298 | 0.29353 | 3 | 2 | 3 | 1 |
| 1481 | 39S ribosomal protein L15, mitochondrial | IPI00023086 | -0.298 | 0.29353 | 2 | 3 | 3 | 1 |
| 1482 | Isoform Long of Deoxyhypusine synthase | IPI00026829 | -0.298 | 0.29353 | 2 | 3 | 2 | 2 |
| 1483 | D-tyrosyl-tRNA(Tyr) deacylase 1 | IPI00152692 | -0.298 | 0.29353 | 3 | 2 | 2 | 2 |
| 1484 | Isoform Alpha of E3 ubiquitin-protein ligase TRIM33 | IPI00010252 | -0.298 | 0.29353 | 3 | 2 | 2 | 2 |
| 1485 | Na(+)/H(+) exchange regulatory cofactor NHE-RF1 | IPI00003527 | -0.298 | 0.29353 | 4 | 1 | 3 | 1 |
| 1486 | Isoform 1 of AP-1 complex subunit sigma-1A | IPI00152898 | -0.298 | 0.29353 | 2 | 3 | 3 | 1 |
| 1487 | Histone H1x | IPI00021924 | -0.298 | 0.29353 | 2 | 3 | 2 | 2 |
| 1488 | Isoform 2 of Oxidoreductase HTATIP2 | IPI00383665 | -0.298 | 0.29353 | 3 | 2 | 3 | 0 |
| 1489 | WD repeat-containing protein 46 | IPI00023126 | -0.298 | 0.29353 | 3 | 2 | 3 | 0 |
| 1490 | V-type proton ATPase subunit G 1 | IPI00025285 | -0.298 | 0.29353 | 2 | 3 | 3 | 1 |
| 1491 | Isoform 3 of F-box only protein 22 | IPI00169168 | -0.298 | 0.29353 | 2 | 3 | 2 | 2 |
| 1492 | Vesicle-fusing ATPase | IPI00006451 | -0.301 | 0.28575 | 9 | 17 | 14 | 10 |
| 1493 | Protein RRP5 homolog | IPI00400922 | -0.301 | 0.28575 | 11 | 15 | 12 | 12 |
| 1494 | Isoform 1 of Cleavage and polyadenylation specificity factor subunit 7 | IPI00550821 | -0.301 | 0.28575 | 13 | 13 | 14 | 10 |
| 1495 | Isoform 3 of Core histone macro-H2A.1 | IPI00059366 | -0.308 | 0.28323 | 31 | 36 | 27 | 37 |
| 1496 | cDNA FLJ77422, highly similar to Homo sapiens RNA binding protein | IPI00011268 | -0.311 | 0.28318 | 14 | 10 | 11 | 11 |
| 1497 | Ras-related protein Rab-14 | IPI00291928 | -0.311 | 0.28318 | 13 | 11 | 11 | 11 |
| 1498 | Isoform 2 of Splicing factor 3B subunit 3 | IPI00179138 | -0.317 | 0.28015 | 13 | 10 | 10 | 11 |
| 1499 | Eukaryotic translation initiation factor 5 | IPI00022648 | -0.317 | 0.28015 | 13 | 10 | 13 | 8 |
| 1500 | U4/U6.U5 tri-snRNP-associated protein 2 | IPI00419844 | -0.323 | 0.27972 | 11 | 11 | 10 | 10 |
| 1501 | GDP-mannose 4,6 dehydratase | IPI00030207 | -0.323 | 0.27972 | 13 | 9 | 10 | 10 |
| 1502 | Alpha-soluble NSF attachment protein | IPI00009253 | -0.323 | 0.27972 | 11 | 11 | 10 | 10 |
| 1503 | Glucosamine--fructose-6-phosphate aminotransferase [isomerizing] 2 | IPI00216159 | -0.329 | 0.27640 | 12 | 9 | 10 | 9 |
| 1504 | Histone H2A.V | IPI00018278 | -0.330 | 0.27640 | 62 | 54 | 65 | 47 |
| 1505 | Aldose reductase | IPI00413641 | -0.330 | 0.27625 | 3 | 1 | 2 | 0 |
| 1506 | FAST kinase domain-containing protein 5 | IPI00414973 | -0.330 | 0.27625 | 2 | 2 | 1 | 2 |
| 1507 | Isoform 1 of Adipocyte plasma membrane-associated protein | IPI00031131 | -0.330 | 0.27625 | 3 | 1 | 0 | 2 |
| 1508 | UDP-glucose:glycoprotein glucosyltransferase 2 | IPI00024467 | -0.330 | 0.27625 | 3 | 1 | 1 | 2 |
| 1509 | Phosphoglucomutase-2 | IPI00550364 | -0.330 | 0.27625 | 2 | 2 | 2 | 0 |
| 1510 | Isoform 3 of Mediator of RNA polymerase II transcription subunit 23 | IPI00413272 | -0.330 | 0.27625 | 2 | 2 | 2 | 1 |
| 1511 | Probable O-sialoglycoprotein endopeptidase | IPI00015809 | -0.330 | 0.27625 | 2 | 2 | 1 | 2 |
| 1512 | Echinoderm microtubule-associated protein-like 4 | IPI00001466 | -0.330 | 0.27625 | 2 | 2 | 2 | 1 |
| 1513 | Histone acetyltransferase type B catalytic subunit | IPI00024719 | -0.330 | 0.27625 | 2 | 2 | 1 | 2 |
| 1514 | Alcohol dehydrogenase class-3 | IPI00746777 | -0.330 | 0.27625 | 2 | 2 | 0 | 2 |
| 1515 | Ethanolamine-phosphate cytidylyltransferase | IPI00015285 | -0.330 | 0.27625 | 1 | 3 | 2 | 0 |
| 1516 | Eukaryotic translation initiation factor 2 subunit 2 | IPI00021728 | -0.330 | 0.27625 | 1 | 3 | 1 | 2 |

| No. | Description | Accession number | STN[1] | p-Value[1] | Con_A[2] | Con_B[2] | LUTEO_A[2] | LUTEO_B[2] |
|---|---|---|---|---|---|---|---|---|
| 1517 | Isoform 1 of Protein timeless homolog | IPI00335541 | -0.330 | 0.27625 | 2 | 2 | 1 | 2 |
| 1518 | EPS8L2 protein | IPI00414315 | -0.330 | 0.27625 | 3 | 1 | 2 | 0 |
| 1519 | SPRY domain-containing protein 4 | IPI00291643 | -0.330 | 0.27625 | 2 | 2 | 2 | 1 |
| 1520 | Beta-hexosaminidase subunit beta | IPI00012585 | -0.330 | 0.27625 | 2 | 2 | 2 | 1 |
| 1521 | cDNA FLJ55543, highly similar to Phosphoacetylglucosamine mutase | IPI00030116 | -0.330 | 0.27625 | 2 | 2 | 2 | 1 |
| 1522 | Isoform 2 of 39S ribosomal protein L39, mitochondrial | IPI00084571 | -0.330 | 0.27625 | 2 | 2 | 2 | 1 |
| 1523 | Transmembrane protein 214 | IPI00477118 | -0.330 | 0.27625 | 3 | 0 | 2 | 1 |
| 1524 | Isoform 4 of E3 ubiquitin-protein ligase UBR2 | IPI00217407 | -0.330 | 0.27625 | 2 | 2 | 0 | 2 |
| 1525 | Isoform 1 of N-acylneuraminate cytidylyltransferase | IPI00303158 | -0.330 | 0.27625 | 2 | 2 | 2 | 1 |
| 1526 | Isoform 1 of GTP-binding protein 10 | IPI00167638 | -0.330 | 0.27625 | 2 | 2 | 2 | 1 |
| 1527 | Farnesyl pyrophosphate synthetase like-4 protein (Fragment) | IPI00382869 | -0.330 | 0.27625 | 2 | 2 | 1 | 2 |
| 1528 | Eukaryotic translation initiation factor 1 | IPI00015077 | -0.330 | 0.27625 | 2 | 2 | 1 | 2 |
| 1529 | twinfilin-1 | IPI00183508 | -0.330 | 0.27625 | 2 | 2 | 1 | 2 |
| 1530 | RNA-binding protein 27 | IPI00292975 | -0.330 | 0.27625 | 2 | 2 | 1 | 2 |
| 1531 | CDGSH iron sulfur domain-containing protein 2 | IPI00166865 | -0.330 | 0.27625 | 2 | 2 | 2 | 1 |
| 1532 | 60S ribosomal protein L34 | IPI00219160 | -0.330 | 0.27625 | 3 | 1 | 2 | 1 |
| 1533 | Bleomycin hydrolase | IPI00219575 | -0.330 | 0.27625 | 2 | 2 | 2 | 1 |
| 1534 | Tubulin-folding cofactor B | IPI00293126 | -0.330 | 0.27625 | 2 | 2 | 1 | 2 |
| 1535 | Cytosolic Fe-S cluster assembly factor NUBP2 | IPI00644674 | -0.330 | 0.27625 | 2 | 2 | 1 | 2 |
| 1536 | SNF2 histone linker PHD RING helicase, isoform CRA_a | IPI00470627 | -0.330 | 0.27625 | 2 | 2 | 0 | 2 |
| 1537 | Isoform 1 of NADH dehydrogenase [ubiquinone] 1 alpha subcomplex subunit 11 | IPI00329301 | -0.330 | 0.27625 | 2 | 2 | 2 | 0 |
| 1538 | Methylmalonyl-CoA mutase, mitochondrial | IPI00024934 | -0.330 | 0.27625 | 2 | 2 | 2 | 0 |
| 1539 | Isoform 1 of HBS1-like protein | IPI00009070 | -0.330 | 0.27625 | 2 | 2 | 1 | 2 |
| 1540 | Isoform 1 of TRM1-like protein | IPI00334914 | -0.330 | 0.27625 | 2 | 2 | 1 | 2 |
| 1541 | D-beta-hydroxybutyrate dehydrogenase, mitochondrial | IPI00025341 | -0.330 | 0.27625 | 2 | 2 | 2 | 0 |
| 1542 | 119 kDa protein | IPI00297178 | -0.330 | 0.27625 | 2 | 2 | 2 | 1 |
| 1543 | Acyl-coenzyme A thioesterase 13 | IPI00020530 | -0.330 | 0.27625 | 2 | 2 | 1 | 2 |
| 1544 | U8 snoRNA-decapping enzyme | IPI00783497 | -0.330 | 0.27625 | 3 | 1 | 2 | 1 |
| 1545 | Isoform 2 of DNA-3-methyladenine glycosylase | IPI00218495 | -0.330 | 0.27625 | 2 | 2 | 0 | 2 |
| 1546 | Gamma-soluble NSF attachment protein | IPI00293817 | -0.330 | 0.27625 | 3 | 1 | 2 | 0 |
| 1547 | Putative uncharacterized protein DKFZp781K1356 | IPI00412545 | -0.330 | 0.27625 | 2 | 2 | 2 | 1 |
| 1548 | 37 kDa protein | IPI00032799 | -0.330 | 0.27625 | 2 | 2 | 1 | 2 |
| 1549 | UPF0428 protein CXorf56 | IPI00005055 | -0.330 | 0.27625 | 1 | 3 | 1 | 2 |
| 1550 | Isoform 1 of Syntaxin-7 | IPI00289876 | -0.330 | 0.27625 | 3 | 0 | 0 | 2 |
| 1551 | Isoform 1 of Phosphatidylinositol glycan anchor biosynthesis class U protein | IPI00026044 | -0.330 | 0.27625 | 2 | 2 | 2 | 1 |
| 1552 | WD repeat-containing protein 43 | IPI00937477 | -0.330 | 0.27625 | 0 | 3 | 0 | 2 |
| 1553 | Serpin H1 | IPI00032140 | -0.336 | 0.25779 | 11 | 9 | 11 | 7 |
| 1554 | cDNA FLJ60299, highly similar to Rab GDP dissociation inhibitor beta | IPI00031461 | -0.339 | 0.25413 | 24 | 29 | 27 | 23 |
| 1555 | Heterogeneous nuclear ribonucleoprotein C-like 1 | IPI00027569 | -0.342 | 0.25380 | 60 | 46 | 60 | 42 |
| 1556 | ATP-dependent RNA helicase DDX18 | IPI00301323 | -0.350 | 0.25342 | 23 | 26 | 25 | 21 |
| 1557 | Aladin | IPI00024143 | -0.351 | 0.25328 | 7 | 11 | 9 | 7 |
| 1558 | Isoform 2 of Microtubule-associated protein 4 | IPI00220113 | -0.360 | 0.24734 | 9 | 8 | 8 | 7 |
| 1559 | Thymidylate kinase | IPI00013862 | -0.360 | 0.24734 | 8 | 9 | 4 | 11 |
| 1560 | Cytosolic purine 5'-nucleotidase | IPI00029054 | -0.360 | 0.24734 | 8 | 9 | 7 | 8 |
| 1561 | Phosphoribosylformylglycinamidine synthase | IPI00004534 | -0.373 | 0.24131 | 21 | 21 | 23 | 16 |
| 1562 | Isoform 1 of Telomere-associated protein RIF1 | IPI00293845 | -0.378 | 0.24022 | 1 | 2 | 1 | 1 |
| 1563 | Golgi-specific brefeldin A-resistance guanine nucleotide exchange factor 1 | IPI00021954 | -0.378 | 0.24022 | 0 | 2 | 0 | 1 |
| 1564 | Isoform 1 of Uridine-cytidine kinase 2 | IPI00065671 | -0.378 | 0.24022 | 2 | 1 | 0 | 1 |
| 1565 | Isoform 1 of Protein dopey-2 | IPI00294653 | -0.378 | 0.24022 | 2 | 1 | 0 | 0 |
| 1566 | Isoform 1 of Ubiquitin conjugation factor E4 B | IPI00005715 | -0.378 | 0.24022 | 1 | 2 | 1 | 0 |
| 1567 | Isoform 2 of Pinin | IPI00002649 | -0.378 | 0.24022 | 1 | 2 | 1 | 0 |
| 1568 | Guanine nucleotide-binding protein-like 3-like protein | IPI00005132 | -0.378 | 0.24022 | 2 | 1 | 1 | 1 |
| 1569 | Putative uncharacterized protein ARAF | IPI00020578 | -0.378 | 0.24022 | 0 | 2 | 1 | 1 |
| 1570 | WD repeat and HMG-box DNA-binding protein 1 | IPI00411614 | -0.378 | 0.24022 | 1 | 2 | 0 | 0 |
| 1571 | NADH dehydrogenase [ubiquinone] 1 alpha subcomplex subunit 6 | IPI00419266 | -0.378 | 0.24022 | 2 | 1 | 0 | 1 |
| 1572 | Vacuolar protein sorting-associated protein 4A | IPI00411356 | -0.378 | 0.24022 | 2 | 1 | 0 | 1 |
| 1573 | Isoform 2 of Membrane magnesium transporter 1 | IPI00166785 | -0.378 | 0.24022 | 1 | 2 | 1 | 1 |
| 1574 | Protein LYRIC | IPI00328715 | -0.378 | 0.24022 | 2 | 1 | 1 | 0 |
| 1575 | Serine/threonine-protein phosphatase 1 regulatory subunit 10 | IPI00298731 | -0.378 | 0.24022 | 1 | 2 | 0 | 1 |
| 1576 | Isoform 1 of Acetoacetyl-CoA synthetase | IPI00217272 | -0.378 | 0.24022 | 1 | 2 | 0 | 1 |
| 1577 | Flotillin-2 | IPI00789008 | -0.378 | 0.24022 | 2 | 0 | 0 | 0 |
| 1578 | Histone deacetylase 4 | IPI00010088 | -0.378 | 0.24022 | 2 | 1 | 0 | 1 |
| 1579 | Chromosome-associated kinesin KIF4B | IPI00175193 | -0.378 | 0.24022 | 1 | 2 | 1 | 1 |
| 1580 | WD repeat domain 57 (U5 snRNP specific), isoform CRA_b | IPI00385642 | -0.378 | 0.24022 | 2 | 0 | 0 | 1 |
| 1581 | EF-hand domain-containing protein D1 | IPI00031091 | -0.378 | 0.24022 | 0 | 2 | 1 | 1 |
| 1582 | Succinate-semialdehyde dehydrogenase, mitochondrial | IPI00019888 | -0.378 | 0.24022 | 1 | 2 | 1 | 0 |
| 1583 | Deoxycytidine kinase | IPI00020454 | -0.378 | 0.24022 | 2 | 0 | 0 | 1 |
| 1584 | Adenylate kinase isoenzyme 6 | IPI00032879 | -0.378 | 0.24022 | 2 | 1 | 1 | 1 |
| 1585 | Isoform 1 of Polyadenylate-binding protein 2 | IPI00005792 | -0.378 | 0.24022 | 2 | 1 | 1 | 1 |
| 1586 | Protein TFG | IPI00294619 | -0.378 | 0.24022 | 2 | 0 | 1 | 0 |
| 1587 | cDNA FLJ78497 | IPI00289535 | -0.378 | 0.24022 | 1 | 2 | 1 | 1 |
| 1588 | Isoform 1 of Kinesin-like protein KIF1B | IPI00029011 | -0.378 | 0.24022 | 1 | 2 | 0 | 0 |
| 1589 | Isoform 1 of Anaphase-promoting complex subunit 5 | IPI00008247 | -0.378 | 0.24022 | 1 | 2 | 1 | 1 |
| 1590 | Ubiquitin carboxyl-terminal hydrolase 13 | IPI00024401 | -0.378 | 0.24022 | 1 | 2 | 1 | 0 |
| 1591 | Isoform 2 of Transcription factor p65 | IPI00219084 | -0.378 | 0.24022 | 1 | 2 | 1 | 1 |
| 1592 | Isoform 2 of Choline-phosphate cytidylyltransferase B | IPI00001562 | -0.378 | 0.24022 | 1 | 2 | 1 | 1 |
| 1593 | Tensin-1 | IPI00307545 | -0.378 | 0.24022 | 2 | 1 | 1 | 1 |
| 1594 | Isoform 1 of Ubiquinone biosynthesis protein COQ9, mitochondrial | IPI00470631 | -0.378 | 0.24022 | 1 | 2 | 1 | 0 |
| 1595 | ERBB2IP protein | IPI00438286 | -0.378 | 0.24022 | 2 | 1 | 0 | 0 |
| 1596 | Isoform 1 of Glucocorticoid receptor DNA-binding factor 1 | IPI00334715 | -0.378 | 0.24022 | 0 | 2 | 0 | 0 |
| 1597 | Isoform 1 of UPF0557 protein C10orf119 | IPI00478758 | -0.378 | 0.24022 | 2 | 1 | 1 | 1 |
| 1598 | Isoform 1 of Mixed lineage kinase domain-like protein | IPI00180781 | -0.378 | 0.24022 | 2 | 1 | 0 | 0 |
| 1599 | Transmembrane protein 41A | IPI00063334 | -0.378 | 0.24022 | 0 | 2 | 1 | 1 |
| 1600 | Ubiquitin-conjugating enzyme E2 T | IPI00023087 | -0.378 | 0.24022 | 2 | 1 | 0 | 1 |
| 1601 | Isoform Beta-1C of Integrin beta-1 | IPI00217561 | -0.378 | 0.24022 | 2 | 1 | 0 | 1 |
| 1602 | mesencephalic astrocyte-derived neurotrophic factor | IPI00328748 | -0.378 | 0.24022 | 2 | 0 | 1 | 1 |
| 1603 | Isoform 1 of Uncharacterized protein FLJ44048 | IPI00410549 | -0.378 | 0.24022 | 2 | 0 | 0 | 0 |
| 1604 | Isoform 3 of Protein LAS1 homolog | IPI00009917 | -0.378 | 0.24022 | 1 | 2 | 1 | 0 |
| 1605 | 28S ribosomal protein S9, mitochondrial | IPI00641924 | -0.378 | 0.24022 | 1 | 2 | 1 | 1 |
| 1606 | Isoform 2 of Uncharacterized protein C3orf63 | IPI00745978 | -0.378 | 0.24022 | 1 | 2 | 1 | 0 |
| 1607 | Isoform DFF45 of DNA fragmentation factor subunit alpha (Fragment) | IPI00010882 | -0.378 | 0.24022 | 1 | 2 | 1 | 1 |
| 1608 | KIF1-binding protein | IPI00477355 | -0.378 | 0.24022 | 2 | 1 | 1 | 1 |
| 1609 | TAF6-like RNA polymerase II p300/CBP-associated factor-associated factor 65 kDa subunit 6L | IPI00007957 | -0.378 | 0.24022 | 1 | 2 | 0 | 0 |
| 1610 | c-Maf-inducing protein isoform C-mip | IPI00028438 | -0.378 | 0.24022 | 1 | 2 | 0 | 1 |
| 1611 | Isoform 1 of Phosphatidylinositol 4-kinase alpha | IPI00070943 | -0.378 | 0.24022 | 1 | 2 | 1 | 0 |

| No. | Description | Accession number | STN[1] | p-Value[1] | Con_A[2] | Con_B[2] | LUTEO_A[2] | LUTEO_B[2] |
|---|---|---|---|---|---|---|---|---|
| 1612 | Isoform 1 of UPF0424 protein C1orf128 | IPI00015351 | -0.378 | 0.24022 | 1 | 2 | 1 | 0 |
| 1613 | Large subunit GTPase 1 homolog | IPI00300094 | -0.378 | 0.24022 | 1 | 2 | 1 | 0 |
| 1614 | U3 small nucleolar RNA-interacting protein 2 | IPI00217862 | -0.378 | 0.24022 | 1 | 2 | 1 | 1 |
| 1615 | Isoform 2 of VIP36-like protein | IPI00218337 | -0.378 | 0.24022 | 2 | 1 | 0 | 0 |
| 1616 | Isoform 1 of Endophilin-B1 | IPI00006558 | -0.378 | 0.24022 | 2 | 0 | 1 | 1 |
| 1617 | Isoform 1 of Liprin-alpha-1 | IPI00163496 | -0.378 | 0.24022 | 2 | 0 | 0 | 1 |
| 1618 | Isoform 1 of Regulatory-associated protein of mTOR | IPI00166044 | -0.378 | 0.24022 | 2 | 0 | 0 | 0 |
| 1619 | Glutamine-dependent NAD(+) synthetase | IPI00306689 | -0.378 | 0.24022 | 2 | 1 | 1 | 1 |
| 1620 | Neudesin | IPI00002525 | -0.378 | 0.24022 | 2 | 0 | 1 | 0 |
| 1621 | DnaJ homolog subfamily C member 17 | IPI00018798 | -0.378 | 0.24022 | 2 | 1 | 0 | 0 |
| 1622 | Isoform 1 of Upstream-binding protein 1 | IPI00005018 | -0.378 | 0.24022 | 1 | 2 | 1 | 1 |
| 1623 | Ubiquitin-associated protein 2 | IPI00171127 | -0.378 | 0.24022 | 0 | 2 | 1 | 1 |
| 1624 | Isoform 2 of Inositol hexakisphosphate and diphosphoinositol-pentakisphosphate kinase 2 | IPI00178375 | -0.378 | 0.24022 | 2 | 1 | 0 | 0 |
| 1625 | Cell division protein kinase 4 | IPI00007811 | -0.378 | 0.24022 | 0 | 2 | 0 | 1 |
| 1626 | Peroxisomal biogenesis factor 3 | IPI00010232 | -0.378 | 0.24022 | 1 | 2 | 1 | 1 |
| 1627 | Isoform 1 of Rab GTPase-activating protein 1 | IPI00016702 | -0.378 | 0.24022 | 1 | 2 | 1 | 1 |
| 1628 | Isoform 1 of DNA repair protein complementing XP-G cells | IPI00477535 | -0.378 | 0.24022 | 1 | 2 | 0 | 0 |
| 1629 | Syntaxin-4 | IPI00029730 | -0.378 | 0.24022 | 2 | 0 | 1 | 1 |
| 1630 | Isoform 1 of Centromere protein M | IPI00031566 | -0.378 | 0.24022 | 2 | 1 | 0 | 0 |
| 1631 | 28S ribosomal protein S30, mitochondrial | IPI00010278 | -0.378 | 0.24022 | 2 | 0 | 1 | 0 |
| 1632 | cDNA FLJ56280, highly similar to Endoplasmic reticulum-Golgi intermediate compartment protein 1 | IPI00003635 | -0.378 | 0.24022 | 0 | 2 | 0 | 0 |
| 1633 | Isoform 2 of Late secretory pathway protein AVL9 homolog | IPI00022042 | -0.378 | 0.24022 | 1 | 2 | 0 | 1 |
| 1634 | Isoform 1 of WD repeat-containing protein 44 | IPI00444371 | -0.378 | 0.24022 | 2 | 1 | 1 | 0 |
| 1635 | Uncharacterized protein C19orf21 | IPI00217121 | -0.378 | 0.24022 | 2 | 1 | 0 | 0 |
| 1636 | Poly(A)-specific ribonuclease PARN | IPI00294744 | -0.378 | 0.24022 | 2 | 1 | 1 | 1 |
| 1637 | Isoform 1 of Phytanoyl-CoA hydroxylase-interacting protein-like | IPI00289396 | -0.378 | 0.24022 | 2 | 1 | 0 | 1 |
| 1638 | Isoform 1 of Set1/Ash2 histone methyltransferase complex subunit ASH2 | IPI00328658 | -0.378 | 0.24022 | 1 | 2 | 1 | 1 |
| 1639 | Isoform 1 of Mps one binder kinase activator-like 1B | IPI00301518 | -0.378 | 0.24022 | 1 | 2 | 0 | 0 |
| 1640 | Isoform 1 of Beta-1-syntrophin | IPI00026059 | -0.378 | 0.24022 | 2 | 1 | 1 | 0 |
| 1641 | bifunctional protein NCOAT isoform b | IPI00181391 | -0.378 | 0.24022 | 1 | 2 | 0 | 0 |
| 1642 | Protein S100-A16 | IPI00062120 | -0.378 | 0.24022 | 1 | 2 | 1 | 1 |
| 1643 | Isoform 1 of Kinesin-like protein KIF15 | IPI00024975 | -0.378 | 0.24022 | 2 | 0 | 1 | 0 |
| 1644 | Histone acetyltransferase p300 | IPI00020985 | -0.378 | 0.24022 | 0 | 2 | 0 | 0 |
| 1645 | BTB/POZ domain-containing protein KCTD14 | IPI00181836 | -0.378 | 0.24022 | 2 | 1 | 1 | 1 |
| 1646 | Ras-related protein Rab-22A | IPI00007756 | -0.378 | 0.24022 | 2 | 1 | 1 | 1 |
| 1647 | cDNA FLJ56469, highly similar to Propionyl-CoA carboxylase alpha chain, mitochondrial | IPI00552419 | -0.378 | 0.24022 | 0 | 2 | 1 | 0 |
| 1648 | NICE-4 protein (Fragment) | IPI00005416 | -0.378 | 0.24022 | 1 | 2 | 1 | 1 |
| 1649 | cDNA FLJ11251 fis, clone PLACE1008813 | IPI00010953 | -0.378 | 0.24022 | 0 | 2 | 1 | 1 |
| 1650 | Caspase-3 | IPI00292140 | -0.378 | 0.24022 | 0 | 2 | 0 | 0 |
| 1651 | Isoform 1 of Multivesicular body subunit 12A | IPI00744702 | -0.378 | 0.24022 | 0 | 2 | 0 | 1 |
| 1652 | cDNA FLJ61655, highly similar to Phosphorylated CTD-interacting factor 1 | IPI00014865 | -0.378 | 0.24022 | 2 | 1 | 1 | 0 |
| 1653 | RAC-alpha serine/threonine-protein kinase | IPI00012866 | -0.378 | 0.24022 | 2 | 1 | 0 | 0 |
| 1654 | Gamma-taxilin | IPI00019994 | -0.378 | 0.24022 | 2 | 1 | 0 | 0 |
| 1655 | Isoform 1 of Elongator complex protein 3 | IPI00165477 | -0.378 | 0.24022 | 1 | 2 | 0 | 0 |
| 1656 | ribonucleotide reductase M2 polypeptide isoform 1 | IPI00011118 | -0.378 | 0.24022 | 2 | 1 | 0 | 0 |
| 1657 | La-related protein 7 | IPI00294742 | -0.378 | 0.24022 | 2 | 1 | 0 | 0 |
| 1658 | HLA class I histocompatibility antigen, B-7 alpha chain | IPI00004657 | -0.378 | 0.24022 | 2 | 0 | 1 | 1 |
| 1659 | Isoform 1 of Polyadenylate-binding protein-interacting protein 1 | IPI00021466 | -0.378 | 0.24022 | 0 | 2 | 0 | 1 |
| 1660 | ADP-ribosylation factor-like protein 8A | IPI00060031 | -0.378 | 0.24022 | 2 | 1 | 1 | 0 |
| 1661 | Isoform 2 of Myosin-XVIIIa | IPI00334410 | -0.378 | 0.24022 | 0 | 2 | 0 | 0 |
| 1662 | Isoform 1 of Quinone oxidoreductase PIG3 | IPI00384643 | -0.378 | 0.24022 | 1 | 2 | 1 | 1 |
| 1663 | Isoform 2 of Uncharacterized protein C3orf21 | IPI00165665 | -0.378 | 0.24022 | 1 | 2 | 1 | 1 |
| 1664 | Isoform 2 of Septin-11 | IPI00019376 | -0.378 | 0.24022 | 0 | 2 | 0 | 1 |
| 1665 | YrdC domain-containing protein, mitochondrial | IPI00384180 | -0.378 | 0.24022 | 0 | 2 | 0 | 1 |
| 1666 | poly [ADP-ribose] polymerase 14 | IPI00291215 | -0.378 | 0.24022 | 0 | 2 | 0 | 1 |
| 1667 | RAB4A, member RAS oncogene family variant | IPI00480056 | -0.378 | 0.24022 | 2 | 1 | 1 | 1 |
| 1668 | Isoform B of Syntaxin-16 | IPI00023149 | -0.378 | 0.24022 | 2 | 0 | 0 | 0 |
| 1669 | DNA-directed RNA polymerase II subunit RPB4 | IPI00007283 | -0.378 | 0.24022 | 1 | 2 | 1 | 0 |
| 1670 | Conserved hypothetical protein | IPI00477526 | -0.378 | 0.24022 | 2 | 1 | 0 | 0 |
| 1671 | 39S ribosomal protein L32, mitochondrial precursor | IPI00011077 | -0.378 | 0.24022 | 2 | 0 | 1 | 0 |
| 1672 | 28 kDa heat- and acid-stable phosphoprotein | IPI00013297 | -0.378 | 0.24022 | 2 | 1 | 1 | 0 |
| 1673 | Sulfide:quinone oxidoreductase, mitochondrial | IPI00009634 | -0.378 | 0.24022 | 2 | 1 | 0 | 0 |
| 1674 | Isoform 1 of F-box/LRR-repeat protein 12 | IPI00015011 | -0.378 | 0.24022 | 2 | 0 | 0 | 0 |
| 1675 | Isoform 2 of Membrane-associated guanylate kinase, WW and PDZ domain-containing protein 1 | IPI00165946 | -0.378 | 0.24022 | 1 | 2 | 0 | 1 |
| 1676 | Isoform 1 of Choline kinase alpha | IPI00409761 | -0.378 | 0.24022 | 2 | 1 | 0 | 0 |
| 1677 | Tetratricopeptide repeat protein 27 | IPI00183938 | -0.378 | 0.24022 | 1 | 2 | 1 | 0 |
| 1678 | cDNA FLJ61386, highly similar to Homo sapiens mitochondrial ribosomal protein L43 (MRPL43) | IPI00334579 | -0.378 | 0.24022 | 0 | 2 | 0 | 0 |
| 1679 | Isoform Delta 6 of Calcium/calmodulin-dependent protein kinase type II subunit delta | IPI00172636 | -0.378 | 0.24022 | 2 | 0 | 1 | 1 |
| 1680 | Biotin--protein ligase | IPI00301907 | -0.378 | 0.24022 | 0 | 2 | 0 | 0 |
| 1681 | Proteasome assembly chaperone 4 | IPI00895892 | -0.378 | 0.24022 | 1 | 2 | 0 | 0 |
| 1682 | Isoform 1 of SWI/SNF-related matrix-associated actin-dependent regulator of chromatin | IPI00017669 | -0.378 | 0.24022 | 1 | 2 | 1 | 0 |
| 1683 | Switch-associated protein 70 | IPI00307200 | -0.378 | 0.24022 | 2 | 0 | 0 | 0 |
| 1684 | Ankyrin repeat and SAM domain-containing protein 1A | IPI00395663 | -0.378 | 0.24022 | 0 | 2 | 0 | 0 |
| 1685 | Squalene synthase | IPI00020944 | -0.378 | 0.24022 | 2 | 1 | 0 | 0 |
| 1686 | Isoform 1 of Signal recognition particle 68 kDa protein | IPI00168388 | -0.378 | 0.24022 | 0 | 2 | 0 | 1 |
| 1687 | Ferritin heavy chain | IPI00554521 | -0.378 | 0.24022 | 0 | 2 | 0 | 0 |
| 1688 | COP9 signalosome complex subunit 8 | IPI00009480 | -0.378 | 0.24022 | 2 | 0 | 1 | 0 |
| 1689 | Formin-binding protein 4 | IPI00170778 | -0.378 | 0.24022 | 0 | 2 | 1 | 0 |
| 1690 | Protein QIL1 | IPI00329373 | -0.378 | 0.24022 | 1 | 2 | 0 | 0 |
| 1691 | Uncharacterized protein C20orf29 | IPI00019941 | -0.378 | 0.24022 | 1 | 2 | 0 | 1 |
| 1692 | 10 kDa heat shock protein, mitochondrial | IPI00220362 | -0.380 | 0.22707 | 6 | 9 | 6 | 7 |
| 1693 | Isoform 1 of RNA-binding protein 14 | IPI00013174 | -0.380 | 0.22707 | 7 | 8 | 7 | 6 |
| 1694 | Isoform 2 of Extended synaptotagmin-2 | IPI00409635 | -0.380 | 0.22707 | 6 | 9 | 7 | 6 |
| 1695 | ATP-dependent Clp protease ATP-binding subunit clpX-like, mitochondrial | IPI00008728 | -0.380 | 0.22707 | 9 | 6 | 5 | 8 |
| 1696 | Isoform 1 of DNA replication licensing factor MCM7 | IPI00299904 | -0.385 | 0.22669 | 21 | 18 | 19 | 17 |
| 1697 | Ras-related protein Rab-11B | IPI00020436 | -0.385 | 0.22669 | 21 | 18 | 17 | 19 |
| 1698 | THO complex subunit 2 | IPI00158615 | -0.385 | 0.22669 | 18 | 21 | 18 | 18 |
| 1699 | 40S ribosomal protein S8 | IPI00216587 | -0.389 | 0.22598 | 17 | 21 | 20 | 15 |
| 1700 | Single-stranded DNA-binding protein, mitochondrial | IPI00029744 | -0.389 | 0.22598 | 20 | 18 | 20 | 15 |
| 1701 | Isoform 2 of Tyrosine-protein phosphatase non-receptor type 11 | IPI00298347 | -0.391 | 0.22569 | 7 | 7 | 7 | 5 |
| 1702 | Isoform 2 of cAMP-dependent protein kinase catalytic subunit alpha | IPI00217960 | -0.391 | 0.22569 | 8 | 6 | 7 | 5 |
| 1703 | 26S protease regulatory subunit 6A | IPI00018398 | -0.391 | 0.22569 | 9 | 5 | 7 | 5 |
| 1704 | Cold-inducible RNA-binding protein | IPI00180954 | -0.391 | 0.22569 | 7 | 7 | 6 | 6 |
| 1705 | Transgelin-2 | IPI00550363 | -0.391 | 0.22569 | 8 | 6 | 8 | 4 |

| No. | Description | Accession number | STN[1] | p-Value[1] | Con_A[2] | Con_B[2] | LUTEO_A[2] | LUTEO_B[2] |
|---|---|---|---|---|---|---|---|---|
| 1706 | ADP-ribosylation factor-like protein 2 | IPI00003326 | -0.391 | 0.22569 | 8 | 6 | 7 | 5 |
| 1707 | Rab GDP dissociation inhibitor alpha | IPI00010154 | -0.398 | 0.21881 | 18 | 18 | 18 | 15 |
| 1708 | Isoform 1 of Host cell factor 1 | IPI00019848 | -0.404 | 0.21791 | 5 | 8 | 6 | 5 |
| 1709 | Isoform 1 of Acidic leucine-rich nuclear phosphoprotein 32 family member B | IPI00007423 | -0.404 | 0.21791 | 6 | 7 | 5 | 6 |
| 1710 | Barrier-to-autointegration factor | IPI00026087 | -0.404 | 0.21791 | 5 | 8 | 5 | 6 |
| 1711 | Isoform 1 of Protein-tyrosine phosphatase mitochondrial 1 | IPI00174190 | -0.404 | 0.21791 | 6 | 7 | 4 | 7 |
| 1712 | Isoform 2 of Histone deacetylase 2 | IPI00289601 | -0.404 | 0.21791 | 5 | 8 | 6 | 5 |
| 1713 | Lamin-B1 | IPI00217975 | -0.405 | 0.21791 | 39 | 31 | 35 | 31 |
| 1714 | Small subunit processome component 20 homolog | IPI00004970 | -0.407 | 0.21781 | 15 | 19 | 13 | 18 |
| 1715 | Isoform 1 of Heterogeneous nuclear ribonucleoprotein H3 | IPI00013877 | -0.407 | 0.21781 | 18 | 16 | 15 | 16 |
| 1716 | Isoform 1 of Filamin-B | IPI00289334 | -0.412 | 0.21724 | 30 | 37 | 37 | 26 |
| 1717 | Vacuolar protein sorting-associated protein 35 | IPI00018931 | -0.417 | 0.21658 | 32 | 33 | 30 | 31 |
| 1718 | Isoform 3 of PCI domain-containing protein 2 | IPI00072541 | -0.418 | 0.21615 | 7 | 5 | 4 | 6 |
| 1719 | Isoform Short of Glycylpeptide N-tetradecanoyltransferase 1 | IPI00218830 | -0.418 | 0.21615 | 6 | 6 | 5 | 5 |
| 1720 | Putative uncharacterized protein | IPI00260769 | -0.418 | 0.21615 | 6 | 6 | 5 | 5 |
| 1721 | Thimet oligopeptidase | IPI00549189 | -0.418 | 0.21615 | 4 | 8 | 6 | 4 |
| 1722 | Cytoplasmic dynein 1 light intermediate chain 1 | IPI00007675 | -0.418 | 0.21615 | 5 | 7 | 5 | 5 |
| 1723 | Isoform A of Nucleoporin SEH1 | IPI00185533 | -0.418 | 0.21615 | 7 | 5 | 6 | 4 |
| 1724 | Isoform 3 of DNA repair protein RAD50 | IPI00107531 | -0.418 | 0.21615 | 6 | 6 | 6 | 4 |
| 1725 | Cytokine-like nuclear factor n-pac, isoform CRA_a | IPI00000155 | -0.418 | 0.21615 | 6 | 6 | 5 | 5 |
| 1726 | N-alpha-acetyltransferase 38, NatC auxiliary subunit | IPI00219871 | -0.418 | 0.21615 | 6 | 6 | 6 | 4 |
| 1727 | 39S ribosomal protein L49, mitochondrial | IPI00013195 | -0.418 | 0.21615 | 6 | 6 | 5 | 5 |
| 1728 | 40S ribosomal protein S15a | IPI00221091 | -0.423 | 0.20571 | 18 | 13 | 16 | 12 |
| 1729 | Peroxiredoxin-4 | IPI00011937 | -0.429 | 0.20528 | 17 | 13 | 14 | 13 |
| 1730 | PNAS-139 | IPI00000477 | -0.434 | 0.20485 | 6 | 5 | 4 | 5 |
| 1731 | Isoform 1 of Abhydrolase domain-containing protein 14B | IPI00063827 | -0.434 | 0.20485 | 5 | 6 | 3 | 6 |
| 1732 | Serine/threonine-protein phosphatase 5 | IPI00019812 | -0.434 | 0.20485 | 5 | 6 | 5 | 4 |
| 1733 | 28S ribosomal protein S10, mitochondrial | IPI00061245 | -0.434 | 0.20485 | 4 | 7 | 4 | 5 |
| 1734 | Putative ATP-dependent Clp protease proteolytic subunit, mitochondrial | IPI00003870 | -0.434 | 0.20485 | 7 | 4 | 5 | 4 |
| 1735 | tropomyosin alpha-1 chain isoform 2 | IPI00000230 | -0.434 | 0.20485 | 5 | 6 | 4 | 5 |
| 1736 | Isoform 1 of LETM1 and EF-hand domain-containing protein 1, mitochondrial | IPI00017592 | -0.435 | 0.20485 | 17 | 12 | 11 | 15 |
| 1737 | Actin-related protein 2 | IPI00005159 | -0.435 | 0.20485 | 14 | 15 | 15 | 11 |
| 1738 | Ribose-phosphate pyrophosphokinase 1 | IPI00219616 | -0.442 | 0.20438 | 14 | 14 | 14 | 11 |
| 1739 | Proteasome subunit beta type-4 | IPI00555956 | -0.442 | 0.20438 | 15 | 13 | 12 | 13 |
| 1740 | ATP-dependent RNA helicase DDX1 | IPI00293655 | -0.450 | 0.20324 | 26 | 28 | 27 | 23 |
| 1741 | Isoform 1 of ATP-dependent RNA helicase DDX42 | IPI00409671 | -0.453 | 0.20300 | 5 | 5 | 5 | 3 |
| 1742 | Isoform 1 of Ataxin-2-like protein | IPI00456359 | -0.453 | 0.20300 | 6 | 4 | 2 | 6 |
| 1743 | Cell division protein kinase 6 | IPI00023529 | -0.453 | 0.20300 | 5 | 5 | 5 | 3 |
| 1744 | Glutathione S-transferase omega-1 | IPI00019755 | -0.453 | 0.20300 | 5 | 5 | 5 | 3 |
| 1745 | Isoform 2C of Cytoplasmic dynein 1 intermediate chain 2 | IPI00216348 | -0.453 | 0.20300 | 4 | 6 | 5 | 3 |
| 1746 | Mitogen-activated protein kinase 1 | IPI00003479 | -0.453 | 0.20300 | 6 | 4 | 5 | 3 |
| 1747 | Isoform 1 of HEAT repeat-containing protein 2 | IPI00242630 | -0.453 | 0.20300 | 5 | 5 | 5 | 3 |
| 1748 | Isoform 1 of U4/U6 small nuclear ribonucleoprotein Prp4 | IPI00150269 | -0.453 | 0.20300 | 7 | 3 | 4 | 4 |
| 1749 | COP9 signalosome complex subunit 4 | IPI00171844 | -0.453 | 0.20300 | 5 | 5 | 6 | 2 |
| 1750 | Protein | IPI00892529 | -0.453 | 0.20300 | 6 | 4 | 4 | 4 |
| 1751 | 40S ribosomal protein S16 | IPI00221092 | -0.456 | 0.18999 | 13 | 13 | 14 | 9 |
| 1752 | Nucleosome assembly protein 1-like 1 | IPI00023860 | -0.461 | 0.18833 | 24 | 27 | 23 | 24 |
| 1753 | Heterogeneous nuclear ribonucleoprotein F | IPI00003881 | -0.461 | 0.18833 | 29 | 22 | 24 | 23 |
| 1754 | Isoform Short of Proteasome subunit alpha type-1 | IPI00016832 | -0.463 | 0.18824 | 14 | 11 | 11 | 11 |
| 1755 | Isoform 2 of U5 small nuclear ribonucleoprotein 200 kDa helicase | IPI00168235 | -0.469 | 0.18733 | 22 | 27 | 22 | 23 |
| 1756 | Thioredoxin | IPI00216298 | -0.471 | 0.18733 | 13 | 11 | 11 | 10 |
| 1757 | Eukaryotic translation initiation factor 6 | IPI00010105 | -0.471 | 0.18733 | 11 | 13 | 11 | 10 |
| 1758 | Isoform 1 of Clathrin heavy chain 1 | IPI00024067 | -0.472 | 0.18619 | 135 | 134 | 132 | 129 |
| 1759 | cDNA FLJ35809 fis, clone TESTI2006016, highly similar to Eukaryotic translation initiation factor 3 | IPI00647650 | -0.475 | 0.18524 | 5 | 4 | 4 | 3 |
| 1760 | Aminoacyl tRNA synthase complex-interacting multifunctional protein 2 | IPI00011916 | -0.475 | 0.18524 | 3 | 6 | 4 | 3 |
| 1761 | Histidine triad nucleotide-binding protein 1 | IPI00239077 | -0.475 | 0.18524 | 5 | 4 | 3 | 4 |
| 1762 | NADPH--cytochrome P450 reductase | IPI00470467 | -0.475 | 0.18524 | 4 | 5 | 5 | 2 |
| 1763 | Isoform 1 of Nicalin | IPI00470649 | -0.475 | 0.18524 | 5 | 4 | 3 | 4 |
| 1764 | Mitochondrial ribonuclease P protein 1 | IPI00099996 | -0.475 | 0.18524 | 4 | 5 | 4 | 3 |
| 1765 | Isoform F of Protein SON | IPI00000192 | -0.475 | 0.18524 | 6 | 3 | 2 | 5 |
| 1766 | AP-1 complex subunit mu-1 | IPI00032516 | -0.475 | 0.18524 | 3 | 6 | 3 | 4 |
| 1767 | cDNA FLJ56357, highly similar to Homo sapiens apolipoprotein A-I binding protein (APOA1BP), mRNA | IPI00168479 | -0.475 | 0.18524 | 5 | 4 | 4 | 3 |
| 1768 | TDP43 | IPI00025815 | -0.475 | 0.18524 | 5 | 4 | 3 | 4 |
| 1769 | Isoform Epsilon of Apoptosis regulator BAX | IPI00071059 | -0.475 | 0.18524 | 6 | 3 | 3 | 4 |
| 1770 | Probable fructose-2,6-bisphosphatase TIGAR | IPI00006907 | -0.475 | 0.18524 | 4 | 5 | 3 | 4 |
| 1771 | OTU domain-containing protein 6B | IPI00182180 | -0.475 | 0.18524 | 6 | 3 | 4 | 3 |
| 1772 | Guanine nucleotide-binding protein subunit alpha-13 | IPI00290928 | -0.475 | 0.18524 | 4 | 5 | 3 | 4 |
| 1773 | Isoform 1 of Filamin-C | IPI00178352 | -0.480 | 0.18506 | 10 | 13 | 8 | 12 |
| 1774 | Ubiquitin-conjugating enzyme E2 N | IPI00003949 | -0.480 | 0.18506 | 13 | 10 | 10 | 10 |
| 1775 | E3 SUMO-protein ligase RanBP2 | IPI00221325 | -0.480 | 0.18506 | 10 | 13 | 6 | 14 |
| 1776 | Spermidine synthase | IPI00292020 | -0.480 | 0.18506 | 14 | 9 | 13 | 7 |
| 1777 | Proteasome activator complex subunit 1 | IPI00479722 | -0.486 | 0.18401 | 21 | 24 | 23 | 18 |
| 1778 | U1 small nuclear ribonucleoprotein A | IPI00012382 | -0.489 | 0.18392 | 12 | 10 | 9 | 10 |
| 1779 | 87 kDa protein | IPI00220365 | -0.489 | 0.18392 | 10 | 12 | 12 | 7 |
| 1780 | annexin A4 | IPI00793199 | -0.490 | 0.18311 | 24 | 20 | 20 | 20 |
| 1781 | Isoform 2 of Transportin-3 | IPI00395694 | -0.499 | 0.18225 | 10 | 11 | 9 | 9 |
| 1782 | Isoform 1 of PC4 and SFRS1-interacting protein | IPI00028122 | -0.501 | 0.18130 | 3 | 5 | 3 | 3 |
| 1783 | Histone-binding protein RBBP7 | IPI00395865 | -0.501 | 0.18130 | 4 | 4 | 3 | 3 |
| 1784 | Protein dpy-30 homolog | IPI00028109 | -0.501 | 0.18130 | 6 | 2 | 3 | 3 |
| 1785 | TRIP12 protein | IPI00032342 | -0.501 | 0.18130 | 5 | 3 | 3 | 3 |
| 1786 | Small nuclear ribonucleoprotein Sm D3 | IPI00017964 | -0.501 | 0.18130 | 4 | 4 | 3 | 3 |
| 1787 | Isoform 2 of Isochorismatase domain-containing protein 2, mitochondrial | IPI00003031 | -0.501 | 0.18130 | 5 | 3 | 4 | 2 |
| 1788 | Pyruvate dehydrogenase protein X component, mitochondrial | IPI00298423 | -0.501 | 0.18130 | 4 | 4 | 3 | 3 |
| 1789 | Isoform 1 of Chromosome-associated kinesin KIF4A | IPI00178150 | -0.501 | 0.18130 | 3 | 5 | 3 | 3 |
| 1790 | Putative high mobility group protein 1-like 10 | IPI00018755 | -0.501 | 0.18130 | 6 | 2 | 2 | 4 |
| 1791 | X-Pro aminopeptidase 1, soluble isoform 2 | IPI00607814 | -0.501 | 0.18130 | 5 | 3 | 2 | 4 |
| 1792 | cDNA FLJ56184, highly similar to Proto-oncogene tyrosine-protein kinase LCK | IPI00394952 | -0.501 | 0.18130 | 4 | 4 | 4 | 2 |
| 1793 | N(G),N(G)-dimethylarginine dimethylaminohydrolase 2 | IPI00000760 | -0.501 | 0.18130 | 5 | 3 | 4 | 2 |
| 1794 | Probable methylthioribulose-1-phosphate dehydratase | IPI00549730 | -0.501 | 0.18130 | 4 | 4 | 4 | 2 |
| 1795 | SRA stem-loop-interacting RNA-binding protein, mitochondrial | IPI00009922 | -0.501 | 0.18130 | 5 | 3 | 3 | 3 |
| 1796 | Protein C20orf11 | IPI00016634 | -0.501 | 0.18130 | 6 | 2 | 3 | 3 |
| 1797 | Isoform 1 of Peroxisomal acyl-coenzyme A oxidase 1 | IPI00296907 | -0.501 | 0.18130 | 3 | 5 | 2 | 4 |
| 1798 | Ribosome biogenesis protein BOP1 | IPI00028955 | -0.501 | 0.18130 | 2 | 6 | 4 | 2 |
| 1799 | Isoform 2 of DnaJ homolog subfamily C member 2 | IPI00455199 | -0.501 | 0.18130 | 5 | 3 | 4 | 2 |

| No. | Description | Accession number | STN[1] | p-Value[1] | Con_A[2] | Con_B[2] | LUTEO_A[2] | LUTEO_B[2] |
|---|---|---|---|---|---|---|---|---|
| 1800 | Isoform A of Peptidyl-prolyl cis-trans isomerase E | IPI00009316 | -0.501 | 0.18130 | 5 | 3 | 3 | 3 |
| 1801 | Ewing sarcoma breakpoint region 1 isoform 1 | IPI00009841 | -0.501 | 0.18130 | 5 | 3 | 3 | 3 |
| 1802 | Isoform 1 of Low molecular weight phosphotyrosine protein phosphatase | IPI00219861 | -0.501 | 0.18130 | 4 | 4 | 2 | 4 |
| 1803 | proteasome subunit beta type-5 isoform 3 | IPI00383971 | -0.501 | 0.18130 | 4 | 4 | 3 | 3 |
| 1804 | Isoform 1 of Pyruvate dehydrogenase E1 component subunit beta, mitochondrial | IPI00003925 | -0.510 | 0.16640 | 11 | 9 | 10 | 7 |
| 1805 | Poly(rC)-binding protein 1 | IPI00016610 | -0.521 | 0.16507 | 11 | 8 | 9 | 7 |
| 1806 | Cytochrome b-c1 complex subunit 2, mitochondrial | IPI00305383 | -0.521 | 0.16507 | 10 | 9 | 8 | 8 |
| 1807 | SWI/SNF complex subunit SMARCC1 | IPI00234252 | -0.521 | 0.16507 | 11 | 8 | 8 | 8 |
| 1808 | Laminin receptor-like protein LAMRL5 | IPI00411639 | -0.521 | 0.16478 | 20 | 18 | 19 | 15 |
| 1809 | Isoform 1 of Nucleoside diphosphate kinase B | IPI00026260 | -0.527 | 0.16445 | 21 | 16 | 18 | 15 |
| 1810 | Isoform 1 of Serine/arginine repetitive matrix protein 2 | IPI00782992 | -0.533 | 0.16436 | 3 | 4 | 1 | 4 |
| 1811 | Probable ATP-dependent RNA helicase DHX37 | IPI00217630 | -0.533 | 0.16436 | 5 | 2 | 2 | 3 |
| 1812 | 60S ribosomal protein L31 | IPI00026302 | -0.533 | 0.16436 | 3 | 4 | 3 | 2 |
| 1813 | Protein S100-A6 | IPI00027463 | -0.533 | 0.16436 | 4 | 3 | 4 | 1 |
| 1814 | cDNA FLJ55177, highly similar to Ras-related protein Ral-B | IPI00004397 | -0.533 | 0.16436 | 4 | 3 | 3 | 2 |
| 1815 | Isoform 1 of Kinesin-like protein KIF2A | IPI00010368 | -0.533 | 0.16436 | 4 | 3 | 4 | 1 |
| 1816 | Cleavage and polyadenylation specificity factor subunit 2 | IPI00419531 | -0.533 | 0.16436 | 6 | 1 | 4 | 0 |
| 1817 | Isoform 1 of OCIA domain-containing protein 2 | IPI00555902 | -0.533 | 0.16436 | 3 | 4 | 3 | 2 |
| 1818 | Transcription initiation factor IIE subunit beta | IPI00019981 | -0.533 | 0.16436 | 3 | 4 | 2 | 3 |
| 1819 | Dihydrofolate reductase | IPI00030357 | -0.533 | 0.16436 | 4 | 3 | 2 | 3 |
| 1820 | sphingomyelin phosphodiesterase 4 isoform 1 | IPI00743121 | -0.533 | 0.16436 | 1 | 6 | 3 | 2 |
| 1821 | Cytovillin 2 (Fragment) | IPI00384282 | -0.533 | 0.16436 | 3 | 4 | 3 | 2 |
| 1822 | NADH dehydrogenase [ubiquinone] 1 beta subcomplex subunit 4 | IPI00220059 | -0.533 | 0.16436 | 4 | 3 | 3 | 2 |
| 1823 | Ras-related protein Rab-5B | IPI00017344 | -0.533 | 0.16436 | 5 | 2 | 3 | 2 |
| 1824 | Serine/threonine-protein phosphatase PP1-beta catalytic subunit | IPI00218236 | -0.533 | 0.16436 | 3 | 4 | 3 | 2 |
| 1825 | Sec1 family domain-containing protein 1 | IPI00165261 | -0.533 | 0.16331 | 8 | 10 | 6 | 9 |
| 1826 | 26S protease regulatory subunit 7 | IPI00021435 | -0.533 | 0.16331 | 9 | 9 | 7 | 8 |
| 1827 | Isoform 2 of Cat eye syndrome critical region protein 5 | IPI00011511 | -0.533 | 0.16331 | 9 | 9 | 8 | 7 |
| 1828 | ADP-ribosylation factor-like protein 1 | IPI00219518 | -0.540 | 0.16208 | 19 | 16 | 19 | 12 |
| 1829 | Activated RNA polymerase II transcriptional coactivator p15 | IPI00221222 | -0.547 | 0.16103 | 9 | 8 | 8 | 6 |
| 1830 | Alpha-centractin | IPI00029468 | -0.547 | 0.16103 | 9 | 8 | 8 | 6 |
| 1831 | UPF0468 protein C16orf80 | IPI00001655 | -0.547 | 0.16103 | 8 | 9 | 7 | 7 |
| 1832 | Regulation of nuclear pre-mRNA domain-containing protein 1B | IPI00009659 | -0.547 | 0.16103 | 9 | 8 | 7 | 7 |
| 1833 | Ribosomal protein S6 kinase alpha-6 | IPI00007123 | -0.547 | 0.16103 | 8 | 9 | 6 | 8 |
| 1834 | 60S acidic ribosomal protein P0 | IPI00008530 | -0.553 | 0.16037 | 29 | 28 | 27 | 25 |
| 1835 | Isoform 1 of Heterogeneous nuclear ribonucleoprotein U-like protein 1 | IPI00013070 | -0.562 | 0.15890 | 11 | 5 | 8 | 5 |
| 1836 | S-adenosylmethionine synthase isoform type-2 | IPI00010157 | -0.562 | 0.15890 | 8 | 8 | 5 | 8 |
| 1837 | Src substrate cortactin | IPI00029601 | -0.562 | 0.15890 | 8 | 8 | 7 | 6 |
| 1838 | Isoform 1 of 60S ribosomal protein L11 | IPI00376798 | -0.568 | 0.15728 | 16 | 15 | 14 | 13 |
| 1839 | HMT1 hnRNP methyltransferase-like 2 isoform 1 | IPI00018522 | -0.568 | 0.15728 | 16 | 15 | 14 | 13 |
| 1840 | epiplakin | IPI00010951 | -0.574 | 0.15709 | 3 | 3 | 2 | 2 |
| 1841 | Leucine-rich repeat-containing protein 40 | IPI00152998 | -0.574 | 0.15709 | 2 | 4 | 2 | 2 |
| 1842 | Isoform 1 of 14-3-3 protein sigma | IPI00013890 | -0.574 | 0.15709 | 4 | 2 | 2 | 2 |
| 1843 | Isoform 6 of GTPase-activating protein and VPS9 domain-containing protein 1 | IPI00292753 | -0.574 | 0.15709 | 3 | 3 | 2 | 2 |
| 1844 | Histone H2A type 1-H | IPI00081836 | -0.574 | 0.15709 | 2 | 4 | 2 | 2 |
| 1845 | 24 kDa protein | IPI00397611 | -0.574 | 0.15709 | 3 | 3 | 2 | 2 |
| 1846 | Cyclin-G-associated kinase | IPI00298949 | -0.574 | 0.15709 | 4 | 2 | 2 | 2 |
| 1847 | Tyrosyl-tRNA synthetase, mitochondrial | IPI00165092 | -0.574 | 0.15709 | 3 | 3 | 2 | 2 |
| 1848 | Serine/threonine-protein phosphatase 2A 65 kDa regulatory subunit A alpha isoform | IPI00554737 | -0.574 | 0.15709 | 3 | 3 | 1 | 3 |
| 1849 | Mitochondrial import receptor subunit TOM20 homolog | IPI00016676 | -0.574 | 0.15709 | 4 | 2 | 2 | 2 |
| 1850 | Isocitrate dehydrogenase [NADP] cytoplasmic | IPI00027223 | -0.574 | 0.15709 | 3 | 3 | 2 | 2 |
| 1851 | 51 kDa protein | IPI00033025 | -0.574 | 0.15709 | 3 | 3 | 2 | 2 |
| 1852 | Isoform 1 of Acyl-coenzyme A thioesterase 2, mitochondrial | IPI00220906 | -0.574 | 0.15709 | 4 | 2 | 2 | 2 |
| 1853 | cDNA FLJ55034, highly similar to Dihydrolipoyllysine-residue succinyltransferase component | IPI00384122 | -0.574 | 0.15709 | 3 | 3 | 2 | 2 |
| 1854 | Isoform 2 of Sorting nexin-3 | IPI00216508 | -0.574 | 0.15709 | 3 | 3 | 2 | 2 |
| 1855 | Transmembrane emp24 domain-containing protein 7 | IPI00032825 | -0.574 | 0.15709 | 3 | 3 | 3 | 1 |
| 1856 | Protein FAM49A | IPI00006574 | -0.574 | 0.15709 | 3 | 3 | 2 | 2 |
| 1857 | Guanine nucleotide-binding protein subunit alpha-14 | IPI00000695 | -0.574 | 0.15709 | 3 | 3 | 2 | 2 |
| 1858 | Ras-related protein Rap-2b | IPI00018364 | -0.574 | 0.15709 | 3 | 3 | 2 | 2 |
| 1859 | ELMO domain-containing protein 2 | IPI00217918 | -0.574 | 0.15709 | 3 | 3 | 2 | 2 |
| 1860 | Isoform 1 of 28S ribosomal protein S5, mitochondrial | IPI00169400 | -0.574 | 0.15709 | 3 | 3 | 2 | 2 |
| 1861 | NADH dehydrogenase [ubiquinone] 1 beta subcomplex subunit 7 | IPI00219772 | -0.574 | 0.15709 | 3 | 3 | 2 | 2 |
| 1862 | Pre-mRNA-splicing factor SPF27 | IPI00025178 | -0.574 | 0.15709 | 3 | 3 | 1 | 3 |
| 1863 | Isoform 1 of Protein kinase C and casein kinase substrate in neurons protein 2 | IPI00027009 | -0.574 | 0.15709 | 3 | 3 | 0 | 3 |
| 1864 | Isoform 1 of Caprin-1 | IPI00783872 | -0.576 | 0.13568 | 14 | 16 | 13 | 13 |
| 1865 | Developmentally-regulated GTP-binding protein 1 | IPI00031836 | -0.578 | 0.13483 | 7 | 8 | 6 | 6 |
| 1866 | 3-mercaptopyruvate sulfurtransferase | IPI00165360 | -0.578 | 0.13483 | 7 | 8 | 6 | 6 |
| 1867 | Isoform 1 of Aldehyde dehydrogenase family 16 member A1 | IPI00217920 | -0.578 | 0.13483 | 7 | 8 | 8 | 4 |
| 1868 | 40S ribosomal protein S3 | IPI00011253 | -0.578 | 0.13430 | 37 | 43 | 38 | 36 |
| 1869 | Eukaryotic translation initiation factor 5A-2 | IPI00006935 | -0.594 | 0.13364 | 16 | 12 | 12 | 12 |
| 1870 | Eukaryotic initiation factor 4A-III | IPI00009328 | -0.594 | 0.13364 | 13 | 15 | 12 | 12 |
| 1871 | Isoform 1 of Apoptotic chromatin condensation inducer in the nucleus | IPI00007334 | -0.596 | 0.13217 | 6 | 8 | 5 | 6 |
| 1872 | Isoform 1 of Protein canopy homolog 2 | IPI00443909 | -0.596 | 0.13217 | 7 | 7 | 7 | 4 |
| 1873 | Vitamin K epoxide reductase complex subunit 1-like protein 1 | IPI00166079 | -0.596 | 0.13217 | 8 | 6 | 6 | 5 |
| 1874 | Vesicle-trafficking protein SEC22b | IPI00006865 | -0.596 | 0.13217 | 8 | 6 | 6 | 5 |
| 1875 | Peroxiredoxin-6 | IPI00220301 | -0.597 | 0.13089 | 53 | 55 | 50 | 51 |
| 1876 | 60S acidic ribosomal protein P2 | IPI00008529 | -0.603 | 0.13089 | 13 | 14 | 14 | 9 |
| 1877 | Isoform 3 of DNA topoisomerase 2-alpha | IPI00218753 | -0.611 | 0.13036 | 37 | 33 | 35 | 29 |
| 1878 | Proliferating cell nuclear antigen | IPI00021700 | -0.615 | 0.12875 | 37 | 32 | 29 | 34 |
| 1879 | Heat shock protein HSP 90-beta | IPI00414676 | -0.615 | 0.12875 | 94 | 93 | 94 | 84 |
| 1880 | Thyroid hormone receptor-associated protein 3 | IPI00104050 | -0.616 | 0.12861 | 8 | 5 | 4 | 6 |
| 1881 | Adenine phosphoribosyltransferase | IPI00218693 | -0.616 | 0.12861 | 7 | 6 | 5 | 5 |
| 1882 | YLP motif-containing protein 1 | IPI00165434 | -0.616 | 0.12861 | 6 | 7 | 5 | 5 |
| 1883 | 60S ribosomal protein L26-like 1 | IPI00007144 | -0.616 | 0.12861 | 9 | 4 | 5 | 5 |
| 1884 | Small nuclear ribonucleoprotein E | IPI00029266 | -0.616 | 0.12861 | 8 | 5 | 5 | 5 |
| 1885 | Eukaryotic translation initiation factor 3 subunit I | IPI00012795 | -0.616 | 0.12861 | 7 | 6 | 3 | 7 |
| 1886 | Calcium-binding mitochondrial carrier protein Aralar1 | IPI00386271 | -0.616 | 0.12861 | 6 | 7 | 4 | 6 |
| 1887 | protein arginine N-methyltransferase 5 isoform b | IPI00064328 | -0.623 | 0.12709 | 11 | 14 | 11 | 10 |
| 1888 | Isoform 1 of Mitochondrial inner membrane protein | IPI00009960 | -0.628 | 0.12704 | 20 | 22 | 21 | 16 |
| 1889 | Pumilio domain-containing protein KIAA0020 | IPI00791325 | -0.629 | 0.12690 | 2 | 3 | 1 | 2 |
| 1890 | ATP-binding cassette sub-family F member 2 | IPI00005045 | -0.629 | 0.12690 | 2 | 3 | 2 | 1 |
| 1891 | DKFZP586J0619 protein | IPI00740961 | -0.629 | 0.12690 | 1 | 4 | 1 | 2 |
| 1892 | Isoform 1 of Putative ATP-dependent RNA helicase DHX57 | IPI00168885 | -0.629 | 0.12690 | 3 | 2 | 1 | 2 |
| 1893 | Isoform 3 of Protein VPRBP | IPI00181396 | -0.629 | 0.12690 | 3 | 2 | 1 | 2 |
| 1894 | Band 4.1-like protein 2 | IPI00015973 | -0.629 | 0.12690 | 3 | 2 | 1 | 2 |

| No. | Description | Accession number | STN[1] | p-Value[1] | Con_A[2] | Con_B[2] | LUTEO_A[2] | LUTEO_B[2] |
|---|---|---|---|---|---|---|---|---|
| 1895 | UPF0553 protein C9orf64 | IPI00170972 | -0.629 | 0.12690 | 2 | 3 | 1 | 2 |
| 1896 | Exosome complex exonuclease RRP43 | IPI00552920 | -0.629 | 0.12690 | 2 | 3 | 2 | 0 |
| 1897 | Protein phosphatase 1 regulatory subunit 14B | IPI00398922 | -0.629 | 0.12690 | 4 | 1 | 2 | 1 |
| 1898 | Isoform 2 of Syntaxin-5 | IPI00386786 | -0.629 | 0.12690 | 3 | 2 | 1 | 2 |
| 1899 | Heme oxygenase 2 | IPI00026824 | -0.629 | 0.12690 | 2 | 3 | 1 | 2 |
| 1900 | Probable asparaginyl-tRNA synthetase, mitochondrial | IPI00101664 | -0.629 | 0.12690 | 2 | 3 | 2 | 1 |
| 1901 | Small glutamine-rich tetratricopeptide repeat-containing protein alpha | IPI00013949 | -0.629 | 0.12690 | 2 | 3 | 1 | 2 |
| 1902 | inosine-5'-monophosphate dehydrogenase 1 isoform a | IPI00375527 | -0.629 | 0.12690 | 1 | 4 | 0 | 2 |
| 1903 | V-type proton ATPase subunit d 1 | IPI00034159 | -0.629 | 0.12690 | 3 | 2 | 1 | 2 |
| 1904 | Isoform 1 of 39S ribosomal protein L47, mitochondrial | IPI00030820 | -0.629 | 0.12690 | 2 | 3 | 1 | 2 |
| 1905 | Isoform 1 of DAZ-associated protein 1 | IPI00165230 | -0.629 | 0.12690 | 3 | 2 | 2 | 1 |
| 1906 | Isoform 1 of YTH domain family protein 2 | IPI00306043 | -0.629 | 0.12690 | 3 | 2 | 2 | 0 |
| 1907 | Histone acetyltransferase MYST2 | IPI00180764 | -0.629 | 0.12690 | 2 | 3 | 1 | 2 |
| 1908 | Survival of motor neuron-related-splicing factor 30 | IPI00025176 | -0.629 | 0.12690 | 2 | 3 | 2 | 1 |
| 1909 | Isoform 1 of Required for meiotic nuclear division protein 1 homolog | IPI00329591 | -0.629 | 0.12690 | 3 | 2 | 1 | 2 |
| 1910 | Isoform 1 of Nucleolar protein 14 | IPI00022613 | -0.629 | 0.12690 | 3 | 2 | 1 | 2 |
| 1911 | PDZ domain-containing protein GIPC1 | IPI00024705 | -0.629 | 0.12690 | 3 | 2 | 2 | 0 |
| 1912 | Isoform 1 of Heterogeneous nuclear ribonucleoprotein L-like | IPI00103247 | -0.629 | 0.12690 | 3 | 2 | 1 | 2 |
| 1913 | N-alpha-acetyltransferase 20, NatB catalytic subunit | IPI00007174 | -0.629 | 0.12690 | 3 | 2 | 2 | 0 |
| 1914 | Proteasome maturation protein | IPI00006377 | -0.629 | 0.12690 | 3 | 2 | 0 | 2 |
| 1915 | Isoform Long of Ancient ubiquitous protein 1 | IPI00001891 | -0.629 | 0.12690 | 3 | 2 | 2 | 1 |
| 1916 | Gem-associated protein 7 | IPI00003027 | -0.629 | 0.12690 | 4 | 0 | 2 | 1 |
| 1917 | DNA polymerase subunit gamma-1 | IPI00004317 | -0.629 | 0.12690 | 2 | 3 | 1 | 2 |
| 1918 | cDNA FLJ58573, highly similar to Exonuclease 3'-5' domain-like-containing protein 2 | IPI00465113 | -0.629 | 0.12690 | 2 | 3 | 2 | 1 |
| 1919 | Actin, aortic smooth muscle | IPI00008603 | -0.630 | 0.12690 | 38 | 57 | 43 | 45 |
| 1920 | Isoform 1 of Adenylyl cyclase-associated protein 1 | IPI00008274 | -0.632 | 0.12690 | 52 | 42 | 48 | 39 |
| 1921 | Isoform A of Lamin-A/C | IPI00021405 | -0.634 | 0.12690 | 36 | 28 | 29 | 29 |
| 1922 | Actin-related protein 2/3 complex subunit 4 | IPI00554811 | -0.634 | 0.12676 | 14 | 10 | 9 | 11 |
| 1923 | Isoform 1 of Apolipoprotein O | IPI00042580 | -0.634 | 0.12676 | 15 | 9 | 11 | 9 |
| 1924 | Sepiapterin reductase | IPI00017469 | -0.634 | 0.12676 | 13 | 11 | 10 | 10 |
| 1925 | CTP synthase 1 | IPI00290142 | -0.638 | 0.12481 | 35 | 28 | 28 | 29 |
| 1926 | Mitochondrial-processing peptidase subunit alpha | IPI00166749 | -0.639 | 0.12481 | 5 | 7 | 5 | 4 |
| 1927 | Isoform 1 of Transcription elongation factor SPT5 | IPI00298058 | -0.639 | 0.12481 | 5 | 7 | 4 | 5 |
| 1928 | EH domain-containing protein 4 | IPI00005578 | -0.639 | 0.12481 | 7 | 5 | 5 | 4 |
| 1929 | Helicase SKI2W | IPI00414819 | -0.639 | 0.12481 | 6 | 6 | 5 | 4 |
| 1930 | Isoform 3 of Protein transport protein Sec31A | IPI00305152 | -0.639 | 0.12481 | 6 | 6 | 4 | 5 |
| 1931 | Isoform 2 of Condensin-2 complex subunit G2 | IPI00396058 | -0.639 | 0.12481 | 7 | 5 | 5 | 4 |
| 1932 | Dihydropteridine reductase | IPI00014439 | -0.639 | 0.12481 | 6 | 6 | 4 | 5 |
| 1933 | Glutamate-rich WD repeat-containing protein 1 | IPI00027831 | -0.639 | 0.12481 | 5 | 7 | 5 | 4 |
| 1934 | Isoform 1 of Ubiquitin-conjugating enzyme E2 variant 1 | IPI00019599 | -0.639 | 0.12481 | 5 | 7 | 6 | 3 |
| 1935 | Isoform Short of Adenosine kinase | IPI00234368 | -0.646 | 0.12177 | 12 | 11 | 10 | 9 |
| 1936 | cDNA FLJ60076, highly similar to ELAV-like protein 1 | IPI00301936 | -0.646 | 0.12177 | 12 | 11 | 9 | 10 |
| 1937 | Nascent polypeptide-associated complex subunit alpha | IPI00023748 | -0.648 | 0.12177 | 21 | 18 | 16 | 18 |
| 1938 | Interleukin enhancer-binding factor 2 | IPI00005198 | -0.656 | 0.12087 | 43 | 43 | 39 | 40 |
| 1939 | Isoform 2 of ATPase family AAA domain-containing protein 3A | IPI00295992 | -0.659 | 0.12087 | 12 | 10 | 9 | 9 |
| 1940 | Isoform 1 of Protein unc-45 homolog A | IPI00072534 | -0.666 | 0.11793 | 6 | 5 | 5 | 3 |
| 1941 | Isoform 1 of Cullin-4A | IPI00419273 | -0.666 | 0.11793 | 5 | 6 | 6 | 2 |
| 1942 | regulator of chromosome condensation 1 isoform a | IPI00001661 | -0.666 | 0.11793 | 7 | 4 | 5 | 3 |
| 1943 | Isoform A1 of Tight junction protein ZO-2 | IPI00003843 | -0.666 | 0.11793 | 5 | 6 | 4 | 4 |
| 1944 | Isoform 1 of 39S ribosomal protein L22, mitochondrial | IPI00414410 | -0.666 | 0.11793 | 7 | 4 | 4 | 4 |
| 1945 | ATP synthase subunit O, mitochondrial | IPI00007611 | -0.666 | 0.11793 | 5 | 6 | 5 | 3 |
| 1946 | Isoform 1 of Tropomyosin alpha-4 chain | IPI00010779 | -0.666 | 0.11793 | 6 | 5 | 4 | 4 |
| 1947 | Nuclear RNA export factor 1 | IPI00033153 | -0.673 | 0.11664 | 10 | 11 | 10 | 7 |
| 1948 | Isoform 1 of Cytosol aminopeptidase | IPI00419237 | -0.679 | 0.11660 | 17 | 18 | 15 | 15 |
| 1949 | Isoform 2 of Eukaryotic translation initiation factor 5A-1 | IPI00376005 | -0.683 | 0.11626 | 38 | 40 | 33 | 38 |
| 1950 | Eukaryotic translation initiation factor 3 subunit E | IPI00013068 | -0.687 | 0.11607 | 11 | 9 | 8 | 8 |
| 1951 | Isoform Long of Antigen KI-67 | IPI00004233 | -0.696 | 0.11294 | 5 | 5 | 4 | 3 |
| 1952 | Isoform 1 of Nuclear pore complex protein Nup214 | IPI00183294 | -0.696 | 0.11294 | 4 | 6 | 4 | 3 |
| 1953 | Paladin | IPI00297212 | -0.696 | 0.11294 | 4 | 6 | 3 | 4 |
| 1954 | 60S ribosomal protein L32 | IPI00395998 | -0.696 | 0.11294 | 5 | 5 | 4 | 3 |
| 1955 | SNW domain-containing protein 1 | IPI00013830 | -0.696 | 0.11294 | 6 | 4 | 4 | 3 |
| 1956 | Amidophosphoribosyltransferase | IPI00029534 | -0.696 | 0.11294 | 5 | 5 | 3 | 4 |
| 1957 | RuvB-like 2 | IPI00009104 | -0.696 | 0.11052 | 16 | 17 | 14 | 14 |
| 1958 | 14-3-3 protein theta | IPI00018146 | -0.696 | 0.11052 | 17 | 16 | 16 | 12 |
| 1959 | Isoform Gamma-1 of Serine/threonine-protein phosphatase PP1-gamma catalytic subunit | IPI00005705 | -0.703 | 0.11033 | 39 | 34 | 37 | 29 |
| 1960 | 26S protease regulatory subunit S10B | IPI00021926 | -0.703 | 0.11033 | 10 | 9 | 8 | 7 |
| 1961 | Malate dehydrogenase | IPI00916111 | -0.703 | 0.11033 | 10 | 9 | 8 | 7 |
| 1962 | FACT complex subunit SSRP1 | IPI00005154 | -0.706 | 0.11000 | 17 | 15 | 13 | 14 |
| 1963 | 40S ribosomal protein S17 | IPI00221093 | -0.706 | 0.11000 | 16 | 16 | 15 | 12 |
| 1964 | Isoform 1 of Ral GTPase-activating protein subunit beta | IPI00409601 | -0.709 | 0.10957 | 2 | 2 | 1 | 1 |
| 1965 | Putative uncharacterized protein TMX2 | IPI00329596 | -0.709 | 0.10957 | 2 | 2 | 0 | 0 |
| 1966 | Isoform 1 of Dynamin-like 120 kDa protein, mitochondrial | IPI00006721 | -0.709 | 0.10957 | 1 | 3 | 0 | 0 |
| 1967 | Beta-adrenergic receptor kinase 1 | IPI00012497 | -0.709 | 0.10957 | 3 | 1 | 1 | 1 |
| 1968 | 28S ribosomal protein S34, mitochondrial | IPI00169413 | -0.709 | 0.10957 | 2 | 2 | 1 | 1 |
| 1969 | Isoform 1 of Translation initiation factor eIF-2B subunit gamma | IPI00006504 | -0.709 | 0.10957 | 2 | 2 | 0 | 0 |
| 1970 | Cohesin subunit SA-1 | IPI00025158 | -0.709 | 0.10957 | 1 | 3 | 0 | 1 |
| 1971 | Isoform 1 of Anaphase-promoting complex subunit 7 | IPI00008248 | -0.709 | 0.10957 | 2 | 2 | 1 | 1 |
| 1972 | ATP synthase subunit g, mitochondrial | IPI00027448 | -0.709 | 0.10957 | 2 | 2 | 1 | 1 |
| 1973 | Isoform 2 of Carbohydrate kinase domain-containing protein | IPI00645172 | -0.709 | 0.10957 | 2 | 2 | 1 | 1 |
| 1974 | Major centromere autoantigen B | IPI00010388 | -0.709 | 0.10957 | 2 | 2 | 1 | 1 |
| 1975 | Acyl-CoA desaturase | IPI00299468 | -0.709 | 0.10957 | 3 | 1 | 1 | 0 |
| 1976 | Isoform 2 of Transducin-like enhancer protein 3 | IPI00177938 | -0.709 | 0.10957 | 3 | 1 | 1 | 1 |
| 1977 | Isoform 1 of Mitochondrial antiviral-signaling protein | IPI00020719 | -0.709 | 0.10957 | 3 | 1 | 1 | 1 |
| 1978 | Isoform Long of Metastasis-associated protein MTA1 | IPI00012773 | -0.709 | 0.10957 | 2 | 2 | 1 | 0 |
| 1979 | Isoform 1 of DEP domain-containing protein 7 | IPI00163266 | -0.709 | 0.10957 | 2 | 2 | 1 | 0 |
| 1980 | Isoform 1 of Rhotekin | IPI00029834 | -0.709 | 0.10957 | 1 | 3 | 1 | 1 |
| 1981 | Isoform 2 of Peptidyl-prolyl cis-trans isomerase-like 3 | IPI00032473 | -0.709 | 0.10957 | 2 | 2 | 0 | 1 |
| 1982 | Tryptophanyl-tRNA synthetase, mitochondrial | IPI00025050 | -0.709 | 0.10957 | 2 | 2 | 1 | 1 |
| 1983 | Putative uncharacterized protein TXNRD2 | IPI00157820 | -0.709 | 0.10957 | 3 | 1 | 1 | 1 |
| 1984 | Ethanolamine kinase 1 | IPI00030090 | -0.709 | 0.10957 | 2 | 2 | 1 | 0 |
| 1985 | Phosphomevalonate kinase | IPI00220648 | -0.709 | 0.10957 | 3 | 1 | 1 | 1 |
| 1986 | Isoform 1 of Protein VPRBP | IPI00329528 | -0.709 | 0.10957 | 3 | 1 | 0 | 0 |
| 1987 | Ribosomal protein L1 | IPI00035167 | -0.709 | 0.10957 | 2 | 2 | 1 | 1 |
| 1988 | Isoform 2 of 1,2-dihydroxy-3-keto-5-methylthiopentene dioxygenase | IPI00470791 | -0.709 | 0.10957 | 3 | 1 | 0 | 0 |
| 1989 | Sorting nexin-9 | IPI00001883 | -0.709 | 0.10957 | 1 | 3 | 0 | 1 |

| No. | Description | Accession number | STN[1] | p-Value[1] | Con_A[2] | Con_B[2] | LUTEO_A[2] | LUTEO_B[2] |
|---|---|---|---|---|---|---|---|---|
| 1990 | Peptidyl-prolyl cis-trans isomerase-like 4 | IPI00642862 | -0.709 | 0.10957 | 3 | 0 | 0 | 1 |
| 1991 | Diablo homolog, mitochondrial precursor | IPI00008418 | -0.709 | 0.10957 | 2 | 2 | 1 | 0 |
| 1992 | Isoform 1 of Putative deoxyribonuclease TATDN1 | IPI00012463 | -0.709 | 0.10957 | 2 | 2 | 1 | 1 |
| 1993 | Isoform Long of Transcription intermediary factor 1-alpha | IPI00005184 | -0.709 | 0.10957 | 2 | 2 | 1 | 1 |
| 1994 | 13kDa differentiation-associated protein variant (Fragment) | IPI00005966 | -0.709 | 0.10957 | 2 | 2 | 1 | 0 |
| 1995 | Isoform 1 of Protein 4.1 | IPI00003921 | -0.709 | 0.10957 | 2 | 2 | 0 | 1 |
| 1996 | Endonuclease G, mitochondrial | IPI00290614 | -0.709 | 0.10957 | 2 | 2 | 0 | 0 |
| 1997 | Isoform 1 of Transcriptional repressor p66-alpha | IPI00410330 | -0.709 | 0.10957 | 0 | 3 | 1 | 0 |
| 1998 | Isoform 2 of WASH complex subunit FAM21C | IPI00456853 | -0.709 | 0.10957 | 2 | 2 | 0 | 0 |
| 1999 | Keratin, type II cuticular Hb5 | IPI00032541 | -0.709 | 0.10957 | 3 | 0 | 0 | 0 |
| 2000 | Isoform 1 of Serologically defined colon cancer antigen 1 | IPI00301618 | -0.709 | 0.10957 | 3 | 0 | 0 | 0 |
| 2001 | GTP-binding protein GUF1 homolog | IPI00296563 | -0.709 | 0.10957 | 1 | 3 | 1 | 0 |
| 2002 | Isoform 1 of Presenilin-1 | IPI00028077 | -0.709 | 0.10957 | 2 | 2 | 1 | 1 |
| 2003 | 39S ribosomal protein L53, mitochondrial | IPI00061531 | -0.709 | 0.10957 | 2 | 2 | 0 | 1 |
| 2004 | Isoform 1 of Chaperone activity of bc1 complex-like, mitochondrial | IPI00176469 | -0.709 | 0.10957 | 3 | 1 | 0 | 0 |
| 2005 | Integrator complex subunit 9 | IPI00290514 | -0.709 | 0.10957 | 1 | 3 | 0 | 0 |
| 2006 | Isoform 1 of Structural maintenance of chromosomes protein 4 | IPI00411559 | -0.709 | 0.10957 | 2 | 2 | 1 | 1 |
| 2007 | Isoform 1 of Transcription elongation factor A protein 1 | IPI00333215 | -0.709 | 0.10957 | 1 | 3 | 1 | 1 |
| 2008 | Growth arrest and DNA damage-inducible proteins-interacting protein 1 | IPI00552587 | -0.709 | 0.10957 | 3 | 1 | 1 | 1 |
| 2009 | Isoform 1 of AP-3 complex subunit delta-1 | IPI00411453 | -0.709 | 0.10957 | 2 | 2 | 1 | 1 |
| 2010 | Isoform 1 of U4/U6 small nuclear ribonucleoprotein Prp3 | IPI00005861 | -0.709 | 0.10957 | 2 | 2 | 0 | 0 |
| 2011 | nucleoside diphosphate kinase type 6 | IPI00218214 | -0.709 | 0.10957 | 2 | 2 | 0 | 1 |
| 2012 | Diphthamide biosynthesis protein 2 | IPI00106549 | -0.709 | 0.10957 | 2 | 2 | 0 | 0 |
| 2013 | Methylmalonate-semialdehyde dehydrogenase [acylating], mitochondria | IPI00024990 | -0.709 | 0.10957 | 3 | 1 | 0 | 0 |
| 2014 | Isoform 1 of Transmembrane protein 192 | IPI00855873 | -0.709 | 0.10957 | 2 | 2 | 1 | 1 |
| 2015 | HDCMD34P | IPI00001672 | -0.709 | 0.10957 | 2 | 2 | 1 | 1 |
| 2016 | Ribosomal RNA processing protein 1 homolog A | IPI00550766 | -0.709 | 0.10957 | 2 | 2 | 0 | 0 |
| 2017 | Isoform 1 of Protein FAM65A | IPI00418799 | -0.709 | 0.10957 | 2 | 2 | 0 | 0 |
| 2018 | Isoform 1 of tRNA 2'-phosphotransferase 1 | IPI00328580 | -0.709 | 0.10957 | 2 | 2 | 1 | 1 |
| 2019 | perilipin-3 isoform 3 | IPI00106668 | -0.709 | 0.10957 | 2 | 2 | 1 | 1 |
| 2020 | Vacuolar protein sorting-associated protein 52 homolog | IPI00166132 | -0.709 | 0.10957 | 0 | 3 | 1 | 1 |
| 2021 | interferon alpha responsive protein | IPI00007096 | -0.709 | 0.10957 | 2 | 2 | 1 | 1 |
| 2022 | cDNA FLJ56221, highly similar to YTH domain protein 3 | IPI00396131 | -0.709 | 0.10957 | 2 | 2 | 1 | 0 |
| 2023 | Isoform 1 of tRNA guanosine-2'-O-methyltransferase TRM11 homolog | IPI00470606 | -0.709 | 0.10957 | 2 | 2 | 1 | 1 |
| 2024 | Isoform Short of NADPH:adrenodoxin oxidoreductase, mitochondrial | IPI00026958 | -0.709 | 0.10957 | 2 | 2 | 1 | 1 |
| 2025 | cDNA FLJ54848, highly similar to tRNA-splicing endonuclease subunit Sen34 | IPI00451941 | -0.709 | 0.10957 | 3 | 1 | 1 | 1 |
| 2026 | Isoform 1 of Vesicle-associated membrane protein 7 | IPI00020887 | -0.709 | 0.10957 | 2 | 2 | 0 | 0 |
| 2027 | Keratin, type II cytoskeletal 6B | IPI00293665 | -0.709 | 0.10957 | 2 | 2 | 1 | 1 |
| 2028 | Isoform Beta-1 of Protein phosphatase 1B | IPI00026612 | -0.709 | 0.10957 | 1 | 3 | 0 | 0 |
| 2029 | Peptidyl-prolyl cis-trans isomerase A | IPI00419585 | -0.714 | 0.08626 | 75 | 55 | 70 | 51 |
| 2030 | UPF0027 protein C22orf28 | IPI00550689 | -0.716 | 0.08612 | 14 | 17 | 15 | 11 |
| 2031 | Eukaryotic translation initiation factor 3 subunit C | IPI00016910 | -0.721 | 0.08555 | 11 | 7 | 7 | 7 |
| 2032 | Mitochondrial import receptor subunit TOM22 homolog | IPI00024976 | -0.721 | 0.08555 | 11 | 7 | 6 | 8 |
| 2033 | Isoform 5 of Interleukin enhancer-binding factor 3 | IPI00219330 | -0.724 | 0.08299 | 34 | 34 | 35 | 26 |
| 2034 | Talin-1 | IPI00298994 | -0.728 | 0.08270 | 37 | 56 | 39 | 46 |
| 2035 | Glutathione S-transferase P | IPI00219757 | -0.730 | 0.08232 | 109 | 93 | 96 | 95 |
| 2036 | Mitochondrial ribosomal protein L21 isoform d | IPI00375677 | -0.733 | 0.08227 | 6 | 3 | 2 | 4 |
| 2037 | Niban-like protein 1 | IPI00456750 | -0.733 | 0.08227 | 6 | 3 | 4 | 2 |
| 2038 | Mitochondrial fission 1 protein | IPI00007052 | -0.733 | 0.08227 | 5 | 4 | 3 | 3 |
| 2039 | Probable ATP-dependent RNA helicase DDX27 | IPI00293078 | -0.733 | 0.08227 | 6 | 3 | 4 | 2 |
| 2040 | Crk-like protein | IPI00004839 | -0.733 | 0.08227 | 6 | 3 | 3 | 3 |
| 2041 | Putative deoxyribose-phosphate aldolase | IPI00219677 | -0.733 | 0.08227 | 4 | 5 | 3 | 3 |
| 2042 | Isoform 3 of Splicing factor, arginine/serine-rich 13A | IPI00009071 | -0.737 | 0.08109 | 17 | 12 | 13 | 11 |
| 2043 | Signal recognition particle 72 kDa protein | IPI00215888 | -0.740 | 0.08075 | 7 | 10 | 8 | 5 |
| 2044 | Isoform 1 of Acetyl-CoA carboxylase 1 | IPI00011569 | -0.740 | 0.08075 | 6 | 11 | 5 | 8 |
| 2045 | Dolichyl-diphosphooligosaccharide--protein glycosyltransferase subunit STT3B | IPI00152377 | -0.740 | 0.08075 | 6 | 11 | 7 | 6 |
| 2046 | Estradiol 17-beta-dehydrogenase 11 | IPI00329598 | -0.740 | 0.08075 | 8 | 9 | 8 | 5 |
| 2047 | Interferon-induced, double-stranded RNA-activated protein kinase | IPI00019463 | -0.740 | 0.08075 | 9 | 8 | 8 | 5 |
| 2048 | Histone deacetylase 1 | IPI00013774 | -0.740 | 0.08075 | 8 | 9 | 7 | 6 |
| 2049 | T-complex protein 1 subunit epsilon | IPI00010720 | -0.752 | 0.07976 | 47 | 39 | 39 | 39 |
| 2050 | Isoform 1 of Protein SET | IPI00072377 | -0.752 | 0.07971 | 34 | 28 | 28 | 27 |
| 2051 | Isoform 1 of Hydroxyacyl-coenzyme A dehydrogenase, mitochondrial | IPI00294398 | -0.760 | 0.07867 | 14 | 13 | 11 | 11 |
| 2052 | Uncharacterized protein C17orf25 | IPI00007102 | -0.760 | 0.07867 | 17 | 10 | 13 | 9 |
| 2053 | Coatomer subunit zeta-1 | IPI00032851 | -0.760 | 0.07867 | 14 | 13 | 12 | 10 |
| 2054 | Golgi phosphoprotein 3 | IPI00005490 | -0.760 | 0.07843 | 7 | 9 | 6 | 6 |
| 2055 | GMP synthase [glutamine-hydrolyzing] | IPI00029079 | -0.766 | 0.07605 | 20 | 21 | 20 | 15 |
| 2056 | Serine/threonine-protein phosphatase 2A 56 kDa regulatory subunit epsilon isoform | IPI00002853 | -0.777 | 0.07501 | 3 | 5 | 2 | 3 |
| 2057 | Isoform SRP40-1 of Splicing factor, arginine/serine-rich 5 | IPI00012341 | -0.777 | 0.07501 | 4 | 4 | 3 | 2 |
| 2058 | ADP-ribosylation factor-like protein 3 | IPI00003327 | -0.777 | 0.07501 | 4 | 4 | 3 | 2 |
| 2059 | Isoform 1 of C-terminal-binding protein 2 | IPI00010120 | -0.777 | 0.07501 | 5 | 3 | 3 | 2 |
| 2060 | 39S ribosomal protein L23, mitochondrial | IPI00293476 | -0.777 | 0.07501 | 5 | 3 | 3 | 2 |
| 2061 | Isoform 2 of NSFL1 cofactor p47 | IPI00022830 | -0.777 | 0.07501 | 4 | 4 | 2 | 3 |
| 2062 | Emerin | IPI00032003 | -0.777 | 0.07501 | 5 | 3 | 3 | 2 |
| 2063 | Lanosterol synthase | IPI00009747 | -0.777 | 0.07501 | 5 | 3 | 2 | 3 |
| 2064 | T-complex protein 1 subunit eta | IPI00018465 | -0.782 | 0.07354 | 66 | 69 | 65 | 60 |
| 2065 | 26S proteasome non-ATPase regulatory subunit 3 | IPI00011603 | -0.782 | 0.07354 | 21 | 18 | 17 | 16 |
| 2066 | Splicing factor 3A subunit 1 | IPI00017451 | -0.783 | 0.07321 | 8 | 7 | 6 | 5 |
| 2067 | Galectin-3-binding protein | IPI00023673 | -0.783 | 0.07321 | 9 | 6 | 6 | 5 |
| 2068 | NEDD8 | IPI00020008 | -0.783 | 0.07321 | 8 | 7 | 5 | 6 |
| 2069 | Isoform 1 of Solute carrier family 12 member 2 | IPI00022649 | -0.783 | 0.07321 | 5 | 10 | 5 | 6 |
| 2070 | Tumor protein, translationally-controlled 1 | IPI00009943 | -0.783 | 0.07321 | 7 | 8 | 5 | 6 |
| 2071 | Mortality factor 4-like protein 2 | IPI00014174 | -0.783 | 0.07321 | 6 | 9 | 7 | 4 |
| 2072 | WD repeat-containing protein 61 | IPI00019269 | -0.786 | 0.07321 | 12 | 13 | 10 | 10 |
| 2073 | Protein RCC2 | IPI00465044 | -0.801 | 0.07221 | 13 | 11 | 9 | 10 |
| 2074 | coatomer subunit epsilon isoform b | IPI00399318 | -0.801 | 0.07221 | 13 | 11 | 11 | 8 |
| 2075 | Isoform Long of Eukaryotic translation initiation factor 4H | IPI00014263 | -0.801 | 0.07221 | 12 | 12 | 8 | 11 |
| 2076 | U2 small nuclear ribonucleoprotein B'' | IPI00029267 | -0.809 | 0.07192 | 8 | 6 | 6 | 4 |
| 2077 | SAP domain-containing ribonucleoprotein | IPI00014938 | -0.809 | 0.07192 | 5 | 9 | 6 | 4 |
| 2078 | Serpin B6 | IPI00413451 | -0.809 | 0.07192 | 7 | 7 | 5 | 5 |
| 2079 | Immunoglobulin-binding protein 1 | IPI00019148 | -0.809 | 0.07192 | 5 | 9 | 3 | 7 |
| 2080 | Cathepsin D | IPI00011229 | -0.816 | 0.06703 | 13 | 10 | 8 | 10 |
| 2081 | Elongation factor 1-beta | IPI00178440 | -0.816 | 0.06703 | 11 | 12 | 11 | 7 |
| 2082 | Isoform 1 of Transformer-2 protein homolog beta | IPI00301503 | -0.820 | 0.06675 | 20 | 15 | 14 | 15 |
| 2083 | Isoform 1 of Eukaryotic translation initiation factor 3 subunit B | IPI00396370 | -0.831 | 0.06661 | 16 | 18 | 16 | 12 |
| 2084 | Isoform 1 of Drebrin | IPI00003406 | -0.832 | 0.06618 | 4 | 3 | 2 | 2 |

| No. | Description | Accession number | STN[1] | p-Value[1] | Con_A[2] | Con_B[2] | LUTEO_A[2] | LUTEO_B[2] |
|---|---|---|---|---|---|---|---|---|
| 2085 | Succinyl-CoA ligase [GDP-forming] subunit alpha, mitochondrial | IPI00872762 | -0.832 | 0.06618 | 3 | 4 | 2 | 2 |
| 2086 | Talin-2 | IPI00219299 | -0.832 | 0.06618 | 3 | 4 | 2 | 2 |
| 2087 | Phosphoinositide 3-kinase regulatory subunit 4 | IPI00024006 | -0.832 | 0.06618 | 3 | 4 | 1 | 3 |
| 2088 | Methylosome protein 50 | IPI00012202 | -0.832 | 0.06618 | 3 | 4 | 3 | 1 |
| 2089 | Isoform 1 of Retinol dehydrogenase 11 | IPI00339384 | -0.832 | 0.06618 | 3 | 4 | 2 | 2 |
| 2090 | Isoform 1 of Calcium-binding mitochondrial carrier protein SCaMC-1 | IPI00337494 | -0.832 | 0.06618 | 4 | 3 | 2 | 2 |
| 2091 | Procollagen-lysine,2-oxoglutarate 5-dioxygenase 3 | IPI00030255 | -0.832 | 0.06618 | 4 | 3 | 2 | 2 |
| 2092 | NADH dehydrogenase [ubiquinone] 1 beta subcomplex subunit 9 | IPI00255052 | -0.832 | 0.06618 | 3 | 4 | 3 | 1 |
| 2093 | Superoxide dismutase [Mn], mitochondrial | IPI00022314 | -0.832 | 0.06618 | 4 | 3 | 2 | 2 |
| 2094 | Isoform 1 of Microtubule-associated protein 4 | IPI00396171 | -0.832 | 0.06618 | 4 | 3 | 3 | 1 |
| 2095 | 39S ribosomal protein L50, mitochondrial | IPI00329036 | -0.832 | 0.06618 | 4 | 3 | 2 | 2 |
| 2096 | Aminopeptidase B | IPI00642211 | -0.832 | 0.06618 | 3 | 4 | 3 | 0 |
| 2097 | 39S ribosomal protein L18, mitochondrial | IPI00160421 | -0.832 | 0.06618 | 4 | 3 | 2 | 2 |
| 2098 | Glutathione S-transferase kappa 1 | IPI00219673 | -0.833 | 0.06480 | 11 | 11 | 7 | 10 |
| 2099 | Isoform 1 of Paraspeckle component 1 | IPI00103525 | -0.833 | 0.06480 | 13 | 9 | 10 | 7 |
| 2100 | Eukaryotic translation initiation factor 3 subunit G | IPI00290460 | -0.838 | 0.06457 | 7 | 6 | 5 | 4 |
| 2101 | Valyl-tRNA synthetase | IPI00000873 | -0.840 | 0.06428 | 36 | 30 | 28 | 30 |
| 2102 | Isoform 2 of Filamin-A | IPI00302592 | -0.841 | 0.06419 | 105 | 111 | 100 | 103 |
| 2103 | cDNA FLJ51909, highly similar to Serine-threonine kinase receptor-associatedprotein | IPI00294536 | -0.870 | 0.06338 | 10 | 10 | 7 | 8 |
| 2104 | Putative uncharacterized protein FUBP3 | IPI00063245 | -0.870 | 0.06338 | 9 | 11 | 8 | 7 |
| 2105 | Thioredoxin domain-containing protein 17 | IPI00646689 | -0.871 | 0.06338 | 5 | 7 | 4 | 4 |
| 2106 | Histone-binding protein RBBP4 | IPI00328319 | -0.871 | 0.06338 | 5 | 7 | 4 | 4 |
| 2107 | Isoform 3 of Rapamycin-insensitive companion of mTOR | IPI00166528 | -0.871 | 0.06338 | 7 | 5 | 5 | 3 |
| 2108 | cDNA FLJ14239 fis, clone NT2RP5003512, highly similar to Exportin-5 | IPI00549861 | -0.871 | 0.06338 | 7 | 5 | 3 | 5 |
| 2109 | Telomeric repeat-binding factor 2-interacting protein 1 | IPI00008961 | -0.871 | 0.06338 | 6 | 6 | 5 | 3 |
| 2110 | 39S ribosomal protein L1, mitochondrial | IPI00549381 | -0.871 | 0.06338 | 6 | 6 | 5 | 3 |
| 2111 | nardilysin isoform a | IPI00243221 | -0.871 | 0.06338 | 7 | 5 | 4 | 4 |
| 2112 | Isoform 1 of Methionine adenosyltransferase 2 subunit beta | IPI00002324 | -0.871 | 0.06338 | 7 | 5 | 4 | 4 |
| 2113 | DNA-directed RNA polymerase II subunit RPB1 | IPI00031627 | -0.878 | 0.05811 | 12 | 18 | 12 | 12 |
| 2114 | SUMO-activating enzyme subunit 1 | IPI00033130 | -0.891 | 0.05725 | 9 | 10 | 7 | 7 |
| 2115 | Microtubule-associated protein RP/EB family member 1 | IPI00017596 | -0.891 | 0.05725 | 10 | 9 | 8 | 6 |
| 2116 | Isoform 1 of Splicing factor, arginine/serine-rich 7 | IPI00003377 | -0.891 | 0.05716 | 16 | 13 | 12 | 11 |
| 2117 | Isoform 1 of RNA-binding protein 39 | IPI00163505 | -0.891 | 0.05716 | 16 | 13 | 11 | 12 |
| 2118 | Isoform 1 of Heterogeneous nuclear ribonucleoprotein D0 | IPI00028888 | -0.898 | 0.05697 | 21 | 20 | 18 | 16 |
| 2119 | Isoform 1 of Sodium-coupled neutral amino acid transporter 2 | IPI00410034 | -0.905 | 0.05668 | 14 | 14 | 12 | 10 |
| 2120 | cDNA FLJ50992, highly similar to Coronin-1C | IPI00798401 | -0.906 | 0.05588 | 3 | 3 | 1 | 2 |
| 2121 | Isoform Mitochondrial of Glutathione reductase, mitochondrial | IPI00016862 | -0.906 | 0.05588 | 4 | 2 | 1 | 2 |
| 2122 | Brefeldin A-inhibited guanine nucleotide-exchange protein 2 | IPI00002186 | -0.906 | 0.05588 | 4 | 2 | 2 | 0 |
| 2123 | N-acetylgalactosaminyltransferase 7 | IPI00328391 | -0.906 | 0.05588 | 3 | 3 | 2 | 1 |
| 2124 | Isoform 1 of THO complex subunit 1 | IPI00305374 | -0.906 | 0.05588 | 4 | 2 | 1 | 2 |
| 2125 | 39S ribosomal protein L19, mitochondrial | IPI00027096 | -0.906 | 0.05588 | 4 | 2 | 2 | 0 |
| 2126 | 4-hydroxyphenylpyruvate dioxygenase-like protein | IPI00063762 | -0.906 | 0.05588 | 4 | 2 | 2 | 1 |
| 2127 | Ras-related protein Rab-8A | IPI00028481 | -0.906 | 0.05588 | 2 | 4 | 1 | 2 |
| 2128 | Full-length cDNA 5-PRIME end of clone CS0DJ009YL13 of T cells (Jurkat cell line) of Homo sapiens | IPI00384016 | -0.906 | 0.05588 | 3 | 3 | 1 | 2 |
| 2129 | 26S proteasome non-ATPase regulatory subunit 10 | IPI00003565 | -0.906 | 0.05588 | 2 | 4 | 2 | 1 |
| 2130 | Isoform 3 of Nuclear transcription factor Y subunit gamma | IPI00071697 | -0.906 | 0.05588 | 4 | 2 | 1 | 2 |
| 2131 | Isoform 1 of Alpha-parvin | IPI00018963 | -0.906 | 0.05588 | 3 | 3 | 1 | 2 |
| 2132 | NADH dehydrogenase [ubiquinone] iron-sulfur protein 2, mitochondrial | IPI00025239 | -0.906 | 0.05588 | 3 | 3 | 1 | 2 |
| 2133 | Isoform 2 of Fumarylacetoacetate hydrolase domain-containing protein 1 | IPI00440828 | -0.906 | 0.05588 | 3 | 3 | 1 | 2 |
| 2134 | Membrane-associated progesterone receptor component 1 | IPI00220739 | -0.906 | 0.05588 | 3 | 3 | 2 | 1 |
| 2135 | cytochrome b5 type B precursor | IPI00303954 | -0.910 | 0.05588 | 5 | 6 | 4 | 3 |
| 2136 | Actin-related protein 2/3 complex subunit 3 | IPI00005162 | -0.910 | 0.05588 | 4 | 7 | 4 | 3 |
| 2137 | Isoform 1 of Protein phosphatase 1 regulatory subunit 12A | IPI00183002 | -0.910 | 0.05588 | 7 | 4 | 3 | 4 |
| 2138 | Nucleolar protein 16 | IPI00032849 | -0.910 | 0.05588 | 5 | 6 | 6 | 1 |
| 2139 | SWI/SNF-related matrix-associated actin-dependent regulator of chromatin subfamily A member 5 | IPI00297211 | -0.913 | 0.05583 | 9 | 9 | 7 | 6 |
| 2140 | Isoform 2 of Nipped-B-like protein | IPI00026466 | -0.913 | 0.05583 | 7 | 11 | 6 | 7 |
| 2141 | Isoform 1 of DNA primase large subunit | IPI00027705 | -0.913 | 0.05583 | 9 | 9 | 8 | 5 |
| 2142 | UPF0368 protein Cxorf26 | IPI00107104 | -0.913 | 0.05583 | 9 | 9 | 6 | 7 |
| 2143 | Eukaryotic translation initiation factor 5B | IPI00299254 | -0.916 | 0.05555 | 29 | 25 | 25 | 21 |
| 2144 | UMP-CMP kinase isoform a | IPI00219953 | -0.920 | 0.05531 | 13 | 14 | 9 | 12 |
| 2145 | Dipeptidyl peptidase 1 | IPI00022810 | -0.920 | 0.05531 | 15 | 12 | 12 | 9 |
| 2146 | Isoform 2 of Structural maintenance of chromosomes flexible hinge domain-containing protein 1 | IPI00465022 | -0.936 | 0.05517 | 11 | 15 | 10 | 10 |
| 2147 | Galectin-3 | IPI00465431 | -0.938 | 0.05403 | 10 | 7 | 5 | 7 |
| 2148 | Isoform 1 of Pescadillo homolog | IPI00003768 | -0.938 | 0.05403 | 8 | 9 | 6 | 6 |
| 2149 | cDNA FLJ60124, highly similar to Mitochondrial dicarboxylate carrier | IPI00005537 | -0.938 | 0.05403 | 8 | 9 | 6 | 6 |
| 2150 | Exportin-5 | IPI00640703 | -0.938 | 0.05403 | 7 | 10 | 6 | 6 |
| 2151 | retinol-binding protein 1 isoform a | IPI00219718 | -0.938 | 0.05403 | 8 | 9 | 6 | 6 |
| 2152 | Isoform 1 of Replication factor C subunit 2 | IPI00017412 | -0.938 | 0.05403 | 8 | 9 | 7 | 5 |
| 2153 | Serine hydroxymethyltransferase, mitochondrial | IPI00002520 | -0.947 | 0.05369 | 26 | 24 | 21 | 21 |
| 2154 | Isoform 2 of Guanine nucleotide-binding protein G(i) subunit alpha-2 | IPI00217906 | -0.953 | 0.05346 | 14 | 11 | 11 | 8 |
| 2155 | Isoform 1 of Large proline-rich protein BAT3 | IPI00465128 | -0.953 | 0.05346 | 13 | 12 | 8 | 11 |
| 2156 | Isoform 1 of Transcription elongation regulator 1 | IPI00247871 | -0.955 | 0.05346 | 5 | 5 | 5 | 1 |
| 2157 | Thioredoxin-related transmembrane protein 1 | IPI00395887 | -0.955 | 0.05346 | 5 | 5 | 2 | 4 |
| 2158 | Cell differentiation protein RCD1 homolog | IPI00023101 | -0.955 | 0.05346 | 5 | 5 | 3 | 3 |
| 2159 | Isoform Long of Tight junction protein ZO-1 | IPI00216219 | -0.955 | 0.05346 | 5 | 5 | 4 | 2 |
| 2160 | Ribosomal protein S6 kinase alpha-1 | IPI00017305 | -0.955 | 0.05346 | 6 | 4 | 4 | 2 |
| 2161 | Unhealthy ribosome biogenesis protein 2 homolog | IPI00028980 | -0.955 | 0.05346 | 4 | 6 | 3 | 3 |
| 2162 | 26S proteasome non-ATPase regulatory subunit 5 | IPI00002134 | -0.955 | 0.05346 | 4 | 6 | 3 | 3 |
| 2163 | Isoform 1 of Prolyl 4-hydroxylase subunit alpha-1 | IPI00009923 | -0.955 | 0.05346 | 6 | 4 | 4 | 2 |
| 2164 | Isoform 1 of Insulin-like growth factor 2 mRNA-binding protein 2 | IPI00179713 | -0.955 | 0.05346 | 7 | 3 | 2 | 4 |
| 2165 | Phosphoglycerate mutase 2 | IPI00218570 | -0.955 | 0.05346 | 4 | 6 | 3 | 3 |
| 2166 | SF3A2 protein (Fragment) | IPI00017341 | -0.955 | 0.05346 | 6 | 4 | 2 | 4 |
| 2167 | Plakophilin-3 | IPI00026952 | -0.955 | 0.05346 | 5 | 5 | 3 | 3 |
| 2168 | 39S ribosomal protein L48, mitochondrial | IPI00295066 | -0.955 | 0.05346 | 5 | 5 | 3 | 3 |
| 2169 | Isoform 1 of Melanoma-associated antigen D2 | IPI00009542 | -0.955 | 0.05346 | 6 | 4 | 3 | 3 |
| 2170 | Isoform 1 of UBX domain-containing protein 1 | IPI00027378 | -0.955 | 0.05346 | 6 | 4 | 3 | 3 |
| 2171 | DNA replication licensing factor MCM5 | IPI00018350 | -0.963 | 0.04743 | 28 | 20 | 22 | 18 |
| 2172 | Isoform 1 of Protein-L-isoaspartate(D-aspartate) O-methyltransferase | IPI00411680 | -0.966 | 0.04686 | 10 | 6 | 6 | 5 |
| 2173 | Putative uncharacterized protein NAPRT1 | IPI00412498 | -0.966 | 0.04686 | 8 | 8 | 6 | 5 |
| 2174 | Isoform 1 of Calcineurin-like phosphoesterase domain-containing protein 1 | IPI00305010 | -0.966 | 0.04686 | 9 | 7 | 6 | 5 |
| 2175 | Isoform 2 of DNA replication licensing factor MCM7 | IPI00219740 | -0.971 | 0.04681 | 10 | 14 | 7 | 11 |
| 2176 | Isoform 5 of Dynamin-1-like protein | IPI00037283 | -0.972 | 0.04572 | 24 | 23 | 20 | 19 |
| 2177 | Heat shock protein beta-1 | IPI00025512 | -0.982 | 0.04539 | 24 | 22 | 21 | 17 |
| 2178 | Isoform 1 of 3-hydroxyacyl-CoA dehydrogenase type-2 | IPI00017726 | -0.982 | 0.04539 | 22 | 24 | 20 | 18 |
| 2179 | Isoform M2 of Pyruvate kinase isozymes M1/M2 | IPI00479186 | -0.982 | 0.04529 | 87 | 91 | 86 | 78 |

| No. | Description | Accession number | STN[1] | p-Value[1] | Con_A[2] | Con_B[2] | LUTEO_A[2] | LUTEO_B[2] |
|---|---|---|---|---|---|---|---|---|
| 2180 | Ribonucleoside-diphosphate reductase large subunit | IPI00013871 | -0.989 | 0.04510 | 16 | 17 | 16 | 10 |
| 2181 | Probable phosphoglycerate mutase 4 | IPI00374975 | -0.990 | 0.04505 | 13 | 10 | 8 | 9 |
| 2182 | Serine/threonine-protein kinase mTOR | IPI00031410 | -0.997 | 0.04491 | 7 | 8 | 5 | 5 |
| 2183 | Isoform 1 of Inorganic pyrophosphatase 2, mitochondrial | IPI00301109 | -0.997 | 0.04491 | 8 | 7 | 5 | 5 |
| 2184 | Isoform 1 of Dipeptidyl peptidase 3 | IPI00020672 | -1.001 | 0.04486 | 24 | 20 | 19 | 17 |
| 2185 | cDNA FLJ56307, highly similar to Ubiquitin thioesterase protein OTUB1 | IPI00000581 | -1.003 | 0.04472 | 18 | 14 | 13 | 12 |
| 2186 | Isoform Heart of ATP synthase subunit gamma, mitochondrial | IPI00395769 | -1.003 | 0.04472 | 16 | 16 | 13 | 12 |
| 2187 | Neuroblast differentiation-associated protein AHNAK | IPI00021812 | -1.009 | 0.04458 | 6 | 3 | 2 | 3 |
| 2188 | Protein VAC14 homolog | IPI00025160 | -1.009 | 0.04458 | 5 | 4 | 2 | 3 |
| 2189 | Isoform 1 of Protein virilizer homolog | IPI00036742 | -1.009 | 0.04458 | 4 | 5 | 2 | 3 |
| 2190 | Isoform 2 of Suppressor of G2 allele of SKP1 homolog | IPI00791573 | -1.009 | 0.04458 | 6 | 3 | 2 | 3 |
| 2191 | 28S ribosomal protein S22, mitochondrial | IPI00013146 | -1.009 | 0.04458 | 7 | 2 | 2 | 3 |
| 2192 | Isoform 1 of General transcription factor 3C polypeptide 1 | IPI00414482 | -1.009 | 0.04458 | 3 | 6 | 1 | 4 |
| 2193 | Inosine triphosphate pyrophosphatase | IPI00018783 | -1.009 | 0.04458 | 4 | 5 | 4 | 1 |
| 2194 | Nucleolysin TIAR | IPI00005615 | -1.009 | 0.04458 | 5 | 4 | 3 | 2 |
| 2195 | Isoform 1 of Crooked neck-like protein 1 | IPI00177437 | -1.009 | 0.04458 | 5 | 4 | 2 | 3 |
| 2196 | sideroflexin-3 | IPI00793874 | -1.009 | 0.04458 | 4 | 5 | 3 | 2 |
| 2197 | Mitochondrial import inner membrane translocase subunit Tim23 | IPI00007309 | -1.009 | 0.04458 | 4 | 5 | 4 | 1 |
| 2198 | Isoform Delta-1 of Serine/threonine-protein phosphatase 2A 56 kDa regulatory subunit delta isoform | IPI00000030 | -1.011 | 0.04458 | 1 | 4 | 1 | 0 |
| 2199 | Isoform 2 of Ubiquitin-conjugating enzyme E2 K | IPI00019894 | -1.011 | 0.04458 | 2 | 3 | 0 | 0 |
| 2200 | Nuclear pore complex protein Nup153 | IPI00292059 | -1.011 | 0.04458 | 3 | 2 | 1 | 1 |
| 2201 | ATP-dependent RNA helicase DDX51 | IPI00217541 | -1.011 | 0.04458 | 4 | 1 | 1 | 0 |
| 2202 | Selenide, water dikinase 1 | IPI00029056 | -1.011 | 0.04458 | 1 | 4 | 1 | 1 |
| 2203 | Isoform 1 of CD109 antigen | IPI00152540 | -1.011 | 0.04458 | 2 | 3 | 0 | 0 |
| 2204 | 28S ribosomal protein S25, mitochondrial | IPI00013167 | -1.011 | 0.04458 | 2 | 3 | 1 | 1 |
| 2205 | SEC24B protein | IPI00030851 | -1.011 | 0.04458 | 2 | 3 | 1 | 1 |
| 2206 | DNA-directed RNA polymerase III subunit RPC1 | IPI00024163 | -1.011 | 0.04458 | 3 | 2 | 1 | 0 |
| 2207 | Metastasis-associated in colon cancer protein 1 | IPI00376087 | -1.011 | 0.04458 | 3 | 2 | 1 | 0 |
| 2208 | Isoform 1 of Regulation of nuclear pre-mRNA domain-containing protein 2 | IPI00384541 | -1.011 | 0.04458 | 3 | 2 | 0 | 0 |
| 2209 | 39S ribosomal protein L17, mitochondrial | IPI00172591 | -1.011 | 0.04458 | 2 | 3 | 0 | 1 |
| 2210 | 39S ribosomal protein L38, mitochondrial | IPI00783656 | -1.011 | 0.04458 | 3 | 2 | 1 | 1 |
| 2211 | Isoform 2 of RNA-binding protein 47 | IPI00005042 | -1.011 | 0.04458 | 1 | 4 | 0 | 1 |
| 2212 | Protein LLP homolog | IPI00031615 | -1.011 | 0.04458 | 2 | 3 | 1 | 1 |
| 2213 | Isoform 1 of Cytochrome c oxidase assembly protein COX15 homolog | IPI00419869 | -1.011 | 0.04458 | 2 | 3 | 1 | 1 |
| 2214 | Isoform 2 of 5'-3' exoribonuclease 1 | IPI00657645 | -1.011 | 0.04458 | 2 | 3 | 0 | 1 |
| 2215 | Phosphoglycolate phosphatase | IPI00177008 | -1.011 | 0.04458 | 2 | 3 | 1 | 0 |
| 2216 | ATP-dependent RNA helicase DDX54 isoform 1 | IPI00152510 | -1.011 | 0.04458 | 3 | 2 | 0 | 0 |
| 2217 | Protein Red | IPI00011875 | -1.011 | 0.04458 | 2 | 3 | 1 | 0 |
| 2218 | NDUFB10 protein | IPI00074489 | -1.011 | 0.04458 | 2 | 3 | 1 | 1 |
| 2219 | Isoform 1 of Ubiquitin carboxyl-terminal hydrolase 19 | IPI00016589 | -1.011 | 0.04458 | 2 | 3 | 1 | 1 |
| 2220 | Atlastin-1 | IPI00103530 | -1.011 | 0.04458 | 3 | 2 | 1 | 0 |
| 2221 | Calcium-binding protein 39-like | IPI00026359 | -1.011 | 0.04458 | 3 | 2 | 0 | 1 |
| 2222 | Isoform 2 of Diphosphoinositol polyphosphate phosphohydrolase 2 | IPI00021408 | -1.011 | 0.04458 | 2 | 3 | 1 | 0 |
| 2223 | Charged multivesicular body protein 4b | IPI00025974 | -1.011 | 0.04458 | 2 | 3 | 0 | 0 |
| 2224 | Isoform 2 of DNA-directed RNA polymerase I subunit RPA2 | IPI00026445 | -1.011 | 0.04458 | 3 | 2 | 0 | 1 |
| 2225 | Guanine nucleotide-binding protein G(i) subunit alpha-1 | IPI00337415 | -1.011 | 0.04458 | 3 | 2 | 1 | 1 |
| 2226 | cDNA: FLJ22686 fis, clone HSI10987 | IPI00306532 | -1.011 | 0.04458 | 1 | 4 | 0 | 0 |
| 2227 | Pterin-4-alpha-carbinolamine dehydratase | IPI00218568 | -1.011 | 0.04458 | 3 | 2 | 1 | 1 |
| 2228 | Hydroxymethylglutaryl-CoA lyase, mitochondrial | IPI00293564 | -1.011 | 0.04458 | 3 | 2 | 1 | 0 |
| 2229 | Ribosome biogenesis regulatory protein homolog | IPI00014253 | -1.011 | 0.04458 | 2 | 3 | 1 | 1 |
| 2230 | Nucleoside-triphosphatase C1orf57 | IPI00031570 | -1.011 | 0.04458 | 3 | 2 | 1 | 0 |
| 2231 | cDNA FLJ43556 fis, clone PROST2018511, highly similar to Growth factor receptor-bound protein 7 | IPI00448767 | -1.011 | 0.04458 | 3 | 2 | 1 | 0 |
| 2232 | Threonine synthase-like 1 | IPI00016287 | -1.011 | 0.04458 | 3 | 2 | 1 | 1 |
| 2233 | Isoform 1 of Lysine-specific demethylase 2A | IPI00166009 | -1.011 | 0.04458 | 3 | 2 | 1 | 0 |
| 2234 | Putative high mobility group protein B3-like-1 | IPI00006437 | -1.011 | 0.04458 | 4 | 1 | 1 | 1 |
| 2235 | 39S ribosomal protein L41, mitochondrial | IPI00217553 | -1.011 | 0.04458 | 3 | 2 | 0 | 1 |
| 2236 | Serine/threonine-protein phosphatase 2A 56 kDa regulatory subunit alpha isoform | IPI00014978 | -1.011 | 0.04458 | 2 | 3 | 0 | 0 |
| 2237 | NudC domain-containing protein 2 | IPI00103142 | -1.011 | 0.04458 | 3 | 2 | 0 | 0 |
| 2238 | Tubulin--tyrosine ligase-like protein 12 | IPI00029048 | -1.011 | 0.04287 | 23 | 20 | 19 | 16 |
| 2239 | Peptidyl-prolyl cis-trans isomerase FKBP4 | IPI00219005 | -1.012 | 0.04282 | 48 | 44 | 48 | 33 |
| 2240 | Probable ATP-dependent RNA helicase DDX5 | IPI00017617 | -1.017 | 0.04282 | 45 | 46 | 46 | 34 |
| 2241 | Isoform 1 of Catenin alpha-1 | IPI00215948 | -1.031 | 0.04244 | 6 | 8 | 4 | 5 |
| 2242 | Splicing factor, arginine/serine-rich 9 | IPI00012340 | -1.031 | 0.04244 | 7 | 7 | 5 | 4 |
| 2243 | Isoform 1 of Nucleolar protein 6 | IPI00152890 | -1.031 | 0.04244 | 6 | 8 | 4 | 5 |
| 2244 | DnaJ homolog subfamily C member 8 | IPI00003438 | -1.031 | 0.04244 | 7 | 7 | 5 | 4 |
| 2245 | Isoform 3 of Protein PRRC1 | IPI00217053 | -1.031 | 0.04244 | 7 | 7 | 5 | 4 |
| 2246 | 26S proteasome non-ATPase regulatory subunit 6 | IPI00014151 | -1.033 | 0.04230 | 10 | 11 | 8 | 7 |
| 2247 | proteasome-associated protein ECM29 homolog | IPI00157790 | -1.056 | 0.04197 | 22 | 17 | 15 | 16 |
| 2248 | Protein mago nashi homolog 2 | IPI00059292 | -1.057 | 0.04197 | 12 | 8 | 8 | 6 |
| 2249 | Serine/threonine-protein phosphatase 2A catalytic subunit alpha isoform | IPI00008380 | -1.057 | 0.04197 | 11 | 9 | 6 | 8 |
| 2250 | 40S ribosomal protein S4, X isoform | IPI00217030 | -1.060 | 0.04111 | 26 | 25 | 23 | 19 |
| 2251 | Ras GTPase-activating protein-binding protein 1 | IPI00012442 | -1.068 | 0.04097 | 17 | 21 | 17 | 13 |
| 2252 | Rho-associated protein kinase 2 | IPI00307155 | -1.070 | 0.04073 | 6 | 7 | 3 | 5 |
| 2253 | Probable ATP-dependent RNA helicase DDX52 | IPI00032423 | -1.070 | 0.04073 | 5 | 8 | 5 | 3 |
| 2254 | Eukaryotic translation elongation factor 1 epsilon-1 | IPI00003588 | -1.070 | 0.04073 | 7 | 6 | 4 | 4 |
| 2255 | 14-3-3 protein eta | IPI00216319 | -1.070 | 0.04073 | 6 | 7 | 5 | 3 |
| 2256 | DnaJ homolog subfamily C member 9 | IPI00154975 | -1.070 | 0.04073 | 8 | 5 | 5 | 3 |
| 2257 | 1,4-alpha-glucan-branching enzyme | IPI00296635 | -1.077 | 0.04040 | 4 | 4 | 3 | 0 |
| 2258 | Eukaryotic translation initiation factor 2A | IPI00012462 | -1.077 | 0.04040 | 4 | 4 | 2 | 2 |
| 2259 | Isoform 1 of V-type proton ATPase subunit H | IPI00296191 | -1.077 | 0.04040 | 3 | 5 | 2 | 2 |
| 2260 | Ladinin-1 | IPI00514234 | -1.077 | 0.04040 | 3 | 5 | 2 | 2 |
| 2261 | Protein SCO1 homolog, mitochondrial | IPI00027233 | -1.077 | 0.04040 | 5 | 3 | 2 | 2 |
| 2262 | Probable ribosome biogenesis protein NEP1 | IPI00025347 | -1.077 | 0.04040 | 5 | 3 | 0 | 3 |
| 2263 | Programmed cell death protein 6 | IPI00025277 | -1.077 | 0.04040 | 4 | 4 | 0 | 3 |
| 2264 | Guanine nucleotide-binding protein G(k) subunit alpha | IPI00220578 | -1.077 | 0.04040 | 6 | 2 | 2 | 2 |
| 2265 | 28S ribosomal protein S31, mitochondrial | IPI00294242 | -1.077 | 0.04040 | 4 | 4 | 1 | 3 |
| 2266 | Isoform 1 of Protein POF1B | IPI00103242 | -1.077 | 0.04040 | 3 | 5 | 3 | 1 |
| 2267 | Delta-1-pyrroline-5-carboxylate dehydrogenase, mitochondrial | IPI00217871 | -1.077 | 0.04040 | 5 | 3 | 2 | 2 |
| 2268 | Protein kinase, cAMP-dependent, regulatory, type II, alpha, isoform CRA_b | IPI00063234 | -1.077 | 0.04040 | 4 | 4 | 2 | 2 |
| 2269 | 74 kDa protein | IPI00290439 | -1.077 | 0.04040 | 4 | 4 | 3 | 0 |
| 2270 | Sphingosine-1-phosphate lyase 1 | IPI00099463 | -1.077 | 0.04040 | 3 | 5 | 2 | 2 |
| 2271 | Heterogeneous nuclear ribonucleoprotein H2 | IPI00026230 | -1.077 | 0.04040 | 4 | 4 | 1 | 3 |
| 2272 | Isoform Long of Uncharacterized protein C21orf2 | IPI00014472 | -1.077 | 0.04040 | 5 | 3 | 2 | 2 |
| 2273 | Putative RNA-binding protein 3 | IPI00024320 | -1.084 | 0.03565 | 10 | 9 | 6 | 7 |

| No. | Description | Accession number | STN[1] | p-Value[1] | Con_A[2] | Con_B[2] | LUTEO_A[2] | LUTEO_B[2] |
|---|---|---|---|---|---|---|---|---|
| 2274 | Isoform ASF-1 of Splicing factor, arginine/serine-rich 1 | IPI00215884 | -1.087 | 0.03565 | 36 | 26 | 25 | 27 |
| 2275 | 26S proteasome non-ATPase regulatory subunit 12 | IPI00185374 | -1.103 | 0.03518 | 14 | 12 | 11 | 8 |
| 2276 | Isoform 2 of SWI/SNF complex subunit SMARCC2 | IPI00150057 | -1.110 | 0.03518 | 22 | 24 | 19 | 18 |
| 2277 | Isoform 2 of Proteasome subunit alpha type-3 | IPI00171199 | -1.112 | 0.03518 | 10 | 8 | 6 | 6 |
| 2278 | Isoform 1 of Peripherin | IPI00013164 | -1.112 | 0.03518 | 8 | 10 | 6 | 6 |
| 2279 | Eukaryotic translation initiation factor 3 subunit K | IPI00033143 | -1.112 | 0.03518 | 10 | 8 | 6 | 6 |
| 2280 | Isoform 1 of Fragile X mental retardation syndrome-related protein 1 | IPI00016249 | -1.116 | 0.03418 | 7 | 5 | 3 | 4 |
| 2281 | Isoform 1 of Glomulin | IPI00074604 | -1.116 | 0.03418 | 5 | 7 | 4 | 3 |
| 2282 | Actin-related protein 3 | IPI00028091 | -1.116 | 0.03418 | 6 | 6 | 3 | 4 |
| 2283 | Isoform 1 of LIM and SH3 domain protein 1 | IPI00000861 | -1.116 | 0.03418 | 7 | 5 | 4 | 3 |
| 2284 | Protein FRG1 | IPI00004655 | -1.116 | 0.03418 | 5 | 7 | 4 | 3 |
| 2285 | Isoform 1 of Syntenin-1 | IPI00299086 | -1.116 | 0.03418 | 6 | 6 | 4 | 3 |
| 2286 | Isoform GTBP-alt of DNA mismatch repair protein Msh6 | IPI00106847 | -1.121 | 0.03361 | 25 | 20 | 19 | 17 |
| 2287 | Vigilin | IPI00022228 | -1.123 | 0.03361 | 18 | 16 | 15 | 11 |
| 2288 | Pre-mRNA-splicing factor ATP-dependent RNA helicase PRP16 | IPI00294211 | -1.139 | 0.03314 | 19 | 14 | 13 | 12 |
| 2289 | Cytochrome c oxidase subunit 2 | IPI00017510 | -1.144 | 0.03304 | 8 | 9 | 6 | 5 |
| 2290 | Stathmin | IPI00479997 | -1.144 | 0.03304 | 10 | 7 | 5 | 6 |
| 2291 | Probable ATP-dependent RNA helicase DDX23 | IPI00006725 | -1.144 | 0.03304 | 7 | 10 | 6 | 5 |
| 2292 | Protein NipSnap homolog 2 | IPI00016077 | -1.144 | 0.03304 | 9 | 8 | 5 | 6 |
| 2293 | Isoform A1-B of Heterogeneous nuclear ribonucleoprotein A1 | IPI00215965 | -1.144 | 0.03304 | 84 | 87 | 77 | 78 |
| 2294 | Isoform 1 of Nuclear autoantigenic sperm protein | IPI00179953 | -1.145 | 0.03304 | 10 | 14 | 7 | 10 |
| 2295 | transcription activator BRG1 isoform D | IPI00029822 | -1.155 | 0.03290 | 10 | 22 | 13 | 11 |
| 2296 | Isoform 1 of Heterogeneous nuclear ribonucleoprotein D-like | IPI00011274 | -1.156 | 0.03271 | 23 | 19 | 20 | 13 |
| 2297 | Bifunctional aminoacyl-tRNA synthetase | IPI00013452 | -1.157 | 0.03271 | 41 | 42 | 32 | 39 |
| 2298 | Elongation factor 1-alpha 2 | IPI00014424 | -1.161 | 0.03247 | 403 | 377 | 386 | 364 |
| 2299 | Isoform 1 of Elongation factor 1-delta | IPI00023048 | -1.164 | 0.03247 | 28 | 25 | 23 | 20 |
| 2300 | Isoform 1 of Plectin-1 | IPI00014898 | -1.165 | 0.03247 | 142 | 141 | 133 | 130 |
| 2301 | Isoform 1 of Spectrin beta chain, brain 2 | IPI00012645 | -1.166 | 0.03247 | 2 | 5 | 2 | 0 |
| 2302 | Isoform 1 of Ribonuclease H2 subunit C | IPI00382985 | -1.166 | 0.03247 | 5 | 2 | 2 | 1 |
| 2303 | Tyrosine-protein phosphatase non-receptor type 1 | IPI00297261 | -1.166 | 0.03247 | 2 | 5 | 1 | 2 |
| 2304 | Putative uncharacterized protein DKFZp313O211 | IPI00552186 | -1.166 | 0.03247 | 4 | 3 | 2 | 0 |
| 2305 | Isoform 1 of Zinc finger MYM-type protein 3 | IPI00029484 | -1.166 | 0.03247 | 3 | 4 | 1 | 2 |
| 2306 | Isoform 1 of Cullin-3 | IPI00014312 | -1.166 | 0.03247 | 3 | 4 | 1 | 2 |
| 2307 | ANKHD1-EIF4EBP3 protein | IPI00217442 | -1.166 | 0.03247 | 2 | 5 | 2 | 1 |
| 2308 | 39S ribosomal protein L9, mitochondrial | IPI00307409 | -1.166 | 0.03247 | 4 | 3 | 2 | 1 |
| 2309 | Uncharacterized protein C3orf26 | IPI00031679 | -1.166 | 0.03247 | 3 | 4 | 2 | 1 |
| 2310 | lanosterol 14-alpha demethylase isoform 1 | IPI00295772 | -1.166 | 0.03247 | 3 | 4 | 2 | 1 |
| 2311 | Isoform 1 of Uncharacterized methyltransferase WBSCR22 | IPI00013810 | -1.166 | 0.03247 | 3 | 4 | 1 | 2 |
| 2312 | Isoform 1 of Huntingtin-interacting protein K | IPI00335001 | -1.166 | 0.03247 | 3 | 4 | 1 | 2 |
| 2313 | myosin regulatory light polypeptide 9 isoform b | IPI00030929 | -1.166 | 0.03247 | 4 | 3 | 1 | 2 |
| 2314 | Isoform 1 of Nuclear pore complex protein Nup155 | IPI00026625 | -1.168 | 0.03247 | 13 | 10 | 10 | 6 |
| 2315 | 26S proteasome non-ATPase regulatory subunit 13 isoform 2 | IPI00375380 | -1.168 | 0.03247 | 20 | 21 | 16 | 16 |
| 2316 | Isoform 1 of SAM domain and HD domain-containing protein 1 | IPI00294739 | -1.169 | 0.03247 | 5 | 6 | 4 | 2 |
| 2317 | Probable dimethyladenosine transferase | IPI00004459 | -1.169 | 0.03247 | 5 | 6 | 5 | 1 |
| 2318 | Isoform 2 of CDK5 regulatory subunit-associated protein 3 | IPI00018780 | -1.169 | 0.03247 | 6 | 5 | 3 | 3 |
| 2319 | Epithelial cell adhesion molecule | IPI00296215 | -1.169 | 0.03247 | 5 | 6 | 3 | 3 |
| 2320 | Villin-1 | IPI00218852 | -1.175 | 0.03242 | 34 | 31 | 30 | 24 |
| 2321 | ribonucleoprotein PTB-binding 1 | IPI00217661 | -1.179 | 0.03242 | 9 | 7 | 6 | 4 |
| 2322 | Aconitate hydratase, mitochondrial | IPI00017855 | -1.179 | 0.03242 | 7 | 9 | 6 | 4 |
| 2323 | Isoform 1 of Alpha-aminoadipic semialdehyde dehydrogenase | IPI00221234 | -1.179 | 0.03242 | 7 | 9 | 7 | 3 |
| 2324 | Serine/threonine-protein kinase PAK 2 | IPI00419979 | -1.193 | 0.03053 | 9 | 13 | 7 | 8 |
| 2325 | Tripeptidyl-peptidase 2 | IPI00020416 | -1.205 | 0.03034 | 24 | 25 | 18 | 21 |
| 2326 | Peptidyl-prolyl cis-trans isomerase D | IPI00003927 | -1.219 | 0.03024 | 8 | 7 | 5 | 4 |
| 2327 | 60S ribosomal protein L4 | IPI00003918 | -1.221 | 0.03024 | 10 | 11 | 8 | 6 |
| 2328 | Chloride intracellular channel protein 1 | IPI00010896 | -1.228 | 0.03015 | 25 | 22 | 19 | 18 |
| 2329 | Chromobox protein homolog 1 | IPI00010320 | -1.228 | 0.03015 | 27 | 20 | 21 | 16 |
| 2330 | Glutaredoxin-3 | IPI00008552 | -1.229 | 0.03015 | 13 | 15 | 11 | 9 |
| 2331 | Isoform 1 of BRCA2 and CDKN1A-interacting protein | IPI00002203 | -1.232 | 0.02948 | 6 | 4 | 1 | 4 |
| 2332 | Sorting and assembly machinery component 50 homolog | IPI00412713 | -1.232 | 0.02948 | 5 | 5 | 4 | 1 |
| 2333 | Isoform 1 of Wings apart-like protein homolog | IPI00375330 | -1.232 | 0.02948 | 5 | 5 | 3 | 2 |
| 2334 | cDNA FLJ59712, highly similar to Golgi reassembly-stacking protein 2 | IPI00743931 | -1.232 | 0.02948 | 7 | 3 | 2 | 3 |
| 2335 | Isoform 2 of Lysine-specific histone demethylase 1A | IPI00217540 | -1.232 | 0.02948 | 4 | 6 | 2 | 3 |
| 2336 | Isoform 1 of Actin-like protein 6A | IPI00003627 | -1.232 | 0.02948 | 5 | 5 | 3 | 2 |
| 2337 | UBX domain-containing protein 4 | IPI00293946 | -1.232 | 0.02948 | 5 | 5 | 3 | 2 |
| 2338 | Signal recognition particle 14 kDa protein | IPI00293434 | -1.232 | 0.02948 | 7 | 3 | 4 | 1 |
| 2339 | Isoform 1 of Vinculin | IPI00291175 | -1.247 | 0.02915 | 52 | 49 | 45 | 42 |
| 2340 | Isoform 1 of F-actin-capping protein subunit beta | IPI00026185 | -1.250 | 0.02915 | 14 | 13 | 8 | 11 |
| 2341 | Isoform 1 of Cullin-associated NEDD8-dissociated protein 1 | IPI00100160 | -1.250 | 0.02915 | 41 | 43 | 38 | 33 |
| 2342 | Moesin | IPI00219365 | -1.261 | 0.02905 | 32 | 36 | 30 | 26 |
| 2343 | Nucleolar GTP-binding protein 1 | IPI00385042 | -1.264 | 0.02896 | 6 | 8 | 3 | 5 |
| 2344 | Isoform 1 of DNA-binding protein A | IPI00031801 | -1.264 | 0.02896 | 6 | 8 | 4 | 4 |
| 2345 | Isoform 1 of Heterogeneous nuclear ribonucleoprotein K | IPI00216049 | -1.270 | 0.02768 | 122 | 108 | 101 | 109 |
| 2346 | Nicotinamide phosphoribosyltransferase | IPI00018873 | -1.273 | 0.02768 | 10 | 16 | 10 | 8 |
| 2347 | Adenosylhomocysteinase | IPI00012007 | -1.273 | 0.02730 | 20 | 14 | 14 | 11 |
| 2348 | Ubiquitin carboxyl-terminal hydrolase isozyme L3 | IPI00011250 | -1.283 | 0.02725 | 12 | 7 | 6 | 6 |
| 2349 | Isoform 1 of CUGBP Elav-like family member 1 | IPI00034015 | -1.283 | 0.02725 | 10 | 9 | 7 | 5 |
| 2350 | Isoform 5 of Protein polybromo-1 | IPI00023097 | -1.290 | 0.02720 | 3 | 3 | 1 | 1 |
| 2351 | Dual specificity mitogen-activated protein kinase kinase 2 | IPI00003783 | -1.290 | 0.02720 | 3 | 3 | 0 | 1 |
| 2352 | Isocitrate dehydrogenase [NADP], mitochondrial | IPI00011107 | -1.290 | 0.02720 | 4 | 2 | 1 | 0 |
| 2353 | Coronin-1B | IPI00007058 | -1.290 | 0.02720 | 2 | 4 | 1 | 0 |
| 2354 | Isoform Del-701 of Signal transducer and activator of transcription 3 | IPI00306436 | -1.290 | 0.02720 | 3 | 3 | 1 | 1 |
| 2355 | Tubulin beta-3 chain | IPI00013683 | -1.290 | 0.02720 | 3 | 3 | 1 | 1 |
| 2356 | Diphosphoinositol polyphosphate phosphohydrolase 1 | IPI00009148 | -1.290 | 0.02720 | 3 | 3 | 0 | 1 |
| 2357 | RNA polymerase-associated protein CTR9 homolog | IPI00477468 | -1.290 | 0.02720 | 3 | 3 | 0 | 0 |
| 2358 | NADH dehydrogenase [ubiquinone] iron-sulfur protein 4, mitochondrial | IPI00011217 | -1.290 | 0.02720 | 3 | 3 | 1 | 1 |
| 2359 | Isoform 1 of Uncharacterized protein KIAA0528 | IPI00465142 | -1.290 | 0.02720 | 3 | 3 | 1 | 1 |
| 2360 | Isoform 1 of Nuclear-interacting partner of ALK | IPI00301421 | -1.290 | 0.02720 | 4 | 2 | 1 | 1 |
| 2361 | peroxisomal 3,2-trans-enoyl-CoA isomerase isoform 1 | IPI00419263 | -1.290 | 0.02720 | 4 | 2 | 1 | 1 |
| 2362 | Isoform 1 of Structural maintenance of chromosomes protein 6 | IPI00154528 | -1.290 | 0.02720 | 3 | 3 | 1 | 1 |
| 2363 | Isoform 1 of Probable ATP-dependent RNA helicase DHX40 | IPI00410110 | -1.290 | 0.02720 | 3 | 3 | 1 | 1 |
| 2364 | Isoform 2 of Serine/threonine-protein kinase PAK 1 | IPI00289746 | -1.290 | 0.02720 | 3 | 3 | 1 | 0 |
| 2365 | Isoform 1 of Ligatin | IPI00013160 | -1.290 | 0.02720 | 3 | 3 | 1 | 0 |
| 2366 | F-box only protein 7 | IPI00294567 | -1.290 | 0.02720 | 3 | 3 | 0 | 0 |
| 2367 | Integrin-linked protein kinase | IPI00013219 | -1.290 | 0.02720 | 3 | 3 | 1 | 1 |
| 2368 | Isoform 1 of Chromodomain-helicase-DNA-binding protein 4 | IPI00000846 | -1.291 | 0.02060 | 33 | 45 | 36 | 29 |

| No. | Description | Accession number | STN[1] | p-Value[1] | Con_A[2] | Con_B[2] | LUTEO_A[2] | LUTEO_B[2] |
|---|---|---|---|---|---|---|---|---|
| 2369 | Isoform 1 of U2-associated protein SR140 | IPI00143753 | -1.297 | 0.02046 | 12 | 13 | 10 | 7 |
| 2370 | Calpain-2 catalytic subunit | IPI00289758 | -1.297 | 0.02046 | 13 | 12 | 6 | 11 |
| 2371 | splicing factor 3B subunit 2 | IPI00221106 | -1.309 | 0.02046 | 19 | 13 | 12 | 11 |
| 2372 | Protein BUD31 homolog | IPI00013180 | -1.311 | 0.02037 | 6 | 3 | 2 | 2 |
| 2373 | Vacuolar protein sorting-associated protein 26A | IPI00411426 | -1.311 | 0.02037 | 3 | 6 | 3 | 1 |
| 2374 | Activity-dependent neuroprotector homeobox protein | IPI00022215 | -1.311 | 0.02037 | 4 | 5 | 2 | 2 |
| 2375 | Peptidyl-prolyl cis-trans isomerase NIMA-interacting 1 | IPI00013723 | -1.311 | 0.02037 | 5 | 4 | 2 | 2 |
| 2376 | 3-hydroxyisobutyrate dehydrogenase, mitochondrial | IPI00013860 | -1.311 | 0.02037 | 3 | 6 | 2 | 2 |
| 2377 | Claudin-1 | IPI00000691 | -1.311 | 0.02037 | 6 | 3 | 2 | 2 |
| 2378 | NADH dehydrogenase [ubiquinone] flavoprotein 2, mitochondrial | IPI00291328 | -1.311 | 0.02037 | 4 | 5 | 2 | 2 |
| 2379 | Peroxiredoxin-1 | IPI00000874 | -1.314 | 0.02022 | 33 | 29 | 26 | 24 |
| 2380 | Phosphatidylinositol transfer protein alpha isoform | IPI00216048 | -1.315 | 0.02022 | 8 | 5 | 3 | 4 |
| 2381 | Isoform 1 of HEAT repeat-containing protein 3 | IPI00100984 | -1.315 | 0.02022 | 6 | 7 | 2 | 5 |
| 2382 | Isoform 2 of PERQ amino acid-rich with GYF domain-containing protein 2 | IPI00647635 | -1.315 | 0.02022 | 5 | 8 | 4 | 3 |
| 2383 | Isoform Alpha-6X1X2B of Integrin alpha-6 | IPI00010697 | -1.318 | 0.02022 | 9 | 9 | 5 | 6 |
| 2384 | Metastasis-associated protein MTA2 | IPI00171798 | -1.318 | 0.02022 | 9 | 9 | 6 | 5 |
| 2385 | Isoform 1 of Cytoplasmic FMR1-interacting protein 1 | IPI00644231 | -1.318 | 0.02022 | 8 | 10 | 6 | 5 |
| 2386 | Isoform 1 of RuvB-like 1 | IPI00021187 | -1.329 | 0.01946 | 13 | 18 | 9 | 13 |
| 2387 | Ribosomal protein S6 kinase alpha-3 | IPI00020898 | -1.358 | 0.01899 | 8 | 9 | 4 | 6 |
| 2388 | Isoform 1 of Regulator of nonsense transcripts 1 | IPI00034049 | -1.369 | 0.01899 | 19 | 18 | 12 | 15 |
| 2389 | Nestin | IPI00010800 | -1.369 | 0.01899 | 19 | 18 | 14 | 13 |
| 2390 | 39S ribosomal protein L13, mitochondrial | IPI00022403 | -1.375 | 0.01899 | 7 | 5 | 2 | 4 |
| 2391 | Ras-related protein Rap-2c | IPI00009607 | -1.375 | 0.01899 | 6 | 6 | 2 | 4 |
| 2392 | myosin-IXb isoform 1 | IPI00306933 | -1.375 | 0.01899 | 5 | 7 | 3 | 3 |
| 2393 | Isoform 2 of Structural maintenance of chromosomes protein 4 | IPI00328298 | -1.397 | 0.01595 | 28 | 26 | 26 | 16 |
| 2394 | Eukaryotic peptide chain release factor GTP-binding subunit ERF3A | IPI00218829 | -1.402 | 0.01595 | 8 | 8 | 4 | 5 |
| 2395 | Dihydrolipoyl dehydrogenase, mitochondrial | IPI00015911 | -1.406 | 0.01595 | 16 | 19 | 12 | 13 |
| 2396 | UPF0687 protein C20orf27 | IPI00101095 | -1.412 | 0.01595 | 3 | 5 | 1 | 2 |
| 2397 | Protein DEK | IPI00020021 | -1.412 | 0.01595 | 4 | 4 | 2 | 1 |
| 2398 | Serine/threonine-protein kinase 38-like | IPI00237011 | -1.412 | 0.01595 | 5 | 3 | 2 | 1 |
| 2399 | DNA mismatch repair protein Msh3 | IPI00329605 | -1.412 | 0.01595 | 4 | 4 | 2 | 1 |
| 2400 | 39S ribosomal protein L44, mitochondrial | IPI00009680 | -1.412 | 0.01595 | 4 | 4 | 2 | 1 |
| 2401 | Serine/threonine-protein phosphatase 4 catalytic subunit | IPI00012833 | -1.412 | 0.01595 | 4 | 4 | 2 | 1 |
| 2402 | Ribonuclease H2 subunit A | IPI00290192 | -1.412 | 0.01595 | 3 | 5 | 2 | 1 |
| 2403 | Isoform 1 of RNA polymerase II-associated factor 1 homolog | IPI00300333 | -1.412 | 0.01595 | 5 | 3 | 2 | 1 |
| 2404 | programmed cell death 4 isoform 2 | IPI00240675 | -1.412 | 0.01595 | 3 | 5 | 1 | 2 |
| 2405 | Keratin, type I cytoskeletal 14 | IPI00384444 | -1.412 | 0.01595 | 4 | 4 | 2 | 1 |
| 2406 | Ras-related protein Rab-13 | IPI00016373 | -1.412 | 0.01595 | 6 | 2 | 2 | 1 |
| 2407 | Isoform Beta-2 of DNA topoisomerase 2-beta | IPI00027280 | -1.434 | 0.01519 | 26 | 25 | 16 | 23 |
| 2408 | Tubulin beta-1 chain | IPI00006510 | -1.445 | 0.01519 | 19 | 14 | 12 | 11 |
| 2409 | Isoform 1 of Kinectin | IPI00328753 | -1.447 | 0.01519 | 5 | 6 | 4 | 1 |
| 2410 | mortality factor 4 | IPI00001955 | -1.447 | 0.01519 | 5 | 6 | 3 | 2 |
| 2411 | Calpain small subunit 1 | IPI00025084 | -1.447 | 0.01519 | 6 | 5 | 3 | 2 |
| 2412 | Early endosome antigen 1 | IPI00329536 | -1.447 | 0.01519 | 15 | 11 | 11 | 6 |
| 2413 | Probable ATP-dependent RNA helicase DDX47 | IPI00023972 | -1.450 | 0.01495 | 11 | 9 | 7 | 5 |
| 2414 | DNA-directed RNA polymerase II subunit RPB3 | IPI00018288 | -1.452 | 0.01457 | 9 | 6 | 5 | 3 |
| 2415 | Putative uncharacterized protein NAP1L4 | IPI00017763 | -1.460 | 0.01453 | 24 | 25 | 19 | 18 |
| 2416 | Isoform 1 of Catenin beta-1 | IPI00017292 | -1.461 | 0.01453 | 29 | 30 | 25 | 21 |
| 2417 | T-complex protein 1 subunit beta | IPI00297779 | -1.464 | 0.01443 | 118 | 112 | 90 | 117 |
| 2418 | Ubiquitin carboxyl-terminal hydrolase 7 | IPI00003965 | -1.467 | 0.01443 | 14 | 18 | 11 | 11 |
| 2419 | Radixin, isoform CRA_a | IPI00017367 | -1.472 | 0.01405 | 36 | 33 | 31 | 24 |
| 2420 | Isoform 1 of Fanconi anemia group I protein | IPI00019447 | -1.480 | 0.01396 | 18 | 21 | 14 | 14 |
| 2421 | cDNA FLJ59367, highly similar to Adenylosuccinate lyase | IPI00026904 | -1.489 | 0.01396 | 9 | 10 | 6 | 5 |
| 2422 | Isoform 1 of Extended synaptotagmin-1 | IPI00022143 | -1.498 | 0.01396 | 37 | 41 | 30 | 33 |
| 2423 | 60S ribosomal protein L38 | IPI00215790 | -1.506 | 0.01396 | 14 | 10 | 7 | 8 |
| 2424 | Isoform 1 of KH domain-containing, RNA-binding, signal transduction-associated protein 1 | IPI00008575 | -1.509 | 0.01391 | 8 | 6 | 5 | 2 |
| 2425 | DnaJ homolog subfamily B member 1 | IPI00015947 | -1.509 | 0.01391 | 7 | 7 | 3 | 4 |
| 2426 | Nuclear pore complex protein Nup133 | IPI00291200 | -1.513 | 0.01391 | 16 | 14 | 10 | 10 |
| 2427 | Ribosomal L1 domain-containing protein 1 | IPI00008708 | -1.513 | 0.01391 | 14 | 16 | 11 | 9 |
| 2428 | 60S ribosomal protein L23a | IPI00021266 | -1.534 | 0.01315 | 27 | 26 | 19 | 21 |
| 2429 | S-formylglutathione hydrolase | IPI00411706 | -1.535 | 0.01315 | 5 | 5 | 2 | 2 |
| 2430 | Calponin-3 | IPI00216682 | -1.535 | 0.01315 | 6 | 4 | 3 | 1 |
| 2431 | Isoform 1 of Uridine 5'-monophosphate synthase | IPI00003923 | -1.535 | 0.01315 | 6 | 4 | 2 | 2 |
| 2432 | Peptidylprolyl isomerase domain and WD repeat-containing protein 1 | IPI00149650 | -1.535 | 0.01315 | 4 | 6 | 2 | 2 |
| 2433 | telomerase-binding protein EST1A isoform 2 | IPI00014252 | -1.535 | 0.01315 | 6 | 4 | 2 | 2 |
| 2434 | Tropomodulin-3 | IPI00005087 | -1.535 | 0.01315 | 5 | 5 | 2 | 2 |
| 2435 | Isoform 2 of Golgi apparatus protein 1 | IPI00414717 | -1.535 | 0.01315 | 6 | 4 | 0 | 3 |
| 2436 | Isoform Alpha of Signal transducer and activator of transcription 1-alpha/beta | IPI00030781 | -1.539 | 0.01087 | 14 | 15 | 10 | 9 |
| 2437 | HSR1 protein | IPI00384745 | -1.553 | 0.01063 | 5 | 2 | 0 | 1 |
| 2438 | Uncharacterized protein KIAA1797 | IPI00748360 | -1.553 | 0.01063 | 3 | 4 | 0 | 1 |
| 2439 | Isoform 2 of Basigin | IPI00019906 | -1.553 | 0.01063 | 2 | 5 | 1 | 1 |
| 2440 | Rho-associated protein kinase 1 | IPI00022542 | -1.553 | 0.01063 | 4 | 3 | 1 | 0 |
| 2441 | Probable ATP-dependent RNA helicase YTHDC2 | IPI00010200 | -1.553 | 0.01063 | 4 | 3 | 1 | 1 |
| 2442 | cDNA FLJ53927, highly similar to Beta-hexosaminidase alpha chain | IPI00027851 | -1.553 | 0.01063 | 3 | 4 | 1 | 1 |
| 2443 | Isoform 1 of Pleiotropic regulator 1 | IPI00002624 | -1.553 | 0.01063 | 4 | 3 | 1 | 1 |
| 2444 | Aldo-keto reductase family 1 member C1 | IPI00029733 | -1.553 | 0.01063 | 5 | 2 | 0 | 1 |
| 2445 | cDNA FLJ10824 fis, clone NT2RP4001086 (Fragment) | IPI00294810 | -1.553 | 0.01063 | 3 | 4 | 0 | 0 |
| 2446 | Isoform 2 of SWI/SNF-related matrix-associated actin-dependent regulator of chromatin subfamily A | IPI00008422 | -1.553 | 0.01063 | 5 | 2 | 0 | 0 |
| 2447 | Protein naked cuticle homolog 1 | IPI00056339 | -1.553 | 0.01063 | 4 | 3 | 1 | 1 |
| 2448 | Isoform 3 of Tyrosine-protein kinase-like 7 | IPI00168813 | -1.553 | 0.01063 | 5 | 2 | 0 | 0 |
| 2449 | Isoform 1 of Protein syndesmos | IPI00031650 | -1.553 | 0.01063 | 3 | 4 | 1 | 1 |
| 2450 | dynactin subunit 2 | IPI00220503 | -1.553 | 0.01063 | 4 | 3 | 1 | 0 |
| 2451 | UPF0600 protein C5orf51 | IPI00374272 | -1.553 | 0.01063 | 4 | 3 | 1 | 1 |
| 2452 | Tubulin, beta | IPI00645452 | -1.556 | 0.01044 | 30 | 31 | 23 | 24 |
| 2453 | Isoform 1 of Heterogeneous nuclear ribonucleoprotein R | IPI00012074 | -1.560 | 0.01044 | 40 | 43 | 33 | 34 |
| 2454 | T-complex protein 1 subunit alpha | IPI00290566 | -1.561 | 0.01044 | 74 | 67 | 57 | 64 |
| 2455 | Nuclease-sensitive element-binding protein 1 | IPI00031812 | -1.575 | 0.01006 | 11 | 11 | 6 | 7 |
| 2456 | Nucleolar protein 58 | IPI00006379 | -1.575 | 0.01006 | 26 | 24 | 17 | 20 |
| 2457 | Isoform 1AB of Catenin delta-1 | IPI00182469 | -1.575 | 0.01006 | 6 | 7 | 3 | 3 |
| 2458 | Isoform 3 of Ribosome-binding protein 1 | IPI00215743 | -1.575 | 0.01006 | 5 | 8 | 3 | 3 |
| 2459 | Protein flightless-1 homolog | IPI00031023 | -1.595 | 0.00973 | 17 | 10 | 9 | 8 |
| 2460 | Isoform 1 of 60S ribosomal protein L12 | IPI00024933 | -1.606 | 0.00954 | 21 | 19 | 15 | 13 |
| 2461 | X-ray repair cross-complementing protein 6 | IPI00644712 | -1.619 | 0.00940 | 101 | 100 | 91 | 86 |
| 2462 | NADH dehydrogenase [ubiquinone] iron-sulfur protein 3, mitochondrial | IPI00025796 | -1.627 | 0.00926 | 16 | 16 | 12 | 9 |

| No. | Description | Accession number | STN[1] | p-Value[1] | Con_A[2] | Con_B[2] | LUTEO_A[2] | LUTEO_B[2] |
|---|---|---|---|---|---|---|---|---|
| 2463 | Isoform 2 of Microtubule-actin cross-linking factor 1, isoforms 1/2/3/5 | IPI00256861 | -1.648 | 0.00878 | 2 | 7 | 2 | 1 |
| 2464 | Thymidylate synthetase, isoform CRA_a | IPI00103732 | -1.648 | 0.00878 | 5 | 4 | 2 | 1 |
| 2465 | Isoform 1 of ATPase family AAA domain-containing protein 2 | IPI00170548 | -1.648 | 0.00878 | 4 | 5 | 1 | 2 |
| 2466 | cDNA FLJ38069 fis, clone CTONG2015434, highly similar to DOUBLE-STRAND BREAK REPAIR PROTEIN | IPI00029159 | -1.648 | 0.00878 | 6 | 3 | 1 | 2 |
| 2467 | DNA-directed RNA polymerase, mitochondrial precursor | IPI00298738 | -1.648 | 0.00878 | 5 | 4 | 2 | 1 |
| 2468 | DNA-directed RNA polymerase II subunit RPB2 | IPI00027808 | -1.653 | 0.00878 | 17 | 14 | 10 | 10 |
| 2469 | Isoform 1 of Far upstream element-binding protein 1 | IPI00375441 | -1.653 | 0.00878 | 16 | 15 | 11 | 9 |
| 2470 | Glutathione synthetase | IPI00010706 | -1.653 | 0.00878 | 16 | 15 | 10 | 10 |
| 2471 | Scavenger mRNA-decapping enzyme DcpS | IPI00335385 | -1.654 | 0.00878 | 6 | 6 | 3 | 2 |
| 2472 | Actin-related protein 2/3 complex subunit 2 | IPI00005161 | -1.656 | 0.00878 | 9 | 11 | 4 | 7 |
| 2473 | Isoform 1 of Protein diaphanous homolog 1 | IPI00852685 | -1.680 | 0.00859 | 14 | 16 | 11 | 8 |
| 2474 | Histone H1.2 | IPI00217465 | -1.690 | 0.00855 | 27 | 24 | 19 | 18 |
| 2475 | TUBA1C protein | IPI00166768 | -1.692 | 0.00855 | 60 | 57 | 48 | 49 |
| 2476 | Protein of unknown function DUF410 family protein | IPI00419575 | -1.698 | 0.00836 | 8 | 7 | 4 | 3 |
| 2477 | Putative uncharacterized protein ZFR | IPI00748303 | -1.703 | 0.00826 | 10 | 9 | 6 | 4 |
| 2478 | Isoform 2 of Signal recognition particle 68 kDa protein | IPI00102936 | -1.730 | 0.00826 | 20 | 21 | 15 | 13 |
| 2479 | Isoform 1 of Myosin-Ib | IPI00376344 | -1.737 | 0.00821 | 16 | 18 | 12 | 10 |
| 2480 | UPF0160 protein MYG1, mitochondrial | IPI00029444 | -1.751 | 0.00793 | 5 | 6 | 3 | 1 |
| 2481 | Isoform 1 of RNA polymerase II-associated protein 3 | IPI00002408 | -1.751 | 0.00793 | 6 | 5 | 3 | 1 |
| 2482 | Scaffold attachment factor B2 | IPI00005648 | -1.751 | 0.00793 | 7 | 4 | 3 | 1 |
| 2483 | Pre-mRNA branch site protein p14 | IPI00032827 | -1.756 | 0.00788 | 9 | 9 | 5 | 4 |
| 2484 | Ras GTPase-activating-like protein IQGAP1 | IPI00009342 | -1.757 | 0.00788 | 91 | 75 | 73 | 69 |
| 2485 | Phosphatidylinositol-4-phosphate 3-kinase C2 domain-containing subunit alpha | IPI00002580 | -1.770 | 0.00783 | 7 | 7 | 3 | 3 |
| 2486 | Dynein light chain 1, cytoplasmic | IPI00019329 | -1.770 | 0.00783 | 7 | 7 | 4 | 2 |
| 2487 | Elongator complex protein 1 | IPI00293735 | -1.775 | 0.00731 | 12 | 10 | 8 | 4 |
| 2488 | Isoform 1 of Polyadenylate-binding protein 1 | IPI00008524 | -1.801 | 0.00679 | 45 | 45 | 36 | 35 |
| 2489 | Tetratricopeptide repeat protein 37 | IPI00005634 | -1.803 | 0.00679 | 5 | 3 | 1 | 1 |
| 2490 | Phosducin-like protein 3 | IPI00031629 | -1.803 | 0.00679 | 4 | 4 | 1 | 0 |
| 2491 | Seryl-tRNA synthetase, mitochondrial | IPI00328361 | -1.803 | 0.00679 | 5 | 3 | 1 | 1 |
| 2492 | Protein ERGIC-53 | IPI00026530 | -1.803 | 0.00679 | 4 | 4 | 1 | 1 |
| 2493 | Phosphatidylinositol 4-kinase type 2-alpha | IPI00020124 | -1.803 | 0.00679 | 3 | 5 | 0 | 0 |
| 2494 | Carboxypeptidase D | IPI00027078 | -1.803 | 0.00679 | 3 | 5 | 0 | 0 |
| 2495 | Isoform Long of Tyrosine-protein kinase SYK | IPI00018597 | -1.803 | 0.00679 | 4 | 4 | 1 | 0 |
| 2496 | ATP-dependent RNA helicase DHX8 | IPI00031508 | -1.803 | 0.00679 | 5 | 3 | 1 | 0 |
| 2497 | Ribosome biogenesis protein NSA2 homolog | IPI00007089 | -1.803 | 0.00679 | 5 | 3 | 1 | 1 |
| 2498 | Isoform 2 of Peptidyl-prolyl cis-trans isomerase NIMA-interacting 4 | IPI00006658 | -1.803 | 0.00679 | 4 | 4 | 0 | 0 |
| 2499 | Keratin, type I cytoskeletal 17 | IPI00450768 | -1.803 | 0.00679 | 5 | 3 | 1 | 1 |
| 2500 | Putative uncharacterized protein PSME2 | IPI00384051 | -1.809 | 0.00532 | 14 | 12 | 9 | 6 |
| 2501 | cDNA FLJ53975, highly similar to Acetyl-CoA acetyltransferase, cytosolic | IPI00291419 | -1.815 | 0.00532 | 8 | 9 | 4 | 4 |
| 2502 | Isoform 1 of Replication factor C subunit 1 | IPI00375358 | -1.815 | 0.00532 | 8 | 9 | 3 | 5 |
| 2503 | septin-9 isoform e | IPI00455033 | -1.855 | 0.00508 | 6 | 7 | 3 | 2 |
| 2504 | Isoform 2 of Ubiquitin-associated protein 2-like | IPI00029019 | -1.855 | 0.00508 | 8 | 5 | 2 | 3 |
| 2505 | SH3 domain-binding glutamic acid-rich-like protein | IPI00025318 | -1.855 | 0.00508 | 4 | 9 | 4 | 1 |
| 2506 | Putative annexin A2-like protein | IPI00334627 | -1.864 | 0.00508 | 49 | 45 | 40 | 34 |
| 2507 | Tubulin beta-2A chain | IPI00013475 | -1.871 | 0.00498 | 11 | 9 | 4 | 6 |
| 2508 | Isoform 1 of Polyadenylate-binding protein 4 | IPI00012726 | -1.873 | 0.00494 | 5 | 5 | 2 | 1 |
| 2509 | Isoform 1 of Pogo transposable element with ZNF domain | IPI00410717 | -1.873 | 0.00494 | 5 | 5 | 2 | 0 |
| 2510 | Eukaryotic translation initiation factor 3 subunit M | IPI00102069 | -1.882 | 0.00494 | 28 | 27 | 22 | 17 |
| 2511 | Isoform 1 of Protein strawberry notch homolog 1 | IPI00023649 | -1.882 | 0.00494 | 8 | 8 | 3 | 4 |
| 2512 | Vacuolar protein-sorting-associated protein 25 | IPI00031655 | -1.882 | 0.00494 | 9 | 7 | 4 | 3 |
| 2513 | Isoform 1 of Neurochondrin | IPI00549543 | -1.882 | 0.00494 | 9 | 7 | 4 | 3 |
| 2514 | NADH dehydrogenase [ubiquinone] 1 alpha subcomplex subunit 9, mitochondrial | IPI00003968 | -1.888 | 0.00494 | 11 | 13 | 6 | 7 |
| 2515 | Aldehyde dehydrogenase, mitochondrial | IPI00006663 | -1.892 | 0.00494 | 23 | 24 | 16 | 16 |
| 2516 | Isoform 1 of Myb-binding protein 1A | IPI00005024 | -1.911 | 0.00489 | 38 | 41 | 31 | 29 |
| 2517 | Isoform 1 of General transcription factor II-I | IPI00054042 | -1.913 | 0.00489 | 32 | 29 | 21 | 23 |
| 2518 | ATP-dependent RNA helicase DDX3X | IPI00215637 | -1.922 | 0.00475 | 36 | 42 | 32 | 27 |
| 2519 | Isoform 1 of Cullin-4B | IPI00179057 | -1.927 | 0.00475 | 9 | 10 | 6 | 3 |
| 2520 | Gem-associated protein 5 | IPI00291783 | -1.927 | 0.00475 | 12 | 7 | 7 | 2 |
| 2521 | histone deacetylase complex subunit SAP18 | IPI00011698 | -1.927 | 0.00475 | 11 | 8 | 5 | 4 |
| 2522 | Isoform 1 of 39S ribosomal protein L4, mitochondrial | IPI00023334 | -1.927 | 0.00475 | 10 | 9 | 5 | 4 |
| 2523 | Thiosulfate sulfurtransferase | IPI00216293 | -1.933 | 0.00475 | 14 | 9 | 7 | 5 |
| 2524 | Acylamino-acid-releasing enzyme | IPI00337741 | -1.933 | 0.00475 | 13 | 10 | 9 | 3 |
| 2525 | Isoform 2 of S-phase kinase-associated protein 1 | IPI00172421 | -1.933 | 0.00475 | 12 | 11 | 6 | 6 |
| 2526 | ubiquitin and ribosomal protein S27a precursor | IPI00179330 | -1.947 | 0.00475 | 69 | 62 | 50 | 57 |
| 2527 | Ubiquitin-conjugating enzyme E2 L3 | IPI00021347 | -1.959 | 0.00475 | 5 | 7 | 2 | 2 |
| 2528 | Isoform Long of FAS-associated factor 1 | IPI00070643 | -1.959 | 0.00475 | 4 | 8 | 3 | 1 |
| 2529 | 71 kDa protein | IPI00062599 | -1.959 | 0.00475 | 6 | 6 | 3 | 1 |
| 2530 | SERPINE1 mRNA binding protein 1, isoform CRA_d | IPI00410693 | -1.982 | 0.00389 | 10 | 12 | 6 | 5 |
| 2531 | Lamina-associated polypeptide 2, isoform alpha | IPI00216230 | -1.990 | 0.00389 | 9 | 9 | 3 | 5 |
| 2532 | cohesin subunit SA-2 isoform a | IPI00470883 | -1.990 | 0.00389 | 11 | 7 | 5 | 3 |
| 2533 | Isoform 3 of LIM domain only protein 7 | IPI00291802 | -1.990 | 0.00389 | 11 | 7 | 5 | 3 |
| 2534 | Histone H4 | IPI00453473 | -1.995 | 0.00361 | 256 | 250 | 233 | 230 |
| 2535 | cDNA FLJ36192 fis, clone TESTI2027450, highly similar to Eukaryotic translation initiation factor 3 | IPI00654777 | -2.024 | 0.00342 | 22 | 19 | 12 | 14 |
| 2536 | Putative uncharacterized protein DKFZp686L20222 | IPI00026689 | -2.027 | 0.00342 | 27 | 27 | 18 | 19 |
| 2537 | Isoform 1 of CLIP-associating protein 1 | IPI00396279 | -2.041 | 0.00342 | 5 | 4 | 0 | 1 |
| 2538 | Isoform 2 of Bromodomain adjacent to zinc finger domain protein 1A | IPI00383565 | -2.041 | 0.00342 | 5 | 4 | 0 | 0 |
| 2539 | Nitric oxide synthase-interacting protein | IPI00006408 | -2.041 | 0.00342 | 5 | 4 | 1 | 0 |
| 2540 | 60S ribosomal protein L23 | IPI00010153 | -2.042 | 0.00342 | 37 | 40 | 29 | 28 |
| 2541 | Calreticulin | IPI00020599 | -2.048 | 0.00342 | 33 | 35 | 26 | 23 |
| 2542 | Isoform 1 of DNA (cytosine-5)-methyltransferase 1 | IPI00031519 | -2.049 | 0.00342 | 21 | 19 | 10 | 15 |
| 2543 | Programmed cell death protein 10 | IPI00298558 | -2.051 | 0.00342 | 8 | 6 | 2 | 3 |
| 2544 | Keratin, type I cytoskeletal 16 | IPI00217963 | -2.062 | 0.00342 | 8 | 9 | 4 | 3 |
| 2545 | 14-3-3 protein zeta/delta | IPI00021263 | -2.062 | 0.00323 | 38 | 29 | 26 | 22 |
| 2546 | Peptidyl-prolyl cis-trans isomerase FKBP5 | IPI00218775 | -2.091 | 0.00313 | 5 | 6 | 1 | 2 |
| 2547 | poly(rC) binding protein 2 isoform b | IPI00012066 | -2.122 | 0.00294 | 23 | 20 | 15 | 12 |
| 2548 | Putative uncharacterized protein SPTAN1 | IPI00745092 | -2.132 | 0.00294 | 16 | 21 | 11 | 11 |
| 2549 | THO complex subunit 4 | IPI00328840 | -2.145 | 0.00290 | 16 | 11 | 9 | 5 |
| 2550 | Activator of 90 kDa heat shock protein ATPase homolog 1 | IPI00030706 | -2.147 | 0.00290 | 21 | 21 | 11 | 15 |
| 2551 | Condensin complex subunit 1 | IPI00299524 | -2.151 | 0.00290 | 39 | 38 | 32 | 24 |
| 2552 | Isoform Cytoplasmic of Lysyl-tRNA synthetase | IPI00014238 | -2.151 | 0.00290 | 42 | 35 | 27 | 29 |
| 2553 | Isoform 2 of Nucleophosmin | IPI00220740 | -2.161 | 0.00275 | 46 | 48 | 36 | 35 |
| 2554 | Putative uncharacterized protein THADA | IPI00412647 | -2.162 | 0.00275 | 7 | 6 | 2 | 2 |
| 2555 | Isoform 1 of Proteasome activator complex subunit 4 | IPI00005260 | -2.191 | 0.00275 | 11 | 15 | 6 | 7 |
| 2556 | Isoform 1 of Heterogeneous nuclear ribonucleoprotein A3 | IPI00419373 | -2.195 | 0.00275 | 33 | 33 | 21 | 25 |

| No. | Description | Accession number | STN[1] | p-Value[1] | Con_A[2] | Con_B[2] | LUTEO_A[2] | LUTEO_B[2] |
|---|---|---|---|---|---|---|---|---|
| 2557 | Aldehyde dehydrogenase X, mitochondrial | IPI00103467 | -2.241 | 0.00247 | 12 | 13 | 8 | 4 |
| 2558 | Isoform 1 of Rho GTPase-activating protein 18 | IPI00296353 | -2.270 | 0.00247 | 7 | 3 | 1 | 1 |
| 2559 | Palmitoyl-protein thioesterase 1 | IPI00002412 | -2.270 | 0.00247 | 5 | 5 | 1 | 1 |
| 2560 | Putative myosin-XVB | IPI00786880 | -2.270 | 0.00247 | 6 | 4 | 0 | 0 |
| 2561 | cDNA FLJ78679, highly similar to Homo sapiens DEAD (Asp-Glu-Ala-Asp) box polypeptide 46 (DDX46) | IPI00329791 | -2.292 | 0.00180 | 14 | 14 | 7 | 7 |
| 2562 | Pyruvate carboxylase, mitochondrial | IPI00299402 | -2.302 | 0.00176 | 5 | 7 | 1 | 2 |
| 2563 | cDNA: FLJ22728 fis, clone HSI15617 (Fragment) | IPI00386139 | -2.302 | 0.00176 | 5 | 7 | 1 | 2 |
| 2564 | Isoform 2 of Nucleoporin NUP188 homolog | IPI00385001 | -2.325 | 0.00171 | 10 | 7 | 3 | 3 |
| 2565 | Isoform 1 of Transcription factor BTF3 | IPI00221035 | -2.331 | 0.00171 | 11 | 9 | 5 | 3 |
| 2566 | cDNA FLJ34068 fis, clone FCBBF3001918, highly similar to SERINE/THREONINE PROTEIN PHOSPHATASE | IPI00168184 | -2.337 | 0.00157 | 25 | 21 | 13 | 15 |
| 2567 | Guanine nucleotide-binding protein subunit beta-2-like 1 | IPI00848226 | -2.343 | 0.00157 | 72 | 74 | 61 | 55 |
| 2568 | Isoform 1 of Tyrosine-protein kinase BAZ1B | IPI00069817 | -2.363 | 0.00142 | 20 | 15 | 9 | 10 |
| 2569 | Isoform 5 of Glycogen debranching enzyme | IPI00219065 | -2.363 | 0.00142 | 26 | 19 | 13 | 14 |
| 2570 | Proteasome subunit alpha type-2 | IPI00219622 | -2.363 | 0.00142 | 20 | 25 | 12 | 15 |
| 2571 | Electron transfer flavoprotein subunit alpha, mitochondrial | IPI00010810 | -2.422 | 0.00119 | 27 | 27 | 17 | 17 |
| 2572 | NADH-ubiquinone oxidoreductase 75 kDa subunit | IPI00604664 | -2.422 | 0.00119 | 11 | 11 | 5 | 4 |
| 2573 | Isoform 2 of Heat shock protein HSP 90-alpha | IPI00382470 | -2.422 | 0.00119 | 159 | 160 | 145 | 131 |
| 2574 | Isoform 2 of Splicing factor 1 | IPI00294627 | -2.441 | 0.00104 | 16 | 17 | 9 | 8 |
| 2575 | Histone H2B type 2-E | IPI00003935 | -2.453 | 0.00104 | 85 | 92 | 71 | 72 |
| 2576 | Calpain-1 catalytic subunit | IPI00011285 | -2.487 | 0.00104 | 27 | 30 | 21 | 15 |
| 2577 | Protein SGT1 | IPI00027034 | -2.491 | 0.00104 | 5 | 6 | 1 | 1 |
| 2578 | CLASP2 protein | IPI00168165 | -2.491 | 0.00104 | 6 | 5 | 0 | 1 |
| 2579 | Isoform 1 of RNA-binding protein Musashi homolog 2 | IPI00073713 | -2.491 | 0.00104 | 6 | 5 | 1 | 1 |
| 2580 | Putative uncharacterized protein INF2 | IPI00872508 | -2.496 | 0.00104 | 9 | 12 | 4 | 4 |
| 2581 | Keratin-8-like protein 1 | IPI00017870 | -2.547 | 0.00095 | 64 | 67 | 54 | 46 |
| 2582 | DNA polymerase alpha catalytic subunit | IPI00220317 | -2.551 | 0.00095 | 7 | 8 | 2 | 2 |
| 2583 | Heterogeneous nuclear ribonucleoprotein A0 | IPI00011913 | -2.625 | 0.00095 | 20 | 17 | 11 | 8 |
| 2584 | Trifunctional enzyme subunit alpha, mitochondrial | IPI00031522 | -2.680 | 0.00095 | 46 | 47 | 33 | 32 |
| 2585 | Calponin-2 | IPI00015262 | -2.705 | 0.00062 | 7 | 7 | 1 | 2 |
| 2586 | 40S ribosomal protein S10 | IPI00008438 | -2.740 | 0.00062 | 29 | 40 | 22 | 22 |
| 2587 | Heterogeneous nuclear ribonucleoprotein L | IPI00027834 | -2.769 | 0.00062 | 43 | 51 | 31 | 34 |
| 2588 | Eukaryotic translation initiation factor 2 subunit 3 | IPI00297982 | -2.787 | 0.00062 | 9 | 9 | 3 | 2 |
| 2589 | Dihydropyrimidinase-like 2 | IPI00106642 | -2.842 | 0.00062 | 18 | 18 | 8 | 9 |
| 2590 | 32 kDa protein | IPI00176692 | -2.938 | 0.00057 | 149 | 158 | 130 | 126 |
| 2591 | Prohibitin-2 | IPI00027252 | -2.938 | 0.00057 | 109 | 96 | 88 | 74 |
| 2592 | Inorganic pyrophosphatase | IPI00015018 | -2.941 | 0.00057 | 58 | 53 | 41 | 37 |
| 2593 | Glyceraldehyde-3-phosphate dehydrogenase | IPI00219018 | -3.054 | 0.00047 | 468 | 469 | 430 | 423 |
| 2594 | Isoform 1 of La-related protein 1 | IPI00185919 | -3.055 | 0.00047 | 18 | 21 | 9 | 9 |
| 2595 | Isoform 1 of Tensin-3 | IPI00658152 | -3.083 | 0.00047 | 29 | 26 | 14 | 16 |
| 2596 | Heterogeneous nuclear ribonucleoprotein H | IPI00013881 | -3.091 | 0.00047 | 21 | 25 | 12 | 11 |
| 2597 | Isoform B of Serine/threonine-protein kinase 24 | IPI00002212 | -3.100 | 0.00047 | 9 | 9 | 2 | 2 |
| 2598 | Isoform 1 of Heat shock cognate 71 kDa protein | IPI00003865 | -3.108 | 0.00047 | 162 | 161 | 136 | 132 |
| 2599 | Isoform B1 of Heterogeneous nuclear ribonucleoproteins A2/B1 | IPI00396378 | -3.112 | 0.00047 | 229 | 234 | 201 | 198 |
| 2600 | Isoform Long of Spectrin beta chain, brain 1 | IPI00005614 | -3.178 | 0.00038 | 69 | 77 | 53 | 53 |
| 2601 | Ezrin | IPI00843975 | -3.181 | 0.00038 | 34 | 32 | 17 | 21 |
| 2602 | Isoform 1 of Keratin, type I cytoskeletal 13 | IPI00009866 | -3.231 | 0.00038 | 18 | 21 | 10 | 7 |
| 2603 | Isoform M1 of Pyruvate kinase isozymes M1/M2 | IPI00220644 | -3.248 | 0.00038 | 247 | 255 | 216 | 217 |
| 2604 | Putative uncharacterized protein ALB | IPI00022434 | -3.311 | 0.00038 | 8 | 7 | 1 | 1 |
| 2605 | Histone H2A type 1-B/E | IPI00026272 | -3.347 | 0.00024 | 93 | 87 | 66 | 68 |
| 2606 | Actin, cytoplasmic 1 | IPI00021439 | -3.394 | 0.00019 | 200 | 193 | 164 | 164 |
| 2607 | Fatty acid synthase | IPI00026781 | -3.415 | 0.00014 | 218 | 229 | 196 | 182 |
| 2608 | L-lactate dehydrogenase B chain | IPI00219217 | -3.415 | 0.00014 | 128 | 122 | 105 | 91 |
| 2609 | Tubulin alpha-4A chain | IPI00007750 | -3.434 | 0.00014 | 169 | 173 | 129 | 151 |
| 2610 | Isoform Beta-4C of Integrin beta-4 | IPI00027422 | -3.546 | 0.00014 | 15 | 16 | 4 | 6 |
| 2611 | Isoform 1 of Structural maintenance of chromosomes protein 2 | IPI00007927 | -3.555 | 0.00014 | 23 | 20 | 8 | 10 |
| 2612 | ATP synthase subunit beta, mitochondrial | IPI00303476 | -3.650 | 0.00014 | 98 | 100 | 73 | 73 |
| 2613 | 60S ribosomal protein L10a | IPI00412579 | -3.699 | 0.00014 | 42 | 38 | 24 | 21 |
| 2614 | Profilin-1 | IPI00216691 | -3.717 | 0.00014 | 56 | 68 | 37 | 44 |
| 2615 | Isoform 2 of Nuclear mitotic apparatus protein 1 | IPI00006196 | -3.776 | 0.00014 | 28 | 28 | 13 | 13 |
| 2616 | Histone H1.5 | IPI00217468 | -3.807 | 0.00014 | 30 | 29 | 14 | 14 |
| 2617 | Isoform 2 of Inverted formin-2 | IPI00876962 | -3.836 | 0.00014 | 15 | 13 | 3 | 4 |
| 2618 | Hydroxymethylglutaryl-CoA synthase, cytoplasmic | IPI00008475 | -3.976 | 0.00009 | 12 | 9 | 2 | 1 |
| 2619 | Isoform 1 of L-lactate dehydrogenase A chain | IPI00217966 | -3.981 | 0.00009 | 204 | 217 | 170 | 173 |
| 2620 | DNA polymerase delta catalytic subunit | IPI00002894 | -4.230 | 0.00009 | 10 | 10 | 1 | 1 |
| 2621 | Tubulin beta-2C chain | IPI00007752 | -4.238 | 0.00009 | 133 | 124 | 98 | 92 |
| 2622 | Isoform 3 of Spectrin alpha chain, brain | IPI00843765 | -4.331 | 0.00009 | 57 | 65 | 37 | 36 |
| 2623 | Isoform DPI of Desmoplakin | IPI00013933 | -4.410 | 0.00009 | 37 | 44 | 23 | 17 |
| 2624 | Galectin-1 | IPI00219219 | -4.533 | 0.00009 | 21 | 19 | 6 | 5 |
| 2625 | Signal recognition particle 54 kDa protein | IPI00009822 | -4.574 | 0.00009 | 12 | 10 | 1 | 1 |
| 2626 | Eukaryotic initiation factor 4A-I | IPI00025491 | -5.014 | 0.00000 | 80 | 73 | 48 | 43 |
| 2627 | Isoform 1 of Heterogeneous nuclear ribonucleoprotein M | IPI00171903 | -5.218 | 0.00000 | 65 | 72 | 41 | 35 |
| 2628 | Eukaryotic translation initiation factor 3 subunit A | IPI00029012 | -5.294 | 0.00000 | 49 | 47 | 20 | 24 |
| 2629 | Beta-actin-like protein 2 | IPI00003269 | -5.382 | 0.00000 | 81 | 82 | 46 | 49 |
| 2630 | Keratin, type I cytoskeletal 18 | IPI00554788 | -8.306 | 0.00000 | 245 | 277 | 162 | 189 |
| 2631 | Keratin, type I cytoskeletal 19 | IPI00479145 | -8.409 | 0.00000 | 98 | 105 | 46 | 46 |
| 2632 | Keratin, type II cytoskeletal 8 | IPI00554648 | -11.979 | 0.00000 | 519 | 553 | 370 | 372 |
